# Comprehensive genomic characterization of NAC transcription factor family and their response to salt and drought stress in peanut

**DOI:** 10.1186/s12870-020-02678-9

**Published:** 2020-10-02

**Authors:** Cuiling Yuan, Chunjuan Li, Xiaodong Lu, Xiaobo Zhao, Caixia Yan, Juan Wang, Quanxi Sun, Shihua Shan

**Affiliations:** Shandong Peanut Research Institute, Qingdao, 266100 China

**Keywords:** Peanut, NAC gene family, Genome-wide characterization, RNA-seq, RT-qPCR, Salt stress, Drought stress

## Abstract

**Background:**

Peanut is one of the most important oil crop species worldwide. NAC transcription factor (TF) genes play important roles in the salt and drought stress responses of plants by activating or repressing target gene expression. However, little is known about NAC genes in peanut.

**Results:**

We performed a genome-wide characterization of NAC genes from the diploid wild peanut species *Arachis duranensis* and *Arachis ipaensis*, which included analyses of chromosomal locations, gene structures, conserved motifs, expression patterns, and *cis*-acting elements within their promoter regions. In total, 81 and 79 NAC genes were identified from *A. duranensis* and *A. ipaensis* genomes. Phylogenetic analysis of peanut NACs along with their *Arabidopsis* and rice counterparts categorized these proteins into 18 distinct subgroups. Fifty-one orthologous gene pairs were identified, and 46 orthologues were found to be highly syntenic on the chromosomes of both *A. duranensis* and *A. ipaensis*. Comparative RNA sequencing (RNA-seq)-based analysis revealed that the expression of 43 NAC genes was up- or downregulated under salt stress and under drought stress. Among these genes, the expression of 17 genes in cultivated peanut (*Arachis hypogaea*) was up- or downregulated under both stresses. Moreover, quantitative reverse transcription PCR (RT-qPCR)-based analysis revealed that the expression of most of the randomly selected NAC genes tended to be consistent with the comparative RNA-seq results.

**Conclusion:**

Our results facilitated the functional characterization of peanut NAC genes, and the genes involved in salt and drought stress responses identified in this study could be potential genes for peanut improvement.

## Background

Cultivated peanut (*Arachis hypogaea*) is an important economic oil crop species worldwide and used to provide vegetable oil and proteins for human nutrition [1]. During the growth period of peanut plants, their yield is adversely affected by several environmental factors, such as salt and drought stresses, which prevent plants from realizing their full genetic potential [2]. Screening stress-resistant varieties is an important guarantee for achieving targets crop yields [3]. and the identification and utilization of resistant genes is fundamental for the production of new varieties. Transcription factors (TFs), which play roles in activating or repressing gene expression by binding to specific *cis*-acting elements within the promoters of target functional genes, regulate many biological processes [4, 5]. As members of one of the largest plant-specific TF families, NAC [no apical meristem (NAM), *Arabidopsis thaliana* transcription activation factor (ATAF1/2) and cup-shaped cotyledon (CUC2)] proteins have been shown to regulate several biological processes, including responses to salt and drought stresses [6–8]. Remarkably, NAC TFs are considered to be very important for plant adaptations to land [9]. NAC proteins typically have a conserved NAM domain at the N-terminus and a highly variable domain at the C-terminus, the latter of which is related to specific biological functions. NAC family genes have been studied extensively in a variety of plant species, including gymnosperms and embryophytes [10–19]. However, until recently, comprehensive analyses of peanut NAC family genes and their response patterns to salt and drought stresses have been limited.

Increasing evidences have indicated that NAC proteins are involved in plant biotic and abiotic responses. For example, the poplar *NAC13* gene plays a vital role in the salt stress response [20]. Over-expression of a wheat *NAC* (*TaNACL-D1*) enhances resistance to Fusarium head blight disease [21], *TaNAC30* negatively regulates the resistance of wheat to stripe rust [22], and *TaNAC29* can provide salt stress tolerance by enhancing the antioxidant systems [23]. Over-expression of *TsNAC1* from the halophyte *Thellungiella halophila* was shown to improve abiotic stress resistance, especially salt stress tolerance [24]. *SlNAC35* from *Solanum lycopersicum* can promote root growth and development under salt and drought stresses [25], and rice *ONAC033* is induced by drought and can provide strong resistance to both salt and drought stresses in transgenic plants [26]. In peanut, NAC TFs are known to be involved in responses to abiotic stresses. For example, *AhNAC2* and *AhNAC3* can improve salt and drought tolerance in transgenic *Arabidopsis* and tobacco [27, 28], and *AhNAC4* confers enhanced drought tolerance to transgenic tobacco [29]. In addition, over-expression of the *MuNAC4* transgene from horsegram was shown to confer enhanced drought tolerance to transgenic peanut [30].

The genomes of allotetraploid *A. hypogaea* (AABB) and its two wild diploid ancestors *Arachis duranensis* (AA) and *Arachis ipaensis* (BB) were recently sequenced [1, 31–35]. The A and B genomes of the two diploid peanut species are similar to the A and B sub-genomes of cultivated peanut and could be used to identify candidate resistance genes [32, 35]. The availability of genomic information provides opportunities to perform genome-wide analyses of NAC genes and to explore the potential genes involved in peanut biotic and abiotic responses. With the decreasing cost of RNA sequencing (RNA-seq), transcriptome sequencing has become a powerful high-throughput sensitive technique for the analyses of differentially expressed genes. Several peanut RNA-seq datasets containing information on different tissues or responses to different treatments have been published [36–39]. For example, RNA-seq data generated from 22 different tissues and from the development stage of the diploid peanut species *A. duranensis* and *A. ipaensis* have made it convenient to analyse peanut NAC homologue expression profiles [36]. Differential gene expression in response to salt and drought stress has also been analysed, which can help in the identification of NAC genes involved in salt and drought responses [37, 39].

In this paper, we present the results of a genome-wide identification and characterization of NAC genes from wild peanut genomes and their orthologous genes in response to salt and drought stresses in cultivated peanut. We analysed their phylogenetic relationships, structural characteristics, chromosomal locations and gene orthologous gene pairs. We also determined their expression characteristics in different tissues and in response to salt and drought stresses on the basis of RNA-seq data [36, 37, 39]. Seventeen genes were identified as being involved in the response to both salt and drought stresses in cultivated peanut, and these results were confirmed by quantitative reverse transcription PCR (RT-qPCR). The objectives of this study were to provide a theoretical basis for further functional analysis of NAC proteins in peanut and to explore orthologous NAC genes involved in the response to salt and/or drought stresses in cultivated peanut.

## Results

### Identification of NAC proteins from *A. duranensis* and *A. ipaensis*

In total, 81 and 79 NAC genes (Table 1, Additional files 1 and 2) were identified from the diploids *A. duranensis* (~ 1.25 Gb) and *A. ipaensis*(~ 1.56 Gb), respectively, which were less than the totals identified in *Arabidopsis* (105) [40] and rice (141) [41]. However, 164 NAC proteins (Additional files 3 and 4) were identified in the cultivated allotetraploid *A. hypogaea* (~ 2.54 Gb). The number was close to the sum of gene numbers from *A. duranensis* and *A. ipaensis*. The density of NAC genes in *A. duranensis* (0.07/Mb) was greater than that (0.05/Mb) in *A. ipaensis*. The density of NAC genes in *A. hypogaea* was 0.06/Mb, which was approximately the average number between *A. duranensis* and *A. ipaensis*.

**Table 1:** NAC TF gene family members in wild *Arachis*

Gene symbol	Gene model name	Gene location	Length (aa)	MW (kDa)	Theoretical pI	Putative ***Arabidopsis*** orthologues	Closest genes	E-value	Othologous genes with known function
AdNAC1	Aradu.08GFU.1	Chr7:4217194..4220440	367	42.7	6.19	ANAC42		4e-85	
AdNAC2	Aradu.08TAH.1	Chr10:5997735..5999148	229	26.7	5.56	ANAC104/XND1		2e-90	
AdNAC3	Aradu.0MJ0X.1	Chr3:11724103..11725980	384	44.0	7.45	ANAC70		3e-157	
AdNAC4	Aradu.13D06.1	Chr1:100229649..100231496	396	45.3	6.86	ANAC35		1e-119	
AdNAC5	Aradu.15JI0.1	Chr8:28519479..28520527	150	16.7	8.69		ANAC90	1e-28	
AdNAC6	Aradu.15QQT.1	Chr1:17654260..17657374	135	32.8	9.35		ANAC14	3e-28	
AdNAC7	Aradu.1AJ4F.1	Chr7:46474022..46478416	350	40.4	6.93	ANAC33		5e-123	
AdNAC8	Aradu.215DG.1	Chr10:2443477..2446668	322	36.8	8.14	ANAC73		1e-114	
AdNAC9	Aradu.22647.1	Chr10:106757870..106759333	274	31.6	6.00	ANAC87		4e-100	
AdNAC10	Aradu.30S8W.1	Chr1:42645387..42650347	288	33.6	6.94	ANAC7/VND4		2e-103	
AdNAC11	Aradu.3R7A3.1	Chr6:99554879..99559186	481	53.7	5.05	ANAC44		3e-92	
AdNAC12	Aradu.46U1T.1	Chr6:8759633..8760991	251	28.0	6.37		ANAC28/TIP	5e-13	
AdNAC13	Aradu.47JQU.1	Chr8:49202066..49203551	321	36.3	8.99	ANAC100		3e-124	
AdNAC14	Aradu.4RJ0E.1	Chr6:90892652..90894340	355	40.2	9.35	ANAC47		7e-104	
AdNAC15	Aradu.58D1A.1	Chr8:48242228..48244188	193	22.8	10.13		ANAC83	4e-63	
AdNAC16	Aradu.5D5JN.1	Chr10:66508689..66512014	592	67.0	5.52	ANAC9		1e-94	
AdNAC17	Aradu.60 U13.1	Chr10:95255502..95259031	374	41.6	8.64	ANAC38		3e-111	
AdNAC18	Aradu.66XRP.1	Chr3:118432883..118434275	318	34.9	7.79	ANAC25		1e-107	
AdNAC19	Aradu.6H4PP.1	Chr10:84012608..84013897	230	26.1	5.23		ANAC104/XND1		
AdNAC20	Aradu.79PL2.1	Chr3:106298423..106299692	211	23.6	9.45	ANAC41		4e-64	
AdNAC21	Aradu.7NI41.1	Chr3:20188210..20192587	286	32.6	8.19	ANAC73		2e-110	
AdNAC22	Aradu.7X5EV.1	Chr8:36760639..36761970	328	36.3	8.67	ANAC2		9e-121	
AdNAC23	Aradu.ZT2TE.1	Chr5:108980829..108983109	341	39.3	6.30	ANAC7/VND5		2e-114	
AdNAC24	Aradu.8Q7DY.1	Chr10:100727698..100729562	313	36.1	8.50	ANAC94		3e-87	
AdNAC25	Aradu.9FF24.1	Chr9:104552010..104554828	583	65.2	4.72	ANAC53		2e-88	
AdNAC26	Aradu.9T4H8.1	Chr3:129693427..129694223	228	25.8	4.95	ANAC104/XND1		3e-77	
AdNAC27	Aradu.9Y6NH.1	Chr3:126203898..126205566	432	48.2	6.86	ANAC94		7e-96	
AdNAC28	Aradu.AF9FZ.1	Chr3:11453829..11456527	373	43.2	5.89	ANAC7/VND5		6e-118	
AdNAC29	Aradu.B5XXI.1	Chr5:89010001..89014271	405	50.5	6.97	ANAC75		2e-149	
AdNAC30	Aradu.BPK98.1	Chr2:5418916..5424040	463	52.5	5.81		ANAC9	2e-50	
AdNAC31	Aradu.BS3JU.1	Chr8:32409380..32411511	300	34.1	8.55	ANAC73		2e-116	
AdNAC32	Aradu.C1Q0A.1	Chr8:28596303..28598519	372	41.9	7.53	ANAC40/NTL8		4e-99	
AdNAC33	Aradu.DII8L.1	Chr4:123542703..123544293	245	27.8	9.00	ANAC83		1e-102	
AdNAC34	Aradu.DQR3M.1	Chr10:3280639..3282098	347	39.5	6.81	ANAC25		6e-96	
AdNAC35	Aradu.EP425.1	Chr10:83305166..83307784	367	40.4	4.75	ANAC82		7e-91	
AdNAC36	Aradu.ETZ8K.1	Chr5:5429577..5431170	356	40.3	5.20	ANAC71		2e-109	
AdNAC37	Aradu.F2DT2.1	Chr8:26149830..26151706	360	41.0	5.95	ANAC25		1e-78	
AdNAC38	Aradu.F48KW.1	Chr9:118016952..118020856	557	63.0	4.58	ANAC16		1e-131	
AdNAC39	Aradu.F6Z4G.1	Chr1:105899702..105901040	330	37.2	8.16	ANAC100		7e-139	
AdNAC40	Aradu.F8VRL.1	Chr3:30065645..30068198	382	43.0	7.69	ANAC75		2e-123	
AdNAC41	Aradu.H2YS3.1	Chr6:113116..11132912	226	26.1	7.22		ANAC74	2e-61	
AdNAC42	Aradu.H5KV7.1	Chr10:101199649..101202008	317	36.6	4.96	ANAC79/ANAC80/ATNAC4		4e-85	
AdNAC43	Aradu.JV7AK.1	Chr5:103968982..103973619	451	50.9	5.21	ANAC8		4e-150	
AdNAC44	Aradu.JZK1S.1	Chr7:10652666..10652977	95	11.0	9.22		ANAC14	5e-06	
AdNAC45	Aradu.K2UJH.1	Chr7:28089666..28093564	681	75.4	4.57	ANAC14		7e-58	
AdNAC46	Aradu.KF8UQ.1	Chr3:111110289..111111452	184	21.1	5.66		ANAC104/XND1	3e-36	
AdNAC47	Aradu.L3QY1.1	Chr1:27306711..27309985	331	37.2	4.82	ANAC71		2e-97	
AdNAC48	Aradu.L6S7Y.1	Chr2:14425633..14431626	246	27.8	5.89	ANAC74		1e-81	
AdNAC49	Aradu.L8SVN.1	Chr3:126544444..126545867	286	32.8	8.32	ANAC2		2e-109	
AdNAC50	Aradu.LG4RX.1	Chr6:95430437..95431819	218	25.2	9.18		ANAC83	3e-21	
AdNAC51	Aradu.LZ0D8.1	Chr7:70232857..70235212	351	40.3	8.49	ANAC42		2e-86	
AdNAC52	Aradu.M7213.1	Chr5:11521321..11523020	324	37.4	5.38	ANAC1		2e-140	
AdNAC53	Aradu.M8PFR.1	Chr9:104514608..104520535	425	47.9	8.63	ANAC52		1e-86	
AdNAC54	Aradu.M9GL4.1	Chr5:50856073..50857364	308	35.3	7.68	ANAC2		8e-118	
AdNAC55	Aradu.N8F6V.1	Chr5:82433539..82435658	363	40.2	9.35	ANAC040		3e-81	
AdNAC56	Aradu.N8MU8.1	Chr5:93368562..93371821	362	41.0	7.21	ANAC58		2e-125	
AdNAC57	Aradu.NEU1C.1	Chr2:5363506..5368149	255	29.6	5.41		ANAC14	3e-39	
AdNAC58	Aradu.R9F07.1	Chr2:4630145..4632702	463	51.1	6.05	ANAC66		1e-104	
AdNAC59	Aradu.RP61F.1	Chr6:110760391..110763962	306	34.5	5.60		ANAC7	1e-19	
AdNAC60	Aradu.RRT20.1	Chr5:13469204..13471956	394	45.6	6.98	ANAC7		3e-116	
AdNAC61	Aradu.S13QQ.1	Chr6:25318703..25322833	344	39.3	6.33	ANAC25		2e-83	
AdNAC62	Aradu.TGA11.1	Chr3:7357966..7359851	315	36.3	6.62	ANAC36		2e-117	
AdNAC63	Aradu.TI0Z7.1	Chr7:34924555..34930380	322	36.4	7.57	ANAC1		1e-124	
AdNAC64	Aradu.U974Q.1	Chr3:122754747..122758369	633	71.7	6.34	ANAC28		2e-141	
AdNAC65	Aradu.USH95.1	Chr8:38011875..38013744	369	40.7	7.22	ANAC100		4e-90	
AdNAC66	Aradu.UXN6T.1	Chr8:46083445..46085088	304	35.1	6.42	ANAC032		3e-96	
AdNAC67	Aradu.VUC67.1	Chr9:118496634..118500190	321	37.5	6.76	ANAC7		2e-113	
AdNAC68	Aradu.W3GLH.1	Chr7:15758174..15764123	679	77.4	5.43	ANAC28		8e-167	
AdNAC69	Aradu.WIT0W.1	Chr7:44242604..44246557	346	39.5	5.05	ANAC20		2e-96	
AdNAC70	Aradu.WS3DN.1	Chr6:71790233..71791501	213	24.4	5.39	ANAC90		1e-45	
AdNAC71	Aradu.XE8WZ.1	Chr3:111523591..111525548	300	33.0	5.09		ANAC103	2e-50	
AdNAC72	Aradu.XJF09.1	Chr5:86074509..86078409	396	43.6	6.47	ANAC44		2e-87	
AdNAC73	Aradu.XQ4VP.1	Chr5:98563335..98567633	167	19.5	8.87	ANAC57		4e-90	
AdNAC74	Aradu.Y1DM8.1	Chr6:90691784..90693411	396	44.5	6.21	ANAC46		5e-111	
AdNAC75	Aradu.Y9JNS.1	Chr8:4371901..4373364	369	41.6	7.84	ANAC100		5e-75	
AdNAC76	Aradu.YFQ3P.1	Chr3:110319904..110321231	260	29.8	7.71	ANAC102		7e-113	
AdNAC77	Aradu.YIQ80.1	Chr8:36879860..36881784	349	39.1	8.20	ANAC19		4e-120	AhNAC4 (HM776131) [29]
dNAC78	Aradu.YXW0Z.1	Chr3:119828022..119831252	342	38.6	8.66	ANAC10/SND3		1e-120	
AdNAC79	Aradu.Z4K97.1	Chr9:120442436..120446530	493	55.0	5.11	ANAC8		3e-127	
AdNAC80	Aradu.Z5H58.1	Chr3:25915995..25917258	335	37.7	6.61	ANAC3		3e-110	
AdNAC81	Aradu.Z9Y3J.1	Chr4:117994993..117996740	330	38.0	5.72	ANAC100		6e-89	
AiNAC1	Araip.0550R.1	Chr3:197325..198893	330	37.5	9.04	ANAC100		3e-123	
AiNAC2	Araip.0S3JI.1	Chr5:139720050..139724356	358	40.3	6.40	ANAC75		1e-143	
AiNAC3	Araip.1N7IP.1	Chr10:4025791..4028779	324	36.9	8.41	ANAC73		8e-115	
AiNAC4	Araip.1Z0SD.1	Chr3:33051241..33054714	381	42.7	7.33	ANAC75		1e-123	
AiNAC5	Araip.2BL8E.1	Chr8:5815169..5817042	320	36.2	9.08	ANAC40		9e-84	
AiNAC6	Araip.2W5R5.1	Chr10:93270958..93274136	592	67.0	5.46	ANAC14		2e-91	
AiNAC7	Araip.310 T2.1	Chr7:118782789..118785019	301	33.7	6.54	ANAC32		2e-108	
AiNAC8	Araip.31EFM.1	Chr5:142199068..142203628	410	45.4	5.76	ANAC85		2e-85	
AiNAC9	Araip.333QY.1	Chr3:28574038..28575297	335	37.7	6.61	ANAC19		3e-109	AhNAC3 (EU755022) [28]
AiNAC10	Araip.4A49L.1	Chr5:144854742..144856564	285	32.5	6.97	ANAC40		9e-74	
AiNAC11	Araip.6CI1F	Chr10:127061923..127064519	391	45.5	5.07	ANAC79		5e-73	
AiNAC12	Araip.609WS.1	Chr5:134972759..134976087	363	41.1	7.21	ANAC58		2e-123	
AiNAC13	Araip.64GCN.1	Chr5:45189298..45190788	308	35.4	7.69	ANAC25		1e-82	
AiNAC14	Araip.67R8V	Chr6:123068423..123072685	471	52.6	5.11	ANAC44		3e-93	
AiNAC15	Araip.6Y0GY	Chr7:4085598..4089132	280	32.7	5.49	ANAC042		2e-27	
AiNAC16	Araip.714GL	Chr9:138000623..138005210	559	63.3	4.57	ANAC016		8e-134	
AiNAC17	Araip.71CS3	Chr10:107295010..107298155	367	40.4	4.72	ANAC103		6e-87	
AiNAC18	Araip.77ISR	Chr2:17820382..17822891	257	28.9	8.49	ANAC74		1e-63	
AiNAC19	Araip.78PTT	Chr8:24667634..24669998	366	40.4	7.22	ANAC100		8e-90	
AiNAC20	Araip.79TDF	Chr7:16494343..16499499	678	77.3	5.51	ANAC086		1e-115	
AiNAC21	Araip.7L9YW	Chr5:110392178..110396183	497	56.8	5.05	ANAC8		6e-121	
AiNAC22	Araip.8NR3H	Chr3:111878869..111880200	260	29.9	7.09	ANAC032		8e-98	
AiNAC23	Araip.92BTQ	Chr10:108502499..108503880	213	24.3	5.25		ANAC104	3e-55	
AiNAC24	Araip.9BR1Z	Chr3:112774516..112775399	202	23.2	5.19		ANAC104	3e-47	
AiNAC25	Araip.9MG9F	Chr3:22778530..22786094	321	36.6	7.37	ANAC75		1e-95	
AiNAC26	Araip.9N5S4	Chr3:14030155..14031366	338	39.0	5.91	ANAC7		4e-116	
AiNAC27	Araip.9W6SR	Chr3:123433544..123436914	633	71.6	6.09	ANAC86		2e-113	
AiNAC28	Araip.A6QWC	Chr2:6650205..6654171	481	54.6	5.61		ANAC14	3e-51	
AiNAC29	Araip.AVV74	Chr4:127912708..127914469	330	38.1	5.82	ANAC100		3e-88	
AiNAC30	Araip.AWF0A	Chr7:95692641..95694174	354	39.7	7.39	ANAC100		6e-64	
AiNAC31	Araip.CC7W1	Chr2:5953755..5956019	461	51.0	6.05	ANAC33		1e-90	
AiNAC32	Araip.D25HB	Chr8:70326624..70335981	390	44.0	6.27	ANAC58		3e-66	
AiNAC33	Araip.D7N1Q	Chr6:34266288..34267077	142	16.1	9.78		ANAC25	2e-60	
AiNAC34	Araip.DEH65	Chr5:14567806..14570484	343	39.8	6.24	ANAC7		7e-117	
AiNAC35	Araip.DL86S	Chr8:21485570..21487407	349	39.1	8.20	ANAC19		2e-120	AhNAC2 (EU755023) [27]
AiNAC36	Araip.DR280	Chr10:126350971..126352623	304	34.7	6.66	ANAC94		3e-65	
AiNAC37	Araip.E0NQ0	Chr9:131326018..131330122	494	55.1	5.05		ANAC8	5e-132	
AiNAC38	Araip.F5AGL	Chr1:114413161..114414326	330	37.2	8.16	ANAC100		3e-137	
AiNAC39	Araip.F8I62	Chr9:127070762..127076419	425	47.9	8.63	ANAC51		1e-87	
AiNAC40	Araip.FR0NA	Chr8:128783925..128786313	116	13.5	10.25		ANAC83	1e-24	
AiNAC41	Araip.FRS32	Chr6:22593548..22597918	259	29.9	6.70	ANAC74		4e-92	
AiNAC42	Araip.G3ZLR	Chr5:12372525..12374201	321	36.7	5.21	ANAC7		2e-97	
AiNAC43	Araip.G88UP	Chr3:120758733..120762390	330	37.3	8.47	ANAC75		4e-97	
AiNAC44	Araip.HIJ9F	Chr6:113252685..113254364	418	47.1	9.44	ANAC47		8e-98	
AiNAC45	Araip.HYM8C	Chr6:113117048..113118424	395	44.5	6.21	ANAC46		4e-111	
AiNAC46	Araip.I60BC	Chr8:5732262..5733688	234	26.3	6.09	ANAC90		3e-64	
AiNAC47	Araip.I6LH9	Chr8:126952889..126954575	318	36.7	6.01	ANAC32		2e-96	
AiNAC48	Araip.J93FI	Chr8:10780467..10782598	294	33.5	8.82	ANAC75		2e-98	
AiNAC49	Araip.J9WH5	Chr3:14391485..14393315	375	42.5	7.28	ANAC70		2e-159	
AiNAC50	Araip.KI83M	Chr5:144883533..144885223	306	34.4	5.48	ANAC40		6e-83	
AiNAC51	Araip.KM0ZG	Chr3:130519333..130520361	143	16.6	7.84		ANAC104	1e-57	
AiNAC52	Araip.KP5QZ	Chr3:119580802..119582423	362	40.1	8.83	ANAC25		2e-111	
AiNAC53	Araip.ZUP60	Chr8:105359001..105363289	350	40.5	6.73	ANAC33		4e-125	
AiNAC54	Araip.Z57SD	Chr9:127100454..127103707	593	66.4	4.66	ANAC2		6e-82	
AiNAC55	Araip.L222I	Chr3:127136414..127138105	423	47.3	6.49	ANAC94		8e-97	
AiNAC56	Araip.MQD5S	Chr2:6609921..6613742	289	33.1	5.65		ANAC14	1e-39	
AiNAC57	Araip.NB7HU	Chr8:21185319..21187122	332	36.9	8.67	ANAC25		2e-102	
AiNAC58	Araip.NL359	Chr5:126310932..126316255	255	29.4	5.16	ANAC86		1e-78	
AiNAC59	Araip.PNX61	Chr6:135601578..135605205	308	34.8	5.61		ANAC96	8e-19	
AiNAC60	Araip.PT231	Chr3:113538059..113544219	343	38.0	5.85	ANAC103		1e-58	
AiNAC61	Araip.PW8UQ	Chr3:10649536..10655226	256	29.8	5.83	ANAC36		5e-98	
AiNAC62	Araip.PX0QP	Chr7:29146174..29149324	573	63.3	4.59		ANAC14	1e-28	
AiNAC63	Araip.Q1JTJ	Chr1:50655721..50661455	377	43.5	6.24	ANAC7		2e-133	
AiNAC64	Araip.Q3R6H	Chr8:3508743..3510623	360	41.0	5.95	ANAC25		2e-78	
AiNAC65	Araip.QS7JY	Chr9:136867581..136871254	291	34.1	6.97	ANAC7		1e-83	
AiNAC66	Araip.R0657	Chr3:107857753..107858791	211	23.7	9.45	ANAC83		4e-63	
AiNAC67	Araip.T6ICI	Chr3:127434557..127435546	286	32.8	8.04	ANAC25		7e-78	
AiNAC68	Araip.TL0B5	Chr5:15820769..15822869	234	26.7	5.45		ANAC62	7e-20	
AiNAC69	Araip.U9RGH	Chr1:124432208..124434079	403	46.1	6.86	ANAC35		1e-116	
AiNAC70	Araip.UA0W9	Chr10:133594504..133595933	277	31.8	6.32	ANAC87		9e-100	
AiNAC71	Araip.WV14F	Chr8:79712569..79714789	342	39.4	8.70	ANAC42		4e-87	
AiNAC72	Araip.X2KK1	Chr5:5593161..5595535	352	39.9	5.26	ANAC71		3e-108	
AiNAC73	Araip.XJ3T4	Chr10:118962456..118966247	342	37.6	7.70	ANAC38		4e-82	
AiNAC74	Araip.XJX1I	Chr8:97499988..97504182	319	36.9	5.64	ANAC20		3e-96	
AiNAC75	Araip.XK9AB	Chr1:33384496..33387453	277	31.3	5.37	ANAC71		5e-99	
AiNAC76	Araip.XQA0A	Chr5:149488712..149490936	339	39.1	6.30	ANAC7		1e-113	
AiNAC77	Araip.XT8UZ	Chr10:4890767..4892438	365	41.6	6.43	ANAC25		2e-93	
AiNAC78	Araip.ZX5IX	Chr6:7136977..7138554	259	29.1	6.27		ANAC62	3e-10	
AiNAC79	Araip.YS3WM	Chr10:10330806..10332162	229	26.7	5.69	ANAC104		1e-90	

Owing to the lack of a designated standard annotation for NAC genes in *Arachis*, we named these genes *AdNAC1-AdNAC81* and *AiNAC*1-*AiNAC79*. The NAC genes identified in *A.duranensis* and *A.ipaensis* encoded proteins ranging from 95 to 681 amino acid (aa) residues in length, with an average of 345 aa, and the molecular weights (MWs) varied from 11 kDa to 77.4 kDa. The isoelectric points (pIs) of the predicted proteins ranged from 4.57 to 10.25. Detailed information on the NAC genes in *A.duranensis* and *A. ipaensis* is provided in Table 1, including gene location, and putative *Arabidopsis* orthologues.

**Fig. 1:**
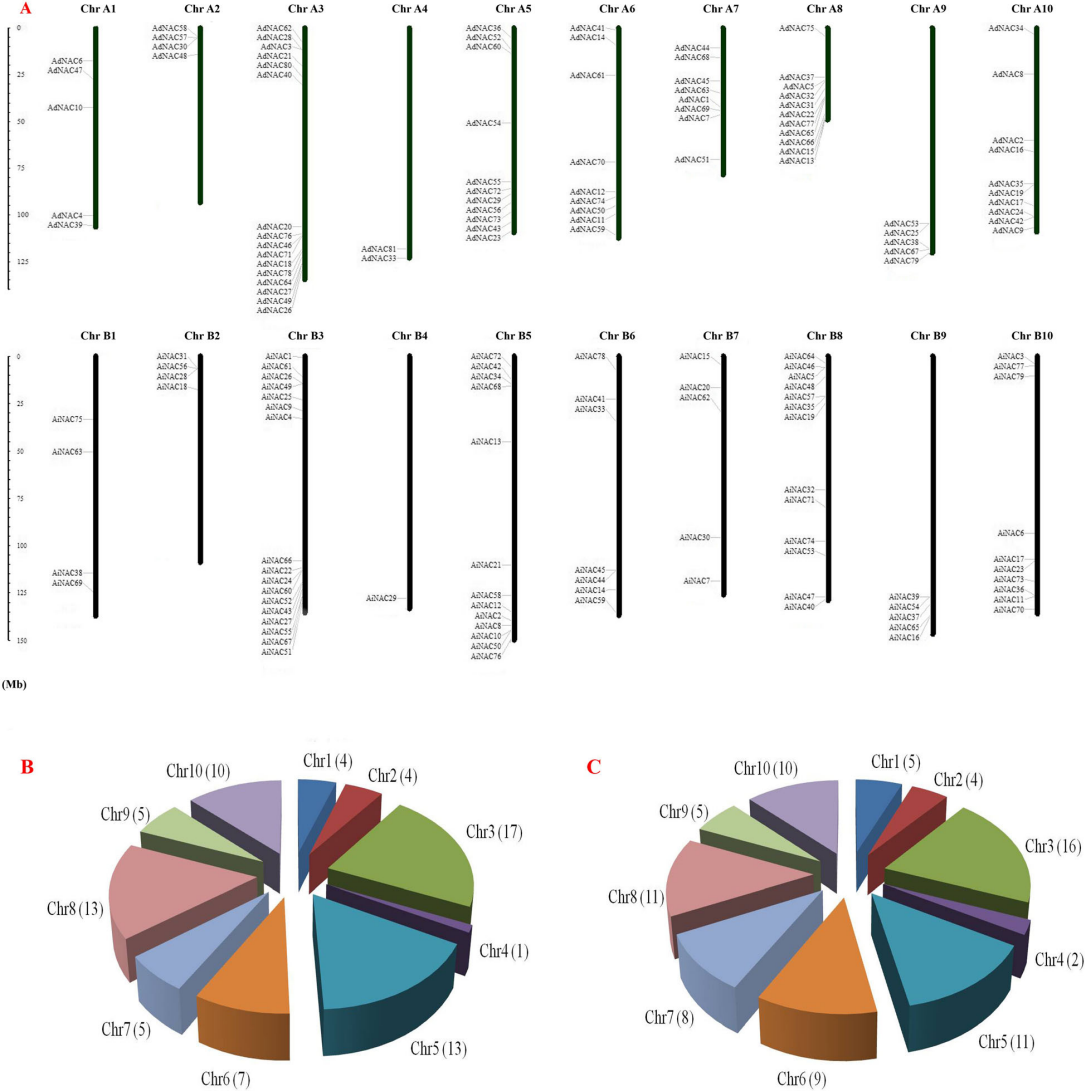
Chromosome location of NAC genes on each chromosomes of *A. duranensis* and *A. ipaensis*. **a** Diagrammatic sketch of distribution of NAC genes on each chromosome (black bars). The approximate location of each NAC gene are shown at the left side of each chromosome. **b-c** The NAC genes’ distribution on each chromosome. The number of NAC genes on each chromosome is shown in brackets

As shown in Fig. 1, the *AdNAC* and *AiNAC* genes are distributed non-randomly across 10 chromosomes of *A. duranensis* (A genome) and *A. ipaensis* (B genome). In these species, chromosome A3 contained the most NAC genes (16), while chromosome A4 contained the fewest NAC genes (2) (Fig. 1b). In *A. ipaensis*, 17 genes were distributed on chromosome B3, whereas only one NAC gene was found on chromosome B4 (Fig. 1c).

### NAC orthologues are located at syntenic loci within the *A. duranensis* and *A. ipaensis* genomes

**Fig. 2:**
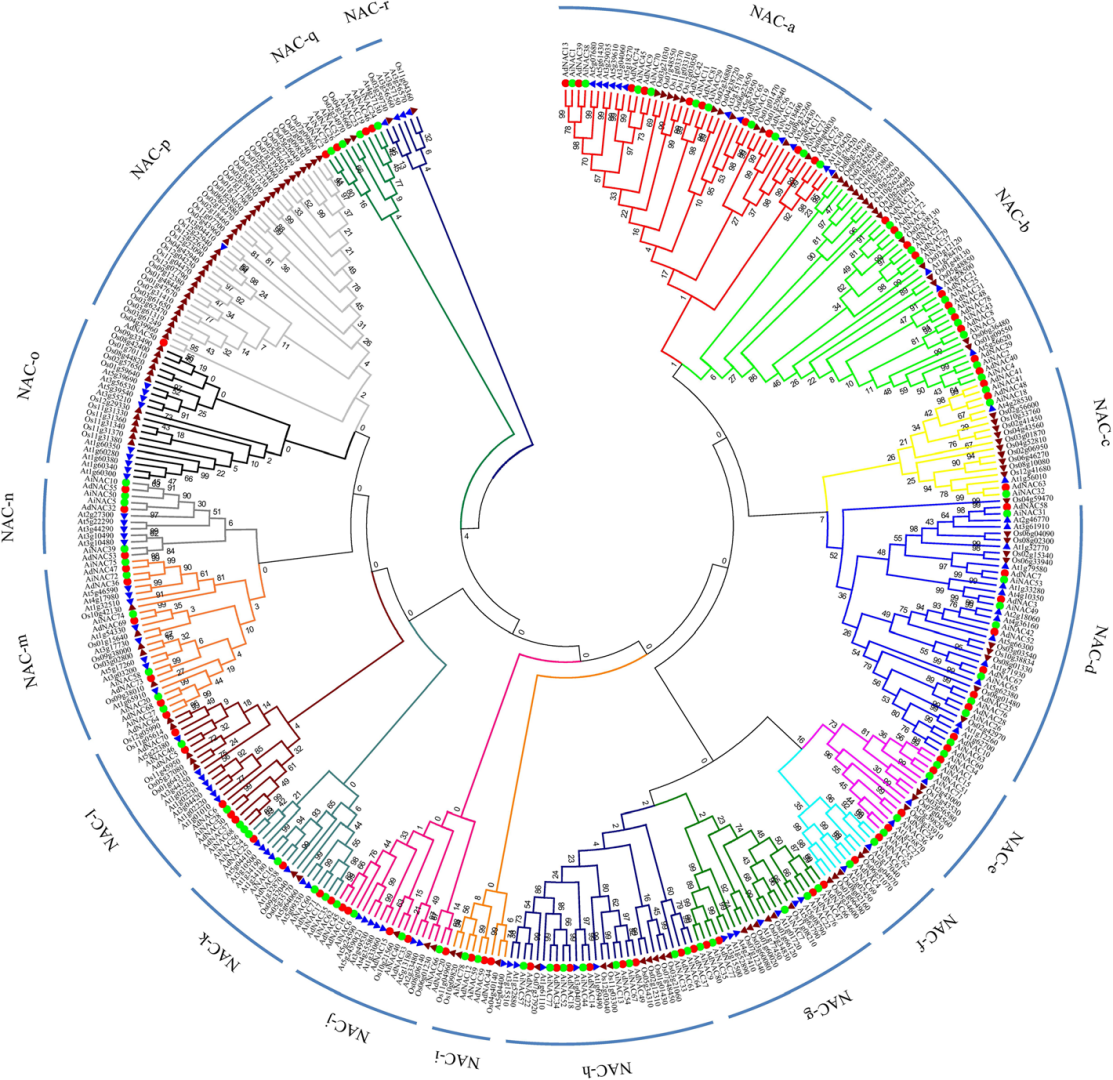
Phylogenetic analysis of NAC proteins among *Arachis*, *Arabidopsis* and rice. Multiple sequence alignment of NAC proteins was performed using ClustalW. The phylogenetic tree was constructed via MEGA 6.0 using NJ method with 1000 bootstrap replicates. The tree was divided these NAC proteins into 18 subgroups, designated NAC-a to NAC-r. NAC protein members of *A. duranensis*, *A. ipaensis*, *Arabidopsis* and rice are distinguished by red circles, green circles, blue triangles, and brown triangles, respectively

We detected 51 orthologous gene pairs according to the phylogenetic relationships of the *AdNAC* and *AiNAC* genes (Fig. 2, Table 2) and further confirmed through their chromosomal location and gene structure. Among these orthologous gene pairs, 46 were located at syntenic loci on the *A. duranensis* and *A. ipaensis* chromosomes (Fig. 1a). However, the location of 9 *AdNAC* genes did not correspond to the location of their orthologous gene in *A. ipaensis*. For example, *AdNAC7* located on chromosome A7, while its orthologous gene in *A. ipaensis, AiNAC53*, is located on chromosome B8. This finding suggested that large chromosomal rearrangement in the diploid peanut genomes has occurred. Moreover, gene pairs with low identity might result from different splicing patterns or premature stop codons that originated from the released incomplete genome draft [1].

**Table 2:** Putative orthologous gene pairs in *A. duranensis* and *A. ipaensis*

Gene pairs	Groups	Chromosome	CDS identity (%)	Protein identity (%)
AdNAC1-AiNAC15	IX-IX	7–7	62.33	73.17
AdNAC2-AiNAC79	IX-IX	10–10	96.73	99.13
AdNAC3-AiNAC49	VIII-VIII	3–3	96.09	96.35
AdNAC4-AiNAC69	IX-IX	1–1	95.51	96.30
AdNAC7-AiNAC53	VI-VI	7–8	97.68	97.43
AdNAC10-AiNAC63	X-X	1–1	79.17	73.47
AdNAC12-AiNAC78	VIII-VIII	6–6	93.89	85.71
AdNAC16-AiNAC6	V-V	10–10	98.67	99.16
AdNAC17-AiNAC73	III-III	10–10	90.02	88.27
AdNAC18-AiNAC52	V-V	3–3	84.50	86.74
AdNAC20-AiNAC66	I-I	3–3	96.68	98.58
AdNAC21-AiNAC25	IX-IX	3–3	89.14	84.78
AdNAC22-AiNAC57	I-I	8–8	95.67	94.58
AdNAC23-AiNAC76	X-X	5–5	93.51	95.07
AdNAC24-AiNAC36	III-III	10–10	86.73	84.76
AdNAC25-AiNAC54	II-II	9–9	94.42	94.01
AdNAC28-AiNAC26	VI-VI	3–3	87.92	87.77
AdNAC29-AiNAC2	VIII-VIII	5–5	76.79	74.10
AdNAC34-AiNAC77	XI-XI	10–10	91.48	88.28
AdNAC35-AiNAC17	XII-XII	10–10	98.64	98.91
AdNAC36-AiNAC72	XII-XII	5–5	97.76	97.19
AdNAC37-AiNAC64	III-III	8–8	98.89	99.17
AdNAC39-AiNAC38	III-III	1–1	98.39	99.39
AdNAC40-AiNAC4	VI-VI	3–3	97.46	96.60
AdNAC47-AiNAC75	XII-XII	1–1	82.03	80.36
AdNAC48-AiNAC41	VI-VI	2–6	61.05	55.21
AdNAC49-AiNAC67	XII-XII	3–3	95.76	98.60
AdNAC52-AiNAC42	XII-XII	5–5	94.43	92.97
AdNAC53-AiNAC39	II-II	9–9	98.54	98.82
AdNAC54-AiNAC13	VIII-VIII	5–5	97.08	99.35
AdNAC55-AiNAC10	III-III	5–5	69.45	43.90
AdNAC56-AiNAC12	II-II	5–5	96.01	95.91
AdNAC57-AiNAC56	XI-XI	2–2	85.78	80.97
AdNAC58-AiNAC31	I-I	2–2	97.00	97.85
AdNAC59-AiNAC59	III-III	6–6	97.70	97.08
AdNAC62-AiNAC61	I-I	3–3	79.21	79.05
AdNAC63-AiNAC32	XII-XII	7–8	76.35	68.29
AdNAC64-AiNAC27	XII-XII	3–3	97.04	97.79
AdNAC65-AiNAC19	IV-IV	8–8	97.82	97.57
AdNAC67-AiNAC65	X-X	9–9	83.21	84.80
AdNAC68-AiNAC20	XII-XII	7–7	98.61	97.94
AdNAC69-AiNAC74	XII-XII	7–8	80.24	76.57
AdNAC73-AiNAC58	XII-XII	5–5	65.10	58.43
AdNAC74-AiNAC45	I-I	6–6	98.99	98.74
AdNAC75-AiNAC30	IV-IV	8–7	89.36	85.87
AdNAC76-AiNAC22	X-X	3–3	98.19	99.23
AdNAC77-AiNAC35	I-I	8–8	97.32	99.71
AdNAC78-AiNAC43	IV-IV	3–3	93.50	94.07
AdNAC79-AiNAC37	V-V	9–9	97.04	95.98
AdNAC80-AiNAC9	I-I	3–3	98.80	99.40
AdNAC81-AiNAC29	IV-IV	4–4	97.32	96.97

### Phylogenetic analysis, gene structure and conserved motifs of *Arachis* NAC genes

To explore the relationships among the NACs of two wild *Arachis* species and predict their potential functions, the full-length NAC proteins from *A. duranensis* (Additional file 5), *A. ipaensis* (Additional file 5), *Arabidopsis* (dicot) (Additional file 6) and rice (monocot) (Additional file 7) were subjected to a multiple sequence alignment. The phylogenetic tree divided NACs from wild peanut into 18 distinct subgroups (NAC-a to NAC-r) along with their *Arabidopsis* and rice homologues (Fig. 2). In general, the *Arabidopsis*, rice and peanut NAC proteins were distributed uniformly in all subgroups. However, the NAC-o and NAC-r subgroups contained only *Arabidopsis* and rice NACs and no peanut NACs. Remarkably, the NAC-p subfamily included 36 rice NACs but only 1 *AdNAC* and 1 *Arabidopsis* NAC, while no rice NAC was found in the NAC-n subgroup. Another phylogenetic tree based on the conserved NAM domain is shown in Additional file 8.

**Fig. 3:**
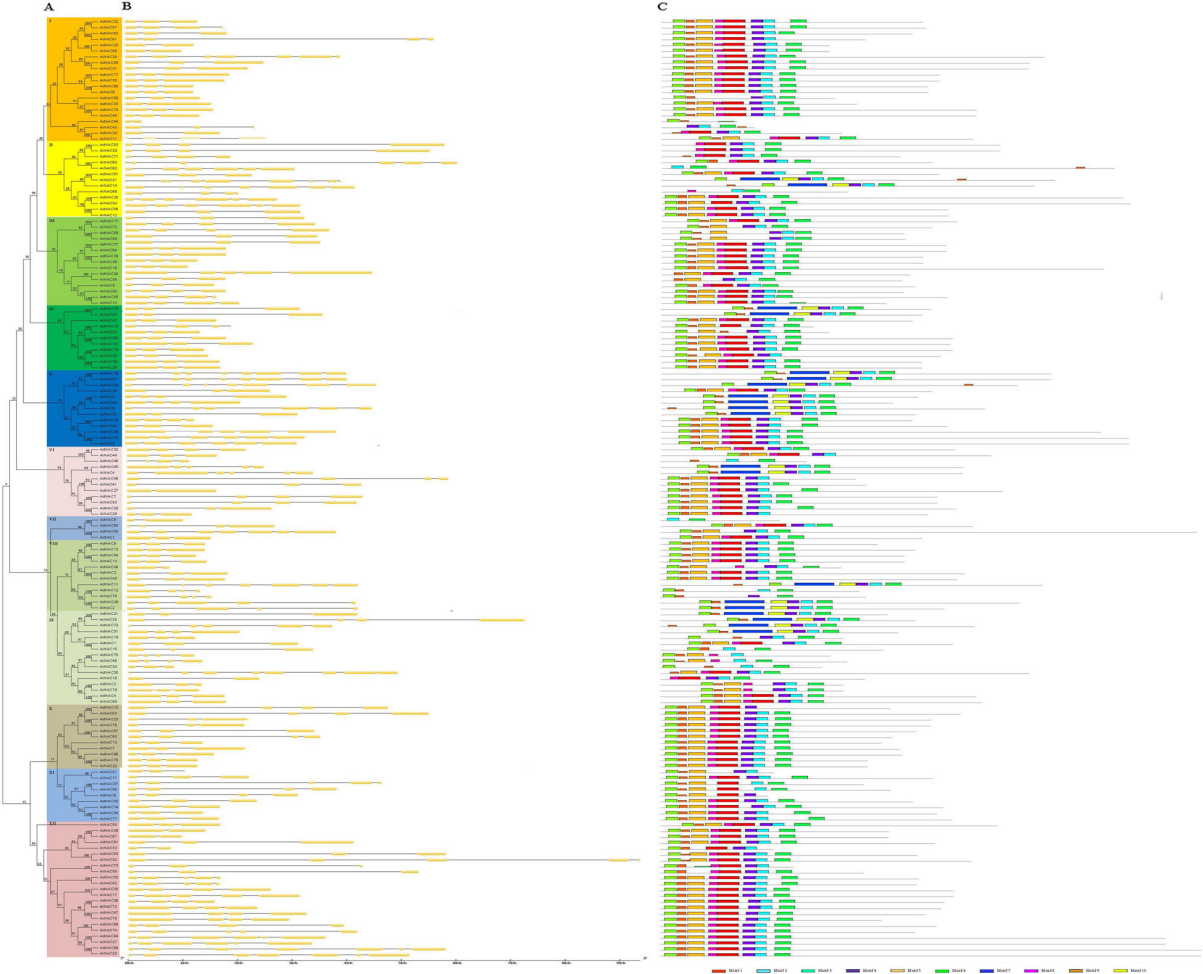
Gene structure and motif compositions of NAC genes from *A. duranensis* and *A. ipaensis*. **a** NAC proteins from two wild peanut were divided into twelve phylogenetic subgroups via MEGA 6.0 using NJ method with 1000 bootstrap replicates, designated as I to XII in different colour backgrounds. **b** Gene structure of peanut NAC genes was analysed using the online GSDS tool. The exons and introns are indicated by yellow boxes and black lines, respectively. The scale at bottom represents the sizes of exons and introns. **c** The distribution of conserved motifs within peanut NAC proteins was explored by MEME. Each motif is distinguished by a number in the coloured box. The black lines show the non-conserved sequences. Detailed information of each motif is listed in Additional file 10

To investigate the structural diversity of NAC genes, the exon/intron structure among the peanut NAC genes was analysed accompanying with their phylogenetic similarities (Fig. 3). All the NAC genes from *A.duranensis* and *A. ipaensis* were classified into twelve subfamilies (Fig. 3a). Commonly, orthologous genes from *A.duranensis* and *A. ipaensis* shared similar exon/intron structures including intron number and exon length, for example, *AdNAC80* and *AiNAC9* in subfamily I, *AdNAC59* and *AiNAC59* in subfamily III, while *AdNAC81* and *AiNAC29* in subfamily IV (Additional file 9). Gene structural analysis indicated that the intron distribution within the peanut NAC genes was diverse and varied from 1 to 9 (Fig. 3b). In general, most of the *NAC*s contained 2–3 introns; for instance, 77 genes contained 2 introns, and 43 genes contained 3 introns.

To determine the diversification of NAC genes further, putative motifs were predicted, and ten conserved motifs within the *Arachis* NAC proteins were analysed (Additional file 10). As expected, the motif compositions among the closely related members were common. For instance, the majority of NAC proteins in subfamily XII contained 8 motifs. Notably, most of the predicted motifs were located in the N-terminal region of the NAC domain, which indicated that the N-terminal region was critical for the function of NAC genes (Fig. 3c).

### *Cis*-acting elements in the promoter region of *Arachis* NAC genes

NAC genes play critical roles in the response to numerous stresses. The putative *cis*-acting elements involved in the response to biotic or abiotic stresses within the 2.5-kb sequence upstream of the start codon (ATG) (Additional file 11) were analysed. As shown in Additional files 12, 14 known stress-related *cis*-acting elements within the promoters of these NAC genes were identified. The numbers of *cis*-acting factors ranged from 0 to 10, and there were 10 different types of *cis*-acting elements within the promoter region of *AdNAC34*, *AdNAC30*, and *AiNAC30*. Only promoters of 4 genes (*AdNAC7*, *AdNAC15*, *AdNAC44*, and *AiNAC15*) contained the TC-rich motif, which is involved in defence and stress responses [42]. Of the 160 promoters, 133 had 1–9 copies of AREs, which are essential for anaerobic induction [43]. The CGTCA motif, which is involved in stress responses mediated by the hormone methyl jasmonate (MeJA) [44], was present within 93 genes. Several other elements related to abiotic and biotic stress responses, such as TGA, W1, HSE, and LTR elements, were also found in these 2.5-kb promoter regions. These results indicated that NAC genes were transcriptionally regulated in response to biotic and abiotic stresses.

### Expression profile of NAC genes in different tissues of *A. duranensis* and *A. ipaensis*

**Fig. 4:**
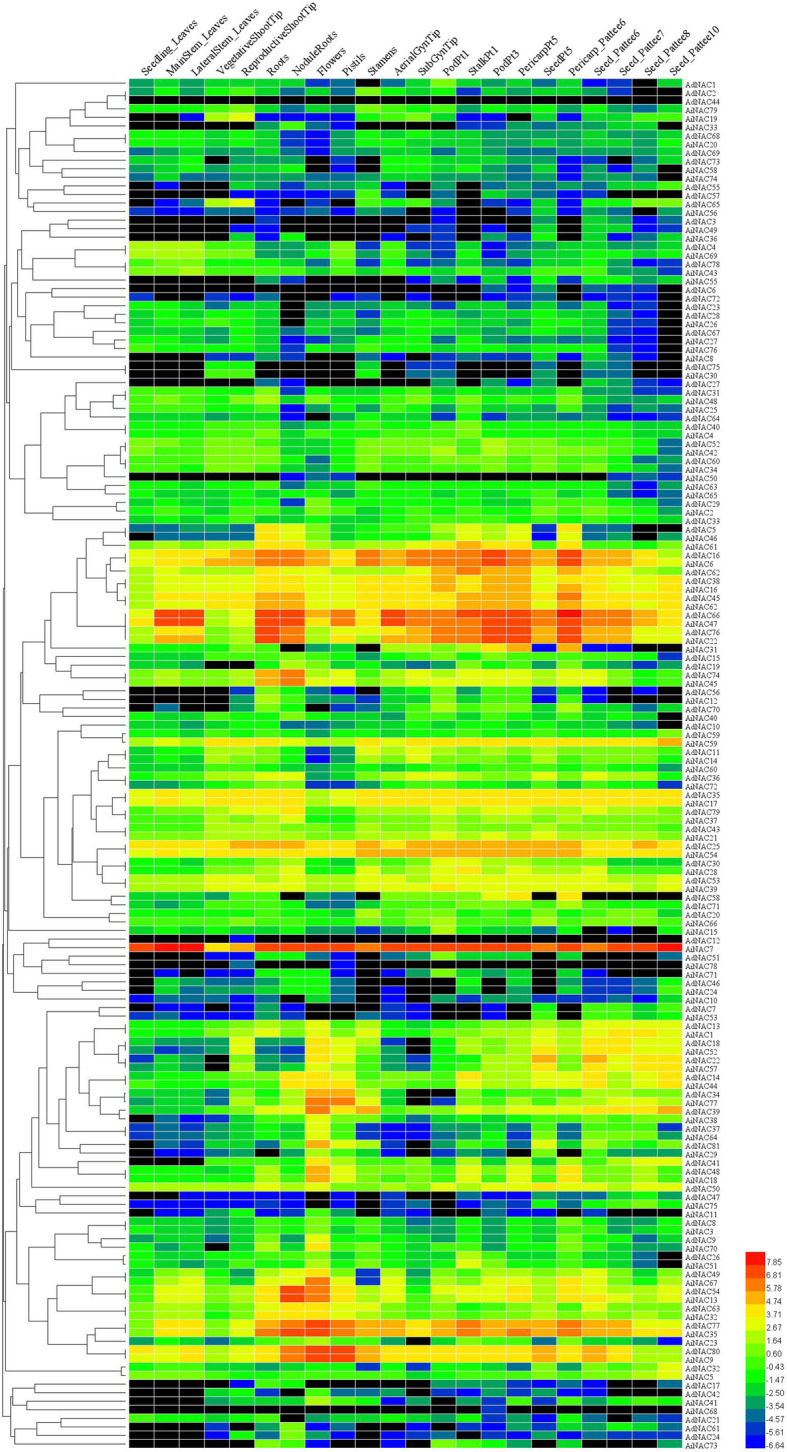
Tissue-specific expression pattern of NAC genes in 22 different tissues and development of two wild peanuts. The illumina RNA-seq data from Clevenger et al. [36] were reanalysed, the average FPKM values were log2 transformed and a heatmap was obtained using HemI. The expression intensity shows in different colours (red, high expression; green, low expression; black, no expression). The bar at the top represents 22 different tissues and developmental stages; NAC genes from *A. duranensis* and *A. ipaensis* are shown on the right

To investigate the tissue-specific expression profile of NAC genes, we utilized transcriptome data from Clevenger et al. [36]. The examined 22 tissues encompassed nearly all tissues and developmental stages. As shown in Fig. 4, there was no detection of *AdNAC44* expression in any of the 22 tissues. Twenty-three NAC genes were expressed at a relatively high level in the 22 tissues. Among these 23 genes, *AiNAC7* exhibited relatively high expression levels in all 22 tissues, while its homologue *AdNAC12* was expressed only in reproductive shoot tip tissue. The genes with the same expression patterns, for example, *AdNAC16* and *AiNAC6*, were classified into the same group (group V, Fig. 3). Moreover, some NAC genes displayed tissue-specific or preferential expression patterns. For example, *AdNAC58* was not expressed in the seeds, pistils or stamens. This tissue-specific expression data analysis could ultimately help determine the locations of the regulatory function of NAC genes.

### Mining NAC genes involved in the response to salt and drought stresses

**Fig. 5:**
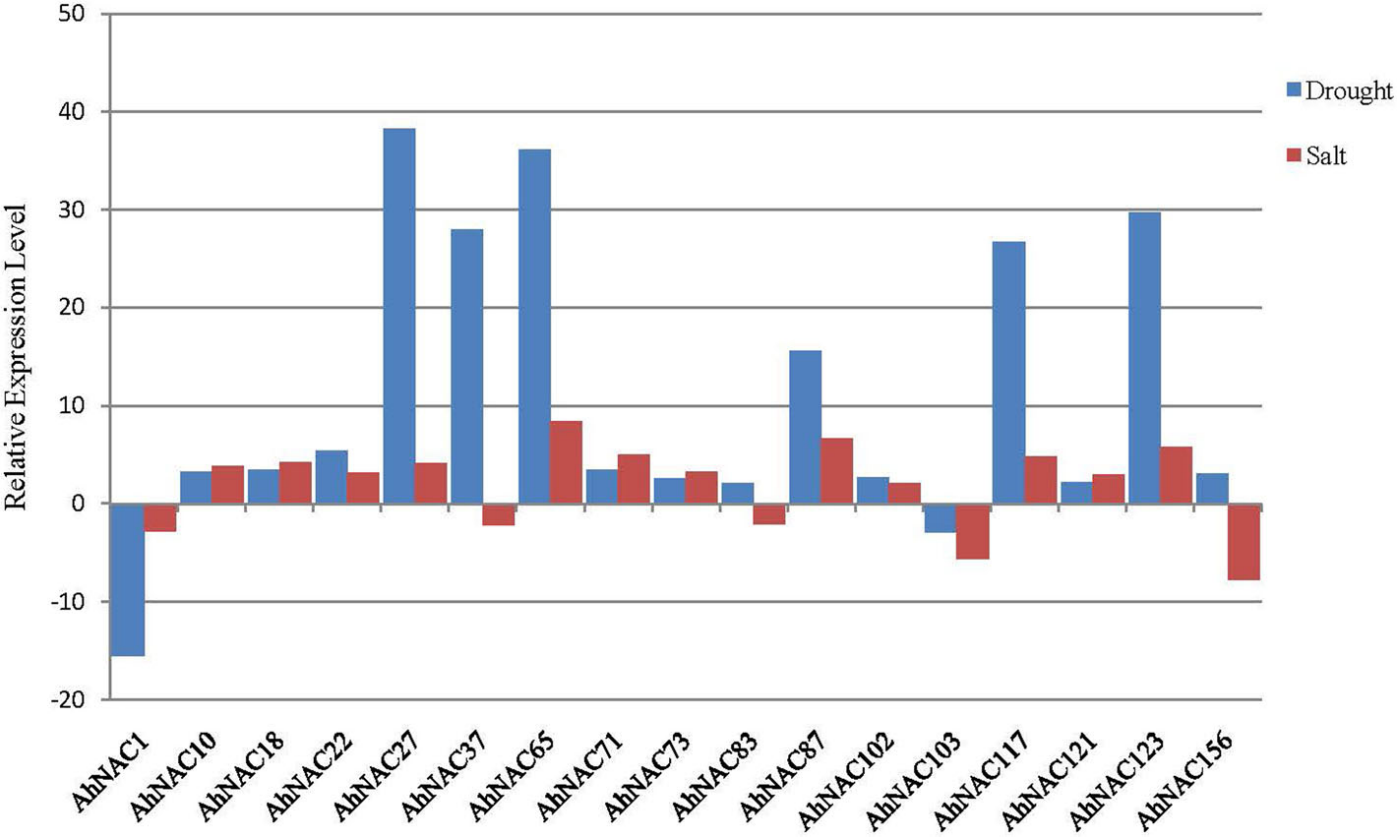
Expression patterns of AhNAC genes under drought and salt stresses based on RNA-seq data. The Y-axis indicates the relative expression level. The X-axis represnts the genes whose expression was upregulated or downregulated more than 2-fold under both salt and drought treatments in cultivated peanut

Many NAC genes are considered to be abiotic stresse-responsive genes. To explore NAC genes involved in the response to salt and/or drought stresses, we analysed the published transcriptome sequencing results of cultivated peanut under salt [39] and drought [37] treatments. Under salt treatment, the expression level of 28 genes was upregulated by 2-fold, whereas the expression of 15 genes was downregulated more than 2-fold. The expression of 8 genes was significantly upregulated more than 5-fold, and the greatest expression reached 17-fold, and the expression of 6 genes was downregulated more than 5-fold (Fig. 5, Additional file 13). Under drought treatment, the expression of 30 genes was up-regulated more than 2-fold, the expression of 9 genes was up-regulated more than 10-fold, and the greatest expression reached 38-fold. The expression of 13 genes was down-regulated more than 2-fold, and the greatest expression reached 15-fold (Fig. 5, Additional file 14). The expression of 17 genes was found to be responsive to both salt and drought stresses. Four genes (*AhNAC1*, *AhNAC37*, *AhNAC83* and *AhNAC156*) displayed the opposite response to salt and drought stresses (Fig. 5). Information concerning these NAC genes from cultivated *A. hypogaea* is listed in Additional file 3. These observations indicated that some of the NAC proteins may function in multiple stress responses.

### RT-qPCR of NAC genes under salt and drought stresses in cultivated peanut

**Fig. 6:**
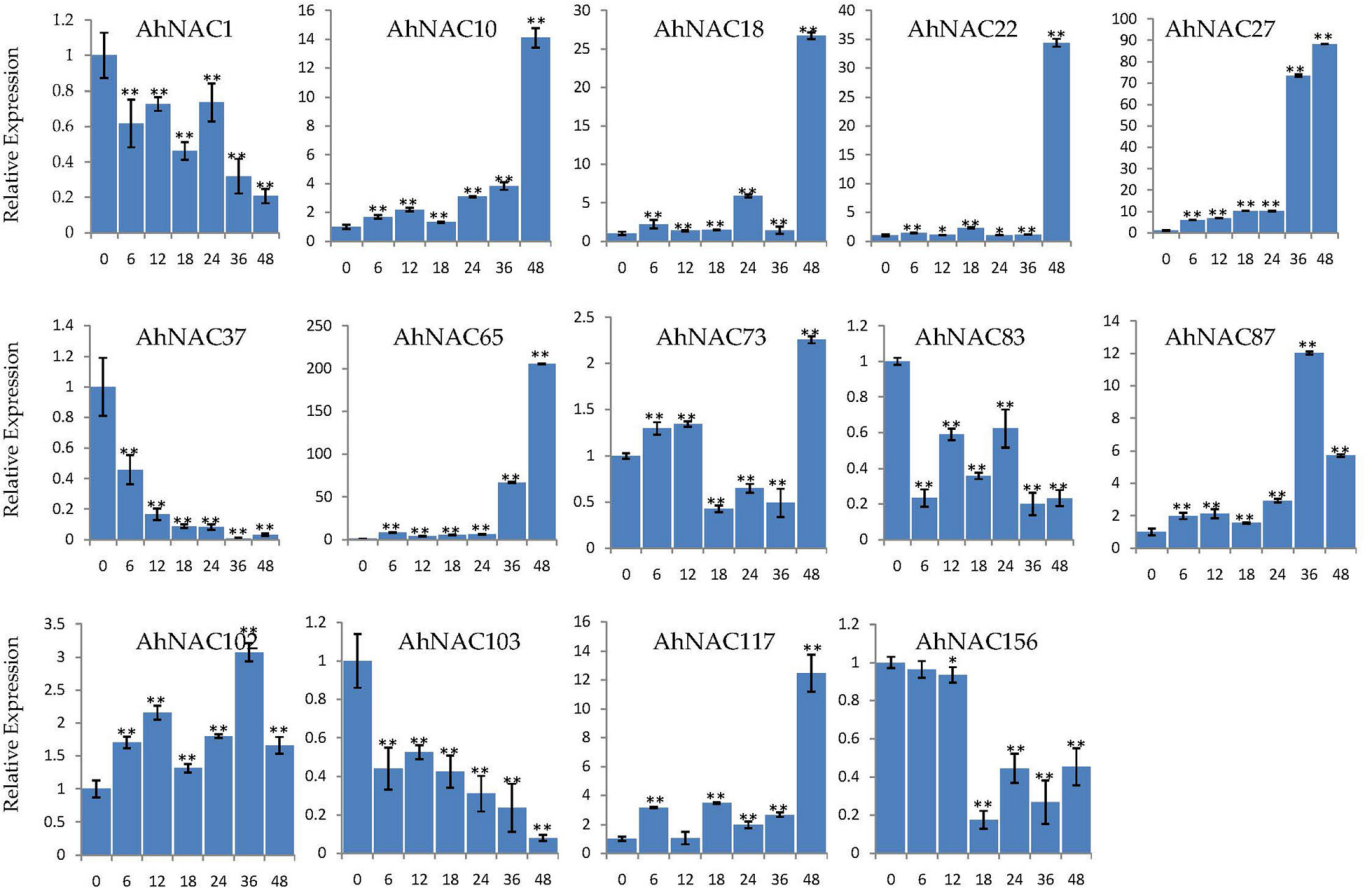
Expression profiling of *AhNAC* genes under salt stress. The Y-axis indicates the relative expression level. The X-axis represents hours (0, 6, 12, 18, 24, 36, and 48) after salt treatment in cultivated peanut. The *actin* gene was used as an internal control. The error bars were obtained from three biological replicates, and asterisks represnt the genes whose expression was significantly up- or downregulated under salt stress, according to t-tests (*, *p* < 0.05; **, *P* < 0.01)

**Fig. 7:**
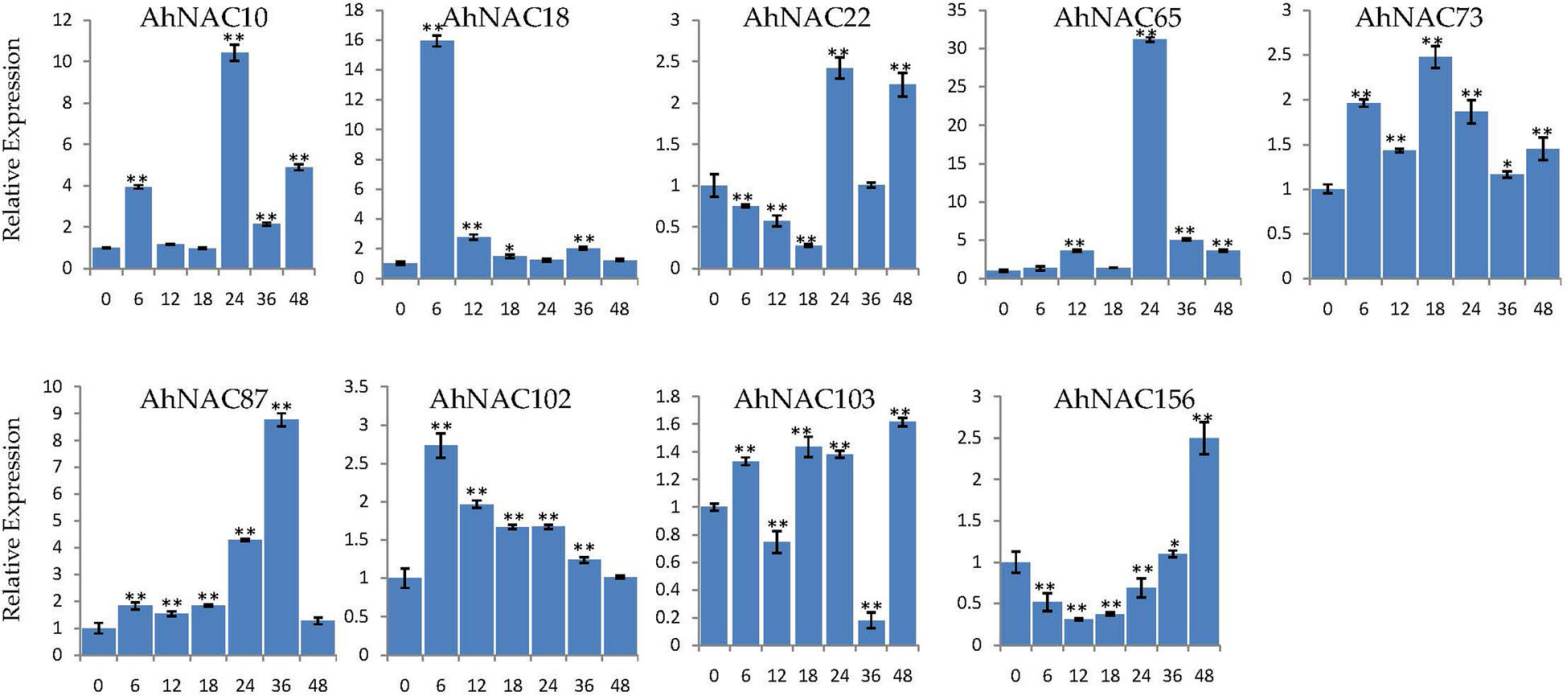
Expression profiling of *AhNAC* genes under drought stress. The Y-axis indicates the relative expression level. The X-axis represents hours (0, 6, 12, 18, 24, 36, and 48) after drought treatment in cultivated peanut. The *actin* gene was used as an internal control. The error bars were obtained from three biological replicates, and the asterisks represent the genes whose expression was significantly up- or downregulated under salt stress, according to t-tests (*, *p* < 0.05; **, *P* < 0.01)

To confirm which genes respond to stress for further genetic engineering of cultivated peanut with improved stress resistance, we performed RT-qPCR expression analysis of the root. Several genes were randomly selected from the 17 NAC genes that were involved in both salt and drought stress responses. Under salt stress (51.33 mM) treatment, the expression trends of most of the detected NACs in roots (except the trends of *AhNAC73*) were identical to the RNA-seq results. For example, the expression of *AhNAC1*, *AhNAC37*, *AhNAC103*, and *AhNAC156* was downregulated under salt stress at all detected time points, while the expression levels of *AhNAC10*, *AhNAC18*, *AhNAC22*, *AhNAC27*, *AhNAC65*, *AhNAC87*, *AhNAC102*, and *AhNAC117* were upregulated. Notably, the expression of *AhNAC10*, *AhNAC18*, *AhNAC22*, *AhNAC27*, *AhNAC65*, and *AhNAC117* peaked at 48 h after salt stress treatment, and the increase in expression of *AhNAC65* reached more than 200-fold (Fig. 6). Under 20% PEG6000 treatment, the expression levels of *AhNAC10*, *AhNAC18*, *AhNAC65*, *AhNAC73*, *AhNAC87*, and *AhNAC102* increased at all subsequent time points after treatment, and the expression level of *AhNAC65* increased by nearly 30-fold after treatment for 24 h (Fig. 7). These results were consistent with the RNA-seq results (Fig. 5). Overall, these results indicated that the response of these genes to salt and drought treatment could potentially improve peanut.

## Discussion

### Characterization of *Arachis* NAC genes

NAC genes are members of one of the largest plant TF families and play critical roles in numerous stress responses [4, 5]. The NAC gene family has been characterized from several plant genomes [10–19, 40, 41]. However, little is known about NAC genes in *Arachis* species. Cultivated peanut *A. hypogaea* originated via hybridization of two diploid wild peanut. The A and B genomes of wild peanut *A. duranensis* (AA) and *A. ipaensis* (BB) are highly identical to the A and B sub-genomes of cultivated peanut (AABB) [32]. The diploid wild peanuts are more convenient for gene cloning than the allotetraploid cultivated peanut (which contains A and B sub-genomes) because the diploids contain only one genome set (AA or BB). The available RNA-seq data of 22 distinct tissue types of the wild peanut *A.duranensis* and *A.ipaensis* made it convenient for gene expression profiling analysis [36]. Therefore, in this study, we performed a genome-wide analysis of NAC TFs from wild peanut and explored their orthologous genes’ potential functions in response to salt and drought stress in cultivated peanut. Information (for example, chromosomal location, gene structure, tissue expression profiles) of NAC genes from cultivated peanut could be deduced from the orthologous genes of wild peanut from this study.

In total, 81, 79 and 164 NAC TFs were identified from the wild peanut species *A.duranensis*, *A. ipaensis* and cultivated peanut *A. hypogaea*, respectively. Two or more peanut NAC genes were found for every orthologue in *Arabidopsis*. Detailed information on the *Arachis* NAC gene family, including model name, location, nucleotide acid length, molecular weight and theoretical pI, as well as *Arabidopsis* orthologues is listed in Table 1 and Additional file 3. A previous study showed that the number of nucleotide-binding site (NBS) domains characteristic of biotic stress resistance genes in tetraploid peanut was less than the sum of them between *A. duranensis* and *A. ipaensis* and caused some resistance abilities lost in cultivated peanut [32]. However, in our study, the number (164) of NACs in *A.hypogaea* was nearly the sum of those between wild *A. duranensis* (81) and *A. ipaensis* (79). This expansion might arise from multiple gene duplication events, including whole-genome duplication in the *Arachis* lineage followed by multiple segmental and tandem duplication events [27, 32]. These results were identical to those NAC from cultivated cotton *Gossypium barbadense* and two diploid cotton species, *Gossypium rainondii* and *Gossypium arboreum* [45]. Previous studies revealed that the involvement of NAC genes performed major functions in transcription regulation [45]. Thus, we speculated that NACs might perform functions through regulating stress-resistant-related genes or proteins, while not performing functions like a “on-off” switch. The number of NAC genes in cultivated peanut (164) was larger than that in other plant species (for example, 105 in *Arabidopsis* [40], 141 in rice [41], and 101 in soybean [46]), which was approximately 1.56-fold than that in *Arabidopsis*, and a similar result was found in *Populus* [10]. The NAC gene density in *A.duranensis*, *A. ipaensis* and *A.hypogaea* (0.07/Mb, 0.05/Mb, 0.06/Mb) was lower than that in *Arabidopsis* (0.87/Mb) and rice (0.37/Mb) [11]. This may be attributed to *Arachis* large genome sizes, which suggested that the genome size and number of NAC family members were not always correlated. These NAC genes were unevenly distributed on each *Arachis* chromosome (Fig. 1). The numbers on each chromosome ranged from 1 to 17, which indicated that there was no positive correlation between chromosome length and the number of NAC genes. Some NAC genes, such as *AdNAC58*, *AdNAC57* and *AdNAC30*, tended to be located in clusters on the chromosome, these gene therefore might function cooperatively [47].

Tissue-specific expression profiling were useful because it identified the genes that were involved in defining the precise nature of individual tissues [48]. In this study, we utilized the published available RNA-seq data of 22 tissue types to examine the specific expression patterns of *Arachis* NAC genes [36]. Twenty-three NAC genes were ubiquitously expressed, which could serve as a platform to regulate a broad set of genes that were subsequently fine tuned by specific regulators. Notably, we found that *AdNAC58* was not expressed in seeds, pistils or stamens, which indicated that its promoter could be used for non-seed genetic engineering.

### Phylogenetic analysis and expression profiling of *Arachis* NAC genes under salt and drought stress

We performed phylogenetic analysis of *Arachis* NAC with monocot (rice) and dicot (*Arabidopsis*) model plant species to investigate the evolutionary relationships and predict drought- or salt-responsive genes. In the present study, these NACs were classified into 18 subgroups, which was largely consistent with the results of previous analyses [10, 40, 41]. Remarkably, the subfamily NAC-p included 36 rice *NAC*s but only 1 *AdNAC* and 1 *Arabidopsis NAC* (Fig. 2), which suggested that they might have been either acquired in the rice or lost in *Arabidopsis* and *Arachis* when they split from their common ancestor. In contrast, there was no rice NAC gene in the subfamily NAC-n (Fig. 2), suggesting that diversification and expansion of this subgroup occurred after the monocot-dicot divergence. This phenomenon has also been found in radish, *Populus* and other species [10, 11].

If the *AdNAC* and *AiNAC* genes were clustered in pairs in phylogenetic tree, the gene pairs were considered as orthologous genes [49, 50]. In this study, 51 orthologous genes were identified from two wild peanut according to the phylogenetic relationship of the *AdNAC* and *AiNAC* genes (Fig. 2, Table 2), which accounted for more than 57% of the entire family, with sequence identities ranging from 61 to 99% (Table 2), Forty-six genes were located at syntenic loci and exhibited high collinearity on the *A. duranensis* and *A. ipaensis* chromosomes (Table 2, Fig. 1). Several putative orthologous gene pairs exhibited low coding DNA sequence (CDS) or low protein identity, which could be attributed to wrong exon-intron splicing originating from genome sequencing mistakes (for example, *AdNAC55* and its orthologous *AiNAC10*). Several NAC genes from both wild peanut species were not located in the corresponding chromosome regions, suggesting the occurrence of large chromosomal rearrangement in the diploid genomes. Orthologous genes ususally exhibit similar characteristics and expression patterns [49, 51]. The functions of orthologous NAC genes of cultivated species which derived from two wild species may be redundant. For example, *AdNAC54* and *AiNAC13* from subfamily VIII have 3 exons and shared the same conserved motif. Both were highly expressed in nodule roots and flowers, but expression at a relatively low levels of in the other organs, which was similar to the results of its corresponding *Arabidopsis* orthologs *NAC2* which expressed in roots and flowers with respect to regulating the salt stress response and lateral root development [52]. Additionally, *ANAC2* can also be induced by abscisic acid (ABA), 1-aminocyclopropane-1-carboxylic acid (ACC) and 1-naphthylacetic acid (NAA) [52]. Their corresponding orthologous genes in cultivated peanut may function together. Orthologous genes from different plant species showed a tendency to fall into one subgroup and shared similar functions. Many NAC genes have been functionally characterized in *Arabidopsis*, and their orthologous genes in *Arachis* were identified in this study (Table 1). Together with the phylogenetic results, it was possible to predict the functions of peanut NAC genes on the basis of the functions of their *Arabidopsis* and rice orthologues, which could also be potentially utilized for further functional studies. For example, *AdNAC77*, *AiNAC9*, and *AiNAC35*, together with their *Arabidopsis* orthologous gene, *ANAC19* (At1g52890) gene were clustered into the same NAC-g subfamily (Fig. 2). The expression of *ANAC19* was induced by drought, high salinity, and abscisic acid (ABA). In the same subfamily, the expression of *Arabidopsis ANAC55* (At3g15500) and *ANAC72* (At4g27410) was also induced by drought and high salinity [8]. Therefore, we speculated that *AdNAC77*, *AiNAC9*, and *AiNAC35* are drought- and high salinity-responsive genes that regulate peanut survival under adverse growth conditions. Not surprisingly, *AhNAC87* (the orthologous gene of *AdNAC77* and *AiNAC35* in cultivated peanut) was induced under both salt and drought treatments based on RNA-seq analysis (Fig. 5), and the RT-qPCR-based results confirmed that, in cultivated peanut, the expression of *AhNAC87* was upregulated under both salt and drought stress treatments (Figs. 6 and 7). Additionally, *Arabidopsis ANAC2* (At1g01720, also known as *ATAF1*), which is orthologous to *AdNAC22*, was induced by drought stress [53]. The expression of their orthologue *AhNAC37*, was upregulated approximately 27.5-fold under drought stress according to the comparative RNA-seq analysis (Fig. 5). These findings strongly supported that the functions of *Arachis* NAC genes could be deduced from these orthologous genes from *Arabidopsis* and rice.

Previous reports have provided strong evidence for phylogenetic analysis based prediction of the stress-related function of several gene family members. The dehydration-induced gene *AhNAC3* (EU755022, *AhNAC117* in our study) provided hyper-resistance to dehydration and drought stresses [27]. In our study, the expression of *AhNAC117* was induced under salt treatment based on the comparative RNA-seq data (Fig. 5), and was confirmed by RT-qPCR (Figs. 6 and 7). Similar results were found for *AhNAC4* (HM776131, the orthologue of *AhNAC87* in our study, and orthologous to *AdNAC77* and *AiNAC35*) and *AhNAC2* (EU755023) [28, 29]. These two genes shared 97.78% similarity, were highly induced by drought and salt stresses, and conferred drought and salt tolerance to transgenic plants.

## Methods

### Sequence database searches

The sequences of all NAC genes in this study were retrieved from the PeanutBase database (www.peanutbase.org) using the NAM domain (PF02365) as a search query. We verified the putative candidate proteins manually using the NCBI database (https://www.ncbi.nlm.nih.gov/) to confirm the presence of NAM domain. Each protein sequence was examined via the Simple Modular Architecture Research Tool (SMART; http://smart.embl-heidelberg.de/) domain analysis program and the Pfam (Protein family: http://pfam.xfam.org/) database to confirm the reliability of the search results. Only the sequences containing these domains were retained. The MWs and pIs of each protein were predicted by proteomic and sequence analysis tools on the ExPASy Proteomics Server (http://web.expasy.org/compute_pi/). The putative *Arabidopsis* orthologues of peanut NACs were identified via BLASTp searches.

### Sequence alignment and phylogenetic analysis

To study the phylogenetic relationships between NAC proteins from peanut and those from dicot *Arabidopsis* and monocot rice, the *Arabidopsis* NAC protein sequences were downloaded from The Arabidopsis Information Resource (TAIR; https://www.arabidopsis.org/) and the rice NAC protein sequences were downloaded from the Rice Genome Annotation Project (RGAP; http://rice.plantbiology.msu.edu/). Full length amino acid sequence multiple alignments were performed by the ClustalW program. Unrooted phylogenetic trees were constructed using the neighbour-joining (NJ) method by MEGA 6.0 software, and the bootstrap test was carried out with 1000 iterations.

### Chromosomal locations, gene structure and conserved motif analysis

The chromosomal location information of NAC genes was retrieved from the PeanutBase website (www.peanutbase.org). These genes were mapped onto the chromosomes via the MapInspect program (http://mapinspect.software.informer.com). Information concerning both the mRNA and gDNA of the peanut NAC genes was obtained from the PeanutBase database (www.peanutbase.org). We used the GSDS (http://gsds.cbi.pku.edu.cn) online program to explore the exon/intron organization of the NAC genes. The MEME (http://meme-suit.org) program was used to investigate the motifs within the NAC protein sequences. The domains in all the protein sequences were analysed via Pfam 31.0 (http://pfam.xfam.org/) based on the hidden Markov model.

### Prediction of *cis*-acting elements within promoters

Promoter sequences (2.5 kb in length) were download from the PeanutBase website (www.peanutbase.org) for *cis*-acting element analyses. The numbers of several elements related to biotic and abiotic stress responses were identified via New PLACE (https://sogo.dna.affrc.go.jp/cgi-bin/sogo.cgi?lang=en&pj=640&action=page&page=newplace) [54].

### RNA-seq-based expression profiling of NAC genes in peanut

The average fragments per kilobase per million reads mapped (FPKM) values of 22 distinct tissue types and developmental stages were obtained from the study by Clevenger et al. [36]. The FPKM values of each NAC gene were log2 transformed and displayed in the form of heatmaps via HemI [55].

To investigate the expression patterns of NAC genes under salt and drought stress treatments, the average FPKM values of each gene under salt [37] and drought [39] treatments were obtained from our previous work. The average FPKM values of these NAC genes whose expression changed by more than twofold were compared via Excel software, log2 transformed and displayed in the form of heatmaps using HemI [55].

### Plant materials, growth conditions and stress treatments

‘Huayu 9303’, a cultivated peanut bred by our team, was grown in a temperature-controlled chamber at 20 °C with a photoperiod of 16 h of light and 8 h of darkness unless stated otherwise. After approximately 1 month, the plants were treated with 51.33 mM NaCl (for salt treatment) or 20% polyethylene glycol (PEG) 6000 (for drought treatment). The roots were collected after 0, 6, 12, 18, 24, 36, and 48 h of treatment, immediately frozen in liquid nitrogen and stored at − 80 °C.

### RNA extraction and RT-qPCR based analysis

Total RNA was extracted with a MiniBEST Plant RNA Extraction Kit (Takara, Dalian, China). First-strand cDNAs were synthesized using a PrimeScript RT-PCR Kit (Takara), and qPCR was carried to check the expression levels of AhNAC genes under salt and drought treatments. The reactions mixtures consisted of 2 μL of cDNA (10.3 ng/μL), forward and reverse primers (400 nM each), 10 μL of TB Green Premix Ex Taq II (Takara), and added sterile water to bring total volume to 20 μL. Amplification was performed on an ABI 7500 Fast Real-Time System (Applied Biosystems, CA, USA) as follows: 50 °C for 2 min; 95 °C for 2 min; and 40 cycles of 95 °C for 15 s and 60 °C for 34 s. The specificity of the reactions was verified by melting curve analysis. Gene specific primers for each detected NAC gene for RT-qPCR were designed based on the basis of the difference between othologous genes and were listed in Additional file 15. Each gene was performed with three biological replicates. Gene transcript levels were calculated using ^ΔΔ^Ct method [56]. Student’s t-test was performed to calculate the P values using SPSS software. When P was < 0.05, we considered the NAC genes were differentially expressed genes. To normalize the expression level of the selected NAC genes, *actin* gene was used as an internal control [47].

## Conclusion

In the present study, a comprehensive analysis including phylogeny, chromosomal location, gene structure, conserved motif, *cis*-acting elements within promoter regions, and expression profiling of NAC gene family members in two diploid *Arachis* species was performed. These results provide a useful foundation for future research on *Arachis* NAC genes. On the basis of comparative RNA-seq and RT-qPCR-based analysis, we also identified NAC genes involved in drought and/or salt stress responses, which could be potentially used for peanut improvement.

## Supplementary information

**Additional file 1.** mRNA sequence of NAC genes from two wild peanuts.

**Additional file 2.** gDNA sequence of NAC genes from two wild peanuts

**Additional file 3.***NAC* TF gene family members in cultivated peanut.

**Additional file 4.** mRNA sequence of NAC genes from cultivated peanut.

**Additional file 5.** NAC proteins of two wild peanuts.

**Additional file 6.** *Arabidopsis* NAC proteins.

**Additional file 7.** Rice NAC proteins.

**Additional file 8.** Phylogenetic tree analysis of NAC proteins among *Arachis*, *Arabidopsis* and rice based on conserved NAM domains.

**Additional file 9.** Exon-intron structure comparison between *AdNAC59*, *AdNAC80*, *AdNAC81* and their orthologues *AiNAC59*, *AiNAC9*, *AiNAC29*.

**Additional file 10.** Sequence logos for the conserved motifs within NAC proteins.

**Additional file 11.** 2500 bp promoter region of NAC genes from two wild peanuts.

**Additional file 12.** Number of different cis-acting elements present within the promoter of NAC genes.

**Additional file 13.** Genes involved in the salt response based on comparative RNA-seq data. The Y-axis represents the fold change compared with the level in un-treated plants. The X-axis shows the genes whose expression was upregulated and downregulated more than 2-fold under salt treatment in cultivated peanut.

**Additional file 14.** Genes involved in the drought response based on comparative RNA-seq data. The Y-axis represents the fold change compared with the level in untreated plants. The X-axis shows the genes whose expression was upregulated or downregulated more than 2-fold under drought treatment in cultivated peanut.

**Additional file 15.** Primers used in this study.

## Data Availability

All data generated or analysed during this study are included in this published article and its supplementary information files.

## Abbreviations

NAC: NAM, ATAF1/2, and CUC2
NAM: No apical meristem
ATAF1/2: *Arabidopsis thaliana* transcription activation factor
CUC2: Cup-shaped cotyledon
TF: Transcription factor
RNA-seq: RNA sequencing
RT-qPCR: Real time quantitative polymerase chain reaction
Gb: Giga-base pair
Mb: Million base pair
aa: Amino acid
kDa: Kilo Dalton
MeJA: Methyl jasmonate
ABA: Abscisic acid
ACC: 1-aminocyclopropane-1-carboxylic acid
NAA: 1-naphthylacetic acid
CDS: Coding DNA sequence
MW: Molecular weights
PI: Isoelectric point
RGAP: Rice Genome Annotation Project
TAIR: The Arabidopsis Information Resource
NJ: Neighbor-joining
FPKM: Fragments per kilobase of transcript per million mapped reads
PEG: Polyethylene glycol
