# Supplementary material for: Comprehensive genomic characterization of NAC transcription factor family and their response to salt and drought stress in peanut
Source: BMC Plant Biol. 2020 Oct 2;20:454. doi: 10.1186/s12870-020-02678-9 (PMC7532626; doi:10.1186/s12870-020-02678-9)
Supplement: Supplementary file 1 — Additional file 1. mRNA sequence of NAC genes from two wild peanuts. [file 12870_2020_2678_MOESM1_ESM.docx]

>AdNAC1--Aradu.08GFU

ATGGATGTGGCTAAGTTGTACATGAACAACGACTACTCCGAAGAACATGAACATGAATATGAACATGATGAAGATGATGATGAGATGATGAAAGAGGAGAAAGAAGTTGTGCTTCCTGGGTTTAGATTCCACCCAACAGATGAAGAGCTTGTTGGGTTTTATCTTCGGAGGAAGGTTGAGAAGAAGCCTCTTAAGATTGAACTTATCAAACATGTTGATATCTACAAATATGATCCATGGGATCTTCCAAAAGTTGGTTCATCAATGGGGGAGAAGGAATGGTATTTCTTTTGCATAAGAGGGAGAAAGTACAGGAACAGCATAAGGCCTAATAGGGTTACAGGATCAGGGTTTTGGAAAGCCACAGGGATTGATAAACCTATATACAGTGCTAATTATAATAATAATTATAATAATAATAGTAATAATAATAGTAAGGAGCATGGTGATCATCATGAATGCATTGGACTGAAGAAATCATTGGTTTATTACCGGGGAAGTGCTGGAAAAGGCACCAAAACTGATTGGATGATGCATGAGTTTCGCCTCCCACCCAATAATAATAATGGAGCAAAATTATTAAGCAATAATCAAGAAGCTAATAATGCTACCAAGGATCTTCATGAAGCTGAAGTGTGGACACTATGCAGAATATTCAAAAGGATTCCAACATACAAAAAGTACACACCAAATTTGAAAGATTCATCAACATCACCACTCATGAACAAACCCATCAATAACATTAACCACCAAACTGATTCCTCAGTAACTTCCATATCATGCAGCTTAGAATCTGACAACAACAATAGCAAGCAATTCTTGACTTTCACTAACACTATGGGGATTCAACAATGTGAAAGGAAGCCTCTTGTTATTGGACATGTTGATGAAAGGAACAACAACTTTTTCTTAGACCATTCATCAATACATCATCAACAAGCTCCAACAACAATTACTACTACTGCTTTGTCATCATCATCATACTCATCATGGAACCAGCACCATGTTGTGGAGGATTACTTGTTTGCAAATGAGAATTGGGATGATCTTAGATCTGTGGTTGAGTTTGCCACTGACCCCAATAATTCCAAGGTTTATCTATGATTGTAATTAAATATATGTTTTGAGAGGGTATTATTATTAGGGTATTTAGCTTCTTAAA

>AdNAC2--Aradu.08TAH

TATTCATCACCAATGTATTAACTAAGTCCTGTTAGATCATAAATCCTTGTCAAGATAATATGATCTGCTTTCACTCCTAATGAAATTGTTTCAATCTGGTGTCACACACTCCTTCCAAGTATTCTGTTTATAAATTATAATGAAAATATTTGGTTGATGATAAACACCAGCTTGGGTTTCACGTCAACACCCTTATTATAAATACCTTAGCTTCTCTATTTGTACTATAAATACCACTCATCTTCACCATTACTCTAACTTGACACATTCCCTCCTCTTGTTTTCTCTGCTTATCTTCTTAATGGGAGATAACAATGTGAACCTTCCACCGGGGTTTCGATTTTATCCAACAGATGAAGAGCTTGTGGTCCATTTTCTTCATAGAAAGGCAGCACTCTTACCTTGCCACCCTGATGTCATCCCTGATCTTGATCTCTATCCTTATGATCCTTGGGAACTTGATGGTAGAGCGTTAGCAGAGGGAAATCAATGGTACTACTACAGCAGGAGAACACAGAGTAGGGTGACTGAGAATGGATATTGGAAAGCAACAGGAATGGAAGAACCAGTGATGACAAGCTCAACCAACAAGAGAGTTGGCATCAAGAAATACTTTGTGTTTCATCTTGGTGAATCCCCTTCTGCTATCAAAACAAATTGGATAATGCAAGAATATTGCCTTTCCGATTATTCTGCTTCCTCTAGCAGATCCTCCAAAAGAAAATCAGATTATAGTAAATGGGTGATATGTCGTGTTTATGAGCGCAATGGAGATGATGATGATGGAACGGAGCTCTCTTGTTTGGATGAAGTTTTCTTGTCACTCGATGATCTTGATGAAATAAGCTTACCAAATTAAATTAATTAATCAAGCTAGCTAGCTGCAT

>AdNAC3--Aradu.0MJ0X

TTCCTTCCTTCCAAGTTCAAATTAAATTTCAGCTAAGTAGTAGTAGTAGTAAGTGATTATTCTAGCTTGATGGGTTCTTCTAATAACGGTGGTGTGCCACCGGGGTTTCGATTTCATCCAACTGATGAGGAATTGCTTCATTACTACTTGAAGAAGAAGGTGTCGTTTCAGAAGTTTGACATGGATGTTATTAGAGAGGTCGACCTCAACAAGATGGAGCCTTGGGACTTGCAAGAAAGATGCAGAATAGGGTCAACACCACAAAACGAGTGGTATTTCTTCAGCCACAAGGATAGAAAGTACCCAACAGGGTCAAGGACAAACCGAGCAACGAACGCAGGGTTCTGGAAAGCCACGGGAAGAGACAAGTGCATAAGGAACACCTACAAGAAGATTGGGATGAGAAAGACACTAGTGTTCTACAAAGGTAGAGCCCCTCATGGCCAGAAGACTGATTGGATCATGCACGAGTACCGTCTTGAAGATTCCAATGATCCTCAAGCAAATGCCAACGAAGATGGGTGGGTGGTGTGCAGGGTGTTCAAGAAGAAGAACCTATTCAAGATTGGAAATGAAGGAGGTGGTGGCTCCACACACACCTCATCGGACCAGCAACTCAACAACTCAACGGCCACCAATGCTCGTTCCTTCATGCAAAGGGAAAACCACTACCTACTGCATCACCACCAACAACAGCAAAACCCTAGGAATGGGAACCCATCGTCTTCATCCTCAGGCTTTGATGAGCTCGATAAACCCGAACTCGGTCTCCATCACTATCCTCACATGCAAAACCCACACTATTCACTCTTCCATCACTCCCAACCACTTCTTCATCCCCAGGCCCACAAGCCCATCGTCTATGACTACTCTTATACACCCGCGCTTCCCTCAGACCCTCCTGTCACCGCTAAGCAGCTCATGACTAACCCTAGAGACTGCGATAGCGGTGGCAGCGAGAGTCTGAGGTACCAGCAGGTTTCCGAGCCTGGTATGGAGGTTGGATCATGTGAACAAGCCCAGGAAATGGGCGCCGCCGCGGCCGCAAGAGGAGGAGGAGAAGGAATGAATGAATGGGGTGTGCTTGATAGGCTTGTAACCGGGAACCTTGGAAATGAAGATTCAGCCAACAAAGGGATTAGGTTTGAAGATGCAAATCCACACCAGATTAACCAGCTTTCTTTGAGGGGAGAGATGGATTTCTGGGGCTATGGAAAACAATAA

>AdNAC4--Aradu.13D06

CTACCCTCATCCCCATGTAGGGCCTGAAAGCTATGTAGATCTTTCTATTAACTCCCATCCTCTCTCTCCTCTTTTTTCTCAACACCTATTTAAAACACACGCCTCATTTTCTCCTCTTTTTTATATATTAACTACTCTGCCTCAAACCCTCAGTTTAATTTCTTCAATTTCTACATTTTAACCATCAATCTCTTATCTCTGCCTAACACCACTGCACCACCCAATTCGTACAACTATTACACTCTCTAAGTATAAAGACAGAGAGAGAAATTAAAGGGGGTCCAAACTCGAAGCACTAGTTACTACTACGATAGCTAGATATAGTATTATTATCAGTGTTGGATGGCAATTGCAGCGCCGAATTCATCTCCGACAATGAGTCTGAGTCACAGCCACAGCCACGAGGACGGGGGGACGACGACGGCGGCCTCCACCACGAACGACAACCTGAACGGGAACGGGAAGCAAGAGGATGATGATCACGAGCATGACATGGTGATGCCGGGGTTTCGTTTTCACCCGACAGAAGAAGAGCTGGTGGAGTTCTACCTTCGCCGTAAGGTGGAGGGCAAACGTTTCAACGTTGAGCTCATTACTTTCCTTGATCTTTATCGCTATGACCCTTGGGAGCTTCCTGCGTTGGCGGCGATAGGAGAGAAGGAATGGTATTTCTATGTGCCTCGAGACAGAAAATACAGAAACGGAGATCGTCCGAATCGAGTGACGACGTCGGGTTATTGGAAGGCAACGGGAGCAGATAGGATGATAAGGACGGAGAATTTCAGGTCCATCGGGCTGAAGAAAACCCTAGTTTTCTACTCTGGGAAAGCTCCTAAAGGCATCCGTACAAGTTGGATTATGAACGAGTACCGTTTGCCCCAACACGAAACTGAACGATACCAAAAGGCGGAGATATCGCTGTGCCGGGTTTACAAAAGAGCTGGAGTTGAAGATCATCCGTCGTTGCCGCGGTGTCTGCCAACGAGGGCTCCATCTTCAAGAACTGTTGATCATCAGAAGAACAAGCAGCAGCACCACAACGATCAACTCAACATGGGATTTGCGGGGAACACCGCCGACGGAGCTTCTGATAATCGTGATCATGATGTAACCACCGCTCTCGCCCTCTCCAAACACAACACAAATGCTTATCGTGCTCCTTCCATGGGACTCCCACCGCTGCTTCTTCCCTTGGACGACGAAGCCGCCTTCGTCCTCATGCAGCAGCAGCAGCACCATGCTGGCCCTTCTTCAGGAACCACCACCATGATGGATGATCTCAACAGACTTGTAAGCTATCAACACCAGTACTACAACAGCAGCAGTAGCAGTAGTAACAATAATAATCCCAATCATCATCATCACCTGTTAATGCAACAACAACAACAGCAGCAGCAGCAAACTCCTCCTGCAATAATGTCTCTGAATAACACTCCTTCTCCGCTTGCAACCGCCTTCTCTGACCGCCTGTGGGAGTGGAATCCACTCCCGGAGGCCAACCAGCGCCAGTACAGCAACATGTCCTTCAAGTAA

>AdNAC5--Aradu.15JI0

CAAGTGATCGGAGTGAAGAAAACCATGGTTTTCTACAAAGGAAAAGCTCCCACCGGTCGCAAAACTAAATGGAAGATGCATGAATACCGCGCCATCGTTCAAGCCCCTAACCAATCTCCCACGGCTATTCCTCAGTTGAGGCACGAATTCAGCTTGTGTCGCGTGTACGTGATATCCGGAAGCTTCAGAGCATTTGATCGACGGCCACGGGAGGTGGTAGTGCCAAGAGTTCTTCATCATGGTTCTTCTACAACAAGTGCTCAGCAGCATCAAGGAGAATCATCAGCAAGGGTGCAGGCCAATAATAATAATAATGGGTCGAGCTCGTCGGAAACTTCCCTTTCATCAGGTGGTCCTGATTTGCCACCAGATACTGGAGGAGGAGGGTCATGTAGCAATTGGAATAGTAGTGAGGTTCAAGTTCAAGCTCAAGTTCAAGAACCACTATGGGAATGGGAACAACTCGATTGGCTATAAGCATGACTAATAAGAATATCAATTCGTCAAGCCATGCATGCATTTCATCTCTCATCAAGACCCCTTCTCCACCACTTCAATAATTCCTCTGTTTTAAATTAATGGTCGCTTTCACTTTTTCGTTTTAACTTCATTTATTTAGAAATAGTAAAATAGGGTATTTGTATAATTAACATGTGTAACACTATCAGGATTTGATTTTCACCGAGAAAATAAAGTAGGTGTAACTCTAATTAAGGAGAGATCATGTAATATTAGAACTTAATTAGAAGCTAAAGTATAGTTTTTTACTTTTTTTAAGCATATTGTATCATTTTATTTAGTTTGCATAAAGGTTAATTTGTAACCAAATTCTTCTCAT

>AdNAC6--Aradu.15QQT

ATGACAGAAACTACAATTTTACCTGTTGGATATAGGTTTCGTCCAACAGAAGAAGAACTTTTAGTTCACTATCTCAATAACAAGCATTTGAGAAATGATGCCGAGATCAAGAACACTGTTTCCCAAATTGATCTTTATAACTTTGATCCTTGGGATTTGCCAGAACAATCGAAGGTGAAATGGGATGATCAAGAATGGTTTTTCTTCAATGAATTGAAACACATAAAAAACAAGCGGTGTAACGGAAAAACTAACGCCGGTTATTGGAAGATCACCGGAAAAGAGCGGATCGTCAAAAGAACAGGGATAGACAATGTGATAGGTACAAAAAGAACACTAGTTTTCTATAAGCGTCCACATAGTGTCAAACCAATTGGGTTCTTCATGAATATCATGCACTTCATCTAAAGAGCAACGTCGTGTTGAGCCGTGTAATAAAGAATGTTGAGAAAAGGGAAAAGAAGGTTAAGAGAAAAGCAAGCAACATAATCGAAGAGGAAGTAACATGTGAAGAAGATGAACCATGCAGCGAAATTACTGGCTATGTTACCGAAGCAACTACAGAAGATGCAATAATTCCTGATGCATGTGTTTCATCCGAACCGCAAGCACCTCAAGATGTTGACTATGAAATTCTTTCGTCGGAACAACAATCTTCGGTGGCCCATTCCGGTAATGGAAGCAACAATTCTCCGTTGCTTCCATTTGAAGGTATGTGGAAGCAAGATGTCGAGATGAACACGGAATGTTTTTGAAATTTGCTGTTTTCTAGCATCGATGCTGACCTTGATGCGGAGTTCTTGAATTCGGTGTTGGCAGGGGATGATTAACTCCATGTTGATTCCGGCCACCATTGACTTTACATAGGTACAAGTAAAGATGTAAAAAAATTCTCTTGCATGGACACGCACAACTTTCAAGCATGTTTTTGAGCAGCTTTATTTTGACAAGAAATTTGACACCCACGTTGTAGTTGACTAGTCGTTATACTCGAACAACATTAGATCTAATCTAATGAATAGAATGTATATATATTCTCAACCT

>AdNAC7--Aradu.1AJ4F

CCCCTCTTTCCTGTAAGCTTCTTTGTCTTCTTTCCTTTGTCCTCAAACGCTCATTTCCCTTGTTGCTTCTCACTAAAGGTAATTAATTAAAAGCAGAAGAATAATCAGAATGATGGCAGGTAGTGGACAACTAACAGTTCCACCAGGGTTCCGGTTCCATCCAACTGATGAGGAGCTTCTCTACTATTACCTAAGGAAGAAAGTTTCTTATGAAGCCATTGACCTTGATGTCATTAGAGAGGTTGATCTCAACAAACTTGAACCTTGGGACCTCAAAGATAAATGCAGAATAGGATCAGGGCCTCAGAACGAGTGGTATTTCTTCAGTCACAAAGACAAGAAGTACCCAACAGGAACAAGGACCAATAGGGCAACCACTGCTGGTTTCTGGAAAGCCACTGGGAGGGACAAGGCCATATACCATACTAGCAATTCCAAGAGGATCGGGATGAGAAAAACCCTAGTTTTCTACACCGGCCGTGCGCCCCACGGCCAGAAGACTGACTGGATCATGCATGAGTACCGCCTCGACGAAGACGAGGCCGAGGTTCAGGAGGATGGGTGGGTCGTGTGCAGGGTTTTCAAGAAGAAAAACCAAAGCAGAGGGTTTCAACAAGAAATTGAAGAAGAGGAACATCATCACTTAGCAGCAGCACATCAACACATGAGAGGAGTAGCAAGCCAACAAGTTCTGGACCCAAAACACCACCACCACTTGCAACATCATCAAGGACTCTATGATAATGAAAATAATAATAATTACACCAATAATTTTGATGGATCCATGCATCTTCCACAGTTGTTCAGTCCAGAATCTTCCGTGGCTACCGCGGCGGCGCACACTTCCATGAATGCCATGGACATTCTTGAATGCTCCCAGAACCTTCTAAGGCTCACAACAACAAGTGGATGTGGACTCAATCTCATGCAACAACAACATGGAGAGAGGTTCAATGGTGATTGGTCTTTCTTGGATAAGCTTCTTGCTTCACACCATGGCAGCACCATGGATCATCATCAGCATCATCATCATCATAGCAAATGTAACAATAATCTTCATCATCAGCATTCTGCAATTGCTATTGGAACTACTTCATCTCAGAAATTCCCATTTCACCACCTTGGTTGTGACAACCATGATATCATGAAGTTTTCCAAGTAG

>AdNAC8--Aradu.215DG

CATCAGTAATCACCAATATCATCAAGCACATAAGAACCAATTCAAGGCGGTGGTAATCATAAGCTAAGCCAAGGGAGATAAAACGTGTCCCGAAAGAGATACAAATAAAGTACTACTATGCTTTCTACCTTACTTTACCTTTCTTTTAACTACATACTTAACTTGTAATTTTTTCTCCTGTTCAATTTTTGGGTATTTTTTTTTTTACAATTCTGAGTATAAGGGAAGAAAAATTAGAGGGTGGTCTAATTTATTGTGATGGCATGGTGCAATGAGACTCATGAGAAAGAGATCATTGCTTCCAATAATAGTACTATTACTCTTAGACCTAAATCCGACCAAGAAATTCGAAACATAAGTTGCCCCTCATGTAGCCATAACATTCAAATAATCCAAGAGCAGGGTGGAATTCATGAGTTGCCAGGGTTACCAGCTGGAGCGAAGTTTGACCCAAATGACATTGAAATATTGGAGCATTTGGAGGCAAAAGTTATGTCTCATGTGCCCAACCTTCATCCTCTCATTGATGAATTCATACCAACGCTTCAAGACGAGAATGGCATCTGTTATACACACCCAGAGAAGCTACCAGGAGTAAAGAAAGATGGGCAGATTCGGCACTTCTTCCACAGGCCTTCAAAAGCATACACAACAGGAACAAGGAAGAGAAGAAAGGTTCACACCGATGAAGATGGAAGCGAAACAAGGTGGCACAAAACCGGAAAAACAAGAGCGGTGGTGGCCGGCGGCCTAGTCAAGGGGTTCAAGAAGATTCTAGTACTATACACCAACTATGGGAGGCAAAAAAAGCCTGAGAAAACTAACTGGGTGATGCATCAATACCATCTTGGAAGCAATGAAGAAGAGAGAGATGGAGAACTAGTAGTTTCAAAAGTGTTCTATCAAACACAACCTAGACAATGCGGCAATTCCATTGTTATAAAGGAAGATGATGATGATCTTCCCTATGGAAAGATATTGATGATGAATAACAGAAAGAAGCACAAAAATAATGATGATGCTGCTCCTGTTGTGGACTACTACATAAATTATGACCATGTTGAGCATCATCATAATCACAATCATAATAGCCAAAGATGTTCATCACCTACTCAACTTATTCCAAACTTGGTTCTCCAAGGTGATTCCTCTTCTCTTTTTCGCTTTGCTTCATCATCACTGGATGGGAATGCCAACAAAACAAGACTTTTTGAGAGAAAGTTGTAGTAATTCGTTCATTATTATTATTTCTTTGACTAAGTGACAATATTTATTCTATACATATATGATATATG

>AdNAC9--Aradu.22647

GTGTATTTAAGCACACCTTCTTCCCTTCATTTTACTTTCTTAATCTTTCATCACTTCATAATTAATTCCTCATCGCTATAATGGATGCGGAAGATCATAATCATGCTTTGGATTTGCCTCCCGGTTTCAGGTTCCACCCTACAGATGAGGAGATCATCTCTTATTATCTCACTCACAAGGTTTTGAACACAAGTTTCACCGCAACTGCCATTGGAGAAGTTGATCTCAATAAGTGTGAGCCTTGGGACTTGCCTCAGAAAGCAAAGATGGGGGAGAAAGATTGGTACTTCTTCTGGCAAAGAGATAAAAAGTACCCAACTGGGATCAGAACGAATCGAGCCACGGAATCCGGCTACTGGAAGGCCACAGGAAAAGACAAAGAGATTTACAAAGGGAGAAACCTTGTTGGTATGAAGAAAACCCTTGTGTTCTATAGAGGTAGAGCCCCTCATGGACACAAAACCAATTGGGTTATGCATGAATTCAGATTGGAAGGCCTTTTTGCTACTTACAACCTCCCTAAACCTGCTAAGGAGGAATGGGTTGTGTCGAGGGTTTTCCATAAGAATACAACAGAAAAATTGAACCCAACTATTCCATCTGGCCTCTTTAGGATAATGAAGAACGTGAACTCAATTGAGGATGATGATCTTGTAGATTTTTCTTCTCTCCCACCTCTCATGGATCCTTCTAATAATTATGATGATGAACACACCACCACCACCAACAATATGTTTGCATCATCATCAGATTATAATATTACTATTCAGCAAAACAAGAAGGATATGATGGGAATAAGGAATAATAATATTAGAGCATTATTAATGTATGACGGTCCATCATCATCATCAGAAGTAGTTGCTCCTCCTCTCTCTGACTTGGAATTATGCCTCTGGGATTTATAACTTCTTACCAAGATTATTATGACAAGACACAGACATATTTAATCA

>AdNAC10--Aradu.30S8W

CTCTCATCTTCTCCGTTCTTCAAACCTTTTTCACTTTTTTTTTCTTTATCACCAAAATCCTCGGGATCTGATCTTCACTCTCTTTCTCAAGTTCAATTTCCGAGGATCATATTCATAACAAAAACTACTACTACTTTTCTTATTGTCTCTCTGTTCTTTTGTTCGCTTAATTACCTTTCTTTCAGTGCTGTTTTCTTGTCTGCTTCTCTTTCCATTCCACAAATTGTAGTGTCACTACAAAAAAGCCTTTCAACAATTATCAATAACTTCCCATCAAAACCAAGTTTTAGTGCGTGTTTACTTAGATCGCTACCGGATTCTATTTGGGGAAAGAGCAACATAGATCAAGGTTCAAGGTTTTTGGTTGTTTTGGTGGCGACATGAACACCTTCTCCCACGTACCTCCAGGCTTTCGTTTTCATCCGACTGATGAAGAATTAGTTGACTACTACCTTAGGAAAAAGGTAGCATCCAAAAAGATTGATCTAGATGTCATCAAAGACGTTGATCTCTATAAAATTGAGCCATGGGATCTTCAAGAACTATGCAAAATAGGAAGCGATGAAGAAAATGACTGGTATTTCTTCAGTCATAAAGATAAGAAGTACCCAACAGGAACAAGAACGAATAGGGCAACAAAAGCAGGGTTTTGGAAAGCCACGGGAAGAGATAAAGCAATATACTCAAAGCAGCATTGCCTTATTGGAATGAGAAAGACTCTTGTCTTCTACAAAGGAAGAGCTCCTAATGGCCACAAAGTGTTCAAGAAGAAAATGGCAACAGTGAGGAAAATTGGAGACTATGATTCACCATGTTCTTGGTACGATGAACAAGTTCCCTTCATGCAAGATCTTGAATCTTCATCCCCAATAAAGCCACCAATAATTAACAACAACCATTATGCTTCTTCATACAACCATCACCAGTTACAATTACCCTGCAAACCGGAATTCCATCAACCAATGCAATACAACAACATGAACATGCCACGTCACGACGACGCTGCTGATAATAACAACAACTTCCTCCAACTTCCTCAGCTTGAAAGCCCTAATGCTGGAATTAGCCCCTTCTTGCAACAACAAGATCCTCATCAGCTATTGCAACAACAAAATTCCAACAACAGCAATTATCATCTTGATCAAGTAACCGATTGGCGAGTTCTCGATAAATTCGTTGCGTCGCAGCTCATGAGTCATGGTGATGATGATGGCCACAACCACAATAATAATGTTTCCAAAGAA

>AdNAC11--Aradu.3R7A3

TTGGCGACCAAGGATGCAAGGTTAAACTTGTAATTCTGGCCCTTACACGGTTTGCGTCTTTACGAAACTCGACTTTCTCTTTCCGGCTCAATATCCGAACCAATAGGCTCGGTTTTGCTTTGGCTCATTTGTTCGACAGAACAGCAATGACGAAGCACAGGTTCGAATGCACAGTCTCTTCCCATTACCAACAAAAACCCCAGGCTACAATTTCCCGCTCACTCTACCGACTGTCGCTTTCTTTGTCTCTCTGTCGATTGAAAATGGGAAACCGATGCTGAGGCTCAATACTCTCCACAGTTCTGCTTCAGCTCAAGCTTCTTCCTCTTCCTCTCTGCAATGGCTAGGTTTAGAAGGGGAAGGGAAAGAAGTGAAAGGTTTGAAGAGTAAAAGGAGCTGGCTTATTGACATAGGTGGATTTGCAAAGAAAGTGAAAAGCACTAATTTATCTCCAGCTGATCAAATCAAAGATTGTGGGGCATATCGTGATTGTCCAAACTGCCATTACCGTATTGATAACCGTGATGTTTCTACTGAGTGGCCTGGCTTTCCTCTTGGTGTGAAGTTTGATCCTTCTGATGTAGAACTCCTAGAACATTTAGCAGGAAAATGTGGCATTGGAAATGCTCAGCTGCATATGTTTATTAATGAGTTCATTCCAACAATAGAAGAAGAAGAAGGTATTTGCTATACACATCCAGAAAATCTTCCAGGTGTCAAGAAAGATGGGAGCAGTGCCCATTTCTTTCACAGAACAACCAATGCCTATACTACTGGTCAACGGAAGCGTCGAAAGATTCATCATGAATGTTTGACTGAAGAGCATGTACGGTGGCATAAGACTGGTAAGACAAAAGCTATATTGGAGGATGGAGTGCATAAGGGCTTTAAGAAGATCATGGTTCTTTATATAAGACCTAAGAAAGGGTCCAAACCTGATAAAACGAATTGGGTGATGCACCAATACCATTTAGGAACTGATGAAGAGGAGAAGAACGGTGAATATGTGGTTTCAAAGATTTTTCAGAAGCAAACTGAGAAAAATGAGGAGAATCCAGCGGTCGAAGATTCTGACCAGACTGAGAAAAATGAGAATCGATTGGCTGATGATTCCAACTGTATAGCATCCCGAACCAGTCCTAGAACTCCGAAACCAAATCCCCCAAATCCACCTCGAGCTGGAAATTTTGTTGACAATGATGATAATATTGACGAAACTGAACTTCCATTCACTCAGGATGTGAAATGTGTCCCACTATGTGATGTTCTGGATCAGAACAATGCTGGTGACCCTGCATGGCTGGCAGGTGAATCGCAGGCTGTGGAAAACTTCGACTTTGATGGCTTGGATGACATCTTGTTCTGCAATGAAATATTTGATTCATCATCTCTACTAGATGTTTCTGGAACGGAAACCATGATAAATGGATCTGCTTCAAACGATATGCTTGGGAATGATAGTTTATCATACGGAACTTCCGTTCTTGATACCCTTGACTTGGGTACTCCCCCAGATTTTGATCTTTCAAATCTGAATTTTTACTCTCAAGATAGTATTTTCGACTGGGTCGACAGATTATGAAGTGATTTCTGAAGTTTGAGTCCATCTGAATGCATGTTTGTTCAGCTCTTAGATGCTTCAGATTCAAATATAGTAAGTGAAACATAGGTTCTCACGTGATGATTTGCAAGCAGAAATAACAGTATTTGTTTGTCCCTGGTTCTTGAAATTCTGCAAATTTTGTAATCCCATGTAAACACATTTGGGCATTGTTCAGGTGGGTGTATTTTGGTGTTCATGTGGATTTTAGGCATTATTCACGTGGGTTGTATTTGTGTGTCAAATAGCTTTTGTGGTATATGTTGTTTCCATAGCCATCTAAACTTGGTTGCCTCATTCTGTTGTGGTAGATACATCTCTGGTAGCAAAGTCCAAACTTATATAAGTACTTGATATTTGACTGTTTTTATGTCAATACTAGAATAACTATCCGC

>AdNAC12--Aradu.46U1T

ATGGCAGAGCTAAGTGCGGCTGCAACCTTCACACCCAGCGATGAAGAACTCATTCATTTCCTCTCCGACAAGGTGAAAGGCCAATCCATGGACGAGGACGCCGCCATCAACATCCACGAATGTGAATACTTGTACGGCCGCAACAAGAACCCTTGGGACATTTGGCGTGACTTCGCGGGCGACGTTGATGCCGGCAGGACCGCCCTTTTCTTCTTCTCCCCCAACAAGAAGCACCATTCCACCGCCTCTCGCCCCATCGGAGCCGGCGTCTGGGAAGCTGAAGCAGAAACCATTGACGGCGAAGGCATCGTTGGCAAGGGCAAGAACCGCCGTATTGGGACCAAGAAATGTTTCATCTTTGACAAGAGTGGCACCTCCTACGATGGTGCATGGATCTTGCATGAATACACTCTTCATGGATCGTCGCTCCACACTAATACTTCAGTGGATAATAGCTATGTTATATGCAAATTGATAAAGAATGTAGAAGGTGAAGCTCATCCAGTTGAGGTGCAGTTTGGAGACAAAAGAAAAAGGCATGCGCAATCCGCCACCACCAGCGGCGTTCAAATTGATGTTAACGCTCCTCATTCATATAGGAACACTAAAGAACAAGAGGTCCAATTCATACCAAATGAACTTGGCAGGCGAATGTTGTTGGAAAGGTTTGAGGGATTACCAATCACGAACGAGAACCTCATCAGAAACCTCATGCAGCTGCAAGGGGGAACAAGAAGAGGTGGAAAAGGCCTCTAA

>AdNAC13--Aradu.47JQU

TTCTCTCAGCTTTTTTTACAAATCTCTCTCGATCACTTCCTTTCTGCATACTCGATCTCTTCTCTTTCGATTCTCATCACCAATTCCTAGAATTCACCCTTTCATTTTGGGGGTTCTTACATATCAAATGGAAAACATTTGTTCAGAGGTTGAGATGGATTTGCCACCAGGATTCAGGTTTCACCCAACTGATGAAGAGCTTATAAGTCATTACCTTTACAACAAGGTCATTGACACTAACTTTTCAGCCAGAGCCATTGCTGAGGTGGACTTGAATAGGTCTGAGCCTTGGGATTTGCCATGGAAGGCGAAAATGGGTGAAAAAGAGTGGTACTTTTTCTGTGTAAGGGACAGAAAATACCCAACAGGATTGAGGACAAACAGAGCAACAGAAGCAGGGTATTGGAAGGCCACTGGAAAAGACAAGGAGATATACAGAGGCAAATCACTTGTTGGCATGAAGAAGACCCTTGTCTTCTACAAAGGTAGGGCTCCCAAAGGTGAGAAATCTGATTGGGTCATGCATGAGTTCAGGCTTCATGGTAAATTCAATCCCCACAACCTCCCCAAATCTGCAAAGAACGAGTGGGTGATTTGCAGGGTGTTTCAGAAGTCTTCAGCCGCCAAGAAAATCCATCTTACCGGGATAATGAGGTTGGACTCTTCTGTTTTCTTGCCACCATTGGCAGATTCCTCATCATCACCTTCCAACACTGCTACTACAGCACCTTACGTGCCCTGCTTCTCCAATCCAATCATTCACAACCAAGTTGGGATCTTTGATCCATTTAGCAACACCCCTTTTGGTGCTGATTCATTCTACTCTTCTCAAGGGATGCCAATGCAACATGCTCAGCCACCAAGTTCCACATATTCCGCTTCCGGCTACACCACTCATGACCATTCAATTCTCAGAGCCTTGCTTCAAAACAATTCTTCAAACCTCAGGAGTGGTTTCAAGCCTGCAGAGAGGGAAATGTCCCATCATCAAACTTCTCTTGTTGATGCCAATAACAACAACAATGGAATCACTTCTGTTGTTGCCCCACAGGACCTTTCTAGCCTCTGGAATTACCAGGTTCAGATCAAGTAGCTATGGAAAGTGAATTCTGAGGGTACAAGAGAAGCCATTCTGTTGTGATTTATTCTCTGTCTTTTGGTGTTGTTGCTTGTCTTAATTGATTTTAAGTAAACCATGTATGAATATTTTGTGATGTCAGTAATAATCGTGTGAATTTATACTATTTTGGTCCTTTTGATACTTTGCTATTGTTATGGTTGCAATGACCTAAATTATCATTATC

>AdNAC14--Aradu.4RJ0E

CTTCTTCTCATTCATTCATGCCCCCTTCCCCTCTTGGCCTAATCTCTTTCTCCACATGGCGACAAATTCCGTCAACCATAAAATCCCCCTCACTTCTCACCTCCCCAAAAATTTCAATCCTCTCTTCTTCCCTTTTCCTACACTTTAGTTTGCTACACCAAACTTCTCTCTCCCTTCTCTTATTCTTATTATTCCTCACCAAGCCTATTAACAAATATTTTCTTACACCAAATTATTAGCCTCTCAAAAATGGGATCACCCGAATCAAATTTGCCACCAGGTTTTAGGTTCCATCCAACGGATGAAGAACTCATTCTTCACTACCTTAGGAAGAAGGTAGCATCCATACCCTTACCTGTTTCCATCATCGCTGAGGTTGATATCTACAAATTGGATCCATGGGAATTACCAGCTAAGGCTGCGTTTGGTGAGAAAGAATGGTACTTCTTCAGTCCAAGAGACCGGAAGTACCCAAACGGTGCGAGGCCAAACAGGGCAGCTGCTTCAGGGTATTGGAAGGCTACGGGTACCGACAAGACCATCGTGGTGTCGCCGGCGGCCACAGTTACACGTAGAGTAGGCCAAGAGAGCAGCGTTGGTGTCAAGAAGGCTCTTGTTTTCTACAAAGGAAGGCCTCCAAAGGGTGTCAAAACCAATTGGATCATGCACGAATATCGTCTTGTAGACAACAACAGACCCATTAAGCTCAAAGATACCTCCATGAGATTGGATGACTGGGTTCTATGCCGGATTTATAAGAAGTCGAAATTCTCAGTATCTTCACCGGAGGAATCACCGTCGAGTGAAGTACAGGCTGCAGAAGAAAATGGTTTATTCAAGAACACCATTTTAAGGAGTCCAATTCCAACACCGTCGCCATCGCCACCGCCGCCGCTGCCGCAGCCACTGCTCTCTCAAAAATCTGTGTCCTTCTCAAACCTCTTAGATGCCATGGACTACTCCATGCTCAGCACCATCTTATCGGAGAACAATAACAACAGCACCCTTGATCAGCAACAATACTCGCAGATCAACACCAACCAATTGAACCATTCATCGAACATGGAGAACACTAGTAACAGCAACATGATGGTGATGAGGTCAAAGCGCCAGATAGAGGAGGAAACAACAACGGTGTTGCACCCATCAAAGAAGTTCCATCACCAACTTATGGGCTCTTCTTCTTGCAGCTTCCCTAATAACATTAACAACACAAACACTGCACAATACGAGAACCCGCAATGGAACTACCTTGTCAAGCAATCCTTCTTGAACCAGCACTTACTTCTCGCTCCTCATCTTCGATTTCAAGGATAG

>AdNAC15--Aradu.58D1A

ATGATGAGCAAGAAGATGAGGTTTGTTAAGAAGAACAAGAATGGAGTGAGATTATTGCCACCTGGATTTAGGTTCCAACCAACAGAAGAGGAGCTTCTATTTCAGTATTTGAAATGCAAGGTTTTCTCTTTTCAGTTGCCAGCTTCAATCATTCCTGAGATCAATGTATGCAACTATGATCCTTGGGATTTGCCAGAGAGATACTTGTTCAGCTCAAAGGAAGTTAAGTATAGAAACGGTAACCGAATGAACAGAATAACGAAATCTGGATATTGGAAAGCAACTGGATCAGACAAAAGAATAATTTCAACATCATCCAATAATAATAATAATAATAATATTGTTGGGATAAGAAAAACTCTTGTATTCTATCATGGAAAATCTCCAAATGGCTCTAGAACTCATTGGATCATGCGTGAGTATCGACTTGTCACTACTCCTTCTAATTCATCCCAGAAGTATGTAGAAGACTTAGGGAATTGGGTTCTTTGCCGCATATTCAAGAAGAAAAGAAGCATAGAAAGTCAACATCACATGGTCAAGAACAAAATTAATAATAATGTTGTCGAGAGTTTTCAATAA

>AdNAC16--Aradu.5D5JN

GTTGGCGACTACTTTGTTAGTCTGATAAATAAAAAGTGAGCAAAAGCTAAAGGGGCCACTCACCCATCACACCCCAAATTCTCACCAAATATTGAATCGAATTATTCCTCTGATTCCTTCCTCATCTCTTCATTTGCTTTTATAATTCATCATCCCATTCTCTTTCGCTTCTTTTTTCTCTCTTTCGTTTCTCTCTGTTTTCGTCTCTGCTGCAAAATCATAAACCGGAAATATTAGAGCTCGTCCAATGGGAGCCGTTGTTGACTGTTATCCGCCGCACGCCGGCGAGGTTGCAGTTTTGTCTCTCAACTCGCTTCCCTTAGGTTTCCGATTTCGACCTTCCGACGAAGAGCTTGTTGATTATTATCTGAGACAGAAAATCAACGGAAATGGAGAAGAAGTCTGGGTTATTCGAGAAATCGATGTTTGCAAATGGGAGCCTTGGGACTTGCCAGATTTGTCGGTGATAAGAAACAAGGATCCGGAGTGGTTCTTCTTCTGTCCACAGGACCGGAAGTATCCAAATGGTCACCGGTTGAACCGAGCAACCAATCATGGGTACTGGAAGGCCACAGGAAAAGATCGTAAGATCAAGTCAGGTTCCACCTTGATTGGGATGAAGAAGACTCTGGTGTTCTACACAGGTCGTGCTCCCAAAGGGAAGAGAACCAATTGGGTCATGCATGAGTACCGCCCCACCCTCAAGGAGCTTGATGGCACCAACCCTGGACAGAATGCGTATGTACTCTGCCGATTATTCAAGAAACAAGATGAGAGTCTTGAGGTTTCAAACTGTGATGAGGTGGAACAAACAGATTCCGCTCCCATGGCGGCCAATTACTCCCCTGAAGAAATACAGTCTGATCAGGCTCTGGCTGAAGTATCGCCGTCTCAAGTTACAGATGAGAAGCACCAGGGTGTTATCCCTGAGATCTCTGAGGAAGCGGTTTCCAACGTTATAACCTCTGCTGATTGCCATAGTGACGGATATGATGCTTGTGAAAGGCGAAATCAAGCTTTTGAACTACCTGCTGAGGACATTCCGCCGTTGAATTGGGACATATTCAATGACCCCGAGGACAAGATATTTGATGACAAATTATTCTCCCCAGTCCATAGCCATATTCCACCAGAATTTTACTACCAAGCAAACAATGAGACAAATATTGCAGACATCTTAAATTCTGTCAATTGGGATGAGATCTCCTATGAGGATCCCTATAGTCAAGCACAGAACAACTTTTTTAATAATGTTAAGCAAAGTGTATCAGGTAGCGAACCAGATGCAGGGCTGACCAATATGACATGCATACACCCGACGAATGTTGTTTATCCCGAGGAGGCAATTCACAGAAAGGTTGCTTTGGCAACAACTCCGCAATTTTGCAGCACCTTCACGTCTGACTTCAGTGCTGATGAGCAGAAGAGCAGTGTCGCGTTAATTCAAAACAATTCCCAGATGGCTTCTTTTCCGGATGCCAGAACAGGCCAAGTGTATAACGTATTCAATGATTATGAGCAGCCGAGAAACCTTAATACCTATGTTAGTGGTGATACTGGAATCAAGATAAGGACTCGACAAGTGCGAAATGAACAACCAGCAATGATCTTTACAGATCAAGGTAATGCAGCAAGGAGAATCCGATTGTTAAAGCAGTGTGCAAATGTCTCAAACAAGATGGCAGATGATGGGAGTCCTAAACAAGAGCATGATTCAAAACCAATAATTGCAGGGAACAAAAACAAAACTTTCAAAAGTCACACTGCAGATAAGCATGATACTGCTAATGATCTGAATGAACGCCAGGAGAAAACTGAGTCAACTGATAAAAGAAACATGATATCTAAACTTGCAAAAGGAGGTTCTTCCATGTTGGGGTTGAAGGGATTATTGCGCAGAAGGCTTAGTTACATATCAAAGGCCTCCTCCAATTTCAAAATGTGGTCATGTGTTGTTGTGGCTTCTGCCTTTGTATTGGTCTCGTTTCTGTTCTTTGCTAACATATGGGGATATATTAACTTATGAACTTCTAGGAGATCATTCCTTTATGCGTGTAATGTGCCTCCATTTTTTTTTTCCTTTCTTTTTTTAGGGCCAGTTGTGGAGGCTTTTAGGATTGACTCCTAGATTTGTTGATAACTCTATTATGAGAGAGTATGCGTTTCAATATGTAGCTGTATAATATACTTAGTTGCTTACCTCGCTGGCTAGTAGAATTTAGATCAACACTTGTGCTACTGTTTTCTTCTCTCTTAATTACTTGCTTACTGCCTATGAATGCCAAGAAAGTTATTTGTGAAATAATGATGAGAGT

>AdNAC17--Aradu.60U13

TGGTAACACTATATGGTGATGCATGCAGTTCAAGAGGGTTAAAATCGTCATTGCAGTTAGTTCAACATTGCTAGGTGTCTCCTTAGAATTTACAACTTGCTATAAACCTGTGGCAACTTGATGATGAAGACCCACATTTGGTGGAACCTTGAGAAACACACTTATCCATGGTTTTTCTTTCTTCATTTGCCTTTGTATACTTAAACCCTAATTAACTTTAGTTTCTTTCATGCATGATTGATTAGTAGTAGCAGTGATAACGAAACACCCTACTCTTCCTTCTCTTTTAATTTGATCTTATTATTTTGTGGTAACCCTCAAGTCAAGTTTTTTCTTCTCAAAGTGAATCATAAAAATTCAACCCAAAATTGAACAAAGAGTTTTGTCTCTCCATGCAAAAGGAAAAGGAATCAATCACCACCAATAAGGAGGGGACTAATAAGAAGGAGATAGAAATAATGGAAGGTTGCAATGGAAAGGAGGAAACCCTACCACCTGGGTTTCGATTTCATCCAACCGACGAAGAACTCATTACTTGCTATCTCATAAACAAGATCTCGGATTCAAACTTTTCAGGCAGGGCAATAACTGATGTTGATCTCAATAAATGTGAGCCATGGGAGCTTCCAGGGAAGGCGAAGATGGGAGAAAAAGAATGGTACTTCTTCAGCCTGAGGGACCGGAAGTACCCAACTGGGGTGCGAACGAACCGAGCCACGAACACCGGGTATTGGAAGACCACCGGAAAAGACAAAGAGATCCTTAATAGTGTTACATCGGAGCTAATTGGGATGAAGAAAACTTTGGTTTTCTACAAAGGAAGAGCCCCCAGGGGAGAGAAGAGTAATTGGGTCATGCATGAATATCGCATTCATTCTAAATCCACCTTTCGAACAACCAAGCAGGATGAATGGGTGGTTTGCCGTGTGTTCCAGAAGAGTGCCGGGGCAAAGAAGTACCCTTCTTCCAACCATGCAAGTAGGGCAATGAACCCTTTCAACCTTGAAATAGGTCACCACAATATTGTGCCGCCGCCGCCAATGATGCAACTCGGAGACCCTGCCGCCGCTCATTTCCTCTATGGAAGGAACTATATGAATACTGCAGAGTTAGCAGAAGTAGCTAGGGTTTTGCGTGTTGGTACTGGATCAACCAGTACCAACCTGCCCGGGATGCAGCCTCAGATAAATTATCCAGTGGCTGCGTCTCCAGGAATTGGATTCACAATTTCAGGACTCAATTTAAATCTAGGAGGCGGAGGAGGCGAAACAGTAGTGGCCACAACACAACCAGTTTTGCGGCCCATGCAGCCGACTCCTCCGTCCCAAACATTGGGTATGGTTCCTCATCATCAAGTTCATCATGATGTGAGTTCCAACATGATTTCAACAAATTCCCTTGGTGCTGAGAATGTGGGTTACGTCAATGAAATAAGCAACACAAATGGTGGTCATGGAAATAGGTTTATGGGCATGGATCATTGCATGGATCTTGATAATTACTGGCCTTCCTACTAAATAAAGGAGAGAGACCCTACTTCATTTTGTTTAATTAATTATTATACATAATTTAGTGTTATAAGCTAAGTTGGATAATTAAGCTACGTAAAAC

>AdNAC18--Aradu.66XRP

AGACTATATATGCATGAGATATAAATAGAGAAGAGAATAATAATAATATTCATTCACTGATCCATGATCATGGTGGATAATAGCACAGATTCATCATCAGGAGCGGGTGATCAGCATCATCACCCTCAGCTTCCTCCAGGCTTTCGATTCCACCCCACAGACGAAGAACTCGTCGTTCACTACCTTAAAAAGAAAGCTTCTTCTTCACCACTCCCTGTCGCCATCATCGCCGACGTTGATCTCTATAAGTTCGATCCATGGGAGCTCCCAAGTAAGGCAGCGTTTGGGGATCAAGAGTGGTACTTTTTCAGTCCTCGGGATAGGAAGTACCCGAATGGAGCTCGGCCAAACAGGGCGGCTACTTCTGGGTATTGGAAAGCCACCGGGACGGATAAGCCTATTCTCTCTTCTGATGGGAACAAGCAGAAAGTTGGAGTCAAGAAAGCGCTTGTTTTTTATGGTGGCAAGCCCCCCAAAGGTGTCAAAACCAATTGGATTATGCATGAGTATAGGCTCACCGATAACAATAACAATGCTTCTTCTTCTATTTCATCTAAGCCTCCTTCTATCCCTCTTGATCCACTCAAGAAGACTTCTCTCAGGCTTGATGATTGGGTTTTGTGCCGAATATACAAGAAGAGCAACAGCAGTAGTAGCAGCCTTCCAATTCCAAGGCCAGCGTTTTTAATGGATGAAGAGAAGGATCTAATTTCCATGGAGAACAGCATGGTGCCAACTATGTCAATGTCAAAACCAAGAAGCACTTCAACAACAGGTTGTTATGGACCCATGGCACTTGAAAACGATGACAACTTCTTCGATGGTATATTGGCAGCATCAACCGATCATCACACCATGCAAAATGGGTCTCCAGGGTCCTCATCTTCAAGCAAGAGATTCCATGGTGATCTTAATAATGGAGACAACACCTCCTTCGTTTCTCTTCTTAACCAGCTTCCTCACAACACACCGTTTCACCCAAACTCCATTCTTGGCTCCGTGGGAGACGCTGTCTCTTGA

>AdNAC19--Aradu.6H4PP

CTTTAATTAACCTTGTGATATTATGACATGTTCCAAGTACCGCCGCCTTTATAATTTCCAGCAATCTATCCATCCATTATTATAATTGTGAAAAATAATCAACCCGAATCTTTATCTCTCTACTCAGCATATATATAGTGACAAAATAAATGGATTATGGAAGTGTAGTTATTACTATACCAAACTCAACAATTCATACATACACCACCAATTCTATGATGATGATGACTGCAGATTATGAAAGCGTGAAGCAGCTTCCTCCTGGGTTTTTGTTCTCTCCAACGGATGAAGAACTTGTCCTTCACTTTCTCTATGCCAAGGCTTCTCTTTTGCCATGCCATCCCAACATCATCCCTGATCTTGATGTCTCTCTCGCTCATCCTTCCCAACTCAACGGTAAAGCGTTGTCAAGCGGAAATCAATACTATTTCTTCAGCAAAGTGAAGGAAAAAAGAATAACAGAAAATGGGTATTGGAAGGAAATAGGTGAAAGTGAAGCAATATTGTCATCAACGTTTGAGAAGAAAGTAGGGACAAAGAAGAACCTTGTATTCCACATAGGAGAAGCTCCACACGGCATTGAAACCAGTTGGGTCATGCAAGAATATCATATTTGCCCATCCTCTAACATTATTTCTACAACTAGAGCCAGAAGAAAACACGATCATCAAATTTGGAGCAAATGGGTTTTGTGCAAAGTGTATGAAAAGAAGGGGTCCGTACGAGGTGTAAACTACTGTAGCGACGATGATGACAGTGGGACAGAGCTATCTTGGCTTGACGAAATTTATCTCTCGTTGGATGATGATCTGGAAGAAATTAGCGTCTCCATTTTAGATTGAATATGTATCAATTACCTGAATTTTAGGTTTAATATATATGTAACGGTGCCGCTATATATGTTGATACTACTGTATATTGAATTGGGAGTTCAATGAAAATGATTATACCTTCACTAAATATTTTAAATGGAAGACAATTGAACAATAAATTCTAAAATTTGGTTCAATAAAATAAAAATACATTGCATTTTATTTTATCCACCTAAATAAAATACTTTA

>AdNAC20--Aradu.79PL2

GCACGATAAGAGAAGAAGATTTGTTTAATTAATTTGTCAATAGAACAAATTAAAGTGGATGCATGCTATCATATATACTATCTATGTGTCGCTTTTCTCTTGTATAAATATAGTTCCATGTTAGCTTGACTGAATTTCATCGATGTCCATGCATGTATAGTGTCACATAGAGTAGAGAGAATATAATACAATCAAATAATGGAAAAGCTAAATTTTGTGAAGAAGAACGGGGTAAGTAGAATGCCTCCTGGATTCAGATTCCAGCCAACGGATGAAGAGCTTGTGTTTCAGTATTTGAAATGTAAGGTCTTCTCATTCCCCTTGCCCGCTTCCATGATTCCTGACATCAATCTCTCCAACTATGATCCTTGGGATTTGCCAGGAAATTGTGATGAACATCAAGAGATGTATTTCTTCAGCAGCAAGGAACCCAAGTATAGAAATGGAAGCCGCATGAACCGAACAACCACCACTGGCTATTGGAAGGCAACAGGATCCGACAAAAGAATCATTTCATCTTCTAATAATAGTGACGATAATAGCATTCTTGGCATTAGAAAAACCCTAGTGTTTTACCAAGGGAAATCTCCCAATGGCACTAGAACTCACTGGGTCTTGCATGAATATCGCCTGGCTAGTACTACTCTACATGCTAATAACAATGCTTGCGATATAGGAGATTGGGTTCTGTGCCGCTTATCGGTGAAGAAAAGGAGTGTTGGGAGTGGTAGCATCATCATAAGCAAGAAAGCACGTTCTTCAGCATCTTCATCCTCATCTTCTTCCACTTCAAGTAATAACGTCATGGAAGTATCTTCTTCATATGCTTCTTAATATCAACGCAACAAACAATGCACTTCCTCCTGATGGTCTTGCTGACTATTGATTAAAACCACCATACATATTCTCACTCAGACCCCGTCGATTCTGCAACTAAATGGATTCATACCCTTTAAAATTAACAA

>AdNAC21--Aradu.7NI41

GATCCCTCTTTTGAAGTACATACTCAACCCTTCTATCTCTCACACACACAAACACATACAACTTTCTATCTTTCTCTTTTTAGTTTTTGTTCTATTTGTTTTATAGAACAAGAGAAAAACAAAGGAAGGAAAAGAAGATCTCCATAAGTGGAATGTTGTGTGAGAAGGATAATAAGGAGGGAAATTAAACAATAAAGAGAAATGAGTGAGTCCAATGAACATGAAAACAATCATGGCAACATCATAGTGGAGGGAAGAAAAGACAGTTTAATTAGAACTTGTCCAACATGTGGTCATCACATCAAATGCCAAGATCAGGGTGGTGGAATTCATGACTTACCTGGACTTCCTGCTGGAGTGAAGTTTGATCCAACAGATCAAGAGATTCTTGAACATTTGGAAGCAAAAGTGCGATCTGATATTCACAAGCTTCACCCTTTAATTGATGAGTTCATCCCAACTCTTGAAGGAGAGAATGGAATCTGCTATACTCATCCAGAGAACTTGCCAGGAGTAAGCAAGGATGGGTTGATCCGGCACTTCTTCCACCGGCCGTCGAAAGCATACACAACCGGAACAAGGAAGAGGAGGAAGGTGAATTCGGACGAAGAGGGAAATGAAACCCGTTGGCACAAAACAGGGAAGACCAGACCAGTCTATATTAGGGGGAAGCTGAAAGGATACAAGAAAATCCTTGTTCTCTACACAAACTATGGTGGGAAGCAAAGGAAGCCAGAGAAAACCAATTGGGTGATGCACCAATACCACCTTGGCAATGATGAAGAGGAGAAAGAAGGAGAGTTGGTTGTTTCCAAAGTGTTCTACCAAACACATCCTAGACAATGTTCTTCACTCTTGATCAATAACAACAAAGACTCTTCAACAGCCGCACTTGTCAAGGGTAATAATAATAACGGGTTTGTTGAGTATTACCATTCAAATTTCATATCATTTGATCAAGGGGAACACCAACATAGATCTAGTGGGGCTCAAGTCGTCATTTCACATTTTCCTGTCCATGAAGGTGCTCCTAATTATCATTCTTTGAATCGAAAGGAGTAGTGTAGAAAAAATGGTAAATTACCATTTTATCTATGAAAGAATTAGCTTGCTGATAAAATGGTTTTTTGGAGATTGATAATTATTTTATGGTACTAGATTTTTTTTTGTTTTGTTTAACAAAATTACTTAAAGCGATCAAAATTTGTTGTTGAAATGGTAATTTACTTGGGGAAATAGACATATAAGGTGAAAGAGTTTGTGAAAGGAGCTGAGGAGAGGAAAATCATAAATAGGAGTTTTCTATATAATACTGAGGCATTATTGTAACATATTATAAAAAATAGTTAAGAGGGCACGTAAGTGA

>AdNAC22--Aradu.7X5EV

GTGAGAGATCCATAAATATGGAGAGCACCGACTCGTCCACCGGTTCGCAACAACCGAACCTTCCACCGGGTTTCCGGTTCCACCCCACCGACGAGGAGCTCGTTGTTCACTACCTGAAGAAGAAAGCTGCATCAGCTCCTCTCCCAGTCGCCATCATCGCCGAGGTTGATCTCTACAAGTTCGATCCATGGGAGCTACCAGCTAAGGCAGCGTTTGGGGAGCAAGAGTGGTACTTCTTTAGCCCAAGGGACAGGAAGTATCCGAACGGTGCTCGGCCAAACAGGGCGGCAACTTCCGGGTACTGGAAGGCAACCGGGACGGATAAGCCGGTGCTGACCTCCGGTGGGACCCAGAAGGTCGGCGTGAAGAAGGCTTTGGTCTTCTATGGAGGGAAGCCCCCCAGAGGGATAAAGACAAATTGGATCATGCATGAGTATAGACTTGCTGATAACAAACCTAACAATAGGCCTCCTGGTTGTGACTTGGGTAACAAGAAAAACTCTCTAAGGCTTGATGATTGGGTATTGTGCCGAATCTACAAGAAGAACAACACACATAGGTCTCCAATGGAACATGAGAGGGAAGATTCCATGGATGACATGATTGGAGGGATTCCTCCTTCCATCAACGTGGGCCAAATGAATGCAAGATTTCATCTCTCAAAAATGTCAACAAGCTTTAGCAACGCTTTGTTGGAAAACGACCATCACCATCACCAGAATCTTCTGGAAGGTATGATGCTAGGAGGAGGAACCAACAACAGCAATCCAAACATGTTGGGATTGGGATCAGCCTCAAACACCATTAACAATAATAGTAATAAGGCAGAGCTTTCATTTGTACCAACCATGACTACATCTTCAAACACCAAGAGGACTCTATCATCACTCTATTGGAATGAAGATGATGTTGCTGCTTCCAACAAAAGATTCAATTTGGAAAGTGGAGATCATAACCATGGAGAGAATAATGGTACTAGTGCTAGTTCTATTGCTACTCTG

>AdNAC23--Aradu.ZT2TE

ATTTGTTTAGGGTTAGGGCTTCCTTCCTAGCTAGCTAGCTAGCTACTTCATTCTCTATACTTGATCCAACAACATGAATCAAAGTAGACCAACAAATCATTGAGGACATTCGTCTGAGTTAGGAGCTTCATGATCTTGGGAGGCAGATTTGTTACTGCACCACCCTCATCTCTTATTACCTTGCTTCATCTCTAATCAGATAGAGAGAAGATGAATACATTTTGTCATGTTCCACCGGGTTTTAGGTTCCATCCAACGGATGAAGAACTCGTTGATTACTACCTTAGAAAAAAAGTTAATTCATGTAGGATTGACCTTGATGTCATCAAAGATGTTGACCTCTACAAAATCGAACCCTGGGATCTTCAAGAGCTATGCAGACTAGGAACAGAAGAGCAAAATGAGTGGTACTTCTTTAGCCATAAAGATAAAAAATATCCAACAGGAACTCGCACAAATAGAGCAACTGCAGCAGGGTTTTGGAAAGCAACAGGGAGGGACAAAGCTATATATTCCAAGCATGACTTGATTGGGATGAGAAAGACTCTCGTCTTCTACAAAGGTCGAGCCCCTAATGGCCTCAAATCTGATTGGATTATGCACGAATATCGTCTTGAAACCGATCAAACTTCGGCTGCTACTCCTCACGAAGAAGGATGGGTTGTGTGTAGAGTGTTCAAGAAGAGAGTGACTTCCATTATGCGTAAGATGAGTGATCATGATTCCCCTTCCTGCACTTGGTATGATGACTCCTCTTTCATGCACCAACAACCAGATCACTTTGACAACTCTTGTTCTTCTTCATCAAAGCACCAACTAATCCCTAATAATAACTGTGATGTCTTCTACCAACAACAAAATAACAACTTGCCTCTTCATCATCTTCCACTTCTTCATCAAACTGCTGCTCTTTCCAATCATAATAATAATCCAATCATGGCACCACCATTTGCTGCTATTAATAATAATGAAACTACTGCTTTTCAAGAACAAGGGAAAAGCTTAATTCATCATCAGGCACTACTCTATGGAAATTTAAATGAAGAGCAAGCTTCTTCTTCAGCTGCTGCTGCTGATTGGAGACTTGTTGACAAGTTTGTTTCATCACAGCTTAGAGAAGATCATCATGTCTCCAAACAAGAATTGATGATGCCAGAAAATAATAATAATAATAATAATGATAATGGTGCCTCAACATCAAACTCAAGCTGTCCAATAATGGACGTGTGGAAATAG

>AdNAC24--Aradu.8Q7DY

ATGCCAGGTTTCAGATTTCACCCAACAGATGAAGAGATTGTTGGTTTTTATCTAAAAAGAAAAATTCAGCAAAAATCTCTTCCTATTGAATTGATCAAGCAAGTTGATATCTATAAGTATGAGCCATGGGACCTTCCAAGGGTGGCAAGTAATGGAGAGAAAGAATGGTATTTCTACTGTCCAAGAGACAGGAAATACAGAAACAGTGCAAGGCCCAACAGAGTGACAAGATGTGGGTTTTGGAAGGCCACTGGAACTGACAGGCCCATATACTCATCTGAGGCCCAATCCATTATTGGTTTGAAGAAATCACTTGTTTTCTACAGAGGCAGAGCTGCTAAGGGTTTCAAAACTGATTGGATGATGCATGAGTTTAGGCTCCCTTCACTTTCTTCTGATTCAGCCAAGAAATGCTCTGACAAAACTACCCCTGCTTCTGATTCATGGGCAATATGTAGGATATTCAAGAAAACAAACACAATGTCCATGGCGCAAAAAGCCTCATTACCTCATCATCCTTATAATTGGAATCATCATAATCAATTATTTGATGATATACTCACACATCAACAACACCAACACCAACACCCTATTATTCCAAACTCCAACAACAACTTCATCTTCTACAATTCCAATTCTACCCTTGAACCCACAAAAGAAATTGATGCCACTACTACTAGTAGCTCCATTGTTATTTCTTCCAACATAGGCCTTCATGAAGATCCAAATCATCATCATTACAATAATAATAGTAGTGGGTTCTCATATGATGATGATTCAGGTGTAATTACAACAATTGCTGGGTTCCCATTCAATTTGCCTCCAAATGATGATGATGCTGCTGCTTGGAATAATAATAATAATAAGCCTAATACTACTCTGCCATGGGATTACTCATCAGACATGTCCACTACCTATTCCACTAATAAATCTTACACTTAA

>AdNAC25--Aradu.9FF24

AATCATACATGAGTCGAATACTCGGTCCCGGTTTCCGCTTCCACCCTACGGACGACGAACTAGTTCAATACTATCTCCGCCGGAAGGTCATCGGAAAACTCAACCACCACGACCACATCGGCGTCATCAATATCTACGACTATGAGCCATGGCAACTCCCCGAATTGTCGAAGCTGAATACTAGGGATTTGGAATGGTATTTCTTCACGGTTCTGGACAAGAAGTACGAGAAGGGGGAGAAGACAAAACGCGCCACCGTCAACGGTTACTGGAAGACCACCGGCAAGGATCGTGGAATCAAGTATGGCGATCGCCAAGTAGGCATGAAGAAGACCCTCGTTTACCATGAAGGAAGGGCCCCGACTGGCAAAAGATCAAATTGGGTCATGCACGAGTACCGGATGGTCGATGAGCAATTGGCGGAAGTCGGATATCAGCTGGACGCTTTTGTGCTGTGTAGAATTTTTGAGAAGAGCGGGATGGGCCCTAAGAATGGAGAGAAGTATGGTGCTCCCTTTAGAGAGGAGGACTGGGTGGAGGATGGCGACCTGCTTGAACCGATTGCTGATGAACCTGTGGTTGAGCTGTCTGTTGACCAGAGTGATGCTTTCCTTGAAACTGATGACCTTGAGAAGAAACTTGGTACGCATGTGGTCGATGGAAGTGCTGATTTACCACCAAACCCTCCCAACTACTTTTATGGGGAGTGTAGTCACTATCCTCAGCATCAAGAAGAATTTGTTGAAGTTCCGAAACCTTTGGAAGGCACTGAAGGCCGGAATTTCGATGTAACTGGGCCATATGCTGAGGATACCTGTTTAGAAAATCATGAAATGAACCATAATGGGAATTCTTCAGAATTCATTTATGGTGACGTTAATTCAGATGAATTCATGGATTCCATTGTTGATCCTCTGATTGGTGCTGAATTATTCCTGGAAACAGATGATCTTCTGAACCCAATCGAGGGAAATTCCTCTGGGGCAGATCCTTATACAGTTGAGGGAAATCACCCTAGGGCCGATCCCTACGCAACTGAGGGAAATCCCCCTGGGCCAGATCCCTACACAGCTGAGGGAAATTATCCTGGGACAGATCCTTATGCTGTGGATATGTTAGATGAGTATCTTGCACTTCCAGATGATGATATTCTGAGATATATATCTTTTGATGATTCTCCTCCATCAATGGAGGGTGAACACCCTATTCTAGAGCAGATACCACCTCTTATCCAGCAGAATGTGGAGGAAGAGGCCAAGGATGTTTCAGAGGAGAAACAACAAAAGGTGGAGGGAGAAGCTGCAAATATTTTCAAGACAAACAAACATGACCTTGAAGCAAATTCTAGCCGTGGAGGAGCTGCTTCAGATGATGCAAATCCAATTGCAAAACGCTTCAAGAAATGGTTGGAAGACATCCCAGCTGCTCCCGCATTTGCTGCAGAGCTTCCATCCAAGAAGGATGCACTCCGGCTTCATTCTGCACCTCAGTCTTCAAATACTACTCATGTAACTGCAGGAATGGTCAGCATTACAAACATTACTGCAAGAGGCAATCACATGAATCCGATGGTGGAAAAGATTGGAGGAGGGTTCAACCATCCCATTATCTCTGCTGTTGTTTTGATACCTGTTTCTGGCTTACTTTGTGGCAAGACTCTGTTTGTGCTGACATATGGATGGGCTTTTCTGGTGACGTTTTCATTTCTGTTTGCCACCGTGACTTGCAAAATTGGAACCTTCATGTATTCTGGAAAATGAGAGTTATGGGGTCTGCTGTGAGACAATGGGGTGCGGTTGGTTATCAATTGGTAGTGATGGTACTCATTGTGACTCAAAGATACTACTCTATTTGAAAGTTGCACAACGAATACCCATAACGGCCTGCTGATATCCAGCCACCGCCCATTGGCTCCTT

>AdNAC26--Aradu.9T4H8

ATGGGGGATAGCAATAATGTCAATCTTCCACCGGGGTTTCGATTTTATCCCACTGATGAAGAGCTTGTAGTCCATTTCCTTCAGAGAAAAGCAGCACTTCTACCTTGCCACCCTGATGTCATTCCTGATCTTGATCTCTACCCTTTTGATCCATGGGAACTTGATGGTATCATTCTTTCTCTATGTTCCAATACTTGCATGCACATTGCAAGAGAATTGAAGTTGATGAATACAATCATGTGTGTGTCTGTGTGTGTATGTGCAGGTAGAGCTTTGGCAGAGGGGAACCAATGGTACTACTACAGCAGAAGGACACAAAATAGGGTCACTGCCAATGGTTATTGGAATCCAATGGGAATTGAAGAGGCAGTGGTTTCAAACTCAAGCAACAGGAGAGTTGGTATCAAGAAATTTTATGTGTTCTATGTTGGAGAAGCCCCTCATGGTAACAGAACCAATTGGATCATGCAAGAGTATCGTCTTTCAGATTCTGCAGCATCCTCTAGCAGATCATCAACCAAAAGAAAATCACAACCAAAAACAGATCATAGTAAATGGGTGGTATGTAGAGTTCATGAAAGTGATGAAAATGATGATGATGGTGATGGTGATGGAACAGAACTCTCTTGTTTGGATGAAGTTTTCTTGTCATTGGATGATCTTGATGAAGTAAGCTTGCCAAATTAG

>AdNAC27--Aradu.9Y6NH

GTGGTACTTAATGATGAAAATAATGTGAATTAATTGGGTTTGATTTCTAATTAAGTTGGTAGTTGTTCTCTTTTTAAGAGCTTCATAGCAACGCGATCCTCGCAAAACACACGCTCCTTTAAGAGTCACCACAAAAGACGGCTGAACCTTCGGGTGCAAGATCCCTTCTCAGGAAGCATATAATCACTTCTTCACTACTTCAACTGTGTTCTCTCTCTATCTATCTCTTCATTATTAGGGTTTTCATTTCTATATATCCACCTCTTTCAACTCTCTACTCTTTCTTGAGGAGATGGAAGGTGAGAAGCTTGACGAGATCATGTTACCAGGTTTCAGGTTCCACCCAACTGATGAGGAGCTTGTCGGGTTCTACCTTAAGAGAAAGATTCAGCAAATGCCTCTGTCCATTGAGCTCATCAAGCAACTTGATATCTATAAATATGATCCTTGGGATCTTCCAAAAGTGGCAGGTACAGGAGAGAAAGAGTGGTATTTCTACTGTCCAAGAGACAGAAAATACAGGAACAGTGCAAGGCCAAATAGGGTAACTGGAGCTGGGTTCTGGAAAGCCACAGGGACTGACAGGCCTATATACTCCTCAGAGGGTTCAAAGTGCATTGGACTCAAGAAATCTTTGGTCTTCTACAAAGGCAGAGCTGCCAAAGGTGTTAAAACTGATTGGATGATGCATGAGTTTAGGCTTCCTTCTCTTGTTGACTCTTCTTCATCCGACAAGACCACTATTCCTGCTAATGTCGGTATTTATCACTCTCTCAAACCTTTCTTAACAGGATCCATCTCACACTTTCTAGCTGACTTCAATTTCAACATGCAGGACTCTTGGGCAATCTGCAGAATATTCAAGAAAACAAATGCTACAGCTCAAAGAGCACTCTCTCACTCTTGGGTTTCTACCTTACCTGAAACAACACCAACCACCACTACCAATGATACAGATCACATATTCAACATGCCAACGATGATGGCAAAGAAAACTAGCTTCATGACCCAGTTTTGCACTAACTACACTAGTGACACACAAATCCAAGATGTTGCATCATCTTATAAACCACCCTTTATTAATATTAATCCATTGCTTTACAAGCAGTTTGATCATCATCATCATCAGTTACCACCTATTATTTCAAATGGAGATCTTATAAGCAACGACTGCTTAATACCCTCTTCTACTACTCCACTTGAAACATCCTCTAATAGTGCAAAACCTACTATGGATTTTTCTTCATTGTTGCTGAACATGTCATCTTCTGTTCTTGGAGATTTTGCTGGAAAGACATCGTCGTCGTCCTCATCCCAAGAGGGTACAGCAGCAGCAGCAACAGCAACAACAATCACGAGTAGCTTCGGTGGTGGAATGCAGGAGCACTACCCAACAATACCATTACTGCGTCAGATGCATCAAGGGAACAACAACAACAACAACAACATTGGCATCAACAACAACAACGTGTCTGCTGGCGGTGAAGAACAAGAGTTGGAGAAAGTTGGATCCATTGTTGGGTTCCCATTCATGAACATTGGGGATGCATGGAAGTCAAATATGCTTTGGGATACTTCTTGTCCCTTGTGA

>AdNAC28--Aradu.AF9FZ

CGGGATCTGAAACGCTTTCCATTCCCGACGGACTTATTGTCTTTGCTACTATCTTTTCATCTCATTCTCAAACTCAAACTCAAGCACTACTCACCATTTCCGGAGCAAGATTATCGATTTCTGAGTGAAAAATTGCATGGAGAATATGAATAGTTTTTGTCATGTTCCCCCGGGTTTTAGATTCCACCCCACGGATGAAGAACTTGTTGATTACTACCTTAGGAAGAAGGTTAGTTCAAGGAAGATTGAGCTTGATGTAATCAAAGATGTTGACCTCTACAAAATTGAGCCATGGGACCTTCAAGAGATATGCAGGATAGGAAGAGAAGAGGAGAATGAATGGTACTTCTTTAGCCACAAGGATAAGAAGTATCCAACAGGAACAAGAACAAATAGGGCAACAGCAGCTGGGTTTTGGAAAGCAACGGGAAGAGACAAAGCTATATATTCAAAGCATGATCTCATAGGGATGAGGAAGACATTAGTCTTTTATAAAGGTAGAGCTCCTAATGGACAAAAATCCGATTGGATCATGCACGAATATCGCCTTGAAACTGATGAAAATGCCGCACCACAGGCAAGTTTAATTCTATATTTTTCGGTCATAGAAATATTTGCATACATACATAATTAAAGACATGAATGCATGCATGCATGGATGCAGGAAGAAGGATGGGTGGTGTGTAGAGTATTCAAGAAGAGAGTAACAACGATGCGTAAAGTGATGATGAGAGAGCATGATGAGTCTCCTAATTCTTCTTGTTGGTACGATGAGCAAGAATTGATGATGATGGAATCGCCAACAAAGCAACAATCCTCTATTCTTCTTCATCAATCCACCAATAATAACCATTCCAATTTGATGCAGCTACCACCATATCCTCTCATCAAGAAAGAGCTTCATCACCCATCATCATCATACCCCTTCCTTCAGCTTCCACTCTTAGAGTCTCATCAACAATCTGCTGCTGCACCTTCCTCCATTTCTGAACAACTCATCATGCCACCACCAATTGGAGGAGGAGGAGGAGAACAAGTCCCTAGTTTTCAGTCATTCTTCAATAATGAACAACAAGAAGTAGGAGTTCTTGATTGGAGAGTTCTTGACAAGTTTGTTGCTTCACAACTTAGTCAAGATGATAATCATGCATCCTCTAATAGTATTGTACAAGATCTCACACAGGAAATTGTTATGGTGCCTCACAATGATGCTGCATCAACATCAAACTCCCTCACCTGCCCAATTGATTTGTGGAAATAG

>AdNAC29--Aradu.B5XXI

ATGAATAATAATAAGATAAGCAACATGAGCTCCGTGAGTAGCTCCGATCTCATAGATGCCAAGCTTGAAGAGCATCAGTTGTGTGGAGGATCCAAGCAGTGCCCCGGTTGCGGCCACAAGTTTGAATCCAAACCGGATTGGCTAGGTTTACCAGCAGGAGTGAAGTTTGATCCAACAGATCAAGAACTAATAGAGCATCTTGAAGCCAAAGTGGAGTCAAAGAACATGAAATCACACCCTTTGATAGACGAATTCATTCCCACCATTGAAGGTGAAGATGGGATTTGTTACACCCATCCTGAGAAACTTCCAGGTATGCCTTCAAAGGCATACACAACAGGAACAAGAAAGAGGAGAAAGATTCAAAATGAGTGTGACTTGCAAGGTGGAGAAACGAGGTGGCATAAGACCGGTAAAACAAGACCGGTCATGGTTAACGGAAAACAGAAGGGTTGCAAGAAGATTTTGGTACTCTACACTAACTTCGGCAAGAATCGGAAGCCGGAGAAGACGAATTGGGTCATGCATCAATACCATTTGGGGCAGCATGAGGAGGAGAAAGAAGGAGAGCTTGTTGTGTCCAAGATATTCTACCAAACTCAGCCTAGGCAGTGTAATTGGTCGTCCGATCGGAGCGCCACCACCACCATCGCTACGGCCGAAGGGAGTGGAGAGCCACTACAAAATAGTAGAAGAGATAGCGGAAGTGGAAGTTGTTCTTCTAAGGAAATTAACATAGGTCATAAGGATGAGATGTCTGCTGTGGTTGGAGTTACTAATACTCCAATCACAGGCTTCGCTCATCCCTTGGACATTCATCATCATCTCAAATCGGATCATTTTAGCTTCATCCCATTTAGGAAAAGCTTTGATGAGGTTGGAATAGGAGAGGCTTCAACAGCAAGAGAAGTTCAAGCATCAGGGTCATGTGATGAAGTAGTACATGAACATGTAAATCATCATCATCAACAACAACAACAACATCATCATCATCATCAAATTNNNNNNNNNNNNNNACATCCAATCTCTACCTTGATATCTCCACCACCACTTCACCACACATCCATCATCTTAGACGATAATTCCTACCAAGTCTCCAGAATAATGCTCCAAAATGAACATTTCCAGGAGTCATCCATCACAAACCCACAAGAAGCTGAATGGTTGAAATACTCTTCCTATTGGCCAGACCCTGACAACCCGGATCATCATGGGTAG

>AdNAC30--Aradu.BPK98

ATGCGGTTCAAGCCAACAGATGAAGAGCTTGTGAGCTATTACCTCAATCACAAGCTCCTAAATGACAATTTTCCAATCAATATTATCCCTGACATTGATCTTTGCAAGGTTGAACCTTGGCAAATTCCAGCATTATCAAAGATAAAATCGGATGATCCAGAATGGTTCTTCTTTAGTGGACGTGATTACAAGTATGGAAAGAGCAAAAGATCAAACAGGGCAACCAAAGGAGGATATTGGAAAGCCACAGGACAAGATAGATACATAAAGGAAAGAGGAACTACCAATGTAATTGGGAGCAAGAAGACACTGGTTTTCTATAGTGGCCGTGTTCCTAATGGTGTCAAAACCAATTGGGTTATCCATGAGTATCATGCTACTACCTTTGATGATAGCCAGAGGAATTTTGTTTTGTGTCGCTTGATGAAGAAAGCCGAGAGAAAATCCGAAGATGGAACTGATGCACAAGCTTGTGATGAGGGGGAACCTAGCACTCATATGGAAGAAGCAGATGAGAGTGTCCCAAGTATGTTTGAGTCGCCAGATGTGGACATGGGTTCGATCTTCCACACACTGCCTCAAGACGGATCATCATCACAGCACTCTCCAGTCAGCATTGAACAGCAAGAATCCTTCCCATTCTCCCCATCTGAAAATTATTACCTTGTAAATGAAGATAGAAGTATGCATATACAATTCGAAACAAACGAAGAGAAGCAAGATGCTGAGAAATTTGCGGATTCGATTTTGGATGGTGGAAATATAGCTATGTTTGAAGAAAGACAGCAGCATCATACTTTCATGAATAATCACCTCCGCTCAGTCCCATCAATGAGGGTATGCTATGAAAGCAGTGACACAGATGCTGAAGTAGTCTCTAGACGGGCTGGTTCAAGAGAATACCATGTATCAAAAATGGTTCAATCATCACATGGTGCTGCGTGCACAGATAAAACTAGAAGTATCTCTTCAGAAGACTTTTGGGGATTGGATTCATCTTCATGTGACTCAAATGCAGATAAACCTTTTGAGATCAATTCTATTGAAATTTCTAGTCCTCCATCGGCTCTAAGTAGATCGAAAAATCAATATATTCCGAGGCTATCTCAAACACATAGGAAGGTTTCAAGCAATGCAATTCCCCATCTTGAGGATAAGAAGAAATTGACCACTGTGGAACAATCAAGAAGAGATCAAGAAAAAGCTCAAAAAACTAGTCCAGGAAAGAAGCTAGAAACCAGAAGCTCTGATGTTAATAGAATTGGTAGTTTCATCCACCTAGAGCCGTGTTCATCGAGCGAAAGCCTGACTCCACGAGCGGTATACCTTGTTAATGTAGTTATTGGGATTTTGTTGCTTCTAGCCATTAGTTGGGATGTGCTATCTTGTTAGAGAAGTGTTGCCATGCAAGATTTGTTTTAGGAGTTTCATAGTTATATACATAGGTTAAGTAATAATATAGTTTATATAGATCTTGCACTGATGCAAAGATTACTGCACCTTTCTTATCATAATGAAAATTATGCTTGTCATTAGTAGTAACTTCATATTGTCATTAATTCTATAATTGCAGTCTTTAAGTCACAAAAATCATTTAATGTTAGAACTAGTTGAGTTCTAGACTTCTAGTGAAAAGATTCCTTTTTTCCATTCAATAAATGTTTGATTAGCTTGATTCCCAAAATGTGCTGACTTCAAATTCTCCATTTAGTAGAAACAGACCAAAGTCAATGTTCCAAAAGAAGAAAATTTGATACAAAAGTGCATAAAGTCAAAAGCAGATTACACACCTTTGAATAGGATCTTTGTTTCATTTTACAATTCTCTCAAGAACCAAAATCATTGAATGTATCTGAGAAAGTTTATGTTCATAGCAGAGTTAATTCAACTAGAAGAGTTTGATTTAACCATAAGGTCAAGGTTGAACTGCAATTGCAGAATCAATGATAACAAAAAAGAACTACTAGATGCAGGTTTAGAAATTGTTACATAAATTTGGATATTGGATTCAACTTTTAGCAGATATTCTATGATATTTTTTTTTTTCAAATTGTAATAAAGAAATGCTTCTCTCTACTTTCATATGGCACATGGAACAAACTAGTTTTATACTTATAGTTAAAATATAAATTTAATTATTCTTTTAGTTCATGTACTTT

>AdNAC31--Aradu.BS3JU

TTTAATTAAAGCTACCTTTAGTCAGAGTAGAAAGGACAAAAACTTGAATTTAGTCTTCTACAAGCTTTCATACAAATGATATATACAAAGCAAATAAACCACAAAACTTGAGATCAAAGCAACCTTATTATGCATTATTTCTCATGTGCAACTAACCCTATCTATCTCATCTTTCTAAATAGTTAGTAGTTTCATTCCTCCTCCATTTAGAATTTTCAGGGAAGGAGGGAGTACAATATTATTCACCTCATGCAAAAGAAAGAAAGCACCAAAAAAAGTGTGAGGAGAATTTAATTCCTTCCCAACCCTTAACAAGAACTTGCATGAGGGGAGCAACCTTTAGAAGAGACAACTTCTTCTTTATTATTTCCCCTTCTTATATACTCAGCGTGAAGAAGGAAAAAGAAGATATGTATCCTGAAATGGAATGAGAGAGGAGAAGGGAAACATAGGGTAACACATGCAAGAGGAAATAATGACTCAGTGCAACAGTAATAATGATTACCCTGAAAACAATCATAGCACCATTGTGGAGAGGAACAAAGATAGCTTAATTAGTAGAACTTGTCCATCATGTGGTCATCATATCAAATGCCAACAAGACCACCAGGGTGCTGGAATTCACGATTTACCTGGGTTACCAGCTGGAGTGAAGTTTGATCCAACAGATCAGGAAATCCTAGAACATTTGGAAGCCAAGGTGAGGTCTGATATTCACAAGCTTCACCCCTTAATTGATGAGTTTATCCCTACTCTTGAAGGCGAGAATGGAATCTGCTGCACCCATCCAGAAAAGTTGCCAGGAGTAGGCAAAGATGGGTTGATCCGTCACTTCTTTCACCGGCCATCGAAAGCATACACAACAGGAACAAGGAAAAGAAGAAAGGTTCACACTGATGCTGATGGCAGTGAAACAAGGTGGCACAAAACAGGTAAAACTAGACCAGTCTACATCAGTGGCAAGTTGAAAGGTTACAAGAAAATCCTTGTTCTTTACACCAACTACAAGAAGCAAAGGAAGCCTGAGAAAACAAACTGGGTCATGCACCAGTACCACCTTGGCAACAATGAAGAGGAGAAGGAAGGTGAGTTAGTTGTGTCTAAGGTTTTCTACCAGACACAACCTAGACAATGTGCAGGTTCACTACTCATCAAAGATTCATCATCATTCCCTGCTAAACTAAAGGATCAGGGTGGTGTTCATCATCATGAAGTGACTAATAATCATAAGAACAATGGGTTTGTGGAATACTACAATGCATCCTTTATAAGTTTTGCTCAAGGGGAACAACAACATAGGTCAAACAATCCCACATTGATTTCCCATTTTCCTGCTCATGATGGGGCTCCTTTCATTCCTTGATATATTTTATGTTAAACATAACTTTTATTACTTGTACATTTTTATTATTGATTAGAAATTCTTTGTGGGACCAAGTTTAGGGCTCACATTTTTATCAATAATAAAAATGTAAGTAAGATAAAGTTATGTAAAATTACATATGAGAAAATCTATTCTTAAAGGATGAACAAAATACTAAATAGGACTTATTACAGTTATAAGCTAAGGCATTACAATTGTATTCTGCAATGAAGAAATAAGTTAATGACCAATTACAGTTAGGAGGGGAAAGAATAAGGAGAAGCTACTTCATTGTAAAGCTGGTGGTCAGATACTCCTATTTTGGGAAAAATTGGGCTAAGAAACTAAAAGAGAAGGATGTAACTG

>AdNAC32--Aradu.C1Q0A

TGGGATGTCAGAATGGGGTGATAGATAAAAGCGGAAGCACTGACTAGTTGCCATCGAAGTAGATAACATGCGAATGCCACGTCATAAGAATGACACCGAACCTGCTTGATTTTCTAGCCGACCCTGTTTCACTCAAAGTCTAAAGCCTAAATTGCCATACCAAGTTGTTGTTACTCACAAACAAGTAAAGAACAGAACAACATAACCCTCTCTGACACCAAACCCTTTTATCTTCACCTTCGAATCATGGTGGACACGGATTCAAGCGAAGCACACATGTCAATAGCAGCTTCTTCCATATTCCCTGGCTTCAGGTTCTGTCCCACTGACGAGGAGTTGATCTCTTATTACCTCAGGAAAAAGCTGGACGGTGATGAAGACAGTGTTCAAGTCATTTCGGAGCTTGAACTTTGCACTTTTGAGCCTTGGGATTTGCCAGGAAAATCTTTCATTCAATCAGACACCGAGTGGTTTTTCTTCTCGCCACGGGGGAGAAAGTATCCCAATGGTTCACAGAGTAAAAGGGCAACTGAATGTGGGTATTGGAAGGCCACAGGAAAAGAACGCATCGTAAAATCCGGTCAGGATGTTATTGGTACCAAACGCACTTTAGTATTTCATCTTGGTCGAGCTCCTAAAGGCGAAAGAACTGAATGGATTATGCATGAGTACTGTGTCAATGACAAATCTCAGGATTCATTGGTGATTTGTCGGCTGAAGAAAAATACAGAATTTCGTGCTAATGATCATTCTAATAAAACTTCACATGATAGTGATTGTGGAGTCTCAGAAGAAGTTACAGTTCAAGGGGGCACTTATGTGCCTATTCAAGATAAAGAAACTGGATGCAGCTCCAAGAGAACTAGCAGTAGTAATAGTTCTCCTTCTACTACTACTGGCCAAATTGAATCCAGTCATAGAGTTGTCAATGAAGCCAATCAAGCCAATGAAGTGGACGAAGATGATTGTTATGCAGAGATCCTAAATGATGATATCATCAAGTTAGATGAATCAACACTCTCACGGCCATCGCCACCACAAGGGACAGCAAACAGGAGAATCAGGCTAAGGGTTCCCAAATCAACAGTTCCCAAATCAAGGGTTCCCAAATCAACGGTTCCAACAGGAAATGGTTGCCATTGCTCCAAGCAATCATCAAACAAAATTAACACCTTCCTGCCATATGCTTTGGTGGTCTTCACTTTCTTCGTTTTCACTTTGTTAGCTCTAGGCTTCTTTCTTATTATTAGGAGGTCCCAAACTACTGCGCAATATTCTCGAGACCTCTCTAGAGTTAATTAAGACGATTAATTAATTAGTGTTTAGTGTTGCATAGCCTTTGGATCGATGTTAGTATGTTACGTTGTATACAAAATTACAAACAAATATGCACTCTTCATAGTGAAATAGTAAGGATAAGGA

>AdNAC33--Aradu.DII8L

TGTGGCTCTCTACTTTGATGGGCTAACATAGGGAATTGGAGGCTGCACGAGTGCCTTCAGTCTCTTCGCTGTTTGTCACCCAAGCGGACAAACATAAATACATAGTCTTACCACTGACCATTTTGGGGAGCATAGAACAGAAAAGGATGGAGGGGAGAGGGAGTAGTTTTGTGAAGAATGGGGAGCTGAGATTGCCTCCAGGATTCCGGTTTCACCCGACGGATGAAGAGCTGGTGGCTCAATACTTAAAGCGCAAGGTCTTCTCCTGCCCGTTGCCGGCCTCTTTCATTCCTGAGGTTGATATTTGCAAGTCCGATCCATGGGATTTACCAGGTGATTTGGAGCAAGAGAGGTACTTCTTCAGCACGAGGGAGGCCAAATACCCCAACGGGAACCGATCCAACAGAGCCACTAACTCGGGCTACTGGAAGGCCACGGGCTTGGACAAACACATCGCAACTTCAAAAGGCCACCAACTTATTGGCATGAAGAAGACTCTCGTCTTTTACAGAGGCAAGCCTCCTTATGGATCAAGAACAGATTGGATCATGCACGAGTATCGCCTTGTCTCCCACTCCCACCTGCTTCCCATGCAAAATTGGGTTCTCTGTCGCATATTCTTCAAGAGGAGAGCACCGGCTAATGCTAAGAATGTTCTACTGGATCACAATTCCCATTCGGCATCAGCATCAGAGGCCTTCACCATCAGCCATGAGGGCAGCAACTCTAAGGTGGTTTTCTACGATTTCTTGGCACAGAACAGGGCTGATTTGAACCGCGTGCCCCCTCCTGCTTCTTCGACCTCTGGCACCAGTGGAATCACCACCGAATCCGATGAGCATGAAGACAGCAGTAGCTGCAACAGCTTTCCTTTTTTCAGGTGAAAACAACCTTAAATATAATTTTACTTGTTACTCTTGTCTTAGTAATTGGCTAGCTAGCTAGTGCACTTCCACACATGAACTGTGTGCACGCTGCGCTCTGCCTCTATCTTTCCCTTTTCTGTTTAGGTGTAACTAATTAATTAATTAACTATCGCCTCTTCCCTTGATGGAATGGATGTAGCAAGCATCAATCCCGCAGGAAACCGACCGGCATTAAAGGTAGTCTAAATCTGAATTTGAAGAGTATCCCGTTCTACTCATACTAGTATGCAATACAATTCTAAACTCTTCCATTTTCCCGTCTTTTCTTCTAGTTGGTACTAGTAATGCTCTCACCATGCATTGTTCTTTCACCCTACGGAATAAAAAGAAAAGAAAAGAAAAGAAAATCAGTCCCTTTTGAAGAAGGTGCTTTTACAGAGAAAAAGTGGGAGGAAAGAAAAAGATGAAAAACAGGGAGAAAAAGAGGGTGGAACCGATTGTGGAATCTAACGATTGTGTTTTAACATTTAAGGAGAACGACCAAATAACGAATAAGAGCATATTATCTTTGCGAAATCAGCATCCCACTTGTTAATTGCACTTATTCCCCTTTCCCCATGGGTTAACTTTGCCGAAAGGGAAAATAAAAAATTGA

>AdNAC34--Aradu.DQR3M

CATTTATCTGTACCACAAAGTCCCATATACACTGATGAATGAATACTCATCTATCTGATCCTACCCCACCCACGCATCACGCATGGATTCATGTCAACCCCAACTCCCACCGGGATTCAGGTTCCACCCAACCGACGAAGAACTCATCGTTCACTACCTCAAGAGAAAAGCTTCCTCTGCTCCTCTCCCCGTTGCCATCATCGCCGACGTTGATCTCTACAAGTTCGACCCATGGGAGCTTCCAAGTAAGGCCACGTTTGGGGAGCAAGAATGGTATTTCTTTAGTCCGAGGGATCGCAAGTATCCGAATGGGGCTCGCCCAAACAGAGCAGCTACGTCCGGATATTGGAAGGCCACCGGCACTGATAAGCCTATTATTGCGTCTGATGGCCAACACCGACTCGGCGTCAAGAAAGCTCTCGTTTTCTATGGCGGCAAGCCTCCTAAAGGGGTCAAAACCAATTGGATCATGCACGAATATAGACTCACTACTACTCATAACAATAATTCTATCTCATCAAAGTCTTTTCCTTCTCTTCCTTCTCATCTTCCTTCCTCCAATCAGAAGAATAATTCCTTGAGGGTATCTATCCTTGATGATTGGGTGTTGTGCCGAATATATGAAAAAAGCAACCGTGGCAATTTTGCAAGAACAGCGTTGATGGAGCACCATGATCATGATGATGATGATGACAATAATAAGGATCAGCTTTCCGCGGAAACAACGAGCATGATAGAAAACATGTCCACCATGAGTCAGAATTCCAAGCCCACACAACATTATGGACCATTGCTGGTTCAAAACGATGACAACTTCTTCAATGGAATCTTAGTCGCTGATCATCAAAATCATCAACATCACAACTTGCCAATGAAGAGGGCACTGGTGAATATGAATAATTCGCAGTTTTGGAATGAGACAAGCAAGAGGTTCCATTGTGATCTCAATAACAACACTAACACTACTGTTGCTAATAATGATGAGGATAACAGTTCCTTTGTTTCACTGCTTAGCCATAATCAGATTCCTCATCCTACTAACAATGCTTCTCTTCTTGGCCCTACTGTTGCTGATGGTGTTTTCAGGCAACACTTTCAACTTCAAACAATTAATTGGAACTTATAA

>AdNAC35--Aradu.EP425

GGGAGTTCCTACGAAGTTGGTTTCTTTCTTCACTGCTGGTGAAGTGGTTCTTTGTTGTTGGATTGTTTGATTCAAGGGAAGTTGTTAGATTCTGAAGGTTTTACTATTTAAGCTTTCTTTATTCTTCTGCGATGGCCGCAATGAAGTCAATTCCAGGGTACCGGTTTCATCCAACCGATGTTGAGCTGGTTCAGTACTTTCTGAAAAGGAAGGTGATGGGGAAGAGATTCCCTTGTGATGTGATTGCTGAACTTGATATATACAAATATCCGCCGTGGGATCTACCAGATCATTCTTTGCTTAAAACTGGAGATTTGGAATGGTACTTCTTTTGCCCTCGAGGGAAGAAGTATTCGAGCGGAGGGAGGATGAATAGGGCCACAGAATGTGGGTACTGGAAGACTACTGGCAAAGATAGATCCGTTGAGAACAAGAAGCTTGTTGTGGGCATGATAAAGACTCTGGTGTTTCACATGGGTAAAGCACCCAAGGGAGATCGAACTGATTGGGTTTTGCATGAATACCGACTTCAAGATAAGGACCTTGCTGATAAGGGTGTTCAACAGGATTCTTATGTGATATGTAAAGTGTTTCAAAAGGATGGTCCTGGTCCTAGGAATGGTGCACAATATGGAAGGCCGTTTAATGAGGAAGACTGGGATAAAGAGGATGAAATTGACTGTGTAGAATCTGCACCTGTTGCTGCTCTACCTGCTGCAGTTCCTATACAACCCGCTTCATGTCATAGCTCTGTTGTGAATAACGTGAATCTCTCTGTGAGTGAATGCTATGGGTTGACCTCTGTTTCGTGTTTAACAGGGCCAATGCCTTCTTGCTCAGCACATCCTTCAGCTCCAAGTAATCAAGTTGATGGTGACATTACACCAGTGCCTGGTTCCTCCATAGAAGATAACATAATGGCTCCTACTCAGAACACCACAACTGAAAAGGTTGACAATCCTCCTGACATAAACAATGCTGAAGGAACACCTTGCTTTGATCCCAATGAGATTTTTGGGGGTCTGGGTGACCTTGATGGTTTGTTCGAAATGGGTGGAATTGGACATGGTTTTTCCTGCGGCCAAAATGGTGGATATACTGTGAATGAAATGCTTTCTGCGGGTGATGGGTTGCGTTTCCCTGATCCCCTGGACTACTTGGAGTTGGGTGACCTCGACACTCCATTGTTATGGGAGACTAATGAACAAGGAAATTGGAGCCAGGACAATAAGTGAGGCTTCAAAATTTGACGAAGCATCAACATGAAGTTGGTGGAAGAACCATTGCCGTGTGTTGCTGATCTTGATTGTCTTTCCATTCTCATTTATAAACTCTACTGAAGCATATATTCTGTAGAAACATTCCTTTCTGTAATAAGCTAACACATGATAGCAGTTGATAAATCTTTAGTGTTAGTTCTATTTACTAGGTTGGATTAATCATTAGCCTCACAAATTTTCTTCTGATATTTTCTAAGTTGTGTTTGATAGAGTGAGTTTTCAACATCTATTACGTTGAAATCTATTCATGTCTTTCGTAGTAATGTCAAATGATGGAAGTTGACGCATTTAACCCTAAACTTCTAGTATTCTCAATTTTGTTTTATTGAAGGGTTAGTTGATGAGGGAACTGTGATAGGCCTTTGCCCCTTTACTTTTCGTCGCGTTGGAAACTGGAAAGTACGTTATCTAAACCAGTATAGC

>AdNAC36--Aradu.ETZ8K

ATGGGAGGGGCATCACTGCCTCCGGGTTTTCGTTTCCACCCTACTGATGAAGAACTGATAGGATACTACCTGAAAAGAAAAGTTGAGGAGCTTGAAATTGAACTTGAAGTTATCCCTGTGATTGATTTGTACAAGTTTGATCCTTGGGAGTTGCCGGAGAAGTCATTCTTACCAAAAAGAGACTTGGAATGGTTCTTCTTTTGTCCAAGGGATCGAAAGTATCCGAATGGATCAAGAACAAACAGAGCTACCAAAGCAGGATACTGGAAAGCCACTGGAAAAGACAAAAAGGTTGTGTGCCAATCTAGTCCATCAACATCAATCATGAAAGCCACCGGATATCGCAAGACCCTTGTTTTCTATCGCGGAAGAGCCCCTTTAGGCGACCGAACGGATTGGGTTATGCACGAGTATCGCCTCTGTGATGATCTTGGCCAAGACTCACCAAGTTTTCAGGGTGCTTATGCTTTGTGCCGGGTTATTAAGAAGAATGACAAGGCCAGTGATTACAAGGGTAAAAGAGGTGTCAGCAGTTCCAAGAATGAGAATGAGAATGAGAATGAGAGCTCAATGAGATTGTCATCCTCTAAGGAGCACTTGAGCATCTCTGCTGATGTTTCTTCTCAAGCAAGTCAGCTATGCAGCGAGAGTCGTTATTCGAGCCCTATAGCTTCTCCTTGTGCATACAATGTGGCTGCAACGGCCGGGTTTGAGCCACCTTCTGTGGACACTAATCCTTCAACCTTCTTGGTCTCCCCTGATATGATTCTTGATTCTTCAAAGGACTTTGCTCAAACACAAGATGTTATTTCAGGATTCTTTCCGCATCATGAATTGCCAAGTACAATGACACCATGGCAATCATTGGAACATACAGAGATTTCATCCAGTTCATCCTACTCAAATTTCAATGGGGAGATAGAATTCTCTGATGAACTCGGCCTAATTGGCCGAATGTCGCGTTACTCAGGACAAGTAGACATGTTAGACTTCTATGGAAATGAGGAAGTGCTGTATGAATATGAAGGATATGACCAGATCAATTCAATCAGAGATCCAAGACAATTCTGAAGTGAAAAGGAAAATTTCTCCTTTTCTTGTAATGCTAAGCATATATGGGAATGGAAGTAAGGGAGATCTTGATGATGGAGGGATCTCTGAGGCAACGCC

>AdNAC37--Aradu.F2DT2

CACTAACATATTATGGCAGAAAACCACTGTGTATTGTGCTGATGAGGTGTTAAGCAGATAATATAATTTTAATTTGGTAGAGTGGTGCCGCTAGGTGAAGGTCAATTTCAACGAGTAAAACCAATTTGGTTGGTTTTCTCTGTCTTTTCTTACAAACTTTACATGCAAGTGATTGGTTCCTCTCCCTTTCAATCTTCACATGTGCATGGGACTAGAGCCATATATATAATGTGGATGACACACTTTGGTTTCTTATTAAAAGCAAGCCAAAAATTCATATTGGTATCATCGGCACTTGTTGATTCCTTTGTCAAGAACAAAGAAAATTCTTTTCATGGAAGAAGGAGGAGGAGATCAACATGCCTCTAACAGCAGCTACACTTTTCCACCAGGTTTCAGATTCCACCCTTCTGATGAAGAACTCATAGTTCATTACCTACAAAACAGAATCAGCTCTCGTCCACTTCCAGCTTCCATTATAGCTGAGATTGATCTTTATAAGTATAACCCTTGGGATTTGCCAAAGAAGGCTTTGTTTGGAGAGGAAGAATGGTACTTCTTTAGCCCGAGAGATCGCAAGTATCCAAATGGATTGAGGCCAAACAGGGCAGCAGGTTCAGGGTACTGGAAGGCTACTGGAACTGACAAGCCGATTCTCACATCTTATGGATCGAAGCGCATCGGAGTGAAGAAAGCTCTTGTCTTCTATTTAGGTAGACCTCCAAAGGGGACTAAAACTGATTGGATCATGAATGAGTATAGATTGGTTGACACAATCACCAGCCCCTCCAGGCTCAAAGGTTCCATGCGCTTAGATGACTGGGTACTCTGTCGCGTTCGACACAAAGGCTACTCATCGAAGAACTCATGTGAGAATCAAGATAATCCTTGTGAACCAAACATGCTATCAAATCTGCCAAGGTGTGATGAAGGTTATCCAGCAACAAACATGAACTTCCATGCTGATATGATCACTGATTATCAATACAAAGACTATCAGATCCTAGCTTCTATCCTTGTTGGTGGCCATGTTCCTACCACTGAGAGCATGTCAAGTTTGAACTTGAAGGATGGCAAAGGCAATGATCCAATAACTTCAGTTCATGAAGATGGTTTCCACAGAGAAGATTCTTCTACAACAGTTTCTCCTTTGGACTGTTACTTCAACTCACTGAAAAGAAAATCTAATGAGGATAGCCAATATGAGAATCTCATTTCCTTTAACAGGAAGTTGAACATGGAGACCACAATGGATGATGAATCTTCTATCATCAATGGAGGTTTGAACTTCTACAATCAAAACCAGTCTCAAGATGACATAATATTCAATAAGAGAGCAGCAGAGCCTAGCATCAACTTTCAAGAGCTAAAGCAATCAGCTTTTATAGGAAGATACCCGCAATGCTCAAGTGATTGACCATAAGACATATTCAATACTCTCTATTCCACTTGTATATTTTCATGGATATACAACTAAGAATATAGGCCTCAAATATTAAAGTGAATTATTGTTCTTTAAGCGTGGTTGTAATAAATATATATAGAACTTTTCTATATACTTATGAAATTCCTTGAAAACATTGTGAATAATGCTAGCTTGTGCAGAATCTGCACAATGTCATTTCAACTAGAATTTTATCCTACTCAGTAATAATAAAACTTGTACAAACAATTTATATTCTTCCCACAAAATAGTTGC

>AdNAC38--Aradu.F48KW

TACCACGCAATCACATCATGTCACGGAGCTCACACTCTCTCCCTACTCCCTCATCGCACTGAATCACTCTCCCATATGAAGGTTCCCAGTCATTACTGCTAGGAGTCTAAGTACTACACCAAGCTTCCTTCTTTGACCATAACACTATCCAATTCCAACCCCCCAGACCCCCTTTCTTCTTTTTCACACCGCAATTTTTTCCACAATCGATCCCATTTTAAGCTTCTCTCTCCCTCTAGGTTTTTTTTTTTCATTCGACGGTTCATTCCTCGAATTCTCGTACCCCGAGGATTCGATTCGGGTCATGTAGTCGTAGGTTTTCGGTTTCTACAACCGCTTGATCCACAAAGCTCGGATTCTTTTTCTTCTTCACTTTTTTGGGGGGTTGTGAATTCTGATCCTCTATTTTTGGGGGTTTCGGTTCCGTGATTCAATTTCTTCTTCCGGATTCCTCTAATCGCATCGATTAACTGGGTGTTTGGTTTCGTAGCCTGTCGATTCAGAAAGTTTCGGTTTGTTCCTTTGGGTGTTTGGTCTAGGGTTTTTCGGATTGAGTATGGGTGCTGAAGCTGGTGCAACTGAGTGTTTCAGTAAGGCCATGGCGTCGATGCCTGGGTTTCGGTTCCATCCCACGGATGAGGAGCTGGTTATGTACTATCTGAAGAGGAAGATATGTGGGAAGAAGCTGAAACTCGACGTGATTCTAGAAACCGATGTTTACAAGTGGGATCCTGAGGAATTGCCAGAGATATCTGTACTGAGGACTGGAGATAGGCAATGGTTCTTTTTCACTCATAGAGATAGGAAGTATCCTAATGGTGCGAGGTCCAACCGAGCAACAAGGCAAGGTTACTGGAAAGCAACAGGAAAGGATCGTAATGTGACCTGCAATTCTCGGTCAGTTGGAGTGAAAAAGACTCTGGTTTTCTATAGAGGCAGAGCTCCTAATGGTGAGCGGACCGATTGGGTTATGCATGAATACACCATGGATGAAGAAGAGCTGAATAGGTGCCAAGATATTAAGGACTATTTTGCACTTTACAAGCTATACAAGAAAAGTGGACCTGGTCCTAAAAATGGAGAACAGTATGGTGCACCATTTAAAGAAGAGGAGTGGGCTGATGACGAATGTGTAGATTTCAATATTAACTCAGCAGATCGGGAGGAAGTAAATACTGTCCCTGTTAATGATCAGCTGCCTCCTTTGGCCGATGATGAAGTCACGGATATGATTAATCAAATTTTGGATAATGAGCTTGCCCTTGACCAGCAATTTGGTGACGGCCTTGAATTTCCTCAGGTTGTTGCTGAAGAAACACAAAGTACTGTGGTGGATCAGTTCTCTGAGGCAGTGACGGACCCCGAGTACAACGATATTTACCACTCAACCAGTCAGCACTATGATGTGCAGAATGTCAACTTCAATCAGTCGGTTGCATCTCACCTTCATGCCCCGGAAGGATCAGAAGTTATTTCTACTGCCAACATTCAAGTAGAAGACTATAACTTTCAGGAGGATGACTTCTTGGAAATCAACGATCTCAATGGTAGTGAACTTACAATTCCAAATATGGAAACACCAGTGGAGAACCTGCAGTTTGAAGATGGATTGTGTGAACTTGATCTGTTCCAAGATGCAGAGATGTTTCTTCGTGACTTGGGACCAATCAATGAGGAAACCATTCCACATTCATATATGAATAATGCCGCTGGAAGCAACATTGAAAATCAGAATTATCACTTGCTGCCCAATCCAGAGGACGCTACTCAAAATGTTCATGAATTTTGGATGCATGATGAAAGAAACACCTCGAGTCTGTTTGAAGGCTTTGATGATTCTCTCTCTCAACAAAATCCAGGTGCTGTATCTACTGAAGGCTATGATAATCAAAGTAGCATTGCAGAAGATGTTGCTACAAGTAGATTCTCTTCGGCTCTCTGGTCCTTTGTTGAGTCAATACCTACCACTCCTGCATCAGCTGCGGAAAATGCTCTAGTGAATCGGGCTTTGAATCGAATGTCTAGCTTCAGCCGAGTGAAGATTAATATCAAGCCGACGAACACAGCTGCAGGTAAAGACACTGCAACTACGAAGAGAGTGGGCAGAAAAGGATTTTCATTCCTTTTCTTCCCAATTATTATTGCTTTATGTGCTTTCTTATGGGTTTCTCTTGGAACTTTTAGATTATTAGGGAGATGCATCGCTCCTTGAATATGTAAATCAAAAGTTTTGATGCTATTATGCTATATATGTTCTATCTGGGATCTCCCCAAGATGACTGAGATAGTATTGTTCTAACATATTCAAAAAATAAAAAATAATATTTTATTCTTTGGGACTGTTAGAAGTTTTAAGTTTAGGCCATATTAGGACTATGATTGGGAGCAATGGTGGAGAGGGGAGGGTGAGGTGTGGCTGGGACCGGTGTTCGAAAACCGTATTACGGAGAGTTTGATGGGGAATGCAAAGCATGGCATAGGAAGGGCATGTGGGAACCCTTGATCAATGACCCCTCTGCAAATTGGAGGGGTGGATACAGAACACTTAGCTGTCTCTCCCAGGCCTTCATCATCTTCCAAACAATAAATCACCAGCCATTCCTTTCCACTCTTCCACTTCATCAAGGACCCTTCCCCTCAACCCCCTCCAAACTTCCAAACATAGCCTAGGTGAGTCTTAACAATCTTAATCCAAGAGAAAGGGGTTTTATATGAATTTGCAGGGTGGATTGGTAATGCACTAAG

>AdNAC39--Aradu.F6Z4G

TCTTTCTCTCTCTTCCCCCAGATAGCAGTAGTATTTAGGTGCCTCCTTTTTCTCTCCTTCGTTTTCCCTTCTCATTATTCACATCCATCTCCATATTCCATCTCTCTTTCCTCTCCCCCCCTCTCTCTATATATTGAAAGTACAATAATATAAGTAAGAAAGAAAGATCAGAGCGAGTGAGTGAATGAAATGGAAAAAGTGGCATCATTGGTGTTGAAGGAAGAGGAGCAGATGGATTTGCCACCGGGGTTTCGGTTTCACCCAACTGATGAAGAGCTCATAACCCATTACCTATACAAGAAAGTTATTGACACCAACTTCGCCGCGAGAGCCATTGGCGAAGTCGACCTTAACCGCTGCGAGCCTTGGGATTTGCCATGGAAAGCGAAAATGGGAGAGAAAGAATGGTATTTCTTCTGTGTGAGGGATAGGAAGTATCCGACCGGGTTGAGGACAAACCGGGCGACCGAGTCTGGGTACTGGAAGGCAACCGGGAAAGACAAGGAGATTTTTCGAGGCAAATCGCTCGTCGGAATGAAGAAAACGCTTGTCTTCTACAAAGGAAGAGCACCAAAAGGAGAGAAAACAAACTGGGTCATGCACGAGTTCAGGCTGGAGGGGAAATTCTCCATCCATAACCTCCCAAAAACCGCAAAGAACGAGTGGGTGATTTGCAGGGTGTTTCGGAAGAGTTCAGCTGGCAAGAAGGTTCACATCTCTGGAATCATGAGGCTCGACACTTTCCGAACCGAATTGGATTCTTCCGGTCTACCTCCCTTGACGGAGACCTCGCCCTCTTTCGACACCATCCATGACGAATCGCCTTACGTGCCCTGCTTCTCCAATCCAATTGATGTTCCAAGAAACCAAGCCGCAGGCGGAAGCGGAGGAGGAGGAGGAGTCTTTGGTGGTTCCTTCCCCAACAACTCCTCCTCTTCTGTTCCCGCCTATGCGGTTTCTTCCAACATTCTCCCAAGGATGCCGATTTGCGGCGGTTCCTTGTACTCTACTCAACATCAAGATCAGAGTATCCTGAGGGCGTTGTATGAATCGAACGAAAGGGAAATGATCAGTGTGTCACAGGAAACAGGCCTCACTACTGAGATGAACGTGGAAACCAATTCCGTGGTGTCCAATTTTGATTTAGGGAGGGCTCACTTTGAGAGTTTGTGGAATTACTGA

>AdNAC40--Aradu.F8VRL

ATGAATAAGATGGATCTGATAGATGCGAAGCTGCAAGAAGAGCATCAATTGTGTGCATCATCGTGGAAACAGTGCCCCGCTTGTGGACATAAGTTTGAAGGCAGCAGCGGGAAGAAGGGGGAGTGGGAGTGGGTAGGTCTGCCAGCAGGAGTGAAGTTCGATCCAACAGACCAAGAACTGATAGAGCATCTAGAAGCAAAAGTAGAGGCAAAGAGATCGCACCCTTTGATCGATGAGTTCATTCCCACCATCGAAGGAGAAGATGGAATCTGTTACACCCATCCCGAGAAACTTCCAGGTGTGACGAGGGATGGGTTGAGCAGACACTTCTTTCACAGGCCATCAAGGGCGTACACCACTGGAACACGGAAGAGAAGAAAGATTCTTCAAAACGATGAGGCAGAGGCCGAGAGAGGAGAGACACGGTGGCACAAGACCGGTAAGACAAGGGCCGTTATGCTCAAGGGAAAGCAGAAGGGGTGCAAGAAGATTCTGGTGCTGTACACCAACTTCGGCAAGAACAGGAAGCCCCAGAAGACCAACTGGGTCATGCATCAGTACCACCTCGGACTCCATGAAGAGGAGAAAGACGGGGAGCTCGTCGTCTCTAAGATTTTCTACCAAACTCAGCCCAGGCAATGCAGTTGGTCTTCTTCTTCTTCTTCTTCTTCAATTACTGCTGCTGCTCCCCCTGTCAAAACTAATAATGACACTTGTCCCGTTCTTGGATTCCCTCCTATGGAACATTTCAGCAGCTTCATCCCTCTCAGAAAAACCCTCCATAATGAGAACTTGTTTTATTTAATAGGGGAAACTTGCACACCAGCGTCACATATTCCTTCATCAAATCCTCTTGGAGTCTTCCATCACAACACTTCCATCATCCTTGACGACCTTATCTCCGCTAGATTCATGACTCCTCCTCCTCCTCCTCATTTCCACCAGCAGCATGATAATAAAGTAGTAGGAGGAACCTCTGCTTCTGGTTTAGAGGAACTCATCATGGGCTGCACTTCAACTTCAACCACTCATAATATCACCAAAGAGGCATCAATGACAAACACAAACCCACAAGAAGCTGAGTGGTTGAAGTACTCTTCTTATTGGGCTGACCCTCAGCCTCAGCCTCAGCCTCATCTTCATGGGTAATAATAACAGACCCCATTTGGCAAAGGCGTGGCTTTTCGAGTCTCAACGGAAATAACTGCTGCATGTGTGCACTGTAACGGCACATACATCAAGTTGGAAACAAACAACAAAGGAGGGAAGAACAAATTGAATCAAATTCAAGAAAATGAAAATAACCGCAAGAAAATTAAAGCTGCTGTTCTACCTCCTCATCACACAAAGAAACAGTTTGATAATGCATCAAA

>AdNAC41--Aradu.H2YS3

ATGATATCAGAGCTTCTACGATCGAATGGAGTAATGAAATACGTGGTATATGCAGAGGTGGCAAAGCTAAATGCAAATGAATGGTACTTCTTCAGCTTCAGAGACAGGAAATATGCGACGGGGTTCAGAACGAACAGAGCGACGACAAGTGGTTATTGGAAAGCAACGGGGAAGGATCGAACGGTGCAGGATCCACTTACGCAAGAGGTTGTAGGGATGCGCAAGACTCTGGTGTTCTACCGGAACAGAGCTCCCAATGGCATCAAAACTGGCTGGATCATGCATGAGTTTCGGTTGGAGACCCCACACATGCCCCCTAAGGAAGATTGGGTGTTGTGTAGAGTGTTCCACAAAAGCAAAGAAGAAAACAGTGGCAAACTTATCATGTATGATTCCATTTCCACACATCATGAATCATCAAACTCTATGGCATTGGTATCAACCCATCATTTGAACCCCATCAACAACCATAATGCCATGAATAATTTCCTTCATCACTTCTCATCATCAAGGGATGATAGTCAAACAAATAACGCCAATAATAATAATAATAATAGTCCCAAGGGTTATGATGGATATGGCTTCATATGGGATCACATGGATCTTGAAGATCATAGTGTGCCCTCATCAGACTTCCAGGTCGACAATAATAATAATAATAATAATATTGCATTGCTATAAGAAGCTAAGATACTAATTAGGTGTATTATTGTATTATTTGTAAATATGCATATTGTATATGGAAAGTGTGAAGAATTCGTTGTTGTATGTATTGATTATGGTGTGTGTGTGTGTGGCTTCGAGTGGCCAAGTCACGTGATATGTACTATATTATGCATATATATGCTCGTTGTGTAATTAGTAGTGTTAGTGTACGTATTATTGGATTAATTAGGCTCTTTATAATTTTCTTATTTTGGTGTATCCTTCTTCTCCTTAAAATTAGCCCTTTCTTTATTTTTCTCATAATGTATGTATCCCAGT

>AdNAC42--Aradu.H5KV7

ATAACAATTTAATACATCAATTATTTTATCATCAAGACCAATAATAATAGAAATAATAACATAGAAATGAATACAAAGATTGAACTGCCACCAGGTTTCAGGTTTCATCCAACAGATGAAGAGCTCATAACTCACTACCTCTCTCAGAAGGTTGTTGCTAGCTGCTTCTATGCAACTGCCATTATTGGAGAGGCTGATTTCAACAAGTGTGAGCCTTGGGATTTACCTTGGAGGGGCAAAATGGGAGAAAAAGAATGGTATTTTTTCTGTTTGAAGGACAAAAAATACCCAACAGGTGAAAGGACAAATAGAGCCACTGGTGCTGGGTACTGGAAGGCCACAGGAAAAGACAGAGAGATATACAATGCAAAAGCAAAAGCACTTATTGGGATGAAGAAAACACTTGTTTTCTACAAAGGAAGAGCTCCAAATGGTGAAAAGACAAATTGGGTCATGCATGAATATAGGTTGGAAGGCGATAATAAACCTTCTATATACAATCTTCCCAAAACAACCAAGAAAGAGTGGGCTTTGTGCAGAGTTCTACACAAAAGTGAAAAGAAAGTAATGCATGTTCCACAACCACAGGGATTGGTTGAGTTCAGCTCTTATGAAAATAAGGAACTTCCCCAATTGATGGATTCTTCACAAGTAACATTCTTTTCATCAGACCCAAATAATCAAAGTGAGGATCCAAATCCAATCACACGTGATGATGATAATAATAATAATGATGACATCATAGTTGATAGCATTGAAACTCCTTTCTTGGAACAACAACCACCTTATTATTCTTCATCCTATGATTCTTCAGATTTAGACACCCTTAACCCTGCCACATGGGATATTTCCGAAAATGCCCCTACAAGTAATGCGTCTAAGGAGACGGACTTTGATGCTGACATGTTCTCTTTGATGTACAACAATAGAGAAGTGTTCCAAACATCATTTGAGAATCAGGAATATTATGCATATGATTCTATGGGACATGTGGACAATGGTTCCCTATGGAATTTTTAG

>AdNAC43--Aradu.JV7AK

CACAATTTTGGGCTTCTTTTTATTTGTATCATTTGAAAATTTTGGATGTTTCTCAGACAGAAAGAGACAGACCTGCTTTTGAATTTGTGACATTGGTTTGAATCATCAAAAACCTCTCTTCGTTCGATGGCAGGAGCATCATGGTTGGTGGACAAAAGCAGAATTGCAACCAAAATCAAGAGTGCATCAGGAGCTTGTGATCCTAACGAAGTTATTTGGAAAACCAATCCTACCAGGATTTGCCCGAATTGTCATCATCCTATTGACAATAGTGATAAAATTGCATATTACCAGTTTAAGCGTTGGTTCAAAGGACCGGTATTGCTTGATATTCATGTAACTCAAGAGTGGCCTGGCTTACCAAAAGGGGTTAAATTTGATCCGTCAGATCAAGAGATAATCTGGCACTTGCTTGTAAAGGCTGGTGTAGGAAATTTAAAACCTCATCCTTTCATTGATGAGTTTATTACTACCCTTGAAGTGGATGATGGGATTTGTTATACTCATCCTCAACATTTACCTGGTGTCAAGCAAGACGGAAGGGCCTCACATTTCTTCCACAGAGCAATAAAGGCTTATAATACCGGCACGCGAAAGCGTCGAAAAGTACATGGTCAGGATGACGTTCGTTGGCACAAGACTGGAAGGACTAAACTGATCACCCTGAACGGGGTTCCAAAGGGTTGCAAAAAAATCATGGTTTTGTATACAAATGCGGTGAGAGGAGGAAAGTCGGTGAAAACTAATTGGGTTATGCACCAATATCACCTTGGGACAGAAGAAGATGAAAAGGAAGGAGCATATGTTATTTCTAAAGTGTTTTATAAAGATGACCAGGATATACCTGAAGCCGCAGAAAGTAAAAATGCAACAGTTGCGAAAGTAGATCCAGTCACTCCCAAATCCACGACTCCTGAACCTCCTCGTAATGAAAGGCAAGATTCAGATCTAGGCCTGGATCTAGACCTAGGGCAAGAAGCACTTGCTTTTACTGAGATGGATTGCTTAGATGAAATTCAAGCTGACTGTGAAGAATCTGCGAAAGCTAATCCACCAGTACTGGAGACACAAGAAAATGAAGGGATGGACAACAAGGAAACTAATGCTTATGAAGCACAACCGTGGTGGGATAGTGATTCACAGAATCTATTAGATTCACAACAACTCGTTGAAGCATTAACTCTCTGCGAAGATATATTTCACAGCCAATCTTCCAACAAAGACGATGAAAATGATAAGAACCAAACCGGTCTATCGGTGTATGCTCATCTAGGACCAGAGCATCTGAAGAAGGATATTGAGGAGTGTCAAAAGCTTGCTCCTGCAGGACCAGAGCATCAGAAGGATATTGAAGACGGCCAAAATCTTGACATCGACCTTGCAAATATAGAGCGGGATACTCCTCCTGAGCATCGACTAAGTCAGCTGGAATTTGGTTCGCAGGACAGCTATACTTACTGGGGTTTTCAAGGCGTAAACTAATATCTTCCTTGATCTCTCGGCTTTGAACCAATTTTTTTGCAACTACATTTTGTTGATGTGTTATTTGTCTTTTGCCTTTGTGTTATGCTTAACTATGGGGCAAACTTAACTGTTACGAGTAAGTGGTTACTACTAACTATGCAGTAATGTAAGACTGTGAACCTGATATATATTTTGATCAACCTTGGTGTAAATGTAATTCAAACTTGCAATTCTCGGCTGGTTGAAGTTTTGTTCTATATTCTTGCAGTTCTGGATGTGGACTCTAGAGAGAAAGATGCATTTAGATTTCTACTCTATTCAAGCGCAGCTATGGAATTATCAAAGTTGCTTTTAGATTTCTGATAATGGCTTAAGACTGAAAAATAGTAATGTTGGCTTTGTTCTACAGTACCAAGTAGTATCAGGATGTGAATTTTATCCAAAAATAATTTCTTCAAGACCAAAAATGATATATAACCAGATTTATGTGAAAATTT

>AdNAC44--Aradu.JZK1S

ATGACTTCAACAAAATTGCCTTCAGGTGCAAGCAAGAAGTTCAAACCTACAGATGAAGAACTCATTCAAGATTTTTTCCGTAATAAAATCAATGGGCGGTCTCTACCAAATTATGGAACCATTCTTGAAGAATCCATGGAAAATTTGGGAAAAAACGTTAAAAATTCTTATGACGGGAAGGACCTCTATTTCTTCACTACTCTGAAGAGGAAGTTCTCGACTAACAACTTGAGGATGGTTCGCACCATCGAGTTTGGTTCTTGGGAAGGTGAAGACATCGGAAAATAG

>AdNAC45--Aradu.K2UJH

ATGGGTGCGGTGGAGGTGTTTCAACAGCAGCCGCTGGTGGTGGACGCTGCTCCGGTTTTGTCGCTGAACTCGCTGCCGTTGGGGTTCCGTTTCCGACCGACAGACGAGGAGCTCGTTGACTTTTACTTGCGGCAGAAGATCAACGGCAATGGCGATGAGGTTTGGGTCATTCGAGAAATCGATGTTTGCAAATGGGAACCTTGGGATTTGCCTGGTACAATTCTCAATTCTCTGTTTTTTCAGATTTGTCAGTGGTACGGAACAAGGATCCGGAGTGGTTCTTCTTCTGTCCACAGGACAGAGTCCGGCTCGACCGTGATTGGAATGAAGAAGACTCTTGTATTCTACACTGGCCGTGCTCCCAAAGGGAAGAGGACCAATTGGGTGATGCATGAGTACAGGCCTACCCTGCAGGAGCTTGATGGTACCAATCCTGGACAGAATCCATATGTCCTTTGTCGATTATTTAAGAAACAAGATGAGAGTCTTGAAGGTTCAAACGGTGAAGAAATGGAGCGAACTACTTCAACTAATTTAACTGCCAATTACTCTCCAGAAGAAATACAATCAGATCCAGCTGTTAAATCGGTTTCTTCTTCACAGGCTACAGAAGATGACAAGAAACTAGCAGTTATCCCTTTGACCCCTGAAGAAGCAATTTCCAATGTTATAACCCCGGTCGGTTGCCAAAGCGATGGATGTGATGCTTATGATGCACAAAATCAAATCGCAGCAGGAGATCCATCTAAGGAGGAGGACTTACAGGTGAACATGGACATATTTTATGACCCGAGTGAGCTATTTGACGATAAATTATTCTCCCCACTTCACAAGCATATTCCAGAAGAACTTTTTCATCAATCAAACAATGAAGCCAATGGACATTTTGGGTTGCAACATCAGTGTGGAACAAATGAGATCAGTATTTCTGACTTCTTTGACTCTGTTATTAATTGGGATGAGATCTCCGGTGACAATTCCAGTGGCCAAACGCCAAACTCTGCTTGGTTTGATGTTCAGCACAATGAATCATGGGGAAACTCAAATGTGGATATGGTCCATGCCAGGCCCCTACAAGTAGGGGGTGCAGATTATCCAGGGGATGCAACCGAGGGAAAGCTCCCTTTGTTGAAAACTAGAGAATTCAATCCCAACACCTCTTATGACAATGCGATCAGCAACAACATGGGATTATTTCAGGACCATTCCCAGATGGCTTTTTCATCTGATGTTAATATGCTCCAAGGTTACCTTGCAACCAACAATTATGAGCAACCGACAAACTTCAATATGGCTATGGCTAATAGTGACAACACTGGAATTAGGATAAGGTCTCGGCCACCAGGTTATGAAGGGCCAAATGCAAACTCCAATATGCAACCACAAGGTACTGCACCTAGGAGAATACGGTTGGCACGAGCTCTTGCACCTCAACACACGTCCAATGAGGCGGCAAAAGATTCGAGTTACGAGTCAAAAGATCGAAATTCACAAGTAACCACTGCCAGAGAGATGGAAACTTCCAAAGACCTTGCAGCTGGTGAGAGTGTTACTGTTACTAGTGATGTGGAGGAACAGGAGTCATCACCAGTTGAAAATAAGGAATTTGAAGACTTCAACACAGTCCAGCAGAGCACATCATCAGCTTCCTCGAATCTTTCCACATGCTCTTCTGATTCTGAAGTTTCTTATGAGGCAGAAAAAGAATCCGGTTGGACATCAGAAGACCATAGTCCAAAACCAGCTGCCGCGGGGGCCAGTAAAGCTTCCGAAGACCAAGTTCCCAGCGAGTGCATCAATGATATCACTGATGATGTGGATGAATCCAGGATACCAAACGCTTATACTCTAGAGGTCTCAAAGGAGGAATCCTTCTCGGACTCAGAGTCGAAAGACTCTCTATTGCGTAGAAAGGTGTGTTACCCATCGAAGTCTTCCTCAAATCTAGCCAAGTGGTATTCGGTTATTGCAATCTCAGCCACTTTGGTGGTGTTACTAGCATTCCTTGTTAATACATGGGGATATGGATATTACCTTAAAGTTTAACTGCATAGTAGGATATGTATCATTGCATAGAGCATATTTTGCCTCCATTGCGTTTTTGGCCTTAGTTAAGGAGGCAAGTATGTAAGGGTTGACTTTGTAGCTTTATTTGAATATAATCTGTATGTGCATATGTTATATATAGCAGAATATGCCTAATCTGCTACTAGCTTGTACTATATGTGGAGTTGTAAACTCATGTTTATAAGTTAGATTCAGAATTTCAGGGTTTCCAACTTTTTGGAATTTAATTTTGATTGCCCGTGGTGACCACCATTGGAGAATATGCCTGCCTAGCCAAGACCCAAGAGTGTATTTTTAGTAAAGAAAAGTTTAGGCAGATTCGGCTGTTTGATTCATTCATTTATTTATTCTATTTTTTGTTCTGTTGGTTTAAAGGTAAGCTTTTAGTTAAGAAGCTAAAAGATATAGAACATTGAAAATCTGGGGGAAAAAAGATAATGTATATATAAGAAATCA

>AdNAC46--Aradu.KF8UQ

ATGAAGGTAGTTATAGCTAGAATTTCTCTACCATTCCATCCCAGCATCATACCGGACCTTGATCCCTCTCAACTTCATCCATGTAAGGCGTTTTCAAGTGGGAATCAACACTATTTCTTCACCAATAAAGTGAAGGAAAACAGAAGCACAGAAAATGGGTATTGGAAGGAAATAGGTTTGAGCGAACCTATAATCTCAGCTGATGCAAACAAGAAATTGGGAATCAAGAAGTATTTTGTCTTCACTCTTAATGAAGGCACAGAAACCAATTGGGTCATGCAGGAATACCATATTTCCTCATCTATGTTCCACAACCCTATTTCATGTTATGCAAATGGAACTGCTCACCGAAGATTATTAAGACCTGATCAAAATCAGAACAATAAATGGGTTTTGTGCAGAGTGTATGAGAAGAACAAGTCACAATCACAACAAGGTGCAACTGCAAACTCCTACTATAGCGACGAGGATGATTGTGGATCAGAACTTTCATATCTAGATGAGGTTTATCTGTCACTTGATGATGATCTTGAAGTCATAAGCCCCCCTAATTAA

>AdNAC47--Aradu.L3QY1

ACATATATGGTTATGGTGTGAACTGGGTTTTGAAGTGGTGGGCCATTTTTCCTTGGAATTTTTGGGAGCACTTTGGGACAAGTTATCTCATACTTTCGGTGTGTTTTGTGCAATTTTGGACTGTTGGTTCCCCTTCCCTTCTCTCCCTCCAAATGCTATAAATATCACTCCACCCATGCCTTATGCTTTTACTACTCAAATTGTTAACATACTACTCAAATTAAACTTGTTTTGAGTTTAACCAATTTGATTTCTTAATTTTCTTGTTATGCAAAAAATAATTTATTAGAGGTCAAAAGTAGTTGGTGGTTGAGTTAGAGTAATTTGAACTTTCTGGGTAAGTTAGTTCATTTTATCATTTGAGGGTGGGTGGTTTGGGATTAGGATAGTGGTAGAGATAGTGTTATTTCAAAGAGAATGATCATTGCATACAGTGGTTGCAGACTACTGTGGAAGTATCACTTGAAAACTATTGTTATCCACAAAAGCTTCTATTTTTGGATCTTCCAAATCTTCAATACATATGGGCATGCCACTTAGCATCATAAAGCTTTGAGCTTTCTTGCTGGAAAATTGTGGGTTTATTTTTTTGGTTACTTTCTTGCTAATAAGTTTTCATAAACTTTGGTTGGTCTTTTTTGTTCTTTTAATCATAATGGGAGGGGCATCACTGCCACCTGGATTTCGTTTCCACCCAACGGATGAAGAATTATTGGGATATTACCTAAAAAGAAAAGTGGAAGGGCTTGAAATTGAGCTTGAGGTTATTCCTGTGATTGATTTGTGCAAGTTTGATCCTTGGGAATTGCCCGAGAAATCATTGTTGGCAAACAGAGACATGGAATGGTTCTTCTTTTGTCCAAGGGACCGCAAGTACCCAAATGGATCAAGAACTAACAGAGCCACCAAAGCTGGTTATTGGAAAGCCACTGGAAAAGACAAGAAAGTTGTGTGCCAATTTGATACTCCTTCCACTGTCACAGGATATAGAAAAACCCTTGTCTTCTACCGTGGCAGAGCCCCTTTAGGTGACAGAACTGATTGGCTCATGCATGAGTATCACCTCGCCGATGATCTCGGCCTAGCATCTACATGTTTTCAGGGTGGTTATGCCTTGTGTCGGGTTATTAAGAAGAATGAGAAGGTGAACAATGGGAATGATGCGTCAATGAGATTCTCCAATGAGCCCTTCTCCATTTCTGCTGATGCTTCATCTTCTCAACCAAGTTATTTGAACAGTGAGAGTGTTTACTCAAGCCCCAATGCTTCTTCACACAATGTGGCTCCAATGGCAGACTCTAACCAAGCTTCTATAAACACCAGTTCTTCATCAGAATTTTGGGTGTCCCCTGATCTGATTCTTGATTCTTCAAAGGACTACCCGCAACTAGAAAATACTTTTACAAGGTGTGACATACCAAGTAGTACAATGACACCATGCCTCTCATTGGATCAACCTGAAATTTCACCTTGTTCATCATACTCAAATTTTAATGGGCAACTTGGATTTTCCGATGATTTCAATATGATTGGCGGCATGTCACCTTACTCAATACAAGAAGATTTTATGTACTTTCATGGAAATGATGGGGATGTTTCTTATGGAAGTTATGATCATATTAATTCAGTTGAGTACCCTGAATACTTCTGAAAACAAAGAAATATCATTCTACAACTTTTCTTGATATGCAAGGTGTCATGACTGAAGTCGCTAGCAATCGAAATTTCAAGCGCAAACATAGTTTAGATGTTAGCACCAAAGAGCGAGTTACGATTTGGAAAGTGGAAAAGAAAATCAAGGGTTATAAGGTACAATACTAGCATGATTGTGCACATTACTATTTAAAGTGTAATTGAAGTTTAAAGGAGCTAAATCTTGGGATCAAATTCTTGTGGGGGATAGTTATTTTAGATGAATTTTATTTGGTGTGATCTGATAAAGGTTGCCTAAGACAACTCTTTTTAATGTATGTCTTAGACTTAAGAGTGATAAATAATGTGTACAATACTCTTTTGATGTCATAGTATCATGTTTACTTATCTGT

>AdNAC48--Aradu.L6S7Y

AAAAAAAAAAAAAATCTTAATTAGATTTCCCTCCATATTCTTCCCTATTACAAGAAAATAAAAATATAATCATGGGTCTTAGAGATATTGGTGCTTCATTGCCACCTGGGTTTCGGTTCTATCCAAGTGATGAAGAATTAGTTCTTCACTATCTTTACAAGAAGATCACAAATGAGGAAGTTCTCAAGGGTACTTTGATGGAAATTGATTTGCACACATGTGAGCCATGGCAGCTTCCTGAGGTGGCTAAGCTCAATGCAAATGAATGGTACTTCTTCAGCTTCCGTGACCGCAAATACGCCACCGGCTTTCGCACCAATCGCGCCACGACATCTGGCTACTGGAAAGCGACCGGCAAGGATCGTACGGTTCTCGATCCCCTCACCCGCGAGGTCGTAGGGATGCGGAAGACCTTGGTGTTCTACAAGAATAGAGCCCCAAATGGCATCAAAACTGGTTGGATCATGCATGAGTTTCGCTTGGAGACCCCACACATGCCACCTAAGGGCAAAACAGACAATAGTGCCAAACTAAGCCCACAATTCATGTATGAGGCCACACCTTCATCCCTAACTTTGGCTTCATCATCATCATCCCCACCAACAAACCAAACAAATTGCAACAATTTGCATGAAAATAGTACCATTACTCAACTAAGTCCCAAAGGTGGCGGTGGCGGTGACGACGGCGGCTATGGGTTCATGTGGGACATGGATCTTGAGGAAAATAGCTTCCATGATGGTGGGGTTATTGCATCAAACTTGAACGACATGAGATTTGAGGTTGATAATAACACTATGGTTATGTTGTAG

>AdNAC49--Aradu.L8SVN

TATATCTTGTAATGGTCCCCTTCCCCAAATGGATAACGGCAAGGCATTGCCAGATGACTAGGTATAACAATAACAAAGTGTTGTCTATCAATCAATTAAATCCATGCAACTTCACAAAACGACAAAGAGACCCTCACATAAAAGGAACACACATCTCATTGCAAACAGTTCAAACTCCTTAGCTTGTTCAATTTCACATAACAACAATGGAAGGAAGTAGTAAAAGTTGTGAACTACTACCACCAGGGTTTAGATTCCACCCAACAGATGAGGAGCTAATTGTGTATTACCTTTGTAACCAAGCAACATCAAAGCCCTGCCCTGCTTCCATCATCCCTGAAGTTGACATCTACAAATTTGATCCATGGGAATTGCCAGGGAAGGCTGAGTTTGGGGAGAAAGAATGGTACTTCTTTAGCCCAAGGGACAGGAAGTATCCCAATGGGGTTCGGCCGAACCGCGCAACGGTTTCTGGGTATTGGAAGGCCACAGGGACAGACAAGGCTATTTACAGCAAGTGTAAGCATGTTGGTGTCAAGAAGGCCTTGGTTTTCTACAAGGGTAGACCTCCAAAGGGGATCAAGACTGATTGGATTATGCACGAATATCGTCTTCTTCAACAATCTAATCACAACAGCAGGACCACTGGTTCTATGAGACTGGATGACTGCGTCTTGTGTAGGATATATAAGAAAAAACATGCTGCTAAAGCATTGGATCAAGGACAGGAATACCCAACAACGGTTCAAATTAATCTAAATGCATCAACCAACAATGATGATCAGAAGGAGTTGATGATGATGAAGAATCTTCCAAGGACTTGTTCCCTTACTTATCTTTTGGACATGAATTACTTTGGTCCAATCTCACAGCTATTGTCTGATGGATCCTACAACAACTCATCAACCTTTGAAATATTTCAACATAGCAATAGTGTTGACAACATTGGAATAGTGGATCCTCTTGTCAAAACTCAAATGGTTGAAATGGATGATAGCTATTATGCTCAAGATTCAGGCAAGTCCCAAGTGATGAAACAAGGGAATGATTTAAGAGGATATTACTAAGTAATTATTAACAAAAGAAAAAAGAAAAAATGTTATATTGTTTAGATTAGACATGATGAATTATGATAAGAGTCTTCTGTAAGATAATCATGAAGATTTTAAATATGTCAAATGTGTCCTATCACAGAAATGAAATTTTCAAATAGAATTTAGTATTCTAGTTCACGTTGACATTGTTCACCTTATCTGTACATAATAAGGACAAGACTATTACTGGTTCTTAATTGAATTTTCGGACTTATGAAAATAGTTTAAAAGATGCCTGGATCACGCCTTCTTAAGA

>AdNAC50--Aradu.LG4RX

ATGGATAACAGGTTGGCCACAAACTCCTCTTATGCTTCTCTTAGATTGCCCGTTGGCTACAGATTCTGCCCCTCTGACGAGGTTTTTGTCTCTTGCTACCTCAAAAACAAGGCCCTTTCAAAAACATTGGATTTTGATGTTGTTCCTGTCTTCGATGTCTTCAACACTGAGCCCAAGAATCTCCCTTCAGGAGGAAAGGTGTTTCTGGAGACAAAGTACTTTTACTTTGATCTGAAAGAGCGTGTGTTTGAAGACAATAACAAGATTGAAGCAGGGAAAGGGCACTGGAAAAGGGTGGGGAAAGGGAATCAGGAGCTTCTAAATAACAACAACAAACTCATTGGGTTCAAGACCAAGTTTGTTTTTTGGAGGAAGAAGAACCGCACTCAATTTCTTAAAACTAAGTGGGTTATGTTTGAGTTCCGTGTTTTTCTCAACCCCTCTCAGATAATGTCATCATGGGCTGGCTACAAAATATATCTGAAGAAGGATAAGAGGAGGAACAAGAAGGCAAAGTTTTCTTGCGAGGAAAGCAGTGACGATGATGAAGAAGAAGCAGAAAGAGCAAGTGAAGTAAATTTTGCAGATGAGATAAGTGGAATTAACACAGGACCTCCTTCACCAACTTCATCTAATGAATCCTCTGTTACAAATTAATTATTAGAGCAGCAAAAATTGTTCAGATGAGTTAGGAAATTCAATAGGAGTTGCTTGCAGCATAGGTCTTTTAATTTACTAGTATGAGAAAAATCATTTAGAGCCCTTTGGATACCCATTAACAACTTTTTTTTTTCCTTTTTAGTCATTAGAGATTAGGCAAATGAGAATTGTATATGGGCGTTTATTTTGTGATCAAGGGCTCTAACATTTGGTGGTGGTTATAATAATAATAAGAAGCAACTTGTTCATAATTTTATTAGTAGTACTCTTCGCAAATATATATGCTTTAATTTATTTGCTA

>AdNAC51--Aradu.LZ0D8

CTTATTAACCGTTTTTGTCTTCTAATAATATCCCTTACTAGCTTCATCATCTTCCTTAGAGAGAAAGAAAAAACACACACACACAAAACACAAAAAGAATAAATAAGAGAACATTATTATTGTTATAATTATTGTTATTATAACTTTTATATATGGGGGTTAATGAAGATTTGATGATGAAGGATGATTCATATGCATCATCAGTGATGGAGGAAGAGGATGATGTTCCACTTCCAGGGTTTAGATTCCACCCAACAGATGAAGAACTTGTGAGTTTCTATCTAAAGAGGAAGCTTGACAAGAAACCAATCAGCATCGAACTCATCAAACAGATTGATATCTACAAGTATGATCCTTGGGATCTTCCAAAAGCGAGTGGAAGTGGAGGAGAGAAGGAAGGTTACTTCTTTTGCAAGAGAGGGAGGAAGTATAGGAACAGCATAAGGCCTAACAGAGTCACCAGTTCCGGCTTCTGGAAAGCAACCGGGATAGACAAGCCGGTGTACTCCCATGGCGGCGAAGGAAGCGACTGCATTGGACTCAAGAAGACGCTTGTCTACTACCGCGGCAGCGCCGGAAAAGGTACCAAGACTGATTGGATGATGCACGAGTTTCGCCTCCCTTCTGCCACCACCGAAAACAAAACAAGCCTACTTGCCAACAACAAGAATAATAATAATATCAACAATGCCGATGTTGCCCAAGAAGCTGAAATCTGGACATTGTGTAGAATATTCAAGCGAAATGTGTCACAAAGGAAGCACACAGCAGACTTGAGATCACATTTAGTAACAGCTAATAGTAACAAGCACAAAACCACTAGAACCCATGTTGTTCAATCCAATAATAACAATATTAATCAACATCAAGAATCTTACATCAACTTTGGTGCAACAATCATTGGCCATCACCATTACCATCATCAAAATGAACAGAAGCCAGTGACTAACTACACAGCATGCAATAACAACACTGATCAAATCCAAAGGAACAATAGCAATCATCATCATCATCATCAGTTGAACTATCACCCTTCTTCAGCGGTGGCTACTACTGTGCCACAGCAACAACAACAACAATATCATCATCATCATCAGCTAATGACGGCTCCAGCTTCTAACATGTGGATTAATCCTTCTGCGATGAACGATTTGTTTGCATTTGATGATAACTGGGATGAGCTTGGATCCGTTCTCAAATTCGCT

>AdNAC52--Aradu.M7213

ATGCAGAAGTAGTACAAGTAGATGGAATCATCGTGTGTCCCACCTGGGTTTCGCTTCCACCCAACGGATGAAGAGCTTGTTGGTTATTATCTGAGGAAGAAAGTGGCATCTCAGAAGATAGACCTTGACGTTATCAGAGAGATCGATCTCTATCGTATTGAACCCTGGGATCTCCAAGAGAGATGTAGGATCGGGTATGAAGAGCAGAACGAGTGGTACTTCTTCAGCCACAAAGACAAGAAGTATCCGACGGGGACTCGAACGAACAGGGCCACCATGGCGGGGTTCTGGAAGGCCACGGGAAGAGACAAGGCGGTGTACGACAAGGCGAAGCTGATCGGGATGAGGAAGACTCTGGTCTTCTACAAAGGGAGAGCCCCTAACGGCCAGAAAACAGACTGGATCATGCACGAGTACAGACTTGAATCCGATGAAAACGGACCCCCTCAGGCAAGCCTTCTAGATTACTTGTATTATGTGTATGAGGAAGGGTGGGTTGTTTGTAGAGCATTCAAGAAAAGGACGACAAACGGGCAAACGAAGACTATGGAAGGATGGGATTCAAGCTACTTCTTCATGTGCAAGCAAGAAATAGAGAACATGCATGCAAATATAGCAGCAGAGCAATTTGTACAGCTTCCACAGCTTGAGAGCCCAAGTTTGCCGCTAGTTAAGAGGCCAACAACAACAACAAGCACAATGGCACTAGTCTCAGAAAGCAATGAAGAGCATAACATGTTATCTTGCAATAACACGAAGAAAGTAGTGACTGATTGGAGGGATCTTGATAAGTTTGTGGCATCTCAACTGAGTCATGGAGGAGACAATAGTAGGCACGAAACTGAAACCGATGATGCAGCAGTGCTCCCAAGCTTTATGGATAATAACAACCATGACAACAATGGTAGCATCTCGGACATGTTGATGATGAGCATGAGCCCCTTTCTAAACACAAGCTCTGACTGTGATATTGGGATATGCGTCTTCGAAAATTAA

>AdNAC53--Aradu.M8PFR

GTTTCAGGTTCCATCCTACTGATGAGGAACTTGTTATCTATTACCTCAAGCGCAAGGTTTGCGGCAAAAGCTTCCGATTTGATGCAATTTCTGAGGTTGACATCTACAGGAGCGAACCCTGGGACCTTGCAGTCGAGGTTGAAGACTAGGGACCAAGAATGGTACTTCTTTAGTGCACTGGACAAGAAGTATGGCAATGGTGGGAGGATGAACAGGGCCACAAGCAAAGGATACTGGAAGGCTACAGGGAACGATCGTCCGGTTAAGCATGAACAAAGGACTGTGGGGTTGAAGAAAACTCTGGTGTTCCATAGTGGAAGAGCCCCAGATGGTAAGAGGACCAATTGGGTCATGCATGAGTACCGACTCGTCGACGAAGAGCTGGAGAGGGCTAGGTCTGGATCCTCTCAGCCTCAGAAGGATGCATATGTTTTGTGTAGAGTTTTTCACAAAAATAACATAGGACCTCCGAATGGGCAACGTTATGCACCTTTCGTTGAAGAGGAGTGGGATGATGCATCGGCATTGGTTCCTGGGGCAGAACCTGTGGAGGATGTTACCGTCACTGTTGCCCATCCTCTACGCATTGAAAGCAACGGTCGCACTTTATGCAGCGACAGGAGAAACAATGTTGCACAGGATACTCAATCTAACAACAAAGTTCCATTTGATGTGAACAAGCTTCCCATTGAAACTCAAAGTCTGCTAGCTGTCTGCAAAAGGGAGAGTATGGCCGAGTTTCCATCACCTGAAAAGGAGGATAACTCGAAGCGTCAGATCGATGAGTATCCTTTGCCACAAACAGAAAACACCAAGCCTATCTCTCAAATATACAAGAGGAGGAGGCATTATTTGAATGTCAACCATTCAAATGTTAACGGAGATTCAGTCCGAACCATCCAAGAACCGCCATGTTCATCAACAATAACCACCGCCGCAACGACACTCCCGACGGTCGCCACCACAGCCTCCACTGCAATAACCAACGTTGCACCTAAAAAACATTTCTTGTCTGCACTGGTGGAGTTTTCTTTGATGGAATCCCTCGAATCAAAGGGAAATGCATCCGTTCAACCACCAGAGTTTGATGATGCTTCCTTGGAGGCATCCGTGCCGCCAAATTGTGTTAAGCTCATCAAACGCATGCAGGGCGAGATTTACAAACTTTCCGAGGAAAGGGAGACTATGAGGTTTGAGATGATGAGCGCACAAGCAATGATTAACATGCTCGAGTCGCGCATTGAAATTTTGAGCAAAGAAAATGAGGAACTGAAGAGCATGATTAATAACAATCCTTAGGGGATTAGCGCAGTGGCCATGTAATCTTGCTTATTGCAACAGAGTGATGGTGCTCTCAGTCTACCTGATCTCTTGTTATCTTTTAAGAGTTAAGAACTGTAGTCTTCTTCATTTGGCTGAACTTTCTGAGTGGTATTTAAACTTTGCCATTTGTGTAGCCTTGTAGCTTATGACTTGTTACCCTTGTTCATCAATGGCTATGTTAGTCCCTCCTTCCTTTAAGGCTTGTCCTTTCTTGTGAATGAATAGCAACTCAATTAGTTAGGCCATTCTGAACAGATTTTTCTCATAGAGGGGTTTGGCAAGTTGGGAATGGATCCTGTGCATAGATTTCACAAGATGAGT

>AdNAC54--Aradu.M9GL4

CATTCATCTCATTGCAAACATTTCAAACACCTTGATATCTCTTCAACCCTTATCTTCAATTCATCCCCATCCTCTATCTCTTGTTGTGTCCCCACTATAATTAATGGAGGGTAGTAGAAGAAGCTCAAATTCTGAACTCCCGCCTGGGTTTCGGTTTCACCCAACTGATGAAGAACTAATCGTTCACTACCTTTGTAACCAAGCCACTTCAAAGCCTTGCCCTGCTTCCGTCATCCCTGAAGTTGATATCTATAAGTTTGATCCATGGGAATTACCCGATAAAACAAGCTTTGGAGAGAACGAATGGTACTTCTTTAGCCCAAGGGATAGGAAGTACCCAAATGGGGTGAGGCCTAATAGGGCAACGGTTTCAGGGTATTGGAAGGCCACTGGTACGGACAAAGCAATCTATAGTGGGTCTAAGCATGTTGGGGTCAAGAAAGCTTTGGTCTTTTACAAGGGTAGGCCCCCAAAGGGTATCAAGACTGATTGGATCATGCATGAGTATAGATTGGTTGGATCAAGAAGGCAACCCACTAAACAAATTGGATCCATGAGGCTAGATGACTGGGTGTTATGCAGGATCTACAAGAAGAGGAGCATAGCAAAATCAATGTTGGAGCCTAAAGAGGAATTCCCAACAATGCCCCAAATCAATCATCATCTAACATCATCATCAAATGATGGGAATGATAATAATGATGATGAGCAAGAAATGATGATGAAATTCCCAAGGACATGTTCCCTTACACATCTCTTGGAAATGGACTACTTGGGCCCAATATCACAAATACTCTCTGATGGATCATATAACTCAACCTTTGATTTTCAACTAAACAGTGCCAATGTTGGCAACATGATTATGGACCCTTTTGTGAAACAACCTCAGATCCTTGAAATCCCTAACAAAAATAATCCTAACAATCCTTATTATGATGTGGATTCAGGGAAGAACAACCTAGTGAAACAGAATAGCACCATAAACCCTACTATATTTGTGAACCAATTCTTTGATCATAGTGGTAGTTAA

>AdNAC55--Aradu.N8F6V

CAGAAATTAAGGAAACAATGGGAGAAGTGTGAGAGTGGGGATGAAGATAAAAGCGGAAGCACGGACTAGTTGCGATCGAAGTGTACATGCGAATGACACGTTAAAAGAATGGCATCAAACATGCTTGGTTCCCTCAAAGTACAAAAGACCCAATTGTTGTCACTCACCAATAAACAAAGAACAGAACAACATATCTCTCTAACACCAAACCCTTTTACTTTCACCTTAGAATCATGGTGGATAGGGATTCAAGCGGAGCACACATGTCAATAGCAGCTTCTTCCATGTTTCCTGGCTTTAGGTTCTGTCCCACTGACCATGAGTTGATCTCTTATTACCTCAGAAAAAAATTGGACGGTGACGAGGACAGTGTTCAGATTATTTCCGAGCTTGAACTTTGCACCTTTGAGCCTTGGGATTTGCCTGAAAAATCGTTCATTAAATCAAACGATGAGTGGTTTTTTTTCTCGCGACGAGGGAGAAAGTATCCTAATGGTTCACAAAATAAAAGGGCAACTAAACATGGGTATTGGAGGGTCACATGCAATGAACGACAGATAAAGTCTGGTCAAAATGTGATTGGTACCAAACGCACCTTGGTATTCCATGTCGGCCGAGCTCCTAAAGGCCAGAGAACTGAATGGATTATTCATGAGTACTGCATCAATGACAAATTTCAGGATTCTTTGGTGGTTTGTCGGCTCAAGAGAAACACAAAATTCCATGCGAGTGATAGTTCTAACAAAGCTTTACGCAAGAGTGGTGGTGGAGTCTCAGAAGGGGTTACAGTTCAAAGGAGCACTTGTGTGCCTATTCAAGATCGTTCTAACAAAACTTCATGCAAGAGTAGTGCTTCACGCAAGAGTAGTTGTGGAGTCTCGGAAGGGATTACAATTCAAAGGAGCACTTGTGCGCCTATTCAAGATCGTTCTAACAAAGCTTCACGCAAGAGTAGTTCTTCACGCAAGAGTAGTTGTGGAGTTTCGAAAGGGGTTACGGTTCAAAGGAGCACTTGTGTGCTTATTCAAGATCGTATTAACAAAGCTTCACGCAAGAATTGTTGTGGAGTCTCAGAAGAAGGAGTTACAGTTCAAAGGAGCACTTGTGTGCCTATTCAAGATAAAGAGGTTGGATGTAGTTCCAAGAAGGGTAACAATAATAATAGTTCTCCTTCTACTACTGCCCAAATTGAATCCAGTCGTATAGTTGCCAATGAAGCCAATCCCAAAGCTTCTTCCGGTCATTCTAAGGTGGTGGACGAAGTGGGTTATTATGCAGAGATCAACTTAGTTGATATCATCAACTTAGATGAAACAGCACTCTGACGGCCATAGCCCATAGCCACCACAAGGGACAACAAATAGAGAATCACGCGGTTTCTAGATCAAAGGTTTCAACACAACTTCTTGACCAATGCTCCAACCAATCATCAAACAAAATCAACACTTTCCTTTCCTGCTATACGGTTTGCTGCTCTTCACTTTCTTCGTTTGCACTTTACTAGCTCTAAGCTTTTTTCTTATCGGGAAGTCTCAAACAACTGTATAATATTCTCCAGACCTCTCTCAGAGTTTAATTATGATTATTAATTAGTTAAAA

>AdNAC56--Aradu.N8MU8

GTTAGATATATGCATTTAACTATGATATCTAATATGAGGATTCTTCTTGTTGAAGTTGTAAGGAGTTATAAAACCATAAAGTGGAGAACCATTTAAGAACCTCATCTTGGTCATGTTATAATTGATGAGGATTCTTCACATCATAGCTTGGTAAACTGAAATTAATTAAGCTCATCATCAAAGGGGTTTTAGGGTTCTTCATAGGAAGAGTAATTGATGGAAGAGAATCTACCTCCTGGATTCAGATTCCACCCAACAGATGAAGAGCTTATAACATATTATCTTACAAGAAAAGTCTCTGAAAATGGATTCACTTCTAAAGCTATTGCTGTTGTTGATCTCAACAAGTCTGAGCCTTGGGACCTTCCAGGTAAGGCAAGCATGGGTGAGAAGGAATGGTACTTCTTCAGTTTAAGAGATAGAAAGTATCCAACAGGACTAAGAACAAATAGGGCAACAGAATCAGGGTATTGGAAGACCACAGGCAAAGACAAAGAGATATTTCGTGGTGGGGTTTTGGTTGGAATGAAGAAAACCCTAGTCTTTTATAAGGGTAGGGCTCCAAGGGGTGAGAAAAGTAATTGGGTCATGCATGAATATAGACTTGAGAACAAGAATCCCTTTAGAACTAAGGATGAATGGGTAGTGTGCAGGGTATTCCAAAAGAGCACAGCAGCGAAAAAACCGCCGCAACAAACATCATCCTCCCAACCTGAATCCCCATGTGATGACACAACCTCTTTGGTCAATGAATTTGGTGATGTTATTGAGCTTCCAAATCTAAACACCAACATTAATAATAATAATAATTCTTCATCATCATCCTCAAGTGCTTTATTCCCTAACAACATTCTTATTTCAGGACAACACATTCATCATCACCATGACCTAACCAATAATAATAACAATAATAATGTTAACACAAACATGAACTTAGCAATGAATTGGCCACCATCAAGTGATCATAATATTAATAATGTTCCATGGCCTTCAGTAGGGTTGTTGAATCCAAGTATTTCATCAATGAATTCCTTGATTCTCAAGGCATTGCAGCTTAGGAATAATTATCAACAAAGAGAAGTTGCATCCACATTTCCACCATCATCATATATTATGCCTCATCATCAAGGACTAGTAGTTCCTCATCAACAAGTTATTGGAACCAATAATAATGATGACCTAATAACAACTTCTTCAAATCTCATCAATGCTTCTTCTTCTTCATCAAAAGTTTTGGAATGTATGCCACATCAGCAACAACAACAACAACAGGAGCAACCATTCAATTTGGACTCCCTTTGGTAA

>AdNAC57--Aradu.NEU1C

ATGGCAGAAACTCGGGTTCTACCTGTTGGATATAGGTTTCGTCCAACAGAAGAGGAAATTTTAATTCACTATCTCAATAACAAGCATTTGGGAAATGATGCAGAGATTAAGAACACTATTTCCCAAGTTGATCTTTGTAACTTTGATCCTTGGGATTTACCAGAACAATCGAAAGTGAAATCGGATGATCAAGAATGGTTTTTCTTCAATGAATTGAAATACATGAAAAACAAGCGGTGTAACAGAAAAACCAACATGGGATATTGGAAGATCACAGGAAAAGAGAGAATCGGGACAGACAGTGTCATAGGTACAAAAAGAACACTAGTTTTCTACGAGCGTCCACATAATGTCAAAACCAATTGGGTTCTTCATGAATATCATGCATTTGATCAAAAGGTAGGTTCTTGCCAGAGCAACATCGTATTGAGCCGTGTAATAATGAATGCTGAGAAAAGGGAACAGAAGCTAAAGACAAAAGCAAGCAACATAGTCGAAGAGGAGGAAGCATGTGTTTCATCCGAGCGACAACAACCTCAAGTTATAGATTATGAAATTCTCTCATCGGGACAACAATCTTCAGTGGCCCATTCCGGTAATGAAAACAATAATGCCGCGGAAGCAACATGGAGGCAAGATGCCGATATGAATATCGAGTATTTTTGGAATTTGCTGTTTTCTAGCATCGATGCTGACCCTCATGCTGAGTTCTTAAATTCGGTGTTGGCAGGGGATGATCAACTCTATGTTGATTCCGGCCACCATTGACTTTACACAGGTACAAGTAAAGATGTAAAAAAAAAATTTCTTTCATAGACATAGACAACTCATAGGGATGTTTTCGAGCAAGCTTTTTTTGACAAGAAATTTGACCCCCAAGTTGTAGTTGTTATACTTCAACAACATCAGATCTACAATAATGTATGTGGGGTGAGAGAGGCTCGAACTCTCGACGTCAGGATAACTCTAAAGCTATGAGAC

>AdNAC58--Aradu.R9F07

GGGACCCTTTGGTGAATTGAACAACAATCAAGAACAACCAATTTTAGACCTTTATATATTACATACATATCTTTGATTTGTTAGTTGTTGACCTGAATGCCAGAAAGCATGAGTATATCAGTGAATGGACAATCTCAAGTTCCACCTGGATTCAGGTTCCATCCAACTGAGGAAGAACTCCTTCAATACTACTTAAGGAAGAAGGTCTCTTATGAGAAGATTGATCTTGATGTTATTCGTGACGTTGATCTCAACAAGCTTGAACCATGGGACATACAAGAGAAATGTAAGATAGGAACCACCCCACAAAATGATTGGTACTTCTTCAGCCACAAAGACAAGAAGTACCCGACCGGAACCCGGACAAATAGAGCGACCGCGGCCGGGTTCTGGAAGGCCACCGGCCGCGACAAGGTGATATACAGCAACGGGAAGAGGATTGGAATGAGGAAGACGTTGGTATTCTACAAAGGAAGAGCCCCTCATGGCCAAAAATCTGATTGGATCATGCATGAATATAGGCTTGATGATAACACCACCAACGATGCCAATATTGTTTCAAATGTGATGGGAGATGCAGCACAAGAAGAAGGGTGGGTGGTGTGTAGAATATTCAAGAAGAAGAACCATCTAAAAACCCTAGATAGTCCTTTAACCTCTTCCATCTCCGGCGACGGAGGTAGGAGGAGCCACCACCACCACCACCTATTCGACTCGTGCGACGAGGGCGCCTTAGAGCAAATTCTCCAACAAATGGGAAGAGGTGGTGGTAGTGGTGGTTGCAAGGAAGAGATCAACAACTATGATCAATCTAATAACAACAACAACAACAACTATGGTGGATCATCATCGTTAACAACAAGGTATGCAAGACCTTTTGACACAATCAACAACAATGTTGATAGCAGGTTCTTGAAGCTCCCAAGCCTAGAGAGTCCAAAATCAACAAGCATGGATCATAATAATAATAACAACAACAATGATAATGATGATAGCAATGAAAATAATGGGTACCATCCTATTATTCCAGTTGAGATGGTAACTGAAAATGAAGGGTCATTCACATGTGACAATCCCAACAACATGTTTCATCATCACCATTTGGGTGGTGGCGGTGGTGGCAGCAGCGACGGCGGTGGCGGTCTTACAAATTGGGTAGCGCTAGATAGGCTTGTTGCTTCTCAGCTTAACGGTCAGACCGAAGCTTCTAGACAACTCTCTTGCTTCAATGATCCCACCATGGGGTATGGCACTGGAAATCATGATCTTCTATTTCCATCCGTCAGATCTACTTCATCGTTGACGTCATCGTCAGCGTCAATAAATCCAAGGGCTGTTATTAGTGCGGGTGCAGGTGCATACATTTCTCCAGGCGCACAGGATTATACCACCACAAGCGAGATTGACCTGTGGAACTTTGCTAGATCCACTTCTTCGTTGTTGTCATCCTCTGAGCCATTGTGCCACGTGTCAAACACGTCAGTGTAG

>AdNAC59--Aradu.RP61F

ATGGAACAAGAAGAAGAACCACAACAAAATGAGCCACCTCACTCTCACTCTCAATCTCGGTGCGTGACGCTACCTCCCGGTTGCCGGTTCCATCCTTCGGAGGAGCTTCTATTGAGTTACTACCTCACCAACAAAAATGGCACGGGGAACTGGAATGGTAACGGTGGTTTGGGATTCGATGGTTCTGATTTGATTCGGGAGCTGGATTTTTACGATTACGATCCTTTTGAACTTCCGGATTTTGCGTGCTTTGCGTACGGCTACGGCGGGAGGAGGAGGCACTGGTACTGTTTCACCACCGTTAGGGTTTCGAGGGGAGAGAGGTGGAAGAGGAAGAGGAAGGTTAAGAGTGGGTTCTGGTTGAAGAGGGGAAGGGTTTCGAATGTTAACGGTGTTGGGGAGAACGTGGTTTTGGGAACGAGGACAAGGTTCGTTTTCTATATGGGTGATTCGGCGAAGAACGGTGCCAGGACGGATTGGGTTTTGTATGAATACGCATTGGTTGATCATGTTATGGCCTCTTTTGTTCTTTGCCGGGTATTTAGTAAGCCTCGTTATAAGAATAGTGCATCAGACATCGGCCTGAGTTGTTGTGCAGAAGAGAGTGTATCAGCAGTGCGCCATATTGGTATTCAGCATGATGAACATGTTAAATTGGATGCCGTTGAAGCTAAAGTATGTGATGATATCTCCATTGACCACAACAATGAAATATGTGCTGGTGGAAACAGCGATAATCAAGTTAAGAATGCACATGACATAGATGCTTTACGATGTTTGGCGGCTCCTCAGGGCAGTCAGCAGGAAAGGCTTCCTTTACTCCCCAGCGGTAGTACAATGTTCATTGAAGCAATTTCATCTCAACAACAATTACTTTCCATCACGGAGGAAGACTTCATAGAGTTGAATGATCTTACATGAATTGGATGGCTTTGAGAAAGATTTCAATATTAATCCTGCTCTCAGGAGTATTCCCCTGAAACTCATTAGTTCCGAATGGGTAAATATCCCATCAAAGTGGATAAACATTCACAACGTCGGAGAAATATTTACGATATCAGGTAAACCTTTGGCTGTCATATGAAGATCATATTGTCAGATCAGGTGAAGGAAAACATCACAGGGTGATCGCTTCACAAAATAAGGTAGTTACTTTGATGACATTTCAGAGTACGTTAAAGTCTTGAGCCATACCGGAGGATATTTAGTAGATATTGGTTTTGGGCATGCAGATTTTCAACTTTGCCTACTGAAAGGCAAGTTATGCAACTGGCTCCAGAGGAATAGAGTTAGGAGTTAGTTTATTGGCATGGTGATTGCAATATGCAATTTACTTCAAGTATGGCAAACCTGACGTTAGTTCCTCATGCTGGCATTGATCCCTT

>AdNAC60--Aradu.RRT20

CTAAGGATCCTCGGGATCTATCTCATCACCTCCATCATTCTTGAGTGCACAAGGCCGATTCTTATCGATAGAGAAATATCCCATCAAGTTATACCACGTACAAGAAAAATCTACGTACAGCGATTCTACTTGGAGTTCTTAGGTGCAAGATATATCAAGCTAAGGAGGTTTGAAATGAACGCATTTTCACATGTTCCTCCTGGGTTTCGGTTCCATCCTACGGATGAAGAACTTGTTGATTACTACCTGAGGAAAAAGATAGCTTCGAAAAGGATTGATCTGGATGTGATAAAAGATGTGGATCTCTATAAAATTGAGCCATGGGATCTTCAAGAAATATGCAAAATAGGAAGCGATGATGAAAATGAATGGTACTTCTTTAGCCATAAAGATAAGAAGTACCCAACAGGGACTCGCACCAATAGAGCTACAAAGGCAGGATTCTGGAAAGCCACGGGAAGAGACAAAGCCATATACTCAAGCTCAAGCCATTGCCTCGTTGGTATGAGAAAGACTCTTGTGTTCTACAAAGGACGAGCTCCCAATGGCCTCAAATCAAACTGGATCATGCACGAGTATCGTCTTGACTCCAATCAGGAAGATGGCTGGGTTGTGTGCAGAGTCTTCAAGAAGCGGATGCCCACGCTACGCAACGTGGTAGACTATGATGATCAACTTCCCTTCATGCAAGGATCTCCATCCACTCACTATCCCTGCAAGCACGAGCTTCATCAATTCCAATACAACACCCATGATGCTTTTCTCCAACTTCCACACCTTGAGAGCCCTAATCAAGTTTTGAGTTGCGGCAGCCCCGTTATTGCACCCTATGCCTACGCCGAAAACAACAACAACAATAATGGAACTAGTAGTACTAGTGCTTTGCAGTCCTATTCATCTGAACGCATTCAGCAACAACTTCACTTGCTTTATGGTAGCAATATTGAGCAAGCAGTAGTGATGGACCAAGTCACGGATTGGAGAGTGCTTGACAAATTTGTTGCTTCTCAACTCATGAGTCAAGATCAAGATCAGGCTTCCAAGGAAACCTGCAGCGTGGCTGATGAACAACATGTTGCTACTACTGTGCTTCCAAATGGATCCACGAAGCAGGAAATGGTGCCTCAGGACGACTATGTTTCAACGTCTGCCTCCAGTAACTGCGATATTCACCTGTGGAATTGA

>AdNAC61--Aradu.S13QQ

CATGCACCACTTATGTTTGTATGTGTATATATATAACACATGCACAGTGTTTTCAATCTTACCACCACCTTAATCGATAATACCGGCAGCTGATTGATACAGTTTCCTAGTTTTCCTCGAAAGCGTTATAGTAGTTTCCATCTTCTTCATGGACAAGGATACTAGTTTGGAAATCCATCTCCCTCCTGGATTTAGATTCCACCCTTCTGATGAAGAGTTAATTGTTCACTATCTAAGAAACAAAGTCACTTCTTCACCACTTCCTGCCTCATTCATAGCAGAGATAGACCTCTACAAGTTCAATCCATGGGAGCTTCCAAGCAAAGCTTTGTTTGGGGAAGAAGAGTGGTATTTCTTTACTCCAAGAGAGAGGAAGTACCCAAATGGAGTGAGACCAAACAGAGCAGCTGGTGCAGGTTACTGGAAGGCCACTGGAACTGACAAACCAATTATCACGTCAGGTGGTATGAAGAGCATTGGAGTGAAGAAAGCCCTTGTCTTCTACAAGGGACGTCCCCCAAAGGGATCCAAAACTGATTGGATCATGCATGAGTATAGGTTGCATGATTCACTCCTCTCAAATTCTCACAAAAGAGGCTCCATGAGACTTGATGAGTGGGTGCTATGCCGGGTGAGACAGAAAACAGGCAGCCCAAGAAGCACTTTGGAAGATCCAAGTGAACTGATTTATGAACCAACAAAAAAGATTCAACAAATGAATGATGAGAACTTCAATCCTGAACTAGTAAAAGCCTCCATTGTGCACAACGAATTTCCAATGTTACCTTATATTCTGGCTTCTAGGAGTACTTTGCCTAATTCCATTGGTGTGTCCTCAAGCACAGGCTTTGTTAGAAATTGTGATATGAAACAATATGGTTCAGTGCATGAAGACAACAACTTGAATGTAATAGGAGCACAGTTCTTAGCATCTGCAATGGAGGGCTTGTATAATAATCCTCTGAAGAGAAAATTCATTGAACAAGAAGAAAACCATTTGGAATATGCTCCCCCAAATAAGAAAATAAGCCTAGAACTTGGTGATGATGTTGATAATAGTGATGATAAGCCAAGTCTTGTGATGGATACAAACAAAGGCTACAATTTTGGCTTCTTTGATCAGTGGAATTCAATCATACAACCTCAAGAGCTTAACAGCTTAGCCTTCATGGGATATTCATGATCCATGAGTTTTATTATTTGACTACAATAATAAGCTATCTTGATCTGAACTCACTTTCTAAAATCATGTGGTACCCATTATTACCATTTCTCTTTTCATGATATGCTCTTTTTTCACTTGGTGTTGGTGCCTCATCAAGAAGTGAGTTGTACAGGGCATGCTTGCTTGTTCTGCATGGGAATGGATGGTAAGTGAAGTGGTTTATATGTAAATAAGCAAGAATAATGCAGTGTGGTAATTATATTCTTTTCAGCACATACATAAGAATGTACATAGTTGAAAATGTATATCATTACAGGATACTACTGTATCAATTGTGTGTGCATAGAGTAGAGATAGCTTGAAGAATAAAGTGCCTGCATAATGCAGTTTAATTGCTCATTGTATCGTGCCTTCACA

>AdNAC62--Aradu.TGA11

AACATAACAACACAAGAAGAGAACCATAATTAAGCAAGCGACATATACATAATACATAATTGATGATTGATATGGGTTCTTCATCAGTAATAGAAGGTGAAGTTACACTTCCAGGATTCAGGTTTCACCCTACTGAAGAAGAGCTCCTTGATTTCTATCTCAAGAACATGGTCGTTGGAAAGAAGCTCCGTTTCGATGTCATCGGCTTCCTCAACATCTATCACCATGATCCCTGGGACTTGCCAGGATTGGCTAAAGTGGGAGAGAGGGAATGGTATTTCTTTGTGCCTCGGGACAGAAAGCATGGCACCGGGGGAAGGCCAAACCGGACCACCGAGAAAGGGTTCTGGAAAGCAACCGGTTCCGACCGTAAGATCGTTACCTTGTCTGATCCGAAGCGCATAATTGGATTGAGGAAGACACTGGTTTTCTATGAGGGAAGAGCTCCACGTGGATCCAAGACCGATTGGGTCATGAATGAGTACCGTTTACCTGACAATTGCCCCTTGCCTAAGGACATAGTGTTATGCAAGATATATAGGAAGGCGACTTCGTTGAAAGTGTTGGAGCAAAGAGCAGCAATAGAGGAAGAGATGAAGCAAATGGTAGGTTCCCCTGAATCCACACCTTCCTCCACAGACACCATGTCCTATGAAGAACAACAACAGAATCAGAATCAGAATCAGAATCTGCAATTGTTACCACCACAACATGTTGTTACTAAGAAAGAGGTTGAAGCTGAACTTGAAGAGGAAAAAATGGTACATGTTACATTGGCAACAACAAAGCAAGAAAACAAGGACACAACAAAGAACAATAAAAGTAGTTGTTGTGGTAACACAAACACTAACAGTAACACAAGTAGTCTTCAATTGCCATTTGGGAAGGATAAGATCCCAGAGCTTCAAATGCCTATGATGATCACTGATTGGACCCAAGACACATTTTGGGCTCAATTGAATAGTCCTTGGCTCCAAAACTATACCTACTCCAACATATTAAACTTCTAG

>AdNAC63--Aradu.TI0Z7

ATCTCTCAAACCTAATCATCAATAATTCAATCAAACCTTGGGAGTTTGCAACATCTTTCTCTCAGACCTATTCTTTTCTCTCTTCTCCCCTTCTTCCTAGCAAGCAGGCTCTCACTATATATTAATTTAATTTGTTGAAATTAAATAGAAAAAAGAATAATGAGCAACATAAGCTTGGTAGAGGCAAGGCTTCCACCAGGGTTCAGATTTCATCCAAAAGATGAAGAGCTTGTGTGTGATTACTTGATGAAGAAGTTCACGCACAATGAATCCCTTCTCATGATTGATGTCGACCTCAACAAGTGTGAGCCATGGGATATTCCTGAAACAGCATGTGTGGGAGGGAAGGAGTGGTACTTCTACACACAGAGAGACAGAAAGTATGCAACGGGTCTGCGTACAAACAGAGCAACGGCATCAGGATATTGGAAGGCCACTGGCAAGGACAGGCCTATCCTTAGGAAGGGCAGCCTTGTTGGTATGCGAAAGACTCTTGTCTTCTATCAAGGTCGGGCTCCCAAAGGCCGTAAGACTGAGTGGGTCATGCATGAGTTTCGCGTTGAACCTCCTCTTCCTCCCCCCAACACTACTTCTTCTAAGGAAGATTGGGTGTTGTGTAGGGTATTCTACAAGAACAGAGAAGTTGGTGGCAAACCTAATAGCATGGGAAGCTGTTATGATGACACGGGCTCTTCATCTCTTCCAGCATTAATGGATTCTTACATCAGCTTTGACCAACAACAACAACCTCAAACCCATCTTCATGCTGATGAGTATGAGCAAGTGCCCTGCTTCTCCATTTTCTCTCACACCCAAACAAGCCCTATTTTCAACCACATAATGGAGCCTAAGTTATTCCCTACCAACAACAACAACAATAATGCAACTTTATATGGTGGAGGAGGAACTACTACAACACCCAATTTGGGTTCTTGCTTAGACCCTTTTTCATGTGATAGGAAAGTATTGAAAGCTGTTTTGAGTCAGCTCACAAATATGGAAAGAAACATACCTAATAATAATAACAACAATACAAATAGTATAAAAGGGTCACCAAGTTTAGGAGAAGGTAGTTCTGAGAGTTACTTATCTGAGGTTGGCATGCCCAACTTGTGGAACAATTATTGA

>AdNAC64--Aradu.U974Q

CCCTCCTCTTCCCTCACCTATTCTCTCGCTCTCTCTCATTTTTCCACATTAAGGTCTCTACTCTTTTAATATTATGGAAGGGAGAGCATTATTATTGCATCATTGGAGATCATCGCTTGGATTATTGAGGTTTCTGATAGTTTGTTAAGTGGGGTATTAGAGATCATATAATATTTAGATATTATAATGGCACCAGTTTCATTGCCTCCTGGTTTTAGGTTCCACCCTACAGACGAAGAACTGGTTGCTTATTACCTTAAAAGGAAGATCAATGGCCGTAAAATTGATCTCGAGATCATTCCTGAAGTTGATCTATACAAGTGCGAACCGTGGGACTTGCCAGGGAGGTCGTTGTTGCCGGGTAAGGATTTGGAGTGGTACTTCTTTAGCCCTCGGGACAGGAAGTATCCAAATGGGTCAAGAACCAACAGAGCAACGAAATGCGGGTACTGGAAGGCCACTGGAAAGGACAGGAAGGTAAATTCGCAGAGCCGCGCCGTAGGGATGAAGAAAACCCTTGTGTACTACCGAGGCAGGGCGCCTCACGGGTCTCGCACTGGTTGGGTCATGCACGAGTACCGTCTTCATGAGAGGGAATGCGAAACCAATGCTGCTTCTGGCTTGCAGGATGCCTATGCTCTTTGCCGTGTCTTCAAGAAGGCGGCGGTCATACTCCCCAAAGTGGCAGATCACTATGCTGGTAACAATATCATGATGATGACAACTGATTCTCAAGGAACACCCCAAGTCTTTGACACCATGCCTTGGGATCATCATATTGGGCATAATGGTAAATGCCCACACTTATCTCAGGATCCATTCCTCAATAACCTTCCATCTTCATCATCCTCCTCATTTCCTCACTATGGAGCCCTAACTTACTCTCCATCTAAGGTGGATGTAGCACTAGAGTGTGCAAGGATGCAGCACAGTTTTTCCATGCCTCCATTGGAGGTAGTGGAGGAATTCCCTAATGTGGGAATTTCAGAGCTCAACATTATGACACGTGGCACCACTTCAATGTGTGGAGGAAGCATGAACAATAACAATGAATCGGATATCTTGCAACAGATTCTTTCACTTGCTAATGCTAATGTTTCTTCCCATGAATTCACAAATCAATCAAACCATTCACACACATTATTGGCTGGCAACAATAATGCAAATTATTCTGCTCCTCATCATCACGAACATGATTTTGCTTTTAATGCTGGCACAAGTTACACTAATCACGCCGTAAATGATATGAACCCCATGAGATATGAAATCCAACATCAAAACCTAAGAACAATAGAGATTGGAGATCTTGAAAGCGAGTTCAAGAGCTTTATGGAAGAGCAAAAGACGGTTCCGATTGAGGATATATCAAGCTTCCAAACAAACATACAAGAAAATGAGGTTCAAGAATCTGAGCTACACAACAGCAACAAAGAATTCAGTGAAGCTGACATTGACAATTTCTCAATGGGGTTCATCAACGATGGTGACCCAAATGAGAACTTCATCGATGAAGATGACAACATTGATTATTCAAATTCCACAAGCTTTGAGGTCCTTGAAGAAACCAAGGTTAGCCATGGAATGTTTGTGGCGACTCGCCGAGTAGCCGACACATTCTTTCATCAGATTGTTCCTTCACAAACCATCAAAGTTCAACTGAATCCAGTAACAATAATGGGCAACAATTCTTCCATGGAGATGCTCAAGAATAATCAAGAGTCTTTGTTCAAGAAGCTGATGATGATGAAGTCACCAAATACATTAGCAAGTGCTATTGTATTTATCTTTGCACTATTGTTGACGCTTTGTGTTAATTTGAAGGGGCAAGTTGAAAATTATTGGGCATCAAGAAGTGATGATGATACGATTAATGTGAAGAAGAAATGTTGCTATGGTGCTAATAGAAGCATGAAGAGAATGAAACAAGTAGCTCACAAGATCATATGGAACCAGCAAGAAAAATCTTGGTGTGTTGGAATTAAAAGTGGGAGAGGATTTAGTGTGGTGTTGAAGAAAATTGGTATTTTCCTCTCCATATCTTTGGCTCTTTGTACCATGTGGGTTAACCATGTTACAATTAGTCCTTAATTGACCTAGTAATTTTATTTTCTTTTAGCTTCTAACTTAATTAAGGATTTCATTATTTAGAAGGATACTTGTATTTTCTTCTCACAACAATTCTTGTATTTTACTTGGTCAATATGTTATTTAATTAAGA

>AdNAC65--Aradu.USH95

AGAAAACACAACCACCAAGATTCTCCTCATTCATTCATTCCAACAAAAGGATCTTTACTTAGTTCCTTTTGTTCCTCCCCAAAAAAGAACACTCTTTATTTATTTTCCTTTCAACACTCTTCACCATGGACAGCTTCTACCACCACCATAACCACCACTTTGACAACAGCGACACTCACTTGCCACCTGGATTCCGATTCCACCCCACCGATGAAGAACTCATCACCTACTACCTCGTCAAGAAGGTTCTCGACAACACCTTCACCGGAAGAGCCATAGCTGAAGTTGACCTCAACAAGTGTGAGCCATGGGAGCTCCCTGAGAAAGCGAAGATGGGTGAGAAAGAATGGTACTTCTTCAGCTTAAGGGACAGGAAGTACCCAACTGGGTTGCGCACAAATAGAGCGACGGAAGCTGGGTACTGGAAAGCCACTGGAAAAGACAGAGAGATCTACAGCTCCAAGACTTGTGCTCTTGTTGGAATGAAGAAGACACTTGTTTTCTACAGAGGAAGAGCTCCCAAGGGTGAGAAGAGCAACTGGGTGATGCATGAGTATCGGCTTGAAGGCAAATTTGCATACCATTATCTCTCTAGAAGCTCCAAGGATGAGTGGGTGATTTCTCGTGTGTTCCAGAAGACAACCACCGGGGGTGGTGGTGGAGGTGGAGGTGGAGGCTCAGCCGTGTCAACCACCGCCGGCGGGTCCAAGAAGGCGAAAATGAGCACTTCCACCACTTCTACAATGAGCTTCTGCCCTGAACCAAGTTCTCCCTCTTCGGTTTACCTTCCACCACTTCTCGACTCTTCACCTTACACTACCGCCACCACCGGCTCCGTCACCTCCGCCGCCGCAGCATACGACGGCCGCCAGAGCTCCTCCTTCGACAACAACAACAACAACAACGATAGCACAAGGGAGCACGTGTCCTGTTTCTCCACAATCTCCAACAACTTTGTCAATGGGTTCTTCGATCTTGCTCCTATGGATTCCTTCGCTCGATTCCAAAGAAACAACAATGTCGGTGTTTCTGCATTCCCAAGTCTAAGGTCGCTGCAAGATAACCTTCAAATTAACCCTTTGTTTTTCTCCGCCGCAGCGGCGCAGCCTCTCCACGGCGGCGAGCTTCACGCCGCGGGGACCTGGCCGGTGCCGGATGATCAGAGGGTTGCCGAGGCTGCTGCCGCCGGCATGGCTTTGGGACATTCCGAGCTTGATTGCATGTGGGGCTATTGA

>AdNAC66--Aradu.UXN6T

GAATAATGCAAGGTGGATTAGAGTTACCGCCAGGGTTCAGGTTTCACCCGAGCGACGAGGAATTGGTGAACCACTATCTCTGCAAGAAATGCGCAAAGCAATCAATTGCTGCTCCAATAATTAAAGAAATCGATTTGTACAAGTTCGATCCGTGGCAGCTTCCAGAGATGGCGTTGTACGGAGAGAAGGAGTGGTACTTTTTTTCGCCGAGGGATAGGAAATATCCGAACGGATCCCGGCCGAACCGGGCGGCTGGGAGCGGGTACTGGAAGGCGACCGGGGCGGATAAGCCGATAGGGAAGCCGAAGGCGTTAGGGATAAAGAAAGCGCTAGTGTTCTACGCCGGAAAGGCCCCGAAAGGAGTGAAGACTAATTGGATTATGCATGAGTACCGTCTCGCCAATGTTGACAGATCCGCCGCCAACAAACTCAACAACAACAACTTGAGGCTTGATGATTGGGTGTTGTGTCGAATCTACAACAAGAAAGGGAAGATTGAGAAATTCAACTCTGCCACAACAGGGTTGGAACAGAAACTACCAAAGTTTTCCCCAGGAGAGATACTTCACTATGATCATGAGCATGAGCATGAGACCAAGCCAAAGATTATCCACAATTTCTCCAACAATGAGCACCAATTGTACATGGACACATCAGATTCCGTTCCAAGGCTGCACACGGACTCTAGCTGCTCGGATCACGCGGTTTCGCCGGACGCCACCTGCGACAAGGAGGTGGAGAGCAACCCAAAGTGGAGCAATGAGCTAGATATGCAGCTGTTTGATACCTTTGATTTTCAGCTCAACAACTATGATAATAACCTCCCAATGAATGATGATGACCTTTTTGGAAATCAGTTCCAAATGAATCAGCTCATGTCTTTCCAAGACACATTCTTGTTCCCACAAAAGCCATTTTGATTCTTTTGACCATTAAGAATTATTGGGAATTGGAAGAAGAGGGGGAGAAAAAAATTGAACCTTTTTTTCTGGGAAAATGTGGTGGAGGACTATTGCCTAATGAGATCATGGGGACCAAAGCATATGTTCATTTTTTGTAGTGAAAACTCAGCATTTACACAATTCACACCTACCTAACAATTTCACTTTTGGACCATTGGGTTTTGGTGGCTATTCTTTGTTCTTGGGAGATTAGGGAGGGAGATGTGAAGG

>AdNAC67--Aradu.VUC67

ATAATATTTTTCGATCTCTATTCATTAATTAACAAAAGTAAAATAGAGATATTAGTGAAATGAGTTATTATTGGTTGTTTTTTATCAGGAGAGAGCATCAATGGATATGGAATCATGTGTGCCTCCAGGATTTAGATTTCACCCAACAGAAGAAGAACTTGTGGGGTATTACCTCAAGAGGAAAATTAACTCCCTCAAAATTGATCTAGATGTTATAGTTGAGATCGATCTCTACAAAATGGAACCATGGGACATACAAGATAGATGCAAGCTAGGATATGAGGAACAAAATGAGTGGTACTTTTTCAGCCACAAAGACAAGAAGTATCCAACAGGAACAAGAACAAACAGAGCAACTGCTGCTGGATTCTGGAAAGCAACTGGGAGAGACAAGGCTGTTATGTCCAAGAACAGGATCATTGGTATGAGGAAGACTTTGGTCTTCTACAAAGGACGTGCCCCTAATGGCCGCAAAACTGATTGGATTATGCACGAATATCGCCATCAAACCTCTGAACATGGCCCTCCTCAGGCAAGATGGGTTGTGTGTAGAGCATTTCGAAAACCAAGTCCAAGTCATCAAAGGCAATTAGGGTTTGATCCATGGTGTAGTAATCATCATCATCAAGCACATTATTTCAGAGATCAAAGTAGCTATGGTGGAAGGCCCTTATCAATCACAGATCTTCTAACTTCAGAAACTCATCATCATCATCTTCTGAGTCACCCTACCGAAGGTACAAATTTTAGTCATTCCTTCGGTTCAGATCATCACCATCATCAAGAACAACAAGAGTTTGTAATATCAAATAATCATCAACAACTCATTGAGCTTCCACAGCTAGATAGCCCAACAACAACAAGTTTTGCAGTCAAAGAATCATCATCCATTAATAATAACAATGAAGAGTATTGCAGTGATGACAGGAACAACAACAACAACAACATTGATTGGAAAAGCTTGGATAACCTGTTTGCTGATACTTCTAATTACTTCTCATCAAATCCAAACATGTCCCAATTCATGACCATCAATCATCATCTAGGTTGTTTCCCTGGTTCATAA

>AdNAC68--Aradu.W3GLH

CCACATTCCAACCCCCCTCTCCTCTTTTGCTCTTTCATCTATTCTCTCCTTAGCCTCTCCCACACTTTCTACCTTTGGATCTTCTTCTTCCCCAATTGAGTTATACTTTGTACTTTTGTTTATAAGAGGAAGAAGGAGGTCCAAAGCTAATTAGGTCATTCGGATTCAAATTATATATAGAGAGAACATATGGCACCAGTTTCATTACCCCCAGGTTTCAGGTTCCATCCAACTGATGAAGAACTTGTTGCTTATTACCTCAAAAGGAAGATCAATGGCCGTAAGATTGAGTTGGAGATCATTGCTGAAGTTGATCTCTACAAGTGTGAACCATGGGACTTGCCAGGGAAGTCATTGTTACCGGGGAAGGATTTGGAGTGGTATTTCTTTAGTCCTCGAGATAGGAAGTATCCAAATGGGTCAAGAACGAACCGTGCAACAAAATCTGGGTATTGGAAGGCGACAGGGAAGGACAGAAAAGTAAATTCACAATGTCGTGCTGTGGGTATGAAGAAAACCCTAGTTTACTATCGTGGAAGGGCACCTCATGGCTCTCGCACTGATTGGGTTATGCATGAATATCGTCTTGATGATAGAGAATGTGAAAATGCTTCTTCTGGCTTGCAGGATGCATATGCACTTTGTCGTGTGTTCAAGAAGAGTGCAGTTATAACCCCTAAAGTTGATGAGGAACATCATCATCACCATCACTATGTTAATGCTAATAATCACAATAATAGCAGCCATGCTTTGCCAATTACAAGTGATCAATCGTCAAGTATGGAGTTATATTCTGAAGGAAGGGGTGAAGATTTGGATAATAGCTCTAATTATTTGGTTCCCATTGATACTACTTCCACACTACCCCTCAACAACATGGTGATGAACAATAATAATAGTGATGCTTCTTTCAATAGTAGGGATAATAATGGAAAATGGTCACAATTTATTTCAGAAGATCCATTGTTCAGCTTTCCAACTTCCTCCTCATCATTTGCTAATAGTTATGGATCTATAACATACCCTCCATCCAAGGTGGATATAGCACTAGAGTGTGCAAGGATGCAACATAGGTTCACCATGCCTCCATTGGAGGTAGAGGACTTCCCTCATGTTGGAACCTCGGAGCTGAAAATGACAGAATTAGCCTCGGGTGCCGGATCCACCGTGCACGGAACCCGAAACGAAACCGATATCTTGCAGGAAATTCTTTCGGTTGCTCATGCTTCCCAGGAGTTGATAAACCACTCCAGCTACTCATCATCATGGGGTGGTGATGGTGGTGGCAACCATGAAAATTGTGCAACTCATGGAGATGATTTCACTTTCATGGTTGGTAGCACTAACTACAATAATAATAATTTGAATGACATTAACTCCATGAGATATGTTGATAGAAATTGGGAAGATCCAAACACTTCAAGATCCATTGATATTGGATATTTGGATGAAGAATTTAAGGGAGAGAGGATGGTAGAGAATTTAAGATGGGTTGGAATGTCTACAAAAGATTTAGAAAAGAACTTCACGGAAGAGCAAAAGATTGTTCCAATAGAGGATATATCAAGCTTCCAGACAAATAATAAAGAAGAAAATGAGGTGCAAGAATCTGAGCAACACCATAGCAACAAGGAACTATTGATCAATGATTTCTCATTAGGGTTCAACCCTAATAACAACAACAGCGAGAACTTTCTAGATGATGATCATAACAACATGGATAATGATGATTACTCAAGTTCTCCAAGCTTTGAAGTCATTGAGGAAATAAGGGTCAGCCATGGATCAATGTTTGTTTCAACTCGCCGCGTCGCCGACACATTCTTCCACCAAATAGTTCCTTCACAAACCGTCCAGGTTCACCTCCTCAATCCAGTGATAACAAGCAATGAAGAAGAGACATTGATGATGATAATGGAGAGGAATCAAGGGTATTTCGGGGATTTTCTTTTCAAGACAATAGCAACTGCGTTTGTGCTCATCTTTGAACTTCTATTCATGCATTGTGATTATTTGAAGGAAGAAGTGGAATTGGTGAAGAGAAAGAGATCATCACAATCATCATCTAAGATCATGAAATGGAACAACAATAATAATAAGGTTTGGTTTGTTGGTTTCAAGAGTAGTGAGAAGGGCTTTGGTGCAATTTTGAAGAAAATAGGGATTTTTCTCACAATATCTTTGGCTCTTTGTACCATGTGGGCTAACCATGTTATAGTTAACCCTTGA

>AdNAC69--Aradu.WIT0W

CTCTCTTAACCATTATTGCTCTAATGGCTCCAATGAGTCTCCCACCTGGTTTTAGGTTTCACCCCACAGATGAAGAGCTCGTTGCTTACTACTTAGAAAGGAAGATAACAGGTCGCTCTATAGAGCTTGACGTAATAGCTGAAGTTGATTTATACAAATGTGAACCATGGGATTTGCCAGATAAGTCATTTCTACCAAGCAAGGATATGGAGTGGTATTTCTACAGTCCAAGGGATAGGAAGTATCCAAATGGATCAAGAACGAACAGGGCAACAAGAGGCGGGTACTGGAAAGCGACTGGAAAGGACAGGGCAGTGCAGTCTCAGAAGAAGGCAGTTGGTATGAAGAAGACTTTGGTGTATTACAAAGGAAGAGCTCCACATGGAATTAGAACCAACTGGGTCATGCATGAGTACCGCTTGATTGAATCCCTCCCTGGAACTCCTCACTCCTCTTTCAAGGATTCCTTTTCATTGTGTCGGATTTTCAAAAAGACAATTCAAGTTCAAGACAAATCTAAAGAAGAGAAAGAACATCAAGCATTACTAGAGGAAGATCACTCAAGTGGCATTGAGATTTCAAGAGAAATGGAAGCCATGAATGATAATAATAACAATAATATAACTCTAAATAGTAATGAACAATATCCTAATAATAATAATAATAAACTCCCTAATTGTGATGCTTCATCTTCTGATCTCACTCAAGGAACATGTACACCCACAGAAACCGGTATAGCAGATGATTTTCATGCCCAATTTGCTTGTGATGAAGCAAATAGTGCCGCTAATTCTTACTCAATGGGAATAGCATACCCCTCAAACGACATAGAGATGTCCATGTATGGAAGCATGCATAATTATCAATTCCCACAAACGCCTTTGGTGATGGAAGATTTTCCACAAATAGATTTTGCTGAGACAAAGTCATTGAAGCCAGAGGTGACTGAAGACTGCATGTTCTATGATAGATACGGTAGGGATTGTATGAATGGAACACTAGAAGAAATCATCTCATTGTGTTCCTCTCAAGACAACTCTGTGGCTTTGCCCATGCTAGAATGA

>AdNAC70--Aradu.WS3DN

TCATGAGAAGTGCACTACACAAAATTGAATTAAAGAGGTGGAGAAGATGGAAAACATGCCACCAGGTTATCGTTTCTACCCTACGGAAGAGGAGCTGATTTCATTCTATCTACGGAACAAGCTTGAAGGAGTGAGGGAGGACATGAATCGGGTTATTCCAGTTCTTGATATATATGAATATAGTCCAAGCGAACTCCCACAAATATCGGGAGAGGCGAGTGTTAGAGACAGCGAGCAGTGGTTCTTTTTCATTCCGCGGCAAGAAAGCGAAGCGCGTGGAGGGAGGCCGAAGAGGCTCACAACAACTGGAGCTCCTAACGGAACTAAAACCGATTGGAAGATGAATGAGTATAAAGCCATTGACACTCATCACCCTTCTTCTTCCTCCAACAACAGGGCGGTTCCTATGAAAGCAAAATGTTTGAGGGCATTTGATAGAAGACCACCTCCAAGGAGGGACACGTATCCTCCTAGCCAGAACAACGGTTCATCATCTTTTGATCATCATCATCAACATAATCAAACGGTGGAGAAATCTTCAGGTGCAGGTAGCTCACCGGAGAGTTCGTGCTCCGAAGACCACGGACAGTGTTCTCATCGGACGGAGGATGTGGAAAACGCTAACGAGCCATTTCTCGATTGGGAGCAAATCGATTGGTTCTTAGGATCTTCACTGCCGGAACCATGA

>AdNAC71--Aradu.XE8WZ

ATGAGTACACAAAAGTATACACAACAAGCCAAGAGTGGCTTCTTCAATGTTGCCGGTGCCAATAGCATATTTAACCAGGATTTTTCTGTTATGAAAATAAGTAGTAAGTCACATCTGAAAAGTGGGGATTTGGAGTGGTACTTCTTCTGTGCAAGAGGAAAAAATTATGGAATTGGGTCGAAGACGAACCGTGCTATAAAGAATGGGTACTGGAAAGCTACTGGTATGGACAAAGCTATTGTTCAGCATGACAAGCAAACAGTGGGGATGATGAAAACCCTTGTTTTCCACACTGGGAAACCGCCTCATGGGACCCGAACTGATTGGGTTATGCATGAGTATAGGCTTCAAGATAAAGACCTTACTGATAAAGGAATTGCTCAGGACTCTTATGTTATCTGTAAGGTGTTCCGAAAGGAGGGTCATGGCCCACGGAGCGGTGCACAATATAGGAAACCATTTAATGAAGAAGATTGGGATGATGATGATGACGATGATGATGATCATCACGTTGTTGAGGAGGGGGGAACCCCTTCAACTGCTTTGGTTGCGCCGGTTTCCATTCAGTCTATGACATTGGATGGCTCTTCTTACATGAAGGCAACCTCTGTTTCATGTGAGTCGGGACCTGTGGCTACTTCTCCTGTCCCATCAACTCCTTCTTCAGATGCAAGCATTCACACGGTTAATAATTCAACAGTGACTGATCTCTCCAAAGACGAAAAAACAGTACCTAAGGAAAATATTGCTGCCGGTGACCTCTTGAGTAAGTTTTTTGAGGGTTTGGAAGACCTCGAGTCTGAATACACTCCAAATGGAATGGGCTTGGATGGCTTCGCCCCAAATGGAATAAACTATGATGACTTGGGGCACTTGGATTTGATTGATTGCAATTTCCTCTAGTTCTACTTCTAGCCGTCTCACAATCTAAAAGTTGTAGATTTTTGTTTAGATTTAACAGTTGCTTTTTCATTTTGTAAATGTCTAATTTGTTGTTTATATTACATGTTTTTT

>AdNAC72--Aradu.XJF09

GGTTTCTCCATTTTTGGCAAGTTGTTTCTTGAATTGCAATAATGGCTAGAAGCTGGGTTATTGACATTGGAGGACTTGCAAAGAAAGTGAAGAATAATACATTGCCATTAGCTGATCAAATCAATGACTGTGGAGCATATTGTGAATGTCCAATATGTCATTATCACATTGATAACATTGATGTTTCTCCTGAGTGGCCAGGCTTTCCGGCCGGCGTGAAGTTTGATCCTTCCGACATAGAACTGTTAGAACATTTGGCAGCAAAATGTTGTGTTGGGAACAAAGTGCCTCATGCCTTTATCCAAGATTTCATCCCAACACTAGAAGGAGACCAAGGAATATGCTACACACATCCAGAAAATCTTCCAGGTGCTAAGAAAGATGGGGCCAGTGTTCATTTCTTTCACAAAACAACTAATGCATATGCTACCGGACAACGAAAGCGTCGAAAGATTAATCATCAACTAGGCCTAAGTGAGGAGCATGTTCGCTGGCATAAGACCGGTAAGACCAAAGCTGTAACAGAAAATGGAGTACACAAGGGCTTCAAGAAGATCATGGTTCTTTACGTAAGGCCTAAGAGAGGAGCAAAGCCGAATAAATCGAAATGGGTGATGCATCAGTACCATCTAGGGAGTGAAGAAGGTGAGAAGGAAGGTGAATATGTGGTTTCAAAGATTTTCTATCAGCAGCAGAAGAAAACTAAGAAGAATAAGCTGAATCCATTGGTGGCTGAAGATTCTGTCATGGCATTACAAGCAAGTCCAAGAACACCGAATCCAAATCCACCAAAACGGCCTCGGACAGGAAAATCTGTTGATTTTGAAGAAACTGACCTTATGCTATTCACTCAGGGTGGAAAGCCTACTATTCATGGAGAATCACTTGAACCACCACCATCTGAAGTTCATGGTGATGAAAATAATGGAGGCTTTAACAACACTGCATTGTTATCTGTTGAAACACAACCTGTCAACTCTGACTTTATTGGATTGGATGATATCTTACTATGCAAGGAACAGACATTAGATTCTTCTTCTGCTCACCTAAATGATTCGGGTTTGAAGTCCAACAATCTGAAAGGCTTTGCTTGCAATGCAAATGGAAATGCAGGTGAATTGTTTGGGGATGTGAATGATTGTTATGGAATTTCAGTGCTGGATAACCTGGGACTGGACAGTCCTCCCGATTTTGATCTTTCTGTAAGCACTCGATCACCCTTGTCGTCGTAG

>AdNAC73--Aradu.XQ4VP

ATGTCACCAGTTGGATTACCACCTGGGTTTAGGTTTCATCCAACAGATGAAGAGCTTGTTAACTATTATCTAAAGAGGAAGATCAATGGCCAAGAAATTGAACTTGATATCATTCCTGAGATGAATGATTGTATATCTAAAAGTCGAACATTTTGCTTGTTTCTAGAAAAATCATTTTTGCCGAGTAGAGATCCAGAGTGGTATTTCTTTGGACCAAGGGACAGAAAATACCCTAACGGATTCAGAACAAATAGAGCAACACGAGCAGGGTACTGGAAATCAACAGGTAAAGACAGGAGAGTTTCAAGCCAAAGCAGACCAATTGGTATGAAGAAGACTTTGGTTTATTATAGAGGAAGGGCTCCTCAGGGAATCAGAACTGATTGGGTTATGCACGAATATCGTCTAGATGACAAGGACTCTGAAGACACCACCGGTTTACAGAAAAATGGAATATGTACGGATGTTGAAGAGCAAGGCATTGTAGTAACATGTCTTCACTAA

>AdNAC74--Aradu.Y1DM8

GAGGAAGTGCCTTGCATACCACGATTCTCATCTCTCTCATCTCTCACAGCGCGTCACACCATCCTCCTTTCTTTCCACTTTCTTAAAACCTCTCCAATTCTTCTTCTTCTTCGCTTTCCGATCCTAATATAACCCCAATTTCTCTATTCCACTATATATATCGTTGAATTCAAAAAGATATTTAGTTTGATTGTGTTAGATGGAAGAGCCTGTCGTAGTTAACAAAGGCGAGGAGCCGCTGGATTTGCCACCAGGTTTCAGATTCCACCCAACAGACGAAGAAATCATCACTTATTACCTCACCGAGAAGGTCATGAACAGCAGCTTCAGTGCAACTGCCATAGGTGAAGCCGATTTGAACAAATCCGAACCCTGGGATTTACCAAAGAAAGCAAAGATGGGAGAGAAGGAGTGGTACTTCTTTTGTCAGAAGGACAGGAAATACCCGACGGGGATGAGGACCAATAGAGCAACGGATTCCGGTTACTGGAAGGCAACCGGAAAAGACAAAGAGATTTTCAAAGGGAAAGGGAATCTCGTTGGGATGAAGAAAACGCTTGTTTTCTACCGAGGTAGAGCTCCCAAGGGTGAAAAGACCAATTGGGTCATGCATGAGTTCAGATTGGAAGGCAAATTTGCCAATTATAACCTCCCCAAGGCTGCAAAGGATGAATGGGTTGTGTCGAGGGTTTTTCACAAGAACACAGATGTAAAGAAGACTACTACCCCATCATCATCATCATCAATAATTCCTGGCCTTTTGAGGATCAACTCAATAGGCGATGATCTTCTAGATTGTTCCACACTCCCACCTCTCATGGACCCTCACCCTACTCCTCTCGATACAACAAAATCCGACGGCTATTATTTCCCCTCCTTCTCATCATCACATCAGATTCTCAATATCAAGCCCGAAGAACACAACACAAGCCACCAAATTCCCATCACCAACTACCAGATTCCAAATTTCAATACCACACTCTCTTCTTCATCATCTCATCAAATCAGACTCCAAAATCATCTCAACTTGTTCTCATCATCATCATCAAATAACAATTACCATAATAGCTCGTGGCCAAGCTATTATGATGAGGTCCACCACCATCATCAAGATGATATTCTACTAAGAGCAATCGCCTCAAAGAACTATAGCAACGGAGGAGGAGGAGGCGGCGGCGAGTGCAAGGTGGAGCAATTCTCCTCCGGCAACCAGTCAGTGGTGAGCGTTTCGCAAGACACGGGGCTGAGCAACGACAGAACCACCAATGACACGTCATCGGTGGTTTCGAAGCAGCAGCATAATAATAAAACATTGTACGAGGATCTTGAAGGTCCTTCTTCATCAGTTGCACCTCTTTCAGATTTGGAATGCTTGTGGGATACCTACTGA

>AdNAC75--Aradu.Y9JNS

ATGGAAGAGCTAGCGTGTGAGCTGAGTGATCATGAAAAGAGAAACGCTCAAGGTTTGCCACCCGGTTTCAGGTTTCACCCAACTGATCAAGAACTCATTACCTTCTATTTGGCTTCCAAGGTCTTCAATACTACTACTACTACTACTCATGTCAACTTTGTGGAGGTTGATCTCAATCGATGCGAGCCATGGGAACTTCCAGAAGTGGCAAAGATGGGGGAGAGAGAGTGGTATCTGTACAGTGTGAGAGACAGAAAATACCCAACGGGCCTCAGAACTAACAGAGCAACCGCTGCTGGGTACTGGAAGGCTACCGGCAAGGACAAGCAAGTATACGGCGGCGGTGGCCTTGTTGGGATGAAGAAGACGTTGGTGTTCTACAAAGGGAGGGCCCCCCGCGGTCAGAAGACTAAATGGGTAATGCATGAGTTCCGGTTGGACCCTCACAGCTCTCCTTCCCTCTCTAAGGATGAGTGGGTAATATGCAGAATATTTCATAAAAGTGGGGAAAAGAGAACTCCTACTACTACTCCTGCTCCTCTGCTACTTCATCATCAACAACACGATCCATCATCACTGTTCAATGACCATATCTCCCACTCTCATAATCATAATCAAAACCTCCTCTCGCCATTGCTTCATCCTTTCCCAATCCCTGAAGAAACCACTAAAACCAAATCATCAACAATTAACAGCAACCATTACCCTCCACCACCACCTTCCTCCCAACACTTGCTTAAGCTCAATAAGTCTACTAAATTAACAAAAACAGTGCCTCCTTCTCCATCATTCTTCCAATACCAACAGCTTCTAGAAGATGATCCCAACTTATTGCATTGGATGGACAGTGGTAATAATAATAATAATAACTGCAAGGCTAATAATACTGCTAGTAGTGTTGAGATAATGGATGCTGCTGCTGCTGGCTTGATAGCATTCTCATCAGGAGGACCTTCACCTACTCCTACTAATAATAATAATAACAATTCTGAAATAATAAGGGACATGATGATGATGTCTTCTTCTTCGGCTTCTATGCTGCACATACTCGACGATGCTCCTCTTGGAATTCAATCTTGGCCTCATCATCATCATCATCACCTTCTGTAA

>AdNAC76--Aradu.YFQ3P

TTTAATTAACCCTCCCTATATAGACCTCAGCTTCTCCAAGCCTCTCTCTCACGTTCCAACTTCTAAGTTCTAAGTCGCAAACAAGAAGAAAAAAAGAAAGGAAGAGGCCGCAATGAAGAGTGAATTGGAATTACCACCTGGATTCAGGTTCCACCCCACTGATGAGGAGCTTGTGAATCACTACTTGTGCAAGAAATGTGCTTCACAGTCAATTGCTGTTCCTATCATCAAGGAGATCGATTTGTACAAGTTTGATCCATGGCACCTTCCAGAGATGGCTCTATACGGCGAGAAAGAGTGGTATTTCTTCTCTCCCAGGGACCGCAAATATCCGAACGGATCACGCCCAAACCGGGCTGCGGGTACAGGGTACTGGAAGGCCACAGGTGCCGATAAGCCCATTGGAAAGCCCAAGGCCCTGGCCATCAAGAAGGCACTGGTGTTTTACGCTGGCAAAGCCCCCAAAGGAGTGAAAACCAATTGGATCATGCATGAATATAGACTCGCTAATGTTGACAGATCCGCAGGCAACAAGAAAAATAACTTAAGGCTTGATGATTGGGTGCTATGCCGAATTTACAACAAGAAAGGAAAGATTGAGAAATACAACCATCTTGGGGCGGCGGATCACAAATCAGCATCTTCGTCGGAGGAGAATGAGAGGAAGCCGGAGGTGAAGGAGCGATTGCATATGGATACGTCGTCGGATTCGGTGGTATCGGCGGATGTGACGTGGGAGAGTAGGGAGGTGCAGAGCGAGCCAAAGTGGAATGACCTGCTTGACCAAGTCTTTGATTTCCAGTTAGGCAGTTTCGTTGATTTCTCATCGGCTGGAGATGACCCTTTTGCCCCCCAGCTCTCTCCTTGGCATCAGGACACGTTCATCACATTTTAATTCATTCTTTCCAACACAACAAGAAAAACTAAGATTGTTTAGTTTAGCCACCATGACTCATTTGGATTTAACTCGGGAACCAAGGACGATGAGATCATATATGTAAAAATAAATAAGAAAAATAATCTCACCAATGCCACCTAGTTTGTTTTCATGTTACCATTGTTGGAGTGTTAAGCATCTACTCCTGTGGAAAATTCTTCCTACCATTTTTTAAAATATATGTTATTATTTAATTCAACATATTCAAGGAATGATTTCG

>AdNAC77--Aradu.YIQ80

TGATTGGAGAAGAAGATTAAAACAAAATGGGAATTCAAGAGAAAGACCCTCTCTCGCAATTGAGTTTACCGCCGGGTTTCCGATTTTATCCGACGGACGAGGAGCTTCTCGTTCAGTATCTGTGCCGCAAGGTTGCTGGCCACCATTTCTCCCTGGAAATCATTGGCGAAATTGATTTGTATAAGTTCGACCCTTGGGTTCTTCCAAGTAAGGCAATTTTTGGCGAGAAAGAATGGTACTTCTTTAGTCCGAGGGATAGGAAGTATCCGAATGGATCGCGACCCAATCGAGTAGCCGGGTCGGGTTACTGGAAAGCTACCGGAACCGATAAGACTATCACGACCGAAGGAAGGAAAGTTGGTATCAAGAAAGCTCTGGTTTTCTACATTGGTAAGGCACCCAAAGGCACCAAAACAAACTGGATCATGCACGAGTATCGCCTCCTAGACTCTACCCGCAAGAACGGGAGCACCAAGCTTGACGATTGGGTTCTGTGCCGGATATACAAGAAGAATTCAAGCGCACAGCAGAAGGTACCAAACGGCGTCGTTTCGAGTAGCGAGCAATATGCCACGCAATACAGCAACGGATCTTCTTCAAACTCCTCTTCCTCCCACCTCGACGAGGTGCTCGAGTCCCTGCCAGAGATCGACGACCGTTGCTTCGCCTTGCCACGTGTCAACTCCTTAAGAGCGCTGCAGCAGCAGCGCCATCACCAAGAAGACACCAAGGTCGGCCTACTCCAACAGCAACAGCAACAGGGTCTCGTAGCCGGCACCGGTAGTTTCTTGGACTGGGCTTCCGGGCCGGGGATTCTGAACGATTTGGGCCAGGCCCAACAGGGGATTGTTAACTACGGAAATGACCTCTTTGTCCCTTCGGTGTGCCACGTGGATTCCAATTTGGTGCCAGCAAAGATTGAAGAGGAGGTTCAGAGCGGCGTGAAGACTCAATCCGGATTCTTTCAGCAGGGACCGAACCCCAATGACTTTACACAAGCATTCTCAAACCAGCTAGATCCTTACGGGTTTAGTAGGTACTCGGTTCAACCGGTGGGGTTCGGGTTCAGGCAATGAACCAGGGTGAGGAACTAGATGTGAAATAACTAAAAGAAAGTGTATTGAATTTTTGACTATTTGTTGAGGTGCAATTGGGGGGTGTAAATAGGGATTCTTTTGGAATATTCCAAGAAAGAAATGTCTTGCATTTAGAAAAAGGGTTGGGTTGTAATTTTCTTTCTTGGTGGTCCTCTTTTTCGGCTGGAGATAGAGTGGAGAAAAGAAGAATTCAATGCATCGAGAGGTGGAAAAAAGGGGGAAAAATTGTGATTGCACTAAATTACTAGTCTTTGGAATTTTAATTAGTTTGAAGATGTACACAATTTTTGGCTGCACCGGCCGTGGTGTGAGCCACATGAATCACGTTTTGTACTTTTGGAAGATTTAGCATCAATCGATATACAATTGTTTACAATTCCAATGTTAGGTTGCTTTTCAAATGTTTGATGTGATCATTTTAGCTAATAACCACAACATTGGTGGCCACAAACTCATTCGTGTTCATGGGATAGGACTATGTTATAAGAGTATTTTCAATGCGTAATTTTAGTTTAGTTTGCTTTTGAGTTTTACAAAATGGTATATTATGTAAAAAATTAATTTGCGATTCAATGTGTGATTATCTATAAAATATAAAATATGAATATTTTGTTAAATTATCGTGTTTGCATG

>AdNAC78--Aradu.YXW0Z

AACTTGTGGAATTGAAAGGGCCGGGTCTGGAAAAATCAAAAGAAATGACATGGTGCAATAGATCATCAGTTGTGGAGAGGGGAATCGAAATAATCAACCACCCTAATCTCAATATTATTGCAATCCCTAGAAATAGTAATGACAATAATAATAGTGTTATTAGTGTTACTCATCACGCAAATACTCCTCCTAAACCAACCGAAATCCGAGCCGTTACTTGCCCCTCTTGTGGTCATAACATTCAAATACAACAAGATCAGGGTGGGGGAATTCAAGACTTGCCAGGATTGCCAGCTGGAGTGAAATTTGATCCGAATGACCAAGAGATACTGGAGCATTTGGATGCAAAAGTGCAGTCTGATGTGAGAAAGCTTCATCCTCTAATTGATGAGTTCATACCAACTCTTGAGGGCGAGAATGGAATTTGCTATACTCACCCAGAGAAGCTTCCAGGAGTAAGCAAAGATGGACAAGTGCGTCACTTCTTTCACAGGCCTTCAAAAGCATACACAACGGGAACAAGGAAGAGAAGAAAGGTTCACACCGACCAAGAAGGAAGCGAGACTAGGTGGCACAAAACTGGCAAAACCAGACCCATCTCTGTGGCTGGTTCCGTTAAGGGTTTCAAGAAGATTCTTGTTCTCTACACCAACTATGGCAGGCAGAAGAAGCCCGAGAAGACCAACTGGGTCATGCATCAGTACCATCTCGGCTCCAACGAAGAAGAAAAAGACGGCGAGCTCGTCGTTTCTAAGGTTTTCTATCAAACACAGCCTCGACAATGTGCAAATAAGGATCCTTATGATGAAAGATTATTGATGACTTCACAAATCAACAGTGTCAATGACATTAGCATGCACGCACTACCCAAGAACAATGCAGGTTTTGTGGATTATTATAACCCCGGTTTCATGAATATGAATTATGAACAGATGAACGAGACTACCTCACCGCAACTGATTCCGAATATGGTGGTGCAAGGTGACAGCTCTTCTTTCATTCGGTTAGCCATGGATGCAAACAAACCCAGGTTGGACAGAAAGTAGTATGACAATGACTGCATGTATGCATGTTGATGTTTTTTTTAATGCC

>AdNAC79--Aradu.Z4K97

GTGTTGAATACATGCGTCATAAGCATTTCTTTTTCTTTTTCTTTTTTAATGTTATATTTACAAAATTAAAGGAAAATTACACGTGTAGAAGTACTTGGAAGTTTCAGAGAAAGAGACAAATTCTCAAACATAAGAACAGGAACATAAATATATGATTTTGAATCTCCGTCTCTCTACTTGCATCACCAGCCACTTTCTCTTTCTCTCTCTATGGCCGGATCATCGTGGTTGGTAGACAAAAGTAGAATTGCAACCAAAATAAAGAGTGCATCTGGAGCAAGTGGGAAAGTTTTATGGAAAAGCAATCCTACCAGAACTTGTCCGAATTGTCAACATGTTATTGATAACAGTGATGTTGCGTTCACGATCACTTGTTCGCCATCACCCTCGTCACATTTGTGTTTTCATTATTGTGCTTGTGTTCCATTCATTTACTCTAGTTTTCATGATTTCTTTGATCATGATTCTGTTACTCTTGCTGGTGCAACCAATTTCATAGGAAGTGTTGTATTGTGGATTCTAATATTTCTCTACGAAGCTAGACACATGAAAATCTTTGAAAGGGCTACAGTGACTGTTCAAGTGGCACAAGAGTGGCCTGGATTACCAAAAGGTGTGAAATTTGATCCATCTGATCAAGAAATAATATCGCACTTGCTTGCAAAAGTTGGTGCAGCAGGTTCAGAGCCTCACCCTTTCATTGATGAATTTATTGCTACTCTTGAAGTGGATGATGGAATTTGTTATACACATCCTAAACATTTACCAGGTGTCAAGCAAGATGGTAGTGCTACACACTTTTTCCACAGATCAATCAAGGCTTATAATACCGGCAATCGAAAGCGTCGGAAAATAAATGACCAGGACTCTGGCGATGTCCGTTGGCACAAGACTGGAAAAACTAAACCTGTCATCTCGGACGGGGTTCAGAGAGGCTGTAAAAAGATTATGGTTCTATATATGACTTCAGTTAGAGGAGTAAAAGCTGAGAAAACTAACTGGGTTATGCATCAATATCACCTCGGAACAGACGAAGATGAAAAGGAAGGAGAGTATGTTATCTCTAAAGTGTTTTACCAGCAACAAGTTAAGTTTGCTGAAAAAGATGATCATGATGTTCCTGGAACCAATGAAGCAACTGTTGTGAAAGATGATCCAGTCACTTCGGAACCTCCTCATAGTGAAAAGCAATGTTCAGATCTCGACATAGGAGAAAAATCACATCAGATTCCTCAGGGTCCTCAGACAGATTGTGTAGAAGACATTCAAGCCGAGTGTGAAGAGATTGTGAAAACTGATGTAGCCATGGCAGATGCTCAAAATAATGAAGGAATGGATAATGTAGAAAATAATGCTGATGGAGAACAAAAATGGTGGGACAGTGAGTCACAGAATTTGTTAGATTCACAACAACTTGTTGAAGCATTGGCCTTGTGTGATGATCTCCTCCATAGCCAGTGTTCCAATAAGGATGATGAAAATGTAGAACACAAGGAGCACTTGAGTCTTTCCATCTATGCTCATCTAGGACCAGAGCATCTGAAGAAAGATCTTGAAGAGTGCCAAAACCTTAATCTTGATCCTGCAAACGTAGAGCTCGAGACACCACCTTCAGAGTTTCGACTAAGTCAGCTGGAATTTGGTTCACAGGATAGCTTTGTTTCCTTGAGCGGCGGCAAGGCAGTCGACTAATCCGGCCCTTCTTCGGCTAATATGGGAAACAAACACATTTCTAACTACTTTTGTTGTAACATTTGATCAACAGTGGTGTAATTAATACTGTCTTTTGGTGTAGTGAAAGCCATGGTATGTTCCTTACAAGTTCTCTAGAAAGTTGCATGTGGACCTGACTTGTCCAAAAATCACTACATGTTTTTATTTCATTTTGAAAGTAACATATGGAGATGAACATGTACATTTATAATTTTATATCCCTATCTTGAAATGTGCTGTCTTGTCTCCCATAAAAATCTATTGTATGTATATTTGGTGGCGACAGATTCATGAATGC

>AdNAC80--Aradu.Z5H58

AGAAATAATAGAAAGCCCTTACAAAAATAAAAACAGAGGAAGGAAAAGAGTTCAAGAAACCAAAAACGTTTTAGCATAATGGGAGTTCCGGAGAAGGATCCTCTTTCTCAATTGAGCTTACCTCCTGGTTTTAGATTTTACCCCACAGATGAGGAGCTTCTTGTTCAGTACCTATGTCGCAAGGTTGCTGGCAACCATTTCTCACTTCCTATCATCGCGGAAATCGATTTGTATAAATTCGACCCTTGGATCCTCCCAGGTAAAGCAATATTTGGGGAGAAAGAATGGTACTTTTTCAGCCCCAGGGATAGAAAGTATCCGAACGGTTCGCGACCGAACAGGGTTGCTGGCTCTGGGTACTGGAAAGCCACAGGAACAGATAAAGTAATCACTACCGAAGGCAGAAAAGTTGGAATCAAGAAAGCACTTGTTTTCTACATTGGCAAAGCACCCAAAGGCACCAAAACAAACTGGATCATGCACGAGTACCGTCTCCTCAACGGTTCTCAAAAGAGCCTCGGCAGCACCAAGCTAGATGATTGGGTTTTGTGTCGGATATACAAGAAGAACTTGAGCTCATCGCAAAAAGTCAATATGCCAAGCTTTACGAGCAAAGAATGGAGCAATGGATCGTCTCCTTCTTCATCGTCTCACATCGACGACATGCTCGAATTGCCGGAGATCGACGACCGGTGCTTCGCCTTACCGCGGGTTAACTCGCTGCAGCACGAGGAAAAGCTCACCCTTGGCGCCACAGGCAATAATTTCCCGGACTGGGTCAACTCGGGGGGTCTCGACTCGGTCCCTGAGTTCGGGAGCCAAACTCAGGGGATGACAAGTTACGATGGAAATGACCTATATGTCCCCTCCGCGTCACAGTTCTGCCACGTCAACACAATGGTTGTGCCGGGTAACCCGACGGAGGAGGAAGTCCAGAGCGGCATCAGGACCCAGCGGATTGATGAGAATTTCGGGTTATTTCAACAGAATTCGAATGTATTCACCCACCGGTATTTGTCGAGTTCGGGTGACTCATTCGGATTCGGATACCCGAATCAGCAATTTGGATTCGGATTCAGAGAATGA

>AdNAC81--Aradu.Z9Y3J

CGGTTCTATGCACCTAAAAACCCAGTATTTAAGGATTCTGTTTCTCATAACATAGATCTTTGTTTTGAGTGTCTCTCAATTGCATTTTTTTTCCCATCAAATTCCTCAAAAAAGAAAAATTAAACCCTCCACACTAAAAAAGAAGAACTGCTCAATTTATTCACTCTCTTAACTTGTCTTCACACCCATGATATATGCATCAAAAAGTGTAGCCACCCTAACCCCAAAACGTTCCTTTTTTAACTCCTTGCAACACACAAGTGTAAAATTGTCCACTACCCTTTAAAGGTTGGTTTCTCTGCATTTAGCCCACTTGAATTCCGGCGCCGAATGTTCGATAGCTGCCAAGTTTAACCGACATATTTGTCGTGTTTCGATGGTTTTTATGAACCAAAGGAGCCTAGCATAGAGATGGAATCAATGGAGAATGTTAGAATGCAAAGAGAGAAAGATCAGAAGTTCGAATTGCCGTCCGGCTTTCGATTTCATCCCACCGATGTAGAGCTCATAAATTACTACCTTGTTAAGAAGGTTCTTGATGATAAGCACTTCTGTTCTATAGCAATTGCTGATGCTGATATGAACAAGTCTGAGCCATGGGATTTACCCGGTTTAGCGAAAATGGGCGAAACAGAATGGTATTTTTTTAGTATGAAGGATAGAAAATACCCAACTGGCCAAAGGACTAATAGGGCGACCGAGGCCGGATATTGGAAGGCCACAGGCAAAGACAAGGAGATATCAAAGGAGAATTCAAAGATTGGGATGAAGAAGACCCTTGTTTTCTACAAAGGAAGAGCTCCAAGAGGTGAAAAGACTAATTGGGTCATGCATGAATATAGATTGGAAGGGAACAAATCTGTTTATAATCTGTCACAACCTGAAAGAGGTGAATGGGTTATATGCAGAGTATTTGAGAAGGGCAATAATGGAAAAAGACTGAATATTGCAAAGTTGGAGAGGCTCAACTCTTCGGGAAAGGAACCATTACCATTGCCAAAACCTACTCCTTTGATGCCTCCATTGATGGATTCTTCATCATCGAGAACCACCCCCGGCGAGTTATCTCAGGCGACGTGCTACTCCTCGGATCCAAATCAAGCCGATGTCCAGAACAATTTGCATGATGACATAGTTGAAAGCAGGGAAACTCCTATCTTGAACTTTTCCCCTGCTTCCATCAATGAAGAATTAATTCAGATTCCAAACCAAATTGAGAATCCGGATTATTATACTCTGCCTCAAGAAAACAACGGATCAATTGCAAGGCAGAATCAGAAATCAGAGTTTGATGCTGATATATCATCTTTGATTTACAACAATGACATGTTTTACAGGTTCTTTGGGAACCAAGAACATTCATCTTCAGCTTCCGCAGACATTTGCAACCTATGGAATTACTAGATAAGGTTGAAGGATATTAAATAATATTATTACTCTCTCTTTTTTTAAGACAAATTAAAGTAAACATGTCATTTATATCATACCTTTAAATTCTGAATTGATTACCTGCAAATGCATGAACTTATTATTAGGTTGGTAACTTTAATTGAAAAAAAGGTGG

>AiNAC1--Araip.0550R

GCAATAAACGACCAACTGGGTTGCCTTACATTCCACGTTTCTTTCTTTCTTTGAAATACAAACACAAACACAGAGAGAGTGGTATTTAGGGGGATTCTTTTTCTCTCAGCTTTTTTTACAAATCTCTCTCGATCACTTCCATGATTCTCATCACCTTTTCTTTCCCACTTTCTGCATACTCGATCTCTTCTCCTTCGATTCTCATCAGCAATTCCTAGAAAATTCTTATATTGAAAACCAAGGGTGACCCTTTTCATTTTGGGGGTTCTTACATATCAAATGGAAAACATTTGTTCAGAGGTTGAGATGGATTTGCCACCAGGATTCAGGTTTCACCCAACTGATGAAGAGCTTATAAGTCATTACCTTTACAACAAGGTCATTGACACTAACTTTTCAGCCAGAGCCATTGCTGAGGTGGACTTGAATAGGTCTGAGCCTTGGGATTTGCCATGGAAGGCGAAAATGGGTGAAAAAGAATGGTACTTTTTCTGTGTAAGGGACAGAAAGTACCCAACAGGATTGAGGACAAACAGAGCAACAGAAGCAGGGTATTGGAAGGCCACTGGAAAAGACAAGGAGATATACAGAGGCAAATCACTTGTTGGCATGAAGAAGACCCTTGTCTTCTACAAAGGTAGGGCTCCCAAAGGTGAGAAATCTGATTGGGTCATGCATGAGTTCAGGCTTCATGGTAAATTCAATCCCCACAACCTCCCCAAATCTGCAAAGAACGAGTGGGTGATTTGCAGGGTGTTTCAGAAGTCTTCAGCCGCCAAGAAAATCCATCTTACCGGGATAATGAGGTTGGACTCTTCTGTTTTCTTGCCACCATTGGCAGATTCCTCATCATCACCTTCCAACACTGCTACTACAGCACCTTACGTGCCCTGCTTCTCCAATCCAATCATTCACAACCAAGTTGGGATCTTTGATCCATTTAGCAACACCCCTTTTGGTGCTGATTCATTCTACACTTCTCAAGGGATGCCAATGCAACATGCTCAACCACCAAGTTGCTACACCACTCAGGACCATTCAATTCTCAGAACCTTGCTTCAAAACAATTCTTCAAACCTCAGGAGTGGTTTCAAGCCTGCAGAGAGGGAAATGGCCCATCATCAAACTTCTCTTGTTGATGCCAACAACAACAACAACAACAATGGAATCACTTCTGTTGTTGCCCCACAGGACCTTTCTAGCCTCTGGAATTACCAGGTTCAGATCAAGTAGCTATGGAAAGTGAATTCTGAGGGTACAAGAGAAGCCATTCTGTTGTGATTTATTCTGTCTTTTGGTGTTGTTGCTTGTCTGAATTGATTTTAAGTAAATCATGTATGAATATTTTGTGATGTCAGTAATAATCTTGTGAATTTATACTATTTTGGTCCTTTTGATACTTTGCTATTGTTATGGTTG

>AiNAC2--Araip.0S3JI

ATGAATAATAATAAGATAAGCAACTTGAGCTCCGTGAGTAGCTCCGATCTCATAGATGCCAAGCTTGAAGAGCATCAGTGGTGTGGAGGATCCAAGCAGTGCCCCGGTTGCGGCCACAAGTTTGAATCCAAACCGGATTGGCTAGGTTTACCAGCAGGAGTGAAGTTTGATCCAACAGATCAAGAACTAATAGAGCATCTTGAAGCCAAAGTGGAGTCAAAGAACATGAAATCACACCCTTTGATAGATGAATTCATTCCCACCATTGAAGGTGAAGATGGGATTTGTTACACCCATCCTGAGAAACTTCCAGGAGTAACAAGAGATGGATTAAGTAAACACTTCTTCCATAGGCCTTCAAAGGCATACACAACAGGAACAAGAAAGAGGAGAAAGATTCAAAATGAGTGTGACTTGCAAGGTGGAGAAACAAGGTGGCATAAGACCGGTAAAACAAGACCGGTCATGGTTAACGGAAAACAGAAGGGTTGCAAGAAGATTTTGGTACTCTACACTAACTTCGGCAAGAATCGGAAGCCGGAGAAGACGAATTGGGTGATGCATCAATACCATTTGGGGCAACATGAGGAGGAGAAAGAAGGAGAGCTTGTTGTGTCTAAGATATTCTACCAAACTCAGCCTAGGCAGTGTAATTGGTCGTCCGATCGGAGCGCCACCACCACCATCGCAACGGCCGAAGGGAGTGGAGAGCCATTACAAAATAGTAGAAGGGATAGTGGGAGTGGAAGTTGTTCTTCTAAGGAAATTAACATCGGTCATAGGGATGAGATGTCTGCTGTGGTTGGAGTTAATAATACTCCAATCACGAGCTTCACTCATCCCTTGGACATTCATCATCACCTCAAATCGGATCATTTTAGCTTCATTCCATTTAGGAAAAACTTTGATGAGGTTGGAATAGGAGAGGCTTCAACAGCAAGAGAAGTTCAAGCATCAGGGTCATGTGATGAAGTAGTACATGAACATGAGTCATCCATCACAAACACACAAGAAGCTGAATGGTTGAAATACTCTTCCTATTGGCCAGACCCTGACAACCCGGATCATCATGGGTAG

>AiNAC3--Araip.1N7IP

ATCTATCGCTTATACCTTTTGTCCTCTTCTTTATCTGATCTTCCCTCTTTCTCTCTCTATCAGTGATCACCAATATCATCAAGCACATAAGAACGAATTCAAGACGGTGGCCATGGTAATCATAAGCTAAGGGAGATAGAACGTGTCCCAAAAGAGTACAAATAAATATAAGGGAAGAAAAATTAGAGGGTGGTCTCATTTATTGTGATGGCATGGTGCAATGACACTCATGAGAAAGAGATAATTGCTTCCAATAATAGTACTATTACTCTTAGACCTAAATCCGACCCAAACGTAACTTGCCCCTCATGTGGCCATAACATTCAAATAATCCAAGAGCAGGGTGGAATTCATGAGTTGCCGGGGTTACCAGCTGGAGTGAAGTTTGACCCAAATGACATTGAAATATTGGAGCATTTGGAGGCAAAAGTTATGTCTCATGTGCCCAACCTTCATCCTCTCATTGATGAGTTCATACCAACACTTCAAGACGAGAATGGCATCTGTTATACACACCCAGAGAAGCTACCAGGAGTAAAGAAAGATGGGCAGATCCGGCACTTCTTCCACAGGCCTTCAAAAGCATACACAACAGGAACAAGGAAGAGAAGAAAGGTTCACACCGATGAAGATGGAAGCGAAACAAGGTGGCACAAAACCGGAAAAACAAGACCGGTGGTGGCCGGCGGCGGCCTAGTGAAGGGTTTCAAGAAGATTCTAGTACTATACACCAACTATGGGAGGCAAAAAAAGCCTGAGAAAACTAACTGGGTGATGCATCAATACCATCTTGGAAGCAATGAAGAAGAGAGAGATGGAGAACTAGTAGTTTCAAAAGTGTTCTACCAAACACAACCTAGACAATGCGGCAATTCCATTGTTATAAAGGAAGATAATGATGATGATCTTCCCTATGGAAAGATATTGATGATGAATAACAGTAGTAAGAAGCACAAAAATAATAACGATAGAAATGTTGCTGCTCCTGTTGTGGACTACTACATCAACTATGACCATGTTGATCATCATAATCACAATCATAATAGTCAAAGATGTTCATCACCTACTCAACTTATTCCAAACTTGGTTCTCCAAGGTGATTCCTCTTCTCTTTTTCGCTTTGCTTCATCATCACTCGATGGGAATACCAACAAAACAAGACTTTTTGAGAGAAAGTTGTAGTAATTCGTTCATTATTATTATTTCTTTCACTAAGTGACAATATTTATTCTATACATATATGATATATGGGATTAATATAATTAGAGTGACATGTAATTAAGTTGATTGGCTTTGATGTGAATTAAGGACTCACTCATATAATCAAAGATTGGTTTTTATTATGTTAATTTTAGTGCTTAGGTACATTCAAATATTGTGGTATAAATAGTAGGGTTATTATTGTCCATTCATAACTTTGTTAAATAATGAATTAAAATTTTTCTCTTTCTCTTTTTTGTTTTTCTCCAATAATATATCTTGAA

>AiNAC4--Araip.1Z0SD

CACATAATTAAACCGAGCAGCCTAGCTATTACTACATACATCCGCTTCATAAATTAGCAAAGTCTTAAAGGTGCTATTCTGTGCTATGTGTTTGAAGCCACACCCAACTTTTCCTTTATTATAAATTTTTATTCGTCGCTGCAGAGAGAGAGAGAGAGAGAGAGAGCTCTATGTGGTTTATGCTTAATTTCTATGGTTAAAGCAGCAGCCTGGAGCCACTAGCTAGTTTCTAAATAATCGAATCGTTATATGATGAAGTAGATGATTGGAAGATGTGATCCAGTTCCAATATATATTAATCAAAGTGTTGGATTTGTGAGTTATAGATAGAAGAGAATGATCATGAGTAGTAGCAGTAGTGGATTGTTGCATAGAAAAAGAATTGAAAATAACAAGGAAAGAAGTAGAGTCGATGAATAAGATGGATCTGATAGATGCGAAGCTGCAAGAAGAGCATCAATTGTGTGCATCATCGTTGAAACAGTGCCCCGCTTGTGGACATAAGTTTGAAGGCAGCAGCGGGAAGAAGGCGGAGTGGGAGTGGGTAGGTCTGCCAGCGGGAGTGAAGTTCGATCCAACAGACCAAGAACTGATAGAGCATCTAGAAGCAAAAGTAGAGGCAAAGAGATCGCACCCTTTGATCGATGAGTTCATTCCCACCATTGAAGGAGAAGATGGAATCTGTTACACCCATCCCGAGAAGCTCCCAGGTGTGACGAGGGATGGGTTGAGCAGACACTTCTTTCACAGGCCATCAAGGGCGTACACCACTGGAACACGGAAGAGAAGAAAGATTCTTCAAAACGATGAGGCGGAGGCCGAGAGAGGAGAGACACGGTGGCACAAGACCGGTAAGACAAGGGCCGTTATGCTCAAGGGAAAGCAGAAGGGGTGCAAGAAGATTCTGGTGTTGTACACCAACTTCGGCAAGAACAGGAAGCCCCAGAAGACCAACTGGGTCATGCATCAGTACCACCTCGGACTCCATGAAGAGGAGAAAGACGGGGAGCTCGTCGTCTCTAAGATTTTCTACCAAACTCAGCCGAGGCAATGCAGTTGGTCTTCTTCTTCTTCTTCTTCTTCAATTACTGCTGTTGCTCCCCCTGTCAAAACTAATAATGACACCTGTCCCGTTCTTGGATTCCCTCCTATGGAACATTTCAGCAGCTTCATCCCTCTCAGAAAAACCCTCCATAATGAGGAAGTTGGAATAGGAGGGGAAACTTGCACACCAGCGTCACATATTCCTTCATCAAATCCTGTTGGAGTCTTCCATCACAACACTTCCATCATCCTTGACGACCTTATCTCCGCTAGATTCATGACTCCTCCTCCTCCTCCTCAGTTCCACCAGCAGCATGATAATAAAGTAGTAGGAGGAACCTCTGCTTCTGGTTTAGAGGAACTCATCATGGGCTGCACTTCAACTTCAACCACTCATAATATCACCAAAGAGGCATCAATGTCAAACACAAACCCACAAGAAGCTGAGTGGTTGAAGTACTCTTCTTATTGGGCTGACCCTCAGCCTCAGCCTCAGCCTCATCTTCATGGGTAATAATAACAGACCCCTGGCAAAGGCGTGGCTTTTCGAGTCTCAACGGAAATAACTGCTGCATGTGTGCACTGTAACGGCACATACATCAAGTTGGAAACAAACAACAAAGGAGGGAAGAACAAATTGAATCAAATTCAAGAAAATGAAAATAACCGCAAGAAAATTAAAGCTCGCTGCTGTTCTACCTCCTCATCACACAAAGAAACAGTTTGATAATGCATCAAAATCATCACTATTACCGATATAGTATCTTTTTTTCTCTCATTATTCATATTATTTTATTATTTTAATTAAGATGTTTGTATAATTTCCCTTTTACTGACTTGCTTCTTTTACACATTACATGAAATATCAAATATACCGA

>AiNAC5--Araip.2BL8E

ATGGTGGACAGGGATTCAAGCGAAGCACACATGTCAATAGCAGCTTCTTCGATATTCCCTGGCTTTAGGTTCTGTCCCACTGACGAGGAGTTGATCTCTTATTACCTCAGGAAAAAGCTGGACGGTGATGAGGACAGAAAATCCTTCATTCAATCTGACAACGAGTGGTTTTTCTTCTCGCCACGGGGGAGAAAGTATCCCAATGGTTCACAGAGTAAAAGGGCAACTGAATGTGGATATTGGAAGGCCACAGGGAAAGAACGCGTTGTAAAGTCTGGTCAGAATGTTATTGGTACCAAACGCACTTTGGTATTCCATCTCGGTCGAGCTCCTAAAGGCGAAAGAACTGAATGGATTATGCATGAGTACTGCGTCAATGACAAATCTCAGGATTCATTGGTAATTTGTCGGCTCAAGAGGAATACAGAATTCCGTGCAAGTGATCATTCTAACAGAACTTCACACGATAGTGATTGTGGAGTCTCAGAAGGAGTTACAGTTCAAGGGGACACTTATGTGCCTATTCAAGATAAAGAGACTGGATGCAGCTCCAAGAGGACTAGCAGTAGTAATAGTTCTCCATCTACTACTGGCCAAATTGAATCCAGTCATAGAGTGAATGAAGAAGAGGATTGTTATGCAGAGATCCTAAATGATGATATCATCAAGTTAGATGAATCAACACTCTCACGGCCATCGCCACCACAAGGGACAGCAAACAGGAGAATCAGGCTAAGGGTTCCCAAATCAACAGTTCCCAAATCAAGGGTTCCCAAATCAAGGGTTCCAACGGGAAATGGTTGTCAATGTTCCAAGCAATCATCAAACAAAATTAACACCTTCCTGTCATACCCTTTGGTGGTCTTCACTTTCTTCGTTTTCACTTTGTTAGCTCTAGGCTTCTTTTTTATTAGGAGGTCCCAAACTACTGCCCAATATTCTCAAGACCTCTCTAGAGTTTAATTATGACTATTAATTAATTAGTGTTTAGCGTTGCATAGCCTTTGGATCAATGTTACGTTGTATACAAAATTACAAACAAATGATGCACTCTTCATAGTAAAATAGTAAGGATAAGGA

>AiNAC6--Araip.2W5R5

GCAAAATCATAAACCGGAAATATAAGAGCTCGTCCAATGGGAGCCGTTGTTGACTGTTATCCGCCGCACGCCGGCGAGGTTGCAGTTTTGTCTCTCAATTCGCTTCCCTTAGGTTTCCGATTTCGACCTTCCGACGAGGAGCTTGTTGACTATTATCTGAGACAGAAAATCAACGGAAATGGAGAAGAAGTCTGGGTTATTCGAGAAATCGATGTTTGCAAATGGGAGCCTTGGGACTTGCCAGATTTGTCGGTGATAAGAAACAAGGATCCGGAGTGGTTCTTCTTCTGTCCACAGGACCGGAAGTATCCAAATGGTCACCGGTTGAACCGAGCAACGAATCATGGGTACTGGAAGGCCACAGGAAAAGATCGTAAGATCAAGTCAGGTTCCACCTTGATTGGGATGAAGAAGACTCTGGTGTTCTACACAGGTCGTGCTCCCAAAGGGAAGAGAACCAATTGGGTCATGCATGAGTACCGCCCCACCCTCAAGGAGCTTGATGGCACCAACCCTGGACAGAATGCGTATGTACTCTGCCGGTTATTCAAGAAACAAGATGAGAGTCTTGAGGTTTCAAACTGTGATGAGGTGGAACAAACAGATTCGGCTCCCATGGCGGCCAATTACTCCCCTGAAGAAATACAGTCTGATCAGGCTCTGGCTGAAGTATCGCCGTCTCAAGTTACAGATGAGAAGCACCAGGGTGTTATCCCTGAGAACTCTGAGGAAGCGGTTTCCAACGTTATAACCTCTGCTGATTGCCATAGTGACGGATATGATGCTTGTGAAAGGCGAAATCAAGCTTTTGAACTACCTGCTGAGGACATTCCGCCGTTGAATTGGGACATATTCAATGACCCCGAAGACAAGATATTTGATGACAAATTATTCTCCCCAGTCCATAGCCATATTCCACCAGAATTTTACTACCAAGCAAACAATGAGACAAATATTGCAGACATCTTAAATTCTGTCAATTGGGATGAGATCTCCTATGAGGATCCCTATAGTCAAGCACAGAACAACTTTTTTAATAATGTTAAGCAAAGTGTATCAGGTAGCGAACCAGATGCAGGGCTGACCAATATGACATGTGTACACCCGACGAATGTTGTTTATCCCGAGGAGGCAATTCACAGAAAGGTTGCTTTGGCAACAACTCCGCAATTTTGCAGCACCTTCACGTCTGACTTCAGTGCTGATGAGCAGAAGAGCAGTGTCGCGTTAATTCAAAACAATTCCCAGATGGCTTCTTTTCCGGATGCCAGAACAGTCCAAGTGTATAACGTATTCAATGATTATGAGCAGCCGAGAAACCTTAATACCTATGTTAGTGGTGATACTGGAATCAAGATAAGGACTCGACAAGTGCGAAATGAACAACCAGCAATGATCTTTACAGATCAAGGTAATGCAGCAAGGAGAATCCGATTGTTAAAGCAGTGTGCAGATGTCTCAAACAAGATGGCAGATGATGGGAGTCCTAAACAAGAGCATGATTCAAAACCAATAATTGCAGGGAACAAAAACAAAACTTTCAAAAGTCATACTGCAGATAAGCATGATACTGCTAATGATCTGAATGAACGCCAGGAGAAAACTGAATCAACTGATAAAAGAAACATGATATCTAAACTTGCTAAAGGAGGTTCTTCCATGTTGGGGTTGAAGGGATTATTGCGCAGAAGGCTTAGTTACATATCAAAGGCTTCCTCCAATTTCAAAATGTGGTCTTGTGTTGTTGTGGCTTCTGCCTTTGTATTGGTCTCGTTTGTGTTCTTTGCTAACATATGGGGATATATTAACTTATGAACTTCTAGGAGATCATTCCTTTATGCGTGTAATGTGCCTCCATTTTTTTTTCCTTTCTTTTTTTAGGGCCAGTTGTGGAGGCTTTTAGGATTGACTCCTAGATTTGTTGATAACTCTATTGTGAGAGAGTATGCGTTTCAATATGTAGCTGTATAATATACTTAGTTGCTTACCTCGCTGGCTAGTAGAATTTAGATCAACACTTGTGTTACCGTTTTCTTCTCTCTTAATTACTTGCTTACTACCTATGAATGCCAAGAAAGCTATTCGTGTAATAATGATGAGAGTAAAGCACTAAAAATGTTCTTTAATTATCAAAACATTAACAGAAATGTATTAATAATTAAAATATCCTCAA

>AiNAC7--Araip.310T2

CCGCTTCACGCTCACCACCACCACCAGAGAAAACGTTATCGCCCACGTGTATTCTAGAAACAGCTGGCTTACCATGCCACGTGTCCCCCTTCGCTCGCTATAAATACCCTTCTCTCCCTTACGTTTTAAGTTTCAAGCGCAGCCGTAGTTCTTCTAAGTCTTTCAGAGGGGCTAAAAGCTTCTTATCTACCTTCACCAGATCTCACTCTAATTATTTAGGTTTCACTAAAGTATCTCAGTTCTTTCAAAAATGGCATCGGAGCTTCAATTGCCGCCTGGATTTCGATTCCATCCAACAGATGAAGAACTCGTGTTGCACTATCTCTGCCGTAAATGCACTTCACAACCGATTGCTGTTCCGATCATTGCCGAGATCGACCTTTATAAATATGATCCCTGGGACCTTCCTGGTATGGCATCCTATGGTGAGAAGGAGTGGTATTTTTTTTCACCTAGAGATAGAAAATATCCGAATGGGTCTAGACCGAATCGGGCTGCAGGAACTGGTTACTGGAAGGCAACCGGAGCCGATAAGCCTATTGGACAACCCAAACCAGTTGGGATTAAGAAAGCTCTGGTCTTTTACTCTGGAAAGGCTCCCAAAGGAGATAAGACCAATTGGATTATGCACGAATATCGTCTTGCAGACGTAGATCGTTCTGTTCGCAAAAAGAATAGCCTCAGGCTGGATGACTGGGTGCTGTGTCGCATCTACAATAAGAAAGGATCAATCGAGAAGCAACAACCGAGCAGCGGCGTCAGTACCGTCGTGAACCAGAAGGCCGAATCTTCGGAAGTCGAAGACAAGAAGCCCGATATAGTTCCACGTGGCGGCGGCGGGGGCGTACTTCCACCGCATCCTCCTACGGCTCAGGCTTCGGCAGGCGGCGTGACGACAGATTATATGTACTTCGACAACTCCGATTCGGTTCCGAAGCTTCACACGGACTCGAGTTGCTCGGAGCAGGTGGTGTCGCCGGAGTTTGCGAGCGAGGTTCAGAGCGAGCCAAAGTGGAACGAGTGGGATAAAAACCTCGAAAGTGCGTATAATTACCTCGATGCCACACTCACCAACGGTTTTGGGTTCCCGTTTCAGGGTAACAATCAGATGTCGCCGCTCCAGGATATGTTCATGAACCTTCCGAAACCGTTCTGAACGCGCTAGTAATAGTAGAACACCAGAGAAAGAATCTTTTTATGGAAAACCTTTGATATGCAGTATGGTGCATTCCACGGCAGCACGAGACGTTAAGGTCCATGAGTGTGCCTGTGCGGGTGCGAGTCCATGGATGGGTCAACGAGATGGAACAGCAAGGGGAGAATCTGAAGAAGGGAAAATGAAAAAATGCATGCGTTAGGTGTGCAGCATTGTGCGCCCATTGATTTGGGTCGTGGGATTAGAATATCGTTTGGGGAGTCTGACGGTGGATGTTGGGTAGTCGCTAGGTTAGGCTGTGTATAGTTTATTTTGTACAATGTAGAGTGTAAGACCGGAGGGGGTAAACTATACGGTGGATGGTGGTGTATGTTGTTTCCATCTATTTATGATTAGTCAATATAAATGGTTAATTATTGATTTTGACACCGAATTAAAATATATGGTGGCAAGAATAAAATATTATGATGTGATAATGGTGACATTATGTCAA

>AiNAC8--Araip.31EFM

CTCTTTTTGATTATCTCTCTATCTATCTATGATCATTTCTTCTTTGTGTCTTAGATTCTGATTCTTGATTGGTTTCTCCATTTTTGGCAAGTTGTTTCTTGAATTGCAATAATGGCTAGCTGGGTTATTGACATTGGAGGACTTGCAAAGAAAGTGAAGAATAATACACTGCCATTAGCTGATCAAATCAATGACTGTGGAGCATATTGTGAATGTCCAATATGTCATTATCACATTGATAACATTGATGTTTCTCCTGAGTGGCCAGGCTTTCCGGCCGGCGTGAAGTTTGATCCTTCCGACGTAGAACTGTTGGAACATTTGGCAGCAAAATGTTGTGTTGGAAACAAAGTGCCTCATGCCTTTATCCAAGATTTCATCCCAACACTAGAAGGAGACCAAGGAATATGCTACACACATCCAGAAAATCTTCCAGGTGCTAAGAAAGATGGGGCCAGTGTTCATTTCTTTCACAAAACAACTAATGCATATGCTACCGGACAACGAAAGCGTCGAAAGATTAATCATCAACTAGGCCTAAGTGAGGAGCATGTTCGCTGGCATAAGACCGGTAAGACCAAAGCTGTAACAGAAAATGGAGTACACAAGGGCTTCAAGAAGATCATGGTTCTTTACATAAGGCCTAAAAGAGGTGCAAAGCCGAATAAATCGAAATGGGTGATGCATCAGTACCATCTAGGGAATGATGAAGGTGAGAAGGAAGGTGAATATGTGGTTTCAAAGATTTTCTATCAGCAGAAGAAGAAAACTAAGAAGAATAAGCTGAATCCATTGGTGGCTGAAGATTCTGTCATGGCATTACAAGCAAGTCCAAGAACACCGAATCCAAATCCACCAAAACGGCCTCGGACAGGAAAATCTGTTGACTGTGATGACAATTTTGATGAAACTGACCTTATGCTATTCACTCAGGATGGAAAGCCTACTATTCATGGAGAATCTCTTGCCCCACCACCATCTGAAGTTCATGGTGATGAAAATAATGGAGGCTTTAACAACACTGCATTGTTATCTGTTGAAACACAACCTGTTGAAAACTCTGACTTTATTGGATTGGATGATATCTTACTATGCAAGGAACAGACATTAGATTCTTCTTCTGCTTACCTAAATGATTCTGGTTTGAAGTCCAACAATCTGAAAGGCTTTGCTTGCAATGCAAATGGAAATGCAGCTGAATTGTTTGGGGATGTGAATGATTGTTATGGAATTTCAGTGCTGGATAACCTGGGACTGGACAGTCCTCCTGATTTTGATCTTTCTAATTTGCAATTTTGTTCTCAAGACAATATCATTGACCTTCAATGGCTGGACATACCATAATGTATTTGCTGAACCAAGTTGTATTTGACACCAATGCTAATCCCAGAAGAGATATCATCATATAGTTGAATCATTTAGCTTAAGTAGGTGTAAGGTTTTTCTATATGTGAACTGTTTCACTCTACCATGTTGTATTGTGAGAAGTATTGTCATTGTTTGTCTGAGTTTAGAATTTAGAATTTAGAA

>AiNAC9--Araip.333QY

AGAAATAATAGAAAGCCCTTATAAAAATAAAAAACAGAGGAAGGAAAAGAGTTCAAGAAACCAAAAAAGTTTTAGCATAATGGGAGTTCCAGAGAAGGATCCTCTCTCTCAATTGAGCTTACCTCCTGGTTTTAGATTTTATCCCACAGATGAGGAGCTTCTTGTTCAGTACCTATGTCGCAAGGTTGCTGGCAACCATTTCTCACTTCCTATCATCGCGGAAATCGATTTGTATAAATTCGACCCTTGGATCCTCCCAGGTAAAGCAATATTTGGGGAGAAAGAATGGTACTTTTTCAGCCCCAGGGATAGAAAGTATCCGAACGGTTCGCGACCGAACAGGGTTGCTGGCTCTGGGTACTGGAAAGCCACAGGAACAGATAAAGTAATCACTACCGAAGGCAGAAAAGTTGGAATCAAGAAAGCACTTGTTTTCTACATTGGCAAAGCACCCAAAGGCACCAAAACAAACTGGATCATGCACGAGTACCGTCTCCTCAACGGTTCTCAAAAGAGCCTCGGCAGCACCAAGCTAGATGATTGGGTTTTGTGTCGGATATACAAGAAGAACTTGAGCTCATCGCAAAAAGTCAATATGCCAAGCTTTACGAGCAAAGAATGGAGCAATGGATCGTCGCCTTCTTCATCGTCTCACATCGACGACATGCTCGAATTGCCGGAGATCGACGACCGGTGCTTCGCCTTACCGCGGGTTAACTCACTGCAGCACGAAGAAAAGCTCACCCTTGGCGGCACAGGCAATAATTTCCCGGACTGGGTCAACTCGGGGGGTCTCGACTCGGTCCCTGAGTTTGGGAGCCAATCTCAGGGGATGACAAGTTACGATGGAAATGACCTATATGTCCCCTCCGCGTCACAGTTCTGCCACGTCAACACAATGGTTGTACCGGGTAACCCGACGGAGGAGGAAGTCCAGAGCGGCATCAGGACCCAGCGGATTGATGAGAATTTCGGGTTATTTCAACAGAATTCGAATGTATTCACCCACCGGTATTTGTCGAGTTCGGGTGACTCATTCGGATTCGGATACCCGAATCAGCAATTTGGATTCGGATTCAGAGAATGA

>AiNAC10--Araip.4A49L

CAGAAGTTAAGGAAACAGTGTGAGAGGTGTGAGAGTGGGGATGAAGATAAAAGCGGAAGCACGGACTAGTTGCGATCGAAGTACTTGCTAATGACACGTTAAAAGAAAAATAATGGCATCAAACATGCTTGGTTCCCTCGCCGACCCTATTCCACTCAAAGACCAAACGCCTAAATTGCCATACTAAATTGTTGTCACTCACCAACAAACAAAGAACAGAACAACATAACTCTCTCTAACACCAAACCCTTTTACTTTCACCTTAGAATCATGGTGGATAGGGATTCAAGTGAAGCACACATGTCAATAGCCGCTTCTTCCATGTTCCCTGGCTTCAAGTTTTGTCCCACTGACGGTGAATTAATCTCTTATTACCTCAGAAAAAAATTGGACGGTGACGAGGACAGTGTTCAGATCATTTCGGAGCTTGAGCTTTGCACCTTTGAGCCTTGGGATTTGCCTGAAAAATCTTTCATTAAATCAAACGATGAGTGGTTTTTTTTCTCGCGACGGGGGAGAAAGTATCCGAATAGTTCACAAAACAAAAGGGCAACTAAAAGTGGGTATTGGAAGGTCACAGGAAAGGAGCGACAGATAGAGTCCGGTCAGAATGTGATTGGTACCAGACGCACTTTGGTATTCCATGTCGGTCGAGTTCCTAAAGGCGAAAGAACTGAATGGATTATTCATGAGTACTGCATCAATGACAAATTTCAGTCCAGTAACCAAATCAGGATTCTTTGGTGGTTTGTCGGCTCAAGAAGAACACAAAATTTCATACAAATGATGATTCTAACAAAGCTTCACGCGAGAGTGGTTGTGGAGTCTCAGAAGGGGGTTACAGTTCAAAGGAGCACTTGTGTGCCTATTCAAGATAAAGAGGTTGGGTGTAGTTCCAAGAGGAGTAACAATAGTAATAGTTCTCCTTCTATTACTGTCCAAATTGAATCCAGTAATAGAGTTGCTAATGAAGTCAATCCCAAAGCTTCTTCCAATGAATCCAGTGATAGAGTTGCCAATGAAGCCAATCCCAAAGCTTCTTCCAATCACTCTAAGGTGGATGAAGTGGATTATTATGCAGAGATCAACTTAGATGATATCATCAACTTAGATGAACCAGCACTCTGACTGCCATAGCCCATAGCCACCACAAGGGACAGCAAATAGAGATCAACAATCATCAAACAAAATCAAAGGTTTCAACGGAACTTCTTGACCAATGCTCCAACCAATCATCAAACAAAATTAACACTTTCCTGCCATACCGTTTAATGCTCTTCACTTTCTTTGTTTTCACTTTACTAGCTCTTAAGCTTTATCCTTATCGGGAGGTCTCAAACTGCTGTATAATATTCTCCAG

>AiNAC12--Araip.609WS

ATGGAAGAGAATCTACCTCCTGGATTCAGATTCCACCCAACAGATGAAGAGCTTATAACATATTATCTTACAAGAAAAGTCTCTGAAAATGGATTCACTTCTAAAGCTATTGCTGTTGTTGATCTCAACAAGTCTGAGCCTTGGGATCTTCCAGGTAAGGCAAGCATGGGTGAGAAGGAATGGTACTTCTTCAGTTTAAGAGATAGAAAGTATCCAACAGGACTAAGAACAAATAGGGCAACAGAATCAGGGTATTGGAAGACCACAGGCAAAGACAAAGAGATATTTCGTGGTGGGGTTTTGGTTGGAATGAAGAAAACCCTAGTCTTCTATAAGGGTAGGGCTCCAAGGGGTGAGAAAAGTAATTGGGTCATGCATGAATATAGACTTGAGAACAAGAATCCCTTTAGAACTAAGGATGAATGGGTAGTGTGCAGGGTATTCCAAAAGAGCACAGCAGCGAAAAAACCGCCGCAACAAACATCATCCTCCCAACCTGAATCCCCATGTGATGACACAACCTCTTTGGTCAATGAATTTGGTGATGTTATTGAGTTTCCAAATCTAAACACCAACATTAATAATACCAATAATAATAATAATAATTCTTCATCATCATCCTCAAGTGCATTATTCCCTAACAACATTCTTATTTCAGGACAACACATTCATCATCACCATGACCTAACCAATAATAACAATAATAACAATGTTAACACAAACATGAACTTAGCAATGAATTGGCCACCATCAAGTGATCATAATGTTCCATGGCCTTCAGTAGGGTTGTTGAATCCAAGTATTTCATCAATGAATTCCTTGATTCTCAAGGCATTGCAGCTTAGGAATAATTATCAACAAAGAGAAGTTGCATCCACATTTGCACCATCATCATATATTATGCCTCATCATCAAGGAGTAGTAGTTCCTCATCAACAAGTTATTATTGGAACCAATAATGATGACCTAATAACAACTTCTTCAAATCTCATCAATGCTTCTTCTTCTTCTTCATCAAAAGTTTTGGAATGTATGCCACATCAGCAACAACAACAACAGGAGCAACCATTCAATTTGGACTCCCTTTGGTAA

>AiNAC13--Araip.64GCN

ACCTTGATTTCTCTTCAACCCTTATCTTCAATTCATCCCCATCCTCTTTCTCTTGTTGTGTCCCCACTATAGTCCCAAAAATAATAACCAATTTAATTAATGGAGGGTAGTAGAAGAAGCTCAAATTCTGAACTCCCTCCTGGGTTTCGGTTTCACCCAACTGATGAAGAACTAATCGTTCACTACCTTTGTAACCAAGCCACTTCAAAGCCTTGCCCTGCTTCCGTCATCCCTGAAGTTGATATCTATAAGTTTGATCCATGGGAATTACCCGATAAAACAAGCTTTGGAGAGAACGAATGGTACTTCTTTAGCCCAAGGGATAGGAAGTACCCAAATGGGGTGAGGCCTAATAGGGCAACGGTTTCAGGGTATTGGAAGGCCACTGGTACGGACAAAGCAATCTATAGTGGGTCTAAGCATGTTGGGGTCAAGAAAGCTTTGGTCTTTTACAAGGGTAGGCCCCCAAAGGGTATCAAGACTGATTGGATCATGCATGAGTATAGATTGGTTGGATCAAGAAGGCAACCCACTAAACAAATTGGATCCATGAGGCTAGATGACTGGGTGTTATGCAGGATCTACAAGAAGAGGAGCATAGCAAAATCAATGTTGGAGCCTAAAGAGGAATTCCCAACAATGCCCCAAATCAATCATCATCTAACATCATCATCAAATGATGGGAATGATAATAATGATGATGAGCAAGAAATGATGATGAAATTCCCAAGGACATGTTCCCTTACACATCTTTTGGAAATGGACTACTTGGGCCCAATATCACAAATACTCTCTGATGGATCATATAACTCAACCTTTGATTTTCAACTAAACAGTGCCAATGTTGGCAACATGATTATGGACCCTTTTATGAAACAACCTCAGATCCTTGAAATCCCTAACAAAAATAATCATAACAATCCTTATTATGATGTGGATTCAGGGAAGAACAACCTAGTGAAACAGAATAGCACCATAAACCCTACTATATTTGTGAACCAATTCTTTGATCATAGTGGTAGTTAA

>AiNAC14--Araip.67R8V

TTGGCGACCAAGGATGCAAGGTTAAACTTGTAATTCTGGCCCTTACACGGTTTGCGTCTTTACGAAACTCGATTTTCTCTTTCTGACTCGATATCCGAACCAATAGGCTCGGTTTTGCTTTGGCTCACTTCTTCGACAGAACAGCAATGACGAAGCACAGGTTCGAATGCACAGTCTCTTCCCATTACCAACAAAAACCCCAGGCTACAATTTCCCGCTCACTCTACCGACTGTCGCTTTCTTTGTCTCTCTGTCGATTGAAAATGGGAAACCGATGCTGAAGCTCAATACTCTCCACAGTTCTGCTTCAGCTCAAGCTTCTTCCTCTTCCTCTCTGCAATGGCTAGGTTTAGAAGGGGAAGGGAAAGAAGTGAAAGGTTTGAAGAGTAAAAGGAGCTGGCTTATTGACATAGGTGGATTTGCAAAGAAAGTGAAAAGCACTAATTTATCTCCAGCTGATCAAATCAAAGATTGTGGGGCATATCGTGATTGTCCAAACTGCCATTACCGTATTGATAACCGTGATGTTTCTACTGAGTGGCCTGGCTTTCCTGTTGGTGTGAAGTTTGATCCTTCTGATGTAGAACTCCTAGAACATTTAGCAGGAAAATGTGGCATTGGAAATGCTCAGCTGCATATGTTTATTAATGAGTTCATTCCAACAATAGAAGAAGAAGAAGGTATTTGCTATACACATCCAGAAAATCTTCCAGGTGTCAAGAAAGATGGGAGCAGTGCCCATTTCTTTCATAGAACAACCAATGCCTATACTACTGGTCAACGGAAGCGTCGAAAGATTCATCATCAATGTTTGACTGAAGAGCATGTACGGTGGCATAAGACTGGTAAGACAAAAGCTATATTGGAGGATGGAGTGCATAAGGGCTTTAAGAAGATCATGGTTCTTTATATAAGACCTAAGAAAGGGTCCAAACCTGATAAAACGAATTGGGTGATGCACCAATATCATTTAGGAACTGATGAAGAGGAGAAGAACGGTGAATATGTGGTTTCAAAGATTTTTCAGAAGCAAACTGAGAAAAATGAGGAGAATCCAGCGGTCGAAGATTCTGACCAGAATGAGAAAAATGAGAATCGATTGGCTGATGATTCCAACTGTATAGCATCCCGAACCAGTCCTAGAACTCCGAAACCAAATCCCCCAAATCCACCTAGAGCTGGAAATTTTGTTGACAATGATGATAATATTGACGAAACTGAACTTCCATTCACTCAGGATGTGAAATGTGTCCCACAATGTGATGTTCTGGATCAGAACAATGCTGGTGACCCTGCATGGCTGGCAGGTGAATCGCAGGCTGTGGAAAACTTCGACTTTGATGGCTTGGATGACATCTTGTTCTGCAATGAAATATTCGATTCATCATCTCTACTAGATATTTCTGGAACGGAAACCATGATAAATGGATCTGCTTCAAACGATATGCTTGGGAATGATGGTTTATCATACGGAACTTCCGTTCTTGATACCCTTGACTTGGGTACTCCCCCAGATTTTGATCTTTCAAATCTGAATTTTTACTCTCAAGATAGTATTTTCGACTGGGTCGACAGATTATGAAGTGATTTCTGAAGTTTGAGTCCATCAGAATGCATGTTTGTTCAGCTCTTAGATGCTTCAGATTCAAATATAGTAAGAGAAACATAGGTTCTCACGTGATTTGCAAGCAGAAATAACAGTATTTGTTTGTCCCTGGTTCTTGAAATTCTGCAAATTTTGTAATCCCACACATTTGGGCATTGTTCAGGTGGGTGTATTGTTCATGTGGATTTTAGGTATTATTCACGTGGGTTGTATTTGTGTCAAATAGCTTTTGTGGTATATGTTGTTTCCATAGCCATCTAAACTTGGTTGCCTCATTCTGTTGTGGTAGATACATCTCTGGTAGCAAAGTCCAAACTTATATAAGTACTTGATATTCGACTGTTTTTATGTCAATACTAGAATAACTATCCGC

>AiNAC11--Araip.6CI1F

ATGCACAAAAACACAAGGGATGTAAACAGTTACGTGAAAGGCTATCTAATTAATTGGGTGAAGTGTTCTTGTTACATAACAATTTAATACATCAAGACCAATAATAATAGAAATAATAACATAGAAATGAATACAAAGATTGAACTGCCACCAGGTTTCAGGTTTCATCCAACAGATGAAGAGCTCATAACTCACTACCTCTCTCAGAAGGTTGTTGGTAGCTGCTTCTATGCAACTGCCATTATTGGAGAGGCTGATTTCAACAAGTGTGAGCCTTGGGATTTACCTTGTCAGTATCTCAATTTTTTTAATGATATTATTTTTTATTTAGCATCCTTTGATTTATTTTGACTTCAAAATTTGTGATTTTTATTTATTATTTTAATTTTTTTCAATGAAGGGAGGGGCAAAATGGGAGAAAAAGAATTGTATTTTTTCTGTTTGAAGGACAAAAAATACCCAACAGGTGAAAGGACAAATAGAGCCACTGGCGCTGGGTACTGGAAGGCCACAGGGAAAGACAGAGAGATATACAATGCAAAAGCAAAAGCACTTATTGGGATGAAGAAAACACTTGTTTTCTACAAAGGAAGAGCTCCAAATGGTGAAAAGACAAATTGGGTCATGCATGAATATAGGTTGGAAGGCGATAATAAACCTTCTATATACAATCTACCCAAAGAAACCAAGAAAGAGTGGGCTTTGTGCAGAGTTCTACACAAAAGTGAAAAGAAAGTAATGCATGTTCCACAACCACAGGGATTGGTTGAGTTCAGCTCCTATGAAAATAAGGAACTTCCCCAATTAATGGATTCTTCACAAGTAACATTCTTTTCATCAGACCCAAATAATCAAAGTGAGGATCCAAATCCAATCACACGTGATGATGATAATAATAATGATGACATCATATTTGATAGCATTGAAACTCCTTTCTTGGAACAACAACCACCTTATTCTTCATCCTATGATTCTTCAGATTTAGACACCCTTAACCCTGCCACATGGGATATTTCCGAAAATGCCCCTACAAGTAATGCGTCTAAGGAGACGGACTTTGATGCTGACATGTTCTCTTTGATGTACAACAATAGAGAAGTGTTCCAAACATCATTTGAGAATCAGGAATATTATGCATATGATTCTGTAGGACATGTGGACAATGGTTCCCTATGGAATTTTTAG

>AiNAC15--Araip.6Y0GY

GAGAAAGCATAGAGAATAGTTTTCACATGTATGTGTTGTTTTACTTGAGACTTTAAGTGTTTTTAGAGTCATGGATGTGGCTAAGTTGTACATGAACAACGACTACTCCGAAGAACATGAACATGAACATGAAGATGAAGATCATGAGATGATGAAAGAGGAGAAAGAAGTTGTGCTTCCTGGGTTTAGATTCCACCCAACAGATGAAGAGCTTGTTGGGTTTTATCTTCGGAGGAAGGTTGAGAAGAAGCCTCTTAAGATTGAACTTATCAAACATGTTGATATCTACAAATATGATCCATGGGATCTTCCAAGCACCAAAACTGATTGGATGATGCATGAGTTTCGTCTCCCACCCAATAATAATAATGGAGCAAAATTATTAAGCAATAATCAAGAAGCTAATAATGCTACCAAGGATCTTCATGAAGCTGAAGTGTGGACACTATGCAGAATATTCAAAAGGATTCCAACATACAAAAAGTACACACCAAATTTGAAAGATTCATCAACATCACCACTGATGAACAAACCCATCAATAACATTAACCACCAAACTGATTCCTCAGTAACTTCCATATCATGCAGCTTAGAATCTGACAACAACAATAGCAAGCCATTCTTGACTTTCACTAACACTATGACTATGGGCATTCAACAATGTGAAAGGAAGCCTCTTGTTATTGGACATGTTGATGAAAGGAACAACAACTTTTTCTTAGACCATTCATCAATACATCATCAACAAGCTCCAACAACAATTACTACTACTGCTTTGTCATCATCATCATACTCATCATGGAACCAGCACCATCTTGTGGAGGATTACTTGTTTGCAAATGAGAATTGGGATGATCTTAGATCTGTGGTTGAGTTTGCCACTGACCCTAATAATTCCAAGGTTTATCTATGATTGTAATTAAAGAGTTTAATATATATTTTGAGAGGGTATTATTATTAGGGTATTTAGCTTCTTAAA

>AiNAC16--Araip.714GL

CTCAAACACCCTAATCACACCCTTAATCAACGACTTGAATACCACGCAATCACATCATGTCACGGAGCTCACACTCTCTCCCTGCTCCCTCATCGCACTGAATCACTCTCCCATATGAAGGTTCCCAGTCATTACTGCTAGGAGTCTAAGTACTACGCCAAGCTTCCTTCTTTGACCATAACACTATCCAATTCCAACCCCCCAGACCCCCTTTCTTCTTTTTCACACCGCAATTTTTTCCACAATCGATCCCATTTTAAGCTTCTCTCTCCCTCTAGGTTTTTTTTTTTTTTCATTCGACGGTTCATTCCTCGAATTCTCGTACCCCGAGGATTCGATTCGGGTCATGTAGTCGTAGGTTTTCGGTTTCTACAACCGCTTGATCCACAAAGCTCGGATTCTTTTTTTTCTTCACTTTTTTGGGGGGTTGTGAATTCTGATCCTCTATTTTTGGGGGTTTCGATTCCGTGATTCAATTTCTTCTTCCGGATTCCTCTAATCGCATCGATTAACTGGGTGTTTGGTTTCGTAGCCTGTCGATTCAGAAAGTTTCGGTTTGTTCCTTTGGGTGTTTGGTCTAGGGTTTTTCGGATTGAGTATGGGTGCTGAAGCTGGTGCAACTGAGTGTTTCAGTAAGGCCATGGCGTCGATGCCTGGGTTTCGGTTTCATCCCACGGATGAGGAGCTGGTTATGTACTATCTGAAGAGGAAGATATGTGGGAAGAAGCTGAAACTCGACGTGATTCTAGAAACCGATGTTTACAAGTGGGATCCTGAGGAATTGCCAGGGATCTCTGTACTGAGGACTGGAGATAGGCAATGGTTCTTTTTCACTCATAGAGATAGGAAGTATCCTAATGGTGCGAGGTCCAACCGAGCAACAAGGCAAGGTTACTGGAAAGCAACAGGAAAGGATCGTAATGTGACCTGCAATTCTCGGTCAGTTGGAGTGAAAAAGACTCTGGTTTTCTATAGAGGCAGAGCTCCTAATGGTGAGCGGACCGATTGGGTTATGCATGAATACACCATGGATGAAGAAGAGCTGAATAGGTGCCAGGATATTAAGGACTATTTTGCACTTTACAAGCTATACAAGAAAAGTGGACCTGGTCCTAAAAATGGAGAACAGTATGGTGCACCATTTAAAGAAGAGGAGTGGGCTGATGATGAATGTGTAGATTTCAATATTAACTCAGCAGATCGGGAGGTAGTAAATACTGTCCCTGTTAATGATCAGCTGCCTCCTTTGGCCGATGATGAAGTCACGGATATGATTAATCAAATTTTGGATAATGAGCTTGCCCTTGACCAGCAATTTGGTGACGACCTTCTTGAATTTCCTCAGGTTGTTGCTGAAGAAACACAAAGTACTGTGGTGGATCAGTTCTCTGAGGCAGTGACGGACCCCGAGTACAATGATATTTACCACTCAACCAGTCAGCACTATGATGCGCAGAATGTCAACTTCAATCAGTCGGTTGCATCTCACCTTCATGCCCCGGAAGGATCAGAAGTTATTTCTACTGCCAACATTCAAGTAGAAGACTATAACTTTCAGGAGGATGACTTCTTGGAAATCAACGATCTCAATGGTAGTGAACTTACAATTCCAAATATGGAAACACCAGTGGAGAACCTGCAGTTTGAAGATGGATTGTGTGAACTTGATCTGTTCCAAGATGCAGAGATGTTTCTTCGTGACTTGGGACCAATCAATGAGGAAACCATTCCACATTCATATATGAATAATGCCCCTGGAAGCAACATTGAAAATCAGAATTATCACTTGCTACCCAATCCAGAGGACACTACTCAAAATGTTCATGAATTTTGGATGCATGATGAAAGAAACACCCTGAGTCTGTTTGAAGGCTTTGATGATTCTGTCTCTCAACAAAATCCAGGTGCTGTATGTGACTCTGCCAGCTTTCCTACTACTGAAGGCTATGATAATCAAAGTAGCATTGCAGAAGATGTTGCTACAAGTAGATTCTCTTCGGCTCTCTGGTCCTTTGTTGAGTCAATACCTACCACTCCTGCATCAGCTGCGGAAAATGCTCTAGTGAATCGGGCTTTGAATCGAATGTCTAGCTTCAGCCGAGTGAAGATTAATATCAAGCCGACGAACACAGCTGCAGGTAAAGACACTGCAACTACGAAGAGAGTGGGCAGAAAAGGATTTTCATTCCTTTTCTTCCCAATTCTTATTGCTTTATGTGCTTTCTTATGGGTTTCTCTTGGAACTTTTAGATTATTAGGGAGATGCATCGCTCCTTGAATATGTAAATCAAAAGTTTTGATGCTATTATGCTATATATGTTCTATCTGGGATCTCCCCAAGATGACTGAAATAGTATTGTTCTAACATATTCAAAAAATAAAAAATAATATTTTATTCTTTGGGACTGTTAGAAGTTTTAAGTTTAGGCAATATTAGGACTATGGTTGGGAGCAACGGTGGAGAGGGGAGGGTGAGGTGTGGCTGGGACAGGTGTTCGAAAACCGTATTTCGGGGAGTTTGATGGGGAATGCAAAGCATGGCATAGGAAGGGCATGTGGGAACCCTTGATCATGACCCCTCTCCAAATTGGAGGGGTGGATACAGAACACTTAGCTGTCCCTCCCAGGCCTTCTTCATCTTCCAAACAATAAATCACCAGCCATTCCTCTCCACTCTTCCACTTCATCAAGGACCCTTCCCCTCAACCCCCTCCATACTTCCAAACGTAGCCTAGGTGAGTCTTAACAATCTTAATCCAAGAGAAAGGGGTTTATATGAATTTGCAGGGTGGATTGGTAATGCACCAAGTTATTTATTATTTTTGTTTATTTATCGGACCCATGTAAATATCTTGTTTGGGTTTTTAAGTCAGACAGCACTACCATTGGATGTTGATTGTACATTGTAAAGCTGAACCCTTTGTGAGATATCGTAGTATTGATGTATCTATACCTATACCCTTTAAGACATTTGTAATATTATATGCAATTGATAGTATATATGCCTTGGGCATTATATTTTTGTTGTTAGAAGCACGGTAACCCGGAAAATAAAGCTGTGTGTACTGTGTAGTTCTCGATAGCTGTTGTGTAAGCAATTGATGGTTACTACTCCCTTAACATGGTTGCTAACCCGAAATGCGTTTGCGGTATGCTTTGTATTTTTTGACAATCGAGATATTTCATATGGCTTAACACTCGAAAATTCTAAGCTTGAAAGGATATGCAATGCGCATACCATAATTTCGGATGATTGGTATGCTCCCATGAGGAAGGAATCTGTCTCCTTGCCTTCTGATCACTCAAAAGTCAAAACTACAATCCCTTTCTGTACGGACTAGCTTAAAAAACTCGCACCAAAAGTTTATCTAAATTAACATAATCTTGTGAGTTTTATTGACTGGTTGTAATTCTTTTATCAATTAT

>AiNAC17--Araip.71CS3

CCCCAATTAAAACCACATATATAAACAAAACCCTGGCCGCTTCTCACTCTCTTGGTAGTCCAAAGATTCAACGATTAGGAGTGCGAAGTTTCAAGTCTGTGTTCTTCTCACTTCCCTCTTTCCTTCCCTGGGTTCCACTGTGCCGATGAATTTTGTTCTCAGAAAGGGACCGTCTTCATCCATTGTGGGTTTCCAGTTCCATTTTACCTTCATGAACCGGGGAAGGCTTCGCTGTTCTCGCAATTTCTTTCGCCACTGGACTTGACGCTTCGCACCCTCGATTCTTTTAGGGTTATTTAATTCCGTTCATCATTCTCTAGTTAAATTTCTCGTCTTTTTTATTTTTTATTTTTATTTTTAATTGGGAGTACCTACAAAGTTGGTTTCTTTCTTCACTGCTGGTGAAGTGGTTCTTTGTTGTTGGATTGTTTGATTTAAGGGAAGTTGTTAGATTCTGAAGGTTTTACTATTTAAGCTTTCTTTATTCTTCTGCGATGGCCGCAATGAAGTCAATTCCAGGGTACCGGTTTCATCCAACCGATGTTGAGCTGGTTCAGTACTTTCTGAAAAGGAAGGTGATGGGGAAGAGATTCCCTTGTGATGTGATTGCTGAACTTGATATATACAAATATCCGCCGTGGGATCTACCAGATCATTCTCTGCTTAAAACTGGAGATTTAGAATGGTACTTCTTTTGCCCTCGAGGGAAGAAGTATTCGAGCGGAGGGAGGATGAATAGGGCCACAGAATGTGGGTACTGGAAGACTACTGGCAAGGATAGATCCGTCGAGAACAAGAAGCTTGTTGTGGGCATGATAAAGACTCTGGTGTTTCACAATGGTAAAGCACCCAAGGGAGATCGAACTGATTGGGTTTTGCATGAATACCGACTTCAAGATAAGGACCTTGCTGATAAGGGTGTTCAACAGGATTCTTATGTGATATGTAAAGTGTTCCAAAAGGATGGTCCTGGTCCTAGGAATGGTGCACAATACGGAAGGCCATTTAATGAGGAAGACTGGGATAAAGAGGATGAAATTGACTGTGTAGAATCTGCACCTGTTGCTGCTCTACCTGCTGCAGTTCCTATACAACCCGCTTCATGTCATAGCTCTGTTGTGAATAACGTGAATCTCTCTGTGAGTGAATGCTATGGGTTGACCTCTGTTTCGTGTTTAACAGGGCCAATGCCTTCTTGCTCAGCACATCCTTCAGCTCCAAGTAATCAAGTTGATGGTGATATTACACCAGTGCCTGGTTCCGCCATAGAAGATAACATAATGGCTCCTACTGAGAACACCACAACTGAAAAGGTTGACAATCCTCCTGACATAAACAATGCTGAAGGAACACCTTGCTTTGATCCCAATGAGATTTTTGGGGGTCTGGGTGACCTTGATGGTTTGTTCGAAATGGGTGGAATTGGACATGGTTTTTCCTGCGGCCAAAATGGTGGATATACTGTGAATGAAATGCTTTCTGCGAGTGATGGGTTGCGTTTCCCTGATCCCCTGGACTACTTGGAGTTGGGTGACCTCGACACCCCATTGTTATGGGAGACTAATGAACAAGGAAATTGGAGCCAGGACAATAAATGAGGCTTCAAAATTTGACGAAGCATCAACATGAGGTTGGTAGAAGAACCGTTGCCGTGTGTTGCTGATCTTGATTGTCTTTCCATTCTCATTTATAAACTCTACTGAAGCATATATTCTGTACAAACATTCCTTTCTGTAATAAGCTAACACATGATAGCAGTTGATAAAATCTTTAGTGTTAGTTCTATTTACTAGGTTGGATTAATCATTAGCCTCACAAATTTTCTTCGGATATTTTCTGAGTTGTGTTTGTTAGAGTGAGTTTTCAACATCTATTACGTTGAAATCTATGCATGTCTTTCGTAGTAATATCAAATGATGGAAGTTGACACATTTAACCCTAAACTTCTAGTATTCTCAATTTTGTTTTATTGGAGAACGGTTAGTTGATGAGGGAACTGTGATAGGCCTTTGCCCCTTTACTTTTCGTCGCGCTGGAAAGTACGTTATCTAAACCAGTATAGC

>AiNAC18--Araip.77ISR

ATGCGTACTCTACACACAGAGGTGGCTAAGCTCAATGCAAATGAATGGTACTTCTTCAGCTTCCGTGACCGCAAATACGCCACCGGGTTTCGCACCAATCGCGCCACGACATCTGGCTATTGGAAAGCGACCGGCAAGGATCGTACGGTTCTCGATCCCCTCACCCGCGAGGTCGTAGGGATGCGGAAGACTTTGGTGTTCTACAAGAATAGAGCCCCAAATGGCATCAAAACTGGTTGGATCATGCATGAGTTTCGCTTGGAGACCCCACACATGCCACCTAAGGAGGATTGGGTTTTGTGTAGAGTGTTTCACAAGGGCAAAACAGACAATAGTGCCAAACTAAGCCCACAATTCATGTATGAGGCCACACCTTCATCCCTAACTTTGGCTTCATCATCATCATCCCCACCAACAAACCAAACAAATTGCAACAATTTGCATGTTATTGGGTATAACCAACTTCCCAATTTCTCATCATCATCATCACCAATGGCAATCCACCATAATCATCATCATCATCATCATCATCAAAACCAAAACGGTTCTTCCTCTTTGATGAATCTCCTTCAATTTTCCACTAAGGAAAATAGTACCATTACTCAACTAAGTCCCAAAGGTGGTGGTGGCGGCGGCGGCGGCGACGACGGCGGCTATGGGTTCATGTGGGACATGGATCTTGAGGAAAATAGCTTCCATGATGGTGGGGTTATTGCATCAAACTTGAACGACATGAGATTTGAGGTTGATAATAACACTATGGTTATGTTGTAG

>AiNAC19--Araip.78PTT

CTCTACCATAAAGAAAAAACACTACTTTCTGTTGCCACTTTCTTCACTGACAAAGGAATACCTGCACACCTTCCTCACACACGCACGCATACTCTCTTTCTCTCTCTCTCTCTCTAGCATTGTGGATTATGATAAGGACCCTGTCAGGGAGAGGAGAGTAGAGAGAGATTTTCAGAATAGGACACTGCACACACAAGAAGAAAACACACACATTTTTCCAACCCCATATCATACAAAGACACCAATAATAATAATAACATGAAGCCCTTATAAACCAAGCACCCTCCTCCATACCAAAAGGCACCACATACAGCAGAACAACAGAAGAAAGAAAAAAGTGATCCAAACCCCATTATTAATCTCCTTTCACTCTTTTGCTCCTTCTGCTTTTTCTTCAAGTTCTTTCCATTCTCATACAATCCAAATGGCCCCTTAAGAAATACAAATAGATCGAAGAAGAAAGAAAACACAACCACCAAGATTCTCCTCATTCATTCATTCCAACAAAAGGATCTTTACTTAGTTCCTTTTGTTCCTCCCCAAAAAAGAACACTCTTTATTTATTTTCCTTTGAACACTCTTCACCATGGACAGCTTCTACCACCACCATAACCACCACTTTGACAACAGCGACACTCACTTGCCACCTGGATTCCGATTCCACCCCACCGATGAAGAACTCATCACCTACTACCTCGTCAAGAAGGTTCTCGACAACACCTTCACCGGAAGAGCCATAGCTGAAGTTGACCTCAACAAGTGTGAGCCATGGGAGCTCCCTGAGAAAGCGAAGATGGGTGAGAAAGAATGGTACTTCTTCAGCTTAAGGGACAGGAAGTACCCAACTGGGTTGCGCACAAATAGAGCGACGGAAGCTGGGTACTGGAAAGCCACTGGAAAAGACAGAGAGATCTACAGCTCCAAGACTTGTGCTCTTGTTGGAATGAAGAAGACACTTGTTTTCTACAGAGGAAGAGCTCCCAAGGGTGAGAAGAGCAACTGGGTGATGCATGAGTATCGCCTTGAAGGCAAATTTGCCTACCATTATCTCTCTAGAAGCTCCAAGGATGAGTGGGTGATTTCTCGTGTGTTCCAGAAGACAACCACCGGGGGTGGCGGTGGAGGCGGAGGCTCAGCCGTGTCAACCACCGCCGGCGGGTCCAAGAAGGCAAAAATGAGCACTTCAACCACCACTTCTACAATGAGCTTCTGCCCTGAACCAAGTTCTCCCTCTTCGGTTTACCTTCCACCACTTCTCGACTCCTCACCTTACACTACCGCCACCACCGGCTCCGTCACCTCCGCCGCCGCAGCATACGACGGCCGCCAGAGCTCCTCCTTCGACAACAACAACAACGATAGCACAAGGGAGCACGTGTCCTGTTTCTCCACAATCTCCAACAACTTTGTCAATGGGTTCTTCGATCTTGCTCCTATGGACTCCTTCGCTCGATTCCAAAGAAACAACAATGTCGGTGTTTCTGCATTCCCAAGTCTAAGGTCTCAGCAAGATAACCTTCAAATTAACCCTTTGTTTTTCTCCGCCGCAGCGGCGCAGCCTCTCCACGGCGGCGAGCTTCACGCCGCGGGGGCCTGGCCGGTGCCGGAGGATCAGAGGGTTGCCGAGGCTGCTGCCGCCGGCATGGCTTTGGGACATTCCGAGCTTGATTGCATGTGGGGCTATTGA

>AiNAC20--Araip.79TDF

AGCCTCTCCCACACTTTCTACCTTTGGATCTTCTTCTTCCCCAATTGAGTTATACTTTGTACTTTTGTTTATAAGAGGAAGAAGGAGGTCCAAAGCTAATTAGGTCATTCGGATTCAAATTATATATAGAGAGAATATATGGCACCAGTTTCATTACCCCCAGGTTTCAGGTTCCATCCAACCGATGAAGAACTTGTTGCTTATTACCTCAAAAGGAAGATCAATGGCCGTAAGATTGAGTTGGAGATCATTGCTGAAGTTGATCTCTACAAGTGTGAACCATGGGACTTGCCAGGGAAGTCATTGTTACCGGGGAAGGATTTGGAGTGGTATTTCTTTAGTCCTCGAGATAGGAAGTATCCAAATGGGTCAAGAACGAACCGTGCAACAAAATCTGGGTATTGGAAGGCGACAGGGAAGGACAGAAAAGTAAATTCACAATGTCGTGCTGTGGGTATGAAGAAAACCCTAGTTTACTATCGTGGAAGGGCACCTCATGGCTCTCGCACTGATTGGGTTATGCATGAATATCGTCTTGATGATAGAGAATGTGAAAATGCTTCTTCTGGCTTGCAGGATGCATATGCACTTTGTCGTGTGTTCAAGAAGAGTGCAGTGATAACCCCTAAAGTTGATGAGGAACATCATCATCACCATCACTATGTTAATGCTAATAATCACAATAATAGCAGCCATGCTTTGCCAATTACAAGTGATCAATCGTCAAGTATGGAGTTATATTCTGAAGGAAGGGGTGAAGATTTGGATAATAGCTCTAATTATTTGGTTCCCATCGATACTACTTGCACACTACCCCTCAACAACATGGTGATGAACAATAATAATAGTGATGCTTCTTTCAATAGTAGGGATAATAATGGGAAATGGTCACAATTTGTTTCAGAAGATCCATTGTTCAGCTTTCCAACTTCCTCCTCATCATTTGCTAATAGTTATGGATCTATAACATATCCTCCATCCAAGGTGGATATAGCACTAGAGTGTGCAAGGATGCAACATAGGTTCACCATGCCTCCATTGGAGGTAGAGGACTTCCCTCATGTTGGAACCTCGGAGCTGAAAATGACAGAATTAACCTCGGGTGCCGCATCCGCCGTGCACGGAACCCGAAACGAAACGGATATCTTGCAGGAAATTCTTTCGGTTGCTCATGCTTCCCAGGAGTTGATAAACCACTCCAGCTACTCATCATCATGGGGTGGTGATGGTGGTGGCAACCATGAAAATTGTGCAACTCATGGAGATGATTTCACTTTCATGGTTGGTAGCACTAACTACAATAATAATAATTTGAATGACATTAACTCCATGAGATATGTTGATAGAAATTGGGAAGATCCAAACAGTTCAAGATCCATTGATATTGGATATTTGGATGAAGAATTTAAGGGAGAGAGGATGGTAGAGAATTTAAGATGGGTTGGAATGTCTACAAAAGATTTAGAAAAGAACTTCACGGAAGAGCAAAAGATTGTTCCAATAGAGGATATATCAAGCTTCCGGACAAATAATAAAGAAGAAAATGAGGTGCAAGAATCTGAGCAACACCATAGCAACAAGGAACTATTGATCAATGATTTCTCATTAGGGTTCAACCCTAATAACAACAACAGCGAGAACTTTCTAGATGATGATCATAACAACATGGATAACGATGATTACTCAAGTTCTCCAAGCTTTGAAGTCATTGAGGAAATAAGGGTCAGCCATGGATCAATGTTTGTTTCGACTCGCCGCGTCGCTGACACATTCTTCCACCAAATAGTTCCTTCACAAACCGTCCAGGTTCACCTCCTCAATCCAGTGATAACAAGCAATGAAGAAGAGACATTGATGATGATAATGGAGAGGAATCAAGGGTATTTCGGGGATTTTCTTTTCAGGACAATAGCAACTGCGTTTGTGTTCATCTTTGCACTTGTATTCGTGCATTGTGATTATTTGAAGGAAGAAGTGGAATTGGTGAAGAGAAAGAGATCATCACAATCATCATCTAAGATCATGAAATGGAGCAACAATAATAAGGTTTGGTTTGTTGGTTTCAAGAGTAGTGAGAAGGGATTTGGTGCAATTTTAAAGAAAATAGGGATTTTTCTCACAATATCTTTGGCTCTTTGTACCATGTGGGCTAACCATGTTATTGTTAACCCTTGATATCTTCCTCCTAGTTTCGTTTATTTTTGTCTCTACCTTATGGAATTCTCTTGACATGGCTCAAATTAAGTTTGGGCTTTTTCCTAATTAAATTAAGTAGGAGGCTGATGTATCCATATACTTATTATAATT

>AiNAC21--Araip.7L9YW

ATGAAAGCTACTGAAAGTTGGGTAACATCATTTTGTATGCTAGTTTGTGATCTATTGAGTGGTCTTTGTTACATGGGAATTAATGGTTTCAACTTCAAACTAAACATGATCTGCTTGGAAAAAATTGCATATTACCAGTTTAAGCGTTGGTTCAAAGGACCGGTATTGCTTGATATTCATGTAACTCAAGAGTGGCCTGGCTTACCAAAAGGGGTTAAATTTGATCCGTCAGATCAAGAGATAATCTGGCACTTGCTTGTAAAAGCTGGTGTAGGAAATTTAAAACCTCATCCTTTCATTGATGAGTTTATTACTACCCTTGAAGTGGATGATGGAATTTGTTATACTCATCCTCAACATTTACCTGGTGTCAAGCAAGACGGAAGGGCCTCACATTTCTTCCACAGAGCAATAAAGGCTTATAATACCGGCACTCGAAAGCGTCGAAAAGTACATGGTCAGGATGACGTTCGTTGGCACAAGACTGGAAGGACTAAACTGATCACCCTGAACGGGGTTCAAAAGGGTTGCAAAAAAATCATGGTTTTATATACAAATGCGGTGAGAGGAGGAAAGTCAGAGAAAACTAATTGGGTTATGCACCAATATCACCTTGGGACAGAAGAAGATGAAAAGGAAGGAGAATATGTTATTTCTAAAGTTTTTTATAAAGAAGACCAGGATATACCTGAAGCCGCAGAAAGTAAGAATGCAACAGTTGCGAAAGTAGATCCAGTCACTCCCAAATCCACGACTCCTGAGCCTCCTCGTAATGAAAGGCAAGATTCAGATCTAGGCCTGGATCTAGACCTAGGGCAAGAAGCACTTGCTTTTCCTGAGATGGATTGCTTAGATGAAATTCAAGCTGACTGTGAAGAATCTGCGAAAGCTAATCCACCAGTACTGGAGACACAAGAAAATGAAGGGATGGACAACAAGGAAACTAATGCTTATGAAGGACAACTGTGGTGGGATAGTGATTCACAGAATCTATTAGATTCACAACAACTCGTTGAAGCATTAACTCTCTGCGAAGATATATTTCACAGCCAATCTTCCAACAAAGACGATGAAAATGATAAGAACCAAACCGGTCTATCTGTGTATGCTCATCTAGGACCAGAGCATCTGAAGAAGGATATTGAGGAGTGTCAAAAGCTTGCTCCTGCAGGACCAGAGCATCAGAAGGATATTGAAGACGGCCAAAATCTTGACATCGACCTTGCAAATATAGAGCGGGATACTCCTCCTGAGCATCGACTAAGTCAGCTGGAATTTGGTTCGCAGGACAGCTATACTTACTGGGGTTTTCAAGGCTTCTGGATGTGGACTCGAGAGAGAAAGATGCATTTAGATTTCTACTCTATTCAAGCAGCTATGGAATTATCAAACTTAAGACTGAAAAATAGTAATGTTGGCTTTGTTCTACAGTACCAAGTAGTATCAGGATGTGAATTTTATCCAAACCCTCAGTTATACGATGGTGTGGGTTAA

>AiNAC22--Araip.8NR3H

TTTAATTAACCCTCCCTATATAGACCTCAGCTTCTCCAAGCCTCTCTCTCACGTTCCAACTTCTAAGTTCTAAGTCGCAAACAAGAAGAAAAAAGAAAGGAAGAGGCCGGAATGAAGAGTGAATTGGAATTACCACCTGGATTCAGGTTCCACCCCACTGATGAGGAGCTTGTGAATCACTACTTGTGCAAGAAATGTGCTTCACAGTCAATTGCTGTTCCTATCATCAAGGAGATCGATTTGTACAAGTTTGATCCATGGCACCTTCCAGAGATGGCTCTATACGGCGAGAAAGAGTGGTATTTCTTCTCTCCTAGGGACCGCAAATATCCGAACGGATCACGCCCAAACCGGGCTGCGGGTTCAGGGTACTGGAAGGCCACAGGTGCTGATAAGCCCATTGGAAAGCCCAAGGCCCTGGCCATCAAGAAGGCACTGGTGTTTTACGCGGGGAAAGCCCCCAAGGGAGTGAAAACCAATTGGATTATGCATGAATATAGACTCGCTAATGTTGACAGATCCGCAGGCAACAAGAAAAATAACTTAAGGCTTGATGATTGGGTGCTATGCCGAATTTACAACAAGAAAGGAAAGATTGAGAAATACAACCATCTTGGAGCGGCGGATCACAAATCAGCATCGTCGGAGGAGGAGAATGAGAGGAAGCCGGAGGTGAAGGAGCGATTGCATATGGATACGTCGTCGGATTCGGTGGTATCGGCGGATGTGACGTGGGAGAGTAGGGAGGTGCAGAGCGAGCCAAAGTGGAATGACCTGCTTGACCAGGTCTTTGATTTCCAGTTAGGCAGTTTCGTTGATTTCTCATCGGCTGGAGATGACCCTTTTGCCCCCCAGCTCTCTCCTTGGCACCAGGACACGTTCATCACATTTTAATTCATTCTTTCCAACACAACAAGAAAAACTAAGATTGTTTAGTTTAGCCACCATGACTCATTTGGATTTAACTCGGGAACCAAGGACGATGAGATCATATATGTAAAAATAAATAAGAAGAAAAATAATCTCACCAATGCCACCTAGTTAGTTTTCATGTTACCATTGTTGGAGTGTTAAGCATCTACTCCTGTAGAAAATTCTTCCTACCATTTTTTAAAATATATTTTATTATTTAATTCAACATATTCAAGGAATGATTTCG

>AiNAC23--Araip.92BTQ

CCAAGTACTACCGCCGCCTTTATAATTTCCAGCAATCTATCCATCCATTATTATAGTTAGCTTTTGTGAAAAATAATCAACCCGAATCTTTATCTCTCTACTCAGCATCTCAAACTATATATATAGTGACAAAATAAATGGATTATGGAAGTGTAGTTATTACTATACCAAACTCAACAATTCATACACCAATTCTATGATGATGATGACTACTACTTCTACTACTGCAGATTATGAAAGCGTGAAGCAGCTTCCTCCTGGGTTTTTGTTCTCTCCAACGGATGAAGAACTTGTCCTTCACTTTCTCTATGCCAAGGCTTCTCTTTTGCCATGCCATCCCAACATCATCCCTGATCTTGATCTCTCTCTCGCTCATCCTTCCCAACTCAACGATAAAGCGTTGTCAAGCGGAAATCAATACTATTTCTTCAGCAAAGTGAAGGAAAAAAGAATAACAGAAAATGGGTATTGGAAGGAAATAGGTGAAAGTGAAGCAATATTGTCATCAACGGTTGAGAAGAAAGTAGGGACAAAGAAGAACCTTGTATTCCACATAGGAGAAGCTCCACACGGCATTGAAACCAGTTGGGTCATGCAAGAATATCATATTTGCCGATCCTCTAACATTATTTCTACAAGTAGAGCCAGAAGAAAACACGATCATCAAATTTGGAGCAAATGGGTTTTGTGCAAAGTGTATGAAAAGAAGGGGTCCGTACGAGGTGTAAACTACTGTAGCGACGATGATGACAGTGGGACAGAGCTATCTTGGCTTGACGAAATTTATCTCTCGTTGGATGATGATCTCGAAGAAATTAGCGTCTCCATTTTAGATTGAACTAGTGAAGTACCCGTGCATCGCACGGGTATGATAAGCATAAGGGAAAGTATGAGGAAACAATACACTTATTGTACAATACATACAATGTGGGTGTAATGGAAATTAAAAATAAATAATGGGTTATTAATTAATTTCATATTATTAATAATTTGAAATTTGAAATTTAAAACTATTTAGAATTAATCTTAATTATAGATTCAAATCAAATATTTAGGTTAAAAATAGAATCTCCCTGACGGCATATTCTCCTGGAGTACACCGTTGCCCCATGCGCTGATTTCCACCTTCTCCAGCAGTCAATTACCCATGCCTGCGTCCTGTTGATCGGCTTCTCCCTTCAGCTCCG

>AiNAC24--Araip.9BR1Z

ATGACGAACCTTCCTCCTGGGTTTTGCTTCTCTCCAACAGATGAAGAACTCATCCTTCACTTTCTTTATTCCAGAATTTCTCTACCATTCCATCCCAGCATCATACCGGACCTTGATCCCTCTCAACTTCATCCATGTAAGGCGTTTTCAAGTGGGAATCAACACTATTTCTTCACCAATAAAGTGAAGGAAAACAGAAGCACAGAAAATGGGTATTGGAAGGAAATAGGTTTGAGCGAACCTATAATCTCAGCTGATGCAAACAAGAAATTGGGAATCAAGAAGTATTTTGTCTTCAATCTTAACGAAGGCACAGAAACCAATTGGGTCATGCAGGAATACCATATTTCCTCATCTATGTTCCACAACCCTATTTCGTGTTATGCAAATGGAACTGCTCATCGAAGATTATTAAAACCTGATCAAAATCAGAACAATAAATGGGTTTTGTGCAGAGTGTATGAGAAGAACAAGTCACAATCACAACAAGGTGCAACTGCAAACTCCTACTATAGCGACGAGGATGATTGTGGATCAGAACTTTCATATCTAGATGAGGTTTATCTGTCACTTGATGATGATCTTGAAGTCATAAGCCCCCCTAATTAA

>AiNAC25--Araip.9MG9F

GATCCCTCTTTTGAAGTACATACTCAACCCTTCTATCTCTCACACACACACACACACAAACACAACTTTCTATCTTTCTCTTTTTAGTTTTTGTTCTATTTGTTTTATAGAACAAGAGAAAAACAAAGGAAGGAAAAGAAGATATCCATAAGTGGAATATGAATGTGTGAGAAGGATAATAAACAATAAGGAGAAATGACTGAGTGCAATGAACATGAAAACAATCATGGCAACATCATAGTGGAGGGAAGAAAAGACAGTTTAATTAGAACTTGTCCAACATGTGGTCATCACATCAAATGCCAAGATCAGTGCAATGAACATGAAAACAATCATGGCAACATCATAGTGGAGGGAAGAAAAGACAGTTTAATTAGAACTTGTCCAACATGTGGTCATCACATCAAATGCCAAGATCAGGGTGGTGGACTTCATGACTTACCTGGACTTCCAGCTGGAGTGAAGTTTGATCCAACAGATCAAGAGATTCTTGAACATTTGGAAGCAAAAGTGCGATCTGATATTCACAAGCTTCACCCTTTAATTGATGAGTTCATCCCAACTCTTGAAGGAGAGAATGGAATCTGCTATACTCATCCAGAGAACTTGCCAGGAGTAAGCAAGGATGGGTTGATCCGGCACTTCTTCCACCGGCCGTCGAAAGCATACACAACCGGAACAAGGAAGAGGAGGAAGGTGAACTCGGACGAAGAGGGAAACGAAACCCGTTGGCACAAAACAGGCAAGACCAGACCAGTCTATATTAGGGGGAAGCTGAAAGGATACAAGAAAATCCTTGTTCTCTACACAAACTATGGTGGGAAGCAAAGGAAGCCAGAGAAAACCAATTGGGTGATGCACCAATACCACCTTGGCAATGATGAAGAGGAGAAAGAAGGGGAGTTGGTTGTTTCCAAAGTGTTCTACCAAACACATCCTAGACAATGTTCTTCACTCTTGGTCAATAACAACAAAGACTCTTCAACAACACTTGTCAAGGGTAATAATAATAATGGGTTTGTTGAGTATTACCATTCAAATTTCATATCATTTGATCAAGGGGAACACCAACATAGATCTAGTGGGGCTCAAGTCGTCATTTCACATTTTCCTCTCCATGAAGCTGCTCCTAATTATCATTCTTTGAATCAAAAGGAGTAGTGTAGAAAAAATAGTAAATTACCATTTTATCTATGAAAGAATTAGCTTGCTGATAAAATGGTTTTCTGGAGATTGATAATTATTTTATGGTACTCGATTTTTTTTTTTTGTTTAACAAAATTACTTAAAGCAATCATAATTTGTTGTTGTTGAAATGGTAATTTATTTGGGGAAATAGACATATAAGGTGAAAGAGTTTGTGAAAAGAGCTGAGGAGAGGAAAATCATAAATAGGAGTTTTTTTTATATAATATTGAGGCATTATTGTAACATATTATGAAAAGTAGTTAAGAGGGCACATAAGTGA

>AiNAC26--Araip.9N5S4

ATGGAGAATATGAATAGTTTTTGTCATGTTCCCCCGGGTTTTAGATTCCACCCGACGGATGAAGAACTTGTTGATTACTACCTTAGGAAGAAGGTTAGTTCAAGGAAGATTGAGCTTGATGTAATCAAAGATGTTGACCTCTACAAAATTGAGCCATGGGACCTTCAAGAGATATGCAGGATAGGAAGAGAAGAGGAGAATGAATGGTATTTCTTTAGCCACAAGGATAAGAAGTATCCAACAGGAACAAGAACAAATAGGGCAACAGCAGCTGGGTTTTGGAAAGCAACGGGAAGAGACAAAGCTATATATTCAAAGCATGATCTCATAGGGATGAGGAAGACATTAGTCTTTTATAAAGGGAGAGCTCCTAATGGACAAAAATCGGATTGGATCATGCACGAATATCGCCTTGAAACCGATGAAAATGCCGCACCACAGGCAATATTCAAGAAGAGAGTAACAACGATGCGTAAAATGATGATGAGAGAGCATGATGAGTCTCCTAATTCTTCTTGTTGGTACGATGACCAAGAATTCATGATGATGGAATCGCCAACAAAGCAACAATCCTCTATTCTTCTTCATCAATCCACCAATAACCATTCCAATTTGATGCAGCTACCACCGTATCCTCTCATCAAGAAAGAGCTTCATCACCCATCATCATCATCATCGTCATACCCCTTCCTTCAGCTTCCACTCTTAGAGTCTCATCAACAATCTGCTGCTGCACCTTCCTCCATTTCTGAACAACTCATCATGCCACCACCAATTGGAGGAGGAGAACAAGTCCCGAGTTTTCAGTCATTCTTCAATAATGAACAACAAGAAGTAGGAGTTCTTGATTGGAGAGTTCTTGACAAGTTTGTTGCTTCACAACTTAGTCAAGATGATAATCATGCATCCTCTAATAGTATTGTACAAGATCTCACACAGGAAATTGTTATGGTGCCTCACAATGATGCTGCATCAACATCAAACTCCCTCACCTCCCCAATTGATTTGTGGAAATAG

>AiNAC27--Araip.9W6SR

CCCAACATTCCACCCTCCTCTTCCCTCACCTATTCTCTCGCTCTCTCTCATTTTTCTAGCTCCCAACGTTCCACATTAAGGGAGGGGAGAGCATTATTATTGCATCATTGGAGATCATCGCTTGGATTATTGAGGTTTCTGATAGTTTGTTAAGTGGGGTATTAGAGATCATATAATATTTAGATATTATAATGGCACCAGTTTCATTGCCTCCTGGTTTTAGGTTCCACCCTACAGACGAAGAACTGGTTGCTTATTACCTTAAAAGGAAGATTAATGGCCGTAAAATTGATCTGGAGATCATTCCTGAAGTTGATCTATACAAGTGCGAACCGTGGGACTTGCCAGGGAGGTCGTTGTTGCCGGGTAAGGATTTGGAGTGGTACTTCTTTAGCCCTCGGGACAGGAAGTATCCAAATGGGTCAAGAACTAACAGAGCAACGAAATGTGGGTACTGGAAGGCCACTGGAAAGGACAGGAAGGTAAATTCGCAGAGCCGCGCCGTAGGGATGAAGAAAACCCTTGTGTACTACCGAGGCAGGGCGCCTCACGGGTCTCGCACTGGTTGGGTCATGCACGAGTACCGTCTTCATGAGAGGGAATGCGAAACCAATGCTGCTTCTGGCTTGCAGGATGCCTATGCTCTTTGCCGTGTCTTCAAGAAGGCGGCGGTCATTATCCCCAAAGTGGCAGATCACTATGCTGCTGATAATATCATGATGATGACAACTGATTCTCAAGGAACACCCCAAGTCTTTGACACCATGCCTTGGGATCATCATATTGGGCATAATGGTAAATGCCCACACTTATCTCAAGATCCATTCCTCAATAACCTTCCATCTTCGTCATCCTCCTCATTTCCTCATTATGGAGCCCTAACTTACTCTCCATCTAAGGTGGATGTAGCACTAGAGTGTGCAAGGATGCAGCACAGTTTTTCCATGCCTCCATTGGAGGTAGTGGAGGAATTCCCTAATGTGGGAATTTCAGAGCTCAACATCATGACACGTGGCACCACTTCAATGTGTGGAGGAAGCATGAACAATAACAATGAATCGGATATCTTGCAACAGATTCTTTCACTTGCTAATGCTAATGTTTCTTCCCATGAATTCACAAATCATTCAAACCATTCACACACATTATTGGGTGGCAACAATGCAAATTATGCTGCTCCTCATCATCACGAACATGATTTTGCTTTTAATGCTGGCACAAGTTACACTAATCACGCCGTAAATGATATGAACCCCATGAGATATGAAATCCAAGATCAAAACCTAAGAACAATAGAGATTGGAGATCTTGAAAGCGAGTTCAAGAGCTTTATGGAAGAGCAAAAGACGGTTCCGATTGAGGATATATCAAGCTTCCAAACAAACATACAAGAAAATGAGGTTCAAGCAGAATCTGAGCTACACAATAGCAACAAAGAATTCAGTGAAGCTGACATTGACAATTTCTCAATGGGGTTCATCAACGATGGTGACCCAAATGAGAACTTCATCGATGATGATGACAACATTGATTATTCAAATTCCACAAGCTTTGAGGTCCTTGAAGAAACCAAGGTTAGCCATGGAATGTTTGTGGCGACTCGCCGAGTAGCCGACACATTCTTTCATCAGATTGTTCCTTCACAAACCATCAAAGTTCAACTGAATCCAGTAACAATAATGGGCAACAATTCTTCCATGGAGATGCTCAAGAATAATCAAGAGTCTTTGTTCAAGAAGCTGATGATGATGAAGTCACCAAATACATTAGCAAGTGCTATTGTATTTATCTTTGCACTATTGTTGACGCTTTGTGTTAATTTGAAGGGGCAAGTTGAAAATTATTGGGCATCAAGAAGTGATGATGACACAATTAATGTGAAGAAGAAATGTTGTTATGATGCTAATAGCAGCATGAAGAGAATGAAACAAGTAGCTCACAAGATCATATGGAATCAGCAAGAAAAATCTTGGTGTGTTGGAATTAAAAGTGGGAGAGGATTTAGTGTGGTGTTGAAGAAAATTGGTATTTTCCTCTCCATATCTTTGGCTCTTTGTACCATGTGGGTTAACCATGTTACAATTAGTCCTTAATTGACCTAGTAATTTTATTTTCTTTTAGCTTCTAACTTAATTAAGGATTTCATTATTTAGAAGGATACTTGTATTTTCTTCTCACAACAATTCTTGTATTTTACTTGGTCAATATGTTATTTAATTAAGA

>AiNAC28--Araip.A6QWC

TAACCAAAGCTTTAAACAATTTTCATCTTCAAAAAAACAAAAACAAAAATGGCTAGAAGTGCCACAATTCCATTTCCAATACTTGATTTCATTCCTGTTGGATTCAGGTTCAAGCCAACAGATGAAGAGCTTGTGAGCTATTACCTCAATCACAAGCTCCTAAATGACAATTTTCCAATCGATATTATCCCTGACATTGATCTTTGCAAGGTTGAACCTTGGCAAATTCCAGCATTATCAAAGGTAAAATCGGATGATCCAGAATGGTTTTTCTTTAGTGGACGTGATTACAAGTATGGAAAGAGCAAAAGATCAAACAGGGCAACCAAAGGAGGATATTGGAAAGCCACAGGACAAGATAGATTCATAAAGGAAAGAGGAACTATGAATGTAATTGGGAGCAAGAAGACACTTGTTTTCTATAGTGGCCGTGTTCCTAATGGTGTCAAAACCAATTGGGTTATCCATGAGTATCATGCTACTACCTTTGATGATAGCCAGAGGAATTTTGTTTTGTGTCGCTTGATGAAGAAAGTCGAGAGAAAATCCGAAGATGGAACTGATGCACAAGCCTGTGATGAGGGGGAACCTAGCACTCACATGGAAGAAGCAGATGAGAGTGTCTCAACTATGTTTGATTCGCCAGATGTGGACATGGATTCAATCTTCCACACACTGCCTCAAGACAGATCATCATCACAGCATTCTCCAGTCGGCATTGAACAGCAAGAATCCTTCCCATTCTCCCCATCTGAAAATTATTACCTTGTAAATGAAGATAGCAGTATGCATATACAATTCGAAACAAACGAAGAGAAGCAAGATGCTGAGAAATTTGCGGATTCGATTTTGGATAGTGGCAATATAGCTATGTTTGAAGAAAGACAGCAGCATCATACTTTCATGAATAATCACCTCCGCTCGGTCCCATCGATGAGGGTATGCTATGAAAGCAGTGACACAGATGCTGAAGTAGTCTCTAGACGGGCTGATTCAAGAGAATACCATGTATCAAAAATGGTTCAATCATCACATAGTGCTGCGCGCACAGATAAAACTAGAAGTATCTCTTCAGAAGACTTTTGGGGAGTGGATTCATCTTCATGTGACTCAAATGCAGATAAACCTTTTGAGATCAATTCTATTGAAATTTCTAGTCCTCCACCGGCTCTAAGTGGATCGAAAAATCAATATAATCCGAGACTATCTCAAACACATAGGAAGGTTTCAAGCAATGCAATTCCCAATCTTGAGGATAAGAAGAAATTGACCACTGTGGAACAATCAAGAAGAGATCAAGAAAAAGCTCGAAAAACTAGTCCAGGAAAGAAGTTAGAAACCAGAAGCTCTGATGTTAATAGAATTGGTAGTTTCATCCACCTAGAGCCGTGTTCGTCGAGCGAAAGCCTGACTCCACGAGCGGTATACCTTGTTAATGTAGTTATTGGGATTTTGTTGCTTCTAGCCATTAGTTGGGATGTGCTATCTTGTTAGAGAAGTGTTGCCATGCAAGATTTGTTTTAGGAGTTTCATAATTATATACATAGTTCAAGTAATAATATAGTTTACATAGATCTTGCACTGATGCAAAGATTACTGCACCTTTCTTATCATAATGAAATTTATGCTTGTGAATTGTGATTAGTGGTAACTTCATATTGTCATTAATTCTATAATTGCAGTCTTTAAGTCACAAAATTCATTTAATGTTAGAACTAGTTGAGTTCTAGACTCTAGTGAAAAGATTCCTTTTTTCCATTCAATAAATGTTTGATTAGCTTGATTCCCAAAATGTGCTGACTTCAAATTCTCCATTTAGTAGAAACAGATCAAAGTCAATGTTCCAAAAGAAGAAAATTTGATACAAAAGTGCATAAAGTCAAAAGCAGGTTACACACCATGTGACAGTAGCAGTGTTACTTTGAATAGGATCTTTGTTTCATTTTAC

>AiNAC29--Araip.AVV74

CGGTTCTATGCACCTAAAAAACCAGTATTTAAGGATTCTGTTTCTCATAACATAGATCTTTGTTTTGAGTGTCTCTCAATTGCATTTTTTTCCATCAAATTCCTCAAAAAAGAAAAATTAAACCCTCCACACCAAAAAAGAAGAACTGCTCAATTTATTCACTCTCTTAACTTGTCTTCACACCCATGATTTAGGCATCAAAAAGTGTAGCCACCCTAACCCCAAAAGCTTCCTTTTTTAACTCCTTGCAACACACAAGTGTGAAATTGTCCACTACCCTTTAAAGGTTGGTTTCTCTGCATTTAGCCCACTTGAATTCCGGCGCCGAATGTTCGATAGCTGCCGAGTTTAACCGACATATTTGTCGTGTTTCGATGCTTTTTATGAACCAAAGGAGCCTAGCATAGAGATGGAATCAATGGAGAATGTTAGAATGCAAAGAGAGAAAGATCAGAAGTTCGAATTGCCGTCCGGCTTTCGATTTCATCCCACCGATGTAGAGCTCATAAATTACTACCTTGTTAAGAAGGTTCTTGATGATAAGCACTTCTGTTCTATAGCAATTGCTGATGCTGATATGAACAAGTCTGAGCCATGGGATTTACCCGGTTTAGCGAAAATGGGCGAAACGGAATGGTATTTTTTCTCTATGAAGGATAGAAAATACCCAACTGGCCAAAGGACTAATAGGGCGACGGAGGCCGGTTATTGGAAGGCCACAGGCAAAGACAAGGAGATATCAAAGGAGAATTCAAAGATTGGGATGAAGAAGACCCTTGTTTTCTACAAAGGAAGAGCTCCAAGAGGTGAAAAGACTAATTGGGTCATGCATGAATATAGATTGGAAGGGAACAAATCTGTTTATAATCTGTCACAACCTGAAAGAGGTGAATGGGTTATATGCAGAGTATTTGAGAAGGGCAATCATGGAAAAAGACTGAATATTGCAAAGTTGGAGAGGCTCAACTCTTTGGGAGAGGAACCATTACCATTGCCAAAACCTACTCCTTTGATGCCTCCATTGATGGATTCTTCATCATCGAGAACCACCCCCGGCGAGTTATCTCAGGCGACGTGCTACTCCTCTGATCCGAATCAAGCCGAAGTCCGGAACAATTTGCATGATGACATAGTTGAAAGCAGGGAAACTCCTATCTTGAACTTTTCCCCTGCTTCCATCAATGAAGAATTTTTTCAGATTCCCAACCAAATTGAGAAACCGGATTGTTATACTCCGCCTCAAGAAAACAACGGATCAATTGCAAGGCAGAATCAGAAATCAGAGTTTGATGCTGATATATCATCTTTGATTTACAACAATGACATGTTTTACAGGTTCTTTGGGAACCAAGAACATTCATCTTCAGCTTCTGCAGACATTTGCAACCTATGGAATTACTAGATAAGGTTGAAGGATATTAAATAATATTATTACTCTTTCTTTTTTTTTTTTAAAGACAAATTAAAGTAAATATGTCATTTATATCATACCTTTAAATTCTAAATTGATTACCTGCAAATGCATGAACTTATTATTAGGTTGGTAACTTTAATTGAAAAAAAAATGG

>AiNAC30--Araip.AWF0A

ATGGAAGAGCTAGCGTGTGAGCTGAGTGATCATGAAAAGAGAAACGCTCAAGGTTTGCCACCGGGTTTCAGGTTTCACCCAACTGATCAAGAACTCATTACCTTCTATTTGGCTTCCAAGGTCTTCAATAATACAAATGCTACTACTGCTACTACTACTACTACTCATGTCAACTTTGTGGAGGTTGATCTCAATCGATGCGAGCCATGGGAACTTCCAGAAGTGGCAAAGATGGGGGAGAGAGAGTGGTATCTGTACAGTGTGAGAGACAGAAAATACCCAACGGGCCTCAGAACTAACAGAGCAACCGCTGCTGGGTACTGGAAGGCTACCGGCAAGGACAAGCAAGTCTACGGCGGCGGTGGCCTTGTTGGGATGAAGAAGACGTTGGTGTTCTACAAAGGGAGGGCCCCCCGCGGTCAGAAGACTAAATGGGTAATGCATGAGTTCCGGTTGGACCCTCACAGCTCTCCTTCCCTCTCTAAGCATCAGCAACAACACGATCCATTATTATTATTCCAAACCCCATCATCCCTGTTCAATGACCATATCTCCCACTCTCATAATCATAATCAAAACCTCCTCTCGCCATTGCTTCATCCTTTCCCAATCCCTGAAGAAACCACTAAAACCAGATCATCAACAATTAACAGCAACCATTACCCTCCACCACCACCTTCCTCCCAACACTCGCTTAAGCTCAACAAGTCTACTAAATTAACAAAAACAGTGCCTCCTTCTCCATCATTCTTCCAATACCAACAGCTTCTAGAAGATTATCCCAACTTATTGCATTGGATCGACAGTGGTAATAACAATAATAATAACTGCAACGCTAATAATACTGCTAGTAGTGTTGAGATAATGGATGCTGCTGCTGCTGGCTTGATAGCATTCTCATCAGGAGGACCTTCACCTACTCCTAATAATAATAATAATAATAATAATAACAATGCTGAAATAATGATGATGTCTTCTTCTTCGGCTTCTATGCTGCACATACTCGACGATGCTCCTCTTGGGATTCAATCTTGGCCTCATCATCATCATCATCACCTTCTGTAA

>AiNAC31--Araip.CC7W1

ATGCCAGAAAGCATGAGTATATCAGTGAATGGACAATCTCAAGTTCCACCTGGATTCAGGTTCCATCCAACTGAGGAAGAACTCCTTCAATACTACTTAAGGAAGAAGGTCTCTTATGAGAAGATTGATCTTGATGTTATTCGTGATGTTGATCTCAACAAGCTTGAACCATGGGACATACAAGAGAAATGTAAGATAGGAACCACCCCACAAAATGATTGGTACTTCTTCAGCCACAAAGACAAGAAGTACCCGACCGGAACCCGGACGAATAGAGCGACCGCGGCCGGGTTCTGGAAGGCCACCGGCCGTGACAAGGTGATATATAGCAACGGGAAGAGGATTGGAATGAGGAAGACGTTGGTATTCTACAAAGGAAGAGCCCCTCATGGCCAAAAATCTGATTGGATCATGCATGAGTATAGGCTTGATGATAACACCACCAACGATGCCAATATTGTTTCAAATGTGATGGGAGATGCAGCACAAGAAGAAGGGTGGGTGGTGTGTAGAATATTCAAGAAGAAGAACCATCTAAAAACCCTAGATAGTCCTTTAACCTCTTCCATCTCTGGCGACGGAGGTAGGAGGAGCCACCACCACCACCACCTATTCGACTCGTGCGACGAGGGCGCCTTAGAGCAAATTCTCCAACAAATGGGAAGAGGTGGTGGTGGTGGTTGCAAGGAAGAGATCAACAACTATGATCAATCTAACAACAACAACAACAATAATAACAACAACTATGGTGGATCATCATCGTTAACAACAAGGTATGCAAGACCTTTTGACACAATCAACAACAATGTTGATAGCAGGTTCTTGAAGCTCCCAAGCCTAGAGAGTCCAAAATCAACAAGCATGGATCATAACAATAATAACAACAATGATAATGATGATAGCAATGAAAATAATGGGTACCATCCTATTATTCCAGTTGAGATGGTAACTGAAAACGAAGGGTCATTCACATGTGACAATCCCAACAACATGTTTCATCATCACCATTTGGGTGGTGGCGGTGGCGGCGGCAGCGACGGCGGTGGCGGTCTTACAAATTGGGTAGCGCTAGATAGGCTTGTTGCTTCTCAGCTTAACGGTCAGACCGAAGCTTCTAGACAACTCTCTTGCTTCAATGACCCCACCATGGGGTATGGCACTGGAAATCATGATCTTCTATTTCCAACCGTCAGATCTACTTCATCGTTGACGTCATCGTCAGCGTCAATAAATCCAAGGGCTGTTATTAGTGCGGGTGCAGGTGCATACATTTCTCCAGGCGCACAGGATTATACCACCACAAGCGAGATTGACCTGTGGAACTTTGCTAGATCCACTTCTTCGTTATTGTCATCCTCTGAGCCATTGTGCCACGTGTCCAAC

>AiNAC32--Araip.D25HB

ATCTCTTAAACCTAATCATCAATAATTCAATCAAACCTTGGGAGTTTGCAACATCTTTCTCTCAGACCTATTCTTTTCTCTCTTCTCCCCTTCTTCCTAGCAAGCAGGCTCTCACTATATATTAATTTAATTTGTTGAAATTAAATAGAAAAAAGAATAATGAGCAACATAAGCTTGGTAGAGGCAAGGCTTCCACCAGGGTTCAGATTTCATCCAAAAGATGAAGAGCTTGTGTGTGATTACTTGATGAAGAAGTTCACGCACAATGAATCCCTTCTCATGATTGATGTCGACCTCAACAAGTGTGAGCCATGGGATATTCCTGAAACAGCATGTGTGGGAGGGAAGGAGTGGTACTTCTACACACAGAGAGACAGAAAGTATGCAACGGGGCTGCGTACAAACAGAGCAACGGCATCAGGATATTGGAAGGCCACTGGCAAGGACAGGCCTATCCTTAGGAAGGGCAGCCTTGTTGGTATGCGAAAGACTCTTGTCTTCTATCAAGGTCGGGCTCCCAAAGGCCGTAAGACTGAGTGGGTCATGCATGAGTTTCGCATTGAACCTCCTCTTCCTCCCCCCAACACTTCTTCTAAGGAAGATTGGGTGTTGTGTAGGGTGTTTTACAAGAACAGAGAAGTTGGTGGCAAACCTAATAGCATGGGAAGCTGCTATGATGACACAGGCTCTTCATCTCTTCCAGCATTAATGGATTCTTTCATCAGCTTTGACCAACAACAACAACCTCAAACCCATCTTCATGCTGATGAGTATGAGCAAGTGCCCTGCTTCTCCATTTTCTCTCACACCCAAACAAGCCCTATTTTCAACCACATAATGGAGCCTAAGTTATTCCCTACCAACAACAACAATAATAATGCAACTTTATATGGTGGAGGAGGAACTACTACAACATCCAATTTGGGTTCTTGCTTAGACCCTTTTTCATGTGATAGGAAAGAAGATTGGGTGTTGTGTAGGGTGTTTTACAAGAACAGAGAAGTTGGTGGCAAACCTAATAGCATGGGAAGCTGCTATGATGACACAGGCTCTTCATCTCTTCCAGCATTAATGGATTCTTTCATCAGCTTTGACCAACAACAACAACCTCAAACCCATCTTCATGCTGATGAGTATGAGCAAGTGCCCTGCTTCTCCATTTTCTCTCACACCCAAACAAGCCCTATTTTCAACCACATAATGGAGCCTAAGTTATTCCCTACCAACAACAACAATAATAATGCAACTTTATATGGTGGAGGAGGAACTACTACAACATCCAATTTGGGTTCTTGCTTAGACCCTTTTTCATGTGATAGGAAAGCTTTGTAA

>AiNAC33--Araip.D7N1Q

ATGGACAAGGATACTAGTTTGGAAATCCATCTCCCTCCTGGATTTAGATTCCACCCTTCTGATGAAGAGTTAATTGTTCACTATCTAAGAAACAAAGTCACTTCTTCACCACTTCCTGCCTCATTCATAGCAGAGATAGACCTCTACAAGTTCAATCCATGGGAGCTTCCAAGGAAGTACCCAAATGGAGTGAGACCAAACAGAGCAGCTGGTGCAGGTTACTGGAAGGCCACTGGAACTGACAAACCAATTATCACGTCATGTGGTATGAAGAGCATTGGAGTGAAGAAAGCCCTTGTCTTCTACAAGGGACGTCCCCCAAAGGGATCCAAAACTGATTGGATCATGCATGAGTATAGGTTGCATGATTCACTCCTCTCAAATTCTCACAAAAGAGGCTCCATGAGAGTAGGTCAACTGCATCCATGA

>AiNAC34--Araip.DEH65

CCATTCCCTGAACGAACCAACATTTCTTTCTTTTTCCTTTTAATTACTTCTCTTTTTGAAGCTAAGGATCCTCGGGATCTATCTCATCACCTTCATCATTCTTGAGTGCACAAGGCCGTGATCCACTTCAATTCCCGAGGCTCTCATTAATTACCACTTTTTCTGTTCTCTTTTCCCCATCCTGTTCATTCCATTGCTGTTTTTTGGTGCAATTTCTGGAAGTAGCTCTTACAAAGACTTGGGTTATATATATTGATTTAAAAGGATTCTTATCGATAGAGAAATTGTACTACGTACAAGAAAAGTTTGGTTAATTTCATATACAACAGATCTACGTACAGGGATTCTACTTGGAGTTCTTAGGTGCAGGATATATCAAGCTAAGGAGGTTTGAAATGAACGCATTTTCCCATGTTCCTCCTGGGTTTCGGTTCCATCCGACGGATGAAGAACTTGTTGATTACTACCTAAGGAAAAAGATAGCTTCGAAAAGGATTGATCTGGATGTGATAAAAGATGTGGATCTCTATAAGATTGAGCCATGGGATCTTCAAGAAATATGCAAAATAGGAAGCGAGGATGAAAATGAATGGTACTTCTTTAGCCATAAAGATAAGAAGTACCCAACAGGGACTCGCACCAATAGAGCTACAAAGGCAGGATTCTGGAAAGCCACGGGAAGAGACAAAGCCATATACTCAAGCTCAAGCCATTGCCTGGTTGGTATGAGAAAGACTCTTGTGTTCTACAAAGGACGAGCTCCCAATGGCCTCAAATCAAACTGGATCATGCACGAGTATCGTCTTGACTCCAATCAGGAAGATGGCTGGGTTGTGTGCAGAGTCTTCAAGAAACGGATGCCCACGCTACGCAACGTGGTAGACTATGATGATCAACTTCCCTTCATGCAAGGATCTCCATCCACTCACTATCCCTGCAAGCACGACCTTCATCAATTCCAATACAACACCCATGATGCTTTTCTCCAACTTCCACACCTTGAGAGCCCTAATCAAGTTTTGAGTTCACCCTATGCCTACGCCGAAAACAACAACAACAATAATGGAACTTTGCAGTCCTATTCATCTGAACGCATTCAGCAACAACTTCACTTGCTTTATGGTAGCAATATTGAGCAAGCAGTAGTGGTGGACCAAGTCACGGATTGGAGAGTGCTTGACAAATTTGTTGCTTCTCAACTCATGAGTCAAGATCAAGATCAGGCTTCCAAGGAAACCTGCAGCGTGGCTGATGAACAACATGTTGCTACTACTGTGCTTCCAAATGGATCCACCAAGCAGGATGACTATGTTTCAACGTCTGCCTCCAGTAACTGCGATATTCACCTGTGGAATTGA

>AiNAC35--Araip.DL86S

GAGGAGATTTACTACGGTCTGATTTTCGAGAATCTAGCTTTTTGATTATCTGAAGAAGATTAAAACAAAATGGGAATTCAAGAGAAAGACCCTCTCTCGCAATTGAGTTTACCGCCGGGTTTCCGATTTTATCCGACGGACGAGGAGCTTCTCGTTCAGTATCTGTGCCGCAAGGTTGCTGGCCACCATTTCTCCCTGGAAATCATTGGCGAAATTGATTTGTATAAGTTCGACCCTTGGGTTCTTCCAAGTAAGGCAATTTTCGGCGAGAAAGAATGGTACTTCTTTAGTCCGAGGGATAGGAAGTATCCGAATGGTTCGCGACCCAATCGGGTAGCCGGGTCGGGTTACTGGAAAGCCACCGGGACCGATAAGACTATCACGACCGAAGGAAGGAAAGTTGGTATCAAGAAAGCTCTGGTTTTCTACATTGGTAAGGCACCCAAAGGCACCAAAACAAACTGGATCATGCACGAGTATCGCCTCCTAGACTCTACCCGTAAGAACGGGAGCACCAAGCTTGACGATTGGGTTCTGTGCCGGATATACAAGAAGAATTCAAGCGCACAGCAGAAGGTACCAAACGGCGTCGTTTCGAGTAGCGAGCAATATGCCACGCAATACAGCAACGGATCTTCTTCAAACTCCTCTTCCTCCCACCTCGACGAGGTGCTCGAGTCCCTGCCGGAGATCGACGACCGTTGCTTCGCCTTGCCACGTGTCAACTCCTTAAGAGCGCTGCAGCAGCAGCGCCATCACCAAGAAGACACCAAGGTCGGCCTACTCCAACAGCAACAGCAACAGGGTCTCGTAGCCGGCACCGGTAGTTTCTTGGACTGGGCTTCCGGGCCGGGGATTCTGAACGATTTGGGCCAGGCCCAGCAGGGGATTGTTAACTACGGAAATGACCTCTTTGTCCCTTCAGTGTGCCACGTGGATTCCAATTTGGTGCCAGCAAAGATAGAAGAGGAGGTTCAGAGCGGTGTGAAGACTCAATCCGCATTCTTTCAGCAGGGACCGAACCCGAATGACTTCACACAAGCATTCTCAAACCAATTAGATCCTTACGGGTTTAGTAGGTACTCGGTTCAACCGGTTGGGTTCGGGTTCAGGCAATGAACCAGGGTGAGGGACTAGATGTTAAATAACTAAAAGAAAGTGTATTGAATTTTTGACTATTTGTTGAGGTGCAATTGGGGGGTGTAAATAGGGATTCTTTTGCAAGATTCCAAGAAAGAAATGTCTTGCATTGAGAAAAGGGTTGGGTTGTAATTTTCTTTCTTGGTGGTCCTCTTTTTCGGCTGGAGATAGAGTGGAGAAAAAAAGAATTCAATGCATCCAAAGGTGGAAAAAAAGGGGGGAAAAATTGTGATTGCACTAAATTACTAGTCTTTGGAATTTTAATTAGTTTGAAGATGTACACAATTTTTGGCTGCACCGGCCGTGGTGTGAGCCACATGAATCACGTTTTGTACTTTGGAAGATTTAGCATCAATTGATATACAATTGTTTACAATTCCAATGTTAGGTTGCTTTTCAAATGTTTGGTGTGGTCTTTTTAGCTAATAACCACAACATTGGTAGCCACAAACTCAATTCGTGTTCATGGGATAGGACTATTCTATAAGAGTATTTTCAATGCA

>AiNAC36--Araip.DR280

ATGCCAGGTTTCAGATTTCACCCAACAGATGAAGAGATTGTTGGTTTTTATCTAAAAAGAAAAATTCAGCAAAAATCTCTTCCTATTGAATTGATCAAGCAAGTTGATATCTATAAGTATGAGCCATGGGACCTTCCAAATGAAAAAATATGGCCCAACAGAGTGACAAGATGTGGGTTTTGGAAGGCCACTGGAACGGACAGGCCCATTTACTCATCTGAGGCCCAATCCATTATTGGTTTGAAGAAATCACTTGTTTTCTACAGAGGCAGAGCTGCTAAGGGTTTCAAAACTGATTGGATGATGCATGAGTTTAGGCTCCCTTCTCTTTCTTCTGATTCAGCCAAGAAATGCTCTGACAAAACTACCCCTGCTTCTGATTCATGGGCAATATGTAGGATATTCAAGAAAACAAACACAATGTCCATGGCACAAAAAGCCTCATTATCTCATCATCCTTATAATTGGAATCATCATAATCAATTATTTGATGATATACTCACACATCAACAACACCAACACCCTATTATTCCAAACTCCAACAACAACTTCATCTTCTACAATTCCAATTCTACCCTTGAACCCACAAAAGAAATTGATGCTACTACTACTAGTAGCTCCATTGTTATTTCTTCCAACATAGGCCTTCATGAAGATCCAAATCATCATCATTACAATAATAATAGTAGTGGGTTCTCATCTTCTTCAATTATGATGATGCAACCAAACATCATGGCAACCTCAGATGATGATGATTCAGGTGTAATTACAACAATTGCTGGCTTCCCATTCAATTTGCCTCCAAATGATGATGATGCTGCTTGGAATAATAATATTAAGCCTAATACTACTCTGCCATGGGACTACTTATCAGACATGTCCACTACCTATTCCACTAATAAATCTTACACTTAAAAAAAAATTATATACCATAGTTAATTAAAACCAAAATTTGGATTTTATTTTG

>AiNAC37--Araip.E0NQ0

GTGTTGAATACATGCGTCATAAGCATTTCTTTTTTTTTTAATGTTATATTTACAAAATTAAAGGAAAATTACACGTGTAGAAGTACTTGGAAGTTTCAGAGAAAGAGACAAATTCTCAAACATAAGAACAGGAACATAAATATATGATTTTGAATCTCCGTCTCTCTACTTGCATCACCAGCCACTTTCTCTTTCTCTCTCTATGGCCGGATCATCCTGGTTGGTAGACAAAAGTAGAATTGCAACCAAAATAAAGAGTGCATCTGGAGCAAGTGGGAAAGTTTTATGGAAAAGCAATCCTACCAGAACTTGTCCGAATTGTCAACATGTTATTGATAACAGTGATGTTGCGTTCACGATCACTTGTTCGCCATCACCCTCGTCACATTTGTGTTTTCATTGTTGTGCTTGTGTTCCATTCATTTACTCTAGTTTTCATGATTTCTTTGATCATGATTCTGTTACTCTTGCTGGAGCAACCAATTTCATAGGAAGTGTTGTATTGTGGATTCTAATATTTCTCTACGAAGCTAGAGACATGAAAATCTTTGAAAGGGCTACAGTGGCACAAGAGTGGCCTGGATTACCAAAAGGTGTGAAATTTGATCCATCTGATCAAGAAATAATATCGCACTTGCTTGCAAAAGTTGGTGCAGCAGGTTCAGAGCCTCACCCTTTCATTGATGAATTTATTGCTACTCTTGAAGTGGATGATGGAATTTGTTATACACATCCTAAACATTTACCAGGTGTCAAGCAAGATGGTAGTGCTACACACTTTTTCCACAGATCAATCAAGGCTTATAATACCGGCAATCGAAAGCGTCGGAAAATAAATGACCAGGACTCTGGCGATGTCCGTTGGCACAAGACTGGAAAAACTAAACCTGTCGTCTCGGACGGGGTTCAGAGAGGCTGTAAAAAGATTATGGTTCTATATATGACTTCAGTTAGAGGAGTAAAAGCTGAGAAAACTAACTGGGTTATGCATCAATATCACCTCGGGACAGACGAAGATGAAAAGGAAGGAGAGTATGTTATCTCTAAAGTGTTTTACCAGCAACAAGTTAAGTTTGGTGAAAAAGATGATCATGATGTTCCTGGAACCAATGAAGCAACTGTTGTGAAAGATGATCCAGTCACTTCCAAATCTCTGACTTCGGAACCTCCTCATAGTGAAAAGCAATGTTCAGATCTCGACATAGGAGAAAAAACACATCAGATTCTTCAGGGTCCTCAGACAGATTGTGTAGAAGACATTCAAGTCGAGTGTGAAGAGATTGTAAAAACTGATGTATCCATGGCAGATGCTCAAAATAATGAAGGAATGGATAATGTAGAAAATAATGCTGACGGAGAACAAAAATGGTGGGACAGTGAGTCACAGAATTTGTTAGATTCACAACAACTTGTTGAAGCATTGGCCTTGTGTGATGATCTCCTCCATAGCCAGTGTTCCAATAAGGATGATGAAAATGAAGAACACAAGGAGCACTTGAGTCTTTCCATCTATGCTCATCTAGGACCAGAGCATCTGAAGAAAGATCTTGAAGAGTGCCAAAACCTTACTCTTGATCCTGCAAACGTAGAGCTCGAGACACCACCTTCAGAGTTTCGACTAAGTCAGCTGGAATTCGGTTCACAGGATAGCTTTGTTTCCTTTAGCGGCGGCAAGGCAGTCGATTAATCCGGCCCTTCTTCGGCTAATATGGGAAACAAACACATTTCTAACTACTTTTGTTGTAACATTTGATCAACAGTGGTGTAATTAATACTGTCTTTTGGTGTGGTGAAAGCCGTGGTATGTTCCTTACAAGTTCTCTAGAAAGTTGCATGTGGACCTGACTTGTCCAAAAATCACTACATGTTTTTATTTCACTCTGAAAGTAACATATGAAGATGAACATGTACATTTATAATTTTACATCCCTATCTTGAAATGTGTTGTCTTGTCTCCCATAAAAATCTATTGTATGTATATTTGGTGGCGACAGATTCATGAATGC

>AiNAC38--Araip.F5AGL

TCAGAGCAATGGAAAAAGTGGCATCATTGGTGTTGAAGGAAGAGGAGCAGATGGATTTACCTCCGGGGTTTCGGTTTCACCCAACTGATGAAGAGCTCATAACCCATTACCTATACAAGAAAGTTATTGACACCAACTTCGCCGCAAGAGCCATTGGCGAAGTCGACCTTAACCGCTGCGAGCCTTGGGATTTGCCATGGAAAGCGAAAATGGGAGAGAAAGAGTGGTACTTCTTCTGCGTGAGGGATAGGAAGTATCCGACCGGGTTGAGGACAAACCGGGCCACGGAGTCTGGGTACTGGAAGGCAACCGGGAAAGACAAGGAGATTTTTCGAGGCAAATCTCTCGTCGGAATGAAGAAAACGCTTGTCTTCTACAAAGGAAGAGCACCTAAAGGAGAGAAAACAAACTGGGTCATGCACGAGTTCAGGCTGGAGGGAAAATTCTCCATCCATAACCTCCCAAAAACCGCAAAGAACGAGTGGGTGATTTGCAGGGTGTTTCGGAAGAGTTCAGCTGGCAAGAAGGTTCACATCTCTGGAATCATGAGGCTCGACACTTTCCGAACTGAATTGGATTCTTCCGGTCTACCTCCCTTGACGGAGACCTCGCCCTCTTTCGACACCATCCATGACGAATCGCCTTACGTGCCCTGCTTCTCCAATCCAATTGATGTTCCAAGAAACCAAGCCGCAGGCGGAAGCGGAGGAGGAGGAGGAGTCTTTGGTGGTTCCTTCCCCAACAACTCCTCCTCTTCTGTTTCCGCCTACGCGGTTTCTTCCAACATTCTCCCAAGGATGCCGATTTGCGGCGGTTCCTTGTACTCTACTCAACATCAAGATCAGAGTATCCTGAGGGCGTTGTATGAATCGAACGAAAGGGAAATGATCAGTGTGTCACAGGAAACAGGCCTCACTACTGAGATGAACGTGGAAACCAATTCCGTGGTGTCCAATTTTGATATAGGGAGGGCTCACTTTGAGAGCTTGTGGAATTACTGA

>AiNAC39--Araip.F8I62

GTTTCAGGTTCCATCCTACGGATGAGGAGCTTGTTATCTATTACCTCAAGCGCAAGGTTTGTGGCAAAAGCTTCCGATTTGATGCAATTTCTGAGGTTGACATCTACAGGAGCGAACCCTGGGACCTTGCAGTCGAGGTTGAAGACTAGGGACCAAGAATGGTACTTCTTTAGTGCACTGGACAAGAAGTATGGCAATGGTGGGAGGATGAACAGGGCCACAAGCAAAGGATACTGGAAGGCTACAGGGAACGATCGTCCGGTTAAGCATGAACAAAGGACCGTGGGGTTGAAGAAAACTCTGGTGTTCCATAGTGGAAGAGCCCCAGATGGTAAGAGGACCAATTGGGTCATGCATGAGTACCGACTCGTCGACGAAGAGCTGGAGAGGGCTAGGTCTGGATCCTCTCAGCCTCAGAAGGATGCATATGTTTTGTGTAGAGTTTTTCACAAAAATAACATAGGACCTCCGAATGGGCAACGTTATGCACCTTTCGTTGAAGAGGAGTGGGATGATGCATCGGCATTGGTTCCTGGGGCAGAACCTGTAGAGGATGTTACCGTTACTGTTGCTCATCCTCTACGCATTGAAAGCAACGGTCGCACTTTATGCAGCGACAGGAGAAACAATGTTGCACAGGATACTCAATCTAACAACAAAGTTCCATTTGATGTGAACAAGCTTCCCATTGAGACTCAAAGTCTGCTAGCTGTCTGCAAAAGGGAGAGTATGGCCGAGTTTCCATCACCTGAAAAGGAGGATAACTCGAAGCGTCAGATTGATGAGTATCCTTTGCCACAAACAGAAAACACCAAGCCTATCTCCCAAATATACAAAAGGAGGAGGCATTATTTGAATGTCAACCATTCAAATGTCAACGGAGATTCAGTCCGAACCATCCAAGAACCGCCATGTTCATCAACAATAACCACCGCCGCAACGACGCTCCCGACGGCCACCACCACAGCCTCCACTGCGATAACCAACGTTGCACCCAAAAAACATTTCTTGTCTGCACTGGTGGAGTTTTCCTTGATGGAATCCCTCGAATCGAAGGGAAATCCATCCGTTCAACCACCAGAGTTTGATGATGCTTCCTTAGAGGCATCCGTGCCGCCAAATTGTGTTAAGCTCATCAAACGCATGCAGGGCGAGATTTACAAACTTTCCGAGGAAAGGGAGACTATGAGGTTTGAGATGATGAGCGCACAAGCAATGATTAACATGCTCGAGTCGCGCATTGAAATTTTGAGCAAAGAAAATGAGGAACTGAAGAGCATGATTAACAACAATCCTTAGGGGATTAGCGCAGTGGCCATGTAATCTTGCTTATTGCAACAGAGTGATGGTGCTCTCAGTCTACCTGATCTCTTGTTATCTTTAAAGAGTTAAGAACTGTAGTCTTCTTCATTTGGCTGAACTTTCTGAGTGGTATTTAAACTTTGCCATTTGTGTAGCCTTGTAGCTTATGACTTGTTACCCTTGTTCATCAATGGCTATGTTAGTCTCTCCTTCCTTTAAGGCTTGTCCTTTCTTGTGAATGAATAGCAACTCAATTAGTTAGGCCATTCTGAACAGATTTTTCTCATAGAGGGGTTTGGCAAGTTGGGAATGGATCCTGTGCATAGATTTCACAAGATGAGT

>AiNAC40--Araip.FR0NA

GGAATAATAATAATTATGGGGAGCAAGAAGAGAGATACTTGTTCAGCTCAAAGGAAGTTAAGTATAGAAACGGTAACCGAATGAACAGAATAACGAAATCTGGATATTGGAAAGCAACTGGATCAGACAAAAGAATAATTTCAACATCATGTAATAATAATAATAATAGTAATATTGTTGGGATAAGAAAAACTCTTGTATTCTATCATGGAAAATCTCCAAATGGCTCTAGAACTCATTGGATCATGCGTGAGTATCGACTTGTCACTACTCCTTCTAATTCATCCCAGAAGTATGTAGAAGACTTAGGGAATTGGGTTCTTTGCCGCATATTCAAGAAGAAAAGAAGCATAGAAAGTCAACATCACATGGTCAACAACAAAATTAATAATGTTGTCGAGATATTGATAATTTTTCACAATGTTACATGA

>AiNAC41--Araip.FRS32

ATGGGGCTTCGAGACATAGGAGCATCACTTCCACCAGGGTTCAGATTCTACCCAAGTGATGAGGAATTGGTTTGCCATTATCTTTATAAGAAGATCACAAACGAGCAACTTCTTAAAGGCACTCTTGTTGAGATTGATTTACACATATGCGAGCCATGGCAGCTTCCAGAGGTGGCAAAGCTAAACGCAAATGAATGGTACTTCTTCAGCTTCAGAGACCGGAAATATGCGACGGGGTTCAGAACGAACAGAGCGACGACAAGTGGTTATTGGAAAGCAACGGGGAAGGATCGAACGGTGCAGGATCCACTGACACAAGAGGTTGTAGGGATGCGCAAGACTCTGGTGTTCTACCGGAACAGAGCTCCCAATGGCATCAAAACTGGCTGGATCATGCATGAGTTTCGCTTGGAGACCCCACACATGCCCCCTAAGGAAGATTGGGTGTTGTGTAGAGTGTTCCACAAAAGCAAAGAAGAAAACAGTGCCAAACTTATCATGTATGATTCCATTTCCACACATCATCAATCATCAAACCCTATGGCATTGGTATCAACCCATCATTTGAACCCCATCAACAACCATAATAACACTTATCATGCCATGAATAATTTCCTTCATCACTTCTCATCATCAAGGGATGATAGTCAAACAAATAACGCCAATAATAATAATAGCAGTGTTACTCAAATTAGTCCCAAGGGTTATGATGGATATGGCTTCATATGGGATCACATGGATCTTGAAGATGGTGGTGTGCCCTCATCAGACTTCCAG

>AiNAC42--Araip.G3ZLR

ATGCAGAAGTAGTACAAGTAGATGGAATCATCGTGTGTCCCACCTGGTTTTCGGTTCCACCCAACGGATGAAGAGCTTGTTGGTTATTATCTGAGGAAGAAAGTGGCATCTCAGAAGATAGACCTTGACGTTATCAGAGAGATCGATCTCTATCGTATTGAACCCTGGGATCTCCAAGAGAGATGTAGGATCGGGTATGAAGAGCAGAACGAGTGGTACTTCTTCAGCCACAAAGACAAGAAGTATCCGACGGGGACTCGAACGAACAGGGCCACCATGGCGGGGTTCTGGAAGGCCACCGGAAGAGACAAGGCAGTGTACGACAAGGCGAAGCTGATCGGGATGAGGAAGACTCTCGTGTTCTACAAAGGGAGAGCCCCTAACGGCCAGAAAACAGACTGGATCATGCACGAGTACAGACTTGAATCCGATGAAAACGGACCCCCTCAGGCAAGCCTTCTAGATTACTTGTATTATGTGTATGAGGAAGGGTGGGTTGTTTGTAGAGCATTCAAGAAAAGGACGACAAACGGGCAAACGAAGACTATGGAAGGATGGGATTCAAGCTACTTGTACGAGGAAGGGAGCGGCGGCGGTGGCGGCCCGAACATGCATGCAAATATAGCAGCAGAGCAATTTGTACAGCTTCCACAGCTTGAGAGCCCAAGTTTGCCGCTAGTTAAGAGGCCAACAACAAGCACAATGGCACTAGTCTCAGAAAGCAACGAAGATCATAACATGTTATCGAAGAAAGTAGTGACTGATTGGAGGGATCTTGATAAGTTTGTGGCATCTCAACTGAGTCATGGAGGAGACAGTAGTAGGCACGAAACTGAAACCGATGATGCAGCAGTGCTCCCAAGCTTTATGGATAATAACAACCATGACAACAGTGATAGCATCTCGGACATGTTGATGATGAGCATGAGCCCCTTTCTAAACACAAGCTCTGACTGTGATATTGGGATATGCGTCTTCGAAAATTAA

>AiNAC43--Araip.G88UP

GGGAGCTGGTGATCATCGAAAGGGCCGGGTCTGGAAAAATCAAAAGAAATGACATGGTGCAATAGATCATCAGTTGTGGAGGGGGGAATCGAAATAATCAACCACCCCAATCTCAATATTATTGCAATCCCTAGAAATAGTAATGACAATAATAATAGTGTTATTAGTGTTAATCACGCAAATACTCCTCCTAAACCAACCGAAATCCGAGCCGTTACTTGCCCCTCTTGTGGTCATCACATTCAAATACAACAAGATCAGGGTGGGGGAATTCAAGACTTGCCAGGATTGCCAGCTGGAGTGAAATTTGATCCGAATGACCAAGAGATACTGGAGCATTTGGATGCAAAAGTGCAGTCTGATGTGAGAAAGCTTCATCCTCTAATTGATGAGTTCATACCAACTCTTGAGGGCGAGAATGGAATTTGCTATACTCACCCAGAGAAGCTTCCAGGAGTAAGCAAAGATGGACAAGTGCGTCACTTCTTTCACAGGCCTTCAAAAGCATACACAACGGGAACAAGGAAGAGAAGAAAGGTTCACACCGACCAAGAAGGAAGCGAGACTAGGTGGCACAAAACTGGCAAAACCAGACCCATCTCTGTGGCTGGTTCCGTTAAGGGTTTCAAGAAGATTCTTGTTCTCTACACCAACTATGGCAGGCAGAAGAAGCCCGAGAAGACCAACTGGGTCATGCATCAGTACCATCTCGGCTCCAACGAAGAAGAGAAAGACGGCGAACTCGTCGTTTCTAAGGTTTTCTATCAAACACAACCTCGACAGTGTGCAAACAAGGATCCTTATGATGAAAGATTATTGATGACTTCACAAATTAACAGTGTCAATGACATTAGCATTCACGCACTACCCAAGAACAACAACAACAACAATGCAGGTTTTGTGGATTATTATAACCCCGGTTTCATGAATATGAATTATGAACAGATGAACGAGACTACCTCACCGCAACTGATTCCGAATATGGTGGTGCAAGGTGACAGCTCTTCTTTCATTCGGTTACCCAGGTTGGACAGAAAGTAGTATGACAATGACTGCATGCATGTTGATGTTTTTTTAATGCC

>AiNAC44--Araip.HIJ9F

CTTCTTCTCATTCATTCATGCCCCCTTCCCCTCTTGGCCTAATCTCTTTCTCCACATGGCGACAAATTCCGTCAACCATAAAATCCCCCTCACTTCTCACCTCCCCAAAAATTTCAATCCTCTCTTCTTCCCTTTTCCTAGACTTTAGTTTGCTACACCAAACTTCTCTCTCCCTTCTGTTATTCTTATTATTCCTCACCAAGCCTATAACAAATATTTTGTTACACCAAATTATTAGGCTCTCAAAAATGGGATCACCGGAATCAAATTTGCCACCAGGTTTTAGGTTCCATCCAACCGATGAAGAACTCATTCTTCACTACCTTAGGAAGAAGGTAGCATCCATACCCTTACCTGTTTCCATCATCGCTGAGGTTGATATCTACAAATTGGATCCATGGGAATTACCAGCTAAGGCTGCGTTTGGTGAGAAAGAATGGTACTTCTTCAGTCCAAGAGACCGGAAGTACCCAAACGGTGCGAGGCCAAACAGGGCAGCTGCTTCAGGGTATTGGAAGGCTACGGGTACCGACAAGACCATAGTGGTGTCGCCGGCGGCCACAGTTACACGTAGAGTAGGCCAAGAGAGCAGCGTTGGTGTCAAGAAGGCTCTTGTTTTCTACAAAGGAAGGCCTCCAAAGGGTGTTAAAACCAATTGGATCATGCACGAATATCGTCTTTTGGATGACTGGGTTCTGTGCCGGATTTATAAGAAGTCGAAATTCTCAGTATCTTCACCGGAGGAATCACCGTCGAGTGAAGTTCAGGCTGCAGAAGAAAATGGTTTATTCAAGAACACCATTTTGAAGAGTCCAATTCCAACACTGTCGCCGTCGCCACCGCCGCCGCTGCCGCCGCAGCCACTTCTCTCTCAAAAATCTGTGTCATTCTCAAACCTCTTAGATGCCATGGACTACTCCATGCTCAGCACCATCTTATCTGAGAACAATAACAACAGCACCCTTGATCAGGAACAATACTCCCAGATCAACACCAACCAATTGAACCATTCATCGAACATGGAGAACACTAGTAACAGCAACATGATGCTGATGAGGTCAAAGCGCCAGATAGACGAGGAAACAACAACGGTGTTGCACCCATCAAAGAAGTTCCATCACCAACTTATGGGCTCTTCTTCTTGCAGCTTCCCTAATAACATTAACAACACAAACACTGCACAATACGAGAACCCGCAATGGAACTACCTTGTCAAGCAATCCTTCTTGAACCAGCACTTACTTCTCGCTCCTCATCTTCGATTTCAAGGATAA

>AiNAC45--Araip.HYM8C

ATGGAAGAGCCTGTGGTAGTTAACAAAGGCGAGGAGCCGCTGGATTTGCCACCAGGTTTCAGATTCCACCCAACAGACGAAGAAATCATCACTTATTACCTCACCGAGAAGGTCATGAACAGCAGCTTCAGTGCAACTGCCATAGGTGAAGCCGATTTGAACAAATCCGAACCCTGGGATTTACCAAAGAAAGCAAAGATGGGAGAGAAGGAGTGGTACTTCTTTTGTCAGAAGGACAGGAAATACCCGACGGGGATGAGGACCAATAGAGCAACGGATTCCGGTTACTGGAAGGCAACCGGAAAAGACAAAGAGATTTTCAAAGGGAAAGGGAATCTCGTTGGGATGAAGAAAACGCTTGTTTTCTACCGAGGTAGAGCTCCCAAGGGTGAAAAGACCAATTGGGTCATGCATGAGTTCAGATTGGAAGGCAAATTTGCCAATTATAACCTCCCCAAGGCTGCAAAGGATGAATGGGTTGTGTCGAGGGTTTTTCACAAGAACACAGATGTAAAGAAGACTACTACCCCATCCTCATCATCATCAATAATTCCTGGCCTTTTGAGGATCAACTCAATAGGCGATGATCTTCTAGATTGTTCCACACTCCCACCTCTCATGGACCCTCACCCTACTCCTCTCGATACAACAAAATCCGACGGCTATTATTTCCCCTCCTTCTCATCATCACATCAGATTCTCAATATCAAGCCCGAAGAACACAACACAAGCCACCAAATTCCCATCACCAACTACCAGATTCCAAATTTCAACACCACACTCTCTTCTTCATCATCTCATCAAATCAGACTACAAAATCATCTCAACTTGTTCTCATCATCATCATCAAATAACAATTACCATAATAGCTCGTGGCCAACCTATTATGATGAGGTCCACCACCATCATCAAGATGATATTCTACTAAGAGCAATCGCGTCAAAGAACTATAGCAACGGAGGAGGAGGCGCCGGCGAGTGCAAGGTGGAGCAATTCTCCTCCGGCAACCAGTCAGTGGTGAGCGTTTCGCAAGAGACGGGGCTGAGCAACGACAGAACCACCAATGACACGTCATCGGTGGTTTCGAAGCAGCAGCATAATAATAGAACATTGTACGAGGATCTTGAAGGTCCTTCTTCATCAGTTGCACCTCTTTCAGATTTGGAATGCTTGTGGGATACCTACTGA

>AiNAC46--Araip.I60BC

TTCCAAAATGTGCAACACCCCATAAAGTAGTGTAAGTACACATCACCATTTTAATATAGTGTATGGAGGATCCACCAACTGGTTTTCGGTTCTATCCTACAGAAGAAGAGCTAGTTGCTTTCTACCTAAACACCCAGCTTCAACTACAAGGCCACGCCAATAACATCAACAGGGTCATTCCAGTGGTTGACATCAATGGCGTTGAGCCCTGGACTCTTCCATCACTGGCGGGAGAGCTGTGCAGGGAAGAGAAGGAACAATGGTTCTTCTTTGTGCCTCGCCAAGAGAGGGAAGCCAGAGGGGGGAGGATCAACAGAACCACTGCTTCTGGAAAAGCTCCCACCGGTCGCAAAACTAAATGGAAGATGCATGAATACCGCGCCATCGTTCAAGCCCCTAACCAATCTCCCACGGCTATTCCTCAGTTGAGGCACGAATTCAGCTTGTGTCGCGTGTACGTGATATCCGGAAGTTTCAGAGCATTTGATCGACGGCCACGGGAGGTGGTGGTGCCAAGAGTTCTTCATCATGGTTCTTCTACAACAAGTGCTCAGCAGCATCAAGGAGAATCATCAGCAAGGGTGCAGGCTAATAATAATAATAACGGGTCGAGCTCGTCGGAAACTTCCCTTTCATCAGGTGGTCCTGATTTGCCACCAGATACTGGAGGAGGAGGGTCAAGTAGCAATTGGAATAGTAGTGAGGTTCAAGTTCAAGCTCAAGTTCAAGAACCGCTATGGGAATGGGAACAACTCGATTGGCTATAAGCATGACTAACAATATCAATTCGTCAAGCCATGCATGCATTATTTCATCTCT

>AiNAC47--Araip.I6LH9

TCAGCTAGAGTTGAAGCTTCTCGAGAAATCAAGAACAAGAAGAAGAAGGAGGAATAATGCAAGGTGGATTAGAGTTACCGCCAGGGTTCAGGTTTCACCCGAGCGACGAGGAATTGGTGAACCACTATCTCTGCAAGAAATGCGCAAAGCAATCAATTGCTGCTCCAATAATTAAGGAAATCGATTTGTACAAGTTCGATCCGTGGCAGCTTCCAGAGATGGCGTTGTACGGAGAGAAGGAGTGGTACTTCTTTTCGCCGAGGGATAGGAAATATCCGAACGGATCCCGGCCGAACCGGGCGGCTGGGAGCGGGTACTGGAAGGCGACCGGGGCGGACAAGCCGATAGGGAAGCCGAAGGCGCTAGGGATAAAGAAAGCGCTAGTGTTCTACGCCGGAAAGGCCCCGAAAGGAGTGAAGACTAATTGGATTATGCATGAGTACCGTCTCGCCAATGTTGACAGATCCGCCGCCAACAAACTCAACAACAACAACTTGAGGCTTGATGATTGGGTGTTGTGTCGAATCTACAACAAGAAAGGGAAGATTGAGAAATTCAACTCTGCCACAACAGGGTTGGAACAGAAACTACCAAAGTTTTCACCAGGGGAGATACTTCACTATGATCATGATCATGAGCATGAGACAAAGCCAAAGATTATCCACAATTTCTCCAACAATGAGCACCAATTGTACATGGACACATCAGATTCCGTTCCAAGGCTGCACACGGACTCTAGCTGCTCGGATCACGCGGTTTCGCCGGACGCCACCTGCGACAAGGAGGTGGAGAGCAACCCAAAGTGGAGCAATGAGCTAGATATGCAGCTGTTTGATACCTTTGATTTTCAGCTCAACAACTATGATAATAGCCTCCCAATGAATGATGATGACCTTTTTGGAAATCAGTTCCAAATGAATCAGCTCATGTCTTTCCAAGACACATTCTTGTTCCCACAAAAGCCATTTTGATTCTTTTGACCATTAAGAATTATTGGGAATTGGAAGAAGAGGGGGAGAAAAAAAGTTGAACCTTTTTTTCTGGGAAAATGTGGTGGAGGACTAGTGCCTAATGAGATCATGGGACCAAAGCATATGTTCATTTTTTGTAGTGAAAACTCAGCATTTAACACAATTCACACCTACCTAACAATTTCACTTTTGGACCATTGGATTTTGGTGGCTATTCTTTGTTCTTCGGAGATTAGGGAGGGAGATGT

>AiNAC48--Araip.J93FI

AATTAAAGCTACCTTTAGTCAGAGTAGAAAGGACAAAAACTTGAATTTAGTCTTCTACAAGCTTTCATACAAATGATATATACAAAGCAAATAAACCACAAAACTTGAGATCAAAGCAACCTTATTATGCATTATTTCTCATGTGCAACTAACCCTATCTATCTCATCTTTCTAAATAGTTAGTAGTTTCATTCCTCCTCCATTTAGAATTTTCAGGGAAGGAGGGAGTACAATATTATTCACCTCATGCAAAAGAAAGAAAGCACCAAAAAAAGTGAGAGGAGAATTTAATTCCTTCCCAACCCTTAACAAGAACTTGCATGAGGGGAGCAACCTTTAGAAGAGACAACTTCTTCTTTATTATTTCCCCTTCTTATATACTCGTGAAGAAGGAAAAAGAAGATATGTATCCTGAAATGGAATGAGAGAGGAGAAGGGAAACATAGGGTAACACATACAAGAGGAAATAATGACTCAGTGCAACAGTAATGATTACCCTGAAAACAATCATAACACCATTGTGGAGAGGAACAAAGATATCTTAATTAGTAGAACTTGTCCATCATGTGGTCATCATATCAAATGTCAACAAGACCACCAGGGTGCTGGAATTCACGATTTACCTGGGCTACCAGCTGGAGTGAAGTTTGATCCAACAGATCAGGAAATTCTAGAACATTTGGAAGCCAAGGTGAGGTCTGATATTCACAAGCTTCACCCCTTAATTGATGAGTTTATCCCTACTCTTGAAGGCGAGAATGGAATCTGCTGCACCCATCCAGAAAAGTTGCCAGGAGTAGGCAAAGATGGGTTGATCCGTCACTTCTTTCACCGGCCATCCAAAGCATACACAACAGGAACAAGGAAAAGAAGAAAAGTTCACACTGATGCTGATGGCAGTGAAACAAGGTGGCACAAAACAGGTAAAACTAGACCAGTCTACATCAGTGGCAAGTTGAAAGGCTACAAGAAAATCCTTGTTCTCTACACCAACTACAAGAAGCAAAGGAAGCCTGAGAAAACAAACTGGGTCATGCACCAGTACCACCTTGGCAACAATGAAGAGGAGAAAGAAGGTGAGTTAGTTGTGTCTAAGGTTTTCTACCAGACACAACCTAGACAATGTGCAGGTTCACTACTCATCAAAGATTCATCATCATTCCCTGCTAAACTAAAGGATCAAAGTGGTGTTCATCATCATGAAGTGACTAATAATCATAAGAACAATGGGTTTGTGGAATACTACAATGCATCCTTTATAAGCTTTGCTCAAGGGGAACAACAACATAGGTCAAACAATCCCACATTGATTTCCCATTTTCCTGCTCATGATGGGGCTCCGTTCATTCCTTGATATATTTTATGTGAAACATAACTTTTATTACTTGTACATTTTTATTATTGATTAGAAATTCTTTGTGGGACCAAGTTTAAGGCTCACATTTTTATCAATAATAAAAATGTAAGTAAGATAAAGTTATGTAAAATTTCATATGAGAAAATCTATTCTTAAAGGATGAACAAAATACTAAATAGGACTTATTACAGTTATAAGCTAAGGCATTACAATTGTATTCTGCAATGAAGAAATAAGTTAATGACCAATTACAGTTAGGAGGGGAAAGAATAAGGAGAAGCTACTTCATTGTAAAGCTGGTGGTCAGATACTTCTATTTTGGGAAAAATTGGGCTAAGAAACTAAAAGAGAAGGATGTAACTG

>AiNAC49--Araip.J9WH5

TTTCCTTCCTTCCAAGTTCAAATTAAATTTCAGCTAAGTAGTAGTAGTAGTAGTAAGTGATTATTCTAGCTTGATGGGTTCTTCTAATAACGGTGGTGTGCCACCGGGGTTTCGATTTCATCCAACCGATGAGGAATTGCTTCATTACTACTTGAAGAAGAAGGTGTCGTTTCAGAAGTTTGACATGGATGTTATTAGAGAGGTCGACCTCAACAAGATGGAGCCTTGGGACTTGCAAGAAAGATGCAGAATAGGGTCAACACCACAAAACGAGTGGTATTTCTTCAGCCACAAGGATAGAAAGTACCCAACAGGGTCAAGGACAAACCGAGCAACGAACGCAGGGTTCTGGAAAGCGACGGGAAGAGACAAGTGCATAAGGAACACCTACAAGAAGATTGGGATGAGAAAGACACTTGTGTTCTACAAAGGTAGAGCCCCTCATGGCCAGAAGACTGATTGGATAATGCACGAGTACCGCCTTGAAGATTCCAATGATCCACAAGCAAATGCCAACGAAGATGGGTGGGTGGTGTGCAGGGTGTTCAAGAAGAAGAACCTATTCAAGATTGGAAATGAAGGAGGTGGTGGCTCCACACACACCTCATCGGACCAGCAACTCAACAACTCAACGGCCACCAATGCTCGTTCCTTCATGCAAAGGGAAAACCACTACCTACTGCAAAACCCTAGGAATGGGAACCCATCCTCTTCATCCTCAGGCTTTGATGAACTCGATAAACCCGAGCTCGGTCTCCATCACTATCCTCACATGCAAACCCCACACTATTCACTCTTCCATCACTCCCAACCACTTCTTCATCCCCAGGCCCACAAGCCCATCGTCTATGACTACTCTTATGCACCCGCCCTTCCTTCAGACCCTCCTGTCATCGCTAAGCAGCTCATGACTAACCCTAGAGACTGCGATAGCGGTGGCAGCGAGGGTCTGAGGTACCAGCAGGTTTCCGAGCCTGGTATGGAGGTTGGATCATGTGAACAAGCCCAGGAAATGGGCGCCGCAAGAGGAGGAGGAGAAGGAATGAATGAATGGGGTGTGCTTGATAGGCTTGTAACCGGGAACCTTGGAAATGAAGATTCAGCCAACAAAGGGATTAGGTTTGAAGATGCAAATCCACATCAGATTAACCAGCTTTCTTTGAGGGGAGAGATGGATTTCTGGGGCTATGGGAAACAATAACTTCCTCCATGATTGAAGATAAATAAGAGTTCCATCCTTGCCTCTAATTAACCATTTAATTAAATATGGGGAGAAA

>AiNAC50--Araip.KI83M

CTGAAGTTAAGGAAACAGTAGGAGAGGTGTGAGAGTGAGGATGAATATAAAAGCGGAAGCACGGACTAGTTGCCATCGAAGTGTACATGCTAATGACACGTTAAAAGAAAAATAATGGCATCAAACATGCTTGGTTCCCTCGCCGACCCTGTTTCACTGAAAGTCCAAAGGCCTAAATTGCCATACTAAATTGTTGTCACTCACCAACAAACAAAGAACAGAACAACATAACCCTCTCTAACACCAAACCCTTTTACTTTCACCTTAAAATCATGGTGGATAGGGATTCAAGTGAAGCACACATGTCAATAGCCGCTTCTTCCATGTTCCCTGGCTTCAGGTTCTGTCCCACGGACGGTGAATTAATCTCTTATTACCTCAGAAAAAAATTGGACGGTGACGAGGACAGTGTTCAGATTATTTCGGAGCTTGAGCTTTGCACCTTTGAGCCTTGGGATTTGCCTGAAAAATCGTTCATTAAATCAAATGATGAGTGGTTTTTCTTCTCGCGACGGTGGAGAAGGTATCCCAATGGTTCACAGAATAAAAGGGCAACTAAAAGTGGTTATTGGAAGGTCACAGGAAATGAGCGACAGATAGAGTCTGGTCAGAATGTGATTGGTACCAAACGTACTTTGGTATTCCATGTCGGTCGAGCTCCCAAAGGCGAAAGAACTGAATGGATTATTCATGAATACTGTATCAATGACAAATTTCAGGATTCTTTGGTGGTTTGTCGGCTCAAGAGGAACACAAAATTTCGTGCAAATGATGATTCTAACAGAACTTCACGCGAGAGTGGTTGTGGAGTCTCAGAAGGGGTTACAGTTCAAATGAGCACTTGTGTGCCTATTCAAGATAAAGAGGTTGGGTGTAGTTCCAAGAGGAGTAACAATAGTAATAGTTCTCCTTCTATTACTGTCCAAATTAAATCCAGTGATAGAGTTGCCAATGAAGACAATCCCAAAGCTTCTTCCAATGAATCCAGTGATAGAGTTGCCAATGAAGCCAATCCCAAAGATAGAGTTGCCAATGAAGCCAATCCCAAAGCTTCTTCCAATGAATCCAGTGATAGAGTTGCCAACGAAGCCAATCCCAAAGCTTCTTCCAATCACTCTAAGGTGGATGAAGTGGATTATTATGCAGAGATCAACTTAGATGATATCATCAACTTAGATGAACCAGCACTCTAA

>AiNAC51--Araip.KM0ZG

ACACACATACCTTGGACCACCAACACCAACACCTTCATTCTCCATTTTCTCACATACCCTTTTTGTAATAGCAGCAGCTGGTTTCGTTTTCTTGCTTTAATGGGGGATAGCAATGTCAATCTTCCACCGGGGTTTCGATTTTATCCCACTGATGAAGAGCTTGTAGTCCATTTCCTTCAGAGAAAAGCAGCACTTCTACCTTGCCACCCTGATGTCATTCCTGATCTTGATCTCTACTCTTTTGATCCATGGGAACTTGATGAGGGGAACCAATGGTACTATTACAGCAGAAGGACACAAAATAGGGTCACTGCCAATGGTTATTGGAATCCAATGGGAATTGAAGAGGCAGTGGTTTCAAACTCAAGCAACAGGAGAGTTGGTATCAAGAAATTTTATGTGTTCTATGTTGGAGAAGCCCCTCATGGTAACAGAACCAATTGGATCATGCAAGAGTATCGTCTTTCAGATTCTGCAGCATCCTCTAGCAGATCATCAACCAAAAGAAAATCACAACCAAAAACACTTTAG

>AiNAC52--Araip.KP5QZ

GAAAAATATTCATTCACTGATCCATGATCATGGTGGATAATAGCACAGATTCATCATCAGGAGCGGGTGAACAGCATCATCACCCTCAGCTTCCTCCAGGCTTTCGATTCCACCCCACAGACGAAGAACTCGTCGTTCACTACCTTAAAAAGAAAGCTTCTTCTTCACCACTCCCTGTCGCCATCATCGCCGACGTTGATCTCTATAAGTTCGATCCATGGGAGCTCCCAAGTAAGGCAGCGTTTGGGGATCAAGAGTGGTACTTTTTCAGTCCTCGGGATAGGAAGTACCCGAATGGAGCTCGGCCAAACAGGGCGGCTACTTCTGGGTATTGGAAAGCCACCGGAACGGATAAGCCTATTCTGTCTTCTGATGGGAACAAGCAGAAAGTTGGAGTCAAGAAAGCGCTTGTTTTTTATGGTGGCAAGCCTCCCAAAGGCGTCAAAACCAATTGGATTATGCATGAGTATAGGCTCACCGATAACAATAACAATGCTTCTTCTTCTAATTCATCTAAGCCTCCTTCTATCCCTCTTGATCCACTCAAGAAGACTTCTCTCAGGCTTGATGATTGGGTTTTGTGCCGAATATACAAGAAGAGCAACAGCAGTAGTAGCAGCCTTCCAATTCCAAGGCCAGCGTTTTTAATGGATGAAGAGAAGGATCTAATTTCCATGGAGAGCAGCATGGTGCCAACTATGTCAATGTCAAAACCAAGAAGCACTTCAACAACAGGTTGTTATGGACCCATGGCACTTGAAAACGATGACAACTTCTTCGATGGTATATTGGCAGCATCAACCGATCATCACACCATGCAAAATGGTAATTAATTATTACCTTCCTTAATTACTTAATTCAAACAAATTAAACTTACTAGTTAATAATAATCCACAGGGTCTCCAGGGTCCTCATCTTCAAGCAAGAGATTCCATGGTGATCTTAATAATGGAGACAACACCTCCTTCGTTTCTCTTCTTAACCAGCTTCCTCACAACACACCCTTTCACCCAAACTCCATTCTTGGCTCCGTTGGAGACGCCGTCTTGAGGCAACAATTTCAACTTCCAGGCTTAAATTGGAACTAATTAATTAATTAATAATAATATTTTGCGTTGCTTTGGTGGGGGGTATTATAA

>AiNAC53--Araip.ZUP60

CCCCTCTTTCCTGCGAGCTTCTTTGTCTTCTTTCCTTTGTCCTCAAACGCTCCTTTCCCTTATTGCTTCTCACTAAAGGTAATTAATTAGAAGCAAAAGAATAATCAGAATGATGGCAGGTAGTGGACAACTAACAGTTCCACCAGGGTTTCGGTTCCATCCAACTGATGAGGAGCTTCTCTACTATTACCTAAAGAAGAAAGTTTCTTATGAAGCCATTGACCTTGATGTCATTAGAGAAGTTGATCTCAACAAACTTGAACCTTGGGACCTCAAAGATAAATGCAGAATAGGATCAGGGCCTCAAAACGAGTGGTATTTCTTCAGTCACAAAGACAAGAAGTACCCAACAGGAACAAGGACCAATAGGGCAACCACTGCTGGTTTTTGGAAAGCCACTGGGAGGGACAAGGCCATATACCATACTAACAATTCCAAGAGGATTGGAATGAGGAAAACCCTAGTTTTCTACATCGGCCGTGCGCCCCACGGCCAGAAGACTGACTGGATCATGCATGAGTACCGCCTCGATGAAGACGACGCCGAAGTTCAGGAGGATGGGTGGGTGGTGTGCAGGGTTTTCAAGAAGAAAAACCAAAGCAGAGGGTTTCAACAAGAAATTGAAGAAGAGGAACATCATCACTTAGCAGCAGAACATCAACACATGAGAGGAGTAGCAAGCCAACAAGTTCTGGACCCAAAACACCACCACCACTTGCAACATCATCAAGGACTCTATGATAATGATAATGATAATAATTACACCAATAATTTTGATGGATCCATGCACCTTCCACAGTTGTTCAGTCCAGAATCTTCCGTGGCTACCGCGGCGGCGCACACTTCCATGAATGCCATGGACATTCTTGAATGCTCCCAGAACCTTCTAAGGCTCACAACAACAAGTGGATGTGGACTCAATCTCATGCAACAACAACATGGAGAGAGGTTCAATGGTGATTGGTCTTTCTTGGATAAGCTTCTTGCTTCACACCATGGCAGCACCATGGATCATCATCACCATCATCATCATCATAGCAAATGCAACAATAATCTTCATCATCAGCATCCTGCAATTGCTATTGGAACTACTTCATCTCAGAAATTCCCATTTCACCACCTTGGTTGTGACAACCATGATATCATGAAGTTTTCCAAGTAG

>AiNAC54--Araip.Z57SD

AGCTGACAAAAGTATTATTTAGTATCTATGTAAGTATTAAAGTAGTAAAGTGCTAGAACCTTCTTTCTCCTCTAACATGGGGTGGGAACTTTGAACTTTCTATATCACCAAAAACGACTCAAAACGGGTCTCGTTTTGAACCTTGAACCCTAAATCCCCAATTCAAACCACTCTCTCTGATACTCGCAAAGCTCGCTCTTTTAGCTGATTAACCTGCTGTTCAATCGTCATCGTGATCGTCATAATCATACATGAGTCGAATACTCGGTCCCGGTTTCCGCTTCCACCCTACGGACGACGAACTAGTTCAATACTATCTCCGCCGGAAGGTCATCGGAAAACTCAACCACCACGACCACATCGGCGTCATCAATATCTACGACTATGAGCCATGGCAACTCCCCGATTTGGAATGGTATTTCTTCACGGTTCTGGACAAGAAGTACGAGAAGGGGGAGAAGACAAAACGCGCCACCGTCAACGGTTACTGGAAGACCACCGGCAAGGATCGTGGAATCAAGTATGGCGATCGCCAAGTAGGCATGAAGAAGACCCTCGTTTACCATGAAGGAAGGGCCCCGACTGGCAAAAGATCAAATTGGGTTATGCACGAGTACCGGATGGTCGATGAGCAATTGGCGGAAGTCGGATATCAGCTGAGTCAAGGTTTGTTTGTTTGATTGGTTTATTTGTGTTTTTGGTGGTGGTGGGCGAAGGACGCTTTTGTGCTGTGTAGAATTTTTGAGAAGAGCGGGATGGGCCCTAAGAATGGAGAGAAGTATGGTGCTCCCTTTAGAGAGGAGGACTGGGTGGAGGATGGCGACCTGCTTGAACCGATTGCTGATGAACCTGTGGTTGAGCTGTCTGTTGACCAGAGTGATGCTTTCCTTGAAACTGATGACCTTGAGAAGAAACTTGGTACGCATGTGGTTGATGGAAGTGCTGATTTACCACCAAACCCTCCCAACTACTTTTATGGGGAGTGTAGTCACTATCCTCAGCATCAAGAAGAATTTGTTGAAGTTCCGAAACCTTTGGAAGGTACTGAAGGCCGGAATTTCGATGTAACTGGGCCATATGCTGAGGATACCTGTTTAGAAAATCATGAAATGAACCATAATGGGAATTCTTCAGGATTCATTTATGGTGATGTTAATTCAGATGAAATCATGGATTCCATTGTTGATCCTCTGATTGGTGCTGAATTATTCCTGGAAACAGATGATCTTCTGAACCCAATCGAGGGAAATTCCTCTGGGGCAGATCCTTATACAGTTGAGGGAAATCACCCCAGGGCCGATCCCTACGCAGCTGAGGGAAATCCCCCTGGGCCAGATCCCTACACAGCTGAGGGAAATTATCCTGGGACAGATCCTTATGCTGTGGATATGTTAGATGAGTATCTTGCACTTCCAGATGATGATATTCTGAGGTATATATCCTTTGATGATTCTCCTCCATCAATGGAGGGTGAAAACCCTATTCTAGAGCAGATACCACCTCTTATACAGCAGAATGTGGAGGAAGAGGCCAAGGATGTTTCAGAGGAGAAACAACAAAAGGTGGAGGGAGAAGCCACAAATATTTTCAAGACAAACAAACATGACCTTGAAGCAAATTCTAGCCGTGGAGGATCTGCTTCAGATGATGCAAATCCAATTGCAAAACGCTTCAAGAAATGGTTGGAAGACATCCCAGCTGCTCCCGCATTTGCTGCAGAGCTTCCATCCAAGAAGGATGCACTCCAGCTTCATTCTGCACCTCAGTCTTCAAATACTACTCATGTAACTGCAGGAATGGTCAGCATTACAAACATTACTGGAAGAGGCAATGACATGAATCCGATGGTGGCAAAGATTGGAGGAGGGTTCAACCATCCCATTATCTCTGCTGTTGTTTTGATACCTGTTTCTGGCTTACTTTGTGGCAAGACTCTGTTTGTGCTGACATATGGATGGGCTTTTCTGGTGACATTTTCATTTCTGTTTGCCACCGTGACTTGCAAAATTGGAACCTTCATGTATTCTGGAAAATGAGAGTTATGAGGTCTGCTGTGAGACAATGGGGTGCGGTTGGTTATCAATTGGTAGTGATGGTACTAATTGTGTGACTCAAAGATACTACTCTATTTGAAAGTTGCACAACGAATACCCATAACCGCCTGCTGATATCCAACCACCGCCCATTGGCTCCTT

>AiNAC55--Araip.L222I

GTGGTACTTAATGATGAAACTAATGCGACTTAATTGGGTTTGATTTCTAATTAAGTTGGTAGTTGTTCTCTTTTTAAGAGCTTCATAGCAACGCGATCCTCGCAAAACACACGCTCCTTTAAGAGTCACCACAAAAGACGGCTGAACCTTCGGGTACAAGATCCCTTCTCAGGAAGCATATAATCACTTCACTACTTCAACTGTGTTCTCTCTCTATCTATCTATCTCTTCATTATTAGGGTTTTCATTTCTTGATTATTACCAGCTTGCTTCTTCTATATATCCACCTCTTTCAACTCTCTACTCTTTCTTGAGGAGATGGAAGGTGAGAAGCTTGATGAGATCATGTTACCAGGTTTCAGGTTCCACCCAACTGATGAGGAGCTTGTGGGGTTCTACCTTAAGAGAAAGATTCAGCAAATGCCTCTGTCCATTGAGCTCATCAAGCAACTTGATATCTATAAATATGATCCTTGGGATCTTCCAAAAGTGGCAGGTACAGGAGAGAAAGAGTGGTATTTCTACTGTCCAAGAGACAGAAAATACAGGAACAGTGCAAGGCCAAATAGGGTAACTGGAGCTGGGTTCTGGAAAGCCACAGGGACTGACAGGCCTATATACTCCTCAGAGGGTTCAAAGTGCATTGGACTCAAGAAATCTTTGGTCTTCTACAAAGGCAGAGCTGCCAAAGGTGTTAAAACTGATTGGATGATGCATGAGTTTAGGCTCCCTTCTCTTGCTGACTCTTCATCCGACAAGACCACTATTCCTCCTAATGACTCTTGGGCAATCTGCAGAATATTCAAGAAAACAAATGCTACAGCTCAAAGAGCACTCTCTCACTCTTGGGTTTCTACCTTACCTGAAACACCAACCACCACTACCAATGATACAGATCACATATTCCACTTTTGTTCATCCAACATGCCAACAATGATGGCAAAGAAAACTAGCTTCATGACCCAGTTTTGCACTAACTACACTAGTGACACACAAATCCAAGATGTTGCATCATCTTATAAACCACCCTTCATTAATATTAATCCATTGCTTTACAAACACTTTGATCATCATCATCATCAGTTACCACCCATTATTTCAAATGGAGATCTTATAAGCAACGACTGTTTAATACCCTCTTCTACTACTACTCCACTTGAAACATCCTCTAATAGTGCAAAACCTACTATGGATTTTTCTTCATTGTTGCTGAACATGTCATCTTCTGTTCTTGGAGATTTTGCTGGAAAGACATCATCGTCGTCCTCATCCCAAGAGGGTACAGCAGCAACAGCAACAACAATCACAAGTAGCTTCGGTGGTGGAATGCAGGAGCACTACCCAACAATACCATTACTGCGTCAGATGCATCAAGGGAACAACAACAACAACATTGGCATCAACAACAACAACGTGTCTGCTGGCGGTGAAGAACAAGAGTTGGAGAAAGTTGGATCCATTGTTGGGTTCCCATTCATGAACATTGGGGATGCATGGAAGTCAAATATGCTTTGGGATACTTCTTGTCCCTTGTGA

>AiNAC56--Araip.MQD5S

ATGGCAGAAACTAGGGTTCTACCTGTTGGATATAGGTTTCGTCCAACAGAAGAGGAACTTTTAATTCACTATCTCAATAACAAGCATTTGGGAAATGATGCAGAGATTAAGAACACTATTTCCCAAGTTGATCTTTGTAACTTTGATCCTTGGGATTTGCCAGAACAATCGAAGGTGAAATCGGATGATCAAGAATGGTTTTTCTTCAATGAATTGAAATACATGAAAAACAAGCGATGTAACAGAAAAACCAACATGGGATATTGGAAGATCACAGGAAAAGAGAGAATCATCAAAAGAACAGGGACAGACAGTGTCATAGGTACAAAAAGAACACTAGTTTTCTACAAGCGTCCACATAATGTCAAAACCAATTGGGTTCTTCATGAATATCATGCACTTCATCAAAAGGTAGGTTCTTGCCAGAGCAACATCGTGTTGAGCCGTGTAACAATGAATGCTGAGAAAAGGGAAAAGAAGCTCAAGACAAAAGCAAGCAACATAATCGAAGAGGAAGTAAAATGTGAAGATGAACCATGCAGCGAAATTACTGGCTGTGTTACCCAAGCAACTACAGAAGATGCAATCCTTCCTGATAATGCATGTGTTTCATCCGAGTGGCAACAACCTCAAGCTATGGACTATGAAATTCTCTCATCGGGACAACGATCTTCAGTGGCCTATTCCGGTAATGAAAACAATGCCGCGTTGCTTCCAATGGAAGCAACGTGGAGGCAAGATGCCGGTATGAATACCGAGTGTTTTTGGAATTCGCTGTTTTCTAGCATCGATGCTGACCCTCATGCCGAGTTCTTAAATTCAGTGTTGGCAGGGGATGATCAACTCTATGTTGATTCCGGCCACCATTGACTTTACACAGGTAGAAGTAAAGATGTAAAAAAATTTCTTTCATATACATAGACAACTCACAGGATGTTTTTGAGCAAGCTTTTTTTGACAAGAAATTTGACCCCGAAGTTGTAGTTGTTATACTTCAACAAAATCAGATCTAAAATGTTAATTGTGGGGTGAGAGAGGCTCGAACTCTCGACCTCAGGATAACTCTAAAGCTATGAGAC

>AiNAC57--Araip.NB7HU

GTGAGAGATCCATAAATATGGAGAGCACCGACTCATCCACCGGTTCGCAACAACCGAACCTTCCACCGGGGTTCCGGTTCCACCCCACCGACGAGGAGCTCGTTGTTCACTACCTCAAGAAGAAAGCTGCATCAGCTCCTCTCCCAGTCGCCATCATCGCCGAGGTTGATCTCTACAAGTTCGATCCATGGGAGCTACCAGCTAAGGCAACGTTTGGGGAGCAAGAGTGGTACTTCTTTAGCCCAAGGGACAGGAAGTATCCGAACGGTGCTCGGCCAAACAGGGCGGCAACTTCCGGGTACTGGAAGGCAACCGGGACGGATAAGCCGGTGCTGACCTCCGGTGGGACCCAGAAGGTGGGTGTGAAGAAGGCTTTGGTCTTCTATGGAGGGAAGCCACCGAGAGGGATAAAGACAAATTGGATCATGCATGAGTATAGACTTGCTGATAACAAACCTAACAATAGGCCTCCTGGTTGTGACTTGGGTAATAAGAAAAACTCTCTAAGGCTTGATGATTGGGTATTGTGCCGAATCTACAAGAAGAACAACACACATAGGTCTCCAATGGAACATGAGAGGGAAGATTCTATGGATGACATGATTGGAGGGATTCCTCCTTCCATCAACGTGGGGCAAATGAATGCAAGATTTCATCTCTCAAAAATGTCAACAAGCTTTAGCAACGGTTTGTTGGAAAACGACCATCACCATCACCAGAATCTTCTGGAAGGTATGATGCTAGGAGGAGGAAACAACAACAACAACAATGTTGTTCCTCCAAACATGTTGGGGTTGGGATCAGCCTCAAACACCATTAACAATAATAGTAATAAGGCAGAGCTTTCATTTGTACCAACCATGACTACATCTTCAAACACCAAGAGGACTCTATCATCTCTCTATTGGAATGAAGATGATGTTGCTGCTTCCAACAAAAGATTCAATTTGGAAAGTGGAGATCATAACCATGGAGAGAATAATGCTATAGTGGTGGTGGTGATGGTGATACCTTAA

>AiNAC58--Araip.NL359

ATGTCACCAGTTGGATTACCACCTGGGTTTAGGTTTCATCCAACAGATGAAGAGCTTGTTAACTATTATCTAAAGAGGAAGATCAATGGCCAAGAAATTGAACTTGATATCATTCCTGAGATGAATGATTATATATCTAAAAGTCGAACATTTTGCTTGTTTCTAGAAAAATCATTTTTGCCGAGTAGAGATCCAGAGTGGTATTTCTTTGGACCAAGGGACAGAAAATACCCTAACGGATTTAGAACAAATAGAGCAACACGAGCAGGGTACTGGAAATCAACAGGTAAAGACAGGAGAGTTTCAAGCCAAAGCAGACCAATTGGTATGAAGAAGACTTTGGTTTATTATAGAGGAAGGGCTCCTCAAGGAATCAGAACTGATTGGGTTATGCACGAATATCGTCTCGATGACAAGGACTCTGAAGACACCACCGGTTTACAGGATACTTATGCTTTGTGCCGTGTGTTCAAGAAAAATGGAATATGTACGGATGTTGAAGAGCAAGTAGGGCATTGTAGTAACATGTCTTCACTAATTGAGAGCTCACAAACCATAATCAATAATAATAACAATAATAGTAATAATAATAATGAGTATTGTGAAACCATGTCACCAGACATAGCAGGGGTTTCATCTTCATGTTTGGAAGAGGAAGACAAAGATGATTCATGGATGCAGTTCATCACGGAAGATGCATGGTACTCTTCTAATGCACCAAATATGGTTGGTGGTGAAGAAGTTTCACATGTTACATTTACAAGCTAA

>AiNAC59--Araip.PNX61

ATGGAACAAGAAGAAGAACCACAACAAAATGAGCCACCTCACTCTCACTCTCAATCTCGGTGCGTGACGCTACCTCCCGGTTGCCGGTTCCATCCTTCGGAGGAGCTTCTATTGCGTTACTACCTGACCAACAAAAACGGCACGGGGAACTGGAATGGTAACGGTGGTTTGGGATTCGATGGTTCTGATTTGATTCGGGAGCTGGATTTTTACGATTACGATCCTTTTGAACTGCCGGATTTTGCGTGCTTTGCGTACGGCTACGGCGGAAGGAGGAGGCACTGGTACTGTTTCACCTCCGTTAGGGTTTCGAGGGGAGAGAGGTGGAAGAGGAAGAGGAAGGTTAAGAGTGGGTTCTGGTTGAGGAGGGGAAGGGTTTCGAATGTTAACGGTGTTGGGGAGAACGTGGTTTTGGGAACGAGGACGAGGTTCGTTTTCTATATGGGTGATTCGGCGAAGAACGGTGCCAGGACGGATTGGGTTTTGTATGAATACGCATTGGTTGATCATGTTATGGCCTCTTATGTTCTTTGCCGGGTATTTAGTAAGCCTCGTTATAAGAATAGTGCATCAGACATCGGCCTGAGTTGTTGTGCAGAAGAGAGTGTATCAGCAGTGCGCCATATTGGTATTCAGCATGATGAACATGTTAAATTGGATGCCGTTGAAGCTAAAGTATGTGATGATATCTCCATTGACCACAACAATGAAATATGTGCTGGTGGAAACAGCGATAATGATAATCAAGTTAAGAATGCACATGATATAGATGCTCTACGTTGTTTGGCGGGTCCTCAGGGCAGTCAGCAGGAAAGGCTTCCTTTACTCCCCAGCAGTAGTACAATGTTCATTGAAGCAATTTCATCTCCACAACAATTACTTTCCATCACGGAGGAAGACTTCATAGAGTTGAATGATCTTACATGAATTGGATGGCTTTAAGAAAGATTTCAATATTAATCCTGCTCTCAGGAGTATTCCCCTGAAACTCATTAGTTCCGAATGGGTAAATATCCCATCAAAGTGGATAAACATTCGCAACGTCGGAGAAATATTTATGATATCAGGTAACCTTTGGCTGTCATATGAAGATCATATTGTCAGATCAGGTGAAGGAAAACATCACAGGGTGATCGCTTCACAAAATAAGGTAGTTACTTTGATGACATTTCAGAGTACGTTAAAGTCTCAAGCCATACCGGAGGATATTTAGTAGATATTGGTTTTGGGCATGCAGATTTTTCAACTTTGCCTACTGAAAGGCAAGTTATGCAACTGGCTCTAGAGGAATAGAGTTAGGAGTTAGTTTATTGGCATTGGTGATTGCAATATGCAATTTACTTAAGTATGGCAAACCTGACGTTAGTTCCTCATGCTGACATTGATCCCTT

>AiNAC60--Araip.PT231

ATGGCTTCTTCACTCCCTCAAAGTCTGTCTTCGTTTCCCACCACCATTCGTGAAACTGTTCCGTTGCTGTGGAAGGGAGCTGCTGATCAGGTGGCTATTGGAAAAAAAGATTATATGGAGAAAGGAAAATTAAGTCCAGGATTTCATTTCAATCCCACTGATGTAGAGCTTCTAAAGTATTTTCTGAAGAGGAAAGTGACCGGCAAAAAGCTACCCAATGTAATTGCTGAGATTAACTCACATCTGAAAAGTGGGGATTTGGAGTGGTACTTCTTCTGTGCAAGAGGAAAAAAATATGGAATTGGGTCGAAGACGAACCGTGCTATAAAGAATGGGTACTGGAAAGCTACTGGTATGGACAAAGCTATTGTTCAGCATGATAAGCAAACTGTGGGGATGATGAAAATCCTTGTTTTCCACACTGGTAAAGCGCCTCATGGGACCCCAACCGATTGGGTTATGCATGAGTATAGGCTTCAAGATAAAGACCTTACTGACAAAGGAATTGCTCAGGACTCCTATGTTATCTGTAAGGTGTTCCGAAAGGAGGGTCATGGCCCACGGACACGGAGCGGTGCACAATATAGGAAACCATTTAATGAAGAAGATTGGGATGATGATGATCATCACGTTGTTGAGGAGGGGGGAACCCCTTCAACTGCTTTGGTTGCGCCGGTTTCCATTCAGTCTACGACATTCGATGGCTCTTCTTACATGAAGCCAACTTCTGTTTCATGTGAGTCGGGACCTGTGGCTACTTCTCCTGTCCCATCAACTCCTTCTTCAGATGCAAGCATTCACACGGTTAATAATGTTAATAATTCAACAGTGACTGATCTATCCAAAGACGAAAAAACAGTACCTGAGGAAAATATTGCTGCCGGTGACCTCTTGAGTAAGTTTTTTGAGGGTTTGGAAGACCTTGAGTCTGAATACACTCCAAATGGAGTGGGCTTGGATGACTTTTCCCCACATGGAATAAACTATGATGACTTGAGGCACTTGGACTTGATTGATCTCAATTTCCTATAGTTCTACTTCTAGCAGCCTTACAATCTAAAAGTTGTAGATTTTTGTTTAGATTTAAAAGTTATTTTTTCATTTTGTACATATCTCATTTTGTAAAAGTCTAATTTGTTGTTTATATTACATATTTTTTGGTTCAG

>AiNAC61--Araip.PW8UQ

AACATAACAACACAAAAAGAGAACCATAATTAAGCAAGCGACATAATTGATGATTGATATGGGTTCTTCATCAGTAATAGACGGTGAAGTTACACTTCCAGGATTCAGGTTTCACCCTACTGAAGAAGAGCTCCTTGATTTCTATCTCAAGAACATGGTCGTTGGAAAGAAGCTCCGTTTCGATGTCATAGGCTTCCTCAACATCTATCACCATGATCCCTGGGACTTGCCAGGGTTGGCGAAGGTAGGAGAGAGGGAATGGTATTTCTTTGTGCCTCGGGACAGAAAGCATGGAACCGGGGGAAGGCCAAACCGGACCACCGAGAAAGGGTTTTGGAAAGCAACCGGTTCGGACCGTAAGATCGTTACCTTGTCTGATCCGAAGCGCATCATTGGATTGAGGAAGACACTGGTTTTCTACGAGGGAAGAGCTCCACGTGGATCCAAGACCGATTGGGACATAGTACTATGCAAGATATATAGGAAGGCGACTTCGTTGAAAGTGTTGGAGCAAAGAGCAGCAATAGAGGAAGAGATGAAGCAAATGGTAGGTTCCCCTGAATCTACACCTTCCTCCACAGACACCATGTCCTATGAAGAACAACAACAGAATCAGAATCAGAATCTGCAATTGTTACCAACACAACATGTTGTTACTAAGAAAGAGGTTGAAGCTGAAGTTGAAGAGGAAAAAATGGACAAAATCCCAGAGCTTCAAATGCCTATGATGATCACTGATTGGACCCAAGACACATTTTGGGCTCAATTGAATAGTCCATGGCTCCAAAACTATACCTACTCCAACATATTAAACTTCTAG

>AiNAC62--Araip.PX0QP

ATTGGAATGAAGAAGACTCTTGTATTCTACACTGGCCGTGCTCCCAAAGGGAAGAGGACCAATTGGGTGATGCATGAGTACAGGCCTACCCTGCAGGAGCTTGATGGTACCAATCCTGGACAGAATCCATATGTCCTTTGTCGATTATTTAAGAAACAAGATGAGAGTCTTGAAGGTTCAAACGGTGAAGAAATGGAGCGTACTACTTCAACTAATTTAACTGCAAATTACTCTCCAGAAGAAATACAATCAGATCCAGCTGTTAAATCGGTTTCTTCTTCACAGGCTACAGAAGATGACAAGAAACTAGCAGTTATCCCTTTGACCCCTGAAGAAGCAATTTCCAATGTTATAACCCCGGTCGGTTGCCAAAACGATGGATGTGATGCTTATGATGCACAAAATCAAATCGCAGCAGGAGATCCATCTAAGGAGGAGGACTTACAAGTGAACATGGACATATTTTATGACCCGAGTGAGCTATTTGACGATAAATTATTCTCCCCACTCCACAAGCATATTCCAGAAGAACTTTTTCATCAATCAAACAATGAAGCCAATGGACATTTTGGGCTGCAACATCAGTGTGGAACAAATGAGATCAGTATTTCTGACTTCTTTGACTCTGTTATTAATTGGGATGAGATCTCCGGTGACAATTCCAGCGGCCAAACGCCAAACTCTGCTTGGTTTGATGTACAGCACAATGAATCATGGGGAAACTCAAATGTGGATATGGTCCATGCCAGGCCCCTACAAGTAGGGGGTGCAGATTATCCAGGGGATGCAACCGAGGGAAAGCTCCCTTTGTTGAAAACTAGAGAATTCAATCCCAACACCTCTTATGACAATGCGCTCAGCAACAACATGGGATTATTTCATAACCATTCCCAGATGGCTTTTTCATCTGATGTTAATATGCTCCAAGGTTACCATGCAACCAACAATTATGAGCAACCGACAAACTTCAATATGGCTATGGCTAATAGTGACAACACTGGAATTAGGATAAGGTCTCGGCCACCAGGTTATGAAGGGCCAAACGTAAACTCCAATATGCAACCACAAGGTACTGCACCTAGGAGAATACGGTTGGCACGATCTCTTGCACCTCAACACATGTCCAATGAGGCGGCAAAAGATTCGAGTTACGAGTCAAAAGATCAAAATTCACAACTAACCACTGCCAGGGAGATGGAAACTTCCAAAGACCTTGCTGCTGGTGAGAGTGTTACTGTTACTAGTGATGTGGAGGAACAGGAGACATCACCAGTTGAAAATAAGGAATTCGAAGACTTCAACACAGTCCAGCAGAGCACATCATCAGCTTCCTCCAATCTTTCCACGTGCTCTTCTGATTCTGAAGTTTCTTATGAGGCAGAAAAAGAATCTGGTTGGACATCAGAAGACCATAGTCCAAAACCAGCTGCCGCGGGGGCCAGTAAAGCTTCCGAAGACCAAGTTCCCAGCGAGTGCGTCAATGATATCACTGATGATGTGGATGAACCCAGGATACCAAACGCTTATACTCTAGAGGTCTCAAAGGAGGAATCCTTCTCGGACTCTCAGTCGAAAGACTCTCTATTGCGTAGAAAGGTGTGTTACCCATCGAAGTCTTCCTCAAATCTAGCCAAGTGGTATTCGGTTATTGCAGTCTCAGCCACTTTGGTGGTGTTACTAGCATTCCTTGTTAATACATGGGGTTATGGATATTACCTTAAAGTTTAACTGCATAGTAGGATATGTATCATTGCATAGAGTATATTTTGCCTCCATTGCGTTTTTGGCCTTAGTTAAGGAGGCATATATGTAAGGGTTGACTTTGTAGCTTTCTTTGAATATAATCTGTATGTGCATATGTTATATATAGCAGAATATGCCTAATCTGCTACTAGCTTGTACTATATGTGGAGTTGTAAACTCATGTTTATAAGTTAGATTCAGAATTTCAGGGTTTCCAATTTTTTGGAATTTAATTTTGATTGCCCGTGCGTGACCACCATTGGAGAATATGCCTGCCTAGCCAAGAGTGTATTTTTAGTAAAGAAAGGTTTAGGCAGATTCGGCTGT

>AiNAC63--Araip.Q1JTJ

CCTTCTTCAAACCTTTTTCACTTTTTTTTTTCTTTTTCACCAAAATCCTCGGGATCTGATCTTCACTACTCTCTTTCTCAAGTTCAATTCCCGAGGATTCATATTCATAACAAAAACTACTACTACTTTTCTTATTGTCTCTCTGTTCTTTTGTTCGCTTAATTACCTTTCTTTCAGTGCTGTTTTCTTGTCTGCTTCTCTTTCCATTCCACAAATTGTAGTGTCACTACAAAAAAGCCTTTCAACAATTATCAATAACTTCCCATCAAAACCAAGTTTTAGTGCGTGTTTACTTAGATCGCTACCGGATTCTATTTGGGGAAGGAGCAACATAGATCAAGGTTTTTGGTTGTTTTGGGGGCGACATGAACACCTTCTCCCACGTACCTCCAGGCTTTCGTTTTCATCCGACTGATGAAGAATTAGTTGACTACTACCTTAGGAAAAAGGTAGCATCCAAAAAGATTGATCTAGATGTCATCAAAGATGTTGATCTCTATAAAATTGAGCCATGGGATCTTCAAGAACTATGCAAAATAGGAAGCGATGAAGAAAATGACTGGTATTTCTTCAGTCATAAAGATAAGAAGTACCCAACAGGAACAAGAACGAATAGGGCAACAAAAGCAGGGTTTTGGAAAGCCACGGGAAGAGATAAAGCAATATACTCAAAGCAGCATTGCCTTATTGGAATGAGAAAGACTCTTGTCTTCTACAAAGGAAGAGCTCCTAATGGCCACAAGTCTGACTGGATCATGCATGAGTATCGCCTTGAAACCAATGAAAATGGAACTGCTCCGGAAGAAGGGTGGGTTGTATGTAGAGTGTTCAAGAAGAAAATGGCAACAGTGAGGAAAATTGGAGACTATGATTCACCATGTTCTTGGTACGATGAACAAGTTCCCTTCATGCAAGATCTTGAATCCTCATCCCCAATAAAGCCACCAATAATTAACAACAACCATTATGCTTCTTCATACAACTACCACCAGTTACAATTACCCTGCAAACCGGAATTCCATCAACTTATGCAATACAACAACATGAACATGCCACGTCACGACGACGCTGCTGATAATAACAACAACTTCCTCCAACTTCCTCAGCTTGAAAGCCCTAATGGTGGAATTAGCCCCTTCTTGCAACAACAAGATCATCATCATCAGCTATTGCAACAACAAAATTCCAACAGCAATTATCATCTTGATCAAGTAACCGATTGGCGAGTTCTCGATAAATTCGTTGCGTCGCAGCTCATGAGTCATGGTCATGATGATAATGATGACGATGGCCACAACAATAATAATAATGTTTCCAAAGAAGTAATAAACAGTTATTCTGATGCTTCAATTCTCCATGTGGCTCAACAGATTGCTATGCTGGCAAATGGATCGTCATCTTCATCATCATCAAGGAGGCCTCAAATTTCTCATCAGGAATATGCTGCTTCAACTTCCACATCAAGTTCTCAGATTGATCTCTGGAAGTCATCGTCATGA

>AiNAC64--Araip.Q3R6H

CACTAACATATTAAGGCAGAAAACCACTGTGTATTGTGCTGATGAGGTGTTAAGCAGATGATATAATTTTAATTTGGTAGAGTGGTGCCGCTAGGTGAAGGTCAATTTCAACGAGTAAAACCAATTTGGTTGGTTTTCTCTGTCTTTTCTTACAAACTTTACATGCAAGTGATTGGTTCCTCTCCCTTTCAATCTTCACATGTGCATGGGACTAGAGCCATATATATAATGTGGATGACACACTTTGGTTTCTTATTAAAAGCAAGCCAAAAATTCATATTGGTATCATCGGCACTTGTTGATTCCTTTGTCAAGAACAAAGAAAATTCTTTTCATGGAAGAAGGAGGAGGAGATCAACATGCCTCTAACAGCAGCTACACTTTTCCACCAGGTTTCAGATTCCACCCTTCTGATGAAGAACTCATAGTTCATTACCTACAAAACAGAATCAGCTCTCGTCCACTTCCAGCTTCCATTATAGCTGAGATTGATCTTTATAAGTATAACCCTTGGGATTTGCCAAAGAAGGCTTTGTTTGGAGAGGAAGAATGGTACTTCTTTAGCCCGAGAGATCGCAAGTATCCAAATGGATTGAGGCCAAACAGGGCAGCAGGTTCAGGGTACTGGAAGGCTACCGGAACTGACAAGCCGATTCTCACTTCTTATGGATCGAAGCGCATCGGAGTGAAGAAAGCTCTTGTCTTCTATTTAGGTAGACCTCCAAAGGGGACTAAAACTGATTGGATCATGAATGAGTATAGATTGGTTGACACAATCACCAGCCCCTCCAGGCTCAAAGGTTCCATGCGCTTAGATGACTGGGTACTCTGTCGCGTTCGACACAAAGGCTACTCATCGAAGAACTCATGTGAGAATCAAGATAATCCTTGTGAACCAAACATGCTACCAAATCTGCCAAGGTGTGATGAAGGTTATCCAGCAACAAACATGAACTTTCATGCTGATATGATCACTGATTATCAATACAAAGACTATCAGATCCTAGCTTCTATTCTTGTTGGTGGCCATGTTCCTACCACTGAGAGCATGTCAAGTTTGAACTTGAAGGATGGCAAAGGCAATGATCCAATAACTTCAGTTCATGAAGATGGTTTCCACAGAGAAGATTCTTCTACAACAGTTTCTCCTTTGGACTGTTACTTCAACTCACTGAAAAGAAAATCTAATGAGGATAACCAATATGAGAATCTCATTTCCTTTAACAGGAAGTTGAACATGGAGACCGCAATGGATGATGAATCTTCTATCATTAATGGAGGTTTGAACTTCTACAATCAAAACCAGTCTCAAGATGACATAATATTCAATAAGAGAGCAGCAGAGCCTAGCATCAACTTTCAAGAGCTAAAGCAATCAGCTTTTATAGGAAGATACCCACAATGCTCAAGTGATTGACCATAAGACATATTCAATACTCTCTATTCCACTTGTATATTTTCATGGATATACAACTAAGAATATAGGCCTCAAATATTAAAGTGAATTATTGTTCTTTAAGCGTGGTTGTAATAAATATATATAGAACTTTTCTATATACTCATGAAATTCCTTGAAAACATTGTGAATAATGCTAGCTTGTGCAGAATCTGCACAATGTCATTTCAACTAGAATTTTATCCTACTCAGTAATAACTAATGATAAAACTTGTACAAACAATTTATATTCTTCCCACAAAATAGTTGC

>AiNAC65--Araip.QS7JY

ATTCATTAATTAACAAAAGTAAAATTAGAGATATTATTTAAAATTTTATGATCCAAGATAGTAAAATGAGTTATTGGTTGTTTTTCAGGAGAGAGCATCAATGGATATGGAATCATGTGTGCCTCCAGGATTTAGATTTCACCCAACAGAAGAAGAACTTGTGGGGTATTACCTCAAGAGGAAAATTAACTCCCTCAAAATTGATCTAGATGTTATAGTTGAGATCGATCTCTACAAAATGGAACCATGGGACATACAAGATAGATGCAAGCTAGGGTATGAAGAACAAAATGAGTGGTACTTTTTCAGCCACAAAGACAAGAAGTATCCAACAGGAACAAGAACAAACAGAGCCACTGCAGCTGGATTCTGGAAAGCAACTGGGAGAGACAAGGCTGTTATGTCGAAGAACAGGATCATTGGTATGAGGAAGACTTTGGTCTTCTACAAAGGACGTGCCCCTAATGGCCGCAAAACTGATTGGATCATGCACGAATATCGCCATCAAACCTCTGAACATGGCCCTCCTCAGGCAAGATGGGTTGTATGTAGAGCATTTCGAAAACCAAGTCCAAGTCATCAAAGGCAATTAGGTTATGATCCATGGTGTAGTAATCATCATCATCAACCACATTATTTCAGAGATCAGAGTAGCTATGATCATCATCATCAACAAGAACAACAAGAGTTTGTAATATCAAATAATCATCAACAACTCATTGAGCTTCCACAGCTAGATAGCCCTACTAGTGCTTCTCTCTCAGCACCACCAACAACAAGTTTTGCAGTCAAAGAATCATCTTCCATTAATAATAACAATGAAGAGTATTGCAGTGATGAGAGGAACAACAACAACAATATTGATTGGAAAAGCTTGGATAACTTGTTTGCTGATACTTCTAATTACTTCTCAAATCCAAACATGTCCCAATTCATGACCATCAATCATCATCTAGGTTGTTTCCCTGGTTCATAA

>AiNAC66--Araip.R0657

GCACGATAAGAGAAGAAGATTTGTTTAATTAATTTGTCAATAGAACAAATTAAAGTGGATGCATGCTATCATATATACTATATATGTGTCGCTTTTCTCTTGTATAAATATAGTTCCATGTTAGCTTAACTGAATTTCATCGATGTGCATGTATAGTATCAGAGAGTAGAGAGAATATAATACAATCAAATAATGGAAAAGTTAAATTTTGTGAAGAAGAATGGGGTAAGTAGAATGCCTCCTGGATTCAGATTCCAGCCAACGGATGAAGAGCTTGTGTTTCAGTATTTGAAATGTAAGGTCTTCTCATTCCCCTTGCCCGCTTCCATGATTCCTGACATCAATCTCTCCAACTATGATCCTTGGGATTTGCCAGGAAATTGTGATGAACATCAAGAGATGTATTTCTTCAGCAGCAAGGAACCCAAGTATAGAAATGGAAGCCGCATGAACCGAACAACCACCTCTGGCTATTGGAAGGCAACAGGATCCGACAAAAGAATCATTTCATCTTCTAATAATAGTGACGATAATAGCATTCTTGGCATTAGAAAAACCCTAGTGTTTTACCAAGGGAAATCTCCCAATGGCACTAGAACTCACTGGGTCTTGCATGAATATCGCCTGGTTAGTACTACTCTACATGCTAATAACAATGCTTGCGATATAGGAGATTGGGTTCTGTGTCGCTTATCGGTGAAGAAAAGGAGTGTTGGGAGTGGTAGTATCATCATAAGCAAGAAAGCACGTTCTTCACCATCTTCATCCTCATCTTCTTCCACTTCAAGTAATAACGTCATGGAAGTATCTTCTTCATATGCTTCTTAATATCAACAATGCACTTCCTCCTCATGGTCTTGCTGACTATTGATTAAAACCACCATACATATTCTCACTCACAACCCGTCGTTTCTGCAACTAAATGGGTTCAGACCCTTTAAAATTAACAA

>AiNAC67--Araip.T6ICI

GTTCAAACTCCTTAGCTTGTTCATCAAACCAACTTCAATTTCACATAACAATATAATGGAAGGAAGTAGTAAAAGTTGTGAACTACTACCACCAGGGTTTAGATTCCACCCAACAGATGAGGAGCTAATTGTGTATTACCTTTGTAACCAAGCAACATCAAAGCCCTGCCCTGCTTCCATCATCCCTGAAGTTGACATCTACAAATTTGATCCATGGGAATTGCCAGGGAAGGCTGAGTTTGGGGAGAAAGAATGGTACTTCTTTAGCCCAAGGGAAAGGAAGTATCCCAATGGGGTTCGGCCTAACCGCGCAACAGTTTCTGGGTATTGGAAGGCCACAGGGACAGACAAGGCTATTTACAGCAAGTGTAAGCATGTTGGTGTCAAGAAGGCCTTGGTTTTCTACAGGGGTAGACCTCCAAAGGGGATCAAGACTGATTGGATCATGCACGAATATCGTCTTCTTCAACAATCTAATCACAACAGCAGGATCACTGGTTCTATGAGACTGGATGACTGCGTCTTGTGTAGGATATATAAGAAAAAACATGCTGCTAAAGCATTGGATCAAGGACAGGAATACCCAACAACAGTTCAAATTAATCTAAATGCATCAACCAACAATGATGATGAGAAGGAGTTGATGATGATGAAGAATCTTCCAAGGACTTGTTCCCTTACTTATCTTTTGGACATGAATTACTTTGGTCCAATCTCACAGCTATTGTCTGATGGATCCTACAACAACTCATCAACCTTTGAAATATTTCAACATAGCAATAGTGTTGACAACATTGGAATAGTGGATCCTCTTGTCAAAACTCAAATGGTTGAAATGGATGATAGCTATTATGCTCAAGATTCAGGCAAGTCCCAAGTGATGAAGCAAGGGAATGATTTAAGAGGATATTACTAA

>AiNAC68--Araip.TL0B5

ATGCTATCCGGGTTTAGATTGTTGTTTTTTATTTTGAGAGTTGTAGTTCATATTTGGTTAAATTATATTATAGAACAATCGAAGGTGAAATCGGATGATCAAGAATGGTTTTTCTTCAATGAATTGAAACACATAAAAAACAAGTGGTGTAACAGAAAAACTAACGCCGGTTATTGGGAGATCACCGGAAAAGAGCGGATCGTCAAAGGAACAGGGACAGACAATGTGATAGGTACAAAAAGAACACTAGTTTTCTACAAGCGTCCACATAGTGTCAAAACCAATTGGGTTCTTCATGAATATCATGCACTTGATCAAAAGAGCAACATCGTGTTGAGCCGTGTAATAAAGAATGTTGAGAAAAGGGAAAAGAAGGTTAAGAGAAAAGCAAGCAACATAATCGAAGAGGAAGTAACATGTGAACCATGCAGCGAAATGACTGGCTATGTTACCGAAGCAACTACAGAAGATGCAATAATTCCTGATGCATGTGTTTCATCCGAACCGCAACCACCTCAAGATATTGACTATGAAATTCTTTCGCCGGGACAACAATCTTCGGTGGCCCATTCCGGTAATGGAAGCAACAATGCTCCGTTGCTTCCATTTGAAGGTATGTGGAAGCAAGATGCCGAGATGAACACGGACATCGATGCTGACCTTGATGCGGAGTTCTTGAATTCGGTGTTGGCAGGGGATGATTAA

>AiNAC69--Araip.U9RGH

CTACCCTCATCCCCATGTAGGGCCTGAAAGCTATGTAGATCTTTCTATTAACTCCCATCCTCTCTCTCCTCTTTTTTCTCAACACCTATTTAAAACACACGCCCCATTTTCTCTTCTTTTTATATATTAACTCTGCCTCAAACACCCAGTTTAATTTCTTCAATTTCTACATTTTAACCATCAATCTCTTATCTCTGCCTAACACCACTGCACCACCCAATTCGTACAACTATTACACTCTCTAAGTATAAAGACAGAGAGAGAAATTAAAGGGGGTCCAAACTCGAAGCACCAACTACTACTACTACGATAGATATAGTATTATTATCAGTGTTGGATGGCAATTGCAGCGCCGAATTCATCTCCGACGATGAGTCTGAGTCACAGCCACAGCCACGAGGACGGAGGGACAACGACGGCGGCGTCCACCACGAACGACAACCTGAACGGGAACGGGAAGCAAGAGGATGATGATCACGAGCATGACATGGTGATGCCGGGGTTTCGTTTTCACCCGACAGAAGAAGAGCTGGTGGAGTTCTACCTTCGCCGTAAGGTGGAGGGCAAACGTTTCAACGTTGAGCTCATTACTTTCCTTGATCTTTATCGCTATGACCCTTGGGAGCTTCCTGCTTTGGCGGCGATAGGAGAGAAGGAGTGGTATTTCTATGTGCCTCGAGACAGAAAATACAGAAACGGAGATCGTCCGAATCGAGTGACGACGTCGGGTTATTGGAAGGCAACGGGAGCAGATAGGATGATAAGGACGGAGAATTTCAGGTCCATCGGCCTCAAGAAAACCCTAGTTTTCTACTCTGGGAAAGCTCCTAAAGGCATCCGTACAAGTTGGATTATGAACGAGTACCGTTTGCCCCAACACGAAACTGAACGATACCAAAAGGCGGAGATATCGCTGTGCCGGGTTTACAAGAGAGCTGGAGTTGAAGATCATCCATCGTTGCCGCGGTGTCTGCCAACAAGGGCTCCATCTTCAAGAACTGTTGATCACCAGAAGAACAAGCAGCAGCCTCACAACGATCAACTCAACATGGGATTTGGGGGGAACACCGCCGATGGAGCTTCTGATAATCGTGATCATGATGTAACCACCGCTCTCGCCCTCTCCAAACACAACACAAATAATACTAATAATGCTTATCGTGCTCCTTCAATGGGACTCCCACCGCTGCTTCTTCCCTTGGACGACGAAGCCGCCTTCGTCCTCATGCAGCAGCAGCACCATGCTGGCCCTTCTTCCGGCACCACCATGATGGATGATCTCAACAGGCTTGTAAGCTATCAACACCAGTACTACAACAGCAGCAGTAGCAGTAGTAACAATAATAATCCCAATCATCATCATCACCTGTTAATGCATCAACAACAACAACAACAACAACAACAGCAGCAGCAGCAAAATCCTCCTGCAATAATGTCTCTGAATAACACTCCTTCTCCGCTTGCAACCGCCTTCTCTGACCGCCTGTGGGAGTGGAATCCACTCCCGGAGGCCAACCAGCGGCAGTACAGCAACATGTCCTTCAAGTAA

>AiNAC70--Araip.UA0W9

GTATTTAAGCACACCTTCTTCCCTTCATTATTTTACTTTCTTAATCTTTCATCACTTCATAATTAATTGCTCATCGCTATGGATGCGGCTTTGGATTTGCCTCCCGGTTTCAGGTTCCACCCTACAGATGAGGAGATCATCTCTTATTATCTCACTCACAAGGTTTTGAACACAAGTTTCACCGCAACTGCCATTGGAGAAGTTGATCTCAATAAGTGTGAGCCTTGGGACTTGCCTAAGAAAGCAAAGATGGGGGAGAAAGATTGGTACTTCTTCTGGCAAAGAGATAGAAAGTATCCAACTGGGATCAGAACGAATCGAGCCACGGAATCCGGCTATTGGAAGGCCACCGGAAAAGACAAAGAGATTTACAAAGGGAGAAACCTTGTTGGTATGAAGAAAACCCTTGTGTTCTATAGAGGTAGAGCCCCTAATGGACACAAAACCAATTGGGTTATGCATGAATTCAGATTGGAAGGCCTTTTTGCTACTTACAACCTCCCTATACCTGCTAAGGAGGAATGGGTTGTGTCGAGGGTTTTCCATAAGAACACAACAGAAAAATTGAACCCAACTATTCCATCTGGCCTCTTTAGGATAATGAAGAACATGAACTCAATTGGAGATGATGATCTTGTAGATTTTTCTTCTCTCCCACCTCTCATGGATCCTTCTAATAATTATGATGATGAACACACCACCACCACCAACAACAACTATGTCAATAGTATGTTTGCATCATCATCAGATTATAATATTACTATTCAGCAAAACAAGAAAGATATGATGGGAATAAGGAATAATAATAATAATATTAGAGCATTGTTAATGTACGACGGTCCATCATCATCATCATCAGAAGTAGTTGCTCCTCCTCTCTCTGACTTGGAATTAGGCCTCTGGGATTTATAACTTCTTACCAAAACATTATTAAACATTATTTCTGATGATAT

>AiNAC71--Araip.WV14F

ATGGAGGAAGAGGATGATGTTCCACTTCCAGGGTTTAGATTCCACCCAACAGATGAAGAACTTGTGAGTTTCTATCTAAAGAGGAAGCTTGACAAGAAACCAATCAGCATCGAACTCATCAAACAGATTGATATCTACAAGTATGATCCTTGGGATCTTCCAAAAGCGAGTGGAAGTGGAGGAGAGAAGGAAGGTTACTTCTTTTGCAAGAGAGGGAGGAAGTATAGGAACAGCATAAGGCCTAACAGAGTCACCAGTTCCGGCTTCTGGAAAGCAACCGGGATAGACAAGCCGGTATACTCCCATGGAGGCGAAGGAACCGACTGCATTGGACTCAAGAAGACGCTTGTCTACTACCGCGGCAGCGCCGGTAAAGGTACCAAGACTGATTGGATGATGCACGAGTTTCGCCTCCCTTCTGCCACAACCGAAAACAACACAAGCCTACTTGCCAACAACAAGAATAATAATAATATCAACAATGCCGATGTTGCCCAAGAAGCTGAAATCTGGACATTGTGTAGAATATTCAAGCGAAATGTGTCACAAAGGAAGCACACAGTAGACTTGAGATCACATTTAGTAACAGCTAATAGTAACAAGCACAAAACCACTAGAACCCATGTTGTTCAATCCAATAATAACAATATTAATCAACATCAAGAATCTTACATCAACTTTGGTGCAACAATCATTGGCCATCACCATTACCATCATCATCGTCATCAAAATGAACAGAAGCCAGTGACTAACTACACAGCATGCAATAACAACACTGATCAAATCCAAAGGAACAATAGCAATCATCATCATCATCATCAGTTGAACTATCACCCTTCTTCAGCGGTGGCTACTACTGTGCCACAGCAACAACAACAACAATATCATCATCATCATCAGCTAATGACGGCTCCAGCTTCTAACATGTGGATTAATCCTTCTGCGATGAACGATTTGTTTGCATTTGATGATGACTGGGATGAGCTTGGATCTGTTCTCAAATTCACTGTTGATACCCCTAGCTTGTAA

>AiNAC72--Araip.X2KK1

TCCTTATTTCTATCAATTTTTTTTCTGTTTCCAACTCAATTTCACATTCTCCATACAAAGTAACTACATTACTATTTAATTTAGTTCATCTTAACTTCTGGGGCTTTCAAAAGCTTCCAACTTTTTTGGAAAATCTTTCCTACTCAAATTTTGGACTTGTGCCCATTTTTCTTTTGTGAAAGTTGGTATTTGGGTTACACTGATTTGAACATCTGGTTACTTACACCTCAGTTTGGTTCATTTTTTCTTAACGGGGATTAATATCAAAGGGGTGTTGGTGGGGGAAGGGTGTGATAGCTTCATAAGAGACCTCAAATTTCTTTTTAAGACCATGATTCTGGCACACAACGGGTGCCTTCTGTTGAATTCTCGCCTTATTCTTCACAAAGAATATATCATAGACAAAAGTTTCTACTTTTGGATCTTTCAGATTTTTAATAGCTATAGTCTTGGTACCTAACAGGAAAATTCTATATCCTTCACCAACATTCTTGAAAATACTATCTTTTTTGTTGTGTTAAATTGAGACCGGAAGAGACTAGAAAACTGCATATACCTTTGGTTCCTTGTTTCTCAATTATACACTTTCCATTCTGTAAGTGAGAATGGGAGGGGCATCACTGCCTCCGGGTTTTCGTTTCCACCCTACTGATGAAGAACTGATAGGATACTACCTGAAAAGAAAAGTTGAGGAGCTTGAAATTGAACTTGAAGTTATCCCTGTGATTGATTTGTACAAGTTTGATCCTTGGGAGTTGCCGGAGAAGTCATTCTTACCCAAAAGAGACTTGGAATGGTTCTTCTTTTGTCCAAGGGATCGAAAGTATCCGAATGGATCAAGAACAAACAGAGCTACCAAAGCAGGATATTGGAAAGCCACTGGAAAAGACAAAAAGGTTGTGTGCCAATCTAGTCCATCAACATCAATCATGAAAGCCACCGGATATCGCAAGACCCTTGTTTTCTATCGGGGAAGAGCCCCTTTAGGCGACCGAACGGATTGGGTTATGCACGAGTATCGCCTCTGTGATGATCTTGGCCAAGACTCACCAAGTTTTCAGGGTGCTTATGCTTTGTGCCGGGTTATTAAGAAGAATGACAAGGCCAGTGATTACAAGGGTAAAAGAGGTGTCAGCAGTTCCAAGAATGAGAATGAGACCTCAATGAGATTGTCATCCTCTAAGGAGCACTTGAGCATCTCTGCTGATGTTTCTTCTCAAGCAAGTCAGCTATGCAGCGAGAGTCGTTATTCGAGCCCTATAGCTTCTCCTTGTGCATACAATGTGGCTGCAACGGCTGTGTTTGAGCCACCTTCTGTGGACACTAATCCTTCAACCTTCTTGGTCTCCCCTGATATGATTCTTGATTCTTCAAAGGACTTTGCTCAAACACAAGATGCTATTTCAGAATTCCTTCCGCATCATGAATTGCTAAGTACAATGACACCATGGCAATCATTGGAACATACAGAAATTTCATCCAGTTCATCCTACTCAAATTTCAATGGGGAGATAGAATTCTCTGATGAACTTGGCCTAATTGGCCGAATGTCGCGTTACTCAGGACAAGTAGACATGTTAGACTTCTATGGAAATGAGGAAGTGCTGTATGAATATGAAGGATATGACCAGATCAATTCAATCAGAGATCCAAGACAATTCTGAAGTGAAAAGGAAATATATGGGAATGAAAGTAAGGGAGATCTTGATGATGGAGGGATCTCTGAGGCAACGCCTTTTAATGTGTGTCTTGGAGTGAAAAATAATGAGTACAAGCTCCTTCTTCTGCACAAATGCTCTTAGCTATGTTTATCATTTTATGCAGTTGTGTAAGATGCTCGGGTGACATCACATTTTTTAAATTGCACTCGGTGTTTGGTCTTAAATTCATTCACCTTATGTAATGAAGGAGAATTTCTTATGTTTCTTATATTCATTCACCTTTCTTTACATACTAACTTAGTAACTTACATGAA

>AiNAC73--Araip.XJ3T4

ACATTTGGTGGAACCTTGAGAAACACACTTATCCATGGTTTTTCTTTCTTCATTTGCCTTTGTATACTTAAACCCTAATTAACTCTAGTTTCTTTCATGCATAATTGATTAGTAGTAGCAGTGATAAGGAAGCTCCATACTCTTCCCTCTCTTTTAATTTGATCTTATTATTTTGTGGTAACCCTCAAGTCAAGTGTTTTCTTCTCAAAGTGAATCACAAAAATTCAACCCAAAATTGAACAAAGAGTTTTGTCTCTCCATGCAAAAGGAAAAGGAAGCAATCACCGCCAATAAGGAGGGCACTAATAAGAAGGACATAGGAATAATGGAATGTTGCCATGGAAAAGAGGAAACCCTACCACCTGGGTTTCGATTTCATCCAACCGACGAAGAACTCATTACTTGCTATCTCATAAACAAGATCTCGGATTCAAACTTTTCAGGCAGGGCAATAACTGATGTTGATCTCAATAAATGTGAGCCATGGGAGCTTCCAGCCACAAACACGGGGTATTGGAAGACCACCGGAAAAGACAAAGAGATCCTTAATAGTGTTACATCGGAGCTAGTTGGGATGAAGAAAACTTTGGTTTTCTACAAAGGAAGAGCCCCAAGGGGAGAGAAGAGTAATTGGGTCATGCATGAATATCGCATTCATTCTAAATCCACCTTTCGAACAACCAAGGATGAATGGGTGGTTTGCCGTGTGTTCCAGAAGAGTGCCGGTGCAAAGAAGTACCCTTCTTCCAACCATGCAAGTAGGGCAATGAACCCTTTCAACCTTGAAATAGGTCACCACAATATTGTGCCGCCGCCGCCAATGATGCAACTCGGAGACCCCGCCGCCGCTCATTTCCTCTATGGAAGGAACTATATGAATACTGCAGAGTTAGCAGAAGTAGCTAGGGTTTTGCGTGTTGGTACTGGATCAACCAGTACCAACCTACCCGGGATGCAGCCTCAGATAAATTATCCAGTGGCTGCATCATCCCCAGGAGTTGGATTCACAATTTCAGGGCTCAATTTAAATCTAGGAGGCGGAGGAGGCGGAACAGTAGTGGCCACAACACAACCAGTTTTGCGGCCCATGCAGCCGACTCCTCCGTCCCAAACATTGGGTATGGTTCCTCATCATCAAGTTCATCATGATGTGAGTTCCAACATGATTTCTGGTGCTGAGAATGTGGGTTACGTCAATGAAATAAGCAACACAAATGGTGGTCATGGAAATAGGTTTATGGGCATGGATCATTGCATGGATCTTGATAATTACTGGCCTTCCTACTAAATAAAGGAGAGAGACCCTACTTCATTTTGTTTAATTAATTATTATACATAATTTAGTGTTATAAGCTAAGTTGGATAATTAAGCTACGTAAAAC

>AiNAC74--Araip.XJX1I

TAACCATTATTGCTCTATGGCTCCAATGAGTCTCCCACCTGGTTTTAGGTTTCACCCCACAGATGAAGAGCTCGTTGCTTACTACTTAGAAAGGAAGATAACAGGTCGCTCTATAGAGCTTGACATTATAGCTGAAGTTGATTTATACAAATGTGAACCATGGGATTTGCCAGATAAGTCATTTCTACCAAGCAAGGATATGGAGTGGTATTTCTACAGTCCAAGGGATAGGAAGTATCCAAATGGATCAAGAACGAACAGGGCAACAAGAGGTGGGTACTGGAAAGCGACTGGAAAGGACAGGGCAGTGCAGTCTCAGAAGAAGGCAGTTGGTATGAAGAAGACTTTGGTGTATTACAAAGGAAGAGCTCCACATGGAATTAGAACCAACTGGGTCATGCATGAGTACCGCTTGATTGAATCCCTCCCTGGTACTCCTCACTCCTCTTTCAAGGATTCCTTTTCATTGTGTCGGATTTTCAAAAAGACAATTCAAGTTCAAGACAAATCTAAAGAAGAGAAAGAACATCAAGCATTACTAGAGGAAGATCACTCAAGAACATGTACACCCACAGAAACCGGTATAGCAGATGATTTTCATGCCCAATTTGCTTGTGATGAAGCAAACAGTAGTGCCGCTAATTCTTACTCAATGGGAATAGCATACCCCTCAAACGACATAGAGATGTCTATGTATGGAAGCATGCATAATTATCAATTCCCACAAACGCCTTTGGTGATGGAAGATTTTCCACAAATAGATTTTGCTGAGACAAAGTTATTGAAGCCAGAGGTGACTGAAGATTGCATGTTCTATGATAGATACGGTAGGGATTGTATGAATGGAACACTAGAAGAAATCATCTCATATTCATTTGACACAGAAGAAACTATAGCTACTTGTGTTGGCCAAAATAACCACACACAAAAGGAGACATTTTTTCTAGCAAGAGAGAAGCAGCAGCTACATATATAA

>AiNAC75--Araip.XK9AB

ATAAGAAAACTATATACATATATGGTTATGGTGTGAACTGGGTTTTGAAGTGGTGGGCCATTTTTCCTTGGAATTTTTGGGAGCACTTTGGGACAAGTTATCTCATACTTTCGGTGTGTTTTGTGCAATTTTGGACTGTTGGTTCCCCTTCCCTTCTCTCCCTCCAAATGCTATAAATATCACTCCACCCATGCCTTATGCTTTTACTACTCAAATTGTTAACATACTACTCAAATTAAACTTGTTTTGAGTTTAACCAATTTGTTTTCCTTAATTTTCTTGTTTTGCAAAAAATAATTTATTAGAGGTCAAAAGTAGTTGGTGGTTGAGTTAGAGTAATTTGAACTTTCTGGGTAAGTTAGTTCATTTTATCATTTGAGGGTGGGGTGGTTTGGGATTAGGATAGTGGTAGAGATAGTGTTATTCCAAAGAGAATGATCATTATTGCATACAATGGTTGCCGACTACTGTGGAAGTATCACTTGAAAACTATTGTTATGCACAAAAGCTTCTATTTTTGGATCTTCCAAATCTTCAATACATATGGGCATGCCACTTAGCATCATAAAGTTTTGAGCTTTCTTGCTGGAAAATTGTGGGTTTATTTTTTTGGTTACTCTCTTGCTATTAAGTTTTCATAAACTTTGGTTGGTCTTTTTTGTTCTTTTAATCATAATGGGAGGGGCATCACTGCCACCTGGATTTCGTTTCCACCCAACAGATGAAGAATTATTGGGATATTACCTAAAAAGAAAAGTGGAAGGGCTTGAAATTGAGCTTGAGGTTATTCCTGTGATTGATTTGTGCAAGTTTGATCCTTGGGAATTGCCTGAGAAATCATTGTTGGCAAATAGAGACATGGAATGGTTCTTCTTTTGTCCAAGGGACCGCAAGTACCCAAATGGATCAAGAACTAACAGAGCCACCAAAGCTGGTTATTGGAAAGCCACTGGAAAAGACAAGAAAGTTGTGTGCCAATTTGATACTCCTTCCACTGTCACAGGATATAGAAAAACCCTTGTCTTCTACCGTGGCAGAGCCCCTTTAGGTGACAGAACTGATTGGCTCATGCATGAGTATCGCCTCGCCGATGATCTCGGCCTATCATCTACATGTTTTCAGGGTGGTTATGCCTTGTGTCGGGTTATTAAGAAGAATGAGAAGGTGAACAATGAGAATGACGCGTCAATGAGATTCTCCAATGAGCCCTTCGCCATTTCTGCTGATGCTTCATCCTCTCAACCAAGTTATTTGAACAATGAGAGTGTTTACTCAAGCCCCAATGCTTCTCCACACAATGTGGACTCTAACCAAGCTTCTATAAACACCAGTTCTTCATCAGAGTTTTGGGTGTCCCCTGATCTGATTCTTGATTCTTCAAAGGACTACCCGCAACTACAAAATACTTTTACAAGGTGTGACATACCAAGTAGTACAATGACACCATGGCTCTCATTGGATCAACCTGAAATTTCATCTAGTTCATCATACTCAAATTTTAATGGGTAACTTCGATTTTCCGATGATTTCAATATGATTGGCGGCATGTCACCTTACTCAATACAAGAAGATTTTATGTACTTTCATGGAAATGATGGGGATGTTTCTTATGGAAGTTATGATCATATTAATTCAGTTGAGTATCCTGAATACTTCTGAAAACAAAGAAATATCATTCTACAAGTTTTCTTGATATGCAAGGTGTCAAGACTGAAGTCGCTAGCAATCGAAATTTCAAGCACAAACATAGTTTAGATGTTAGCACCAAAGAGCGAGTTACGATTTGGAAAGTAAACTGCACAATTATAACATTATGGGGGTAAAAGAAAATCAAGGGTTATAAGGTACAATACTAGAATGATTGTGCGCATTACTATTTAAAGTGTAATTGAAGTTTAAAGGAGCTAAATCTTGGGATCAAATTCTTGTGGGGGATAGTTATTTTAGATGAATTTTATTTGGTGTGATCTGATAAAGATTGCCTAAGACAACTCTTTTTAATGTATGTCTTAGAATTAAGAGTGATAAATAATGTGTACAATACTCTTTTTATGATGTCATGTAGGATGCATAGTATCATGT

>AiNAC76--Araip.XQA0A

ATTTGTTTAGGGTTAGGGCTAGCTACTTCATTCTCTATACTTGATCCAACAACATCATAGGTTGCACAAGAATCAAAGTAGACCAACAAATCATTGAGGACATTTGTCTGAGTTAGGAGCTTCATGATCTTGGGAGGCAGATTTGTGACTGCACCACCCTCATCTCTTATTACCTTGCTTCATCTCTAATCAGATAGAGAGAAGATGAATACATTTTGTCATGTTCCACCGGGTTTTAGGTTCCATCCGACTGATGAAGAGCTCGTTGATTACTACCTTAGAAAAAAAGTTAATTCAACTAGGATTGACCTTGATGTCATCAAAGATGTTGATCTCTACAAAATCGAACCCTGGGATCTTCAAGAGCTATGCAGACTAGGAACAGAAGAGCAAAATGAGTGGTACTTCTTTAGCCATAAAGATAAAAAATATCCAACAGGAACTCGCACAAATAGAGCAACTGCAGCAGGGTTTTGGAAAGCAACAGGGAGGGACAAAGCTATATATTCCAAGCATGATTTGATTGGGATGAGAAAGACTCTCGTCTTCTACAAAGGTCGAGCCCCTAATGGCCTCAAATCTGATTGGATTATGCACGAATATCGTCTTGAAACGGATCAAACTGCGGCTGCTACTCCTCAGGAAGAAGGATGGGTTGTGTGTAGAGTGTTCAAGAAGAGAGTGACTTCCATTATGCGTAAGATGAGTGATCATGATTCCCCTTCTTGCACTTGGTATGATGACTCCTCTTTCATGCACCAACAACCAGATCATCACTTTGACAACTCTTGTTCTTCTTCATCAAAGCACCAACTAATCCCTAATAATAACTGTGATGTCTTCTACCAACAACACAACAACAACAACTTGCCTCTTCATCATCTTCCACTTCTTCATCAAAATAACAATAATCCAATCATGGCACCACCATTTGCTGCTATTAATAATAATGAAACTACTGCTTTTCAAGAACAAGGGAAAAGCTTAATTCATCATCAGGCACTACTCTATGGAAATTTAAATGAAGAGCAAGCTTCTTCTTCAGCTGCTGCTGCTGCTGCTGATTGGAGACTTGTTGACAAGTTTGTTTCATCACAGCTTAGAGAAGATCATCATGTCTCCAAACAAGAATTGATGATGCCAGAAAATACTAATAATAATAATAATGATAATGGTGCCTCAACATCAAACTCAAGCTGTCCAATAATGGACGTGTGGAAATAG

>AiNAC77--Araip.XT8UZ

CATTTATCTGTACCACAAAGTCCCATACACACTGAATACTCATCTATCTGATCCTACCCCACCCACGCATCACGCATGGATTCATGTCAACCCCAACTCCCACCGGGATTCAGGTTCCACCCAACCGACGAAGAACTCATCGTTCACTACCTCAAGAGAAAAGCTTCCTCTGCTCCTCTCCCCGTCGCCATCATCGCCGACGTTGATCTCTACAAGTTCGACCCATGGGAGCTTCCAAGTAAGGCCACGTTTGGGGAGCAAGAATGGTATTTCTTTAGTCCGAGGGATCGCAAGTATCCGAATGGGGCTCGCCCAAACAGAGCAGCTACGTCCGGATATTGGAAGGCCACCGGCACTGATAAGCCTATTATTGCGTCTGATGGCCAACACCGACTCGGCGTCAAGAAAGCTCTCGTCTTCTATGGTGGCAAGCCTCCTAAAGGGGTTAAAACCAATTGGATCATGCACGAATATAGACTCACTACTACTCATAACAACAATTCTATCTCATCATCAAAGTCTTTTCCTTCTCTTCCTTCTCATCTTCCTTCCGCCAATAACAAGAAGAATTCCTTGAGGGTATCTATCATTCATTTCCTTCCTACAAACATATTTGACTTGGATATTTCAATTAAAAATCTTGATGATTGGGTGTTGTGCCGAATATATGAAAAAAGCAACCGTGGCAATTTTGCAAGAACAGCGTTGATGGAGCACCATGATCATGATGATGATGATGATGACAATAAGGATCAGCTTTCCGCGGAAACAACGAGTATGATAGAAAACATGTCCACGATGAGTAGTCAGAATTCCAAGCCCACACAACATTATGGACCATTGCTGGTTCAAAACGATGACAACTTCTTCGATGGAATCTTAGCTGCTGATCATCATAATCAACAACACAACTTGCCAATGAAGAGGACACTGGTGAATATGAATAATTCACAGTTTTGGAATGAGACAAACAAGAGGTTCCATTGTGATCTCAATAACAACACTAACATTGTTGCTAATAATGATGAGGATAACACTTCCTTTGTTTCACTGCTTAGCCATAATCAGATTCCTCATCATCCTACTAACAATGCTTCTCTTCTTGACCCTACTGTTGCTGATGGTGTTTTCAGGCAACACTTTCAACTTCAAGCAATTAATTGGAACTTATAG

>AiNAC78--Araip.ZX5IX

ATGGCAGAGCTAAGTGCGGCCGCAACCTTCACACCCAGCGATGAAGAACTCATTCATTTCCTTTCCGACAAGGTGAAAGGCCAATCAATGGACGAGGACGCCGCCATCAACATCCACGAATGTGAATACTTGTACGGCCGCAACAAGAACCCTTGGGACATTTGGCGGGACTTCGCCGGCGACGTTGATGCCGGCAGGACCGCCCTTTTCTTCTTCTCTCCCACCAAAAAGCACCATTCCACAGCCTCTCGCCCCATCGGAGCCGGCGTCTGGGAAGCTGAAGCCGAAACCATTGACGGCGAAAGCATCGTTGGCAAGGGCAAGAACCGCCGTATTGGGACCAAGAAATGTTTCGTCTTTGACAAGAGTGGCACCTCCTACGATGGTGCATGGATCTTGCATGAATACACTCTTCATGGATCCTCGCTCCACACTAATACTTCAGTGGATCATAGCTATGTTATATGCAAATTGATAAAGAATGTAGAAGGTGAAGCTCATCCAGTTGAGGTGCAGTTTGGAGACAAAAGAAAAAGGCACGGTCAATCTGCCACCACCAGCGGCGTTCAAATTGATGTTAACGCTCCTCATTCATATAGGAACACTAAAGAGCAAGAGGTCCAATTCATACCAAATGAACTTGGCAGGCGAATGTTGTTGGAAATGTTTGAGGATGATGAGGATGGTTTAACTCTTTCTGATGGTTTAACTCATCAGAAACCTCATGCAGCTGCAAGGGGGAACAAGAAGAGGTGGAAAAGGCATCTAATTATTGCATAA

>AiNAC79--Araip.YS3WM

TATTCATCACCAATGTATTAACTAAGTCCTGTTAGATCATAAATCCTTGTCAAGATAACATGATCTGCTTTCACTCCTAATGAAATTGTTTCAATCTGGTGTCACACACTCCTTCCAAGTATTCTGTTTATAAATTATAATGAAATTATACATGATAAACACCAGCTTGGGTTTCACGTCAACACCCTTATTATAAATACCTTAGCTTCTCTATTTGTACTATAAATACCACTCATCTTCACCATTACTCTAACTTGACACATTCCCTCCTCTTGTTTTCTCTGCCTATCTTCTTAATGGGAGATAACAATGTGAACCTTCCACCGGGGTTTCGATTTTATCCAACAGATGAAGAGCTTGTGGTCCATTTTCTTCATAGAAAGGCAGCACTCTTACCTTGCCACCCTGATGTCATCCCTGATCTTGATCTCTATCCTTATGATCCTTGGGAACTTGATGGTAGAGCGTTGGCAGAGGGAAAGCAATGGTACTACTACAGCAGGAGAACACAGAGTAGGGTGACTGAGAATGGATATTGGAAAGCAACGGGAATGGAAGAACCAGTGATGACAAGCTCAACTAACAAGAGAGTTGGCATCAAGAAATACTTTGTGTTTCATCTTGGTGAATCCCCTTCTGCTATCAAAACAAATTGGATAATGCAAGAATATTGCCTTTCCGATTATTCTGCTTCCTCTAGCAGATCCTCCAAAAGAAAATCAGATTATAGTAAATGGGTGATATGTCGTGTTTATGAGCGGAATGGAGATGATGATGATGGAACGGAGCTGTCTTGTTTGGATGAAGTTTTCTTGTCACTGGATGATCTTGATGAAATAAGCTTACCAAATTAAATTAATCAAGCTAGCTGCATTAATTAAGATAATCCAAAATGGATAATTATTAAGTATATAGCAGTGTGATGTTGCAAGTATAGGTAGATTTAGATTTCTATAGCAGCCCTTTAACACAAGAGGAAGTGGGGCACTATGTGAAATTCATGACCAAACAATATGTACTATGACGTTATGCTTTCAAAGATTCCATTTATCATTTTACCACAAATTTTTCTCTCCTCTTTTCTCCATATAAACACACGTGGACAATATTAATCCAAAACATACATGCCATCATGCATGTTGTTTAATTTCTTTGTTTATTATCATCATATCATATATAGTGCATGTTTG
