# Supplementary material for: Comprehensive genomic characterization of NAC transcription factor family and their response to salt and drought stress in peanut
Source: BMC Plant Biol. 2020 Oct 2;20:454. doi: 10.1186/s12870-020-02678-9 (PMC7532626; doi:10.1186/s12870-020-02678-9)
Supplement: Supplementary file 2 — Additional file 2. gDNA sequence of NAC genes from two wild peanuts [file 12870_2020_2678_MOESM2_ESM.docx]

>AdNAC22

GTGAGAGATCCATAAATATGGAGAGCACCGACTCGTCCACCGGTTCGCAACAACCGAACCTTCCACCGGGTTTCCGGTTCCACCCCACCGACGAGGAGCTCGTTGTTCACTACCTGAAGAAGAAAGCTGCATCAGCTCCTCTCCCAGTCGCCATCATCGCCGAGGTTGATCTCTACAAGTTCGATCCATGGGAGCTACCAGGTTTGTTAACAACAATTTTCACCCCACCCCACAAGAACACAATTACAAAGTTTGAATCTTTACATCCCAATGAAAAATGGGTTTTATGCTTTCTTTTGTTTTAATTATGAATTTTTTTTACTTTTCAGCTAAGGCAGCGTTTGGGGAGCAAGAGTGGTACTTCTTTAGCCCAAGGGACAGGAAGTATCCGAACGGTGCTCGGCCAAACAGGGCGGCAACTTCCGGGTACTGGAAGGCAACCGGGACGGATAAGCCGGTGCTGACCTCCGGTGGGACCCAGAAGGTCGGCGTGAAGAAGGCTTTGGTCTTCTATGGAGGGAAGCCCCCCAGAGGGATAAAGACAAATTGGATCATGCATGAGTATAGACTTGCTGATAACAAACCTAACAATAGGCCTCCTGGTTGTGACTTGGGTAACAAGAAAAACTCTCTAAGGGTATGTCAATTATTTTTTACCATCCATATTAAATATACACTAAAAATCCATCATCATATAAAATATATACATATTTATAAACAAATATTAATAGTTAATTTTTTTATTGTTGATAACTTTTAAATTTTATGTGATGTTTAATTATATGTAAACACGTATTAGTTACTTATTAATTTGTTTTGATTTGGATGATTTTGTGTAGCTTGATGATTGGGTATTGTGCCGAATCTACAAGAAGAACAACACACATAGGTCTCCAATGGAACATGAGAGGGAAGATTCCATGGATGACATGATTGGAGGGATTCCTCCTTCCATCAACGTGGGCCAAATGAATGCAAGATTTCATCTCTCAAAAATGTCAACAAGCTTTAGCAACGCTTTGTTGGAAAACGACCATCACCATCACCAGAATCTTCTGGAAGGTATGATGCTAGGAGGAGGAACCAACAACAGCAATCCAAACATGTTGGGATTGGGATCAGCCTCAAACACCATTAACAATAATAGTAATAAGGCAGAGCTTTCATTTGTACCAACCATGACTACATCTTCAAACACCAAGAGGACTCTATCATCACTCTATTGGAATGAAGATGATGTTGCTGCTTCCAACAAAAGATTCAATTTGGAAAGTGGAGATCATAACCATGGAGAGAATAATGGTACTAGTGCTAGTTCTATTGCTACTCTG

>AiNAC57

GTGAGAGATCCATAAATATGGAGAGCACCGACTCATCCACCGGTTCGCAACAACCGAACCTTCCACCGGGGTTCCGGTTCCACCCCACCGACGAGGAGCTCGTTGTTCACTACCTCAAGAAGAAAGCTGCATCAGCTCCTCTCCCAGTCGCCATCATCGCCGAGGTTGATCTCTACAAGTTCGATCCATGGGAGCTACCAGGTTTGTTAGCAACAATTTTCACCCCACCCCACAAGAACACAATTACAAAGTTTGAATCTTTACATCCCAATGAAAAATGGGTTTTGCTTTCTTTTGTTTTAATTATGAATTTTTTTACTTTTCAGCTAAGGCAACGTTTGGGGAGCAAGAGTGGTACTTCTTTAGCCCAAGGGACAGGAAGTATCCGAACGGTGCTCGGCCAAACAGGGCGGCAACTTCCGGGTACTGGAAGGCAACCGGGACGGATAAGCCGGTGCTGACCTCCGGTGGGACCCAGAAGGTGGGTGTGAAGAAGGCTTTGGTCTTCTATGGAGGGAAGCCACCGAGAGGGATAAAGACAAATTGGATCATGCATGAGTATAGACTTGCTGATAACAAACCTAACAATAGGCCTCCTGGTTGTGACTTGGGTAATAAGAAAAACTCTCTAAGGGTATGTCAATTATTTTTTATCATCCATATTAAATATACACTAAAAATCCATCGTAATATAAAATATATACATATAACAGCTAATTTAATAATTAATTTTTTGTATATAAGTAATATTTAATTTTTTACCTTTTTTTTTTTTTATTATTGATAACTTGTAAATTTTATGTGATGTTTAATTATATGTAAACACGTATTAGTTACTTATTAATCTGTTTAGATATGAGGAAGATTATTTTGATAAAATTTGGCTAATTTGATTCGGATGATTTTGTGTAGCTTGATGATTGGGTATTGTGCCGAATCTACAAGAAGAACAACACACATAGGTCTCCAATGGAACATGAGAGGGAAGATTCTATGGATGACATGATTGGAGGGATTCCTCCTTCCATCAACGTGGGGCAAATGAATGCAAGATTTCATCTCTCAAAAATGTCAACAAGCTTTAGCAACGGTTTGTTGGAAAACGACCATCACCATCACCAGAATCTTCTGGAAGGTATGATGCTAGGAGGAGGAAACAACAACAACAACAATGTTGTTCCTCCAAACATGTTGGGGTTGGGATCAGCCTCAAACACCATTAACAATAATAGTAATAAGGCAGAGCTTTCATTTGTACCAACCATGACTACATCTTCAAACACCAAGAGGACTCTATCATCTCTCTATTGGAATGAAGATGATGTTGCTGCTTCCAACAAAAGATTCAATTTGGAAAGTGGAGATCATAACCATGGAGAGAATAATGGTACTAGTGCTAGTTCTNNNNNNNNNNNNNNNNNNNNNNNNNNNNNNNNNNNNNNNNNNNNNNNNNNNNNNNNNNNNNNNNNNNNNNNNNNNNNNNNNNNNNNNNNNNNNNNNNNNNNNNNNNNNNNNNNNNNNNNNNNNNNNNNNNNNNNNNNNNNNNNNNNNNNNNNNNNNNNNNNNNATTTTATCAAATTCAAGGGATGAATTGGTATGCTTAGGTACACATACATACAATTCCCATGTTTAATTAATTTCTAAGGTGGTGATTAATTAGTTAATTAATTAATTAATTATACTATATTGAATATTGATGATGATGACATGCACATGTAGAAATTAAATTATTGAACTTAATTAGTTTAGAAGGGTGAATGAGTATTCTCAGCTATAGTGGTGGTGGTGATGGTGATACCTTAA

>AdNAC62

AACATAACAACACAAGAAGAGAACCATAATTAAGCAAGCGACATATACATAATACATAATTGATGATTGATATGGGTTCTTCATCAGTAATAGAAGGTGAAGTTACACTTCCAGGATTCAGGTTTCACCCTACTGAAGAAGAGCTCCTTGATTTCTATCTCAAGAACATGGTCGTTGGAAAGAAGCTCCGTTTCGATGTCATCGGCTTCCTCAACATCTATCACCATGATCCCTGGGACTTGCCAGGTACTAATAAATTAATTAAACTTGGTATTCTTTGTTTTTGTTATTAACACTGTTATTGTGTCAAACTGTCAGTGCATTAAAATTAAATATTTTTGTATAAAGGATTGGCTAAAGTGGGAGAGAGGGAATGGTATTTCTTTGTGCCTCGGGACAGAAAGCATGGCACCGGGGGAAGGCCAAACCGGACCACCGAGAAAGGGTTCTGGAAAGCAACCGGTTCCGACCGTAAGATCGTTACCTTGTCTGATCCGAAGCGCATAATTGGATTGAGGAAGACACTGGTTTTCTATGAGGGAAGAGCTCCACGTGGATCCAAGACCGATTGGGTCATGAATGAGTACCGTTTACCTGACAATTGCCCCTTGCCTAAGGTATCTTACTTTTAAAACAACAAACTACTCGTATAAAAATATTTTCGTCACAAAATGTCTAGATTATTATTTTCACATAAAAATATTATATAAATAATTATTATATCTATTAATTTCGATAAAATAATATAATAAGATACGAAAATATAAATATAGATATATAAAATTATATTTAATAGATGAGATATGAATAAAATTATTATATTTTGAGATATTAAATTAGTATATTTTGTATTTTTGTTATGAAAATCATAAAGATATTAAACAAAAAAAGACTATTAATTTTTATAATTATACATGGAGTTAACAATATAAAAGAAGCACATATTTAACTTTAATTAATAATATTTAGTTACTTTTCGATTTTTACTTCTAAATGTCTTGTGCTAGACCATGCTCTGTTAAATTACAGAGGAAACTTCCTATGATGTTTTTAGTGGCATTAATTGGTTCACCTATGAGTTATTCTATATTAAAATAAATAAAACTGTCCTACATAGGGAAGGAAACTTCCATGGCAAAAGATGGATAGTGATAGGAGTAAGTCATGGTACTTTCTATATTTATATTCTAGTTTATGGTTCCGGGTCCCTCATATATGAACCTCAACTTCCATTTTTAACTTTTATGCCATTAAATTAATAATACTAGTAGGATCATCACTAGTAGTTAAAGTACTATTATATTAATTAAACTGTTTGAATTGTATAATAAACATAATTAAATATGTGTATAAAATATATTTTACACTGACAGTACATGCATTTCTGTAGGACATAGTGTTATGCAAGATATATAGGAAGGCGACTTCGTTGAAAGTGTTGGAGCAAAGAGCAGCAATAGAGGAAGAGATGAAGCAAATGGTAGGTTCCCCTGAATCCACACCTTCCTCCACAGACACCATGTCCTATGAAGAACAACAACAGAATCAGAATCAGAATCAGAATCTGCAATTGTTACCACCACAACATGTTGTTACTAAGAAAGAGGTTGAAGCTGAACTTGAAGAGGAAAAAATGGTACATGTTACATTGGCAACAACAAAGCAAGAAAACAAGGACACAACAAAGAACAATAAAAGTAGTTGTTGTGGTAACACAAACACTAACAGTAACACAAGTAGTCTTCAATTGCCATTTGGGAAGGATAAGATCCCAGAGCTTCAAATGCCTATGATGATCACTGATTGGACCCAAGACACATTTTGGGCTCAATTGAATAGTCCTTGGCTCCAAAACTATACCTACTCCAACATATTAAACTTCTAG

>AiNAC61

AACATAACAACACAAAAAGAGAACCATAATTAAGCAAGCGACATAATTGATGATTGATATGGGTTCTTCATCAGTAATAGACGGTGAAGTTACACTTCCAGGATTCAGGTTTCACCCTACTGAAGAAGAGCTCCTTGATTTCTATCTCAAGAACATGGTCGTTGGAAAGAAGCTCCGTTTCGATGTCATAGGCTTCCTCAACATCTATCACCATGATCCCTGGGACTTGCCAGGTACTAATAGATTAATTAAACTCGGTGTTCTTTGTTTTTGTTATTATCACTGTCACTGTATCGTACTGTCAGTGTATTAAAATTAAATATTTTTGTTTAAAGGGTTGGCGAAGGTAGGAGAGAGGGAATGGTATTTCTTTGTGCCTCGGGACAGAAAGCATGGAACCGGGGGAAGGCCAAACCGGACCACCGAGAAAGGGTTTTGGAAAGCAACCGGTTCGGACCGTAAGATCGTTACCTTGTCTGATCCGAAGCGCATCATTGGATTGAGGAAGACACTGGTTTTCTACGAGGGAAGAGCTCCACGTGGATCCAAGACCGATTGGGTCATGAATGAGTACCGTTTACCTGACAATAGCCCCTTGCCTAANNNNNNNNNNNNNNNNNNNCGAGTATAATTTTAAGGATAATAATAAAAAAGTTTGTTATTATTAACTCTAAAAATATATGTATTTGTTTTGTCAAAAATAAAAGAAAGTTAGTTATTATTCTTCTTCATTCTATAACAGGAAAGGAGACCTCAAGATCTTTGTTCTAACCCATGCTAGGGGGCCACAACTTGGAAATCAATTTCCCGACCAACGGAATGGAAATTAAATTCCACATTATTTTCCAACAAATTCAACTTTCTTTTTCACTAACACTAATTTACGGAAATGTATTCTCTCAATTGATGTCATTAATATTATATACAACGAATTTTCTTACATATTTTTTCTATGTATTATAGAATATATACTTGACGATCAATTCTCTTAACTTAGAATTTAGACAATTAATGTCTATTATTGCTAACCTGGCTAATTAACTACAAGAGGAATCTTATTTTGCTACAGTTTGCCCTTTTATTAGTATATAGTCAAGTTGATTAATTATTATCAAAAGTCATAATATTTACTATCACGATATGATTAGAAAGATGAAAATGGAACATGGTTATAAGATTGACCACCTTATCATTTCTATTTTGATAATAATGTTGAAGGAAAAGGAATGTACATGAGATTGAGTAACCAACAAACCAAAACGAGTTCAACTTTTTCTTTTTCCCCTTAAAGAAAGACAAAAGTTGAATTCGTAGTAAAAAAATTATTTTATTAATAGGTTCAAATTAAATTGGCACACTTGATCAAAAACACAGAAAAAAAAAAATCATCATATACACACATTTTTAGTAGACATTACATAGATTCTTCTTCTTTCTTTCTTTTTTTTTTTTTAAAAAAAATATTTACAAACACTTCTGTTACTCACTCAAGCATATGCAAATATGGAACCATGTACCTTAATAGTTATTCCTTTTCATTATATATATACGTAATGTGTCTGTGTTTAAATTAATCTTAAAAAAAATACTCAAATTAAAGACTGAAAGGTTTAATTGAGCACTTATGACTATCTAGAACTTATGAATATCCCGTAAATGCATTAGAACCGAATAGTTAGAGCATGGAATCCCAAGACTTACACCAACATTTCAAAAATCCACCTGGTCATTGAACCAATAAATTTAGAAATTTAAAACTTTAATTGGTATTCAACCGAATATAATAAAATAATATATTATTATATAAAATATATAATTTTTGTATATTTTATAATTTTAAATTGATTAACTGCTAAAATTTAAATTAATTTATTGTCAATCAATTTTTTATCCGAATCGAACTAATCAGATAGTCGATTTTTTATTAACTTGATTCAGTCGACCGGTTCGACTCCATTACAACAAATACAAAATATGCCTTAATATTACATTAGAATTTAGTAGGTTTATTTTTTATTTTTTTTATTTTATACTTTGCAAAACATGATAGTTTCATTTGAATTTTTATCTGACTAGTTAATTAGTTTATTTGATATCTAGACTCAACCGAGAAAACCAAAACACACAATAGCACAGAAATATTTCCTTTCCAAACATGAACCTGCTAAATACTCTAAATTGGAGTTCTACTTTTTAGGTTCCTCCTCTTGGCACCTAAATCAAAGGTTATTAGTCACTAGCTCTAATTATTTACCTAAAAACGATAGGTTATAAATGAAAATTAAACTAATTCTTATAATAAGTAACACGCTTGAATCTTATGCCTAAACTAAGTAGAGACCAATTTAAATTAACTAGAGAGTTTAACATCCGTAATAAAAATAGATAATGCGACTACATGATAAATTAAAGTTTTTTTCATAGGAAGAAGTAGAACGTAGAATAATGTAAATAGACAATGTAGGACAATTAAAAAATTGGCAACTTTGCTCGTATTTATTATCCATTATCTCTATTTATTACATATGTAAAAAAAATTGAAAGAAATTTAGTGCACAATTTTTGTTTGAAGTATTTTTTATTGGGAGCCATTCAAATAAAGATGCTGAAAACGTCTTTTTATAAAGATTTTTTAATAATTAAAATTTAACATATAAAATCGATTAAATCGTGTTATTTTTTGTCAAAATTAGACTAGACAAATTGATTTGACCAAAAAATGGTGAATCAAATCTTAAATTGATCTAAATTAATATTTTTTTTTATAGAAAATGACTAAAATACTTCTATTATATATATTAATTTTGAGAATTCTAAATTTTAGTCCTTTATTTTTCTATCGTAGGATTAGGATTTAGAATTTTTAAAATTAATATATATATAATAAGAGTATTTTAATCATTCTCTATAATAAGGGTATTGTAGTCATTTTTTATAAAAAAAATAATATTAATTTAGACTAGTTCAAGATTTGATTCACCATTTTTTCGGTCAAATCAATTTGTCTAGCATAATTTTGATAAAAATAACATGATTTAATCGATTATATATGTTAAATCTTAATTACTGAAAAACATCTTTAAAAAGAGATGTTTTAGACGTCTTTATCTGAGTGGCTCCCTTAATTATTATATTTTAAACACATATATAAAGAGACACATCTAGAAAATATATCTATAAAGACACTTCTATTAAACACAGTCATAAAAAAGATATTTTTATTAGACATATCTACAAAGACACTTTTATTAAACACAGTTATAAATAAGAATTGGCAGAAGTTGATAGAAATACTGTTGGTAACGTAACGAGATTGTAATTAATTATGTGACATGACCAAGTTGCTTTCAAACTTTGGTTGATATAATAATAGTTATAATGTTCAAATAGCTTCATCATTCATTTTAAGGAAAATTTCTTAGCAAATGCATGCTAATAATGGTTACGTTTTAGGAACTCTCACATGTATCCCTATACTCATCAAAAGGGAACCAATCATAGAATAGGTTATAGGACCTTACCCTAATGCACATGCCCATGTTTTATATGTCCTTTCATTTCCTTGTAAACTTTCCTTTTATCTTCCTCTCTTTTGTTTTGAGACATTGGCATGCAACTTTGCCTTGGCTTGATTTGGCAATTACTAGCTTTTGCAATTACTCCATCATCATTCATAAATCACAATCTTCATTTCAGGTAAGATCCACCCATATACACCAACAATATGTCATTAGTTCATAGTGCTTACTTGATATTTTTGGTACCTAAGAAAGTTACTCAATTTAATCACTTTTTAAGTAATAAAACAAAATAAAAGACATTGGAATAATAGGATCGGAAATGACCATCCAATAATTGATACTACAGATGATATAAATCCGATAAGTGAATGGATATTGGAATCTTATCGGAATTCCTTTAATTAGTTGAGTCAACCTACACTTAGCTTTGTATATGTGTTAGACCAGTCACAGAAATTGAGAAGAATAGAATCACCGTGTATTTGGTTTATATTCTTTTAATATTTTTTGTTTTTAACCTTTTATTAAAAAAAATTGTGAGATAATAAAATTTAATTTTTTGTTTTTTGTTTTACAAAATATTAAAAATAAAAAAATACAGATCAAATATATTTTTTTATAAAATAGTATTAACTAGACTTGTTAAAATTGGTCAAATTCGTCAAATAAGCATATTTATCATTTAAACGGGCAAATTTTTACTTTTAAAATGAGTCTTATCTAAATTTTGAGTTAAATGGATTAAGCCTGATGAACAACAAAAAGAGGGTTAAATAGGCCAATTTTTATAAACTAAATAGACATCTCATTTTTTCTTTCACATTTTTTTTCAAAAATAAAAAATAAACGAGTTAACTCATATAATCGTCGTACTTGTCATGAACAGTTTTGACCTAAAAATTAAGATCTATAAATAAACAAAGAATAAATAAGAGTTTAGATTTTCTCAATTTTTTTTTATTTAAAAAGATAAAGTTAATTATATATTTTATTATTTTATTTTATATTCAATTTATCAATAAATAAAATATAAAATTATTTATTTTTATGTATTTATTTTTTATACCATATTTTTAATATCTTGCCTCGTTCTAATTTTTAAAATCAAACACAATCTATATGATTGTAAATCACTACTTTATCCACATGTATTAGCTATGAATTAAGCTTTCTTTGTCCATTAATTAATTAATTTTTCAAATTATACATGGAGTTAGCACATATTTAACTTTAATTAATAACATTTAGTTACTTTTCAGTTTTTACTTCTAAATGTCTTGTGCTAGACCATGCTCTGTTAAATTACAGAGGAAGCTTCCTATGATGTTTTTAGTAGCATTAATTGGTTCACCTATGAGTTATCCTATGTTAAAATAAATAAAACTGTCCTATATAGGGAAGGAAACTTCCATGGCAAAAGATGGATAGTGATAGGAGTAAGTCATGGTACTTTCTATATTTATATTCTAGTTTATGGTTCCGGGTCCCTCATATATAAACCTCAACTTCCATTTTTAACTTTTATGCCATTAAATTAGAAAGTAATAATACTAGTAGGATTATCACTAGTAGTTGTTAAAGTACTATTATATTAATTAAATTGTTTGAATTGTATAATAACACATAATTAAATACGTGTATAAAATATATTTTACACTGACAGTACATACATTATTGTAGGACATAGTACTATGCAAGATATATAGGAAGGCGACTTCGTTGAAAGTGTTGGAGCAAAGAGCAGCAATAGAGGAAGAGATGAAGCAAATGGTAGGTTCCCCTGAATCTACACCTTCCTCCACAGACACCATGTCCTATGAAGAACAACAACAGAATCAGAATCAGAATCTGCAATTGTTACCAACACAACATGTTGTTACTAAGAAAGAGGTTGAAGCTGAAGTTGAAGAGGAAAAAATGGTACATGTTACATTGACAACAACAAAGCAAGAAAACAAGGACACAACAAAGAACAATAAAAGTAGTTGTTGTGGTAACACAAACACTAACACTAACACAAGTAGTCTTCAATTGCCATTTGGGAAGGACAAAATCCCAGAGCTTCAAATGCCTATGATGATCACTGATTGGACCCAAGACACATTTTGGGCTCAATTGAATAGTCCATGGCTCCAAAACTATACCTACTCCAACATATTAAACTTCTAG

>AdNAC20

GCACGATAAGAGAAGAAGATTTGTTTAATTAATTTGTCAATAGAACAAATTAAAGTGGATGCATGCTATCATATATACTATCTATGTGTCGCTTTTCTCTTGTATAAATATAGTTCCATGTTAGCTTGACTGAATTTCATCGATGTCCATGCATGTATAGTGTCACATAGAGTAGAGAGAATATAATACAATCAAATAATGGAAAAGCTAAATTTTGTGAAGAAGAACGGGGTAAGTAGAATGCCTCCTGGATTCAGATTCCAGCCAACGGATGAAGAGCTTGTGTTTCAGTATTTGAAATGTAAGGTCTTCTCATTCCCCTTGCCCGCTTCCATGATTCCTGACATCAATCTCTCCAACTATGATCCTTGGGATTTGCCAGGTACGTACATAGCTACCCGTATACATATGTATAAAATTGATAATAAAAATTTAAATTGATTATTAAAAGTCTTTAGATAAAAATTTAGGTAAATTTATCATATTATTTAATAGTTTTAATTATCAATTTTAAAAATAATGCATGTAAATTTTATAATTAAAAATCATTAAATGACTATATAAACAAATATATTAAATTATTTAACGATTTTTAACCATTAATTTTACATGAAAAATAACTAGCTTGTAGATAAATAGTCACATACATAACATCATGGTTAATATATTGTTATTTTGATACATAGGAAATTGTGATGAACATCAAGAGATGTATTTCTTCAGCAGCAAGGAACCCAAGTATAGAAATGGAAGCCGCATGAACCGAACAACCACCACTGGCTATTGGAAGGCAACAGGATCCGACAAAAGAATCATTTCATCTTCTAATAATAGTGACGATAATAGCATTCTTGGCATTAGAAAAACCCTAGTGTTTTACCAAGGGAAATCTCCCAATGGCACTAGAACTCACTGGGTCTTGCATGAATATCGCCTGGCTAGTACTACTCTACATGCTAATAACAATGCTTGCGATATAGGAGATTGGGTTCTGTGCCGCTTATCGGTGAAGAAAAGGAGTGTTGGGAGTGGTAGCATCATCATAAGCAAGAAAGCACGTTCTTCAGCATCTTCATCCTCATCTTCTTCCACTTCAAGTAATAACGTCATGGAAGTATCTTCTTCATATGCTTCTTAATATCAACGCAACAAACAATGCACTTCCTCCTGATGGTCTTGCTGACTATTGATTAAAACCACCATACATATTCTCACTCAGACCCCGTCGATTCTGCAACTAAATGGATTCATACCCTTTAAAATTAACAA

>AiNAC66

GCACGATAAGAGAAGAAGATTTGTTTAATTAATTTGTCAATAGAACAAATTAAAGTGGATGCATGCTATCATATATACTATATATGTGTCGCTTTTCTCTTGTATAAATATAGTTCCATGTTAGCTTAACTGAATTTCATCGATGTGCATGTATAGTATCAGAGAGTAGAGAGAATATAATACAATCAAATAATGGAAAAGTTAAATTTTGTGAAGAAGAATGGGGTAAGTAGAATGCCTCCTGGATTCAGATTCCAGCCAACGGATGAAGAGCTTGTGTTTCAGTATTTGAAATGTAAGGTCTTCTCATTCCCCTTGCCCGCTTCCATGATTCCTGACATCAATCTCTCCAACTATGATCCTTGGGATTTGCCAGGTACCTACATATGTACCCGTATACGTATGTATATTAAATAGTCACATACATAACATGATGGTTAATATATTGTTATTTTGATACATAGGAAATTGTGATGAACATCAAGAGATGTATTTCTTCAGCAGCAAGGAACCCAAGTATAGAAATGGAAGCCGCATGAACCGAACAACCACCTCTGGCTATTGGAAGGCAACAGGATCCGACAAAAGAATCATTTCATCTTCTAATAATAGTGACGATAATAGCATTCTTGGCATTAGAAAAACCCTAGTGTTTTACCAAGGGAAATCTCCCAATGGCACTAGAACTCACTGGGTCTTGCATGAATATCGCCTGGTTAGTACTACTCTACATGCTAATAACAATGCTTGCGATATAGGAGATTGGGTTCTGTGTCGCTTATCGGTGAAGAAAAGGAGTGTTGGGAGTGGTAGTATCATCATAAGCAAGAAAGCACGTTCTTCACCATCTTCATCCTCATCTTCTTCCACTTCAAGTAATAACGTCATGGAAGTATCTTCTTCATATGCTTCTTAATATCAACAATGCACTTCCTCCTCATGGTCTTGCTGACTATTGATTAAAACCACCATACATATTCTCACTCACAACCCGTCGTTTCTGCAACTAAATGGGTTCAGACCCTTTAAAATTAACAA

>AiNAC28

TAACCAAAGCTTTAAACAATTTTCATCTTCAAAAAAACAAAAACAAAAATGGCTAGAAGTGCCACAATTCCATTTCCAATACTTGATTTCATTCCTGTTGGATTCAGGTTCAAGCCAACAGATGAAGAGCTTGTGAGCTATTACCTCAATCACAAGCTCCTAAATGACAATTTTCCAATCGATATTATCCCTGACATTGATCTTTGCAAGGTTGAACCTTGGCAAATTCCAGGTAAACCCATAATAAATAAATAAATAAAACCACCCTATCATTTTCATGTATCAAAGTTTGCACCTTTTTCATGTTTGATGATCCTATCCGTTATTAAAAATTTAAGAAAATGAAGTAATAAATATACAATTATTCAACAACCCCATCATCTTCATGAGACAAAGCTTGTACCTTTTTGTTTTTTGTCACTACCCCATGATCTTAATGTGACAAAGTTTGAAACTTTTCTTCATGTTAGTATGATGCTGTTTGATGATCGTTGAATTGGGTTCTGTGTGTCAGCATTATCAAAGGTAAAATCGGATGATCCAGAATGGTTTTTCTTTAGTGGACGTGATTACAAGTATGGAAAGAGCAAAAGATCAAACAGGGCAACCAAAGGAGGATATTGGAAAGCCACAGGACAAGATAGATTCATAAAGGAAAGAGGAACTATGAATGTAATTGGGAGCAAGAAGACACTTGTTTTCTATAGTGGCCGTGTTCCTAATGGTGTCAAAACCAATTGGGTTATCCATGAGTATCATGCTACTACCTTTGATGATAGCCAGGTACTTATGGTTTTTCTTTTTCCATAAAAGTTTTGCCTTGATTAGTTTTCATCTGTTTTGGAAATTGATCTTTGTTTCTATAATAAATACCCAAGTTGATTCTATTTCATATCATACTTTGGTTGTTCAACAAATTTTGAGGTACTCTAGAATTACTTTTTGAAGGGGACTAATTTCTTTTAGACCTAATTGTCTTTGATAGGGACTTGTTTGTTCAAAATGCATAATTGAAAATGACCGAGAGAGTAAAAAAATGGTCGGAGAACAAAGTGGTAGATCTAAATTTCTTTGGACCAATTTAAGTGCTCACTTTTGTTTTTTATCTATATGTTTTGTTGGTATAAATTACTATTATAATGTATGATTGTTGTTGTTGCCCCTTCATATGATTTTGATGTTTCCTTATCTGATTGGTGATTATGTCATTGGAATTATTCCCATGTTTGTATTTTGTGTCATAGTCTATTCATGAATCATGACTCACTTGTTTTTCCCTAATTCATTCTTTTTTGTTAATTGGTGAAACAATTCATCATTGATTTTCATGAAATAGTGGAATACTATTGAATTGTTGATTTTGCTATTTAAGCTGTTTCTAGATGAAGTCTTCAATGAACTGAATTTGTGATAGTCTCCATTGTAGTATCAATTTTTCAGTGCTGATTATACTCAACATTCTGCAGAGGAATTTTGTTTTGTGTCGCTTGATGAAGAAAGTCGAGAGAAAATCCGAAGATGGAACTGATGCACAAGCCTGTGATGAGGGGGAACCTAGCACTCACATGGAAGAAGCAGATGAGAGTGTCTCAACTGTAAGCAGAAGCATCATTTTGATCTCTATTTCTTGGTTTATGCATTTAAAAAAAGTTTGGGGATAGTTAATATATCTTTTATGTCTGTTTTAGATGTTTGATTCGCCAGATGTGGACATGGATTCAATCTTCCACACACTGCCTCAAGACAGATCATCATCACAGCATTCTCCAGTCGGCATTGAACAGCAAGAATCCTTCCCATTCTCCCCATCTGAAAATTATTACCTTGTAAATGAAGATAGCAGTATGCATATACAATTCGAAACAAACGAAGAGAAGCAAGATGCTGAGAAATTTGCGGATTCGATTTTGGATAGTGGCAATATAGCTATGTTTGAAGAAAGACAGCAGCATCATACTTTCATGAATAATCACCTCCGCTCGGTCCCATCGATGAGGGTATGCTATGAAAGCAGTGACACAGATGCTGAAGTAGTCTCTAGACGGGTAAAGATAAATCCTTAAAAATGATATTGGGGAGTTATATCTCCAATTGTTTATGAGCTATTTGTTGGTTATGTACATTAATTGTTGTTCACCAGTCTATGAAAAATGTTTAGTTAGTTCCTTCTATAGGATGTTAGAGAGATACTTAGCTGAAATGTCTGGTTTTTGTTTGTGTCCTGTTTGATACATGGCAGTTGGCACCTTAACAATACATCAGTGCATAAAGTGGAGAAAAGGGCAGGGAATGGAAATTAAGAAAATACTTCAAAAGCAATATTTTTCCTGGATTCATGATCCTTCTTTGTTTCGAGCCCTTTCAAGAACTGACTTATAGCATGCTCGGTTTATGCTACTGATGCAACCCAACCATGGTTTTAGGTTTTCGTAAGCTTATACCGAGCATGCTATATGAAACCGAAGGAGTATACTTTTTGGTGGCCCAAAACCATAGTTTGGGAAACAACTATATGGAGATAAGTTTAGCTGTTTCTCAAACCATGGTTTAGAGTTTCCAAAAGCTAAATCAGCCATGGTATCCATTAATTCTCTGCTGAAATTATATAGCATGTTTAGTTTAGTTTTTGATGACCCTGAGCCATGGTTTTAGAAAAAGTTCATCTTAAATAGCTTGTTCCAAAATCATGGTTCGGGGTTTTTAAAAACTAATCCAAACATGCTATTGATCCATCCGTCCGCTTCTGAAATTATACAGTATGGCAGTATTCCTGATTCTTCAACAATGTGTAATGATCAGGCTGATTCAAGAGAATACCATGTATCAAAAATGGTTCAATCATCACATAGTGCTGCGCGCACAGATAAAACTAGAAGTATCTCTTCAGAAGACTTTTGGGGAGTGGATTCATCTTCATGTGACTCAAATGCAGATAAACCTTTTGAGATCAATTCTATTGAAATTTCTAGTCCTCCACCGGCTCTAAGTGGATCGAAAAATCAATATAATCCGAGACTATCTCAAACACATAGGAAGGTTTCAAGCAATGCAATTCCCAATCTTGAGGTGAGAACACCGAGCAGCCAGACTAAACGACGAAGAAGCCTATCTTGACTGAGTAGGCTGTGATGCTTCATTTGAAGAAAATTAAATTCTAACATGCCACTGATCATACCATGCTCCTATGTGATGTAATATGCAGGATAAGAAGAAATTGACCACTGTGGAACAATCAAGAAGAGATCAAGAAAAAGCTCGAAAAACTAGTCCAGGAAAGAAGTTAGAAACCAGAAGCTCTGATGTTAATAGAATTGGTAGTTTCATCCACCTAGAGCCGTGTTCGTCGAGCGAAAGCCTGACTCCACGAGCGGTATACCTTGTTAATGTAGTTATTGGGATTTTGTTGCTTCTAGCCATTAGTTGGGATGTGCTATCTTGTTAGAGAAGTGTTGCCATGCAAGATTTGTTTTAGGAGTTTCATAATTATATACATAGTTCAAGTAATAATATAGTTTACATAGATCTTGCACTGATGCAAAGATTACTGCACCTTTCTTATCATAATGAAATTTATGCTTGTGAATTGTGATTAGTGGTAACTTCATATTGTCATTAATTCTATAATTGCAGTCTTTAAGTCACAAAATTCATTTAATGTTAGAACTAGTTGAGTTCTAGACTCTAGTGAAAAGATTCCTTTTTTCCATTCAATAAATGTTTGATTAGCTTGATTCCCAAAATGTGCTGACTTCAAATTCTCCATTTAGTAGAAACAGATCAAAGTCAATGTTCCAAAAGAAGAAAATTTGGAAGCATACATGGAATTTAAGCCAATCAAGTTTCCTTTCTTTAGAGAATATGTCTGCCTCTCTGCATCTTATACAAAAGTGCATAAAGTCAAAAGCAGGTTACACACCATGTGACAGTAGCAGTGTTACTTTGAATAGGATCTTTGTTTCATTTTAC

>AdNAC58

GGGACCCTTTGGTGAATTGAACAACAATCAAGAACAACCAATTTTAGACCTTTATATATTACATACATATCTTTGATTTGTTAGTTGTTGACCTGAATGCCAGAAAGCATGAGTATATCAGTGAATGGACAATCTCAAGTTCCACCTGGATTCAGGTTCCATCCAACTGAGGAAGAACTCCTTCAATACTACTTAAGGAAGAAGGTCTCTTATGAGAAGATTGATCTTGATGTTATTCGTGACGTTGATCTCAACAAGCTTGAACCATGGGACATACAAGGTACCATATCATTTTTATTTATAGAGTGTTTACTTCTTTTCTATTTGTTTCCGGCGATAGCATCGCTCGAAACAATTTCAGATGATTTTATTATTCTTTCCGAGTAATAATTTTAGTTTGTGCTTTTGTGTTTGAATTTTTATTTCTAAGAAAATTTTGTAACTCCTTAAATTTTCTTATTCTTGTTGAAAATCAAACATTATTTAACTAATTATATATAGTACCAAAAGAGATTATATAGTTAGCTATAATCTTCTTTATTTATTTTTTTTCTTAGTTCTTCCAATTTTCCTTTTTCTTTTCCCTTTCTCCTTTTGGTAATTATGGGTGGCTATCTTTTTTGTGTTTTCCTTCTTCTTCCTTTGATTGCGTTCGTTTCATGTGGGGGATGGATAACTTCATAACTATAATAATGATGACATATTGGAATATTGTACTATATATTATTCTTCTATGGTTTCATGGAGTCTTCATCATCTCATTGATTAATTACAATTAGTAAAAAAAATTGAGAAGCTAATTATTTTCTTTGTTTCTTGAATTTTGGCAGAGAAATGTAAGATAGGAACCACCCCACAAAATGATTGGTACTTCTTCAGCCACAAAGACAAGAAGTACCCGACCGGAACCCGGACAAATAGAGCGACCGCGGCCGGGTTCTGGAAGGCCACCGGCCGCGACAAGGTGATATACAGCAACGGGAAGAGGATTGGAATGAGGAAGACGTTGGTATTCTACAAAGGAAGAGCCCCTCATGGCCAAAAATCTGATTGGATCATGCATGAATATAGGCTTGATGATAACACCACCAACGATGCCAATATTGTAAGCACCTCACAAAAACTACACTCAAATTGTTTTAAATTGAAGTTATAGTCTTCCCTACTATCATAGTATTTGTAAAATTTACAAATTTTTTTTAATATTTAAATTATTTAAAATCAACCCTGTTAAATATACACTAAAATCAATTACTAATATAAAATAAATATTAAAATATAAAATATACGAAAAAATATATATATACAACTGATTTTAATATATAAATAATATTTTTAATTTTTAGTTTTGGTATAATTCTTAATCATTTTAGACGTTTATTTATATAACATTGTATTACTCATAATTTATATTTTAGTAATATTATATAAAATAATCTAAAAGTAACTGTATGTTAGATGTTCTATATATGCTCAATTTAAGACTTTCAATTACATGCATGTATGATAGATTAATTTCTTAGTTTCAATAAAATATTCTTGAAAGAGACATGAAAATTTATACAAGTTTGTGAAATTTTCTGTGTGAGAAAAAAAATTGAAATATATATAAAAATATCTATAGGTTTCAAATGTGATGGGAGATGCAGCACAAGAAGAAGGGTGGGTGGTGTGTAGAATATTCAAGAAGAAGAACCATCTAAAAACCCTAGATAGTCCTTTAACCTCTTCCATCTCCGGCGACGGAGGTAGGAGGAGCCACCACCACCACCACCTATTCGACTCGTGCGACGAGGGCGCCTTAGAGCAAATTCTCCAACAAATGGGAAGAGGTGGTGGTAGTGGTGGTTGCAAGGAAGAGATCAACAACTATGATCAATCTAATAACAACAACAACAACAACTATGGTGGATCATCATCGTTAACAACAAGGTATGCAAGACCTTTTGACACAATCAACAACAATGTTGATAGCAGGTTCTTGAAGCTCCCAAGCCTAGAGAGTCCAAAATCAACAAGCATGGATCATAATAATAATAACAACAACAATGATAATGATGATAGCAATGAAAATAATGGGTACCATCCTATTATTCCAGTTGAGATGGTAACTGAAAATGAAGGGTCATTCACATGTGACAATCCCAACAACATGTTTCATCATCACCATTTGGGTGGTGGCGGTGGTGGCAGCAGCGACGGCGGTGGCGGTCTTACAAATTGGGTAGCGCTAGATAGGCTTGTTGCTTCTCAGCTTAACGGTCAGACCGAAGCTTCTAGACAACTCTCTTGCTTCAATGATCCCACCATGGGGTATGGCACTGGAAATCATGATCTTCTATTTCCATCCGTCAGATCTACTTCATCGTTGACGTCATCGTCAGCGTCAATAAATCCAAGGGCTGTTATTAGTGCGGGTGCAGGTGCATACATTTCTCCAGGCGCACAGGATTATACCACCACAAGCGAGATTGACCTGTGGAACTTTGCTAGATCCACTTCTTCGTTGTTGTCATCCTCTGAGCCATTGTGCCACGTGTCAAACACGTCAGTGTAG

>AiNAC31

ATGCCAGAAAGCATGAGTATATCAGTGAATGGACAATCTCAAGTTCCACCTGGATTCAGGTTCCATCCAACTGAGGAAGAACTCCTTCAATACTACTTAAGGAAGAAGGTCTCTTATGAGAAGATTGATCTTGATGTTATTCGTGATGTTGATCTCAACAAGCTTGAACCATGGGACATACAAGGTACCATATCATTTTTATTTATAGACTATTTACTTCTTCTCTATCTGTTTACTTCTTCCCTATCTGTTTCGGGCGATAGCAATCGCTCGAAACAAATTCGGATAATATTATTATTCATCCTTAGTAGTAATTTTACTCTGTTTTTTTGAATATTTATTTCTAAGAAAATTTTGCAACTCCTTAAATTTTTTTTTTTAATGAAAATCAAACATTATTAACTAATTATATATAGTACCAAAAGAGATTATTATATAGTTAGCTATAATCTTCTTTATTTTTTTTCCCTTAGTTCTTCCAATTTTCCTTTTTCTTTTCCTTTTCTCCTTTTGGTAATTATGGGTGGCTATCTTTTTTGTGTTTTCCTTCTTCTTCCTTTGATTGCGTTCGTTTCATGTGGGGGATGGATAACTTCATAACTAAAATAATGATGACATATTGAAATATTGTACTATATATTATTCTTCTATGGTTTCATGGAGTCTTCATCATCTCATTGATTAATTACAATTAGTAAAAAAAAATTGAGAAGCTAATTATTGTCTTTGATTCTTGAATATTGGCAGAGAAATGTAAGATAGGAACCACCCCACAAAATGATTGGTACTTCTTCAGCCACAAAGACAAGAAGTACCCGACCGGAACCCGGACGAATAGAGCGACCGCGGCCGGGTTCTGGAAGGCCACCGGCCGTGACAAGGTGATATATAGCAACGGGAAGAGGATTGGAATGAGGAAGACGTTGGTATTCTACAAAGGAAGAGCCCCTCATGGCCAAAAATCTGATTGGATCATGCATGAGTATAGGCTTGATGATAACACCACCAACGATGCCAATATTGTAAGCACCTCACCAAAACTACACTCAAATTGTTTTAAATTGAAGTTATATTCTTCCCTACTATCATAGTATTATATTACTCATAATTTATATTTTAGTAATATTATATAAAATAATCTAAAAGTAACTGTATGTGTTAGATGTTCTATATATGCTCAATTTAAGACTTTGAATTACATGTATGATAGATTAATTTCTTAGTTTCAATCTTGTTACATATAAAATATCCTTGAAAGAGACATGAAAATTTATACACGTTTATGAAATTTTCTGTGTGAGAAAAAAAAATTGAAATAATATATATAAAAATATCTATAGGTTTCAAATGTGATGGGAGATGCAGCACAAGAAGAAGGGTGGGTGGTGTGTAGAATATTCAAGAAGAAGAACCATCTAAAAACCCTAGATAGTCCTTTAACCTCTTCCATCTCTGGCGACGGAGGTAGGAGGAGCCACCACCACCACCACCTATTCGACTCGTGCGACGAGGGCGCCTTAGAGCAAATTCTCCAACAAATGGGAAGAGGTGGTGGTGGTGGTTGCAAGGAAGAGATCAACAACTATGATCAATCTAACAACAACAACAACAATAATAACAACAACTATGGTGGATCATCATCGTTAACAACAAGGTATGCAAGACCTTTTGACACAATCAACAACAATGTTGATAGCAGGTTCTTGAAGCTCCCAAGCCTAGAGAGTCCAAAATCAACAAGCATGGATCATAACAATAATAACAACAATGATAATGATGATAGCAATGAAAATAATGGGTACCATCCTATTATTCCAGTTGAGATGGTAACTGAAAACGAAGGGTCATTCACATGTGACAATCCCAACAACATGTTTCATCATCACCATTTGGGTGGTGGCGGTGGCGGCGGCAGCGACGGCGGTGGCGGTCTTACAAATTGGGTAGCGCTAGATAGGCTTGTTGCTTCTCAGCTTAACGGTCAGACCGAAGCTTCTAGACAACTCTCTTGCTTCAATGACCCCACCATGGGGTATGGCACTGGAAATCATGATCTTCTATTTCCAACCGTCAGATCTACTTCATCGTTGACGTCATCGTCAGCGTCAATAAATCCAAGGGCTGTTATTAGTGCGGGTGCAGGTGCATACATTTCTCCAGGCGCACAGGATTATACCACCACAAGCGAGATTGACCTGTGGAACTTTGCTAGATCCACTTCTTCGTTATTGTCATCCTCTGAGCCATTGTGCCACGTGTCCAAC

>AdNAC77

TGATTGGAGAAGAAGATTAAAACAAAATGGGAATTCAAGAGAAAGACCCTCTCTCGCAATTGAGTTTACCGCCGGGTTTCCGATTTTATCCGACGGACGAGGAGCTTCTCGTTCAGTATCTGTGCCGCAAGGTTGCTGGCCACCATTTCTCCCTGGAAATCATTGGCGAAATTGATTTGTATAAGTTCGACCCTTGGGTTCTTCCAAGTAATCAGCTAACTATATCTTCCCTCTTAGTTATTATATTCTAATTTCTAATCTCTGGACAATGTTTCTTACGGTGTAATTGTGTTTTCATGGTGCAGGTAAGGCAATTTTTGGCGAGAAAGAATGGTACTTCTTTAGTCCGAGGGATAGGAAGTATCCGAATGGATCGCGACCCAATCGAGTAGCCGGGTCGGGTTACTGGAAAGCTACCGGAACCGATAAGACTATCACGACCGAAGGAAGGAAAGTTGGTATCAAGAAAGCTCTGGTTTTCTACATTGGTAAGGCACCCAAAGGCACCAAAACAAACTGGATCATGCACGAGTATCGCCTCCTAGACTCTACCCGCAAGAACGGGAGCACCAAGGTAATTGATTAACATTTTTGTTTAATATAAAAAAATAATTAAATTTGTAAAAGCATGAAAGTGAAACTGAAAATTAATTGTTGTATTGTTATGCAGCTTGACGATTGGGTTCTGTGCCGGATATACAAGAAGAATTCAAGCGCACAGCAGAAGGTACCAAACGGCGTCGTTTCGAGTAGCGAGCAATATGCCACGCAATACAGCAACGGATCTTCTTCAAACTCCTCTTCCTCCCACCTCGACGAGGTGCTCGAGTCCCTGCCAGAGATCGACGACCGTTGCTTCGCCTTGCCACGTGTCAACTCCTTAAGAGCGCTGCAGCAGCAGCGCCATCACCAAGAAGACACCAAGGTCGGCCTACTCCAACAGCAACAGCAACAGGGTCTCGTAGCCGGCACCGGTAGTTTCTTGGACTGGGCTTCCGGGCCGGGGATTCTGAACGATTTGGGCCAGGCCCAACAGGGGATTGTTAACTACGGAAATGACCTCTTTGTCCCTTCGGTGTGCCACGTGGATTCCAATTTGGTGCCAGCAAAGATTGAAGAGGAGGTTCAGAGCGGCGTGAAGACTCAATCCGGATTCTTTCAGCAGGGACCGAACCCCAATGACTTTACACAAGCATTCTCAAACCAGCTAGATCCTTACGGGTTTAGTAGGTACTCGGTTCAACCGGTGGGGTTCGGGTTCAGGCAATGAACCAGGGTGAGGAACTAGATGTGAAATAACTAAAAGAAAGTGTATTGAATTTTTGACTATTTGTTGAGGTGCAATTGGGGGGTGTAAATAGGGATTCTTTTGGAATATTCCAAGAAAGAAATGTCTTGCATTTAGAAAAAGGGTTGGGTTGTAATTTTCTTTCTTGGTGGTCCTCTTTTTCGGCTGGAGATAGAGTGGAGAAAAGAAGAATTCAATGCATCGAGAGGTGGAAAAAAGGGGGAAAAATTGTGATTGCACTAAATTACTAGTCTTTGGAATTTTAATTAGTTTGAAGATGTACACAATTTTTGGCTGCACCGGCCGTGGTGTGAGCCACATGAATCACGTTTTGTACTTTTGGAAGATTTAGCATCAATCGATATACAATTGTTTACAATTCCAATGTTAGGTTGCTTTTCAAATGTTTGATGTGATCATTTTAGCTAATAACCACAACATTGGTGGCCACAAACTCATTCGTGTTCATGGGATAGGACTATGTTATAAGAGTATTTTCAATGCGTAATTTTAGTTTAGTTTGCTTTTGAGTTTTACAAAATGGTATATTATGTAAAAAATTAATTTGCGATTCAATGTGTGATTATCTATAAAATATAAAATATGAATATTTTGTTAAATTATCGTGTTTGCATG

>AiNAC35

GAGGAGATTTACTACGGTCTGATTTTCGAGAATCTAGCTTTTTGATTATCTGAAGAAGATTAAAACAAAATGGGAATTCAAGAGAAAGACCCTCTCTCGCAATTGAGTTTACCGCCGGGTTTCCGATTTTATCCGACGGACGAGGAGCTTCTCGTTCAGTATCTGTGCCGCAAGGTTGCTGGCCACCATTTCTCCCTGGAAATCATTGGCGAAATTGATTTGTATAAGTTCGACCCTTGGGTTCTTCCAAGTAATCAGCTAACTATATCTTCCCTCTTAGTTATTATATTCTAAGTTCTAATCTCTGGACAATGTTTCTTACGGTGTAATTGTGTTTTCATGGTGCAGGTAAGGCAATTTTCGGCGAGAAAGAATGGTACTTCTTTAGTCCGAGGGATAGGAAGTATCCGAATGGTTCGCGACCCAATCGGGTAGCCGGGTCGGGTTACTGGAAAGCCACCGGGACCGATAAGACTATCACGACCGAAGGAAGGAAAGTTGGTATCAAGAAAGCTCTGGTTTTCTACATTGGTAAGGCACCCAAAGGCACCAAAACAAACTGGATCATGCACGAGTATCGCCTCCTAGACTCTACCCGTAAGAACGGGAGCACCAAGGTAATTGATTAACATTTTTATTTAATATAAAAAATTAATTAAATTTGTAAAAGCATGAAAGTGAAACTGAAAATTAATTGTTGTATTGTTATGCAGCTTGACGATTGGGTTCTGTGCCGGATATACAAGAAGAATTCAAGCGCACAGCAGAAGGTACCAAACGGCGTCGTTTCGAGTAGCGAGCAATATGCCACGCAATACAGCAACGGATCTTCTTCAAACTCCTCTTCCTCCCACCTCGACGAGGTGCTCGAGTCCCTGCCGGAGATCGACGACCGTTGCTTCGCCTTGCCACGTGTCAACTCCTTAAGAGCGCTGCAGCAGCAGCGCCATCACCAAGAAGACACCAAGGTCGGCCTACTCCAACAGCAACAGCAACAGGGTCTCGTAGCCGGCACCGGTAGTTTCTTGGACTGGGCTTCCGGGCCGGGGATTCTGAACGATTTGGGCCAGGCCCAGCAGGGGATTGTTAACTACGGAAATGACCTCTTTGTCCCTTCAGTGTGCCACGTGGATTCCAATTTGGTGCCAGCAAAGATAGAAGAGGAGGTTCAGAGCGGTGTGAAGACTCAATCCGCATTCTTTCAGCAGGGACCGAACCCGAATGACTTCACACAAGCATTCTCAAACCAATTAGATCCTTACGGGTTTAGTAGGTACTCGGTTCAACCGGTTGGGTTCGGGTTCAGGCAATGAACCAGGGTGAGGGACTAGATGTTAAATAACTAAAAGAAAGTGTATTGAATTTTTGACTATTTGTTGAGGTGCAATTGGGGGGTGTAAATAGGGATTCTTTTGCAAGATTCCAAGAAAGAAATGTCTTGCATTGAGAAAAGGGTTGGGTTGTAATTTTCTTTCTTGGTGGTCCTCTTTTTCGGCTGGAGATAGAGTGGAGAAAAAAAGAATTCAATGCATCCAAAGGTGGAAAAAAAGGGGGGAAAAATTGTGATTGCACTAAATTACTAGTCTTTGGAATTTTAATTAGTTTGAAGATGTACACAATTTTTGGCTGCACCGGCCGTGGTGTGAGCCACATGAATCACGTTTTGTACTTTGGAAGATTTAGCATCAATTGATATACAATTGTTTACAATTCCAATGTTAGGTTGCTTTTCAAATGTTTGGTGTGGTCTTTTTAGCTAATAACCACAACATTGGTAGCCACAAACTCAATTCGTGTTCATGGGATAGGACTATTCTATAAGAGTATTTTCAATGCA

>AdNAC80

AGAAATAATAGAAAGCCCTTACAAAAATAAAAACAGAGGAAGGAAAAGAGTTCAAGAAACCAAAAACGTTTTAGCATAATGGGAGTTCCGGAGAAGGATCCTCTTTCTCAATTGAGCTTACCTCCTGGTTTTAGATTTTACCCCACAGATGAGGAGCTTCTTGTTCAGTACCTATGTCGCAAGGTTGCTGGCAACCATTTCTCACTTCCTATCATCGCGGAAATCGATTTGTATAAATTCGACCCTTGGATCCTCCCAGGTATATATTTGATAAGGGATAATGACATTATTTCTGTGATTGAGAGCAAGATGTTTATCTATTTGGGTTCTTTTTCTGCAGGTAAAGCAATATTTGGGGAGAAAGAATGGTACTTTTTCAGCCCCAGGGATAGAAAGTATCCGAACGGTTCGCGACCGAACAGGGTTGCTGGCTCTGGGTACTGGAAAGCCACAGGAACAGATAAAGTAATCACTACCGAAGGCAGAAAAGTTGGAATCAAGAAAGCACTTGTTTTCTACATTGGCAAAGCACCCAAAGGCACCAAAACAAACTGGATCATGCACGAGTACCGTCTCCTCAACGGTTCTCAAAAGAGCCTCGGCAGCACCAAGGTACAACATTATTCCATCAGAAGTATTATATATCATTTAAAAAATATATTTTCAATGAGTATTAACATTTGTTTTATGAATATTGGTGTTTTGCAGCTAGATGATTGGGTTTTGTGTCGGATATACAAGAAGAACTTGAGCTCATCGCAAAAAGTCAATATGCCAAGCTTTACGAGCAAAGAATGGAGCAATGGATCGTCTCCTTCTTCATCGTCTCACATCGACGACATGCTCGAATTGCCGGAGATCGACGACCGGTGCTTCGCCTTACCGCGGGTTAACTCGCTGCAGCACGAGGAAAAGCTCACCCTTGGCGCCACAGGCAATAATTTCCCGGACTGGGTCAACTCGGGGGGTCTCGACTCGGTCCCTGAGTTCGGGAGCCAAACTCAGGGGATGACAAGTTACGATGGAAATGACCTATATGTCCCCTCCGCGTCACAGTTCTGCCACGTCAACACAATGGTTGTGCCGGGTAACCCGACGGAGGAGGAAGTCCAGAGCGGCATCAGGACCCAGCGGATTGATGAGAATTTCGGGTTATTTCAACAGAATTCGAATGTATTCACCCACCGGTATTTGTCGAGTTCGGGTGACTCATTCGGATTCGGATACCCGAATCAGCAATTTGGATTCGGATTCAGAGAATGA

>AiNAC9

AGAAATAATAGAAAGCCCTTATAAAAATAAAAAACAGAGGAAGGAAAAGAGTTCAAGAAACCAAAAAAGTTTTAGCATAATGGGAGTTCCAGAGAAGGATCCTCTCTCTCAATTGAGCTTACCTCCTGGTTTTAGATTTTATCCCACAGATGAGGAGCTTCTTGTTCAGTACCTATGTCGCAAGGTTGCTGGCAACCATTTCTCACTTCCTATCATCGCGGAAATCGATTTGTATAAATTCGACCCTTGGATCCTCCCAGGTATATATTTGATAAGGGATAATGACATTATTTCTGTGATTGAGAGCAAGATGTTTATTTATTTGGTTTGTTTTTGTGCAGGTAAAGCAATATTTGGGGAGAAAGAATGGTACTTTTTCAGCCCCAGGGATAGAAAGTATCCGAACGGTTCGCGACCGAACAGGGTTGCTGGCTCTGGGTACTGGAAAGCCACAGGAACAGATAAAGTAATCACTACCGAAGGCAGAAAAGTTGGAATCAAGAAAGCACTTGTTTTCTACATTGGCAAAGCACCCAAAGGCACCAAAACAAACTGGATCATGCACGAGTACCGTCTCCTCAACGGTTCTCAAAAGAGCCTCGGCAGCACCAAGGTACAACATTATTCGATCACAAGTTTTATATATGATTTTAAAAAAATTATTTATTTGTCGCATTTGTTTTATGAATATTGGTGTTTTGCAGCTAGATGATTGGGTTTTGTGTCGGATATACAAGAAGAACTTGAGCTCATCGCAAAAAGTCAATATGCCAAGCTTTACGAGCAAAGAATGGAGCAATGGATCGTCGCCTTCTTCATCGTCTCACATCGACGACATGCTCGAATTGCCGGAGATCGACGACCGGTGCTTCGCCTTACCGCGGGTTAACTCACTGCAGCACGAAGAAAAGCTCACCCTTGGCGGCACAGGCAATAATTTCCCGGACTGGGTCAACTCGGGGGGTCTCGACTCGGTCCCTGAGTTTGGGAGCCAATCTCAGGGGATGACAAGTTACGATGGAAATGACCTATATGTCCCCTCCGCGTCACAGTTCTGCCACGTCAACACAATGGTTGTACCGGGTAACCCGACGGAGGAGGAAGTCCAGAGCGGCATCAGGACCCAGCGGATTGATGAGAATTTCGGGTTATTTCAACAGAATTCGAATGTATTCACCCACCGGTATTTGTCGAGTTCGGGTGACTCATTCGGATTCGGATACCCGAATCAGCAATTTGGATTCGGATTCAGAGAATGA

>AdNAC50

ATGGATAACAGGTTGGCCACAAACTCCTCTTATGCTTCTCTTAGATTGCCCGTTGGCTACAGATTCTGCCCCTCTGACGAGGTTTTTGTCTCTTGCTACCTCAAAAACAAGGCCCTTTCAAAAACATTGGATTTTGATGTTGTTCCTGTCTTCGATGTCTTCAACACTGAGCCCAAGAATCTCCCTTCAGGTTCGTGTAAATTAATATATATATTTAAATAATATTTTCCAGTTCTTTATGTATTTATTTATTTCAATATTAAATCTTCTATGACAGTGTTGTTTATGGTTACCTCGTTAATAATCAGTATTTTCACACTCATTAAAAATTAAAAAAAGAAATTACGTGATCAACATTTTCTTAAATTAATTCTTAATTTTTTATTACCGTTAGTCCTTTCTTGAGCCCTTAAACCTGAATGAATTTTTTTACTTTTGTGGTCTCTTTTTCCAATTTAATTATTTGTTTTTCAGGAGGAAAGGTGTTTCTGGAGACAAAGTACTTTTACTTTGATCTGAAAGAGCGTGTGTTTGAAGACAATAACAAGATTGAAGCAGGGAAAGGGCACTGGAAAAGGGTGGGGAAAGGGAATCAGGAGCTTCTAAATAACAACAACAAACTCATTGGGTTCAAGACCAAGTTTGTTTTTTGGAGGAAGAAGAACCGCACTCAATTTCTTAAAACTAAGTGGGTTATGTTTGAGTTCCGTGTTTTTCTCAACCCCTCTCAGGTAATTCATTTCTTTAGAAGATAGAATCTAAATGCAAAATGAAAGATCATGTTGCCATATACTATGCCACATTATCTTATCTGAATAATTATTTTGGTATATATTTCATATTCGTAACATGTGGTTTTTGGAAACAGATAATGTCATCATGGGCTGGCTACAAAATATATCTGAAGAAGGATAAGAGGAGGAACAAGAAGGCAAAGTTTTCTTGCGAGGAAAGCAGTGACGATGATGAAGAAGAAGCAGAAAGAGCAAGTGAAGTAAATTTTGCAGATGAGATAAGTGGAATTAACACAGGACCTCCTTCACCAACTTCATCTAATGAATCCTCTGTTACAAATTAATTATTAGAGCAGCAAAAATTGTTCAGATGAGTTAGGAAATTCAATAGGAGTTGCTTGCAGCATAGGTCTTTTAATTTACTAGTATGAGAAAAATCATTTAGAGCCCTTTGGATACCCATTAACAACTTTTTTTTTTCCTTTTTAGTCATTAGAGATTAGGCAAATGAGAATTGTATATGGGCGTTTATTTTGTGATCAAGGGCTCTAACATTTGGTGGTGGTTATAATAATAATAAGAAGCAACTTGTTCATAATTTTATTAGTAGTACTCTTCGCAAATATATATGCTTTAATTTATTTGCTA

>AdNAC33

TGTGGCTCTCTACTTTGATGGGCTAACATAGGGAATTGGAGGCTGCACGAGTGCCTTCAGTCTCTTCGCTGTTTGTCACCCAAGCGGACAAACATAAATACATAGTCTTACCACTGACCATTTTGGGGAGCATAGAACAGAAAAGGATGGAGGGGAGAGGGAGTAGTTTTGTGAAGAATGGGGAGCTGAGATTGCCTCCAGGATTCCGGTTTCACCCGACGGATGAAGAGCTGGTGGCTCAATACTTAAAGCGCAAGGTCTTCTCCTGCCCGTTGCCGGCCTCTTTCATTCCTGAGGTTGATATTTGCAAGTCCGATCCATGGGATTTACCAGGTATGACTAGTTGACAACTCTTTAAATTTTATTTCTCAAGCCTGTTTTCCTCACGTACACATACATCAGGTGATTTGGAGCAAGAGAGGTACTTCTTCAGCACGAGGGAGGCCAAATACCCCAACGGGAACCGATCCAACAGAGCCACTAACTCGGGCTACTGGAAGGCCACGGGCTTGGACAAACACATCGCAACTTCAAAAGGCCACCAACTTATTGGCATGAAGAAGACTCTCGTCTTTTACAGAGGCAAGCCTCCTTATGGATCAAGAACAGATTGGATCATGCACGAGTATCGCCTTGTCTCCCACTCCCACCTGCTTCCCATGCAAAATTGGGTTCTCTGTCGCATATTCTTCAAGAGGAGAGCACCGGCTAATGCTAAGAATGTTCTACTGGATCACAATTCCCATTCGGCATCAGCATCAGAGGCCTTCACCATCAGCCATGAGGGCAGCAACTCTAAGGTGGTTTTCTACGATTTCTTGGCACAGAACAGGGCTGATTTGAACCGCGTGCCCCCTCCTGCTTCTTCGACCTCTGGCACCAGTGGAATCACCACCGAATCCGATGAGCATGAAGACAGCAGTAGCTGCAACAGCTTTCCTTTTTTCAGGTGAAAACAACCTTAAATATAATTTTACTTGTTACTCTTGTCTTAGTAATTGGCTAGCTAGCTAGTGCACTTCCACACATGAACTGTGTGCACGCTGCGCTCTGCCTCTATCTTTCCCTTTTCTGTTTAGGTGTAACTAATTAATTAATTAACTATCGCCTCTTCCCTTGATGGAATGGATGTAGCAAGCATCAATCCCGCAGGAAACCGACCGGCATTAAAGGTAGTCTAAATCTGAATTTGAAGAGTATCCCGTTCTACTCATACTAGTATGCAATACAATTCTAAACTCTTCCATTTTCCCGTCTTTTCTTCTAGTTGGTACTAGTAATGCTCTCACCATGCATTGTTCTTTCACCCTACGGAATAAAAAGAAAAGAAAAGAAAAGAAAATCAGTCCCTTTTGAAGAAGGTGCTTTTACAGAGAAAAAGTGGGAGGAAAGAAAAAGATGAAAAACAGGGAGAAAAAGAGGGTGGAACCGATTGTGGAATCTAACGATTGTGTTTTAACATTTAAGGAGAACGACCAAATAACGAATAAGAGCATATTATCTTTGCGAAATCAGCATCCCACTTGTTAATTGCACTTATTCCCCTTTCCCCATGGGTTAACTTTGCCGAAAGGGAAAATAAAAAATTGA

>AdNAC74

GAGGAAGTGCCTTGCATACCACGATTCTCATCTCTCTCATCTCTCACAGCGCGTGTAAACGCTCTCTCTATTTAAGCACACCATCCTCCTTTCTTTCCACTTTCTTAAAACCTCTCCAATTCTTCTTCTTCTTCGCTTTCCGATCCTAATATAACCCCAATTTCTCTATTCCACTATATATATCGTTGAATTCAAAAAGATATTTAGTTTGATTGTGTTAGATGGAAGAGCCTGTCGTAGTTAACAAAGGCGAGGAGCCGCTGGATTTGCCACCAGGTTTCAGATTCCACCCAACAGACGAAGAAATCATCACTTATTACCTCACCGAGAAGGTCATGAACAGCAGCTTCAGTGCAACTGCCATAGGTGAAGCCGATTTGAACAAATCCGAACCCTGGGATTTACCAAGTAAGAAGTAGTAGTAATATTATGTCCGTACATGAGCAGTTAGTTAGTTAGTTATGAGTTATGAGTGATTATTTTCATTATTATTTGGTGTGCAGAGAAAGCAAAGATGGGAGAGAAGGAGTGGTACTTCTTTTGTCAGAAGGACAGGAAATACCCGACGGGGATGAGGACCAATAGAGCAACGGATTCCGGTTACTGGAAGGCAACCGGAAAAGACAAAGAGATTTTCAAAGGGAAAGGGAATCTCGTTGGGATGAAGAAAACGCTTGTTTTCTACCGAGGTAGAGCTCCCAAGGGTGAAAAGACCAATTGGGTCATGCATGAGTTCAGATTGGAAGGCAAATTTGCCAATTATAACCTCCCCAAGGCTGCAAAGGTCATATATATTATTATCACCTTTTACAAAAGTGCCTTTATTTCTTCTATGTTGGTTTTGGAGTTCCTTTCATGTTTGGTCCTTCTTAATTTTATTTCTATTTATTTTTTCTTCTTGTAGGATGAATGGGTTGTGTCGAGGGTTTTTCACAAGAACACAGATGTAAAGAAGACTACTACCCCATCATCATCATCATCAATAATTCCTGGCCTTTTGAGGATCAACTCAATAGGCGATGATCTTCTAGATTGTTCCACACTCCCACCTCTCATGGACCCTCACCCTACTCCTCTCGATACAACAAAATCCGACGGCTATTATTTCCCCTCCTTCTCATCATCACATCAGATTCTCAATATCAAGCCCGAAGAACACAACACAAGCCACCAAATTCCCATCACCAACTACCAGATTCCAAATTTCAATACCACACTCTCTTCTTCATCATCTCATCAAATCAGACTCCAAAATCATCTCAACTTGTTCTCATCATCATCATCAAATAACAATTACCATAATAGCTCGTGGCCAAGCTATTATGATGAGGTCCACCACCATCATCAAGATGATATTCTACTAAGAGCAATCGCCTCAAAGAACTATAGCAACGGAGGAGGAGGAGGCGGCGGCGAGTGCAAGGTGGAGCAATTCTCCTCCGGCAACCAGTCAGTGGTGAGCGTTTCGCAAGACACGGGGCTGAGCAACGACAGAACCACCAATGACACGTCATCGGTGGTTTCGAAGCAGCAGCATAATAATAAAACATTGTACGAGGATCTTGAAGGTCCTTCTTCATCAGTTGCACCTCTTTCAGATTTGGAATGCTTGTGGGATACCTACTGA

>AiNAC45

ATGGAAGAGCCTGTGGTAGTTAACAAAGGCGAGGAGCCGCTGGATTTGCCACCAGGTTTCAGATTCCACCCAACAGACGAAGAAATCATCACTTATTACCTCACCGAGAAGGTCATGAACAGCAGCTTCAGTGCAACTGCCATAGGTGAAGCCGATTTGAACAAATCCGAACCCTGGGATTTACCAAGTAAGAAGTAGTAATATTATGTCCGTACATGAGCAGTTAGTTAGTTAGTTATGAGTTATGACTGATTATTTGGTGTGCAGAGAAAGCAAAGATGGGAGAGAAGGAGTGGTACTTCTTTTGTCAGAAGGACAGGAAATACCCGACGGGGATGAGGACCAATAGAGCAACGGATTCCGGTTACTGGAAGGCAACCGGAAAAGACAAAGAGATTTTCAAAGGGAAAGGGAATCTCGTTGGGATGAAGAAAACGCTTGTTTTCTACCGAGGTAGAGCTCCCAAGGGTGAAAAGACCAATTGGGTCATGCATGAGTTCAGATTGGAAGGCAAATTTGCCAATTATAACCTCCCCAAGGCTGCAAAGGTCACCTTTTACAAAAGTGCCTTTATTTCTTGTATGTTGGTTTTCGAGTTCCTTTCATGTTTGGTCCTTCTTAATAATTTTATTTCTATTTCTTTTTTCTTCTTGTAGGATGAATGGGTTGTGTCGAGGGTTTTTCACAAGAACACAGATGTAAAGAAGACTACTACCCCATCCTCATCATCATCAATAATTCCTGGCCTTTTGAGGATCAACTCAATAGGCGATGATCTTCTAGATTGTTCCACACTCCCACCTCTCATGGACCCTCACCCTACTCCTCTCGATACAACAAAATCCGACGGCTATTATTTCCCCTCCTTCTCATCATCACATCAGATTCTCAATATCAAGCCCGAAGAACACAACACAAGCCACCAAATTCCCATCACCAACTACCAGATTCCAAATTTCAACACCACACTCTCTTCTTCATCATCTCATCAAATCAGACTACAAAATCATCTCAACTTGTTCTCATCATCATCATCAAATAACAATTACCATAATAGCTCGTGGCCAACCTATTATGATGAGGTCCACCACCATCATCAAGATGATATTCTACTAAGAGCAATCGCGTCAAAGAACTATAGCAACGGAGGAGGAGGCGCCGGCGAGTGCAAGGTGGAGCAATTCTCCTCCGGCAACCAGTCAGTGGTGAGCGTTTCGCAAGAGACGGGGCTGAGCAACGACAGAACCACCAATGACACGTCATCGGTGGTTTCGAAGCAGCAGCATAATAATAGAACATTGTACGAGGATCTTGAAGGTCCTTCTTCATCAGTTGCACCTCTTTCAGATTTGGAATGCTTGTGGGATACCTACTGA

>AdNAC44

ATGACTTCAACAAAATTGCCTTCAGGTGCAAGCAAGAAGTTCAAACCTACAGATGAAGAACTCATTCAAGATTTTTTCCGTAATAAAATCAATGGGCGGTCTCTACCAAATTATGGAACCATTCTTGAAGGTGAATTATTTGGTACGGAGAAGAATCCATGGAAAATTTGGGAAAAAACGTTAAAAATTCTTATGACGGGAAGGACCTCTATTTCTTCACTACTCTGAAGAGGAAGTTCTCGACTAACAACTTGAGGATGGTTCGCACCATCGAGTTTGGTTCTTGGGAAGGTGAAGACATCGGAAAATAG

>AiNAC40

GGAATAATAATAATTATGGGGAGCAAGAAGAGAGATACTTGTTCAGCTCAAAGGAAGTTAAGTATAGAAACGGTAACCGAATGAACAGAATAACGAAATCTGGATATTGGAAAGCAACTGGATCAGACAAAAGAATAATTTCAACATCATGTAATAATAATAATAATAGTAATATTGTTGGGATAAGAAAAACTCTTGTATTCTATCATGGAAAATCTCCAAATGGCTCTAGAACTCATTGGATCATGCGTGAGTATCGACTTGTCACTACTCCTTCTAATTCATCCCAGGTGATTATTTTTTCAGTTATTACTACAAAGTTTGTTTTTACAAGTTTTTTTTCTTTTATAATGGCCTAATTAATGGGTAGCCAAAATTAGGTTAAATTACACAGTTGGTCTATACATTTTTAGTGAAATTACAAATTAGTCCCTACATTTTAAAAGTTTGTAATTGAGTTTCTAAAGAGAATTAAAATTTGCAATTTAGTCCTTACCGTTCAAAAATGTTAATTTAACAGATATTCTCAGCGTATGCTAAGAATATTCTGTTAAATCAAACACTTTTTTGAACGACGGAGATTAAATTACAAATTTTAATTTTCTTTAAGGACTCAATTACAAACTTTTAAAATGTAGGGACCAATTTACAATTTCACTGAAAGTGTAGTACCAACTGTGTAATTTAATCCCAAAATTATTAGCATTAATTATATTCCATTTTTTTTAACTTTCATGTAATATTACACTCATCAATAATATTCTTTGATTTGTTATATTTACACCCACATTATATTTTACAACATACAAAAAATAAATGGTAGAGATCATTATTAATAAAATATTTATTTATCATCTTTATAATAGACATACGATTTAATTAATAAGCATAATCAATTAATTTCTCGTGTTAATATTTTGTTATTCTGTTTTCGCGACACTCTTATTATTTAAATTCTCAACATTATTGTCAATTTTTTTCCTTCAAAAAAATTATATTATTTTTGGAGATTATAAATATACTAGTGTGACTAAAGAATCTGATCAAGCTAAAACAGAATTTAGTATTGCTTATTTAACTAACTAACTAACTAACTTAACATTTAAGTAACATAACTTTTCTTAATTATAACACTAGATTTTATTTTGGCTTGGTCGTTATTCTTTAGTTACACTAATCTTTACAAAAATAATAGTAAATTATATATTAACTAAAATAGTAAGATTATTGTGTGTATGACCATATTTTATTTAATTTATGTATGATTGATTTTATGTTGGCAGAAGTATGTAGAAGACTTAGGGAATTGGGTTCTTTGCCGCATATTCAAGAAGAAAAGAAGCATAGAAAGTCAACATCACATGGTCAACAACAAAATTAATAATGTTGTCGAGGTGGCTAATAATAATAATAAGCCAATATTCTTTGATTTTATGAGGCTATATGACTCGCCAATATCTTCTTCTTCATCCTCATCTTCTTCTTGTTTAAGTTCTGATTTCATAACTCAAGTGTAAGAACATATGTGGTTATTATGTATGAATTTTTATATACTTTTGTANNNNNNNNNNNNNNNNNNNNNNNNNNNNNNNNNNNNNNNNNNNNNNNNNNNNNNNNNNNNNNNNNNNNNNNNNNNNNNNNNNNNNNNNNNNNNNNNNNNNNNNNNNNNNNNNNNNNNNNNNNNNNNNNNNNNNNNNNNNNNNNNNNNNNNNNNNNNNNNNNNNNNNNNNNNNNNNNNNNNNNNNNNNNNNNNNNNNNNNNNNNNNNNNNNNNNNNNNNNNNNNNNNNNNNNNNNNNNNNNNNNNNNNNNNNNNNNNNNNNNNNNNNNNNNNNNNNNNNNNNNNNNNNNNNNNNNNNNNNNNNNNNNNNNNNNNNNNNNNNNNNNNNNNNNNNNNNNNNNNNNNNNNNNNNNNNNNNNNNNNNNNNNNNNNNNNNNNNNNNNNNNNNNNNNNNNNNNNNNNNNNNNNNNNNNNNNNNNNNNNNNNNNNNNNNNNNNNNNNNNNNNNNNNNNNNNNNNNNNNNNNNNNNNNNNNNNNNNNNNNNNNNNNNNNNNNNNNNNNNNNNNNNNNNNNNNNNNNNNNNNNNNNNNNNNNNNNNNNNNNNNNNNNNNNNNNNNNNNNNNNNNNNNNNNNNNNNNNNNNNNNNNNNNNNNNNNNNNNNNNNNNNNNNNNNNNNNNNNNNNNNNNNNNNNNNNNNNNNNNNNNNNNNNNNNNNNNNNNNNNNNNNNNNNNACTAATTAAACCTACATATAATTATATATATATGACAATATTGTTATATATCATCTTTTAATGATTAATGTCTGAGAATTATTATAACTAACTTTAATTTTATAACTAACTTTAATTTCTATTGAAGATATTGATAATTTTTCACAATGTTACATGA

>AdNAC41

ATGATATCAGAGCTTCTACGATCGAATGGAGTAATGAAATACGTGGTATATGCAGAGGTGGCAAAGCTAAATGCAAATGAATGGTACTTCTTCAGCTTCAGAGACAGGAAATATGCGACGGGGTTCAGAACGAACAGAGCGACGACAAGTGGTTATTGGAAAGCAACGGGGAAGGATCGAACGGTGCAGGATCCACTTACGCAAGAGGTTGTAGGGATGCGCAAGACTCTGGTGTTCTACCGGAACAGAGCTCCCAATGGCATCAAAACTGGCTGGATCATGCATGAGTTTCGGTTGGAGACCCCACACATGCCCCCTAAGGTACTTTCCTTTTTCTCTATTTTCAAACTTCTTAATTAATTACTTCTCATACCTTCATATGCATCCCGTGACAAAACTTCATCATCATTATTAGCTCTAGTGCAAATTTTGAACTTGATACACACCATCACAAAACCATTATGTTGTTGAATGGTTTAATTTAGGGTATGTTTGGCAAAATAACAGAATTTTATGGATTTAAATGTTCTAAATTTTGAATTATTTTAGAGGATACAGAATAATCTCTCATCTTTAAATATTTTTTTTCTTATATTTTTTCTTAGTTACGCCTATAAAATAAATGATGAGAAATCACACTTTATCCTCTAAAATGAAAATTTAAAATTTAGAGGATCCAAATTCGAATTTTATTGTTTCATTTTTTTTCACAAAATATTAAAAATAAAAAAAAAAACAAAACAAAACCAAATACGCCTTTAAGTTTTTTCTTTTTAAATGAAAAGACTAGTTTAATTTTAGGTGGAACTCAGTTACAGTCGGGTTCACATAAAGTTAATAACTAAGAGTGTTAGATGATTTGACTAAATTTTTATTTAACGACTCTCAACTATCAACTTCACGTGAAGTCGACCGCACACGAAGTTTTACCTTAATTTTATTAGGAAAAGTTGTGAAGGGTAAATAAAAAAGTGAGTAGCAAGGGAAAAGGTTTGATATATAGGGTTTGTTTGGCGTGTGTATGTGTGTATTATATTTTTGGTGTGAGGTTAAAGGGTCTTTAAGGAAGCGTGTGTTTGATTACACAGGAAGATTGGGTGTTGTGTAGAGTGTTCCACAAAAGCAAAGAAGAAAACAGTGGCAAACTTATCATGTATGATTCCATTTCCACACATCATGAATCATCAAACTCTATGGCATTGGTATCAACCCATCATTTGAACCCCATCAACAACCATAATGCCATGAATAATTTCCTTCATCACTTCTCATCATCAAGGGATGATAGTCAAACAAATAACGCCAATAATAATAATAATAATAGTCCCAAGGGTTATGATGGATATGGCTTCATATGGGATCACATGGATCTTGAAGATCATAGTGTGCCCTCATCAGACTTCCAGGTCGACAATAATAATAATAATAATAATATTGCATTGCTATAAGAAGCTAAGATACTAATTAGGTGTATTATTGTATTATTTGTAAATATGCATATTGTATATGGAAAGTGTGAAGAATTCGTTGTTGTATGTATTGATTATGGTGTGTGTGTGTGTGGCTTCGAGTGGCCAAGTCACGTGATATGTACTATATTATGCATATATATGCTCGTTGTGTAATTAGTAGTGTTAGTGTACGTATTATTGGATTAATTAGGCTCTTTATAATTTTCTTATTTTGGTGTATCCTTCTTCTCCTTAAAATTAGCCCTTTCTTTATTTTTCTCATAATGTATGTATCCCAGT

>AiNAC11

ATGCACAAAAACACAAGGGATGTAAACAGTTACGTGAAAGGCTATCTAATTAATTGGGTGAAGTGTTCTTGGTCAAGTTTTCAGTTTTGGATATCAAAACTTAACTTATCCCATAAAAGTAATAAAGACACAAACCTAAAATATAAAAAGCATGAAACTTCCCAATAGTTTTTCTATCTATAACTATAATAAAGCCAAGGTTCTCTGAACTATGCACCCAATTTTTCAAATATATATATAAAATCCACTATTTATAAGCACTTAATAAGAACAACATCTTCATTCAACTCTACTCCTTCCATTATTCTCTATCTCTCTTTTTTCTTCTCTTCAGTTACATAACAATTTAATACATCAAGACCAATAATAATAGAAATAATAACATAGAAATGAATACAAAGATTGAACTGCCACCAGGTTTCAGGTTTCATCCAACAGATGAAGAGCTCATAACTCACTACCTCTCTCAGAAGGTTGTTGGTAGCTGCTTCTATGCAACTGCCATTATTGGAGAGGCTGATTTCAACAAGTGTGAGCCTTGGGATTTACCTTGTCAGTATCTCAATTGTTATTTTCCCTTTCAGAAATAAATTGGGTTTTCATCATTTGATTCTCTTTTTATTATGTGCCTTTGAAATATATTTTAAAAATATTCATCATTATTCAATCATTCTTTTTTTAATGTAAATCTTAATATTATAAATGAGATTTTAACTAGTTCTGAATATTTAAATAACATGTAAATGACTTTTTCCTGAAAATTTCTTAAATGCCTATTTAAGTAGAAAATATGTATAATAAGTAACTTTTTGTTTATGGCATTATATTTTTTAGTTATAAACTGCCGCCAATAGTTACTGGAAATTACTGTGAATTTGTTTCTCTGTGTCTTAAAATATGATGTTTAAAATGTTGATATTTCATTTTGCAGCGGTTTAAAATCGTCGTAAAATATTTAAAAAATTGTTGTTATTCTATCTTGGTGTATACTTATATATATGTAGAAATATAGTTTTTAATGATATTATTTTTTATTTAGCATCCTTTGATTTATTTTGACTTCAAAATTTGTGATTTTTATTTATTATTTTAATTTTTTTCAATGAAGGGAGGGGCAAAATGGGAGAAAAAGAATTGTATTTTTTCTGTTTGAAGGACAAAAAATACCCAACAGGTGAAAGGACAAATAGAGCCACTGGCGCTGGGTACTGGAAGGCCACAGGGAAAGACAGAGAGATATACAATGCAAAAGCAAAAGCACTTATTGGGATGAAGAAAACACTTGTTTTCTACAAAGGAAGAGCTCCAAATGGTGAAAAGACAAATTGGGTCATGCATGAATATAGGTTGGAAGGCGATAATAAACCTTCTATATACAATCTACCCAAAGAAACCAAGGTAATTAGAGTTCAATCAGTATGCATCAGCCATGTCAGAAAAAAAAATTATATTGTCATTTAATTTGATGCATGTATTTATAACTTTTGAATTAGTAATACATTAATCTCTTTGGATGGAGTAGTTATTTTTGTCGATATAACCGTATTTGTACATAAAAAGTATGAATACACGTGTTGTATATATGTGACATTTAACCAAAAAATAAAATAAAAAACTATAAAAGTACATTTTTAGTATGAATAATATTAAGTCATTAACTTTTTTTAGCTAACAACAGTTAATTTTTTAAAATTATTTTATTTATTTTAAATTTCAGACTTTAAATCATAAATTTTTTATTTTTAATTATAAATTATAAATTTAAATATATAAAATATATATTAAAAATAATTTAAATAACACATGTATTTATATATAAAATATATGAAAACTTTTCGTGTATACATATCCGGATACCTATATATTCATGCAACTTTCGTTCAATTTTACACTATTAATTTTATACATAATGCATATGTATGGCTTATTTTTGAGATAGTTAATAATTGAGTTAACATTTATATATACTGATATAGTAATACATAACTGAATATCTATATAAAATAAAATATTTAACATTAACAATTCATGCAAATTAATTTGATTTGAATTTAGTATTTGTTAACATGGTTCCTTTTCTATTTTTATTTTTTCATTTCAGAAAGAGTGGGCTTTGTGCAGAGTTCTACACAAAAGTGAAAAGAAAGTAATGCATGTTCCACAACCACAGGGATTGGTTGAGTTCAGCTCCTATGAAAATAAGGAACTTCCCCAATTAATGGATTCTTCACAAGTAACATTCTTTTCATCAGACCCAAATAATCAAAGTGAGGATCCAAATCCAATCACACGTGATGATGATAATAATAATGATGACATCATATTTGATAGCATTGAAACTCCTTTCTTGGAACAACAACCACCTTATTCTTCATCCTATGATTCTTCAGATTTAGACACCCTTAACCCTGCCACATGGGATATTTCCGAAAATGCCCCTACAAGTAATGCGTCTAAGGAGACGGACTTTGATGCTGACATGTTCTCTTTGATGTACAACAATAGAGAAGTGTTCCAAACATCATTTGAGAATCAGGAATATTATGCATATGATTCTGTAGGACATGTGGACAATGGTTCCCTATGGAATTTTTAG

>AdNAC53

GTTTCAGGTTCCATCCTACTGATGAGGAACTTGTTATCTATTACCTCAAGCGCAAGGTTTGCGGCAAAAGCTTCCGATTTGATGCAATTTCTGAGGTTGACATCTACAGGAGCGAACCCTGGGACCTTGCAGGTTACTCCCTTTTACCTAAATCTGTCTCTTTTTTTTCTTCATTTGTGCTGTAAAAATCAAAGCTTGTTGAATTTGTTGTTCTTCGCTGAATGTGTGTTTTTTTTGTCATGGGTTTTGATTTTCTTAGTTGAATTTGTAATGGGTTGTGAAAAAAATTTAGACTTGGTGATGTTTTGTTTCTTGGGGCGTGTTGTTGTTGTGCTTGTTAACTCATTTAGATGATGTTTGGTTGAATGTTTGTTTAGATAAGTCGAGGTTGAAGACTAGGGACCAAGAATGGTACTTCTTTAGTGCACTGGACAAGAAGTATGGCAATGGTGGGAGGATGAACAGGGCCACAAGCAAAGGATACTGGAAGGCTACAGGGAACGATCGTCCGGTTAAGCATGAACAAAGGACTGTGGGGTTGAAGAAAACTCTGGTGTTCCATAGTGGAAGAGCCCCAGATGGTAAGAGGACCAATTGGGTCATGCATGAGTACCGACTCGTCGACGAAGAGCTGGAGAGGGCTAGGTCTGGATCCTCTCAGCCTCAGGTGAGATTATTGAGTTTACAACTTGAAGTATGAGGTGAGCCTAATGTGTTTTTTACCACGGCGAACCACGTATGAGGGCTATCCTCATTTTGATGTTGATAAAATAAGGAGCTTTGTTGTTGCTTAAAAAAAGGAGCTTTGTTGTTTTACTTGGTTCCAAAATCCAAATTGTTGTGTAAAACTTGGATGCAGGAGTTTGAAACTTTGTTGTTTAGTAAACGTGGAAAGTTTAGTTTGCTATGTGCTCTACTACAGTATGGCCATTGTTATTATATCTCAACAGCTGGTTAATCATTTTTGTGCATTTCTCTTTCGGATGGAGAAGGATGCATATGTTTTGTGTAGAGTTTTTCACAAAAATAACATAGGACCTCCGAATGGGCAACGTTATGCACCTTTCGTTGAAGAGGAGTGGGATGATGCATCGGCATTGGTTCCTGGGGCAGAACCTGTGGAGGATGTTACCGTCACTGTTGCCCATCCTCTACGCATTGAAAGCAACGGTCGCACTTTATGCAGCGACAGGAGAAACAATGTTGCACAGGTTTGTTCCCTTTGGTGCTCCCTCCATCCAACAATATTGTTAGCTTTTGACAAACCGAGTACATTTCAAAAGTTGATGTATTTGAGCACAAATTGATCTTAGATACATTGACTTTTGAATGTACTTGGTTTGTCAAAAGCGACCTATATTATTGGAAGGAGGGGATACTTTTTGGTTATCATTTAATCATTGTGATTATATTTAGTTAATTGTGTTGATTCACCTGGAAATAATTGTGTTGATGCTTTATGTTTTATGTGTGCAATGACGTATTGAATTCACGTGGTTTGTCAAAAGCGATCTATATTAATGGAAGGAGGGGATACTTTTTGGTTATCATTTAATCATTGTGAATATGGTTAATTGTGTTGATTCACCTGGAAATAATTGTGTTGATGCTTTATGTTCTATGTGTGCAACGACGTATTGAATTCACGTGCTAAAAAAGAATTGGATATATGAAAAGTCGGCGACATTTGTGCGCGTTAATTAATAGTACAATTGGTAACTATGCCGAGTGGTTCGGAACCTTTGCAGCATTTTCTATTTTCTTCCTTGTCTTAATGAACTTCTTATTATAACGAATAATGAAAATAATAATTGCAATATTCCCATATTTTAAAAGGTTTTGTTAAGGAGTGCCCTTAGAGCAATAATTATGGATACAAATGGGTTTCTTATTCAAAAAAATTAAGTTTCAAGTCTTCACTGACATTAAATGCATTAAATCTTGTTTTCTAAATATAAAAGATTCCCCTTTTGTACCCTTAATGCTTGCCCGATCCTTAACGCACTTGTTAGCAAGACCTATTGTAAATGCGCAATGTAAATGCGCAATAAGAGTATTTCCCCCGACTACTTCCGCCCTTCTAGCAGAACCATTGCCCTTTTGCATTTCATTTGATCATCTTAGAAATTGTAGTTTCTTATGACCTTTCTAGAAGATCAGGAATTTGGGAACTGAGTACTATTCCGTTTTGAGTAAAATTGTAGGTGATACTTTTGTTTATCTGGTCAGAATCCATTATGAGCAGCAACTTTTTCCCCCATCTATACTTGTGTACTTAGCTTGTAATTAAAATCTGATTGCTTTTCCTTACTTTAAATAAATTTTATCTTGGAGAGGTTTTTGCAACAAGGAAAGTTGCATTTCCAGAGAGACAATGAGGTAATTAATCTGGGCCTTGTCTTTCTTGCCAATTTACAAGAATCATGTCACTGCTTTTATTGTTATTATTATAAAGAGAAGACTCTATCGTCAGTAAGGAGGATGGAGCTTCTAACTGGTGCTTCAGATAATTTGGTAAACTTCCTTGATTGTTTGGGTCAATTGACAAGGGGCTTGATAGTATCTAATGCTACTGACATGAAGTTGGAATTGCCAATGCTATTTTACTTCAGGAAAGTAGCCTCTGTTGCCCCTATGAGTATGGTGCATTGGGTGGTTATCTCATATTTGTTGATTTCTGGTTTCGCAATAAACATTTTGAAGTTTATTATATGGGCCTCTATAACCCATACTTACCAAAGAAGAGCAGAGAAGAGAAGCAAGAACGAGATGGTCTATAAGGCTACGTGCCCAAGGGAGGACATTGAAGGTCTATGAGGGAAGGAAGAGAGTGAATGATCAAGGTTAGAAATCGGAATGAGCTAGAAAAAGGCCCCATCGAGGAAATGCTTATACTGATGGGAGAATTGTTGAACCGAATTTACACCTGGCAGCAATGGAGGGACTTGAAAATGTGGGGAAATGTGAATATGGAAGCTTGAGAGGGTAGTGTGACATTGGCAATTTAGCATTGGGTTGTCTGGGCCGTGTGGGTTGGCTTGAGCCACTGGACAGATTTAGCCTCCTGCTTCTCTGATCTCCACCCCCCTTTCTCTCTTTTCTCGTATTAAAACTGCAGCTGCGCATACTCATTATGATTGACAGCTCTATAACTGTAATTGAGGGCTTGCCTCGGACTATCTATCAACCCGGCTTACCATTAGTTCCATATTCTATCATATGACTAGTAGTGGAAAGTGGGTAAATACTTGACAGATCATACTTATACCCTAGAAGATATTGAAGTAACTGAATGATCATTGATTTTATTGCGGAGATTTCACGCTATCCTTGACGGAGAATTTTGACCTAGTCCTTGGTAGGTATTCTTTTTTCCCTAAATGAGGTAGCGTTTGGAAGAGAGAGAGACTGGGAGACAGAGACTGAGAGACAGGCCGTGCCACTGCATTTAGGACCTCTTCACTCTCTCTCTCCCTCTCTGCCGCCGTCGCCCAGCACCCATGGTCCACTCTGATCCAGCACCCGTCGGCGGCTCCTCTTTCTGCGCCGCTGTCTCCTCCTCCTTCCTTCCTCTCCTTTATTCTCTTCTCTTCTCTTCTCCCTCTTCTGATTTTGAGATATGTTTGATATCGTTGTTGTTGGTATTATTGTTCTGATTCTGATATTGTTCTTGGTATTATTGTTGTTGGTGGTGATGGATTGCAAGGAGGGATGATGGCAGAAGTTTGGTGGTAGGGATGAGAGATAGGGTGGAGTTGGGAGATGAGAGGTGGTGATCCTGGCGGCCGCGATAGCCGATAGGGCAGTGGTGGTTGAAGAACTGAACGAGGGTAAACTTGGAATTATTATTTTGGGATAAGGGTATTTTGGGAAGAAATTGTATGTTAAAGTTTCAGTATCTGTCCACAAAATTTCAATTTGCGGTGTCTCTACTTTCTGGAGATACTAAAAGAACTGAAATTTTGGAGACGAAGACCAAAATATTAGTTCGAATCTCTGGCTATCAAACACAATATTGAGTCCTAGTCTCCCAGTCTCAGTTCCAGTACCGTTACCAAACGCTACCTGAGAGAAGTTGTTCATCTTCATAGAATTGAAGCTGTTGGGAGATATTCAGAAAATGGAGATCAATGGATATTCTCCTGTATTAAGGATATGTCAGTTACTTTGTATTACTGTAAGGAAAATTGCAACCTACCTGTTGGGATTATCCTGAATCCAACAGCTTCTTGAACTGACGTATATCGAGAGCAAATTGATGTTCTCTTGATTAGTATAGTGGAGATCATGAAAACAAATTGAGGATGACTTGTGAGCAAGAGGAACCCCATCTACAATGGTTTTCCAGCACACACTGATAAGTTTGTTGTTTCTTTGAAAGTTCACCTATATTAGGGGGAAATGGTCTTGGGGAATATTACACCCTGCTGATTCCAGAGATACATAAGAAAAGTTTCAAGAGATAGGCTTTGTATGATCTATTAAGGGTGAAAGGATACTCACCCATTCGATGCTTCAAGTTTGTGCTTTATGGGCAATCAAAACTTGGCCGCACTCTCCCTTAGTTCAAAGGCTCAATTTAGTCGTCTTTACTCCTTGTCAACTTCACCCATTGTTCCTGTGTAGTTCTGGTTTCTCTTCCTTTGATAGTTCTTCATTGCAATTGTGGCTCACTTTTCTTCTCTTTCACTACTTGTGTTCTATTTTGTTCTGTTTTAGATTTCATCTTTTGTTTTAGTTTCGTTTCTCCTACTGTAATTGATACCAGATTGGTTGCCTCTAACTTCTGTTAAATTTTGAAAATGATGTAATGATCAATCTGGAGTTAACTTATATTGTATACATCATTAATTGGATTTAATGTCTGTTACTTGTTACAGCACTTTGCATTCTAACATGTCATTGTTGTATTCTAATTTCAGGATACTCAATCTAACAACAAAGTTCCATTTGATGTGAACAAGCTTCCCATTGAAACTCAAAGTCTGCTAGCTGTCTGCAAAAGGGAGAGTATGGCCGAGTTTCCATCACCTGAAAAGGAGGATAACTCGAAGCGTCAGATCGATGAGTATCCTTTGCCACAAACAGAAAACACCAAGCCTATCTCTCAAATATACAAGAGGAGGAGGCATTATTTGAATGTCAACCATTCAAATGTTAACGGAGATTCAGTCCGAACCATCCAAGAACCGCCATGTTCATCAACAATAACCACCGCCGCAACGACACTCCCGACGGTCGCCACCACAGCCTCCACTGCAATAACCAACGTTGCACCTAAAAAACATTTCTTGTCTGCACTGGTGGAGTTTTCTTTGATGGAATCCCTCGAATCAAAGGGAAATGCATCCGTTCAACCACCAGAGTTTGATGATGCTTCCTTGGAGGCATCCGTGCCGCCAAATTGTGTTAAGCTCATCAAACGCATGCAGGGCGAGATTTACAAACTTTCCGAGGAAAGGGAGACTATGAGGTTTGAGATGATGAGCGCACAAGCAATGATTAACATGCTCGAGTCGCGCATTGAAATTTTGAGCAAAGAAAATGAGGAACTGAAGAGCATGATTAATAACAATCCTTAGGGGATTAGCGCAGTGGCCATGTAATCTTGCTTATTGCAACAGAGTGATGGTGCTCTCAGTCTACCTGATCTCTTGTTATCTTTTAAGAGTTAAGAACTGTAGTCTTCTTCATTTGGCTGAACTTTCTGAGTGGTATTTAAACTTTGCCATTTGTGTAGCCTTGTAGCTTATGACTTGTTACCCTTGTTCATCAATGGCTATGTTAGTCCCTCCTTCCTTTAAGGCTTGTCCTTTCTTGTGAATGAATAGCAACTCAATTAGTTAGGCCATTCTGAACAGATTTTTCTCATAGAGGGGTTTGGCAAGTTGGGAATGGATCCTGTGCATAGATTTCACAAGATGAGT

>AiNAC39

GTTTCAGGTTCCATCCTACGGATGAGGAGCTTGTTATCTATTACCTCAAGCGCAAGGTTTGTGGCAAAAGCTTCCGATTTGATGCAATTTCTGAGGTTGACATCTACAGGAGCGAACCCTGGGACCTTGCAGGTTACTCCGTTTTACCTAAATCTGTCTCCTTTTTTTCTTCATTTGTGCTGTAAAAATCAAAGCTTGTTGAATTTGTTGTTCTTCGCTGAATGTGTGTTTTTTTGTCATGGGTTTTGATTTTCTAGTTGAATTTGTAATGGGTTGTGAAAAAAATTTAGACTTGGTGATGTTTTGTTTCTTGGGGTGTGTTGTTGTTGTGCTTGTTAACTCATTTAGATGATGTTTGGTTGAATGTTTGTTTAGATAAGTCGAGGTTGAAGACTAGGGACCAAGAATGGTACTTCTTTAGTGCACTGGACAAGAAGTATGGCAATGGTGGGAGGATGAACAGGGCCACAAGCAAAGGATACTGGAAGGCTACAGGGAACGATCGTCCGGTTAAGCATGAACAAAGGACCGTGGGGTTGAAGAAAACTCTGGTGTTCCATAGTGGAAGAGCCCCAGATGGTAAGAGGACCAATTGGGTCATGCATGAGTACCGACTCGTCGACGAAGAGCTGGAGAGGGCTAGGTCTGGATCCTCTCAGCCTCAGGTGAGATTATTGAGTTTACAACTTGAAGTGTGAGGTGAGCCTAATGTGTTTTTTACCATGGCGAACCACGTATGAGGGCTATCCTCATTTTGATGTTGATAAAATAAGGAGCTTTGTTGTTGCTTAAAAAAAGGGAGCTTTGTTGTTTTATTTGGTTCCAAAATACAAATTGTTGTGTAAAACTTGGATGCAGGAGTTTGAAACTTTGTTGTCTAGTAAACGTGGAAAGTTTAGTTTGCTATGTGCTCTACTACAGTATGGCCATTGTTATTATATCTCAACAGCTGGTTAATCATTTTTGTGCATTTCTCTTTCGGATGGAGAAGGATGCATATGTTTTGTGTAGAGTTTTTCACAAAAATAACATAGGACCTCCGAATGGGCAACGTTATGCACCTTTCGTTGAAGAGGAGTGGGATGATGCATCGGCATTGGTTCCTGGGGCAGAACCTGTAGAGGATGTTACCGTTACTGTTGCTCATCCTCTACGCATTGAAAGCAACGGTCGCACTTTATGCAGCGACAGGAGAAACAATGTTGCACAGGTTTGTTCCCTTTGGTACTCCCTCCATCCAACAATATTGTTAGCTTTTGACAAACTGAGTACATTTCAAAAGTTGATGTATTTGAGCACAAATTGATCTTAGATACATTGACTTTTGAATTTACTGATTTGTCAAAAGCTATCTATATTATTGGAAGGAGGGGATACTTTTTGGTTATCATTTAATCATTGTGAATATGGTTAATTGTGTTGATTCACCTGGAAATAATTGTGTTGATGCTTTATTGAATTCACGTGCTAAAAAAGAATTCGATATATGAAAAGTCGGCGACATTTGTGCACGTTAATTAATAGTAGAATTGGTAACTATGCCGAGTGGTTCGGAACCTTTGCAGCATTCCCATATTTTAAAAGGTTTTGTTAAGGAGTGCCCTTAGAGCAATAATTATGGATACAAATGGGTTTCTTATTCCAAAAAATTAAGTTTCAAGTCTTCACTGACATTAAATGCATTAAATCTTGTTTTCTCAATATAAAAGTTTCCCCTTTTGTACCCTTAATGCTTGCCCATCCTTAACGCACTTGTAAATGCGCAATGTAAATGCCCAATAAGAGTATTTCCCCGATTACTTCCGCCCTTCTAGCAGATCAGGAATTGTTGTTTCTTATGACCTTTCTAGAAGATCAGGAATTTGAGAACTGAGTACTATTCTGTTTTGAGTAAAATTGTAGGTGATACTTTTGTTTATCTGGTCAGAATCCATTATGAGCAGCAACTTTTTCCCCATCTATACTTGTGTACTTAGCTTGTAATTAAAATCTGATTGCTTTTCCTTACTTTAAATAATTTTTATCTTGGAGAGGTTCTTGCAACATGGAAAGTTGCATGTCCAGAGAGACAATGAGGTAATTAATCTGGACCTTGTCTTTCTTGCCAATTTACAAGAATCATGTCACCCCTTTTATTGTTATTATTATAAAGAGAAGACTCTATCGTCAGTAAGGAGGATGGAGCTTCTAACTGGTGCTTCAGATAATTTGGTAAACTTCCTTGATTGTTTGGGTCAATTGACAAGGGGCTTGATAGTATCTAATGCTACTGACATGAAGTTGGAATTGCCAATGCTATTTTACTTCAGGAAAGTAGCCTCTGTTGCCCCTATGAGTATGGTGCATTGGGTGGTTATCTCATATTTGTTGATTTCTGGTTTTGCAATAAACATTTTGAAGTTTATTATATGGGCCTCTATAGCACATACTTACCAAAGAAGAGCAGAGAAGAGAAGCAAGAAGGAGATGGTCTATAAGGCTACGTGCCCAAGGGAGGACATTGAAGGTCTATGAGGGAAGGAAGAGAGGGAATGATCAAGGTTAGAAATCGGAATGAACTAGAAAAAGGCCCCATCGAGGAAATGCTTATACTGATGGGAGAATTGTTGAACCGAATTTACACTTGGCAGCAATAGAGGGACTTGAAAATGTGGGGAAATGTGAATATGGAAGCTTCAGAGGGTAGTGTGACATTGGCAATTCAACATTGGGTTGTCTGGGTCCTGTGGGTTGGCTTGAGCCACTGGACAGATTTAGCCTCCTGCTTCTCTGATCTCCACCCCCCTCTCTCTTGTACTAAAACTGCAGCTGCGCATACTCGTTATGATTGACAGCTCTATAACTGTAATTGAGGGCTTGCCTCGGACTATCTATCAACCGGCTTACCATTAATTCCATATTCTATCATATGACTAGTAGTGGAAAGTGGGTAAATACTTGACAGATCATACTTATACCCTAGAAGATATTAGAAGATATTGAAGTAACTGAATGATCATTGATTTTATTGCGGAGATTTCACGCTATCCTTGACGGAGAATTTTGACCTAGTCCTTAGTTGGTAGGTATTCTTTTTTCCCTAAATGAGGTAGCGTTTGGAAGAGAGAGAGACTGGGAGGCAGAGACTGAGAGACAGGCCGTGCCACCGCATTTAGGACCTCTTCACTCTCTCTCCCCCTCTCCGCCGCTGTCGCCCAGCACCCATGGTCCACTCTGATCCAGCACCCGTCGGCGGCTCCTCTTTCTGCGCCGCTGTCTCCTCCTCCTTCCTTCCTCTCCTTTCTTCTCTTCTCTTCTCTTCTCTTCTCTTCTCCCTCTGTTCTGATTTTGAGATATGTTTGATATCGTTGTTGTTAGTATGATTGTTCTGATTTTGATATTGTTCTTGGTATTATTGTTGTTGGTGGTGATGGATTGCAAGGAGGGATGATGGCAGAAGTGTGGTGGTAGGGATGAGAGATAGGGTGGAGTTGGGAGGTGAGAGGTGGTGGTCCTGGCAGCGGCGATAGCCGATAGGGCAGAGGTGGTGGTTGAAGAACTGAACGAGGGTAAACTTAGAATTATTATTTTGGGATAAGGGTATTTTGGGAAGAAATTATATGTTAAAGTTTCAGTATCTGTCCACAAAATTTCAATTTGCGGTGTCTCCACTTTTTGGAGATACTGAAAGGATTGAAATTTTGGATACGAAGACTGAAATATTAGTTCCAATCAAACACAATACTGAGTCTCAGTCTCCCAGTCTCAGTTCCAGTACCCATTACCAAACGCTGCCTGAGAGAAGTTGTTCATCTTCATAGAATTGAAGCTGTTGGGAGATATTCAGAAAATGGAGATCAATGGATATTCTCCAGTATTAAGGATATGTCAGTTACTTTGTATTACTGTAAGGAAAATTGCAACCTACCTGTTGGGATTATCCTGAATCCAACAGCTTCTTGAACTGACGTATATCGAGAGCAAATTGATGTTCTTTTAATTAGTATAGTGGAGATCATGAAAGCAAATTGAGGATGACTTGTGAGCAAGAGGAACCCCATCTACAATTGGTTTTCCAGCTCACACTGATAAGTTTGTTGTTTCTTTGAAAGTTTACCTATATTAGGGGAAAATTGTCTTGGGGAATATTACACCTGCTGATTCCAGAGATACATAAGAAACGTTTTAAGAGATAGGCTTTGTATGATCTATTTAGGTGAAAGGATACTCACCCATTCGATGCTTCAAGTTTGTGCTTTATGGGCGATCAAAATTTGGCCGCACTCTCCCTTAGTTCAAAGGCTCAATTTAGTCGTCTTTACTCCTTGTCAACTTCACCTATTGTTCCTGTGTAGTTCTGGTTTCTCTTCCTTTGATAGTTCTTCATTGCAATTGTGGCTCACCTTTCTTCTCTTGCATTACTTGTGTTCTATTTTGTTCTGTTTTAGATTTCATCTTTTGTTTTAGTTTCGTTTATCTTTTGTTTTAGTTTCGTTTCTCCTACTGTAATTGATACCAGATTGGTTGCCTCTAACTTATGGTAAATTTTGAAAATGATGTAATGATCAATCTGGAGTTAACTTATACTGTATACATCATGAATTGGATTTAATGTATGTTACTTTGTACAGCACTTTGCATTCTAACATGTCATTGTTGTATTTTAATTTCAGGATACTCAATCTAACAACAAAGTTCCATTTGATGTGAACAAGCTTCCCATTGAGACTCAAAGTCTGCTAGCTGTCTGCAAAAGGGAGAGTATGGCCGAGTTTCCATCACCTGAAAAGGAGGATAACTCGAAGCGTCAGATTGATGAGTATCCTTTGCCACAAACAGAAAACACCAAGCCTATCTCCCAAATATACAAAAGGAGGAGGCATTATTTGAATGTCAACCATTCAAATGTCAACGGAGATTCAGTCCGAACCATCCAAGAACCGCCATGTTCATCAACAATAACCACCGCCGCAACGACGCTCCCGACGGCCACCACCACAGCCTCCACTGCGATAACCAACGTTGCACCCAAAAAACATTTCTTGTCTGCACTGGTGGAGTTTTCCTTGATGGAATCCCTCGAATCGAAGGGAAATCCATCCGTTCAACCACCAGAGTTTGATGATGCTTCCTTAGAGGCATCCGTGCCGCCAAATTGTGTTAAGCTCATCAAACGCATGCAGGGCGAGATTTACAAACTTTCCGAGGAAAGGGAGACTATGAGGTTTGAGATGATGAGCGCACAAGCAATGATTAACATGCTCGAGTCGCGCATTGAAATTTTGAGCAAAGAAAATGAGGAACTGAAGAGCATGATTAACAACAATCCTTAGGGGATTAGCGCAGTGGCCATGTAATCTTGCTTATTGCAACAGAGTGATGGTGCTCTCAGTCTACCTGATCTCTTGTTATCTTTAAAGAGTTAAGAACTGTAGTCTTCTTCATTTGGCTGAACTTTCTGAGTGGTATTTAAACTTTGCCATTTGTGTAGCCTTGTAGCTTATGACTTGTTACCCTTGTTCATCAATGGCTATGTTAGTCTCTCCTTCCTTTAAGGCTTGTCCTTTCTTGTGAATGAATAGCAACTCAATTAGTTAGGCCATTCTGAACAGATTTTTCTCATAGAGGGGTTTGGCAAGTTGGGAATGGATCCTGTGCATAGATTTCACAAGATGAGT

>AdNAC71

ATGAGTACACAAAAGTATACACAACAAGCCAAGAGTGGCTTCTTCAATGTTGCCGGTGCCAATAGCATATTTAACCAGGATTTTTCTGTTATGAAAATAAGTAGTGAGGTTTGGTTTGGTAAAAAAAAAAAAAAATATTGAAATGAAAAAAGATGAGTGCTGCAAAGACATTCTCTTTTTACATAAAAATATATCTTTGGTATAGAAAACTGTTTTTGAAATAAATTAAAATAATTCATTATTAACATCTTTATATACATAGATCTATTTTTTTGAACTTAAATTGTCTAAATGATAAGCCTTCAAACAAATGCATATCGAATACTGCATGTAGATTGCCCAAACAAATGTGTTGGCGCAACAAATGCATAAGAATTTAAACTATATCACTTCTTATATAAATTTTTTTAAATAATTTGTTACTTACATGTTTGCTGAAGCAAAAAATAAATAAATAAATAAACAAACGTTTTTTGTTTATTTATTTCCAGGTAAGTCACATCTGAAAAGTGGGGATTTGGAGTGGTACTTCTTCTGTGCAAGAGGAAAAAATTATGGAATTGGGTCGAAGACGAACCGTGCTATAAAGAATGGGTACTGGAAAGCTACTGGTATGGACAAAGCTATTGTTCAGCATGACAAGCAAACAGTGGGGATGATGAAAACCCTTGTTTTCCACACTGGGAAACCGCCTCATGGGACCCGAACTGATTGGGTTATGCATGAGTATAGGCTTCAAGATAAAGACCTTACTGATAAAGGAATTGCTCAGGTGATCACCAATCAATTGTTTCTTAAGAAGATATTTGCAGCTTGTATTTGTCTAATTTCGCTGTTTGTATTAACAACATTCTTGACATTGTTTGTTTACTTATACATCTAGTGACCATTGTGAATGTCTTTTAATTAGTTCTCCTGATTTATTTTCATGGCAGGACTCTTATGTTATCTGTAAGGTGTTCCGAAAGGAGGGTCATGGCCCACGGAGCGGTGCACAATATAGGAAACCATTTAATGAAGAAGATTGGGATGATGATGATGACGATGATGATGATCATCACGTTGTTGAGGAGGGGGGAACCCCTTCAACTGCTTTGGTTGCGCCGGTTTCCATTCAGTCTATGACATTGGATGGCTCTTCTTACATGAAGGCAACCTCTGTTTCATGTGAGTCGGGACCTGTGGCTACTTCTCCTGTCCCATCAACTCCTTCTTCAGATGCAAGCATTCACACGGTTAATAATTCAACAGTGACTGATCTCTCCAAAGACGAAAAAACAGTACCTAAGGTATGTAATCTGGTCTTCTTTCTCATGTGCTTGCTTTTACCTTCTTTTTAGGAAATGCTTTTAATTTTAGTTTTATTATTTTGAACTTCTTCAAACCGCAAACACCGCCCCGGGGGACTTTTAGTGACTTATTTTGGATATTATTCCAATCTTCTTTAGTCCTCCATTGCTGGTATAATCCTCTCTAAATTCGATTGTGTTTTTTAGCTCTTCCTATTATAAGTATATTTGAGTTTGCAGTATAAATTAAGTTTGATGGTAATGAGATATTCAATTGAAGGGCTTGTGATCTCATTAAATTTGTCTTATGATATGTTTGCACAATCTTTTTATTCATATTGAAATATGTTGTGAATCTTGAGGACTAACATAAGTTTGATGTTTGTAATCAGGAAAATATTGCTGCCGGTGACCTCTTGAGTAAGTTTTTTGAGGGTTTGGAAGACCTCGAGTCTGAATACACTCCAAATGGAATGGGCTTGGATGGCTTCGCCCCAAATGGAATAAACTATGATGACTTGGGGCACTTGGATTTGATTGATTGCAATTTCCTCTAGTTCTACTTCTAGCCGTCTCACAATCTAAAAGTTGTAGATTTTTGTTTAGATTTAACAGTTGCTTTTTCATTTTGTAAATGTCTAATTTGTTGTTTATATTACATGTTTTTT

>AiNAC60

ATGGCTTCTTCACTCCCTCAAAGGTGAAAGTGTTTCTCGCCTTCCACACATTCCAGCACAGTATCGCTACTAAAAGGGTCTCTCTGCTTTCATTCTGTCTTCGTCCAAAGTCTGTCTTCGTTTCCCACCACCATTCGTGAAACTGTTCCGTTGCTGTGGAAGGGAGCTGCTGATCAGGTGGCTATTGGAAAAAAAGGTAAACTCCTCGATACCCAGACAGAAGCCACATCATATTTTATTTATTTATTATATTATTTTACAAAGCAAAACAAATACATCCATCATTGATGAAGTTGAACAAGTCAATTTTAAAATTAATAGACTTATTTATCTTTTATTCTTTTATTTAGAAGTTCTGAATTTGAAATAGATTTTTATTTTTTTTAATTATTGCTATTTTTTATAGTATTAAAAATATTAAAATTAGTTGATGGAAAATTGAAATTGGTTACACAGGTATACTTATACTAACTAGTCTTAAATATAAGAAACATTTAAATTTAATAAGAGATATTCTAATCAATTTTTTTTCTAAATTAAAAATTAAAAACTCAACACAAATAAAAGTATTTAACTGTTTACTCTAATCTGTGATACTTAGCGCAAGATCCTTTCCTTTTTACACAACTAGATACTTTAAAGTTCAAACTACAACATAAAAACCAACAACCCACTTCTTCTCAGTTTTCTTATTGGAGCCAACACCAACGGGTAATCCTATTTTCCATTCTTCTTCTGTCCTTTCCCGTTTTTTTCTTTTTTTTTTTTCTTTTACATATATCAATTGTGATTCTCCTCTTTTATCAATTTTGTTTCGCACTATCTCCATCATTATTACCCTTCACAATTCATCTTGTTATTAATCTTGACTACCCTCCCTCCTTTGTGAGGCACAGCAGACTCTGTTCTTCTCCCATAGTCTTAGGATTTATGATTGTTCTCTCGTCTAAAATCAGGATTTAGAATTTAAAAATTAAAATTTGACCTTTTTTTATATTGACAAATTATAGATTATATGGAGAAAGGAAAATTAAGTCCAGGATTTCATTTCAATCCCACTGATGTAGAGCTTCTAAAGTATTTTCTGAAGAGGAAAGTGACCGGCAAAAAGCTACCCAATGTAATTGCTGAGATTAACGTTTATCAGTATTGTCCCTGGGACCTCCGAGGTTAGAACTAAGCGCATCTCTTTGTCTTTGGCTGTGTTTGGTTTAGCATTGGTAAAGAGAAAAGTGTCTTTTAAATTTCTTTAACGCTTTTATTTTGTGTTTGATTATTATTTCTTCTCTTTTGAATGCAGATGTGATTTTACATTTTAAACCCATTTTTATGTGAAAAAAAATTATTTTCAAAAACACTTTATTTCAACTTTAATCTTCTGAGTGCGTTTTTTTTGTTTAAGATGAATTGGGCCCAACTCTTGGTGAACTTCTATAATTTTATAGAGTTCGCCCAATCCTCTAGCGAATTTTATCCAAATTCTCTCTAAAAATTTGACCGAGAACAAAATTAAAATTTCTAAGTCATTATTATTGTTTCCAATAATATAGAAGAATAGACAAAAGTACACACAACAAGCCAAAAATGACTTTTCAATGTCGCCGGTGCTAATGGCATAAGCCATGTTTAACCAGGATTTTTCTATTATGAAAATAAGTAGTGAGGCTTGGTTTGGTAAAACTTTTACTTTTTGAAAGTAATTTATGAAAATTGTCGTTTAAAAGACAGCTTTTAGCTGCAGGCGTTTGGTAAAATCAAATTAAAAGTAACTTTTAATAAGCACAAGCACCACAATTATGTGTGTGTTTAGATTAGGGTTTATAAACGAGAGTTTGCATAAAATTGATTTTGCAAATTTGATTTTGATGAAAAGTAAGTTTGTGTTAAAGTAATTTATGTTTGTCAATTTTTATATCAAAATGGATTATCATAAAATAAATGTTGTTTGGATTACACACTTAAAATCACTTTTAGATAAAAAATTACTAAAATAAACTATCAACTTAAATAATTTTTGTATATTATCTTATCATTTTAATTTAGATATTTGAATAGATCTTATTAATTAATTTTATAATAAAATTANNNNNNNNNNNNNNNNNNNNNNNNNNNNNNNNNNNNNNNNNNNNNNNNNNNNNNNNNNNNNNNNNNNNNNNNNNNNNNNNNNNNNNNNNNNNNNNNNNNNNNNNNNNNNNNNNNNNNNNNNNNNNNNNNNNNNNNNNNNNNNNNNNNNNNNNNNNNNNNNNNNNNNNNNNNNNNNNNNNNNNNNNNNNNNNNNNNNNNNNNNNNNNNNNNNNNNNNNNNNNNNNNNNNNNNNNNNNNNNNNNNNNNNNNNNNNNNNNNNNNNNNNNNNNNNNNNNNNNNNNNNNNNNNNNNNNNNNNNNNNNNNNNNNNNNNNNNNNNNNNNNNNNNNNNNNNNNNNNNNNNNNNNNNNNNNNNNNNNNNNNNNNNNNNNNNNNNNNNNNNNNNNNNNNNNNNNNNNNNNNNNNNNNNNNNNNNNNNNNNNNNNNNNNNNNNNNNNNNNNNNNNNNNNNNNNNNNNNNNNNNNNNNNNNNNNNNNNNNNNNNNNNNNNNNNNNNNNNNNNNNNNNNNNNNNNNNNNNNNNNNNNNNNNNNNNNNNNNNNNNNNNNNNNNNNNNNNNNNNNNNNNNNNNNNNNNNNNNNNNNNNNNNNNNNNNNNNNNNNNNNNNNNNNNNNNNNNNNNNNNNNNNNNNNNNNNNNNNNNNNNNNNNNNNNNNNNNNNNNNNNNNNNNNNNNNNNNNNNNNNNNNNNNNNNNNNNNNNNNNNNNNNNNNNNNNNNNNNNNNNNNNNNNNNNNNNNNNNNNNNNNNNNNNNNNNNNNNNNNNNNNNNNNNNNNNNNNNNNNNNNNNNNNNNNNNNNNNNNNNNNNNNNNNNNNNNNNNNNNNNNNNNNNNNNNNNNNNNNNNNNNNNNNNNNNNNNNNNNNNNNNNNNNNNNNNNNNNNNNNNNNNNNNNNNNNNNNNNNNNNNNNNNNNNNNNNNNNNNNNNNNNNNNNNNNNNNNNNNNNNNNNNNNNNNNNNNNNNNNNNNNNNNNNNNNNNNNNNNNNNNNNNNNNNNNNNNNNNNNNNNNNNNNNNNNNNNNNNNNNNNNNNNNNNNNNNNNNNGTTTATATAAATAGAAATAATAAGAATTCTATAAATAATACAATAATATGCATAAAGGATAAAGTTGGTAAGAAGTAAATAAAGGTTGAGTGATAATGGCTAAAAGCCCGTTGATGAAAAGCAGAAGCTCAAAATTGTTGCTTCTTGTAAACGTGATTTTGAATGCAAAATCACTTATACGTTCATGGTAAAAAATTTGCCAAACTAAAAATTGAAGCTAGAAGCTTAAACGGGATTCTTCTCTTCTAACGCGTTTGCCAAACACACCCTATGTTTGGTAAAACAGTTTTTAAAACTTAAAAAAATTATAATAAATATATTTATAACGAAAAATAATTTATTTTTTCAAATTCTTTAAAATTTTATATAATATTTTAAAATAAAAAATTCATAATAAACATAAATATAATAAATAAACTCTTTTATTTTTTAACTTTAAAATTTTATATAAGTATCATAAAAATTTATTTTATCCTAAACATCATTAAAAATACTAAATTACCTAATTCAAATTTTAGACTAAGTCATCCTCTTCACATTTCAAAGAACAGATAAATTTGTGAAGAGTAGATTGAGAAATAAAAAATATATAGAAAAATGTAACACTAATTCTCTAATACTCCATAAACTTCCATTGTATACGTTGTACAAATATTTCATTGACTCCTCATACTTTTCCTAGCGGAAAATATGTGTAGAAAAATAAATAAAATTTGATAAGTACAAGCTAGCTTTGAAAGCTCCCGCTTAGGTGTTTTCAAAAGCATCCCTAACTTTTAAAAGCCGCAAGCACAAGCACAAGCACTTGGACTTTTTAATTTACCAAAATGAGGTGTTTGTGCTTTTTAAAAACACAAGCACCTCTTGAAAAAATTTTACCAAATTCAGCCTGAATCTGGTGTAGGCGAATTGTATATAAATTATAGAGTTTTCAGAGGTGCAAAATAATTATATTTTTAGGAATGGTTTTAGGACAATAATTATTTTTTAAAATTCATTTNNNNNNNNNNNNNNNNNNNNNNNNNNNNNNNNNNNNNNNNNNNNNNNNNNNNNNNNNNNNNNNNNNNNNNNNNNNNNNNNNNNNNNNNNNNNNNNNNNNNNNNNNNNNNNNNNNNNNNNNNNNNNNNNNNNNNNNNNNNNNNNNNNNNNNNNNNNNNNNNNNNNNNNNNNNNNNNNNNNNNNNNNNNNNTTACAAATTTTAATTCAAACACACACAAAATTGCAAAGAAAAAAATATTGAAATGAAAGAAGATGTGAGTGCTGCAAAGACATTCTCTTTTTACATAAAAATATATCTTTGGTATAGAAAACTGTTTTTGAAATAAATTAAAATAATTCATTATTAACATCTTTATATACATAGATCTATTTTTTTGAATTTAAATTGTCTAAATCATAAGCCTTCAAACAAATGCATATCGATTACTGCATGTAGATCGCCCAAACAAATGTGTTGAAATGCATAAGAATTTAAATTATATCACTTCTTATATAAATTTTTTTAATTAATTTGTAACTTACATGTTTGCTGAAACAAAAAATAAATAAATAAATAAATAAAATAAATAAACGGTTTTTGTTTATTTATTTCCAGGTAAGTCACATCTGAAAAGTGGGGATTTGGAGTGGTACTTCTTCTGTGCAAGAGGAAAAAAATATGGAATTGGGTCGAAGACGAACCGTGCTATAAAGAATGGGTACTGGAAAGCTACTGGTATGGACAAAGCTATTGTTCAGCATGATAAGCAAACTGTGGGGATGATGAAAATCCTTGTTTTCCACACTGGTAAAGCGCCTCATGGGACCCCAACCGATTGGGTTATGCATGAGTATAGGCTTCAAGATAAAGACCTTACTGACAAAGGAATTGCTCAGGTGATCACCAATCAATTGTTTCTTAAGAAGATATTTGCAGCTTGTATTTGTCTAATTTTGCTGTTTGTCTTAACAACATTCTTAACATTGTTTGTTTACTTATACATCTAGTGACCATTGTGAATGTATTTTAATTAGTTCTCCTGATTTATTTTCATGGCAGGACTCCTATGTTATCTGTAAGGTGTTCCGAAAGGAGGGTCATGGCCCACGGACACGGAGCGGTGCACAATATAGGAAACCATTTAATGAAGAAGATTGGGATGATGATGATCATCACGTTGTTGAGGAGGGGGGAACCCCTTCAACTGCTTTGGTTGCGCCGGTTTCCATTCAGTCTACGACATTCGATGGCTCTTCTTACATGAAGCCAACTTCTGTTTCATGTGAGTCGGGACCTGTGGCTACTTCTCCTGTCCCATCAACTCCTTCTTCAGATGCAAGCATTCACACGGTTAATAATGTTAATAATTCAACAGTGACTGATCTATCCAAAGACGAAAAAACAGTACCTGAGGTATGTAATCTGGTCTTCTTTCTCGTGTGGTTGCTTTTACCTTCTTTTAGGAAATACTTTTTAAATTTTAGTGACTTATTTTGGATATTATTTCCAATCTTCTTTAGTCCTCCATTGCTGGTATAATCCCCTCAAAATTCGATTGTTTTTAAGCTCTTCCTATTAATAAGTATATTTGAGTTTGCAGTATAAATTAAGTTTGATGGTAGGTGAAAAATGAAAGATTCAATTGAAGGGCTTGTGATCTCATCAAATTTGTCTTATAATATGTTTGCACAATTTTTTTATTCATATTGAAGTATGTTGTGAATCTTGAGGACTAACATAAGTTTGATGTTTGTGATCAGGAAAATATTGCTGCCGGTGACCTCTTGAGTAAGTTTTTTGAGGGTTTGGAAGACCTTGAGTCTGAATACACTCCAAATGGAGTGGGCTTGGATGACTTTTCCCCACATGGAATAAACTATGATGACTTGAGGCACTTGGACTTGATTGATCTCAATTTCCTATAGTTCTACTTCTAGCAGCCTTACAATCTAAAAGTTGTAGATTTTTGTTTAGATTTAAAAGTTATTTTTTCATTTTGTACATATCTCATTTTGTAAAAGTCTAATTTGTTGTTTATATTACATATTTTTTGGTTCAG

>AiNAC62

ATTGGAATGAAGAAGACTCTTGTATTCTACACTGGCCGTGCTCCCAAAGGGAAGAGGACCAATTGGGTGATGCATGAGTACAGGCCTACCCTGCAGGAGCTTGATGGTACCAATCCTGGACAGGTATAGTCTTATCTTTGGTTTCTGTTGTTCTGTGTTTAGCTTTCGATCCGTCTTTATTATTATAGGGATGGCTGACATTGAATTGTCATTAACTCAAATCAATATGACTTCACCACTAAATTCAAGTGTATGAAATGTGTGGGTCTTAAATTATATGATTGCCATATTTGATGAATATTGTGTTAAGTTTGTGCTTCTTCCAATTTGGTTCATAAAAATGATCATTTTTCATATTTGTGTTGATTTAATAAAGGAAATATATTCAATTATTATTTCATCATTTATTTGTCTCTTCACTTACTTGGTGGTTGCTAGCTTGTTATTTCTTGAGTTCTTGTAATAGCTTCTCATAATAATTCTGCACCTGATTATTGTGGTTCTAAATTTTCTTGGTGATCTTTTATTTCTCTAGTTCAATTTCTAAGGTGCAACCTGCAATTGTTCACAGCCTACAGGTGTATGTAGGAATTAGCATTAAGCTACTGCAAATTATGACTAGCTCAGATGTTATATGAACTCATGAAGATAAGATATATTGCTAACTTTTCAATTTGGTTGATTTGACGTTTACCAGAATCCATATGTCCTTTGTCGATTATTTAAGAAACAAGATGAGAGTCTTGAAGGTTCAAACGGTGAAGAAATGGAGCGTACTACTTCAACTAATTTAACTGCAAATTACTCTCCAGAAGAAATACAATCAGATCCAGCTGTTAAATCGGTTTCTTCTTCACAGGCTACAGAAGATGACAAGAAACTAGCAGTTATCCCTTTGACCCCTGAAGAAGCAATTTCCAATGTTATAACCCCGGTCGGTTGCCAAAACGATGGATGTGATGCTTATGATGCACAAAATCAAATCGCAGCAGGAGATCCATCTAAGGAGGTTTGATGAGCAAACTAATTTTTTGTCCCCCATTTAGTTGATTTGCAGACTTTGTGTATGTTCAGTTATTAACCATAATTTGTTGACTAATATAACTTTAGGAGGACTTACAAGTGAACATGGACATATTTTATGACCCGAGTGAGCTATTTGACGATAAATTATTCTCCCCACTCCACAAGCATATTCCAGAAGAACTTTTTCATCAATCAAACAATGAAGCCAATGGACATTTTGGGCTGCAACATCAGTGTGGAACAAATGAGATCAGTATTTCTGACTTCTTTGACTCTGTTATTAATTGGGATGAGATCTCCGGTGACAATTCCAGCGGCCAAACGCCAAACTCTGCTTGGTTTGATGTACAGCACAATGAATCATGGGGAAACTCAAATGTGGATATGGTCCATGCCAGGGTGAGTGACAGTGTTAACCCTGTTTATCTTTTGGTCAGTTGAGGTTTTGCCACCAAACCAACATGTTACCTTTGAACTTGGAAGCACATACTGGTGCTTAAGCCTTTTGTTAATTAATTTTTTTATTTCCCTCTAAATTGACGCAAATTATATGTGATATTATATTTGATTTTCTTTGGCATGGTTTGCTTGTGTCGCTATATAATTTTAGCTGCTGTTATTTACTGCAGCCCCTACAAGTAGGGGGTGCAGATTATCCAGGGGATGCAACCGAGGGAAAGCTCCCTTTGTTGAAAACTAGAGAATTCAATCCCAACACCTCTTATGACAATGCGCTCAGCAACAACATGGGATTATTTCATAACCATTCCCAGATGGCTTTTTCATCTGATGTTAATATGCTCCAAGGTTACCATGCAACCAACAATTATGAGCAACCGACAAACTTCAATATGGCTATGGCTAATAGTGACAACACTGGAATTAGGATAAGGTCTCGGCCACCAGGTTATGAAGGGCCAAACGTAAACTCCAATATGCAACCACAAGGTACTGCACCTAGGAGAATACGGTTGGCACGATCTCTTGCACCTCAACACATGTCCAATGAGGCGGCAAAAGATTCGAGTTACGAGTCAAAAGATCAAAATTCACAACTAACCACTGCCAGGGTAAGCAGTCTTTTTGAATAACAACCTGTGTATCCAACTTGAGAGATGTCACTGCATGTGGCGTAAGATTTTGTTGGTTCTTTTGCAGGAGATGGAAACTTCCAAAGACCTTGCTGCTGGTGAGAGTGTTACTGTTACTAGTGATGTGGAGGAACAGGAGACATCACCAGTTGAAAATAAGGAATTCGAAGACTTCAACACAGTCCAGCAGAGCACATCATCAGCTTCCTCCAATCTTTCCACGTGCTCTTCTGATTCTGAAGTTTCTTATGAGGCAGAAAAAGAATCTGGTTGGACATCAGAAGACCATAGTCCAAAACCAGCTGCCGCGGGGGTAAGAAGTCTGTTTGATTTACTTGTGTATATACTCTTAGATGTTGCTGCATATTCATCAAGATTTTTGCTGGTTCTTCTTTTGCAGGCCAGTAAAGCTTCCGAAGACCAAGTTCCCAGCGAGTGCGTCAATGATATCACTGATGATGTGGATGAACCCAGGATACCAAACGCTTATACTCTAGAGGTCTCAAAGGAGGAATCCTTCTCGGACTCTCAGTCGAAAGACTCTCTATTGCGTAGAAAGGTGTGTTACCCATCGAAGTCTTCCTCAAATCTAGCCAAGTGGTATTCGGTTATTGCAGTCTCAGCCACTTTGGTGGTGTTACTAGCATTCCTTGTTAATACATGGGGTTATGGATATTACCTTAAAGTTTAACTGCATAGTAGGATATGTATCATTGCATAGAGTATATTTTGCCTCCATTGCGTTTTTGGCCTTAGTTAAGGAGGCATATATGTAAGGGTTGACTTTGTAGCTTTCTTTGAATATAATCTGTATGTGCATATGTTATATATAGCAGAATATGCCTAATCTGCTACTAGCTTGTACTATATGTGGAGTTGTAAACTCATGTTTATAAGTTAGATTCAGAATTTCAGGGTTTCCAATTTTTTGGAATTTAATTTTGATTGCCCGTGCGTGACCACCATTGGAGAATATGCCTGCCTAGCCAAGAGTGTATTTTTAGTAAAGAAAGGTTTAGGCAGATTCGGCTGT

>AdNAC51

CTTATTAACCGTTTTTGTCTTCTAATAATATCCCTTACTAGCTTCATCATCTTCCTTAGAGAGAAAGAAAAAACACACACACACAAAACACAAAAAGAATAAATAAGAGAACATTATTATTGTTATAATTATTGTTATTATAACTTTTATATATGGGGGTTAATGAAGATTTGATGATGAAGGATGATTCATATGCATCATCAGTGATGGAGGAAGAGGATGATGTTCCACTTCCAGGGTTTAGATTCCACCCAACAGATGAAGAACTTGTGAGTTTCTATCTAAAGAGGAAGCTTGACAAGAAACCAATCAGCATCGAACTCATCAAACAGATTGATATCTACAAGTATGATCCTTGGGATCTTCCAAGTATGTATTTATTCTTTTCTTTTTTAATTAGCTAGTATTCCTCTTTAAATTCAAGAATATCATTTTCTCCCCTTAAGATCTAATTATTATTACGAGTAACTAATAGCAGGGTTTCATCTAAACACAATTACTGAATCAGCAATATTAATAAAACAATAATGTAAAATTATTTTATTTAATCTGATTTTTATTACTCAGTTAATTATAATATAATATATTATTTCTTAGCCTACCTTGGCTTAATTAACTAATTAGTAGCTAGTGATAAAATTATTAAATGAAAGCACTAAATGCATAAACCCAGTTTGATTATTAAGATATAATTAGTAGAATTATGAGGTATAGCCACAATATTTTACAGGAAATGAATTGTAAATAGAGTATGATTTATCAACCTTTTGATTTAGGAATAAAGAGAATCGCACTTAGCATGCATGGTGAATATATCATATAAATTTCTTTCCCTCTCTAATGATTATTAATCCATTATTATATATAAACTAATAGACATAGAATAAATATAGAAAAAAAACAAACATTTTCCTTTCCATTGTTAGTAACTACTTTACATATGTATGTTGCTAACACTATTGAATTTGGTTAATTAGAAGCGAGTGGAAGTGGAGGAGAGAAGGAAGGTTACTTCTTTTGCAAGAGAGGGAGGAAGTATAGGAACAGCATAAGGCCTAACAGAGTCACCAGTTCCGGCTTCTGGAAAGCAACCGGGATAGACAAGCCGGTGTACTCCCATGGCGGCGAAGGAAGCGACTGCATTGGACTCAAGAAGACGCTTGTCTACTACCGCGGCAGCGCCGGAAAAGGTACCAAGACTGATTGGATGATGCACGAGTTTCGCCTCCCTTCTGCCACCACCGAAAACAAAACAAGCCTACTTGCCAACAACAAGAATAATAATAATATCAACAATGCCGATGTTGCCCAAGAAGCTGTAAGTAACTTAATTAGATAAATGGCTTTAATTTATTATAGTTGTGATTCAATCTGTGATTGCAATTCGCAGGCTCAATTATTGTTATGATCATTATGATTATTACATTAAACTCCATGTGAATATTTCCGTCGCAATTATATGATTAGGTATTCAGTAATGGATAGTCATGTACACTTATGTTGATATATAAATACTATATGTAATTTAGATTAAGAAAAACATTTTCCGTTTTTTGATACGAAAAAAAAAAAAACATTTTCCTTCCATCATAGGTTTAATTTTATATATACTATTATTCTTTTATACGCATGCTCTCACTTAACCTTATTAGCAATATACTCGTAGTCTTCTATGTATATATATAACTTCTTTCATAAAAAGAAAACCTTCGTAATATATTTCCCTCTTACCATGTAATATAGCAAACCCAAAGAATACAATAATAATAATAATATTTCAATTCTTGACAACTTACTTTCATAGTTAATAGTAGTATTTGATTTCTGAGTTAACTAATAATAAGCTTGTGATGGATGCAGGAAATCTGGACATTGTGTAGAATATTCAAGCGAAATGTGTCACAAAGGAAGCACACAGCAGACTTGAGATCACATTTAGTAACAGCTAATAGTAACAAGCACAAAACCACTAGAACCCATGTTGTTCAATCCAATAATAACAATATTAATCAACATCAAGAATCTTACATCAACTTTGGTGCAACAATCATTGGCCATCACCATTACCATCATCAAAATGAACAGAAGCCAGTGACTAACTACACAGCATGCAATAACAACACTGATCAAATCCAAAGGAACAATAGCAATCATCATCATCATCATCAGTTGAACTATCACCCTTCTTCAGCGGTGGCTACTACTGTGCCACAGCAACAACAACAACAATATCATCATCATCATCAGCTAATGACGGCTCCAGCTTCTAACATGTGGATTAATCCTTCTGCGATGAACGATTTGTTTGCATTTGATGATAACTGGGATGAGCTTGGATCCGTTCTCAAATTCGCT

>AiNAC21

ATGAAAGCTACTGAAAGTTGGGGTTTCTATTTCTTGTATTATTTTTACCCATTTGATAATATGCATTTATGGTAGTGTGTGCCATAAGAATTAGTTCTTACAATTTTTGAGTTTCACATTGTTTTTCTTGCTTTATTATCTTTGTCAGTTTTGACTTTTGAGTTCACTTAATAACGTAATGAGTGGCAATTTTGTGTATGTGTATGTGTGAGCTTTGTGTTCGTATGTTTGTTCCAAGTTCTGAAATTTATTAGGTTGCTTAAAATTGGTACAATGAACATATGCATTTCTTGATAAGTATCAGTTCCTTTTTTGGTGGTGAGGTGATCATAGATTGCATTGTTTCTTTCTCTATGTTACCAAGCCATCTTTGTAGAAAAATAAGAGGAGTCTGGTGATTGGGACTTTTATGACTCATTGTTTTATCTATTTTGCTATTCCTTTGCCATAGAGGACTCATGATCATTTTGATTTATTGTTTCGTTTTTCTTCGGGATGTTAGAATCTATACATGGTCATAGACTTCTATAACTTTGTGGCTCTTATATTTTTATGCAGAGCATCATGGTTGGTGGACATTAGATTTCATGATAATTGTTTTTTTTTTCTTTCTATTTTTATATTTTGGCCTTTCAGAAATATAAATTTTATATTTTCATGTATTTAAGTAACATCATTTTGTATGCTAGTTTGTGATCTATTGAGTGGTCTTTGTTACATGGGAATTAATGGTTTCAACTTCAAACTAAACATGATCTGCTTGGAAGTATAATTTCTTTTGTAGAAAATTGCATATTACCAGTTTAAGCGTTGGTTCAAAGGACCGGTATTGCTTGATATTCATGTAAGTGTGAAGATAACTTATCAACTGCATTAAGGGTAATGCCTTCACGTCAAGGTCAAAATTAATGGAATTCCATTGCTTCTCTAAATTTTGCATATGCCAATATGAGTTGAACCTGTTAAATTGGCTTAATCCACCATGCTTTTGGCACAAAAGTTGGAACATGTTTCAATCACCACTTTGGTACTTCTAGTATTTTATTTTCTTCTTCATGATAAAGTTATATGCCATTCTTCAGAAATATGTTTAGAAAGTTTATTACCAATAAAGTTAAAAGATATTGGTTTGATAGTTCAAACTTAATCCTTTCTATTTCACAAATAATGGCACTTAAAAATTTATAGATGATTTAACATAAACTTCCTGTTGTTAGGTAACTCAAGAGTGGCCTGGCTTACCAAAAGGGGTTAAATTTGATCCGTCAGATCAAGAGATAATCTGGCACTTGCTTGTAAAAGCTGGTGTAGGAAATTTAAAACCTCATCCTTTCATTGATGAGTTTATTACTACCCTTGAAGTGGATGATGGAATTTGTTATACTCATCCTCAACATTTACCTGGTTTGTAGCACATAAGTTTTATTCATAGTTGAATTCTTATTTGATTTTCAATCAATATGATATGTTGCGATTAAAATTTAGGTGTCAAGCAAGACGGAAGGGCCTCACATTTCTTCCACAGAGCAATAAAGGCTTATAATACCGGCACTCGAAAGCGTCGAAAAGTACATGGTCAGGATGACGTTCGTTGGCACAAGACTGGAAGGACTAAACTGATCACCCTGAACGGGGTTCAAAAGGGTTGCAAAAAAATCATGGTTTTATATACAAATGCGGTGAGAGGAGGAAAGTCAGAGAAAACTAATTGGGTTATGCACCAATATCACCTTGGGACAGAAGAAGATGAAAAGGAAGGAGAATATGTTATTTCTAAAGTTTTTTATAAAGAAGACCAGGATATACCTGAAGCCGCAGAAAGTAAGAATGCAACAGTTGCGAAAGTAGATCCAGTCACTCCCAAATCCACGACTCCTGAGCCTCCTCGTAATGAAAGGCAAGATTCAGATCTAGGCCTGGATCTAGACCTAGGGCAAGAAGCACTTGCTTTTCCTGAGGTAAAACCTTCAATGTTTTCCTGCTATTGAATATCAGAATCTGTTTTCTGTTATCATTTTGCTGTGATCATTTTCATATTGCGTGTTTTTTCTTTTGTTATTGGAGCGAGATCTGTCAGAAAATGGATGCAACTTTATAATATTCTTTTGTTTCTTTGGGGAAAAAAAAAGGGGGGGGGGGATGATGGATTTGTTGGAAGTACTTCTTTTCATTTATAAAGTTCGAAACCTATTTTCTCAAAGTAAATATCGATGATTCTACCTGCTAAAAGAATCACCATTAGTTCTGCATTAGATACTTCTTGTCTTAGCTGTTTTTCTGTCTCAAGTTATTATTGCTTGCCATTGAAGTCTTTTATGATAGTTGTACAATTTATATGAAAGTAGAAATAAAGCGAAAGCCTAAAATTGATTTGTTGTATTCCACTTTTAAGTAGTCTTTGTCCCTTGATTCATCTTGTGATGTTAGTTTGATTAAGAAAGGAAGCTTTTTGCTTAACTCTACATCTCATTGAACTCTCGTGCTTGAAATTCTGAATACTGAACAGTTATTTTGCAATCATATCTTGCCTCCTTTTTTGCCTTTTCCCAGAAACTGCCCTAAGCATATAATCTTGACATATAATTTGATGATATAGTTCTGTGGAATCTGAAAATTGCACTAACAGAAGTTGATTATTCCCGTTCTATGTTTCTTTTTTTTTTTAAAAAAAAAAATTATACCCTTAGATGGATTGCTTAGATGAAATTCAAGCTGACTGTGAAGAATCTGCGAAAGCTAATCCACCAGTACTGGAGACACAAGAAAATGAAGGGATGGACAACAAGGAAACTAATGCTTATGAAGGACAACTGTGGTGGGATAGTGATTCACAGAATCTATTAGATTCACAACAACTCGTTGAAGCATTAACTCTCTGCGAAGATATATTTCACAGCCAATCTTCCAACAAAGACGATGAAAATGATAAGAACCAAACCGGTCTATCTGTGTATGCTCATCTAGGACCAGAGCATCTGAAGAAGGATATTGAGGAGTGTCAAAAGCTTGCTCCTGCAGGACCAGAGCATCAGAAGGATATTGAAGACGGCCAAAATCTTGACATCGACCTTGCAAATATAGAGCGGGATACTCCTCCTGAGCATCGACTAAGTCAGCTGGTATCTCCATGTTCTTGATGATTTTGCATTACAATGAATGAATTCATTTAAATCACCATTACTAATTACTAGAATGCTTCAAATTAAGTTTTCTTTCATTTGGTTTTGAAAGTAGTTATGAATTGTTTTTCTTTTGGAAAAAAAAGTGTCACTTGTGTAATAGCTAGCTTAATTGAAAATTGCGCACATAATCAGTAGTTACTTTACATATATGTTAGGAAGTTATCTCAGGGGGTAAAAGTTTTGTACTAATGGAGAAAAAATATTTGGACACCAATCATCATAAATCCTGTTTATTAATGATGCATTTTCATGTTTCTGTAGGAATTTGGTTCGCAGGACAGCTATACTTACTGGGGTTTTCAAGGCGTAAACTAATATCTTCCTTGATCTCTCGGCATTGAACCAATTTTTTTGCAACTACATTTTGTTGATGTGTTATTTGTCTTTTGCCTTTGTGTTATGCTAAACTATGGGGCAAACTTAACTGTTACTAGTAAGTGGTTACTACTAACTATGCAGTAATGTAAGACTGAACCTGATAGATATTTTGATCAACCTTGGTGTAAATGTAATTCAAACTTGCAATTCTCGGCTGGTTGAAGTTTTGTTCTATATTCTTGCAGTTCTGGATGTGGACTCGAGAGAGAAAGATGCATTTAGATTTCTACTCTATTCAAGCAGCTATGGAATTATCAAAGTTGCTTTTAGATTTCTGATAATAGCTTAAGACTGAAAAATAGTAATGTTGGCTTTGTTCTACAGTACCAAGTAGTATCAGGATGTGAATTTTATCCAAACCCTCAGGTATCCATAGTAATTTTGGAATTTAACAGTTAGATAAATACAATTTATTGCGTTTAAGTTATACGATGGTGTGGGTTAA

>AiNAC14

TTGGCGACCAAGGATGCAAGGTTAAACTTGTAATTCTGGCCCTTACACGGTTTGCGTCTTTACGAAACTCGATTTTCTCTTTCTGACTCGATATCCGAACCAATAGGCTCGGTTTTGCTTTGGCTCACTTCTTCGACAGAACAGCAATGACGAAGCACAGGTTCGAATGCACAGTCTCTTCCCATTACCAACAAAAACCCCAGGCTACAATTTCCCGCTCACTCTACCGACTGTCGCTTTCTTTGTCTCTCTGTCGATTGAAAATGGGAAACCGATGCTGAAGCTCAATACTCTCCACAGTTCTGCTTCAGCTCAAGCTTCTTCCTCTTCCTCTCTGCAATGGCTAGGTGAGGTTTTAGAATCAGATTCAGATATGGCCACATTGCATAATGAGATACACGTTTTCTTCCTTAGTAACTTTTGTGTGTGTGTTACGCTTTCATTTTTTTTCTTAAAAAATCATTTTTGATTTTTTATTTTAACTTAATTGTGGTTTGGATACATTTTGTGGCATTTTATTTTCCTATTTTGGGGTTATTGACATGGACGTGCTTCAGTTTTTGGAAAGCAAACTGTAGTCTGTAGTGTAGGGTTTGACATGCTTGAGTTTGGTAAATAGCTGCTGCTACTTTTTTCTCTTGTTCCAATTAAAGTAAACAAAGTGTTTCTTGAAGTGGTCCTGATTGCTGAACATGATTACATGAATCCTTATTGGAATATTTCAAATGACATTATGACAGTGTTAGTCACAGTATACTATGTTCTTACACAAGAAGTTACATTTTGTGCTTTGGCTTTGTATGAGTCCTTTTTTCTACCCTGATTGAAGCTGTTGGTGAAGAAAAAGCTTTCTGAAGGGAATTTCATGGTGAAAATCTCACTATGTTGTAAATGATTAATAATGAGTTTCCTGGTATCTTGCCCTTCTAATTTACTCCTTGCCATGCATTTCTAATTTTGATTACATCTGTAATATGCTGTGTTTTTGTGGTCATGGAATCTTTAAGCTGTGTTTGTGAATTGAGGTTTAGAAGGGGAAGGGAAAGAAGTGAAAGGTTTGAAGAGTAAAAGGTAATTGAATCATAATTATATGACTCTCTTTCACTCTACAAGCCCTTCTCATTACTCTTCAAACTCTTCCTATCCCATCTTTCGTTAGAAAACCTCATATTGACATTACTGGCTTCTGGCCTCCGGATTCTGTGCAGTACAGAATTCCCTGTGACTTTGTTTCTTTTTTTCATTTGACTTCATTTCTACTTCTCTTTAACTGTATTTGACCTAGAAATTCGCCATTGTTATTCCTAATTATTTATTCCATTTTCGTATTTATCATGTAAAGAAGCTGAATTTTCACACTTTTATTTAAATCTAGTTATGCTTTTAAAGTTCTTCAGCTAACAATCTCTTCTTTATCTTCCAATGGTATGATGAACTTAGGAATGTTTGCTATTTTATTTGGCAGGAGCTGGCTTATTGACATAGGTGGATTTGCAAAGAAAGTGAAAAGCACTAATTTATCTCCAGCTGATCAAATCAAAGATTGTGGGGCATATCGTGATTGTCCAAACTGCCATTACCGTATTGATAACCGTGATGTACGTTAAAATTCAGCATAATATTCATTCTCATTTTATAGTTGTTAGGTAAATTGACACACACAACTCAACACGGAGTCAACATAGAGGCAATGGACTGGCTTTTATATAATAAAGCCTTCAATGATCATTTGCAGTTTTGTTATGATCCATTCTCTTTTCCCTATATTTTACTTTTTAACTTTGGATTTAGCTAACAAGGGCACATGTTAAAGTTACGAATTAAGAAAGTTATTATAGAAAAATGTGTAGAGTTTAGTTTTCAATGCAAATGTTGTGCATCTATTAAAATAAAACATTTAAGACTTTCTACTAATACTAATCTTAAGTTGTGCCCTTTAGTAAAACCCTTTAACTTTTGGCTAATACAATTCCTTCGTTTATTTGATGTTGTGCTTGTGTGAGTTTGCAAACTTCACCATCATACCAGGATTGAATTCTAGAAAATTTTGGCATGGGTTTGAATGATTGCAGATGTTTAGTTATGAATATTTCTTCTTCTTCTTCTATTTATTTTTTTAAATAGGTTTCTACTGAGTGGCCTGGCTTTCCTGTTGGTGTGAAGTTTGATCCTTCTGATGTAGAACTCCTAGAACATTTAGCAGGAAAATGTGGCATTGGAAATGCTCAGCTGCATATGTTTATTAATGAGTTCATTCCAACAATAGAAGAAGAAGAAGGTATTTGCTATACACATCCAGAAAATCTTCCAGGTGAGGTTTCTCTATGCTTTTGCTTCCAAAAATTTAAACATTAAACATGTGATACAGAAAAGAATCCTTTCACATTGACCTTATCAGACTTGTGTTTTGCACTATAAAGCAAAACCCAATCAATGTTCAGATCATGTTAATTGCCATGTTTGAAATACATTACATGTTGCTTTTACTGTTATATGTTGAGGGTTAGGGATCAACAGGTTTATGCTTGCCGAACTTAATGATGTTGCTTTTATGAATGATAATCATTTCATAATCCCTTATGTGTTCTGAATATTGAGTTTTTCTTGTGGACCTACATTGGCCCACTTTTCTGGAGCTATGTCTCTAATCATCATGACTGTTTTATTTCAGGTGTCAAGAAAGATGGGAGCAGTGCCCATTTCTTTCATAGAACAACCAATGCCTATACTACTGGTCAACGGAAGCGTCGAAAGATTCATCATCAATGTTTGACTGAAGAGCATGTACGGTGGCATAAGACTGGTAAGACAAAAGCTATATTGGAGGATGGAGTGCATAAGGGCTTTAAGAAGATCATGGTTCTTTATATAAGACCTAAGAAAGGGTCCAAACCTGATAAAACGAATTGGGTGATGCACCAATATCATTTAGGAACTGATGAAGAGGAGAAGAACGGTGAATATGTGGTTTCAAAGATTTTTCAGAAGCAAACTGAGAAAAATGAGGAGAATCCAGCGGTCGAAGATTCTGACCAGAATGAGAAAAATGAGAATCGATTGGCTGATGATTCCAACTGTATAGCATCCCGAACCAGTCCTAGAACTCCGAAACCAAATCCCCCAAATCCACCTAGAGCTGGAAATTTTGTTGACAATGATGATAATATTGACGAAACTGAACTTCCATTCACTCAGGTAGTAGCTGTATTTCATAATTATTAATACAACTGTTAAATCATCTTCTAGAAAAATCAAATGGAAAAGAGAAGTCAAATCTGATATATTTCCTCCCTAACTAGTTCGCACATAATCTAAGGATTAACTTGGCTTTCAACATGTATTTATGTATGACTGATGGAACAAGTTCTACGCTTTGTTTTATCTAAAACTTGTCCACATTTAGTTTATAAAATTTGAACTTTTTGAACTAGGATGTGAAATGTGTCCCACAATGTGATGTTCTGGATCAGAACAATGCTGGTGACCCTGCATGGCTGGCAGGTGAATCGCAGGCTGTGGAAAACTTCGACTTTGATGGCTTGGATGACATCTTGTTCTGCAATGAAATATTCGATTCATCATCTCTACTAGATATTTCTGGAACGGAAACCATGATAAATGGATCTGCTTCAAACGATATGCTTGGGAATGATGGTTTATCATACGGAACTTCCGTTCTTGATACCCTTGACTTGGGTACTCCCCCAGATTTTGATCTTTCAGTAAGCATCCAGTTACCCTGCTCCCTGTATCTTTGATTTTGAGTTTGTACATTTACTAACAAGCTCTACGATTCTCACTTTGGATGCAGAATCTGAATTTTTACTCTCAAGATAGTATTTTCGACTGGGTCGACAGATTATGAAGTGATTTCTGAAGTTTGAGTCCATCAGAATGCATGTTTGTTCAGCTCTTAGATGCTTCAGATTCAAATATAGTAAGAGAAACATAGGTTCTCACGTGATTTGCAAGCAGAAATAACAGTATTTGTTTGTCCCTGGTTCTTGAAATTCTGCAAATTTTGTAATCCCACACATTTGGGCATTGTTCAGGTGGGTGTATTGTTCATGTGGATTTTAGGTATTATTCACGTGGGTTGTATTTGTGTCAAATAGCTTTTGTGGTATATGTTGTTTCCATAGCCATCTAAACTTGGTTGCCTCATTCTGTTGTGGTAGATACATCTCTGGTAGCAAAGTCCAAACTTATATAAGTACTTGATATTCGACTGTTTTTATGTCAATACTAGAATAACTATCCGC

>AiNAC68

ATGCTATCCGGGTTTAGATTGTTGTTTTTTATTTTGAGAGTTGTAGTTCATATTTGGTTAAATTATATTATAGAACAATCGAAGGTGAAATCGGATGATCAAGAATGGTTTTTCTTCAATGAATTGAAACACATAAAAAACAAGTGGTGTAACAGAAAAACTAACGCCGGTTATTGGGAGATCACCGGAAAAGAGCGGATCGTCAAAGGAACAGGGACAGACAATGTGATAGGTACAAAAAGAACACTAGTTTTCTACAAGCGTCCACATAGTGTCAAAACCAATTGGGTTCTTCATGAATATCATGCACTTGATCAAAAGGTAGGTTCTTGCTAGGTAAGAGATTGTATTGGAAATGAAATAAATTCTCCAATTTTTTTTTTATCTCAGTTGGTTTTGTTTTTGTTGTAAACTGTGTAATCTTTTCACATCATTTCTTCTCTGTTTTAAGTCTTTTTTAAGTGTTGTAGGGTATGAAGAATAAGATGAAGATCGGATCCTCTTGAGTCCCAACTTCATTTTCTAGATAAACGCACCTTGAATGTTGGTTTTTGAGTAATTTTAATGGTTGATTTTTATTAATATATATTATATATTTTTTTATATTTAAAATTAACAGTTTAANNNNNNNNNNNNNNNNNNNNNNNNNAAATTATTAGAATATCAATATTTTAGGTACATCTAAAACTTTTTTGAAATTTTTTTATTAGTTTATATGATAGAGCGATGGGTTTTGTTTTTTGAGGGTGGAAGTTACGAATTTAAAAATTAATCACTTTTTTTTAAAATTAAAAGATTGATAAATAATAAAAAGTTTTTCTGGGAGTTTTAGTTTGTAATAAGTATTTTTTTAAGTTATATTTAAAATATTATTTTATTATTTTACTAATTTTTCATAGGAAAATGTATTTTTCCCTTTGACGAGGGTGTGGTACTAATTAATTTGAGTTGCATGAAAATGGGTATTTTTTTTTTATTTTGATTAATCTTTTTTCCCCTCTTCTACACATTTGACAAAGAAAAATTCAAAGGATTCATGCTGCATATATCTATTGATTTAATGACATGGTATAATAATGATTCATGCATTTCCATTTACCAAGAACAGTAATTATTTCAAAAATAGACTTTTATTTACACATTAAAATCTTTAAAAATTAAAAAAAGATATTTTGGTTTAATTTGGATAAATAATTTAATTAAGTTCTTTTGAAAAAATAGTTTAAACAATAAATTGTTATATTAAAAGTAATTTATACAACATATTTTATATTATATGAAACAATTTTTTTAATTTTATAGTACAGTTGATAGACATGGTTTAATCTTATTGATTCATTGTCTTGAGTGGTACAATTTTTAAGATCTACACAAATCAGTTTGATGCACACAAATTTTGTATGCAGAGCAACATCGTGTTGAGCCGTGTAATAAAGAATGTTGAGAAAAGGGAAAAGAAGGTTAAGAGAAAAGCAAGCAACATAATCGAAGAGGAAGTAACATGTGAACCATGCAGCGAAATGACTGGCTATGTTACCGAAGCAACTACAGAAGATGCAATAATTCCTGATGTGAGTGGAAACATATTTTTGACACTTAAAAATGTTTTTTATATACTAAAACTAGTCATTATATATTTGTGTTTAAATATANNNNNNNNNNNNNNNNNAATTTCTGTAAATATAAATACAAATTATTGTCAGTAAAATATTGGTCTAAAATAATAATATTTACTAGTTAAGCAATATTATTAGTATTTTATATTAATAGGTAAGTTTAATGACTGATTTTAATACTCCTTATTTCTCGACAAATTTTAATGTCGTGACTAAACATTACGGGATATGTTTTCAGGCATGTGTTTCATCCGAACCGCAACCACCTCAAGATATTGACTATGAAATTCTTTCGCCGGGACAACAATCTTCGGTGGCCCATTCCGGTAATGGAAGCAACAATGCTCCGTTGCTTCCATTTGAAGGTATGTGGAAGCAAGATGCCGAGATGAACACGGAGTGTTTTTGAAATTTGCTGTTTTCTAGCATCGATGCTGACCTTGATGCGGAGTTCTTGAATTCGGTGTTGGCAGGGGATGATTAA

>AdNAC25

AATCATACATGAGTCGAATACTCGGTCCCGGTTTCCGCTTCCACCCTACGGACGACGAACTAGTTCAATACTATCTCCGCCGGAAGGTCATCGGAAAACTCAACCACCACGACCACATCGGCGTCATCAATATCTACGACTATGAGCCATGGCAACTCCCCGGTTAGCTTTCTCTTAATAGCTGAATTTCGACCCTAATTTGTTTTCGGTGTCGTGTATCTTAGGGTTTCATAGTGAAATTAGAATTTTATGGTGGTGGTTGTTGTTGTTGTATGTGTTTTTTTAGAATTGTCGAAGCTGAATACTAGGGATTTGGAATGGTATTTCTTCACGGTTCTGGACAAGAAGTACGAGAAGGGGGAGAAGACAAAACGCGCCACCGTCAACGGTTACTGGAAGACCACCGGCAAGGATCGTGGAATCAAGTATGGCGATCGCCAAGTAGGCATGAAGAAGACCCTCGTTTACCATGAAGGAAGGGCCCCGACTGGCAAAAGATCAAATTGGGTCATGCACGAGTACCGGATGGTCGATGAGCAATTGGCGGAAGTCGGATATCAGCTGGTATATATATGTATATGAATTTGAACACGGCTATATTTTTTATTTTGAAATCTTTCTAAATTCGTTCGTATTTTTAATTAAATATCTGGCGTTTCGAAAGTTTGGAAAGTTTATAATTCAATATAGCTGGATACAATGTTGGAAATGGTGGTGGGCGAAGGACGCTTTTGTGCTGTGTAGAATTTTTGAGAAGAGCGGGATGGGCCCTAAGAATGGAGAGAAGTATGGTGCTCCCTTTAGAGAGGAGGACTGGGTGGAGGATGGCGACCTGCTTGAACCGATTGCTGATGAACCTGTGGTTGAGCTGTCTGTTGACCAGAGTGATGCTTTCCTTGAAACTGATGACCTTGAGAAGGTTCGTATACAATCTTCGAGTCACTGATGATAGATTTCATAATAGTCATATTTCATTAATGCAGATGTGTGCCCCTTAACATTCACGAGGATTTGCGTGGCCATATGTTTCCACATTGTTATGTTAATTGTCTTGTTTTTGTCTCAGTCAAATCATTGATTTGTATGGTCAATAGAGATTAGGCTGATTTCAAAGATTGATTTTGCAATTCGTACCCATTTCTGATTCTGTCCTGTTTCTGTATCACTGGGATGATGAATCAAATTTATTCATGCGATTTGTATCGCGAATTGTCTGATACTGTCAACCTTGGTCAATGGTAGATATCCTCTTTTTCAGCTTTACTTTCTGTTCTAAGTTGATGATTTGAAATATTGTCTTAACAGAAACTTGGTACGCATGTGGTCGATGGAAGTGCTGATTTACCACCAAACCCTCCCAACTACTTTTATGGGGAGTGTAGTCACTATCCTCAGCATCAAGAAGAATTTGTTGAAGTTCCGAAACCTTTGGAAGGCACTGAAGGCCGGAATTTCGATGTAACTGGGCCATATGCTGAGGATACCTGTTTAGAAAATCATGAAATGAACCATAATGGGAATTCTTCAGAATTCATTTATGGTGACGTTAATTCAGATGAATTCATGGATTCCATTGTTGATCCTCTGATTGGTGCTGAATTATTCCTGGAAACAGATGATCTTCTGAACCCAATCGAGGGAAATTCCTCTGGGGCAGATCCTTATACAGTTGAGGGAAATCACCCTAGGGCCGATCCCTACGCAACTGAGGGAAATCCCCCTGGGCCAGATCCCTACACAGCTGAGGGAAATTATCCTGGGACAGATCCTTATGCTGTGGATATGTTAGATGAGTATCTTGCACTTCCAGATGATGATATTCTGAGATATATATCTTTTGATGATTCTCCTCCATCAATGGAGGGTGAACACCCTATTCTAGAGCAGATACCACCTCTTATCCAGCAGGTTAGATCTAGCTACAGTAGCTTATTTACCTGCCACACTAATTACGACTTTATCTGCCTATTAAACATCTTTGTTCATTGACAGAATGTGGAGGAAGAGGCCAAGGATGTTTCAGAGGAGAAACAACAAAAGGTGGAGGGAGAAGCTGCAAATATTTTCAAGACAAACAAACATGACCTTGAAGCAAATTCTAGCCGTGGAGGAGCTGCTTCAGGTGAGACTATTTCACTTAGCATCCTAATTTCTCCAGAATCTCACATATACCATGCAAAGCTTGTGTTCTAGTGGATCTAAGTAGCTTGTGAACTTGCATCCTATATTTGTACTGAGATGAGGAAACTCTTTGTTCACTGCCTGGCAGATGATGCAAATCCAATTGCAAAACGCTTCAAGAAATGGTTGGAAGACATCCCAGCTGCTCCCGCATTTGCTGCAGAGCTTCCATCCAAGAAGGATGCACTCCGGCTTCATTCTGCACCTCAGTCTTCAAATACTACTCATGTAACTGCAGGAATGGTCAGCATTACAAACATTACTGCAAGAGGCAATCACATGAATCCGATGGTGGAAAAGATTGGAGGAGGGTTCAACCATCCCATTATCTCTGCTGTTGTTTTGATACCTGTTTCTGGCTTACTTTGTGGCAAGACTCTGTTTGTGCTGACATATGGATGGGCTTTTCTGGTGACGTTTTCATTTCTGTTTGCCACCGTGACTTGCAAAATTGGAACCTTCATGTATTCTGGAAAATGAGAGTTATGGGGTCTGCTGTGAGACAATGGGGTGCGGTTGGTTATCAATTGGTAGTGATGGTACTCATTGTGACTCAAAGATACTACTCTATTTGAAAGTTGCACAACGAATACCCATAACGGCCTGCTGATATCCAGCCACCGCCCATTGGCTCCTT

>-AiNAC54

AGCTGACAAAAGTATTATTTAGTATCTATGTAAGTATTAAAGTAGTAAAGTGCTAGAACCTTCTTTCTCCTCTAACATGGGGTGGGAACTTTGAACTTTCTATATCACCAAAAACGACTCAAAACGGGTCTCGTTTTGAACCTTGAACCCTAAATCCCCAATTCAAACCACTCTCTCTGATACTCGCAAAGCTCGCTCTTTTAGCTGATTAACCTGCTGTTCAATCGTCATCGTGATCGTCATAATCATACATGAGTCGAATACTCGGTCCCGGTTTCCGCTTCCACCCTACGGACGACGAACTAGTTCAATACTATCTCCGCCGGAAGGTCATCGGAAAACTCAACCACCACGACCACATCGGCGTCATCAATATCTACGACTATGAGCCATGGCAACTCCCCGGTTAGCTTTCTCTCAATAGCTGAATTTCGACCCTAATTTGTTTTTGGTGTCGTGTATCTTAGGGTTTCATAGTGAAATTAGAATTTTATGGTGGTGGTTGTTGTTGTTGTATGTGGTTTTTTAGAATTGTCGAAGCTGAATACTAGAGATTTGGAATGGTATTTCTTCACGGTTCTGGACAAGAAGTACGAGAAGGGGGAGAAGACAAAACGCGCCACCGTCAACGGTTACTGGAAGACCACCGGCAAGGATCGTGGAATCAAGTATGGCGATCGCCAAGTAGGCATGAAGAAGACCCTCGTTTACCATGAAGGAAGGGCCCCGACTGGCAAAAGATCAAATTGGGTTATGCACGAGTACCGGATGGTCGATGAGCAATTGGCGGAAGTCGGATATCAGCTGGTATATATATGTATATGAATTTGCACACGGATATATTTTTTATTTTGAAATCTTTCTAAATTCGTTCGCATTTTTAATTAAATATCTGGCGCTTCGAAAGTTTGGAAAGTTTATAANNNNNNNNNNNNNNNNNNNNNNNNNNNNNNNNNNNNNNNNNNNNNNNNNNNNNNNNNNNNNNNNNNNNNNNNNNNNNNNNNNNNNNNNNNNNNNNNNNNNNNNNNNNNNNNNNNNNNNNNNNNNNNNNNNNNNNNNNNNNTTTTTGTTTTTTTGGTGGGGGGGGGGGGGGTTTGCAGAGTCAAGGTTTGTTTGTTTGATTGGTTTATTTGTGTTTTTGGTGGTGGTGGGCGAAGGACGCTTTTGTGCTGTGTAGAATTTTTGAGAAGAGCGGGATGGGCCCTAAGAATGGAGAGAAGTATGGTGCTCCCTTTAGAGAGGAGGACTGGGTGGAGGATGGCGACCTGCTTGAACCGATTGCTGATGAACCTGTGGTTGAGCTGTCTGTTGACCAGAGTGATGCTTTCCTTGAAACTGATGACCTTGAGAAGGTTCGTATACTATCTTCGAGTCACTGATGATAGATTTCATAATAGTCATATTTCATTAATGCAGATGTGTGCCCCTTAACATTCACGAGGATTTGTGTGGCCATATGTTTCCACATTGTTATGTTAATTGTCTTGTTTTTGTCTCAGTCAAATCATTGATTTGTATGGTCAATACAGATTAGGCTGATTTCAAAGATTGATTTTGCGATTCGTACCCCTTTCTGATTCTGTCCTGTTTCTGTATCACTGGGATGATGAATCAAATTTATTCATGCGATTTGTATCGCGAATTGTCTGATACTGTCAACCTTGGTCAATGGTAGATATCCTCTTTTTCAGCTTTAGTTTATGTGCTAAGTTGATGATTTGAAATATTGCCTTAACAGAAACTTGGTACGCATGTGGTTGATGGAAGTGCTGATTTACCACCAAACCCTCCCAACTACTTTTATGGGGAGTGTAGTCACTATCCTCAGCATCAAGAAGAATTTGTTGAAGTTCCGAAACCTTTGGAAGGTACTGAAGGCCGGAATTTCGATGTAACTGGGCCATATGCTGAGGATACCTGTTTAGAAAATCATGAAATGAACCATAATGGGAATTCTTCAGGATTCATTTATGGTGATGTTAATTCAGATGAAATCATGGATTCCATTGTTGATCCTCTGATTGGTGCTGAATTATTCCTGGAAACAGATGATCTTCTGAACCCAATCGAGGGAAATTCCTCTGGGGCAGATCCTTATACAGTTGAGGGAAATCACCCCAGGGCCGATCCCTACGCAGCTGAGGGAAATCCCCCTGGGCCAGATCCCTACACAGCTGAGGGAAATTATCCTGGGACAGATCCTTATGCTGTGGATATGTTAGATGAGTATCTTGCACTTCCAGATGATGATATTCTGAGGTATATATCCTTTGATGATTCTCCTCCATCAATGGAGGGTGAAAACCCTATTCTAGAGCAGATACCACCTCTTATACAGCAGGTTAGATCTAACTACAGTAGCTTATTTACCTGCCACACTAATTACGACTTTATCTTCCTATTAAACATCTTTGTTCATTGACAGAATGTGGAGGAAGAGGCCAAGGATGTTTCAGAGGAGAAACAACAAAAGGTGGAGGGAGAAGCCACAAATATTTTCAAGACAAACAAACATGACCTTGAAGCAAATTCTAGCCGTGGAGGATCTGCTTCAGGTGAGACTATTTCATTTGGCATCCTAATTTCTCCAGAATCTCACATATACCATGCAAAGCTTGTGTTCTAGTGGATCTAAGTAGCTTGTGAACTTGCATCCTATATTTGTACTGAGATGAGGAAACTCTTTGTTCACTGCCTGGCAGATGATGCAAATCCAATTGCAAAACGCTTCAAGAAATGGTTGGAAGACATCCCAGCTGCTCCCGCATTTGCTGCAGAGCTTCCATCCAAGAAGGATGCACTCCAGCTTCATTCTGCACCTCAGTCTTCAAATACTACTCATGTAACTGCAGGAATGGTCAGCATTACAAACATTACTGGAAGAGGCAATGACATGAATCCGATGGTGGCAAAGATTGGAGGAGGGTTCAACCATCCCATTATCTCTGCTGTTGTTTTGATACCTGTTTCTGGCTTACTTTGTGGCAAGACTCTGTTTGTGCTGACATATGGATGGGCTTTTCTGGTGACATTTTCATTTCTGTTTGCCACCGTGACTTGCAAAATTGGAACCTTCATGTATTCTGGAAAATGAGAGTTATGAGGTCTGCTGTGAGACAATGGGGTGCGGTTGGTTATCAATTGGTAGTGATGGTACTAATTGTGTGACTCAAAGATACTACTCTATTTGAAAGTTGCACAACGAATACCCATAACCGCCTGCTGATATCCAACCACCGCCCATTGGCTCCTT

>AdNAC56

GTTAGATATATGCATTTAACTATGATATCTAATATGAGGATTCTTCTTGTTGAAGTTGTAAGGAGTTATAAAACCATAAAGTGGAGAACCATTTAAGAACCTCATCTTGGTCATGTTATAATTGATGAGGATTCTTCACATCATAGCTTGGTAAACTGAAATTAATTAAGCTCATCATCAAAGGGGTTTTAGGGTTCTTCATAGGAAGAGTAATTGATGGAAGAGAATCTACCTCCTGGATTCAGATTCCACCCAACAGATGAAGAGCTTATAACATATTATCTTACAAGAAAAGTCTCTGAAAATGGATTCACTTCTAAAGCTATTGCTGTTGTTGATCTCAACAAGTCTGAGCCTTGGGACCTTCCAGGTATATATATACACACACATATAGTGCTTTAACTAATTTTAATTTATTTTTTCTTTTTATTTTTTCTTCTATAACGAAGAAAACTGAGGTGCAATTAATTTTATGTGAAATTGATAAGTGTGAATCGTTAAATGATTTAATAAGTTTGATTAAATAATTATTATCAAATAACTCTCAACTATCAACTTCACAAAGTGCACATAAATTTTCACTTTCTTCTATGTATACATAATAATGGACTAGTACTCTCTTTCTTGAGAAAATTAAACTAGCTTTATTTGTTTTTTTTTTTTTTGTTCTAAGGATTATTTTTACAATTGATTTAATTTGCAAATAAAGGCTTATAGATTTTAAGTTTAATCATTGAACCAAAGCTTTTAACTTGCCATTGATTGTATCGAAAAGACTCCAAATTTTTGAAAATATATATAGGAACAAATTGAATTATGAAATTTGGAAAATAGAAAACATTGATGAGCAGAAGTAAATTTTCAACCATATATAAGAGTTAATAAGATCCATGCATGGTGATCTTTTCAATTTGTCTCTCTATAATATATATTGTAATTGATTAAAAAGAAAACACAGCTTTGATCTGATGAGTTTCATAAATCAACTAAGAAAATTATTTCTTTAATTTTTACACTATGCATATAGTTTTGTGTTTTGACCCGTTCTACTTTTGCTGCGCATATATATATATATATGCTTCATAATCCATGAAATAATAATAAATAATAATTAATAACTAAATAACAACATTTCCCAATGTGCATACGCGCATATAGTACTAACTAATTTATTAGTCATACTCATATATATAAATAACTCAACTATTTACCTTTTAAATGGTAGTAAAAGTATTAGTTAATATATATTGGTATACCATACCACTCTCTTAATTTGGTGAACCAAAATAAATAAATAAATGATTATGATTGTGTTGGAACAGGTAAGGCAAGCATGGGTGAGAAGGAATGGTACTTCTTCAGTTTAAGAGATAGAAAGTATCCAACAGGACTAAGAACAAATAGGGCAACAGAATCAGGGTATTGGAAGACCACAGGCAAAGACAAAGAGATATTTCGTGGTGGGGTTTTGGTTGGAATGAAGAAAACCCTAGTCTTTTATAAGGGTAGGGCTCCAAGGGGTGAGAAAAGTAATTGGGTCATGCATGAATATAGACTTGAGAACAAGAATCCCTTTAGAACTAAGGTAATAATTATATGTTATTAATTTATTATCACTCATCACAAGCAATTCACAAACACTATATTAATTAATTAGATTTTCTTAGTTACATACAGAGAATTATGTTCTTTTTTTAAAATTAATATAAAAATTTAATTTTTAATCAGATAATTTTAACTTATTTTTGTAAAACTATTATTTGACATTCATTTAATTTTTGAGGATTTAGAGTTGAGGATTTAACTTTAGAATTTAAAATAAAAAATAATTTTAAAAGAATTAATTTATGTTAGCTGACATTATATAAATAACGACAGATAGGATAATTTTAAAGTTAGATGGTAACAATTTTTATATATATCCTACCAAAATATGGTTAGAGCAGTTGATAATTTTTTTTCTATTAATCATTAATGCCAATTTTGAGTTTTAGGCAAAAAAAAAATCTTAAAATCACGGAAAAAAATGAATTAAAGTGTTCATACTAGGATAGTTATAGTTTTACAAAAATATTAAAGGGAAAAAAGTTAATATTTGTGGTTAGTAGTAGGGGTGTTAAAAATCTGGTCCGAATTCGAGGTGTATTTTGTCGGGCTCGAATTTGTAATTTCTACTCAACATTATTTTTGGGTCGGATTCGGATTATGTTTTGGGTTGTCCGACTTAGTTGGAAGTTATTTTTTACAATGAAATCTATATTTTAGAGTGAAATTAGTAATATCATATTATACATCGAATAGATCTAGTTTAATCGAATTTATTCGGGTCGAGTCAAAATAAATTCTGTTACCTTTTAAAGCCGGATTCACTTAAATCCAGTCCGATCCGACACATAAACACTCCTAGTTAATACATACACTAATGATGAAAAAACAATGTAATTTTATATTTTAATTTCTTTCCATCGTTAATTATGAAAGTTTATAGTATCATAGGCAACTATTCATACATATGTGAATAGATATGTCTTCTGAAATGAATTAATTTACTATTTTTTTAATATAAATTTAATTATGGTTATTGTAGGATGAATGGGTAGTGTGCAGGGTATTCCAAAAGAGCACAGCAGCGAAAAAACCGCCGCAACAAACATCATCCTCCCAACCTGAATCCCCATGTGATGACACAACCTCTTTGGTCAATGAATTTGGTGATGTTATTGAGCTTCCAAATCTAAACACCAACATTAATAATAATAATAATTCTTCATCATCATCCTCAAGTGCTTTATTCCCTAACAACATTCTTATTTCAGGACAACACATTCATCATCACCATGACCTAACCAATAATAATAACAATAATAATGTTAACACAAACATGAACTTAGCAATGAATTGGCCACCATCAAGTGATCATAATATTAATAATGTTCCATGGCCTTCAGTAGGGTTGTTGAATCCAAGTATTTCATCAATGAATTCCTTGATTCTCAAGGCATTGCAGCTTAGGAATAATTATCAACAAAGAGAAGTTGCATCCACATTTCCACCATCATCATATATTATGCCTCATCATCAAGGACTAGTAGTTCCTCATCAACAAGTTATTGGAACCAATAATAATGATGACCTAATAACAACTTCTTCAAATCTCATCAATGCTTCTTCTTCTTCATCAAAAGTTTTGGAATGTATGCCACATCAGCAACAACAACAACAACAGGAGCAACCATTCAATTTGGACTCCCTTTGGTAA

>AiNAC12

ATGGAAGAGAATCTACCTCCTGGATTCAGATTCCACCCAACAGATGAAGAGCTTATAACATATTATCTTACAAGAAAAGTCTCTGAAAATGGATTCACTTCTAAAGCTATTGCTGTTGTTGATCTCAACAAGTCTGAGCCTTGGGATCTTCCAGGTATATATATATACACACACATATACTATAGTGCTTTAACTAATTTCAATTTATTTTTTATTTTATTTCTTTCTTCTGTATATAATGAAAACTCAGGTGCAGTTAATTTCATGTGAAATTGATAGTTGAGAATCATTAAATAATTTAATAAGTTTGATTAAATAATTATGATCTAATAATTTTCAACTATCAACTTCACATGAAATTCACGTGAGTTTTTACTTTCTTCTATATATACATAATAATGGACTAGTACTCTCTTTCTTGAGAAAATTAAACTAGCTTTAATTTATTTGTTATTTTTTGTTCTAAGGATTATTTTTACAATTGATTTAATTTGCAAATAAAGGCTTATAGATTTTAAGTTTAATTATTGAAACAAAGCTTTTAACTTGCCATTGATTGTATCGAAAAGACTCCAAATTTTTGAAAACATATATAGGAACAAATTGAATTATGAAATTAGGAAAATAGAAAACATTGATGAGCAGAAGTAATTTTTCAACCATATATATATAAGAGTTAATAAGATCCATGCATGGTGATCTTTTCAATTTGTCTCTCTATAATATATATTGTAATTGATTAAAAAAAAAAACACAGCTTTGATCTGATGAGTTTCATAAATCAACAAGGAAAATTATTTCTTTAATTTTTACACTATGCATATAGTGTTGTATTTTGACCCGTTCTACTTTTGCTGCGCATATATATGCTTCATAATCCATGAAATAATAATAAATAATAATTAATAACTAAATAACAACATTTCCCAATGTGCATACGCGCATGTAGTACTACCTAATTTATTAGCAATCCCGCTAAGTTACCAACAGTATTTCCACTAACTTCTATCAACTCTTGTTTATAACTGTGTTTAATGGAAGTGTCTTTGTGGATGTGTCTAATAAAAATATCTTTTTTATGATTGTGTCTAATAGAAGTGTCTTTATAGATATATTTCCTGGATGTGTCTTTTTATACATGTGTTTAAAACATAATAATTAATTATTCTTAGTAATAAATTGACAGATAATATGTTAATACCCTATACTTTTTCATTTATTAGTCATACTCATATATATAAATAACTCAACTATTTACCTTTTAAATGGTAGTAAAAGTATTGGTTAATATATATTGGTGTACCATACCGTAGTAGCTATAAGCTAAGCCACTCTCTTAATTTGGTGAACCAAAATAAATAAATAAATAAATGATTATTATTGTGTTGGAACAGGTAAGGCAAGCATGGGTGAGAAGGAATGGTACTTCTTCAGTTTAAGAGATAGAAAGTATCCAACAGGACTAAGAACAAATAGGGCAACAGAATCAGGGTATTGGAAGACCACAGGCAAAGACAAAGAGATATTTCGTGGTGGGGTTTTGGTTGGAATGAAGAAAACCCTAGTCTTCTATAAGGGTAGGGCTCCAAGGGGTGAGAAAAGTAATTGGGTCATGCATGAATATAGACTTGAGAACAAGAATCCCTTTAGAACTAAGGTAACAATTATATGTTATTAATTTATTATCATGCATGCATACCTCATCACTCATCACAAGCAATTCACAAACACTATATTAATTAATTAGATTTTCTTAGTTACATATAGAAGATATATAGCGACAATTTTAAAGTCGGATTGTGATAATTTTAATATATATTCTACCAAAATATGGTTAGAGCAGTAGATTTTTTTTTCTATTAATCATTAATGCCAAGTTTGAGTTTTAGGTACAGAAAAAAAAATCTAAAAATCATGAAAAAAAATTGAATAAAAGTGTTCATAATAGGAAGATAGTTATAGTTTTACAAAAATATTAAAAAAAAAAAGTTAATATCCATAGTTAGAAATAGGGCTGTTTAAGACCTAGTCCGGATTTGAGGTATATTTTGTTGGATTTGGGTTTGTAATTTCTACTCAGCATTATTTTTGGATTAGATTCGACTTATGTTTTAGGCATCCAACTTGATTAGAAGTTATTTTTAACAATAAAATCTATATTTTAGAGTGAAATTAGTAATGTCATATTATATGTTTATATTTCAATTAATATTTTTTATATTATACGGTATTATTTTTATTTTAAAATAATTTATTTTGATATAAATATAATATAATGTTTATTAAATTTATTTTTAAAAATAAAATTTTATTACTTTAATATATAAAATTTACAAATTTGTATATATTAATTTTACATCGAATCGACTTGGTTTAATCCAATTTGTTTAGGTCATTTTCGAGTCAGATCAAAATAAATCCTGCTGTCTTTTAAGACCGGATCCACTTAAATTCAGTCATATCCAATACATAAACACTCTTAGTTAATATGTACACTAATGATGAAAAAATAATGTGATTTTATATTTTAATTTCTTTCCATCGTTAATTATGAAAGTTTATAGTATGATAGGCAACTATTCATACATATGTGAATAGATATGTCTTCTGAAATTAATTAATTTACTATTTTTTTAATATATGGTTATTGTAGGATGAATGGGTAGTGTGCAGGGTATTCCAAAAGAGCACAGCAGCGAAAAAACCGCCGCAACAAACATCATCCTCCCAACCTGAATCCCCATGTGATGACACAACCTCTTTGGTCAATGAATTTGGTGATGTTATTGAGTTTCCAAATCTAAACACCAACATTAATAATACCAATAATAATAATAATAATTCTTCATCATCATCCTCAAGTGCATTATTCCCTAACAACATTCTTATTTCAGGACAACACATTCATCATCACCATGACCTAACCAATAATAACAATAATAACAATGTTAACACAAACATGAACTTAGCAATGAATTGGCCACCATCAAGTGATCATAATGTTCCATGGCCTTCAGTAGGGTTGTTGAATCCAAGTATTTCATCAATGAATTCCTTGATTCTCAAGGCATTGCAGCTTAGGAATAATTATCAACAAAGAGAAGTTGCATCCACATTTGCACCATCATCATATATTATGCCTCATCATCAAGGAGTAGTAGTTCCTCATCAACAAGTTATTATTGGAACCAATAATGATGACCTAATAACAACTTCTTCAAATCTCATCAATGCTTCTTCTTCTTCTTCATCAAAAGTTTTGGAATGTATGCCACATCAGCAACAACAACAACAGGAGCAACCATTCAATTTGGACTCCCTTTGGTAA

>AdNAC17

TGGTAACACTATATGGTGATGCATGCAGTTCAAGAGGGTTAAAATCGTCATTGCAGTTAGTTCAACATTGCTAGGTGTCTCCTTAGAATTTACAACTTGCTATAAACCTGTGGCAACTTGATGATGAAGACCCACATTTGGTGGAACCTTGAGAAACACACTTATCCATGGTTTTTCTTTCTTCATTTGCCTTTGTATACTTAAACCCTAATTAACTTTAGTTTCTTTCATGCATGATTGATTAGTAGTAGCAGTGATAACGAAACACCCTACTCTTCCTTCTCTTTTAATTTGATCTTATTATTTTGTGGTAACCCTCAAGTCAAGTTTTTTCTTCTCAAAGTGAATCATAAAAATTCAACCCAAAATTGAACAAAGAGTTTTGTCTCTCCATGCAAGTAAGCTCTATCTCTTTAATTATTTACTTCTTAGGGTTTTTGTCTAAGCTTTTTCTTTGAAGAAGAAATCAAAGTTAATTTCTTTAATTTAAATTTTTTCCTAAGGGTATTGATGACTCTTTTTGAGAGGTGGATATATTTAATTAGAAAATTGGAGAGTTAGATGTGTAATTGAACTTTGTCTAATTTTTTCTTCAATGATAAATCTATGTTTTGCTTAGCTATAATTTTTCTCTAAGAAAGTTCATTATGATGTGCAAATTTGTATGCTATAGAAGGAAAAGGAATCAATCACCACCAATAAGGAGGGGACTAATAAGAAGGAGATAGAAATAATGGAAGGTTGCAATGGAAAGGAGGAAACCCTACCACCTGGGTTTCGATTTCATCCAACCGACGAAGAACTCATTACTTGCTATCTCATAAACAAGATCTCGGATTCAAACTTTTCAGGCAGGGCAATAACTGATGTTGATCTCAATAAATGTGAGCCATGGGAGCTTCCAGGTGCATATAGTTTGCAACTATTATTATTAGTTCAATTTCTTTGATTTTTTTATATGCATATTCATTTATATTTGAGAAATAAATATCCATCGAATTCAAGATATTTAATTTTCTTAATTTATTTCTTCAATTATTCTCTTAATAGATAGTGCTTATTTTTATATCCATGTTATCATTATTTTTAGATTACTCAACACTATCTAAGGTTTTTATGAGAAATTAATTAATTGATGCACCAAATTTTAATCCCATTCTACTAACTAACGTATATTTTAGTCCATTAAGAAGAGATTTGGATAAGATTTAATTTAAGTGAATGATATACATTTGTTAAATACCTTCTATTACTTTTCTAATTAAACAAAAGGACCACAACAGATTCTCATTGAATCTAATATATTATGTTCCTTGGATGTTTATTTTTTTTTCATAGTTATTTAACAAAAATTTCCATGAATTAGGGAAGGCGAAGATGGGAGAAAAAGAATGGTACTTCTTCAGCCTGAGGGACCGGAAGTACCCAACTGGGGTGCGAACGAACCGAGCCACGAACACCGGGTATTGGAAGACCACCGGAAAAGACAAAGAGATCCTTAATAGTGTTACATCGGAGCTAATTGGGATGAAGAAAACTTTGGTTTTCTACAAAGGAAGAGCCCCCAGGGGAGAGAAGAGTAATTGGGTCATGCATGAATATCGCATTCATTCTAAATCCACCTTTCGAACAACCAAGGTATATATAAATATAATGAAAAAATATTAGTTACTTAATATTTCTGTAAATGATTTTTCTTTCAAACATGTAGTAAAATGCAAATACAAATAACGTATCTTTTATGACAAAACCCTACCCATCTTCTTCTTCCACTAAACTCCAAGAAGTGTCCGCATATCATATCATATATTGCAAATTCAAAAAACCAAACAATTATTAGTCAAGAACCATAACTTAAGGGTTAATTAATCCTTAAGAAAAGATGGCGAGTGGTTTTATATTTACTTAAACATGCTATTTATGTTAAAGTTTTTTGGGGCTTAAAATTTGGAAGAATCAAAGGGGAAGCTACCTTGGTTTATTTCTCTTTTTCTTTTTTTTTTTACTCTTTGTCTCTTTCAAATATGTAGCACTATTTGGCTACCTTTTATTTCTTTTAAAATTTGTCCATGTCAGTTTCATTTTTCTTTAACCCCAATCGAACATAGAATCATTATTAGGTGCTATGATAAAATGTTTTATTCTCTTCAGCAATTTATATCACATAAACAAATAATTTTATTATAAACATTCATTAATGGCTTATCATTCAATTAATACATCAATACACGAGTTTTAATTTACACTAAGAATATCTACTAATTTTTCTCAAACATATTCCTAATATTAAGAGTTTACTCTATTTCGGTATACAAACAAGAATTTAATCAACAAGAACCAAATTGTAAATTATTTGCTTTAAATTTAATGCTACAGAGTTAATAATCTAAAATTATTTTATTTAACTTAACATCTATAATTTTATGCATCTATAATTTTATACATATAATATTTGTAATTACACATTTATTACATCTAATATTTTCTAATTATTCTAACGATAATTAATGAATACAAAATAAATTACTTTTGACTAATTTAAAATAAGTTTTTATTATCTCTTAAATATTTAAATTTTAATCACCTTTATGCCATGTTAATGGATCAATTTCCTCAAAGACTTTACTTAGACTATTTATTGAAGGAAGAAAAATGTTTTCTAACAATCATCATAAGCGTCTTTAATAGTCAATATTAACAATTCTGGATGAACATTAACAACCTTTGATTATTATAGGTGACCAATTTAATGGAGATATAGATTAACTGTCATTAATATTCAACATAACAAACCCTAACACCCTTTTGTGATATATACAGCAGGATGAATGGGTGGTTTGCCGTGTGTTCCAGAAGAGTGCCGGGGCAAAGAAGTACCCTTCTTCCAACCATGCAAGTAGGGCAATGAACCCTTTCAACCTTGAAATAGGTCACCACAATATTGTGCCGCCGCCGCCAATGATGCAACTCGGAGACCCTGCCGCCGCTCATTTCCTCTATGGAAGGAACTATATGAATACTGCAGAGTTAGCAGAAGTAGCTAGGGTTTTGCGTGTTGGTACTGGATCAACCAGTACCAACCTGCCCGGGATGCAGCCTCAGATAAATTATCCAGTGGCTGCGTCTCCAGGAATTGGATTCACAATTTCAGGACTCAATTTAAATCTAGGAGGCGGAGGAGGCGAAACAGTAGTGGCCACAACACAACCAGTTTTGCGGCCCATGCAGCCGACTCCTCCGTCCCAAACATTGGGTATGGTTCCTCATCATCAAGTTCATCATGATGTGAGTTCCAACATGATTTCAACAAATTCCCTTGGTGCTGAGAATGTGGGTTACGTCAATGAAATAAGCAACACAAATGGTGGTCATGGAAATAGGTTTATGGGCATGGATCATTGCATGGATCTTGATAATTACTGGCCTTCCTACTAAATAAAGGAGAGAGACCCTACTTCATTTTGTTTAATTAATTATTATACATAATTTAGTGTTATAAGCTAAGTTGGATAATTAAGCTACGTAAAAC

>AiNAC73

ACATTTGGTGGAACCTTGAGAAACACACTTATCCATGGTTTTTCTTTCTTCATTTGCCTTTGTATACTTAAACCCTAATTAACTCTAGTTTCTTTCATGCATAATTGATTAGTAGTAGCAGTGATAAGGAAGCTCCATACTCTTCCCTCTCTTTTAATTTGATCTTATTATTTTGTGGTAACCCTCAAGTCAAGTGTTTTCTTCTCAAAGTGAATCACAAAAATTCAACCCAAAATTGAACAAAGAGTTTTGTCTCTCCATGCAAGTAAGCTCTATCTCTTTAATTATTTACTTCTTAGGGTTTTTGTCTAAGCTTTTTCTTTGAAGAAGAAATCAAAGTTATTATGTTTAATTTCTTTAGTTTAAATTTTTTCCCAAGGGTATTGATGACTCTTTTTGAAAGGTGGATATATTTAATTAGAACATTGGAGAGCTAGATGTGTAATTGAACTTTGTCTAATTTTTTCTTCAATGATAAATCTATGTTTTGTTTAGCTATAATTTTTCTCTAAGAAAGTTCATTATGATGTGCAAATTTGTATGCTATAGAAGGAAAAGGAAGCAATCACCGCCAATAAGGAGGGCACTAATAAGAAGGACATAGGAATAATGGAATGTTGCCATGGAAAAGAGGAAACCCTACCACCTGGGTTTCGATTTCATCCAACCGACGAAGAACTCATTACTTGCTATCTCATAAACAAGATCTCGGATTCAAACTTTTCAGGCAGGGCAATAACTGATGTTGATCTCAATAAATGTGAGCCATGGGAGCTTCCAGGTGCGTATAGTTTGCAACTATTATTATTAGTTCAATTTCTTTGATTTTTTTTATGCATATTCTTTTATATTTGAGAAATAAATATCCATCAAATTCAAGATATTTAATTTTCTTAATTTACTCCTTCAATTATTCTCCTAAATAGATAGTGCTTATTTTTATATCCATGTTATCATTATTTTTAGAGTACTCAACACTATCTAAGGTTTCTATGAGACAATAATTGACTGATGCACCAAATTTTAATCCCATTATACTAACTAAGGTATATTTTAGTCCATTTAATTCATTAAGAAGAGATTTGCCTTTTTCTTTTTTTAGACATGGATAAGATTTAATTTAAGTGAATGATATACATTTGTTAAATACCTTCTATTACTTTTCTAATTAAACAAAAAGACCACAACAGATTCTCATTGAATCTAATATATTATGTTCCTTGGATTTTTCTTTTTTTTTTTTTTTCTATTTATTTAACNNNNNNNNNNNNNNNNNNNNNNNNNNNNNNNNNNNNNNNNNNNNNNNNNNNNNNNNNNNNNNNNNNNNNNNNNNNNNNNNNNNNNNNNNNNNNNNNNNNNNNNNNNNNNNNNNNNNNNNNNNNNNNNNNNNNNNNNNNNNNNNNNNNNNNNNNNNNNNNNNNNNNNNNNNNNNNNNNNNNNNNNNNNNNNNNNNNNNNNNNNNNNNNAAGTACCCAACTGGGGTGCGAACGAACCGAGCCACAAACACGGGGTATTGGAAGACCACCGGAAAAGACAAAGAGATCCTTAATAGTGTTACATCGGAGCTAGTTGGGATGAAGAAAACTTTGGTTTTCTACAAAGGAAGAGCCCCAAGGGGAGAGAAGAGTAATTGGGTCATGCATGAATATCGCATTCATTCTAAATCCACCTTTCGAACAACCAAGGTATAAATATAAACAAAAAATATTAATTACTTAATATTTCTGTAAATGATTTTTGTTTCTTTTATATATATATATAATGAAACTCAGTCTTTCAAACATGTAGTAAAATGCAAATAAAAATAACGTATCTTTTATGACAAAACCCTACCCATCTTCTTCTTCCACTAAACTCCAAGAAGTGTCCGCATATCATACCATATATTGCAAATTCAAAAAAGCAAACAATTATTAGTCAAGAACCATAACTTAAGGGTTAATTAATCCTTAAGAAAAGATGGCGACTGGTTTTATATTTACTTAAACATGCTATTTTAAAGTTTTTTCAGGCTTAAAATTTGGAAGAATCAAAGGTGAAGCTACCTTGGTTTATATCTCTTTTTCTTTTTTTCTTTTTTTTATTCTTTGTCTCTTTCAAATATATAGCATTAATTGACTACCTTTTATTTCAATTCTTTTAAAATTTGTCCATGTCGTTTCATTTTTCTTTAACCCCAATCGAACATAGAATCATTATTAGGTGCTAAAATAAAATGTTTTATTCTCTTCAGCAATTTATATCACATAAACTAAAATAATTTTATTATAAACATTCATTAATGACTACTTATCATTCAATTAATACATCAATGCACAAGTTATAATTTACACTAAGAATACCTACTAATTTTTCTCAAACATATTCCTAATATTAAGAGTTTACTCTATTTCGGTATACAAACAAGAATTTAATCAACAAGAACCAAATTGTAAATTATTTGCTTTAAATTTAATGCTACAGAGTTAATAATCTAAAATTATTTTATTTAACTTAACATCTATAATTTTATACATTTATAATTTTATACATATAATATTTATAATTACACATTTATTATATCTAATATTATGTATTTATTTTAATAATAATTAAAAAATATAAAATAAGAAAATAAATGAATTAAATAAACATCCAAAAAAGCAAGTTATGGCTCTAAAACAATGTCTCCTAAATATTTTTCTTATTCTATTATTAGCTAATTAATTCTATTACGAGAGCTATATTTCATATGTAATTCAGGAAAAGAAATTGTGGTAGATTAAAATAACGAACTATAATTAAATTATTTTTGACTGATTTAAAATAATTTTTTATTATCTCTTAAATATTTAAATTTTAATCACCTTTATGCCATGTTAATGGATCAATTTCCCCAAAGACTTTATTTAGACTATTTATTGAAGGAAGAAAAATGTTCTCTAAAAATCATCATAAGCGTCTTTAATAGTCAATATTAACAATTCTGGATGAACATTAACAACCTTTGATTATAGGTGACCATTTTGATGGAGATATAGATTAACTGTCATTAATATTCAACATAACAAACCCTAACATGTAATCCTACACCCTTTTGTGATATATATAGCAGGATGAATGGGTGGTTTGCCGTGTGTTCCAGAAGAGTGCCGGTGCAAAGAAGTACCCTTCTTCCAACCATGCAAGTAGGGCAATGAACCCTTTCAACCTTGAAATAGGTCACCACAATATTGTGCCGCCGCCGCCAATGATGCAACTCGGAGACCCCGCCGCCGCTCATTTCCTCTATGGAAGGAACTATATGAATACTGCAGAGTTAGCAGAAGTAGCTAGGGTTTTGCGTGTTGGTACTGGATCAACCAGTACCAACCTACCCGGGATGCAGCCTCAGATAAATTATCCAGTGGCTGCATCATCCCCAGGAGTTGGATTCACAATTTCAGGGCTCAATTTAAATCTAGGAGGCGGAGGAGGCGGAACAGTAGTGGCCACAACACAACCAGTTTTGCGGCCCATGCAGCCGACTCCTCCGTCCCAAACATTGGGTATGGTTCCTCATCATCAAGTTCATCATGATGTGAGTTCCAACATGATTTCTGGTGCTGAGAATGTGGGTTACGTCAATGAAATAAGCAACACAAATGGTGGTCATGGAAATAGGTTTATGGGCATGGATCATTGCATGGATCTTGATAATTACTGGCCTTCCTACTAAATAAAGGAGAGAGACCCTACTTCATTTTGTTTAATTAATTATTATACATAATTTAGTGTTATAAGCTAAGTTGGATAATTAAGCTACGTAAAAC

>AdNAC59

ATGGAACAAGAAGAAGAACCACAACAAAATGAGCCACCTCACTCTCACTCTCAATCTCGGTGCGTGACGCTACCTCCCGGTTGCCGGTTCCATCCTTCGGAGGAGCTTCTATTGAGTTACTACCTCACCAACAAAAATGGCACGGGGAACTGGAATGGTAACGGTGGTTTGGGATTCGATGGTTCTGATTTGATTCGGGAGCTGGATTTTTACGATTACGATCCTTTTGAACTTCCGGATTTTGCGTGCTTTGCGTACGGCTACGGCGGGAGGAGGAGGCACTGGTACTGTTTCACCACCGTTAGGGTTTCGAGGGGAGAGAGGTGGAAGAGGAAGAGGAAGGTTAAGAGTGGGTTCTGGTTGAAGAGGGGAAGGGTTTCGAATGTTAACGGTGTTGGGGAGAACGTGGTTTTGGGAACGAGGACAAGGTTCGTTTTCTATATGGGTGATTCGGCGAAGAACGGTGCCAGGACGGATTGGGTTTTGTATGAATACGCATTGGTTGATCATGTTATGGTACGAATTTCGGAAATCAGAACGCTTTTGATTGTTATCTATATTGTTTCGTTAAAGTTTCTGGTTTGTTGCGTTGTTTGATAATCCTTTGGGGTTTAGGATTTAGAGTTTAGTTTAAGATTGAAGAGAAATTGCTATACGAGAGGTTTAGTAATCTTAATCAGATTGGTTAGTTTCGGCAGAATTCAAATATTGGTGATTTCAATAGGAAAGTAATTTTGTTACTTAGAAAGTCATGTGTTGGTTGAGTTCATGCACTAAACACGTTGGGGTGGGGACACAGTACCCATTTGATTAGAGATGGTGGAATTTTGCTGTAGTTGGTTTTGTGTCGAATAACCGGATGAATCCAATTAGTGTAGATTCTAGCACGGAAGTGGGAAATAATGGGAGAAAGAGGAAACTACATGAAATAATTTAGATGTTCATAGTTTTTCTATATATATACATAAGGTTTATTGGTATATATGGTTAATGTCGAGTGATGTACTGTGACATTGTCTGATTTATCAGGCTAATATTACATAGAGAAGCGAGGTTTTCTTCGCAATGGAAATATATTTTGTTACTTGTTAGTTGGGATGTTTTTAAATATCCATTCAAATGCTGTGTTGTTGATTTTATGCTTGTGAGTTCTGTGTTGGATACTCCTTTATTTCTTCTACTTGAGTTGGACAGTGCAGCAGTTAATTGTGGATATTCTTTGTTGTAATGAAGATTGTGATACTCTTGGATAATTTGGATGTTCGTAATTTGCAGCTGATTCCGAACTTCTGATATATGATTTGGCACGGTTTTGCCTACCATGTTTCAACTCTTAAAAGAGTTTGATGAAAGCCTTTTTTTATTTGGAACAATTTTGAAGAAAGTTTATATACTTAAACTATTTTAACATTTTGGTATTTGTAACTTTATATTACTCCAGATTTTTTCTTACCATTTGAATTTGCTAAACCATGAATTCACAAAAGATTTTAATCTCTTGGGTGACTCGTTGGAGGGTTTCTTCACTACCAAATATAGTAAATTCATAACACTTACGAAGTAACCACTCTGCCTTGGCATCCTCATTTACTCTGCCCTTGTTATCTAGTATTCTGTTCTTTCATTATAGTATTTTAAAGATTCATGTTTTATCTATTTATTCCCTTTCTATTGAAAATTTCACGTGTAGGCCTCTTTTGTTCTTTGCCGGGTATTTAGTAAGCCTCGTTATAAGAATAGTGCATCAGACATCGGCCTGAGTTGTTGTGCAGAAGAGAGTGTATCAGCAGTGCGCCATATTGGTATTCAGCATGATGAACATGTTAAATTGGATGCCGTTGAAGCTAAAGTATGTGATGATATCTCCATTGACCACAACAATGAAATATGTGCTGGTGGAAACAGCGATAATCAAGTTAAGAATGCACATGACATAGATGCTTTACGATGTTTGGCGGCTCCTCAGGGCAGTCAGCAGGTACCCTATCTACCTTTGATTGATCTGAAATATTTAGAGCTGGTTTGGTTTCTATTTTCATTTACTGTATTTATTTTCTCTGTTTTTTTTTTTCCGATTTTAAAGATTTTTGTGAATAAAATAATGGAAACATCAAATAAGGGTTTATCAAATAATGACAACACATACAAATCCATGATTTAGTGAGAAACCATTGTTGTTTTCTTTTACTTTTTTCCTTTCGTTTTATTTCTTTCTTTTTTATCCATTCATGCTAGATGTGTATTTTGCTTACTGCTATCAAGATTTTATTGGTATTTTTTTTCTTTTTTGGGTTAAAACTGAATAGTTCAAGTACTATAATTGGGGTTATTTTTGTTATGCAGCTATCAATTGCAAACTTCTACGTCAGCAATAATCTGAAAAGTGTAGTCAGAAATGGGGCTATCAGTGTCGGGGAGTCATTTAGCGTTTCTATCTTCTTTACAATTACCTTCCCCCTTTTTAGATTTGCTAGATTTGAGAACTCATTCATATGTTATTGGTTGCGTGCCCCTTTTTTTCCCTGCTAATATCATTATTAATATTTGATTAAATATGTTATGCTTTCTTCTGAATGGGAAAACATATTACTGGTCAGTAGAAACCGCTATAAGTAAATTTAGCAGTAATATGTCCTTCCATCCAGCTTGCTTTCCAGCAGACTTTCGCTTTAGCATGCTCTTTTGCCAGTGAACCTGTTCTTTTCATTTTGTAATTGGTAATAGAATTTAATTTTGATGCACTATTAGTGTAGAAAAGTTCTACATAAGCATCCAGTCATATATTGTTACATGAACACAAATGTTTGACTCAACTACTCACTAATCACTACATTAAAAATCTAGCAAATAAATATATATGATTGGATGATAATGTAAAGTTTTCTTTTAAACTCACAGTGCATCAAAATTAATATCTAGTTATTATGCGGTAGAAAGGAAATTATTGCTAAAAACATTAAACCCTTTTGTGTGTTATTGATGATAATCAGGAAAGGCTTCCTTTACTCCCCAGCGGTAGTACAATGTTCATTGAAGCAATTTCATCTCAACAACAATTACTTTCCATCACGGAGGAAGACTTCATAGAGTTGAATGATCTTACATGAATTGGATGGCTTTGAGAAAGATTTCAATATTAATCCTGCTCTCAGGAGTATTCCCCTGAAACTCATTAGTTCCGAATGGGTAAATATCCCATCAAAGTGGATAAACATTCACAACGTCGGAGAAATATTTACGATATCAGGTAAACCTTTGGCTGTCATATGAAGATCATATTGTCAGATCAGGTGAAGGAAAACATCACAGGGTGATCGCTTCACAAAATAAGGTAGTTACTTTGATGACATTTCAGAGTACGTTAAAGTCTTGAGCCATACCGGAGGATATTTAGTAGATATTGGTTTTGGGCATGCAGATTTTCAACTTTGCCTACTGAAAGGCAAGTTATGCAACTGGCTCCAGAGGAATAGAGTTAGGAGTTAGTTTATTGGCATGGTGATTGCAATATGCAATTTACTTCAAGTATGGCAAACCTGACGTTAGTTCCTCATGCTGGCATTGATCCCTT

>AiNAC59

ATGGAACAAGAAGAAGAACCACAACAAAATGAGCCACCTCACTCTCACTCTCAATCTCGGTGCGTGACGCTACCTCCCGGTTGCCGGTTCCATCCTTCGGAGGAGCTTCTATTGCGTTACTACCTGACCAACAAAAACGGCACGGGGAACTGGAATGGTAACGGTGGTTTGGGATTCGATGGTTCTGATTTGATTCGGGAGCTGGATTTTTACGATTACGATCCTTTTGAACTGCCGGATTTTGCGTGCTTTGCGTACGGCTACGGCGGAAGGAGGAGGCACTGGTACTGTTTCACCTCCGTTAGGGTTTCGAGGGGAGAGAGGTGGAAGAGGAAGAGGAAGGTTAAGAGTGGGTTCTGGTTGAGGAGGGGAAGGGTTTCGAATGTTAACGGTGTTGGGGAGAACGTGGTTTTGGGAACGAGGACGAGGTTCGTTTTCTATATGGGTGATTCGGCGAAGAACGGTGCCAGGACGGATTGGGTTTTGTATGAATACGCATTGGTTGATCATGTTATGGTACGAATTTCGGAAATCAGAACGCTTTTGAATGTTATCTATATTGTTTCCGTAAAGTTTTTGGTTTGTTGAGTTGTTTGATAATCCTTTTGGGTTTAGGATTTAGAGTTTAGTTTAAGATTGAAGAGAAATTGCTATACGAGAGGTTTAGTAATCTTAATCAGATTGGTTAGTTTCGGCAGAATTCAAATATTGGTGATTTCAATAGGAAAGTAATTTTGTTACTTAGAAAGTCATGTGTTGGTTGAGTTCATGCACTAAACACGTTGGGGTGGGGACACAGTACTCATTTGATTAGAGATGGTGGAATTTTGCTTTAGTTGGTTTTGTGTAGAATATCCGGATGAATCCAATTAGTGTAGATTCTAGCACGGAAGTGGGAAATAATGAGAGAAAGAGGAAACTACATGAAATAATTTAGATGTTCATAGTTTTTCTATATATATACATAAGGTTTATTGGTTTATATGGTTAATGTCGAGTGATATACTGTGACATTGTCTGATTTATCAGGCTAATATTACATAGAGAAGCAAGGTTTTCTTCGCAATGGAAATATATTTTGTTACTTGTTAGTTGGGATGTTTTTAAATATCCATTCAAGTGCTGTGTTGTTGATTTTATGCTTGTGAGTTCTGTGTTGGATATTTGTTTCTGCTTGAGTTGAACAGTGCAGCAGTTAATTGGGGATATTCTTTGTTGTAATAAAGATTTTGATACTCTTGGATAATTTTGATGTTCGTAATTTTGCAGCTGATTCCGAACTTCTGATATATGATTTAGCACGGTTTTGCCTGCCATATTTCAACTCTTAAAAGAGTTTGATGAAAGCCTTTTTTTATTTGGAACAATTTTGAAGAAAGTTTATGTACTTAAACTATTTTAACATTTTGGTATTTGTAACCTTATATTACTCCAGATATTTTCTTACCATTTGAATTGGCTAAACCATGAATTCACAAAAGATTTTAATCTCTTGGGTGACTCGTTGGAGGGTTTCTTCACTACCAAAATATAGTAAATTCATAACACTTACGGAGTAACCACTCTGCCTTGGTATCCTCATTTACTCTGCCCTTGTTATCTAGTATTCTGTTCTTTCATTATAGTATTTTAAAGATTCATGTTTTATCTATTTATTCCCTTTCCACTGAAAATTTCACGTGTAGGCCTCTTATGTTCTTTGCCGGGTATTTAGTAAGCCTCGTTATAAGAATAGTGCATCAGACATCGGCCTGAGTTGTTGTGCAGAAGAGAGTGTATCAGCAGTGCGCCATATTGGTATTCAGCATGATGAACATGTTAAATTGGATGCCGTTGAAGCTAAAGTATGTGATGATATCTCCATTGACCACAACAATGAAATATGTGCTGGTGGAAACAGCGATAATGATAATCAAGTTAAGAATGCACATGATATAGATGCTCTACGTTGTTTGGCGGGTCCTCAGGGCAGTCAGCAGGTATCCTATCAACCTTTGATTGATCTGAAATATTTAGAGCTGGTTTGGTTTCTATTTTCATTTACTGTTTTTATTTTCTCTGTTTTTTTCCGATTAAAGATTTTGTGAACAAAATAATGGAAACATCAAATAAGGGTTTATCACTTTATTGTTTTTACTGTGTTCCTTCACAAAAGTCTAGTAAATAGGAAATTAAAGTAGGAAATGACAACAAACACTTACAAATTCATGATTTAGTGAGAAACCATTGTTGTTTTCTTTTATCTTTCCTTTCATTTTATTTCTTTCTTTTTTATCCATTCATGCTAGATGTGTATTTTGCTTACTGCTATTAAGATTTTATTGGTAATTTTTTTTTCTTTTTTGGGTTAAAACTGAATAGTTCAAGTACTATAATTGGGGTTATTTTTGTTATGCAGCTATCAATTGCAAACTTCTACATCAGCAATAATCTGAAAAGTCTAGTCAGAAATGGGGCTATCAGTGTCGGGAGTCAATTAGCGTTTCTATCTTCTTTACAATTACCTTCCCCCTTTTTAGATTTGCTAGATTTGAGAACTCATTCATATGATCTTGGTTGCATTCCCCTTTTTTTCCCTGCTAATATCATTATTAATATTAGATTAAATGTGTTATTCTTTCTTCTGAATGGGAAAACATATTACTGTTCAGTAGAAACCGCTATAAGTAAATCTAGCAGTAATATGTCCTTCCATCCAGCTCTTGCTTTCCAGCAGACTTTTGCTTTAGCATGCTCTTTTGCCAGTGAATCTGTTCATTTCATTTTGTAATTGGTAATAGAATTTAATTATGATGCACTATTAGTATAGAAAAGTTCTACATAGGCATCCAGTCATATATTGTTACATGAACACAAATGTTTGACTCAACTACTCACGAATCACTACATTAAAAATCTAGCAAATAAATATATATGATTGGATGATAATGTAAAGTTTTCTTTTAAACTCACAGTGCATCAAAATTAATATCTTGTTATTATGTGGTAGAAAGGAAATTATTGCTAAAAACATTAAACCCTTTTGTGTGTTATTGATGATAATCAGGAAAGGCTTCCTTTACTCCCCAGCAGTAGTACAATGTTCATTGAAGCAATTTCATCTCCACAACAATTACTTTCCATCACGGAGGAAGACTTCATAGAGTTGAATGATCTTACATGAATTGGATGGCTTTAAGAAAGATTTCAATATTAATCCTGCTCTCAGGAGTATTCCCCTGAAACTCATTAGTTCCGAATGGGTAAATATCCCATCAAAGTGGATAAACATTCGCAACGTCGGAGAAATATTTATGATATCAGGTAACCTTTGGCTGTCATATGAAGATCATATTGTCAGATCAGGTGAAGGAAAACATCACAGGGTGATCGCTTCACAAAATAAGGTAGTTACTTTGATGACATTTCAGAGTACGTTAAAGTCTCAAGCCATACCGGAGGATATTTAGTAGATATTGGTTTTGGGCATGCAGATTTTTCAACTTTGCCTACTGAAAGGCAAGTTATGCAACTGGCTCTAGAGGAATAGAGTTAGGAGTTAGTTTATTGGCATTGGTGATTGCAATATGCAATTTACTTAAGTATGGCAAACCTGACGTTAGTTCCTCATGCTGACATTGATCCCTT

>AdNAC37

CACTAACATATTATGGCAGAAAACCACTGTGTATTGTGCTGATGAGGTGTTAAGCAGATAATATAATTTTAATTTGGTAGAGTGGTGCCGCTAGGTGAAGGTCAATTTCAACGAGTAAAACCAATTTGGTTGGTTTTCTCTGTCTTTTCTTACAAACTTTACATGCAAGTGATTGGTTCCTCTCCCTTTCAATCTTCACATGTGCATGGGACTAGAGCCATATATATAATGTGGATGACACACTTTGGTTTCTTATTAAAAGCAAGCCAAAAATTCATATTGGTATCATCGGCACTTGTTGATTCCTTTGTCAAGAACAAAGAAAATTCTTTTCATGGAAGAAGGAGGAGGAGATCAACATGCCTCTAACAGCAGCTACACTTTTCCACCAGGTTTCAGATTCCACCCTTCTGATGAAGAACTCATAGTTCATTACCTACAAAACAGAATCAGCTCTCGTCCACTTCCAGCTTCCATTATAGCTGAGATTGATCTTTATAAGTATAACCCTTGGGATTTGCCAAGTATGTTCTTTAATTCCTCATCCACTTTGTGTGTTCACTAAAATCTTTCCTTGTCATTATAAAGATAGTATTTGCTTTTTCTTGTCATTGTGCAGAGAAGGCTTTGTTTGGAGAGGAAGAATGGTACTTCTTTAGCCCGAGAGATCGCAAGTATCCAAATGGATTGAGGCCAAACAGGGCAGCAGGTTCAGGGTACTGGAAGGCTACTGGAACTGACAAGCCGATTCTCACATCTTATGGATCGAAGCGCATCGGAGTGAAGAAAGCTCTTGTCTTCTATTTAGGTAGACCTCCAAAGGGGACTAAAACTGATTGGATCATGAATGAGTATAGATTGGTTGACACAATCACCAGCCCCTCCAGGCTCAAAGGTTCCATGCGCGTAAGTTTTCATAAAGAAATATTTGTGCCACTCATTTTTCTTTTCTTTTATTGTGTAAAATCATCATTCCATGCTCTTACAGTTAGATGACTGGGTACTCTGTCGCGTTCGACACAAAGGCTACTCATCGAAGAACTCATGTGAGAATCAAGATAATCCTTGTGAACCAAACATGCTATCAAATCTGCCAAGGTGTGATGAAGGTTATCCAGCAACAAACATGAACTTCCATGCTGATATGATCACTGATTATCAATACAAAGACTATCAGATCCTAGCTTCTATCCTTGTTGGTGGCCATGTTCCTACCACTGAGAGCATGTCAAGTTTGAACTTGAAGGATGGCAAAGGCAATGATCCAATAACTTCAGTTCATGAAGATGGTTTCCACAGAGAAGATTCTTCTACAACAGTTTCTCCTTTGGACTGTTACTTCAACTCACTGAAAAGAAAATCTAATGAGGATAGCCAATATGAGAATCTCATTTCCTTTAACAGGAAGTTGAACATGGAGACCACAATGGATGATGAATCTTCTATCATCAATGGAGGTTTGAACTTCTACAATCAAAACCAGTCTCAAGATGACATAATATTCAATAAGAGAGCAGCAGAGCCTAGCATCAACTTTCAAGAGCTAAAGCAATCAGCTTTTATAGGAAGATACCCGCAATGCTCAAGTGATTGACCATAAGACATATTCAATACTCTCTATTCCACTTGTATATTTTCATGGATATACAACTAAGAATATAGGCCTCAAATATTAAAGTGAATTATTGTTCTTTAAGCGTGGTTGTAATAAATATATATAGAACTTTTCTATATACTTATGAAATTCCTTGAAAACATTGTGAATAATGCTAGCTTGTGCAGAATCTGCACAATGTCATTTCAACTAGAATTTTATCCTACTCAGTAATAATAAAACTTGTACAAACAATTTATATTCTTCCCACAAAATAGTTGC

>AiNAC64

CACTAACATATTAAGGCAGAAAACCACTGTGTATTGTGCTGATGAGGTGTTAAGCAGATGATATAATTTTAATTTGGTAGAGTGGTGCCGCTAGGTGAAGGTCAATTTCAACGAGTAAAACCAATTTGGTTGGTTTTCTCTGTCTTTTCTTACAAACTTTACATGCAAGTGATTGGTTCCTCTCCCTTTCAATCTTCACATGTGCATGGGACTAGAGCCATATATATAATGTGGATGACACACTTTGGTTTCTTATTAAAAGCAAGCCAAAAATTCATATTGGTATCATCGGCACTTGTTGATTCCTTTGTCAAGAACAAAGAAAATTCTTTTCATGGAAGAAGGAGGAGGAGATCAACATGCCTCTAACAGCAGCTACACTTTTCCACCAGGTTTCAGATTCCACCCTTCTGATGAAGAACTCATAGTTCATTACCTACAAAACAGAATCAGCTCTCGTCCACTTCCAGCTTCCATTATAGCTGAGATTGATCTTTATAAGTATAACCCTTGGGATTTGCCAAGTATGTTCTTTAATTCCTCATCCACTTTGTGTGTTCACTAAATCTTTCCTTGTCATTATAAAGATAGTATTTGCTTTTTCTTGTCATTGTGCAGAGAAGGCTTTGTTTGGAGAGGAAGAATGGTACTTCTTTAGCCCGAGAGATCGCAAGTATCCAAATGGATTGAGGCCAAACAGGGCAGCAGGTTCAGGGTACTGGAAGGCTACCGGAACTGACAAGCCGATTCTCACTTCTTATGGATCGAAGCGCATCGGAGTGAAGAAAGCTCTTGTCTTCTATTTAGGTAGACCTCCAAAGGGGACTAAAACTGATTGGATCATGAATGAGTATAGATTGGTTGACACAATCACCAGCCCCTCCAGGCTCAAAGGTTCCATGCGCGTAAGTTTTCATAAAGAAATATTTGTGCCACTCATTTTTCTTTTCTTTTATTGTGTACTCATCATTCCATGCTCTTACAGTTAGATGACTGGGTACTCTGTCGCGTTCGACACAAAGGCTACTCATCGAAGAACTCATGTGAGAATCAAGATAATCCTTGTGAACCAAACATGCTACCAAATCTGCCAAGGTGTGATGAAGGTTATCCAGCAACAAACATGAACTTTCATGCTGATATGATCACTGATTATCAATACAAAGACTATCAGATCCTAGCTTCTATTCTTGTTGGTGGCCATGTTCCTACCACTGAGAGCATGTCAAGTTTGAACTTGAAGGATGGCAAAGGCAATGATCCAATAACTTCAGTTCATGAAGATGGTTTCCACAGAGAAGATTCTTCTACAACAGTTTCTCCTTTGGACTGTTACTTCAACTCACTGAAAAGAAAATCTAATGAGGATAACCAATATGAGAATCTCATTTCCTTTAACAGGAAGTTGAACATGGAGACCGCAATGGATGATGAATCTTCTATCATTAATGGAGGTTTGAACTTCTACAATCAAAACCAGTCTCAAGATGACATAATATTCAATAAGAGAGCAGCAGAGCCTAGCATCAACTTTCAAGAGCTAAAGCAATCAGCTTTTATAGGAAGATACCCACAATGCTCAAGTGATTGACCATAAGACATATTCAATACTCTCTATTCCACTTGTATATTTTCATGGATATACAACTAAGAATATAGGCCTCAAATATTAAAGTGAATTATTGTTCTTTAAGCGTGGTTGTAATAAATATATATAGAACTTTTCTATATACTCATGAAATTCCTTGAAAACATTGTGAATAATGCTAGCTTGTGCAGAATCTGCACAATGTCATTTCAACTAGAATTTTATCCTACTCAGTAATAACTAATGATAAAACTTGTACAAACAATTTATATTCTTCCCACAAAATAGTTGC

>AdNAC39

TCTTTCTCTCTCTTCCCCCAGATAGCAGTAGTATTTAGGTGCCTCCTTTTTCTCTCCTTCGTTTTCCCTTCTCATTATTCACATCCATCTCCATATTCCATCTCTCTTTCCTCTCCCCCCCTCTCTCTATATATTGAAAGTACAATAATATAAGTAAGAAAGAAAGATCAGAGCGAGTGAGTGAATGAAATGGAAAAAGTGGCATCATTGGTGTTGAAGGAAGAGGAGCAGATGGATTTGCCACCGGGGTTTCGGTTTCACCCAACTGATGAAGAGCTCATAACCCATTACCTATACAAGAAAGTTATTGACACCAACTTCGCCGCGAGAGCCATTGGCGAAGTCGACCTTAACCGCTGCGAGCCTTGGGATTTGCCATGTAACTAACACCGCCAACATCAATTCATTATCTTCTATCTCTCTCTCTAATTTTCTGATTATTTTACTTTTATTTGTTTAGGGAAAGCGAAAATGGGAGAGAAAGAATGGTATTTCTTCTGTGTGAGGGATAGGAAGTATCCGACCGGGTTGAGGACAAACCGGGCGACCGAGTCTGGGTACTGGAAGGCAACCGGGAAAGACAAGGAGATTTTTCGAGGCAAATCGCTCGTCGGAATGAAGAAAACGCTTGTCTTCTACAAAGGAAGAGCACCAAAAGGAGAGAAAACAAACTGGGTCATGCACGAGTTCAGGCTGGAGGGGAAATTCTCCATCCATAACCTCCCAAAAACCGCAAAGGTAAACCGATATACCCTGTTTACTGTTTAATTTCCTCTGTTTTTCCAACTTGGAATTTAACAATCTTTCTGGCAGAACGAGTGGGTGATTTGCAGGGTGTTTCGGAAGAGTTCAGCTGGCAAGAAGGTTCACATCTCTGGAATCATGAGGCTCGACACTTTCCGAACCGAATTGGATTCTTCCGGTCTACCTCCCTTGACGGAGACCTCGCCCTCTTTCGACACCATCCATGACGAATCGCCTTACGTGCCCTGCTTCTCCAATCCAATTGATGTTCCAAGAAACCAAGCCGCAGGCGGAAGCGGAGGAGGAGGAGGAGTCTTTGGTGGTTCCTTCCCCAACAACTCCTCCTCTTCTGTTCCCGCCTATGCGGTTTCTTCCAACATTCTCCCAAGGATGCCGATTTGCGGCGGTTCCTTGTACTCTACTCAACATCAAGATCAGAGTATCCTGAGGGCGTTGTATGAATCGAACGAAAGGGAAATGATCAGTGTGTCACAGGAAACAGGCCTCACTACTGAGATGAACGTGGAAACCAATTCCGTGGTGTCCAATTTTGATTTAGGGAGGGCTCACTTTGAGAGTTTGTGGAATTACTGA

>AiNAC38

TCAGAGCAATGGAAAAAGTGGCATCATTGGTGTTGAAGGAAGAGGAGCAGATGGATTTACCTCCGGGGTTTCGGTTTCACCCAACTGATGAAGAGCTCATAACCCATTACCTATACAAGAAAGTTATTGACACCAACTTCGCCGCAAGAGCCATTGGCGAAGTCGACCTTAACCGCTGCGAGCCTTGGGATTTGCCATGTAACTAACACCACCAACATCAATTCATTATCTTCTCTCATCTCTCTCTCTCTCTAATTCTCTGATTATTTTACTTTTATTTGTTTAGGGAAAGCGAAAATGGGAGAGAAAGAGTGGTACTTCTTCTGCGTGAGGGATAGGAAGTATCCGACCGGGTTGAGGACAAACCGGGCCACGGAGTCTGGGTACTGGAAGGCAACCGGGAAAGACAAGGAGATTTTTCGAGGCAAATCTCTCGTCGGAATGAAGAAAACGCTTGTCTTCTACAAAGGAAGAGCACCTAAAGGAGAGAAAACAAACTGGGTCATGCACGAGTTCAGGCTGGAGGGAAAATTCTCCATCCATAACCTCCCAAAAACCGCAAAGGTAAACCAATATACCCTGTTTACTGTTTAATTTCCTCTGTTTTTTCCAACTTGGAATTGAACAATCTTTGTGGCAGAACGAGTGGGTGATTTGCAGGGTGTTTCGGAAGAGTTCAGCTGGCAAGAAGGTTCACATCTCTGGAATCATGAGGCTCGACACTTTCCGAACTGAATTGGATTCTTCCGGTCTACCTCCCTTGACGGAGACCTCGCCCTCTTTCGACACCATCCATGACGAATCGCCTTACGTGCCCTGCTTCTCCAATCCAATTGATGTTCCAAGAAACCAAGCCGCAGGCGGAAGCGGAGGAGGAGGAGGAGTCTTTGGTGGTTCCTTCCCCAACAACTCCTCCTCTTCTGTTTCCGCCTACGCGGTTTCTTCCAACATTCTCCCAAGGATGCCGATTTGCGGCGGTTCCTTGTACTCTACTCAACATCAAGATCAGAGTATCCTGAGGGCGTTGTATGAATCGAACGAAAGGGAAATGATCAGTGTGTCACAGGAAACAGGCCTCACTACTGAGATGAACGTGGAAACCAATTCCGTGGTGTCCAATTTTGATATAGGGAGGGCTCACTTTGAGAGCTTGTGGAATTACTGA

>AiNAC16

CTCAAACACCCTAATCACACCCTTAATCAACGACTTGAATACCACGCAATCACATCATGTCACGGAGCTCACACTCTCTCCCTGCTCCCTCATCGCACTGAATCACTCTCCCATATGAAGGTTCCCAGTCATTACTGCTAGGAGTCTAAGTACTACGCCAAGCTTCCTTCTTTGACCATAACACTATCCAATTCCAACCCCCCAGACCCCCTTTCTTCTTTTTCACACCGCAATTTTTTCCACAATCGATCCCATTTTAAGCTTCTCTCTCCCTCTAGGTTTTTTTTTTTTTTCATTCGACGGTTCATTCCTCGAATTCTCGTACCCCGAGGATTCGATTCGGGTCATGTAGTCGTAGGTTTTCGGTTTCTACAACCGCTTGATCCACAAAGCTCGGATTCTTTTTTTTCTTCACTTTTTTGGGGGGTTGTGAATTCTGATCCTCTATTTTTGGGGGTTTCGATTCCGTGATTCAATTTCTTCTTCCGGATTCCTCTAATCGCATCGATTAACTGGGTGTTTGGTTTCGTAGCCTGTCGATTCAGAAAGTTTCGGTTTGTTCCTTTGGGTGTTTGGTCTAGGGTTTTTCGGATTGAGTATGGGTGCTGAAGCTGGTGCAACTGAGTGTTTCAGTAAGGCCATGGCGTCGATGCCTGGGTTTCGGTTTCATCCCACGGATGAGGAGCTGGTTATGTACTATCTGAAGAGGAAGATATGTGGGAAGAAGCTGAAACTCGACGTGATTCTAGAAACCGATGTTTACAAGTGGGATCCTGAGGAATTGCCAGGTATACTGTTATACTCTGTGAACTCGAGTGTTTCTGACTGTTATAGCGTTGACTTAGGGTTTGCGTTATTTGTGTAATTGTGTTCTTTTGTTTGATTCTTATCAAGTTGTTGATTGATACTTTAGGATGTATGTGTTTAAACTTGGGTCCTCGTGTTGCGAATAGTGTGATTGCTTGGGTTAGAATACTAATAAACCAATTATAGTTGATATTCTGAATGCTTGTGTTGAGATATCTTGACTGATTTGGTGCATATTCCATATAAATTGAATATATGGCTTTCGTGCGATGATCTGTCAGATTATGGCTTTCTTGGTCCGTCCAAACTGGGATTTTTTACTTACCATATAATTACGGTTAATAACGTTTGCTAATAATTTATCTGTTCATGGGATTTGTGGTTTTGGTGTTCGAAGGTTGCAGTTACTCTTATGATGCGAACAAATTATACAGATAGGGTATGATGCTGGTACACTGCTTGCACCATTGTCAAACTTAGTCCAGATAGGGTTTTAAGTTGATATTAAAGATTGTATGGAACTTTTTTTTTTCCACTTTTCTTTATTTTCTTTTCGGTTGATTGCTGATAGTTTGTAATATTTCGCAGGGATCTCTGTACTGAGGACTGGAGATAGGCAATGGTTCTTTTTCACTCATAGAGATAGGAAGTATCCTAATGGTGCGAGGTCCAACCGAGCAACAAGGCAAGGTTACTGGAAAGCAACAGGAAAGGATCGTAATGTGACCTGCAATTCTCGGTCAGTTGGAGTGAAAAAGACTCTGGTTTTCTATAGAGGCAGAGCTCCTAATGGTGAGCGGACCGATTGGGTTATGCATGAATACACCATGGATGAAGAAGAGCTGAATAGGTGCCAGGATATTAAGGTATCTTCCTGAAGTAACATTTTTTATTTAAATTTACTGGATGATTGTTTTCTATACTCTATTCTCATTCATCATGGAAAGAACATTGCTTACTGATCATCTTTTTTTCGTTATCAGGACTATTTTGCACTTTACAAGCTATACAAGAAAAGTGGACCTGGTCCTAAAAATGGAGAACAGTATGGTGCACCATTTAAAGAAGAGGAGTGGGCTGATGATGAATGTGTAGATTTCAATATTAACTCAGCAGATCGGGAGGTAGTAAATACTGTCCCTGTTAATGATCAGCTGCCTCCTTTGGCCGATGATGAAGTCACGGATATGATTAATCAAATTTTGGATAATGAGCTTGCCCTTGACCAGCAATTTGGTGACGACCTTCTTGAATTTCCTCAGGTATGTGGTCATAACAAATTTTGTTACTTATTTAGTTTTGATTGCCTAACTTCTACTTTATTTCCATAAATGATCGTCTGAATCAAATTCCTTATGAATCATATAGGTTGTTGCTGAAGAAACACAAAGTACTGTGGTGGATCAGTTCTCTGAGGCAGTGACGGACCCCGAGTACAATGATATTTACCACTCAACCAGTCAGCACTATGATGCGCAGAATGTCAACTTCAATCAGTCGGTTGCATCTCACCTTCATGCCCCGGAAGGATCAGAAGTTATTTCTACTGCCAACATTCAAGTAGAAGACTATAACTTTCAGGAGGATGACTTCTTGGAAATCAACGATCTCAATGGTAGTGAACTTACAATTCCAAATATGGAAACACCAGTGGAGAACCTGCAGTTTGAAGATGGATTGTGTGAACTTGATCTGTTCCAAGATGCAGAGATGTTTCTTCGTGACTTGGGACCAATCAATGAGGAAACCATTCCACATTCATATATGAATAATGCCCCTGGAAGCAACATTGAAAATCAGAATTATCACTTGCTACCCAATCCAGAGGACACTACTCAAAATGTTCATGAATTTTGGATGCATGATGAAAGAAACACCCTGAGTCTGTTTGAAGGCTTTGATGATTCTGTCTCTCAACAAAATCCAGGTATACTGATCAGTGCAAGACACAGTGTTGCTCAACTCCCAAAAAATATTTTTAATGTCTTTGATTTGGATGGAGATGCAAGCTTCACATCTCATTTGTGCTGAGCTCTTCACAGATTACTGCTCCTGGTTGACATGCCAGCTTATGCATGGCAGAAACCATTTAGGAGTCTAAATTTTGTTGCTCTATTGAGAGTTTTACAAACTAAGATTTCTAAATAGGGAAATGTTAAATTTGAGCCTGTTTAAAGCGGCTCTTATGTAAAAATAAAAAATAGGTCCTAGGCTCAGTAGGCTGTCTCTAATATTTGCTAGTTTCTGGTCATGTTTAGGTGCTGTATGTGACTCTGCCAGCTTTCCTACTACTGAAGGCTATGATAATCAAAGTAGCATTGCAGAAGATGTTGCTACAAGTAGATTCTCTTCGGCTCTCTGGTCCTTTGTTGAGTCAATACCTACCACTCCTGCATCAGCTGCGGAAAATGCTCTAGTGAATCGGGCTTTGAATCGAATGTCTAGCTTCAGCCGAGTGAAGATTAATATCAAGCCGACGAACACAGCTGCAGGTAAAGACACTGCAACTACGAAGAGAGTGGGCAGAAAAGGATTTTCATTCCTTTTCTTCCCAATTCTTATTGCTTTATGTGCTTTCTTATGGGTTTCTCTTGGAACTTTTAGATTATTAGGGAGATGCATCGCTCCTTGAATATGTAAATCAAAAGTTTTGATGCTATTATGCTATATATGTTCTATCTGGGATCTCCCCAAGATGACTGAAATAGTATTGTTCTAACATATTCAAAAAATAAAAAATAATATTTTATTCTTTGGGACTGTTAGAAGTTTTAAGTTTAGGCAATATTAGGACTATGGTTGGGAGCAACGGTGGAGAGGGGAGGGTGAGGTGTGGCTGGGACAGGTGTTCGAAAACCGTATTTCGGGGAGTTTGATGGGGAATGCAAAGCATGGCATAGGAAGGGCATGTGGGAACCCTTGATCATGACCCCTCTCCAAATTGGAGGGGTGGATACAGAACACTTAGCTGTCCCTCCCAGGCCTTCTTCATCTTCCAAACAATAAATCACCAGCCATTCCTCTCCACTCTTCCACTTCATCAAGGACCCTTCCCCTCAACCCCCTCCATACTTCCAAACGTAGCCTAGGTGAGTCTTAACAATCTTAATCCAAGAGAAAGGGGTTTATATGAATTTGCAGGGTGGATTGGTAATGCACCAAGTTATTTATTATTTTTGTTTATTTATCGGACCCATGTAAATATCTTGTTTGGGTTTTTAAGTCAGACAGCACTACCATTGGATGTTGATTGTACATTGTAAAGCTGAACCCTTTGTGAGATATCGTAGTATTGATGTATCTATACCTATACCCTTTAAGACATTTGTAATATTATATGCAATTGATAGTATATATGCCTTGGGCATTATATTTTTGTTGTTAGAAGCACGGTAACCCGGAAAATAAAGCTGTGTGTACTGTGTAGTTCTCGATAGCTGTTGTGTAAGCAATTGATGGTTACTACTCCCTTAACATGGTTGCTAACCCGAAATGCGTTTGCGGTATGCTTTGTATTTTTTGACAATCGAGATATTTCATATGGCTTAACACTCGAAAATTCTAAGCTTGAAAGGATATGCAATGCGCATACCATAATTTCGGATGATTGGTATGCTCCCATGAGGAAGGAATCTGTCTCCTTGCCTTCTGATCACTCAAAAGTCAAAACTACAATCCCTTTCTGTACGGACTAGCTTAAAAAACTCGCACCAAAAGTTTATCTAAATTAACATAATCTTGTGAGTTTTATTGACTGGTTGTAATTCTTTTATCAATTAT

>AdNAC24

ATGCCAGGTTTCAGATTTCACCCAACAGATGAAGAGATTGTTGGTTTTTATCTAAAAAGAAAAATTCAGCAAAAATCTCTTCCTATTGAATTGATCAAGCAAGTTGATATCTATAAGTATGAGCCATGGGACCTTCCAAGTAAGAACTAATATCTTTTCTTGTTGAGTTTTTTTGTTAATCTCTTTTATCTGTCTTGGTACATCTTTTTTCACATATATTCGTTTAATCAAGGATGTAAAAGAATAAAAGATAAAAAAAAATTACAAATAGACTATTAAAATACAAACATAAAATTATTCCTCTAATATCTATATGTTCCTTAATAAAAATCAAACCCATTTTTATACTTGCTTCCTAATAATCAATAATGAGTTCTCAAAAATCAAAACTGCACCATCTTTTTCTAAAGATTAAAAAATAGTTAATCTATTTTCTGTTTGTGATATTTTCTTTTTGAGTTATGCTGAAATTCTCAAACAAGTTTTTTTTCCGTTTACATTCTCTTTAAAATAAACATAAATTTAATTAATTTCATACGTTAAAAAAAAATTAATATAATCTAAACATAATTGAATGAAAAAATTCCTTGATTTTGACTTGATTCTTTTCAAATTTTAATAAGCTGATAATTTTTTTAATTTATTTATATATTTTTTTATTAAAAAAGGGGTGGCAAGTAATGGAGAGAAAGAATGGTATTTCTACTGTCCAAGAGACAGGAAATACAGAAACAGTGCAAGGCCCAACAGAGTGACAAGATGTGGGTTTTGGAAGGCCACTGGAACTGACAGGCCCATATACTCATCTGAGGCCCAATCCATTATTGGTTTGAAGAAATCACTTGTTTTCTACAGAGGCAGAGCTGCTAAGGGTTTCAAAACTGATTGGATGATGCATGAGTTTAGGCTCCCTTCACTTTCTTCTGATTCAGCCAAGAAATGCTCTGACAAAACTACCCCTGCTTCTGTAAGTTTTATTTTTTATTTATTTATTTCCAAGGGTTTTATATAGGATATAATGATTAATTTGGTCTGTTAGCTTTTATATATTTAATTAATTTGGTTTTGAATTTTAAAAATATTTGTTTTGGTTTTTGAACTTTTACCTACAAATTAATATCTCTTACATATTTTATTTTAACGCTGCTAAAAAGATATTATTATGCATCGTTAAATAGTATGTGATATAAAATATATTTTTTTAATATTTAAAGATATTTTAAAAAATTCTAAAAATTTTAAAAATAAAAAATATATTTTACCCTTAACCTAAGAATGAGAAATTATTAAAAGTATATTTATACAGTGGAAGTGAATTACAGAAAATTAATTAATGAAATTTGGTTGATTGGAATTTCAGGATTCATGGGCAATATGTAGGATATTCAAGAAAACAAACACAATGTCCATGGCGCAAAAAGCCTCATTACCTCATCATCCTTATAATTGGAATCATCATAATCAATTATTTGATGATATACTCACACATCAACAACACCAACACCAACACCCTATTATTCCAAACTCCAACAACAACTTCATCTTCTACAATTCCAATTCTACCCTTGAACCCACAAAAGAAATTGATGCCACTACTACTAGTAGCTCCATTGTTATTTCTTCCAACATAGGCCTTCATGAAGATCCAAATCATCATCATTACAATAATAATAGTAGTGGGTTCTCATATGATGATGATTCAGGTGTAATTACAACAATTGCTGGGTTCCCATTCAATTTGCCTCCAAATGATGATGATGCTGCTGCTTGGAATAATAATAATAATAAGCCTAATACTACTCTGCCATGGGATTACTCATCAGACATGTCCACTACCTATTCCACTAATAAATCTTACACTTAA

>AiNAC36

ATGCCAGGTTTCAGATTTCACCCAACAGATGAAGAGATTGTTGGTTTTTATCTAAAAAGAAAAATTCAGCAAAAATCTCTTCCTATTGAATTGATCAAGCAAGTTGATATCTATAAGTATGAGCCATGGGACCTTCCAAGTAAGAACTAATATCTTTTCTTGTTGAGTTTTTTTGTTAATCTCTTTTATCTGTTATTATTGTCACCACTTCTTGGCACATCTTTTTTCACATATATTCATTTAATCAAGGATGTAAAGAATAAAAGATAAAAAAAAAATTACAAATAGACTATTAAAATACAAACATAAAATTATTCCTCTAATATCTATATGTTCCTTAATAAAAATCAAACCCATTTTTATACTTGCTTCCTAATAATCAATAATGAGTTCTCAAAAATCAAAACTGCACCATCTTTTTCTAAAGATGAAAAAATAGTTAATCTATTTTCTGTTTGTGATATTTTCTTTTTGAGTGGCCCAACAGAGTGACAAGATGTGGGTTTTGGAAGGCCACTGGAACGGACAGGCCCATTTACTCATCTGAGGCCCAATCCATTATTGGTTTGAAGAAATCACTTGTTTTCTACAGAGGCAGAGCTGCTAAGGGTTTCAAAACTGATTGGATGATGCATGAGTTTAGGCTCCCTTCTCTTTCTTCTGATTCAGCCAAGAAATGCTCTGACAAAACTACCCCTGCTTCTGTAAGTTTATTTTTTTATTTATTTATTTCCAAGGGTTTTATATAGGATATAATAATTTGGTCTGTTAGCTTTTATATATTTGATTAATTTGGTTTTGAATTTTAAAAATATTTGTTTTGGTTTTTGAACTTTTACCTACAAATTAATATCTCCTACATATTTTATTTTGACACTGTTAAAAAGAAAAAAGANNNNNNNNNNNNNNNNNNNNNNNNNNNNNNNNNNNNNNNNNNNNNNNNNNNNNNNNNNNNNNNNNNTTGCCCTTAATCTAAGAATGAGAAATTGTTAAAAGTATATTTATACAATGGAAGTGAATTACAGAAAATTAATTAATGAAATTTGGTTGGTTGGAATTTCAGGATTCATGGGCAATATGTAGGATATTCAAGAAAACAAACACAATGTCCATGGCACAAAAAGCCTCATTATCTCATCATCCTTATAATTGGAATCATCATAATCAATTATTTGATGATATACTCACACATCAACAACACCAACACCCTATTATTCCAAACTCCAACAACAACTTCATCTTCTACAATTCCAATTCTACCCTTGAACCCACAAAAGAAATTGATGCTACTACTACTAGTAGCTCCATTGTTATTTCTTCCAACATAGGCCTTCATGAAGATCCAAATCATCATCATTACAATAATAATAGTAGTGGGTTCTCATCTTCTTCAATTATGATGATGCAACCAAACATCATGGCAACCTCAGATGATGATGATTCAGGTGTAATTACAACAATTGCTGGCTTCCCATTCAATTTGCCTCCAAATGATGATGATGCTGCTTGGAATAATAATATTAAGCCTAATACTACTCTGCCATGGGACTACTTATCAGACATGTCCACTACCTATTCCACTAATAAATCTTACACTTAAAAAAAAATTATATACCATAGTTAATTAAAACCAAAATTTGGATTTTATTTTG

>AiNAC5

ATGGTGGACAGGGATTCAAGCGAAGCACACATGTCAATAGCAGCTTCTTCGATATTCCCTGGCTTTAGGTTCTGTCCCACTGACGAGGAGTTGATCTCTTATTACCTCAGGAAAAAGCTGGACGGTGATGAGGACAGTGTTCAGGTCATTTCGGAGCTTGAGCTTTGCACATTTGAGCCTTGGGATTTGCCAGGTCAGCCTTGTTCTTTCTCTTTGAATGATATATCTACTTATTTCTTGAACCACATAAATATTTATTATAAAAATGTTTGAAAACAGGAAAATCCTTCATTCAATCTGACAACGAGTGGTTTTTCTTCTCGCCACGGGGGAGAAAGTATCCCAATGGTTCACAGAGTAAAAGGGCAACTGAATGTGGATATTGGAAGGCCACAGGGAAAGAACGCGTTGTAAAGTCTGGTCAGAATGTTATTGGTACCAAACGCACTTTGGTATTCCATCTCGGTCGAGCTCCTAAAGGCGAAAGAACTGAATGGATTATGCATGAGTACTGCGTCAATGACAAATCTCAGGTTAATTCGTTGATATTCTTCTTCTGTTGATTGTGTAAGAGAATTCATGTATTATCAAGATTTAAACAAGTCAAAATTTGATTAAGTACCTGTGAATTAGTTAGTTAGAGTGAATCTTGGTTTCATTCTTGAGAGTACATAGCACTTTGCATGTGTTAATTCTCCGTATAAATAACTATGTATAAGACTTGTGTAAACACTAATTTAGCATACATTTTCATAAAAATAATTTATGTACTTCCGATTCACTGGCTACCTTTATATGGCATCAAACCTAGTATTCATATAAGTGCAACAAGTAAATTTTGTGTTTTGAATTAGGATTCATTGGTAATTTGTCGGCTCAAGAGGAATACAGAATTCCGTGCAAGTGATCATTCTAACAGAACTTCACACGATAGTGATTGTGGAGTCTCAGAAGGAGTTACAGTTCAAGGGGACACTTATGTGCCTATTCAAGATAAAGAGACTGGATGCAGCTCCAAGAGGACTAGCAGTAGTAATAGTTCTCCATCTACTACTGGCCAAATTGAATCCAGTCATAGAGTTGCCAATGAAGGCAATGAAGCCAATCAAGCAAATGAAGCCAATGTAGCCAATGAAATCAATCAAGTCAATGAAGCCATTGAAGCTAATGTAGCCAATATAGCCAAGGAAGCCAATCAAGCCAATGAAGTCAGTGTAGCCAATGAAGTTAATATAGCCAATGAAGCCAATCAAGTCAATCAAGCCAATGAAGCCAATGTAGCCAATCCCAGAAATCACCCTCAGGTAACTTGAACAATTGTATAAGAAAAACAGAAAAATGATTTGATATATTCTGATGTGTAGTTGGAAAGTGTGATGGAATTGAATAAAATTATGGTAACATGTAGGTGAATGAAGAAGAGGATTGTTATGCAGAGATCCTAAATGATGATATCATCAAGTTAGATGAATCAACACTCTCACGGCCATCGCCACCACAAGGGACAGCAAACAGGAGAATCAGGCTAAGGGTTCCCAAATCAACAGTTCCCAAATCAAGGGTTCCCAAATCAAGGGTTCCAACGGGAAATGGTTGTCAATGTTCCAAGCAATCATCAAACAAAATTAACACCTTCCTGTCATACCCTTTGGTGGTCTTCACTTTCTTCGTTTTCACTTTGTTAGCTCTAGGCTTCTTTTTTATTAGGAGGTCCCAAACTACTGCCCAATATTCTCAAGACCTCTCTAGAGTTTAATTATGACTATTAATTAATTAGTGTTTAGCGTTGCATAGCCTTTGGATCAATGTTACGTTGTATACAAAATTACAAACAAATGATGCACTCTTCATAGTAAAATAGTAAGGATAAGGA

>AiNAC50

CTGAAGTTAAGGAAACAGTAGGAGAGGTGTGAGAGTGAGGATGAATATAAAAGCGGAAGCACGGACTAGTTGCCATCGAAGTGTACATGCTAATGACACGTTAAAAGAAAAATAATGGCATCAAACATGCTTGGTTCCCTCGCCGACCCTGTTTCACTGAAAGTCCAAAGGCCTAAATTGCCATACTAAATTGTTGTCACTCACCAACAAACAAAGAACAGAACAACATAACCCTCTCTAACACCAAACCCTTTTACTTTCACCTTAAAATCATGGTGGATAGGGATTCAAGTGAAGCACACATGTCAATAGCCGCTTCTTCCATGTTCCCTGGCTTCAGGTTCTGTCCCACGGACGGTGAATTAATCTCTTATTACCTCAGAAAAAAATTGGACGGTGACGAGGACAGTGTTCAGATTATTTCGGAGCTTGAGCTTTGCACCTTTGAGCCTTGGGATTTGCCTGGTCAGCTTTATTCTTTCTTTTTTGAATGATGTTGTTGCTTTGTTTCTTGAGCCACATAAATATTTATTATAAAAATGTTTGAAAACAGAAAAATCGTTCATTAAATCAAATGATGAGTGGTTTTTCTTCTCGCGACGGTGGAGAAGGTATCCCAATGGTTCACAGAATAAAAGGGCAACTAAAAGTGGTTATTGGAAGGTCACAGGAAATGAGCGACAGATAGAGTCTGGTCAGAATGTGATTGGTACCAAACGTACTTTGGTATTCCATGTCGGTCGAGCTCCCAAAGGCGAAAGAACTGAATGGATTATTCATGAATACTGTATCAATGACAAATTTCAGGTTAATTGATTCACATATTCTCCTTCCACTGATTGTGTAAGAGAGTACATGTATTATCAAGATTTAAACTAAGTCAAAATCTATTTAGGTAGCTTTGAATTAGTTCGTTAGAGTGAATCTTGGTTTAATTCTTGGGAGTACATAGCACTTTGCATGTGTCAATTCTCCTTATAACTAGCTTTGTTCATTCTTGGGAGTACATAGCATTTAGCATATATTTTCATAAAAACAATACCTTTACATGGCATAAATATGATATTTATGTAAGTCCAGTAACTAGTAAGTAACACTTTGTGTCGAATCAGGATTCTTTGGTGGTTTGTCGGCTCAAGAGGAACACAAAATTTCGTGCAAATGATGATTCTAACAGAACTTCACGCGAGAGTGGTTGTGGAGTCTCAGAAGGGGTTACAGTTCAAATGAGCACTTGTGTGCCTATTCAAGATAAAGAGGTTGGGTGTAGTTCCAAGAGGAGTAACAATAGTAATAGTTCTCCTTCTATTACTGTCCAAATTAAATCCAGTGATAGAGTTGCCAATGAAGACAATCCCAAAGCTTCTTCCAATGAATCCAGTGATAGAGTTGCCAATGAAGCCAATCCCAAAGATAGAGTTGCCAATGAAGCCAATCCCAAAGCTTCTTCCAATGAATCCAGTGATAGAGTTGCCAACGAAGCCAATCCCAAAGCTTCTTCCAATCACTCTAAGGTAACTTGAACAATTGCATGAGGAAAACAGAGAAATTACTTTATATGTTGTGATGTGTGGTTAAAAAGTGTGATGGAATTGAATGAGATTGTGATAACATGCAGGTGGATGAAGTGGATTATTATGCAGAGATCAACTTAGATGATATCATCAACTTAGATGAACCAGCACTCTAA

>AdNAC55

CAGAAATTAAGGAAACAATGGGAGAAGTGTGAGAGTGGGGATGAAGATAAAAGCGGAAGCACGGACTAGTTGCGATCGAAGTGTACATGCGAATGACACGTTAAAAGAATGGCATCAAACATGCTTGGTTCCCTCAAAGTACAAAAGACCCAATTGTTGTCACTCACCAATAAACAAAGAACAGAACAACATATCTCTCTAACACCAAACCCTTTTACTTTCACCTTAGAATCATGGTGGATAGGGATTCAAGCGGAGCACACATGTCAATAGCAGCTTCTTCCATGTTTCCTGGCTTTAGGTTCTGTCCCACTGACCATGAGTTGATCTCTTATTACCTCAGAAAAAAATTGGACGGTGACGAGGACAGTGTTCAGATTATTTCCGAGCTTGAACTTTGCACCTTTGAGCCTTGGGATTTGCCTGGTCAGTCTTATTCTTTCTTTTTGAATGATGTTGTTGCTTGTTTCTTGAGCCACGTAAATATTTATTATAAAAATGTTTGAAAACAGAAAAATCGTTCATTAAATCAAACGATGAGTGGTTTTTTTTCTCGCGACGAGGGAGAAAGTATCCTAATGGTTCACAAAATAAAAGGGCAACTAAACATGGGTATTGGAGGGTCACATGCAATGAACGACAGATAAAGTCTGGTCAAAATGTGATTGGTACCAAACGCACCTTGGTATTCCATGTCGGCCGAGCTCCTAAAGGCCAGAGAACTGAATGGATTATTCATGAGTACTGCATCAATGACAAATTTCAGGTTAATTGATTCACATATTCTCCTTCCGTTGATTGTGTAAGAGAGTACATGTATTATCAAGATTTAGACTACATCAAATCAAAATCTATTTAGGTAGTTTTGAATTAGTTGGTTAGAGTGAATCTTGGTTTAATTCTTGGGAGTACATAGCACTTTACAAGTGTCAATTCTCCTTATAACTAGCTTTGTTCATTCTTGGGAGTACATAGTATTTAGCATATATTTTCATAAAGACAATTTCACTACTTTCGGTTCACTAGTTACCTTTACATGGCATAAATTTGATATTTACTTAAGTACAATAAGTAACACTTTTGTGTCTCGAATCAGGATTCTTTGGTGGTTTGTCGGCTCAAGAGAAACACAAAATTCCATGCGAGTGATAGTTCTAACAAAGCTTTACGCAAGAGTGGTGGTGGAGTCTCAGAAGGGGTTACAGTTCAAAGGAGCACTTGTGTGCCTATTCAAGATCGTTCTAACAAAACTTCATGCAAGAGTAGTGCTTCACGCAAGAGTAGTTGTGGAGTCTCGGAAGGGATTACAATTCAAAGGAGCACTTGTGCGCCTATTCAAGATCGTTCTAACAAAGCTTCACGCAAGAGTAGTTCTTCACGCAAGAGTAGTTGTGGAGTTTCGAAAGGGGTTACGGTTCAAAGGAGCACTTGTGTGCTTATTCAAGATCGTATTAACAAAGCTTCACGCAAGAATTGTTGTGGAGTCTCAGAAGAAGGAGTTACAGTTCAAAGGAGCACTTGTGTGCCTATTCAAGATAAAGAGGTTGGATGTAGTTCCAAGAAGGGTAACAATAATAATAGTTCTCCTTCTACTACTGCCCAAATTGAATCCAGTCGTATAGTTGCCAATGAAGCCAATCCCAAAGCTTCTTCCGGTCATTCTAAGGTAACTTGAACAATTGCATGAGAAAAGCAAGGAAATTACTTTATATGTTGTGATGTGTGGTTAGAAAGTGCGATGAAATTGAATGAAATTGTGATAACATGTAGGTGGTGGACGAAGTGGGTTATTATGCAGAGATCAACTTAGTTGATATCATCAACTTAGATGAAACAGCACTCTGACGGCCATAGCCCATAGCCACCACAAGGGACAACAAATAGAGAATCACGCGGTTTCTAGATCAAAGGTTTCAACACAACTTCTTGACCAATGCTCCAACCAATCATCAAACAAAATCAACACTTTCCTTTCCTGCTATACGGTTTGCTGCTCTTCACTTTCTTCGTTTGCACTTTACTAGCTCTAAGCTTTTTTCTTATCGGGAAGTCTCAAACAACTGTATAATATTCTCCAGACCTCTCTCAGAGTTTAATTATGATTATTAATTAGTTAAAA

>AiNAC10

CAGAAGTTAAGGAAACAGTGTGAGAGGTGTGAGAGTGGGGATGAAGATAAAAGCGGAAGCACGGACTAGTTGCGATCGAAGTACTTGCTAATGACACGTTAAAAGAAAAATAATGGCATCAAACATGCTTGGTTCCCTCGCCGACCCTATTCCACTCAAAGACCAAACGCCTAAATTGCCATACTAAATTGTTGTCACTCACCAACAAACAAAGAACAGAACAACATAACTCTCTCTAACACCAAACCCTTTTACTTTCACCTTAGAATCATGGTGGATAGGGATTCAAGTGAAGCACACATGTCAATAGCCGCTTCTTCCATGTTCCCTGGCTTCAAGTTTTGTCCCACTGACGGTGAATTAATCTCTTATTACCTCAGAAAAAAATTGGACGGTGACGAGGACAGTGTTCAGATCATTTCGGAGCTTGAGCTTTGCACCTTTGAGCCTTGGGATTTGCCTGGTCAGCCTTATTCTTTCTTTTTTGAATGATGTTGTTGTTTGTTTCTTGAGCCACATAAATATTTATTATAAGAATGTTTGAAAACAGAAAAATCTTTCATTAAATCAAACGATGAGTGGTTTTTTTTCTCGCGACGGGGGAGAAAGTATCCGAATAGTTCACAAAACAAAAGGGCAACTAAAAGTGGGTATTGGAAGGTCACAGGAAAGGAGCGACAGATAGAGTCCGGTCAGAATGTGATTGGTACCAGACGCACTTTGGTATTCCATGTCGGTCGAGTTCCTAAAGGCGAAAGAACTGAATGGATTATTCATGAGTACTGCATCAATGACAAATTTCAGGTTAATTGATTCACATTTTCTCCTTCCACTGATTGTGTAAGAGAGTACATGTATTATCAAGATTCTATTTAGGTAGCTTTGAATTAGTTCGTTAGAGTGAATCTTGGTTTAATTCTTGGGAGTACATAGCACTTTGCATGCGTCAATTCTCCTTGTAACTAGCTTTGTTCATTCTTGGGAGTACATAGCATTTAGCATATATTTTCATAAAAACAATACTTTTACATGCATAAATCTGATATTTATGTAAGTCCAGTAACCAGTAAGTGACACTTTATGTAGAATCAGGATTCTTTGGTGGTTTGTCGGCTCAAGAAGAACACAAAATTTCATACAAATGATGATTCTAACAAAGCTTCACGCGAGAGTGGTTGTGGAGTCTCAGAAGGGGGTTACAGTTCAAAGGAGCACTTGTGTGCCTATTCAAGATAAAGAGGTTGGGTGTAGTTCCAAGAGGAGTAACAATAGTAATAGTTCTCCTTCTATTACTGTCCAAATTGAATCCAGTAATAGAGTTGCTAATGAAGTCAATCCCAAAGCTTCTTCCAATGAATCCAGTGATAGAGTTGCCAATGAAGCCAATCCCAAAGCTTCTTCCAATCACTCTAAGGTAACTTGAACAATTGAATGAGAAAAACAGAGAAATTACTTTATATGTTGTGATGTGTGGTTAGAAAGTGTGATGGAATTGAATGAGATTGTGATAACATGTAGGTGGATGAAGTGGATTATTATGCAGAGATCAACTTAGATGATATCATCAACTTAGATGAACCAGCACTCTGACTGCCATAGCCCATAGCCACCACAAGGGACAGCAAATAGAGATCAACAATCATCAAACAAAATCAAAGGTTTCAACGGAACTTCTTGACCAATGCTCCAACCAATCATCAAACAAAATTAACACTTTCCTGCCATACCGTTTAATGCTCTTCACTTTCTTTGTTTTCACTTTACTAGCTCTTAAGCTTTATCCTTATCGGGAGGTCTCAAACTGCTGTATAATATTCTCCAG

>AdNAC78

AACTTGTGGAATTGAAAGGGCCGGGTCTGGAAAAATCAAAAGAAATGACATGGTGCAATAGATCATCAGTTGTGGAGAGGGGAATCGAAATAATCAACCACCCTAATCTCAATATTATTGCAATCCCTAGAAATAGTAATGACAATAATAATAGTGTTATTAGTGTTACTCATCACGCAAATACTCCTCCTAAACCAACCGAAATCCGAGCCGTTACTTGCCCCTCTTGTGGTCATAACATTCAAATACAACAAGATCAGGTACTCATATCATTAATTAATCCCAAGATTTGCTTCGTTATATCTTATGAAATTAAATGTTTTGATAACACATACAGGGTGGGGGAATTCAAGACTTGCCAGGATTGCCAGCTGGAGTGAAATTTGATCCGAATGACCAAGAGATACTGGAGCATTTGGATGCAAAAGTGCAGTCTGATGTGAGAAAGCTTCATCCTCTAATTGATGAGTTCATACCAACTCTTGAGGGCGAGAATGGAATTTGCTATACTCACCCAGAGAAGCTTCCAGGTTCAATTTCTCATTCTTTTCTTCTTCTTTTTTTAAGTAATATTGATTAATTATTTTTCTGGTGATGGCTTATTTAATTAATTTGGTAATAATAATACCATCATGATGCATAGGTTACGTTACTTTCATCACTGAAATTCTAATTTATTCGGAGACATCAGATTGACGTGCTGCATGAAATGAGGATTATTTAGATGAGAATGACTGCCAAGTGCCAACACATTTATTCATATCTCTAAAACGATATATTAATACTAATGATATTATCATAATAAATTTCTGTTTATAAATGCACTTTCAATCATGTTAAAGCACTTGTTGCGGTATAAATTCTAAAAGTGATCTCAAATTATATATAATAAAGGGGAGGTAGGGGTCTTGGTTTGTGAAAATTAAGAAGACACCGGGGTTAGACATGTTTATCCGTCTAATAGAAATGATGATGCATGTCTCTTTAGCTTAGCCTTTTAAGTTGTTCCTTTTAATTATATATGTGTGTCATTCTGAGAGATGTTATATGATTATTAGGGGTATGTAAGGAGTAAATAGGTGGAAGAATCACATCTTTTAACCTACTCGTTCATGTATGTTACAAGGAAATGGAGATGCTTTTTAATTAGTACAGTTGTCAATGACTACCAAACTCAATGGTGGCATGATATCTCATGCATGTAGCAAAATGAAATAATGTAAATAAATGTGAGAATTTATTAGATGCAACCTCATCATAATAAGAAAGATAACATTCTCAATGAATCATAGTTCAATATTTAAGCTATATTATATATGATACATGATAGAGTTCAATGACATTTGATCTATGTAGTCGATCCTACTTAGTGTGTTAAGACTTTGTTTTAGTTTTAATTTGCAACAAACAATGTCTTGAGTTATCATACAATAGAAAATTGTTTTGTAACAATGTATGTACACTTAAAAAATTAGATGATATGAATAAATCGTCATTTAACACTACTTTACTAGTTATATATATAGTAGGAGGATTATTTATACGAAATTGATCTACTGAACAACCATTAAAGAAGAGAGGGTTATTTTTCTGTATGTTAAGAATGTATATTTAAAAGATGATGTTTAAATATGTTATAGTTTGTTTACCAATTCTTCATCTATAACAAAATATATTAAAAGTAAATAGTTAGGGTAGTGACTGCAATTCATACAAAATTCATATGTCAATTTATTAATAGAATGGAGGATTATTTCTCTGTTACTAAAACATTTAAGAATAATGCCTTAAGTAATTAACGATTCTGAACCTTAAATGGTATAAATAATAAGCAATGTTATATGATCAATAAAATCGATTATCTTTTATTTAATAATGCTGGTTAAGAATTATTTATTTATATTTTTTATTTAATTATATTAAATATATACTAAAATCAGCTACTAATATAAAATATATGTTAAAATATAAATACATCTTAAAAATAAATTAAATAACACATATATTTAATTTATACATAAATATATTGATATAAAATATTAACTAATATTAATAAAAAATATTAGTCTCTCATCATCTTTTATAAATAATACATGATAATGAGAAATTAAGATGATTTTGTAAAAGAGAAATTTGTTGGTATTTTGTGATGAACAGTTGAGAATTTTTCAAATGAAAACTAATATTTGAATTGAAAGNTAACAATATTGAATCTAACTAACTTAGCTAGTTATAAGTGTTGAGAAGGATTTTAACAACTGATATATATAACTACAAAAATGTTATTTATATACTAAAATCAATCATTATAATCAGTTATTAATGTATTTGTATATAAATATATGTAATTTAATTTATTTTAAATATATTTATATTTTAGCATATATTTTATATTAGTGGCTGATTTTGGTATACGCGGCATAATTTATATAACCATCTTCCTTCTCCCAATCCGTTGTGTTAATGCTAAAGAAAAAGTTTTAGAAACTAATATTTGAGGCTGAATATTATGCTATAGTATTAAGTAGCTAAGCAATAATTAGTGTAATGTTAATAAAGGTTGGATGAATTGATATTATTGGTTGGCACAGGAGTAAGCAAAGATGGACAAGTGCGTCACTTCTTTCACAGGCCTTCAAAAGCATACACAACGGGAACAAGGAAGAGAAGAAAGGTTCACACCGACCAAGAAGGAAGCGAGACTAGGTGGCACAAAACTGGCAAAACCAGACCCATCTCTGTGGCTGGTTCCGTTAAGGGTTTCAAGAAGATTCTTGTTCTCTACACCAACTATGGCAGGCAGAAGAAGCCCGAGAAGACCAACTGGGTCATGCATCAGTACCATCTCGGCTCCAACGAAGAAGAAAAAGACGGCGAGCTCGTCGTTTCTAAGGTTTTCTATCAAACACAGCCTCGACAATGTGCAAATAAGGATCCTTATGATGAAAGATTATTGATGACTTCACAAATCAACAGTGTCAATGACATTAGCATGCACGCACTACCCAAGAACAATGCAGGTTTTGTGGATTATTATAACCCCGGTTTCATGAATATGAATTATGAACAGATGAACGAGACTACCTCACCGCAACTGATTCCGAATATGGTGGTGCAAGGTGACAGCTCTTCTTTCATTCGGTTAGCCATGGATGCAAACAAACCCAGGTTGGACAGAAAGTAGTATGACAATGACTGCATGTATGCATGTTGATGTTTTTTTTAATGCC

>AiNAC43

GGGAGCTGGTGATCATCGAAAGGGCCGGGTCTGGAAAAATCAAAAGAAATGACATGGTGCAATAGATCATCAGTTGTGGAGGGGGGAATCGAAATAATCAACCACCCCAATCTCAATATTATTGCAATCCCTAGAAATAGTAATGACAATAATAATAGTGTTATTAGTGTTAATCACGCAAATACTCCTCCTAAACCAACCGAAATCCGAGCCGTTACTTGCCCCTCTTGTGGTCATCACATTCAAATACAACAAGATCAGGTACTCATATCATTAATCCCAAGATTTGCTTCGTTATATCTTATGAAATTAAATGTTTTGATAACACATACAGGGTGGGGGAATTCAAGACTTGCCAGGATTGCCAGCTGGAGTGAAATTTGATCCGAATGACCAAGAGATACTGGAGCATTTGGATGCAAAAGTGCAGTCTGATGTGAGAAAGCTTCATCCTCTAATTGATGAGTTCATACCAACTCTTGAGGGCGAGAATGGAATTTGCTATACTCACCCAGAGAAGCTTCCAGGTTCAATTTCCCATTCTTTCCTTCTTCTTTTTTTTTTTTTTTTTTTTTAAAATAATATTGATTAATTATTTTTCTTGTGATGGCTTATTTAATTAATTTGGTAATAATAATACCATCATGATGCATAGGTTACGTTACTTTCATCATTGAAATTCTAATTTATTCGGAGACATCAGATTGATGTCCTGCATGAAATGGGGATTATTTAGCTGAGAATGACTGCCAACACATTTATTCATATCTCTAAAACGATATATTAATACTAATGATATTATCATTAATAAATTTCTGTTTATAAATGCACTTTCAATCATGTTAAGGCACTTGTTGCGGAATAAATTCTAAAAATGATCTCAAATTATATATAATAAAGGGGAGGGTCTTGGTTTAATTTGTGAAAATTAAGGAAGACACTGGGGTTAGACATGTTTATCCGTCTAATAGAAATGATGATGCATGTCTATTCTTTAGCTTAGCCTTTTAAGTTGTTCCTGCCTCCTTTTAATTATATATGTGTGTCATTCGGAGAGATGTTATATGATTATTAGGGGTAGTAAGGAGTAAATAGGTGGAAGAATCACATCTTTTAACCTACTCGTTCATGTATGTTACAAGGAAATGGAGATGCTTTTTAATTAGTACAGTTGTCAATGACTACCAAACTCAATGGTGGCATGATATCTCATGCATGTAGCAAAATGAATAATGTAAATAAATGTGAGAATTTATTAGATGCAACCTCATCATCATAATAAGAAAGATAACATTCTCAATGAATCATAGTTCAATATTTAAGCTATATTATATGATATATGGTAGAGTTCAATAATATTATTTGATCCATATAATTGACTCTACTTAGTGTGATAAGACTTTATTGTGGTTTTAATTTGTAATAAACAATGTCTTGAGTTATCCATACAATAGAAAATTGTTTTGTAAGAATGTATGTACACTTAAAAAATTAGATGATGTGAATAAATCGTCATTTAACACTTTACTAGTTATATATATAGTAGGAGGATTATTTATACGAAATTGATCTACTGAACAACCATTAAAGAAGAGTGGGTTATTTTTCTGTATGTTAAGAATGTATATTTAAAAGATGATGTTTAAATATGTTATAGTTTGTTTACCAATTCTTCATCTATAACAAAATATATTAAAAGTAAATAGTTAGAGTAGTGACTGCAATTCATACAAAATTCATATGTCAATTTATTAATAGAATGGAGGATTATTTCTCTGTTACTAAAACATTTAAGAATAATGCCTTAAGTAATTAACATTCTGAACTTTAATTTAAATGGTATAAATAAATAATAAACAATGTTATATGATCAATAAAATTGATTATCTTTTATTTAATAATGCTGGTTNNNNNNNNNNNNNNNNNNNNNNNNNNNNNNNNNNNNNNNNNNNNNNNNNNNNNNNNNNNNNNNNNNNNNNNNNNNNNNNNNNNNNNNNNNNNNNNNNNNNNNNNNNNNNNNNNNNNNNNNNNNNNNNNNNNNNNNNNNNNNNNNNNNNNNNNNNNNNNNNNNNNNNNNNNNNNNNNNNNNNNNNNNNNNNNNNNNNNNNNNNNNNNNNNNNNNNNNNNNNNNNNNNNNNNNNNNNNNNNNNNNNNNNNNNNNNNNNNNNNNNNNNNNNNNNNNNNNNNNNNNNNNNNNNNNNNNNNNNNNNNNNNNNNNNNNNNNNNNNNNNNNNNNNNNNNNNNNNNNNNNNNNNNNNNNNNNNNNNNNNNNNNNNNNNNNNNNNNNNNNNNNNNNNNNNNNNNNNNNNNNNNNNNNNNNNNNNNNNNNNNNNNNNNNNNNNNNNNNNNNNNNNNNNNNNNNNNNNNNNNNNNNNNNNNNNNNNNNNNNNNNNNNNNNNNNNNNNNNNNNNNNNNNNNNNNNNNNNNNNNNNNNNNNNNNNNNNNNNNNNNNNNNNNNNNNNNNNNTATTTTTTTATTTAATTATATTAAATATATACTAAAATCAGTTATTAATATAAAATATATATTAAAATATAAATATATCTTATAAATTAAATAACACATATATTTAATTTAGAGGAGTGCTAGGGGGTCAGTAATTTTTGTGTTTTGTAATCATCAATTGGCCATCAATAGTGTTTTTAATAGTGTGAGATTAAATCTAATGGTGTAAGATCATTCAATTTTCTTTTGATAGTTAAATGTTGGCCAACTTTTAAAAAGTTGCTGGCCCTAGACTTTTCCATTATGAATAATACAAGATAATGAGAAATTAAGATGATTTTGTAAAAGAGAATTTTGTTGGTATTTTGTGATATATAGTTTAATTTATTTTTAATGTGTTTATATTTTAGCATATATTTTATATTAGTGGCTGATTTTGGTAACCAGCTATCATAACTCATATAGAAAAAGTTTTAGAAACTAATATTTCAGGCTGAATATTATGCTATAGTATTAAGTAGCTAAGCAATAATTAGTGTAATGTTAATAAAGGTTGGATGAATTGATATTATTGGTTGGCACAGGAGTAAGCAAAGATGGACAAGTGCGTCACTTCTTTCACAGGCCTTCAAAAGCATACACAACGGGAACAAGGAAGAGAAGAAAGGTTCACACCGACCAAGAAGGAAGCGAGACTAGGTGGCACAAAACTGGCAAAACCAGACCCATCTCTGTGGCTGGTTCCGTTAAGGGTTTCAAGAAGATTCTTGTTCTCTACACCAACTATGGCAGGCAGAAGAAGCCCGAGAAGACCAACTGGGTCATGCATCAGTACCATCTCGGCTCCAACGAAGAAGAGAAAGACGGCGAACTCGTCGTTTCTAAGGTTTTCTATCAAACACAACCTCGACAGTGTGCAAACAAGGATCCTTATGATGAAAGATTATTGATGACTTCACAAATTAACAGTGTCAATGACATTAGCATTCACGCACTACCCAAGAACAACAACAACAACAATGCAGGTTTTGTGGATTATTATAACCCCGGTTTCATGAATATGAATTATGAACAGATGAACGAGACTACCTCACCGCAACTGATTCCGAATATGGTGGTGCAAGGTGACAGCTCTTCTTTCATTCGGTTACCCAGGTTGGACAGAAAGTAGTATGACAATGACTGCATGCATGTTGATGTTTTTTTAATGCC

>AiNAC47

TCAGCTAGAGTTGAAGCTTCTCGAGAAATCAAGAACAAGAAGAAGAAGGAGGAATAATGCAAGGTGGATTAGAGTTACCGCCAGGGTTCAGGTTTCACCCGAGCGACGAGGAATTGGTGAACCACTATCTCTGCAAGAAATGCGCAAAGCAATCAATTGCTGCTCCAATAATTAAGGAAATCGATTTGTACAAGTTCGATCCGTGGCAGCTTCCAGGTACTTAAACGCTCGATTTCTGTTTTCAGTGGATATTTATTCGTAAGAATTCGAGTTTGACTTATTTATTGATTGCAGAGATGGCGTTGTACGGAGAGAAGGAGTGGTACTTCTTTTCGCCGAGGGATAGGAAATATCCGAACGGATCCCGGCCGAACCGGGCGGCTGGGAGCGGGTACTGGAAGGCGACCGGGGCGGACAAGCCGATAGGGAAGCCGAAGGCGCTAGGGATAAAGAAAGCGCTAGTGTTCTACGCCGGAAAGGCCCCGAAAGGAGTGAAGACTAATTGGATTATGCATGAGTACCGTCTCGCCAATGTTGACAGATCCGCCGCCAACAAACTCAACAACAACAACTTGAGGGTATTAACTACTAGAACCTTCTCTTCAAACCTTTTTTTTACCCTAATTTATTTTATTTAATAATAATAATAATAATAATTAAGGTTTTTGATTACAAAAATTGGTTGTGGAGAGAGGTTGATATTTAAATTCTTAGTATTAGAATTATTTTTTAAAAATAATTTGAGCATAACTGTTTTTTAGTTTTTATTGGATCCAATTAACACATTCTTTTAATTTTTTTTTATTCTATATGGGTGGTTCATTTGGGGAATATTTTGTGTGCATCTAGTCTGGTTATTTATAATATCATTTGATCAAAGTATTTGTGATGCAAGATTTTTGAAGTACTGTTGATGAGAATCAACTTTTCTTTTAGTATGATCTGATTGGAATATGTTTTGGTGCAGCTTGATGATTGGGTGTTGTGTCGAATCTACAACAAGAAAGGGAAGATTGAGAAATTCAACTCTGCCACAACAGGGTTGGAACAGAAACTACCAAAGTTTTCACCAGGGGAGATACTTCACTATGATCATGATCATGAGCATGAGACAAAGCCAAAGATTATCCACAATTTCTCCAACAATGAGCACCAATTGTACATGGACACATCAGATTCCGTTCCAAGGCTGCACACGGACTCTAGCTGCTCGGATCACGCGGTTTCGCCGGACGCCACCTGCGACAAGGAGGTGGAGAGCAACCCAAAGTGGAGCAATGAGCTAGATATGCAGCTGTTTGATACCTTTGATTTTCAGCTCAACAACTATGATAATAGCCTCCCAATGAATGATGATGACCTTTTTGGAAATCAGTTCCAAATGAATCAGCTCATGTCTTTCCAAGACACATTCTTGTTCCCACAAAAGCCATTTTGATTCTTTTGACCATTAAGAATTATTGGGAATTGGAAGAAGAGGGGGAGAAAAAAAGTTGAACCTTTTTTTCTGGGAAAATGTGGTGGAGGACTAGTGCCTAATGAGATCATGGGACCAAAGCATATGTTCATTTTTTGTAGTGAAAACTCAGCATTTAACACAATTCACACCTACCTAACAATTTCACTTTTGGACCATTGGATTTTGGTGGCTATTCTTTGTTCTTCGGAGATTAGGGAGGGAGATGT

>AdNAC15

ATGATGAGCAAGAAGATGAGGTTTGTTAAGAAGAACAAGAATGGAGTGAGATTATTGCCACCTGGATTTAGGTTCCAACCAACAGAAGAGGAGCTTCTATTTCAGTATTTGAAATGCAAGGTTTTCTCTTTTCAGTTGCCAGCTTCAATCATTCCTGAGATCAATGTATGCAACTATGATCCTTGGGATTTGCCAGGTAATCAATATCAAAATCTTTTTTTTTTTTTTAAGTTTTAGTTTCACTCTTATTAAAGTTACCAAATTATAGTGAAGTATATATAATTCCGTTTAGTCTTTGCTTCTTAAAATGTTTAATTTAATAATTTCTTCAAAAAAAGAAAAAGCTAAAGATAGACTAAATTAATTAAATTAAATTGAAACTAAATTGACATTATCTAAGGTTAGAATGAAACTAAGTTAAATTAAAATATTATTCAACTTTTACTCATTTTTTTTTTTTACATATAGCTCTAGGAATAATTAACTGATTAAGGTATTTTTACAATATGCAGTATAAATTCATCATCATTATTGACATATTTTCCATGATTATTGACATATTTTCTATGCAAGAGCTCATTAGTATATGTAATATAAAAATATTACGTGTACATTAAAAATTATCGATTAAAATCAGTCATTATATATTTTATATAAATATATATTGTTGTTAAACTTAATTTTAGTATATATTTTATATTTTAATATACATTATACGTACAGATATAGTAATATCTTTCTATATCTTTTAATTATTACTAAATTGATTAAAAGATGAAGAGAAACAAATATAAATGAACAAAAGCACATTAACATAACATTATTTATTAATGTCATATATATGCTCTTATAACTATGATCTTATTATTTTTTTATTTTATTATTAGGGAATAATAATAATTATGGGGAGGAAGAAGAGAGATACTTGTTCAGCTCAAAGGAAGTTAAGTATAGAAACGGTAACCGAATGAACAGAATAACGAAATCTGGATATTGGAAAGCAACTGGATCAGACAAAAGAATAATTTCAACATCATCCAATAATAATAATAATAATAATATTGTTGGGATAAGAAAAACTCTTGTATTCTATCATGGAAAATCTCCAAATGGCTCTAGAACTCATTGGATCATGCGTGAGTATCGACTTGTCACTACTCCTTCTAATTCATCCCAGGTGATTATTTTTTCAATTATTACTACAAAGTTTGTTTTTACAATTTTTTTCTTTTATAATGGCCTAATATTACACTCATCAATAATATTCTTTGATTTGTTATATTTACACTCGCATTATATTTTACAACATACAAAAAATAAATGGTAGAGATCATTATTAATAAAATATTTATTTATCATCTTTATATAATAGACATACGATTTAATTAATAAGCATAATCAATTAATTTGGTATTACTTATCTAACTAATTAACTTAACATCTAAGTAACATAATTTTTCTTATAACACTAGATTTTATTTTAGCTTGATGGTTATTCTTTAGTTACACTAATCTTTACAAAAATAATAGTAATTAAATTATATATTAACTAAAATAGTAAGATTATTGTGTGTATGGGCATATTTAATTTATGTATGATTGATTTTATGTTGGCAGAAGTATGTAGAAGACTTAGGGAATTGGGTTCTTTGCCGCATATTCAAGAAGAAAAGAAGCATAGAAAGTCAACATCACATGGTCAAGAACAAAATTAATAATAATGTTGTCGAGGTGGCTAATAATAATAATAATAAGCCAATATTCTTTGATTTTATGAGGCTATATGACTCGCCAATATCTTCTTCTTCATCTTCATCTTCTTCTTGTTTAAGTTCTGATTTCATAACTCAAATGTAAGAACATATGTGGTTATATTATGTATGAATTTTGATTAAGTGCGTATATATAAGGTTTAATTACCTTGTAGGTTTTTTATATAGTTTTGTAGAGTTTTCAATAA

>AiNAC23

CCAAGTACTACCGCCGCCTTTATAATTTCCAGCAATCTATCCATCCATTATTATAGTTAGCTTTTGTGAAAAATAATCAACCCGAATCTTTATCTCTCTACTCAGCATCTCAAACTATATATATAGTGACAAAATAAATGGATTATGGAAGTGTAGTTATTACTATACCAAACTCAACAATTCATACACCAATTCTATGATGATGATGACTACTACTTCTACTACTGCAGATTATGAAAGCGTGAAGCAGCTTCCTCCTGGGTTTTTGTTCTCTCCAACGGATGAAGAACTTGTCCTTCACTTTCTCTATGCCAAGGCTTCTCTTTTGCCATGCCATCCCAACATCATCCCTGATCTTGATCTCTCTCTCGCTCATCCTTCCCAACTCAACGGTATCTTTAACCTACTCAATTTCTTATCACCACTTTTCATATAACAACCAAGTTAACACCTTTTACTTTAAACCAAAATAATTTATTTTAAAATTGAGCAGTGTAAATATTTGGCAGATAAAGCGTTGTCAAGCGGAAATCAATACTATTTCTTCAGCAAAGTGAAGGAAAAAAGAATAACAGAAAATGGGTATTGGAAGGAAATAGGTGAAAGTGAAGCAATATTGTCATCAACGGTTGAGAAGAAAGTAGGGACAAAGAAGAACCTTGTATTCCACATAGGAGAAGCTCCACACGGCATTGAAACCAGTTGGGTCATGCAAGAATATCATATTTGCCGATCCTCTAACATTATTTCTACAAGTAGAGCCAGAAGAAAACACGTAAGTCACTACCCACATCCACTATAGGTTTTTTTTCCTTTGTGAAAATTTACTAACATCTAATTAAATTAAAACAGGATCATCAAATTTGGAGCAAATGGGTTTTGTGCAAAGTGTATGAAAAGAAGGGGTCCGTACGAGGTGTAAACTACTGTAGCGACGATGATGACAGTGGGACAGAGCTATCTTGGCTTGACGAAATTTATCTCTCGTTGGATGATGATCTCGAAGAAATTAGCGTCTCCATTTTAGATTGAACTAGTGAAGTACCCGTGCATCGCACGGGTATGATAAGCATAAGGGAAAGTATGAGGAAACAATACACTTATTGTACAATACATACAATGTGGGTGTAATGGAAATTAAAAATAAATAATGGGTTATTAATTAATTTCATATTATTAATAATTTGAAATTTGAAATTTAAAACTATTTAGAATTAATCTTAATTATAGATTCAAATCAAATATTTAGGTTAAAAATAGAATCTCCCTGACGGCATATTCTCCTGGAGTACACCGTTGCCCCATGCGCTGATTTCCACCTTCTCCAGCAGTCAATTACCCATGCCTGCGTCCTGTTGATCGGCTTCTCCCTTCAGCTCCG

>AdNAC65

AGAAAACACAACCACCAAGATTCTCCTCATTCATTCATTCCAACAAAAGGATCTTTACTTAGTTCCTTTTGTTCCTCCCCAAAAAAGAACACTCTTTATTTATTTTCCTTTCAACACTCTTCACCATGGACAGCTTCTACCACCACCATAACCACCACTTTGACAACAGCGACACTCACTTGCCACCTGGATTCCGATTCCACCCCACCGATGAAGAACTCATCACCTACTACCTCGTCAAGAAGGTTCTCGACAACACCTTCACCGGAAGAGCCATAGCTGAAGTTGACCTCAACAAGTGTGAGCCATGGGAGCTCCCTGGTATGTATAACAAGAACTCAAATCAAAATCACTTTTTTATCACACCTCATGTTGTAAATCCATGAAAATGTTGAAACTTTCAGAGAAAGCGAAGATGGGTGAGAAAGAATGGTACTTCTTCAGCTTAAGGGACAGGAAGTACCCAACTGGGTTGCGCACAAATAGAGCGACGGAAGCTGGGTACTGGAAAGCCACTGGAAAAGACAGAGAGATCTACAGCTCCAAGACTTGTGCTCTTGTTGGAATGAAGAAGACACTTGTTTTCTACAGAGGAAGAGCTCCCAAGGGTGAGAAGAGCAACTGGGTGATGCATGAGTATCGGCTTGAAGGCAAATTTGCATACCATTATCTCTCTAGAAGCTCCAAGGTTAGTCATGGAATCGGTAGCTAGATTCTTTGCTTATCATGAGCCGCATGCTAGAGCTATGGAGGCTATAGTAGGGGTGATCGGAAATCTCTGGTTTTATAATTAGGTTCCGTTTATCAATGTTGATTGTTTTTTTCTTGAGGACTCTTGGTTCTTTCATTTGTACTTAGGACTACAACCGCAGGTAGGAGGGTTTCTAAGTTGCTTGCTTTTTTGATTCATATAACTGTTAAAAAGAAGTTTGCATGGGGAGTGTAAACAATGACGCATTAATTGCAGAAAATAAAAGAAAAAATTAGTTGCCAAAAGGTGTGCTACTAATAAGTACTAGTAACATGTATGAAAGAAGCTACTATATTGCATCATGTTATAGGGTTTTGGTTTAGCTTCCATTAATTACACTATTTTCCATAACACTGCTATCAATCTCAAAGATCCTAATCCTATACTATTAGCTTCATGCTCATTCTTCTATTATTCCATTTTTTATACCAACAAATTAATGCTTACAATTATCATTAATTTCAAAAATGTGCTATTTTTTATTTTAGGATGAGTGGGTGATTTCTCGTGTGTTCCAGAAGACAACCACCGGGGGTGGTGGTGGAGGTGGAGGTGGAGGCTCAGCCGTGTCAACCACCGCCGGCGGGTCCAAGAAGGCGAAAATGAGCACTTCCACCACTTCTACAATGAGCTTCTGCCCTGAACCAAGTTCTCCCTCTTCGGTTTACCTTCCACCACTTCTCGACTCTTCACCTTACACTACCGCCACCACCGGCTCCGTCACCTCCGCCGCCGCAGCATACGACGGCCGCCAGAGCTCCTCCTTCGACAACAACAACAACAACAACGATAGCACAAGGGAGCACGTGTCCTGTTTCTCCACAATCTCCAACAACTTTGTCAATGGGTTCTTCGATCTTGCTCCTATGGATTCCTTCGCTCGATTCCAAAGAAACAACAATGTCGGTGTTTCTGCATTCCCAAGTCTAAGGTCGCTGCAAGATAACCTTCAAATTAACCCTTTGTTTTTCTCCGCCGCAGCGGCGCAGCCTCTCCACGGCGGCGAGCTTCACGCCGCGGGGACCTGGCCGGTGCCGGATGATCAGAGGGTTGCCGAGGCTGCTGCCGCCGGCATGGCTTTGGGACATTCCGAGCTTGATTGCATGTGGGGCTATTGA

>AiNAC19

CTCTACCATAAAGAAAAAACACTACTTTCTGTTGCCACTTTCTTCACTGACAAAGGAATACCTGCACACCTTCCTCACACACGCACGCATACTCTCTTTCTCTCTCTCTCTCTCTAGCATTGTGGATTATGATAAGGACCCTGTCAGGGAGAGGAGAGTAGAGAGAGATTTTCAGAATAGGACACTGCACACACAAGAAGAAAACACACACATTTTTCCAACCCCATATCATACAAAGACACCAATAATAATAATAACATGAAGCCCTTATAAACCAAGCACCCTCCTCCATACCAAAAGGCACCACATACAGCAGAACAACAGAAGAAAGAAAAAAGTGATCCAAACCCCATTATTAATCTCCTTTCACTCTTTTGCTCCTTCTGCTTTTTCTTCAAGTTCTTTCCATTCTCATACAATCCAAATGGCCCCTTAAGAAATACAAATAGATCGAAGAAGAAAGAAAACACAACCACCAAGATTCTCCTCATTCATTCATTCCAACAAAAGGATCTTTACTTAGTTCCTTTTGTTCCTCCCCAAAAAAGAACACTCTTTATTTATTTTCCTTTGAACACTCTTCACCATGGACAGCTTCTACCACCACCATAACCACCACTTTGACAACAGCGACACTCACTTGCCACCTGGATTCCGATTCCACCCCACCGATGAAGAACTCATCACCTACTACCTCGTCAAGAAGGTTCTCGACAACACCTTCACCGGAAGAGCCATAGCTGAAGTTGACCTCAACAAGTGTGAGCCATGGGAGCTCCCTGGTATGTATAACAAGAACTCAAATCAAAATCAATTTTTTATCACACCCCATGTTCTAAATCCATGAAAATCTTGAAACTTTCAGAGAAAGCGAAGATGGGTGAGAAAGAATGGTACTTCTTCAGCTTAAGGGACAGGAAGTACCCAACTGGGTTGCGCACAAATAGAGCGACGGAAGCTGGGTACTGGAAAGCCACTGGAAAAGACAGAGAGATCTACAGCTCCAAGACTTGTGCTCTTGTTGGAATGAAGAAGACACTTGTTTTCTACAGAGGAAGAGCTCCCAAGGGTGAGAAGAGCAACTGGGTGATGCATGAGTATCGCCTTGAAGGCAAATTTGCCTACCATTATCTCTCTAGAAGCTCCAAGGTTAGTCATGGAATCGCTAGCTAGATTCTTTGCTTATCATGAGCCGCATGCAATAATAATCATGCTAGAGATATGGAGGCTAGTAGGGGTGATCGGAAATCTCTGGTTTTATAATTTGGTTCCGTTTATCAATGTTGATTGCTTTTTTCTTGAGGACTCTTGGTTCTTTCATTTGTACTTAGGACTACAACCGCAGGTAGGAGGGTTTCTAAGTTGCTTGCTTTTTTGATTCATAAAACTGTTAAAAAGAAGTTTGCATGGGGAGTGTAAACAATGACGCATTAATTGCAGAAAATAAAAGAAAAAATGATAAAAATGTGGGTTGTAATTACTTGCCAAAAGGTGTGCTACTAATAAGTACTAGTAACATGTATCAAAGCAGCTACTATATTGCATCATGTTATAGGGTTTTGGTTTAGCTTCCATTAATTACACTATTTTCCATAACACTGCTATCAATCTGAAAGATCCTAATCCTATACTATTAGCTTCATGCTCATTCTTCTATTATTCCATTACTTCCCATTTTTTATACCAACAAATTAATGCTTACAATTATCATTAATTTCAAAAATGTCCTATTTTTTATTTTAGGATGAGTGGGTGATTTCTCGTGTGTTCCAGAAGACAACCACCGGGGGTGGCGGTGGAGGCGGAGGCTCAGCCGTGTCAACCACCGCCGGCGGGTCCAAGAAGGCAAAAATGAGCACTTCAACCACCACTTCTACAATGAGCTTCTGCCCTGAACCAAGTTCTCCCTCTTCGGTTTACCTTCCACCACTTCTCGACTCCTCACCTTACACTACCGCCACCACCGGCTCCGTCACCTCCGCCGCCGCAGCATACGACGGCCGCCAGAGCTCCTCCTTCGACAACAACAACAACGATAGCACAAGGGAGCACGTGTCCTGTTTCTCCACAATCTCCAACAACTTTGTCAATGGGTTCTTCGATCTTGCTCCTATGGACTCCTTCGCTCGATTCCAAAGAAACAACAATGTCGGTGTTTCTGCATTCCCAAGTCTAAGGTCTCAGCAAGATAACCTTCAAATTAACCCTTTGTTTTTCTCCGCCGCAGCGGCGCAGCCTCTCCACGGCGGCGAGCTTCACGCCGCGGGGGCCTGGCCGGTGCCGGAGGATCAGAGGGTTGCCGAGGCTGCTGCCGCCGGCATGGCTTTGGGACATTCCGAGCTTGATTGCATGTGGGGCTATTGA

>AdNAC75

ATGGAAGAGCTAGCGTGTGAGCTGAGTGATCATGAAAAGAGAAACGCTCAAGGTTTGCCACCCGGTTTCAGGTTTCACCCAACTGATCAAGAACTCATTACCTTCTATTTGGCTTCCAAGGTCTTCAATACTACTACTACTACTACTCATGTCAACTTTGTGGAGGTTGATCTCAATCGATGCGAGCCATGGGAACTTCCAGGTCTGAATTTAATTTAATTTATTCTTCAATCGTAATAAGCTTGGCTGTTTCTTTGTTTAAAAAAGAAAAAATGTGAATGTGATGTTGGTGGATGAAATGGCATGTACGACAGAAGTGGCAAAGATGGGGGAGAGAGAGTGGTATCTGTACAGTGTGAGAGACAGAAAATACCCAACGGGCCTCAGAACTAACAGAGCAACCGCTGCTGGGTACTGGAAGGCTACCGGCAAGGACAAGCAAGTATACGGCGGCGGTGGCCTTGTTGGGATGAAGAAGACGTTGGTGTTCTACAAAGGGAGGGCCCCCCGCGGTCAGAAGACTAAATGGGTAATGCATGAGTTCCGGTTGGACCCTCACAGCTCTCCTTCCCTCTCTAAGGTACATCACTCTTACATTGGCTTCTGTGTACCGTGTGAATCAAGTTATGTCTTCACTTTTGGGTTGACTAATGAATCATTTCCTGTTCCTAAACTTGACCCTTTTTACTCTCTGCTCTTCTTTCTCTTGTCTTGATGTGATCCAATCCATCTTTTCTACCTACTATTCATTCCCACATGTGAATTCCTAGAGAGCCATCTTAATTAATTAATCAGCTTGTTGCCTTGTTTGAGAGATGCAGGATGAGTGGGTAATATGCAGAATATTTCATAAAAGTGGGGAAAAGAGAACTCCTACTACTACTCCTGCTCCTCTGCTACTTCATCATCAACAACACGATCCATCATCACTGTTCAATGACCATATCTCCCACTCTCATAATCATAATCAAAACCTCCTCTCGCCATTGCTTCATCCTTTCCCAATCCCTGAAGAAACCACTAAAACCAAATCATCAACAATTAACAGCAACCATTACCCTCCACCACCACCTTCCTCCCAACACTTGCTTAAGCTCAATAAGTCTACTAAATTAACAAAAACAGTGCCTCCTTCTCCATCATTCTTCCAATACCAACAGCTTCTAGAAGATGATCCCAACTTATTGCATTGGATGGACAGTGGTAATAATAATAATAATAACTGCAAGGCTAATAATACTGCTAGTAGTGTTGAGATAATGGATGCTGCTGCTGCTGGCTTGATAGCATTCTCATCAGGAGGACCTTCACCTACTCCTACTAATAATAATAATAACAATTCTGAAATAATAAGGGACATGATGATGATGTCTTCTTCTTCGGCTTCTATGCTGCACATACTCGACGATGCTCCTCTTGGAATTCAATCTTGGCCTCATCATCATCATCATCACCTTCTGTAA

>AiNAC30

ATGGAAGAGCTAGCGTGTGAGCTGAGTGATCATGAAAAGAGAAACGCTCAAGGTTTGCCACCGGGTTTCAGGTTTCACCCAACTGATCAAGAACTCATTACCTTCTATTTGGCTTCCAAGGTCTTCAATAATACAAATGCTACTACTGCTACTACTACTACTACTCATGTCAACTTTGTGGAGGTTGATCTCAATCGATGCGAGCCATGGGAACTTCCAGGTCTGAATTTAATTTAATTTATTCTTCAATCGTAATAAGCTTGGCTGTAAGTGTTGAGTTCTTTCTTTCTTTAAAAAAGAAAAAATGTGAATGTGATGTTGGTGGATGAAATGGCATGTACGGCAGAAGTGGCAAAGATGGGGGAGAGAGAGTGGTATCTGTACAGTGTGAGAGACAGAAAATACCCAACGGGCCTCAGAACTAACAGAGCAACCGCTGCTGGGTACTGGAAGGCTACCGGCAAGGACAAGCAAGTCTACGGCGGCGGTGGCCTTGTTGGGATGAAGAAGACGTTGGTGTTCTACAAAGGGAGGGCCCCCCGCGGTCAGAAGACTAAATGGGTAATGCATGAGTTCCGGTTGGACCCTCACAGCTCTCCTTCCCTCTCTAAGGTACATACACCCAAAGCAATCACTCTTACTTACATTGGCTTCTGTGTACCGTGTGAATCAAGTTATGTCTTCACTTTTGGGTTGACTAATGAATCATTTCCTGTTCCTAAACTTGACCCTTTTTACTCTCTGCTCTTGTTTCTCTTGTCTTCATGTGATCCAATCCATCTTTTCTACCTACTATTCATTCCCACATGTGAATTCCTAGAGAGCCATCTTAATTAATTAATTGTTTGAGAGATGCAGGATGAGTGGGTAATATGCAGAATATTTCATAAAAGTGGGGAAAAGAGAGCTCCTACTACTACTCCTCCTCCTCTGCTACTTCATCATCAACTGCAGCATCAGCAACAACACGATCCATTATTATTATTCCAAACCCCATCATCCCTGTTCAATGACCATATCTCCCACTCTCATAATCATAATCAAAACCTCCTCTCGCCATTGCTTCATCCTTTCCCAATCCCTGAAGAAACCACTAAAACCAGATCATCAACAATTAACAGCAACCATTACCCTCCACCACCACCTTCCTCCCAACACTCGCTTAAGCTCAACAAGTCTACTAAATTAACAAAAACAGTGCCTCCTTCTCCATCATTCTTCCAATACCAACAGCTTCTAGAAGATTATCCCAACTTATTGCATTGGATCGACAGTGGTAATAACAATAATAATAACTGCAACGCTAATAATACTGCTAGTAGTGTTGAGATAATGGATGCTGCTGCTGCTGGCTTGATAGCATTCTCATCAGGAGGACCTTCACCTACTCCTAATAATAATAATAATAATAATAATAACAATGCTGAAATAATGATGATGTCTTCTTCTTCGGCTTCTATGCTGCACATACTCGACGATGCTCCTCTTGGGATTCAATCTTGGCCTCATCATCATCATCATCACCTTCTGTAA

>AdNAC81

CGGTTCTATGCACCTAAAAACCCAGTATTTAAGGATTCTGTTTCTCATAACATAGATCTTTGTTTTGAGTGTCTCTCAATTGCATTTTTTTTCCCATCAAATTCCTCAAAAAAGAAAAATTAAACCCTCCACACTAAAAAAGAAGAACTGCTCAATTTATTCACTCTCTTAACTTGTCTTCACACCCATGATATATGCATCAAAAAGTGTAGCCACCCTAACCCCAAAACGTTCCTTTTTTAACTCCTTGCAACACACAAGTGTAAAATTGTCCACTACCCTTTAAAGGTTGGTTTCTCTGCATTTAGCCCACTTGAATTCCGGCGCCGAATGTTCGATAGCTGCCAAGTTTAACCGACATATTTGTCGTGTTTCGATGGTTTTTATGAACCAAAGGAGCCTAGCATAGAGATGGAATCAATGGAGAATGTTAGAATGCAAAGAGAGAAAGATCAGAAGTTCGAATTGCCGTCCGGCTTTCGATTTCATCCCACCGATGTAGAGCTCATAAATTACTACCTTGTTAAGAAGGTTCTTGATGATAAGCACTTCTGTTCTATAGCAATTGCTGATGCTGATATGAACAAGTCTGAGCCATGGGATTTACCCGGTAAAACTTTGATACTTTCTCATCATTACATATCTTTTTAGATGGCAATTTTATTACCTAAATTGCTCCCTTTTATTATGTTTAAAGGTTTAGCGAAAATGGGCGAAACAGAATGGTATTTTTTTAGTATGAAGGATAGAAAATACCCAACTGGCCAAAGGACTAATAGGGCGACCGAGGCCGGATATTGGAAGGCCACAGGCAAAGACAAGGAGATATCAAAGGAGAATTCAAAGATTGGGATGAAGAAGACCCTTGTTTTCTACAAAGGAAGAGCTCCAAGAGGTGAAAAGACTAATTGGGTCATGCATGAATATAGATTGGAAGGGAACAAATCTGTTTATAATCTGTCACAACCTGAAAGAGTTAGTATATTTTACTCTGTTTTTGCTCTGTTTTCCTCTGTTTTAATACTCTGTTTTAAAATGAAAATTTTGCTAACAAGTGTTTACCATTCATAGGGTGAATGGGTTATATGCAGAGTATTTGAGAAGGGCAATAATGGAAAAAGACTGAATATTGCAAAGTTGGAGAGGCTCAACTCTTCGGGAAAGGAACCATTACCATTGCCAAAACCTACTCCTTTGATGCCTCCATTGATGGATTCTTCATCATCGAGAACCACCCCCGGCGAGTTATCTCAGGCGACGTGCTACTCCTCGGATCCAAATCAAGCCGATGTCCAGAACAATTTGCATGATGACATAGTTGAAAGCAGGGAAACTCCTATCTTGAACTTTTCCCCTGCTTCCATCAATGAAGAATTAATTCAGATTCCAAACCAAATTGAGAATCCGGATTATTATACTCTGCCTCAAGAAAACAACGGATCAATTGCAAGGCAGAATCAGAAATCAGAGTTTGATGCTGATATATCATCTTTGATTTACAACAATGACATGTTTTACAGGTTCTTTGGGAACCAAGAACATTCATCTTCAGCTTCCGCAGACATTTGCAACCTATGGAATTACTAGATAAGGTTGAAGGATATTAAATAATATTATTACTCTCTCTTTTTTTAAGACAAATTAAAGTAAACATGTCATTTATATCATACCTTTAAATTCTGAATTGATTACCTGCAAATGCATGAACTTATTATTAGGTTGGTAACTTTAATTGAAAAAAAGGTGG

>AiNAC29

CGGTTCTATGCACCTAAAAAACCAGTATTTAAGGATTCTGTTTCTCATAACATAGATCTTTGTTTTGAGTGTCTCTCAATTGCATTTTTTTCCATCAAATTCCTCAAAAAAGAAAAATTAAACCCTCCACACCAAAAAAGAAGAACTGCTCAATTTATTCACTCTCTTAACTTGTCTTCACACCCATGATTTAGGCATCAAAAAGTGTAGCCACCCTAACCCCAAAAGCTTCCTTTTTTAACTCCTTGCAACACACAAGTGTGAAATTGTCCACTACCCTTTAAAGGTTGGTTTCTCTGCATTTAGCCCACTTGAATTCCGGCGCCGAATGTTCGATAGCTGCCGAGTTTAACCGACATATTTGTCGTGTTTCGATGCTTTTTATGAACCAAAGGAGCCTAGCATAGAGATGGAATCAATGGAGAATGTTAGAATGCAAAGAGAGAAAGATCAGAAGTTCGAATTGCCGTCCGGCTTTCGATTTCATCCCACCGATGTAGAGCTCATAAATTACTACCTTGTTAAGAAGGTTCTTGATGATAAGCACTTCTGTTCTATAGCAATTGCTGATGCTGATATGAACAAGTCTGAGCCATGGGATTTACCCGGTAAAACTTTGATACTTTCTCATCATTACATATCTTTTTAGATGGCGATTTTATTACCTAAATTGCTCCCTTTCATTATGTTTAAAGGTTTAGCGAAAATGGGCGAAACGGAATGGTATTTTTTCTCTATGAAGGATAGAAAATACCCAACTGGCCAAAGGACTAATAGGGCGACGGAGGCCGGTTATTGGAAGGCCACAGGCAAAGACAAGGAGATATCAAAGGAGAATTCAAAGATTGGGATGAAGAAGACCCTTGTTTTCTACAAAGGAAGAGCTCCAAGAGGTGAAAAGACTAATTGGGTCATGCATGAATATAGATTGGAAGGGAACAAATCTGTTTATAATCTGTCACAACCTGAAAGAGTTAGTATATTTTACTCTGTTTTTGCTCTGTTTTCCTCTGTTTTCCTCTGTTTTAATACTCTGTTTTAAAATGAAAATTTTGCTAACAAGTGTTTACCATTCATAGGGTGAATGGGTTATATGCAGAGTATTTGAGAAGGGCAATCATGGAAAAAGACTGAATATTGCAAAGTTGGAGAGGCTCAACTCTTTGGGAGAGGAACCATTACCATTGCCAAAACCTACTCCTTTGATGCCTCCATTGATGGATTCTTCATCATCGAGAACCACCCCCGGCGAGTTATCTCAGGCGACGTGCTACTCCTCTGATCCGAATCAAGCCGAAGTCCGGAACAATTTGCATGATGACATAGTTGAAAGCAGGGAAACTCCTATCTTGAACTTTTCCCCTGCTTCCATCAATGAAGAATTTTTTCAGATTCCCAACCAAATTGAGAAACCGGATTGTTATACTCCGCCTCAAGAAAACAACGGATCAATTGCAAGGCAGAATCAGAAATCAGAGTTTGATGCTGATATATCATCTTTGATTTACAACAATGACATGTTTTACAGGTTCTTTGGGAACCAAGAACATTCATCTTCAGCTTCTGCAGACATTTGCAACCTATGGAATTACTAGATAAGGTTGAAGGATATTAAATAATATTATTACTCTTTCTTTTTTTTTTTTAAAGACAAATTAAAGTAAATATGTCATTTATATCATACCTTTAAATTCTAAATTGATTACCTGCAAATGCATGAACTTATTATTAGGTTGGTAACTTTAATTGAAAAAAAAATGG

>AdNAC79

GTGTTGAATACATGCGTCATAAGCATTTCTTTTTCTTTTTCTTTTTTAATGTTATATTTACAAAATTAAAGGAAAATTACACGTGTAGAAGTACTTGGAAGTTTCAGAGAAAGAGACAAATTCTCAAACATAAGAACAGGAACATAAATATATGATTTTGAATCTCCGTCTCTCTACTTGCATCACCAGCCACTTTCTCTTTCTCTCTCTATGGCCGGGTATGTACTTTACTACTTTTCTTTTCCTTTCCCTGCTTCAACTTCAAACTAGTTCATTTTCCCAGTAGAAGCTGAACTTGGGCATCTACTTTTCTTTCTATGTTTAGAATGCTAAATAGGGTTAACTCTTACATCTGAAAAATCATAGCTTTTTCTGTTTTTGGGTTTAATTTTTGTGGTGGGGTTCGCCTCTTCCTTGTTGATTGTTCTATGGTTGAGAACTAAGTTGGATGTCAGCATAAGCATATGAAAGTTGAGATTTTTAGTTTCTATGTACCTTCCCCCTTTTTATCATGCAGTTGCCGGATGCACCATTGAAATGAATGTTGTGCTTGTTTAGCTTAATGTTTATGTATTGTTCTCTAAATATAATGGAAATGTCTACTAGGTTCCGGTTTTTATTACATTCTCCAAAATAATAATAATAAATAAATAAATATAAATTATTACTCAGTGGACTTATGCAGTTCCAAACAACTGAAACTTGTGATAAGTTTTTTGTTCTTTTGTGCAACCATTGTGTCTTACGCATTTCTAACTTCTAAGACATCTTCGTACTGATATCAGAGAAGTAGGAATTTTCCTCCCTTTTCGGAATTAGTCTTTGTCAAATTTTACAATAGTAACTTCCTTTGAGGTTCTGTATATGTTCTTGTTTATGCAGATCATCGTGGTTGGTAGACAAAAGTAGAATTGCAACCAAAATAAAGAGTGCATCTGGAGCAAGTGGGAAAGTTTTATGGAAAAGCAATCCTACCAGAACTTGTCCGAATTGTCAACATGTTATTGATAACAGTGATGTATGTATATCTTTACAATGTACCATTTCATTTGAGGTTCTATATATTGACACATATGTTCTGTACTTCTGTGTGGATATTGCACATGTGTGTGTAAGGTTGCGTTCACGATCACTTGTTCGCCATCACCCTCGTCACATTTGTGTTTTCATTATTGTGCTTGTGTTCCATTCATTTACTCTAGTTTTCATGATTTCTTTGATCATGATTCTGTTACTCTTGCTGGTATCATATGGTTCTGAGTTACACTTTGTCAGTGATATTGCATGCATGTGATAATATATAGAAAGAAAGAAATCAGTTGTACCGTAGTTGAACTACCATAAACGGAAAGCAAAATTGATAGAAGTGTTTTATCCTAATTGAACTAAATGTAAAATAGTCATGAAAGAAAGCAATTCATTACTATTTAAACTGTACTGTATCTAAGGACTGCCACTTTTCTTGACAGTTGTTTCTATGAGATAGAATGAGCATCGAGATATCGTCTTTTGGAATACAAAATAAATAATAGCCCCCTTTTGGTATCCCCATCTTCCTTTATGCATATAAATATTTAGTTGTTGAAGCTGTTTCTATCATATAATTTTTCCTAATTGATGTACTATACTTGGAAATCATCAACCGAATAATCAAAATACCTTTTGCATGTGTTTGTGATTTTAAAGCTTTCTAGTTTTCATTGCATCGTTAGATTTCATGCATTAATTATAATTAGGGCACTCTTGTTATAACTTTCCGTTAACAATAATGTTTCTGTGGCTTGGGAGGTGCAACCAATTTCATAGGAAGTGTTGTATTGTGGATTCTAATATTTCTCTACGAAGCTAGACACATGAAAATCTTTGAAAGGGCTACAGTGACTGTTCAAGTATTTTTTCGTTACATCACACATTCTGAACAATGCGTGTTGATGGTTTCAAATGCTTGTCACTTTGGTATTTTCTTTACTTGTTTTCAGTTTGTTTGTTGCCTTATATATGGTATCATAGAAATGTATCCTATAAATTTAATTTGCATTATCAAGTTGAAAGTTTAAACACATACATAAATGTGAAATTTCTTCTTATCTCAGTCTCAATGTATTGCTCTTATGAATGTTCTAACATAGTAACACTTGACAATTTTCTATTGTCAGGTGGCACAAGAGTGGCCTGGATTACCAAAAGGTGTGAAATTTGATCCATCTGATCAAGAAATAATATCGCACTTGCTTGCAAAAGTTGGTGCAGCAGGTTCAGAGCCTCACCCTTTCATTGATGAATTTATTGCTACTCTTGAAGTGGATGATGGAATTTGTTATACACATCCTAAACATTTACCAGGTGGATATCATGACTTTCATTCAAATACGATGATTATTTTTGTAAACATAATAACATGTGATTTTTTTCCCCTTAAAAGGTGTCAAGCAAGATGGTAGTGCTACACACTTTTTCCACAGATCAATCAAGGCTTATAATACCGGCAATCGAAAGCGTCGGAAAATAAATGACCAGGACTCTGGCGATGTCCGTTGGCACAAGACTGGAAAAACTAAACCTGTCATCTCGGACGGGGTTCAGAGAGGCTGTAAAAAGATTATGGTTCTATATATGACTTCAGTTAGAGGAGTAAAAGCTGAGAAAACTAACTGGGTTATGCATCAATATCACCTCGGAACAGACGAAGATGAAAAGGAAGGAGAGTATGTTATCTCTAAAGTGTTTTACCAGCAACAAGTTAAGTTTGCTGAAAAAGATGATCATGATGTTCCTGGAACCAATGAAGCAACTGTTGTGAAAGATGATCCAGTCACTTCGGAACCTCCTCATAGTGAAAAGCAATGTTCAGATCTCGACATAGGAGAAAAATCACATCAGATTCCTCAGGTAATACCTCCAACCATGTTTTTTAAAAAGCATATAACTTAATCTAAGTGCCGTTCTTTCACGAAAATATTGTCTTAATTGTGTAGATAGACATTGCTTTTTCTTGCAACCAATGCATACTTGGCTGGAGTCGATGCTCTTAAATAATTTGTGGCCCTTCTAATCATTCTTTTTTATTAGTAGTACTGCGGAATGTCACTTAACCACTTAGCTAACATTCTAATTTTTAATGTCCTACATTTTAAGGCAATTTGAGGTTGACAATGATTCAGTAGTCTTATGATATTCGTGACGGTTATTAATGCTGTTTTCCTATTTTGTTTTCTGCAATATATTTTACCAAAGGGTCCTCAGACAGATTGTGTAGAAGACATTCAAGCCGAGTGTGAAGAGATTGTGAAAACTGATGTAGCCATGGCAGATGCTCAAAATAATGAAGGAATGGATAATGTAGAAAATAATGCTGATGGAGAACAAAAATGGTGGGACAGTGAGTCACAGAATTTGTTAGATTCACAACAACTTGTTGAAGCATTGGCCTTGTGTGATGATCTCCTCCATAGCCAGTGTTCCAATAAGGATGATGAAAATGTAGAACACAAGGAGCACTTGAGTCTTTCCATCTATGCTCATCTAGGACCAGAGCATCTGAAGAAAGATCTTGAAGAGTGCCAAAACCTTAATCTTGATCCTGCAAACGTAGAGCTCGAGACACCACCTTCAGAGTTTCGACTAAGTCAGCTGGTATTCCCATGTTCTCAATCATTCTTCTGTTTTGTTACGAATGCCGAATGTCATTGACTGATAACCTGTTTCCCATTTGCTATAGGAATTTGGTTCACAGGATAGCTTTGTTTCCTTGAGCGGCGGCAAGGCAGTCGACTAATCCGGCCCTTCTTCGGCTAATATGGGAAACAAACACATTTCTAACTACTTTTGTTGTAACATTTGATCAACAGTGGTGTAATTAATACTGTCTTTTGGTGTAGTGAAAGCCATGGTATGTTCCTTACAAGTTCTCTAGAAAGTTGCATGTGGACCTGACTTGTCCAAAAATCACTACATGTTTTTATTTCATTTTGAAAGTAACATATGGAGATGAACATGTACATTTATAATTTTATATCCCTATCTTGAAATGTGCTGTCTTGTCTCCCATAAAAATCTATTGTATGTATATTTGGTGGCGACAGATTCATGAATGC

>AiNAC37

GTGTTGAATACATGCGTCATAAGCATTTCTTTTTTTTTTAATGTTATATTTACAAAATTAAAGGAAAATTACACGTGTAGAAGTACTTGGAAGTTTCAGAGAAAGAGACAAATTCTCAAACATAAGAACAGGAACATAAATATATGATTTTGAATCTCCGTCTCTCTACTTGCATCACCAGCCACTTTCTCTTTCTCTCTCTATGGCCGGGTATGTACTTTTCTTTTCCTTTCCCTGCTTCAACTCCAAACTAGTTCATTTTCCCAGTAGAAGCTGAACTTGGGCATCTACTTTTCTTTCTATGTTTAGAATGCTAAATAGGGTTAACTTTTACATCTGAAAAATCATAGCTTTTTCTGTTTTTGGGTTTAATTTTTGTGGTGGGGTTCGCCTCTTCCTTGTTGAATGTTCTATGGTTGAGAACTAAGTTGGATGTCAGCATAAGCAGATGAAAGTTGAGATTTTTAGTTTCTATGTACCTTCCCCCTTTTTATCATGCAGTTGCTGGATGCACCATTGAAATGAATGTTGTGCTTATTTAGCTTAATGTTTATGTATTGTTCTCTAAATATAATGGAAATGTCTACTAGGTTCCGATTTTTATTACATTCTCCAAAATAATAATAATAAATAAATAAATAAATATAAATTATTACTCAGTGGACTTATGCAGTTCCAAACAACTTAAACTTGTGATAAGTTTTTTGTTCTTTTGTGCAACCATTGTGTCTTACGAATTTCTAACTTCTAGGACATCTTCGTACTGATATCAGAGAAGTAGGAATTTTCCTCCCTTTTTTGAATTAGTCTTTGTAAATTTTACAATAGTAACTTCCTTTGAGGTTCTTTATATGTTCTTGTTTATGCAGATCATCCTGGTTGGTAGACAAAAGTAGAATTGCAACCAAAATAAAGAGTGCATCTGGAGCAAGTGGGAAAGTTTTATGGAAAAGCAATCCTACCAGAACTTGTCCGAATTGTCAACATGTTATTGATAACAGTGATGTATGTATATCTTTACAATGTACCGTTTCATTTGAGGTTCTATATATTGACACATATGTTCTGTACTTCTGTGTGGATATTGCACATGTGTGTGTGTAAGGTTGCGTTCACGATCACTTGTTCGCCATCACCCTCGTCACATTTGTGTTTTCATTGTTGTGCTTGTGTTCCATTCATTTACTCTAGTTTTCATGATTTCTTTGATCATGATTCTGTTACTCTTGCTGGTATCATATGGTTCTGAGTTACACTTTGTCAGTGATATCGCATGCATGTGATTATATATAGAAAGAAAGAAATCAGTTGTACCGTAGTTGAACTACCATAAACAGAAAGCAAAATTCATAGAAGTGTTTTATCCTAATTGAACTAAATGTAAAATAGTCATAGAAGAAAGCAATACATTACTATTTAAACTGTACTGTATCTAAGGACTGCCACTTTTCTTGACAGTTGTTTCTATGAGATAGAATGAGCATTGAGATATGGTCATATGGAATACAAAATAAATAATAGCCCCCTTTTGGTATCCCCATCTTCCTTTATGCATATAAATATTTAGTTGTTGAAGCTGTTTCTATCATAAAGTTTTTCCTAATTGATGTACTATACTTGGAAATCATCAACCGAATAATCAAAATACCTTTTGTGTGTGTGTATGTGATTTTAAAGCGTTCTAGTTTTCATTGCATCATTAGATTTCATGCATTAATTAGAGCACACTTGTTATAACTTTCCGTTAACAATAATGTTTCTGTGGCTGTGGGAGGAGCAACCAATTTCATAGGAAGTGTTGTATTGTGGATTCTAATATTTCTCTACGAAGCTAGAGACATGAAAATCTTTGAAAGGGCTACAGTGACTGTTCAAATATTTTTTCGTTACATCACACATTCTGAACAATGCGTATTGATGGTTTCAAATGCTTGTCACTTTGGTATTTTCTTTACTTGTTTTCAGTTTGTTTGTTGCCTTATATACAGTATCATAGAAATGTATCCTATAAATTTAATTTGCATTATCAAGTTGAAAGTTTAAACACATACATAAATGTGAAATTTCTTCTTATCTCAGTCTCAATGTATTGCTCTTATGAATGTTCTAACATAGTAACACTTGACAATTTTCTATTGTCAGGTGGCACAAGAGTGGCCTGGATTACCAAAAGGTGTGAAATTTGATCCATCTGATCAAGAAATAATATCGCACTTGCTTGCAAAAGTTGGTGCAGCAGGTTCAGAGCCTCACCCTTTCATTGATGAATTTATTGCTACTCTTGAAGTGGATGATGGAATTTGTTATACACATCCTAAACATTTACCAGGTGGATATCATGACTTTCATTCAAATACGATGATTATTTTTGTAAACATAATAACATGTGATTTTTTTTCCCTTAAAAAGGTGTCAAGCAAGATGGTAGTGCTACACACTTTTTCCACAGATCAATCAAGGCTTATAATACCGGCAATCGAAAGCGTCGGAAAATAAATGACCAGGACTCTGGCGATGTCCGTTGGCACAAGACTGGAAAAACTAAACCTGTCGTCTCGGACGGGGTTCAGAGAGGCTGTAAAAAGATTATGGTTCTATATATGACTTCAGTTAGAGGAGTAAAAGCTGAGAAAACTAACTGGGTTATGCATCAATATCACCTCGGGACAGACGAAGATGAAAAGGAAGGAGAGTATGTTATCTCTAAAGTGTTTTACCAGCAACAAGTTAAGTTTGGTGAAAAAGATGATCATGATGTTCCTGGAACCAATGAAGCAACTGTTGTGAAAGATGATCCAGTCACTTCCAAATCTCTGACTTCGGAACCTCCTCATAGTGAAAAGCAATGTTCAGATCTCGACATAGGAGAAAAAACACATCAGATTCTTCAGGTAATACCTCCAACCATGTTTTTTAAAAAGCATATAGCTTAATCTAAGTGCCATTCTTTCACGAAAATATTGTCTTAATTGTGTGGATAGACATTAATTGATTTGCTTTTTCTTGCAACCAATGCATACTTGGCCGGAGTCGATGCTCTTAAATAATTCGTGGCCCTTCTAATCATTCTTTTTTATTAGTAGTACTGCGGAATGCCACTTAACCACTTAGCTAACATTCTAATTTTTAATGTCTTACATTTTAAGGCAATTTGAGGTTGACAATGATTCAGTAGTCTTATGATATTAGTGATGGTTATTAATGCTGTTCTCCTATTTTGTTTTCTGCAATATATTTTACCAAGGGTCCTCAGACAGATTGTGTAGAAGACATTCAAGTCGAGTGTGAAGAGATTGTAAAAACTGATGTATCCATGGCAGATGCTCAAAATAATGAAGGAATGGATAATGTAGAAAATAATGCTGACGGAGAACAAAAATGGTGGGACAGTGAGTCACAGAATTTGTTAGATTCACAACAACTTGTTGAAGCATTGGCCTTGTGTGATGATCTCCTCCATAGCCAGTGTTCCAATAAGGATGATGAAAATGAAGAACACAAGGAGCACTTGAGTCTTTCCATCTATGCTCATCTAGGACCAGAGCATCTGAAGAAAGATCTTGAAGAGTGCCAAAACCTTACTCTTGATCCTGCAAACGTAGAGCTCGAGACACCACCTTCAGAGTTTCGACTAAGTCAGCTGGTATTCCCATGTTCTCAATCATTCTTCTGTTTTGTCACGAATGCCGAATGTCATTGACTGATAACCTGTTTCGCATTTGCTATAGGAATTCGGTTCACAGGATAGCTTTGTTTCCTTTAGCGGCGGCAAGGCAGTCGATTAATCCGGCCCTTCTTCGGCTAATATGGGAAACAAACACATTTCTAACTACTTTTGTTGTAACATTTGATCAACAGTGGTGTAATTAATACTGTCTTTTGGTGTGGTGAAAGCCGTGGTATGTTCCTTACAAGTTCTCTAGAAAGTTGCATGTGGACCTGACTTGTCCAAAAATCACTACATGTTTTTATTTCACTCTGAAAGTAACATATGAAGATGAACATGTACATTTATAATTTTACATCCCTATCTTGAAATGTGTTGTCTTGTCTCCCATAAAAATCTATTGTATGTATATTTGGTGGCGACAGATTCATGAATGC

>AdNAC43

CACAATTTTGGGCTTCTTTTTATTTGTATCATTTGAAAATTTTGGATGTTTCTCAGACAGAAAGAGACAGACCTGCTTTTGAATTTGTGACATTGGTTTGAATCATCAAAAACCTCTCTTCGTTCGATGGCAGGGTATGTACCTACTATGCTTGATCATCAAAATCCATTTTTTTATCCGCACATCGTTTCGTAGCAATACTATGTGAACTGAGAGAGATTATTTTTCCACTTTCATTCTATATTAGAAGTGGGGTTAACTTGTTCATGTGGAAAGTTATACCCTTTTTGCGTTTTGGTGAGATTTCTTAGTTGTGTTGGTCGTCTTGGCGATTTTCTGTTTATTGATTGAAAGAAAAGTGAACGGATTTAATGGGATTAGTTAGTTTTATGGGAAAAATGACTTCCTTTTTTCTATTTTTTTTTTGGGATAAAATTTTTTACTTGGAGTTCGTTTCCACGTTGTTGACTGTTCTATGATCGAAAGATAAATGGATGATCCTGAAAGCTACTGAAAGTTGGGGTTTCTATTTCTTGTATTATTTTTACCCATTTGATAATATGCATTTATGGTAGTGTGTGCCATAAGAATTAGTTCTTACAATTTTTTAGTTTCACATTGTTTTTCTTGCTTTATTATCTTTGTCAGTTTTGACTTTTGAGTTCACTTAATAACGTAATGAGTGGCAATTTTGTGTATGTGTGTGTGTGAGCTTTGTGTTTGTATTTTTGTTCCAAGTTCTGAAATAGTGAAATTTATTAGGTTGCTTAAAATTGGTACAATGAACATATGCATTTCTTGATAAGTATCAGTTCCTTTTTTGGTGGTGAGGTGATCATAGATTGCATTGTTTCTTTCTCTATGTTACCAAGCCAACTTTGTAGAAAAATAAGAGGAGTCTGGTGATTGGGACTTTTATGACTCATTATTTTATCTATTTTGCTATTCTTTTGCCATAGAGGACTCTTGATCATTTTGATTTATTGTTTCGTTTTTCTTCGGGGTGTTAGAATGTATACATGGTCATGGACTTCTATAACTTTGTGGCTCTTATATTTTTATGCAGAGCATCATGGTTGGTGGACAAAAGCAGAATTGCAACCAAAATCAAGAGTGCATCAGGAGCTTGTGATCCTAACGAAGTTATTTGGAAAACCAATCCTACCAGGATTTGCCCGAATTGTCATCATCCTATTGACAATAGTGATGTATGTCTGACACCCCCCCCCTTTCTCTTTTTAAACTTAATTCTGATTAGATTTCATGATAATTGTTTTTTTTTCTTTCTATTTTTATATTTTGGCCTTTCAGAAATATAAATTTTATATTTTCATGTATTTAAGTAACATCATTTTGTATGCTAGTTTGTGATCTATTGAGTGGTCTTTGTTACATGGGAATTAATGGTTTCAACTTCAAACTAAACATGATCTGCTTGGAAGTATAATTTCTTTTGTAGAAAATTGCATATTACCAGTTTAAGCGTTGGTTCAAAGGACCGGTATTGCTTGATATTCATGTAAGTGTGAAGATAACTTATCAACTGCATTAAGGGTAATGCCTTCACATCAAGGTCAAAATTAATGGAATTCCATTGCTTCCCTAAATTTTGCATATGCCAATATGAGTTGAACCTGTTAAATTGGCTTAATCCACCATGCTTTTGGGACAAAAGTTGGAACATGTTTTAATCACCACTTTGGTACTTCTAGTATTTTATTTTCTTCTTCATGATAAAGTTATATGCCATTCTTCAGAAATATGTTTAGAAAGTTTATTACCAATAAAGTTAAAAGATATTGGTTTGATAGTTCAAACTTCATCCTTTCTATTTCACAAATAATGGCACTTAAATAGTTATAGATGATTTAATATAAACTTCCTGTTGTTAGGTAACTCAAGAGTGGCCTGGCTTACCAAAAGGGGTTAAATTTGATCCGTCAGATCAAGAGATAATCTGGCACTTGCTTGTAAAGGCTGGTGTAGGAAATTTAAAACCTCATCCTTTCATTGATGAGTTTATTACTACCCTTGAAGTGGATGATGGGATTTGTTATACTCATCCTCAACATTTACCTGGTTTGTAGCACATAAGTTTTATTCATAGTTGAATTCTTGTTTGATTTTCAATCAATATGATATGTTGCGATTAAAATTTAGGTGTCAAGCAAGACGGAAGGGCCTCACATTTCTTCCACAGAGCAATAAAGGCTTATAATACCGGCACGCGAAAGCGTCGAAAAGTACATGGTCAGGATGACGTTCGTTGGCACAAGACTGGAAGGACTAAACTGATCACCCTGAACGGGGTTCCAAAGGGTTGCAAAAAAATCATGGTTTTGTATACAAATGCGGTGAGAGGAGGAAAGTCGGTGAAAACTAATTGGGTTATGCACCAATATCACCTTGGGACAGAAGAAGATGAAAAGGAAGGAGCATATGTTATTTCTAAAGTGTTTTATAAAGATGACCAGGATATACCTGAAGCCGCAGAAAGTAAAAATGCAACAGTTGCGAAAGTAGATCCAGTCACTCCCAAATCCACGACTCCTGAACCTCCTCGTAATGAAAGGCAAGATTCAGATCTAGGCCTGGATCTAGACCTAGGGCAAGAAGCACTTGCTTTTACTGAGGTAAAACCTTCAATGTTTTCCTGCTATTGAATATCAGAATCTGTTTTCTGTTATCATTTTGCTGTGATCATTTTCATATTGCGTGTTTTTTCTTTTGTTATTGGAGCGAGATCTGTCAGAAAATGGATGCAACTTTATAATATATTTTTGGAAAAGAAATNNNNNGCTGGATTTGGTGGAAGTACTTTTCATTTATAAAGTTCGAAACCTATTTTCACAAAGTAAATATCCATGATTCTACCTGCTAAAAGAATCACCATTAGTTCTGCATTAGATACTTTTTGTCTTAGCTGTTTTTCTGTCTCAAGTTATTATTGCTTGGCATTGAAGTCTTTTATGATAGTTGTACAATTTATATGAAAGTAGAAATAAAGCGAAAGCCTAAAATTAATTTGTTGTATTCCACTTTTAAGTAGTCTTTGTCCCTTGATTCATCTTGTGATGTTAATTTGATTAAGAAAGGAAGCTTTGTGCTTAACTTTACATCTCATTGAACTCTCATGCTTGAAGAATACTGAACAGTTATTTTGCAATCATATCTTGCCTCCTTTTTTACCTTTTCCCAGAAACTGCCCTAAGCATATAATCTTGACATATAATTTGATGATATAGTTCTGTGGAATCTGAAAATTGCACTAACAGAAGTTGATTATTCCCGTTCATTGTTTCTTTTTTTTTTTTTTAATTTANNNNNNNNNTTTTAATTTATACCCTTAGATGGATTGCTTAGATGAAATTCAAGCTGACTGTGAAGAATCTGCGAAAGCTAATCCACCAGTACTGGAGACACAAGAAAATGAAGGGATGGACAACAAGGAAACTAATGCTTATGAAGCACAACCGTGGTGGGATAGTGATTCACAGAATCTATTAGATTCACAACAACTCGTTGAAGCATTAACTCTCTGCGAAGATATATTTCACAGCCAATCTTCCAACAAAGACGATGAAAATGATAAGAACCAAACCGGTCTATCGGTGTATGCTCATCTAGGACCAGAGCATCTGAAGAAGGATATTGAGGAGTGTCAAAAGCTTGCTCCTGCAGGACCAGAGCATCAGAAGGATATTGAAGACGGCCAAAATCTTGACATCGACCTTGCAAATATAGAGCGGGATACTCCTCCTGAGCATCGACTAAGTCAGCTGGTATCTCCATGTTCTTGATGATTTTGCATTACAATGAATGAATTCATTTAAATCACCATTACTAATTACTAGAATGCTTCAAATTAAGTTTTCTTTCATTTGGTTTTGAAAGTAGTTATGAAATTTTTTTCTTTTGGAAAAAAAAGTGTCACTTGTGTAATAGCTAGCTTAATTGAAAATTGCGCACATAATCAGTAGTTACTTTACATATGTTAGGAAGTTATCTCAGGGGGTAAAAGTTTTGTACTAATGGAGAAAAAATATTTGGACACCAATCATCATAAACCCTGTTTATTAATGATGCATTTTCATGTTTCTGTAGGAATTTGGTTCGCAGGACAGCTATACTTACTGGGGTTTTCAAGGCGTAAACTAATATCTTCCTTGATCTCTCGGCTTTGAACCAATTTTTTTGCAACTACATTTTGTTGATGTGTTATTTGTCTTTTGCCTTTGTGTTATGCTTAACTATGGGGCAAACTTAACTGTTACGAGTAAGTGGTTACTACTAACTATGCAGTAATGTAAGACTGTGAACCTGATATATATTTTGATCAACCTTGGTGTAAATGTAATTCAAACTTGCAATTCTCGGCTGGTTGAAGTTTTGTTCTATATTCTTGCAGTTCTGGATGTGGACTCTAGAGAGAAAGATGCATTTAGATTTCTACTCTATTCAAGCGCAGCTATGGAATTATCAAAGTTGCTTTTAGATTTCTGATAATGGCTTAAGACTGAAAAATAGTAATGTTGGCTTTGTTCTACAGTACCAAGTAGTATCAGGATGTGAATTTTATCCAAAAATAATTTCTTCAAGACCAAAAATGATATATAACCAGATTTATGTGAAAATTT

>AiNAC34

CCATTCCCTGAACGAACCAACATTTCTTTCTTTTTCCTTTTAATTACTTCTCTTTTTGAAGCTAAGGATCCTCGGGATCTATCTCATCACCTTCATCATTCTTGAGTGCACAAGGCCGTGATCCACTTCAATTCCCGAGGCTCTCATTAATTACCACTTTTTCTGTTCTCTTTTCCCCATCCTGTTCATTCCATTGCTGTTTTTTGGTGCAATTTCTGGAAGTAGCTCTTACAAAGACTTGGGTTATATATATTGATTTAAAAGGATTCTTATCGATAGAGAAATTGTACTACGTACAAGAAAAGTTTGGTTAATTTCATATACAACAGATCTACGTACAGGGATTCTACTTGGAGTTCTTAGGTGCAGGATATATCAAGCTAAGGTTGTTCTTTAAACTTGTTCTCATTATATATAATGCATATCTACTTTAGCATATTATTAATATGTGAGTATCTCTATCTATATTCTTAAGGCATGACGAGAGAAAATATTACTGGTTTGTTTCTTTTAATAACTCAAGTCAACCAGCATAAGTCCAAAAAACAAAAAGAGACAACAATAAAAGACTGATGAAAACATGACATAATTAAAATCTTTGACATGACTTCGATCTGTGTGTTTCGGTTGCAATTTGTGTGTTCTACACCTACCGTGATTCATGTGCTTTACTTCAGCTTTTTATTTATTTATTTATTATTCCCTTTGTTCTGGTTTTAATTTGCACCCGACAGATATTGATCTCTTTGCTTATTCATTCGACCGCAAAACAACCATGCTAGCTTTAGTTTATCTGCCTGTGATAGATGCCATGATCATTAGATTTTCCCGAACCGTTTTTATATATGTTTGAATGATCAGTCATCAGTTCCTAAGTCAAAAACTTCGCCTCTTCTCTTAGACAATTTTAGCAAACTATGCTGTAAGCCATTACGTACGTTCGTGAAATGCTTGAGTGCTCTACCAGTTTTGCTGTAGGGTTGGCTTCAAATAGGGTAAATATTTGATGCTATATAATCAAACCCTAAATCTAATGGAGTTGTTTTGGAGAAATTTTATTAGCTGACGAGACCGTAATTGTTTACGGTTGATTTTTCCATCCTTCTATATTAGTTCTATAGTGTTTGTATATAGCAACAGTATCTAAAGGGGAACATCAATTATCTGCTGTTGTTGAACCTTTTTTTTTTTTTTAATTTCAATTTTTAACCGTGAACTTCCATTCAACACTACTATCATATCTTTTTTCCTCTTTGTTAAACTTCTAACTTGTAAGCACAAATCGAATTCAGGCAGCAGAAGATTCCTTCGAATTAGACCCTATCAAATACAGTCAACACTACTAGCTGTTGCTGTTATTCATTTTTATTTTAAAACTGTTGTTACAGAATTTAACATATTCCTGTGAAATCTACCACACAATACGTATATACATATGTAGATCATGTTATTATTTGCAAACAAAAAACCAATTATCAATCAATCATCACTGATATAAAATACATGTTAAAATATATAATACAAACATTAATAAACATAAATTAAATTACGTGGCTAATAACACATTAATACACAGAAATTTTGCTGAAATTTTACAACTAACGGAAGTGATATGATTTATTGAAATGCAGGAGGTTTGAAATGAACGCATTTTCCCATGTTCCTCCTGGGTTTCGGTTCCATCCGACGGATGAAGAACTTGTTGATTACTACCTAAGGAAAAAGATAGCTTCGAAAAGGATTGATCTGGATGTGATAAAAGATGTGGATCTCTATAAGATTGAGCCATGGGATCTTCAAGGTACGTATCAGAGCCACTAACATACATTAGTAGATAGAAATGAATTATAACTGGAGTGAGATGCATGCATGCAGAAATATGCAAAATAGGAAGCGAGGATGAAAATGAATGGTACTTCTTTAGCCATAAAGATAAGAAGTACCCAACAGGGACTCGCACCAATAGAGCTACAAAGGCAGGATTCTGGAAAGCCACGGGAAGAGACAAAGCCATATACTCAAGCTCAAGCCATTGCCTGGTTGGTATGAGAAAGACTCTTGTGTTCTACAAAGGACGAGCTCCCAATGGCCTCAAATCAAACTGGATCATGCACGAGTATCGTCTTGACTCCAATCAGGAAGATGGCTGGGTTGTGTGCAGAGTCTTCAAGAAACGGATGCCCACGCTACGCAACGTGGTAGACTATGATGATCAACTTCCCTTCATGCAAGGATCTCCATCCACTCACTATCCCTGCAAGCACGACCTTCATCAATTCCAATACAACACCCATGATGCTTTTCTCCAACTTCCACACCTTGAGAGCCCTAATCAAGTTTTGAGTTCACCCTATGCCTACGCCGAAAACAACAACAACAATAATGGAACTTTGCAGTCCTATTCATCTGAACGCATTCAGCAACAACTTCACTTGCTTTATGGTAGCAATATTGAGCAAGCAGTAGTGGTGGACCAAGTCACGGATTGGAGAGTGCTTGACAAATTTGTTGCTTCTCAACTCATGAGTCAAGATCAAGATCAGGCTTCCAAGGAAACCTGCAGCGTGGCTGATGAACAACATGTTGCTACTACTGTGCTTCCAAATGGATCCACCAAGCAGGATGACTATGTTTCAACGTCTGCCTCCAGTAACTGCGATATTCACCTGTGGAATTGA

>AiNAC3

ATCTATCGCTTATACCTTTTGTCCTCTTCTTTATCTGATCTTCCCTCTTTCTCTCTCTATCAGTGATCACCAATATCATCAAGCACATAAGAACGAATTCAAGACGGTGGCCATGGTAATCATAAGCTAAGGGAGATAGAACGTGTCCCAAAAGAGTACAAATAAAGTACTACTATGCTTTCTACCTTATTTTACCTTTCTCTTAACTATATACTTAACTTGTAATTTTTTCTCCTGTTCAATTTTTGGGGGTTTTTTGTTTTTACAATTCTAAGTATAAGGGAAGAAAAATTAGAGGGTGGTCTCATTTATTGTGATGGCATGGTGCAATGACACTCATGAGAAAGAGATAATTGCTTCCAATAATAGTACTATTACTCTTAGACCTAAATCCGACCCAAACGTAACTTGCCCCTCATGTGGCCATAACATTCAAATAATCCAAGAGCAGGTACTCAAATTAAATTATCTTCTTTGTTTGTGGTCTTATGGTTTATTAATTAGGAACTTGAATATATAATAAATTGGTTTAATTGTTTTGATAGGGTGGAATTCATGAGTTGCCGGGGTTACCAGCTGGAGTGAAGTTTGACCCAAATGACATTGAAATATTGGAGCATTTGGAGGCAAAAGTTATGTCTCATGTGCCCAACCTTCATCCTCTCATTGATGAGTTCATACCAACACTTCAAGACGAGAATGGCATCTGTTATACACACCCAGAGAAGCTACCAGGTATTATTCTTTTATTTCTTCTATTTAATTCATTTTCCCTAATTAATATAATTAGGACATAGGTTATTATAAACTTATTATATCATTTATAATTTAAGGTAGGCACGCTGCACGCACGTCTTGCTGTGCACGGCATGCATAAAATTAATTACTCTTTTTGTAATAATAATAATGAGAATGATTTAATTTGATCTTCTCTTTGAAAAGGATCTAATTATTATATATACACACCACAAGCGGGATCTTAGAATTTTATTTAAATTATTGTGATATCTTTTAAGCTTTTAAAACATTTTTTTAAACTTGTTATATACTATAAATTACCGGGTGATTAAAAGAGTCTGAAAATATAAAGATTTCACCTTTCAAATGGTTGAGTAGTGGCAATAACTACCAAACTCTTAAGTTTAGCCATGTAGCATGCCGAATGAAAACGTTATTAACCCTTAATTATGTTCTAAAAAATGTTTATATTTTACGTCAAATTTGAACATGGCGTGGGGTGTCGGCCAAAATCTATCTGTCAACACCAAAAAATGGGGTTGCTGCCCTGCTACTCTTCTATTTTCATGAGCCTTCGTTATACTCAATATATATATATACCCAGATCATTTCATATATAGCTCCTATATATCCTATATAACAAATTTAAATTGCTGTTGATTGATATCTTAGTGTAGCATTATTTCATATATATTACCCTAAATTTATCCTTGTAACCAAAACTGGAGAGAAAAGAAAAGATTGATGATTTAAAAATTCAACTTGGTCAATGATCACCCAATTTAATTTTTAAGATAAAATTAAATATAGAATTAAATTTTAATATACTAATTATACACGTAAGTTTAATTCCGTATTGTTACTTAGCTAAAATTTTAAAAACATTTTTTAAGGTGAGTACATTAAAATTAAACAATTAAATATATTAATTTATTTTTCGATATCAATACATATATAGTCTAACAAAAATGTTATTTATACATCAAAATTAATATATTTGTATATTATATATGTGTGGTTTAATTATATTTCAACATACATTTTAGTACACGTAGCATAGTCGATAATCCAATTATCTGATTCTAAAGTTAATGATTGATTATAGCATTATGAATGACGAAAATCATGTTAATTATACCATAACTATAATAATGGTTTGCAACATTTTTACACTATATCTCATCTCCTTTTGTTTATATTTACATGTTAAGATTATTTGCATACATTTCTTAACATAAGAGTTTCTTTATGTAATAAGTATGAGACAAATGAAAGATTATTATTAATTAATGATGTAGGAGTAAAGAAAGATGGGCAGATCCGGCACTTCTTCCACAGGCCTTCAAAAGCATACACAACAGGAACAAGGAAGAGAAGAAAGGTTCACACCGATGAAGATGGAAGCGAAACAAGGTGGCACAAAACCGGAAAAACAAGACCGGTGGTGGCCGGCGGCGGCCTAGTGAAGGGTTTCAAGAAGATTCTAGTACTATACACCAACTATGGGAGGCAAAAAAAGCCTGAGAAAACTAACTGGGTGATGCATCAATACCATCTTGGAAGCAATGAAGAAGAGAGAGATGGAGAACTAGTAGTTTCAAAAGTGTTCTACCAAACACAACCTAGACAATGCGGCAATTCCATTGTTATAAAGGAAGATAATGATGATGATCTTCCCTATGGAAAGATATTGATGATGAATAACAGTAGTAAGAAGCACAAAAATAATAACGATAGAAATGTTGCTGCTCCTGTTGTGGACTACTACATCAACTATGACCATGTTGATCATCATAATCACAATCATAATAGTCAAAGATGTTCATCACCTACTCAACTTATTCCAAACTTGGTTCTCCAAGGTGATTCCTCTTCTCTTTTTCGCTTTGCTTCATCATCACTCGATGGGAATACCAACAAAACAAGACTTTTTGAGAGAAAGTTGTAGTAATTCGTTCATTATTATTATTTCTTTCACTAAGTGACAATATTTATTCTATACATATATGATATATGGGATTAATATAATTAGAGTGACATGTAATTAAGTTGATTGGCTTTGATGTGAATTAAGGACTCACTCATATAATCAAAGATTGGTTTTTATTATGTTAATTTTAGTGCTTAGGTACATTCAAATATTGTGGTATAAATAGTAGGGTTATTATTGTCCATTCATAACTTTGTTAAATAATGAATTAAAATTTTTCTCTTTCTCTTTTTTGTTTTTCTCCAATAATATATCTTGAA

>AiNAC48

AATTAAAGCTACCTTTAGTCAGAGTAGAAAGGACAAAAACTTGAATTTAGTCTTCTACAAGCTTTCATACAAATGATATATACAAAGCAAATAAACCACAAAACTTGAGATCAAAGCAACCTTATTATGCATTATTTCTCATGTGCAACTAACCCTATCTATCTCATCTTTCTAAATAGTTAGTAGTTTCATTCCTCCTCCATTTAGAATTTTCAGGGAAGGAGGGAGTACAATATTATTCACCTCATGCAAAAGAAAGAAAGCACCAAAAAAAGTGAGAGGAGAATTTAATTCCTTCCCAACCCTTAACAAGAACTTGCATGAGGGGAGCAACCTTTAGAAGAGACAACTTCTTCTTTATTATTTCCCCTTCTTATATACTGTACTCATCACTATTACTTTAAATATATTCATCTCTTAACTCAATCTACACATAAAATCACTCTATCTCCTTTGCTTGATGCATATATCTCTTTTTTTTCTTTATTATTTTCTTATGGCAGCGTGAAGAAGGAAAAAGAAGATATGTATCCTGAAATGGAATGAGAGAGGAGAAGGGAAACATAGGGTAACACATACAAGAGGAAATAATGACTCAGTGCAACAGTAATGATTACCCTGAAAACAATCATAACACCATTGTGGAGAGGAACAAAGATATCTTAATTAGTAGAACTTGTCCATCATGTGGTCATCATATCAAATGTCAACAAGACCACCAGGTTATTAATTCTTTTACATTTTTGTACTTCAATTATTTTGCATGTGTTATATTCATTTTTGTACAATTGGGGTGCTCATGATTATTATTTATTATGATATGATAATTAGGGTGCTGGAATTCACGATTTACCTGGGCTACCAGCTGGAGTGAAGTTTGATCCAACAGATCAGGAAATTCTAGAACATTTGGAAGCCAAGGTGAGGTCTGATATTCACAAGCTTCACCCCTTAATTGATGAGTTTATCCCTACTCTTGAAGGCGAGAATGGAATCTGCTGCACCCATCCAGAAAAGTTGCCAGGTCTTCATCAATTTATTTAAAGTAAAAGCATTTCATAATTCATGTATCAAGACAAGTCTTTTCTGATCTTATTTCCTTCCTTATAATCTTGTTATCCCATATATTTTACATTAACTCCGATTCATATATGCATGTATGAATCAAGATATAGTTATTGGTCTAATAAATTGTAGAACTGCAGGAGTAGGCAAAGATGGGTTGATCCGTCACTTCTTTCACCGGCCATCCAAAGCATACACAACAGGAACAAGGAAAAGAAGAAAAGTTCACACTGATGCTGATGGCAGTGAAACAAGGTGGCACAAAACAGGTAAAACTAGACCAGTCTACATCAGTGGCAAGTTGAAAGGCTACAAGAAAATCCTTGTTCTCTACACCAACTACAAGAAGCAAAGGAAGCCTGAGAAAACAAACTGGGTCATGCACCAGTACCACCTTGGCAACAATGAAGAGGAGAAAGAAGGTGAGTTAGTTGTGTCTAAGGTTTTCTACCAGACACAACCTAGACAATGTGCAGGTTCACTACTCATCAAAGATTCATCATCATTCCCTGCTAAACTAAAGGATCAAAGTGGTGTTCATCATCATGAAGTGACTAATAATCATAAGAACAATGGGTTTGTGGAATACTACAATGCATCCTTTATAAGCTTTGCTCAAGGGGAACAACAACATAGGTCAAACAATCCCACATTGATTTCCCATTTTCCTGCTCATGATGGGGCTCCGTTCATTCCTTGATATATTTTATGTGAAACATAACTTTTATTACTTGTACATTTTTATTATTGATTAGAAATTCTTTGTGGGACCAAGTTTAAGGCTCACATTTTTATCAATAATAAAAATGTAAGTAAGATAAAGTTATGTAAAATTTCATATGAGAAAATCTATTCTTAAAGGATGAACAAAATACTAAATAGGACTTATTACAGTTATAAGCTAAGGCATTACAATTGTATTCTGCAATGAAGAAATAAGTTAATGACCAATTACAGTTAGGAGGGGAAAGAATAAGGAGAAGCTACTTCATTGTAAAGCTGGTGGTCAGATACTTCTATTTTGGGAAAAATTGGGCTAAGAAACTAAAAGAGAAGGATGTAACTG

>AiNAC8

CTCTTTTTGATTATCTCTCTATCTATCTATGATCATTTCTTCTTTGTGTCTTAGATTCTGATTCTTGATTGGTTTCTCCATTTTTGGCAAGTTGTTTCTTGAATTGCAATAATGGCTAGGTGAGCTATAGATTTTTAACAGAATGTGGAGATTTTTTATTTTTGCATCTGATAAAGCTTTCACTACCTTCTTCTTCTTGTGATTTGGAGACCCTTTATAGCGTTGATGAATAAAAATTCAACTTTTTCAACTGGGGTTTTTTTTAGGGTGGTGTCAGAGGAATGTTCCCCTGTTTCTCAGCTTTGTTTTTGTTTTCTCTTCTTTTCAGTTTCCTAACAAATATATATATTAAAATATAAAACTATGTGGTTGAGTTTTGATGATGAATGGATCATGGATGATAGCATGTTTTGGCTTTTATATTCCCCCTAAATAATAATCATGGGAAAATGGATGATGGATTATGTTACATATTCCCATTACAATGAGTTTACCTTAGCTTATTTTTTGTGTTTTTGAAGAAAATGTGATTGTTAAACCTAAATTTTAAGCAAATTTGGACATGCATGCACTGAACTGGGGCTTAGAAAGGATATATAGATGTCCTAAATCTTATTTTCTGGCGCGAAAGAAACATTTGAGAGGTACTCAATGATGATCATAGTTACCTGGATGATTGAAATGTGAAGTAAAAAAGCCAAGATGGTTTAGCTGGATTTCCAAATATACTACATTGTTTATGAACAAGAATTAATTCATAAATAAAGAACTTCAGTTCATCATCCTTGAGTCTGAAATCTGTCCTTTGTGTTCCTCAAAATGAAGTTGGTAGAACAAATTTTCTGAAGAGGATACAGTTGGAGATCACTTACAATGCACAAGCCTTTCTTAATTATTACCCTAATTTTAATGATCAATGGCATCACATAAAAACTAGTTTTCATGTCTAAATTTAACTCATAGGTTTGAAAGATCAAGGCTTCTATCATTGATGTCCTTAAACATGATCAGTGCAATCATTAGAAATAGTAGATTTTTCACTTGACGTATAGTTATATCTTAGAAACTATATAGAACACAAAGCATGCTAATTCTAATTTCTATGAATCCTTCTTATGATATTATGGCTCATTTCTTCATATCTTATCTAATACAGTTTCACAACCTCTTTATGTATCAGTCTAGTTGCTTCTTTTTCTTTTTTATTTCTCAAAACCCAAATATGCTTCTTTTTCTAGCTGGGTTATTGACATTGGAGGACTTGCAAAGAAAGTGAAGAATAATACACTGCCATTAGCTGATCAAATCAATGACTGTGGAGCATATTGTGAATGTCCAATATGTCATTATCACATTGATAACATTGATGTATGCTATAATGAATTCAACAAAATCTTCAATCTCTGTTCATAACTTCATGTATAAATTCATGATTACAGATGTTTCATTAAGAACATTCCTTTGCCTTTTGTAGGTTTCTCCTGAGTGGCCAGGCTTTCCGGCCGGCGTGAAGTTTGATCCTTCCGACGTAGAACTGTTGGAACATTTGGCAGCAAAATGTTGTGTTGGAAACAAAGTGCCTCATGCCTTTATCCAAGATTTCATCCCAACACTAGAAGGAGACCAAGGAATATGCTACACACATCCAGAAAATCTTCCAGGTGAGGTTTCTCTAGTTACCTTCTCTTTCAATGTTAGCTAATAACTTAAAATCAGGGTTCCTTATGAACCTTATTCCTTAAAGATATTGCTCTTTTTTTGTGAACATTTATACTTGACCAAGAAACTAGAAAAGCATGAAAAATACAATACATTCTAGCATGTTGGGATATATAATGTAGTGGTTTAATCAAATATCAACAGGCATATAACAAACTTGCTTGGATTGTGGATCTTACTTTCGCATGAAATTCCAAGTTGAGTGTTAGGATTTTAGATAGTAAGAGCAAGTAAGCTGAATAACAAAAAGTGACACAAAATACATTAACGTGGTTCGGCTTATGCCTACGTGGCAAAGACGACAAACAAATATGAAGGAAATACAAAATATGTGATACCAAAATAATGACCAAATACTTAAACTAAACCAAGTCTCAAATACACCAAAAATACTTTATAGTACTATCAAATAGTATAAAGTATAAACTCTTGGCTCTCAGGAGAAGTAAACAAGAACAAAATACTTCTCCCTATACAAAGAGAGACACGAATAATTAATACGAATCTTCTCTAACAACCAAAAGAGTAACCAAATAGACTTGGTACACACTAATGTGGAGAAACAACACTCAAAATGAGACCTTACAAATTTTCACTAACAACACTAATATTAACTCATAATTCAAATTCCATATATCTAAATCACCAAACTCTTAGTCTTTTATATAAGCAACTAATGTGAACAAGATGGCGAAGTATATCTACCATGTATGCATATGAACACACAAGTTTCAACTTGTGATATGTTTGAAAGTTGAACAATTTCAATTTGATCTTTGTTCATTTCACCATGCATGTTCAAACTCCTTTTGAATAGTAGCCACAATTTCAATTTGATCTTTGTTCCTTTTGATCACCTTGCATCCAAATTGATTTTGATTTGGCAGTAGTTAAGTAATACAATCCGAAGTGTAAATTATAAGATTCGAATCATGACTGTAACAGTTATCTCAGCAAGGCAAAGTGTGATAAACCAATGTCCTAAGACTCAGAGTAGTTCATGCACATAGCAGAAACAAGAGAAGGAAAGAGAAGAACAATGAATATGAAATTCATGTAGCATTTGGAATCAATGAATTGTGTATAGTTCTCAAAGTTGTGAACCATATATAATTAACTACCTCATAACAATGACATTGCAACATTCTCAAAAAAAATTAGACCTGCAATGTTTGCAGGTGCTAAGAAAGATGGGGCCAGTGTTCATTTCTTTCACAAAACAACTAATGCATATGCTACCGGACAACGAAAGCGTCGAAAGATTAATCATCAACTAGGCCTAAGTGAGGAGCATGTTCGCTGGCATAAGACCGGTAAGACCAAAGCTGTAACAGAAAATGGAGTACACAAGGGCTTCAAGAAGATCATGGTTCTTTACATAAGGCCTAAAAGAGGTGCAAAGCCGAATAAATCGAAATGGGTGATGCATCAGTACCATCTAGGGAATGATGAAGGTGAGAAGGAAGGTGAATATGTGGTTTCAAAGATTTTCTATCAGCAGAAGAAGAAAACTAAGAAGAATAAGCTGAATCCATTGGTGGCTGAAGATTCTGTCATGGCATTACAAGCAAGTCCAAGAACACCGAATCCAAATCCACCAAAACGGCCTCGGACAGGAAAATCTGTTGACTGTGATGACAATTTTGATGAAACTGACCTTATGCTATTCACTCAGGTAGGTAGCTATTGAAGGCTGATCATCCTTTTAGTGAACTCAAATGAAAAAGAGAAATGAAATACTCCCTCAATTCTATGAACATTTCTTGATTGTACTAATAGGCCTAGAACTAAGTTATGCTAAATTTTTTTACTATATACGCGTTTTGATTTGTTTTAATCGAATTTTCATGTGATGGACTAGGATGGAAAGCCTACTATTCATGGAGAATCTCTTGCCCCACCACCATCTGAAGTTCATGGTGATGAAAATAATGGAGGCTTTAACAACACTGCATTGTTATCTGTTGAAACACAACCTGTTGAAAACTCTGACTTTATTGGATTGGATGATATCTTACTATGCAAGGAACAGACATTAGATTCTTCTTCTGCTTACCTAAATGATTCTGGTTTGAAGTCCAACAATCTGAAAGGCTTTGCTTGCAATGCAAATGGAAATGCAGCTGAATTGTTTGGGGATGTGAATGATTGTTATGGAATTTCAGTGCTGGATAACCTGGGACTGGACAGTCCTCCTGATTTTGATCTTTCTGTAAGCACTCGATCACCCTTGTCGTCGTAGGGTATGTTCGGTTCGCAGTGTAAAATTAAAATGGAACGAAAGTTTTCATGGATGAGCACTGTATGTAAAAGTTTAAAAGATGAAAGTTGTTCCATGAAATTGTCCATCCTATTGTCACCATGCAACCAAACATATCATTAGCTTATATTCGATAAGTGTCTTAGAACAGAAAATACAAAGATACCGAAATAATTTTTGTTCTGTTATAAACACATTTAGTCATAGAGTAGAAATTTCTATAAATAAGTTTGTGTTTTCTAATATATCTGTATTTCTGTTCTAAGACAATCATCAAATATGACCTTTGTGGTTTTTTGATTATGTTAACATGCTCTCACTTATATGTGATGCAGAATTTGCAATTTTGTTCTCAAGACAATATCATTGACCTTCAATGGCTGGACATACCATAATGTATTTGCTGAACCAAGTTGTATTTGACACCAATGCTAATCCCAGAAGAGATATCATCATATAGTTGAATCATTTAGCTTAAGTAGGTGTAAGGTTTTTCTATATGTGAACTGTTTCACTCTACCATGTTGTATTGTGAGAAGTATTGTCATTGTTTGTCTGAGTTTAGAATTTAGAATTTAGAA

>AdNAC8

CATCAGTAATCACCAATATCATCAAGCACATAAGAACCAATTCAAGGCGGTGGTAATCATAAGCTAAGCCAAGGGAGATAAAACGTGTCCCGAAAGAGATACAAATAAAGTACTACTATGCTTTCTACCTTACTTTACCTTTCTTTTAACTACATACTTAACTTGTAATTTTTTCTCCTGTTCAATTTTTGGGTATTTTTTTTTTTACAATTCTGAGTATAAGGGAAGAAAAATTAGAGGGTGGTCTAATTTATTGTGATGGCATGGTGCAATGAGACTCATGAGAAAGAGATCATTGCTTCCAATAATAGTACTATTACTCTTAGACCTAAATCCGACCAAGAAATTCGAAACATAAGTTGCCCCTCATGTAGCCATAACATTCAAATAATCCAAGAGCAGGTACTCAAATTAAATTATCTTCTTTCTTTGTTTGTGGTCTTATGGTTTATTAAATAATAAATTGGTTTAATTGTTTTGATAGGGTGGAATTCATGAGTTGCCAGGGTTACCAGCTGGAGCGAAGTTTGACCCAAATGACATTGAAATATTGGAGCATTTGGAGGCAAAAGTTATGTCTCATGTGCCCAACCTTCATCCTCTCATTGATGAATTCATACCAACGCTTCAAGACGAGAATGGCATCTGTTATACACACCCAGAGAAGCTACCAGGTATTATTTTTTTATTTCTTCTATTTAATAATTTATTTTCCTAATTAATATAATTAGGACATAGGTTATTATAAACTTATTATATCATTTCTAATTTAAGGTAGGCATGCTGCACGCACGTCTTGCTGTGCACGGCATGCATGAAATTAATTACTCTTTTTGTAATAATAATAACGAGAATGATTTAATTTGATCTTCTCTTTGAAAAGAATCTAATAATTATTATATATGCACCACGGGATCTTAGAATTTTATTTAAATTATTGTGAACGCACGTCTTGCTGTGGACGGCATGCATGAAATTAATTACTCTTTTTGTAATAATAATAACGAGAATGATTTAATTTGATCTTCTCTTTGAAAAGAATCCTCAGGTTTAACCATGTAAGATGTCAAATGAAAACGTTATTAACCCTTAATTATGTTCTCAGCAATGTTTATATTTTACGTCAAATTTAAACATGCCATACCAAATAGAAGGTGGGGTGTCGGCCAAAATCTATATGTCAACACCAAAAAGTGGAGTTGCTGCCATGCTACTCTTCTATTTTCATGAGCCTTCGTTATACTCAATAGATATATATACCTACCTAGATTGTTTCATATATAGCTCCTATATATCCTATATGACAAATTTAAATTTCTATTGATTGATATCTTACTGCAGCATTATTTCATGCATGACCCTAAATTTATCCTTGTAACCAAAACGGGAGAGAAAAGAAAAGATTGATGATTTCAAAATTCAACTTGGTCAATGATCACCTAATTTAATTTTTAAAATAAATTAAATATAGAATAAAATTTTAATATACTAATTATATACATACATTTAATTACGTATTGTTACTTTAACTAAAAATTTAAAAACATTTTGAAAGGTGAGTACATCAAAATTAAACATTTAAATTTATTAATTTATTTTTCGATATCGATACATATATAATCTAATAAAAATGCTATTCGTAAAATCAGTTACTAATATATTTGTGTATAATACATGTGTGGTTTAATTATATTTCAACGTACATTTTATATTAAATACTGATTTTAGTGTACACGTAGTATAGTGATAATTCAATTATCTGATTCTAAAGTTAATGATTGATTATAGCATTATGAATGACGAAAATCATGTTAATTATACCATAACTATAAGGAAAAGTATAAGTAACCAACAACATTTTTGAATAATGTGTGAACAATGTGAATTAATAGGGTTAAAAGAGTAAATTAATCTTAAATTTAATTAATAGCCTTAAATTAGGGTGTAGTATATTTTTATTTGATTGGTGGTTATTTATGTTGTTTAAGATGGTCATTGTTTACCTAACACTACCCTAACTATAATAATGGTCCGTAACAAAATTCAAAGAGTACGCAAAGAAAACAAAACTTTAGTATGTATTTCTTGATTGATTGAATTGAATTGAATTATATTTTTGTAAACTATGAGGATAAAATTAAATATATATAAAGTATTGTCCTATAAAAGATAAAAGTAAATAAAGATAAGATAAGAACAACTAAAGATAAAATAAAAATAAGATAATAAAGACTAAAATTGTAATTGAATTTATGTATTCTGTTAGACTGAATTTGTAGGTCATAAGACTTCTTTATTGTTGTCATTGAATAAGGTGGAAGAGTAATTTAATAGTTTGAAACTTTTTAATTTTACACCATATCTCATCTCCTTTTATCAAGATTATTTGCATAAATTTCTGAACATAAGAGTTTCTTTATATAATAAGTATGAGACAAATGAAAGATTATTATTAAACTGTTCCTCTGTGAATGAATTAATGATGTAGGAGTAAAGAAAGATGGGCAGATTCGGCACTTCTTCCACAGGCCTTCAAAAGCATACACAACAGGAACAAGGAAGAGAAGAAAGGTTCACACCGATGAAGATGGAAGCGAAACAAGGTGGCACAAAACCGGAAAAACAAGAGCGGTGGTGGCCGGCGGCCTAGTCAAGGGGTTCAAGAAGATTCTAGTACTATACACCAACTATGGGAGGCAAAAAAAGCCTGAGAAAACTAACTGGGTGATGCATCAATACCATCTTGGAAGCAATGAAGAAGAGAGAGATGGAGAACTAGTAGTTTCAAAAGTGTTCTATCAAACACAACCTAGACAATGCGGCAATTCCATTGTTATAAAGGAAGATGATGATGATCTTCCCTATGGAAAGATATTGATGATGAATAACAGAAAGAAGCACAAAAATAATGATGATGCTGCTCCTGTTGTGGACTACTACATAAATTATGACCATGTTGAGCATCATCATAATCACAATCATAATAGCCAAAGATGTTCATCACCTACTCAACTTATTCCAAACTTGGTTCTCCAAGGTGATTCCTCTTCTCTTTTTCGCTTTGCTTCATCATCACTGGATGGGAATGCCAACAAAACAAGACTTTTTGAGAGAAAGTTGTAGTAATTCGTTCATTATTATTATTTCTTTGACTAAGTGACAATATTTATTCTATACATATATGATATATG

>ADNAC18

AGACTATATATGCATGAGATATAAATAGAGAAGAGAATAATAATAATATTCATTCACTGATCCATGATCATGGTGGATAATAGCACAGATTCATCATCAGGAGCGGGTGATCAGCATCATCACCCTCAGCTTCCTCCAGGCTTTCGATTCCACCCCACAGACGAAGAACTCGTCGTTCACTACCTTAAAAAGAAAGCTTCTTCTTCACCACTCCCTGTCGCCATCATCGCCGACGTTGATCTCTATAAGTTCGATCCATGGGAGCTCCCAAGTACCCAACTTTTCCACATGCCCAATTCGTATTAGTAGCATGTGAATTAAAGTGATTATTTTTAATCTTGATTTTGTGAATATATAGGTAAGGCAGCGTTTGGGGATCAAGAGTGGTACTTTTTCAGTCCTCGGGATAGGAAGTACCCGAATGGAGCTCGGCCAAACAGGGCGGCTACTTCTGGGTATTGGAAAGCCACCGGGACGGATAAGCCTATTCTCTCTTCTGATGGGAACAAGCAGAAAGTTGGAGTCAAGAAAGCGCTTGTTTTTTATGGTGGCAAGCCCCCCAAAGGTGTCAAAACCAATTGGATTATGCATGAGTATAGGCTCACCGATAACAATAACAATGCTTCTTCTTCTATTTCATCTAAGCCTCCTTCTATCCCTCTTGATCCACTCAAGAAGACTTCTCTCAGGGTATCTATATTATTATTATTAGTTTTTTAGTTTAGTTCCTTTAATTAATAATTTAACAAAATATTTTATCTCGTACTTTTGTAGCTTGATGATTGGGTTTTGTGCCGAATATACAAGAAGAGCAACAGCAGTAGTAGCAGCCTTCCAATTCCAAGGCCAGCGTTTTTAATGGATGAAGAGAAGGATCTAATTTCCATGGAGAACAGCATGGTGCCAACTATGTCAATGTCAAAACCAAGAAGCACTTCAACAACAGGTTGTTATGGACCCATGGCACTTGAAAACGATGACAACTTCTTCGATGGTATATTGGCAGCATCAACCGATCATCACACCATGCAAAATGGTACCAACCTTATTATTATTATTACCTTCCTTAATTACTTAATTCAAACAAATTAAACTTACTAGTTAATAATAATAATCCACAGGGTCTCCAGGGTCCTCATCTTCAAGCAAGAGATTCCATGGTGATCTTAATAATGGAGACAACACCTCCTTCGTTTCTCTTCTTAACCAGCTTCCTCACAACACACCGTTTCACCCAAACTCCATTCTTGGCTCCGTGGGAGACGCTGTCTCTTGA

>AiNAC52

GAAAAATATTCATTCACTGATCCATGATCATGGTGGATAATAGCACAGATTCATCATCAGGAGCGGGTGAACAGCATCATCACCCTCAGCTTCCTCCAGGCTTTCGATTCCACCCCACAGACGAAGAACTCGTCGTTCACTACCTTAAAAAGAAAGCTTCTTCTTCACCACTCCCTGTCGCCATCATCGCCGACGTTGATCTCTATAAGTTCGATCCATGGGAGCTCCCAAGTACCCAACTTTTCCACACCCACATGCCCAATTCGTCTATCGGTTTCGTATTAGTAGCATGTGAATTAAAGTGATTATTTTTAATGTTGATTTTGTGAATATATAGGTAAGGCAGCGTTTGGGGATCAAGAGTGGTACTTTTTCAGTCCTCGGGATAGGAAGTACCCGAATGGAGCTCGGCCAAACAGGGCGGCTACTTCTGGGTATTGGAAAGCCACCGGAACGGATAAGCCTATTCTGTCTTCTGATGGGAACAAGCAGAAAGTTGGAGTCAAGAAAGCGCTTGTTTTTTATGGTGGCAAGCCTCCCAAAGGCGTCAAAACCAATTGGATTATGCATGAGTATAGGCTCACCGATAACAATAACAATGCTTCTTCTTCTAATTCATCTAAGCCTCCTTCTATCCCTCTTGATCCACTCAAGAAGACTTCTCTCAGGGTATCTATATTATTATTATTATTATTAGACAAAATGGATGTTAAATATTAAGATTCTATCAGAATTTATTGTTTTTAGTCGTCACTTAATTATCAATTTATTTTTTTTAGAGAAATGCTATGAGTCAGTAATTTTTGTGATTTGTAACCATTAAATAGCCATCAATGATGGTTTTAATGGTGTGAGATTGGTATAAGATTTCATCCAATGGCTCACTTTTCTTTGTTGGTTATATGCTGGCCAAAATTTAACAAAATTGCTAGTCCCTAAACTTTTCCTTTTTTTTAATCTGCTGCTTTTAGTTAATAATTCAACAACATACTTTATCTGTTAAATAAATAATTAAGGTTGTTATGGGGTGTGTGTGGTTTTGTAGCTTGATGATTGGGTTTTGTGCCGAATATACAAGAAGAGCAACAGCAGTAGTAGCAGCCTTCCAATTCCAAGGCCAGCGTTTTTAATGGATGAAGAGAAGGATCTAATTTCCATGGAGAGCAGCATGGTGCCAACTATGTCAATGTCAAAACCAAGAAGCACTTCAACAACAGGTTGTTATGGACCCATGGCACTTGAAAACGATGACAACTTCTTCGATGGTATATTGGCAGCATCAACCGATCATCACACCATGCAAAATGGTAATTAATTATTACCTTCCTTAATTACTTAATTCAAACAAATTAAACTTACTAGTTAATAATAATCCACAGGGTCTCCAGGGTCCTCATCTTCAAGCAAGAGATTCCATGGTGATCTTAATAATGGAGACAACACCTCCTTCGTTTCTCTTCTTAACCAGCTTCCTCACAACACACCCTTTCACCCAAACTCCATTCTTGGCTCCGTTGGAGACGCCGTCTTGAGGCAACAATTTCAACTTCCAGGCTTAAATTGGAACTAATTAATTAATTAATAATAATATTTTGCGTTGCTTTGGTGGGGGGTATTATAA

>AdNAC38

TACCACGCAATCACATCATGTCACGGAGCTCACACTCTCTCCCTACTCCCTCATCGCACTGAATCACTCTCCCATATGAAGGTTCCCAGTCATTACTGCTAGGAGTCTAAGTACTACACCAAGCTTCCTTCTTTGACCATAACACTATCCAATTCCAACCCCCCAGACCCCCTTTCTTCTTTTTCACACCGCAATTTTTTCCACAATCGATCCCATTTTAAGCTTCTCTCTCCCTCTAGGTTTTTTTTTTTCATTCGACGGTTCATTCCTCGAATTCTCGTACCCCGAGGATTCGATTCGGGTCATGTAGTCGTAGGTTTTCGGTTTCTACAACCGCTTGATCCACAAAGCTCGGATTCTTTTTCTTCTTCACTTTTTTGGGGGGTTGTGAATTCTGATCCTCTATTTTTGGGGGTTTCGGTTCCGTGATTCAATTTCTTCTTCCGGATTCCTCTAATCGCATCGATTAACTGGGTGTTTGGTTTCGTAGCCTGTCGATTCAGAAAGTTTCGGTTTGTTCCTTTGGGTGTTTGGTCTAGGGTTTTTCGGATTGAGTATGGGTGCTGAAGCTGGTGCAACTGAGTGTTTCAGTAAGGCCATGGCGTCGATGCCTGGGTTTCGGTTCCATCCCACGGATGAGGAGCTGGTTATGTACTATCTGAAGAGGAAGATATGTGGGAAGAAGCTGAAACTCGACGTGATTCTAGAAACCGATGTTTACAAGTGGGATCCTGAGGAATTGCCAGGTATACTGTTATACTCTGTGAACTCAAGTGTTTCTGACTGTTACAGCGTTGACTTAGGGTTTGTGTTATTTGTGTAATTGTGTTCTTTTGTTTGATTCTTATCAAGTTGTTGATTGATACTTTAGGATGTATGTGTTTAAACTTGGGTCCTCGTGTTGCGAATTGTGTGATTGCTTGGGTTAGAATACTAATAAACCAATTATAGTTGATACTCTGAATGCTTGTGTTGAGATATCTTGACTGATTTGGTGCATATTCCATATAAATTGAATATATGGCTTTCGTGCGATGATCTGTCAGATTATGGCTTTCTTGGTCCGTCCAAACTGGGATTTTTTACTTACCATATAATTATGGTTAATAACGTTTGCTAATAATTTATCTGTTCATGGGATTTATGGTTTTGGTGTTCGAAGGTTGCAGTTACTCTTATGATGCGAACAAATTATACAGATAGGGTATGATGCTGGTACACTGCTTGCACCACTGTCAAATTTAGTCCAGATAGGGTTTTAAGTTGATATTAAAGATTGTATGGAACTTTTTTTTTCCCACTTTTCTTTATTTTCTTTTCGGTTGATTGCTGATAGTTTGTAATATTTTGCAGAGATATCTGTACTGAGGACTGGAGATAGGCAATGGTTCTTTTTCACTCATAGAGATAGGAAGTATCCTAATGGTGCGAGGTCCAACCGAGCAACAAGGCAAGGTTACTGGAAAGCAACAGGAAAGGATCGTAATGTGACCTGCAATTCTCGGTCAGTTGGAGTGAAAAAGACTCTGGTTTTCTATAGAGGCAGAGCTCCTAATGGTGAGCGGACCGATTGGGTTATGCATGAATACACCATGGATGAAGAAGAGCTGAATAGGTGCCAAGATATTAAGGTATCTTCCTGAAGTAACATTTTTTATTTAAATTTACTGGATGATTGTTTTCTATACTCTATTCTCATTCATCATGGAAAGAGCATTGCTTACTGATCATCTTTTTTTCGTTATCAGGACTATTTTGCACTTTACAAGCTATACAAGAAAAGTGGACCTGGTCCTAAAAATGGAGAACAGTATGGTGCACCATTTAAAGAAGAGGAGTGGGCTGATGACGAATGTGTAGATTTCAATATTAACTCAGCAGATCGGGAGGAAGTAAATACTGTCCCTGTTAATGATCAGCTGCCTCCTTTGGCCGATGATGAAGTCACGGATATGATTAATCAAATTTTGGATAATGAGCTTGCCCTTGACCAGCAATTTGGTGACGGCCTTGAATTTCCTCAGGTATGTGGTCATAACAAATTTTGTTACTTATTTAGTTTTGATTGCCTAACTTCTACTTTATATCCATAAATGATCGTCTGAATCGAATTCCTTATGAATCATATAGGTTGTTGCTGAAGAAACACAAAGTACTGTGGTGGATCAGTTCTCTGAGGCAGTGACGGACCCCGAGTACAACGATATTTACCACTCAACCAGTCAGCACTATGATGTGCAGAATGTCAACTTCAATCAGTCGGTTGCATCTCACCTTCATGCCCCGGAAGGATCAGAAGTTATTTCTACTGCCAACATTCAAGTAGAAGACTATAACTTTCAGGAGGATGACTTCTTGGAAATCAACGATCTCAATGGTAGTGAACTTACAATTCCAAATATGGAAACACCAGTGGAGAACCTGCAGTTTGAAGATGGATTGTGTGAACTTGATCTGTTCCAAGATGCAGAGATGTTTCTTCGTGACTTGGGACCAATCAATGAGGAAACCATTCCACATTCATATATGAATAATGCCGCTGGAAGCAACATTGAAAATCAGAATTATCACTTGCTGCCCAATCCAGAGGACGCTACTCAAAATGTTCATGAATTTTGGATGCATGATGAAAGAAACACCTCGAGTCTGTTTGAAGGCTTTGATGATTCTCTCTCTCAACAAAATCCAGGTATACTGATCAGTGCAAGACAGTGTTGCTCAACTCTCAAAAAATATTTTTAATGTCTTTGATTTGGATGGAGATGCAAGCTTCACATCTCATTTGTGCTGAGCTCTTCGCAGATTACTGCTCCTGGTTGACATGCCAGCTTATGCATGGCAGAAACCATTTAGGAGTCTAAATTTTGTTGCTCTATTGAGAGTTTCACAAACTAAGATTTCTAAATAGGGAAATGTTAAATTTGAGCCTGTTTAAAGCGGCTCTTATGTAAAAATAAAAAATAGGTCCTAGGCTCAGTAGGCTGTCTCTAATATTTGCTAGTTTCTGGTCATGTTTAGGTGCTGTATCTACTGAAGGCTATGATAATCAAAGTAGCATTGCAGAAGATGTTGCTACAAGTAGATTCTCTTCGGCTCTCTGGTCCTTTGTTGAGTCAATACCTACCACTCCTGCATCAGCTGCGGAAAATGCTCTAGTGAATCGGGCTTTGAATCGAATGTCTAGCTTCAGCCGAGTGAAGATTAATATCAAGCCGACGAACACAGCTGCAGGTAAAGACACTGCAACTACGAAGAGAGTGGGCAGAAAAGGATTTTCATTCCTTTTCTTCCCAATTATTATTGCTTTATGTGCTTTCTTATGGGTTTCTCTTGGAACTTTTAGATTATTAGGGAGATGCATCGCTCCTTGAATATGTAAATCAAAAGTTTTGATGCTATTATGCTATATATGTTCTATCTGGGATCTCCCCAAGATGACTGAGATAGTATTGTTCTAACATATTCAAAAAATAAAAAATAATATTTTATTCTTTGGGACTGTTAGAAGTTTTAAGTTTAGGCCATATTAGGACTATGATTGGGAGCAATGGTGGAGAGGGGAGGGTGAGGTGTGGCTGGGACCGGTGTTCGAAAACCGTATTACGGAGAGTTTGATGGGGAATGCAAAGCATGGCATAGGAAGGGCATGTGGGAACCCTTGATCAATGACCCCTCTGCAAATTGGAGGGGTGGATACAGAACACTTAGCTGTCTCTCCCAGGCCTTCATCATCTTCCAAACAATAAATCACCAGCCATTCCTTTCCACTCTTCCACTTCATCAAGGACCCTTCCCCTCAACCCCCTCCAAACTTCCAAACATAGCCTAGGTGAGTCTTAACAATCTTAATCCAAGAGAAAGGGGTTTTATATGAATTTGCAGGGTGGATTGGTAATGCACTAAG

>AdNAC16

GTTGGCGACTACTTTGTTAGTCTGATAAATAAAAAGTGAGCAAAAGCTAAAGGGGCCACTCACCCATCACACCCCAAATTCTCACCAAATATTGAATCGAATTATTCCTCTGATTCCTTCCTCATCTCTTCATTTGCTTTTATAATTCATCATCCCATTCTCTTTCGCTTCTTTTTTCTCTCTTTCGTTTCTCTCTGTTTTCGTCTCTGCTGCAAAATCATAAACCGGAAATATTAGAGCTCGTCCAATGGGAGCCGTTGTTGACTGTTATCCGCCGCACGCCGGCGAGGTTGCAGTTTTGTCTCTCAACTCGCTTCCCTTAGGTTTCCGATTTCGACCTTCCGACGAAGAGCTTGTTGATTATTATCTGAGACAGAAAATCAACGGAAATGGAGAAGAAGTCTGGGTTATTCGAGAAATCGATGTTTGCAAATGGGAGCCTTGGGACTTGCCAGGTATCAATCATAGATGAAGTTTCTTTGCTTTACTAATTTTCTGCTCATGTTCCCTGTTTCTGAATTTGATGACAATAACGTGATCGTAACTTCTTTTTATTTTGTTGCAGATTTGTCGGTGATAAGAAACAAGGATCCGGAGTGGTTCTTCTTCTGTCCACAGGACCGGAAGTATCCAAATGGTCACCGGTTGAACCGAGCAACCAATCATGGGTACTGGAAGGCCACAGGAAAAGATCGTAAGATCAAGTCAGGTTCCACCTTGATTGGGATGAAGAAGACTCTGGTGTTCTACACAGGTCGTGCTCCCAAAGGGAAGAGAACCAATTGGGTCATGCATGAGTACCGCCCCACCCTCAAGGAGCTTGATGGCACCAACCCTGGACAGGTACCTGTACCTATAGTTACACATGTATATGTATGTATATCATCTTTTTATGTATGTTGTGATTTACCATTGGATCTTTGTAGAGGTTATTGGGATCTTGTATTTCATTTTATATGTTCAATTTTTTACCACTATTATCATATCCACTGCCTCCTACAGGATATGTTCAAAAATGGTATACACCAAAATCGGCCACCAAACACCGCCACCATCTATTTGTGTATAAAGATGTGTTGTTTAACTTATTTTCAATGTGCATTTTATATTTTAGCATGTATTTTATACTGGCGGTGATTTTGGTAAATATTTAACATGGTTGGGATATGTTTATTGATTCCTACAAATCTGTTTATTTGGTTTGGTTTGTAACAGAATGCGTATGTACTCTGCCGATTATTCAAGAAACAAGATGAGAGTCTTGAGGTTTCAAACTGTGATGAGGTGGAACAAACAGATTCCGCTCCCATGGCGGCCAATTACTCCCCTGAAGAAATACAGTCTGATCAGGCTCTGGCTGAAGTATCGCCGTCTCAAGTTACAGATGAGAAGCACCAGGGTGTTATCCCTGAGATCTCTGAGGAAGCGGTTTCCAACGTTATAACCTCTGCTGATTGCCATAGTGACGGATATGATGCTTGTGAAAGGCGAAATCAAGCTTTTGAACTACCTGCTGAGGTTAGATTTGACTGACTCAGTTTGAATATTTTACTGGATCATCATCGTGTTCTTTCGGGGGAAAAAATAAATCCTAAAGTCACAATGGAGAATTCTGACTACCTAGAGAATAATTTAGAGTGATTTCAATGTTCACTTGCTATGAGAATAGCAGTTTATCTTTCAGTTAGGTTTCAGTATACATTACTTTAAAATAATGTTTTTATCACGATAAAAAAATTGAGTATCTCCACTTCTCTATATGTATGGATTTATTAGTGTGTATTTTATTTATTTTAGTAACTGAATTTATGTTTCTGACAGGACATTCCGCCGTTGAATTGGGACATATTCAATGACCCCGAGGACAAGATATTTGATGACAAATTATTCTCCCCAGTCCATAGCCATATTCCACCAGAATTTTACTACCAAGCAAACAATGAGACAAATATTGCAGACATCTTAAATTCTGTCAATTGGGATGAGATCTCCTATGAGGATCCCTATAGTCAAGCACAGAACAACTTTTTTAATAATGTTAAGCAAAGTGTATCAGGTAGCGAACCAGATGCAGGGCTGACCAATATGACAGTGAGTAACAGCTCTTTTGCTATTTATATTTTGGTCATTGTATTGAGTGTGGTTCATCAACCCTGAGCAGCAGCTATATTATTTAGCATTGTTTCTTGAAAATTCTTTATAATTATAGTTCTCTGTATTCTTCAGTGCATACACCCGACGAATGTTGTTTATCCCGAGGAGGCAATTCACAGAAAGGTTGCTTTGGCAACAACTCCGCAATTTTGCAGCACCTTCACGTCTGACTTCAGTGCTGATGAGCAGAAGAGCAGTGTCGCGTTAATTCAAAACAATTCCCAGATGGCTTCTTTTCCGGATGCCAGAACAGGCCAAGTGTATAACGTATTCAATGATTATGAGCAGCCGAGAAACCTTAATACCTATGTTAGTGGTGATACTGGAATCAAGATAAGGACTCGACAAGTGCGAAATGAACAACCAGCAATGATCTTTACAGATCAAGGTAATGCAGCAAGGAGAATCCGATTGTTAAAGCAGTGTGCAAATGTCTCAAACAAGATGGCAGATGATGGGAGTCCTAAACAAGAGCATGATTCAAAACCAATAATTGCAGGGGTAAGAAGCTAATTACGTTTATTTAGCTTTGGTTTATATAAAGAATTGTCTGTGCACGTGTTCATGAGATTTGCTTTTTCGCTTTTGTAGAACAAAAACAAAACTTTCAAAAGTCACACTGCAGATAAGCATGATACTGCTAATGATCTGAATGAACGCCAGGAGAAAACTGAGTCAACTGATAAAAGAAACATGATATCTAAACTTGCAAAAGGAGGTTCTTCCATGTTGGGGTTGAAGGGATTATTGCGCAGAAGGCTTAGTTACATATCAAAGGCCTCCTCCAATTTCAAAATGTGGTCATGTGTTGTTGTGGCTTCTGCCTTTGTATTGGTCTCGTTTCTGTTCTTTGCTAACATATGGGGATATATTAACTTATGAACTTCTAGGAGATCATTCCTTTATGCGTGTAATGTGCCTCCATTTTTTTTTTCCTTTCTTTTTTTAGGGCCAGTTGTGGAGGCTTTTAGGATTGACTCCTAGATTTGTTGATAACTCTATTATGAGAGAGTATGCGTTTCAATATGTAGCTGTATAATATACTTAGTTGCTTACCTCGCTGGCTAGTAGAATTTAGATCAACACTTGTGCTACTGTTTTCTTCTCTCTTAATTACTTGCTTACTGCCTATGAATGCCAAGAAAGTTATTTGTGAAATAATGATGAGAGT

>AiNAC6

GCAAAATCATAAACCGGAAATATAAGAGCTCGTCCAATGGGAGCCGTTGTTGACTGTTATCCGCCGCACGCCGGCGAGGTTGCAGTTTTGTCTCTCAATTCGCTTCCCTTAGGTTTCCGATTTCGACCTTCCGACGAGGAGCTTGTTGACTATTATCTGAGACAGAAAATCAACGGAAATGGAGAAGAAGTCTGGGTTATTCGAGAAATCGATGTTTGCAAATGGGAGCCTTGGGACTTGCCAGGTATCAATCATAGATGAAGTTTCTTGGCTTTACTAATTTTCTGCTCATGTTCCCTGTTTCTGAATTTGATGACAATAACGTGATCGTAACTTCTTTTTATTTTGTTGCAGATTTGTCGGTGATAAGAAACAAGGATCCGGAGTGGTTCTTCTTCTGTCCACAGGACCGGAAGTATCCAAATGGTCACCGGTTGAACCGAGCAACGAATCATGGGTACTGGAAGGCCACAGGAAAAGATCGTAAGATCAAGTCAGGTTCCACCTTGATTGGGATGAAGAAGACTCTGGTGTTCTACACAGGTCGTGCTCCCAAAGGGAAGAGAACCAATTGGGTCATGCATGAGTACCGCCCCACCCTCAAGGAGCTTGATGGCACCAACCCTGGACAGGTACCTGTACCTATACTTACATATGTATATGTATATCATCTTTTTATGTATGTTGTGATTTACCATTGGATCTTTGTAGAGGTTATTGGGATCTTGTATTTCATTTTATATGTTCAATTTTTTACCACTATTATCATATCCACTGCCTCCTACAGGATATGTTCGAAAATGGTGTACACCAAAATCGGCCACCAAACAGCGCCACCATCTATTTGTGTATAAATATGTGTTGTTTAACTTATTTTCCATGTGTATTTTGTATTTTAGCATGTATTTTATACTGGTGGTGATTTTGGTAAATATTTAGCATGGTTGGGATATGTTTATTGATTCCTACAAGTCTGTTTATTTGGTTTGGTTTGTAACAGAATGCGTATGTACTCTGCCGGTTATTCAAGAAACAAGATGAGAGTCTTGAGGTTTCAAACTGTGATGAGGTGGAACAAACAGATTCGGCTCCCATGGCGGCCAATTACTCCCCTGAAGAAATACAGTCTGATCAGGCTCTGGCTGAAGTATCGCCGTCTCAAGTTACAGATGAGAAGCACCAGGGTGTTATCCCTGAGAACTCTGAGGAAGCGGTTTCCAACGTTATAACCTCTGCTGATTGCCATAGTGACGGATATGATGCTTGTGAAAGGCGAAATCAAGCTTTTGAACTACCTGCTGAGGTTAGATTTGACTGACTCAGTTTGAATATTTTACTGGATCATCATCATGTTCTTTTGGGGGAAAAAATAAATCCTAAAGTCACAATGGAGAATTCTGACTACCTAGAGGATAATTTAGAGTGATTTCAATGTTCACTTGCTATGAGAATAGCAGTTTATCTTTCAGTTAGGTTTCAGTATACATTACTTTAAAATAATGTTTTTATCACCATAAAAAAATTGAGTATCTCCACTTCTCTATATGTATGGATTTCTTAGTGTGCATTTTATTTATTTTAGTAACTGAATTTATGTTTCTGACAGGACATTCCGCCGTTGAATTGGGACATATTCAATGACCCCGAAGACAAGATATTTGATGACAAATTATTCTCCCCAGTCCATAGCCATATTCCACCAGAATTTTACTACCAAGCAAACAATGAGACAAATATTGCAGACATCTTAAATTCTGTCAATTGGGATGAGATCTCCTATGAGGATCCCTATAGTCAAGCACAGAACAACTTTTTTAATAATGTTAAGCAAAGTGTATCAGGTAGCGAACCAGATGCAGGGCTGACCAATATGACAGTGAGTAACAGCTCTATTGCTATTTATATTTTGGTCATTGTATTGAGTGTGGTTCATCAACCCTGAGCAGCAGCTATATTATTTAGCATTGTTTCTTGAAAATTCTTTATAATTATAGTTCTCTGTATTCTTCAGTGTGTACACCCGACGAATGTTGTTTATCCCGAGGAGGCAATTCACAGAAAGGTTGCTTTGGCAACAACTCCGCAATTTTGCAGCACCTTCACGTCTGACTTCAGTGCTGATGAGCAGAAGAGCAGTGTCGCGTTAATTCAAAACAATTCCCAGATGGCTTCTTTTCCGGATGCCAGAACAGTCCAAGTGTATAACGTATTCAATGATTATGAGCAGCCGAGAAACCTTAATACCTATGTTAGTGGTGATACTGGAATCAAGATAAGGACTCGACAAGTGCGAAATGAACAACCAGCAATGATCTTTACAGATCAAGGTAATGCAGCAAGGAGAATCCGATTGTTAAAGCAGTGTGCAGATGTCTCAAACAAGATGGCAGATGATGGGAGTCCTAAACAAGAGCATGATTCAAAACCAATAATTGCAGGGGTAAGAAGCTAATTTCGTTTATTTAGCTTTGGTTTATATAAAGAATTGTCTGTGCACGTGTTCATGAGATTTGTTTTTTGCTTTTGTAGAACAAAAACAAAACTTTCAAAAGTCATACTGCAGATAAGCATGATACTGCTAATGATCTGAATGAACGCCAGGAGAAAACTGAATCAACTGATAAAAGAAACATGATATCTAAACTTGCTAAAGGAGGTTCTTCCATGTTGGGGTTGAAGGGATTATTGCGCAGAAGGCTTAGTTACATATCAAAGGCTTCCTCCAATTTCAAAATGTGGTCTTGTGTTGTTGTGGCTTCTGCCTTTGTATTGGTCTCGTTTGTGTTCTTTGCTAACATATGGGGATATATTAACTTATGAACTTCTAGGAGATCATTCCTTTATGCGTGTAATGTGCCTCCATTTTTTTTTCCTTTCTTTTTTTAGGGCCAGTTGTGGAGGCTTTTAGGATTGACTCCTAGATTTGTTGATAACTCTATTGTGAGAGAGTATGCGTTTCAATATGTAGCTGTATAATATACTTAGTTGCTTACCTCGCTGGCTAGTAGAATTTAGATCAACACTTGTGTTACCGTTTTCTTCTCTCTTAATTACTTGCTTACTACCTATGAATGCCAAGAAAGCTATTCGTGTAATAATGATGAGAGTAAAGCACTAAAAATGTTCTTTAATTATCAAAACATTAACAGAAATGTATTAATAATTAAAATATCCTCAA

>AdNAC32

TGGGATGTCAGAATGGGGTGATAGATAAAAGCGGAAGCACTGACTAGTTGCCATCGAAGTAGATAACATGCGAATGCCACGTCATAAGAATGACACCGAACCTGCTTGATTTTCTAGCCGACCCTGTTTCACTCAAAGTCTAAAGCCTAAATTGCCATACCAAGTTGTTGTTACTCACAAACAAGTAAAGAACAGAACAACATAACCCTCTCTGACACCAAACCCTTTTATCTTCACCTTCGAATCATGGTGGACACGGATTCAAGCGAAGCACACATGTCAATAGCAGCTTCTTCCATATTCCCTGGCTTCAGGTTCTGTCCCACTGACGAGGAGTTGATCTCTTATTACCTCAGGAAAAAGCTGGACGGTGATGAAGACAGTGTTCAAGTCATTTCGGAGCTTGAACTTTGCACTTTTGAGCCTTGGGATTTGCCAGGTCAGTCTTGTTCTTTCTCTTTGAATGATGTATCTGCTTATTTTTTGAGCCACATAAATATTTATTATAAGAATGTTTGAAAACAGGAAAATCTTTCATTCAATCAGACACCGAGTGGTTTTTCTTCTCGCCACGGGGGAGAAAGTATCCCAATGGTTCACAGAGTAAAAGGGCAACTGAATGTGGGTATTGGAAGGCCACAGGAAAAGAACGCATCGTAAAATCCGGTCAGGATGTTATTGGTACCAAACGCACTTTAGTATTTCATCTTGGTCGAGCTCCTAAAGGCGAAAGAACTGAATGGATTATGCATGAGTACTGTGTCAATGACAAATCTCAGGTTCATTCGTTCATATTTTTCTTCTATTGATTGTGTAAGAGAATTCATGTATTATTAAGATTTAAACCAAATCAAAATTTGATTAAGTAGCTGTGAATTAGTTAGTTAGAGTGAATCTTGATTTCATTTTTGAGAGTACATAGCACTTTGCATGTGTTAATTTTCCGTATAAATAGCTATGTATAAGATTTGTTTAAATACTAATTTAGCATATATTTTCATAAAAACATTTCTGTACTTCCCAATTCACTACCTTTACATGACATCAAACCTGGTATTCATTTTGTCTTCTGAATTAGGATTCATTGGTGATTTGTCGGCTGAAGAAAAATACAGAATTTCGTGCTAATGATCATTCTAATAAAACTTCACATGATAGTGATTGTGGAGTCTCAGAAGAAGTTACAGTTCAAGGGGGCACTTATGTGCCTATTCAAGATAAAGAAACTGGATGCAGCTCCAAGAGAACTAGCAGTAGTAATAGTTCTCCTTCTACTACTACTGGCCAAATTGAATCCAGTCATAGAGTTGTCAATGAAGCCAATCAAGCCAATGAAGTCAACATAGCAAATGAAACCAATCAAANNNNNNNNNNNNNNNNNNNNNNNNNNNNNNNNNNNNNNNNNNNNNNNNNNNNNNNNNNNNNNNNNNNNNNNNNNNNNNNNNNNNNNNNNNNNNNNNNNNNNNNNNNNNNNNNNNNNNNNNNNNNNNNNNNNNNNNNNNNNNNNNNNNNNNNNNNNNNNNNNNNNNNNNNNNNNNNNNNATAGGTAATGAAGTCAATCAAGCCAATGAAGTCAATATAGTCCATGAAGCCAATCAAGCCAATGTAGCCAATCAAGCCAATCCCAGAAATCACCCTCAGGTAACTTGAACAATTGCATAAGAAAAACAGAAAAATGATTTGATATGTTGTGATGTGTGGTTGGAAAGTGTGATGGAATTGAATAAGATTATGGTAACATGTAGGTGGACGAAGATGATTGTTATGCAGAGATCCTAAATGATGATATCATCAAGTTAGATGAATCAACACTCTCACGGCCATCGCCACCACAAGGGACAGCAAACAGGAGAATCAGGCTAAGGGTTCCCAAATCAACAGTTCCCAAATCAAGGGTTCCCAAATCAACGGTTCCAACAGGAAATGGTTGCCATTGCTCCAAGCAATCATCAAACAAAATTAACACCTTCCTGCCATATGCTTTGGTGGTCTTCACTTTCTTCGTTTTCACTTTGTTAGCTCTAGGCTTCTTTCTTATTATTAGGAGGTCCCAAACTACTGCGCAATATTCTCGAGACCTCTCTAGAGTTAATTAAGACGATTAATTAATTAGTGTTTAGTGTTGCATAGCCTTTGGATCGATGTTAGTATGTTACGTTGTATACAAAATTACAAACAAATATGCACTCTTCATAGTGAAATAGTAAGGATAAGGA

>AiNAC44

CTTCTTCTCATTCATTCATGCCCCCTTCCCCTCTTGGCCTAATCTCTTTCTCCACATGGCGACAAATTCCGTCAACCATAAAATCCCCCTCACTTCTCACCTCCCCAAAAATTTCAATCCTCTCTTCTTCCCTTTTCCTAGACTTTAGTTTGCTACACCAAACTTCTCTCTCCCTTCTGTTATTCTTATTATTCCTCACCAAGCCTATAACAAATATTTTGTTACACCAAATTATTAGGCTCTCAAAAATGGGATCACCGGAATCAAATTTGCCACCAGGTTTTAGGTTCCATCCAACCGATGAAGAACTCATTCTTCACTACCTTAGGAAGAAGGTAGCATCCATACCCTTACCTGTTTCCATCATCGCTGAGGTTGATATCTACAAATTGGATCCATGGGAATTACCAGGTATAATATATATATATAATAGCATTTTTTTTTTGTTTCAATTCTTGTTAGTAAGTATTTTGTGTTTGTTATGAGGATAAGATTGTGCCATGTCGTGTCATAATATAATCAATGACGCAACGTATCATTGATTTTTATTGTATATTATAGCATACAGTGTTGTGATGTGGAAGCTTATTATTATTGGTTTGTTGTGGTTTCTTTATCACCAGCTAAGGCTGCGTTTGGTGAGAAAGAATGGTACTTCTTCAGTCCAAGAGACCGGAAGTACCCAAACGGTGCGAGGCCAAACAGGGCAGCTGCTTCAGGGTATTGGAAGGCTACGGGTACCGACAAGACCATAGTGGTGTCGCCGGCGGCCACAGTTACACGTAGAGTAGGCCAAGAGAGCAGCGTTGGTGTCAAGAAGGCTCTTGTTTTCTACAAAGGAAGGCCTCCAAAGGGTGTTAAAACCAATTGGATCATGCACGAATATCGTCTTGTAGACAACAATAGACCCATTAAGCTCAAAGATACCTCCATGAGAGTAAGTTTACTAACTACCCTTCCTCTTGATATTTTTTTTTACAAACAGGTGATTCGAATCCGAAACCTCTTAAGTGAGTATAGAGAGATCATGCCATTTGAGCTATAACTGATTTGCCCAATTTGCTAATTCATGTGCCTTTGTGCAGTTGGATGACTGGGTTCTGTGCCGGATTTATAAGAAGTCGAAATTCTCAGTATCTTCACCGGAGGAATCACCGTCGAGTGAAGTTCAGGCTGCAGAAGAAAATGGTTTATTCAAGAACACCATTTTGAAGAGTCCAATTCCAACACTGTCGCCGTCGCCACCGCCGCCGCTGCCGCCGCAGCCACTTCTCTCTCAAAAATCTGTGTCATTCTCAAACCTCTTAGATGCCATGGACTACTCCATGCTCAGCACCATCTTATCTGAGAACAATAACAACAGCACCCTTGATCAGGAACAATACTCCCAGATCAACACCAACCAATTGAACCATTCATCGAACATGGAGAACACTAGTAACAGCAACATGATGCTGATGAGGTCAAAGCGCCAGATAGACGAGGAAACAACAACGGTGTTGCACCCATCAAAGAAGTTCCATCACCAACTTATGGGCTCTTCTTCTTGCAGCTTCCCTAATAACATTAACAACACAAACACTGCACAATACGAGAACCCGCAATGGAACTACCTTGTCAAGCAATCCTTCTTGAACCAGCACTTACTTCTCGCTCCTCATCTTCGATTTCAAGGATAA

>AdNAC46

ATGAAGGTAGTTATAGCTAGGTTACTTAAACAGTAGTGAAGCAACGCACAATAATTTGACCTTAATCAATTACATTACATATATAGCAGCTAACTTGTATTGTTAGCTTCAATGGCCGTCTATCTAGCTACCAAGCTTTGTTGTTTGGCCCTACTTAATTCCTTATGATGGAATTCCACGTACAGCCGTCTTTCTTCAAACAGCTCAACGCTTTTTTTGAATCCTAATCAATCCAACCATAAATCTCTCTCTCTCTCTCAGTCTCTCTGTGTGTATATATATATATATATATATATATATCCTCCTGGGTTTTGCTTCTCTCCAACAGATGAAGAACTCATCCTTCACTTTCTTTATTCCAGAATTTCTCTACCATTCCATCCCAGCATCATACCGGACCTTGATCCCTCTCAACTTCATCCATGTCAATTAAATGGTACATACATACATAATAATCTATGCATGCATGCATCCAATTCCACATACACATATAACATGGAGTTTGAAATTTAATAAAATATATTTTTTTTTATATGTACTTATATATATATACACAAACACAGGTAAGGCGTTTTCAAGTGGGAATCAACACTATTTCTTCACCAATAAAGTGAAGGAAAACAGAAGCACAGAAAATGGGTATTGGAAGGAAATAGGTTTGAGCGAACCTATAATCTCAGCTGATGCAAACAAGAAATTGGGAATCAAGAAGTATTTTGTCTTCACTCTTAATGAAGGCACAGAAACCAATTGGGTCATGCAGGAATACCATATTTCCTCATCTATGTTCCACAACCCTATTTCATGTTATGCAAATGGAACTGCTCACCGAAGATTATTAAGACCTGTAAGCTATGGTTTTAATTTCCCTACCATATAGCCCTAGATATTATTTCAATAATAACCTCTCTATTAATTTGTTTGTTATTGTGTAATTGTGCAATTTATTAATGATTATATTTATAATATTACAGGATCAAAATCAGAACAATAAATGGGTTTTGTGCAGAGTGTATGAGAAGAACAAGTCACAATCACAACAAGGTGCAACTGCAAACTCCTACTATAGCGACGAGGATGATTGTGGATCAGAACTTTCATATCTAGATGAGGTTTATCTGTCACTTGATGATGATCTTGAAGTCATAAGCCCCCCTAATTAA

>AdNAC40

ATGAATAAGATGGATCTGATAGATGCGAAGCTGCAAGAAGAGCATCAATTGTGTGCATCATCGTGGAAACAGTGCCCCGCTTGTGGACATAAGTTTGAAGGCAGCAGCGGGAAGAAGGGGGAGTGGGAGTGGGTAGGTCTGCCAGCAGGAGTGAAGTTCGATCCAACAGACCAAGAACTGATAGAGCATCTAGAAGCAAAAGTAGAGGCAAAGAGATCGCACCCTTTGATCGATGAGTTCATTCCCACCATCGAAGGAGAAGATGGAATCTGTTACACCCATCCCGAGAAACTTCCAGGTGTGACGAGGGATGGGTTGAGCAGACACTTCTTTCACAGGCCATCAAGGGCGTACACCACTGGAACACGGAAGAGAAGAAAGATTCTTCAAAACGATGAGGCAGAGGCCGAGAGAGGAGAGACACGGTGGCACAAGACCGGTAAGACAAGGGCCGTTATGCTCAAGGGAAAGCAGAAGGGGTGCAAGAAGATTCTGGTGCTGTACACCAACTTCGGCAAGAACAGGAAGCCCCAGAAGACCAACTGGGTCATGCATCAGTACCACCTCGGACTCCATGAAGAGGAGAAAGACGGGGAGCTCGTCGTCTCTAAGATTTTCTACCAAACTCAGCCCAGGCAATGCAGTTGGTCTTCTTCTTCTTCTTCTTCTTCAATTACTGCTGCTGCTCCCCCTGTCAAAACTAATAATGACACTTGTCCCGTTCTTGGATTCCCTCCTATGGAACATTTCAGCAGCTTCATCCCTCTCAGAAAAACCCTCCATAATGAGGTGATTAATTTATCATAACAACTATATATATATAGTAGAAATATTTAATAACAACAAAAATTTAACCAAAAATAATTAGAACTTGTTTTATTTAATAGGGGAAACTTGCACACCAGCGTCACATATTCCTTCATCAAATCCTCTTGGAGTCTTCCATCACAACACTTCCATCATCCTTGACGACCTTATCTCCGCTAGATTCATGACTCCTCCTCCTCCTCCTCATTTCCACCAGGTAGGTACTTTCTATGTGCAGATTGGTTATTGTTGTTGTTGAGTGCCACGTTGACCTTTGAAAATCCACATGATGCAGCAGCATGATAATAAAGTAGTAGGAGGAACCTCTGCTTCTGGTTTAGAGGAACTCATCATGGGCTGCACTTCAACTTCAACCACTCATAATATCACCAAAGTAAGAACAATGAGACTAATTCACTCACCTCTCTTCACATCAACTTTGTCACCTATTATGCGCACTATACAAACTATATCACACTAAACATGGTCCAGTTTTTTATTTATTCTTGTTTTGAATTAAAGACTGGAACAATTCAACGTGCTGCTTCTCTTCTCTTCTTGTTTCTGTATCTAAGAACAGACTATAATCTTTTCTTTACACAATAATGGAGGAAATTAAAAGAATGATTACATCCATAAAAATAAGAATTCCCAATACAATAACTTGTTATATGAATTTCTTTAAGTTACATTAATACCGTGTGTATACAAGGTCATCAAACCACGTCACACATTATTGCACCATTTACTTTGTCATACTTGTTTTAATCTTTCTACATCAAAGAACTCTATGTCATATTTATTGTTTAATATTAGATTATTTTACAAATGAAATGATTGATTAAAGAATTATTATTTGTCATCTAAACTTTATTACTAAGTTTGAATATGAAAGAATATATTCCTACCTTTGTTAATAGCTTACGGGGTTAATTTTGCTTTATGTTGATGTTATATTTCTTTTTTCCCATTGTAAAGTAATAAGAATATATCATATCCAGAAGGAAATAAAGAAAAGAAAAAAGTAAGGAGAATATGTAGACTAATAAATCCGTCAACTATGGTTATTTGAAGGAAAGAGATCTCCAAGACAAGGAGGGAAAGAATCCTTCTCTTTATTAATTTTCATGTTTGAACTCTCTAAGTGCCAATTGAAAAAATTAAAAAACAAACAAACACACACATGATTATGCAACATCTAGCTAGTAGGTGGCAATAAAAACATCACTACTGCATGAGAATAAGTAGATTGTTAGAAAGAAATGATTGTTTAATTTGGATGAGCTTCAATGCAATTGGAAGTGTGCAGAAGGCTAACCCCTAAATTAAATTACATTGCAGGAGGCATCAATGACAAACACAAACCCACAAGAAGCTGAGTGGTTGAAGTACTCTTCTTATTGGGCTGACCCTCAGCCTCAGCCTCAGCCTCATCTTCATGGGTAATAATAACAGACCCCATTTGGCAAAGTAATCATCCACACTTACGCTTTCCTCTTTTTTTTTTTCTTTTTTTTTTTCATTAATGCAAATATATATATAAAAGGGCGTGGCTTTTCGAGTCTCAACGGAAATAACTGCTGCATGTGTGCACTGTAACGGCACATACATCAAGTTGGAAACAAACAACAAAGGAGGGAAGAACAAATTGAATCAAATTCAAGAAAATGAAAATAACCGCAAGAAAATTAAAGCTGCTGTTCTACCTCCTCATCACACAAAGAAACAGTTTGATAATGCATCAAA

>AiNAC4

CACATAATTAAACCGAGCAGCCTAGCTATTACTACATACATCCGCTTCATAAATTAGCAAAGTCTTAAAGGTGCTATTCTGTGCTATGTGTTTGAAGCCACACCCAACTTTTCCTTTATTATAAATTTTTATTCGTCGCTGCAGAGAGAGAGAGAGAGAGAGAGAGCTCTATGTGGTTTATGCTTAATTTCTATGGTTAAAGCAGCAGGTTTATTGCTCCTCCAANNNNNNNNNNNNNNNNNNNNNNNNNNNNNNNNNNNNNNNNNNNNNNNNNNNNNNNNNNNNNNNNNNNNNNNNNNNNNNNNTTTTTTTTTTTTTACATCCTTCCCTTCTCTCCTTTAATTTCTAGGTTTCCTGCATATAGTGCATACTTAATTTCATATATTCAGCACAAATTAATTTTCTGTTGATACTCTAAATAAAATATATATGTAGCCTAGCTACCACTGGTTGCTTTTCTTGAGAAAAAGTTATTAGTATTTTCGATTAAGGTATAATAATAATTTTGTTTTTATTATTCGTTGTAGCCTGGAGCCACTAGCTAGTTTCTAAATAATCGAATCGTTATATGATGAAGTAGATGATTGGAAGATGTGATCCAGTTCCAATATATATTAATCAAAGTGTTGGATTTGTGAGTTATAGATAGAAGAGAATGATCATGAGTAGTAGCAGTAGTGGATTGTTGCATAGAAAAAGAATTGAAAATAACAAGGAAAGAAGTAGAGTCGATGAATAAGATGGATCTGATAGATGCGAAGCTGCAAGAAGAGCATCAATTGTGTGCATCATCGTTGAAACAGTGCCCCGCTTGTGGACATAAGTTTGAAGGCAGCAGCGGGAAGAAGGCGGAGTGGGAGTGGGTAGGTCTGCCAGCGGGAGTGAAGTTCGATCCAACAGACCAAGAACTGATAGAGCATCTAGAAGCAAAAGTAGAGGCAAAGAGATCGCACCCTTTGATCGATGAGTTCATTCCCACCATTGAAGGAGAAGATGGAATCTGTTACACCCATCCCGAGAAGCTCCCAGGTGTGACGAGGGATGGGTTGAGCAGACACTTCTTTCACAGGCCATCAAGGGCGTACACCACTGGAACACGGAAGAGAAGAAAGATTCTTCAAAACGATGAGGCGGAGGCCGAGAGAGGAGAGACACGGTGGCACAAGACCGGTAAGACAAGGGCCGTTATGCTCAAGGGAAAGCAGAAGGGGTGCAAGAAGATTCTGGTGTTGTACACCAACTTCGGCAAGAACAGGAAGCCCCAGAAGACCAACTGGGTCATGCATCAGTACCACCTCGGACTCCATGAAGAGGAGAAAGACGGGGAGCTCGTCGTCTCTAAGATTTTCTACCAAACTCAGCCGAGGCAATGCAGTTGGTCTTCTTCTTCTTCTTCTTCTTCAATTACTGCTGTTGCTCCCCCTGTCAAAACTAATAATGACACCTGTCCCGTTCTTGGATTCCCTCCTATGGAACATTTCAGCAGCTTCATCCCTCTCAGAAAAACCCTCCATAATGAGGTGATTAATTTATCGTAACAACTATATATTGCAACAATTAATGAATATTAAATAAAAAGTTTTGACTATTTTTTTTTTATTTCTCTAACATTACCAATATATAGTGACTTGGGTGCATGTTAACATAGAGCAATGACCTTGTAGCAGGAAGTTGGAATAGGAGGGGAAACTTGCACACCAGCGTCACATATTCCTTCATCAAATCCTGTTGGAGTCTTCCATCACAACACTTCCATCATCCTTGACGACCTTATCTCCGCTAGATTCATGACTCCTCCTCCTCCTCCTCAGTTCCACCAGGTAGGTACTTTCTATGTGCAGATTGGTTATTGTTGTTGTTGAGTGCTACGTTGACCTTTGAAAATCCACATGATGCAGCAGCATGATAATAAAGTAGTAGGAGGAACCTCTGCTTCTGGTTTAGAGGAACTCATCATGGGCTGCACTTCAACTTCAACCACTCATAATATCACCAAAGTAAGAACAATGAGACTAATTCACTCAGCTCTCTTCACATCAACTTTGTCACCTATTATGCGCACTATACAAACTATATCACACTAAACATGGTCCAGTTTTTTATTTATTCTTGTTTTGAATTAAAGACTGGAATAATTCAACATGCTGCTTCTCTTCTCTTCTTGTTTCTGTATCTAAGAACAGAATATAATCTTTTCTTTGCACAATAATGGAGGAAATTAAAAGAATGAATTACATCCAAGAATAAGAATTCCCAATACAATTATTTGTTATATGAATTTCTTTAAGTTACATTAATACCGTGTGTATACAAGGTCATCAAACCACTTCACATATTATTGCACCATTTACTTTGTCATACTTGTTTTAATCTTTCTACATCAAAGAACTCTATGTCATATTTATTGTTTAACATTAGATTATTTTACAAATGAAATTATTGATTAAAGAATTATTATTTGTCATCTAAACTTTATTACTAAGTTTGAATATGAAAGAATATATTCCTACCTTTGTTAATAGCTTACGGGGTTAATTTTGCTTTATGTTGATGTTATATTTCTTTTTTCCCATTGTAAAGTAATAAGAATATATCATATGCAGAAGGAAATAAAGAAAAGAAAAAAGTAAGGAGAATATGTAGACTAATAAATCCGTCAAACTATGGTAATTTGAAGGAAAGAGATCTCCAAGACAAGGAGGGAAAGAATCCTTCTCTTTATTAATTTTCATCTTTGAACTTTCTAAGTGCCAATTGAAAAAGTTAGAAAACAAACACACACACATGATTATGCAACTTCTAGCTAGTAGGTGGCAATAAAAACATCACTGCTGCATGAGAATGAGTAGATTGTTAGAAAGAAATGATTGTTTGGATGAGCTTCAATGCAATTCAAAGTGTGCAGAAGGCTAACCCTAAATTAAATTACATTGCAGGAGGCATCAATGTCAAACACAAACCCACAAGAAGCTGAGTGGTTGAAGTACTCTTCTTATTGGGCTGACCCTCAGCCTCAGCCTCAGCCTCATCTTCATGGGTAATAATAACAGACCCCTGGCAAAGTAATCATCCACACTTGCGCTTTCCTCTTCTTTTTTTTTTTCATTAATGCAAATATATATATATAAAGGGCGTGGCTTTTCGAGTCTCAACGGAAATAACTGCTGCATGTGTGCACTGTAACGGCACATACATCAAGTTGGAAACAAACAACAAAGGAGGGAAGAACAAATTGAATCAAATTCAAGAAAATGAAAATAACCGCAAGAAAATTAAAGCTCGCTGCTGTTCTACCTCCTCATCACACAAAGAAACAGTTTGATAATGCATCAAAATCATCACTATTACCGATATAGTATCTTTTTTTCTCTCATTATTCATATTATTTTATTATTTTAATTAAGATGTTTGTATAATTTCCCTTTTACTGACTTGCTTCTTTTACACATTACATGAAATATCAAATATACCGA

>AdNAC48

AAAAAAAAAAAAAATCTTAATTAGATTTCCCTCCATATTCTTCCCTATTACAAGAAAATAAAAATATAATCATGGGTCTTAGAGATATTGGTGCTTCATTGCCACCTGGGTTTCGGTTCTATCCAAGTGATGAAGAATTAGTTCTTCACTATCTTTACAAGAAGATCACAAATGAGGAAGTTCTCAAGGGTACTTTGATGGAAATTGATTTGCACACATGTGAGCCATGGCAGCTTCCTGGTACATACTAATCTAATTATTAATTAATAGTATATATAGTAATATCAATTATAATTACCTTATTATTATTCCTTAATATATCCTTAATTAACCTTTATCCTAATGCATGATGCATGCCTCCTCTTCAATTCCTCCTCCATTTTATACACAACATTAAAGTTATAATCATAAATCATCTTTTTTTTTTTAAATTTCCCATGTTTTATTTGTTGTGGTTTTTTTGTCTTTTGCCTTTGTCTTTTTGATAGAAAATAGACATAGATATTTGGTTCACATTCAGTCACATATATATAGAGCTATAGTCTAATCTAATCTAAACCTTGTCTCCTTATATTAACATGGTGTCCATAGATCTTGAGTTTTGTTTCCTTAAACAAAAAATATGCAAAATAAATATAAGATTTAACATAAAAAGAAAAGCTAGCTAGCATGTTTAGAATAAAGTAGGAGGAAGCAGAAGAGGGAGAGAAGAGAAAGAAAACTCAAAGCAAAGGGAGAAAGAAACAAGAAGGTGAGGTGTTACTATTAAGTGTAATAATTTATTCAGTTCCTCAATGATTAGGGTAATGGCAACTTCTTCAGGAATACACGGCCAAAATAAATTAATTAATTATTATGTTCTTTATACAATTATTTTATATATAATATTCTTCCACTTGATTTATCTACTTGTGTGTATTATTATTATTGCTTTTGTCTTTAATTTGTAAAGTTTGTATGTGATTGAATTCTTTGGCTGTAAAGCGCGTCCAAAACACGTTCCCTTTTTCTTTCTAATAAAATCATTTTAAAGATTGAAATTCGATAAAGGATGTCTTTTGTCTACCTCTTGTAAAGATTCTCTGTTTTTTTTTTTAAATTTTTCCTTGCACCACTTTATTACACCACTTTAATTTTCAAGCATTCTTTTTTTTATTATTATTATTTTATTATCTTCTCTTTTAGTAAAATTCAAATTGCAAACAATAATTACTTTTTTTTGGAATTAATTATTTTTACCTATCCTCTTTCATACTTTATTTTATATACATGAGAATATATATATACACGAAATTATCTGTGTTTAGAATGAAAACAAATCTAATTTACATGCATGGCCTATATATATGTATAACATGAGGTACTTGTCATGTGCAAATATCGTCTCTATTGCATGTTTAGTTTCCACAAATACCAAAACCGTTGACACCAAACCTATTTTGCTGGTCCATATTCATGTCTCTATATATATGAATACTTGTATAGATGCACATGCACTACACATTAGAATTATTTCATTTCTTAATATATATAATTTATTTTTGGATTTTTGCCCATGATCATTATATGGCCTTATTTAAATTAATTTTAGAAGAGTTGTAGTATGTGTTGTTATATGTGTATGTAGTGTTCACTCTTCTCTTCTTATGAAAATAATATTTAGTTTTATTTATTTTACAAAAAATAATATTACTTGTAGTGTTAAATAACCTCCTAATTTTGTGATATTGATGATTATTAAGATTACCTCAACATCGTGTAATTAAGAGAAACCGGACATTTGTAATTAACAACATATAGGGCCTAGCTAGCTAGAAACTACTAACTATATATATCACAATTCTCTTTCTCTCTCTTGGGATTCAAGTCATCATTCTCACTAATGCATGTCATGCTTGTCACTTCAATTACACGCTTGTTATTATTATTATTATTATTATTATTATTATTATTATTGCATATATATATATATACACTATGCACTATTATTATGAAGTTGACAAAAGTTATTAATTCATTCTCATTTCTCATCAAGCAACAAATATTGATTGTTAAAGAGTATGCAATTATTTTACCTATATATAATATATATATTATTTCAGTAACTCTCAATTACTTTTCTCGAACATGGCTTAGTATCTAAGTGATTTTGACTAGTTAACTTTAATATATAATTTTGTTTTTACAATATATAATCAACTATAGATTGTGCCATGAAATTTATTCTTTTTAAAATAAAGAAGCACATAAATTGTTATAATACGCCTCTATCATATAAGAGTTTTTTTTTTTGGATTTGTGTAAAATTTTATGCAAGGTTTATTCTTTTTCTATTAGTTTTATACTAAAATTATTATTTTTTTGTCAAGAAAAATCGAACTCTAAATGTTTAGATTATAGAAATTTGATACCATGTCATAAAATTAATTTTTTTTCAAAAATTTAAACTAATAAAAAAATATACAAGTAATTATTTATTTTTAGTTTTTTTTTCTAATCTAAATTAACTGATTTTCAAATTTTGAAAACGGTAAAAGGGATAAAAGCAGAGAGAGAATTTTTTTTTAAAAGATCATTACAACACAATCACAAATATGTGGCAAACACATTGAAACTTCAAAAGTGCCATTGTTTAGGTCATCTTTATCTGATGGGGATTGGAAACTTGAAAATTGCAAGGTCATTGATGAACAAGATGACATACATGAAAGTGATTTCACACACAAGACCTAATTATTACTGAGATAGTGAGATGATCATAATTATATTCACATGGAAACAATTAATTCTACAATACGATAACTAATGTATAGTATCTATCAAATTATCTATATAAAGTATGTATCTTCTTGTGGAAAATATTTCTTTCTTCATCTAAATTAAATTATCTATGTATGTATGACTTCATTATATATGTGCATATTTATTTTTTATCATCAATATTTAAATTTTCATGTATAATTTACAGTCTTACACAAGAGCATCTTTTATGATTTTATTCTATATATTATACCTATCTGTCTATCTTTTTTGTATGTTAATCTTTATCATTGATTATTAAAACCTTTTCTGAATCCCCTATTTAATTTTACTGTTGAGATATTCAATCATGAAAATCACCTATGCATGACAAAATCGTATGGAATTTGCATTAAAAAAAAAAAACTCTATTAATTACCTTATGAATTGTCTTGTCGAATNNNNNNNNNNNNNNNNNNNNNNNTTCGACAAAAAATTTTCGTATGATCTTATCTGATAATAAAAAATATATATATATATATTAATTTAAAGAATAAATGCCATTTTTATTTTTGATAATTTAACTGAAGAATAAAATAGTCTTTTACAGTGTAAAATTTTTTATTCGATCTATAGACTTAACGATTTTTGACCATTAATTAAAAATTGATTAAAAATTTTATAGTTGTAAAAAAGTTTTTAATCTTTAAAGCAAATGATTAAGAAGTGGAAATAATATTTAGTTTAATTTAAATAAAATATAAGATTTCTCTTTCGTGATACCTACCTTACTTAAGTGATGTTGCTGTCCACATATGCTAGAACCATAAACTGACCCATCATATGTAATGTATTTTCATGCATGCGTACTCTACACACAGAGGTGGCTAAGCTCAATGCAAATGAATGGTACTTCTTCAGCTTCCGTGACCGCAAATACGCCACCGGCTTTCGCACCAATCGCGCCACGACATCTGGCTACTGGAAAGCGACCGGCAAGGATCGTACGGTTCTCGATCCCCTCACCCGCGAGGTCGTAGGGATGCGGAAGACCTTGGTGTTCTACAAGAATAGAGCCCCAAATGGCATCAAAACTGGTTGGATCATGCATGAGTTTCGCTTGGAGACCCCACACATGCCACCTAAGGTTAACTCATTTTTCTCACACTCAAATTTCAATTTTTTCGCTCTCGTTTAATTACATTTATTTTTTTAAATAATTATTTATGTAGTTAAAGTAAAAAATAATTATTTTTTTAATATAACAGTATGTAATTATTAGATTTATATGTAAATTTGATTTATGTTGATTGTACATTAAAAATTAAATTTAAAAATATTTATAAATATTTTCAAAGGTGCATATAGGTATCATGATTAATTGATTATGATATGGTTTACACGCTTACTTAATAATAGTAATGGCCACTACCATATTGGGAGTGTACGGTCTATGATAAATGGGGAATGAGATTAGGGTATAGTGGAGAGAAGGATCTTAATTAGTTTCAATATGTTCCCACGTAAGAGATATATGTTAAAGCTTATGATTGTGATTTCATTTATATCTTAATGAAACCTTTAGATGAAATGACTAATATCCGATGATAAGATTTTTTTTAAAGAAACATATTAGGCACTTAAGTTAGGCATGTATTTAACTAATTTGTTTGCTAATACAAATTTGTTTTTAGGAGTTAAGTGTTTACATACTAATAATATGAAAAAAAAAATTATAAGAAAAGAAATAATTGCATTGTCAATCACTAAAATTATAAAAAGTATGTTAAATTAATATAAATAAATACCTATTATTATCTATAATAACTATACATTCTGATTTCTTATTTTCTTGTAATTTATGGATTCAAAATGTAAAACTCTACTACATTCTCAACGACTTATAAAACATGATCTCTTATTTTTAATAATAAACGGGTTTATATATAACACCATAAAAATATGGAAAGAACTTGACCTGTATTTCATCCTAATGTGACAACCTTGTAGATAATTGAAAACAACTTTGATTTCTGGTTGGAAAGTTGAAAACTTAATTAGCCTATGTCATGTAAAAGATTAGGTAGAATCTAATGTCTTTTCTCCATGTGAACGTTTGAGTTAGGATCATATTTGGCTTCTGCATCGATCATTTAGATTATTGAGATAAAAAGGGAACCAGCATGCGTCTGTGACAACTTATATATATTAATATAAGGATGTTACGGTGACTATAATATTTTAAAAAATACTACTAAAATTAAAATAATATTTTATTATTATTAAATAATAATTTTTAAAAAATATTACTAAAATAAAAAATATCATAATTAATATAGAATATTCATATATATTAGAATATATATATGCGCCAATGAAATTTGTTACAGCTATGCATGCATTGCTATGAGTGTTTGAGTTGGATGGAACAGAATAATTTCATTATGGGAAAGAAAAAGAAATTGGTTTCGTTTGACGCGTATTTTTGGTGTGAGGATAAAGTTCCTTTAAGTCCTAAACTTTGGTTTCACATTTATTTTTGCTAATTAGGTCATCTATTTATTTACTGTGTATTTATTTATTCATTTATGAATTTAATTTGTATATACAACAAATATAAAAAAAAAAAAATTATCCATACCTACCAATTTTAAAAGTCTTGTTATTACAATAACTGGACCACAACTTAAAAACTCATTTAACTTTTAGAAAAAGAATTTTGATTACACATATGAATTAATTAATTAATTAATTAAATTTATTTGGTGATTACACAGGAGGATTGGGTTTTATGTAGAGTGTTTCACAAGGGCAAAACAGACAATAGTGCCAAACTAAGCCCACAATTCATGTATGAGGCCACACCTTCATCCCTAACTTTGGCTTCATCATCATCATCCCCACCAACAAACCAAACAAATTGCAACAATTTGCATGTTATTGGGTATAACCAACTTCCCAATTTCTCATCATCATCATCACCAATGCCAATCCACCATAATCACCATCATCATCATCATCAAAACCAAAACGGTTCTTCCTCTTTGATGAATCTCCTTCAATTTTCCACTAAGGAAAATAGTACCATTACTCAACTAAGTCCCAAAGGTGGCGGTGGCGGTGACGACGGCGGCTATGGGTTCATGTGGGACATGGATCTTGAGGAAAATAGCTTCCATGATGGTGGGGTTATTGCATCAAACTTGAACGACATGAGATTTGAGGTTGATAATAACACTATGGTTATGTTGTAG

>AiNAC41

ATGGGGCTTCGAGACATAGGAGCATCACTTCCACCAGGGTTCAGATTCTACCCAAGTGATGAGGAATTGGTTTGCCATTATCTTTATAAGAAGATCACAAACGAGCAACTTCTTAAAGGCACTCTTGTTGAGATTGATTTACACATATGCGAGCCATGGCAGCTTCCAGGTATGTTGTTTTCTCAATCAATAAACAAACAAAAACAATCACATGGGCTTTTCTTCCATATCATTCCTTGTCTTCATTCACATAAATTAAAACCTTGTATTTGTATTTCTATTGTGTATTGTGTTATTGTCTATTTGTGTCTTTTCTTTGCCTTTCATTAATCCTGCATAATACAAACTTGTCTCATCATCATCAATTAAAAACGGAAGGGCTAGCTAGAAACATGTTCATAATTACTTTCAAAACATGTATTATAATCTACTCATCAATATTAATCAAGGTAATAATGCTAATTTTCTTCATATAATTTCTGCCTTTTCCTCTCTTCAAAAAATATTGAACCACTTATATCTTCTCTCTCACTCAATAATTTCTCTTCTCGTGATCAATATTTTTTTTTACTTTATATTTAAATCAAGACTAGCAAGACTAGNNNNNNNNNNNNNNNNNNNNNNNNNNNNNNNNNNNNNNNNNNNNNNNNNNNNNNNNNNNNNNNNNNNNNNNNNNNNNNNNNNNNNNNNNNNNNNNNNNNNNNNNNNNNNNNNNNNNNNNNNNNNNNNNNNNNNNNNNNNNNNNNNNNNNNNNNNNNNNNNNNNNNNNNNNNNNNNNNNNNNNNNNNNNNNNNNNNNNACTCTTAACATTTTTTATTTACTTACAATTTTATAGCCTTATTCCTTTGAGTTATATATGAACTTGAATGTTGTTGGGTGTAACTACTTCCAGTGTTCTACACATTTATTATTTTACTTTATCTCGTTGGAGAATAGAATGATGCAATTTTAATTTGCTTCTGTATTTCGAGAGTCAAAATATAATAAATCCTTTTTTTTTCTTCCCTAACCTAATTGATGTTAGAAAGTGAATATTGAAGCCATTTGTTTTAAGCTCTATTGTCATCGGCATGAAGCTTTCATCAGTAATCTTTTTTTTTTATTTATTTATTAGATCCATTCTCNNNNNNNNNNNNNNNNNNTCTAGAATTGGTAGAATATATATAAGACCGAAGATTTATTTTCCTTCAATTATATTCTAACATTACTTCGTTTTATGGTCTTTAATGATTTTGTCTTTTGTATTTTAGTTGCTTGGTTTATAGAATAATAATAACAACAATAAAAAATTGTATAGTGGCTAGTGGTTGGTGTATATTATATATAACTATAGATCACACAGCATAAACTTGTTTTCTCCTCTATATTCGGATTATTATTTAATTTCTTTAAAAATAATTGTGTGTGTGATTATATATTATAAGGCGTATATATCCGCCTTATGTATGTATATATGTATGTATACAGTATTTTTAGAATAATACAGGGATCTCTAGCTAGCTGGCAGATATATATTCAAGAGTAGGTTCAAACATAGAATGAATAATGATCATATTTTATTTTTTCTTTAATTTAGGTATAACGTCGTCCCTTTAATTTTTAATTTTACAACATATATAAACATGGTAATTATATTATTATTTAATTATAACAAAGTTGACAAAGCAATACATTCTTATTAAGCAACCAAAATATATATATAGAGATGATTAATTATAAAGATAATTCAGTTAATTATTATATGTATATATATACACACTAACATACATACGTGTATAAATGTCTCATTTACCTTTCTTGTGCATGATTCGCTATCTTCATGATTTTGACTCTTTAGCACTCATAAATATATAATTAGTAATTAAATGTTCCCTCAAGAAATGTTAAGAAAATCTAACTTTACACATGAAAAGACATACTTGATCTTTCTTGCGTAGAATAATTAATCAGCCCTCATCTCTCTGATTATATATTATGATGATTCATGCACAACATTATGAAATATGTGAAAAGAGGGTGAAAACAAGGGAGAGGATAAAAAAGGTCACGTATTTACAGAAATAGCAACCTTGAAACTTCCAAACACTGTGTTGGTGTATGTCATGTACCACTATTTCTCTTGTCACCTTTGTCTGCTGAGATTGGAAACTTGAAAATATCATGTCATTCACGACCTAGTTGACACATGATGCTGATTCCACACAACACCTAATTATTGTTATATTATTTTAGGATTTTTGGATTAGGATTTCTTTAATAAAACTAACCCTTAGTCGTTAAAGATTTGGAAATTTAATCATATAAATTAATTTAATATTTATTAATTAATGATTAAATTAGTTCTTTCANNNNNNNNNNNNNNNNNNNNNNNNNNNNNNNNNNNNNNNNNNNNNNNNNNNNNNNNNNNNNNNNNNNNNNNNNNNNNNNNNNNNNNNNNNNNNNNNNNNNNNNNNNNNNNNNNNNNNNNNNNNNNNNNNNNNNNNNNNNNNNNNNNNNNNNNNNNNNNNNNNNNNNNNNNNNNNNNNNNNNNNNNNNNNNNNNNATAATATCACTTTTTCCAAAATTTTAAATTGATAAAAGATACATATATTCTTCTATTATGGTATCAGAGCTTCTATGATTGAAAAATCTAAAATTCAATAATTGTTGAATAAAAAAAAATTAATCCATTATAAAAGAGGCTTTTTTTTTTGTTTGCGTGAATTTCTTGCATAATTTGTCTTTTTTTTTCCTTATCCTTATACTGAATGTTTTCTTTCAACATTAAGTTATAATAAAAACTCTAAAATTATATCATGATATATTATTTCTCTTAAAAGTTTAAATTGATAGAAGAAGATATATGTATAACAATTTTGAGAAAGCTATTAAACAAGTATCGAAAATGAAGAGATAATTTGGGTATAGAGAAGAATGGAGTAATGAAATATATGGATGGATGTGGTATGCAGAGGTGGCAAAGCTAAACGCAAATGAATGGTACTTCTTCAGCTTCAGAGACCGGAAATATGCGACGGGGTTCAGAACGAACAGAGCGACGACAAGTGGTTATTGGAAAGCAACGGGGAAGGATCGAACGGTGCAGGATCCACTGACACAAGAGGTTGTAGGGATGCGCAAGACTCTGGTGTTCTACCGGAACAGAGCTCCCAATGGCATCAAAACTGGCTGGATCATGCATGAGTTTCGCTTGGAGACCCCACACATGCCCCCTAAGGTACTTTTCTTTTTCTCTCTTTTCAAACTTCCACTTTCATCATCATTATATTTCTTTTTCTTCTTAATTACTTGCATCCCGTGACAAAACTTCATCATCATTATTAACTCTAGTGCAAATTTTTAAGTTCATACACACCATCACAAAACCATTATGTTGTTGACTCATCCATATATATATTCTTAATCAAAATGGTTTTGCTTAGTATTCATCGGTTTGTAACTAACTGTTTAAATGTTAGGCATGAATGGTTTAATTTAGGGTATGTTTGGCAAAATAACAAAATTTTATGAATTTGGATCCTCTAAATTTTGAATTTCACTTAGAAGATACAGTATAAATATAATCTCTCATTTTTGAATAGTTTCTCTCTCATATTTTTTCTTAGTCCCACCTATAAAATAAATGATGAGAAATCACACTTTATATATCCTCTAAAGTGAAAATTTAAAATTTAGAGGATCCAAATTCGAATTTCATTGTTTCATTTTTTTTTCACAAAATATTAAAAATAAAACAAAAACAAAACAAAACCAAATACGCCTTTAAGTTTTTTTTTTCTTTTTAAATGAAAAGACTAGTTTAATTTTATTAGGAAAAGTTGTGAAGGGTAAATAAAAAAGTGAGTAGAGCAAGGGAAAAGGTTTCATATATTATATATAGGGTTTGTTTATTTGGCGTGTATGTGTATGTGTGTATTATATTTTTGGTGTGAGGTTAAAGGGTCTTTAAGCAAGCGTGTGTTTGGGTTTCACATTTTACAATATATATTATTTTGTTCCCCTAAAATTCTATTTGTTTGATTACACAGGAAGATTGGGTGTTGTGTAGAGTGTTCCACAAAAGCAAAGAAGAAAACAGTGCCAAACTTATCATGTATGATTCCATTTCCACACATCATCAATCATCAAACCCTATGGCATTGGTATCAACCCATCATTTGAACCCCATCAACAACCATAATAACACTTATCATGCCATGAATAATTTCCTTCATCACTTCTCATCATCAAGGGATGATAGTCAAACAAATAACGCCAATAATAATAATAGCAGTGTTACTCAAATTAGTCCCAAGGGTTATGATGGATATGGCTTCATATGGGATCACATGGATCTTGAAGATGGTGGTGTGCCCTCATCAGACTTCCAG

>AdNAC27

GTGGTACTTAATGATGAAAATAATGTGAATTAATTGGGTTTGATTTCTAATTAAGTTGGTAGTTGTTCTCTTTTTAAGAGCTTCATAGCAACGCGATCCTCGCAAAACACACGCTCCTTTAAGAGTCACCACAAAAGACGGCTGAACCTTCGGGTGCAAGATCCCTTCTCAGGAAGCATATAATCACTTCTTCACTACTTCAACTGTGTTCTCTCTCTATCTATCTCTTCATTATTAGGGTTTTCATTTCTATATATCCACCTCTTTCAACTCTCTACTCTTTCTTGAGGAGATGGAAGGTGAGAAGCTTGACGAGATCATGTTACCAGGTTTCAGGTTCCACCCAACTGATGAGGAGCTTGTCGGGTTCTACCTTAAGAGAAAGATTCAGCAAATGCCTCTGTCCATTGAGCTCATCAAGCAACTTGATATCTATAAATATGATCCTTGGGATCTTCCAAGTAAGTATATAATGGTTATGATCTGAATCAACTTAATGATAATAAGGCAATTAATTAAAAAAGCACTGTATGTGTAGAAGTGGCAGGTACAGGAGAGAAAGAGTGGTATTTCTACTGTCCAAGAGACAGAAAATACAGGAACAGTGCAAGGCCAAATAGGGTAACTGGAGCTGGGTTCTGGAAAGCCACAGGGACTGACAGGCCTATATACTCCTCAGAGGGTTCAAAGTGCATTGGACTCAAGAAATCTTTGGTCTTCTACAAAGGCAGAGCTGCCAAAGGTGTTAAAACTGATTGGATGATGCATGAGTTTAGGCTTCCTTCTCTTGTTGACTCTTCTTCATCCGACAAGACCACTATTCCTGCTAATGTCGGTATTTATCACTCTCTCAAACCTTTCTTAACAGGATCCATCTCACACTTTCTAGCTGACTTCAATTTCAACATGCAGGACTCTTGGGCAATCTGCAGAATATTCAAGAAAACAAATGCTACAGCTCAAAGAGCACTCTCTCACTCTTGGGTTTCTACCTTACCTGAAACAACACCAACCACCACTACCAATGATACAGATCACATATTCAACATGCCAACGATGATGGCAAAGAAAACTAGCTTCATGACCCAGTTTTGCACTAACTACACTAGTGACACACAAATCCAAGATGTTGCATCATCTTATAAACCACCCTTTATTAATATTAATCCATTGCTTTACAAGCAGTTTGATCATCATCATCATCAGTTACCACCTATTATTTCAAATGGAGATCTTATAAGCAACGACTGCTTAATACCCTCTTCTACTACTCCACTTGAAACATCCTCTAATAGTGCAAAACCTACTATGGATTTTTCTTCATTGTTGCTGAACATGTCATCTTCTGTTCTTGGAGATTTTGCTGGAAAGACATCGTCGTCGTCCTCATCCCAAGAGGGTACAGCAGCAGCAGCAACAGCAACAACAATCACGAGTAGCTTCGGTGGTGGAATGCAGGAGCACTACCCAACAATACCATTACTGCGTCAGATGCATCAAGGGAACAACAACAACAACAACAACATTGGCATCAACAACAACAACGTGTCTGCTGGCGGTGAAGAACAAGAGTTGGAGAAAGTTGGATCCATTGTTGGGTTCCCATTCATGAACATTGGGGATGCATGGAAGTCAAATATGCTTTGGGATACTTCTTGTCCCTTGTGA

>AdNAC7

CCCCTCTTTCCTGTAAGCTTCTTTGTCTTCTTTCCTTTGTCCTCAAACGCTCATTTCCCTTGTTGCTTCTCACTAAAGGTTTGTATCTATCTATCTCTGTGTTTTTGCTATCACTACTTAATTTCTTCTTCTTCTTCTTTAATTTCTTTTTTGACATGCATGAGTCTTGGTTTTCTTATTTTTCACTTTATTAATAAAATCTATTTTTGTTTCTTATTCTTCCTTTTTGTTAGCTTCTATATGTATTGCCTTTTGATCTATCATATGCTTCATTTCAGAAAATGAACTAACCCTTTTCTTTAATTTTTTTCTTGGTGTTTTAATTCTGTAGTATTAGTTACACTTAAACTCTAGCATACATAAATCATAAATATTGCGCTTTCTCCTAGCTAGCTCTTGTATTAATTAGGTTTGGGACAAAAAGGATTATATATATTCATATTATTTGGTTGAAGCTAAAAGCTTTAGAGAGAAGAAACAACAAGCTTAGGGTTTTAGCTAATGGGTTCTATATCTTAAATTATACAAAACTTTTCAAGATATGAGATGAAGATACTAGTTACTTTTCTTTAATTTTATTAGTTTACTTCCTTTCTCTCACGTGATTTCTTTCAACTTTTGGTTGTAGTATGTGCTTAATTATCCCTCCAATAATACACGTATAGTAGTCCAAAGTTCAAATTGATATGCATGAACGTTGGTGTTTGTTTCTAAGGCAATCAGCATGGACATAACTTATCATTTTTTTTTCCTTATAGTTTCTGATTGGTTCTGGGTCAAATATCAGTCCAAGTTTAATATAGTCATCATTGGAAAAAAGATCTCTAGCTTTTTTCTGAGGATCTTCCCTAGGGTTATATTATAGATAGATCTCATAGATTGAAAATTCCTTGTGTCAGAAGAAAATGTCTCTTATATTGTGTATGGACACAACTTTAAATGAAAAACTGATATTTGTATTATTTTAAATAATTTTTTGTACTCCTTTATTTATTTATTTTTATGGGTGTACTCCTTTATTTATAGTATAAAAGACAAATAGTTTATCTTTTTTCAATTTTTTTTTATCAATCATCTTTAATTTTAAAAGTAGATGTACAAAAATATGTACACAGAGTGATGTATATAACATTCCTCGTTTTAAATCATATATACTTAGTATTTTTCGATTTGATATATATATATCTACTTTTGTATATTTCTTATATACTTAATCATTTTCTATTTGAAGTATATATATAATATATATATTCTTAAACTTAATTAGTTACAATAATAATTAAAGTCGTATCTCTCTAAATAAATTGGATCACATAAATCACACTACACTTTCCAATATTACATTTGTATTTTATGTTATTTTTTAATAAATTATGGAAGGTGTGATTTTATTTAGACTAATTTTCTATATAATTGTTATTTCCTGCAAGTTCTCCTTATTTTTTAGTTATATAAAGTAGCATTTTCACACCATGACGGTCAGGAAGAACACACATACACATATGTTCTCGAGATTATCAATTTTTGGGCCACTAATTTTGAATTTCTACAACATAAACTACCGCTAGCTAGTTCCATTCATTCATACTTGCTATGTTCTTAATTAATTAACTATAGAAAAACGTTTCACATCTACCTTTTAATTATTTATTCAACAATGTTAAATTACTAGCTAATAAATATTATTATTTTAAATCATCACATAATTAACAACAATTTATACTAATATTTACAAAATTATATAATTTAAATTTATATTTACTAAAATTTACACACATAAATAAATATATTTTATATTCATATTTATCATAATTTATACTAATAAAATAATAAAATTTATTTATTAAAAATAATTTAATATTTATATAGTCAAAATTATTAAAAAGTACTCACTCCCAAAATTTTTTAATATTTATTTATTTATTTTGGTGTAGATATCCACCATCTATTGCTATCTATGAAATAAACATACAATCAATAATAATCCAAGGATTAAGTAAAGCTTAATGATTATTATTATTCTTGATTTTATTTATCAGGTAATTAATTAAAAGCAGAAGAATAATCAGAATGATGGCAGGTAGTGGACAACTAACAGTTCCACCAGGGTTCCGGTTCCATCCAACTGATGAGGAGCTTCTCTACTATTACCTAAGGAAGAAAGTTTCTTATGAAGCCATTGACCTTGATGTCATTAGAGAGGTTGATCTCAACAAACTTGAACCTTGGGACCTCAAAGGTATCTTTCCATCTAACTACATTCATTACATATATCAATATAAATTCTTAGCTTCAGTATGATAAAACTAATTATAGTAGTTGGAAAACCTTAACTTTTTTGTTCACTCCTCTATCTAATTAGGGCTTAACGTAGCATGGGTCAATTATATTAATCGTGATGGTTAAAAATGGAGGACTACAATAATTGTTGCAAGCCGCAAGCCCCTGCAACTGTGGTGTGTTCTCGATTTACATTAAATTTGTAATGAATAAAAAAAATTTAAAGAAACCATTAATTTGACATTAAACGAAAGGAATATTTAGATGTTAATTATACATGTACGATCATACTAGTATTAATAGAATAAATTCCATCAAAACTTCATAGTTAAGTGTACTAAGACAAAAGTAGTACTAGGATGAATGAATTTTCAGTAAATCTCTGTGATAATTGAAAAATAAACAGTGCTTCATCTTTGTTTGAAATAGTTAAGTACCGTACTAAGATGTTGGCATTGAATGATGATTGAAATATACGTGTGGAGTTGTGCAGATAAATGCAGAATAGGATCAGGGCCTCAGAACGAGTGGTATTTCTTCAGTCACAAAGACAAGAAGTACCCAACAGGAACAAGGACCAATAGGGCAACCACTGCTGGTTTCTGGAAAGCCACTGGGAGGGACAAGGCCATATACCATACTAGCAATTCCAAGAGGATCGGGATGAGAAAAACCCTAGTTTTCTACACCGGCCGTGCGCCCCACGGCCAGAAGACTGACTGGATCATGCATGAGTACCGCCTCGACGAAGACGAGGCCGAGGTTCAGGTGACAAAGAAAAATAATTCCTTACATGCATGCAATGCCATTCAATAATAACAATGTTTGGAGAAAAATTCAAGATTGGAAAAATTTATTATTTTTTATTAGCATTTTTACTCATTAATTTATTTTATTTAGTCTAATAATTTAATAATGTATTTTTAATTCATATATATTTTTATAATTATGTTATACAGACCCTAAAATTAGTCATTAAAATTAATTATTAGTATAAAATATATATTAAAATATAAATATACATTTGAAAAAAATTAAACCTATATACAAATATATTAGTGATTGATTTTAGTAATTAATTTTAGTGTACAGATAATATTTTTAATATTTTTAAAAATTAATGACTAGCTACTAGTAAAAAATAATAATTTATTAATTTTCTAGTATTTCTCTAATGTTGAAGATGAAATATTATCTTCTGTAAAAAAATATGTATTATACATAAATATACTATGACTGATTTTGATAATTTAGTATAGGTAGGAAATTTTGCCGTACAAATATGGTTTATGTTCATCTTATAACAACAAAATGAGTCATTTTATTTGTGTACACAAAAGATAAGTTATATAAATAACTTTTTGTTTTTGAAGATATTGATATAGCCAATAAGTCATTCTATATTCCTAGTGAAATTTGACATGCTTCAAACAATTAGTGAGTGCTATATACATATAACGAGAAAATTGAAGTTGCAATAAAAAATGATTTTGTATGTATTGCAGGAGGATGGGTGGGTCGTGTGCAGGGTTTTCAAGAAGAAAAACCAAAGCAGAGGGTTTCAACAAGAAATTGAAGAAGAGGAACATCATCACTTAGCAGCAGCACATCAACACATGAGAGGAGTAGCAAGCCAACAAGTTCTGGACCCAAAACACCACCACCACTTGCAACATCATCAAGGACTCTATGATAATGAAAATAATAATAATTACACCAATAATTTTGATGGATCCATGCATCTTCCACAGTTGTTCAGTCCAGAATCTTCCGTGGCTACCGCGGCGGCGCACACTTCCATGAATGCCATGGACATTCTTGAATGCTCCCAGAACCTTCTAAGGCTCACAACAACAAGTGGATGTGGACTCAATCTCATGCAACAACAACATGGAGAGAGGTTCAATGGTGATTGGTCTTTCTTGGATAAGCTTCTTGCTTCACACCATGGCAGCACCATGGATCATCATCAGCATCATCATCATCATAGCAAATGTAACAATAATCTTCATCATCAGCATTCTGCAATTGCTATTGGAACTACTTCATCTCAGAAATTCCCATTTCACCACCTTGGTTGTGACAACCATGATATCATGAAGTTTTCCAAGTAG

>AiNAC53

CCCCTCTTTCCTGCGAGCTTCTTTGTCTTCTTTCCTTTGTCCTCAAACGCTCCTTTCCCTTATTGCTTCTCACTAAAGGTTTGTATCTATCTATCTCTATGTTTTTGCTATCACTACTTAGTTTCTTCTTCTTCTTCTTCTTTAGTTTCTTTTTTTGACATGCATGAGTCTTGGTTTTCTTATTTTTCACTTTATTAATAGAATCTATTATTGTTTCTTATTCTTCCTTTTCATTAGTTTCTGTATGTATTGTCTGTTGATCTATCATATGCTTCATTTCAGAAAATGAACTAACACTTTTCTTTAATTTTTTTTTAGGTGTTTTAATTCTGTAGTATTAGTTACAGTTAAACTCTAACATACATAAATCGTAAATATTGTGCTTTCTCCTAGCTAGCTCTTATATTAATTAGGTTTGGGAAAAAAAGATTATATATATTCATATTATTTGGTTGTAGCTAAAAGCGTTAGAGAAAAGGAACAACAAGCTTAGGGTTTTAGCTAATGGATTCTATATCTTAATTATACAAAACTTTTTTAAGATATAAGATGTACTTTTCTTTAATTTTATTAGTTTACTTCCTTTCTCTTATGTGATTTCTTTCAACATTTGGTTGTGGTATGTGCTTAATTATCCCTCCAATAATACACGTACAGTAGTCCAAAGTTCAAACTGATATGTATGAACCTTGGTGTTTGTTTCTAAGGCAATCAGCATGCACATAACTTATCATTTTTTTCTTATAGTTTCTGATTGGTTCTCGGTCAAATATCAGTCCAAGTTTAACATAGTCATCATTAGAAAAAAAGATCTCTAGCTTTTCTCTAAGAATCTTCCCTAGGGTTATATTATAGATAGATCTCATAAATTCAAAATTCTGTGTCAGAAAAAAATGCCTCTTATATTTTATTGCATGGACACAATGTTAAATGAAAAATAATATTTGTATTATTTTTAAATTAATTTTTGTACTCCTTTATTTACAGTATAAAAGACAAATATTTTATCTTCTTTCAAATTTTCTTTATCAATCATCTTTAATTTTAAAAGTAGAAAATGTACAAAGAAAATGTACACAGAGTGATGTATATAACATTCCTCATCTTAAACCATACATACTTAATATTTTTCGATTTGATATATATATATACTTTTATATATTTCTCATATACTTATTAATTTTTTATTTGATATATATATATATATAAACTTAATTAGTTACAATAATAATTAAAGTCTTATCCCTCTAAATAAATTGGATGACATAAATCACACTACACTTTTCAATATTACATTTGTATTTATGTTATTTTTCAATAAATTATGGAAAGTGTGATTTTATTTGGACCAATTTTCTATATAATTGTTATTTCCTCCAAGTTCTCCTTATTTTTTAGTTATATAAAGTAGCATTTTCACACAATGACTGTCAGGAAGAACACACATACATATATGTTCCCGAGATTATCAAGTTTTGGGCCACTAACTTTGAATTTCTACAACATAGACTATACCGCTAGCTAGTTCCATTCATTCATACTTGCTATGTCCTTAATTAATTAACTATAACAAAAAACCGTTTCACATCTACCTTTTAATTATTTATTCAACAATGTTAAATTATTAGCTAATAAATATTATTATNNNNNNNNNNNNNNNNNNNNNNNNNNNNNNNNNNNNNNNNNNNNNNNNNNNNNNNNNNNNNNNNNNNNNNNNNNNNNNNNNNNNNNNNNNNNNNNNNNNNNNNNNNNNNNNNNNNNNNNNNNNNNNNNNNNNNNNNNNNNNNNNNNNNNNNTCCTAATCCTAAAATCTTTTAATATTTATTTATTTATTTTGGTGTAAATATCTGCCTTCTATTGTATCTATAAAATAAACATATAATCAATAATAATCCAAGGATTAAGCAAAGCTTAATGATTATTATTATCCTTGATTTTATTTGTCAGGTAATTAATTAGAAGCAAAAGAATAATCAGAATGATGGCAGGTAGTGGACAACTAACAGTTCCACCAGGGTTTCGGTTCCATCCAACTGATGAGGAGCTTCTCTACTATTACCTAAAGAAGAAAGTTTCTTATGAAGCCATTGACCTTGATGTCATTAGAGAAGTTGATCTCAACAAACTTGAACCTTGGGACCTCAAAGGTATCTTTCTATCTAGTTACATTCATTACATCTATCAATATAAATTCTTAGCTTTAGTATGGTGGTTAAGAATGGAGGACTACAATCATTGTTGCAAGTGCAAGCCCCGGTGGTGTGTTCTCGATTTACATTAAATTTGTAATGAATAAAAAAATTGAAAGAAACCATTAATTTGACATTAAACGAGAGGATTACTTAGATGTTATACATGTACGATTATACTAGTACTAATAGAATGAATTCCATCAAAACTTCATAGTTAAGTGTACTAAGACAAAAGTAGTACTAGGATTAATGACTTTTCAATAAATTTGTGTTACACATCTTTTTTAGTATTTAATCAATGTGGTTGGTCGAATGGTTAGTTCTTTTGTTCTTTGAAGCAAGTTTGTCAGAGTTCGAATCTTACTTTATATATGCAGTAATAATTTATTGATTAATAACAAATTTTTAAATAAATACCAGGTCCGCTTTGAATTAATTTTTAATTTTTTGGATTAAAAAATACTGTGAGAATTGAAAAAAAATAGTATTTCATTCTTGTTTGAAATGGTTAAGTACCATACTAAGATGTTGGCATTGAATGATGATTGAAATATATGTGTGGAGTTGTGCAGATAAATGCAGAATAGGATCAGGGCCTCAAAACGAGTGGTATTTCTTCAGTCACAAAGACAAGAAGTACCCAACAGGAACAAGGACCAATAGGGCAACCACTGCTGGTTTTTGGAAAGCCACTGGGAGGGACAAGGCCATATACCATACTAACAATTCCAAGAGGATTGGAATGAGGAAAACCCTAGTTTTCTACATCGGCCGTGCGCCCCACGGCCAGAAGACTGACTGGATCATGCATGAGTACCGCCTCGATGAAGACGACGCCGAAGTTCAGGTGACAGAGAAAAATAATTCCTTACATGCATGCAATGCCATTCAATAATAACAATAATGTTTGGAGAAAAATTCAAGACGGCANNNNNNNNNNNNNNNNNNNNNNNNNNNNNNNNNNNNNNNNNNNNNNNNNNNNNNNNNNNNNNNNNNNNNNNNNNNNNNNNNNNNNNNNNNNNNNNNNNNNNNNNNNNNNNNNNNNNNNNNNNNNNNNNNNNNNNNNNNNNNNNNNNNNNNNNNNNNNNNNNNNNNNNNNNNNNNNNNNNNNNNNNNNNNNNNNNNNNNNNNNNNNNNNNGTATTTCTCTAATGTTTAAGATGAAATATAATCTTCTGTAAAAAAAATATGTATTATACATAAATATATGATGACTGATTTTGATAATTAATTTAATGTAGATATGACATTTTACCGTACAAATATGGTTTATGCTCATCTTATAACAACAAAATGATTCATTTTATTTGTGTACACAAAAGATAAGTTATATAAATAACTCTTTGTTTTTGAAGTTATTGATATAGCCAATAAGTCATTCTATATTCCTAGTGAAATTTGACATGCTTCAAACAATTAGTGAGTGCTATATACATAAAATGAGAAAATTGAAGTTGCAATAAAAAAATGATTTTGTATGTATTGCAGGAGGATGGGTGGGTGGTGTGCAGGGTTTTCAAGAAGAAAAACCAAAGCAGAGGGTTTCAACAAGAAATTGAAGAAGAGGAACATCATCACTTAGCAGCAGAACATCAACACATGAGAGGAGTAGCAAGCCAACAAGTTCTGGACCCAAAACACCACCACCACTTGCAACATCATCAAGGACTCTATGATAATGATAATGATAATAATTACACCAATAATTTTGATGGATCCATGCACCTTCCACAGTTGTTCAGTCCAGAATCTTCCGTGGCTACCGCGGCGGCGCACACTTCCATGAATGCCATGGACATTCTTGAATGCTCCCAGAACCTTCTAAGGCTCACAACAACAAGTGGATGTGGACTCAATCTCATGCAACAACAACATGGAGAGAGGTTCAATGGTGATTGGTCTTTCTTGGATAAGCTTCTTGCTTCACACCATGGCAGCACCATGGATCATCATCACCATCATCATCATCATAGCAAATGCAACAATAATCTTCATCATCAGCATCCTGCAATTGCTATTGGAACTACTTCATCTCAGAAATTCCCATTTCACCACCTTGGTTGTGACAACCATGATATCATGAAGTTTTCCAAGTAG

>-AdNAC28

CGGGATCTGAAACGCTTTCCATTCCCGACGGACTTATTGTCTTTGCTACTATCTTTTCATCTCATTCTCAAACTCAAACTCAAGCACTACTCACCATTTCCGGAGCAAGATTATCGATTTCTGTGAGCTCTCATACATACACTTTCTCTCATTTCATTACTTTTTTTAGTTTTTCTTTTTTACACATTCATGATATCTTCAGTTATAAATTAAATATACAGAGTCTCTAATATATGTATGACATGTATTGTGGCACTAATTAAATAGAGCATGCTTACTTATGAATTTTGTTAATAACCGATGTTCTTAAAATAATGACTAACATTTTCAATGAGAGAAAATAGCATATCTTTTGATATTTTGATTCTTATTTTTATTTCAAATATTTAATTTTAGTAACCTTGGCTTCGTAATTAGTACTCTCTTAAAATACTGACTAATTAATAATACCTTAAAGTCATTATTATGTGCAGTTTGTTCTATAAGATGGAGGTATTGAAACTTTTCTTTAGATTAATTTGGAGATGTTAAAAACAAGAAATACTTAAAAGTGATCAAAATTAATTTAAAAAGCTAAAGGGCAGATTTGATTTCCATATTCATATTTTGAATGGTTGATGATCTCGGATGTTGTGTCTTATTTGTCTAAACATACTGTGACACATAATAAGAAGGTTCATGAACTTTATTCTTTTGAAGCATGGGTCACATAAATCAGTGGGAATCACAATGAGTGACACGCACTATATTGTGTGTATATATATGTTGATCTAGTAACATACTTAGTTAGTATTGTGTGTGTGCATGTCCATTATCACATGTGTGTTGACTATGTGAACATTTTCACACGCATAATCATATCATCATGGTCTTGCTTTTTTCTCCATCTACACTTTATAACGACAAACCACATTGGATATCAATTCTTATTTGTTATATTTCCGTCTTCCAATATCAGAAATTAGGTTAATTAAGCATTGTTGTTTATGCTTATAATTGGATTAATTATGTCGTCATTATACTACTACATACTGTAGGAGGTGTAATTAATTTGCTATTGTTTTTCATTATTGTTGGGCACTTGTTGATATAATCAAAAGTAAATATTAGATGGTGTAAGTTTATCGTTTAAAAGACGCTACTCTGTAAATATTTGGAAAATGTCCAAATTCGCCTTATTATGAAATAGTTAGGGATTGAAAAAGCAAGGAGAATAAAAGTAACTTGTAAAGAAATTATTATAAAGTGGAAACTCAGTCGACTTCACGTGAAGTTAATACCTGAGAGCCATTAGATGATTTGACTAATTTGACTAAATTTTCATCTAACAGCTCTTATGTATCAACTTCACGTGAAGTTGACTGCACCTGAGTTTTCACCTTATTATTATAAAGGTGTTCTTGCATTGTCACTAATAAATTGGGCAAGCCACAATGCATTAAGGGATGGAAGTATCTTACTATAAATTTTGTGCAGGAGTGAAAAATTGCATGGAGAATATGAATAGTTTTTGTCATGTTCCCCCGGGTTTTAGATTCCACCCCACGGATGAAGAACTTGTTGATTACTACCTTAGGAAGAAGGTTAGTTCAAGGAAGATTGAGCTTGATGTAATCAAAGATGTTGACCTCTACAAAATTGAGCCATGGGACCTTCAAGGTACATACACATGCATGCATATACTATATGTATTGTGAGGATATATATTGTTAATTAATGAATAAGTTGTTCAAATTTTGAAGAGATATGCAGGATAGGAAGAGAAGAGGAGAATGAATGGTACTTCTTTAGCCACAAGGATAAGAAGTATCCAACAGGAACAAGAACAAATAGGGCAACAGCAGCTGGGTTTTGGAAAGCAACGGGAAGAGACAAAGCTATATATTCAAAGCATGATCTCATAGGGATGAGGAAGACATTAGTCTTTTATAAAGGTAGAGCTCCTAATGGACAAAAATCCGATTGGATCATGCACGAATATCGCCTTGAAACTGATGAAAATGCCGCACCACAGGCAAGTTTAATTCTATATTTTTCGGTCATAGAAATATTTGCATACATACATAATTAAAGACATGAATGCATGCATGCATGGATGCAGGAAGAAGGATGGGTGGTGTGTAGAGTATTCAAGAAGAGAGTAACAACGATGCGTAAAGTGATGATGAGAGAGCATGATGAGTCTCCTAATTCTTCTTGTTGGTACGATGAGCAAGAATTGATGATGATGGAATCGCCAACAAAGCAACAATCCTCTATTCTTCTTCATCAATCCACCAATAATAACCATTCCAATTTGATGCAGCTACCACCATATCCTCTCATCAAGAAAGAGCTTCATCACCCATCATCATCATACCCCTTCCTTCAGCTTCCACTCTTAGAGTCTCATCAACAATCTGCTGCTGCACCTTCCTCCATTTCTGAACAACTCATCATGCCACCACCAATTGGAGGAGGAGGAGGAGAACAAGTCCCTAGTTTTCAGTCATTCTTCAATAATGAACAACAAGAAGTAGGAGTTCTTGATTGGAGAGTTCTTGACAAGTTTGTTGCTTCACAACTTAGTCAAGATGATAATCATGCATCCTCTAATAGTATTGTACAAGATCTCACACAGGAAATTGTTATGGTGCCTCACAATGATGCTGCATCAACATCAAACTCCCTCACCTGCCCAATTGATTTGTGGAAATAG

>AiNAC26

ATGGAGAATATGAATAGTTTTTGTCATGTTCCCCCGGGTTTTAGATTCCACCCGACGGATGAAGAACTTGTTGATTACTACCTTAGGAAGAAGGTTAGTTCAAGGAAGATTGAGCTTGATGTAATCAAAGATGTTGACCTCTACAAAATTGAGCCATGGGACCTTCAAGGTATAATACACATGCATGCATAGACTATATGTATTGTGAGGCATATATATTGTTAATTAATGAATAAGTTGTTCAAATTTTGAAGAGATATGCAGGATAGGAAGAGAAGAGGAGAATGAATGGTATTTCTTTAGCCACAAGGATAAGAAGTATCCAACAGGAACAAGAACAAATAGGGCAACAGCAGCTGGGTTTTGGAAAGCAACGGGAAGAGACAAAGCTATATATTCAAAGCATGATCTCATAGGGATGAGGAAGACATTAGTCTTTTATAAAGGGAGAGCTCCTAATGGACAAAAATCGGATTGGATCATGCACGAATATCGCCTTGAAACCGATGAAAATGCCGCACCACAGGCAAGTTTTAACTCTATATTTTTCGCTCATACAAATATTTGCATACATATATACATATACACATGAATGCATGCATGCATGGATGCAGGAAGAAGGATGGGTGGTGTGTAGAGTATTCAAGAAGAGAGTAACAACGATGCGTAAAATGATGATGAGAGAGCATGATGAGTCTCCTAATTCTTCTTGTTGGTACGATGACCAAGAATTCATGATGATGGAATCGCCAACAAAGCAACAATCCTCTATTCTTCTTCATCAATCCACCAATAACCATTCCAATTTGATGCAGCTACCACCGTATCCTCTCATCAAGAAAGAGCTTCATCACCCATCATCATCATCATCGTCATACCCCTTCCTTCAGCTTCCACTCTTAGAGTCTCATCAACAATCTGCTGCTGCACCTTCCTCCATTTCTGAACAACTCATCATGCCACCACCAATTGGAGGAGGAGAACAAGTCCCGAGTTTTCAGTCATTCTTCAATAATGAACAACAAGAAGTAGGAGTTCTTGATTGGAGAGTTCTTGACAAGTTTGTTGCTTCACAACTTAGTCAAGATGATAATCATGCATCCTCTAATAGTATTGTACAAGATCTCACACAGGAAATTGTTATGGTGCCTCACAATGATGCTGCATCAACATCAAACTCCCTCACCTCCCCAATTGATTTGTGGAAATAG

>AdNAC5

CAAGTGATCGGAGTGAAGAAAACCATGGTTTTCTACAAAGGAAAAGCTCCCACCGGTCGCAAAACTAAATGGAAGATGCATGAATACCGCGCCATCGTTCAAGCCCCTAACCAATCTCCCACGGCTATTCCTCAGGTATGCATACATAAGGTCAATGTGAAATTAGTTAAGATGGTAATTGGTAAATATTTTATAACGAGAAATTTCAAGTGACTATTAGAATTTACTATTTTTAATCATCAAATTGATGATTAAAAATAGTAAATTCTAATAGTCTATAGAATTTCTCTTTATAATTTAACTAATATATTTTTTTAAGTTTTAAATTTAATTTTATACGTGTAGTTGAGGCACGAATTCAGCTTGTGTCGCGTGTACGTGATATCCGGAAGCTTCAGAGCATTTGATCGACGGCCACGGGAGGTGGTAGTGCCAAGAGTTCTTCATCATGGTTCTTCTACAACAAGTGCTCAGCAGCATCAAGGAGAATCATCAGCAAGGGTGCAGGCCAATAATAATAATAATGGGTCGAGCTCGTCGGAAACTTCCCTTTCATCAGGTGGTCCTGATTTGCCACCAGATACTGGAGGAGGAGGGTCATGTAGCAATTGGAATAGTAGTGAGGTTCAAGTTCAAGCTCAAGTTCAAGAACCACTATGGGAATGGGAACAACTCGATTGGCTATAAGCATGACTAATAAGAATATCAATTCGTCAAGCCATGCATGCATTTCATCTCTCATCAAGACCCCTTCTCCACCACTTCAATAATTCCTCTGTTTTAAATTAATGGTCGCTTTCACTTTTTCGTTTTAACTTCATTTATTTAGAAATAGTAAAATAGGGTATTTGTATAATTAACATGTGTAACACTATCAGGATTTGATTTTCACCGAGAAAATAAAGTAGGTGTAACTCTAATTAAGGAGAGATCATGTAATATTAGAACTTAATTAGAAGCTAAAGTATAGTTTTTTACTTTTTTTAAGCATATTGTATCATTTTATTTAGTTTGCATAAAGGTTAATTTGTAACCAAATTCTTCTCAT

>AdNAC60

CTAAGGATCCTCGGGATCTATCTCATCACCTCCATCATTCTTGAGTGCACAAGGCCGTGATCCACTTCAATTCCCGAGGATCACTTTTAGTTGCTCTCATTAATTACCACTTTTTCTGTTCTCTTGTCCCCATCCTGTTCATTCCATTGCTGTTTTTTGGTGCAATTTCTGGAAGTAGCTCTTACAAAGACTTGGGTTATATATATTGATTTAAAAGGATTCTTATCGATAGAGAAATATCCCATCAAGTTATACCACGTACAAGAAAAGTTTGGTTAATTTCATATACAACAGATCTACGTACAGCGATTCTACTTGGAGTTCTTAGGTGCAAGATATATCAAGCTAAGGTTGTTCTTTAAACTTGTTCTCATTATTATATATAATGCATATCTACTTTAGCATATTATTATGTGAGTATCTCTATCTATATTCTTAAGGCATGACGAGAGAAAATATTACTGGTTTGTTTCTTTTAATAATTCAAGTCAACCAGCATAAGTCCAAAAAACAAAAAGAGACAACAATAAAAGACTGATGAAAACATGACATAATTAAAATCTTTGACATGACTTCGATCTGTGTGTTTCGGTTGGAATTTGTGTGTTCTACACCTACCGTGATTCATGTGCTTTACTTCAGCTTTTTATTTATTTATTTATTATTCCCTTTGTTCTGGCTTTAATTTGCACCCGACAGATATTGATCTCTTTGCTTATTCATTCGACCGCAAAACAACCATGCTAGCTTTAGTTTATCTGCCTGTGATAGATGCCATGATCATTAGATTTTCCCAAACCGTTTTTATATATGTTTGAATGATCAGTCATCAGTTCTTAAGTCAGTAACATCGCCTCTTCTCTTAGGGATTTTAGCAAACTATGCTGTAAGCCATTACGTACGTGCGTGAAATGCTTGAGTGCTCTACCAATTTTGCTGTAGGGTTGGCTTCAAATAGGGTAAATATTTGATGCTATATAATCAAACCCTAAATCTAATGGAGTTGTATGTTTTGGAGAAATTTTATTAGCTGACGAGGCCGTAATTGTTTACGGTTGATTTTTCCATCCTTCTATATTAATTCTATAGTGTTTGTATATAGCAACAGTATCTAAAGGGGAACATCAATTATCAACAATTATATATGTTATTATCTTCTATTCTTCATTTTCTTGTCCTTTGCTGTTGTTGAACTTTTTTTTTTTATTTCAATTTTTAACCGTGAACTTCCATTCAACACTACTGTCATATCTTTTTTCCTCTTTGTTAAACTTCTAACTTGTAAGCACAAATCGAATTCAGCCATCAGAAGAGTCCTTCGAATTAAACCCTATCAAATACAGTCAACACTACTAGCTGTTGCTGTTCTTCATCTTTATTTTAAAACTGTTATTACAGAATTTAACATATTCCTGTGAAATCTACCACACAATACGTATATACATATGTACATCATGTTATTATTTGCAAAAAAAAAAAAATTATCAATAATCAATCACTTATATAAAATACATGTTAAAATATATAATACAATTTAAAAATATGTAAAATAACACATTAATAAACACAAATTAAATTACGTGGCTAATAACACATTAATACACAGAAAATTTGCTGAAATTTTACAACTAACGGAAGTGATATGATTTATTGAAATGCAGGAGGTTTGAAATGAACGCATTTTCACATGTTCCTCCTGGGTTTCGGTTCCATCCTACGGATGAAGAACTTGTTGATTACTACCTGAGGAAAAAGATAGCTTCGAAAAGGATTGATCTGGATGTGATAAAAGATGTGGATCTCTATAAAATTGAGCCATGGGATCTTCAAGGTACGTATGAGAACCACTAACATACATTAGTAGATAGAAATGAATTATAACTGGAGTGAGATGCATGCAGAAATATGCAAAATAGGAAGCGATGATGAAAATGAATGGTACTTCTTTAGCCATAAAGATAAGAAGTACCCAACAGGGACTCGCACCAATAGAGCTACAAAGGCAGGATTCTGGAAAGCCACGGGAAGAGACAAAGCCATATACTCAAGCTCAAGCCATTGCCTCGTTGGTATGAGAAAGACTCTTGTGTTCTACAAAGGACGAGCTCCCAATGGCCTCAAATCAAACTGGATCATGCACGAGTATCGTCTTGACTCCAATCAGGAAGATGGCTGGGTTGTGTGCAGAGTCTTCAAGAAGCGGATGCCCACGCTACGCAACGTGGTAGACTATGATGATCAACTTCCCTTCATGCAAGGATCTCCATCCACTCACTATCCCTGCAAGCACGAGCTTCATCAATTCCAATACAACACCCATGATGCTTTTCTCCAACTTCCACACCTTGAGAGCCCTAATCAAGTTTTGAGTTGCGGCAGCCCCGTTATTGCACCCTATGCCTACGCCGAAAACAACAACAACAATAATGGAACTAGTAGTACTAGTGCTTTGCAGTCCTATTCATCTGAACGCATTCAGCAACAACTTCACTTGCTTTATGGTAGCAATATTGAGCAAGCAGTAGTGATGGACCAAGTCACGGATTGGAGAGTGCTTGACAAATTTGTTGCTTCTCAACTCATGAGTCAAGATCAAGATCAGGCTTCCAAGGAAACCTGCAGCGTGGCTGATGAACAACATGTTGCTACTACTGTGCTTCCAAATGGATCCACGAAGCAGGAAATGGTGCCTCAGGACGACTATGTTTCAACGTCTGCCTCCAGTAACTGCGATATTCACCTGTGGAATTGA

>AdNAC45

ATGGGTGCGGTGGAGGTGTTTCAACAGCAGCCGCTGGTGGTGGACGCTGCTCCGGTTTTGTCGCTGAACTCGCTGCCGTTGGGGTTCCGTTTCCGACCGACAGACGAGGAGCTCGTTGACTTTTACTTGCGGCAGAAGATCAACGGCAATGGCGATGAGGTTTGGGTCATTCGAGAAATCGATGTTTGCAAATGGGAACCTTGGGATTTGCCTGGTACAATTCTCAATTCTCTGTTTTTTCAGGTTTCTATTGTAGTTTAATTCTTTGATTTTGCAGTGGAAAGGAGATTTTAAAGTTTTCTTGGTTTATGCTGTTGCAATTGTTTAACTGACTTAGTTTGACTTCATAATTCTAATTATGTGATTCATCTTAAAGTATGACAAGCTGAATTTTCTAATTATGCTCTTGAATGAAATAATAGAACTATGTTAGAATAATTTGAGTGAAATTGCAAGAGTTAATTTGGATGGCCATGCTATTTGCTCATTGTTTGTTCCTTTTGGTGGGGGTGTGGACTGTGGGATTTGTGTTTCTAGATTTGTCAGTGGTACGGAACAAGGATCCGGAGTGGTTCTTCTTCTGTCCACAGGACAGAGTCCGGCTCGACCGTGATTGGAATGAAGAAGACTCTTGTATTCTACACTGGCCGTGCTCCCAAAGGGAAGAGGACCAATTGGGTGATGCATGAGTACAGGCCTACCCTGCAGGAGCTTGATGGTACCAATCCTGGACAGGTATAGTCTTATCTTTGGTTTCTATTGTTCTGTGTATAGCTTTCGATCCGTCTTTATTATTATAGGGATGGCAGACATTGAATTGTCATTAACTCAAATCAATATGACTTCTCCACTAAATTCAAGTGTATGAAATGTGTGGGTCTTAAATTATATGATTGCCATATTTGATGAATATTGTGTTAAGTTTGTGCTTCTTCCAATTTGGTTCATAAAAATGATCATTTTTCATATTTGTGTTGATTTAATAAAGGAAAATATATTCAATTATTATTTCATCATTTATTTGTTTCTTCACTTACTTGGTGGTTGCTAGCTTGTTACTTCTTGAGTTCTTGTAATAGCTTCTCATAATAATTCTGCACCTGATTATTGTGGTTCTAAATTTTCTTGGTGATCTTTTATTTCTCTAGTTCAATTTCTAAGGTGCAACCTGCAATTGTTCACAGCCTACAGGTGTATGTAGGAATTAGCATTAAGCTACTGCAAATTATGACTAGCTCAGATGTTATATGAACTCATGAAGATAAGATATATTGCTAACTTTTCAATTTGGTTGATTTGACGTTTACCAGAATCCATATGTCCTTTGTCGATTATTTAAGAAACAAGATGAGAGTCTTGAAGGTTCAAACGGTGAAGAAATGGAGCGAACTACTTCAACTAATTTAACTGCCAATTACTCTCCAGAAGAAATACAATCAGATCCAGCTGTTAAATCGGTTTCTTCTTCACAGGCTACAGAAGATGACAAGAAACTAGCAGTTATCCCTTTGACCCCTGAAGAAGCAATTTCCAATGTTATAACCCCGGTCGGTTGCCAAAGCGATGGATGTGATGCTTATGATGCACAAAATCAAATCGCAGCAGGAGATCCATCTAAGGAGGTTTGATGAGCAAACTAATTTTTTGCACCCCATTTAGTTGATTTGCAGACTTTGTGTATGTTCAGTTATTAACCATAATTTGTTGACTAATGTAACTTTAGGAGGACTTACAGGTGAACATGGACATATTTTATGACCCGAGTGAGCTATTTGACGATAAATTATTCTCCCCACTTCACAAGCATATTCCAGAAGAACTTTTTCATCAATCAAACAATGAAGCCAATGGACATTTTGGGTTGCAACATCAGTGTGGAACAAATGAGATCAGTATTTCTGACTTCTTTGACTCTGTTATTAATTGGGATGAGATCTCCGGTGACAATTCCAGTGGCCAAACGCCAAACTCTGCTTGGTTTGATGTTCAGCACAATGAATCATGGGGAAACTCAAATGTGGATATGGTCCATGCCAGGGTGAGTGACAGTGTTAACCCTGTTTATCTTTTGGTCAGTTGAGGTTTTGCCACCCAACCAACATGTTACCTTTGAACTTGGAAGCACATGCTGGTGCTTAAGCCTTTTGTTAATTAATTTTTTTATTTCCCTCTAAATTGACGCAAATTATATGTGATATTATATTTGATTTTCTTTGGCATGGTTTGCTTATGTCGCTATATAATTTTAGCTGCTGTTATTTACCACAGCCCCTACAAGTAGGGGGTGCAGATTATCCAGGGGATGCAACCGAGGGAAAGCTCCCTTTGTTGAAAACTAGAGAATTCAATCCCAACACCTCTTATGACAATGCGATCAGCAACAACATGGGATTATTTCAGGACCATTCCCAGATGGCTTTTTCATCTGATGTTAATATGCTCCAAGGTTACCTTGCAACCAACAATTATGAGCAACCGACAAACTTCAATATGGCTATGGCTAATAGTGACAACACTGGAATTAGGATAAGGTCTCGGCCACCAGGTTATGAAGGGCCAAATGCAAACTCCAATATGCAACCACAAGGTACTGCACCTAGGAGAATACGGTTGGCACGAGCTCTTGCACCTCAACACACGTCCAATGAGGCGGCAAAAGATTCGAGTTACGAGTCAAAAGATCGAAATTCACAAGTAACCACTGCCAGAGTAAGCAGTCTTTTTGAATAACAACCTGTGTATCCAACTTGAGAGATGTCACTGCGTGTGGCGTAAGATTTTGTTGGTTCTTTTGCAGGAGATGGAAACTTCCAAAGACCTTGCAGCTGGTGAGAGTGTTACTGTTACTAGTGATGTGGAGGAACAGGAGTCATCACCAGTTGAAAATAAGGAATTTGAAGACTTCAACACAGTCCAGCAGAGCACATCATCAGCTTCCTCGAATCTTTCCACATGCTCTTCTGATTCTGAAGTTTCTTATGAGGCAGAAAAAGAATCCGGTTGGACATCAGAAGACCATAGTCCAAAACCAGCTGCCGCGGGGGTAAGAAGTCTGTTTGATTTACTTGTGTATATACTCTTAGATGTTGCTGCATATTCATTAAGATTTTTGCTGGTTCTTCTTTTGCAGGCCAGTAAAGCTTCCGAAGACCAAGTTCCCAGCGAGTGCATCAATGATATCACTGATGATGTGGATGAATCCAGGATACCAAACGCTTATACTCTAGAGGTCTCAAAGGAGGAATCCTTCTCGGACTCAGAGTCGAAAGACTCTCTATTGCGTAGAAAGGTGTGTTACCCATCGAAGTCTTCCTCAAATCTAGCCAAGTGGTATTCGGTTATTGCAATCTCAGCCACTTTGGTGGTGTTACTAGCATTCCTTGTTAATACATGGGGATATGGATATTACCTTAAAGTTTAACTGCATAGTAGGATATGTATCATTGCATAGAGCATATTTTGCCTCCATTGCGTTTTTGGCCTTAGTTAAGGAGGCAAGTATGTAAGGGTTGACTTTGTAGCTTTATTTGAATATAATCTGTATGTGCATATGTTATATATAGCAGAATATGCCTAATCTGCTACTAGCTTGTACTATATGTGGAGTTGTAAACTCATGTTTATAAGTTAGATTCAGAATTTCAGGGTTTCCAACTTTTTGGAATTTAATTTTGATTGCCCGTGGTGACCACCATTGGAGAATATGCCTGCCTAGCCAAGACCCAAGAGTGTATTTTTAGTAAAGAAAAGTTTAGGCAGATTCGGCTGTTTGATTCATTCATTTATTTATTCTATTTTTTGTTCTGTTGGTTTAAAGGTAAGCTTTTAGTTAAGAAGCTAAAAGATATAGAACATTGAAAATCTGGGGGAAAAAAGATAATGTATATATAAGAAATCA

>AiNAC1

GCAATAAACGACCAACTGGGTTGCCTTACATTCCACGTTTCTTTCTTTCTTTGAAATACAAACACAAACACAGAGAGAGTGGTATTTAGGGGGATTCTTTTTCTCTCAGCTTTTTTTACAAATCTCTCTCGATCACTTCCATGATTCTCATCACCTTTTCTTTCCCACTTTCTGCATACTCGATCTCTTCTCCTTCGATTCTCATCAGCAATTCCTAGAAAATTCTTATATTGAAAACCAAGGGTGACCCTTTTCATTTTGGGGGTTCTTACATATCAAATGGAAAACATTTGTTCAGAGGTTGAGATGGATTTGCCACCAGGATTCAGGTTTCACCCAACTGATGAAGAGCTTATAAGTCATTACCTTTACAACAAGGTCATTGACACTAACTTTTCAGCCAGAGCCATTGCTGAGGTGGACTTGAATAGGTCTGAGCCTTGGGATTTGCCATGTGAGTTCACACTAAACTAATTCAGTTTCTTATTTGGTTTTGTTGAAATATATATATATAAAGATTATATTGTTTAGGGAAGGCGAAAATGGGTGAAAAAGAATGGTACTTTTTCTGTGTAAGGGACAGAAAGTACCCAACAGGATTGAGGACAAACAGAGCAACAGAAGCAGGGTATTGGAAGGCCACTGGAAAAGACAAGGAGATATACAGAGGCAAATCACTTGTTGGCATGAAGAAGACCCTTGTCTTCTACAAAGGTAGGGCTCCCAAAGGTGAGAAATCTGATTGGGTCATGCATGAGTTCAGGCTTCATGGTAAATTCAATCCCCACAACCTCCCCAAATCTGCAAAGGTACCTCCTTTTCTTCTCCTCTGTTTTCAAACTCATACATCCATCATCATTAACTAGTTCTAGCTTGTGCAGAACGAGTGGGTGATTTGCAGGGTGTTTCAGAAGTCTTCAGCCGCCAAGAAAATCCATCTTACCGGGATAATGAGGTTGGACTCTTCTGTTTTCTTGCCACCATTGGCAGATTCCTCATCATCACCTTCCAACACTGCTACTACAGCACCTTACGTGCCCTGCTTCTCCAATCCAATCATTCACAACCAAGTTGGGATCTTTGATCCATTTAGCAACACCCCTTTTGGTGCTGATTCATTCTACACTTCTCAAGGGATGCCAATGCAACATGCTCAACCACCAAGTTGCTACACCACTCAGGACCATTCAATTCTCAGAACCTTGCTTCAAAACAATTCTTCAAACCTCAGGAGTGGTTTCAAGCCTGCAGAGAGGGAAATGGCCCATCATCAAACTTCTCTTGTTGATGCCAACAACAACAACAACAACAATGGAATCACTTCTGTTGTTGCCCCACAGGACCTTTCTAGCCTCTGGAATTACCAGGTTCAGATCAAGTAGCTATGGAAAGTGAATTCTGAGGGTACAAGAGAAGCCATTCTGTTGTGATTTATTCTGTCTTTTGGTGTTGTTGCTTGTCTGAATTGATTTTAAGTAAATCATGTATGAATATTTTGTGATGTCAGTAATAATCTTGTGAATTTATACTATTTTGGTCCTTTTGATACTTTGCTATTGTTATGGTTG

>AdNAC9

GTGTATTTAAGCACACCTTCTTCCCTTCATTTTACTTTCTTAATCTTTCATCACTTCATAATTAATTCCTCATCGCTATAATGGATGCGGAAGATCATAATCATGCTTTGGATTTGCCTCCCGGTTTCAGGTTCCACCCTACAGATGAGGAGATCATCTCTTATTATCTCACTCACAAGGTTTTGAACACAAGTTTCACCGCAACTGCCATTGGAGAAGTTGATCTCAATAAGTGTGAGCCTTGGGACTTGCCTCGTAAGTCACCATTTCTTGCTTCTTTCTCTTTATTTCGTGAAAGCAAACTACATATTTGATTAAATTGTTTTGTTATTGTGAATGTTTAGAGAAAGCAAAGATGGGGGAGAAAGATTGGTACTTCTTCTGGCAAAGAGATAAAAAGTACCCAACTGGGATCAGAACGAATCGAGCCACGGAATCCGGCTACTGGAAGGCCACAGGAAAAGACAAAGAGATTTACAAAGGGAGAAACCTTGTTGGTATGAAGAAAACCCTTGTGTTCTATAGAGGTAGAGCCCCTCATGGACACAAAACCAATTGGGTTATGCATGAATTCAGATTGGAAGGCCTTTTTGCTACTTACAACCTCCCTAAACCTGCTAAGGTACTCATCAATTTCTTTAGTAAAAATTCACCAACAATTGTGTTCAATTGTTAGATGATTTAATATGTTGATTAAATTGTTATTTAACGATTTTTAAATATTAATTTTATAAAAATAACAAGTGTACGTGAATGGTCACCTAATTATATATATGTAGTCTCAAAAATAATAACTTAAAACTGTTAAATAATTTAATATATTTAACTAAACTGTTTAAAGATTATTTGATTTTGGTGAAAACTCAGGTTCAGTTAACTTCACGTGAAATTGATACCTGAAAGTCGTTACATGATTTGATTGATTTGATTAAATTTTCATCTAACAGACTCTCAGGTATCAGCTTCACGTAAAGTCGACTGTATCTGAGTTTTCACTTTGATTTTTGTATATGTTGTTTTTCAAATATTGCTTTTCTCTTTTGCAGGAGGAATGGGTTGTGTCGAGGGTTTTCCATAAGAATACAACAGAAAAATTGAACCCAACTATTCCATCTGGCCTCTTTAGGATAATGAAGAACGTGAACTCAATTGAGGATGATGATCTTGTAGATTTTTCTTCTCTCCCACCTCTCATGGATCCTTCTAATAATTATGATGATGAACACACCACCACCACCAACAATATGTTTGCATCATCATCAGATTATAATATTACTATTCAGCAAAACAAGAAGGATATGATGGGAATAAGGAATAATAATATTAGAGCATTATTAATGTATGACGGTCCATCATCATCATCAGAAGTAGTTGCTCCTCCTCTCTCTGACTTGGAATTATGCCTCTGGGATTTATAACTTCTTACCAAGATTATTATGACAAGACACAGACATATTTAATCA

>AdNAC13

TTCTCTCAGCTTTTTTTACAAATCTCTCTCGATCACTTCCTTTCTGCATACTCGATCTCTTCTCTTTCGATTCTCATCACCAATTCCTAGAATTCACCCTTTCATTTTGGGGGTTCTTACATATCAAATGGAAAACATTTGTTCAGAGGTTGAGATGGATTTGCCACCAGGATTCAGGTTTCACCCAACTGATGAAGAGCTTATAAGTCATTACCTTTACAACAAGGTCATTGACACTAACTTTTCAGCCAGAGCCATTGCTGAGGTGGACTTGAATAGGTCTGAGCCTTGGGATTTGCCATGTGAGTTCACACTAAACTAAACTAATTCAGTTTCTTAGGTTTTGTTGAAATATATATATATATAAAGATTATGTTGTTTAGGGAAGGCGAAAATGGGTGAAAAAGAGTGGTACTTTTTCTGTGTAAGGGACAGAAAATACCCAACAGGATTGAGGACAAACAGAGCAACAGAAGCAGGGTATTGGAAGGCCACTGGAAAAGACAAGGAGATATACAGAGGCAAATCACTTGTTGGCATGAAGAAGACCCTTGTCTTCTACAAAGGTAGGGCTCCCAAAGGTGAGAAATCTGATTGGGTCATGCATGAGTTCAGGCTTCATGGTAAATTCAATCCCCACAACCTCCCCAAATCTGCAAAGGTACCTCCTTTTCTTCTCCTCTGTTTTCAAACTCATACATCCATCATCATTAACTAGTTCTAGCTTGTGCAGAACGAGTGGGTGATTTGCAGGGTGTTTCAGAAGTCTTCAGCCGCCAAGAAAATCCATCTTACCGGGATAATGAGGTTGGACTCTTCTGTTTTCTTGCCACCATTGGCAGATTCCTCATCATCACCTTCCAACACTGCTACTACAGCACCTTACGTGCCCTGCTTCTCCAATCCAATCATTCACAACCAAGTTGGGATCTTTGATCCATTTAGCAACACCCCTTTTGGTGCTGATTCATTCTACTCTTCTCAAGGGATGCCAATGCAACATGCTCAGCCACCAAGTTCCACATATTCCGCTTCCGGCTACACCACTCATGACCATTCAATTCTCAGAGCCTTGCTTCAAAACAATTCTTCAAACCTCAGGAGTGGTTTCAAGCCTGCAGAGAGGGAAATGTCCCATCATCAAACTTCTCTTGTTGATGCCAATAACAACAACAATGGAATCACTTCTGTTGTTGCCCCACAGGACCTTTCTAGCCTCTGGAATTACCAGGTTCAGATCAAGTAGCTATGGAAAGTGAATTCTGAGGGTACAAGAGAAGCCATTCTGTTGTGATTTATTCTCTGTCTTTTGGTGTTGTTGCTTGTCTTAATTGATTTTAAGTAAACCATGTATGAATATTTTGTGATGTCAGTAATAATCGTGTGAATTTATACTATTTTGGTCCTTTTGATACTTTGCTATTGTTATGGTTGCAATGACCTAAATTATCATTATC

>AdNAC54

CATTCATCTCATTGCAAACATTTCAAACACCTTGATATCTCTTCAACCCTTATCTTCAATTCATCCCCATCCTCTATCTCTTGTTGTGTCCCCACTATAATTAATGGAGGGTAGTAGAAGAAGCTCAAATTCTGAACTCCCGCCTGGGTTTCGGTTTCACCCAACTGATGAAGAACTAATCGTTCACTACCTTTGTAACCAAGCCACTTCAAAGCCTTGCCCTGCTTCCGTCATCCCTGAAGTTGATATCTATAAGTTTGATCCATGGGAATTACCCGGTACATATAATTATTCTCCTTAATTTATTAGCCTAGTCTCTCTGGTTGTTTTAATTAATTCTTGTGATTTCAGATAAAACAAGCTTTGGAGAGAACGAATGGTACTTCTTTAGCCCAAGGGATAGGAAGTACCCAAATGGGGTGAGGCCTAATAGGGCAACGGTTTCAGGGTATTGGAAGGCCACTGGTACGGACAAAGCAATCTATAGTGGGTCTAAGCATGTTGGGGTCAAGAAAGCTTTGGTCTTTTACAAGGGTAGGCCCCCAAAGGGTATCAAGACTGATTGGATCATGCATGAGTATAGATTGGTTGGATCAAGAAGGCAACCCACTAAACAAATTGGATCCATGAGGGTAACCCCTTTTCGCTCTTTTCTGACCAAAACACAAAATAAAAAATATTATTATACAAAGATAGAAATATTGTCTCTGCTAATAAATTTGGGTTGATGAAATACAATACTTCATGATTTTGTATTTGTATGATATTTTGATGTGTTGGATTAAGTGGAAGCTTACCCTTTTGGATGTGTCATCATCAGCTAGATGACTGGGTGTTATGCAGGATCTACAAGAAGAGGAGCATAGCAAAATCAATGTTGGAGCCTAAAGAGGAATTCCCAACAATGCCCCAAATCAATCATCATCTAACATCATCATCAAATGATGGGAATGATAATAATGATGATGAGCAAGAAATGATGATGAAATTCCCAAGGACATGTTCCCTTACACATCTCTTGGAAATGGACTACTTGGGCCCAATATCACAAATACTCTCTGATGGATCATATAACTCAACCTTTGATTTTCAACTAAACAGTGCCAATGTTGGCAACATGATTATGGACCCTTTTGTGAAACAACCTCAGATCCTTGAAATCCCTAACAAAAATAATCCTAACAATCCTTATTATGATGTGGATTCAGGGAAGAACAACCTAGTGAAACAGAATAGCACCATAAACCCTACTATATTTGTGAACCAATTCTTTGATCATAGTGGTAGTTAA

>AiNAC13

ACCTTGATTTCTCTTCAACCCTTATCTTCAATTCATCCCCATCCTCTTTCTCTTGTTGTGTCCCCACTATAGTCCCAAAAATAATAACCAATTTAATTAATGGAGGGTAGTAGAAGAAGCTCAAATTCTGAACTCCCTCCTGGGTTTCGGTTTCACCCAACTGATGAAGAACTAATCGTTCACTACCTTTGTAACCAAGCCACTTCAAAGCCTTGCCCTGCTTCCGTCATCCCTGAAGTTGATATCTATAAGTTTGATCCATGGGAATTACCCGGTACATATCATTATTATTTAATTTATTAGCCTAGTCTCTGTGGTTGTTTTAATTAATTCTTGTGATTTCAGATAAAACAAGCTTTGGAGAGAACGAATGGTACTTCTTTAGCCCAAGGGATAGGAAGTACCCAAATGGGGTGAGGCCTAATAGGGCAACGGTTTCAGGGTATTGGAAGGCCACTGGTACGGACAAAGCAATCTATAGTGGGTCTAAGCATGTTGGGGTCAAGAAAGCTTTGGTCTTTTACAAGGGTAGGCCCCCAAAGGGTATCAAGACTGATTGGATCATGCATGAGTATAGATTGGTTGGATCAAGAAGGCAACCCACTAAACAAATTGGATCCATGAGGGTAACCCCTTTTCGCTCTTTAACTCATCATCTTTTTCACACCAAAACAGAAAATAAAAATTAGTCATCATATTTTAGAGTAATTATACAAAGATAGAAATATTGTCTCTTAAATTTGGGTTGATTATTCATTCACTTTTATTCACTTTTGGTTTGTATATATAATAATTCATGACTAATAATAGACATTTAAATATTGTTTTTCTATATTTTGGATAAACAGACAAAAGAAAATTCTAAAACTAAAACATCTTGTCTATCTATTGATAATGTTTTATCCTGCAGTCAAATTTGTATATTATTCTCAATATTTTGTATTTGTCTGATATTTTGATGTGTTGGATTAAGTGGAAGCTTACGCTTTTTTGTGATTTTTGGGATGTGTCATCATCAGCTAGATGACTGGGTGTTATGCAGGATCTACAAGAAGAGGAGCATAGCAAAATCAATGTTGGAGCCTAAAGAGGAATTCCCAACAATGCCCCAAATCAATCATCATCTAACATCATCATCAAATGATGGGAATGATAATAATGATGATGAGCAAGAAATGATGATGAAATTCCCAAGGACATGTTCCCTTACACATCTTTTGGAAATGGACTACTTGGGCCCAATATCACAAATACTCTCTGATGGATCATATAACTCAACCTTTGATTTTCAACTAAACAGTGCCAATGTTGGCAACATGATTATGGACCCTTTTATGAAACAACCTCAGATCCTTGAAATCCCTAACAAAAATAATCATAACAATCCTTATTATGATGTGGATTCAGGGAAGAACAACCTAGTGAAACAGAATAGCACCATAAACCCTACTATATTTGTGAACCAATTCTTTGATCATAGTGGTAGTTAA

>AdNAC26

ATGGGGGATAGCAATAATGTCAATCTTCCACCGGGGTTTCGATTTTATCCCACTGATGAAGAGCTTGTAGTCCATTTCCTTCAGAGAAAAGCAGCACTTCTACCTTGCCACCCTGATGTCATTCCTGATCTTGATCTCTACCCTTTTGATCCATGGGAACTTGATGGTATCATTCTTTCTCTATGTTCCAATACTTGCATGCACATTGCAAGAGAATTGAAGTTGATGAATACAATCATGTGTGTGTCTGTGTGTGTATGTGCAGGTAGAGCTTTGGCAGAGGGGAACCAATGGTACTACTACAGCAGAAGGACACAAAATAGGGTCACTGCCAATGGTTATTGGAATCCAATGGGAATTGAAGAGGCAGTGGTTTCAAACTCAAGCAACAGGAGAGTTGGTATCAAGAAATTTTATGTGTTCTATGTTGGAGAAGCCCCTCATGGTAACAGAACCAATTGGATCATGCAAGAGTATCGTCTTTCAGATTCTGCAGCATCCTCTAGCAGATCATCAACCAAAAGAAAATCACAACCAAAAACAGTTAGTACCACAATTTCACCTCAAAAACATAGTTCAAGTTCAATAATGTATGCCACTTAGATTATTATTATTATTGATTTTTTTTCCTTCCTGGTTTTGGTGTATTCAGGATCATAGTAAATGGGTGGTATGTAGAGTTCATGAAAGTGATGAAAATGATGATGATGGTGATGGTGATGGAACAGAACTCTCTTGTTTGGATGAAGTTTTCTTGTCATTGGATGATCTTGATGAAGTAAGCTTGCCAAATTAG

>AdNAC3

TTCCTTCCTTCCAAGTTCAAATTAAATTTCAGCTAAGTAGTAGTAGTAGTAAGTGATTATTCTAGCTTGATGGGTTCTTCTAATAACGGTGGTGTGCCACCGGGGTTTCGATTTCATCCAACTGATGAGGAATTGCTTCATTACTACTTGAAGAAGAAGGTGTCGTTTCAGAAGTTTGACATGGATGTTATTAGAGAGGTCGACCTCAACAAGATGGAGCCTTGGGACTTGCAAGGTAATTAAATTAACTTAATGTAATTTTCTTTAATTCCCTTAAGCTAATAAGTAGGCCTTTTTTCTTTTAAGTTTTGAGCTTAATTCTTGCTTTATCTCCCGTTTTCGTTACTCAATTCATATTATTGATTTTTACTATATAATAAACTAGTATGATAATAAACAAGATGACATAACTTGCATTGATGGATTGTTTTCCAACACCGATCTATCTGCTGAAATTAATCTTGAAACGTAAATTTCTTAATTATTACTACATGGTGGATGAATGACAGGGTTTATTATGAGTTGTTGTAAGCTTATATGTTCGTGAATAAGATAATGGAATTAGTAGCCCATGGTGCATATATTATTTGACTTGTTTTGTTTAATTTGATGCATAACTGCCTCGTAGTATAAGTTGATATGAGGAGAATGTCACTCAAGTACCTAACTTGATGGAAAATCTATGATTTATCATACTCTTGGATTTGTTGGAGGTTCACGAATCGAATCTTGTGCACAATTACTAGGGCGGCATATATGTAGAGACATCATATATTTATATAGAAATACAAATGATGATGTATGCAGAAAGATGCAGAATAGGGTCAACACCACAAAACGAGTGGTATTTCTTCAGCCACAAGGATAGAAAGTACCCAACAGGGTCAAGGACAAACCGAGCAACGAACGCAGGGTTCTGGAAAGCCACGGGAAGAGACAAGTGCATAAGGAACACCTACAAGAAGATTGGGATGAGAAAGACACTAGTGTTCTACAAAGGTAGAGCCCCTCATGGCCAGAAGACTGATTGGATCATGCACGAGTACCGTCTTGAAGATTCCAATGATCCTCAAGCAAATGCCAACGTATGTATATCTAACTCATTTTCTCGTGAATTAAATGACTAGTTAGATATCTAACTATATATTATTGATATTGTTTTGTAGGAAGATGGGTGGGTGGTGTGCAGGGTGTTCAAGAAGAAGAACCTATTCAAGATTGGAAATGAAGGAGGTGGTGGCTCCACACACACCTCATCGGACCAGCAACTCAACAACTCAACGGCCACCAATGCTCGTTCCTTCATGCAAAGGGAAAACCACTACCTACTGCATCACCACCAACAACAGCAAAACCCTAGGAATGGGAACCCATCGTCTTCATCCTCAGGCTTTGATGAGCTCGATAAACCCGAACTCGGTCTCCATCACTATCCTCACATGCAAAACCCACACTATTCACTCTTCCATCACTCCCAACCACTTCTTCATCCCCAGGCCCACAAGCCCATCGTCTATGACTACTCTTATACACCCGCGCTTCCCTCAGACCCTCCTGTCACCGCTAAGCAGCTCATGACTAACCCTAGAGACTGCGATAGCGGTGGCAGCGAGAGTCTGAGGTACCAGCAGGTTTCCGAGCCTGGTATGGAGGTTGGATCATGTGAACAAGCCCAGGAAATGGGCGCCGCCGCGGCCGCAAGAGGAGGAGGAGAAGGAATGAATGAATGGGGTGTGCTTGATAGGCTTGTAACCGGGAACCTTGGAAATGAAGATTCAGCCAACAAAGGGATTAGGTTTGAAGATGCAAATCCACACCAGATTAACCAGCTTTCTTTGAGGGGAGAGATGGATTTCTGGGGCTATGGAAAACAATAA

>AiNAC49

TTTCCTTCCTTCCAAGTTCAAATTAAATTTCAGCTAAGTAGTAGTAGTAGTAGTAAGTGATTATTCTAGCTTGATGGGTTCTTCTAATAACGGTGGTGTGCCACCGGGGTTTCGATTTCATCCAACCGATGAGGAATTGCTTCATTACTACTTGAAGAAGAAGGTGTCGTTTCAGAAGTTTGACATGGATGTTATTAGAGAGGTCGACCTCAACAAGATGGAGCCTTGGGACTTGCAAGGTAATTAAATTAACTTAATGTAATTTCCTTTAGTTCTTATAAGTTTTGAGCTTAATTCTTGTTTTATCTCCCGTTTTCGTTACTCAATTCATATTATTGATTTTTACTATATAATAAACTAGTATGATAATAAACAAGATGACATAACTTGCATTGTATGGATTGTTTTCCAACACCGATCTATCTGCTGAAATTAATCTTGAAACGTAAATTTATTAATTATTACTACATGGTGGATGAATGACAGGGTTTATTATGAGTTGTTGTAAGCTTATATGTTGGTGAATAAAAGAATGGAATTAGTAGGCCATGGTGCATATATTATTTGACTTGTTTTGTTCAATTTGATGCATAACTGCCTCGTAGTATAATTTGATATCAGGAGACTGTCACTCAAGTATATATATTTTCGTGTATGATTTATCATACTCTTGGATTTGTTGGAGGTTCATGAATGGAATCTTGTTATGCAGAAAGATGCAGAATAGGGTCAACACCACAAAACGAGTGGTATTTCTTCAGCCACAAGGATAGAAAGTACCCAACAGGGTCAAGGACAAACCGAGCAACGAACGCAGGGTTCTGGAAAGCGACGGGAAGAGACAAGTGCATAAGGAACACCTACAAGAAGATTGGGATGAGAAAGACACTTGTGTTCTACAAAGGTAGAGCCCCTCATGGCCAGAAGACTGATTGGATAATGCACGAGTACCGCCTTGAAGATTCCAATGATCCACAAGCAAATGCCAACGTATGTATATCTAACTCATTTTGTCGTGAATTAAATGACTAGTTAGATATCTACTATATATTGTTGATATTGTTTTGTAGGAAGATGGGTGGGTGGTGTGCAGGGTGTTCAAGAAGAAGAACCTATTCAAGATTGGAAATGAAGGAGGTGGTGGCTCCACACACACCTCATCGGACCAGCAACTCAACAACTCAACGGCCACCAATGCTCGTTCCTTCATGCAAAGGGAAAACCACTACCTACTGCAAAACCCTAGGAATGGGAACCCATCCTCTTCATCCTCAGGCTTTGATGAACTCGATAAACCCGAGCTCGGTCTCCATCACTATCCTCACATGCAAACCCCACACTATTCACTCTTCCATCACTCCCAACCACTTCTTCATCCCCAGGCCCACAAGCCCATCGTCTATGACTACTCTTATGCACCCGCCCTTCCTTCAGACCCTCCTGTCATCGCTAAGCAGCTCATGACTAACCCTAGAGACTGCGATAGCGGTGGCAGCGAGGGTCTGAGGTACCAGCAGGTTTCCGAGCCTGGTATGGAGGTTGGATCATGTGAACAAGCCCAGGAAATGGGCGCCGCAAGAGGAGGAGGAGAAGGAATGAATGAATGGGGTGTGCTTGATAGGCTTGTAACCGGGAACCTTGGAAATGAAGATTCAGCCAACAAAGGGATTAGGTTTGAAGATGCAAATCCACATCAGATTAACCAGCTTTCTTTGAGGGGAGAGATGGATTTCTGGGGCTATGGGAAACAATAACTTCCTCCATGATTGAAGATAAATAAGAGTTCCATCCTTGCCTCTAATTAACCATTTAATTAAATATGGGGAGAAA

>AdNAC11

TTGGCGACCAAGGATGCAAGGTTAAACTTGTAATTCTGGCCCTTACACGGTTTGCGTCTTTACGAAACTCGACTTTCTCTTTCCGGCTCAATATCCGAACCAATAGGCTCGGTTTTGCTTTGGCTCATTTGTTCGACAGAACAGCAATGACGAAGCACAGGTTCGAATGCACAGTCTCTTCCCATTACCAACAAAAACCCCAGGCTACAATTTCCCGCTCACTCTACCGACTGTCGCTTTCTTTGTCTCTCTGTCGATTGAAAATGGGAAACCGATGCTGAGGCTCAATACTCTCCACAGTTCTGCTTCAGCTCAAGCTTCTTCCTCTTCCTCTCTGCAATGGCTAGGTGAGGTTTAGAATCAGATTCAGATATGGCCACATTGCATAATGTGATACACGTTTTCTTCCTTAGTAACTTTTGTGTGTGTGTTACGCTTTCATTTTTTTTTCTTAAAAAATCATTTTTAATTTTTGATTTTAACTTAATTGTGGTTTGGATGCATTTTGTGGCATTTTATTTTCCTATTTTGGGGTTATTGACATGGACGTGCTTCAGTTTTGGGAAAGCAAACTGTAGTCTGTAGTGTAGGGTTTGACATGCTTGAGTTTGGTAAATAGCTACTGCTACTTTTTTCTCTTGTTCCAATTAAAGTAAACAAAGTGTTTCTTGAAGTGGTCCTGATTGCTAAACATGATTACATGAATCCTTATTGGAATATTTCAAATGACATTATGATAGTGTTAGTCACAGTATACTATGTTCTTACACAAGAAGTTACATTTTGTGCTTTGGCTTTGTATGAGTCCTTTTTTCTACCCTGATTGAAGCTGTTGGTGAAGAAAAAGCTTTCTGAAGGGAATTTCATGGTGAAAATCTCACTATGTTGTAAATGATTAATAATGAGTATCCTGGTATCTTGCCCTTCTAATTTACTCCTTGCCATGCATTTCTAATTTTGATTACAATCTGTAATATGCTGTGTTTTTGTGGTTATGGAATCTTTAAGCTGTGTTTGTGAATTGAGGTTTAGAAGGGGAAGGGAAAGAAGTGAAAGGTTTGAAGAGTAAAAGGTAATTGAATCATAATTATATGACTGTTTCACTCTACAAGCCCTTCTCATTACTCTTCAAACTCTTCCTATCCCTTATTTCGGTAGAAAACCTCATATTGACATTACTGCCTTCTGGCCTCCGGATTCTGTGCAGTACAGAATTCCCTGTGACTGTTTCTTTTTTTCATTTGACTTCATTTCTACTTCTCTTTAACTGTATTGGACCTAGAAATTCGCCATTGTTATTCCTAATTATTTATTCCATTTTCGTATTTATCATGTAAAGAAGCTGAATTTTCACACTTTTATTTAAATCTAGCTATGCTTTTAAAGTTCTTCAGCTAACAATCTCTTCTTCTTTATCTTCCAATGGTATGATGAACTTAGGAATGTTTGCTATTTTATTTGGCAGGAGCTGGCTTATTGACATAGGTGGATTTGCAAAGAAAGTGAAAAGCACTAATTTATCTCCAGCTGATCAAATCAAAGATTGTGGGGCATATCGTGATTGTCCAAACTGCCATTACCGTATTGATAACCGTGATGTACGTTAAAATTCATCATAATATTCATTCTCATTTTATAGTTGTTAGGTAAATTGACACACACAGCTCAACATAGAGTCAACATAGAGGCAATGAACTGGCTTTTATATAATAAAGCCTTCAATGATCATTTGTAGTTTCGTTATGGTCCATTCTCTTTTCCCTATATTTTACTTTTAACTTTGGATTTAGCTAACAAGGGCACATGTTAAAGTTACGAATTAAGAAAGTTATTATAGAAAATGTGTAGAGTTTAGTTTTCAATGCAAATGTTGTGCATCTATTAAAATAAAACATTTAAGACTTTCTACTAATACTAATCTTAACTTGTGCCCTTTAGTAAAACCCTTTAACTTTTGGCTAATATAATTCCTTCATTTATTTGATGTTGTGCTTGTGTGAGTTTGCAAACTTAACTATCATACCAGGATTGAAGAATTTTGGCATGGGTTTGAATGATTGCAGATGTTTAGTTATGAATATTTCTTCTTCTTCTTCCTTTTATTTTTTTAAATAGGTTTCTACTGAGTGGCCTGGCTTTCCTCTTGGTGTGAAGTTTGATCCTTCTGATGTAGAACTCCTAGAACATTTAGCAGGAAAATGTGGCATTGGAAATGCTCAGCTGCATATGTTTATTAATGAGTTCATTCCAACAATAGAAGAAGAAGAAGGTATTTGCTATACACATCCAGAAAATCTTCCAGGTGAGGTTTCTCTATGCTTTTGCTTCCAAAAATTTAAAACATTAAACATGTGAAAGGATTCTTTTCTGTATGCTCTTCACCTTGATACAGAAAATAATTCTTTCACATTGACCTTATGAGACTTGTGTTTTGCACTATAAAGCAAAACCCAATCAATGTTCAGATCATGTTAATTGAAAGCCATGTTGGAAATACATTACATGTTGCTTTTACTGTTATATGTTGAGGGTTAGGGAACAACAGGTTTTATGATTGCCGAACTTAATGATGTTGCTTTTATGAATGATAATCATTTCATAATCCCTTATGTGTTCTGAATATTGAGTTTTTCTTGTGGACTTACATTGGCCCACTTTTCTGGAGCTATGTCTCTAATCATCATGACTGTTTTATTTCAGGTGTCAAGAAAGATGGGAGCAGTGCCCATTTCTTTCACAGAACAACCAATGCCTATACTACTGGTCAACGGAAGCGTCGAAAGATTCATCATGAATGTTTGACTGAAGAGCATGTACGGTGGCATAAGACTGGTAAGACAAAAGCTATATTGGAGGATGGAGTGCATAAGGGCTTTAAGAAGATCATGGTTCTTTATATAAGACCTAAGAAAGGGTCCAAACCTGATAAAACGAATTGGGTGATGCACCAATACCATTTAGGAACTGATGAAGAGGAGAAGAACGGTGAATATGTGGTTTCAAAGATTTTTCAGAAGCAAACTGAGAAAAATGAGGAGAATCCAGCGGTCGAAGATTCTGACCAGACTGAGAAAAATGAGAATCGATTGGCTGATGATTCCAACTGTATAGCATCCCGAACCAGTCCTAGAACTCCGAAACCAAATCCCCCAAATCCACCTCGAGCTGGAAATTTTGTTGACAATGATGATAATATTGACGAAACTGAACTTCCATTCACTCAGGTAGTAGCTGTATTTCATAATTATTAATACAACTGTTAAATCATCTTCTAGAAACATCAAATGGAAAAGAGAAGTCAAATCTGATATATTTCCTCCCTAACTAGTTCGCACATAATCTAAGGATTAACTTGGCTTTCAACATGTATTTATGTATGACTGATGGAACAAGTTCTACGCTTTGTTTTTTCTTAAACTTGTCCACATTTAGTTTCTAAAATTTGAACTTTTTGAACTAGGATGTGAAATGTGTCCCACTATGTGATGTTCTGGATCAGAACAATGCTGGTGACCCTGCATGGCTGGCAGGTGAATCGCAGGCTGTGGAAAACTTCGACTTTGATGGCTTGGATGACATCTTGTTCTGCAATGAAATATTTGATTCATCATCTCTACTAGATGTTTCTGGAACGGAAACCATGATAAATGGATCTGCTTCAAACGATATGCTTGGGAATGATAGTTTATCATACGGAACTTCCGTTCTTGATACCCTTGACTTGGGTACTCCCCCAGATTTTGATCTTTCAGTAAGCATCCAGTTACCCTGCTCCCTGTATCTTTGATTTTGAGTTTGTACATTTACTAACAAGCTCTACTATTCTCACTTTGGATGCAGAATCTGAATTTTTACTCTCAAGATAGTATTTTCGACTGGGTCGACAGATTATGAAGTGATTTCTGAAGTTTGAGTCCATCTGAATGCATGTTTGTTCAGCTCTTAGATGCTTCAGATTCAAATATAGTAAGTGAAACATAGGTTCTCACGTGATGATTTGCAAGCAGAAATAACAGTATTTGTTTGTCCCTGGTTCTTGAAATTCTGCAAATTTTGTAATCCCATGTAAACACATTTGGGCATTGTTCAGGTGGGTGTATTTTGGTGTTCATGTGGATTTTAGGCATTATTCACGTGGGTTGTATTTGTGTGTCAAATAGCTTTTGTGGTATATGTTGTTTCCATAGCCATCTAAACTTGGTTGCCTCATTCTGTTGTGGTAGATACATCTCTGGTAGCAAAGTCCAAACTTATATAAGTACTTGATATTTGACTGTTTTTATGTCAATACTAGAATAACTATCCGC

>AdNAC12

ATGGCAGAGCTAAGTGCGGCTGCAACCTTCACACCCAGCGATGAAGAACTCATTCATTTCCTCTCCGACAAGGTGAAAGGCCAATCCATGGACGAGGACGCCGCCATCAACATCCACGAATGTGAATACTTGTACGGCCGCAACAAGAACCCTTGGGACATTTGGCGTGACTTCGCGGGCGACGTTGATGCCGGCAGGACCGCCCTTTTCTTCTTCTCCCCCAACAAGAAGCACCATTCCACCGCCTCTCGCCCCATCGGAGCCGGCGTCTGGGAAGCTGAAGCAGAAACCATTGACGGCGAAGGCATCGTTGGCAAGGGCAAGAACCGCCGTATTGGGACCAAGAAATGTTTCATCTTTGACAAGAGTGGCACCTCCTACGATGGTGCATGGATCTTGCATGAATACACTCTTCATGGATCGTCGCTCCACACTAATACTTCAGTAATTACCACTAATCAATGCCTCTAGTCTCATTAAAGAGTTCAAAATTTCTTTTCATATTCTTGTGTGTATATGATCAAATTTTTGCTAACATGTGTCTTTTAATATTAATTTTGTCCTATATTTCTTATTTTTAGAATCTATTAGCATTCGCCTTAATAAATACATTAATCTCATTAAAAAAAAGTTAATATTACCTTTTAATATTAGTATATACCTGCTAAATTAAGATTTTATGTATAAGTATTCTAAAAACTTTTTCAATATTTACGCATTTTTCTTTCATCCAATATCAATATCTAAATTATCATTGTGTTGGTAGCAGGTGGATAATAGCTATGTTATATGCAAATTGATAAAGAATGTAGAAGGTGAAGCTCATCCAGTTGAGGTGCAGTTTGGAGACAAAAGAAAAAGGCATGCGCAATCCGCCACCACCAGCGGCGTTCAAATTGATGTTAACGCTCCTCATTCATATGTAAGTGCTCAACACATTGAATATAATATACATTACATGAAACTTTGTAGGTGGTGCTGACCACTTGTCATATATATTTTAAGAGGAACACTAAAGAACAAGAGGTCCAATTCATACCAAATGAACTTGGCAGGCGAATGTTGTTGGAAAGGTTTGAGGGTAAGTATGTATATAATTTATTTTGAATGTGAATTTATATTTTAATATATATTTTATATTAGTGACTGATTTTGATATACACATAGTTATTAAATAATTTGGTATACGTATGTACATTTTGCTTCAAAGTACATTCTGTTATATTAGGAAATATGATGAGGATGGTTTAATTCTTTCTTTCATTGTTAAACAAGGATTACCAATCACGAACGAGAACCTCATCAGAAACCTCATGCAGCTGCAAGGGGGAACAAGAAGAGGTGGAAAAGGCCTCTAA

>AiNAC78

ATGGCAGAGCTAAGTGCGGCCGCAACCTTCACACCCAGCGATGAAGAACTCATTCATTTCCTTTCCGACAAGGTGAAAGGCCAATCAATGGACGAGGACGCCGCCATCAACATCCACGAATGTGAATACTTGTACGGCCGCAACAAGAACCCTTGGGACATTTGGCGGGACTTCGCCGGCGACGTTGATGCCGGCAGGACCGCCCTTTTCTTCTTCTCTCCCACCAAAAAGCACCATTCCACAGCCTCTCGCCCCATCGGAGCCGGCGTCTGGGAAGCTGAAGCCGAAACCATTGACGGCGAAAGCATCGTTGGCAAGGGCAAGAACCGCCGTATTGGGACCAAGAAATGTTTCGTCTTTGACAAGAGTGGCACCTCCTACGATGGTGCATGGATCTTGCATGAATACACTCTTCATGGATCCTCGCTCCACACTAATACTTCAGTAATTACTACTAATCAATGCCTCTAGTCTCATTAAAGAGTTCAAAATTTCTTTTCATATACTTGTGTGTATATGATCACATTTTTGCTAACATGTCTCTTTTAATATTAATTTTGATAATTATCATTAACATTCTTCGTCTTTAAATGTTCTTACTAAAAGAGAAGGAATGCAGTGCTATAATATTTATTAATCAAATAAATTTTAATTTTTTATTGATTATATTTTATATTAATTATCTAAATTTAAAATATTTTATTTTTTATTTTTAATAATAAATTAATTTTTAAGAAATCCTATATTTCTTATTTTTATAATCTATTAGCATTCACCTTAGTAAATACATTAATCTCATTAAAAAAAAAAAAAGTTAAAATTACCTTTTAATATTAGTATATACCTGCCAAATTAAGATTTTATTTATAAGTACTCTAAAAACTTTTTCAATATTTACGCATTTTTCTTTCATTCAATATCAATATCTAAATTATCATCTTTGTGTAAAATATTTTCAAGTAAGCCTCCATCATTGTGTTGGTAGCAGGTGGATCATAGCTATGTTATATGCAAATTGATAAAGAATGTAGAAGGTGAAGCTCATCCAGTTGAGGTGCAGTTTGGAGACAAAAGAAAAAGGCACGGTCAATCTGCCACCACCAGCGGCGTTCAAATTGATGTTAACGCTCCTCATTCATATGTAAGTACTCAACACATTGAATATATAGTTACATGAAACTTTGTAGGTGGTGCTGACCAATTGTCATATATATTTTAAGAGGAACACTAAAGAGCAAGAGGTCCAATTCATACCAAATGAACTTGGCAGGCGAATGTTGTTGGAAATGTTTGAGGGTAAGTATATGTATAAATATATACGTAGTTTAATTTATTTTGAATTTGTATTTATATTTTAGTATATATTTTATATTGATAGCTGATTTTAATATACACCTAGCATAGTTATTAAATAATTTGGTATACTTATATACATTTTGCTTCAAAGTACATTCTGTTATATTAGGAAAGATGATGAGGATGGTTTAACTCTTTCTGATGGTTTAACTCATCAGAAACCTCATGCAGCTGCAAGGGGGAACAAGAAGAGGTGGAAAAGGCATCTAATTATTGCATAA

>AdNAC29

ATGAATAATAATAAGATAAGCAACATGAGCTCCGTGAGTAGCTCCGATCTCATAGATGCCAAGCTTGAAGAGCATCAGTTGTGTGGAGGATCCAAGCAGTGCCCCGGTTGCGGCCACAAGTTTGAATCCAAACCGGTATGATTCAAAAATAAATAATTAATATTCTCATTATTGCTATAGCTTAAATGAAAATTAAAAGCCTTATGGAAAGAAATTAATTTCTTTTAATTTGGCATTAATGTGTTAAAGTTTTTTATCCACTCTCTCTTTATTGATATCTCTTTAATTTGTTCTCTCCAATCTATCTTTCTTTATTTCTTTCTCTACTCCTTCTTTTGCTTTCTTTATTTTAATTTAAATTTAAATTTTGCTTGAAAATAAACTATTATTATTATTATTATTTCAGGATTGGCTAGGTTTACCAGCAGGAGTGAAGTTTGATCCAACAGATCAAGAACTAATAGAGCATCTTGAAGCCAAAGTGGAGTCAAAGAACATGAAATCACACCCTTTGATAGACGAATTCATTCCCACCATTGAAGGTGAAGATGGGATTTGTTACACCCATCCTGAGAAACTTCCAGGTATGTATATATGTTACATCCATTTATTTATATAATTATTTTAATACTTGAGAAATTTATAACATTTAAACGCCAACATTTCTAAATTATTTTTGTTTAGACTACATCTATAAATAGTAATTATTATTAATCATATATTATTGTATTTACAAAATTAAATCTCCCCTTGTATTAACAAAAAAAAAGGATCAAAATAGAAATTTAAGCAAAAGGGTTTTGTGTTGTTGTTGAAGTTAGTTCAGCTAAGTGAACCGAACTTTGATTTTTTTTTAAAATTATTATTATTATGTTATTAGGAGTAACAAGAGATGGATTAAGTAAACACTTCTTCCATAGGCCTTCAAAGGCATACACAACAGGAACAAGAAAGAGGAGAAAGATTCAAAATGAGTGTGACTTGCAAGGTGGAGAAACGAGGTGGCATAAGACCGGTAAAACAAGACCGGTCATGGTTAACGGAAAACAGAAGGGTTGCAAGAAGATTTTGGTACTCTACACTAACTTCGGCAAGAATCGGAAGCCGGAGAAGACGAATTGGGTCATGCATCAATACCATTTGGGGCAGCATGAGGAGGAGAAAGAAGGAGAGCTTGTTGTGTCCAAGATATTCTACCAAACTCAGCCTAGGCAGTGTAATTGGTCGTCCGATCGGAGCGCCACCACCACCATCGCTACGGCCGAAGGGAGTGGAGAGCCACTACAAAATAGTAGAAGAGATAGCGGAAGTGGAAGTTGTTCTTCTAAGGAAATTAACATAGGTCATAAGGATGAGATGTCTGCTGTGGTTGGAGTTACTAATACTCCAATCACAGGCTTCGCTCATCCCTTGGACATTCATCATCATCTCAAATCGGATCATTTTAGCTTCATCCCATTTAGGAAAAGCTTTGATGAGGTATGTTTTAATATTTATTATGAATTAGGATCAAGTTCTTATAAGTAATTGTGTTGAAATTTGATGGATTTTAGCCACCTTTATGAATTAATATCAGTGATGAAGATGTTTCACTTATAATGATAAGCTTTATTGTTGTTGATTGATCCACCAAACATATAATAAGTCACTGGATTTTTTCAACAATTAAAGAGAAAAAAAACCAAAATAGTGAAAAACATGAAAGGGGGTTAAGGTAGGTGCTGACATTAAACTATATATAAAAGCATATATATATATTTTATAGCATATAAATAAATATTTTGCTTGGGTAGAACGCATGAAATTGTGTTGTATGGACATGGCGTGTGATCATAGATGAGTGTCATTCCCAACCACTAATTGTTTTTCTTCACCTACCACTGAAAATTCTCTAACTAATTAATTAACAGAAAATTAGAAGCATATATAGACTCATGCAAAATTCATATAATTAATGAAAACAAAAAAGTGATATGTATGTATTTGAGTAGTGACGGTTTAATTAACAGTTGCAAATGATATATCATATGGTTGGGAGGATTTTCATCAGGTTGGAATAGGAGAGGCTTCAACAGCAAGAGAAGTTCAAGCATCAGGGTCATGTGATGAAGTAGTACATGAACATGTAAATCATCATCATCAACAACAACAACAACATCATCATCATCATCAAATTNNNNNNNNNNNNNNACATCCAATCTCTACCTTGATATCTCCACCACCACTTCACCACACATCCATCATCTTAGACGATAATTCCTACCAAGTCTCCAGAATAATGCTCCAAAATGAACATTTCCAGGTTAGTAATTAATTTTATCTAGTAATACTCATTTTTTACAAATTTATTATATACATTAAATCATATTAAAAGAAATCAAAGCATGGAGTTAATTAGCAGTATTTCATCAACAACTATTTCTTTTTCCCTCTCCCTCTCTCTCTCTTATTATCTATCTATGTACAATTAGTAAAAAAATTTTCCAACATATTAAAACTATATTTAATTTATTATAAAAAAATACTATGTATATAAAAAATTAGTTATTTGTATATCAATATTTGTATATTATTTTATTTATTTTTAATGTGTATTTTATATTATAATATGTATTTTGTATAAATAATTAATTTAAAAAATGATTTTTTATTATCCCTTAGCATGATGCTTTTAAGTTTAAAATAGTCATGGAAAGATTTTTATGCAAATAAAAAGCAATTATATCTTAAAATAAATCTATAATAATAATACTAGTGAAAAAAAAAAGAGTAAAAAATCAAGGTGCAAGATGCTAAGTAGAAAGAGAGGAGTTCATAATCTATAATGTATAAAAATATATAATAGTAGTAAATAATAATTAAAAAGGAGAGAAGAAAAAGTTATTTAATTTATGGTATATTTGTGGTGCAATAAATTAAAAAAGAATGAGTTAAGATGTGATGAGAGAGTAGTAGTATAGGAAGCAATTAAGTTCTGCATGGCATTGTGCATGAGTCAAGAGCCATCAAGTATAGTTGAAATTAAAGGGTTTATTTTTTGAGTGCTTGCTAAAACACAAGCATTGAAAACTGTCTTTTTCACTTTTTGTATGTTATTACTAATAATTTATTATTGTGTGTGTGTGTTGAACATTATGATTGAGAAGTGTGTTTTGAAATCTTGATTGTTATATTGACCCTTTGAAAATGGAACTATGATATGGATACACATGGCATGGCAGCAACAACAACAACAACATCAACAACATCATCATAAAATTGGAGCAAGGTCTGCGTCTGGTTTGGAGGAACTCATCATGGGGTGCACTTCTTCTGATATCAAAGAGGTATATATAAATATGTAACCCTTTTTTGACTCCGAATTCTATGAATTGAACTACTTTGTGTGTCATAATGCCAATGCAATTCTAACAAAAAGAGAAGACAGATAGTGTGTGTGTGTGTTTATATATGCTAAAGCTAAACCAATCAAATTTTCTCCCTAACATTTTGTTTTCTAAATATTATAATGAGAATAATGCTAGAGCTTAATTTTTTTCCAGTATTAATTAATTTCTCAAAATGATTTTATTTATATATCTTAAATAATAAATTCTTAACTATAAATCTTAAATTTTAATATTTCCAAAAATAAAAAATTTTAAAATGAAAAAATATTGATTAATTAAAAATTAATTTTTTATATTTTTTATTATAATGAATGGAGTTTTTCTAAGATTTTCGGTTAAGAATTCAAGGAATGCAATATTTTATGCTTCTATAATTTTCAATGCAAGCAATTTAATTTTTATAATTTTTTAAAACCTAATTTTAAAGATTAGCTAACTAATAACTATATTGGGAAAGTTTCTTTAAACTAAAGGTTTTGTAAGCTCTTAGGGTCTTTCACTAAGCCTATTATTGTAATTATAAATAAGTTAAATTTGGTTAAAGGTAATAATGAGTTAAAATTCTCCTTTCTTTTTAAAATCCATTCTTTGGACTTAGCTTGCTAATTAGCAAAATATAATACCCTTGACTTTTGACTATGAATTGGAAAAGATGATATTATTCCAGACAAAATCCATTTTCTTGTCTTGTGGTTGATAAAAAAGAGAACATGATATTAAATTATTATTATTTTATTTTCATTATACATAAAACTAAACCCTAACCCTAATCTTTTGATTTTTGTATGTAGGAGTCATCCATCACAAACCCACAAGAAGCTGAATGGTTGAAATACTCTTCCTATTGGCCAGACCCTGACAACCCGGATCATCATGGGTAG

>AiNAC2

ATGAATAATAATAAGATAAGCAACTTGAGCTCCGTGAGTAGCTCCGATCTCATAGATGCCAAGCTTGAAGAGCATCAGTGGTGTGGAGGATCCAAGCAGTGCCCCGGTTGCGGCCACAAGTTTGAATCCAAACCGGTATGATTCAAAAATAAAAATATTAGATAATTAATATTCTCATTATGGAAAGCAATTAATTTCTTTGTTTTTTTTTTTAAACTCACTTATTATATCATCACTAAATTAAAGTTTTAATTTAGCATTAATGTGTTAAAGTTTTTTATCCACTCTCTCTTTATTGATATCTCTTTAATTTGTTCTCTCCAATCTATCTTTCTTTATTTCTTTCTCTACTCCTTCTTTTGCTTTCTTTTTATTTTTTTATTTTTTGTTTTAATTTAAATTTAAATTTTGCTTGAAAATAAACTATTATTATTATTATTACAGGATTGGCTAGGTTTACCAGCAGGAGTGAAGTTTGATCCAACAGATCAAGAACTAATAGAGCATCTTGAAGCCAAAGTGGAGTCAAAGAACATGAAATCACACCCTTTGATAGATGAATTCATTCCCACCATTGAAGGTGAAGATGGGATTTGTTACACCCATCCTGAGAAACTTCCAGGTATGTATATATGTTACATACATTTATTTATATAATTATTTTAATACTTGAGAACTNNNNNNNNNNNNNNNNNNNNNNNNNNNNNNNNNNNNNNNNNNNNNNNNNNNNNNNNNNNNNNNNNNNNNNNNNNNNNNNNNNNNNNNNNNNNNNNNNNNNNNNNNNNNNNNNNNNNNNNNNNNNNNNNNNNNNNNNNNNNNNNNNNNNNNNNNNNNNNNNNNNNNNNNNNNNNNNNNNNNNNNNNNNNNNNNNNNNNNNNNNNNNNNNNNNNNNNNNNNNNNNNNNNNNNNNNNNNNNNNNNNNNNNNNNNNNNNNNNNNNNNNNNNNNNNNNNNNNNNNNNNNNNNNNNNNNNNNNNNNNNNNNNNNNNNNNNNNNNNNNNNNNNNNNNNNNNNNNNNNNNNNNNNNNNNNNNNNNNNNNNNNNNNNNNNNNNNNNNNNNNNNNNNNNNNTTATAACATTTAAATGCCAACATTTCTAAATTATTTTTGTTTAGTAATTATTATTAATCATATATTATTGTATTTACAAAATTAAATCTCTCCTTGTATTAACAAAAAAAAAAAGGATCAAAATAGAAATTTAAGCAAAAGGGTTTGTGTTGTTGTTGAAGTTAGTTCAGCTAAGTGAACCTAACTTTGATTTTTTTTTTTTCAAATTATTATTATGTTATTAGGAGTAACAAGAGATGGATTAAGTAAACACTTCTTCCATAGGCCTTCAAAGGCATACACAACAGGAACAAGAAAGAGGAGAAAGATTCAAAATGAGTGTGACTTGCAAGGTGGAGAAACAAGGTGGCATAAGACCGGTAAAACAAGACCGGTCATGGTTAACGGAAAACAGAAGGGTTGCAAGAAGATTTTGGTACTCTACACTAACTTCGGCAAGAATCGGAAGCCGGAGAAGACGAATTGGGTGATGCATCAATACCATTTGGGGCAACATGAGGAGGAGAAAGAAGGAGAGCTTGTTGTGTCTAAGATATTCTACCAAACTCAGCCTAGGCAGTGTAATTGGTCGTCCGATCGGAGCGCCACCACCACCATCGCAACGGCCGAAGGGAGTGGAGAGCCATTACAAAATAGTAGAAGGGATAGTGGGAGTGGAAGTTGTTCTTCTAAGGAAATTAACATCGGTCATAGGGATGAGATGTCTGCTGTGGTTGGAGTTAATAATACTCCAATCACGAGCTTCACTCATCCCTTGGACATTCATCATCACCTCAAATCGGATCATTTTAGCTTCATTCCATTTAGGAAAAACTTTGATGAGGTATGTTTTAATATTTATTATGAATTAGGATCAAGTTCTTATAAGTAATTGTTGTTTACATAACAAGTTGAAATTTGATGGATTTTAGCCACCTTTATGAATTAATATCAGTGATGAAGATGTTTCACTTATAATGATAAGCTTTATTGTTGTTGATTGATCCACCAAACATATAACAAGTCACTGGATTTTTTCAACAATTAAAAAAAAAAAAAAAACCCAAAATAGTGAAAAACATGAAAGCATATATAGCATATAAATATATATATATTGCTTGGGTAGAACGCATGAAATTGTGGTGTATGGACATGGCGTGTGATCATAGATGAGTGTCATTCCCAACCACTAATTGTTTTTCTTCACCTACCACTGAAAATTCTCTAACTAATTAATTAACAGAAAATTAGAACCATATATAGACTCATGCAAAAGTCATATAATTAATGAAAACAAAAAATTGATATGTATGTATTTGAGTAGTGACGGTTTAATTAACAGTTGCAAATGATATATCATATGGTTGGGAGGATTTTCATCAGGTTGGAATAGGAGAGGCTTCAACAGCAAGAGAAGTTCAAGCATCAGGGTCATGTGATGAAGTAGTACATGAACATGTAAATCATCATCATCAGCAACAACAACAACATCATCATCATCAACATCATCAAATTANNNNNNNNNNNNNNNNNNNNNNNNNNNNNNNNNNNNNNNNNNNNNNNNNNNNNNNNNNNNNNNNNNNNNNNNTAGTCATGGAAAGATTTTTATGCAAATAAAAAGCAATTATATCTTAAAATAAATCTATAATAATAATACTAGTGAAAAGAAAGAGTAAAAAATCAAGGTGCAAGATGAGTTCATAATCTATAATGTATAACAATATATAATAATAGTAAATAATAATTAAAAAGGAGAGAAGAAAGAGTTATTTAGGGTATATTTGTGGTGCAATAAATAAAAAGAATGAGTTAAGATGTGATGAGAGAGTAGTAGTAGTATAGGAAGCAATTAAGTTCTGCATGGCATTGTGCATGAGTCAAGAGTAATCAAGTATAGTTGAAATTAAAGGGTTTATTTTTTGAGTGCTTGCTAAATAAAACACAAGCATTGAAAACTGTCTTTTTCACTTTTTGTATGTTATTACTAATAATTTATTATTGTGTGTGTGTGTGTTGAACATTATGATTGAGAAGTGTGTTTTGAAATCTTGATTGTTATATTGACCCTTTGAAAATGGAACTATGATATGGATACACATGGCATGGCAGCAACAACAACAACAACAACATCAACACCATCATCATAAAATTGGAGCAAGGTCTGCGTCTGGTTTGGAGGAACTCATCATGGGGTGCACTTCTTCTGATATCAAAGAGGTATATATAATATATATATATGTAACCCTTTTTTGACTCCGAATTCTATGAATTGAGCTACTTTGTGTGTTTATATATGCTAAGCCAATCAAATTTTCTCCCTAACATTTTGTTTTCTAAATATTATAATGAGAATAATGCTAGAGCTTAATTTTTTTTCAATATTAATTAATTTCTTAGAATTATTTACTTATATATCTTAAATAATAAATTCTTCACTAAAAATCTTAAATTTTAATATTTCAAAAAATAAAAAATTTTAAAATTAAAAAATATTGATTAATTAAAAATTATTTTTTTTAATTTTTTTTATTATAATGATTGGAGTTTTTCTAAGATTTTTGGTTAAGAATTCAAGGAATGCAATATTTTACGCTTCTATAATTTTCAATGCAAGCAATTCAATTTCTATAAGTTTTTAAAACCTATTTTTAAAGATTAGTTAACTAATAACTATATTGAGAAAGTTTCTTTAAAATAAAGGTTTTGTAAGCTCTTAGGGTCTTTTATTAAGCCTATTATTGTAATTATAAATAAGTTAAATTTGGTTAAAGGTAATAATGAGTTAAAATTCTCCTTTCTTTTTAAAATCCATTCTTTGGACTTAGCTTGATAATTAGCAAAATATACCCTTGACTTTTGACTATGAGTTGGAAAAGATGATATTATTCCAGACAAAATCCATTTTCTTGTCTTGTGGTTGATAAAAAAGAGAATATGATATTAAATTATTATTTTAGAGGAGTGTTAGGAGTTAGCAGAATTTGTGATGTTTAGCCATCAATTAACCATCATCAATATTTTTAATAGTGTGAAATAAAATCTAATGGTATATGATTAATCATTTTTTCTTTAATAATTAAGTACTAACCAAATTTCAACAAAAGTACTGGTCCTTAAACTTTCTCTAAAACTAAACCCTAACCCTAATCTTTTGATTTTTGTATGTAGGAGTCATCCATCACAAACACACAAGAAGCTGAATGGTTGAAATACTCTTCCTATTGGCCAGACCCTGACAACCCGGATCATCATGGGTAG

>AdNAC21

GATCCCTCTTTTGAAGTACATACTCAACCCTTCTATCTCTCACACACACAAACACATACAACTTTCTATCTTTCTCTTTTTAGTTTTTGTTCTATTTGTTTTATAGAACAAGAGAAAAACAAAGGAAGGAAAAGAAGATCTCCATAAGTGGAATGTTGTGTGAGAAGGATAATAAGGAGGGAAATTAAACAATAAAGAGAAATGAGTGAGTCCAATGAACATGAAAACAATCATGGCAACATCATAGTGGAGGGAAGAAAAGACAGTTTAATTAGAACTTGTCCAACATGTGGTCATCACATCAAATGCCAAGATCAGGTTCTTAGTTCTAAATACCCCTTTAATTTGTTATTAGTTAGCACATAAAAAAATTTTCATTAGTTTGATAATTGTTCATATCATAAATATATATGAGAGAGAGTTACTTTATTATTATAATAACAATAATAATATATTATATTTTTTTTGGCAACAAGTTATTACACAAATTGATTATCTATATATTTTATTTGGTTTATTTATTATATTTTGTGGGATTTTCTTTATTCATGAAATATAATTCATAATATTATTATTGTTGTTGTATTAATAATATAAATAAGTATAGGGTGGTGGAATTCATGACTTACCTGGACTTCCTGCTGGAGTGAAGTTTGATCCAACAGATCAAGAGATTCTTGAACATTTGGAAGCAAAAGTGCGATCTGATATTCACAAGCTTCACCCTTTAATTGATGAGTTCATCCCAACTCTTGAAGGAGAGAATGGAATCTGCTATACTCATCCAGAGAACTTGCCAGGTCATGCATGCATATATTATTACTACTGGATAAGTCTTTATAATATTGAGAAATATAATGATACTTTTATTATATATAACTATAGTTTTGGATAATTTGCGAGAACTTATTTAATTTGTTACATATATATTCCCATGACATTGGAGATTTGGAGTAGTATTATTACGTCCTTTTATAATAAATTAAAGTCATATTTTAAATAAAGGAAAAGTCTAAGGGCCAGCATGTAACCAAGAAAGGTGAGTCATTGGATAAAATTTTACACCAATCTCACACCATCAAATTATCATTAATGGCTAGTTGATGGCTACCAATCACAAATGTTGCTGGCCCTAGCATTGCTCTTAAATAAAAGCGGTGATTTTAATTTGATGTTTAATTTCACGAGCCTAAAAAAAAATTCTAACAACTAATAATAAGATTTCCCTATCAATTAGTATATTTAATTTCAAGTGAACGTTTTGAAACTTGAAGGTGATTAGATATCTTATAATAAATGTGAAATCTTTGTAATGAATTTACCAATAATGTTGACACTTATGCTTGATTTGATTTGACAAAATTTTTGATGAGGTATTGTGAAAAGTACAAATTGTTTGTTTTATATATGTTAAATAAAAAAAATTCATGTATTTGTATTTAGCAATAGATCTAAAAAAATTTTATAGTGAGAGTAAAATATATATAATATAATAATATTTTGGTTTAAATATTTTACTTGTTCTTATATTTTACTAATTTTTTAGTTAAATTCTTATAGTCTAAAAATTTATAATTAACTTATTATATTAGGTTAAAATTTTTTTAATTAAATCTTTAGTAGTTGTCATCTTTTAGAAAATATACACTAATTCTAAAATATTTTATAATTCATCCTACATAAAATATAAAAACACATGTTAAAAAATTAAAAAGAATCTAATAATTTAAATAATAACATACACTTTAGAATTCAATTATTCTAAGTTTTATATTAACAAAAATTAAATAATTTTTCATTGTATACGTTACTAAACAATTATTTAAAAATATATCTCCAATACCATTACAAAATCAACTCTTTATAATATTCATAACTTAAAAAGTATTTTCAATTGATGTAGTTCTACCACAAAATTAAATCTAACTTAAAAAAAAGGAAATAGTCGTGGATAAAAAATATTTTTTTTAAGTCTTAACCAATTTCTATAATAAAAAATACCAATTTTATTTAAGTTTTAATTTTATTTAAGTTTGAGAATTGATTATGAAAATGCATATATAAAATAAAATTCTCCAATTGATTATCAAGTGCTAAAAATTGAGTAAAAAAACTTTTAATGAGGCCAAATTAATTAATTTAATAAAAAAATTATTATTGTTATATATTACTCAATAAATGTTTAAATTGTAGTGGGGGAGGAGTTACCGGCCCACACCCTAATACATAAATCCGTCTCAATTTATATTTGTGGTTATAATTTTGGTTTAATTAATCTATTGGCTTCTATATTTTCGAAAAATTTTTAATTAGGTCTTTATGCTTTTTTCTTTTTAATTGAGTCTCTGCACCAATTTTTTTTTTAATTAGGTTCTTACACTCTTTTTTTATTTAGATCCCTATACCAATTTTTTTTAGTTGGGTCCCTATAAAATTAAGCCAATTACTACTAAAAAAGACTTAATTGAAAAAAAAATTAGTGTTGGGACTCAATTAAAAAAAAAATAAAGGGATCTAATTGAAAATTTCACGAAACTATAGAAACCAATAGAGTAATTAAACCTATAATTTTTAAAAGGTAAAAATATCAAAAAAAATTAAAAAAATATTTTTAAAGTTAACATGTATTTATCAAAACTTAAAATTTGATATAACCTTATACATTAATAAATATTTAAATTTACTTTTATATTTATATTTATTATAGTCTTTTTAAATTTTAAGAATTATTTTATCAAACACAATTGTTATTATTTGTATTTATTAAAATTTATTTTTAATTTAATTTATGAAACATATATATGTTATAATTTAAAAAAAATCATCTTTCAAAAATTAGTTTAGATAAGATATTTTCAAAAACTAAAACTTTATCAAAAACCAATACGTAGCTAAAGTTTTGCAATCATTAAGGCATGATTTTATATATTATATTATTTTCTAGTGCATTCACCTCAAGTACAATTTAAATCATAATTATTTATTAAATATAATTGTCATAAAATTATTTTTCTTTTTAATTTTAAAGTGGTAAAAAAGGTACATAAATAATTAGGTCTTTAATACGATTATAAAAAAAACTTCTTTTAGAGTTATTTAAATTTTGAATAATTTTTTTTTATTTGTCTTATATTATTTTTTCTCTTATGAAGAATAATAAGAATCGAACTCTAAATGTTTTGGTTATAAAAATTTTGATACCATCTCATAAAATTATTTTTTATAAAAATTTAAACTAATAAACAAATACATAAATAATTATATCTTAAAAAAAAATTCCATTTCCAGGTGTCTAAATAATCTTAGAAATGTAATTTTAAAATCATAGTTTGAACTTTAAAGTGACTTTTTTTTAAGACTTCAGTAACATAATTAAAACGAAGATAAATTACATGTTTTTTCGTATATTTACGAAAACAGTGAATTATCTTTTTTGTTACCCTAACTAGCATTACCTAAGTTAAAATGAAATTTATAATTTTATGTAAATTATTAATATTTTATGATCGAATTTTGGCACAGGAGTAAGCAAGGATGGGTTGATCCGGCACTTCTTCCACCGGCCGTCGAAAGCATACACAACCGGAACAAGGAAGAGGAGGAAGGTGAATTCGGACGAAGAGGGAAATGAAACCCGTTGGCACAAAACAGGGAAGACCAGACCAGTCTATATTAGGGGGAAGCTGAAAGGATACAAGAAAATCCTTGTTCTCTACACAAACTATGGTGGGAAGCAAAGGAAGCCAGAGAAAACCAATTGGGTGATGCACCAATACCACCTTGGCAATGATGAAGAGGAGAAAGAAGGAGAGTTGGTTGTTTCCAAAGTGTTCTACCAAACACATCCTAGACAATGTTCTTCACTCTTGATCAATAACAACAAAGACTCTTCAACAGCCGCACTTGTCAAGGGTAATAATAATAACGGGTTTGTTGAGTATTACCATTCAAATTTCATATCATTTGATCAAGGGGAACACCAACATAGATCTAGTGGGGCTCAAGTCGTCATTTCACATTTTCCTGTCCATGAAGGTGCTCCTAATTATCATTCTTTGAATCGAAAGGAGTAGTGTAGAAAAAATGGTAAATTACCATTTTATCTATGAAAGAATTAGCTTGCTGATAAAATGGTTTTTTGGAGATTGATAATTATTTTATGGTACTAGATTTTTTTTTGTTTTGTTTAACAAAATTACTTAAAGCGATCAAAATTTGTTGTTGAAATGGTAATTTACTTGGGGAAATAGACATATAAGGTGAAAGAGTTTGTGAAAGGAGCTGAGGAGAGGAAAATCATAAATAGGAGTTTTCTATATAATACTGAGGCATTATTGTAACATATTATAAAAAATAGTTAAGAGGGCACGTAAGTGA

>AiNAC25

GATCCCTCTTTTGAAGTACATACTCAACCCTTCTATCTCTCACACACACACACACACAAACACAACTTTCTATCTTTCTCTTTTTAGTTTTTGTTCTATTTGTTTTATAGAACAAGAGAAAAACAAAGGAAGGAAAAGAAGATATCCATAAGTGGAATATGAATGTGTGAGAAGGATAATAAACAATAAGGAGAAATGACTGAGTGCAATGAACATGAAAACAATCATGGCAACATCATAGTGGAGGGAAGAAAAGACAGTTTAATTAGAACTTGTCCAACATGTGGTCATCACATCAAATGCCAAGATCAGGTTCTTAGTTCTAAATACCCCTTTAATTTGTTATTAGTTAGCACATATAATTAAGTCCACTTTATTTGTTATTTTATAGTTTAACTTATTAGAATCTGATTTATTTGTTGTAAAAAGTTTTTCATTAGTTTGATAATTGTTCATATCATAAATATATATGATTTTGTGCTTTCTTTTCAGTTTTCAAGAAGCAGTTTCCTTCCCCCAAAAACAAAACTAAATAGAGAGAATGAGATAGAAGAATAGAAGATAGGATTGGTTGCAATGAGAGAGATAAGAATAATAAAAAAAATTAAAAAGGAATAAAAACATTGCTTTGATTTCAAGTTTAGGTATGCTTTGTATATATTACTTGGTATATGAAAACTTGTAGATGAATAAATGTTGGTTTTTATTCTTTTTAATTTTTGGGACGTGATTTTGTCCATTTTACACTGATTAAAGGTACATAGCTCTGCTCTGATGATGAATTTGATGAAATAAAGAAATGATGTTAAACCTAGAAAGAAACTATTAAAATATGGATTTAGAGCTAGAGTGGGATTGGGGCAACCGAATGTATAAGCTCAAAGAGTAAGAATTTAGGTCCAATGTCACCCTAGAGTGTTGATAAAGTTAAAAATATAAATACCAAGAAGACTCTATTAATAAGGGGGAAAATTGAATTGAAGAGAACAATTGTTTTCATAGCCCAACATAATTCTATTACTTCACGTAGACTTCTCATGCATAGCAAGAAACCCCTAGCGTTCCCACTCAATAGATATTTTCATATTGAGTGGAAATATTTGATTTGTAATGAAAGTATTGGTTTAATCTAATTTGTCTCTACTAATAATAATGGATTAAGTCGTTATTATTATTATTATTATTATTAAGCCAACAACTACGATTGTTACTTAGTATAACATATCCTTTTATAAATTTTTCAAATTGCATTTAATAAAAAACAATGCTAAAGAATTAATATTACGGTAATCAATTTTAATCAATACTATAATATAGTTTGCCGAACAAGTTCAATATAAAACCTACTAAAAATTAACCTTTTTTCAATTTGAATTTCGCATGTATTTTTTAATTGAGATTCGGATATTCTTGTTTTGAAGAGTTTCGAATGTTTTTATCTTGGGAAAATTATTTTAAAGGACCGTTAGAAAGATTTAATTATTAAAAAAGACCTTTAATATCAAAATTATTAAAGAATGACCAAATCTTTTTATCTTTCTAAATTACTGATTATTTATTTATATTCATATATTCATACAACACATTAATTTTTACCCCCTTCGATGAATGACCCATTTAGCTACAGTTTTGATAGGATTTTTGAAAGGAAATTAAAAAAGAATTTCAAATGAGTTATTTGCACTTTGAATGCAGTAAATATATTTAGCATATTAGCATAATTGCTAATTCAATTAGCTTGCTATTGTGAACCGAAAGCTGGATGCAATTTTTCTAAATTTGACTGTCAAACTGAGTAACATAAAGAGTTTGTGTAAAATATGGATATTAAAAAGATTTAAAAAGATTTAATTATTAAAAAGGATCTTTAATACTAAAATTATTAAGAAGGACTAAATCTTTTTACATAATATTTAAGAAGAAGAACCAAATCTTTTTATAAGGAGATAAATATATCCTTAACTATTTTTCTTTAACCATATTTATTTCAGTAGTCTTGACTACAGAAACTGAAAATATGTTTACCATTTTAATACATTCTCCAGTTTCGGGAGTGTTTGTAAATGAATATCTTGCAACTTGATGTTATGTATAACATCTTGTTTTCTATTATCATCATCAGCTTCTTTAAATATAACTTTGATTGACTCACAATTCGTAACCCTCAAACAATTTAAATTTGACAATCTTTTAACCGTATTATGAGAAATTATATGGACCAATTCATCACATTTCTCAATGATCAGAGTGTGTAGTTTGCCAAAAGAATTTCCATGAGCTTCAGAATTTCATATTTTGCACAAATTCTTTATGTTACTCAGTTTGACAGTCAAATTTAAAAAAATTGCATCCTGCTTTTAGTTCACAATAGCAAGCTAATTGAATTAGCAATTATGCTAATATGCTAAATATATTTACTGCATTCAAAGTGCAAATAACTCATTTGAAATTCTTTTTTAATTTCCTTTCAAAAATCCTATCAAAACTGTAGCTAAATGGGTCATTCATCGAAGGGGGTAAAAATTAATGTGTTGTATGAATATATGAATATAAATAAATAATCAGTAATTTAGAAAGATAAAAAGATTTGGTCATTCTTTAATAATTTTGATATTAAAGGTCTTTTTTAATAATTAAATCTTTCTAACGGTCCTTTAAAATAATTTTCCCAAGATAAAAACATTCGAAACTCTTCAAAACAAGAATATCCGAATCTCAATTAAAAAATACATGCGAAATTCAAATTGAAAAAAGGTTAATTTTTAGTAGGTTTTATATTGAACTTGTTCGGCAAACTATATTATAGTATTGATTAAAATTGATTACCGTAATATTAATTCTTTAGCATTGTTTTTTATTAAATGCAATTTGAAAAATTTATAAAAGGATATGTTATACTAAGTAACAATCGTAGTTGTTGGCTTAATAATAATAATAATAATAATAACGACTTAATCCATTATTATTAGTAGAGACAAATTAGATTAAACCAATACTTTCATTACAAATCAAATATTTCCACTCAATATGAAAATATCTATTGAGTGGGAACGCTAGGGGTTTCTTGCTATGCATGAGAAGTCTACGTGAAGTAATAGAATTATGTTGGGCTATGAAAACAATTGTTCTCTTCAATTCAATTTTCCCCCTTATTAATAGAGTCTTCTTGGTATTTATATTTTTAACTTTATCAACACTCTAGGGTGACATTGGACCTAAATTCTTACTCTTTGAGCTTATACATTCGGTTGCCCCAATCCCACTCTAGCTCTAAATCCATATTTTAATAGTTTCTTTCTAGGTTTAACATCATTTCTTTATTTCATCAAATTCATCATCAGAGCAGAGCTATGTACCTTTAATCAGTGTAAAATGGACAAAATCACGTCCCAAAAATTAAAAAGAATAAAAACCAACATTTATTCATCTACAAGTTTTCATATACCAAGTAATATATACAAAGCATACCTAAACTTGAAATCAAAGCAATGTTTTTATTCCTTTTTAATTTTTTTTATTATTCTTATCTCTCTCATTGCAACCAATCCTATCTTCTATTCTTCTATCTCATTCTCTCTATTTAGTTTTGTTTTTGGGGGAAGGAAACTGCTTCTTGAAAACTGAAAAGAAAGCACAAAAAAGAGAAAGTGAGGGAGAATTTAATTCTTTAATTCCTTCCCACACCTCAACAAGTGCTAGCATGAGGGGTGGTGCAATCTTTAGAAGAGACAAGTTTATAAACTTTTTCTCCCTTTAATTTTATATTTAATTAATAATTATTACTACTTACTTTATTATTAGATCCCTCTTTTGAAGTACATACTCAACCCTTCTATCTCTCACACACACACACACACAAACACAACTTTCTATCTTTCTCTTTTTAGTTTTTGTTCTATTTGTTTTATAGAACAAGAGAAAAACAAAGGAAGGAAAAGAAGATATCCATAAGTGGAATATGAATGTGTGAGAAGGATAATAAACAATAAGGAGAAATGACTGAGTGCAATGAACATGAAAACAATCATGGCAACATCATAGTGGAGGGAAGAAAAGACAGTTTAATTAGAACTTGTCCAACATGTGGTCATCACATCAAATGCCAAGATCAGGTTCTAAATACTAAATACCCCTTTAATTTGTTATTAGTTAGCACATATAATTAAGTCCACTTTATTTGTTATTTTATAGTTTAACTTATTAATTTATTTGTTATTTGTTGTAAAAAGTTTTTCATTAGTTTGATAATTGTTCATATCATAAATATATATGAGAGAGAGTTATTTTATTATTATAATAACAATAATAATATATTAGAATTTTTTTTGGCAAGTTGTTGTATTAATAATATAAATAAGTATAGGGTGGTGGACTTCATGACTTACCTGGACTTCCAGCTGGAGTGAAGTTTGATCCAACAGATCAAGAGATTCTTGAACATTTGGAAGCAAAAGTGCGATCTGATATTCACAAGCTTCACCCTTTAATTGATGAGTTCATCCCAACTCTTGAAGGAGAGAATGGAATCTGCTATACTCATCCAGAGAACTTGCCAGGTCATCAATACAATTTATACATGCATGCATATTATTACTATCTTTATAATATTGAGAAATATAATGATACTTTTATTATATTATATAATTATAGTTTTGGATAATTTGCGAGAACTTATTTAATTTGTTACATATATATAATATAGGAAGACTCTTAAGTGTGCCGGAAACATCGTTATTTCAATTGATTTAACCGTTGATCTGAATTATAAAAATATATTAATTAAAATTAACGGTTAAAACAATTAGAATATCGGTGTTTTCGATACACTTGAAATTTTTTCTATAAATAGTCCCATGACATTGGAGATTTGGAGTAGTATTATTACGTCCTTTTATAATAAATTAAAATCATATTTTAAATAAAAGCGGTGATTGTAATTTGATGTTTAATTTCACGAGCCTAAAAAAAATTCTAACAACTAATAAGATTTCCCTATCAATTAGTATATTTAATTTCAAGTGAACGTTTTGAAACTTGAAAGTGATTAGATATCTTATAATAAATCTGAAATCTTTGTAATGAATTTACCAATAATGTTGACACTTATGCTTGATTTGATTTGACAAAATTTTTTGAAGAGGTATTGTGAAAAGTACAAATTGTTTGTTTTGTATATGTTAAATAAAAAAAATTCATGTATTTGTATTTAGCAATAGATCTAAAAAATTTTGACAGCAAGAGTAAAATATATATAATATAATAATATTTTGGTTTAAATATTTTATTTGTTCTTNNNNNNNNNNNNNNNNNNNNNNNNNNNTATTATTTTTTCTCTTACGAAGAATAATAAGAATTGAACTCTAGATCTTTTGGTCATAAAAATTTTGATATCATATCATAAAATTACTTCTTTTAAAAATTTAAACTAATAAAAAAATACATAAATAATTATATCTTAAAAAAAATAATTTCCATTTCCAGGTGTCTAAATAATCTCAGAAATGTAATGTTAAAATCATAGTTTGAACTTTAAAGTGACTTTTCTTTAAGACTTCAGTAACATAATTAAAACGAAGATAAAGTAGCTGGTCTATTAAGCAGCATTTTGTGGCAGATGTAACCGTTTATGGTTTACACATACAACATATTAAACGTAGGTTTTGAAAATTATTTGCATCCCTTCAAACTTAAACCAACCATTCAACCAATGCTATATATACATGTGATCGTGCATGGGATCTAGTATGTATGAAGCTGATATCTTAACATACAATAATCTTACATATGTGAAGTTGATAATAAGTTGTATGCTGCTTGCTTTTATTTTAAAGATTAATTTTGATGCATTATTAGATGTTAGCATAAAATAGTTTAATTGAAGTAATAATAAAGTGACATGTTTTGTTCATGCCACAAACAAATAATTAATTTATTACATTTAACAAGAAAAATGTAAAACACTTAGAGAAAGTACACGGTAATAACAATTGAGGAATGCTAGAGCAAACAATTTTTGTGATTTGTAGCCATCAAATAGCTATCAATGATGGTTTTAATGGTGTAAGATTGGTGTGGTGTGAGATTTCATCCAATGGCTCATTTTTCTTTGCTAGTTACATGCTGGTCAGAATTTAACAAAGTTGTTGGTCCCCTAGACTTTTCCATAACAATTATCACGTATATTTACCAAAAGAGGAAGTATAGGAAGCCAATGGAATATTTCTACAATGTGTACAATGAAGGTTTAGGGATGTCCGATTCAGTATTAGAGATATAACCATTAGTGTTACATTTTTCTATCAGCTTAAATTTTTGGGATGAGTGGTTTCATGACATAGTATTAGAGCTCTAAATTCGAAAGATCAAGAGTTCGATCTTTGGTAAACCCCAAAATTAACTTAAGCTTTTGAAATGAGTGATTTTTTAACTCATAGCCCTATTGTACGGATGATATTCTTTTTTTAACTCATAACCCCGTTGTATTGTACAATAATAGTATATCATTAGTTAAAACAACAATGTGTGAAAGATAAAAAAACAGCCAAAATAATTAAAATTTATTTTGTTAATTGCTACTACAATTAATAAATATTAAATAAGATAAATTTGAGTTATTTTTTTCGTCACCCTAACTAACATTACCCTAGTTAAAATGAAATTTATAATTTTATGTGGATTATTAATACTTTATGATCGAATTTTGGTACAGGAGTAAGCAAGGATGGGTTGATCCGGCACTTCTTCCACCGGCCGTCGAAAGCATACACAACCGGAACAAGGAAGAGGAGGAAGGTGAACTCGGACGAAGAGGGAAACGAAACCCGTTGGCACAAAACAGGCAAGACCAGACCAGTCTATATTAGGGGGAAGCTGAAAGGATACAAGAAAATCCTTGTTCTCTACACAAACTATGGTGGGAAGCAAAGGAAGCCAGAGAAAACCAATTGGGTGATGCACCAATACCACCTTGGCAATGATGAAGAGGAGAAAGAAGGGGAGTTGGTTGTTTCCAAAGTGTTCTACCAAACACATCCTAGACAATGTTCTTCACTCTTGGTCAATAACAACAAAGACTCTTCAACAACACTTGTCAAGGGTAATAATAATAATGGGTTTGTTGAGTATTACCATTCAAATTTCATATCATTTGATCAAGGGGAACACCAACATAGATCTAGTGGGGCTCAAGTCGTCATTTCACATTTTCCTCTCCATGAAGCTGCTCCTAATTATCATTCTTTGAATCAAAAGGAGTAGTGTAGAAAAAATAGTAAATTACCATTTTATCTATGAAAGAATTAGCTTGCTGATAAAATGGTTTTCTGGAGATTGATAATTATTTTATGGTACTCGATTTTTTTTTTTTGTTTAACAAAATTACTTAAAGCAATCATAATTTGTTGTTGTTGAAATGGTAATTTATTTGGGGAAATAGACATATAAGGTGAAAGAGTTTGTGAAAAGAGCTGAGGAGAGGAAAATCATAAATAGGAGTTTTTTTTATATAATATTGAGGCATTATTGTAACATATTATGAAAAGTAGTTAAGAGGGCACATAAGTGA

>AdNAC72

GGTTTCTCCATTTTTGGCAAGTTGTTTCTTGAATTGCAATAATGGCTAGGTGAGCTATAGATTTTTAACAGAATGTGGAGATTTTTTATTTTGTGCATCTGATAAAGCTTTCACTACCTTCTTCTTCTTGTGATTTGGAGACCCTTCATAGCGTTGATGAATAAAAATTCAACTTTTTCAACTGGGGTTTTTTTTTTAGGGTGGTGTCATTGGAATGTTCCCCTGTTTCTCAGCATTGTTTTTGTTTTCTCTTCTTTTCAGTTTCCTAACAAATATATATATTAAAATATAAAACTATATGGTTGAGTTTTGATGATGAATGGATCATGGATGATAGCATGTTTTGGCTTTTATTTTCCCCTAAATAATAATCATGGGAAGATGGATGATGGATTATGTTACATATTCCCATTACAATGAGTTTACCTTAGCTTATTTCTTGTGTTTTTGAAGATAATGTGATTGTTAAACCTAAATTTTAAGCAAATTTGGACATGCATGCACTGAATTGGGGATTAGAAAGGATATATAGATGTACTAACTCTTATTTTCTCGCGCGAAAGAAACATTTGAGAGGTACTAAATGATGATCATAGCTCCTGGATGATTGAAATGTGAAGTAAAAAGCCCAGATGGTTTAGCTGGATTTCCAAATATACTACATTGTTTATGAACAAGAATTAATTCATAAATAAAGAACTTCAGTTCATCATCCTTGAGTCTGAAATCTGTCCTTTGTGTTCCTCAAAATGAAGTTAGTAGAACAAATTTTCTGCAGAGGATACAGTTGGAGATCACTTACAATGCACAAGCCTTTCTTAACTATTAACCTAATTTTAATGATCAATGGCATCACATAAAAACTAGTTTTCATGTCTAAATTTAACTTCATAGGTTTGAAAGATCAAGGCTTCTATCATTGATGTCCTTAAACATGATCAGTGCAATCATTAGAAATAGTAGATTTTTCACTTGACCTATAGTTATATCTTAGAAACTATAGAACACAAAGCATGCTAATTCTAATTTCTACGAATCCTTCTTATGATATTATGGTTCATTTCTTCATATTTAATACAGTTTCACAACCTCTTTATGTATCAGTCTAGTTACTTCTTTTTCTTCTTTTTTATTTCTCAAAACCCAAATATGATTTCGACAATCTGCGAATTCGTATTTGAATTTGCAGAAGCTGGGTTATTGACATTGGAGGACTTGCAAAGAAAGTGAAGAATAATACATTGCCATTAGCTGATCAAATCAATGACTGTGGAGCATATTGTGAATGTCCAATATGTCATTATCACATTGATAACATTGATGTATGCTATAATGAATTCAACAAAATCTTCAATCTCTGTTCATAACTTCATGTATAAATTCATGATTACAGATGTTTCATTAAGAACATTCCTTTGCCTTTTGTAGGTTTCTCCTGAGTGGCCAGGCTTTCCGGCCGGCGTGAAGTTTGATCCTTCCGACATAGAACTGTTAGAACATTTGGCAGCAAAATGTTGTGTTGGGAACAAAGTGCCTCATGCCTTTATCCAAGATTTCATCCCAACACTAGAAGGAGACCAAGGAATATGCTACACACATCCAGAAAATCTTCCAGGTGAGGTTTCTCTAGTTACCTTCTCTTTCAATGTTAGCTAATAACTTAAAATGAGGGTTCCTTATGGAACCTTATTCCTTAAAGATATTGCTCTTTTTTTGTGAACATTTATACTTGACCAAGAAACTAGAAAAGGATGAAAAATACAATACATTCTAGCATGTTGGGATCTATAATGTAGTGGTTTAATCAAATATCAACAGGCATATAACAAACTTGCTTGGATTGTGGATCTTACTTTAGCATGAAATTCCAAGTTGAGTGTTAGGATTTGAGATAGTAAGAGCAAGCAAGCTGAATAACAAAAAGTGACACAAAATAAATTAACGTGGTTCAGCTTATGCCTATGTGGCAAAGACGACAAACAAATATGAAGGAAATACAAAATATGTGATACCAAAGTAATGACCAAATACTTAAACTAAACCAAGTCTCAAATACACCAAAAATACTTTATAGTACTATCAAATAGTATAAAGTATAAACTCTCTATCTCAAGAGAAGTAAACAAGAACAAAATACTCCTCACGATACAAAGAGAGACATGAATAATTAATACGAATCTTCACTAACAACCAAAAGAGTAACCAAATAGACTTGGTACACACTACGTGGAGAAACAACACTCAAAATGAGACCTTACAAATTTTCACTAACAACACTAATATTAACTCATAATTCAAATTCCATACATCTGAATCACCAAACTCTTAGTCTTTTATATTAGCAACTAATGTGAACAGGATGGCGAAGTATATCTACCATGTATGCATATGAACACACAAGTTTCAACTTGTGACATGTTTGAAAGTTGAACAATTTCAATTTGATCTTTGTTCATTTCACCATGCATGTTCAAACTCCTTTTGAATAGTAGCCCCAATTTCAATTTGATCTTTGTTCCTTTTGATCACCTTGCATCCAAATTGATTTTGATTTGGCAGTAGTTAAGTAATACAATCCGATGTGTAAATTATAGGATTCGAATCATGAGTGTAATAGTTATCTCAGCAAGGCAAAGTGTGATAAACCAATGTCCTAAGACTCAGAGTAGTTCATGCACATAGCAGAAACAAGAGAAGGAAAGAGAAGAACAATGAATATGAAATTCATGTAGCATTTGGAATCAATGAATTGTGTATAGTTCTCAAAGTTGTGAACCATATATAATTAACTACCTCATAACAATGACATTTCAACATTCTCAAAAAAAATTAGACCTGCAATGTTTTCAGGTGCTAAGAAAGATGGGGCCAGTGTTCATTTCTTTCACAAAACAACTAATGCATATGCTACCGGACAACGAAAGCGTCGAAAGATTAATCATCAACTAGGCCTAAGTGAGGAGCATGTTCGCTGGCATAAGACCGGTAAGACCAAAGCTGTAACAGAAAATGGAGTACACAAGGGCTTCAAGAAGATCATGGTTCTTTACGTAAGGCCTAAGAGAGGAGCAAAGCCGAATAAATCGAAATGGGTGATGCATCAGTACCATCTAGGGAGTGAAGAAGGTGAGAAGGAAGGTGAATATGTGGTTTCAAAGATTTTCTATCAGCAGCAGAAGAAAACTAAGAAGAATAAGCTGAATCCATTGGTGGCTGAAGATTCTGTCATGGCATTACAAGCAAGTCCAAGAACACCGAATCCAAATCCACCAAAACGGCCTCGGACAGGAAAATCTGTTGATTTTGAAGAAACTGACCTTATGCTATTCACTCAGGTAGGTAGCTATTGAAGGCTGATCATCCTTTTAGTGAATTCAAATGAAAAAGAGAAATGAAATACTCCCTCAATTCTATGAACATTTCTTGATTGTACTAATAGGCCTAGAACTAAGTTATGCTAAATTTTTTTACTATATATGCGTTTTGATTTGTTTTATTCGGAATTTTCATGTGATGGACTAGGGTGGAAAGCCTACTATTCATGGAGAATCACTTGAACCACCACCATCTGAAGTTCATGGTGATGAAAATAATGGAGGCTTTAACAACACTGCATTGTTATCTGTTGAAACACAACCTGTCAACTCTGACTTTATTGGATTGGATGATATCTTACTATGCAAGGAACAGACATTAGATTCTTCTTCTGCTCACCTAAATGATTCGGGTTTGAAGTCCAACAATCTGAAAGGCTTTGCTTGCAATGCAAATGGAAATGCAGGTGAATTGTTTGGGGATGTGAATGATTGTTATGGAATTTCAGTGCTGGATAACCTGGGACTGGACAGTCCTCCCGATTTTGATCTTTCTGTAAGCACTCGATCACCCTTGTCGTCGTAG

>AdNAC31

TTTAATTAAAGCTACCTTTAGTCAGAGTAGAAAGGACAAAAACTTGAATTTAGTCTTCTACAAGCTTTCATACAAATGATATATACAAAGCAAATAAACCACAAAACTTGAGATCAAAGCAACCTTATTATGCATTATTTCTCATGTGCAACTAACCCTATCTATCTCATCTTTCTAAATAGTTAGTAGTTTCATTCCTCCTCCATTTAGAATTTTCAGGGAAGGAGGGAGTACAATATTATTCACCTCATGCAAAAGAAAGAAAGCACCAAAAAAAGTGTGAGGAGAATTTAATTCCTTCCCAACCCTTAACAAGAACTTGCATGAGGGGAGCAACCTTTAGAAGAGACAACTTCTTCTTTATTATTTCCCCTTCTTATATACTGTACTCATCACTATTACTTTAAATATATTCATCTCTTAACTCAATCTACACATAAAATCACTCTATCTCCTTTGCTTGATGCATATATCTCTTTTTTTTCTTTATTTCTTTCTTATAGCAGCGTGAAGAAGGAAAAAGAAGATATGTATCCTGAAATGGAATGAGAGAGGAGAAGGGAAACATAGGGTAACACATGCAAGAGGAAATAATGACTCAGTGCAACAGTAATAATGATTACCCTGAAAACAATCATAGCACCATTGTGGAGAGGAACAAAGATAGCTTAATTAGTAGAACTTGTCCATCATGTGGTCATCATATCAAATGCCAACAAGACCACCAGGTTATTAATTCTTTTACATTTTTGTACTTCAATTTTGCATGTACAATTGGGGTGCACATGATTATTATTTATTATGATTTATGATATGATAATTAGGGTGCTGGAATTCACGATTTACCTGGGTTACCAGCTGGAGTGAAGTTTGATCCAACAGATCAGGAAATCCTAGAACATTTGGAAGCCAAGGTGAGGTCTGATATTCACAAGCTTCACCCCTTAATTGATGAGTTTATCCCTACTCTTGAAGGCGAGAATGGAATCTGCTGCACCCATCCAGAAAAGTTGCCAGGTCTTCATCAATTTATTTAAAGTAAAAGCATTTCATAATTCATGTATCAAGAGAAGTCTTTTCTGATCTTAATTATTTCCTTCCTTAAAATCTTTTTATCGCATATATATCTTTACATTACCTCTGATTCATATATGCATGTATGAATCAAGATATAGTTATTGGTCTAATAAATTGGAGAACTGCAGGAGTAGGCAAAGATGGGTTGATCCGTCACTTCTTTCACCGGCCATCGAAAGCATACACAACAGGAACAAGGAAAAGAAGAAAGGTTCACACTGATGCTGATGGCAGTGAAACAAGGTGGCACAAAACAGGTAAAACTAGACCAGTCTACATCAGTGGCAAGTTGAAAGGTTACAAGAAAATCCTTGTTCTTTACACCAACTACAAGAAGCAAAGGAAGCCTGAGAAAACAAACTGGGTCATGCACCAGTACCACCTTGGCAACAATGAAGAGGAGAAGGAAGGTGAGTTAGTTGTGTCTAAGGTTTTCTACCAGACACAACCTAGACAATGTGCAGGTTCACTACTCATCAAAGATTCATCATCATTCCCTGCTAAACTAAAGGATCAGGGTGGTGTTCATCATCATGAAGTGACTAATAATCATAAGAACAATGGGTTTGTGGAATACTACAATGCATCCTTTATAAGTTTTGCTCAAGGGGAACAACAACATAGGTCAAACAATCCCACATTGATTTCCCATTTTCCTGCTCATGATGGGGCTCCTTTCATTCCTTGATATATTTTATGTTAAACATAACTTTTATTACTTGTACATTTTTATTATTGATTAGAAATTCTTTGTGGGACCAAGTTTAGGGCTCACATTTTTATCAATAATAAAAATGTAAGTAAGATAAAGTTATGTAAAATTACATATGAGAAAATCTATTCTTAAAGGATGAACAAAATACTAAATAGGACTTATTACAGTTATAAGCTAAGGCATTACAATTGTATTCTGCAATGAAGAAATAAGTTAATGACCAATTACAGTTAGGAGGGGAAAGAATAAGGAGAAGCTACTTCATTGTAAAGCTGGTGGTCAGATACTCCTATTTTGGGAAAAATTGGGCTAAGAAACTAAAAGAGAAGGATGTAACTG

>AdNAC19

CTTTAATTAACCTTGTGATATTATGACATGTTCCAAGTACCGCCGCCTTTATAATTTCCAGCAATCTATCCATCCATTATTATAATTGTGAAAAATAATCAACCCGAATCTTTATCTCTCTACTCAGCATATATATAGTGACAAAATAAATGGATTATGGAAGTGTAGTTATTACTATACCAAACTCAACAATTCATACATACACCACCAATTCTATGATGATGATGACTGCAGATTATGAAAGCGTGAAGCAGCTTCCTCCTGGGTTTTTGTTCTCTCCAACGGATGAAGAACTTGTCCTTCACTTTCTCTATGCCAAGGCTTCTCTTTTGCCATGCCATCCCAACATCATCCCTGATCTTGATGTCTCTCTCGCTCATCCTTCCCAACTCAACGGTATCTTTAACCACCTACTCAATTTCTTATAACCACTTTTCATATAACAAGCAAGTTAACACCTTTTACTTTAAACCAAAATAAATTATTTTAAAATTGAGCAGTGTAAATATTTGGCAGGTAAAGCGTTGTCAAGCGGAAATCAATACTATTTCTTCAGCAAAGTGAAGGAAAAAAGAATAACAGAAAATGGGTATTGGAAGGAAATAGGTGAAAGTGAAGCAATATTGTCATCAACGTTTGAGAAGAAAGTAGGGACAAAGAAGAACCTTGTATTCCACATAGGAGAAGCTCCACACGGCATTGAAACCAGTTGGGTCATGCAAGAATATCATATTTGCCCATCCTCTAACATTATTTCTACAACTAGAGCCAGAAGAAAACACGTAAGTCACTACCCACATCCACTATATATAGGTTTAACTTCTTTTTAGATGTTATTTTTTTTTTTTCATTTGTGAAAAATTTACGAACATCTAATTAAATTAAAACAGGATCATCAAATTTGGAGCAAATGGGTTTTGTGCAAAGTGTATGAAAAGAAGGGGTCCGTACGAGGTGTAAACTACTGTAGCGACGATGATGACAGTGGGACAGAGCTATCTTGGCTTGACGAAATTTATCTCTCGTTGGATGATGATCTGGAAGAAATTAGCGTCTCCATTTTAGATTGAATATGTATCAATTACCTGAATTTTAGGTTTAATATATATGTAACGGTGCCGCTATATATGTTGATACTACTGTATATTGAATTGGGAGTTCAATGAAAATGATTATACCTTCACTAAATATTTTAAATGGAAGACAATTGAACAATAAATTCTAAAATTTGGTTCAATAAAATAAAAATACATTGCATTTTATTTTATCCACCTAAATAAAATACTTTA

>AdNAC1

ATGGATGTGGCTAAGTTGTACATGAACAACGACTACTCCGAAGAACATGAACATGAATATGAACATGATGAAGATGATGATGAGATGATGAAAGAGGAGAAAGAAGTTGTGCTTCCTGGGTTTAGATTCCACCCAACAGATGAAGAGCTTGTTGGGTTTTATCTTCGGAGGAAGGTTGAGAAGAAGCCTCTTAAGATTGAACTTATCAAACATGTTGATATCTACAAATATGATCCATGGGATCTTCCAAGTAAGATATTCAATTCTTCTTCTCCTAATTTCAATTCTTCTTCTAATTAATTGTCACATGCATGTTTTAGAGTTTATCCATAGTAGATGATCAATTTCTTCAGTTTGATTATAACTATTCGTATACTTTTTCTTATCTGAGAGGTAATTTTCATAACACTTTTATGATAAAAAAAATTTACAGTTGAATACTTATCATTACAAGAGATACGCTGAATAGCAACAGTTTTTTTAAGAATTTGAGACGATTTTCAAAAAGTTGAATACTTATTACTGCATAGTTAATTACCTTCTCTTATTACATGCATAAAAAATTATTTGTACAAGTATATATGAACCTCATGTATGTTATATAGGTGCAATTTTGATTTTTTTTTTGTCTATGTTATTATATAATTTTGTTTATATAATAATTAATTAACGAATTTATTGTTAATATGTGAAGAAGTTGGTTCATCAATGGGGGAGAAGGAATGGTATTTCTTTTGCATAAGAGGGAGAAAGTACAGGAACAGCATAAGGCCTAATAGGGTTACAGGATCAGGGTTTTGGAAAGCCACAGGGATTGATAAACCTATATACAGTGCTAATTATAATAATAATTATAATAATAATAGTAATAATAATAGTAAGGAGCATGGTGATCATCATGAATGCATTGGACTGAAGAAATCATTGGTTTATTACCGGGGAAGTGCTGGAAAAGGCACCAAAACTGATTGGATGATGCATGAGTTTCGCCTCCCACCCAATAATAATAATGGAGCAAAATTATTAAGCAATAATCAAGAAGCTAATAATGCTACCAAGGATCTTCATGAAGCTGTAAGTCAATTATTATACCCTTTTTTTCTTTTTGCTACATCACTAATTTTGCCTTTCAAACTTTAAACTTTGACAATTTAAGTAACTAGCTTCTAGATAAAAAAAAAAAATTAAATAAGTCTATGTGTTACTTGAAATATATATTTCATTTGACAAGTTCTCTCAATATTTTCCTAATTTTTGTCAAATGAAATACATTTTTGAGTATCACAAGTTTATTTAGTTTTTTTTTTCAAGAATTTAATTACTAATAGGTGGTTTAATTTATTACTATTTTTTATTTGCATATACTATATATATTATTTTAATTAGAGATGGTGTCTCTTTAGGGATTTAAATTCTTTTGTGTTATTTGAAAAATGTGGTGAGGATTATATTATTTAAATAAGAATGCAAACTAGACCATCTAGAAGTAGAAGAATAAAGACAAATTCTAGAGGGAATATATTCAAAATAAATGATTATTACAACAGATGCTGAGGTTCTTTGCTAGAAATCTTGTTCATACTCACCATGACCATAAATATCAATGTTGTCTCTATTCAAAAGTCAGAACCACGTTATTTAGACCCATGTATTTAGGAGAATGCATGATAGTGAATAAATTAAGCAACCAATTTATCAACAAATTAACGAGATAATATGTTACATATTCACACATGCATATATAATAAGTATACAAATACATAGTGTCAGCTAAGTTATATCAATAATTAATCAATTAAAGTGTATTTTATTATATTTATTTGAACTAACGTGGTTCATAGCATATACTTAATTATTTTTGTATTATTTTAATAATTGTATTGTATGTGTATTATTTTAATAAATTGTATTTTAATATTCACATATGTTTATAATTGTATTATTTTAATAAATTGTATTTTAATATTGCCAAGTGAAACTTGCGGTTGATGACACATTAATTAGGTCATTGGTCAATCAGAAGAGTCGTCTGGATTTTGACTGAGAAAAAATCAAATTAATTCTTTTTCTAATTTCTTGTTAGTATTAATATTTTGAATATATTTTATTATTTTGACTGAGAAAAAATAATTAAGCCGCATTAATTGCTTTATTTGTCTCTCTTTAATAAGTCAATTTCTCTCTTTGTTATACTAGTTCTTAATCAACCTTGATTAAATCAAGTAAATTAATCAGTATATTTTAGAGATAAGATGTAAATAAAGTACTAAATCTACTTTTATTATTCCTTTGCTGTATTTATCAATTAAGGTTATTTTTCTTGTGCAAAAAGTCAAAGTCAAGATGATATCTTAAGAGGTTGCATTAAATGATGGATTGAGTTTCTTGCATTACCCCCACCAAACTTGTATTATTAATGGAGTCTGGTCAATTGGCACTTAATGGAACTTGGTATACTTTTACAGGTCTTGATGTTCCATTCTTCATATTGTTACATCTTTGGTCCCTTCATCTCTGACCAAGTCAACTTTCTTCAATTCCTTTGAAGAAAAATAACAAAAAACTTTACAAAATAGTCCATATAAAGTTATAGATATTTATTTTTCTTGATAAAAACTTTATAAAACTAGTTATCTGATTAAGAATTCATGTGGATCATAGATTATATTTACAAAAATTTTATATGTTTTTTATTTGGATGGTTATTATTACAGGAAGTGTGGACACTATGCAGAATATTCAAAAGGATTCCAACATACAAAAAGTACACACCAAATTTGAAAGATTCATCAACATCACCACTCATGAACAAACCCATCAATAACATTAACCACCAAACTGATTCCTCAGTAACTTCCATATCATGCAGCTTAGAATCTGACAACAACAATAGCAAGCAATTCTTGACTTTCACTAACACTATGGGGATTCAACAATGTGAAAGGAAGCCTCTTGTTATTGGACATGTTGATGAAAGGAACAACAACTTTTTCTTAGACCATTCATCAATACATCATCAACAAGCTCCAACAACAATTACTACTACTGCTTTGTCATCATCATCATACTCATCATGGAACCAGCACCATGTTGTGGAGGATTACTTGTTTGCAAATGAGAATTGGGATGATCTTAGATCTGTGGTTGAGTTTGCCACTGACCCCAATAATTCCAAGGTTTATCTATGATTGTAATTAAATATATGTTTTGAGAGGGTATTATTATTAGGGTATTTAGCTTCTTAAA

>AiNAC15

GAGAAAGCATAGAGAATAGTTTTCACATGTATGTGTTGTTTTACTTGAGACTTTAAGTGTTTTTAGAGTCATGGATGTGGCTAAGTTGTACATGAACAACGACTACTCCGAAGAACATGAACATGAACATGAAGATGAAGATCATGAGATGATGAAAGAGGAGAAAGAAGTTGTGCTTCCTGGGTTTAGATTCCACCCAACAGATGAAGAGCTTGTTGGGTTTTATCTTCGGAGGAAGGTTGAGAAGAAGCCTCTTAAGATTGAACTTATCAAACATGTTGATATCTACAAATATGATCCATGGGATCTTCCAAGTAAGATATTCAATTCTTCTTCTAATTAATTGTAACATGCATGTTTTAGGGTTTAAATTTCTTGGGTTTGATTATAACTATTCATATATTTTTTTTTAGAGAGATAATTTCGTAATACTTTTATGATCGAAAAATTTAAAGTTGAATACTTATTAAAGATTACATAGTTAATTACCTTATCTTATTACATGCATAAAAAATTATTTGTACAAGTATATATGAACCTCATGTATGTTATATAGGTGCAATTTTGATTTTTTTTTTTTCTATGTTATTATACAATTTTGTTTATATAATAATTAATTAACGAATTTATTGTTAATATGTGAAGAAGTTGGTTCATCAATGGGGGAGAAGGAATGGTATTTCTTTTGCATAAGAGGGAGAAAGTACAGGAACAGCATAAGGCCTAATAGGGTTACAGGATCAGGGTTTTGGAAAGCCACAGGGAATTGGTTTATTACCGGGGAAGTGCTGGAAAAGGCACCAAAACTGATTGGATGATGCATGAGTTTCGTCTCCCACCCAATAATAATAATGGAGCAAAATTATTAAGCAATAATCAAGAAGCTAATAATGCTACCAAGGATCTTCATGAAGCTGTAAGTCAATTATTATACCCTTTTTTTCTTTTGGCTACATCACTAATTTTGCCTTTCAAACTTTATACTTTGACAATTTAAGTAACTAGCTTCTAGATAAAAAAAAAATTAAATAAGTCTATGTGATACTTGAAATATATATATTTCATTTGAGATTTAATTACTCTGCTAGTCCTATAGTTTCGTAAAATTTTTAATTAGGTCCTTATACTTTTTTTTCTTTTAATTCAGTCCTTATACCAAATTTTTTTTTTAATTGAGTCTCTATATCTTTTTTTCTTTTATTTAGGTTCCTGTACCAAATTTTTTTTTTAGTTTGCTCCTTATAAAATTAAGCCAATTACTACTAAGAGGGACTTAATTGACAAAAAAAATTTGGTACAAGGACCAATTAAAAAGAAAAAAAGTATAGGGATCTAATTAAAAATTTTACGAAACTATAGAAACCAATAGAGTAATTAAACCTTCATTTGACAAGTTCTCTCAATATTTTCCTAATTTTTGTCAAATGAAATACATTTTTTAGTATCACAAGTTTATTTAGTTTTTTTTTCCAAGAATTTAATTACTATATATTTCTTCATTATTTTTTATTTGCATATACTATATATATTATTTTAATTAGAGATGGTGTATGTCTCTTTAGGGATTTAAATTCTTTTGTCTTGTTTGAAAAATGTGGTGAGGATTATATTATTTAAATAAGAATGCAAACTAGACCATCTAGAAGTAGAAGAATAAAGACAAATTCTAGAGGGAATATATTCAAAATAAATGATTATTACAACAGATGCTGAGGTTCTTTGCTAGAAATCTTGTTCATACTCATCACCATGACCATAAATATCAATGTTGTCTCTATTCAAAAGTCAGAACCACGTTATTTAGACCCATGTATTGAGGAGAATGCATGATAGTGAATAAATTAAGCAACCAATTTATCAACAAATTAACGAGATAATATGTTACATATTCACACATGCATATATAAGTATACAAATATACATAGTGTCAGCTAAGTTATATTTGTAATAATTAACCAATTAAAGTGTATTTTATTATATTTATTTGAACTAACGTGATTCATAGCATATACTTAATTAGTTCTGTATTATTTTAATAAATTGTATTTTAATATTCACATATGCTTATAATTGTATTATTTTAATAAATTGTATTTTAATATTTCCAAGTGAAACTTGCGGTTGATGACACATTAATTAGGTCATTGGTCAATCAGAAGAGTCGTCTGGATTTTGACTGAGAAAAAATAATCAAGTTAATTCTTTTTTTTTCTGAATATATTTTATTATTTTGACTGAGAAAAAATAATTAAGCCGCATTAATTGCTTTATTTGTCTCTCTTTAATAAGTCAATTTCTCTCTTTGTTATACTAGTTCTTAATCAGTATATTTTAGAGATAAGATGTAAATAAAGTACTAAATCTACTTTTATTTTTATTTACTATTTAAAAATATTTTTCTTGTGCAAAAAGTCAAAGTCAAGATGACATCTTAAGAGGTTGCATTAAATTATGGATTGAGTTTCTTGCATTACCCCACCAAACTTGTATTATTAATGGAGTCTGGTCAATTGGCACTTAATGGAACTTGGTATACTTTTACAGGTCTTGATGTTCCATTCTTCATATTGTTACATCTTTGGTCCCTTCATCTCTGACCAAGTCAACTTTTTGAAAGAAAAATAACAAAATGATCAATCCATCTATCTATCTATCTATTTTTCTATCTATACATATATAATTTAAAAAAAATTTCAAATGTATCGGAGAACACTGGTGTTCCAGTTGTTTTGGTCGTTGATTTCAATTAATATATATTATATATATTTTTTATAATTCAGATCAACGGTTAAAACAACTGGAACACCGGTGTTTTCGACATACTTAAAACTCTTCCTATAATTTATAAAATAGTCCATTAAAGTTATAGATATTTATTTTTTCTTAATAAAAATTTTACAAAACTAGTTATCTGATTAAGAATTCATGTAGATCATAGATTATATTTACAAAAATTTTATAGGCTTTTTATTTGGATGGTTATTACAGGAAGTGTGGACACTATGCAGAATATTCAAAAGGATTCCAACATACAAAAAGTACACACCAAATTTGAAAGATTCATCAACATCACCACTGATGAACAAACCCATCAATAACATTAACCACCAAACTGATTCCTCAGTAACTTCCATATCATGCAGCTTAGAATCTGACAACAACAATAGCAAGCCATTCTTGACTTTCACTAACACTATGACTATGGGCATTCAACAATGTGAAAGGAAGCCTCTTGTTATTGGACATGTTGATGAAAGGAACAACAACTTTTTCTTAGACCATTCATCAATACATCATCAACAAGCTCCAACAACAATTACTACTACTGCTTTGTCATCATCATCATACTCATCATGGAACCAGCACCATCTTGTGGAGGATTACTTGTTTGCAAATGAGAATTGGGATGATCTTAGATCTGTGGTTGAGTTTGCCACTGACCCTAATAATTCCAAGGTTTATCTATGATTGTAATTAAAGAGTTTAATATATATTTTGAGAGGGTATTATTATTAGGGTATTTAGCTTCTTAAA

>AdNAC70

TCATGAGAAGTGCACTACACAAAATTGAATTAAAGAGGTGGAGAAGATGGAAAACATGCCACCAGGTTATCGTTTCTACCCTACGGAAGAGGAGCTGATTTCATTCTATCTACGGAACAAGCTTGAAGGAGTGAGGGAGGACATGAATCGGGTTATTCCAGTTCTTGATATATATGAATATAGTCCAAGCGAACTCCCACGTAATTATTAACTACAATCTTAATTTCCTTTTATATAATATATAGTTTTGCAAAGATTTTAACATATATATTTATATTGTGTTCAGAAATATCGGGAGAGGCGAGTGTTAGAGACAGCGAGCAGTGGTTCTTTTTCATTCCGCGGCAAGAAAGCGAAGCGCGTGGAGGGAGGCCGAAGAGGCTCACAACAACTGGGTACTGGAAAGCCACTGGCTCCCNNNNNNNNNNNNNNNNNNNNNNNNNNNNNNNNNNNNNNNNNNNNNNNNNNNNNNNNNNNNNNNNNNATCGGAATGAAGAGAACCATGGTTTTCTACTGCGGTAGAGCTCCTAACGGAACTAAAACCGATTGGAAGATGAATGAGTATAAAGCCATTGACACTCATCACCCTTCTTCTTCCTCCAACAACAGGGCGGTTCCTATGGTAACAACCACGTATACTCAACTCTAATCAATCTTATTAGTTTCCAGCATCATGGTTTTTAATTAATATATTTTTATTTTTTTATAATCTAATTAATTGTTAATCTTGTTTGCACACCAAAACTTTAGAGAGTCAAGGTGATGACATCCATTTATTACATCATCCAAGGGATTTTGTTAGAGAACATGAATTCCCAGAATTGAGTTGTAATTATTTAAGTCACAGACCTTATAACGAAGTTTTAGAAAAATGGTTGGAAGTTACAATTTATTCTCTTAATTTATCATAAAAAATATTATTTATCATCAAACTTTGTTACTAAAATTAGTTATTATATATAGAATTCAGTTTATGCCGAGTGTACAAGAAAGCAAAATGTTTGAGGGCATTTGATAGAAGACCACCTCCAAGGAGGGACACGTATCCTCCTAGCCAGAACAACGGTTCATCATCTTTTGATCATCATCATCAACATAATCAAACGGTGGAGAAATCTTCAGGTGCAGGTAGCTCACCGGAGAGTTCGTGCTCCGAAGACCACGGACAGTGTTCTCATCGGACGGAGGATGTGGAAAACGCTAACGAGCCATTTCTCGATTGGGAGCAAATCGATTGGTTCTTAGGATCTTCACTGCCGGAACCATGA

>AiNAC46

TTCCAAAATGTGCAACACCCCATAAAGTAGTGTAAGTACACATCACCATTTTAATATAGTGTATGGAGGATCCACCAACTGGTTTTCGGTTCTATCCTACAGAAGAAGAGCTAGTTGCTTTCTACCTAAACACCCAGCTTCAACTACAAGGCCACGCCAATAACATCAACAGGGTCATTCCAGTGGTTGACATCAATGGCGTTGAGCCCTGGACTCTTCCATGTACGTAACCAAAGTATATGAAACACATATATGTATGCTTCTGTATTTCTGTCTTAAGACAGTTAGTTATTTTGTTGGTTGCAGCACTGGCGGGAGAGCTGTGCAGGGAAGAGAAGGAACAATGGTTCTTCTTTGTGCCTCGCCAAGAGAGGGAAGCCAGAGGGGGGAGGATCAACAGAACCACTGCTTCTGGTTACTGGAAAGCCACCGGATCACCGGNNNNNNNNNNNNNNNNNNNNNNNNNNNNNNNNNNNNNNNNNNNNNNNNNNNNNNNNNNNNNNNNNNNNNNNNNNNNNNNNNNNNNNNNNNNNNNNNNNNNNNNNNNNNNNNNNNNNNNNNNNNNNNNNNNNNNNNNNNNNNNNNNNNNNNNNNNNNNNNNNNNNNNNNNNNNNNNNNNNNNNNNNNNNNNNNNNNNNNNNNNNNNNNNNNNNNNNNNNNNNNNNNNNNNNNNNNNNNNNNNNNNNNNNNNNNNNNNNNNNNNNNNNNNNNNNNNNNNNNNNNNNNNNNNNNAAGAAAACTTTGGTTTTCTACAAAGGAAAAGCTCCCACCGGTCGCAAAACTAAATGGAAGATGCATGAATACCGCGCCATCGTTCAAGCCCCTAACCAATCTCCCACGGCTATTCCTCAGGTATGCAAACATAAGGTCAATGTGAAATTAGTTAAGATGGTAAATATAAGTTATTATCAGAATTTATTATTTTTTGTCATCGTTTAACAGATTGATGATTAAAAATAGTAAATTCTGATTCCTCTTTATAATTTAACTAATATATTTTTCTATGTTTTAAATTTGGTTTTATACGTGTAGTTGAGGCACGAATTCAGCTTGTGTCGCGTGTACGTGATATCCGGAAGTTTCAGAGCATTTGATCGACGGCCACGGGAGGTGGTGGTGCCAAGAGTTCTTCATCATGGTTCTTCTACAACAAGTGCTCAGCAGCATCAAGGAGAATCATCAGCAAGGGTGCAGGCTAATAATAATAATAACGGGTCGAGCTCGTCGGAAACTTCCCTTTCATCAGGTGGTCCTGATTTGCCACCAGATACTGGAGGAGGAGGGTCAAGTAGCAATTGGAATAGTAGTGAGGTTCAAGTTCAAGCTCAAGTTCAAGAACCGCTATGGGAATGGGAACAACTCGATTGGCTATAAGCATGACTAACAATATCAATTCGTCAAGCCATGCATGCATTATTTCATCTCT

>AiNAC24

ATGACGAACCTTCCTCCTGGGTTTTGCTTCTCTCCAACAGATGAAGAACTCATCCTTCACTTTCTTTATTCCAGAATTTCTCTACCATTCCATCCCAGCATCATACCGGACCTTGATCCCTCTCAACTTCATCCATGTCAATTAAATGGTACATACATACATAATAATCTATGCATGCATGCATCCAATTCCACATACACATATATTTGAAATTTAATTAAATATATTTTTTATATAGTTTCTTACATATATGTACTTATATATATATATATATACACACAAACACAGGTAAGGCGTTTTCAAGTGGGAATCAACACTATTTCTTCACCAATAAAGTGAAGGAAAACAGAAGCACAGAAAATGGGTATTGGAAGGAAATAGGTTTGAGCGAACCTATAATCTCAGCTGATGCAAACAAGAAATTGGGAATCAAGAAGTATTTTGTCTTCAATCTTAACGAAGGCACAGAAACCAATTGGGTCATGCAGGAATACCATATTTCCTCATCTATGTTCCACAACCCTATTTCGTGTTATGCAAATGGAACTGCTCATCGAAGATTATTAAAACCTGTAAGCTGTTGGTTTTAATTTCCCTACCATATAGCCCTAGATATTATTTCAATAATAACCTCTCTATTAATTTGTTTGTTATTGTGTAATTGTGCAATTTATTAATGATTATAATATTACAGGATCAAAATCAGAACAATAAATGGGTTTTGTGCAGAGTGTATGAGAAGAACAAGTCACAATCACAACAAGGTGCAACTGCAAACTCCTACTATAGCGACGAGGATGATTGTGGATCAGAACTTTCATATCTAGATGAGGTTTATCTGTCACTTGATGATGATCTTGAAGTCATAAGCCCCCCTAATTAA

>-AdNAC30

ATGCG**GT**AATTGTAACCATTAGATGCG**GT**AATTGTAACCATTAGAAATTCTCAAAGTGAGTCAGTTCAATGGTCATATTCCTATATGACAAGAACAATTTAGATTAAATTATAAAAGATTTATCTCTCAATATTGTGATCACTATCACAATGATAAATCTCTAAATTTAATTAAGGACGTTAATATATTAACATTTTAATATAATAATAATAACAAATTATTTGATACAGGATTGATTTGATTGTGGTCATACTACTTATTCCCAACAAATAATTTAGTGTTCAAATTATTTTTTTTAAATATCTTTCATTGTATATAATTTATAAAATCATATATTTTGTTAATTATTATCTTGATTAATTTAAATTTATTCTCAGTACTATATTTATATAGGAAAACAGTAAATTAGTCAATACACTAATTTCAATTCTGTAAATAACAATTTTTTTTGAAATTATTGTTATTCCATTTCCACCGCTCTATTTCTTTTTCACTGTGATTAAAAGTATTTTTTTATATAAAAATACTTTTTAAATTAAATAATTATTTTTTGAAATTTTTATTGTATCTTTTAGTTTTATATGTGTATAAGTTATACCACAAATTTTCTATATTTGTTTCTCCGACCAACTAATTTGATTAGATAATTCCTAACCGTGTGTTTTGCTTTTCATCTTACCCTACGAAATTACAAAAAAAAAAAAATTGAAACTTCTCCCTTACGCCAAATTTCAAAGTCAAGTCAATGACTAACTTCGTTGCATTACAAATTCGACACAAACCAATCACTCATAACCAACACTCTCTGCTTCGCTTCAGTGCTGCTTCTTCTCTTTACTCTGTTTCTTCATCTTCGTCTTCTTCATCAATCATCATCACCTTCATCACCTTCTTCTTCTTCTTCAACCAAAGCTTTAAACAATTTTCAATTTTCATCTTAAAAAAAAAAAAAAAAAGAAAACAAAAATGGCTAGAAGTGCCACAATTCCATTTCCAATACTTGATTTCATTCCTGTTGGATTC**AG**GTTCAAGCCAACAGATGAAGAGCTTGTGAGCTATTACCTCAATCACAAGCTCCTAAATGACAATTTTCCAATCAATATTATCCCTGACATTGATCTTTGCAAGGTTGAACCTTGGCAAATTCCAGGTAAACCCATAATAAATAAAACCACCCCATCATTTTCATGTATCAAAGTTTGCACCTTTTTCATGTTTGATGATCCTATCCGGTGTTCAAAATTTAAGAAAATGAAGTAATAAATATACAATTATTCAACAACCCCATCATCTTCATGAGACAAAGCTTGTACCTTTTGTTTTTTTGTCACAACCCCATGATCTTAATGTCACAAAGTTTGAAACTTTTCTTCATGTTAGTAACTTAGTATGATGATGTTTGATGATTGATGGATTGGGTTTTGTGTGTCAGCATTATCAAAGATAAAATCGGATGATCCAGAATGGTTCTTCTTTAGTGGACGTGATTACAAGTATGGAAAGAGCAAAAGATCAAACAGGGCAACCAAAGGAGGATATTGGAAAGCCACAGGACAAGATAGATACATAAAGGAAAGAGGAACTACCAATGTAATTGGGAGCAAGAAGACACTGGTTTTCTATAGTGGCCGTGTTCCTAATGGTGTCAAAACCAATTGGGTTATCCATGAGTATCATGCTACTACCTTTGATGATAGCCAGGTACATATGGTTTTTCTTTTTCCAGATAAGTTTTGCCTTGATTAGTTTTCATCTGTTTTGGAAATTGATCTTTGTTTCTATAATAAATACCCAAGTTGATTCTATTTCATATTATACTTTGGTTGTTCAACAAATTTTGAGGTACTCTAGAATTACTTTTTGAAGGGGACTAATTTCTTTTAGACCTAATTGTCTTTGATAGGGACTTATTTGTTCAAAATGCATAATTGAAAATGACCGAGAGAGTAAAAAAATTTGTTGGAGAACAAAGTGGTAGATCTAAATTTCTTTGGACCAATTTAAGTGTTCACTTTTGTTTTTTATCTATATGTTTTGTTGGTATAAATTACTATTATAGTGTATGGTTGTTGTTGCCCCTTCTTGTGATTTTGATGTTTCCTTATCTGATTGGTGATTATGTCATTGGAATTATTCCCATGTTTGTATTTTGTATCATAGTCTATTCATGAATCATGACTCACTTTTTTCCCCCTAATTCATTCTTTTTTGTTGATTGGTGGAACAATTCATCATTGGTTTTCATGAAATAGTGGAATACTATTGAATTGTTGATTTTTGCTATTTAAGCTGTTTCTAGATGAAGTCTCCAATGAACTGAATTTGTGATAGTCTCCATTGTAGTATCAATTTTTCAGTGCTGATTATACTCAACATTCTGCAGAGGAATTTTGTTTTGTGTCGCTTGATGAAGAAAGCCGAGAGAAAATCCGAAGATGGAACTGATGCACAAGCTTGTGATGAGGGGGAACCTAGCACTCATATGGAAGAAGCAGATGAGAGTGTCCCAAGTGTAAGCAGAAGCATCATTTTGATCTCTATTTCATGGTTTATGCATTTTAAAAAAGTTTGGGGATAGTTAATATATCTTTTATGTCTGTTTTAGATGTTTGAGTCGCCAGATGTGGACATGGGTTCGATCTTCCACACACTGCCTCAAGACGGATCATCATCACAGCACTCTCCAGTCAGCATTGAACAGCAAGAATCCTTCCCATTCTCCCCATCTGAAAATTATTACCTTGTAAATGAAGATAGAAGTATGCATATACAATTCGAAACAAACGAAGAGAAGCAAGATGCTGAGAAATTTGCGGATTCGATTTTGGATGGTGGAAATATAGCTATGTTTGAAGAAAGACAGCAGCATCATACTTTCATGAATAATCACCTCCGCTCAGTCCCATCAATGAGGGTATGCTATGAAAGCAGTGACACAGATGCTGAAGTAGTCTCTAGACGGGTAAAGATAAATCCTTAAAAATGATATTGGGGAGTTATATCTCCAATTGTTTATGAGCTATTTGTTGGTTATGTACATTAATTGTTGTTCATCAGTCTATGAAAAATGTTTAGTTAGTTCCTTCTATAGGATGTTAGAGAGATACTTAGCTGAAATGTCTGGTTTTTGTTTGTGTCCTGTTTGATACATGGCAGTTGGCACCTTAACAATACATCAGTGCATAAAGTGGAGAAAGGGGCAGGGAATGGAAATTAAGAAAATACTTCAAAAGCAATATTTTTCCTGGATTCATGATCCTTCTTTGTTTCGAGCCCTTTCAAGAATTGACATATAGCATGCTCGGTTTATGCTACTGATGCAACCCAACCATGGTTTTAGGTTTCATAAGCCTATACCGAGCATGCTATATGAAACCGAAGGAGTATACTTTTTGGTGGCCCAAAACCATAGTTTGGGAAACAACTGAATGGAGATAAGTTTAGCTGTTTTCCAAACCATGGCTTTGAGTTTCCAAAAGCTAAATCAGCCATGGTATCCATTAATTCTCTGCTGAAATTATGTAGCATGTTTAGTTTAGTTTTTGATGACCCTAAGCCATGGTTTTAGAAAAAGTTCATCTTAAATAGCTTTTTCCAAAATCATGGTTCAGGGTTTCTAAAAGCTAATCCAAACATGCTATTGATCCATCGGTCCGCTTCTGAAATTATACAGTATGGCAATATTCCTGATTCTTCAACAATGTGTAATGATCAGGCTGGTTCAAGAGAATACCATGTATCAAAAATGGTTCAATCATCACATGGTGCTGCGTGCACAGATAAAACTAGAAGTATCTCTTCAGAAGACTTTTGGGGATTGGATTCATCTTCATGTGACTCAAATGCAGATAAACCTTTTGAGATCAATTCTATTGAAATTTCTAGTCCTCCATCGGCTCTAAGTAGATCGAAAAATCAATATATTCCGAGGCTATCTCAAACACATAGGAAGGTTTCAAGCAATGCAATTCCCCATCTTGAGGTGAGAACACCGAGCAGCCAGACTAAACGACGAAGAAGCCTATCTTGACTGAGTAGGCTGTGATGCTTCATTTGAAGAAAATTAAATTCTAACATGCCACTGATCATACCATGCTCCTATGTGATGTAATATGCAGGATAAGAAGAAATTGACCACTGTGGAACAATCAAGAAGAGATCAAGAAAAAGCTCAAAAAACTAGTCCAGGAAAGAAGCTAGAAACCAGAAGCTCTGATGTTAATAGAATTGGTAGTTTCATCCACCTAGAGCCGTGTTCATCGAGCGAAAGCCTGACTCCACGAGCGGTATACCTTGTTAATGTAGTTATTGGGATTTTGTTGCTTCTAGCCATTAGTTGGGATGTGCTATCTTGTTAGAGAAGTGTTGCCATGCAAGATTTGTTTTAGGAGTTTCATAGTTATATACATAGGTTAAGTAATAATATAGTTTATATAGATCTTGCACTGATGCAAAGATTACTGCACCTTTCTTATCATAATGAAAATTATGCTTGTCATTAGTAGTAACTTCATATTGTCATTAATTCTATAATTGCAGTCTTTAAGTCACAAAAATCATTTAATGTTAGAACTAGTTGAGTTCTAGACTTCTAGTGAAAAGATTCCTTTTTTCCATTCAATAAATGTTTGATTAGCTTGATTCCCAAAATGTGCTGACTTCAAATTCTCCATTTAGTAGAAACAGACCAAAGTCAATGTTCCAAAAGAAGAAAATTTGATACAAAAGTGCATAAAGTCAAAAGCAGATTACACACCATGTGGCAGTAGCAGTGTTACTTTGAATAGGATCTTTGTTTCATTTTACAATTCTCTCAAGAACCAAAATCATTGAATGTATCTGAGAAAGTTTATGTTCATAGCAGAGTTAATTCAACTAGAAGAGTTTGATTTAACCATAAGGTCAAGGTTGAACTGCAATTGCAGAATCAATGATAACAAAAAAGAACTACTAGATGCAGGTTTAGAAATTGTTACATAAATTTGGATATTGGATTCAACTTTTAGCAGATATTCTATGATATTTTTTTTTTTCAAATTGTAATAAAGAAATGCTTCTCTCTACTTTCATATGGCACATGGAACAAACTAGTTTTATACTTATAGTTAAAATATAAATTTAATTATTCTTTTAGTTCATGTACTTT

>AiNAC18

ATGCGTACTCTACACACAGAGGTGGCTAAGCTCAATGCAAATGAATGGTACTTCTTCAGCTTCCGTGACCGCAAATACGCCACCGGGTTTCGCACCAATCGCGCCACGACATCTGGCTATTGGAAAGCGACCGGCAAGGATCGTACGGTTCTCGATCCCCTCACCCGCGAGGTCGTAGGGATGCGGAAGACTTTGGTGTTCTACAAGAATAGAGCCCCAAATGGCATCAAAACTGGTTGGATCATGCATGAGTTTCGCTTGGAGACCCCACACATGCCACCTAAGGTTAATTCATTTTTCTCGCATCTCAAATTTTAAATTTTTCAAAAGTTTTTAACGATGTCGTTTAATTACATCTATTTTTTTAAATGATTATTTATGTAGTCAAAATAAAATTAATTATTTTTATTGATATAACAATATGTGATTAGATGTATATGTAAATCTATTTTATATAGACAGTATATTAAAATTAAATTTAAAAAATATTTATAAATATTTTCAAAGGTGCATATAGATATCATGATTAATTGATTATGATATGGTTTACACGCTTACTTAATAATAGTAATGGCCACTACTATATTGGGAGTGTACGGTACATGATAAATGGGGAATGAGATTAGGGTATAGTGGAGAGAAGGATCTTAATTAGTTTCAATATGTTCCCACGTAAGAGATATATGTTAAAACTTATGATTGTGATTTCATTTATATCTTAATGAAACCTTTAGATGAAATGACTAATATCCGATGATAAGATTTTTTTTTAAAGAAACATATTAGGCACTTAAGTTAGGCATATATTTAACTAATTTGTTTGCTAATACAAATTTGTTTTTAGGAATTAAGTGTTTACATACTAATTAATAATATGAAAAATATAATAAATAATTGCATTGTCAATCACTAAAATTATAAAAAGTGTGTTAAATCAATATAAATAAGTACCTATTATTATATATATATAATAACTATACATTCTGATTTCTTATTTTCTTGTAATTTATCGATTCAAAATGTAAAACTCTACAACATTCTCAACGACTAATAAAACATGATCTCTTATTTTTAATAATAAACGGGTTTATGTATAACACCATAAATATGGAAAGAACTTGACCTGTATTTCATCTTAAAGTGACAACCTTGTAGATAATTGAAAACAACTTTCATTTCTGGTTGGAAAGTTGAAAACTTAATTAGCCTATGTCATGTAAAAGATTAGGTAGAATCTAATGTCTTTTCTCCATGTGAACGTTTGAGTTAGGATCATATTTGGCTTCAGCATCGATCATTTAGATTATTGAGATAAAAAGGGAACCAGCATGCGTCTGTGACAACTTATATATATTAATATAATAATGTTACGGTGACTATAATATTTTAAAAAGTATTATTGAAATTAAAGTAATATATTATTAATTATTAAATAATAATTTTTAAAAAGTATATTTAAAAAAATTACTTAAGTTTNNNNNNNNNNNNNNNNNNNNNNNNNNNNNNNNNNNNNNNNNNNNNNNNNNNNNNNNNNNNNNNNNNNNNNNNNNNNNNNNNNNNNNNNNNNNNNNNNNNNNNNNNNNNTTCATATTAGAATATATATATGCGCCAATGAAATTTGTTACAGCTATGCATGCATTGCTATGAGTGTTTGAGTTGGATGGAACAGAATAATTTCATTATGGGAAAGAAAAAGAAATTGGTTTCGTTTGACGCGTATTTTTGGTGTGAGGATAAAGTTCCTTTAAGTCCTAAACTTTGGTTTCACATTTATTTCTGCTAATTAGGTCATCTATTTATTTACTGTATAATTATTTATTTATTAATTTATGAATTTAATTTATATATACAATAAATAAAAAAAAATTTACCCGCACCTACCTATTTAAAAGTCTTGTTATTACAAATAAAAGAAAAAAAATTAACTGGACCACAACTTAAAAACTCATTTAACTTTTAGAAAAAGTATTTTCATTACACATATCAATTAATTAATTAAATTTATTTGATGATTACACAGGAGGATTGGGTTTTGTGTAGAGTGTTTCACAAGGGCAAAACAGACAATAGTGCCAAACTAAGCCCACAATTCATGTATGAGGCCACACCTTCATCCCTAACTTTGGCTTCATCATCATCATCCCCACCAACAAACCAAACAAATTGCAACAATTTGCATGTTATTGGGTATAACCAACTTCCCAATTTCTCATCATCATCATCACCAATGGCAATCCACCATAATCATCATCATCATCATCATCATCAAAACCAAAACGGTTCTTCCTCTTTGATGAATCTCCTTCAATTTTCCACTAAGGAAAATAGTACCATTACTCAACTAAGTCCCAAAGGTGGTGGTGGCGGCGGCGGCGGCGACGACGGCGGCTATGGGTTCATGTGGGACATGGATCTTGAGGAAAATAGCTTCCATGATGGTGGGGTTATTGCATCAAACTTGAACGACATGAGATTTGAGGTTGATAATAACACTATGGTTATGTTGTAG

>AdNAC2

TATTCATCACCAATGTATTAACTAAGTCCTGTTAGATCATAAATCCTTGTCAAGATAATATGATCTGCTTTCACTCCTAATGAAATTGTTTCAATCTGGTGTCACACACTCCTTCCAAGTATTCTGTTTATAAATTATAATGAAAATATTTGGTTGATGATAAACACCAGCTTGGGTTTCACGTCAACACCCTTATTATAAATACCTTAGCTTCTCTATTTGTACTATAAATACCACTCATCTTCACCATTACTCTAACTTGACACATTCCCTCCTCTTGTTTTCTCTGCTTATCTTCTTAATGGGAGATAACAATGTGAACCTTCCACCGGGGTTTCGATTTTATCCAACAGATGAAGAGCTTGTGGTCCATTTTCTTCATAGAAAGGCAGCACTCTTACCTTGCCACCCTGATGTCATCCCTGATCTTGATCTCTATCCTTATGATCCTTGGGAACTTGATGGTATATTTATTCTCTCTATTCTCTCTATTCTATATAGTCACATAGGTTGCGTTTAAAGAAAGGACATTGAGACAGGGATACAGAAACACAAAATCGTGTTTGACAGAGGAGACATGGACAGAGAGACAATGTGTCTAGAGACACTGAATTAGTGTATTTTGTGTCTATCCTGACAGGAAAAACACGGAAATACTAACAAGGGATACAACTTTTTTTTTCATTATTTTTGTTAATTTTTTATAATATTTTTTATTATTATATTTTTCATCTCAAATTTTTGAATGAAAAAAAAAATAAGTTTTCATAATTTGTTCTAGTTTATCACTAAATAGAATACAAGAACACAAAATTTTATGTTTCTATTACCTTGTCCTGTCCTATTTTCACAAACAAACGCAACCATAAATACAACAACTGAATGTTTATATATTTATAGGTAGAGCGTTAGCAGAGGGAAATCAATGGTACTACTACAGCAGGAGAACACAGAGTAGGGTGACTGAGAATGGATATTGGAAAGCAACAGGAATGGAAGAACCAGTGATGACAAGCTCAACCAACAAGAGAGTTGGCATCAAGAAATACTTTGTGTTTCATCTTGGTGAATCCCCTTCTGCTATCAAAACAAATTGGATAATGCAAGAATATTGCCTTTCCGATTATTCTGCTTCCTCTAGCAGATCCTCCAAAAGAAAATCAGTAAGCTTTATAAGCATAATATTTATATATATCAACTTATTGAAAATTTATTATTATTGATAACTGTAATTGGTTTGTTTTATTATTCAGGATTATAGTAAATGGGTGATATGTCGTGTTTATGAGCGCAATGGAGATGATGATGATGGAACGGAGCTCTCTTGTTTGGATGAAGTTTTCTTGTCACTCGATGATCTTGATGAAATAAGCTTACCAAATTAAATTAATTAATCAAGCTAGCTAGCTGCAT

>AiNAC79

TATTCATCACCAATGTATTAACTAAGTCCTGTTAGATCATAAATCCTTGTCAAGATAACATGATCTGCTTTCACTCCTAATGAAATTGTTTCAATCTGGTGTCACACACTCCTTCCAAGTATTCTGTTTATAAATTATAATGAAATTATACATGATAAACACCAGCTTGGGTTTCACGTCAACACCCTTATTATAAATACCTTAGCTTCTCTATTTGTACTATAAATACCACTCATCTTCACCATTACTCTAACTTGACACATTCCCTCCTCTTGTTTTCTCTGCCTATCTTCTTAATGGGAGATAACAATGTGAACCTTCCACCGGGGTTTCGATTTTATCCAACAGATGAAGAGCTTGTGGTCCATTTTCTTCATAGAAAGGCAGCACTCTTACCTTGCCACCCTGATGTCATCCCTGATCTTGATCTCTATCCTTATGATCCTTGGGAACTTGATGGTATATCTATCTATTCTCTCTATTCTATATAGTCACATATATAGATACAACAACAACAACTGATCTGAATGTTTATATTTATAGGTAGAGCGTTGGCAGAGGGAAAGCAATGGTACTACTACAGCAGGAGAACACAGAGTAGGGTGACTGAGAATGGATATTGGAAAGCAACGGGAATGGAAGAACCAGTGATGACAAGCTCAACTAACAAGAGAGTTGGCATCAAGAAATACTTTGTGTTTCATCTTGGTGAATCCCCTTCTGCTATCAAAACAAATTGGATAATGCAAGAATATTGCCTTTCCGATTATTCTGCTTCCTCTAGCAGATCCTCCAAAAGAAAATCAGTAAGCTAAGCACAATATTTATATATATATATATATATCAACTTAGTATTATTCATAACTGTTGGTTTGTTTTATTATTCAGGATTATAGTAAATGGGTGATATGTCGTGTTTATGAGCGGAATGGAGATGATGATGATGGAACGGAGCTGTCTTGTTTGGATGAAGTTTTCTTGTCACTGGATGATCTTGATGAAATAAGCTTACCAAATTAAATTAATCAAGCTAGCTGCATTAATTAAGATAATCCAAAATGGATAATTATTAAGTATATAGCAGTGTGATGTTGCAAGTATAGGTAGATTTAGATTTCTATAGCAGCCCTTTAACACAAGAGGAAGTGGGGCACTATGTGAAATTCATGACCAAACAATATGTACTATGACGTTATGCTTTCAAAGATTCCATTTATCATTTTACCACAAATTTTTCTCTCCTCTTTTCTCCATATAAACACACGTGGACAATATTAATCCAAAACATACATGCCATCATGCATGTTGTTTAATTTCTTTGTTTATTATCATCATATCATATATAGTGCATGTTTG

>AdNAC4

CTACCCTCATCCCCATGTAGGGCCTGAAAGCTATGTAGATCTTTCTATTAACTCCCATCCTCTCTCTCCTCTTTTTTCTCAACACCTATTTAAAACACACGCCTCATTTTCTCCTCTTTTTTATATATTAACTACTCTGCCTCAAACCCTCAGTTTAATTTCTTCAATTTCTACATTTTAACCATCAATCTCTTATCTCTGCCTAACACCACTGCACCACCCAATTCGTACAACTATTACACTCTCTAAGTATAAAGACAGAGAGAGAAATTAAAGGGGGTCCAAACTCGAAGCACTAGTTACTACTACGATAGCTAGATATAGTATTATTATCAGTGTTGGATGGCAATTGCAGCGCCGAATTCATCTCCGACAATGAGTCTGAGTCACAGCCACAGCCACGAGGACGGGGGGACGACGACGGCGGCCTCCACCACGAACGACAACCTGAACGGGAACGGGAAGCAAGAGGATGATGATCACGAGCATGACATGGTGATGCCGGGGTTTCGTTTTCACCCGACAGAAGAAGAGCTGGTGGAGTTCTACCTTCGCCGTAAGGTGGAGGGCAAACGTTTCAACGTTGAGCTCATTACTTTCCTTGATCTTTATCGCTATGACCCTTGGGAGCTTCCTGGTATACCTAGCTTCCCTTTTCTTCACGCAATTCAATTCAATTCTTTATAAATATGTTCTTTGTTTACAATTTTGGCTTAATTGGCAGCGTTGGCGGCGATAGGAGAGAAGGAATGGTATTTCTATGTGCCTCGAGACAGAAAATACAGAAACGGAGATCGTCCGAATCGAGTGACGACGTCGGGTTATTGGAAGGCAACGGGAGCAGATAGGATGATAAGGACGGAGAATTTCAGGTCCATCGGGCTGAAGAAAACCCTAGTTTTCTACTCTGGGAAAGCTCCTAAAGGCATCCGTACAAGTTGGATTATGAACGAGTACCGTTTGCCCCAACACGAAACTGAACGATACCAAAAGGTACTTCCTTTTCGATCCATCCTATCTTTATACATCTATATGTTCTTCAAAGATCCAAGAAATGAACGAAGCTATAGCTAGTAGGGAATGGAATCATGGTTATTAATTAAATGGTGGTAGGAATAAATACGAGAGTTGGCAGAATATTAAAGTTAGCACCGCTAGTAATTATGACGACTACACCATCTCCATGCATCAAATTTGAAATTTGAATTAAATTTTTGCAGGCGGAGATATCGCTGTGCCGGGTTTACAAAAGAGCTGGAGTTGAAGATCATCCGTCGTTGCCGCGGTGTCTGCCAACGAGGGCTCCATCTTCAAGAACTGTTGATCATCAGAAGAACAAGCAGCAGCACCACAACGATCAACTCAACATGGGATTTGCGGGGAACACCGCCGACGGAGCTTCTGATAATCGTGATCATGATGTAACCACCGCTCTCGCCCTCTCCAAACACAACACAAATGCTTATCGTGCTCCTTCCATGGGACTCCCACCGCTGCTTCTTCCCTTGGACGACGAAGCCGCCTTCGTCCTCATGCAGCAGCAGCAGCACCATGCTGGCCCTTCTTCAGGAACCACCACCATGATGGATGATCTCAACAGACTTGTAAGCTATCAACACCAGTACTACAACAGCAGCAGTAGCAGTAGTAACAATAATAATCCCAATCATCATCATCACCTGTTAATGCAACAACAACAACAGCAGCAGCAGCAAACTCCTCCTGCAATAATGTCTCTGAATAACACTCCTTCTCCGCTTGCAACCGCCTTCTCTGACCGCCTGTGGGAGTGGAATCCACTCCCGGAGGCCAACCAGCGCCAGTACAGCAACATGTCCTTCAAGTAA

>AiNAC69

CTACCCTCATCCCCATGTAGGGCCTGAAAGCTATGTAGATCTTTCTATTAACTCCCATCCTCTCTCTCCTCTTTTTTCTCAACACCTATTTAAAACACACGCCCCATTTTCTCTTCTTTTTATATATTAACTCTGCCTCAAACACCCAGTTTAATTTCTTCAATTTCTACATTTTAACCATCAATCTCTTATCTCTGCCTAACACCACTGCACCACCCAATTCGTACAACTATTACACTCTCTAAGTATAAAGACAGAGAGAGAAATTAAAGGGGGTCCAAACTCGAAGCACCAACTACTACTACTACGATAGATATAGTATTATTATCAGTGTTGGATGGCAATTGCAGCGCCGAATTCATCTCCGACGATGAGTCTGAGTCACAGCCACAGCCACGAGGACGGAGGGACAACGACGGCGGCGTCCACCACGAACGACAACCTGAACGGGAACGGGAAGCAAGAGGATGATGATCACGAGCATGACATGGTGATGCCGGGGTTTCGTTTTCACCCGACAGAAGAAGAGCTGGTGGAGTTCTACCTTCGCCGTAAGGTGGAGGGCAAACGTTTCAACGTTGAGCTCATTACTTTCCTTGATCTTTATCGCTATGACCCTTGGGAGCTTCCTGGTATAGCTTCCCTTTTCTTCACGCAATTCAATTCAATATATATACACCTTGAATCTTGTTGTTTGTTTACAATTTTGCCTTAATTGGCAGCTTTGGCGGCGATAGGAGAGAAGGAGTGGTATTTCTATGTGCCTCGAGACAGAAAATACAGAAACGGAGATCGTCCGAATCGAGTGACGACGTCGGGTTATTGGAAGGCAACGGGAGCAGATAGGATGATAAGGACGGAGAATTTCAGGTCCATCGGCCTCAAGAAAACCCTAGTTTTCTACTCTGGGAAAGCTCCTAAAGGCATCCGTACAAGTTGGATTATGAACGAGTACCGTTTGCCCCAACACGAAACTGAACGATACCAAAAGGTACTTCCTTTTCGATCCATCATATCTTTATATTCAAACATCCAAGAAATCAACGAAGCTAGATAGTAGGGAGCTAGAGGGAATCGAATCATGGTTATTAATTAAATGGTGGTAGGAATAAATACAAGAGTTGGCAGAATATTAAAGTTAGCACCGCTAGTAATTATGACGACTACACCATCTCCATGCATCAAATTTGAAATTTGAAAATTAATGAATTAAATTTATGCAGGCGGAGATATCGCTGTGCCGGGTTTACAAGAGAGCTGGAGTTGAAGATCATCCATCGTTGCCGCGGTGTCTGCCAACAAGGGCTCCATCTTCAAGAACTGTTGATCACCAGAAGAACAAGCAGCAGCCTCACAACGATCAACTCAACATGGGATTTGGGGGGAACACCGCCGATGGAGCTTCTGATAATCGTGATCATGATGTAACCACCGCTCTCGCCCTCTCCAAACACAACACAAATAATACTAATAATGCTTATCGTGCTCCTTCAATGGGACTCCCACCGCTGCTTCTTCCCTTGGACGACGAAGCCGCCTTCGTCCTCATGCAGCAGCAGCACCATGCTGGCCCTTCTTCCGGCACCACCATGATGGATGATCTCAACAGGCTTGTAAGCTATCAACACCAGTACTACAACAGCAGCAGTAGCAGTAGTAACAATAATAATCCCAATCATCATCATCACCTGTTAATGCATCAACAACAACAACAACAACAACAACAGCAGCAGCAGCAAAATCCTCCTGCAATAATGTCTCTGAATAACACTCCTTCTCCGCTTGCAACCGCCTTCTCTGACCGCCTGTGGGAGTGGAATCCACTCCCGGAGGCCAACCAGCGGCAGTACAGCAACATGTCCTTCAAGTAA

>AdNAC10

CTCTCATCTTCTCCGTTCTTCAAACCTTTTTCACTTTTTTTTTCTTTATCACCAAAATCCTCGGGATCTGATCTTCACTCTCTTTCTCAAGTTCAATTTCCGAGGATCATATTCATAACAAAAACTACTACTACTTTTCTTATTGTCTCTCTGTTCTTTTGTTCGCTTAATTACCTTTCTTTCAGTGCTGTTTTCTTGTCTGCTTCTCTTTCCATTCCACAAATTGTAGTGTCACTACAAAAAAGCCTTTCAACAATTATCAATAACTTCCCATCAAAACCAAGTTTTAGTGCGTGTTTACTTAGATCGCTACCGGATTCTATTTGGGGAAAGAGCAACATAGATCAAGGTTCAAGGTTCTTCTGTCAAGTGTTGTTAATTCTCTACATCTATCTAGTTTTTTTTTTACTATTTTTATTATTATTATTTTCTTGCACATATTTTGATCTATTACAATTGGTATATGTATTAGCTAGTGTCATGTGAATGTTTTTCTAGATCGATCATCACTAGAGTTTGAAGATGGTGTGGATCGATGAATAAAAATTAAATAACTCGTGAAGGCTATTAATTAACAAAAGTCCAAAATAATTAATATGAAAGACAACTAAAAGACAGAAAGAACCTTCTCTTAGTCAAATGCTTGATGTCACCTGCATAAAAATCTTTGACCACACTTTAATTTGATCTCTCGGTGTTTCAGTTGGTCGGAGTTTATATCACTAAACTCCAAATCACATGCTCTCTCTCTCTCTCTCCCTCTCATTTTAATTTTTCTTGTTTTCCTTTTTTTTTTCGGAATTTGGTTTTGTACCTAACGACTATGATATTTTCATGATATCCTTCTTCAGACACGTCTTTTTTCTTCTGCAATTGATGTATATATATTTTTCTCCAATAATCTTTTTGGAACAGAACAAGCTGAAAGTGTTTGCCTTATGTGCTTGCTTGGTATATATTTTGTGCTATTTATTTACACAATTATTATGTGAATAAATGTGACTCACTTTGAGTGCTCTACAACCTAAAGAGGCTATGAAATAAAGTAACATGTTTTAGTGCTATATCATACATATCCTACAACATCATTCAGCTTCCATAATTGCATTCTAAAAAGCAGTGTGCCTTCTCCATAGTGTGTTACAACGTACTTCCATTTCTCTTTTCCTTTAAGCAAGAATTAAACGAGCAACATTTTATTTTTCTCTTGATAATTGAGTAAAATTTTTTGTAACGTAAAGTTATATATATATAACCAATTAATTAAGTAGTATATATAGCTTTATGTTGGTGTTTTATGTTCATATATACAGTAAGGGGCCTTTTTAATTAGTTTCTTTGTTCCTTCTTTATCATTTCATGGATCCAGTGGATCCAAGTTATGGTATGTACATTTTAGAGTTGTATATACGAGTGTTTTAAAAATATAATTATTTATATATTTTTATTAATTTAAACTTTTAAAACAAATAATTTTAGGAGAGAGTAGATATATAAAAGAGAAAATATATATGTGTAATTTTTTATAACTTAACTGTATCATATAGTATAATATAATACGATGAAGTTATTTGGGGTAGATTGTCTATGTATAATCAAATTGATTGATTGCAAATTTAAAGTTGTAATCTATACAAATTATTAGAGATATAGTTATTTATGTTATTTTTTTGTTAATTTAATTTTTTGACAGAAGTAGTTAAATAACACAAATCACAGGTAAAAGAAATAGCTATGTATAACTGCCAATTTTGTCTATTCCTTTCAACTAATTATAATTGCATGTGTACAAGTTATGATAAAAATTTGCATGTAATTATTTTTATATAAAATTAATAGTTAAAAATTATTAAATAATAATTTAATTAAATATGTTAAATTATTTAACGATTATTAACTATCAATTTTATGTAAAAACAACTGCACATAAATCTTTACGATATGATATACTCCCAAATAAAAAAAAAAAAGTAAATATAAAGGGGATGTGTGATAACATTCTTATATGTATTTAGTTTTTGGGTTAACAGATGTACTGCCTTCTTATTACTTGAATGATGTATATGCTTGACTTGACTTTCCATTAATCGTGCACTATTTATTAACATAATTGATTCCTTATTATCAATCTCATTGCTTTATATATGAGGTTCATTAGATGTATATTGAAACGAAGCAACTGTGTAACGCGTATACGTAGTGTCTAAGCGATGAAAAACTACGACTCTTCCTTCTCTCTCTCTTTGCTCTTTAATTATCAAAATAAACACAATCTGCATTAAATATGCTCCTTTGTTTTGGGCTTGGACCATTTTTGTACCAATAATATGAATTTATTCTTACATAATGTTGCTTTGAAGATAATTAACTCTGATCTTTAACTTTTAAAGCATGCATAGATTACTGTTGACATCATTTCAAGAGTAACTTAATGTCCTTAATGTAAATGCATATATGTAGCATCATTATTCATTCCCTATACTTGAAAAAAGATTTGCTAAAATTTTTTACATGAAAAGTGATGTTGTTTTCGTTTTCTGTTGTTGTCTTCCCTATTAATACAGGTTTTTGGTTGTTTTGGTGGCGACATGAACACCTTCTCCCACGTACCTCCAGGCTTTCGTTTTCATCCGACTGATGAAGAATTAGTTGACTACTACCTTAGGAAAAAGGTAGCATCCAAAAAGATTGATCTAGATGTCATCAAAGACGTTGATCTCTATAAAATTGAGCCATGGGATCTTCAAGGTATATATATATAAATATGAGAGCCTTGTTAATGTGGTTGGATTTTAATTAGCTTTACACACTTGAAACAAACATAAGGATGGTACGTTATCTTGTGATAGAATCACTCCTCCAAAAAATTATAGGTAGTTGGAGAAAACACATGAATAATTATATATTTAATATATCTTCTCATACAAAAATTTATTTTAAGTTTGTGTAAATTTTTGGCATATATTTTTTTTATTGGCTTTATACTAAAATTCTTTTTTGTCAAAATCAAGAAAAATCAAATTCTAAATTTTTAGGTTATAAAAATTCTAATATTATATCATCAAATAATTATTCCTAAAACTCAAATGGTTAAATCTACAACGATTGTCTCACGAGATTACATGTATTATTTCACAAATATTTTGAATTATTATATATTAGATATGGCATTATATATTCACGTGTTTTTGCTGAAATTAAACACAGTAAAAAGTAGCGTAACTTAGCGTGTCGTGGCAATCATGATCATAGAAATGATAGTATATATGGAGAATATGAAGTGAGATTTGATAAGTGTTTTGAAGATACTGCAGAACTATGCAAAATAGGAAGCGATGAAGAAAATGACTGGTATTTCTTCAGTCATAAAGATAAGAAGTACCCAACAGGAACAAGAACGAATAGGGCAACAAAAGCAGGGTTTTGGAAAGCCACGGGAAGAGATAAAGCAATATACTCAAAGCAGCATTGCCTTATTGGAATGAGAAAGACTCTTGTCTTCTACAAAGGAAGAGCTCCTAATGGCCACAAGTCTGATTGGATCATGCATGAGTATCGCCTTGAAACCAATGATTCACTCTTAATATAAAAAATTTGTACACATGTCTTCCCTTACATATTATAAAAAAAATGTGATTACATAACTGAAAAAAAAACATATTATATTAATACATCAAAATTAAATGAGAGAGAGAGAGAGATGAACTCATAAATTAAGCAATGCATAAAGAAAGACAGAATGCAAGTGAGAGAAGTATTGAATTGAGAGTTTGTAAAACTCTATTGATTTAGCTATAGCAATGTGGAGTAGCAGCTGAAGCTTGTCCAAGAAAGAAAAGGATCTTTAAAGAAACTATGCTAACCGTATATCTCAACTAACTCATTACCCTTTATAAAATCAATAAAACTTACATTGTTTTAGAAAGCTAAAGACATCCCAATACCCAAATGTCCATAGTGCATAGGAAATTCATAGGTAGGTTCTTCATCAAGGCCTTAAAACTTGAATTCCCTCTATTTCTTCATTTTGATATCTTTTAACTTATGGCATAGGAAATTCATAGGTAGGTTCTTTAAAGAAAATACTAAAAGCTATTGAAAAAAATTCTTTCTAATATATTAAATAAATAAAAAAATTTCTATGATTATTCTTTTAAATATTTTCCTAAAAAATGTTTTAGCTAAATCCATGATTATAAATTTGTAATGCAACAAGCATGTTAGACTTTCTTAACAGAAAAATTTAAAAGTTAGTAAAATTTAATTTTTTTTAGTATTTTTAATCAATAGCCTAATACATTTTATCTAGTAATTTAATAATATATTTTTAGTTGATATTTTTAAATATTATTAAATAACTACTAGTGAAAAATAATAAATTTTACTCATTATTGAGGATGAATTGTTTTTTGCACTAATTTCCTAACAAAGTTAGGGTTAACTGCAACAGGAAGAAGGGTGGGTTGTATGTAGAGTGTTCAAGAAGAAAATGGCAACAGTGAGGAAAATTGGAGACTATGATTCACCATGTTCTTGGTACGATGAACAAGTTCCCTTCATGCAAGATCTTGAATCTTCATCCCCAATAAAGCCACCAATAATTAACAACAACCATTATGCTTCTTCATACAACCATCACCAGTTACAATTACCCTGCAAACCGGAATTCCATCAACCAATGCAATACAACAACATGAACATGCCACGTCACGACGACGCTGCTGATAATAACAACAACTTCCTCCAACTTCCTCAGCTTGAAAGCCCTAATGCTGGAATTAGCCCCTTCTTGCAACAACAAGATCCTCATCAGCTATTGCAACAACAAAATTCCAACAACAGCAATTATCATCTTGATCAAGTAACCGATTGGCGAGTTCTCGATAAATTCGTTGCGTCGCAGCTCATGAGTCATGGTGATGATGATGGCCACAACCACAATAATAATGTTTCCAAAGAA

>AiNAC63

CCTTCTTCAAACCTTTTTCACTTTTTTTTTTCTTTTTCACCAAAATCCTCGGGATCTGATCTTCACTACTCTCTTTCTCAAGTTCAATTCCCGAGGATTCATATTCATAACAAAAACTACTACTACTTTTCTTATTGTCTCTCTGTTCTTTTGTTCGCTTAATTACCTTTCTTTCAGTGCTGTTTTCTTGTCTGCTTCTCTTTCCATTCCACAAATTGTAGTGTCACTACAAAAAAGCCTTTCAACAATTATCAATAACTTCCCATCAAAACCAAGTTTTAGTGCGTGTTTACTTAGATCGCTACCGGATTCTATTTGGGGAAGGAGCAACATAGATCAAGGTTCAAGGTTCTTCTCTCAAGTGTTGTTAATTCTCTACATCTATAGAGTTTGAAGATGGTGTGGATCGATGAATAAAAATTAAATAACAAGTCAAGGCTATTAATTAACAAAAGTCCAAAATAATTAATATGAAAGACAACTAAAAGACAGAAAGAACCTTCTCTTAGTCAATTAATGCTTGATGTCACCTGCATAAAAATCTTTGACCACACTTTAATTTGATCTCTCAGTGTTTCAGTTGGTCGGAGTTTATATCACTAAACTCCAAATCACATGCTCTCTCTTTCTCTCTCTCTCTCTCCCTCTTATTTTAATTTTTCTTGTTTTCCTTTTTTTTTTTTTCGGAATTTGGTTTTGTACCTAACAACTATGATATTTTCATGATATCCTTCTTCAGACACGTCTTTTTTCTTCTGCAATTGATGTATATATATTTTTCTCAAATAATCTTTAATTTGGAACAGAACAAGCTGAAAGTGTTTGCCTTATGTGCTTGCTTGGTATATATTTTGTGCTATTTATTTACACAATTATTATGTGAATAAATGTGACTCACTTTGAGTGCTCTACAACCTAAAGAGGCTATGAAATAAAGTAACATGTTTTAGTGCTATATCATACATATCCTACAACATCATTCAGCTTCCATAATTGCATTCTGAAAAGCAGTGTGCCTTCTCCATAGTGTGTTACAACGTACTTCTATTTCTCTTTTCCTTTAAGCAAGAATTAAACGAGCAACATTTTATTTTTCTCTTGATAATTGAGTAAAATTTTTTGTAACGTAAAGTTATATATATATATAACCAATTAAGTAGTATATATAGCTTTATGTTGGTGTTTTATGTTTATATATATAGTAAGGGGCCTTTTTAATTAGTTTCTTTGTTCCTTCTTTATCATTTCATGGATCCAGTGGATCCAAGTTATGGTATGTACATTTAGAGTTGTATATAAAGAGAAAATATATGTGTAACTTTTTATAATCTTAACTGTATCATATAGTACAATATAATATGATGAGGTTATTTGGGGTAGATTGTGTATGTATAATCAAGTTGATTGATTGCAAATTTAAAGTTCTAATCTATGCAAATGGTTAGAAATATAATTATTTATATTATTTTTTAATTAATTTAAATTTTTGAAAAAAAGTAGTTCAATAACACAAATCACAGGTAAAAGAAACAGCTATATATAGCTGCCAATTTTGTTTATTCCTTTCAACTAATTATAATTGTATATGAATAAGTTATGGTAAAAATTTGCATATAATTGTTTTTATGTAAAATTGATAATTAAAAATTGTTAAATGATAATTTAATTAAATATATTAAATTATTTAATAATTATTAACTATTAATTTTAAGTGAAAATAACNNNNNNNNNNNNNNNNNNNNNNNNNNNNNNNNNNNNNNNNNNNNNNNNNNNNNNNNNNNNNNNNNNNNNNNNNNNNNNNNNNNNNNNNNNNNNNNNNNNNNNNNNNNNNNNNNNNNNNNNNNNNNNNNNNNNNNNNNNNNNNNNNNNNNNNNNNNNNNNNNNNNNNNNNNNNNNNNNNNNNNNNNNNNNNNNNNNNNNNNNNNNNNNNNNNNNNNNNNNNNNNNNNNNNNNNNNNNNNNNNNNNNNNNNNNNNNNNNNNNNNNNNNNNNNNNNNNNNNNNNNNNNNNNNNNNNNNNNNNNNNNNNNNNNNNNNNNNNNNNNNNNNNNNNNNNNNNNNNNNNNNNNNNNNNNNNNNNNNNNNNNNNNNNNNNNNNNNNNNNNNNNNNNNNNNNNNNNNNNNNNNNNNNNNNNNNNNNNNNNNNNNNNNNNNNNNNNNNNNNNNNNNNNNNNNNNNNNNNNNNNNNNNNNNNNNNNNNNNNNNNNNNNNNNNNNNNNNNNNNNNNNNNNNNNNNNNNNNNNNNNNNNNNNNNNNNNNNNNNNNNNNNNNNNNNNNNNNNNNNNNNNNNNNNNNNNNNNNNNNNNNNNNNNNNNNNNNNNNNNNNNNNNNNNNNNNNNNNNNNNNNNNNNNNNNNNNNNNNNNNNNNNNNNNNNNNNNNNNNNNNNNNNNNNNNNNNNNNNNNNNNNNNNNNNNNNNNNNNNNNNNNNNNNNNNAAAAAAAAAAAAAGAAAATATAAAGGGGATGTGTGATAACATTCTTATATGTATTTAGTTTTTTGGTTAACAGATGTACTGCCTTCTTATTACTCGAATGATGTATATGCTTGACATGACTTTCCATTAATCGTGCACTATTTATTAACATAATTGATTCCTTATTATCAATCTCATTGCTTTATATATGAGGTTCATTAGATGTATATTGAAACGAAGCAACTGTGTAACGCGTATACGTAGTGTCTAAGAGATGAAAAACTACGACTCTTGCTTCTCTCTCTCTTTGCTCTTTAATTATCAAAATAAACACAATCTGCATTAAATATGCTCCTTTGTTTTAGGCTTGGACCATTTTGTATCCTTTTTGTACCAATAATATGAATTTATTCTTACATAATGTTGCTTTGAAGATAATTAACTCTGATCTTTAACTTTTAAAGCATGCATAGATTACTGTTGACATCATTTCTAGAGTAACTTAATGTCCTTAATGCAAATGCATATATATAGCATCATTATTCATTCCATATACTTGAAAAAAGATTTGCTAAAAATTTTTACATGAAAAGTGATGTTGTTTTCGTTTTTTGTTGTTGTCTTCCCTATTAATAATACAGGTTTTTGGTTGTTTTGGGGGCGACATGAACACCTTCTCCCACGTACCTCCAGGCTTTCGTTTTCATCCGACTGATGAAGAATTAGTTGACTACTACCTTAGGAAAAAGGTAGCATCCAAAAAGATTGATCTAGATGTCATCAAAGATGTTGATCTCTATAAAATTGAGCCATGGGATCTTCAAGGTATATATATAATTATGAGAGCCTTGTTAATGGGGTTGGATTTTAATTAGCTTTACACACTTGAAACAAACATAAGGATGGTTAAAGCACGTTATCTTGTGATAGAATCACTCCTCCAAAAGATTATAGATAGTTGGAGAAAACACATGAATAATTATATATTTGATATATCTTCTCATACAAAAATTTATTTTAAGTTTGTGTAAATTTTTGGCATATATTTTTTTTATTCTTTTTTGTCAAAATCAAGAAAAATCAATTCTAAATTTTTAGGTTATAAAAATTCTAATATTAAATAATCAAATAATTTTTCCTAAAACTTAAATGGTTAAATCTACAGTAGTGATTCTCTCACGAGATTACATGTATTATTTCACAAATATGAATGTTAGGGGCAGCAACTTTTGTAATTTGTAGCCATCAAATAGCCATTATTGATGGTTTTAATGATGTGAGATTGGTATGAGATTTTATCCAATTCCTCACTTTTTTTTGTTGGTTACATACTGGTCAGAATTTAACAAAGTTGCTGCCCCTAGACTTTTCCAAAGTATATATTAGATATGGCATTATATATTCACGTGTTTTTGCTGAAATTAAACACAGTAAAAAGTAGCGTAACTTAGCGTGTCGCAATCATCATCATAGAAATGATAGTATATAGGGAGAATATGAAGTGAGATTTGATAGGTGTTTTGAAGATACTGCAGAACTATGCAAAATAGGAAGCGATGAAGAAAATGACTGGTATTTCTTCAGTCATAAAGATAAGAAGTACCCAACAGGAACAAGAACGAATAGGGCAACAAAAGCAGGGTTTTGGAAAGCCACGGGAAGAGATAAAGCAATATACTCAAAGCAGCATTGCCTTATTGGAATGAGAAAGACTCTTGTCTTCTACAAAGGAAGAGCTCCTAATGGCCACAAGTCTGACTGGATCATGCATGAGTATCGCCTTGAAACCAATGAAAATGGAACTGCTCCGGTAATTTTCTTTATATAATTATTACTTTCTAATTNNTTACACATGTCTTCACTTACATATTATCAAAAACAAAATGTGATTACATAATTGAAAAAAAAATTATTATATTAATACATCAATATTAAATGAGAGAGAGAGATGAACTCATAATAAGCAATGCATAAAGAAAGACAGAAAGCAAGTGAGAGAAGTATTGAATTGAGAGAATGTAAAACTCTATTGATTTAGCTATAGCAATGTGGAGTAGCAGCTGAAGCTTGTCCAAGAAAGAAAAGGATCTTTAAACAAACTATGCTAACCGTATATCTCATCAACTAACTCTAACCCTTTATAAAATCAATAAAACTTACATTGTTTTAGAAAGCTAAAGATATCCCAATACCCAAATGTCCATAGTGCATAGTAGTGCATAGGAAATTCATAGGTAGGTTCTTTAAAGAAAATACTAAAAGTTATTGAAAAAAATTCTCTTTCTAATATATTAAATAAATAAAAAAAAATTCTATGATTATTCTCTTAAATATTTTCCTAAAAAATGTTTTAGCTAAATCCATGATTATAAATTTGTAATGCAACAAGCATGTTAGACTTTCTTAAGAGAAAAATTTAAAAGTTAGCAAAATGTAATTTTTTTTAGTATTTTTAATCATCAGCCTAATACATTTTATCTAGTAATTTAATAATACATTTTTAGTTGATATTTTTAAATATTATTAAATAATTACTAGCCAAAAATAATAAATTTTACTCATTATTGAGGATGAATTGTTTTTTGCACTAATTTCCTAACAAAGTTAGGGTTAACTGCAACAGGAAGAAGGGTGGGTTGTATGTAGAGTGTTCAAGAAGAAAATGGCAACAGTGAGGAAAATTGGAGACTATGATTCACCATGTTCTTGGTACGATGAACAAGTTCCCTTCATGCAAGATCTTGAATCCTCATCCCCAATAAAGCCACCAATAATTAACAACAACCATTATGCTTCTTCATACAACTACCACCAGTTACAATTACCCTGCAAACCGGAATTCCATCAACTTATGCAATACAACAACATGAACATGCCACGTCACGACGACGCTGCTGATAATAACAACAACTTCCTCCAACTTCCTCAGCTTGAAAGCCCTAATGGTGGAATTAGCCCCTTCTTGCAACAACAAGATCATCATCATCAGCTATTGCAACAACAAAATTCCAACAGCAATTATCATCTTGATCAAGTAACCGATTGGCGAGTTCTCGATAAATTCGTTGCGTCGCAGCTCATGAGTCATGGTCATGATGATAATGATGACGATGGCCACAACAATAATAATAATGTTTCCAAAGAAGTAATAAACAGTTATTCTGATGCTTCAATTCTCCATGTGGCTCAACAGATTGCTATGCTGGCAAATGGATCGTCATCTTCATCATCATCAAGGAGGCCTCAAATTTCTCATCAGGAATATGCTGCTTCAACTTCCACATCAAGTTCTCAGATTGATCTCTGGAAGTCATCGTCATGA

>AdNAC23

ATTTGTTTAGGGTTAGGGCTTCCTTCCTAGCTAGCTAGCTAGCTACTTCATTCTCTATACTTGATCCAACAACATGAATCAAAGTAGACCAACAAATCATTGAGGACATTCGTCTGAGTTAGGAGCTTCATGATCTTGGGAGGCAGATTTGTTACTGCACCACCCTCATCTCTTATTACCTTGCTTCATCTCTGTGAGCTCTCTCATCGTACTTTAATTTGCTTAAACTATATATGTTGAGAACATTCATATTTATTTTCATACATATAATACTCACCATTCACGCTTTCCTTTCGATTCTACTTAAGCTAATAATTAGTCAATATAATAATATGGTCTCATATCTATGTCCCAATTCTTAATTTATGTTTGGAACACTTAGAAATTAAAAAAGATAGCGACAATCAATTTATGTTTAACTACTTTAACTGAAAAGTAAATTTAAGATAATTTACTATGTTTTGAATGTATGAAAAAAGGTTTTTTTTTAAGTAGACAATTACCAAAAATGGATATGAAATAATATTATTTAATATTTAAAATAGTTGATAACAAATTGATAAAATCATTACAATCTAAAAAAAAAACAAACTTTTACTTATATTAATAAGTTTTAGAGCGTTGCTTTTATTTATAAGTTGTATGGTAGTTGAAATTATTGGTTAATAATCTATTTTTTTCTACTCTTCCCAAACATGTTCTTGATTTTCCATTTCCCATTTGTGTCTGGTTTTGTGATTGAGCATGTTACTTTGTATCATTTGGAAGCATGGATCATGAACTAGTGGGAACAGCAATGAGTGGCATTAATTACTTTAGTATTCATGTGCAGTAATATACAAATAAATAATACTGTGATCTATTGTGTTGTGCATGTGTCTAATATATAATCAAGGAATGTTAGGCGCAGCAATTTTTGTGATTTGTAGCCATCAAATAGTTATCAATGATACTTTTAATGGTGTGAGATTTTATCTAATAGTGTAAAATTACTCTATTTTCTTTTACTGGTTACATACGGGTGAGATTTCAATAAAAGTGCTTCCCTCTAGACTTTCTTTCTCGTATAACGCCGCATAATGTAATCATGATGATGAGTTTTATTGATCTTAAAATGAATTCTTGAATAACAGGATTAACCATTGATCAGAATCAGATAGAGAGAAGATGAATACATTTTGTCATGTTCCACCGGGTTTTAGGTTCCATCCAACGGATGAAGAACTCGTTGATTACTACCTTAGAAAAAAAGTTAATTCATGTAGGATTGACCTTGATGTCATCAAAGATGTTGACCTCTACAAAATCGAACCCTGGGATCTTCAAGGTAACATACATATTCTACTACGCAAATTTAATTATTTAACAGAAGATGCATTCTTAAGTATAACTTATTAATTTGATGAATATATAGAGCTATGCAGACTAGGAACAGAAGAGCAAAATGAGTGGTACTTCTTTAGCCATAAAGATAAAAAATATCCAACAGGAACTCGCACAAATAGAGCAACTGCAGCAGGGTTTTGGAAAGCAACAGGGAGGGACAAAGCTATATATTCCAAGCATGACTTGATTGGGATGAGAAAGACTCTCGTCTTCTACAAAGGTCGAGCCCCTAATGGCCTCAAATCTGATTGGATTATGCACGAATATCGTCTTGAAACCGATCAAACTTCGGCTGCTACTCCTCACGAAGAAGGATGGGTTGTGTGTAGAGTGTTCAAGAAGAGAGTGACTTCCATTATGCGTAAGATGAGTGATCATGATTCCCCTTCCTGCACTTGGTATGATGACTCCTCTTTCATGCACCAACAACCAGATCACTTTGACAACTCTTGTTCTTCTTCATCAAAGCACCAACTAATCCCTAATAATAACTGTGATGTCTTCTACCAACAACAAAATAACAACTTGCCTCTTCATCATCTTCCACTTCTTCATCAAACTGCTGCTCTTTCCAATCATAATAATAATCCAATCATGGCACCACCATTTGCTGCTATTAATAATAATGAAACTACTGCTTTTCAAGAACAAGGGAAAAGCTTAATTCATCATCAGGCACTACTCTATGGAAATTTAAATGAAGAGCAAGCTTCTTCTTCAGCTGCTGCTGCTGATTGGAGACTTGTTGACAAGTTTGTTTCATCACAGCTTAGAGAAGATCATCATGTCTCCAAACAAGAATTGATGATGCCAGAAAATAATAATAATAATAATAATGATAATGGTGCCTCAACATCAAACTCAAGCTGTCCAATAATGGACGTGTGGAAATAG

>AiNAC76

ATTTGTTTAGGGTTAGGGCTAGCTACTTCATTCTCTATACTTGATCCAACAACATCATAGGTTGCACAAGAATCAAAGTAGACCAACAAATCATTGAGGACATTTGTCTGAGTTAGGAGCTTCATGATCTTGGGAGGCAGATTTGTGACTGCACCACCCTCATCTCTTATTACCTTGCTTCATCTCTGTGAGCTCTCTCATCGTACTTTAATTTCTGTGCTGTCTCTTTTTTTTTTTTTTTTTTATAAAAAAATATAAGACTCGAACCCGCAACCTCTTAATTAAGTATGGGGAGATTATGCCATTTGAGTTATTACTCATTGGCATTTCTGTGCTGTCTCTTGCTTCTTAATTAATTAGCTTAAACTATATATTTTGAGAATATTCATATTTAGTTGCATATATATAATACTCATCAGTCACCATTCATGCTTTCCTTTCGAAGAAATTACTTAAGCTAATTAGTCAATATAATAATATTGTCTCATATCTATGTCCCAATTCTTAATTTATGTTTGGAACACTTAGAAATTAAAAAAGATAGCGACAATCAATTTTTGTTTAAATATTTTAACTGAAAAGTAATTTTAAGATAATTTACTATGTTTTGAATGTATGAAAAAAGGTTATTTTTAAGTGGACAATTACAAAAAATGGATATGAAATAATATTATTTAATATTTAATATTTGATAACAAATTAATAAAATCATTAAAATTGAAAAAAATAAACTTTTACTTATACTAATAAGTTTTAGAGCGTTGCTTTTATTTATAAGTTGTATGGTAGCTGAAATTATTGGTTAATAATCTATTTTTTTCTACTCTTCCCAAACATGTTCTTGATTTGCCATTTCCCATTTGTGTCTGGTTTTGTGATTGAGCATGTTACTTTGTATCATTTGGAAGCATGGGTCATGAACTAGTGGGAACAGCAATGAGTGGCATTAATTACTTTAGTATTCATGTGCAGTAATATACAAATAAATAATATATACTGTGATCTATATTGTGTTGTGCATGTGTCTAATATATAACGCCGCATAATGTAATCATGACGATGAGTTTTATTGATCTTAAAATGAATTATTGAATTGCAGATTAACCATTGATCAGAATCAGATAGAGAGAAGATGAATACATTTTGTCATGTTCCACCGGGTTTTAGGTTCCATCCGACTGATGAAGAGCTCGTTGATTACTACCTTAGAAAAAAAGTTAATTCAACTAGGATTGACCTTGATGTCATCAAAGATGTTGATCTCTACAAAATCGAACCCTGGGATCTTCAAGGTAACATACATATTGTACTACTTAGATTTAATTATTTAACAAAAGATGCTTATTAATTTGATGAATATATAGAGCTATGCAGACTAGGAACAGAAGAGCAAAATGAGTGGTACTTCTTTAGCCATAAAGATAAAAAATATCCAACAGGAACTCGCACAAATAGAGCAACTGCAGCAGGGTTTTGGAAAGCAACAGGGAGGGACAAAGCTATATATTCCAAGCATGATTTGATTGGGATGAGAAAGACTCTCGTCTTCTACAAAGGTCGAGCCCCTAATGGCCTCAAATCTGATTGGATTATGCACGAATATCGTCTTGAAACGGATCAAACTGCGGCTGCTACTCCTCAGGAAGAAGGATGGGTTGTGTGTAGAGTGTTCAAGAAGAGAGTGACTTCCATTATGCGTAAGATGAGTGATCATGATTCCCCTTCTTGCACTTGGTATGATGACTCCTCTTTCATGCACCAACAACCAGATCATCACTTTGACAACTCTTGTTCTTCTTCATCAAAGCACCAACTAATCCCTAATAATAACTGTGATGTCTTCTACCAACAACACAACAACAACAACTTGCCTCTTCATCATCTTCCACTTCTTCATCAAAATAACAATAATCCAATCATGGCACCACCATTTGCTGCTATTAATAATAATGAAACTACTGCTTTTCAAGAACAAGGGAAAAGCTTAATTCATCATCAGGCACTACTCTATGGAAATTTAAATGAAGAGCAAGCTTCTTCTTCAGCTGCTGCTGCTGCTGCTGATTGGAGACTTGTTGACAAGTTTGTTTCATCACAGCTTAGAGAAGATCATCATGTCTCCAAACAAGAATTGATGATGCCAGAAAATACTAATAATAATAATAATGATAATGGTGCCTCAACATCAAACTCAAGCTGTCCAATAATGGACGTGTGGAAATAG

>AdNAC67

ATAATATTTTTCGATCTCTATTCATTAATTAACAAAAGTAAAATAGAGATATTAGTGAAATGAGTTATTATTGGTTGTTTTTTATCAGGAGAGAGCATCAATGGATATGGAATCATGTGTGCCTCCAGGATTTAGATTTCACCCAACAGAAGAAGAACTTGTGGGGTATTACCTCAAGAGGAAAATTAACTCCCTCAAAATTGATCTAGATGTTATAGTTGAGATCGATCTCTACAAAATGGAACCATGGGACATACAAGGTACATATCACAATATAGAGATACAATGTAATTAAAATTAAAGTACTTATATTATTATAACTTCCTCAACTAATTTTTTCTTATTGGTTTTAAATTTTTAACTTTTGTACCGAATTTATACACTCTTATATAATTTTTATTATTAAATGTGGCGTAAGATTCACATATAATAATGAGATTTACAGTATTTATTATTTAATAAAAAATTAGTCACCAAATTAATCATTATATATTTGTATAAAATATATGTATATTTTATATTTTTAACATATATAAGTGTAAGTGTGGTACATAAGTTGAAATTCCCACATAATAAGTAGATGAATTTGAACAATGTGCAGATAGATGCAAGCTAGGATATGAGGAACAAAATGAGTGGTACTTTTTCAGCCACAAAGACAAGAAGTATCCAACAGGAACAAGAACAAACAGAGCAACTGCTGCTGGATTCTGGAAAGCAACTGGGAGAGACAAGGCTGTTATGTCCAAGAACAGGATCATTGGTATGAGGAAGACTTTGGTCTTCTACAAAGGACGTGCCCCTAATGGCCGCAAAACTGATTGGATTATGCACGAATATCGCCATCAAACCTCTGAACATGGCCCTCCTCAGGCAAGTTCTTTCTCTACATTGTTTTAATTTTTAATTTGATAAAAATTTAGGTGTACTCGTCTTTATATAAAGTTGATAATTAAAAACGGTTAAATACATATAAAAATTTAGGTGCAATTGATTTCACGTGAAGTTGATATTTGAAAATCGACTCTCAGATATCAACTTCACATAAAGTCGACTTCACCTGAATTTTCACCTTAAATAAATTAATTATTAATAAAAAATAACAATTAATAATGCTACAGGACCACAAAATTTATTATTTTCGACTAACAATTAACTAATAAACTTACTGATATAAAAAAAACATTCAAATACATCAACAGAATATAAAGTATTATTTAGTATAAATAATAATTACTTATTTTCGAATTTTTTTTCTTAAAATAATTTAGTATCTAGCTACATCTTAATGGCTAATTAATGCTAGTAATTAAATAAATAACAAAATTTAGACATTAATCAATAATTGATGATCAAAATAATAAATTCTAATAATTCTTTAACATTATTTGTAATTTAATTAACATGAAAAAGCTCTTATGCTTTTCAAAAGTCATAAGTGAAAAGAGAAGGCAAACATAAATTTTTATAGTAAATAGGATGTCAAAAACCATTCTTATTATTCCACTTTTCTTCCTCTTTCACACTCCATCTTTATTTTCAAACATATATACAGTAGTCATCACTCTTTTTTCCAATTACACAATAATGCTTTCAACTTATCTTTTTCGCTTTCTCATACACTATTTAAACTTTTTATTCTTTTCAGAAATAATAATGTGGACCTGGCTATAGTAATTGTTAAATCATATCTTCTTTATTTATTTATTAATTGTTTTGCTAAGTTCTATAAAAGAGCTTCTAGATTTCATCAATTTTTAACCAATATAACATTAAAGGCGTTCTTAGTTCTTACTGATGATGATCATTATTAGGGTGCAATTGAAAATCATTGAGTAATATTTTTGCATGCATTGCGTTTATATTGAGCATTAATTAATTAGCTAAACACAAGAGTTATTCGTAAAATGTTTTGAACATTTTAAATTAAATTAAAGATGATATCCCTAAGAAATTTCATAACAAACATTGTTTTTTGAAGCATGAGGCAGCCAAATTTTGGTGTTATGATTGAGAATCGTTAATACGTAGTAATGTCAACCATGTAATGAAAACATATCGCACTTACTCATAAAAGTTACTTTGCTTTTCATAAAGCCCAAGAGCCAAATGCAAATCATAACACGCTTCTCCTACTTTCTCTATCTTATCTTACACAACTTCATACATAATATCCTTCTAAGCTTATAATTAATATATCTAATTTATTATCTCTACTTACTTTTATTTTATTTTACTTTTAGTCTTGCTTGCTCATACAAATTGATTATTTTAGTTGGTAAAAAAAAATCATAAATAAAGAATAATGTAAATAGTTATATCTCTAACATATTTTGTTTTTTTATTATTTGCTTTGTACTAAAATTTTTTTCTTTTAAGATCATCAAGAATTAAACAGATTAATAGTCAAATTAGTATATGAAAGATAAGATGTTTTTTAAATTCATCCCTAAAAGATTTTTTCAATCAAATTGGTCATTCAAAAATTACGAATTAATCATATTTGTCCTCCAGTCACTCCATTAATAATTTTCTTCAAAGATTAATGTTGTAAAACGTTAATTAATAACATATATAATACCTGATATGTCTAATTAGATATTGACCAAATATGTTTATAAAAATTTATTAATTTAGTCATTTTTTCTAATGATAAAAATCTTATTCCTAATATGACCTAGTGGTTAAATTGATAGATTTTCGTAAACATATTTAGTCAACGTCTAATTGAACATGTTATGTGTCATGTATGCTATCAGTAAACATTTCACATCATCAATTGTTGACGAAAATTATGAATGGAGTAGCTAAAGGACAGATATGATTAATTCGTAATTTTTGAATGACCAATTTGATTGAAAAAATCTTTCAAGAACGAATTTAAAGAATGTCTTATCTTTCAGAAACTAATTTGACTATTAACCCAAAATTAAACTCTGCATCATAAAATTATTTATCTTAAAAATTTACATTAATTTCTTGATAATCAAGCGTTGACTAATATAATTGAATATTACAGGAAGAAGGATGGGTTGTGTGTAGAGCATTTCGAAAACCAAGTCCAAGTCATCAAAGGCAATTAGGGTTTGATCCATGGTGTAGTAATCATCATCATCAAGCACATTATTTCAGAGATCAAAGTAGCTATGGTGGAAGGCCCTTATCAATCACAGATCTTCTAACTTCAGAAACTCATCATCATCATCTTCTGAGTCACCCTACCGAAGGTACAAATTTTAGTCATTCCTTCGGTTCAGATCATCACCATCATCAAGAACAACAAGAGTTTGTAATATCAAATAATCATCAACAACTCATTGAGCTTCCACAGCTAGATAGCCCAACAACAACAAGTTTTGCAGTCAAAGAATCATCATCCATTAATAATAACAATGAAGAGTATTGCAGTGATGACAGGAACAACAACAACAACAACATTGATTGGAAAAGCTTGGATAACCTGTTTGCTGATACTTCTAATTACTTCTCATCAAATCCAAACATGTCCCAATTCATGACCATCAATCATCATCTAGGTTGTTTCCCTGGTTCATAA

>AiNAC65

ATTCATTAATTAACAAAAGTAAAATTAGAGATATTATTTAAAATTTTATGATCCAAGATAGTAAAATGAGTTATTGGTTGTTTTTCAGGAGAGAGCATCAATGGATATGGAATCATGTGTGCCTCCAGGATTTAGATTTCACCCAACAGAAGAAGAACTTGTGGGGTATTACCTCAAGAGGAAAATTAACTCCCTCAAAATTGATCTAGATGTTATAGTTGAGATCGATCTCTACAAAATGGAACCATGGGACATACAAGGTACATATATATAGTATTCATCACAATATAGAGATACAATAATGTAATTAAAAGTACTTATATTATTATAACTTCCTCAACTAATTTTTTCTTGTTGGTTTTAAATTTTTAACTTTTATACCGAATTTATACACTCTTATATAATTTTTATTATTAAATGTGGCGTAAGATTCACATATAAAAAGTGTATGAGAGATGAATTTACCAAAAATGTTGCAAATTAAATATGATTTTCATAATCCACATATATAGATTTGGTTTAATTCAGTGTAAGTAAGTGTGGTACATAAGTTGAAATTCCGATGAATTTGAACAATGTGCAGATAGATGCAAGCTAGGGTATGAAGAACAAAATGAGTGGTACTTTTTCAGCCACAAAGACAAGAAGTATCCAACAGGAACAAGAACAAACAGAGCCACTGCAGCTGGATTCTGGAAAGCAACTGGGAGAGACAAGGCTGTTATGTCGAAGAACAGGATCATTGGTATGAGGAAGACTTTGGTCTTCTACAAAGGACGTGCCCCTAATGGCCGCAAAACTGATTGGATCATGCACGAATATCGCCATCAAACCTCTGAACATGGCCCTCCTCAGGCAAGTTCTTTCCATACATCCTACAAATTTTTAATTAGATAGAAATTTATGTGTAGTTATCTTTATATAAAGTTGATAATAAAAAATAATTAATTATTGATATAAAATAACAATTAATAATGCTACAGGACCACACAATTTATTATTTTCGAAACAATTAACTAATAAACTTATTGATATAAAAAAAAACATTCAAATACACCAACAGAATATAAAGTATTATTATTTAGTATAAATAATAATTACTTATTTTCGAAATTTTTTTCTTAAAATAATTTAGTATCTAGTTACATCTTAATGGCTAATTAATGCTAGTAATTAAATAAATAACAAAAGTATTTCATTTAAAAAAATATTAGGTANNNNNNNNNNNNNNNNNNNNNNNNNNNNNNNNNNNNNNNNNNNNNNNNNNNNNNNNNNNNNNNNNNNNNNNNNNNNNNNNNNNNNNNNNNNNNNNNNNNNNNNNNNNNNNNNNNNNNNNNNNNNNNNNNNNNNNNNNNNNNNNNNNNNNNNNNNNNNNNNNNNNNNNNNNNNNNNNNNNNNNNNNNNNNNNNNNNNNNNNNNNNNNNNNNNNNNNNNNNNNNNNNNNNNNNNNNNNNNNNNNNNNNNNNNNNNNNNNNNNNNNNNNNNNNNNNNNNNNNNNNNNNNNNNNNNNNNNNNNNNNNNNNNNNNNNNNNNNNNNNNNNNNNNNNNNNNNNNNNNNNNNNNNNNNNNNNNNNNNNNNNNNNNNNNNNNNNNNNNNNNNNNNNNNNNNNNNNNNNNNNNNNNNNNNNNNNNNNNNNNNNNNNNNNNNNNNNNNNNNNNNNNNNNNNNNNNNNNNNNNNNNNNNNNNNNNNNNNNNNNNNNNNNNNNNNNNNNNNNNNNNNNNNNNNNNNNNNNNNNNNTTGGACATTAATCATTAATTGATGATCAAAATAATAAATTTTAATAATTCTTTAATATTATTGGTAATTTAATTAACATGGAAAAGCTCTTATGCTTTTCAAAAGTCATAATAAGTGATCAAAAGAGAAGGCAAACATATATTTTTATAGTAAATAGGATGTCAAAAACTATTCTTATTATTCCACTTTTCTTCCTCTTTCACACTCTATCTTTATTTTCAAACATATATACAGTAGTCATCACTCTTTTTTTCCAATTACACAATGCTTTCAACTTATCTTTTTCCCTTTCTCATATACTTAAACTTTTTATTCTTTTCAGAAATAATAATGTGGACCTTGCTAGTGATTGTTAAATTATATCTTCTTTATTTATTTATTAATTGTTTTGCTAAGTTCTATAAAAGAGCTTCTAGATTTCATCAAATTTTAACCAAATAACATTAAAGGCGTTCTTAGTTCTTATTGATGATGATCATTATTAGGGTGCAATTGAAAATCATTGAGTAATGTTCTTGCATGCATTGCGTTTATATTGAGCATTAATAATTAATTAGCTATACACAAGAGTTATTCGTAAAATGTTTTGGACATTTTAAATTAAATTAAAGATATCCCTAAGAAATTTCATAACAAGCATTGTTTTTTGAAGCATGAGGCAGCCATATTTTGGCGTTATGATTGAGAATCGTTAATACGTAGTAATGTCAACCATGTAATGAAAACATATCGCACTTACTCATAAAAGTTACTTTGCTTTTCATAAAGCCCAAGAGCCAAATGCAAATCATAACACGCTTCTCCTACTTTCTCTATCTTATCTTACACAACTTCATATATACATACATGATATATCCTTCTAAGCTTAATTAATATATCTAATTTATTATCTCTACTTATTTTTATTTTATTTTAACTTTTAGTCTTGCTTCCTCATACAAATTGATTATTTTAGTTGGTAAAAAAAATCATAAACAAAGAGTCACGTAAATAGTTATATCTCTAACACACTTCTTTTTTTTATTATTTGCCTTGTACTAAAATTTTTTTCTGATCATCAAAGAATTAAACTCTAGACTTCTAGTATGGTACTGTATCATAAAATTTTTTGGAAAAGTCAGTCTAGCAGCTTTTTTAAATTTTGATCAGCATATAACCAACAAAGAAAAGTGAGTTATTGAATAAAATCTCACACCATTAAAATTATTATTGATGGCTATTTGATGGCTATAAATCACAAAATTGTTGGTGCCCTAACACTCCTCAAATTTTTTATCTTAAAAATTTACATTAATTGATAAAAAGAGACGTATGAATGAGAATATTTATAAGACTCTTGATAATTAAGCGTTGACTAATATAATCGAATATTACAGGAAGAAGGATGGGTTGTATGTAGAGCATTTCGAAAACCAAGTCCAAGTCATCAAAGGCAATTAGGTTATGATCCATGGTGTAGTAATCATCATCATCAACCACATTATTTCAGAGATCAGAGTAGCTATGGTGGAAGGCCCTTATCAATCACAGATCTTCTAACTTCAGAAACTCATCATCATCATCATCTGCTGAATCACCCTACTGAAGGTACAAATTTTAGTCATCCCTTCGGTTCAGATCATCATCATCAACAAGAACAACAAGAGTTTGTAATATCAAATAATCATCAACAACTCATTGAGCTTCCACAGCTAGATAGCCCTACTAGTGCTTCTCTCTCAGCACCACCAACAACAAGTTTTGCAGTCAAAGAATCATCTTCCATTAATAATAACAATGAAGAGTATTGCAGTGATGAGAGGAACAACAACAACAATATTGATTGGAAAAGCTTGGATAACTTGTTTGCTGATACTTCTAATTACTTCTCAAATCCAAACATGTCCCAATTCATGACCATCAATCATCATCTAGGTTGTTTCCCTGGTTCATAA

>AiNAC70

GTATTTAAGCACACCTTCTTCCCTTCATTATTTTACTTTCTTAATCTTTCATCACTTCATAATTAATTGCTCATCGCTATGGATGCGGCTTTGGATTTGCCTCCCGGTTTCAGGTTCCACCCTACAGATGAGGAGATCATCTCTTATTATCTCACTCACAAGGTTTTGAACACAAGTTTCACCGCAACTGCCATTGGAGAAGTTGATCTCAATAAGTGTGAGCCTTGGGACTTGCCTAGTAAGTCACCATTTCTTGCTTCTTCTTTATTTGTTTTCTGGCAGTGAAAACTCTCGTGCAGTTGACATATTTGATAAAATTGTAAATGTTTATTTAGAGAAAGCAAAGATGGGGGAGAAAGATTGGTACTTCTTCTGGCAAAGAGATAGAAAGTATCCAACTGGGATCAGAACGAATCGAGCCACGGAATCCGGCTATTGGAAGGCCACCGGAAAAGACAAAGAGATTTACAAAGGGAGAAACCTTGTTGGTATGAAGAAAACCCTTGTGTTCTATAGAGGTAGAGCCCCTAATGGACACAAAACCAATTGGGTTATGCATGAATTCAGATTGGAAGGCCTTTTTGCTACTTACAACCTCCCTATACCTGCTAAGGTACTCATCAATTTCTTTTGTAAAAATTCACCAACAATTGTGTTCGTGAAAAGCTATTAGTTAAGAATTGTTATATGATTTGATACGTTGACTAAATTATTATTTAATGATTTTTAACTATTAACTTTACAGAAAAATAACAACTATACGTGAATATTCACCTAATTATATATATGTAGTCTCAAAAGGTGGAAACTCAGGTTGCAGTCGACTTCACGTGAAGTTGATACCTGAGAGCCGTTAGATAATTTGACTGATTTGATTAAATTTTTATCCAACAGACTTTCATGTATCAACTTCACGTAAAATCGACTGCACCTGAGTTTTCACCTGATTTTTTAATATTGTATTTTTCAAGGATTGCTTTTCTCTTTTGCAGGAGGAATGGGTTGTGTCGAGGGTTTTCCATAAGAACACAACAGAAAAATTGAACCCAACTATTCCATCTGGCCTCTTTAGGATAATGAAGAACATGAACTCAATTGGAGATGATGATCTTGTAGATTTTTCTTCTCTCCCACCTCTCATGGATCCTTCTAATAATTATGATGATGAACACACCACCACCACCAACAACAACTATGTCAATAGTATGTTTGCATCATCATCAGATTATAATATTACTATTCAGCAAAACAAGAAAGATATGATGGGAATAAGGAATAATAATAATAATATTAGAGCATTGTTAATGTACGACGGTCCATCATCATCATCATCAGAAGTAGTTGCTCCTCCTCTCTCTGACTTGGAATTAGGCCTCTGGGATTTATAACTTCTTACCAAAACATTATTAAACATTATTTCTGATGATAT

>AiNAC7

CCGCTTCACGCTCACCACCACCACCAGAGAAAACGTTATCGCCCACGTGTATTCTAGAAACAGCTGGCTTACCATGCCACGTGTCCCCCTTCGCTCGCTATAAATACCCTTCTCTCCCTTACGTTTTAAGTTTCAAGCGCAGCCGTAGTTCTTCTAAGTCTTTCAGAGGGGCTAAAAGCTTCTTATCTACCTTCACCAGATCTCACTCTAATTATTTAGGTTTCACTAAAGTATCTCAGTTCTTTCAAAAATGGCATCGGAGCTTCAATTGCCGCCTGGATTTCGATTCCATCCAACAGATGAAGAACTCGTGTTGCACTATCTCTGCCGTAAATGCACTTCACAACCGATTGCTGTTCCGATCATTGCCGAGATCGACCTTTATAAATATGATCCCTGGGACCTTCCTGGTAATTACTGTAATGTTAATTAGTTTTTAATTCGTTAATATATACTTGTTGTGACTGACTATTTAATTATAATATTGTTTTTTGCAGGTATGGCATCCTATGGTGAGAAGGAGTGGTATTTTTTTTCACCTAGAGATAGAAAATATCCGAATGGGTCTAGACCGAATCGGGCTGCAGGAACTGGTTACTGGAAGGCAACCGGAGCCGATAAGCCTATTGGACAACCCAAACCAGTTGGGATTAAGAAAGCTCTGGTCTTTTACTCTGGAAAGGCTCCCAAAGGAGATAAGACCAATTGGATTATGCACGAATATCGTCTTGCAGACGTAGATCGTTCTGTTCGCAAAAAGAATAGCCTCAGGGTATATATCTAGCTGATTAGTTGTCAAGTACAGAGTCATAAAAAAAAAATTCATTATGATGGGATGAGATTACCAATTTACCCCTCTGGTCCCTAATCTTCTTGTTTTGCGGCCAGTCAGTGTGGCCAGCCATACAAATAAGCAGTGATTATCTTGTTGTACAGCATAAAGATAAGAAGCTGCGTGTGGACCCGTTGAGAATTTTTGGTGAAAAAACATGTGGCCCAAATTTGTTCTCATGACACACACCTTCGTGATCATTTGATGCGTGACATTGACTGATCACAAGTTGAACATGCAAACTTGTGGCGTGTGTGCATGAACTGTCTCAAATTGATTTTTATTTTTTAAAAAGGAAATTAATCAATTAATACGATCACGAAGAATCACAATCATTACCTTCTACAAGTTTTCTATGGATAGATCTAATAAACATTTTGCGGATTATTGTGCGATTATTTGTTGACTGATAAAACCATTGTGGTTTTTTTCAGCTGGATGACTGGGTGCTGTGTCGCATCTACAATAAGAAAGGATCAATCGAGAAGCAACAACCGAGCAGCGGCGTCAGTACCGTCGTGAACCAGAAGGCCGAATCTTCGGAAGTCGAAGACAAGAAGCCCGATATAGTTCCACGTGGCGGCGGCGGGGGCGTACTTCCACCGCATCCTCCTACGGCTCAGGCTTCGGCAGGCGGCGTGACGACAGATTATATGTACTTCGACAACTCCGATTCGGTTCCGAAGCTTCACACGGACTCGAGTTGCTCGGAGCAGGTGGTGTCGCCGGAGTTTGCGAGCGAGGTTCAGAGCGAGCCAAAGTGGAACGAGTGGGATAAAAACCTCGAAAGTGCGTATAATTACCTCGATGCCACACTCACCAACGGTTTTGGGTTCCCGTTTCAGGGTAACAATCAGATGTCGCCGCTCCAGGATATGTTCATGAACCTTCCGAAACCGTTCTGAACGCGCTAGTAATAGTAGAACACCAGAGAAAGAATCTTTTTATGGAAAACCTTTGATATGCAGTATGGTGCATTCCACGGCAGCACGAGACGTTAAGGTCCATGAGTGTGCCTGTGCGGGTGCGAGTCCATGGATGGGTCAACGAGATGGAACAGCAAGGGGAGAATCTGAAGAAGGGAAAATGAAAAAATGCATGCGTTAGGTGTGCAGCATTGTGCGCCCATTGATTTGGGTCGTGGGATTAGAATATCGTTTGGGGAGTCTGACGGTGGATGTTGGGTAGTCGCTAGGTTAGGCTGTGTATAGTTTATTTTGTACAATGTAGAGTGTAAGACCGGAGGGGGTAAACTATACGGTGGATGGTGGTGTATGTTGTTTCCATCTATTTATGATTAGTCAATATAAATGGTTAATTATTGATTTTGACACCGAATTAAAATATATGGTGGCAAGAATAAAATATTATGATGTGATAATGGTGACATTATGTCAA

>AdNAC66

GAATAATGCAAGGTGGATTAGAGTTACCGCCAGGGTTCAGGTTTCACCCGAGCGACGAGGAATTGGTGAACCACTATCTCTGCAAGAAATGCGCAAAGCAATCAATTGCTGCTCCAATAATTAAAGAAATCGATTTGTACAAGTTCGATCCGTGGCAGCTTCCAGGTACTTAAACGCTCGATTTCTGTTTTCAGTGGATATTTATTCGTAAGAATTCGAGTTTGACTTATTTATTGATTGCAGAGATGGCGTTGTACGGAGAGAAGGAGTGGTACTTTTTTTCGCCGAGGGATAGGAAATATCCGAACGGATCCCGGCCGAACCGGGCGGCTGGGAGCGGGTACTGGAAGGCGACCGGGGCGGATAAGCCGATAGGGAAGCCGAAGGCGTTAGGGATAAAGAAAGCGCTAGTGTTCTACGCCGGAAAGGCCCCGAAAGGAGTGAAGACTAATTGGATTATGCATGAGTACCGTCTCGCCAATGTTGACAGATCCGCCGCCAACAAACTCAACAACAACAACTTGAGGGTATTAACTACTAGAACCTTCTCTTCAAACCTTTTTTACCCTAATTTATTTTATTTAATAATAATAATAATAATAATAATAATAAAGGTTTGTGATTATAAAAATTGGTTATGGAGAGAGGTTGATATTTAAATTCTTAGGATTAGAATTATTTTTTAAAAATAATTTGAGCATAACTGTTTTTTAGTTTTTATTGGATCCAATTAACATATTCTTTTAATTTTTTTATTCTATATGGGTGATTCATTCGGGGACTATTTTTAGTGTGCATCTAGTCTGGTTATTTATAATATCATTTAATCAAATTATTTGTGATGCAAGATTTTTGAAGTACTGTTGATGAGAATCAACTTTTCTTTTAGTATGATCTGATTGGAATATGTTTTGGTGCAGCTTGATGATTGGGTGTTGTGTCGAATCTACAACAAGAAAGGGAAGATTGAGAAATTCAACTCTGCCACAACAGGGTTGGAACAGAAACTACCAAAGTTTTCCCCAGGAGAGATACTTCACTATGATCATGAGCATGAGCATGAGACCAAGCCAAAGATTATCCACAATTTCTCCAACAATGAGCACCAATTGTACATGGACACATCAGATTCCGTTCCAAGGCTGCACACGGACTCTAGCTGCTCGGATCACGCGGTTTCGCCGGACGCCACCTGCGACAAGGAGGTGGAGAGCAACCCAAAGTGGAGCAATGAGCTAGATATGCAGCTGTTTGATACCTTTGATTTTCAGCTCAACAACTATGATAATAACCTCCCAATGAATGATGATGACCTTTTTGGAAATCAGTTCCAAATGAATCAGCTCATGTCTTTCCAAGACACATTCTTGTTCCCACAAAAGCCATTTTGATTCTTTTGACCATTAAGAATTATTGGGAATTGGAAGAAGAGGGGGAGAAAAAAATTGAACCTTTTTTTCTGGGAAAATGTGGTGGAGGACTATTGCCTAATGAGATCATGGGGACCAAAGCATATGTTCATTTTTTGTAGTGAAAACTCAGCATTTACACAATTCACACCTACCTAACAATTTCACTTTTGGACCATTGGGTTTTGGTGGCTATTCTTTGTTCTTGGGAGATTAGGGAGGGAGATGTGAAGG

>AdNAC76

TTTAATTAACCCTCCCTATATAGACCTCAGCTTCTCCAAGCCTCTCTCTCACGTTCCAACTTCTAAGTTCTAAGTCGCAAACAAGAAGAAAAAAAGAAAGGAAGAGGCCGCAATGAAGAGTGAATTGGAATTACCACCTGGATTCAGGTTCCACCCCACTGATGAGGAGCTTGTGAATCACTACTTGTGCAAGAAATGTGCTTCACAGTCAATTGCTGTTCCTATCATCAAGGAGATCGATTTGTACAAGTTTGATCCATGGCACCTTCCAGGTACACTACTTTATTTCTCAAGGGGAAACAAAGACAAAAACAAAAAATAAGTACTGAATTCTTTTTCTATCTAAATAAATGTACAGAGATGGCTCTATACGGCGAGAAAGAGTGGTATTTCTTCTCTCCCAGGGACCGCAAATATCCGAACGGATCACGCCCAAACCGGGCTGCGGGTACAGGGTACTGGAAGGCCACAGGTGCCGATAAGCCCATTGGAAAGCCCAAGGCCCTGGCCATCAAGAAGGCACTGGTGTTTTACGCTGGCAAAGCCCCCAAAGGAGTGAAAACCAATTGGATCATGCATGAATATAGACTCGCTAATGTTGACAGATCCGCAGGCAACAAGAAAAATAACTTAAGGGTATGTCGTGCTCTATGCACCTTAATTTCAATCTCACAACATTTTAAATTTGGTAAAAAATATAATGATTGGGAATTATTGCAGCTTGATGATTGGGTGCTATGCCGAATTTACAACAAGAAAGGAAAGATTGAGAAATACAACCATCTTGGGGCGGCGGATCACAAATCAGCATCTTCGTCGGAGGAGAATGAGAGGAAGCCGGAGGTGAAGGAGCGATTGCATATGGATACGTCGTCGGATTCGGTGGTATCGGCGGATGTGACGTGGGAGAGTAGGGAGGTGCAGAGCGAGCCAAAGTGGAATGACCTGCTTGACCAAGTCTTTGATTTCCAGTTAGGCAGTTTCGTTGATTTCTCATCGGCTGGAGATGACCCTTTTGCCCCCCAGCTCTCTCCTTGGCATCAGGACACGTTCATCACATTTTAATTCATTCTTTCCAACACAACAAGAAAAACTAAGATTGTTTAGTTTAGCCACCATGACTCATTTGGATTTAACTCGGGAACCAAGGACGATGAGATCATATATGTAAAAATAAATAAGAAAAATAATCTCACCAATGCCACCTAGTTTGTTTTCATGTTACCATTGTTGGAGTGTTAAGCATCTACTCCTGTGGAAAATTCTTCCTACCATTTTTTAAAATATATGTTATTATTTAATTCAACATATTCAAGGAATGATTTCG

>AiNAC22

TTTAATTAACCCTCCCTATATAGACCTCAGCTTCTCCAAGCCTCTCTCTCACGTTCCAACTTCTAAGTTCTAAGTCGCAAACAAGAAGAAAAAAGAAAGGAAGAGGCCGGAATGAAGAGTGAATTGGAATTACCACCTGGATTCAGGTTCCACCCCACTGATGAGGAGCTTGTGAATCACTACTTGTGCAAGAAATGTGCTTCACAGTCAATTGCTGTTCCTATCATCAAGGAGATCGATTTGTACAAGTTTGATCCATGGCACCTTCCAGGTACACTACTTTATTTCTCAAGGGGAAACAAAGGCAAAAACAAAAACTAAGTACTGATTATTCTTTTTCTATCTAAATGAATGTACAGAGATGGCTCTATACGGCGAGAAAGAGTGGTATTTCTTCTCTCCTAGGGACCGCAAATATCCGAACGGATCACGCCCAAACCGGGCTGCGGGTTCAGGGTACTGGAAGGCCACAGGTGCTGATAAGCCCATTGGAAAGCCCAAGGCCCTGGCCATCAAGAAGGCACTGGTGTTTTACGCGGGGAAAGCCCCCAAGGGAGTGAAAACCAATTGGATTATGCATGAATATAGACTCGCTAATGTTGACAGATCCGCAGGCAACAAGAAAAATAACTTAAGGGTATGTCGTGCTCTATGCACCTTAATTTCAATCTCACAACATTTTAGATTTGGTAAAAAATATAATGATTGGGAATTATTGCAGCTTGATGATTGGGTGCTATGCCGAATTTACAACAAGAAAGGAAAGATTGAGAAATACAACCATCTTGGAGCGGCGGATCACAAATCAGCATCGTCGGAGGAGGAGAATGAGAGGAAGCCGGAGGTGAAGGAGCGATTGCATATGGATACGTCGTCGGATTCGGTGGTATCGGCGGATGTGACGTGGGAGAGTAGGGAGGTGCAGAGCGAGCCAAAGTGGAATGACCTGCTTGACCAGGTCTTTGATTTCCAGTTAGGCAGTTTCGTTGATTTCTCATCGGCTGGAGATGACCCTTTTGCCCCCCAGCTCTCTCCTTGGCACCAGGACACGTTCATCACATTTTAATTCATTCTTTCCAACACAACAAGAAAAACTAAGATTGTTTAGTTTAGCCACCATGACTCATTTGGATTTAACTCGGGAACCAAGGACGATGAGATCATATATGTAAAAATAAATAAGAAGAAAAATAATCTCACCAATGCCACCTAGTTAGTTTTCATGTTACCATTGTTGGAGTGTTAAGCATCTACTCCTGTAGAAAATTCTTCCTACCATTTTTTAAAATATATTTTATTATTTAATTCAACATATTCAAGGAATGATTTCG

>AiNAC51

ACACACATACCTTGGACCACCAACACCAACACCTTCATTCTCCATTTTCTCACATACCCTTTTTGTAATAGCAGCAGCTGGTTTCGTTTTCTTGCTTTAATGGGGGATAGCAATGTCAATCTTCCACCGGGGTTTCGATTTTATCCCACTGATGAAGAGCTTGTAGTCCATTTCCTTCAGAGAAAAGCAGCACTTCTACCTTGCCACCCTGATGTCATTCCTGATCTTGATCTCTACTCTTTTGATCCATGGGAACTTGATGGTATCATTCTTTCTCTATGTTCCAATACTTGCATGACACATTGCAAGAGAATTGAAGTTGATGAATACAATCATGTGTGTGTGTGTGTGTGTGCAGGTAGAGCTTTGGCAGAGGGGAACCAATGGTACTATTACAGCAGAAGGACACAAAATAGGGTCACTGCCAATGGTTATTGGAATCCAATGGGAATTGAAGAGGCAGTGGTTTCAAACTCAAGCAACAGGAGAGTTGGTATCAAGAAATTTTATGTGTTCTATGTTGGAGAAGCCCCTCATGGTAACAGAACCAATTGGATCATGCAAGAGTATCGTCTTTCAGATTCTGCAGCATCCTCTAGCAGATCATCAACCAAAAGAAAATCACAACCAAAAACA**GT**TAGTACCACAATTTCACCTCAAAAACATAGTTCAAGTTTAATAATGTATGCCACTTAGATTATTATTATTATTATTATTATTGATTTTTTTTCCTTCCTGGTTTTGGTGTATTCAGGATCATAGTAAATGGGTGGTATGTAGAGTCTATGAAAGTGATGAAAATGATGATGATGGTGATGGTGATGGAACAGAACTCTCTTGTTTGGATGAAGTTTTCTTGTCATTGGATGATCTTGATGAAGTAAGCTTGCCAAATTAGATGATGCAAAACTTTTGTTAGCAGCATTATTATCCCAAATGGGGATAATTAATTAAGGCTAAATAGCTGTTTAATGCAAATTTGTTAGTACTTAATGTAGTTAAGTTTGTTTTTTTGTATTT**AG**CTTTAGCTTTAGCTTTAG

>AiNAC71

ATGGAGGAAGAGGATGATGTTCCACTTCCAGGGTTTAGATTCCACCCAACAGATGAAGAACTTGTGAGTTTCTATCTAAAGAGGAAGCTTGACAAGAAACCAATCAGCATCGAACTCATCAAACAGATTGATATCTACAAGTATGATCCTTGGGATCTTCCAAGTATGTATTTATTCTTTTCTTTTTTAATTAGTATTCCTCTTTAAATTCAAGAATATCATTTTCTCCCATTAAGATTTAATTATTATTACGAGTAACTAATAGCAGGGTTTCATCTAAACACAATTACTGAATCAGTAATATTAATAAAACAATAATGTAAACTTATTTTATTTAAACTGATTTTTGTTCTTTATCGAACATTTTTATTACTCAGTTACTTAATTATAATATAATATATTATTTCTTAGCCTACCTTGGGTTAATTAACTAATTAGTAGCTAGTGATCCAAAGTCTATATTAGGAAGTGAAAATTATTAAATGATAGCACTAAATGCATAAACCCAGTTTGATTATTAAGATATAATTAGTAGAATTATGAGGTATAGCCGCAATATTTTACAGGAAATTAATTGTACATAGAGTATGATTTATCAACCTTTTGATTTAGGAATAAACAGAATCGCACTTAGCATGCATGGTGAATATATCATATAGAATTTCTTTCCCTCTCTAATGATTATTAATCCATTATTATATATAAACTAATAGACAGAATAAATATAGAAAAAAACAAACATTTTCCTTTCCATTGTTAGTAACTACTTTACATACATATGTATGTTGCTAACACTATTGAATTTGGTTAATTAGAAGCGAGTGGAAGTGGAGGAGAGAAGGAAGGTTACTTCTTTTGCAAGAGAGGGAGGAAGTATAGGAACAGCATAAGGCCTAACAGAGTCACCAGTTCCGGCTTCTGGAAAGCAACCGGGATAGACAAGCCGGTATACTCCCATGGAGGCGAAGGAACCGACTGCATTGGACTCAAGAAGACGCTTGTCTACTACCGCGGCAGCGCCGGTAAAGGTACCAAGACTGATTGGATGATGCACGAGTTTCGCCTCCCTTCTGCCACAACCGAAAACAACACAAGCCTACTTGCCAACAACAAGAATAATAATAATATCAACAATGCCGATGTTGCCCAAGAAGCTGTAAGTAACTTAATTAGATAAATGGTTTTAAATTATTATAGTTGTGATTCAATCTGTGATTGCAATTCGCAGGCACAATTATTGTTATGATCATTATGATTATTACATTAAACTCCATGTGAATATTTCCGTCGCAATTATGATTAGGTATTCAGTAATAGATAGTCTTGTACACTTAATGTTCATATATAAATACTATATGTAGTTTAAATTAAGAAAAACATTTTCCTTTTTTGACACGGAAAAAAATAAACATTTTCCTTCCATCATAGGTTTAATTTTATATATATTATTATTCTTTTATACGCATGCTCTCACTTAACCTTATTTGCAATATACTCGTAGTCTTCTATGTATATATATAAGTTCTTTCATAAAAAGAAAACATTAGTAATATATTTCCCTCTTACCATGTAATATAGCAAACCCAAGTAATACAATAATAATAATAATATTTCAATTCTTGACAACTTACTTTCATAGTTAATAGTAGTATTTGATTTCTGAGTTAACAAATAATAAGCTTGTGATGGATGCAGGAAATCTGGACATTGTGTAGAATATTCAAGCGAAATGTGTCACAAAGGAAGCACACAGTAGACTTGAGATCACATTTAGTAACAGCTAATAGTAACAAGCACAAAACCACTAGAACCCATGTTGTTCAATCCAATAATAACAATATTAATCAACATCAAGAATCTTACATCAACTTTGGTGCAACAATCATTGGCCATCACCATTACCATCATCATCGTCATCAAAATGAACAGAAGCCAGTGACTAACTACACAGCATGCAATAACAACACTGATCAAATCCAAAGGAACAATAGCAATCATCATCATCATCATCAGTTGAACTATCACCCTTCTTCAGCGGTGGCTACTACTGTGCCACAGCAACAACAACAACAATATCATCATCATCATCAGCTAATGACGGCTCCAGCTTCTAACATGTGGATTAATCCTTCTGCGATGAACGATTTGTTTGCATTTGATGATGACTGGGATGAGCTTGGATCTGTTCTCAAATTCACTGTTGATACCCCTAGCTTGTAA

>AdNAC57

ATGGCAGAAACTCGGGTTCTACCTGTTGGATATAGGTTTCGTCCAACAGAAGAGGAAATTTTAATTCACTATCTCAATAACAAGCATTTGGGAAATGATGCAGAGATTAAGAACACTATTTCCCAAGTTGATCTTTGTAACTTTGATCCTTGGGATTTACCAGGTTCCTCTACCTTATTACTATTGTTTTATAAAAACTTTATATCTTTGATCTTTATAAAGTTTTTATACAAAAACTATGTAAAGTTTTAATTTTTAGTACATATTAGCAATCTTAGATTGTTGTTTTTTATTTTGAGAGTTGTGGTTCATACTTGGTTGAATTATATTATAGAACAATCGAAAGTGAAATCGGATGATCAAGAATGGTTTTTCTTCAATGAATTGAAATACATGAAAAACAAGCGGTGTAACAGAAAAACCAACATGGGATATTGGAAGATCACAGGAAAAGAGAGAATCGGGACAGACAGTGTCATAGGTACAAAAAGAACACTAGTTTTCTACGAGCGTCCACATAATGTCAAAACCAATTGGGTTCTTCATGAATATCATGCATTTGATCAAAAGGTAGGTTCTTGCCAGGTAAGAGATTGTATTGGAAATGAAATTAATTCTCCAATTTTTTTTTTTATCTCTATTATTGGTTTTGTCGTAAATTATGGAATCTTTTCACATCATTTTTTCTGTTTAAAGTCTTTTCTATTTCTAACATGTTTGGTTCAAGCAAAAATAAGAAGAAAGAAAGAGAAAGAGGAAAAATGAATGAAATTTTTTTTTTTTGTTTAGATGTTACAAAAAATGAAAAGGAAAGAAAATTTACACTAGTTTAAGAGAAAATAAAAGAAAAAAATTAAAAAAATGAGTGAAAATTTTTATTTATTTATTTTTGGATATTAAAAGAAAAAAAAATTTAGTGTAGGACTCACTAAAAAAATTTTTTCTTCATTACAAGTAAGAAAATAAAATAAAAATATTATTTTTTGTGTATTTATAATATTATCTATAGTTTTATTATTATTAATAATTATCAATATAAATTAGTTTTAATAGTTTTAATATATTATACTAGAAATTTATCTTTAAACATTATATATATATTAGNNNNNNNNNNNNNNNNNNNNNNNNNNNNNNNNNNNNNNNNNNNNNNNNNNNNNNNNNNNNNNNNNNNNNNNNNNNNNNNNNNNNNNNNNNNNNNNNNNNNNNNNNNNNNNNNNNNNNNNNNNNNNNNNNNNNNNNNNNNNNNNNNNNNNNNNNNNNNNNNNNNNNNNNNNNNNNNNNNNNNNNNNNNNNNNNNNNNNNNNNNNNNNNNNNNNNNNNNNNNNNNNNNNNNNNNNNNNNNNNNNNNNNNNNNNNNNNNNNNNNNNNNNNNNNNNNNNNNNNNNNNNNNNNNNNNNNNNNNNNNNNNNNNNNNNNNNNNNNNNNNNNNNNNNNNNNNNNNNNNNNNNNNNNNNNNNNNNNNNNNNNNNNNNNNNNNNNNNNNNNNNNNNNNNNNNNNNNNNNNNNNNNNNNNNNNNNNNNNNNNNNNNNNNNNNNNNNNNNNNNNNNNNNNNNNNNNNNNNNNNNNNNNNNNNNNNNNNNNNNNNNNNNNNNNNNNNNNNNNNNNNNNNNNNNNNNNNNNNNNNNNNNNNNNNNNNNNNNNNNNNNNNNNNNNNNNNNNNNNNNNNNNNNNNNNNNNNNNNNNNNNNNNNNNNNNNNNNNNNNNNNNNNNNNNNNNNNNNNNNNNNNNNNNNNNNNNNNNNNNNNNNNNNGGAACTAGCCATTGATATGTTGAGGGGCGAAACCTTATGCAACAAAAGCACGGTAATGGTCTCTAAATTTTGATTTGTTTTTTATATAAATTATGTATAAATTTTAGTTTAATATTTTTGAAAATTTTATTTTATTTCTTTATTATAAAATTAAATTTTTAGCTGCTTTAATTTTTGGCTAAATTTTTTAAATAAGTTTTTTTGAAGGAAGATCTTAAAATATAAGAACTTTTATTAATAATAATTTTTAAATAAGTTATTTTGTGTTTTCAAAATTATTATAGAAGTTCTTGTTTTAAAGTTGTGCATTAAATAGGAATAATAAAATAGCTTTTGAAAATAAGAGAAGTCACATTTTTTAATTTTTTCAAAAACTCTTAAATAATTTTTTAAAAAATTAAAAATTTATTTAAAAAATTGTACCAAACATTATTAATATAATTTTTTATAAGTCAAAAACTAAAAAAAATAAAAAAAATAACTTTTAAAGTTTTTCAAATGGACCTTAATCTAGTTTCCCCTTCCATATGTTCATATAACAATATTACCATTAAGTATTTATGTTCATAGAAAAATGCATTTTTTCCTATAACCTTTTTTTCTCTCCTGTACACATTTGACAAAGAAAATTTAAGAGGATTCATGCTGCATCAAAATCTGTCAAAAAAATAATTATCTTATTACTTTTTTTAGTTTTTTTCTATCTTTGGAATTTAAAATTTTTCGTTTATAATTCAACTTGGAAACAAAATTTTTCTATTTATTTTTAAATAATGTGAGAAAATATTATTTTTTTGTCAAACAATTTTTTAAGTAGAATCTAAAAGAAAATATATAGAAAAAGATAAAAAATAAATCCTCTCAAATTTTTTTCATGTATCGTTGTAAAGTAAAATTTTTTATTATGTATTTTAAGTTGAACCAAGAAAAAACATAAAAAAAATGTTGAGTGGAATAAAATTATACTTGAAAATATCAATTGAAAAAATTTATTACCAAAAGAAATATAGTAAAAAATTAATTTTTGTGATCACAAGAAAAAAATTGAGCTAAAATTTATTTCTTATCTTCAAATTAACTTGTTTGAATCCAATTTTTTGGCTCATGTGACAAAAGCTCATCTCTAAGATACAACATATTTTATAGGATTAATTATTCTGTTAGTCCCTATAATTTCGTAAAATTTTTAATTAGTTTCTTATACTTTTTTTTAATTGAGTTATTGCACCAATTTTTTTTTAATTGGATCTCTACATTTTTTTTTATTTAGGTCTTTATACCAAATTTTTTTAGTTGGGCCTCTATAAAATTAAGTCAATTACTACTAAGAGAGACTTAATTGAAAAAAATAGTACAGAGACCCAATTAAAAATAAAAAAAATATAAGAACCTAATTGAAAATTTCACGAAACTATAAAAACTAACAGAATAATTAAACCTATTTTATATTATATGAAACAATTTCTTTAATTTTATACTACAGTTGATAGGCCTCGTCGTTTATGACACTTATGGTTTAATCTCTTGTCGATTCATTGTCTTGGGTGATACAATTTTTGTACGCAGAGCAACATCGTATTGAGCCGTGTAATAATGAATGCTGAGAAAAGGGAACAGAAGCTAAAGACAAAAGCAAGCAACATAGTCGAAGAGGAGGAAGTAAAATGTGAAGATGAACCATGCAGCGAAATTACTGACTGTGTTACCCAAGCAACTACAGAAGATGCAATCATTCCTGATAATGTGAGTGAAAACATTAATATATTCAAAAATATTATTTGTATATTAAAATTAGTTATTATGTATTTGTTTATAAGTACATGTGTTATTTAATTTATTTTTAATATATATTTTATACTAGTAATTGATTTTGATAGTTGATTTTAATGTACAACTAATGCAATTATTTGACATTACAGGTCTATTTGGGAAACACTAAAAAGCTACTTTTTTTTTTTTTACTTTTGACTTATAAAAAGGTACATTAATAATATTTGGTATAATCTTTTAGATAAATTTTTAACTTTTCAAAAAGTTATTTAAGAGTTTTTAAAAAAGTTAAAAAACGTGACTTCATCTATTTTCAAAATCTATTTTATCACTCCTATTTAATGTACAACTTTAATACAAGGACTTTTATAATAACTTTTCAAACATAAAATAACTTATTTAAAAATTATTATTAATAAGATTCTTTATATTTTAAGCTTTTTTTAAAAAAAAATATTTAAAAAATTTAGTCAAAATGGCTCTATATATATCTTGTCGAAAAAATTTTAATCTCGTGACCAAACATTACGAGATATATTTTCAGGCATGTGTTTCATCCGAGCGACAACAACCTCAAGTTATAGATTATGAAATTCTCTCATCGGGACAACAATCTTCAGTGGCCCATTCCGGTAATGAAAACAATAATGCCGCGGAAGCAACATGGAGGCAAGATGCCGATATGAATATCGAGTATTTTTGGAATTTGCTGTTTTCTAGCATCGATGCTGACCCTCATGCTGAGTTCTTAAATTCGGTGTTGGCAGGGGATGATCAACTCTATGTTGATTCCGGCCACCATTGACTTTACACAGGTACAAGTAAAGATGTAAAAAAAAAATTTCTTTCATAGACATAGACAACTCATAGGGATGTTTTCGAGCAAGCTTTTTTTGACAAGAAATTTGACCCCCAAGTTGTAGTTGTTATACTTCAACAACATCAGATCTAATGAATAGAATGGATATATATATTCTCAACATTATTGGATATATATTTTTGATAAATTCGTTTGTAAAAAATTTTATTAATTAATTTATAATTTTTGTTATAAGCAATAATGTATGTGGGGTGAGAGAGGCTCGAACTCTCGACGTCAGGATAACTCTAAAGCTATGAGAC

>AiNAC56

ATGGCAGAAACTAGGGTTCTACCTGTTGGATATAGGTTTCGTCCAACAGAAGAGGAACTTTTAATTCACTATCTCAATAACAAGCATTTGGGAAATGATGCAGAGATTAAGAACACTATTTCCCAAGTTGATCTTTGTAACTTTGATCCTTGGGATTTGCCAGGTTCCTCTACCTTATTACTATTGTTTTATAAAAACTTTATAACTTTGATCTTTATAAAGTTTTCTAACAAAAATATGTAAAGTTTTAATCTTTAGTATATTAGTAATCTTAGATTGTTGTTTTTTATTTTGAGAGTCGTAGTTTATACTTGGTTGAATTATATTATAGAACAATCGAAGGTGAAATCGGATGATCAAGAATGGTTTTTCTTCAATGAATTGAAATACATGAAAAACAAGCGATGTAACAGAAAAACCAACATGGGATATTGGAAGATCACAGGAAAAGAGAGAATCATCAAAAGAACAGGGACAGACAGTGTCATAGGTACAAAAAGAACACTAGTTTTCTACAAGCGTCCACATAATGTCAAAACCAATTGGGTTCTTCATGAATATCATGCACTTCATCAAAAGGTAGGTTCTTGCCAGGTAAGAGATTGTATTGGAAATGAAATTAATTCGCCAATTTTTTTCCCTCTATTATTGGTTTTGTTGTAAACTGTGGAATCTTTGCCTCAATCTTTTGGATATGTAAATTCTGTGTGTGTTATACATAGTTATGAAAGTAGGTGGTGTTAGACATGAACGTGAAAATAATATTTTTTGAGCTATGAAGCGAAGAAAATGGACGATTTTTTTATTAAAAAAATTAAGAACACAAATCAAATGGTCTTATTTGTGTGAGAGAAAAAATCGGAGGGTTTGATTTATTTTTTTAAATTTTTTTTATTTTGAAAACAAAAATCGAATAGTTCGATTTTAATATTAAAATTTTTTTAATTGTCTAAAACACAAATTGGAGTTTGGTTTGTGTACTGTAAATTTTTTTAATTTTTTAAATATAAATCAGAAGTCCAATTTATTCTTGACATCACAACTGAGTAAAGCATTCATACATTTCATAATTGAATTAAAAAGTGAGAATCTTTTCACATCATTTTTTTCTGTCTTAAAGTCTTTTCTATTTCTAACGCTTTTAGTGTGTGTTTGGATTACAGTTTATAAATGAGAGTTTGTATAAAATTGATTTTGCAAACTTGATTTTGATAAAAAGTAAGTTTGTGTTAAAGTGATTTATGTTTGGTAATTTTAAAAAATAGAGTTCATATAAATAGGAATAACAAGAATTTTATAAATAATATAATAACATACATGAAGGATAAAGTTGGTAAAAGATAAATAAATGGTTAGTATCATGGCTAAAAAGCTCGTTAGGAAAACGCAGAAGCTAAACATAGTTGCTTCTTGTAAACGTTGGTTTTGGCAGCAGAATCACTTCTCCATTCATGGAAAAAAATTTTGCCAAACCAAAAGTTGAAGCTTTCAAGAAATCTAAACGTACTTCTTCCCTTCTAACGAGTTTTCCAAACACACCCCAAGAAAAATTTTATTTTATTTTTTTTTGCTTGGATGCTAAAAAAATGAAAGAAAAATTATACTATATTTGGTTCAAGAAAAAATAAGAGAAAAAAAATGAATGAAAAAAAATGAGTGAAAATTTTTATTTTTCTTTATTTGGATGCTAAAAAAATAAAAAGAAAGAAAATTTTCGTATAGGTTTCACTGATAAAAAGAGAAAATTTTTGNTAATTAAATAAAAAAAAATATTATATTTTATTTATTTTCCTTTCATTCATTCTTTTCTCACTCATTTTCTCTCTATTTTTTTAGATAAATTTTTTAGTTTACATGGTAGATGATGGGTTTTGTTCTTTGAGAGGGAAAGTTGCGAATTTAAAAATTAATCACTTCTAAAATTAAAAGATTGATAAATAATAAATTATTCTTGTTTGAGTTTTGTTTTGTAATGAAAATTATTTTTAAAAGTTATATTTAAAATATTAATTTATTAATTTTTTATTTTAGAGTATAAATTTTAGTTTAGTATTTTTTAAAAATTTTATTTTATTTTGTGTTATTATAAAATTAAATTTTTAGCTCCTTCAGTCTTTCCTAAAATTTAATCTAGTTCCGTCTTCAATATGTTCATATAACAATATTACCATTAAGTATTTATGTTCATAGAGAAATGCATTTTTTCCTATAACCTTTTTTTCTCTCCTGTACACATTTGACAAAGAAAATTTAAGAGGATTCATGCTGCATTTATTCATTGATTTAATGACATGGTATAATAATGATTCACGCGTTTCCATCTACCAAGAAGANNNNNNNNNNNNNNNNNNNNNNNNNNNNNNNNNNNNNNNNNNNNNNNNNNNNNNNNNNNNNNNNNNNNNNNNNNNNNNNNNNNNNNNNNNNNNNNNNNNNNNNNNNNNNNNNNNNNNNATTAACTTGGAAACAAAATTTTTCAATTTATTTTTTAAATAATGTGAAAAAATGTTATTTTTTGTCAAACAGTTTTTTAAGTAGAACTGAAAAGAAAATATATAAAAAAAAAGATGGAAAATGAATCCTCTAAATTTTTTCTCATTTATTGTTGTAAAGTAGAAGTTTTTATTATGTATTTTAAGTTGAACCAAGAAAAAACATAAAAAAAAAATGTTGAGCGGAATAAAATTATACCTAAAAATATCAATTGAAAAAATTTATTACCAAAAGAATTATAGTAAAAAATTATTTTTGTGACTTATCTTCAAATTAACTTGTTTGAATCCAATTTATTTCGTCATTTATCACACTTATGGTTTAATCTCTTGTTGATTCATTGCGTTGGGTGATACAATTTTTGTACGCAGAGCAACATCGTGTTGAGCCGTGTAACAATGAATGCTGAGAAAAGGGAAAAGAAGCTCAAGACAAAAGCAAGCAACATAATCGAAGAGGAAGTAAAATGTGAAGATGAACCATGCAGCGAAATTACTGGCTGTGTTACCCAAGCAACTACAGAAGATGCAATCCTTCCTGATAATGTGAGTGGAAACATCAATATACTCAAAAATATTATTTGTATATTAAAATTAATTATTATGTATTTATATAAATACATGTGTTATTTAATTTATTTTTAAAAATATATATTTTAATATATATTTTGTATTAGTAACTGNNNNNNNNNNNNNNNNNNNNNNNNNNNNNNNNNNNNNNNNNNNNNNNNNNNNNNNNNNNNCAATTTAATTGTCGATTTTAATGTATAGCTAATGTGATTGTTTGACCCTATATATATCTTCTCGAGAAAATTTGAATCTCGTCACCAAACATTACGAGATATATTTTCAGGCATGTGTTTCATCCGAGTGGCAACAACCTCAAGCTATGGACTATGAAATTCTCTCATCGGGACAACGATCTTCAGTGGCCTATTCCGGTAATGAAAACAATGCCGCGTTGCTTCCAATGGAAGCAACGTGGAGGCAAGATGCCGGTATGAATACCGAGTGTTTTTGGAATTCGCTGTTTTCTAGCATCGATGCTGACCCTCATGCCGAGTTCTTAAATTCAGTGTTGGCAGGGGATGATCAACTCTATGTTGATTCCGGCCACCATTGACTTTACACAGGTAGAAGTAAAGATGTAAAAAAATTTCTTTCATATACATAGACAACTCACAGGATGTTTTTGAGCAAGCTTTTTTTGACAAGAAATTTGACCCCGAAGTTGTAGTTGTTATACTTCAACAAAATCAGATCTAAAATGTTAATTGTGGGGTGAGAGAGGCTCGAACTCTCGACCTCAGGATAACTCTAAAGCTATGAGAC

>AdNAC6

ATGACAGAAACTACAATTTTACCTGTTGGATATAGGTTTCGTCCAACAGAAGAAGAACTTTTAGTTCACTATCTCAATAACAAGCATTTGAGAAATGATGCCGAGATCAAGAACACTGTTTCCCAAATTGATCTTTATAACTTTGATCCTTGGGATTTGCCAGGTTCTACCTTATTACTATTATTTTATAAAAACTTTTTAACTTTGATGTTTATAAAGTTTTTATTTTTAGTATATTAGTAGTCTTAGATTGTTGTTTTTTATTTTGAGAGTTGTAGTTCATATTTGGTTAAATTATATTATAGAACAATCGAAGGTGAAATGGGATGATCAAGAATGGTTTTTCTTCAATGAATTGAAACACATAAAAAACAAGCGGTGTAACGGAAAAACTAACGCCGGTTATTGGAAGATCACCGGAAAAGAGCGGATCGTCAAAAGAACAGGGATAGACAATGTGATAGGTACAAAAAGAACACTAGTTTTCTATAAGCGTCCACATAGTGTCAAACCAATTGGGTTCTTCATGAATATCATGCACTTCATCTAAAGGTAGGTTCTTGCTAGGTAAGAGATTGTATTGGAAATGAAATAAATTCTCCAAATTTTTTTTATCTCAGTTGGTTTTGTTGTAAATTGTGTAATCTTTTCACATCATTTTTTCTCTGTTTTAAGTCTTTTTTAAGTGTTGTAGGGTATGAAGAATAAGATGAAGATTGGATTCTCTTGAGTCCCAACTTCATTTTCTAGATAAAAGCACCTTGAATGTTGGTTTTTGGATAATTTTAATGGTTGATTTTTATTAATATATATTATATATATTTTTTATATTTAAAATTAACAGTTTAAATTATTAGAATATCAATATTTTAGAAAAATTAATCATTTTTTTTAAATTAAAAGATTGATAAATAATAAAAAGTTTTTCTTTGAGTTTTAGTTTGTAATAAGTATTTTTTTAAGTTATATTTAAAATATTATTTTATTATTTTACTAATTTTTCATAGGAAAACGCGTTTTTCCCTTTGACGAGGGTGTGGTACTAATTAATTTGAGTTGCATGAAAATAGGTATTTTTCTTTATTTTGATTAATCTTTTTTTTTCCCTTTTCTACGCATTTGACAAAGAAAAATTCAGAGGATTCATGCTGCATTTATCTATTGATTTAATGACATGGTATAATAATGATTCATGCATTTCCATTTACCAAGAACAGTAATTATTTCAAAAATAGACTTTTATTTACACATTAAAATCTTTAAAAATTAAAAAGATATTTTGATTTAATTTGAATAAATAATTTAATTATATTCTTTTGAAAAAATAATTTAAATAATAAGTTGTTATATTAAAAATAATTTATAAATAAATTATTTTGTGTTGCTTTCCAAACAAACTTTATATCTACTTATTCTTTTTTTTCTCTAATTTTCTTTAATTTTAAAATTTGTTGTTATAAAGTGTGATTTTTTTTATTAGGTATTTTTAAGTCAAGCCAAGAGAAAAAATAAAAAAAGATGTTGATTAGTATAAAATCACACTTGAAAATATCAATTAAGAAAATTTAAAAAAAATAATTTCAAAAAATATATAGTAAAAAATTATTTGTGATCACAAAAAAAAAAGAAATTGAGCTAAAATTATTTCTCCTTTTCAAATAACTTGTTTGAATTTATGTGACAATACAACATATTTTATATTGTATGAAAGAATTTTTTTTAATTTTATAGTACAGTTGATAGGCATGGTTTAATCTTATTGATTCATTGTCTTGAGTGGTACAATTTTTTAAAAGAAAAAGTCTAAGGGACCAGCGACTTTTGTATTTTCTAGCCAGCACTTAACCATTAAAATAAATAAAAGTAAATGATCTTCCACTATTAGATATAATTTCACACCATTAAAAACATTATTGATGGCCAATTAATAGTTATAAAACACCAAAATTACTGCCTCCTAGCATTCCTTTTTTTAAAATGTACACAAATCAGTTTGATGCACACAAATTTTGTTTGCAGAGCAACGTCGTGTTGAGCCGTGTAATAAAGAATGTTGAGAAAAGGGAAAAGAAGGTTAAGAGAAAAGCAAGCAACATAATCGAAGAGGAAGTAACATGTGAAGAAGATGAACCATGCAGCGAAATTACTGGCTATGTTACCGAAGCAACTACAGAAGATGCAATAATTCCTGATGTGAGTGGAAACATATTTTTGATACCTAAAAATATTTTTTATATACTAAAATTAGTTATTATATATTTGTATTTAAATATACATAAATACATGTTTTGTTTAATTTATTTTTAATTATATTTTAATATTATTTTTAAAAAATTCTTTAAAATTGATAGTTTTTGGTAAATTTGACTATTATTTGATCAGCAAAATATTAAATTGTTTTTAATAAATATATTTTATTAATTTATATGTGTAAATTTTATAAAATCTAAGTATAAACTATATTTTTTATGCAAATTTCTGTAAATATAAATACAAATTATTGTTAATAAAATATTAATTTAAAATGATAATATTTACTGATTAAATAACATTGTTTGTATTTTATATTAATTGTTGAATTTAATTACTGATTTTAGTATACGATACTACTTATTTTTAATCTCGTGACTAAACATTACGAGATATGTTTTCAGGCATGTGTTTCATCCGAACCGCAAGCACCTCAAGATGTTGACTATGAAATTCTTTCGTCGGAACAACAATCTTCGGTGGCCCATTCCGGTAATGGAAGCAACAATTCTCCGTTGCTTCCATTTGAAGGTATGTGGAAGCAAGATGTCGAGATGAACACGGAATGTTTTTGAAATTTGCTGTTTTCTAGCATCGATGCTGACCTTGATGCGGAGTTCTTGAATTCGGTGTTGGCAGGGGATGATTAACTCCATGTTGATTCCGGCCACCATTGACTTTACATAGGTACAAGTAAAGATGTAAAAAAATTCTCTTGCATGGACACGCACAACTTTCAAGCATGTTTTTGAGCAGCTTTATTTTGACAAGAAATTTGACACCCACGTTGTAGTTGACTAGTCGTTATACTCGAACAACATTAGATCTAATCTAATGAATAGAATGTATATATATTCTCAACCT

>AdNAC42

ATAACAATTTAATACATCAATTATTTTATCATCAAGACCAATAATAATAGAAATAATAACATAGAAATGAATACAAAGATTGAACTGCCACCAGGTTTCAGGTTTCATCCAACAGATGAAGAGCTCATAACTCACTACCTCTCTCAGAAGGTTGTTGCTAGCTGCTTCTATGCAACTGCCATTATTGGAGAGGCTGATTTCAACAAGTGTGAGCCTTGGGATTTACCTTGTCAGTATCTCAATTGTTATTTTCCCTTTCAGAAATAAATTGGGTTTTCATTTCATTCTCTTTTTATTATGTGCCTTTGAAATAAATATATTTTAAAAATATTCATTATTATTCAATCATTCTTTTTTTAATGTAAATTTTAATATTATAAATGAGATTAACTAGTTCTGAATATTTAAATAACATGTAAATGACTTTTTCATGAAAATTTTTTAAATGCCTATTTAAGTAGAAAATATGTATAACAAGTAACTTTTTGTTTATGGCATTATATTTTTTAGTTATAAACTACCGCCAATAGTTACTGGAAATTACTGTAAATTTCTTTCTGTGTCTTAAAAGATGATGTTTAAAATGCTGATAATCTATTTTGCAACGGTTTAAAATCGTCGTAAAATATTTAAAAATTTGTTGTTATGCAGCGTGTCTTTTATAGTGTATAATTAAATATATGTAGAAATATATTTTTTAATGATATTATTTTTTATTTAGCGTTGTTTGTTTGATTTATTTTGAGTTCAAAATTTGTGATTTTTATTTATTATTTTAATTATTTTGAATGAAGGGAGGGGCAAAATGGGAGAAAAAGAATGGTATTTTTTCTGTTTGAAGGACAAAAAATACCCAACAGGTGAAAGGACAAATAGAGCCACTGGTGCTGGGTACTGGAAGGCCACAGGAAAAGACAGAGAGATATACAATGCAAAAGCAAAAGCACTTATTGGGATGAAGAAAACACTTGTTTTCTACAAAGGAAGAGCTCCAAATGGTGAAAAGACAAATTGGGTCATGCATGAATATAGGTTGGAAGGCGATAATAAACCTTCTATATACAATCTTCCCAAAACAACCAAGGTAATTAGAGTTCAATCAGTATGCATCAGCCATGTCAGAAAAAAAATATATTATATTGTCATTTAATTTGATGCATGTATTTATAACTTTTGAATTAGTATTACATTAATATTTTTTGATGGAGTAGTTATTTTTGTCGATATAAACGTATTTGGACATAAAAAGTATGAATACACGTGTTGTATATNNNNNNNNNNNNNNNNNNNNNNNNNNNNNNNNNNNNNNNNNNNNNNNNNNNNNNNNNNNNNNNNNNNNNNNNNNNNNNNNNNNNNNNNNNNNNNNNNNNNNNNNNNNNNNNNNNNNNNNNNNNNNNNNNNNNNNNNNNNNNNNNNNNNNNNNNNNNNNNNNNNNNNNNNNNNNNNNNNNNNNNNNNNNNNNNNNNNNNNNNNNNNNNNNNNNNNNNNNNNNNNNNNNNNNNNNNNNNNNNNNNNNNNNNNNNTAAAATATATATTAAATATAATTTAAATAACACATCTATTTATACACAAAATATATGATAACTTTTGGTGTATACATATCCGGATACCTATATATTCATGCAACTTTCGTTCATTTTTACACTATTAATTTTATACATGCATATGTATGGCTTATTTTTTAGATAGTTAATAATTGAGTTAACATTTATATATACTGATATAATAATAATACATAACTGAATATCTATATAAAATAAAATATTTAACATTAACAATTCATGCAAATTAATTTGATTTGAATTTAGTATTTGTTAACATGGTTCCTTTATTTTCTATTTTTATTTTTACATTTCAGAAAGAGTGGGCTTTGTGCAGAGTTCTACACAAAAGTGAAAAGAAAGTAATGCATGTTCCACAACCACAGGGATTGGTTGAGTTCAGCTCTTATGAAAATAAGGAACTTCCCCAATTGATGGATTCTTCACAAGTAACATTCTTTTCATCAGACCCAAATAATCAAAGTGAGGATCCAAATCCAATCACACGTGATGATGATAATAATAATAATGATGACATCATAGTTGATAGCATTGAAACTCCTTTCTTGGAACAACAACCACCTTATTATTCTTCATCCTATGATTCTTCAGATTTAGACACCCTTAACCCTGCCACATGGGATATTTCCGAAAATGCCCCTACAAGTAATGCGTCTAAGGAGACGGACTTTGATGCTGACATGTTCTCTTTGATGTACAACAATAGAGAAGTGTTCCAAACATCATTTGAGAATCAGGAATATTATGCATATGATTCTATGGGACATGTGGACAATGGTTCCCTATGGAATTTTTAG

>AdNAC14

CTTCTTCTCATTCATTCATGCCCCCTTCCCCTCTTGGCCTAATCTCTTTCTCCACATGGCGACAAATTCCGTCAACCATAAAATCCCCCTCACTTCTCACCTCCCCAAAAATTTCAATCCTCTCTTCTTCCCTTTTCCTACACTTTAGTTTGCTACACCAAACTTCTCTCTCCCTTCTCTTATTCTTATTATTCCTCACCAAGCCTATTAACAAATATTTTCTTACACCAAATTATTAGCCTCTCAAAAATGGGATCACCCGAATCAAATTTGCCACCAGGTTTTAGGTTCCATCCAACGGATGAAGAACTCATTCTTCACTACCTTAGGAAGAAGGTAGCATCCATACCCTTACCTGTTTCCATCATCGCTGAGGTTGATATCTACAAATTGGATCCATGGGAATTACCAGGTATAATATATATAATAGCATTTTTTTTTGTTTCAATTCTTGTTAGTAAGTATTTTGTGTTTGTTATGAGGATAAGATTGTGCCATGTCGTGTCATAATATAATCAATGACGCAACGTATCATTGATTTTTATTGTATATTATAGCATACAGTGTTGTGATGTGGAAGCTTACTATTATTGGTTTGTTGTGGTTTCTTTATCACCAGCTAAGGCTGCGTTTGGTGAGAAAGAATGGTACTTCTTCAGTCCAAGAGACCGGAAGTACCCAAACGGTGCGAGGCCAAACAGGGCAGCTGCTTCAGGGTATTGGAAGGCTACGGGTACCGACAAGACCATCGTGGTGTCGCCGGCGGCCACAGTTACACGTAGAGTAGGCCAAGAGAGCAGCGTTGGTGTCAAGAAGGCTCTTGTTTTCTACAAAGGAAGGCCTCCAAAGGGTGTCAAAACCAATTGGATCATGCACGAATATCGTCTTGTAGACAACAACAGACCCATTAAGCTCAAAGATACCTCCATGAGAGTAAGTTTACCTAACTACCCTTCCTTCTGATAATTTTTTTTATATAAAGATTGAGAAACTCGAACTCGTAGCCTCTTAGGTGGGTATAGAGAAATTATGTCATTTGAACTTAATTCATTGGATCTTATAACCGTTAGATTGCTAATTCATGTGCTTTTGTGCAGTTGGATGACTGGGTTCTATGCCGGATTTATAAGAAGTCGAAATTCTCAGTATCTTCACCGGAGGAATCACCGTCGAGTGAAGTACAGGCTGCAGAAGAAAATGGTTTATTCAAGAACACCATTTTAAGGAGTCCAATTCCAACACCGTCGCCATCGCCACCGCCGCCGCTGCCGCAGCCACTGCTCTCTCAAAAATCTGTGTCCTTCTCAAACCTCTTAGATGCCATGGACTACTCCATGCTCAGCACCATCTTATCGGAGAACAATAACAACAGCACCCTTGATCAGCAACAATACTCGCAGATCAACACCAACCAATTGAACCATTCATCGAACATGGAGAACACTAGTAACAGCAACATGATGGTGATGAGGTCAAAGCGCCAGATAGAGGAGGAAACAACAACGGTGTTGCACCCATCAAAGAAGTTCCATCACCAACTTATGGGCTCTTCTTCTTGCAGCTTCCCTAATAACATTAACAACACAAACACTGCACAATACGAGAACCCGCAATGGAACTACCTTGTCAAGCAATCCTTCTTGAACCAGCACTTACTTCTCGCTCCTCATCTTCGATTTCAAGGATAG

>AdNAC34

ATGGATTCATGTCAACCCCAACTCCCACCGGGATTCAGGTTCCACCCAACCGACGAAGAACTCATCGTTCACTACCTCAAGAGAAAAGCTTCCTCTGCTCCTCTCCCCGTTGCCATCATCGCCGACGTTGATCTCTACAAGTTCGACCCATGGGAGCTTCCAAGTACCAATCCTAAATCATTCATCTCAACTTTCTTCTTTCCTTTTCATCTTTTAACATCTATCCATATACAGGTAAGGCCACGTTTGGGGAGCAAGAATGGTATTTCTTTAGTCCGAGGGATCGCAAGTATCCGAATGGGGCTCGCCCAAACAGAGCAGCTACGTCCGGATATTGGAAGGCCACCGGCACTGATAAGCCTATTATTGCGTCTGATGGCCAACACCGACTCGGCGTCAAGAAAGCTCTCGTTTTCTATGGCGGCAAGCCTCCTAAAGGGGTCAAAACCAATTGGATCATGCACGAATATAGACTCACTACTACTCATAACAATAATTCTATCTCATCAAAGTCTTTTCCTTCTCTTCCTTCTCATCTTCCTTCCTCCAATCAGAAGAATAATTCCTTGAGGGTATCTATCGTTCATTTCTTTCTTACACACATATTTTATTTGGATATCACTTATTTATCAACTTAATTTTTTCAGTTTAATAATTTAATAATATATTTTATTTTATATTTTTAAATATCAATAAATAATTATGATCACCAAAAATAAATTTTGATAGTTTTCTAACATTCCTCTTAAAGATATTTTGCTACAGTTTGATATTTTCGATTAAGCTCTTTTAAATAGTTAACTTTATTGTTTGTTTGGTTTGGTTTTATTATTATTATTATAGCTTGATGATTGGGTGTTGTGCCGAATATATGAAAAAAGCAACCGTGGCAATTTTGCAAGAACAGCGTTGATGGAGCACCATGATCATGATGATGATGATGACAATAATAAGGATCAGCTTTCCGCGGAAACAACGAGCATGATAGAAAACATGTCCACCATGAGTCAGAATTCCAAGCCCACACAACATTATGGACCATTGCTGGTTCAAAACGATGACAACTTCTTCAATGGAATCTTAGTCGCTGATCATCAAAATCATCAACATCACAACTTGCCAATGAAGAGGGCACTGGTGAATATGAATAATTCGCAGTTTTGGAATGAGACAAGCAAGAGGTTCCATTGTGATCTCAATAACAACACTAACACTACTGTTGCTAATAATGATGAGGATAACAGTTCCTTTGTTTCACTGCTTAGCCATAATCAGATTCCTCATCCTACTAACAATGCTTCTCTTCTTGGCCCTACTGTTGCTGATGGTGTTTTCAGGCAACACTTTCAACTTCAAACAATTAATTGGAACTTATAA

>AiNAC77

CATTTATCTGTACCACAAAGTCCCATACACACTGAATACTCATCTATCTGATCCTACCCCACCCACGCATCACGCATGGATTCATGTCAACCCCAACTCCCACCGGGATTCAGGTTCCACCCAACCGACGAAGAACTCATCGTTCACTACCTCAAGAGAAAAGCTTCCTCTGCTCCTCTCCCCGTCGCCATCATCGCCGACGTTGATCTCTACAAGTTCGACCCATGGGAGCTTCCAAGTACCAATCCTAAATCATTCATCTCACCTTTCTTCTTTCCTTTTCATCTTTTAACATCTATCCATATACAGGTAAGGCCACGTTTGGGGAGCAAGAATGGTATTTCTTTAGTCCGAGGGATCGCAAGTATCCGAATGGGGCTCGCCCAAACAGAGCAGCTACGTCCGGATATTGGAAGGCCACCGGCACTGATAAGCCTATTATTGCGTCTGATGGCCAACACCGACTCGGCGTCAAGAAAGCTCTCGTCTTCTATGGTGGCAAGCCTCCTAAAGGGGTTAAAACCAATTGGATCATGCACGAATATAGACTCACTACTACTCATAACAACAATTCTATCTCATCATCAAAGTCTTTTCCTTCTCTTCCTTCTCATCTTCCTTCCGCCAATAACAAGAAGAATTCCTTGAGGGTATCTATCATTCATTTCCTTCCTACAAACATATTTGACTTGGATATTTCAATTAAAAATGTTAAAAGATCATTAAAATTTATTATTTTTTATTATTATTTAGTTATTAATTTAATTTTTTTAAATTTTATTTTATATTTTTAAATAAAAGAGTGTTAGAAATAATTTTTGTGATTTGTAGCCATCAAATAGTCATCAATGATAATTTTAATGGTGTGAGATTGGTATGAGATTTCATCCAATAACTCACTTTTTCTTGCTGATTACATGCTGACAAGAATTTGAAAAAATTGCTGGCTCCTTAGATTTTTTTTTTAAATATTGATAACTAATTGATAACTATAAATAATAAATAATTCTGATAATTCTCCAGTATCTGTGTTAAAGATATTTTGCTATAACTAATAGCTAATGCTTTTCCCTTTTTCTTTCTTTTAAATAGTTAACTTTATTGTTTGTTTGGTTGGTTTTATTATTATTATTATAGCTTGATGATTGGGTGTTGTGCCGAATATATGAAAAAAGCAACCGTGGCAATTTTGCAAGAACAGCGTTGATGGAGCACCATGATCATGATGATGATGATGATGACAATAAGGATCAGCTTTCCGCGGAAACAACGAGTATGATAGAAAACATGTCCACGATGAGTAGTCAGAATTCCAAGCCCACACAACATTATGGACCATTGCTGGTTCAAAACGATGACAACTTCTTCGATGGAATCTTAGCTGCTGATCATCATAATCAACAACACAACTTGCCAATGAAGAGGACACTGGTGAATATGAATAATTCACAGTTTTGGAATGAGACAAACAAGAGGTTCCATTGTGATCTCAATAACAACACTAACATTGTTGCTAATAATGATGAGGATAACACTTCCTTTGTTTCACTGCTTAGCCATAATCAGATTCCTCATCATCCTACTAACAATGCTTCTCTTCTTGACCCTACTGTTGCTGATGGTGTTTTCAGGCAACACTTTCAACTTCAAGCAATTAATTGGAACTTATAG

>AiNAC55

GTGGTACTTAATGATGAAACTAATGCGACTTAATTGGGTTTGATTTCTAATTAAGTTGGTAGTTGTTCTCTTTTTAAGAGCTTCATAGCAACGCGATCCTCGCAAAACACACGCTCCTTTAAGAGTCACCACAAAAGACGGCTGAACCTTCGGGTACAAGATCCCTTCTCAGGAAGCATATAATCACTTCACTACTTCAACTGTGTTCTCTCTCTATCTATCTATCTCTTCATTATTAGGGTTTTCATTTCTTGATTATTACCAGCTTGCTTCTTCTATATATCCACCTCTTTCAACTCTCTACTCTTTCTTGAGGAGATGGAAGGTGAGAAGCTTGATGAGATCATGTTACCAGGTTTCAGGTTCCACCCAACTGATGAGGAGCTTGTGGGGTTCTACCTTAAGAGAAAGATTCAGCAAATGCCTCTGTCCATTGAGCTCATCAAGCAACTTGATATCTATAAATATGATCCTTGGGATCTTCCAAGTAAGTATATAGTGGCTATGATCTGAATGAACTTAATGATAAGGCAATTAATTAAAAAAGTACTGTATGTGTAGAAGTGGCAGGTACAGGAGAGAAAGAGTGGTATTTCTACTGTCCAAGAGACAGAAAATACAGGAACAGTGCAAGGCCAAATAGGGTAACTGGAGCTGGGTTCTGGAAAGCCACAGGGACTGACAGGCCTATATACTCCTCAGAGGGTTCAAAGTGCATTGGACTCAAGAAATCTTTGGTCTTCTACAAAGGCAGAGCTGCCAAAGGTGTTAAAACTGATTGGATGATGCATGAGTTTAGGCTCCCTTCTCTTGCTGACTCTTCATCCGACAAGACCACTATTCCTCCTAATGTCAGTATTTATTACTCTCTCAAACCCTTCTTAACAAGATCCATCTCACACTTTCTACCTAACTTCAATTTCAACATGCAGGACTCTTGGGCAATCTGCAGAATATTCAAGAAAACAAATGCTACAGCTCAAAGAGCACTCTCTCACTCTTGGGTTTCTACCTTACCTGAAACACCAACCACCACTACCAATGATACAGATCACATATTCCACTTTTGTTCATCCAACATGCCAACAATGATGGCAAAGAAAACTAGCTTCATGACCCAGTTTTGCACTAACTACACTAGTGACACACAAATCCAAGATGTTGCATCATCTTATAAACCACCCTTCATTAATATTAATCCATTGCTTTACAAACACTTTGATCATCATCATCATCAGTTACCACCCATTATTTCAAATGGAGATCTTATAAGCAACGACTGTTTAATACCCTCTTCTACTACTACTCCACTTGAAACATCCTCTAATAGTGCAAAACCTACTATGGATTTTTCTTCATTGTTGCTGAACATGTCATCTTCTGTTCTTGGAGATTTTGCTGGAAAGACATCATCGTCGTCCTCATCCCAAGAGGGTACAGCAGCAACAGCAACAACAATCACAAGTAGCTTCGGTGGTGGAATGCAGGAGCACTACCCAACAATACCATTACTGCGTCAGATGCATCAAGGGAACAACAACAACAACATTGGCATCAACAACAACAACGTGTCTGCTGGCGGTGAAGAACAAGAGTTGGAGAAAGTTGGATCCATTGTTGGGTTCCCATTCATGAACATTGGGGATGCATGGAAGTCAAATATGCTTTGGGATACTTCTTGTCCCTTGTGA

>-AdNAC49

TATATCTTGTAATGGTCCCCTTCCCCAAATGGATAACGGCAAGGCATTGCCAGATGACTAGGTATAACAATAACAAAGTGTTGTCTATCAATCAATTAAATCCATGCAACTTCACAAAACGACAAAGAGACCCTCACATAAAAGGAACACACATCTCATTGCAAACAGTTCAAACTCCTTAGCTTGTTCAATTTCACATAACAACAATGGAAGGAAGTAGTAAAAGTTGTGAACTACTACCACCAGGGTTTAGATTCCACCCAACAGATGAGGAGCTAATTGTGTATTACCTTTGTAACCAAGCAACATCAAAGCCCTGCCCTGCTTCCATCATCCCTGAAGTTGACATCTACAAATTTGATCCATGGGAATTGCCAGGGAAGGCTGAGTTTGGGGAGAAAGAATGGTACTTCTTTAGCCCAAGGGACAGGAAGTATCCCAATGGGGTTCGGCCGAACCGCGCAACGGTTTCTGGGTATTGGAAGGCCACAGGGACAGACAAGGCTATTTACAGCAAGTGTAAGCATGTTGGTGTCAAGAAGGCCTTGGTTTTCTACAAGGGTAGACCTCCAAAGGGGATCAAGACTGATTGGATTATGCACGAATATCGTCTTCTTCAACAATCTAATCACAACAGCAGGACCACTGGTTCTATGAGAGTAAGACTTCCAATCTTTTAACTATATGTTTCAATTGTTATGTTCAATTTGTAACTGATATTTCTGTCATCAGCTGGATGACTGCGTCTTGTGTAGGATATATAAGAAAAAACATGCTGCTAAAGCATTGGATCAAGGACAGGAATACCCAACAACGGTTCAAATTAATCTAAATGCATCAACCAACAATGATGATCAGAAGGAGTTGATGATGATGAAGAATCTTCCAAGGACTTGTTCCCTTACTTATCTTTTGGACATGAATTACTTTGGTCCAATCTCACAGCTATTGTCTGATGGATCCTACAACAACTCATCAACCTTTGAAATATTTCAACATAGCAATAGTGTTGACAACATTGGAATAGTGGATCCTCTTGTCAAAACTCAAATGGTTGAAATGGATGATAGCTATTATGCTCAAGATTCAGGCAAGTCCCAAGTGATGAAACAAGGGAATGATTTAAGAGGATATTACTAAGTAATTATTAACAAAAGAAAAAAGAAAAAATGTTATATTGTTTAGATTAGACATGATGAATTATGATAAGAGTCTTCTGTAAGATAATCATGAAGATTTTAAATATGTCAAATGTGTCCTATCACAGAAATGAAATTTTCAAATAGAATTTAGTATTCTAGTTCACGTTGACATTGTTCACCTTATCTGTACATAATAAGGACAAGACTATTACTGGTTCTTAATTGAATTTTCGGACTTATGAAAATAGTTTAAAAGATGCCTGGATCACGCCTTCTTAAGA

>AiNAC67

GTTCAAACTCCTTAGCTTGTTCATCAAACCAACTTCAATTTCACATAACAATATAATGGAAGGAAGTAGTAAAAGTTGTGAACTACTACCACCAGGGTTTAGATTCCACCCAACAGATGAGGAGCTAATTGTGTATTACCTTTGTAACCAAGCAACATCAAAGCCCTGCCCTGCTTCCATCATCCCTGAAGTTGACATCTACAAATTTGATCCATGGGAATTGCCAGGGAAGGCTGAGTTTGGGGAGAAAGAATGGTACTTCTTTAGCCCAAGGGAAAGGAAGTATCCCAATGGGGTTCGGCCTAACCGCGCAACAGTTTCTGGGTATTGGAAGGCCACAGGGACAGACAAGGCTATTTACAGCAAGTGTAAGCATGTTGGTGTCAAGAAGGCCTTGGTTTTCTACAGGGGTAGACCTCCAAAGGGGATCAAGACTGATTGGATCATGCACGAATATCGTCTTCTTCAACAATCTAATCACAACAGCAGGATCACTGGTTCTATGAGAGTAAGACTTCCAATCTTTTAACTATATGTTTCAATTGTTATGTTCAATTTGTAACTGATATTTGTGTCATCAGCTGGATGACTGCGTCTTGTGTAGGATATATAAGAAAAAACATGCTGCTAAAGCATTGGATCAAGGACAGGAATACCCAACAACAGTTCAAATTAATCTAAATGCATCAACCAACAATGATGATGAGAAGGAGTTGATGATGATGAAGAATCTTCCAAGGACTTGTTCCCTTACTTATCTTTTGGACATGAATTACTTTGGTCCAATCTCACAGCTATTGTCTGATGGATCCTACAACAACTCATCAACCTTTGAAATATTTCAACATAGCAATAGTGTTGACAACATTGGAATAGTGGATCCTCTTGTCAAAACTCAAATGGTTGAAATGGATGATAGCTATTATGCTCAAGATTCAGGCAAGTCCCAAGTGATGAAGCAAGGGAATGATTTAAGAGGATATTACTAA

>AdNAC61

CATGCACCACTTATGTTTGTATGTGTATATATATAACACATGCACAGTGTTTTCAATCTTACCACCACCTTAATCGATAATACCGGCAGCTGATTGATACAGTTTCCTAGTTTTCCTCGAAAGCGTTATAGTAGTTTCCATCTTCTTCATGGACAAGGATACTAGTTTGGAAATCCATCTCCCTCCTGGATTTAGATTCCACCCTTCTGATGAAGAGTTAATTGTTCACTATCTAAGAAACAAAGTCACTTCTTCACCACTTCCTGCCTCATTCATAGCAGAGATAGACCTCTACAAGTTCAATCCATGGGAGCTTCCAAGTATGTGTACAACTATATATCTGGTTTCTGTTTCCATCTTTTACTTCTCTTTCCCGTACAAAACATTTAGTCTTAATTGTATATGTTCATTTCATGCACATTTTGACTTATAAATGAAAGATAACTCCTCTTATATAACAATCACACTCTAATTTGTATTAATAAAAATTTTGATTTATTTAAATCAACATAATAAACTAATTGCAATTCCACTACTTTTCTTAAAAATTAACCAAATCATTGTCATAAGTCACTTCTCCCAAAAATTTAAATTCATACAACAAGATACATAAATGTTATATTTCTAACACACTACATCAAATAAGAACCTCTTTTTTTACTGTAACTTTGTGTGAAGTCTTTTTTTCTTATAGGCTTTCTTTTTTTTTTTTTCGTTAGCCTTATACCAGAATTCTTTTTTAGGATCAATAAGGATCGGATTCTATACTTTTGCATAATATCATGTTATAATATTATTTCTCTTAAAAGTTTAATCTAAAGGATATATAGTTATATTTTCAGCAAATATACCTAGGTAATCTAAGTCCCAACTCAAAAGAAAATAGATTTATGTAATTAAAGATGTTTTTATTTGGTGTGAAAAATAAAAAATTATTTTAATTCAACTAAATAGCTTTGTACTCTATGTTTTTTTTTTATAGGCAAAGCTTTGTTTGGGGAAGAAGAGTGGTATTTCTTTACTCCAAGAGAGAGGAAGTACCCAAATGGAGTGAGACCAAACAGAGCAGCTGGTGCAGGTTACTGGAAGGCCACTGGAACTGACAAACCAATTATCACGTCAGGTGGTATGAAGAGCATTGGAGTGAAGAAAGCCCTTGTCTTCTACAAGGGACGTCCCCCAAAGGGATCCAAAACTGATTGGATCATGCATGAGTATAGGTTGCATGATTCACTCCTCTCAAATTCTCACAAAAGAGGCTCCATGAGAGTAGGTCAATTGCATGCATGAGTTCTGAATTTCTATCTCTTTTTCATAAAACATGGTTAATGATAAACATTTTCTGGTTTATTTAATAGATTATTTTAAGAGAAAGTATAGGGAGCCAATGACCTAAGCGTACAATGTGTACAATGAAGGTTTAGAAAATATTAGAGATATGATTATTAGTGTTATATTGTCCTATCAGGTTACATTTTTGGGATGAGTGGTTTTAAGACATGGTATTAGAGTTCTAGATCCGGAAGGTCAAGAGTTCGAACTTTGATGAACCCAAAATGTATTCGGATGGTTATTCTGAATAGTATAAGTGATGTTCATTTTATTCATAAACCAAAGATTTAGCCCATTGTACACATTGTACGTTTAGGCCATTGGCTAAAGGAAAGAAGAATCAATTCATTTTAAAGATTTTTTTAATACATTAAAATATCTAATTTTTTACTTATGTAGTGTTAACTCTTTTTTGAAATAGCTATTTTGTTTCTCACATTTTAGATTTGTTAATAATTTAAGTATTTGGACTTAAATTGTGTTCGAATAAACTAAATTATTAATAAAACTAAAACATAAAAGATACTTACTAAGAATGCGGAAGCATAATATTTTTAATAATACTATGTGTATAAAAAAAACAGTGATTCGCATAAAATATATAGTAAAATTGAATTAAATTATACATATATTTATACAATTTTATTAGATAATCAATAATAGGTGTAATCAATTAGCAATTTTTTTTATTTTATTTTTTTTTTTGAAGAGAACATCAATTTAAATTATCACATCCACAAACTAATAGAAAAAAAAAACTAAAATCTAAAATTAAATAAAAACAAACATCTAAAATTATTGTAAATTCATTTTAAAAATTCAAAAAATTAAAAAATTCTTACTGAAAAACAATATTTGAAACACAATAAAAAGAAACATCCAAAATCTAACAAAATAAAATATTCAAAATTTAAAAATTTTTTTTAAAAAACATTAATTCAAATTATCATGAAAAATATTTAAAATTTAATGAAAAATATCTAAAATCTAAAAAAACTATCTATAAAATCAAATAATAACAAACTTTAAATTTAATGTAAAATTATTTTTAAAATTTATTTAAAAGATTCCAAAAATTATTTAAAAAAATTCAAAATTCATTATAAAAATACCATAATTATTTTAAAAATTTTAAAGTAAAAAAAATTCAAAACATAACAAAAAAAACATCCAAAATTTTACAAAACCAGGATATCTTAAAAATTCTAAAAGCGGACAAAACCAACTATAAAAAAAATTAGAAAAAGGAGATGGAGGGAGAGACTGCAGCGTTCTTGCACAAGGAGATGGAAGGGGTGGTGACGCAGTTGCTTTCTAGGTGGTCGTTGCAGTAGTCGAACTCCCGCGGACAGAACCATGACAACACGAGACGATGTGAGCGAGGGTGGTCTGGCTCGGCGACCCCACGTGTTAGACGTTAGGGAGTGCACAAATCGCGACTCTGCAAGAAGATCTTGGATGTCGATGCATGTATTGGCCGTAACCGGTGCGACGTGAGAGGATCCCGTTAGGAATGGCACCGCAACGCGATATGGAGGAGACGAATCATGACAGGAGAGAGGAGAAACAATGTCGCATCGGGAGGAGTCGTTTCTTATGAAAAGAGGTTTTTTTTAATGCAAAATTAAATTTTAGAAATTTTATAATCAGATTTTTTATCTACCAATACATGATGACTAATTTATTGGCTAATTTATAATATGTATCTACTATTTATTTTGTATTTTTGATACACTATATCCTTACAAACATTTGATGGAATATGTTTAAAGCCAGAATAATATCATGAAGTTTTTGTTTAATTGTTTTTCTTTTTGCTACAGCTTGATGAGTGGGTGCTATGCCGGGTGAGACAGAAAACAGGCAGCCCAAGAAGCACTTTGGAAGATCCAAGTGAACTGATTTATGAACCAACAAAAAAGATTCAACAAATGAATGATGAGAACTTCAATCCTGAACTAGTAAAAGCCTCCATTGTGCACAACGAATTTCCAATGTTACCTTATATTCTGGCTTCTAGGAGTACTTTGCCTAATTCCATTGGTGTGTCCTCAAGCACAGGCTTTGTTAGAAATTGTGATATGAAACAATATGGTTCAGTGCATGAAGACAACAACTTGAATGTAATAGGAGCACAGTTCTTAGCATCTGCAATGGAGGGCTTGTATAATAATCCTCTGAAGAGAAAATTCATTGAACAAGAAGAAAACCATTTGGAATATGCTCCCCCAAATAAGAAAATAAGCCTAGAACTTGGTGATGATGTTGATAATAGTGATGATAAGCCAAGTCTTGTGATGGATACAAACAAAGGCTACAATTTTGGCTTCTTTGATCAGTGGAATTCAATCATACAACCTCAAGAGCTTAACAGCTTAGCCTTCATGGGATATTCATGATCCATGAGTTTTATTATTTGACTACAATAATAAGCTATCTTGATCTGAACTCACTTTCTAAAATCATGTGGTACCCATTATTACCATTTCTCTTTTCATGATATGCTCTTTTTTCACTTGGTGTTGGTGCCTCATCAAGAAGTGAGTTGTACAGGGCATGCTTGCTTGTTCTGCATGGGAATGGATGGTAAGTGAAGTGGTTTATATGTAAATAAGCAAGAATAATGCAGTGTGGTAATTATATTCTTTTCAGCACATACATAAGAATGTACATAGTTGAAAATGTATATCATTACAGGATACTACTGTATCAATTGTGTGTGCATAGAGTAGAGATAGCTTGAAGAATAAAGTGCCTGCATAATGCAGTTTAATTGCTCATTGTATCGTGCCTTCACA

>AiNAC33

ATGGACAAGGATACTAGTTTGGAAATCCATCTCCCTCCTGGATTTAGATTCCACCCTTCTGATGAAGAGTTAATTGTTCACTATCTAAGAAACAAAGTCACTTCTTCACCACTTCCTGCCTCATTCATAGCAGAGATAGACCTCTACAAGTTCAATCCATGGGAGCTTCCAAGTATGTGTACAACTATATATCTGGTTTCTGTTTCCATCTTTTACTTCTCTTTCCCGTACAAAACATTTAGTCTTAATTGTATGTTCATTTCATGCACATTTTGACTTATAAATGAAAGATAACTCCTCTTAATAACAATCACACTCTAATTTGTATTAATAAAAATTTTGATTTATTTAAATCAACATAATAAAATAATTACAATTCTACTGCTTTTCTTAAAAATTAACCAAATCACTGTCATAAGCTAAGCCACCTCTCCCAAAAACTTAAATTCATACGAGGAGATACATGAATGTTATATTTCTAACACACTCCGCCAAGCAAAAACCTCTTTTTTTACTGTAACTTTGCGTGAAGGGAAGTACCCAAATGGAGTGAGACCAAACAGAGCAGCTGGTGCAGGTTACTGGAAGGCCACTGGAACTGACAAACCAATTATCACGTCATGTGGTATGAAGAGCATTGGAGTGAAGAAAGCCCTTGTCTTCTACAAGGGACGTCCCCCAAAGGGATCCAAAACTGATTGGATCATGCATGAGTATAGGTTGCATGATTCACTCCTCTCAAATTCTCACAAAAGAGGCTCCATGAGAGTAGGTCAACTGCATCCATGA

>AdNAC63

ATCTCTCAAACCTAATCATCAATAATTCAATCAAACCTTGGGAGTTTGCAACATCTTTCTCTCAGACCTATTCTTTTCTCTCTTCTCCCCTTCTTCCTAGCAAGCAGGCTCTCACTATATATTAATTTAATTTGTTGAAATTAAATAGAAAAAAGAATAATGAGCAACATAAGCTTGGTAGAGGCAAGGCTTCCACCAGGGTTCAGATTTCATCCAAAAGATGAAGAGCTTGTGTGTGATTACTTGATGAAGAAGTTCACGCACAATGAATCCCTTCTCATGATTGATGTCGACCTCAACAAGTGTGAGCCATGGGATATTCCTGGTTAGTCATTAATAATTTTCTTTCCATTTTATATCATGCATTATTCTCTAGCAATACATAATTATTCTTATTATTGTCATTCTTGTTGTTGTTGCCTCATCATATATATGATTGCATATTTCTTAAAAAGTGGCTATTTTGCGAAGAGATCTTTGTATGAGAATATTATGAACCATTAGATGATTTGATATGTTTGACTGAATATATTTAATTGATAAACTATCTAACGATTTTTAACATTATATTCACATAAAAATAGCTACTCTCTTAGAAAGAGCCATCAATTTTTATGCAAAACTCATAAATTAGTGTTTTGGATATGTAATTCATGTGGATTTAATTTTTTAAATTAACTAAAGAAAAAAAAAACTTAGTTGGAGGGCAATGGCATGGGTGCATATGAAATTATTATTATTTAATCCGAAATAAAATTCCCTATAGTTTTCTCTTTTTCTTGATGAGTCTCGTTAGGGACACCCATCACATGATACATACTAATAATGAAAGTGTTTGATCGGTTTGAGTTTGCTATTTTGGTTGGTAAAGTACAAAAGAGTTGCTACTTTCAACCTGTATTGTATTCTTGTGTGCTATAGTTTCAAAATTATGATGATAAGGATAAAGTTTTCTTTTTCTTTCTTTGGCTTTGAGAGTGAGGTTCATAATGATAGCAACTGGAATGGCTATAATAGATTCCAATTCTATAAGACCACTTATTATGTGTATGTTCTTTGATGGGGAAGTGATACCTGCCAACTGTGTGACTTAGATAATTGTAACACATCACAAGCTTTGATTATTGCTCAAATCAATAATTCATTAGAAAATAATTAACATTGAATTTATTAGATTAATTATATTCAACAATAATACAGTGCATTGAATATATATATATATATCTAACAGTATAAATTTTTAGAATAAATGTTTTTATGATATATATGATATAATAGAACTTTAATTTTATAATCATAGGGTTTATAATTTAATTTTTATTATTTTCATTCTTTAAAAATAAAAAATAATTTAAATATAATAAGATAAACAAAAAAAGTAACTGTTAAATTTAATTTTGACTAATTAATACATTCAAAAGTTAATGTATTTCGATGCAATTATGCTTGAATAAATCTGATCTTTTGATGTATTAATTTAATCAAAAGGACATTTTTTTATGAAATGGGAAAAAAAGAAATCACGTTAAGTGAATATTCCTCCATGTGATACCCGTTGACATAAGCCTCTATGCATATTCAAGCGGGTCATATAAGTCATAAGTTTCATTCAATCCAAGCCACTTTCACATAGATATAGATATATAGGTGGCTGCAACCAATCATATTCAATATTATGAAGATCGAACTGAAAATATAATTAATTAAGTTTACTTGCCCACATAGTAAAAGTATAATACATTTTCACATATGAAATAATTTAAAATATTTTATATATAAAAAATTAGATTTTTTTAAATATATTATCAATAATTAATACAATTATTTGATTATATATCTAATTTTAGAAGCTGGTTATTGATTATTGACATATTAGCTACTCAAATGTTTTTTATATATAAAGAAAACTTCACCATGATAGTGATTAATTTTCAGTTTTATACTTAAACATCATTTTGCTATAAAATTTTTTATGGATAACACGTACACACATGCTCAAACTCATACACCCGAGAATGAAAGGAATCTAATTATTTCTGGTTAGAAGCTTTGAATGGGAAGTTCAAAAATATCACACATATATATGGGGGGTAATATTGTGATTGCATCTAAACGGTATAGGCAAATGCATGTATATGTATGTATCATCTTCAAAATTTTTTAGGTGAAATGACGCATTGCCCTTGTCCTATTCTAAAGTGCTTGACAGTGATTTTCATTATTCATTATTCATTATTCATTCATATATAGTGCTAACATTAATTCATTTCAACCATACGCATGTTCATGAACATGCATGATTAACTTCCATATCATAATTTAATTAATTGTTTAAACTTTAAACTCTATGAAGTCTTACGATTATTGTAGCTGTATCCCACCTAGTCTATTATTGAAGTTTCAAAGCAAAAGGAAAGTGTAGTTAAAGTAATAAGACGGTTAAATTAATTTGCAACTTGTTTTTGACTTTAATAGATCCTTTTTTAATCCTTATTAATTGGATAGGTTGAAGTGAGTACTTTCCTAATTAAGAAGTATTACTTGTCTTAAGTAAAATTTTCATGTAGCATGACAAAAGAGATTTCCAGGGCATGATATGATGGGTCAACATTAATGCTTTGGTTTGTGATAGGCCATGTGTTCCTACTTTAATTTATACAAGTTTACATATTATTTTGGAGAAAAATATCAACTGTTTGATTGTGAAAGCACCATAATTAGTTTTTGTATGTTAACACACGCATGTCATCAATGAAAAGAAACTGACAAGGGTGGGCATAATCCGAAGAAACAATTCCAATCAATGCACGAAGCTACAGTTATATATGGGACCTATATAGATAGTAACTAATTTGCCCGCATTTATAATAATAATAATAATAATAATAATAATAATAATAATAATAATAATAATAATAATAATAATAATAATAATGTAGAAGTATGTAATTTTCTAAAGATAAATATGAAACAATGAAAGTCTTATTATAAGTCAATTGACTGAACGAGCGCGCGTGACTTTTAATATTTATTTTCTCCCTTCTGTTCCAGTCAATATTATCAATTCTAATAAGACAAAAGAAGAGAAAGTATAAGAGAATTTGATTAAAGGGTTATATTATAAGCAAGAGAGGATAATATTGTTAATGTCAAAAATTATTCAAGTCGCCACTCATATAGTTGAAATTAAAAAGAAAAAAAAATTCAATTCACACCTTATGTTCAAACGGGACATGTTCCTCAATCTTACCGAACATCATAGACTACGTTAAAGGTTTAATAACTTGGAAAAAAAAAGTTATTATGTTTGTTCTACTGATACGTAAATAGACTTTTATAGTTTAAAAATTTATATAACTAAAAAAATATTTGTAGTTAAAATTCTGTCAAAAATATAATTATCAGTTTATATTAAAAAAGTAATTTTATAACGTAATATTAAAATTTTTATAAAAAATTAGAGTTCAATTTTTGTCATCTTAAAAACAGATGTAAAAGATAAAAAGTTATATAATGACAAAATTTTTAATAGTGTTTGTTTAAAAAACACGCACTCAATAATAAATGTCAAATTAAATAAGTACCTTTTTATTTTTTTAATGATGTTACGCATCTAAATTTTTTTATTAACTAAATTTAATTATTAGTCTAATATTAACAAAAATAATTATAAATAGCATGATTAGAAGTTGGATTTTTGATTGATGGAAACAAAATTTGGTTTGGGCAGAAACAGCATGTGTGGGAGGGAAGGAGTGGTACTTCTACACACAGAGAGACAGAAAGTATGCAACGGGTCTGCGTACAAACAGAGCAACGGCATCAGGATATTGGAAGGCCACTGGCAAGGACAGGCCTATCCTTAGGAAGGGCAGCCTTGTTGGTATGCGAAAGACTCTTGTCTTCTATCAAGGTCGGGCTCCCAAAGGCCGTAAGACTGAGTGGGTCATGCATGAGTTTCGCGTTGAACCTCCTCTTCCTCCCCCCAACACTACTTCTTCTAAGGTATCCCCTCACTTCCANNNNNNNNNNNNNNNNNNNNNNNNNNNTCACTTTCACTCTCTCCTTTTTTTTTTTTACTCTTCATAACTACTTCTTCTTTTATTTTTCTAAATACAACATTTTTTATTTTATTTTTTTAATATTTTCCAAAAATGAATTCAATTTTTCTATCATTATCCGAATTAAGATTTAGCTTATCCAATAATAAGACACTTGAATCATTGTAATGTCTCCAGCTTAATATATATACATGCATTTTTTTATATTATATTCTATTTAAGTTATTAATTTTATCTAAAACTTTTCTCTGATATTATCCTATTATATATATAATATTATTTATTGTATTAATCAATTTAGATTGGTAATAATTTGTCTAATAATAAATTCTTAAATAAAATTTAAATCTGTATTAAATTATTCTTTGGCCTATCAAATTGAAAATACTTTTAAAATTTAAAAAAATTGTTAATATATTAGTTAATTGATTTGGTTAGTATATTAAGGTTAATTTTTAATTTAGTTTTTTATACAAAAATAAAATATTTAAATTATATGCATTTGTCTAAATTATTTTGTATGATATTATTTCATTAGCCATTAGGCGTATGCATTTTTGGTTAATTTTTTATCGGTTATTATTTATAACAGGTGGCTACTCCAAGAAAAATTTTATAATTATAATCGTGTGAAGATATTTTATTTTAATTATTGAATGATAAATTATAAAGTTTGATTTTTATATGTTATTAAAAATACTATCTTTATTTAAAATATAACTAAACAAATAAGTGATATTTTTTAAACAAGAATCATTATTTCATTAAAGTAATTTTCTTTATAATATATAACACATATAGGGTAATAGCAAACGGTATTATTATATTTTATTTTGTATTATTTTCTTTGCACAATTGCATGATTCATGGAAGAAACTTAGCTTGTGATAACGTAATTAATTTCTTTTCAAGGATTTTGACTTTTTGCAATCAGTTTTTGCTAGCTCTACCGAAACCAAATTTGTCTCACATGCTTATTGGCTAGAATGTGACTTCAGAAAGATATGGGGTAGCTGACCTAAGATTCTCTTTGAATTTTTTATTAAATGCCATATGTAAGCAATATGAAAGCACCTAATTCTCATAGATTTATTGATCTAAGAAATATTAATACTCCTTAAGGCTATTACTAAAATTTAGTATCTATACTATATGTTTATACTTTAATTTATCCCACCATTACACTCCATATTTCTTTCCTCCCATTTCCTCAGTGTGTTTTTTTTCCCCATACAGGAAGATTGGGTGTTGTGTAGGGTATTCTACAAGAACAGAGAAGTTGGTGGCAAACCTAATAGCATGGGAAGCTGTTATGATGACACGGGCTCTTCATCTCTTCCAGCATTAATGGATTCTTACATCAGCTTTGACCAACAACAACAACCTCAAACCCATCTTCATGCTGATGAGTATGAGCAAGTGCCCTGCTTCTCCATTTTCTCTCACACCCAAACAAGCCCTATTTTCAACCACATAATGGAGCCTAAGTTATTCCCTACCAACAACAACAACAATAATGCAACTTTATATGGTGGAGGAGGAACTACTACAACACCCAATTTGGGTTCTTGCTTAGACCCTTTTTCATGTGATAGGAAAGTATTGAAAGCTGTTTTGAGTCAGCTCACAAATATGGAAAGAAACATACCTAATAATAATAACAACAATACAAATAGTATAAAAGGGTCACCAAGTTTAGGAGAAGGTAGTTCTGAGAGTTACTTATCTGAGGTTGGCATGCCCAACTTGTGGAACAATTATTGA

>AiNAC32

ATCTCTTAAACCTAATCATCAATAATTCAATCAAACCTTGGGAGTTTGCAACATCTTTCTCTCAGACCTATTCTTTTCTCTCTTCTCCCCTTCTTCCTAGCAAGCAGGCTCTCACTATATATTAATTTAATTTGTTGAAATTAAATAGAAAAAAGAATAATGAGCAACATAAGCTTGGTAGAGGCAAGGCTTCCACCAGGGTTCAGATTTCATCCAAAAGATGAAGAGCTTGTGTGTGATTACTTGATGAAGAAGTTCACGCACAATGAATCCCTTCTCATGATTGATGTCGACCTCAACAAGTGTGAGCCATGGGATATTCCTGGTTAGTCACTAATAATTTCCTCTCCGTTTTATATCATGCATTATTCTCTCGCAATACATAATTATTCTTATTATTCTCATTCTTCTTGTTGTTGCCTCATCGTATATATGATTGCATATTTCTTAAAAAGTGCCTACTTTTGTGAAGACATCTTCACCCTTAGATAATTTGATATTTTTTTTACTGAATATATTTAATTGATGAACTATCTAACGATTTTTAACATTATCTTCACATAAAAATAGCTACTCTCTTAGAAAAAGCGATCAATCACTATGCAAAACTCATAAACATTTAGTGTTTTGTCTATGTATTTCATGTGGATTTAATTTTTTAAATTAATTAAAGAAGAAAAAAAAAACTTAGTTGGAGGGCAATGGCATGGGTGCATATGAAATTATTATTATTTAATCGGAAATAAAATTTCCTATAGTTTTCACTTTTTCTTGATGAGTCTCGTTAGGGACACCCATCACATGATACATACTAATAATGAAAGTGTTTGATCGGTTTGAGTTTGCTATTTTGGTTGGTAAAGTACAAAAGAGTTGTTACTTTCAACCTGTATTGTATTCTTGTGTGCTATAGTTTCAAAATTATGATGATAAGGATAAAGTTTTCTTTTTCTTTCTTTGGCTTTGAGAGTGAGGTTCATAATGATAGCAACTGGAATGGCTATAATAGATTCCAATTCTATAAGACCACTTATTATGTGTGTGTTCTTTGATGGGGAAATTAAATGATACCTGCCAACTGTGTGACTTAGATAATTGTAACACATCACAAGCTTTGATTATTGTTCAAATCAATAATTCATTAGAAAATAATTAACATTGAATTTATTTGATTAATTAATATATAATTTAATTTTTATTATTTTCATTCTTTAAAAATAAAAAATAATTTAAATATAATAAGATAAACAAAAAAATTATTTAAGATTTTAGTTTACTTTTTCTTTTTAATCCATGTAGCCAATAATTATACTTTCTCATTTTCAAAATAACTGTTAAATTTAATCTTGACTAAATTAATACATTCAAAAGTTAATGTATTTCGATGCAATTATGCTTGAATAAATTTTTTGATGTATTAATTTAATCAAAAGGACATTTTTTATGAAATGGGAAAGAAAAGAAATCACGTTAAGTGAATATTCCTTCATGTGATACCCGTTGACATAAGCCTCTATGCATATTCAATCCAAGCCACTTTCACATAGATATAGATATATAGGTGGCTGCAACCAATCATGTTCAATATTATGAAGATCTATAAATAAATTTAATATATTTATTTTCTTTCATTGAATAACAGAAATAATAAATTTTGTCCACCGAAGATTCCATACATATACACCATGATAGTGATTAATTTTCAGTTTTACACTTAAACATCATTTTGCTATAAAATTTTTTATGGATAACACGTACACACATGCTCAAACTCATACACCCGAGAATGAAAGGAATCTAATTATTTCTGGTTAGAAGCTTTGAATGGGAAGTTCAAAAATATCATACGTATATATGGGGGGTAATATTGTAATTGCATCTAAACGATATAGGCAAATGCATGTATATGTATGTATCATCTTCAAAAATTTTTAGGTGAAATGACGCATTGCCCTTGTCCTATTCTAAAGTGCTTAACAGTGATTTTCATTATTCATTATTCATTCATATAGTGCTAACATCAATTCATTTCAACCATACGCATGTTCATGAACATGCATGATTAACTTCCATATCATAATTTAATTAATTGTTTAAACTTTAAACTCTATGAAGTCTTACGATTATTGTAGCTGTATCCCACCTAGTCTATTATTGAAGTTTCAAAGCAAAAGGAAAGTGTAGTTAAAGTAATAAGACGGTTAAATTAATTTGCAACTTGTTTTTGACTTTAATAGATCCTTTTTTAATCCTTATTAATTGGATGGTTGAAGTGAGTACTTTCCTAATTAAGAAGTATTACTTGTCTTAAGTAAAATTTTCATGTAGCATGACAAAAGAGATTTCTAGGGCATGATATGATGGGTCAACATTAATGCTTTGGTTTGTGATAGGCCATGCGTTCCTACTTTAATTTATACAAGTTTACATATTATTTTGGAGAAAAATATCAGCTGTTTGATTGTGAAAGCACCATAATTAGTTTTTGTATGTTAACACACGCATGTCATCAATGAAAAGAAACTGACAAGGAAGTATGTATTTTTCTAAAGATAAATATGAAACAATGAAAGTCTTATTATAAGTCAATTGACTGAACGAGCGCGCGTGACTTTTAATATTTATTTCCCCCCTTCTGTTCCAGTATAGTCAATATTATCAATTCTAATAAGACAAAAGAAGAGAAAGTATAAGAGAATTTGATTAAAGGGTTATATTATAAGCAAGAGAGGATAATATTGTTAATGTCAAAAATTATTCAAGTCGTCACTCATATAGTTGAAATTATAAAAAAAAACAAATTCAATTCACACCTTATGTTGACCAAACGGGACATGTTCCTCAATCTTACCGAACATCATAGACTACGTTAAAGGTTTAATAACTTGGAGAAAAAAATTTATTATGTTTGTTCTACTGAGTACTGATACGTAAATAGACTTTTATAGTTTAAAAATTTACACAACTAAAAAAATATTTGTAATTAAAATTCTGTCAAAAATATAATTACCAGTTTATATTAAAAAGATAATTTTATAATATAATATTAAAATTTTTATAAAATAATTTAGATTAAATTTTTGTTATCTTAAAAACAAATTTAAAAGATAAAAGTTATATAATGACAATTTTTAATAGTATTTGTTTAAAAAACACGGCACTCAATAATACATGTCAACTTAAATAAGTACCTTTTATTTTTTTTCAATGATGTTACGTATGTAAATTTTTTTGTTATTAACTAAATTAGTCTAATATTAACAAAAATAATTATGATTAGAAGTTGGATTTTTGATTGATGGAAGCAAAATTTGGTTTGGTCAGAAACAGCATGTGTGGGAGGGAAGGAGTGGTACTTCTACACACAGAGAGACAGAAAGTATGCAACGGGGCTGCGTACAAACAGAGCAACGGCATCAGGATATTGGAAGGCCACTGGCAAGGACAGGCCTATCCTTAGGAAGGGCAGCCTTGTTGGTATGCGAAAGACTCTTGTCTTCTATCAAGGTCGGGCTCCCAAAGGCCGTAAGACTGAGTGGGTCATGCATGAGTTTCGCATTGAACCTCCTCTTCCTCCCCCCAACACTTCTTCTAAGGTATCCCCTCACTTCAACTCTCCCTTTTTTTACTCTTCATAACTTCTTCTTCTTTTATTTTCTAAATACAACATTTTCAATTTTATTTTTTTTATATTTTCCAAAAATGAATTCAATTTTTCTATCATTATCCAAATTAAGATTTAGCTTATCCAATAATAAGACACTTGAATCATTGNNNNNNNNNNNNNNNNNNNNNNNNNNNNNNNNNNNNNNNNNNNNNNNNNNNNNNNNNNNNNNNNNNNNNNNNNNNNNNNNNNNNNNNNNNNNNNNNNNNNNNNNNNNNNNNNNNNNNNNNNNNNNNNNNNNNNNNNNNNNNNNNNNNNNNNNNNNNNNNNNNNNNNNNNNNNNNNNNNNNNNNNNNNNNNNNNNNNNNNNNNNNNNNNNNNNNNNNNNNNNNNNNNNNNNNNNNNNNNNNNNNNNNNNNNNNNNNNNNNNNNNNNNNNNNNNNNNNNNNNNNNNNNNNNNNNNNNNNNNNNNNNNNNNNNNNNNNNNNNNNNNNNNNNNNNNNNNNNNNNNNNNNNNNNNNNNNNNNNNNNNNNNNNNNNNNNNNNNNNNNNNNNNNNNNNNNNNNNNNNNNNNNNNNNNNNNNNNNNNNNNNNNNNNNNNNNNNNNNNNNNNNNNNNNNNNNNNNNNNNNNNNNNNNNNNNNNNNNNNNNNNNNNNNNNNNNNNNNNNNNNNNNNNNNNNNNNNNNNNNNNNNNNNNNNNNNNNNNNNNNNNNNNNNNNNNNNNNNNNNNNNNNNNNNNNNNNNNNNNNNNNNNNNNNNNNNNNNNNNNNNNNNNNNNNNNNNNNNNNNNNNNNNNNNNNNNNNNNNNNNNNNNNNNNNNNNNNNNNNNNNNNNNNNNNNNNNNNNNNNNNNNNNNNNNNNNNNNNAATTGAGAATACTCTTAAAAATAAAAAAAATTAGTTAATATATTAGTTAATTGATTTGGTTAGTATATTAAGGTTAATTTTTAATTTAGTTTTTTATACAGAAATAAAATATTTAAATTATATGCATTTGTCTAAATCATTTTGTATTATATTATTTCATTAGTCGTATGCATTTTTGATCAATTTTTTATCGGCTACCATTTATAACAGGTGCCTACTCTAATGAAAATTTTATAATTGTCATCGTGTGAAGATATTTTATTTTAATCATTGAATGATAAAATGTAAAACTTGATTTTTATACGTTATTAAAAATATTATCTTTATTTAAAATATGACTAAACAAATAAGTGATATTTTTTAAACAAAAATCATTATTTCATTAGAGTAATTTCTTTTACAGAAATGTTTGGTAACCAAAGAAAATCAGTCAAAAATATTCATAACTTGTCTTATTTAACATTCATTAATTGTTGCGATAATTAATTAATACTAAATAAGACAAGTTCTGACTGATTTTTTTATCTACCTAACATTACCCTTTTCTTTATAATTAACACGAATAGAATAATAGCAAACGGTATTATTATATTTTATTTTGTATTATTTTCTTTGCACAATTGCATGATTCATGGAGGAAACTTAGCTTGTGATAACATAATTAATTTCTTTTCAAGAATTTTGACTTTTTGCAATCAGTTTTTGCTAGCTCTACCGAAACCAAATTTGTCTCACATGCTTATTGGCTAGAATGTGACTTCAGAAAGATATGGGGTAGCTGACCTAAGATTCTCTTTGAATTTTTTATTAAATGCCATATGTAAGCAATATGAAAGCACCTAATTCTCCTAGATTTATTGATCTAAGAAATATTAATACTCCTTAAGGCTATTACTAAAACTTAGTATCTATACTATATGTTTATACTTTAATTTATCCTACCATTACACTATATTTCTTTCCTCCCATTTCCTCAGTGTGTTTTTTTTTCCCATACAGGAAGATTGGGTGTTGTGTAGGGTGTTTTACAAGAACAGAGAAGTTGGTGGCAAACCTAATAGCATGGGAAGCTGCTATGATGACACAGGCTCTTCATCTCTTCCAGCATTAATGGATTCTTTCATCAGCTTTGACCAACAACAACAACCTCAAACCCATCTTCATGCTGATGAGTATGAGCAAGTGCCCTGCTTCTCCATTTTCTCTCACACCCAAACAAGCCCTATTTTCAACCACATAATGGAGCCTAAGTTATTCCCTACCAACAACAACAATAATAATGCAACTTTATATGGTGGAGGAGGAACTACTACAACATCCAATTTGGGTTCTTGCTTAGACCCTTTTTCATGTGATAGGAAAGTATTGAAAGCTGTTTTGAGTCAGCTCACAAATATGGAAAGAAACATACCTAATAATAATAACAACAATACAAATAGTATAAAAGGGTCACCAAGTTTAGGAGAAGGTAGTTCTGAGAGTTACTTATCTGAGGTTGGCATGCCCAACTTGTGGAACAATTATTGATGTGGTAGTCCTTATAGGCTTTGTAATTTGTTTATATACTTTCCCCCAAAATTAATTTATCCATGTTCTTCATATGCCACAAGGAAGTGGAAAAAATATATATGAAGAATCAATAGAATTTTGGAGGAGGGGGAAATTCTCATTGGAATTTTAAGTCATTGTGGTGGGTATCTAGGCTTCACCCATTGTGTGCATGTCCCAATATATTAAGAAAACGGGACTTGTAATTGAAACTCAAAAATATTTGTAGTTAATTTTAGTTAGTAGATGAATTGTTTCTTCGAAATTCAACCTTTTTTTTAATTAATATGAATTAATTTTTTAAAATTATTTTGTTTCTCTTNNNNNNNNNNNNNNNNNNNNNNNNNNNNNNNNNNNNNNNNNNNNNNNNNNNNNNNNNNNNNNNNNNNNNNNNNNNNNNNNNNNNNNNNNNNNNNNNNNNNNNNNNNNNNNNNNNNNNNNNNNNNNNNNNNNNNNNNNNNNNNNNNNNNNNNNNNNNNNNNNNNNNNNNNNNNNNNNNNNNNNNNNNNNNNNNNNNNNNNNNNNNNNNNNNNNNNNNNNNNNNNNNNNNNNNNNNNNNNNNNNNNNNNNNNNNNNNNNNNNNNNNNNNNNNNNNNNNNNNNNNNNNNNNNNNNNNNNNNNNNNNNNNNNNNNNNNNNNNNNNNNNNNNNNNNNNNNNNNNNNNNNNNNNNNNNNNNNNNNNNNNNNNNNNNNNNNNNNNNNNNNNNNNNNNNNNNNNNNNNNNNNNNNNNNNNNNNNNNNNNNNNNNNNNNNNNNNNNNNNNNNNNNNNNNNNNNNNNNNNNNNNNNNNNNNNNNNNNNNNNNNNNNNNNNNNNNNNNNNNNNNNNNNNNNNNNNNNNNNNNNNNNNNNNNNNNNNNNNNNNNNNNNNNNNNNNNNNNNNNNNNNNNNNNNNNNNNNNNNNNNNNNNNNNNNNNNNNNNNNNNNNNNNNNNNNNNNNNNNNNNNNNNNNNNNNNNNNNNNNNNNNNNNNNNNNNNNNNNNNNNNNNNNNNNNNNNNNNNNNNNNNNNNNNNNNNNNNNNNNNNNNNNNNNNNNNNNNNNNNNNNNNNNNNNNNNNNNNNNNNNNNNNNNNNNNNNNNNNNNNNNNNNNNNNNNNNNNNNNNNNNNNNNNNNNNNNNNNNNNNNNNNNNNNNNNNNNNNNNNNNNNNNNNNNNNNNNNNNNNNNNNNNNNNNNNNNNNNNNNNNNNNNNNNNNNNNNNNNNNNNNNNNNNNNNNNNNNNNNNNNNNNNNNNNNNNNNNNNNNNNNNNNNNNNNNNNNNNNNNNNNNNNNNNNNNNNNNNNNNNNNNNNNNNNNNNNNNNNNNNNNNNNNNNNNNNNNNNNNNNNNNNNNNNNNNNNNNNNNNNNNNNNNNNNNNNNNNNNNNNNNNNNNNNNNNNNNNNNNNNNNNNNNNNNNNNNNNNNNNNNNNNNNNNNNNNNNNNNNNNNNNNNNNNNNNNNNNNNNNNNNNNNNNNNNNNNNNNNNNNNNNNNNNNNNNNNNNNNNNNNNNNNNNNNNNNNNNNNNNNNNNNNNNNNNNNNNNNNNNNNNNNNNNNNNNNNNNNNNNNNNNNNNNNNNNNNNNNNNNNNNNNNNNNNNNNNNNNNNNNNNNNNNNNNNNNNNNNNNNNNNNNNNNNNNNNNNNNNNNNNNNNNNNNNNNNNNNNNNNNNNNNNNNNNNNNNNNNNNNNNNNNNNNNNNNNNNNNNNNNNNNNNNNNNNNNNNNNNNNNNNNNNNNNNNNNNNNNNNNNNNNNNNNNNNNNNNNNNNNNNNNNNNNNNNNNNNNNNNNNNNNNNNNNNNNNNNNNNNNNNNAATTGAGAATACTCTTAAAAATAAAAAAAATTAGTTAATATATTAGTTAATTGATTTGGTTAGTATATTAAGGTTAATTTTTAATTTAGTTTTTTATACAGAAATAAAATATTTAAATTATATGCATTTGTCTAAATCATTTTGTATTATATTATTTCATTAGTCGTATGCATTTTTGATCAATTTTTTATCGGCTACCATTTATAACAGGTGCCTACTCTAATGAAAATTTTATAATTGTCATCGTGTGAAGATATTTTATTTTAATCATTGAATGATAAAATGTAAAACTTGATTTTTATACGTTATTAAAAATATTATCTTTATTTAAAATATGACTAAACAAATAAGTGATATTTTTTAAACAAAAATCATTATTTCATTAGAGTAATTTCTTTTACAGAAATGTTTGGTAACCAAAGAAAATCAGTCAAAAATATTCATAACTTGTCTTATTTAACATTCATTAATTGTTGCGATAATTAATTAATACTAAATAAGACAAGTTCTGACTGATTTTTTTATCTACCTAACATTACCCTTTTCTTTATAATTAACACGAATAGAATAATAGCAAACGGTATTATTATATTTTATTTTGTATTATTTTCTTTGCACAATTGCATGATTCATGGAGGAAACTTAGCTTGTGATAACGTAATTAATTTCTTTTCAAGGATTTTGACTTTTTGCAATCAATTTTTGCTAGCTCTACCGAAACCAAATTTGTCTCACATGCTTATTGGCTAGAATGTGACTTCAGAAAGATATGGGGTAGCTGACCTAAGATTCTCTTTGAATTTTTTATTAAATGCCATATGTAAGCAATATGAAAGCACCTAATTCTCCTAGATTTATTGATCTAAGAAATATTAATACTCCTTAAGGCTATTACTAAAACTTAGTATCTATACTATATGTTTATACTTTAATTTATCCTACCATTACACTATATTTCTTTCCTCCCATTTCCTCAGTGTGTTTTTTTTTCCCATACAGGAAGATTGGGTGTTGTGTAGGGTGTTTTACAAGAACAGAGAAGTTGGTGGCAAACCTAATAGCATGGGAAGCTGCTATGATGACACAGGCTCTTCATCTCTTCCAGCATTAATGGATTCTTTCATCAGCTTTGACCAACAACAACAACCTCAAACCCATCTTCATGCTGATGAGTATGAGCAAGTGCCCTGCTTCTCCATTTTCTCTCACACCCAAACAAGCCCTATTTTCAACCACATAATGGAGCCTAAGTTATTCCCTACCAACAACAACAATAATAATGCAACTTTATATGGTGGAGGAGGAACTACTACAACATCCAATTTGGGTTCTTGCTTAGACCCTTTTTCATGTGATAGGAAAGTATTGAAAGCTGTTTTCAGTCAGCTCACAAATATGGAAAGAAACATACCTAATAATAATAACAACAATACAAATAGTATAAAAGGGTCACCAAGTTTAGGAGAAGGTAGTTCTGAGAGTTACTTATCTGAGGTTGGCATGCCCAACTTGTGGAACAATTATTGATGTGGTAGTCCTTAT**AG**GCTTTGTAAGCTTTGTAAGCTTTGTAA

>AdNAC73

ATGTCACCAGTTGGATTACCACCTGGGTTTAGGTTTCATCCAACAGATGAAGAGCTTGTTAACTATTATCTAAAGAGGAAGATCAATGGCCAAGAAATTGAACTTGATATCATTCCTGAGGTTGATCTCTACAAATGTGAACCATGGGAATTAGCAGGTTTCTTTTCTTTTTTTTTTCCTTCTTTCTTATAATAATTAACTTCCTTTAGTTTTCTTAAGTCTTGTTTTTTTCTTCTTTGGTTTAAAACAATTTATGCATTCAAGTTTTAAACTTCTTATATCATTTCCAAGTCATTATATCTTAAGTTCACTAGTCAACTAAGTTAATTCGAAACTAATTATACATTCATGTTTTTAATTATTTCTTCCTTTGTTTTATTTTCCCTTTTTTTCTCAGACGATAATATTTGAACATCACTAGAATTCAACTAATAATATTATTCTAAATGACTCAGTACATCATTCACTTTTCTTTTTATTCCCAGTACATTTACATTTCTAAATTTCAAACATAAAAGAATTTTCATTTTTGATATCTTGCCAAAATAATAATAATAATAATATATAATACTGATTTTCAAATTCCTGTATACATTTTGAACTGTAATGAATCAATTTATGTGTTTTACTTTGTTATCCTCAAATATATTCATGAGATACATTTTGACCTAGCATTCTAGCTACAGCTCTTCAATGTCAAGAACCATTTTATTTTTCAAAAGCCTAAACAATTATTTAATACGCATGCAGATGAATGATTGTATATCTAAAAGTCGAACATTTTGCTTGTTTCTAGAAAAATCATTTTTGCCGAGTAGAGATCCAGAGTGGTATTTCTTTGGACCAAGGGACAGAAAATACCCTAACGGATTCAGAACAAATAGAGCAACACGAGCAGGGTACTGGAAATCAACAGGTAAAGACAGGAGAGTTTCAAGCCAAAGCAGACCAATTGGTATGAAGAAGACTTTGGTTTATTATAGAGGAAGGGCTCCTCAGGGAATCAGAACTGATTGGGTTATGCACGAATATCGTCTAGATGACAAGGACTCTGAAGACACCACCGGTTTACAGGTATTAAGTCTAATTACACCTGCTTTTGGATATTCATAAATGGGCACCATAAGGTTTATTTAGTTGCACCAAATGCTAAGAGTGAAAAATGTTATATGAGAGTTGGTTTCATAAGCTTGCAACCATTTACAATAACTATGATAACAAAAATACTATTCTTATACTAAAATTAATTATTAAAATTATCTATCAATATATTTGTGTATAAATATATTTGTAGTTTAATTTATTTTTTATATATATTTATATTCTAACATATATTTTATATTAATAACTAACTTTAGTTACTGATTTTAGTATACACATAACATAATCGATATGATAATTTTTATAGGTAGATTCTAGGGAGTTCAATTATATACTTAGGATAAATTACATTTAACTTTATTAATTGCTTTAGTCTAGCTTAGGTTATATTAAGGTTATATTAACACAACAACCTTGATTTTTCCTGTCAAGAAGCCTACTATGCTATATGTATAGGAAAATTAATATATATTTTACAGATAAAAAAATAACCATAATATTACATGCTTAAGTTATTTTAATAATTTATTAGCATAATAAAAATTATTTAAAAAATAAAAAGAAGAAAATTTTTTTATTAGATTTGATTTAAAAAAAATATTTACCTAGTATTTCTTTAAAAATAATATTTATAAAATCGTCTAAGTTTATGAGAAGTCAGTGAAACTTATTCTTATTATTTTGTTACAGTTTTAATAACTTTTCATAATAAAGTAGCAGACAGACTTAGTTGTTACTTAGACTTGTATAGTTATTACTGACTTACTGTATCATATCATAGGTTGAACATAAATTTACAAGTCAGTACAGTCTTTTATATAGTTACTTTTACTATTTAAAAAAACGGTTATTCACTACTTAATTAAAATTATCATTTCTCTGTGGCTCAGTTTTAGGGGATATATAATTATTAAGCTATGTTTATCATTCTGAGGTGGTGAATTAATTAAAGACAATTGAAGGGAATAATGATGATGTCCTAACAATTTCCGGGTACAAAATAATTAATTAAGGTGTGTCTCAATTTTATTAGAACTCGGAGAAGCTAAGAAAGGGAATGGTAGTTGAAGAAGTGGGGATGAATTAAAGACTTGAATATGATGATGATGATGACAGAATTGAAAAACCTTTACATGTCACATCGTACAAAAGTAAATGTTTTTGTTAGCTTCAAAACTGTAGTCTCTGGTGGCCCACTTCACCCTTTTCCTACTTTCAGAAACTCAAACACCCTCTACTCTCTTTTTCTTGTGTGTGTATCCACACTTGTCATCACCATATATTTGACCCAAAAAGCATCAAGTTCCATGCTTAAACCAATTACAAAAGCTTTTTTCTTTCATTATATTATTTTCATTTGCAAAAAACTTTATAAATTTCGATAATGATGACAAATAACCAAACTTATACTCTAATGATATATAAAAGTTTATTTTTTATCAATTGCATTAATCAATAATCATCTTTAATTTGGTCCGTCTTTTATACTAAAGACAATATTTTTAAAACAAAAATTTAAAAGCAGTAATTTTCATTCTTATTGTGATAATTAATATTACTTCTATATATTTTTATTTTTTTTATAAAAATATATTAAGCATTCACGTATGAATATCATTAATATATAATTAAACAGTAGAATTATTGAAAAAAAAATATTTAACTTAAATTAATCGAATAATCAGTTCACTTTTGTTTAAATATTAAAAGTTCGATTCATATTCAATGTGTATAAAAATTTATTGGCCAAATTAAGAGTCATAGACACTAGAAGTATAAGATGTTAAATTAGGACGTGTGTAACAAATTGCATGCATGGCTTGCATGGGAAGCCATAACTACTTTTATATAATAGTATAGTAATTCTAATTCTTTCTTCTTTCTCTCTCTATCACACTTTCTCTCACATGTTACTTTCTCTATAAACGTATTGCTGACATATTCGTTGCTTGTGTTACTCTAATACTTCTACATGACAAAGTTCCTCTATACTATTGCGCTTCACATTGTCCTCTATCTCTTTTTTATTTTTTGAGTATTAATTAATGTAATGAGACAAAAGTCCTATACTCCGATCCTTAGGATCCAATAATAATTAACTAAATATTTTAAACTATTCCTTTGATGTCTTTGTCAATTGTCGTTGTTATCTTTCTTTTCATCATCATCCCTGTGTGTATGTTAATGTTATCCTCATCTAGAATTATTAGATTCTATTTCTCTAAGCCATGAATGAAGGGTTGTTTTGGCACCGCATAAAGCAACTCCTTCTCCATCACTTTCTCTCTGTGTCGGTCACTTTTTACTTTATTCCTCCAAACCCCAAAATACATCAATTGATACTCATAGTGTACTATTACACCAACATAGAAGCTGAATAAGAAGAAGAAGAGGAAGAAGAATAAGTAACAATAATAATATTAAATAGAAAAGGTGAGGGGATATGAGGGAAAAAAAATAATCAATAGATTCCAATCAATAATGAATGCATTACTTTATTGTTAGGTTAAGCTGTAATATAATTTAATAAAGAAAATAATTAAAGATCAGAGAACTATTAGACACAAACACTTTTCATAAATATTAATCAATATTATAGAGTAATATTATATTTTTATACCATTAAAATAAAAAATTTTAAAATTTAATCAATTTTACATTTTTTAATATTATATATAAAAATATATTTGTTAACTAAATATTAACTCAAAATAATAAATTTTACTCAACCTATTATAATATATATATATGTTCCTCACTTTTAGTAGGCTATGGTGAGTGTCTTCAAACTTGATTACAAAATGGACAAGACATCTAGTATTCTTTGAATGTTCATCTCATGGATTTGTGTGGAGATTGTACTTTACGGTTTCTACGTACACATTTGTGCAGATCTGTTAGAGCCACAATTTTTGACTAAACTGTTTTTGACCATTGCAAACAATATAAGGCCAAAGAAGACCAAAAAGCAATTTGGGAAGAAATTAAAATATTTTAAATTATTGGATCATCATCACCCTACTCAACAATCTTTGGTGTTATTAATACTTTATAATAAATTATATTTATATTTAATTATTGGTGACTTATTTTATTATTGCAGGATACTTATGCTTTGTGCCGTGTGTTCAAGAAAAATGGAATATGTACGGATGTTGAAGAGCAAGTAGGGCATTGTAGTAACATGTCTTCACTAA

>AiNAC58

ATGTCACCAGTTGGATTACCACCTGGGTTTAGGTTTCATCCAACAGATGAAGAGCTTGTTAACTATTATCTAAAGAGGAAGATCAATGGCCAAGAAATTGAACTTGATATCATTCCTGAGGTTGATCTCTACAAATGTGAACCATGGGAATTAGCAGGTTTCTTTTCTTTTCTTTTTTTTCCTTCTTTCTTATAATAATTAACTTCCTTTAGTTTTCTTAAGTCTTGTTTTTTTCTTTTTCGGTTTAAAACAAGTTACGCATTCAGTTTTAAACTTCTTATGTCATTTCCAAATCATTATGTCTTAAGTTCACTAGTCAACTAAATTAATTCGAAACTAATTATACATTCATGTTTTTAATTATTTCTTCCTTTGTTTTTTTTTTCCCTTTTTTCTCTCAAACGATAATATTATTCTAAATGACTCAGTACATCATTCACTTTTCTTTTTATTCCCTGTACATTTACATTTCTAAATTTCAAACATAAAAGAATTTTCATTTTTGATATCTTGCCAAAATAATAATAATAATAATAATAATAATAATAATAATAATAATAATAATAATATATAATTCTCAATTAAACTNNNNNNNNNNNNNNNNNNNNNNNNNNNNNNNNNNNNNNNNNNNNNNNNNNNNNNNNNNNNNNNNNNNNNNNNNNNNNNNNNNNNNNNNNNNNNACTCTATAGCTGTTGTGGATCATGTAGCCATCTTTTTATATATTGATTTTCAAATTCCTGTATACATTTTGAAGTGTTATGAATGAATTCATGTTTTACTTTGTTATCCTCAAATATATTCAAGCAATACATAGTACTTGATCCATGCAAAATTCATGAGATACATTTTGACCTAGAATTCTAGCAATGTCAAGAAGCATTTTATTTTTCAAAAGCCTAAACAATTTTTTGATACGCATGCAGATGAATGATTATATATCTAAAAGTCGAACATTTTGCTTGTTTCTAGAAAAATCATTTTTGCCGAGTAGAGATCCAGAGTGGTATTTCTTTGGACCAAGGGACAGAAAATACCCTAACGGATTTAGAACAAATAGAGCAACACGAGCAGGGTACTGGAAATCAACAGGTAAAGACAGGAGAGTTTCAAGCCAAAGCAGACCAATTGGTATGAAGAAGACTTTGGTTTATTATAGAGGAAGGGCTCCTCAAGGAATCAGAACTGATTGGGTTATGCACGAATATCGTCTCGATGACAAGGACTCTGAAGACACCACCGGTTTACAGGTATTATCCTTGCGTCCTCATTCATTTTATCATTTTTACAACTCTAATTACACCTACTTTTGGATTTAGTTGCACCAAATGCTATCCTATTAAGAGTGAAAAATGTTATATGAGAGTTGGTTTCATAAGCTTTCAACCATTTACAATAAGCTATATTGGTATATTATAACAAAAATGCTATTTGTACATCAAAATTAGCTACTAAAATCATCTACTAATGTATTTTTGTATAAATACATGTGCAGTTTAGTTTATTTTCGATATGTATTTGTATTCTAATATATATTTTATATTGGTGACTGATTTTAATGTACACATAATATGATTGATATGATAATTTTTATACGTAAATGCTAGGGAGATACATGTCCAGAAGTTCAATTATATACTTAGGATAAATTACATTTATCTTTAATTGCTTTTAGTCTAGCTTAGGTTATATTAAGGTTAATTATATCAACATAACAACCTTGATTTTTCATGTCAAGAAGCCTACTACGCTATATGTATAGGGAAAATAATATATATTTTACAGATAAAAAAAATAACCATAATATTACATGATTAAGTCATTTTAATAATTAAGTTGATTTAGTAATTTATTAACATAATAAAAATCAATTAAAAAAATAAAAAGAGACGTATAAGAAGAAGAAAAAATATTTACCTAGTATTTCTTTAAAAATAATATATATAAAATCGTCTAAGTTTATGAGAAGTCAGTGAAACTTATTCTTATTATTTTGTTACAGTTTTAATAACTTTTCATAATAAAGTAGCAGACAGACTTAGTTGTTAGTTAGACTTGTATAGTTAATTACTGTATCATATCATAGGTTGAAACATAAATTTACAAGTCAGTACAGTCTTTTATATAGTTACTTTTACTATTTTTAAAAAACGGTTATGGACTACTTAATTAAAATTATAATTTCTCTGTGTCTCAGTTTTAGGGGATATATAATTATTAACCTATGTTTATCATTCTGAGGTGGTGAATTAATTAAAGACAATTGAAGGGAATAATGATGATGTCCTAACAATTTCCGGGTACAAAATAATTAATTAAGGTGTGTCTCAATTTTATTAGACCTAGGAGAAGCTAAGAAAGGGAATGGTAGTGAAGTGGGGATGAATTAAAGACTTGAATATGATGATGATGATGAATAATAGAATTGAAAAACCTTTACATGTCACATCCTACAAAAGTAAATGTTTCTGTTAGCTTCAAAACTGTAGTCTCTGGTGGTGGCCCACTTGACCCTTTTCCTACTTTCAGAAACTCAAACACCCTCTACTCTCTTTTTCTTGTGTGTGTATCCACACTTGTCATCACCATATATTTGACCCAAAAAGCATCAAGTTCCATGCTTAAGCCAATTACAAAAGCTTTTTTCTTTCATTATATTATTTCCATTTGCCAAAAAACTTTATAAATTTCCATAATGATGACAAATAACCAAACTTATATTCTAATGATATATATAAGTTTATTTTTTATGAATTGCACGAATCATCTTTAATTTGGCCCATCTTTTATACTAAAGACTAATATTTTTAAAACAAAAACTTAAAAGGAGTAAATTTTCATTCTTATTGTGATAATTAATATNNGTAATAAAATTATTGAAAAAAAAATATTTAACTTAAATTAATCGAATAATTAGTTCACTTTTGTTTAAATATTAAAAGTCCGATTCATATTCAATGTATATAAAAATTCATTGGCCAAATTAAGAGTCACAGACACTAGAAGTATAAGATGTTAAATTAGGACGTGTGTAAACAAATTGCATGCATGGCTTGCATGGGAAGCCATAACTACTTTTATATAATAGTATAGTAATTCTAATTCTATCACACTTTCTCTCACATGTTACTTTCTCTATAAACGTATTGCTGACATATTCGTTGCTTGTGTTACTCTAATACTTGTACATGACAAAGTTCCTCTATACTATTGCGCTTCACATTGTCCTCTATCTCTTTTTTATTTTTTGAGTATTAATTATTGTAATGAGACAAAAGTCCTATACTCCGACCCTTAGGATCCAATAATTAACTAAATATTTTAAACTATTATATTGCTTTGGTGTCTTTGTCAATTGTCGTTGTTATCTTTCTTTTCATCATCATCCCTGTGTGTATGTTAATGTTATCCTCATCTAGAATTATTAGATTCTATTTCTCTTAGCCATGAATGAAGGGTTGTTTTGGCACCGCATAAAGCAACTCCTTCTCCATCACTTTCTCTCTGTGTCGGTCACTTTTTACTTTATTCCTCCAAACCCCAAAATACACCAATTGATACTCATAGTGTACTATTACACCAACATAGAAGCTGAATAAGAAGAAGAAGAGGAAGAAGAATAATATTAAATAGAAAAGGTGAGGGGATATGAGGAAAAAAAATTAATCAATAGATTCCAATCAATAATGAATGCATTACTTTATTATTAGGTTAAGCTGGAATATAATTTAATAAAGAAAATAATTAAAGANNNNNNNNNNNNNNNNNNNNNNNNNNNNNNNNNNNNNNNNNNNNNNNNNNNNNNNNNNNNNNNNNNNNNNNNNNNNNNNNNNNNNNNNNNNNNNNNNNNNNNNNNNNNNNNNNNNNNNNNNNNNNNNNNNNNNNNNNNNNNNNNNNNNNNNNNNNNNNNNNNNNNNNNNNNNNNNNNNNNNNNNNNNNNNNNNNNNNNNNNNNNNNNNNNNNNNNNNNNNNNNNNNNNNNNNNNNNNNNNNNNNNNNNNNNNNNNNNNNNNNNNNNNNNNNNNNNNNNNNNNNNNNNNNNNNNNNNNNNNNNNNNNNNNNNNNNNNNNNNNNNNNNNNNNNNNNNNNNNNNNNNNNNNNNNNNNNNNNNNNNNNNNNNNNNNNNNNNNNNNNNNNNNNNNNNNNNNNNNNNNNNNNNNNNNNNNNNNNNNNNNNNNNNNNNNNNNNNNNNNNNNNNNNNNNNNNNNNNNNNNNNNNNNNNNNNNNNNNNNNNNNNNNNNNNNNNNNNNNNNNNNNNNNNNNNNNNNNNNNNNNNNNNNNNNNNNNNNNNNNNNNNNNNNNNNNNNNNNNNNNNNNNNNNNNNNNNNNNNNNNNNNNNNNNNNNNNNNNNNNNNNNNNNNNNNNNNNNNNNNNNNNNNNNNNNNNNNNNNNNNNNNNNNNNNNNNNNNNNNNNNNNNNNNNNNNNNNNNNNNNNNNNNNNNNNNNNNNNNNNNNNNNNNNNNNNNNNNNNNNNNNNNNNNNNNNNNNNNNNNNNNNNNNNNNNNNNNNNAGTAATTTTTAACCGTTGATCTTAATTATATATATTATATATATTTTTTATAATTAAAATTAACGATTAAAAATCACTAAAACACCAGTATACCAGTACACTTGAAAATTTTCCTATAATATATGTTCCTCACTTTTAGTAGGCTATGGTGAGTGTCTTCAAACTTGATGACAAAATGGACAAGACATCTAGTATTGTTTGAATGTTCATCTCATGGATTTGTGTGGAGATTGTACTTTACGGTTTGTACGTACACATTTGTGCAGATCTGTTAGAGCCACAAATTTTGACTAAACTGTTTTTGACCATTGCAAACAATATAAGGCCAAAGAAGACCAAAAAGCAATTTGGGAAGAAATTAAAATATTTTAAATTATTGGATCATCATCACCCTACTCAACAATCTTTGGTGTTATTAATACTTTATAATAAATTATATTTTTGTTTGAATCATTTATATTTAATTATTGGTGACTTATTTTATTATTGCAGGATACTTATGCTTTGTGCCGTGTGTTCAAGAAAAATGGAATATGTACGGATGTTGAAGAGCAAGTAGGGCATTGTAGTAACATGTCTTCACTAATTGAGAGCTCACAAACCATAATCAATAATAATAACAATAATAGTAATAATAATAATGAGTATTGTGAAACCATGTCACCAGACATAGCAGGGGTTTCATCTTCATGTTTGGAAGAGGAAGACAAAGATGATTCATGGATGCAGTTCATCACGGAAGATGCATGGTACTCTTCTAATGCACCAAATATGGTTGGTGGTGAAGAAGTTTCACATGTTACATTTACAAGCTAA

>AdNAC52

ATGCAGAAGTAGTACAAGTAGATGGAATCATCGTGTGTCCCACCTGGGTTTCGCTTCCACCCAACGGATGAAGAGCTTGTTGGTTATTATCTGAGGAAGAAAGTGGCATCTCAGAAGATAGACCTTGACGTTATCAGAGAGATCGATCTCTATCGTATTGAACCCTGGGATCTCCAAGGTACACATACATATACATGCATATGATATGACAGTGAAAATTGATGACATGATTAACTAGATCTGAACGTGTGAGTGCAGAGAGATGTAGGATCGGGTATGAAGAGCAGAACGAGTGGTACTTCTTCAGCCACAAAGACAAGAAGTATCCGACGGGGACTCGAACGAACAGGGCCACCATGGCGGGGTTCTGGAAGGCCACGGGAAGAGACAAGGCGGTGTACGACAAGGCGAAGCTGATCGGGATGAGGAAGACTCTGGTCTTCTACAAAGGGAGAGCCCCTAACGGCCAGAAAACAGACTGGATCATGCACGAGTACAGACTTGAATCCGATGAAAACGGACCCCCTCAGGCAAGCCTTCTAGATTGTTCTATTAGATCTAATTAATAACTAGTTTGTTGCTATGTTACTCATTAATTTTGCTTTTTTTAGTAGACTTGTATTATGTGTATGTATGTTGAGGAAGCTAAGGGACGTACAAGCTTTCTTGGCACACTACTTTCTCATCTCCCAACAACAAGTCAAAAGTCTCACTTCTTTCAACACCGATATAATTGAACGTTTTCTCTTTTCCTTTTCTGCTACACCCTTTTTCTTACCATTTTTCATTGTCAAACGTTAACAAATTAAAGCTAAAGGAGAACCACTACCAACATTCATAACATCATCATCATCTTTAACTTGGTCAACTCAAAGTCTCCAACATAACAACAAAAAGGAAAAACAAAGTCCACAATTCTTTTATATATTTATATAGAATTAACAAACGATTAATAACTTTGGAGCTTTGAATTGAAATCTCAAATTTGTTGTTGCATGCATGCATATATATGCAGGAGGAAGGGTGGGTTGTTTGTAGAGCATTCAAGAAAAGGACGACAAACGGGCAAACGAAGACTATGGAAGGATGGGATTCAAGCTACTTGTACGAGGAAGCAAGCGGCGGCGGTGGCGGTGGCGGCACGGTTGTTGAGTCAATTGAGCAGCTGCTCTCAAGGCAGGGTCATCATCATCATCATCATCAAAGCAGCTTCATGTGCAAGCAAGAAATAGAGAACATGCATGCAAATATAGCAGCAGAGCAATTTGTACAGCTTCCACAGCTTGAGAGCCCAAGTTTGCCGCTAGTTAAGAGGCCAACAACAACAACAAGCACAATGGCACTAGTCTCAGAAAGCAATGAAGAGCATAACATGTTATCTTGCAATAACACGAAGAAAGTAGTGACTGATTGGAGGGATCTTGATAAGTTTGTGGCATCTCAACTGAGTCATGGAGGAGACAATAGTAGGCACGAAACTGAAACCGATGATGCAGCAGTGCTCCCAAGCTTTATGGATAATAACAACCATGACAACAATGGTAGCATCTCGGACATGTCATTGCTGCAGCTTCTGCAGAGTAGTAGTAGTAGTAATAGTAGGGTCAACAATGAAGGGAACAGGTTGATGATGAGCATGAGCCCCTTTCTAAACACAAGCTCTGACTGTGATATTGGGATATGCGTCTTCGAAAATTAA

>AiNAC42

ATGCAGAAGTAGTACAAGTAGATGGAATCATCGTGTGTCCCACCTGGTTTTCGGTTCCACCCAACGGATGAAGAGCTTGTTGGTTATTATCTGAGGAAGAAAGTGGCATCTCAGAAGATAGACCTTGACGTTATCAGAGAGATCGATCTCTATCGTATTGAACCCTGGGATCTCCAAGGTACACATACATATACATGCATATGATATGATAGTGAAAATTGATGAGATTATTAACTAGATCTGAACGTGTGAGTGCAGAGAGATGTAGGATCGGGTATGAAGAGCAGAACGAGTGGTACTTCTTCAGCCACAAAGACAAGAAGTATCCGACGGGGACTCGAACGAACAGGGCCACCATGGCGGGGTTCTGGAAGGCCACCGGAAGAGACAAGGCAGTGTACGACAAGGCGAAGCTGATCGGGATGAGGAAGACTCTCGTGTTCTACAAAGGGAGAGCCCCTAACGGCCAGAAAACAGACTGGATCATGCACGAGTACAGACTTGAATCCGATGAAAACGGACCCCCTCAGGCAAGCCTTCTAGATTGTTCTATATAGCTATTAGATCTAATTAATAATTAGTTTGTTGCTATGTTACTCATTAATTTTGCTTTTTTGAGTAGACTTGTATTATGTGTATGTATGTTGAGGAAGATAAGGGACGTACAAGCTTTCTTGGCACACTACTTTCTCATCTCCCAACAACAAGTCAAAAGTCTCACTTCTTTCAACACCGATATAATTGAACGTTTTCTCTTTTCCTTTTCTGCTACACCCTTTTTCTTACCATTTTTCATTGTCAAACGTTAACAAATTAAATCCTATCTCATTTATATTATTTGACTGCATCACCTACTGCAAAAGCTAAAGGAGAACCACTAACAACATTCATAACATCATCATCATCATCTTTAACTTGGTCAACTCAAAGTCTCAAACATAACAACAAAAAGGAAAAACAAAGTCCACATTTCTTTTATATATTTATATAGAATTAACAAACGATTAATATGTATGCAGGAGGAAGGGTGGGTTGTTTGTAGAGCATTCAAGAAAAGGACGACAAACGGGCAAACGAAGACTATGGAAGGATGGGATTCAAGCTACTTGTACGAGGAAGGGAGCGGCGGCGGTGGCGGCCCGGTTGTTGAGTCAATTGAGCAGCTGCTCTCAAGGCAGAGTCGTCATCATCATCATCATCAAAGCAGCTTCATGTGCAAGCAAGAAATAGAGAACATGCATGCAAATATAGCAGCAGAGCAATTTGTACAGCTTCCACAGCTTGAGAGCCCAAGTTTGCCGCTAGTTAAGAGGCCAACAACAAGCACAATGGCACTAGTCTCAGAAAGCAACGAAGATCATAACATGTTATCGAAGAAAGTAGTGACTGATTGGAGGGATCTTGATAAGTTTGTGGCATCTCAACTGAGTCATGGAGGAGACAGTAGTAGGCACGAAACTGAAACCGATGATGCAGCAGTGCTCCCAAGCTTTATGGATAATAACAACCATGACAACAGTGATAGCATCTCGGACATGTCATTGCTGCAGCTTCTGCAGAGTAGTAGTAGTAATAGTAGGGTCAACAATGAAGGGAACAGGTTGATGATGAGCATGAGCCCCTTTCTAAACACAAGCTCTGACTGTGATATTGGGATATGCGTCTTCGAAAATTAA

>AdNAC35

GGGAGTTCCTACGAAGTTGGTTTCTTTCTTCACTGCTGGTGAAGTGGTTCTTTGTTGTTGGATTGTTTGATTCAAGGGAAGTTGTTAGATTCTGAAGGTTTTACTATTTAAGCTTTCTTTATTCTTCTGCGATGGCCGCAATGAAGTCAATTCCAGGGTACCGGTTTCATCCAACCGATGTTGAGCTGGTTCAGTACTTTCTGAAAAGGAAGGTGATGGGGAAGAGATTCCCTTGTGATGTGATTGCTGAACTTGATATATACAAATATCCGCCGTGGGATCTACCAGGTTTGCACTCTGATTTCATTTGATTTAGTAGAGTTCTGTGTTCCTCAGAGTTGTTTTTAACTTGATGATATTTTTTCAGATCATTCTTTGCTTAAAACTGGAGATTTGGAATGGTACTTCTTTTGCCCTCGAGGGAAGAAGTATTCGAGCGGAGGGAGGATGAATAGGGCCACAGAATGTGGGTACTGGAAGACTACTGGCAAAGATAGATCCGTTGAGAACAAGAAGCTTGTTGTGGGCATGATAAAGACTCTGGTGTTTCACATGGGTAAAGCACCCAAGGGAGATCGAACTGATTGGGTTTTGCATGAATACCGACTTCAAGATAAGGACCTTGCTGATAAGGGTGTTCAACAGGTAACTACTGATTACTCATTCATGAACATTAAGTGTATTTCTACTTTGGTGAGACTAAGGAAATATATGTTGAAATATATGCTGCTAATTATTGTTATCTGTTAGTGTTTGTGCTTTTCTGCCCATATGGGGATAACTAGGAATCTAGGATTGTTGTTATTTATGTTGTAATCGTCTTATGTTTCACTAGAAGCTATGCTCTTAGCTGATGAGCGTATTTTAATACACGAGGATTAGTGGATTACAGAATTCTGCTGAATTGTGCGCGTTGCAACCTCTATTGTTGGTAATGGCTTCTATGTAGATTGTCTTTTCATGTTAAGCCTGATGCCATAAACTGTTTTTTAGCAGTATATATATGCCTGGTGCTTAAGCTGTATGGTATATAATGGCTAGCTTTACTTAATATAATTTTACCGTATGTCTGACTAGTTTAGCATCTCTTCTGAGACTGTTTATGGTTTTTGCTTATTTTTGTGCTTGTTTTTCTGGTGTGTTTATTTTAATTAGTTCTAATTGTTTACGTTTTTCTCAGGATTCTTATGTGATATGTAAAGTGTTTCAAAAGGATGGTCCTGGTCCTAGGAATGGTGCACAATATGGAAGGCCGTTTAATGAGGAAGACTGGGATAAAGAGGATGAAATTGACTGTGTAGAATCTGCACCTGTTGCTGCTCTACCTGCTGCAGTTCCTATACAACCCGCTTCATGTCATAGCTCTGTTGTGAATAACGTGAATCTCTCTGTGAGTGAATGCTATGGGTTGACCTCTGTTTCGTGTTTAACAGGGCCAATGCCTTCTTGCTCAGCACATCCTTCAGCTCCAAGTAATCAAGTTGATGGTGACATTACACCAGTGCCTGGTTCCTCCATAGAAGATAACATAATGGCTCCTACTCAGAACACCACAACTGAAGTATGCAATCTATTTATTCTTATTTATGCCTGCACAATATTTTGGGGGAGTTTGACTAGATTGTTTTTCTTTTCATGCTTGTTTCAATCTAATCTATGACATCTCTTCTACAGTTTCCTTCCATCTCTTCCCCCATATTGTGGAAACATATCTAGGACCTTGGTTTTTGGAATAACTGCATCTGTCGCATCCATTATTTTTAGTTTCAAACTCATGATGCTTTATTATTATTGTTTTACTTTGTTCCATAAATGTTCTGAGCCTTCATTTCTTTTCTCTTGTTCTAATTTTGTAATTACAGAAGGTTGACAATCCTCCTGACATAAACAATGCTGAAGGAACACCTTGCTTTGATCCCAATGAGATTTTTGGGGGTCTGGGTGACCTTGATGGTTTGTTCGAAATGGGTGGAATTGGACATGGTTTTTCCTGCGGCCAAAATGGTGGATATACTGTGAATGAAATGCTTTCTGCGGGTGATGGGTTGCGTTTCCCTGATCCCCTGGACTACTTGGAGTTGGGTGACCTCGACACTCCATTGTTATGGGAGACTAATGAACAAGGAAATTGGAGCCAGGACAATAAGTGAGGCTTCAAAATTTGACGAAGCATCAACATGAAGTTGGTGGAAGAACCATTGCCGTGTGTTGCTGATCTTGATTGTCTTTCCATTCTCATTTATAAACTCTACTGAAGCATATATTCTGTAGAAACATTCCTTTCTGTAATAAGCTAACACATGATAGCAGTTGATAAATCTTTAGTGTTAGTTCTATTTACTAGGTTGGATTAATCATTAGCCTCACAAATTTTCTTCTGATATTTTCTAAGTTGTGTTTGATAGAGTGAGTTTTCAACATCTATTACGTTGAAATCTATTCATGTCTTTCGTAGTAATGTCAAATGATGGAAGTTGACGCATTTAACCCTAAACTTCTAGTATTCTCAATTTTGTTTTATTGAAGGGTTAGTTGATGAGGGAACTGTGATAGGCCTTTGCCCCTTTACTTTTCGTCGCGTTGGAAACTGGAAAGTACGTTATCTAAACCAGTATAGC

>AiNAC17
[truncated: 39,576 more chars]
