# Supplementary material for: Comprehensive genomic characterization of NAC transcription factor family and their response to salt and drought stress in peanut
Source: BMC Plant Biol. 2020 Oct 2;20:454. doi: 10.1186/s12870-020-02678-9 (PMC7532626; doi:10.1186/s12870-020-02678-9)
Supplement: Supplementary file 3 — Additional file 3.NAC TF gene family members in cultivated peanut. [file 12870_2020_2678_MOESM3_ESM.docx]

| Gene symbol | Gene model name | Gene location | Length (aa) | MW (kDa) | Theoretical pI |
| --- | --- | --- | --- | --- | --- |
| AhNAC1 | Arahy.05SHD0 | Chr11:53546420.. 53550826 | 386 | 44.4 | 6.16 |
| AhNAC2 | [Arahy.09VTBX](https://www.peanutbase.org/feature/Arachis/hypogaea/gene/arahy.Tifrunner.gnm1.ann1.09VTBX) | Chr7:9512104..9514059 | 240 | 27.8 | 7.16 |
| AhNAC3 | Arahy.0UTR6Y | Chr19:145193543..145194694 | 90 | 10.3 | 5.53 |
| AhNAC4 | Arahy.0V68XN | Chr12:19289697..19296599 | 307 | 34.7 | 6.59 |
| AhNAC5 | Arahy.12HUMH | Chr6:100589984..10059170 | 273 | 31.8 | 9.20 |
| AhNAC6 | Arahy.13C0EN | Chr19:141984144..141988045 | 418 | 46.4 | 5.10 |
| AhNAC7 | Arahy.1BXK41 | Chr20:133573663..133576546 | 152 | 17.6 | 4.53 |
| AhNAC8 | Arahy.1GPE0T | Chr17:18202759..18208274 | 678 | 77.3 | 5.51 |
| AhNAC9 | Arahy.1I167B | Chr11:35134950..3513800 | 277 | 31.3 | 5.37 |
| AhNAC10 | Arahy.1IJJ7X | Chr12:7116915..7121461 | 481 | 54.6 | 5.61 |
| AhNAC11 | Arahy.1P6DI5 | Chr6:95240090..95241866 | 256 | 29.0 | 6.30 |
| AhNAC12 | Arahy.1Q9HM | Chr9:118111101..118114121 | 226 | 26.2 | 5.54 |
| AhNAC13 | Arahy.1UE66A | Chr20:99254524..99259219 | 591 | 66.9 | 5.46 |
| AhNAC14 | Arahy.2275VI | Chr1:19572736..19575435 | 127 | 15.2 | 9.63 |
| AhNAC15 | Arahy.23ZMCQ | Chr20:115280112..115281873 | 279 | 37.1 | 6.15 |
| AhNAC16 | Arahy.24LKGZ | Chr13:34282807..34286593 | 381 | 42.7 | 7.33 |
| AhNAC17 | Arahy.2C699W | Chr18:39974479..39980788 | 217 | 25.1 | 9.20 |
| AhNAC18 | Arahy.2FYA1K | Chr18:132283391..132286467 | 304 | 35.1 | 6.42 |
| AhNAC19 | Arahy.2I3PJC | Chr19:137577568..137583637 | 740 | 83.2 | 8.65 |
| AhNAC20 | Arahy.2L619Y | Chr1:44477832..44482133 | 382 | 43.8 | 6.30 |
| AhNAC21 | Arahy.2MPS86 | Chr13:210007..211705 | 317 | 36.0 | 8.94 |
| AhNAC22 | Arahy.2P3Z86 | Chr15:155843327..155845244 | 306 | 34.4 | 5.48 |
| AhNAC23 | Arahy.332M63 | Chr10:102674028..102677702 | 374 | 41.6 | 8.64 |
| AhNAC24 | Arahy.338FND | Chr7:67924103..67926888 | 269 | 31.6 | 6.43 |
| AhNAC25 | Arahy.3F2LJ6 | Chr16:126272371..126274687 | 395 | 44.5 | 6.21 |
| AhNAC26 | Arahy.3G9MJD | Chr20:141213309..141214542 | 133 | 15.5 | 5.58 |
| AhNAC27 | Arahy.3GEX4P | Chr3:120056206..120057711 | 206 | 23.6 | 5.19 |
| AhNAC28 | Arahy.3M9RMX | Chr5:87890742..87893316 | 363 | 40.2 | 9.35 |
| AhNAC29 | Arahy.3N6K0N | Chr5:48399897..48401289 | 213 | 24.4 | 4.81 |
| AhNAC30 | Arahy.3X1GMP | Chr5:88031217..88033817 | 363 | 40.2 | 9.42 |
| AhNAC31 | Arahy.4435CX | Chr3:11227096..11229643 | 384 | 43.6 | 7.33 |
| AhNAC32 | Arahy.4PPW5Y | Chr18:39973832..39974183 | 80 | 9.3 | 8.32 |
| AhNAC33 | Arahy.4QZT53 | Chr13:14914685..14916941 | 375 | 42.5 | 7.28 |
| AhNAC34 | Arahy.563XC3 | Chr3:10904376..10904971 | 64 | 7.8 | 5.17 |
| AhNAC35 | Arahy.5AD6K9 | Chr6:113391620..113395838 | 393 | 44.1 | 6.67 |
| AhNAC36 | Arahy.5P3U81 | Chr18:6544943..6546522 | 264 | 29.6 | 7.03 |
| AhNAC37 | Arahy.65HUV4 | Chr8:38774621..38778136 | 358 | 39.8 | 8.65 |
| AhNAC38 | Arahy.6LHU5T | Chr12:7075943..7082266 | 210 | 23.8 | 6.39 |
| AhNAC39 | Arahy.6RTE4A | Chr5:104880777..104885849 | 275 | 31.7 | 5.12 |
| AhNAC40 | Arahy.70K1B3 | Chr13:142582173..142582608 | 99 | 11.2 | 9.34 |
| AhNAC41 | Arahy.72Q128 | Chr15:59403427..59405511 | 217 | 24.9 | 4.98 |
| AhNAC42 | Arahy.76LABN | Chr15:5793582..5797056 | 356 | 40.3 | 5.2 |
| AhNAC43 | Arahy.78BVZL | Chr7:9526281..9530142 | 319 | 36.9 | 5.27 |
| AhNAC44 | Arahy.798CQ8 | Chr10:114422543..114425830 | 204 | 23.6 | 5.35 |
| AhNAC45 | Arahy.7G7SK7 | Chr4:123511466..123513398 | 330 | 38.0 | 5.72 |
| AhNAC46 | Arahy.7GZ1C0 | Chr12:6271946..6275241 | 464 | 51.3 | 6.05 |
| AhNAC47 | Arahy.7J37F0 | Chr15:153065181..153069211 | 418 | 46.4 | 5.68 |
| AhNAC48 | Arahy.7QMU6B | Chr18:134197800..134200379 | 239 | 27.8 | 9.74 |
| AhNAC49 | Arahy.83A3G6 | Chr5:87976693..87978686 | 351 | 39.1 | 9.06 |
| AhNAC50 | Arahy.83IFXD | Chr16:126403709..126405478 | 325 | 36.6 | 5.98 |
| AhNAC51 | Arahy.83Q9A2 | Chr1:105164904..105167533 | 321 | 36.1 | 6.10 |
| AhNAC52 | Arahy.8AKD3R | Chr6:27881808..27882681 | 62 | 7.2 | 5.42 |
| AhNAC53 | Arahy.8D109F | Chr19:149053837..149058561 | 499 | 56.1 | 4.44 |
| AhNAC54 | Arahy.8E68ZN | Chr16:131914437..131916265 | 270 | 31.4 | 9.28 |
| AhNAC55 | Arahy.8GCY61 | Chr2:14868224..14874967 | 303 | 34.4 | 6.56 |
| AhNAC56 | Arahy.8KC8J6 | Chr2:5663650..5668228 | 481 | 54.4 | 5.81 |
| AhNAC57 | Arahy.90HG3Y | Chr2:5600058..5605255 | 282 | 32.7 | 5.08 |
| AhNAC58 | Arahy.919QYJ | Chr15:119586340..119591026 | 431 | 48.5 | 4.98 |
| AhNAC59 | Arahy.9732XC | Chr18:11907902..11910313 | 294 | 33.5 | 8.82 |
| AhNAC60 | Arahy.9ZTQ0N | Chr18:22975006..22977422 | 269 | 30.0 | 5.22 |
| AhNAC61 | Arahy.A1VWSL | Chr13:11001262..11003864 | 313 | 36.1 | 6.62 |
| AhNAC62 | Arahy.A3J7SX | Chr9:105602762..105609144 | 606 | 67.9 | 7.22 |
| AhNAC63 | Arahy.A5ASCL | Chr6:75207120..75209243 | 256 | 29.5 | 6.32 |
| AhNAC64 | Arahy.A80DKX | Chr13:133805862..133810507 | 62 | 7.0 | 11.28 |
| AhNAC65 | Arahy.AIPG34 | Chr13:138011479..138012935 | 286 | 32.8 | 8.32 |
| AhNAC66 | Arahy.B9XEKF | Chr8:40056889..40059540 | 370 | 40.8 | 7.22 |
| AhNAC67 | Arahy.BEF3I8 | Chr8:34472677..34475068 | 300 | 34.2 | 8.55 |
| AhNAC68 | Arahy.BFU0GS | Chr8:10764843..10766881 | 225 | 25.2 | 4.95 |
| AhNAC69 | Arahy.BN8407 | Chr7:41534099..41538588 | 349 | 39.9 | 5.05 |
| AhNAC70 | Arahy.BPCJ1X | Chr18:6621921..6624293 | 444 | 49.4 | 5.42 |
| AhNAC71 | Arahy.BS3I7W | Chr7:3197672..3201191 | 281 | 32.9 | 5.27 |
| AhNAC72 | Arahy.BX5EMB | Chr15:145545938..145550107 | 363 | 41.1 | 7.21 |
| AhNAC73 | Arahy.CDPA7L | Chr8:30826823..30829116 | 457 | 50.7 | 5.38 |
| AhNAC74 | Arahy.CK11CG | Chr10:108765200..108768180 | 317 | 36.6 | 4.96 |
| AhNAC75 | Arahy.CRX62L | Chr15:136448827..136454210 | 275 | 31.7 | 5.12 |
| AhNAC76 | Arahy.CSHQ77 | Chr1:29507895..29511264 | 331 | 37.2 | 4.82 |
| AhNAC77 | Arahy.CSZ51X | Chr8:50199808..50201900 | 241 | 28.0 | 9.77 |
| AhNAC78 | Arahy.CTTQ97 | Chr5:99556719..99560479 | 376 | 42.6 | 7.21 |
| AhNAC79 | Arahy.D4BTID | Chr3:120646808..120654287 | 317 | 35.3 | 5.21 |
| AhNAC80 | Arahy.D5FDJH | Chr13:129791976..129793880 | 248 | 27.3 | 5.78 |
| AhNAC81 | Arahy.EHBV2Z | Chr15:12754683..12755219 | 151 | 17.8 | 8.36 |
| AhNAC82 | Arahy.F01Q5M | Chr6:1217684..1221909 | 280 | 32.5 | 6.67 |
| AhNAC83 | Arahy.FD63AG | Chr13:131061978..131066907 | 330 | 37.3 | 8.47 |
| AhNAC84 | Arahy.FFKU3L | Chr19:137608276..137611863 | 583 | 65 | 4.67 |
| AhNAC85 | Arahy.FHJ4BK | Chr14:105327338..105329676 | 76 | 8.4 | 4.64 |
| AhNAC86 | Arahy.FKL2A7 | Chr3:138316534..138318357 | 195 | 22.3 | 4.77 |
| AhNAC87 | Arahy.FU1JML | Chr8:38900847..38910580 | 349 | 39.1 | 7.66 |
| AhNAC88 | Arahy.G1V3KR | Chr20:114021918..114025341 | 367 | 40.4 | 4.72 |
| AhNAC89 | Arahy.G3FV2L | Chr3:32455210..32459113 | 382 | 42.8 | 7.34 |
| AhNAC90 | Arahy.G3YZJ0 | Chr10:108271367..108274153 | 139 | 16.2 | 4.54 |
| AhNAC91 | Arahy.GDX8G8 | Chr7:50520786..50525633 | 350 | 40.5 | 7.07 |
| AhNAC92 | Arahy.GPRR9Y | Chr17:102371104..102372749 | 394 | 44.5 | 7.85 |
| AhNAC93 | Arahy.GU1UJS | Chr19:148021172..148024295 | 250 | 29.0 | 5.45 |
| AhNAC94 | Arahy.H91V8V | Chr17:31791028..31795578 | 709 | 78.9 | 4.72 |
| AhNAC95 | Arahy.HHSK2A | Chr7:9707093..9707384 | 96 | 11.7 | 6.29 |
| AhNAC96 | Arahy.HJ0R1G | Chr12:88963124..88964180 | 188 | 21.6 | 9.63 |
| AhNAC97 | Arahy.I1Q9WS | Chr15:155808825..155810967 | 268 | 30.3 | 5.94 |
| AhNAC98 | Arahy.I4FPAQ | Chr17:130540422..130541478 | 188 | 21.7 | 9.76 |
| AhNAC99 | Arahy.ILS8DP | Chr9:105641267..105644874 | 583 | 65.3 | 4.71 |
| AhNAC100 | Arahy.JB9PK4 | Chr18:58337765..58344128 | 321 | 36.3 | 7.57 |
| AhNAC101 | Arahy.JBNT97 | Chr3:131298981..131303032 | 634 | 71.8 | 6.32 |
| AhNAC102 | Arahy.JBU48Q | Chr17:127095479..127097548 | 304 | 34.1 | 7.12 |
| AhNAC103 | Arahy.JE37KP | Chr11:135087590..135090066 | 403 | 46.1 | 6.86 |
| AhNAC104 | Arahy.JHHH6T | Chr16:136652643..136656818 | 491 | 55.0 | 5.01 |
| AhNAC105 | Arahy.JUA047 | Chr8:28456145..28457404 | 360 | 41 | 5.95 |
| AhNAC106 | Arahy.K4491K | Chr6:104660121..104664605 | 471 | 52.6 | 5.08 |
| AhNAC107 | Arahy.K9ZHT4 | Chr18:68815412..68818348 | 78 | 9.2 | 4.42 |
| AhNAC108 | Arahy.KK00U0 | Chr8:4243922..4245422 | 376 | 42.5 | 7.84 |
| AhNAC109 | Arahy.L5HHP2 | Chr8:51200842..51202462 | 322 | 36.5 | 8.99 |
| AhNAC110 | Arahy.L9IK9Y | Chr1:110960044..110961987 | 330 | 37.2 | 8.16 |
| AhNAC111 | Arahy.LV3APC | Chr2:4991064..4994322 | 466 | 51.4 | 6.13 |
| AhNAC112 | Arahy.M99KVR | Chr13:117576838..117578079 | 211 | 23.7 | 9.45 |
| AhNAC113 | Arahy.MFVS6B | Chr7:25230676..25235223 | 698 | 77.7 | 4.69 |
| AhNAC114 | Arahy.MGN032 | Chr4:128745510..128748005 | 248 | 28.2 | 8.61 |
| AhNAC115 | Arahy.MI72XM | Chr10:91687222..91690640 | 367 | 40.4 | 4.75 |
| AhNAC116 | Arahy.N4TQEE | Chr10:92412280..92413664 | 230 | 26.1 | 5.23 |
| AhNAC117 | Arahy.NB8KRW | Chr13:29651467..29653481 | 362 | 40.8 | 6.34 |
| AhNAC118 | Arahy.NLE5K3 | Chr10:5667471..5669359 | 185 | 21.4 | 4.83 |
| AhNAC119 | Arahy.Q3Y7SF | Chr3:127243744..127245897 | 330 | 36.5 | 8.36 |
| AhNAC120 | Arahy.QDSH2R | Chr13:122796079..122797577 | 206 | 23.6 | 5.19 |
| AhNAC121 | Arahy.QL5RCW | Chr20:4143170..4146189 | 324 | 36.9 | 8.41 |
| AhNAC122 | Arahy.QVEY1G | Chr16:27133221..27134709 | 225 | 26.1 | 7.93 |
| AhNAC123 | Arahy.QZZL54 | Chr3:27969655..27971660 | 376 | 42.5 | 8.17 |
| AhNAC124 | Arahy.R9WKT4 | Chr20:134322596..134325308 | 292 | 33.5 | 4.71 |
| AhNAC125 | Arahy.RC5QY0 | Chr13:141206123..141208762 | 237 | 27.0 | 5.50 |
| AhNAC126 | Arahy.RU4C7B | Chr18:101051701..101056198 | 350 | 40.0 | 5.05 |
| AhNAC127 | Arahy.S9FEUH | Chr3:135147747..135149530 | 286 | 32.8 | 8.48 |
| AhNAC128 | Arahy.SJ3Y3C | Chr5:12184443..12184988 | 154 | 18.1 | 8.69 |
| AhNAC129 | Arahy.T50ENK | Chr15:150435586..150441902 | 461 | 52.2 | 6.58 |
| AhNAC130 | Arahy.T5AJQY | Chr13:23543198..23549543 | 285 | 32.6 | 7.75 |
| AhNAC131 | Arahy.TCTP66 | Chr18:4207013..4208270 | 360 | 41 | 5.95 |
| AhNAC132 | Arahy.TY7WD8 | Chr3:7810467..7813061 | 315 | 36.3 | 6.62 |
| AhNAC133 | Arahy.U16Y2L | Chr5:5793582..5797056 | 356 | 40.3 | 5.20 |
| AhNAC134 | Arahy.U487DX | Chr10:2874655..2876604 | 169 | 19.2 | 9.34 |
| AhNAC135 | Arahy.UCK419 | Chr9:120314038..120317924 | 410 | 46.5 | 5.10 |
| AhNAC136 | Arahy.UK39BN | Chr17:5194799..5198834 | 251 | 29.4 | 5.41 |
| AhNAC137 | Arahy.UX5JN7 | Chr5:95117451..95123223 | 460 | 52.0 | 6.58 |
| AhNAC138 | Arahy.V0X4SV | Chr5:115369101..115371675 | 240 | 27.6 | 5.87 |
| AhNAC139 | Arahy.V20ZHW | Chr10:77824836..77828550 | 592 | 67.0 | 5.52 |
| AhNAC140 | Arahy.V6VDUM | Chr3:22261118..22266410 | 286 | 32.7 | 7.78 |
| AhNAC141 | Arahy.V88TU5 | Chr7:72037705..72043979 | 323 | 36.5 | 7.57 |
| AhNAC142 | Arahy.V9YDBD | Chr10:2052796..2056222 | 319 | 36.6 | 8.14 |
| AhNAC143 | Arahy.VCF3H0 | Chr8:48032456..48035512 | 304 | 35.1 | 6.42 |
| AhNAC144 | Arahy.VI6QZG | Chr20:10819553..10821059 | 185 | 21.4 | 4.92 |
| AhNAC145 | Arahy.W5I9MA | Chr3:134840921..134842779 | 201 | 22.0 | 4.56 |
| AhNAC146 | Arahy.W8FFAE | Chr13:137847418..137848474 | 188 | 21.6 | 9.76 |
| AhNAC147 | Arahy.WB1HDB | Chr20:126391430..126395275 | 370 | 41.0 | 8.86 |
| AhNAC148 | Arahy.WF4CBH | Chr14:143181040..143183535 | 248 | 28.2 | 8.61 |
| AhNAC149 | Arahy.WPHD30 | Chr3:119219927..119222377 | 260 | 30.0 | 7.71 |
| AhNAC150 | Arahy.WULW7H | Chr6:95121632..95123973 | 396 | 44.5 | 6.21 |
| AhNAC151 | Arahy.X47CQ0 | Chr13:121867203..121869706 | 260 | 30.0 | 7.09 |
| AhNAC152 | Arahy.XB6K25 | Chr18:26244148..26246859 | 366 | 40.4 | 7.22 |
| AhNAC153 | Arahy.XKF840 | Chr7:14476514..14482811 | 679 | 77.4 | 5.43 |
| AhNAC154 | Arahy.XMHS8A | Chr5:109971297..109975961 | 431 | 48.5 | 4.98 |
| AhNAC155 | Arahy.YB9YM9 | Chr18:22631602..22633194 | 297 | 33.0 | 9.18 |
| AhNAC156 | Arahy.YH9HLJ | Chr20:5058214..5060443 | 345 | 39.4 | 6.51 |
| AhNAC157 | Arahy.YSLF5V | Chr18:109673628..109678502 | 350 | 40.5 | 6.73 |
| AhNAC158 | Arahy.YV5C93 | Chr13:137702108..137703952 | 198 | 21.7 | 4.56 |
| AhNAC159 | Arahy.YXGX3A | Chr8:30758970..30760629 | 264 | 30.0 | 7.06 |
| AhNAC160 | Arahy.YY4A03 | Chr15:160449217..160451778 | 240 | 27.5 | 5.87 |
| AhNAC161 | Arahy.Z03JM7 | Chr3:115179051..115180523 | 211 | 23.6 | 9.45 |
| AhNAC162 | Arahy.Z8VU36 | Chr16:152695625..152700210 | 558 | 62.6 | 6.70 |
| AhNAC163 | Arahy.ZDQ75D | Chr16:100258872..100260783 | 263 | 30.0 | 6.51 |
| AhNAC164 | Arahy.ZM1I0C | Chr5:13979980..13983527 | 368 | 42.6 | 8.01 |
