# Supplementary material for: Comprehensive genomic characterization of NAC transcription factor family and their response to salt and drought stress in peanut
Source: BMC Plant Biol. 2020 Oct 2;20:454. doi: 10.1186/s12870-020-02678-9 (PMC7532626; doi:10.1186/s12870-020-02678-9)
Supplement: Supplementary file 4 — Additional file 4. mRNA sequence of NAC genes from cultivated peanut. [file 12870_2020_2678_MOESM4_ESM.docx]

[> AhNAC1-Arahy.05SHD0.1 4](#_Toc28891)

[> AhNAC2-Arahy.09VTBX.1 5](#_Toc32093)

[> AhNAC3-Arahy.0UTR6Y.1 5](#_Toc14311)

[> AhNAC4-Arahy.0V68XN.1 6](#_Toc20295)

[> AhNAC5-Arahy.12HUMH.1 6](#_Toc16024)

[>AhNAC6-Arahy.13C0EN.1 6](#_Toc28933)

[> AhNAC7-Arahy.1BXK41.1 7](#_Toc13971)

[> AhNAC8-Arahy.1GPE0T.1 7](#_Toc30110)

[> AhNAC9-Arahy.1I167B.1 8](#_Toc22222)

[> AhNAC10-Arahy.1IJJ7X.1 9](#_Toc12278)

[>AhNAC11-Arahy.1P6DI5.1 9](#_Toc26434)

[>AhNAC12-Arahy.1Q9HM8.1 10](#_Toc3652)

[>AhNAC13-Arahy.1UE66A.1 10](#_Toc13055)

[>AhNAC14-Arahy.2275VI.1 11](#_Toc13886)

[>AhNAC15-Arahy.23ZMCQ.1 11](#_Toc23582)

[>AhNAC16-Arahy.24LKGZ.1 11](#_Toc4049)

[>AhNAC17-Arahy.2C699W.1 12](#_Toc10289)

[>AhNAC18-Arahy.2FYA1K.1 12](#_Toc6741)

[>AhNAC19-Arahy.2I3PJC.1 13](#_Toc20752)

[>AhNAC20-Arahy.2L619Y.1 14](#_Toc2203)

[>AhNAC21-Arahy.2MPS86.1 14](#_Toc1337)

[>AhNAC22-Arahy.2P3Z86.1 15](#_Toc16976)

[> AhNAC23-Arahy.332M63.1 15](#_Toc23590)

[> AhNAC24-Arahy.338FND.1 16](#_Toc7556)

[> AhNAC25-Arahy.3F2LJ6.1 16](#_Toc26169)

[> AhNAC26-Arahy.3G9MJD.1 17](#_Toc31063)

[> AhNAC27-Arahy.3GEX4P.1 17](#_Toc32056)

[> AhNAC28-Arahy.3M9RMX.1 17](#_Toc27415)

[> AhNAC29-Arahy.3N6K0N.1 18](#_Toc24936)

[> AhNAC30-Arahy.3X1GMP.1 18](#_Toc31065)

[> AhNAC31-Arahy.4435CX.1 19](#_Toc20683)

[> AhNAC32-Arahy.4PPW5Y.1 19](#_Toc31956)

[> AhNAC33-Arahy.4QZT53.1 19](#_Toc28945)

[> AhNAC34-Arahy.563XC3.1 20](#_Toc15614)

[>AhNAC35-Arahy.5AD6K9.1 20](#_Toc27189)

[>AhNAC36-Arahy.5P3U81.1 21](#_Toc20167)

[>AhNAC37-Arahy.65HUV4.1 21](#_Toc16095)

[>AhNAC38-Arahy.6LHU5T.1 21](#_Toc11742)

[>AhNAC39-Arahy.6RTE4A.1 22](#_Toc6402)

[>AhNAC40-Arahy.70K1B3.1 22](#_Toc3424)

[>AhNAC41-Arahy.72Q128.1 22](#_Toc13399)

[>AhNAC42-Arahy.76LABN.1 23](#_Toc752)

[>AhNAC43-Arahy.78BVZL.1 23](#_Toc7575)

[>AhNAC44-Arahy.798CQ8.1 24](#_Toc1918)

[>AhNAC45-Arahy.7G7SK7.1 24](#_Toc29862)

[>AhNAC46-Arahy.7GZ1C0.1 25](#_Toc25413)

[>AhNAC47-Arahy.7J37F0.1 25](#_Toc4373)

[>AhNAC48-Arahy.7QMU6B.1 26](#_Toc1741)

[>AhNAC49-Arahy.83A3G6.1 26](#_Toc9117)

[>AhNAC50-Arahy.83IFXD.1 27](#_Toc8438)

[>AhNAC51-Arahy.83Q9A2.1 27](#_Toc1152)

[>AhNAC52-Arahy.8AKD3R.1 28](#_Toc594)

[>AhNAC53-Arahy.8D109F.1 28](#_Toc7674)

[>AhNAC54-Arahy.8E68ZN.1 28](#_Toc8283)

[>AhNAC55-Arahy.8GCY61.1 29](#_Toc6945)

[>AhNAC56-Arahy.8KC8J6.1 29](#_Toc24388)

[>AhNAC57-Arahy.90HG3Y.1 30](#_Toc1769)

[>AhNAC58-Arahy.919QYJ.1 30](#_Toc16253)

[>AhNAC59-Arahy.9732XC.1 31](#_Toc9256)

[>AhNAC60-Arahy.9ZTQ0N.1 31](#_Toc13342)

[>AhNAC61-Arahy.A1VWSL.1 32](#_Toc20170)

[>AhNAC62-Arahy.A3J7SX.1 32](#_Toc20942)

[>AhNAC63-Arahy.A5ASCL.1 33](#_Toc30614)

[>AhNAC64-Arahy.A80DKX.1 33](#_Toc16882)

[>AhNAC65-Arahy.AIPG34.1 34](#_Toc5941)

[>AhNAC66-Arahy.B9XEKF.1 34](#_Toc1048)

[>AhNAC67-Arahy.BEF3I8.1 35](#_Toc6746)

[>AhNAC68-Arahy.BFU0GS.1 35](#_Toc6144)

[>AhNAC69-Arahy.BN8407.1 35](#_Toc6203)

[>AhNAC70-Arahy.BPCJ1X.1 36](#_Toc15540)

[>AhNAC71-Arahy.BS3I7W.1 37](#_Toc28090)

[>AhNAC72-Arahy.BX5EMB.1 37](#_Toc5511)

[>AhNAC73-Arahy.CDPA7L.1 38](#_Toc27375)

[>AhNAC74-Arahy.CK11CG.1 39](#_Toc19452)

[>AhNAC75-Arahy.CRX62L.1 39](#_Toc28149)

[>AhNAC76-Arahy.CSHQ77.1 40](#_Toc15835)

[>AhNAC77-Arahy.CSZ51X.1 41](#_Toc23846)

[>AhNAC78-Arahy.CTTQ97.1 41](#_Toc258)

[>AhNAC79-Arahy.D4BTID.1 42](#_Toc17447)

[>AhNAC80-Arahy.D5FDJH.1 43](#_Toc4792)

[>AhNAC81-Arahy.EHBV2Z.1 43](#_Toc12922)

[>AhNAC82-Arahy.F01Q5M.1 43](#_Toc30339)

[>AhNAC83-Arahy.FD63AG.1 44](#_Toc12140)

[>AhNAC84-Arahy.FFKU3L.1 45](#_Toc26838)

[>AhNAC85-Arahy.FHJ4BK.1 46](#_Toc30357)

[>AhNAC86-Arahy.FKL2A7.1 46](#_Toc11286)

[>AhNAC87-Arahy.FU1JML.1 47](#_Toc2376)

[>AhNAC88-Arahy.G1V3KR.1 48](#_Toc27325)

[>AhNAC89-Arahy.G3FV2L.1 49](#_Toc17580)

[>AhNAC90-Arahy.G3YZJ0.1 50](#_Toc16297)

[>AhNAC91-Arahy.GDX8G8.1 50](#_Toc26951)

[>AhNAC92-Arahy.GPRR9Y.1 51](#_Toc10313)

[>AhNAC93-Arahy.GU1UJS.1 51](#_Toc23342)

[>AhNAC94-Arahy.H91V8V.1 52](#_Toc11232)

[>AhNAC95-Arahy.HHSK2A.1 53](#_Toc32212)

[>AhNAC96-Arahy.HJ0R1G.1 53](#_Toc31566)

[>AhNAC97-Arahy.I1Q9WS.1 53](#_Toc10898)

[>AhNAC98-Arahy.I4FPAQ.1 54](#_Toc24735)

[>AhNAC99-Arahy.ILS8DP.1 54](#_Toc537)

[>AhNAC100-Arahy.JB9PK4.1 55](#_Toc25911)

[>AhNAC101-Arahy.JBNT97.1 56](#_Toc28143)

[>AhNAC102-Arahy.JBU48Q.1 57](#_Toc25476)

[>AhNAC103-Arahy.JE37KP.1 58](#_Toc8636)

[>AhNAC104-Arahy.JHHH6T.1 58](#_Toc24479)

[>AhNAC105-Arahy.JUA047.1 59](#_Toc31746)

[>AhNAC106-Arahy.K4491K.1 60](#_Toc9638)

[>AhNAC107-Arahy.K9ZHT4.1 61](#_Toc27011)

[>AhNAC108-Arahy.KK00U0.1 61](#_Toc25605)

[>AhNAC109-Arahy.L5HHP2.1 62](#_Toc18655)

[>AhNAC110-Arahy.L9IK9Y.1 62](#_Toc26907)

[>AhNAC111-Arahy.LV3APC.1 63](#_Toc30779)

[>AhNAC112-Arahy.M99KVR.1 64](#_Toc9937)

[>AhNAC113-Arahy.MFVS6B.1 64](#_Toc17635)

[>AhNAC114-Arahy.MGN032.1 66](#_Toc29724)

[>AhNAC115-Arahy.MI72XM.1 66](#_Toc30030)

[>AhNAC116-Arahy.N4TQEE.1 67](#_Toc914)

[>AhNAC117-Arahy.NB8KRW.1 68](#_Toc10219)

[>AhNAC118-Arahy.NLE5K3.1 69](#_Toc32613)

[>AhNAC119-Arahy.Q3Y7SF.1 69](#_Toc21912)

[>AhNAC120-Arahy.QDSH2R.1 70](#_Toc32394)

[>AhNAC121-Arahy.QL5RCW.1 70](#_Toc31939)

[>AhNAC122-Arahy.QVEY1G.1 71](#_Toc25563)

[>AhNAC123-Arahy.QZZL54.1 72](#_Toc25207)

[>AhNAC124-Arahy.R9WKT4.1 72](#_Toc32620)

[>AhNAC125-Arahy.RC5QY0.1 73](#_Toc226)

[>AhNAC126-Arahy.RU4C7B.1 73](#_Toc19313)

[>AhNAC127-Arahy.S9FEUH.1 74](#_Toc14113)

[>AhNAC128-Arahy.SJ3Y3C.1 75](#_Toc10596)

[>AhNAC129-Arahy.T50ENK.1 75](#_Toc11348)

[>AhNAC130-Arahy.T5AJQY.1 76](#_Toc26956)

[>AhNAC131-Arahy.TCTP66.1 77](#_Toc10299)

[>AhNAC132-Arahy.TY7WD8.1 77](#_Toc29784)

[>AhNAC133-Arahy.U16Y2L.1 78](#_Toc30199)

[>AhNAC134-Arahy.U487DX.1 79](#_Toc32038)

[>AhNAC135-Arahy.UCK419.1 80](#_Toc18855)

[>AhNAC136-Arahy.UK39BN.1 81](#_Toc27502)

[>AhNAC137-Arahy.UX5JN7.1 81](#_Toc10218)

[>AhNAC138-Arahy.V0X4SV.1 82](#_Toc9712)

[>AhNAC139-Arahy.V20ZHW.1 83](#_Toc1693)

[>AhNAC140-Arahy.V6VDUM.1 84](#_Toc13518)

[>AhNAC141-Arahy.V88TU5.1 85](#_Toc22595)

[>AhNAC142-Arahy.V9YDBD.1 85](#_Toc18363)

[>AhNAC143-Arahy.VCF3H0.1 86](#_Toc32765)

[>AhNAC144-Arahy.VI6QZG.1 87](#_Toc18089)

[>AhNAC145-Arahy.W5I9MA.1 87](#_Toc23040)

[>AhNAC146-Arahy.W8FFAE.1 88](#_Toc2264)

[>AhNAC147-Arahy.WB1HDB.1 88](#_Toc9443)

[>AhNAC148-Arahy.WF4CBH.1 89](#_Toc28026)

[>AhNAC149-Arahy.WPHD30.1 90](#_Toc14913)

[>AhNAC150-Arahy.WULW7H.1 91](#_Toc10814)

[>AhNAC151-Arahy.X47CQ0.1 91](#_Toc14716)

[>AhNAC152-Arahy.XB6K25.1 92](#_Toc1931)

[>AhNAC153-Arahy.XKF840.1 93](#_Toc7604)

[>AhNAC154-Arahy.XMHS8A.1 94](#_Toc16173)

[>AhNAC155-Arahy.YB9YM9.1 95](#_Toc16409)

[>AhNAC156-Arahy.YH9HLJ.1 95](#_Toc6587)

[>AhNAC157-Arahy.YSLF5V.1 96](#_Toc4688)

[>AhNAC158-Arahy.YV5C93.1 97](#_Toc2065)

[>AhNAC159-Arahy.YXGX3A.1 97](#_Toc30850)

[>AhNAC160-Arahy.YY4A03.1 98](#_Toc8576)

[>AhNAC161-Arahy.Z03JM7.1 98](#_Toc10566)

[>AhNAC162-Arahy.Z8VU36.1 99](#_Toc29865)

[>AhNAC163-Arahy.ZDQ75D.1 100](#_Toc27021)

[>AhNAC164-Arahy.ZM1I0C.1 100](#_Toc18472)

## **>** AhNAC1-**Arahy.05SHD0.1**

ATGGTTTTTGGTTGTTTTGGGGGCGACATGAACACCTTCTCCCACGTACCTCCAGGCTTTCGTTTTCATCCGACTGATGAAGAATTAGTTGACTACTACCTTAGGAAAAAGGTAGCATCCAAAAAGATTGATCTAGATGTCATCAAAGATGTTGATCTCTATAAAATTGAGCCATGGGATCTTCAAGAACTATGCAAAATAGGAAGCGATGAAGAAAATGACTGGTATTTCTTCAGTCATAAAGATAAGAAGTACCCAACAGGAACAAGAACGAATAGGGCAACAAAAGCAGGGTTTTGGAAAGCCACGGGAAGAGATAAAGCAATATACTCAAAGCAGCATTGCCTTATTGGAATGAGAAAGACTCTTGTCTTCTACAAAGGAAGAGCTCCTAATGGCCACAAGTCTGACTGGATCATGCATGAGTATCGCCTTGAAACCAATGAAAATGGAACTGCTCCGGAAGAAGGGTGGGTTGTATGTAGAGTGTTCAAGAAGAAAATGGCAACAGTGAGGAAAATTGGAGACTATGATTCACCATGTTCTTGGTACGATGAACAAGTTCCCTTCATGCAAGATCTTGAATCCTCATCCCCAATAAAGCCACCAATAATTAACAACAACCATTATGCTTCTTCATACAACTACCACCAGTTACAATTACCCTGCAAACCGGAATTCCATCAACTTATGCAATACAACAACATGAACATGCCACGTCACGACGACGCTGCTGATAATAACAACAACTTCCTCCAACTTCCTCAGCTTGAAAGCCCTAATGGTGGAATTAGCCCCTTCTTGCAACAACAAGATCATCATCATCAGCTATTGCAACAACAAAATTCCAACAGCAATTATCATCTTGATCAAGTAACCGATTGGCGAGTTCTCGATAAATTCGTTGCGTCGCAGCTCATGAGTCATGGTCATGATGATAATGATGACGATGGCCACAACAATAATAATAATGTTTCCAAAGAAGTAATAAACAGTTATTCTGATGCTTCAATTCTCCATGTGGCTCAACAGATTGCTATGCTGGCAAATGGATCGTCATCTTCATCATCATCAAGGAGGCCTCAAATTTCTCATCAGGAATATGCTGCTTCAACTTCCACATCAAGTTCTCAGATTGATCTCTGGAAGTCATCGTCATGA

## **>** AhNAC2-**Arahy.09VTBX.1**

ATGGCTTCAACAAATTTGCCTTCAGGTGCAAGCAAGAAGTTCAAACCTACAGATGAGGAACTCATTCAAGATTTTCTCCGTAACAAAATTAATGGGAGGCCTGTACCAAACTATGGAACCATTCTTGAAGAAAACGTTAAAAATTCTTATGACGGGAAGGACCTCTATTTCTTCACTACTCTGAAGAGGAAGTTTCCAACTAACAGCTTGAGAATGGTTCGCACCATCGGGTTAGGTTCTTGGAAAGGTGAAGACATCAAAAAAGAGATTATGGCCAATAAAACTAACCAGCATATTGGAATGAGAAAACGATATCGCTTTGAGAAGAGTGGTACTAGCCATGATGGTGGATGGATCTTACATCAACATAGCATTGATTCTTCTTTGTTACCAAATCCTTCCAATATGAATAATTATATTTTATGCAGATTTAGAATGAATAACATTAAATCTCGTCAAAAGAAAAGAAAACCTGTGGCTCCTAAAACTATGGCTACTCTAATGCCGATTGATGAGATTCAAAGAGAACTAACAAATCAATTAGAGAATAATAATGGAGATGAAAAGATTGATGAAATTCAGAGAGAACTAACAAATCAATTAGAGAATAATAAGGATGAAATAAAGGTTGAGGCTTTGGTTACTACTATTAGAATTGAGGCAAACTATGACGAAATGGAAGATGGTGATGATGATGAAAATCACAATGATATGACATAG

## **>** AhNAC3-Arahy.0UTR6Y.1

ATGCATCGCCTCCTTTGGTCGCGACACGAGCCTGTCGTAGTTAACAAAGGCAAGGAGCCGCTGGATTTGCCACCAGGTTTCGGACTCCACCCAACAGATGAAGAAATCATCACTTATTACCTCACCGAGAAGGTCATGAACAGCAACTTCAGTGCAACTGCCATAGGTGAAGCCGATTTGAACAAATCCGGACCTTGGGATTTACCAAAAAAAGCAAAGATGGGAGAGAAGGAGTGGTACTTCTTTTGTCAGAAAGGTGACATCACAGATTGA

## **>** AhNAC4-**Arahy.0V68XN.1**

ATGGGTCTTAGAGATATTGGTGCTTCATTGCCACCTGGGTTTCGGTTCTATCCAAGTGATGAAGAATTAGTTCTTCACTATCTTTACAAGAAGATCACAAATGAGGAAGTTCTCAAGGGTACTTTGATGGAAATTGATTTGCACACTTGTGAGCCATGGCAGCTTCCTGAGGTGGCTAAGCTCAATGCAAATGAATGGTACTTCTTCAGCTTCCGTGACCGCAAATACGCCACCGGGTTTCGCACCAATCGCGCCACGACATCTGGCTATTGGAAAGCGACCGGCAAGGATCGTACGGTTCTCGATCCCCTCACCCGCGAGGTCGTAGGGATGCGGAAGACTTTGGTGTTCTACAAGAATAGAGCCCCAAATGGCATCAAAACTGGTTGGATCATGCATGAGTTTCGCTTGGAGACCCCACACATGCCACCTAAGGAGGATTGGGTTTTGTGTAGAGTGTTTCACAAGGGCAAAACAGACAATAGTGCCAAACTAAGCCCACAATTCATGTATGAGGCCACACCTTCATCCCTAACTTTGGCTTCATCATCATCATCCCCACCAACAAACCAAACAAATTGCAACAATTTGCATGTTATTGGGTATAACCAACTTCCCAATTTCTCATCATCATCATCACCAATGGCAATCCACCATAATCATCATCATCATCATCATCATCAAAACCAAAACGGTTCTTCCTCTTTGATGAATCTCCTTCAATTTTCCACTAAGGAAAATAGTACCATTACTCAACTAAGTCCCAAAGGTGGTGGTGGCGGCGGCGGCGGCGACGACGGCGGCTATGGGTTCATGTGGGACATGGATCTTGAGGAAAATAGCTTCCATGATGGTGGGGTTATTGCATCAAACTTGAACGACATGAGATTTGAGGTTGATAATAACACTATGGTTATGTTGTAG

## **>** AhNAC5-**Arahy.12HUMH.1**

ATGTCTTGTATCTATAAATTAACCCACACCCACTTTTCTCTGTCCTCACACATAAAACAACACTGCTCTTTGTCTCATCCTTCAAATCCCTCTATCTTCTTCTTCCAAAGGGGAGTTCCACTCAAACCCCCCTTCTTCTTCTTCTTCTTCTTCTTCTTTGAGATGGATAACAGGTTGGCCACAAACTCCTCTTATGCTTCTCTTAGATTGCCCGTTGGCTACAGATTCTGCCCCTCTGACGAGGTTTTTGTCTCTTGCTACCTCAAAAACAAGGCCCTTTCAAAAACATTGGATTTTGATGTTGTTCCTGTCTTCGATGTCTTCAACACTGAGCCCAAGAATCTCCCTTCAGGAGGAAAGGTGTTTCTGGAGACAAAGTACTTTTACTTTGATCTGAAAGAGCGTGTGTTTGAAGACAATAACAAGATTGAAGCAGGGAAAGGGCACTGGAAAAGGGTGGGGAAAGGGAATCAGGAGCTTCTAAATAACAACAACAAACTCATTGGGTTCAAGACCAAGTTTGTTTTTTGGAGGAAGAAGAACCGCACTCAATTTCTTAAAACTAAGTGGGTTATGTTTGAGTTCCGTGTTTTTCTCAACCCCTCTCAGATAATGTCATCATGGGCTGGCTACAAAATATATCTGAAGAAGGATAAGAGGAGGAACAAGAAGGCAAAGTTTTCTTGCGAGGAAAGCAGTGATGATGATGAAGAAGAAGAAGCAGAAAGAGCAAGTGAAGTAAATTTTGCAGATGAGATAAGTGGAATTAACACAGGACCTCTTTCACCAACTTCATCTAATGAATCCTCTGTTACAAATTAA

## >AhNAC6-Arahy.13C0EN.1

ATGGCCGGATCATCCTGGTTGGTAGACAAAAGTAGAATTGCAACCAAAATAAAGAGTGCATCTGGAGCAAGTGGGAAAGTTTTATGGAAAAGCAATCCTACCAGAACTTGTCCGAATTGTCAACATGTTATTGATAACAGTGATGTGGCACAAGAGTGGCCTGGATTACCAAAAGGTGTGAAATTTGATCCATCTGATCAAGAAATAATATCGCACTTGCTTGCAAAAGTTGGTGCAGCAGGTTCAGAGCCTCACCCTTTCATTGATGAATTTATTGCTACTCTTGAAGTGGATGATGGAATTTGTTATACACATCCTAAACATTTACCAGGTGTCAAGCAAGATGGTAGTGCTACACACTTTTTCCACAGATCAATCAAGGCTTATAATACCGGCAATCGAAAGCGTCGGAAAATAAATGACCAGGACTCTGGCGATGTCCGTTGGCACAAGACTGGAAAAACTAAACCTGTCGTCTCGGACGGGGTTCAGAGAGGCTGTAAAAAGATTATGGTTCTATATATGACTTCAGTTAGAGGAGTAAAAGCTGAGAAAACTAACTGGGTTATGCATCAATATCACCTCGGGACAGACGAAGATGAAAAGGAAGGAGAGTATGTTATCTCTAAAGTGTTTTACCAGCAACAAGTTAAGTTTGGTGAAAAAGATGATCATGATGTTCCTGGAACCAATGAAGCAACTGTTGTGAAAGATGATCCAGTCACTTCCAAATCTCTGACTTCGGAACCTCCTCATAGTGAAAAGCAATGTTCAGATCTCGACATAGGAGAAAAAACACATCAGATTCTTCAGGGTCCTCAGACAGATTGTGTAGAAGACATTCAAGTCGAGTGTGAAGAGATTGTAAAAACTGATGTATCCATGGCAGATGCTCAAAATAATGAAGGAATGGATAATGTAGAAATAATGCTGACGGAGAACAAAAATGGTGGGACAAATTTGTTAGATTCACAACAACTTGTTGAAGCATTGGCCTTGTGTGATGATCTCCTCCATAGCCAGTGTTCCAATAAGGATGATGAAAATGAAGAACACAAGGAGCACTTGAGTCTTTCCATCTATGCTCATCTAGGACCAGAGCATCTGAAGAAAGATCTTGAAGAGTGCCAAAACCTTACTCTTGATCCTGCAAACGTAGAGCTCGAGACACCACCTTCAGAGTTTCGACTAAGTCAGCTGGAATTCGGTTCACAGGATAGCTTTGTTTCCTTTAGCGGCGGCAAGGCAGTCGATTAA

## **>** AhNAC7-**Arahy.1BXK41.1**

ATGGGGGAAAGAAATATTGAGATGGAGAATAAGATTGAAGATGAGATGATGCCAGGTTTCAGATTTCACCCAACAGATGAAGAGATTGTTGGTTTTTATCTAAAAAGAAAAATTCAGCAAAAATCTCTCCCTATTGAATTGATCAAGCAAGTTGATATCTATAAGTATGAGCCATGGGACCTTCCAAGCCTTCATGAAGATCCAAATCATCATCATTACAATAATAATAGTAGTGGGTTCTCATCTTCTTCAATTATGATGATGCAACCAAACATCATGGCAACCTCAGATGATGATGATTCAGGTGTAATTACAACAATTGCTGGCTTCCCATTCAATTTGCCTCCAAATGATGATGATGCTGCTTGGAATAATAATATTAAGCCTAATACTACTCTGCCATGGGACTACTTATCAGACATGTCCACTACCTATTCCACTAATAAATCTTACACT

## **>** AhNAC8-**Arahy.1GPE0T.1**

ATGGCACCAGTTTCATTACCCCCAGGTTTCAGGTTCCATCCAACCGATGAAGAACTTGTTGCTTATTACCTCAAAAGGAAGATCAATGGCCGTAAGATTGAGTTGGAGATCATTGCTGAAGTTGATCTCTACAAGTGTGAACCATGGGACTTGCCAGGGAAGTCATTGTTACCGGGGAAGGATTTGGAGTGGTATTTCTTTAGTCCTCGAGATAGGAAGTATCCAAATGGGTCAAGAACGAACCGTGCAACAAAATCTGGGTATTGGAAGGCGACAGGGAAGGACAGAAAAGTAAATTCACAATGTCGTGCTGTGGGTATGAAGAAAACCCTAGTTTACTATCGTGGAAGGGCACCTCATGGCTCTCGCACTGATTGGGTTATGCATGAATATCGTCTTGATGATAGAGAATGTGAAAATGCTTCTTCTGGCTTGCAGGATGCATATGCACTTTGTCGTGTGTTCAAGAAGAGTGCAGTGATAACCCCTAAAGTTGATGAGGAACATCATCATCACCATCACTATGTTAATGCTAATAATCACAATAATAGCAGCCATGCTTTGCCAATTACAAGTGATCAATCGTCAAGTATGGAGTTATATTCTGAAGGAAGGGGTGAAGATTTGGATAATAGCTCTAATTATTTGGTTCCCATCGATACTACTTGCACACTACCCCTCAACAACATGGTGATGAACAATAATAATAGTGATGCTTCTTTCAATAGTAGGGATAATAATGGGAAATGGTCACAATTTGTTTCAGAAGATCCATTGTTCAGCTTTCCAACTTCCTCCTCATCATTTGCTAATAGTTATGGATCTATAACATATCCTCCATCCAAGGTGGATATAGCACTAGAGTGTGCAAGGATGCAACATAGGTTCACCATGCCTCCATTGGAGGTAGAGGACTTCCCTCATGTTGGAACCTCGGAGCTGAAAATGACAGAATTAACCTCGGGTGCCGCATCCGCCGTGCACGGAACCCGAAACGAAACGGATATCTTGCAGGAAATTCTTTCGGTTGCTCATGCTTCCCAGGAGTTGATAAACCACTCCAGCTACTCATCATCATGGGGTGGTGATGGTGGTGGCAACCATGAAAATTGTGCAACTCATGGAGATGATTTCACTTTCATGGTTGGTAGCACTAACTACAATAATAATAATTTGAATGACATTAACTCCATGAGATATGTTGATAGAAATTGGGAAGATCCAAACAGTTCAAGATCCATTGATATTGGATATTTGGATGAAGAATTTAAGGGAGAGAGGATGGTAGAGAATTTAAGATGGGTTGGAATGTCTACAAAAGATTTAGAAAAGAACTTCACGGAAGAGCAAAAGATTGTTCCAATAGAGGATATATCAAGCTTCCGGACAAATAATAAAGAAGAAAATGAGGTGCAAGAATCTGAGCAACACCATAGCAACAAGGAACTATTGATCAATGATTTCTCATTAGGGTTCAACCCTAATAACAACAACAGCGAGAACTTTCTAGATGATGATCATAACAACATGGATAACGATGATTACTCAAGTTCTCCAAGCTTTGAAGTCATTGAGGAAATAAGGGTCAGCCATGGATCAATGTTTGTTTCGACTCGCCGCGTCGCTGACACATTCTTCCACCAAATAGTTCCTTCACAAACCGTCCAGGTTCACCTCCTCAATCCAGTGATAACAAGCAATGAAGAAGAGACATTGATGATGATAATGGAGAGGAATCAAGGGTATTTCGGGGATTTTCTTTTCAGGACAATAGCAACTGCGTTTGTGTTCATCTTTGCACTTGTATTCGTGCATTGTGATTATTTGAAGGAAGAAGTGGAATTGGTGAAGAGAAAGAGATCATCACAATCATCATCTAAGATCATGAAATGGAGCAACAATAATAAGGTTTGGTTTGTTGGTTTCAAGAGTAGTGAGAAGGGATTTGGTGCAATTTTAAAGAAAATAGGGATTTTTCTCACAATATCTTTGGCTCTTTGTACCATGTGGGCTAACCATGTTATTGTTAACCCTTGA

## **>** AhNAC9-**Arahy.1I167B.1**

ATGGGAGGGGCATCACTGCCACCTGGATTTCGTTTCCACCCAACAGATGAAGAATTATTGGGATATTACCTAAAAAGAAAAGTGGAAGGGCTTGAAATTGAGCTTGAGGTTATTCCTGTGATTGATTTGTGCAAGTTTGATCCTTGGGAATTGCCTGAGAAATCATTGTTGGCAAATAGAGACATGGAATGGTTCTTCTTTTGTCCAAGGGACCGCAAGTACCCAAATGGATCAAGAACTAACAGAGCCACCAAAGCTGGTTATTGGAAAGCCACTGGAAAAGACAAGAAAGTTGTGTGCCAATTTGATACTCCTTCCACTGTCACAGGATATAGAAAAACCCTTGTCTTCTACCGTGGCAGAGCCCCTTTAGGTGACAGAACTGATTGGCTCATGCATGAGTATCGCCTCGCCGATGATCTCGGCCTATCATCTACATGTTTTCAGGGTGGTTATGCCTTGTGTCGGGTTATTAAGAAGAATGAGAAGGTGAACAATGAGAATGACGCGTCAATGAGATTCTCCAATGAGCCCTTCGCCATTTCTGCTGATGCTTCATCCTCTCAACCAAGTTATTTGAACAATGAGAGTGTTTACTCAAGCCCCAATGCTTCTCCACACAATGTGGACTCTAACCAAGCTTCTATAAACACCAGTTCTTCATCAGAGTTTTGGGTGTCCCCTGATCTGATTCTTGATTCTTCAAAGGACTACCCGCAACTACAAAATACTTTTACAAGGTGTGACATACCAAGTAGTACAATGACACCATGGCTCTCATTGGATCAACCTGAAATTTCATCTAGTTCATCATACTCAAATTTTAATGGGTAA

## **>** AhNAC10-**Arahy.1IJJ7X.1**

ATGGCTAGAAGTGCCACAATTCCATTTCCAATACTTGATTTCATTCCTGTTGGATTCAGGTTCAAGCCAACAGATGAAGAGCTTGTGAGCTATTACCTCAATCACAAGCTCCTAAATGACAATTTTCCAATCGATATTATCCCTGACATTGATCTTTGCAAGGTTGAACCTTGGCAAATTCCAGCATTATCAAAGGTAAAATCGGATGATCCAGAATGGTTTTTCTTTAGTGGACGTGATTACAAGTATGGAAAGAGCAAAAGATCAAACAGGGCAACCAAAGGAGGATATTGGAAAGCCACAGGACAAGATAGATTCATAAAGGAAAGAGGAACTATGAATGTAATTGGGAGCAAGAAGACACTTGTTTTCTATAGTGGCCGTGTTCCTAATGGTGTCAAAACCAATTGGGTTATCCATGAGTATCATGCTACTACCTTTGATGATAGCCAGAGGAATTTTGTTTTGTGTCGCTTGATGAAGAAAGTCGAGAGAAAATCCGAAGATGGAACTGATGCACAAGCCTGTGATGAGGGGGAACCTAGCACTCACATGGAAGAAGCAGATGAGAGTGTCTCAACTATGTTTGATTCGCCAGATGTGGACATGGATTCAATCTTCCACACACTGCCTCAAGACAGATCATCATCACAGCATTCTCCAGTCGGCATTGAACAGCAAGAATCCTTCCCATTCTCCCCATCTGAAAATTATTACCTTGTAAATGAAGATAGCAGTATGCATATACAATTCGAAACAAACGAAGAGAAGCAAGATGCTGAGAAATTTGCGGATTCGATTTTGGATAGTGGCAATATAGCTATGTTTGAAGAAAGACAGCAGCATCATACTTTCATGAATAATCACCTCCGCTCGGTCCCATCGATGAGGGTATGCTATGAAAGCAGTGACACAGATGCTGAAGTAGTCTCTAGACGGGCTGATTCAAGAGAATACCATGTATCAAAAATGGTTCAATCATCACATAGTGCTGCGCGCACAGATAAAACTAGAAGTATCTCTTCAGAAGACTTTTGGGGAGTGGATTCATCTTCATGTGACTCAAATGCAGATAAACCTTTTGAGATCAATTCTATTGAAATTTCTAGTCCTCCACCGGCTCTAAGTGGATCGAAAAATCAATATAATCCGAGACTATCTCAAACACATAGGAAGGTTTCAAGCAATGCAATTCCCAATCTTGAGGATAAGAAGAAATTGACCACTGTGGAACAATCAAGAAGAGATCAAGAAAAAGCTCGAAAAACTAGTCCAGGAAAGAAGTTAGAAACCAGAAGCTCTGATGTTAATAGAATTGGTAGTTTCATCCACCTAGAGCCGTGTTCGTCGAGCGAAAGCCTGACTCCACGAGCGGTATACCTTGTTAATGTAGTTATTGGGATTTTGTTGCTTCTAGCCATTAGTTGGGATGTGCTATCTTGTTAG

## **>**AhNAC11-**Arahy.1P6DI5.1**

ATGGGATCACCCGAATCAAATTTGCCACCAGGTTTTAGGTTCCATCCAACGGATGAAGAACTCATTCTTCACTACCTTAGGAAGAAGGTAGCATCCATACCCTTACCTGTTTCCATCATCGCTGAGGTTGATATCTACAAATTGGATCCATGGGAATTACCAGGAAGGCCTCCAAAGGGTGTCAAAACCAATTGGATCATGCACGAATATCGTCTTTCGAAATTCTCAGTATCTTCACCGGAGGAATCACCGTCGATTGAAGTTCAGGCTGCAGAAGAAAATGGTTTATTCAAGAACACCATTTTAAGGAGTCCAATTCCAACACCGTCGCCATCACCACCGCCGCCGCTGCCGCAGTCACTGCTCTCTCAAAAATCTGTGTCCTTCTCAAACCTCTTAGATGCCATGGACTACTCCATGCTCAGCACCATCTTATCTGAGAACAATAACAACAGCACCCTTGATCAGCAACAATACTCGCAGATCAACACCAACCAATTGAACCATTCATCGAACATGGAGAACACTAGTAACAGCAACATGATGGTGATGAGGTCAAAGCGCCAGATAGAGGAGGAAACAACAACGGTGTTGCACCCATCAAAGAAGTTCCATCACCAACTTATGGGCTCTTCTTCTTGCAGCTTCCCTAATAACATTAACAACACAAACACTGCACAATACGAGAACCCGCAATGGAACTACCTTGTCAAGCAATCCTTCTTGAACCAGCACTTACTTCTCGCTCCTCATCTTCGATTTCAAGGATAG

## **>**AhNAC12-**Arahy.1Q9HM8.1**

ATGGATATGGAATCATGTGTGCCTCCAGGATTTAGATTTCACCCAACAGAAGAAGAACTTGTGGGGTATTACCTCAAGAGGAAAATTAACTCCCTCAAAATTGATCTAGATGTTATAGTTGAGATCGATCTCTACAAAATGGAACCATGGGACATACAAGGATGGGTTGTGTGTAGAGCATTTCGAAAACCAAGTCCAAGTCATCAAAGGCAATTAGGGTTTGATCCATGGTGTAGTAATCATCATCATCAAGCACATTATTTCAGAGATCAAAGTAGCTATGGTGGAAGGCCCTTATCAATCACAGATCTTCTAACTTCAGAAACTCATCTTCTGAATCACCCTACTGAAGGTACAAATTTTAGTCATTCCTTCGGTTCAGATCATCATCATCATCATCAAGAACAAGAGTTTGTAATATCAAATAATCATCAACAACTCATTGAGCTTCCACAGCTAGATAGCCCAACAACAACAAGTTTTGCAGTCAAAGAATCATCATCCATTAATAATAACAATGAAGAGTATTGCAGTGATGACAGGAACAACAACAACAACAACATTGATTGGAAAAGCTTGGATAACCTGTTTGCTGATACTTCTAATTACTTCTCATCAAATCCAAACATGTCCCAATTCATGACCATCAATCATCATCTAGGTTGTTTCCCTGGTTCATAA

## **>**AhNAC13-**Arahy.1UE66A.1**

ATGGGAGCCGTTGTTGACTGTTATCCGCCGCACGCCGGCGAGGTTGCAGTTTTGTCTCTCAATTCGCTTCCCTTAGGTTTCCGATTTCGACCTTCCGACGAGGAGCTTGTTGACTATTATCTGAGACAGAAAATCAACGGAAATGGAGAAGAAGTCTGGGTTATTCGAGAAATCGATGTTTGCAAATGGGAGCCTTGGGACTTGCCAGATTTGTCGGTGATAAGAAACAAGGATCCGGAGTGGTTCTTCTTCTGTCCACAGGACCGGAAGTATCCAAATGGTCACCGGTTGAACCGAGCAACGAATCATGGGTACTGGAAGGCCACAGGAAAAGATCGTAAGATCAAGTCAGGTTCCACCTTGATTGGGATGAAGAAGACTCTGGTGTTCTACACAGGTCGTGCTCCCAAAGGGAAGAGAACCAATTGGGTCATGCATGAGTACCGCCCCACCCTCAAGGAGCTTGATGGCACCAACCCTGGACAGAATGCGTATGTACTCTGCCGGTTATTCAAGAAACAAGATGAGAGTCTTGAGGTTTCAAACTGTGATGAGGTGGAACAAACAGATTCGGCTCCCATGGCGGCCAATTACTCCCCTGAAGAAATACAGTCTGATCAGGCTCTGGCTGAAGTATCGCCGTCTCAAGTTACAGATGAGAAGCACCAGGGTGTTATCCCTGAGAACTCTGAGGAAGCGGTTTCCAACGTTATAACCTCTGCTGATTGCCATAGTGACGGATATGATGCTTGTGAAAGGCGAAATCAAGCTTTTGAACTACCTGCTGAGGACATTCCGCCGTTGAATTGGGACATATTCAATGACCCCGAAGACAAGATATTTGATGACAAATTATTCTCCCCAGTCCATAGCCATATTCCACCAGAATTTTACTACCAAGCAAACAATGAGACAAATATTGCAGACATCTTAAATTCTGTCAATTGGGATGAGATCTCCTATGAGGATCCCTATAGTCAAGCACAGAACAACTTTTTTAATAATGTTAAGCAAAGTGTATCAGGTAGCGAACCAGATGCAGGGCTGACCAATATGACATGTGTACACCCGACGAATGTTGTTTATCCCGAGGAGGCAATTCACAGAAAGGTTGCTTTGGCAACAACTCCGCAATTTTGCAGCACCTTCACGTCTGACTTCAGTGCTGATGAGCAGAAGAGCAGTGTCGCGTTAATTCAAAACAATTCCCAGATGGCTTCTTTTCCGGATGCCAGAACAGTCCAAGTGTATAACGTATTCAATGATTATGAGCAGCCGAGAAACCTTAATACCTATGTTAGTGGTGATACTGGAATCAAGATAAGGACTCGACAAGTGCGAAATGAACAACCAGCAATGATCTTTACAGATCAAGGTAATGCAGCAAGGAGAATCCGATTGTTAAAGCAGTGTGCAGATGTCTCAAACAAGATGGCAGATGATGGGAGTCCTAAACAAGAGCATGATTCAAAACCAATAATTGCAGGGAACAAAAACAAAACTTTCAAAAGTCATACTGCAGATAAGCATGATACTGCTAATGATCTGAATGAACGCCAGGAGAAAACTGAATCAACTGATAAAAGAAACATGATATCTAAACTTGCTAAAGGAGGTTCTTCCATGTTGGGGTTGAAGGGATTATTGCGCAGAAGGCTTAGTTACATATCAAAGGCTTCCTCCAATTTCAAAATGTGGTCTTGTGTTGTTGTGGCTTCTGCCTTTGTATTGGTCTCGTTTGTGTTCTTTGCTAACATATGGGGATATATTAACTTATGA

## **>**AhNAC14-**Arahy.2275VI.1**

ATGACAGAAACTACAATTCTACCTGTTGGATATAGGTTTCGTCCAACAGAAGAACTTTTAGTTCACTATCTCAATAACAAGCATTTGAGAAATGATGCACAGATCAAGAACACTGTTTCCCAAATTGATCTTTATAACTTTGATCCTTGGGATTTGCCAGAACAATCGAAGGTGAAATGGGATGATCAAGAATGGTTTTTCTTCAATGAATTGAAACACATAAAAAACAAGCGGTGTAACAGAAAAACTAACGCCGGTTATTGGAAGATCACCGGAAAAGAGCGGATCGTCAAAAGAACAGGGATAGACAATGTGATAGGTACAAAAAGAACACTAGTTTTCTATAAGCGTCCACAGTGTCAAAACCAATTGGGTTCTTCATGA

## **>**AhNAC15-**Arahy.23ZMCQ.1**

ATGATGATGATGACTACTACTTCTACTACTGCAGATTATGAAAGCGTGAAGCAGCTTCCTCCTGGGTTTTTGTTCTCTCCAACGGATGAAGAACTTGTCCTTCACTTTCTCTATGCCAAGGCTTCTCTTTTGCCATGCCATCCCAACATCATCCCTGATCTTGATCTCTCTCTCGCTCATCCTTCCCAACTCAACGATAAAGCGTTGTCAAGCGGAAATCAATACTATTTCTTCAGCAAAGTGAAGGAAAAAAGAATAACAGAAAATGGGTATTGGAAGGAAATAGGTGAAAGTGAAGCAATATTGTCATCAACGGTTGAGAAGAAAGTAGGGACAAAGAAGAACCTTGTATTCCACATAGGAGAAGCTCCACACGGCATTGAAACCAGTTGGGTCATGCAAGAATATCATATTTGCCGATCCTCTAACATTATTTCTACAAGTAGAGCCAGAAGAAAACACGATCATCAAATTTGGAGCAAATGGGTTTTGTGCAAAGTGTATGAAAAGAAGGGGTCCGTACGAGGTGTAAACTACTGTAGCGACGATGATGACAGTGGGACAGAGCTATCTTGGCTTGACGAAATTTATCTCTCGTTGGATGATGATCTCGAAGAAATTAGCCAGTCAATTACCCATGCCTGCGTCCTGTTGATCGGCTTCTCCCTTCAGCTCCGATCGAACATTGCACCGCCAACAACGACTAACCAACCAGTGCCGCACTCTCTTCCGCACACAGCGCAGCCGGTTTTGAATACTTCAAAATGGCAGAATCACAAGGAAAGCGTGTTTCAAGCGAGGATCCTCTTTTATGCACGTCACCTAGCCGAAATTCATTAA

## **>**AhNAC16-**Arahy.24LKGZ.1**

ATGAATAAGATGGATCTGATAGATGCGAAGCTGCAAGAAGAGCATCAATTGTGTGCATCATCGTTGAAACAGTGCCCCGCTTGTGGACATAAGTTTGAAGGCAGCAGCGGGAAGAAGGCGGAGTGGGAGTGGGTAGGTCTGCCAGCGGGAGTGAAGTTCGATCCAACAGACCAAGAACTGATAGAGCATCTAGAAGCAAAAGTAGAGGCAAAGAGATCGCACCCTTTGATCGATGAGTTCATTCCCACCATTGAAGGAGAAGATGGAATCTGTTACACCCATCCCGAGAAGCTCCCAGGTGTGACGAGGGATGGGTTGAGCAGACACTTCTTTCACAGGCCATCAAGGGCGTACACCACTGGAACACGGAAGAGAAGAAAGATTCTTCAAAACGATGAGGCGGAGGCCGAGAGAGGAGAGACACGGTGGCACAAGACCGGTAAGACAAGGGCCGTTATGCTCAAGGGAAAGCAGAAGGGGTGCAAGAAGATTCTGGTGTTGTACACCAACTTCGGCAAGAACAGGAAGCCCCAGAAGACCAACTGGGTCATGCATCAGTACCACCTCGGACTCCATGAAGAGGAGAAAGACGGGGAGCTCGTCGTCTCTAAGATTTTCTACCAAACTCAGCCGAGGCAATGCAGTTGGTCTTCTTCTTCTTCTTCTTCTTCAATTACTGCTGTTGCTCCCCCTGTCAAAACTAATAATGACACCTGTCCCGTTCTTGGATTCCCTCCTATGGAACATTTCAGCAGCTTCATCCCTCTCAGAAAAACCCTCCATAATGAGGAAGTTGGAATAGGAGGGGAAACTTGCACACCAGCGTCACATATTCCTTCATCAAATCCTGTTGGAGTCTTCCATCACAACACTTCCATCATCCTTGACGACCTTATCTCCGCTAGATTCATGACTCCTCCTCCTCCTCCTCAGTTCCACCAGCAGCATGATAATAAAGTAGTAGGAGGAACCTCTGCTTCTGGTTTAGAGGAACTCATCATGGGCTGCACTTCAACTTCAACCACTCATAATATCACCAAAGAGGCATCAATGTCAAACACAAACCCACAAGAAGCTGAGTGGTTGAAGTACTCTTCTTATTGGGCTGACCCTCAGCCTCAGCCTCAGCCTCATCTTCATGGGTAA

## **>**AhNAC17-**Arahy.2C699W.1**

ATGAAACTAAGGAATAATAATAATTATGGGGAGCAAGAAGATAGATACTTGTTCAGCTCAAAGGAAGTTAAGTATAGAAACGGTAACCGAATGAACAGAATAACGAAATCTGGATATTGGAAAGCAACTGGATCAGACAAAAGAATAATTTCAACATCATGCAATAATAATAATAATAGTAATATTGTTGGGATAAGAAAAACTCTTGTATTCTATCATGGAAAATCTCCAAATGGCTCTAGAACTCATTGGATCATGCGTGAGTATCGACTTGTCACTACTCATTCTAATTCATCCCAGATAAATCTAAACATTTACTCCCAAGATTCTCTTGCAAAGCATGCTGGCATGTTTATGTTTATGCTGGGAACAGACAACTGGTTGTTTGATAAATCAAGAAGGCTAAGCCTTGGTGCAGATAAACTTGATGGAATTTCGCCAGAAAGTTTATTCGAGGACAAGTTCAATGAGTTGAGTGTGAGCAAAGATCTAATACCAACTGGTATTTCTCCGGAAAGAGAGTTACCAGAAAAATTACTATCAGTATTCATGTGCAACTACTTCAAAATCTCTCATGCAGTGGCAAAAGCCTATAAATACGTTGAAGAAGAAGATTACATATGCATAACACTTATAAACTATGTGGGGTACTAA

## **>**AhNAC18-**Arahy.2FYA1K.1**

ATGCAAGGTGGATTAGAGTTACCGCCAGGGTTCAGGTTTCACCCGAGCGACGAGGAATTGGTGAACCACTATCTCTGCAAGAAATGCGCAAAGCAATCAATTGCTGCTCCAATAATTAAGGAAATCGATTTGTACAAGTTCGATCCGTGGCAGCTTCCAGAGATGGCGTTGTACGGAGAGAAGGAGTGGTACTTCTTTTCGCCGAGGGATAGGAAATATCCGAACGGATCCCGGCCGAACCGGGCGGCTGGGAGCGGGTACTGGAAGGCGACCGGGGCGGACAAGCCGATAGGGAAGCCGAAGGCGCTAGGGATAAAGAAAGCGCTAGTGTTCTACGCCGGAAAGGCCCCGAAAGGAGTGAAGACTAATTGGATTATGCATGAGTACCGTCTCGCCAATGTTGACAGATCCGCCGCCAACAAACTCAACAACAACAACTTGAGGCTTGATGATTGGGTGTTGTGTCGAATCTACAACAAGAAAGGGAAGATTGAGAAATTCAACTCTGCCACAACAGGGTTGGAACAGAAACTACCAAAGTTTTCACCAGGGGAGATACTTCACTATGATCATGATCATGAGCATGAGACAAAGCCAAAGATTATCCACAATTTCTCCAACAATGAGCACCAATTGTACATGGACACATCAGATTCCGTTCCAAGGCTGCACACGGACTCTAGCTGCTCGGATCACGCGGTTTCGCCGGACGCCACCTGCGACAAGGAGGTGGAGAGCAACCCAAAGTGGAGCAATGAGCTAGATATGCAGCTGTTTGATACCTTTGATTTTCAGCTCAACAACTATGATAATAGCCTCCCAATGAATGATGATGACCTTTTTGGAAATCAGTTCCAAATGAATCAGCTCATGTCTTTCCAAGACACATTCTTGTTCCCACAAAAGCCATTTTGA

## **>**AhNAC19-**Arahy.2I3PJC.1**

ATGGGTCGTGAAACCCTTCTTCATCCACCACCACCATCCACCACCACCGCAACAGCCACACACACCCCACCACCGCCGCCGCCACTGCCGTCTCTACCTTCATTGACCCAAGCAGGACCATCAGGAGGAGTATCAGCATCTGCACCTTCACCATCTGCTTCACCTTCTTCTCCTGCTATTGTTGCTACTACTGCTGTGGCTACTGCAGTTGCTGCTCCTCCTACCTCTCTTGCTCCTGGTTTCAGGTTCCATCCTACGGATGAGGAGCTTGTTATCTATTACCTCAAGCGCAAGGTTTGTGGCAAAAGCTTCCGATTTGATGCAATTTCTGAGGTTGACATCTACAGGAGCGAACCCTGGGACCTTGCAGATAAGTCGAGGTTGAAGACTAGGGACCAAGAATGGTACTTCTTTAGTGCACTGGACAAGAAGTATGGCAATGGTGGGAGGATGAACAGGGCCACAAGCAAAGGATACTGGAAGGCTACAGGGAACGATCGTCCGGTTAAGCATGAACAAAGGACCGTGGGGTTGAAGAAAACTCTGGTGTTCCATAGTGGAAGAGCCCCAGATGGTAAGAGGACCAATTGGGTCATGCATGAGTACCGACTCGTCGACGAAGAGCTGGAGAGGGCTAGGTCTGGATCCTCTCAGCCTCAGGTGAGATTATTGAGTTTACAACTTGAAGTGAGTTTGAAACTTTGTTGTCTAGTAAACGTGGAAAGTTTAGTTTGCTATGTGCTCTACTACAGTATGGCCATTGTTATTATATCTCAACAGCTGGTTAATCATTTTTGTGCATTTCTCTTTCGGATGGAGAAGGATGCATATGTTTTGTGTAGAGTTTTTCACAAAAATAACATAGGACCTCCGAATGGGCAACGTTATGCACCTTTCGTTGAAGAGGAGTGGGATGATGCATCGGCATTGGTTCCTGGGGCAGAACCTGTAGAGGATGTTACCGTTACTGTTGCTCATCCTCTACGCATTGAAAGCAACGGTCGCACTTTATGCAGCGACAGGAGAAACAATGTTGCACAGAGAGACAATGAGGTAATTAATCTGGACCTTGTCTTTCTTGCCAATTTACAAGAATCATGTCACCCCTTTTATTGTTATTATTATAAAGAGAAGACTCTATCGTCAGTAAGGAGGATGGAGCTTCTAACTGGTGCTTCAGATAATTTGGTAAACTTCCTTGATTGTTTGGGTCAATTGACAAGGGGCTTGATAGTATCTAATGCTACTGACATGAAGTTGGAATTGCCAATGCTATTTTACTTCAGGAAAGTAGCCTCTGTTGCCCCTATGAGTATGCACATACTTACCAAAGAAGAGCAGAGAAGAGAAGCAAGAAGGAGATGGTCTATAAGGCTACGTGCCCAAGGGAGGACATTGAAGGTCTATGAGGGAAGGAAGAGAGGGAATGATCAAGCTTCAGAGGGTAGTGTGACATTGGCAATTCAACATTGGGTTGTCTGGGTCCTGGCTTGCCTCGGACTATCTATCAACCGGCTTACCATTAATTCCATATTCTATCATATGACTAGTAGTGGAAAGTGGGATACTCAATCTAACAACAAAGTTCCATTTGATGTGAACAAGCTTCCCATTGAGACTCAAAGTCTGCTAGCTGTCTGCAAAAGGGAGAGTATGGCCGAGTTTCCATCACCTGAAAAGGAGGATAACTCGAAGCGTCAGATTGATGAGTATCCTTTGCCACAAACAGAAAACACCAAGCCTATCTCCCAAATATACAAAAGGAGGAGGCATTATTTGAATGTCAACCATTCAAATGTCAACGGAGATTCAGTCCGAACCATCCAAGAACCGCCATGTTCATCAACAATAACCACCGCCGCAACGACGCTCCCGACGGCCACCACCACAGCCTCCACTGCGATAACCAACGTTGCACCCAAAAAACATTTCTTGTCTGCACTGGTGGAGTTTTCCTTGATGGAATCCCTCGAATCGAAGGGAAATCCATCCGTTCAACCACCAGAGTTTGATGATGCTTCCTTAGAGGCATCCGTGCCGCCAAATTGTGTTAAGCTCATCAAACGCATGCAGGGCGAGATTTACAAACTTTCCGAGGAAAGGGAGACTATGAGGTTTGAGATGATGAGCGCACAAGCAATGATTAACATGCTCGAGTCGCGCATTGAAATTTTGAGCAAAGAAAATGAGGAACTGAAGAGCATGATTAACAACAATCCTTAG

## **>**AhNAC20-**Arahy.2L619Y.1**

ATGGTTTTTGGTTGTTTTGGTGGCGACATGAACACCTTCTCCCACGTACCTCCAGGCTTTCGTTTTCATCCGACTGATGAAGAATTAGTTGACTACTACCTTAGGAAAAAGGTAGCATCCAAAAAGATTGATCTAGATGTCATCAAAGACGTTGATCTCTATAAAATTGAGCCATGGGATCTTCAAGAACTATGCAAAATAGGAAGCGATGAAGAAAATGACTGGTATTTCTTCAGTCATAAAGATAAGAAGTACCCAACAGGAACAAGAACGAATAGGGCAACAAAAGCAGGGTTTTGGAAAGCCACGGGAAGAGATAAAGCAATATACTCAAAGCAGCATTGCCTTATTGGAATGAGAAAGACTCTTGTCTTCTACAAAGGAAGAGCTCCTAATGGCCACAAGTCTGATTGGATCATGCATGAGTATCGCCTTGAAACCAATGAAAATGGAACTGCTCCGGAAGAAGGGTGGGTTGTATGTAGAGTGTTCAAGAAGAAAATGGCAACAGTGAGGAAAATTGGAGACTATGATTCACCATGTTCTTGGTACGATGAACAAGTTCCCTTCATGCAAGATCTTGAATCTTCATCCCCAATAAAGCCACCAATAATTAACAACAACCATTATGCTTCTTCATACAACCATCACCAGTTACAATTACCCTGCAAACCGGAATTCCATCAACCAATGCAATACAACAACATGAACATGCCACGTCACGACGACGCTGCTGATAATAACAACAACTTCCTCCAACTTCCTCAGCTTGAAAGCCCTAATGCTGGAATTAGCCCCTTCTTGCAACAACAAGATCCTCATCAGCTATTGCAACAACAAAATTCCAACAACAGCAATTATCATCTTGATCAAGTAACCGATTGGCGAGTTCTCGATAAATTCGTTGCGTCGCAGCTCATGAGTCATGGTGATGATGATGGCCACAACCACAATAATAATGTTTCCAAAGAAGTAATAAACAGTTATTCTGATGCTTCAATTCTCCATGTGGCTCAACAGATTGCTATGCTGGCAAATGGATCGTCATCTTCATCATCATCAAGGAGGCCTCAAATTTCTCATCAGGAATATGCTGCTTCAACTTCCACATCAAGTTCTCAGATTGATCTCTGGAAGTCATCATCATGA

## **>**AhNAC21-**Arahy.2MPS86.1**

ATGGAAAACATTTGTTCAGAGGTTGAGATGGATTTGCCACCAGGATTCAGGTTTCACCCAACTGATGAAGAGCTTATAAGTCATTACCTTTACAACAAGGTCATTGACACTAACTTTTCAGCCAGAGCCATTGCTGAGGTGGACTTGAATAGGTCTGAGCCTTGGGATTTGCCATGGAAGGCGAAAATGGGTGAAAAAGAATGGTACTTTTTCTGTGTAAGGGACAGAAAGTACCCAACAGGATTGAGGACAAACAGAGCAACAGAAGCAGGGTATTGGAAGGCCACTGGAAAAGACAAGGAGATATACAGAGGCAAATCACTTGTTGGCATGAAGAAGACCCTTGTCTTCTACAAAGGTAGGGCTCCCAAAGGTGAGAAATCTGATTGGGTCATGCATGAGTTCAGGCTTCATGGTAAATTCAATCCCCACAACCTCCCCAAATCTGCAAAGAACGAGTGGGTGATTTGCAGGGTGTTTCAGAAGTCTTCAGCCGCCAAGAAAATCCATCTTACCGGGATAATGAGGTTGGACTCTTCTGTTTTCTTGCCACCATTGGCAGATTCCTCATCATCACCTTCCAACACTGCTACTACAGCACCTTACGTGCCCTGCTTCTCCAATCCAATCATTCACAACCAAGTTGGGATCTTTGATCCATTTAGCAACACCCCTTTTGGTGCTGATTCATTCTACACTTCTCAAGGGATGCCAATGCAACATGCTCAACCACCAAGTTGCTACACCACTCAGGACCATTCAATTCTCAGAACCTTGCTTCAAAACAATTCTTCAAACCTCAGGAGTGGTTTCAAGCCTGCAGAGAGGGAAATGGCCCATCATCAAACTTCTCTTGTTGATGCCAACAACAACAACAACAACAATGGAATCACTTCTGTTGTTGCCCCACAGGACCTTTCTAGCCTCTGGAATTACCAGGTTCAGATCAAGTAG

## **>**AhNAC22-**Arahy.2P3Z86.1**

ATGGTGGATAGGGATTCAAGTGAAGCACACATGTCAATAGCCGCTTCTTCCATGTTCCCTGGCTTCAGGTTCTGTCCCACGGACGGTGAATTAATCTCTTATTACCTCAGAAAAAAATTGGACGGTGACGAGGACAGTGTTCAGATTATTTCGGAGCTTGAGCTTTGCACCTTTGAGCCTTGGGATTTGCCTGAAAAATCGTTCATTAAATCAAATGATGAGTGGTTTTTCTTCTCGCGACGGTGGAGAAGGTATCCCAATGGTTCACAGAATAAAAGGGCAACTAAAAGTGGTTATTGGAAGGTCACAGGAAATGAGCGACAGATAGAGTCTGGTCAGAATGTGATTGGTACCAAACGTACTTTGGTATTCCATGTCGGTCGAGCTCCCAAAGGCGAAAGAACTGAATGGATTATTCATGAATACTGTATCAATGACAAATTTCAGGATTCTTTGGTGGTTTGTCGGCTCAAGAGGAACACAAAATTTCGTGCAAATGATGATTCTAACAGAACTTCACGCGAGAGTGGTTGTGGAGTCTCAGAAGGGGTTACAGTTCAAATGAGCACTTGTGTGCCTATTCAAGATAAAGAGGTTGGGTGTAGTTCCAAGAGGAGTAACAATAGTAATAGTTCTCCTTCTATTACTGTCCAAATTAAATCCAGTGATAGAGTTGCCAATGAAGACAATCCCAAAGCTTCTTCCAATGAATCCAGTGATAGAGTTGCCAATGAAGCCAATCCCAAAGATAGAGTTGCCAATGAAGCCAATCCCAAAGCTTCTTCCAATGAATCCAGTGATAGAGTTGCCAACGAAGCCAATCCCAAAGCTTCTTCCAATCACTCTAAGGTGGATGAAGTGGATTATTATGCAGAGATCAACTTAGATGATATCATCAACTTAGATGAACCAGCACTCTAA

## **>** AhNAC23-**Arahy.332M63.1**

ATGCAAAAGGAAAAGGAAGCAATCACCACCAATAAGGAGGGGATTAATAAGAAGGACATAGAAATAATGGAAGGTTGCAATGGAAAGGAGGAAACCCTACCACCTGGGTTTCGATTTCATCCAACCGACGAAGAACTCATTACTTGCTATCTCATAAACAAGATCTCGGATTCAAACTTTTCAGGCAGGGCAATAACTGATGTTGATCTCAATAAATGTGAGCCATGGGAGCTTCCAGGGAAGGCGAAGATGGGAGAAAAAGAATGGTACTTCTTCAGCCTGAGGGACCGGAAGTACCCAACTGGGGTGCGAACGAACCGAGCCACGAACACCGGGTATTGGAAGACCACCGGAAAAGACAAAGAGATCCTTAATAGTGTTACATCGGAGCTAATTGGGATGAAGAAAACTTTGGTTTTCTACAAAGGAAGAGCCCCCAGGGGAGAGAAGAGTAATTGGGTCATGCATGAATATCGCATTCATTCTAAATCCACCTTTCGAACAACCAAGCAGGATGAATGGGTGGTTTGCCGTGTGTTCCAGAAGAGTGCCGGTGCAAAGAAGTACCCTTCTTCCAACCATGCAAGTAGGGCAATGAACCCTTTCAACCTTGAAATAGGTCACCACAATATTGTGCCGCCGCCGCCAATGATGCAACTCGGAGACCCCGCCGCCGCTCATTTCCTCTATGGAAGGAACTATATGAATACTGCAGAGTTAGCAGAAGTAGCTAGGGTTTTGCGTGTTGGTACTGGATCAACCAGTACCAACCTGCCCGGGATGCAGCCTCAGATAAATTATCCAGTGGCTGCGTCTCCAGGAATTGGATTCACAATTTCAGGACTCAATTTAAATCTAGGAGGCGGAGGAGGCGAAACAGTAGTGGCCACAACACAACCAGTTTTGCGGCCCATGCAGCCGACTCCTCCGTCCCAAACATTGGGTATGGTTCCTCATCATCAAGTTCATCATGATGTGAGTTCCAACATGATTTCAACAAATTCCCTTGGTGCTGAGAATGTGGGTTACGTCAATGAAATAAGCAACACAAATGGTGGTCATGGAAATAGGTTTATGGGCATGGATCATTGCATGGATCTTGATAATTACTGGCCTTCCTACTA

## **>** AhNAC24-**Arahy.338FND.1**

ATGGGGGTTAATGAAGATTTGATGATGAAGGATGATTCATATGCATCATCAGTGATGGAGGAAGAGGATGATGTTCCACTTCCAGGGTTTAGATTCCACCCAACAGATGAAGAACTTGTGAGTTTCTATCTAAAGAGGAAGCTTGACAAGAAACCAATCAGCATCGAACTCATCAAACAGATTGATATCTACAAGTATGATCCTTGGGATCTTCCAATTGTGATTCAATCTGTGATTGCAATTCGCAGGCTCAATTATTGTTATGATCATTATGATTATTACATTAAACTCCATGAAATCTGGACATTGTGTAGAATATTCAAGCGAAATGTGTCACAAAGGAAGCACACAGCAGACTTGAGATCACATTTAGTAACAGCTAATAGTAACAAGCACAAAACCACTAGAACCCATGTTGTTCAATCCAATAATAACAATATTAATCAACATCAAGAATCTTACATCAACTTTGGTGCAACAATCATTGGCCATCACCATTACCATCATCAAAATGAACAGAAGCCAGTGACTAACTACACAGCATGCAATAACAACACTGATCAAATCCAAAGGAACAATAGCAATCATCATCATCATCATCAGTTGAACTATCACCCTTCTTCAGCGGTGGCTACTACTGTGCCACAGCAACAACAACAACAATATCATCATCATCATCAGCTAATGACGGCTCCAGCTTCTAACATGTGGATTAATCCTTCTGCGATGAACGATTTGTTTGCATTTGATGATAACTGGGATGAGCTTGGATCCGTTCTCAAATTCGCTGTTGATACCCCTAGCTTGTAA

## **>** AhNAC25-**Arahy.3F2LJ6.1**

ATGGAAGAGCCTGTGGTAGTTAACAAAGGCGAGGAGCCGCTGGATTTGCCACCAGGTTTCAGATTCCACCCAACAGACGAAGAAATCATCACTTATTACCTCACCGAGAAGGTCATGAACAGCAGCTTCAGTGCAACTGCCATAGGTGAAGCCGATTTGAACAAATCCGAACCCTGGGATTTACCAAAGAAAGCAAAGATGGGAGAGAAGGAGTGGTACTTCTTTTGTCAGAAGGACAGGAAATACCCGACGGGGATGAGGACCAATAGAGCAACGGATTCCGGTTACTGGAAGGCAACCGGAAAAGACAAAGAGATTTTCAAAGGGAAAGGGAATCTCGTTGGGATGAAGAAAACGCTTGTTTTCTACCGAGGTAGAGCTCCCAAGGGTGAAAAGACCAATTGGGTCATGCATGAGTTCAGATTGGAAGGCAAATTTGCCAATTATAACCTCCCCAAGGCTGCAAAGGATGAATGGGTTGTGTCGAGGGTTTTTCACAAGAACACAGATGTAAAGAAGACTACTACCCCATCCTCATCATCATCAATAATTCCTGGCCTTTTGAGGATCAACTCAATAGGCGATGATCTTCTAGATTGTTCCACACTCCCACCTCTCATGGACCCTCACCCTACTCCTCTCGATACAACAAAATCCGACGGCTATTATTTCCCCTCCTTCTCATCATCACATCAGATTCTCAATATCAAGCCCGAAGAACACAACACAAGCCACCAAATTCCCATCACCAACTACCAGATTCCAAATTTCAACACCACACTCTCTTCTTCATCATCTCATCAAATCAGACTACAAAATCATCTCAACTTGTTCTCATCATCATCATCAAATAACAATTACCATAATAGCTCGTGGCCAACCTATTATGATGAGGTCCACCACCATCATCAAGATGATATTCTACTAAGAGCAATCGCGTCAAAGAACTATAGCAACGGAGGAGGAGGCGCCGGCGAGTGCAAGGTGGAGCAATTCTCCTCCGGCAACCAGTCAGTGGTGAGCGTTTCGCAAGAGACGGGGCTGAGCAACGACAGAACCACCAATGACACGTCATCGGTGGTTTCGAAGCAGCAGCATAATAATAGAACATTGTACGAGGATCTTGAAGGTCCTTCTTCATCAGTTGCACCTCTTTCAGATTTGGAATGCTTGTGGGATACCTACTGA

## **>** AhNAC26-**Arahy.3G9MJD.1**

ATGGATGCGGCTTTGGATTTGCCTCCCGGTTTCAGGTTCCACCCTACAGATGAGGAGATCATCTCTTATTATCTCACTCACAAGGTTTTGAACACAAGTTTCACCGCAACTGCCATTGGAGAAGTTGATCTCAATAAGTGTGAGCCTTGGGACTTGCCTAAGAAAGCAAAGATGGGGGAGAAAGATTGGTACTTCTTCTGGCAAAGAGATAGAAAGTATCCAACTGGGATCAGAACGAATCGAGCCACGGAATCCGGCTATTGGAAGGCCACCGGAAAAGACAAAGAGATTTACAAAGGGAGAAACCTTGTTGATTTTTCTTCTCTCCCACCTCTCATGGATCCTTCTAATAATTATGATGATGAACACACCACCACCACCAACAACAACTATGTCAAT

## **>** AhNAC27-**Arahy.3GEX4P.1**

ATGACGAACCTTCCTCCTGGGTTTTGCTTCTCTCCAACAGATGAAGAACTCATCCTTCACTTTCTTTATTCCAGAATTTCTCTACCATTCCATCCCAGCATCATACCGGACCTTGATCCCTCTCAACTTCATCCATGTCAATTAAATGGTAAGGCGTTTTCAAGTGGGAATCAACACTATTTCTTCACCAATAAAGTGAAGGAAAACAGAAGCACAGAAAATGGGTATTGGAAGGAAATAGGTTTGAGCGAACCTATAATCTCAGCTGATGCAAACAAGAAATTGGGAATCAAGAAGTATTTTGTCTTCACTCTTAATGAAGGCACAGAAACCAATTGGGTCATGCAGGAATACCATATTTCCTCATCTATGTTCCACAACCCTATTTCATGTTATGCAAATGGAACTGCTCACCGAAGATTATTAAGACCTGATCAAAATCAGAACAATAAATGGGTTTTGTGCAGAGTGTATGAGAAGAACAAGTCACAATCACAACAAGGTGCAACTGCAAACTCCTACTATAGCGACGAGGATGATTGTGGATCAGAACTTTCATATCTAGATGAGGTTTATCTGTCACTTGATGATGATCTTGAAGTCATAAGCCCCCCTAATTAA

## **>** AhNAC28-**Arahy.3M9RMX.1**

ATGGTGGATAGGGATTCAAGCGGAGCACACATGTCAATAGCAGCTTCTTCCATGTTTCCTGGCTTTAGGTTCTGTCCCACTGACCATGAGTTGATCTCTTATTACCTCAGAAAAAAATTGGATGGTGACGAGGACAGTGTTCAGATTATTTCCGAGCTTGAACTTTGCACCTTTGAGCCTTGGGATTTGCCTGAAAAATCGTTCATTAAATCAAACGATGAGTGGTTTTTTTTCTCGCGACGAGGGAGAAAGTATCCTAATGGTTCACAAAATAAAAGGGCAACTAAACATGGGTATTGGAGGGTCACATGCAATGAACGACAGATAAAGTCTGGTCAAAATGTGATTGGTACCAAACGCACCTTGGTATTCCATGTCGGCCGAGCTCCTAAAGGCCAGAGAACTGAATGGATTATTCATGAGTACTGCATCAATGACAAATTTCAGGATTCTTTGGTGGTTTGTCGGCTCAAGAGAAACACAAAATTCCATGCGAGTGATAGTTCTAACAAAGCTTTACGCAAGAGTGGTGGTGGAGTCTCAGAAGGGGTTACAGTTCAAAGGAGCACTTGTGTGCCTATTCAAGATCGTTCTAACAAAACTTCATGCAAGAGTAGTGCTTCACGCAAGAGTAGTTGTGGAGTCTCGGAAGGGATTACAATTCAAAGGAGCACTTGTGCGCCTATTCAAGATCGTTCTAACAAAGCTTCACGCAAGAGTAGTTCTTCACGCAAGAGTAGTTGTGGAGTTTCGAAAGGGGTTACGGTTCAAAGGAGCACTTGTGTGCTTATTCAAGATCGTATTAACAAAGCTTCACGCAAGAATTGTTGTGGAGTCTCAGAAGAAGGAGTTACAGTTCAAAGGAGCACTTGTGTGCCTATTCAAGATAAAGAGGTTGGATGTAGTTCCAAGAAGGGTAACAATAATAATAGTTCTCCTTCTACTACTGCCCAAATTGAATCCAGTCGTATAGTTGCCAATGAAGCCAATCCCAAAGCTTCTTCCGGTCATTCTAAGGTGGTGGACGAAGTGGGTTATTATGCAGAGATCAACTTAGTTGATATCATCAACTTAGATGAAACAGCACTCTGA

## **>** AhNAC29-**Arahy.3N6K0N.1**

ATGGAGGGTAGTAGAAGAAGCTCAAATTCTGAACTCCCGCCTGGGTTTCGGTTTCACCCAACTGATGAAGAACTAATCGTTCACTACCTTTGTAACCAAGCCACTTCAAAGCCTTGCCCTGCTTCCGTCATCCCTGAAGTTGATATCTATAAGTTTGATCCATGGGAATTACCCGATGACTGGGTGTTATGCAGGATCTACAAGAAGAGGAGCATAGCAAAATCAATGTTGGAGCCTAAAGAGGAATTCCCAACAATGCCCCAAATCAATCATCATCTAACATCATCATCAAATGATGGGAATGATAATAATGATGATGAGCAAGAAATGATGATGAAATTCCCAAGGACATGTTCCCTTACACATCTCTTGGAAATGGACTACTTGGGCCCAATATCACAAATACTCTCTGATGGATCATATAACTCAACCTTTGATTTTCAACTAAACAGTGCCAATGTTGGCAACATGATTATGGACCCTTTTGTGAAACAACCTCAGATCCTTGAAATCCCTAACAAAAATAATCCTAACAATCCTTATTATGATGTGGATTCAGGGAAGAACAACCTAGTGAAACAGAATAGCACCATAAACCCTACTATATTTGTGAACCAATTCTTTGATCATAGTGGTAGTTAA

## **>** AhNAC30-**Arahy.3X1GMP.1**

ATGGTGGATAGGGATTCAAGCGGAGCACGCATGTCAATAGCAGCTTCTTCCATGTTTCCTGGCTTTAGGTTCTGTCCCACTGACCATGAGTTGATCTCTTATTACCTCAGAAAAAAATTGGATGGTGACGAAGACAGTGTTCAGATTATTTCCGAGCTTGAACTTTGCACCTTTGAGCCTTGGGATTTGCCTGAAAAATCGTTCATTAAATCAAACGATGAGTGGTTTTTTTTCTCGCGACGAGGGAGGAAGTATCCCAATGGTTCACAAAATAAAAGGGCAACTAAACATGGGTATTGGAAGGTCACAGGCAATGAACGACAGATAGAGTCTGGTCAAAATGTGATTGGTACCAAACGCACCTTGGTATTCTATGTCGGCCGAGCTCCTAAAGGCCAGAGAACTGAATGGATTATTCATGAGTATTGCATCAATGACAAATTTCAGGATTCTTTGGTGGTTTGTCGGCTCAAGAGAAACACAAAATTCCATGCGAGTGATAGTTCTAACAAAGCTTTACGCAAGAGTGGTGGTGGAGTCTCAGAAGGGGTTACAGTTCAAAGGAGCACTTGTGTGCCTATTCAAGATCGTTCTAACAAAACTTCATGCAAGAGTAGTGCTTCACGCAAGAGTAGTTGTGGAGTCTCGGAAGGGATTACAATTCAAAGGAGCACTTGTGCGCCTATTCAAGATCGTTCTAACAAAGCTTCACGCAAGAGTAGTTCTTCACGCAAGAGTAGTTGTGGAGTTTCGAAAGGGGTTACGGTTCAAAGGAGCACTTGTGTGCTTATTCAAGATCGTATTAACAAAGCTTCACGCAAGAATTGTTGTGGAGTCTCAGAAGAAGGAGTTACAGTTCAAAGGAGCACTTGTGTGCCTATTCAAGATAAAGAGGTTGGATGTAGTTTCAAGAAGGGTAACAATAATAATAGTTCTCCTTCTACTACTGCCCAAATTGAATCCAGTCGTATAGTTGCCAATGAAGCCAATCCCAAAGCTTCTTCCGGTCATTCTAAGGTGGTGGACGAAGTGGGTTATTATGCAGAGATCAACTTAGTTGATATCATCAACTTAGATGAAACAGCACTCTGA

## **>** AhNAC31-**Arahy.4435CX.1**

ATGGGTTCTTCTAATAACGGTGGTGTGCCACCGGGGTTTCGATTTCATCCAACTGATGAGGAATTGCTTCATTACTACTTGAAGAAGAAGGTGTCGTTTCAGAAGTTTGACATGGATGTTATTAGAGAGGTCGACCTCAACAAGATGGAGCCTTGGGACTTGCAAGAAAGATGCAGAATAGGGTCAACACCACAAAACGAGTGGTATTTCTTCAGCCACAAGGATAGAAAGTACCCAACAGGGTCAAGGACAAACCGAGCAACGAACGCAGGGTTCTGGAAAGCCACGGGAAGAGACAAGTGCATAAGGAACACCTACAAGAAGATTGGGATGAGAAAGACACTAGTGTTCTACAAAGGTAGAGCCCCTCATGGCCAGAAGACTGATTGGATCATGCACGAGTACCGCCTTGAAGATTCCAATGATCCTCAAGCAAATGCCAACGAAGATGGGTGGGTGGTGTGCAGGGTGTTCAAGAAGAAGAACCTATTCAAGATTGGAAATGAAGGAGGTGGTGGCTCCACACACACCTCATCGGACCAGCAACTCAACAACTCAACGGCCACCAATGCTCGTTCCTTCATGCAAAGGGAAAACCACTACCTACTGCATCACCACCAACAACAGCAAAACCCTAGGAATGGGAACCCATCGTCTTCATCCTCAGGCTTTGATGAGCTCGATAAACCCGAACTCGGTCTCCATCACTATCCTCACATGCAAAACCCACACTATTCACTCTTCCATCACTCCCAACCACTTCTTCATCCCCAGGCCCACAAGCCCATCGTCTATGACTACTCTTATACACCCGCGCTTCCCTCAGACCCTCCTGTCATCGCTAAGCAGCTCATGACTAACCCTAGAGACTGCGATAGCGGTGGCAGCGAGGGTCTGAGGTACCAGCAGGTTTCCGAGCCTGGTATGGAGGTTGGATCATGTGAACAAGCCCAGGAAATGGGCGCCGCCGCGGCCACAAGAGGAGGAGGAGAAGGAATGAATGAATGGGGTGTGCTTGATAGGCTTGTAACCGGGAACCTTGGAAATGAAGATTCAGCCAACAAAGGGATTAGGTTTGAAGATGCAAATCCACACCAGATTAACCAGCTTTCTTTGAGGGGAGAGATGGATTTCTGGGGCTATGGAAAACAATAA

## **>** AhNAC32-**Arahy.4PPW5Y.1**

ATGGCATCAAAGAAAGAGAACACCAGTAACACCAGAAACAGAATGAATCTTTTGGAGTGCTCTTTCTGCTCAAGTCAGATCAAAGAATATGCCTCATGCACACAGATTAAGAATGGAGTGAGATTATTGCCACCTGTATTTCGGTTCCAACCAACAGAAGAGGAGTTTCTATTTCAGTATTTGAAATGTAAGTTGCCAGCTTCAATCATTTCTGAGATCAATGTTTGCAACTACGATCCATGA

## **>** AhNAC33-**Arahy.4QZT53.1**

ATGGGTTCTTCTAATAACGGTGGTGTGCCACCGGGGTTTCGATTTCATCCAACCGATGAGGAATTGCTTCATTACTACTTGAAGAAGAAGGTGTCGTTTCAGAAGTTTGACATGGATGTTATTAGAGAGGTCGACCTCAACAAGATGGAGCCTTGGGACTTGCAAGAAAGATGCAGAATAGGGTCAACACCACAAAACGAGTGGTATTTCTTCAGCCACAAGGATAGAAAGTACCCAACAGGGTCAAGGACAAACCGAGCAACGAACGCAGGGTTCTGGAAAGCCACGGGAAGAGACAAGTGCATAAGGAACACCTACAAGAAGATTGGGATGAGAAAGACACTTGTGTTCTACAAAGGTAGAGCCCCTCATGGCCAGAAGACTGATTGGATAATGCACGAGTACCGCCTTGAAGATTCCAATGATCCACAAGCAAATGCCAACGAAGATGGGTGGGTGGTGTGCAGGGTGTTCAAGAAGAAGAACCTATTCAAGATTGGAAATGAAGGAGGTGGTGGCTCCACACACACCTCATCGGACCAGCAACTCAACAACTCAACGGCCACCAATGCTCGTTCCTTCATGCAAAGGGAAAACCACTACCTACTGCAAAACCCTAGGAATGGGAACCCATCCTCTTCATCCTCAGGCTTTGATGAACTCGATAAACCCGAGCTCGGTCTCCATCACTATCCTCACATGCAAACCCCACACTATTCACTCTTCCATCACTCCCAACCACTTCTTCATCCCCAGGCCCACAAGCCCATCGTCTATGACTACTCTTATGCACCCGCCCTTCCTTCAGACCCTCCTGTCATCGCTAAGCAGCTCATGACTAACCCTAGAGACTGCGATAGCGGTGGCAGCGAGGGTCTGAGGTACCAGCAGGTTTCCGAGCCTGGTATGGAGGTTGGATCATGTGAACAAGCCCAGGAAATGGGCGCCGCAAGAGGAGGAGGAGAAGGAATGAATGAATGGGGTGTGCTTGATAGGCTTGTAACCGGGAACCTTGGAAATGAAGATTCAGCCAACAAAGGGATTAGGTTTGAAGATGCAAATCCACATCAGATTAACCAGCTTTCTTTGAGGGGAGAGATGGATTTCTGGGGCTATGGGAAACAATAA

## **>** AhNAC34-**Arahy.563XC3.1**

ATGGAGAATATGAATAGTTTTTGTCATGTTCCCCCGGGTTTTAGATTCCACCCCACGGATGAAGAACTTGTTGATTACTACCTTAGGAAGAAGGTTAGTTCAAGGAAGATTGAGCTTGATGTAATCAAAGATGTTGACCTCTACAAAATTGAGCCATGGGACCTTCAAGAAATATTTGCATACATACATAAT

## **>**AhNAC35-**Arahy.5AD6K9.1**

ATGGAACAAGAAGAAGAACCACAACAAAATGAGCCACCTCACTCTCACTCTCAATCTCGGTGCGTGACGCTACCTCCCGGTTGCCGGTTCCATCCTTCGGAGGAGCTTCTATTGCGTTACTACCTGACCAACAAAAACGGCACGGGGAACTGGAATGGTAACGGTGGTTTGGGATTCGATGGTTCTGATTTGATTCGGGAGCTGGATTTTTACGATTACGATCCTTTTGAACTGCCGGATTTTGCGTGCTTTGCGTACGGCTACGGCGGAAGGAGGAGGCACTGGTACTGTTTCACCTCCGTTAGGGTTTCGAGGGGAGAGAGGTGGAAGAGGAAGAGGAAGGTTAAGAGTGGGTTCTGGTTGAGGAGGGGAAGGGTTTCGAATGTTAACGGTGTTGGGGAGAACGTGGTTTTGGGAACGAGGACGAGGTTCGTTTTCTATATGGGTGATTCGGCGAAGAACGGTGCCAGGACGGATTGGGTTTTGTATGAATACGCATTGGTTGATCATGTTATGGCCTCTTATGTTCTTTGCCGGGTATTTAGTAAGCCTCGTTATAAGAATAGTGCATCAGACATCGGCCTGAGTTGTTGTGCAGAAGAGAGTGTATCAGCAGTGCGCCATATTGGTATTCAGCATGATGAACATGTTAAATTGGATGCCGTTGAAGCTAAAGTATGTGATGATATCTCCATTGACCACAACAATGAAATATGTGCTGGTGGAAACAGCGATAATGATAATCAAGTTAAGAATGCACATGATATAGATGCTCTACGTTGTTTGGCGGGTCCTCAGGGCAGTCAGCAGCTATCAATTGCAAACTTCTACATCAGCAATAATCTGAAAAGTCTAGTCAGAAATGGGGCTATCAGTGTCGGGAGTCAATTAGCGTTTCTATCTTCTTTACAATTACCTTCCCCCTTTTTAGATTTGCTAGATTTGAGAACTCATTCATATGATCTTGGTTGCATTCCCCTTTTTTTCCCTGCTAATATCATTATTAATATTAGATTAAATGTGTTATTCTTTCTTCTGAATGGGAAAACATATTACTGTTCAGTAGAAACCGCTATAAGTAAATCTAGCAGTAATATGTCCTTCCATCCAGCTCTTGCTTTCCAGCAGACTTTTGCTTTAGCATGCTCTTTTGCCAGTGAATCTGTTCATTTCATTTTGTAA

## **>**AhNAC36-**Arahy.5P3U81.1**

CCCCATAAAGTAGTGTAAGTACACATCACCATTTTAATATAGTGTATGGAGGATCCACCAACTGGTTTTCGGTTCTATCCTACAGAAGAAGAGCTAGTTGCTTTCTACCTAAACACCCAGCTTCAACTACAAGGCCACGCCAATAACATCAACAGGGTCATTCCAGTGGTTGACATCAATGGCGTTGAGCCCTGGACTCTTCCATCACTGGCGGGAGAGCTGTGCAGGGAAGAGAAGGAACAATGGTTCTTCTTTGTGCCTCGCCAAGAGAGGGAAGCCAGAGGGGGGAGGATCAACAGAACCACTGCTTCTGGTTACTGGAAAGCCACCGGATCACCGGGGTACGTATACTCTTCCGATAACCAAGTGATCGGAGTCAAGAAAACCATGGTTTTCTACAAAGGAAAAGCTCCCACCGGTCGCAAAACTAAATGGAAGATGCATGAATACCGCGCCATCGTTCAAGCCCCTAACCAATCTCCCACGGCTATTCCTCAGTTGAGGCACGAATTCAGCTTGTGTCGCGTGTACGTGATATCCGGAAGTTTCAGAGCATTTGATCGACGGCCACGGGAGGTGGTGGTGCCAAGAGTTCTTCATCATGGTTCTTCTACAACAAGTGCTCAGCAGCATCAAGGAGAATCATCAGCAAGGGTGCAGGCTAATAATAATAATAACGGGTCGAGCTCGTCGGAAACTTCCCTTTCATCAGGTGGTCCTGATTTGCCACCAGATACTGGAGGAGGAGGGTCAAGTAGCAATTGGAATAGTAGTGAGGTTCAAGTTCAAGCTCAAGTTCAAGAACCGCTATGGGAATGGGAACAACTCGATTGGCTATAA

## **>**AhNAC37-**Arahy.65HUV4.1**

ATGGAGAGCACCGACTCGTCCACCGGTTCGCAACAACCGAACCTTCCACCGGGTTTCCGGTTCCACCCCACCGACGAGGAGCTCGTTGTTCACTACCTGAAGAAGAAAGCTGCATCAGCTCCTCTCCCAGTCGCCATCATCGCCGAGGTTGATCTCTACAAGTTCGATCCATGGGAGCTACCAGCTAAGGCAACGTTTGGGGAGCAAGAGTGGTACTTCTTTAGCCCAAGGGACAGGAAGTATCCGAACGGTGCTCGGCCAAACAGGGCGGCAACTTCCGGGTACTGGAAGGCAACCGGGACGGATAAGCCGGTGCTGACCTCCGGTGGGACCCAGAAGGTCGGCGTGAAGAAGGCTTTGGTCTTCTATGGAGGGAAGCCCCCCAGAGGGATAAAGACAAATTGGATCATGCATGAGTATAGACTTGCTGATAACAAACCTAACAATAGGCCTCCTGGTTGTGACTTGGGTAACAAGAAAAACTCTCTAAGGCTTGATGATTGGGTATTGTGCCGAATCTACAAGAAGAACAACACACATAGGTCTCCAATGGAACATGAGAGGGAAGATTCCATGGATGACATGATTGGAGGGATTCCTCCTTCCATCAACGTGGGCCAAATGAATGCAAGATTTCATCTCTCAAAAATGTCAACAAGCTTTAGCAACGCTTTGTTGGAAAACGACCATCACCATCACCAGAATCTTCTGGAAGGTATGATGCTAGGAGGAGGAACCAACAACAGCAATCCAAACATGTTGGGATTGGGATCAGCCTCAAACACCATTAACAATAATAGTAATAAGGCAGAGCTTTCATTTGTACCAACCATGACTACATCTTCAAACACCAAGAGGACTCTATCATCACTCTATTGGAATGAAGATGATGTTGCTGCTTCCAACAAAAGATTCAATTTGGAAAGTGGAGATCATAACCATGGAGAGAATAATGGTACTAGTGCTAGTTCTATTGCTACTCTGCTGAACCAACTTCCTCAAACCCCTTCATTGCACCAACACAACATCATGGGAACAATACCTTATCAAATTCAAGGAATGAATTGGTATGCTTAG

## >AhNAC38-Arahy.6LHU5T.1

ATGAAAAACAAGCGATGTAACAGAAAAACCAACATGGGATATTGGAAGATCACAGGAAAAGAGAGAATCATCAAAAGAACAGGGACAGACAGTGTCATAGGTACAAAAAGAACACTAGTTTTCTACAAGCGTCCACATAATGTCAAAACCAATTGGGTTCTTCATGAATATCATGCACTTCATCAAAAGAGCAACATCGTGTTGAGCCGTGTAACAATGAATGCTGAGAAAAGGGAAAAGAAGCTCAAGACAAAAGCAAGCAACATAATCGAAGAGGAAGTAAAATGTGAAGATGAACCATGCAGCGAAATTACTGGCTGTGTTACCCAAGCAACTACAGAAGATGCAATCCTTCCTGATAATGCATGTGTTTCATCCGAGTGGCAACAACCTCAAGCTATGGACTATGAAATTCTCTCATCGGGACAACGATCTTCAGTGGCCTATTCCGGTAATGAAAACAATGCCGCGTTGCTTCCAATGGAAGCAACGTGGAGGCAAGATGCCGGTATGAATACCGAGTGTTTTTGGAATTCGCTGTTTTCTAGCATCGATGCTGACCCTCATGCCGAGTTCTTAAATTCAGTGTTGGCAGGGGATGATCAACTCTATGTTGATTCCGGCCACCATTGA

## >AhNAC39-Arahy.6RTE4A.1

ATGAATACACTTTTGCAGAACACTAAGATCTCTAACAATCAAATCAATACAAGAGAGAAAAATAATATAATGTCACCAGTTGGATTACCACCTGGGTTTAGGTTTCATCCAACAGATGAAGAGCTTGTTAACTATTATCTAAAGAGGAAGATCAATGGCCAAGAAATTGAACTTGATATCATTCCTGAGGTTGATCTCTACAAATGTGAACCATGGGAATTAGCAGAAAAATCATTTTTGCCGAGTAGAGATCCAGAGTGGTATTTCTTTGGACCAAGGGACAGAAAATACCCTAACGGATTCAGAACAAATAGAGCAACACGAGCAGGGTACTGGAAATCAACAGGTAAAGACAGGAGAGTTTCAAGCCAAAGCAGACCAATTGGTATGAAGAAGACTTTGGTTTATTATAGAGGAAGGGCTCCTCAGGGAATCAGAACTGATTGGGTTATGCACGAATATCGTCTCGATGACAAGGACTCTGAAGACACCACCGGTTTACAGGATACTTATGCTTTGTGCCGTGTGTTCAAGAAAAATGGAATATGTACGGATGTTGAAGAGCAAGTAGGGCATTGTAGTAACATGTCTTCACTAATTGAGAGCTCACAAACCATAATCAATAATAATAACAATAATAGTAATAATAATAATGAGTATTGTGAAACCATGTCACCAGACATAGCAGGGGTTTCATCTTCATGTTTGGAAGAGGAAGACAAAGATGATTCATGGATGCAGTTCATCACAGAAGATGCATGGTACTCTTCTAATGCACCAAATATGGTTGGTGGTGAAGAAGTTTCACATGTTACATTTACAAGCTAA

## >AhNAC40-Arahy.70K1B3.1

AGCGGAGCGGGTGATCAGCATCATCACCCTCAGCTTCCTCCAGGCTTTCGATTCCACCCCACAGACGAAGAACTCGTCGTTCACTACCTTAAAAAGAAAGCTTCTTCTTCACCACTCCCTATCGCCATCATCGCCGACGTTGATCTCTATAAGTTCGATCCATGGGAGCTCCCAAGTAAGGCAGCGTTTGGGGATCAAGAGCGGTACTTTTTCAGTCCTCGGGATAGGAAGTACCCGAATGGAGCTCGGCCAAACAGGCGGCTACTTCTGGGTATTGGAAAGCCACCGGGACATATA

## >AhNAC41-Arahy.72Q128.1

ATGGAGGGTAGTAGAAGAAGCTCAAATTCTGAACTCCCTCCTGGGTTTCGGTTTCACCCAACTGATGAAGAACTAATCGTTCACTACCTTTGTAACCAAGCCACTTCAAAGCCTTGCCCTGCTTCCGTCATCCCTGAAGTTGATATCTATAAGTTTGATCCATGGGAATTACCCGTCAAATTTCTAGATGACTGGGTGTTATGCAGGATCTACAAGAAGAGGAGCATAGCAAAATCAATGTTGGAGCCTAAAGAGGAATTCCCAACAATGCCCCAAATCAATCATCATCTAACATCATCATCAAATGATGGGAATGATAATAATGATGATGAGCAAGAAATGATGATGAAATTCCCAAGGACATGTTCCCTTACACATCTTTTGGAAATGGACTACTTGGGCCCAATATCACAAATACTCTCTGATGGATCATATAACTCAACCTTTGATTTTCAACTAAACAGTGCCAATGTTGGCAACATGATTATGGACCCTTTTATGAAACAACCTCAGATCCTTGAAATCCCTAACAAAAATAATCATAACAATCCTTATTATGATGTGGATTCAGGGAAGAACAACCTAGTGAAACAGAATAGCACCATAAACCCTACTATATTTGTGAACCAATTCTTTGATCATAGTGGTAGTTAA

## >AhNAC42-Arahy.76LABN.1

ATGGGAGGGGCATCACTGCCTCCGGGTTTTCGTTTCCACCCTACTGATGAAGAACTGATAGGATACTACCTGAAAAGAAAAGTTGAGGAGCTTGAAATTGAACTTGAAGTTATCCCTGTGATTGATTTGTACAAGTTTGATCCTTGGGAGTTGCCGGAGAAGTCATTCTTACCAAAAAGAGACTTGGAATGGTTCTTCTTTTGTCCAAGGGATCGAAAGTATCCGAATGGATCAAGAACAAACAGAGCTACCAAAGCAGGATACTGGAAAGCCACTGGAAAAGACAAAAAGGTTGTGTGCCAATCTAGTCCATCAACATCAATCATGAAAGCCACCGGATATCGCAAGACCCTTGTTTTCTATCGCGGAAGAGCCCCTTTAGGCGACCGAACGGATTGGGTTATGCACGAGTATCGCCTCTGTGATGATCTTGGCCAAGACTCACCAAGTTTTCAGGGTGCTTATGCTTTGTGCCGGGTTATTAAGAAGAATGACAAGGCCAGTGATTACAAGGGTAAAAGAGGTGTCAGCAGTTCCAAGAATGAGAATGAGAATGAGAATGAGAGCTCAATGAGATTGTCATCCTCTAAGGAGCACTTGAGCATCTCTGCTGATGTTTCTTCTCAAGCAAGTCAGCTATGCAGCGAGAGTCGTTATTCGAGCCCTATAGCTTCTCCTTGTGCATACAATGTGGCTGCAACGGCCGGGTTTGAGCCACCTTCTGTGGACACTAATCCTTCAACCTTCTTGGTCTCCCCTGATATGATTCTTGATTCTTCAAAGGACTTTGCTCAAACACAAGATGTTATTTCAGGATTCTTTCCGCATCATGAATTGCCAAGTACAATGACACCATGGCAATCATTGGAACATACAGAGATTTCATCCAGTTCATCCTACTCAAATTTCAATGGGGAGATAGAATTCTCTGATGAACTCGGCCTAATTGGCCGAATGTCGCGTTACTCAGGACAAGTAGACATGTTAGACTTCTATGGAAATGAGGAAGTGCTGTATGAATATGAAGGATATGACCAGATCAATTCAATCAGAGATCCAAGACAATTCTGA

## >AhNAC43-Arahy.78BVZL.1

ATGACTTCAACAAATTTACCTTCAGGTGCAAGCAAGAAGTTCAAACCTACAGATGAAGAACTCATTCAAGATTTTCTCCGTAACAAAATCAATGGGAGGCCTCTACCAAACTATGGAACCATTCTTGAAGGTGAATTGTTTGGTACGGAGAAGAATCCATGGGAAATTTGGGAAGAAAACGTTGAAAATTCTTATGATGGGAAGGACCTCTATTTCTTTACTACTCTGAAGAGGAAGTTCTCGACTAACAGCTTGAGGATGGTTCGCACCATCGGGTTGGGTTCTTGGGAAGGTGAAGACATCGGAAAAGAGATTATGGCCAACAAAACTAACCAGCGCATTGGAATGAGGAAACGATATCGCTTTGAGAAGAGTGGTACTAGCCATGATGCGATCGGTGGCGGCAGAGGAATGGTCTCTCCTCTATTTAGAAAGAATAACATTAAACCTTGTCAAAAGAGAAGGAAACTTGTGGCTCCTGAAACTGTGGCTACTGTAATACCGAATGCCAATAATTATGCTGGGATAGTAGAATTTGTTAAACAATCAGAGAATGGAGATGGAAGGATGGAGATTCAAAATAAATCAGCAAACCAATCACGCAATAATGAAGATAAAAAGATTGATGAGATTCAGAGAAAACTAACAAATCAATTAGAGAATAATAATGAAGATGAAAAGATTGATGAGTTTCAGAGAGATCTAACAAATCAATTAGAGAATAATAAGGATGAAATAAAGGTTGATGCTTTGGTTACTACTATTGAAATTGAGGCAAACTGTGACGAAATGGAAGATGGTGATGATGATAAAGATCACAATGATATGACATGGCCAGAGTTTTTTGCAATGCAATTGATAGAAGTTAATAAAGACTGGTTCATCCCAAACGATGATGTCCCCCTAATTCCAAATAAATTTCACAATGACACGTTAAAGAGGTTTAAAAAAGTTTTGTGA

## >AhNAC44-Arahy.798CQ8.1

ATGGATGCGGAAGATCATAATCATGCTTTGGATTTGCCTCCCGGTTTCAGGTTCCACCCTACAGATGAGGAGATCATCTCTTATTATCTCACTCACAAGGTTTTGAACACAAGTTTCACCGCAACTGCCATTGGAGAAGTTGATCTCAATAAGTGTGAGCCTTGGGACTTGCCTCAGAAAGCAAAGATGGGGGAGAAAGATTGGTACTTCTTCTGGCAAAGAGATAAAAAGTACCCAACTGGGATCAGAACGAATCGAGCCACGGAATCCGGCTACTGGAAGGCCACCGGAAAAGACAAAGAGATTTACAAAGGGAGAAACCTTGTTGAAAAATTGAACCCAACAATTCCATCTGGCCTCTTTAGGATAATGAAGAACGTGAACTCAATTGAGGATGATGATCTTGTAGATTTTTCTTCTCTCCCACCTCTCATGGATCCTTCTAATAATTATGATGATGAACACACCACCACCACCAACAATATGTTTCCATCATCATCAGATTATAATATTACTATTCAGCAAAACAAGAAGGATATGATGGGAAAGTTCTGGCCAGCTAGCTTAGTGCCTCACACCAAGGCATGGCTGCAGTTAATAGATCAAAGTTTC

## >AhNAC45-Arahy.7G7SK7.1

ATGGAATCAATGGAGAATGTTAGAATGCAAAGAGAGAAAGATCAGAAGTTCGAATTGCCGTCCGGCTTTCGATTTCATCCCACCGATGTAGAGCTCATAAATTACTACCTTGTTAAGAAGGTTCTTGATGATAAGCACTTCTGTTCTATAGCAATTGCTGATGCTGATATGAACAAGTCTGAGCCATGGGATTTACCCGGTTTAGCGAAAATGGGCGAAACAGAATGGTATTTTTTTAGTATGAAGGATAGAAAATACCCAACTGGCCAAAGGACTAATAGGGCGACCGAGGCCGGATATTGGAAGGCCACAGGCAAAGACAAGGAGATATCAAAGGAGAATTCAAAGATTGGGATGAAGAAGACCCTTGTTTTCTACAAAGGAAGAGCTCCAAGAGGTGAAAAGACTAATTGGGTCATGCATGAATATAGATTGGAAGGGAACAAATCTGTTTATAATCTGTCACAACCTGAAAGAGGTGAATGGGTTATATGCAGAGTATTTGAGAAGGGCAATAATGGAAAAAGACTGAATATTGCAAAGTTGGAGAGGCTCAACTCTTCGGGAAAGGAACCATTACCATTGCCAAAACCTACTCCTTTGATGCCTCCATTGATGGATTCTTCATCATCGAGAACCACCCCCGGCGAGTTATCTCAGGCGACGTGCTACTCCTCGGATCCAAATCAAGCCGATGTCCAGAACAATTTGCATGATGACATAGTTGAAAGCAGGGAAACTCCTATCTTGAACTTTTCCCCTGCTTCCATCAATGAAGAATTAATTCAGATTCCAAACCAAATTGAGAATCCGGATTATTATACTCTGCCTCAAGAAAACAACGGATCAATTGCAAGGCAGAATCAGAAATCAGAGTTTGATGCTGATATATCATCTTTGATTTACAACAATGACATGTTTTACAGGTTCTTTGGGAACCAAGAACATTCATCTTCAGCTTCCGCAGACATTTGCAACCTATGGAATTACTAG

## >AhNAC46-Arahy.7GZ1C0.1

ATGCCAGAAAGCATGAGTATATCAGTGAATGGACAATCTCAAGTTCCACCTGGATTCAGGTTCCATCCAACTGAGGAAGAACTCCTTCAATACTACTTAAGGAAGAAGGTCTCTTATGAGAAGATTGATCTTGATGTTATTCGTGATGTTGATCTCAACAAGCTTGAACCATGGGACATACAAGAGAAATGTAAGATAGGAACCACCCCACAAAATGATTGGTACTTCTTCAGCCACAAAGACAAGAAGTACCCGACCGGAACCCGGACGAATAGAGCGACCGCGGCCGGGTTCTGGAAGGCCACCGGCCGTGACAAGGTGATATATAGCAACGGGAAGAGGATTGGAATGAGGAAGACGTTGGTATTCTACAAAGGAAGAGCCCCTCATGGCCAAAAATCTGATTGGATCATGCATGAGTATAGGCTTGATGATAACACCACCAACGATGCCAATATTGTTTCAAATGTGATGGGAGATGCAGCACAAGAAGAAGGGTGGGTGGTGTGTAGAATATTCAAGAAGAAGAACCATCTAAAAACCCTAGATAGTCCTTTAACCTCTTCCATCTCTGGCGACGGAGGTAGGAGGAGCCACCACCACCACCACCTATTCGACTCGTGCGACGAGGGCGCCTTAGAGCAAATTCTCCAACAAATGGGAAGAGGTGGTGGTGGTGGTTGCAAGGAAGAGATCAACAACTATGATCAATCTAACAACAACAACAACAATAATAACAACAACTATGGTGGATCATCATCGTTAACAACAAGGTATGCAAGACCTTTTGACACAATCAACAACAATGTTGATAGCAGGTTCTTGAAGCTCCCAAGCCTAGAGAGTCCAAAATCAACAAGCATGGATCATAACAATAATAACAACAATGATAATGATGATAGCAATGAAAATAATGGGTACCATCCTATTATTCCAGTTGAGATGGTAACTGAAAACGAAGGGTCATTCACATGTGACAATCCCAACAACATGTTTCATCATCACCATTTGGGTGGTGGCGGTGGCGGCGGCAGCGACGGCGGTGGCGGTCTTACAAATTGGGTAGCGCTAGATAGGCTTGTTGCTTCTCAGCTTAACGGTCAGACCGAAGCTTCTAGACAACTCTCTTGCTTCAATGACCCCACCATGGGGTATGGCACTGGAAATCATGATCTTCTATTTCCAACCGTCAGATCTACTTCATCGTTGACGTCATCGTCAGCGTCAATAAATCCAAGGGCTGTTATTAGTGCGGGTGCAGGTGCATACATTTCTCCAGGCGCACAGGATTATACCACCACAAGCGAGATTGACCTGTGGAACTTTGCTAGATCCACTTCTTCGTTATTGTCATCCTCTGAGCCATTGTGCCACGTGTCCAACACGTCAGTGTAG

## >AhNAC47-Arahy.7J37F0.1

ATGATGATCATAGTTACCTGGATGATTGAAATCTGGGTTATTGACATTGGAGGACTTGCAAAGAAAGTGAAGAATAATACACTGCCATTAGCTGATCAAATCAATGACTGTGGAGCATATTGTGAATGTCCAATATGTCATTATCACATTGATAACATTGATGTTTCTCCTGAGTGGCCAGGCTTTCCGGCCGGCGTGAAGTTTGATCCTTCCGACGTAGAACTGTTGGAACATTTGGCAGCAAAATGTTGTGTTGGAAACAAAGTGCCTCATGCCTTTATCCAAGATTTCATCCCAACACTAGAAGGAGACCAAGGAATATGCTACACACATCCAGAAAATCTTCCAGGTGCTAAGAAAGATGGGGCCAGTGTTCATTTCTTTCACAAAACAACTAATGCATATGCTACCGGACAACGAAAGCGTCGAAAGATTAATCATCAACTAGGCCTAAGTGAGGAGCATGTTCGCTGGCATAAGACCGGTAAGACCAAAGCTGTAACAGAAAATGGAGTACACAAGGGCTTCAAGAAGATCATGGTTCTTTACATAAGGCCTAAAAGAGGTGCAAAGCCGAATAAATCGAAATGGGTGATGCATCAGTACCATCTAGGGAATGATGAAGGTGAGAAGGAAGGTGAATATGTGGTTTCAAAGATTTTCTATCAGCAGAAGAAGAAAACTAAGAAGAATAAGCTGAATCCATTGGTGGCTGAAGATTCTGTCATGGCATTACAAGCAAGTCCAAGAACACCGAATCCAAATCCACCAAAACGGCCTCGGACAGGAAAATCTGTTGACTGTGATGACAATTTTGATGAAACTGACCTTATGCTATTCACTCAGGATGGAAAGCCTACTATTCATGGAGAATCTCTTGCCCCACCACCATCTGAAGTTCATGGTGATGAAAATAATGGAGGCTTTAACAACACTGCATTGTTATCTGTTGAAACACAACCTGTTGAAAACTCTGACTTTATTGGATTGGATGATATCTTACTATGCAAGGAACAGACATTAGATTCTTCTTCTGCTTACCTAAATGATTCTGGTTTGAAGTCCAACAATCTGAAAGGCTTTGCTTGCAATGCAAATGGAAATGCAGCTGAATTGTTTGGGGATGTGAATGATTGTTATGGAATTTCAGTGCTGGATAACCTGGGACTGGACAGTCCTCCTGATTTTGATCTTTCTAATTTGCAATTTTGTTCTCAAGACAATATCATTGACCTTCAATGGCTGGACATACCATAA

## >AhNAC48-Arahy.7QMU6B.1

ATGATGAGCAAGAAGATGAGGTTTGTTAAGAAGAACAAGAATGGAGTGAGATTATTGCCACCTGGATTTCGGTTCCAACCAACAGAAGAGGAGCTTCTATTTCAGTATTTGAAATGTAAGGTTTTCTCTTTTCAGTTGCCAGCTTCAATCATTCCTGAGATCAATGTATGCAACTATGATCCTTGGGATTTGCCAGGGAATAATAATAATTATGGGGAGCAAGAAGAGAGATACTTGTTCAGCTCAAAGGAAGTTAAGTATAGAAACGGTAACCGAATGAACAGAATAACGAAATCTGGATATTGGAAAGCAACTGGATCAGACAAAAGAATAATTTCAACATCATGTAATAATAATAATAATAGTAATATTGTTGGGATAAGAAAAACTCTTGTATTCTATCATGGAAAATCTCCAAATGGCTCTAGAACTCATTGGATCATGCGTGAGTATCGACTTGTCACTACTCCTTCTAATTCATCCCAGAAGTATGTAGAAGACTTAGGGAATTGGGTTCTTTGCCGCATATTCAAGAAGAAAAGAAGCATAGAAAGTCAACATCACATGGTCAACAACAAAATTAATAATGTTGTCGAGGTGGCTAATAATAATAATAAGCCAATATTCTTTGATTTTATGAGGCTATATGACTCGCCAATATCTTCTTCTTCATCCTCATCTTCTTCTTGTTTAAGTTCTGATTTCATAACTCAAGTGTAA

## >AhNAC49-Arahy.83A3G6.1

ATGGTGGATAGGGATTCAAGCGGAGTAAACATGTCAATAGCAGCTTCTTCCATGTTTCCTGGCTTTAGGTTCTGTCCCACTGACCATGAGTTGATCTCTTATTACCTCAGAAAAAAATTGGATGGTGACGAGGACAGTGTTCAGATTATTTCCGAGCTTGAACTTTGCACCTTTGAGCCTTGGGATTTGCCTGAAAAATCGTTCATTAAATCAAACGATGAGTGGTTTTTTTTCTCGCGACGAGGGAGAAAGTATCCCAATGGTTCACAAAATAAAAGGGCTACTAAACATGGGTATTGGAAGGTCACAGGCAATGAACGACAGATAGAGTCTGGTCAAAATGTGATTGGTACCAAACGCACCTTGGTATTCCATGTCGGTCGAGCTCCTAAAGGCCAGAGAACTGAATGGATTATTCATGAGTACTGCATCAATGACAAATTTCAGGATTCTTTGGTGGTTTGTCGGCTCAAGAGAAACACAAAATTCCATGCTAGTGATAGTTCTAACAAAGCTTCACGCAAGAGTGGTGGTGGAGTCTCAGAAGGGGTTACAGTTCAAAGGAGCACTTGTGTGCCTATTCAAGATCGTTCTAATAAAGCTTCACGCAAGAGTAGTTGTGGAGTCTCAGAAGAGGTTACAATTCAAAGGAGCACTTGTGTGCCTATTCAAGATCGTTCTAACAAAGCTTCACGCAAGAGTAGTTGTGGAGTTTCGAAAGGGGTTACGGTTCAAAGGAGCCCTTGTGTGCCTATTCAAGATCGTTTTAACAAAGCTTCACGTAAGAATTGTTGTGGAGTCTCAGAAGAAGGGGTTACAATTCAAAGGAGCACTTATGTGCCTATTCAAGATAAAGAGGTTGGATGTAGTTCCAAGAAGGGTAACAATAATAATAGTTCTCCTTCTACTACTACCCAAATTGAATCCAGTCGTATAGTTGCCAATGAAGCCAATCCCAAAGCTTCTTCCGATCATTCTAAGGTGGTGGACGAAGTGGGTTATTATGCAGAGATCAACTTAGTTGATATCATCAACTTAGATGAAACAGCACTCTGA

## >AhNAC50-Arahy.83IFXD.1

ATGGAAAATTTTGAAGTTGATCCTTCTTCTCATTCATTCATGCCCCCTTCCCCTCTTGGCCTAATCTCTTTCTCCACATGGCGACAAATTCCGTCAACCATAAAATCCCCCTCACTTCTCACCTCCCCAAAAATTTCAATCCTCTCTTCTTCCCTTTTCCTAGACTTTAGTTTGCTACACCAAACTTCTCTCTCCCTTCTGTTATTCTTATTATTCCTCACCAAGCCTATAACAAATATTTTGTTACACCAAATTATTAGGCTCTCAAAAATGGGATCACCGGAATCAAATTTGCCACCAGGTTTTAGGTTCCATCCAACCGATGAAGAACTCATTCTTCACTACCTTAGGAAGAAGGTAGCATCCATACCCTTACCTGTTTCCATCATCGCTGAGGTTGATATCTACAAATTGGATCCATGGGAATTACCAGTATCTTCACCGGAGGAATCACCGTCGAGTGAAGTTCAGGCTGCAGAAGAAAATGGTTTATTCAAGAACACCATTTTGAAGAGTCCAATTCCAACACTGTCGCCGTCGCCACCGCCGCCGCTGCCGCCGCAGCCACTTCTCTCTCAAAAATCTGTGTCATTCTCAAACCTCTTAGATGCCATGGACTACTCCATGCTCAGCACCATCTTATCTGAGAACAATAACAACAGCACCCTTGATCAGGAACAATACTCCCAGATCAACACCAACCAATTGAACCATTCATCGAACATGGAGAACACTAGTAACAGCAACATGATGCTGATGAGGTCAAAGCGCCAGATAGACGAGGAAACAACAACGGTGTTGCACCCATCAAAGAAGTTCCATCACCAACTTATGGGCTCTTCTTCTTGCAGCTTCCCTAATAACATTAACAACACAAACACTGCACAATACGAGAACCCGCAATGGAACTACCTTGTCAAGCAATCCTTCTTGAACCAGCACTTACTTCTCGCTCCTCATCTTCGATTTCAAGGATAA

## >AhNAC51-Arahy.83Q9A2.1

ATGGCAATTGCAGCGCCGAATTCATCTCCGACGATGAGTCTGAGTCACAGCCACAGCCACGAGGACGGGGGGACGACGACGGCGGCCTCCACCACGAACGACAACCTGAACGGAAACGGGAAGCAAGAGGATGATGATCACGAGCATGACATGGTGATGCCGGGGTTTCGTTTTCACCCGACAGAAGAAGAGCTGGTGGAGTTCTACCTTCGCCGTAAGGTGGAGGGCAAACGTTTCAACGTTGAGCTCATTACTTTCCTTGATCTTTATCGCTATGACCCTTGGGAGCTTCCTGGAATAAATACGAGAGTTGGCAGAATATTAAAGTTAGCACCGCTAGCGGAGATATCGCTGTGCCGGGTTTACAAAAGAGCTGGAGTTGAAGATCATCCATCGTTGCCGCGGTGTCTGCCAACGAGGGCTCCATCTTCAAGAACTGTTGATCATCAGAAGAACAAGCAGCAGCACCACAACGATCAACTCAACATGGGATTTGCGGGGAACACCGCCGATGGAGCTTCTGATAATCGTGATCATGATGTAACCACCGCTCTTGCCCTCTCCAAACACAACACAAATGCTTATCGTGCTCCTTCGCTGGGACTCCCACCGCTGCTTCTTCCCTTGGACGACGAAGCCGCCTTCGTCCTCATGCAGCAGCAGCAGCACCATGCTGGCCCTTCTTCAGGAACCACCACCATGATGGATGATCTCAACAGACTTGTAAGCTATCAACACCAGTACTACAACAGCAGCAGTAGCAGTAGTAACAATAATAATCCCAATCATCATCATCACCTGTTAATGCATCAACAACAACAGCAGCAGCAGCAAACTCCTCCTGCAATAATGTCTCTGAATAACACTCCTTCTCCGCTTGCAACCGCCTTCTCTGACCGCCTGTGGGAGTGGAATCCACTCCCGGAGGCCAACCAGCGCCAGTACAGCAACATGTCCTTCAAGTAA

## >AhNAC52-Arahy.8AKD3R.1

ATGGACAAGGATACTAGTTTGGAAATCCATCTCCCTCCTGGATTTAGATTCCACCCTTCTGATGAAGAGTTAATTGTTCACTATCTAAGAAACAAAGTCACTTCTTCACCACTTCCTGCCTCATTCATAGCAGAGATAGACCTCTACAAGTTCAATCCATGGGAGCTTCCAACAAATATACCTAGGTAA

## >AhNAC53-Arahy.8D109F.1

ATGGGTGCTGAAGCTGGTGCAACTGAGTGTTTCAGTAAGGCCATGGCGTCGATGCCTGGGTTTCGGTTTCATCCCACGGATGAGGAGCTGGTTATGTACTATCTGAAGAGGAAGATATGTGGGAAGAAGCTGAAACTCGACGTGATTCTAGAAACCGATGTTTACAAGTGGGATCCTGAGGAATTGCCAGGGATCTCTGTACTGAGGACTGGAGATAGGCAATGGTTCTTTTTCACTCATAGAGATAGGAAGTATCCTAATGGTGCGAGGTCCAACCGAGCAACAAGGCAAGGTTACTGGAAAGCAACAGGAAAGGATCGTAATGTGACCTGCAATTCTCGGTCAGTTGGATATGGTGCACCATTTAAAGAAGAGGAGTGGGCTGATGATGAATGTGTAGATTTCAATATTAACTCAGCAGATCGGGAGGTAGTAAATACTGTCCCTGTTAATGATCAGCTGCCTCCTTTGGCCGATGATGAAGTCACGGATATGATTAATCAAATTTTGGATAATGAGCTTGCCCTTGACCAGCAATTTGGTGACGACCTTCTTGAATTTCCTCAGGTTGTTGCTGAAGAAACACAAAGTACTGTGGTGGATCAGTTCTCTGAGGCAGTGACGGACCCCGAGTACAATGATATTTACCACTCAACCAGTCAGCACTATGATGCGCAGAATGTCAACTTCAATCAGTCGGTTGCATCTCACCTTCATGCCCCGGAAGGATCAGAAGTTATTTCTACTGCCAACATTCAAGTAGAAGACTATAACTTTCAGGAGGATGACTTCTTGGAAATCAACGATCTCAATGGTAGTGAACTTACAATTCCAAATATGGAAACACCAGTGGAGAACCTGCAGTTTGAAGATGGATTGTGTGAACTTGATCTGTTCCAAGATGCAGAGATGTTTCTTCGTGACTTGGGACCAATCAATGAGGAAACCATTCCACATTCATATATGAATAATGCCCCTGGAAGCAACATTGAAAATCAGAATTATCACTTGCTACCCAATCCAGAGGACACTACTCAAAATGTTCATGAATTTTGGATGCATGATGAAAGAAACACCCTGAGTCTGTTTGAAGGCTTTGATGATTCTGTCTCTCAACAAAATCCAGGTGCTGTATGTGACTCTGCCAGCTTTCCTACTACTGAAGGCTATGATAATCAAAGTAGCATTGCAGAAGATGTTGCTACAAGTAGATTCTCTTCGGCTCTCTGGTCCTTTGTTGAGTCAATACCTACCACTCCTGCATCAGCTGCGGAAAATGCTCTAGTGAATCGGGCTTTGAATCGAATGTCTAGCTTCAGCCGAGTGAAGATTAATATCAAGCCGACGAACACAGCTGCAGGTAAAGACACTGCAACTACGAAGAGAGTGGGCAGAAAAGGATTTTCATTCCTTTTCTTCCCAATTCTTATTGCTTTATGTGCTTTCTTATGGGTTTCTCTTGGAACTTTTAGATTATTAGGGAGATGCATCGCTCCTTGA

## >AhNAC54-Arahy.8E68ZN.1

ATGTCTTGTATCTATAAATTAACCCACACCCACTTTTCTCTGTCCTCACACACAAAACAACACTGCTCTTTGTCTCATCCTACAAATCCCTCTCTCTTCTTCTTCCAAAGGGGAGTTCCACTCAAACCCCCCTTCTTCTTCTTCTTCTTCTTTGAGATGGATAACAGGTTGGCCACAAACTCCTCTTATGCTTCTCTTAGATTGCCCGTTGGCTACAGATTCTGCCCCTCTGACGAGGTTTTTGTCTCTTGCTACCTCAAAAACAAGGCCCTTTCAAAAACATTGGATTTTGATGTTGTTCCTGTCTTCGATGTCTTCAACACTGAGCCCAAGAATCTCCCTTCAGGTGGAAAGGTGTTTCTGGAGACAAAGTACTTTTACTTTGATCTGAAAGAGCGTGTGTTTGAAGACAATAACAAGATTGAAGCAGGGAAAGGGCACTGGAAAAGGGTGGGGAAAGGGAATCAGGAGCTTCTAAATAACAACAACAAACTCATTGGGTTCAAGACCAAGTTTGTTTTTTGGAGGAAGAAGAACCGCACTCAATTTCTTAAAACTAAGTGGGTTATGTTTGAGTTCCGTGTTTTTCTCAACCCCTCTCAGATAATGTCGTCATGGGCTGGCTACAAAATATATCTGAAGAAGGATAAGAGGAGGAACAAGAAGGCAAAGTTTTCTTGCGAGGAAAGCAGTGATGATGAAGAAGAAGAAGCAGAAAGAGCAAGTGAAGTGAATTTTCCAGATGAGATAAGTGGAATTAACACAGGACCTCCTTCACCAACTTCATCTAATGAATCCTCTGTTACAAGTTAA

## >AhNAC55-Arahy.8GCY61.1

ATGGGTCTTAGAGATATTGGTGCTTCATTGCCACCTGGGTTTCGGTTCTATCCAAGTGATGAAGAATTAGTTCTTCACTATCTTTACAAGAAGATCACAAATGAGGAAGTTCTCAAGGGTACTTTGATGGAAATTGATTTGCACACATGTGAGCCATGGCAGCTTCCTGAGGTGGCTAAGCTCAATGCAAATGAATGGTACTTCTTCAGCTTCCGTGACCGCAAATACGCCACCGGCTTTCGCACCAATCGCGCCACGACATCTGGCTACTGGAAAGCGACCGGCAAGGATCGTACGGTTCTCGATCCCCTCACCCGCGAGGTCGTAGGGATGCGGAAGACCTTGGTGTTCTACAAGAATAGAGCCCCAAATGGCATCAAAACTGGTTGGATCATGCATGAGTTTCGCTTGGAGACCCCACACATGCCACCTAAGGAGGATTGGGTTTTATGTAGAGTGTTTCACAAGGGCAAAACAGACAATAGTGCCAAACTAAGCCCACAATTCATGTATGAGGCCACACCTTCATCCCTAACTTTGGCTTCATCATCATCATCCCCACCAACAAACCAAACAAATTGCAACAATTTGCATGTTATTGGGTATAACCAACTTCCCAATTTCTCATCATCATCATCACCAATGCCAATCCACCATAATCACCATCATCATCATCATCAAAACCAAAACGGTTCTTCCTCTTTGATGAATCTCCTTCAATTTTCCACTAAGGAAAATAGTACCATTACTCAACTAAGTCCCAAAGGTGGCGGTGGCGGTGACGACGGCGGCTATGGGTTCATGTGGGACATGGATCTTGAGGAAAATAGCTTCCATGATGGTGGGGTTATTGCATCAAACTTGAACGACATGAGATTTGAGGTTGATAATAACACTATGGTTATGTTGTAG

## >AhNAC56-Arahy.8KC8J6.1

ATGGCTAGAAGTGCCACAATTCCATTTCCAATACTTGATTTCATTCCTGTTGGATTCAGATTCAAGCCAACAGATGAAGAGCTTGTGAGCTATTACCTCAATCACAAGCTCCTAAATGACAATTTTCCAATCAATATTATCCCTGACATTGATCTTTGCAAGGTTGAACCTTGGCAAATTCCAGCATTATCAAAGATAAAATCGGATGATCCAGAATGGTTCTTCTTTAGTGGACGTGATTACAAGTATGGAAAGAGCAAAAGATCAAACAGGGCAACCAAAGGAGGATATTGGAAAGCCACAGGACAAGATAGATACATAAAGGAAAGAGGAACTACCAATGTAATTGGGAGCAAGAAGACACTGGTTTTCTATAGTGGCCGTGTTCCTAATGGTGTCAAAACCAATTGGGTTATCCATGAGTATCATGCTACTACCTTTGATGATAGCCAGAGGAATTTTGTTTTGTGTCGCTTGATGAAGAAAGCCGAGAGAAAATCCGAAGATGGAACTGATGCACAAGCTTGTGATGAGGGGGAACCTAGCACTCATATGGAAGAAGCAGATGAGAGTGTCCCAAGTATGTTTGAGTCGCCAGATGTGGACATGGGTTCGATCTTCCACACACTGCCTCAAGACGGATCATCATCACAGCACTCTCCAGTCAGCATTGAACAGCAAGAATCCTTCCCATTCTCCCCATCTGAAAATTATTACCTTGTAAATGAAGATAGAAGTATGCATATACAATTCGAAACAAACGAAGAGAAGCAAGATGCTGAGAAATTTGCGGATTCGATTTTGGATGGTGGAAGTATAGCTATGTTTGAAGAAAGACAGCAGCATCATACTTTCATGAATAATCACCTCCGCTCGGTCCCATCAATGAGGGTATGCTATGAAAGCAGTGACACAGATGCTGAAGTAGTCTCTAGACGGGCTGGTTCAAGAGAATACCATGTATCAAAAATGGTTCAATCATCACATAGTGCTGCGTGCACAGATAAAACTAGAAGTATCTCTTCAGAAGACTTTTGGGGAGTGGATTCATCTTCATGTGACTCAAATGCAGATAAACCTTTTGAGATCAGTTCTATTGAAATTTCTAGTCCTCCATCGGCTCTAAGTAGATCGAAAAATCAATATAATCCGAGGCTATCTCAAACACATAGGAAGGTTTCAAGCAATGCAATTCCCCATCTTGAGGATAAGAAGAAATTGACCACTGTGGAACAATCAAGAAGAGATCAAGAAAAAGCTCAAAAAACTAGTCCAGGAAAGAAGCTAGAAACCAGAAGCTCTGATGTTAATAGAATTGGTAGTTTCATCCACCTAGAGCCGTGTTCGTCGAGCGAAAGCCTGACTCCACGAGCGGTATACCTTGTTAATGTAGTTATTGGGATTTTGTTGCTTCTAGCCATTAGTTGGGATGTGCTATCTTGTTAG

## >AhNAC57-Arahy.90HG3Y.1

ATGGCAGAAACTAGAGTTCTACCTGTTGGATATAGGTTTCGTCCAACAGAAGAGGAACTTTTAATTCACTATCTCAATAACAAGCATTTGAAAAATGATGCAGAGATTAAGAACACTATTTCCCAAGTTGATCTTTGTAACTTTGATCCTTGGGATTTACCAGAACAATCGAAGGTGAAATCGGATGATCAAGAATGGTTTTTCTTCAATGAATTGAAATACATGAAAAACAAGCGGTGTAACAGAAAAACCAACATGGGATATTGGAAGATCACAGGAAAAGAGAGAATCATCAAAAGAAGAGGGACAGACAGTGTCATAGGTACAAAAAGAACACTAGTTTTCTACGAGCCTCCACATAATGTCAAAACCAATTGGGTTCTTCATGAATATCATGCATTTGATCAAAAGAGCAACATCGTATTGAGCCGTGTAATAATGAATGCTGAGAAAAGGGAACAGAAGCTAAAGACAAAAGCAAGCAACATAGTCGAAGAGGAGGAAGTAAAATGTGAAGATGAACCATGCAGCGAAATTACTGGCTGTGTTACCCAAGCAACTACAGAAGATGCAATCATTCCTGATAATGCATGTGTTTCATCCGAGCGGCAACAACCTCAAGTTATAGATTATGAAATTCTCTCATCGGGAGAACAATCTTCAGTAGCCCATTCCGATAATGAAAACAATAATGCCGCGGAAGCAACATGGAGGCAAGATGCCGATATGAATATCGAGTATTTTTGGAATTTGCTGTTTTCTAGCATCGATGCTGACCCTCATGCTGAGTTCTTAAATTCGGTGTTGGCAGGGGATGATCAACTCTATGTTGATTCCGGCCACCATTGA

## >AhNAC58-Arahy.919QYJ.1

ATGGCAGGAGCATCATGGTTGGTGGACAAAAGCAGAATTGCAACCAAAATCAAGAGTGCTTCAGGAGCTTGTGATCCTAACGAAGTTATTTGGAAAACCAATCCTACCAGGATTTGCCCGAATTGTCATCATCCTATTGACAATAGTGATGTAACTCAAGAGTGGCCTGGCTTACCAAAAGGGGTTAAATTTGATCCGTCAGATCAAGAGATAATCTGGCACTTGCTTGTAAAAGCTGGTGTAGGAAATTTAAAACCTCATCCTTTCATTGATGAGTTTATTACTACCCTTGAAGTGGATGATGGAATTTGTTATACTCATCCTCAACATTTACCTGGTGTCAAGCAAGACGGAAGGGCCTCACATTTCTTCCACAGAGCAATAAAGGCTTATAATACCGGCACTCGAAAGCGTCGAAAAGTACATGGTCAGGATGACGTTCGTTGGCACAAGACTGGAAGGACTAAACTGATCACCCTGAACGGGGTTCAAAAGGGTTGCAAAAAAATCATGGTTTTATATACAAATGCGGTGAGAGGAGGAAAGTCAGAGAAAACTAATTGGGTTATGCACCAATATCACCTTGGGACAGAAGAAGATGAAAAGGAAGGAGAATATGTTATTTCTAAAGTTTTTTATAAAGAAGACCAGGATATACCTGAAGCCGCAGAAAGTAAGAATGCAACAGTTGCGAAAGTAGATCCAGTCACTCCCAAATCCACGACTCCTGAGCCTCCTCGTAATGAAAGGCAAGATTCAGATCTAGGCCTGGATCTAGACCTAGGGCAAGAAGCACTTGCTTTTCCTGAGATGGATTGCTTAGATGAAATTCAAGCTGACTGTGAAGAATCTGCGAAAGCTAATCCACCAGTACTGGAGACACAAGAAAATGAAGGGATGGACAACAAGGAAACTAATGCTTATGAAGGACAACTGTGGTGGGATAGTGATTCACAGAATCTATTAGATTCACAACAACTCGTTGAAGCATTAACTCTCTGCGAAGATATATTTCACAGCCAATCTTCCAACAAAGACGATGAAAATGATAAGAACCAAACCGGTCTATCTGTGTATGCTCATCTAGGACCAGAGCATCTGAAGAAGGATATTGAGGAGTGTCAAAAGCTTGCTCCTGCAGGACCAGAGCATCAGAAGGATATTGAAGACGGCCAAAATCTTGACATCGACCTTGCAAATATAGAGCGGGATACTCCTCCTGAGCATCGACTAAGTCAGCTGGAATTTGGTTCGCAGGACAGCTATACTTACTGGGGTTTTCAAGGCGTAAACTAA

## >AhNAC59-Arahy.9732XC.1

ATGACTCAGTGCAACAGTAATGATTACCCTGAAAACAATCATAACACCATTGTGGAGAGGAACAAAGATATCTTAATTAGTAGAACTTGTCCATCATGTGGTCATCATATCAAATGTCAACAAGACCACCAGGGTGCTGGAATTCACGATTTACCTGGGCTACCAGCTGGAGTGAAGTTTGATCCAACAGATCAGGAAATTCTAGAACATTTGGAAGCCAAGGTGAGGTCTGATATTCACAAGCTTCACCCCTTAATTGATGAGTTTATCCCTACTCTTGAAGGCGAGAATGGAATCTGCTGCACCCATCCAGAAAAGTTGCCAGGAGTAGGCAAAGATGGGTTGATCCGTCACTTCTTTCACCGGCCATCCAAAGCATACACAACAGGAACAAGGAAAAGAAGAAAAGTTCACACTGATGCTGATGGCAGTGAAACAAGGTGGCACAAAACAGGTAAAACTAGACCAGTCTACATCAGTGGCAAGTTGAAAGGCTACAAGAAAATCCTTGTTCTCTACACCAACTACAAGAAGCAAAGGAAGCCTGAGAAAACAAACTGGGTCATGCACCAGTACCACCTTGGCAACAATGAAGAGGAGAAAGAAGGTGAGTTAGTTGTGTCTAAGGTTTTCTACCAGACACAACCTAGACAATGTGCAGGTTCACTACTCATCAAAGATTCATCATCATTCCCTGCTAAACTAAAGGATCAAAGTGGTGTTCATCATCATGAAGTGACTAATAATCATAAGAACAATGGGTTTGTGGAATACTACAATGCATCCTTTATAAGCTTTGCTCAAGGGGAACAACAACATAGGTCAAACAATCCCACATTGATTTCCCATTTTCCTGCTCATGATGGGGCTCCGTTCATTCCTTGA

## >AhNAC60-Arahy.9ZTQ0N.1

ATGGGAATTCAAGAGAAAGACCCTCTCTCGCAATTGAGTTTACCGCCGGGTTTCCGATTTTATCCGACGGACGAGGAGCTTCTCGTTCAGTATCTGTGCCGCAAGGTTGCTGGCCACCATTTCTCCCTGGAAATCATTGGCGAAATTGATTTGTATAAGTTCGACCCTTGGGTTCTTCCAAACTCTACCCGTAAGAACGGGAGCACCAAGCTTGACGATTGGGTTCTGTGCCGGATATACAAGAAGAATTCAAGCGCACAGCAGAAGGTACCAAACGGCGTCGTTTCGAGTAGCGAGCAATATGCCACGCAATACAGCAACGGATCTTCTTCAAACTCCTCTTCCTCCCACCTCGACGAGGTGCTCGAGTCCCTGCCGGAGATCGACGACCGTTGCTTCGCCTTGCCACGTGTCAACTCCTTAAGAGCGCTGCAGCAGCAGCGCCATCACCAAGAAGACACCAAGGTCGGCCTACTCCAACAGCAACAGCAACAGGGTCTCGTAGCCGGCACCGGTAGTTTCTTGGACTGGGCTTCCGGGCCGGGGATTCTGAACGATTTGGGCCAGGCCCAGCAGGGGATTGTTAACTACGGAAATGACCTCTTTGTCCCTTCAGTGTGCCACGTGGATTCCAATTTGGTGCCAGCAAAGATAGAAGAGGAGGTTCAGAGCGGTGTGAAGACTCAATCCGCATTCTTTCAGCAGGGACCGAACCCGAATGACTTCACACAAGCATTCTCAAACCAATTAGATCCTTACGGGTTTAGTAGGTACTCGGTTCAACCGGTTGGGTTCGGGTTCAGGCAATGA

## >AhNAC61-Arahy.A1VWSL.1

TCAAATAGTATGTATCAACATTCAAGTCTTTTCTTTTCTGTCCTCTTCTATGTATAGTCTACCTCATGACCAAGTCATAACCCAAATCACCATCATTAATCACAATTATTAAATAAATAAAGCCACAAATTAAATAGTTATGAGCTCCATTAACACAACCAACTCCACCTCCTTATAAATATACACACCCCTCTCAATATCATATTCATTTAAACATAACAACACAAAAAGAGAACCATAATTAAGCAAGCGACATAATTGATGATTGATATGGGTTCTTCATCAGTAATAGACGGTGAAGTTACACTTCCAGGATTCAGGTTTCACCCTACTGAAGAAGAGCTCCTTGATTTCTATCTCAAGAACATGGTCGTTGGAAAGAAGCTCCGTTTCGATGTCATAGGCTTCCTCAACATCTATCACCATGATCCCTGGGACTTGCCAGGGTTGGCGAAGGTAGGAGAGAGGGAATGGTATTTCTTTGTGCCTCGGGACAGAAAGCATGGAACCGGGGGAAGGCCAAACCGGACCACCGAGAAAGGGTTTTGGAAAGCAACCGGTTCGGACCGTAAGATCGTTACCTTGTCTGATCCGAAGCGCATCATTGGATTGAGGAAGACACTGGTTTTCTACGAGGGAAGAGCTCCACGTGGATCCAAGACCGATTGGGTCATGAATGAGTACCGTTTACCTGACAATAGCCCCTTGCCTAAGGACATAGTACTATGCAAGATATATAGGAAGGCGACTTCGTTGAAAGTGTTGGAGCAAAGAGCAGCAATAGAGGAAGAGATGAAGCAAATGGTAGGTTCCCCTGAATCTACACCTTCCTCCACAGACACCATGTCCTATGAAGAACAACAACAGAATCAGAATCAGAATCTGCAATTGTTACCAACACAACATGTTGTTACTAAGAAAGAGGTTGAAGCTGAAGTTGAAGAGGAAAAAATGGTACATGTTACATTGACAACAACAAAGCAAGAAAACAAGGACACAACAAAGAACAATAAAAGTAGTTGTTGTGGTAACACAAACACTAACACTAACACAAGTAGTCTTCAATTGCCATTTGGGAAGGACAAAATCCCAGAGCTTCAAATGCCTATGATGATCACTGATTGGACCCAAGACACATTTTGGGCTCAATTGAATAGTCCATGGCTCCAAAACTATACCTACTCCAACATATTAAACTTCTAG

## >AhNAC62-Arahy.A3J7SX.1

ATGGGTCGTGAAACCCTTCTTCATCCACCACCACCATCCACCACCGCCCCAACAGCCACACACACCCCACCACCACCGCCGCCGCCACTGCCGTCTCTACCTTCATTGACACAAGCAGGACCATCAGGAGGAGTATCAGCATCTGCACCTTCACCATCTGCTTCACCTTCTTCTCCTGCTATTGTTGCTACTACTGCTGTGGCTACTGCAGTTGCTGCTCCTCCTACCTCTCTTGCTCCTGGTTTCAGGTTCCATCCTACTGATGAGGAGCTTGTTATCTATTACCTCAAGCGCAAGGTTTGCGGCAAAAGCTTCCGATTTGATGCAATTTCTGAGGTTGACATCTACAGGAGCGAACCCTGGGACCTTGCAGATAAGTCGAGGTTGAAGACTAGGGACCAAGAATGGTACTTCTTTAGTGCACTGGACAAGAAGTATGGCAATGGTGGGAGGATGAACAGGGCCACAAGCAAAGGATACTGGAAGGCTACAGGGAACGATCGTCCGGTTAAGCATGAACAAAGGACTGTGGGGTTGAAGAAAACTCTGGTGTTCCATAGTGGAAGAGCCCCAGATGGTAAGAGGACCAATTGGGTCATGCATGAGTACCGACTCGTCGACGAAGAGCTGGAGAGGGCTAGGTCTGGATCCTCTCAGCCTCAGCTGGTTAATCATTTTTGTGCATTTCTCTTTCGGATGGAGAAGGATGCATATGTTTTGTGTAGAGTTTTTCACAAAAATAACATAGGACCTCCGAATGGGCAACGTTATGCACCTTTCGTTGAAGAGGAGTGGGATGATGCATCGGCATTGGTTCCTGGGGCAGAACCTGTGGAGGATGTTACCGTCACTGTTGCCCATCCTCTACGCATTGAAAGCAACGGTCGCACTTTATGCAGCGACAGGAGAAACAATGTTGCACAGGAAAGTAGCCTCTGTTGCCCCTATGAGTATGGTGCATTGGGTGGTTATCTCATATTTGTTGATTTCTGGTTTCGCAATAAACATTTTGAAGTTTATTATATGGGCCTCTATAACCCATACTTACCAAAGAAGAGCAGAGAAGAGAAGCAAGAACGAGATGGGCTTGCCTCGGACTATCTATCAACCCGGCTTACCATTAGTTCCATATTCTATCATATGACTAGTAGTGGAAAGTGGGATACTCAATCTAACAACAAAGTTCCATTTGATGTGAACAAGCTTCCCATTGAAACTCAAAGTCTGCTAGCTGTCTGCAAAAGGGAGAGTATGGCCGAGTTTCCATCACCTGAAAAGGAGGATAACTCGAAGCGTCAGATCGATGAGTATCCTTTGCCACAAACAGAAAACACCAAGCCTATCTCTCAAATATACAAGAGGAGGAGGCATTATTTGAATGTCAACCATTCAAATGTTAACGGAGATTCAGTCCGAACCATCCAAGAACCGCCATGTTCATCAACAATAACCACCGCCGCAACGACACTCCCGACGGTCGCCACCACAGCCTCCACTGCAATAACCAACGTTGCACCTAAAAAACATTTCTTGTCTGCACTGGTGGAGTTTTCTTTGATGGAATCCCTCGAATCAAAGGGAAATGCATCCGTTCAACCACCAGAGTTTGATGATGCTTCCTTGGAGGCATCCGTGCCGCCAAATTGTGTTAAGCTCATCAAACGCATGCAGGGCGAGATTTACAAACTTTCCGAGGAAAGGGAGACTATGAGGTTTGAGATGATGAGCGCACAAGCAATGATTAACATGCTCGAGTCGCGCATTGAAATTTTGAGCAAAGAAAATGAGGAACTGAAGAGCATGATTAATAACAATCCTTAG

## >AhNAC63-Arahy.A5ASCL.1

ATGGAAAACATGCCACCAGGTTATCGTTTCTACCCTACGGAAGAGGAGCTGATTTCATTCTATCTACGGAACAAGCTTGAAGGAGTGAGGGAGGACATGAATCGGGTTATTCCAGTTCTTGATATATATGAATATAGTCCAAGCGAACTCCCACAAATATCGGGAGAGGCGAGTGTTAGAGACAGCGAGCAGTGGTTCTTTTTCATTCCGCGGCAAGAAAGCGAAGCGCGTGGAGGGAGGCCGAAGAGGCTCACAACAACTGGGTACTGGAAAGCCACTGGCTCCCCTAACCATGTTTTCTCTTCCGATAATCGCGTCATCGGAATGAAGAGAACCATGGTTTTCTACTGCGGTAGAGCTCCTAACGGAACTAAAACCGATTGGAAGATGAATGAGTATAAAGCCATTGACACTCATCACCCTTCTTCTTCCTCCAACAACAGGGCGGTTCCTATGTTAAGGCAAGAATTCAGTTTATGCCGAGTGTACAAGAAAGCAAAATGTTTGAGGGCATTTGATAGAAGACCACCTCCAAGGAGGGACACGTATCCTCCTAGCCAGAACAACGGTTCATCATCTTTTGATCATCATCATCAACATAATCAAACGGTGGAGAAATCTTCAGGTGCAGGTAGCTCACCGGAGAGTTCGTGCTCCGAAGACCACGGACAGTGTTCTCATCGGACGGAGGATGTGGAAAACGCTAACGAGCCATTTCTCGATTGGGAGCAAATCGATTGGTTCTTAGGATCTTCACTGCCGGAACCATGA

## >AhNAC64-Arahy.A80DKX.1

ATGCGAGTAGTAGTCAAGTGTGAACGCCCAAGCAACCCCAACATTCCACCCTCCTCTTCCCTCACCTATTCTCTCGCTCTCTCTCATTTTTCTAGCTCCCAACGTTCCACATTAAGGGAGGGGAGAGCATTATTATTGCATCATTGGAGATCATCGCTTGGATTATTGAGGTTTCTGATAGTTTGTTAA

## >AhNAC65-Arahy.AIPG34.1

ATGGAAGGAAGTAGTAAAAGTTGTGAACTACTACCACCAGGGTTTAGATTCCACCCAACAGATGAGGAGCTAATTGTGTATTACCTTTGTAACCAAGCAACATCAAAGCCCTGCCCTGCTTCCATCATCCCTGAAGTTGACATCTACAAATTTGATCCATGGGAATTGCCAGGGAAGGCTGAGTTTGGGGAGAAAGAATGGTACTTCTTTAGCCCAAGGGAAAGGAAGTATCCCAATGGGGTTCGGCCTAACCGCGCAACAGTTTCTGGGTATTGGAAGGCCACAGGGACAGACAAGGCTATTTACAGCAAGTGTAAGCATGTTGGTGTCAAGAAGGCCTTGGTTTTCTACAGGGGTAGACCTCCAAAGGGGATCAAGACTGATTGGATCATGCACGAATATCGTCTTCTTCAACAATCTAATCACAACAGCAGGATCACTGGTTCTATGAGACTGGATGACTGCGTCTTGTGTAGGATATATAAGAAAAAACATGCTGCTAAAGCATTGGATCAAGGACAGGAATACCCAACAACAGTTCAAATTAATCTAAATGCATCAACCAACAATGATGATGAGAAGGAGTTGATGATGATGAAGAATCTTCCAAGGACTTGTTCCCTTACTTATCTTTTGGACATGAATTACTTTGGTCCAATCTCACAGCTATTGTCTGATGGATCCTACAACAAGTCATCAACCTTTGAAATATTTCAACATAGCAATAGTGTTGACAACATTGGAATAGTGGATCCTCTTGTCAAAACTCAAATGGTTGAAATGGATGATAGCTATTATGCTCAAGATTCAGGCAAGTCCCAAGTGATGAAGCAAGGGAATGATTTAAGAGGATATTACTAA

## >AhNAC66-Arahy.B9XEKF.1

ATGGACAGCTTCTACCACCACCATAACCACCACTTTGACAACAGCGACACTCACTTGCCACCTGGATTCCGATTCCACCCCACCGATGAAGAACTCATCACCTACTACCTCGTCAAGAAGGTTCTCGACAACACCTTCACCGGAAGAGCCATAGCTGAAGTTGACCTCAACAAGTGTGAGCCATGGGAGCTCCCTGAGAAAGCGAAGATGGGTGAGAAAGAATGGTACTTCTTCAGCTTAAGGGACAGGAAGTACCCAACTGGGTTGCGCACAAATAGAGCGACGGAAGCTGGGTACTGGAAAGCCACTGGAAAAGACAGAGAGATCTACAGCTCCAAGACTTGTGCTCTTGTTGGAATGAAGAAGACACTTGTTTTCTACAGAGGAAGAGCTCCCAAGGGTGAGAAGAGCAACTGGGTGATGCATGAGTATCGGCTTGAAGGCAAATTTGCATACCATTATCTCTCTAGAAGCTCCAAGGATGAGTGGGTGATTTCTCGTGTGTTCCAGAAGACAACCACCGGGGGTGGTGGTGGAGGTGGAGGTGGAGGCTCAGCCGTGTCAACCACCGCCGGCGGGTCCAAGAAGGCGAAAATGAGCACTTCAACCACCACTTCTACAATGAGCTTCTGCCCTGAACCAAGTTCTCCCTCTTCGGTTTACCTTCCACCACTTCTCGACTCTTCACCTTACACTACCGCCACCACAGGCTCCGTCACCTCCGCCGCCGCAGCATACGACGGCCGCCAGAGCTCCTCCTTCGACAACAACAACAACAACAACGATAGCACAAGGGAGCACGTGTCCTGTTTCTCCACAATCTCCAACAACTTTGTCAATGGGTTCTTCGATCTTGCTCCTATGGATTCCTTCGCTCGATTCCAAAGAAACAACAATGTCGGTGTTTCTGCATTCCCAAGTCTAAGGTCGCTGCAAGATAACCTTCAAATTAACCCTTTGTTTTTCTCCGCCGCAGCGGCGCAGCCTCTCCACGGCGGCGAGCTTCACGCCGCGGGGACCTGGCCGGTGCCGGATGATCAGAGGGTTGCCGAGGCTGCTGCCGCCGGCATGGCTTTGGGACATTCCGAGCTTGATTGCATGTGGGGCTATTGA

## >AhNAC67-Arahy.BEF3I8.1

ATGCAAGAGGAAATAATGACTCAGTGCAACAGTAATAATGATTACCCTGAAAACAATCATAGCACCATTGTGGAGAGGAACAAAGATAGCTTAATTAGTAGAACTTGTCCATCATGTGGTCATCATATCAAATGCCAACAAGACCACCAGGGTGCTGGAATTCACGATTTACCTGGGCTACCAGCTGGAGTGAAGTTTGATCCAACAGATCAGGAAATTCTAGAACATTTGGAAGCCAAGGTGAGGTCTGATATTCACAAGCTTCACCCCTTAATTGATGAGTTTATCCCTACTCTTGAAGGCGAGAATGGAATCTGCTGCACCCATCCAGAAAAGTTACCAGGAGTAGGCAAAGATGGGTTGATCCGTCACTTCTTTCACCGGCCATCGAAAGCATACACAACAGGAACAAGGAAAAGAAGAAAGGTTCACATTGATGCTGATGGCAGTGAAACAAGGTGGCACAAAACAGGTAAAACTAGACCAGTCTACATCAGTGGCAAGTTGAAAGGTTACAAGAAAATCCTTGTTCTTTACACCAACTACAAGAAGCAAAGGAAGCCTGAGAAAACAAACTGGGTCATGCACCAGTACCACCTTGGCAACAATGAAGAGGAGAAGGAAGGTGAGTTAGTTGTGTCTAAGGTTTTCTACCAGACACAACCTAGACAATGTGCAGGTTCACTACTCATCAAAGATTCATCATCATTCCCTGCTAAACTAAAGGATCAAAGTGGTGTTCATCATCATGAAGTGACTAATAATCATAAGAACAATGGGTTTGTGGAATACTACAATGCATCCTTTATAAGCTTTGCTCAAGGGGAACAACAACATAGGTCAAACAATCCCACATTGATTTCCCATTTTCCTGCTCATGATGGGGCTCCTTTCATTCCTTGA

## >AhNAC68-Arahy.BFU0GS.1

CTTTCAAAAATGGCATCGGAGCTTCAATTGCCGCCTGGATTTCGATTCCATCCAACAGATGAAGAACTCGTGTTGCACTATCTCTGCCGTAAATGCACTTCACAACCGATTGCTGTTCCGATCATTGCCGAGATCGACCTTTATAAATATGATCCCTGGGACCTTCCTGACGTAGATCGTTCTGTTCGCAAAAAGAATAGCCTCAGGCTGGATGACTGGGTGCTGTGTCGCATCTACAATAAGAAAGGATCAATCGAGAAGCAACAACCGAGCAGCGGCGTCAGTACCGTCGTGAACCAGAGGGCCGAATCTTCGGAAGTCGAAGACAAGAAGCCCGATATAGTTCCACGTGGCGGCGGCGGGGGCGTACTTCCACCGCATCCTCCTACGGCTCAGGCTTCGGCAGGCGGCGTGACGACAGATTATATGTACTTCGACAACTCCGATTCAGTTCCGAAGCTTCACACGGACTCGAGTTGCTCGGAGCAGGTGGTGTCGCCGGAGTTTGCGAGCGAGGTTCAGAGCGAGCCAAAGTGGAACGAGTGGGATAAAAACCTCGAAAGTGCGTATAATTACCTCGATGCCACACTCACGAACGGTTTTGGGTTCCCGTTCCAGGGTAACAATCAGATGTCGCCGCTTCAGGATATGTTCATGAACCTTCCGAAACCGTTCTGA

## >AhNAC69-Arahy.BN8407.1

TTCACTCTCCTCTCTCTCTCTCTCTCTCTCTCTCTCTACTCTCTTAACCATTATTGCTCTAATGGCTCCAATGAGTCTCCCACCTGGTTTTAGGTTTCACCCCACAGATGAAGAGCTCGTTGCTTACTACTTAGAAAGGAAGATAACAGGTCGCTCTATAGAGCTTGACGTTATAGCTGAAGTTGATTTATACAAATGTGAACCATGGGATTTGCCAGATAAGTCATTTCTACCAAGCAAGGATATGGAGTGGTATTTCTACAGTCCAAGGGATAGGAAGTATCCAAATGGATCAAGAACGAACAGGGCAACAAGAGGCGGGTACTGGAAAGCGACTGGAAAGGACAGGGCAGTGCAGTCTCAGAAGAAGGCAGTTGGTATGAAGAAGACTTTGGTGTATTACAAAGGAAGAGCTCCACATGGAATTAGAACCAACTGGGTCATGCATGAGTACCGCTTGATTGAATCCCTCCCTGGAACTCCTCACTCCTCTTTCAAGGATTCCTTTTCATTGTGTCGGATTTTCAAAAAGACAATTCAAGTTCAAGACAAATCTAAAGAAGAGAAAGAACATCAAGCATTACTAGAGGAAGATCACTCAAGTGGCATTGAGATTTCAAGAGAAATGGAAGCCATGAATGATAATAATAACAATAATATAACTCTAAATAGTAATGAACAATATCCTAATAATAATAATAATAAACTCCCTAATTGTGATGCTTCATCTTCTGATCTCACTCAAGGAACATGTACACCCACAGAAACCGGTATAGCAGATGATTTTCATGCCCAATTTGCTTGTGATGAAGCAAATAGTGCCGCTAATTCTTACTCAATGGGAATAGCATACCCCTCAAACGTTTTTCAGGACATAGAGATGTCCATGTATGGAAGCATGCATAATTATCAATTCCCACAAACGCCTTTGGTGATGGAAGATTTTCCACAAATAGATTTTGCTGAGACAAAGTCATTGAAGCCAGAGGTGACTGAAGACTGCATGTTCTATGATAGATACGGTAGGGATTGTATGAATGGAACACTAGAAGAAATCATCTCATTGTGTTCCTCTCAAGACAACTCTGTGGCTTTGCCCATGCTAGAATGATTATATTACTAACGAAAAAAAAAAAAACAAAATGACACGAAAAAAAAAAAAAACAGTATCAAAATATTCATTTGACACAGAAGAAACTATAGCTACTTGTGTTGGCCAAAATAACCACACACAAAAGGAGACATTTTTTCTAGCAAGAGAGAAGCAGCAGCTACATATATAATGTACTAAAGATACATAGTAGAATATGCAGAGCCAGGGAGGCTGGCATATAGTTCACTTGGTTTCTTTTTCCTTTTTTTTTGGGTGTTCATCTTTTTTTTGAACGGGGTGTATGTCTTTCTACAATATAATTTTTAATATATATATATTTGTGTTCAAATCTCTTG

## >AhNAC70-Arahy.BPCJ1X.1

TTTTATATGAATTTTTTATTCGTTAATACAAACAAATACTTAATCGGATTAAGACAAGAAAATAAAAAATGAGATGAAGCAGCTAAGGAAACGGTGGGATGTCAGAATGAGGGTGAAAGATAAAAGCGGAAGCACTGACTAGTTGTAATCGAAGTGGATAACATGCGAATGGCACGTCACAAGAATGACACCGAACCTGCTTGGTTCCCTAGCCGACCCTGTTTCACTCAAAGTCTAAAAGCCTAAATTGCCATACCAAGTTGTTGTTACTCACAAACAAGCAAAGAACAGAACAATATAACCCTCTCTGACACCAAACCCTTCTACCTTCACCTTCGAATCATGGTGGACAGGGATTCAAGCGAAGCACACATGTCAATAGCAGCTTCTTCGATATTCCCTGGCTTTAGGTTCTGTCCCACTGACGAGGAGTTGATCTCTTATTACCTCAGGAAAAAGCTGGACGGTGATGAGGACAGTGTTCAGGTCATTTCGGAGCTTGAGCTTTGCACATTTGAGCCTTGGGATTTGCCAGGAAAATCCTTCATTCAATCTGACAACGAGTGGTTTTTCTTCTCGCCACGGGGGAGAAAGTATCCCAATGGTTCACAGAGTAAAAGGGCAACTGAATGTGGATATTGGAAGGCCACAGGGAAAGAACGCGTTGTAAAGTCTGGTCAGAATGTTATTGGTACCAAACGCACTTTGGTATTCCATCTCGGTCGAGCTCCTAAAGGCGAAAGAACTGAATGGATTATGCATGAGTACTGCGTCAATGACAAATCTCAGGATTCATTGGTAATTTGTCGGCTCAAGAGGAATACAGAATTCCGTGCAAGTGATCATTCTAACAGAACTTCACACGATAGTGATTGTGGAGTCTCAGAAGGAGTTACAGTTCAAGGGGACACTTATGTGCCTATTCAAGATAAAGAGACTGGATGCAGCTCCAAGAGGACTAGCAGTAGTAATAGTTCTCCATCTACTACTGGCCAAATTGAATCCAGTCATAGAGTTGCCAATGAAGGCAATGAAGCCAATCAAGCAAATGAAGCCAATGTAGCCAATGAAATCAATCAAGTCAATGAAGCCATTGAAGCTAATGTAGCCAATATAGCCAAGGAAGCCAATCAAGCCAATGAAGTCAGTGTAGCCAATGAAGTTAATATAGCCAATGAAACCAATGAAGCCAATGTAGCCAATGAAGTCAATATAACCAATGAAGCCAATCAAGTCAATCAAGCCAATGAAGTTAATATAGCCAATGAAGCCAATCAAGTCAATCAAGCCAATGAAGCCAATGTAGCCAATCCCAGAAATCACCCTCAGGTGAATGAAGAAGAGGATTGTTATGCAGAGATCCTAAATGATGATATCATCAAGTTAGATGAATCAACACTCTCACGGCCATCGCCACCACAAGGGACAGCAAACAGGAGAATCAGGCTAAGGGTTCCCAAATCAACAGTTCCCAAATCAAGGGTTCCCAAATCAAGGGTTCCAACGGGAAATGGTTGTCAATGTTCCAAGCAATCATCAAACAAAATTAACACCTTCCTGTCATACCCTTTGGTGGTCTTCACTTTCTTCGTTTTCACTTTGTTAGCTCTAGGCTTCTTTTTTATTAGGAGGTCCCAAACTACTGCCCAATATTCTCAAGACCTCTCTAGAGTTTAATTATGACTATTAATTAATTAGTGTTTAGCGTTGCATAGCCTTTGGATCAATGTTACGTTGTATACAAAATTACAAACAAATGATGCACTCTTCATAGTAAAATAGTAAGGATAAGGATTATATTATTCGTTACATTTCTTTATTGTGCATCTGAATATATATTAAACCAACGTTGAATTGCATT

## >AhNAC71-Arahy.BS3I7W.1

ATAGAGAAAGCATAGAGAATAGTTTTCACATGTATGTGTTGTTTCACTTGAGACTTTAAGTGTTTTTAGAGTCATGGATGTGGCTAAGTTGTACATGAACAACGACTACTCCGAAGAACATGAACATGAATATCAACATGATGAAGATGATGATGATGAGATGATGAAAGAGGAGAAAGAAGTTGTGCTTCCTGGGTTTAGATTCCACCCAACAGATGAAGAGCTTGTTGGGTTTTATCTTCGGAGGAAGGTTGAGAAGAAGACTCTTAAGATTGAACTTATCAAACATGTTGATATCTACAAATATGATCCATGGGATCTTCCAAGCACCAAAACTGATTGGATGATGCATGAGTTTCGCCTCCCACCCAATAATAATAATGGAGCAAAATTATTAAGCAATAATCAAGAAGCTAATAATGCTACCAAGGATCTTCATGAAGCTGAAGTGTGGACACTATGCAGAATATTCAAAAGGATTCCAACATACAAAAAGTACACACCAAATTTGAAAGATTCATCAACATCACCACTCATGAACAAACCCATCAATAACATTAACCACCAAACTGATTCCTCAGTAACTTCCATATCATGCAGCTTAGAATCTGACAACAACAATAGCAAGCAATTCTTGACTTTCACTAACACTATGGGGATTCAACAATGTGAAAGGAAGCCTCTTGTTATTGGACATGTTGATGAAAGGAACAACAACTTTTTCTTAGACCATTCATCAATACATCATCAACAAGCTCCAACAACAATTACTACTACTGCTTTGTCATCATCATCATACTCATCATGGAACCAGCACCATGTTGTGGAGGATTACTTGTTTGCAAATGAGAATTGGGATGATCTTAGATCTGTGGTTGAGTTTGCCACTGACCCTAATAATTCCAAGGTTTATCTATGATTGTAATTAAATATATGTTTTGAGAGGGTATTATTATTAGGGTATTTAGCTTCTTAAAACTACACTACTACTACTACTATTACTACCAATAATAGTGACATGTATTACTATTATGTATATCTATCTTACTCTAACCCCAAATTCTTTTCTTGGGGTTGAAGTGAGAAAGTTTGATAGAGTATGTTTGTATGAAAAATAGGCCTTCAGTTTTGTTTTGACATTGTTCACATACACTTGGTTCTTTTGCTAATTTAATATATAGTTATATACAGATATAGTTTTATTTATAC

## >AhNAC72-Arahy.BX5EMB.1

GTTAGATATATGCATTTAACTATGATATCTAATATGAGGATTCTTCTTGTTGAAGTTGTGAGGAGTTATAAAACCATAAAGTGGAGAACCATTTAATAAGAGCTTCATCTTGGTCATGTATGTTATAATTGATGAGGATTCTTCACATCATAGCTTGGTAAACTGAAATTAATTAAGGTCATCATCAAAGGGGTTTTAGGGTTCTTCATAGGAAGAGTAATTGATGGAAGAGAATCTACCTCCTGGATTCAGATTCCACCCAACAGATGAAGAGCTTATAACATATTATCTTACAAGAAAAGTCTCTGAAAATGGATTCACTTCTAAAGCTATTGCTGTTGTTGATCTCAACAAGTCTGAGCCTTGGGATCTTCCAGGTAAGGCAAGCATGGGTGAGAAGGAATGGTACTTCTTCAGTTTAAGAGATAGAAAGTATCCAACAGGACTAAGAACAAATAGGGCAACAGAATCAGGGTATTGGAAGACCACAGGCAAAGACAAAGAGATATTTCGTGGTGGGGTTTTGGTTGGAATGAAGAAAACCCTAGTCTTCTATAAGGGTAGGGCTCCAAGGGGTGAGAAAAGTAATTGGGTCATGCATGAATATAGACTTGAGAACAAGAATCCCTTTAGAACTAAGGATGAATGGGTAGTGTGCAGGGTATTCCAAAAGAGCACAGCAGCGAAAAAACCGCCGCAACAAACATCATCCTCCCAACCTGAATCCCCATGTGATGACACAACCTCTTTGGTCAATGAATTTGGTGATGTTATTGAGTTTCCAAATCTAAACACCAACATTAATAATACCAATAATAATAATAATAATTCTTCATCATCATCCTCAAGTGCATTATTCCCTAACAACATTCTTATTTCAGGACAACACATTCATCATCACCATGACCTAACCAATAATAACAATAATAACAATGTTAACACAAACATGAACTTAGCAATGAATTGGCCACCATCAAGTGATCATAATGTTCCATGGCCTTCAGTAGGGTTGTTGAATCCAAGTATTTCATCAATGAATTCCTTGATTCTCAAGGCATTGCAGCTTAGGAATAATTATCAACAAAGAGAAGTTGCATCCACATTTGCACCATCATCATATATTATGCCTCATCATCAAGGAGTAGTAGTTCCTCATCAACAAGTTATTATTGGAACCAATAATGATGACCTAATAACAACTTCTTCAAATCTCATCAATGCTTCTTCTTCTTCTTCATCAAAAGTTTTGGAATGTATGCCACATCAGCAACAACAACAACAGGAGCAACCATTCAATTTGGACTCCCTTTGGTAAATTGATGAATGAATTGAAAAGCATATATATAATAATGTATATGAAGCTCTAGCTTGCTAGATAGCTAACATCCATCTCATCTTGTAAAACTTCTTGTGAATTATTATATTATTAATATATATGTCCATGTAACGCTCCCATTCCTACATGAAAAAAAAATTGTAACATTTTCAAAATTGTAATAAACATCCTAATTTTTAGTAGTTATGTATGTGTCCTTTCTTTGCCCTTCGGTAATAAATAAAAAGTCAACCAACCTTAATGTTGATGTTGTGAATAGTGATAATAATCAGTTGTTGTAATTAGGTGTAAAATTAGGGTCCTTTCAGTCGTTTTAGGTTTCATGTTCTAGGATTAACAACTCAAGAATTTTCTATAAAATAACCGTTTTTCTTTCTCCTATTTTAAAGCATAGGTTAGGCATATTA

## >AhNAC73-Arahy.CDPA7L.1

CGGTGGGATGTTAGAATGGGGTGATAGATAAAAGCGGAAGCACTGATTAGTTGCCATCGAAGTAGATAACATGCGAATGCCACGTCACAAGAATGACACCGAACCTGCTTGGTTTTCTAGCCGACCCTGTTTCACTCAAAGTCTAAAGCCTAAATTGCCATACCAAGTTGTTGTTACTCACAAACAAGTAAAGAACAGAACAACATAACCCTCTCTGACACCAAACCCTTTTATCTTCACCTTCGAATCATGGTGGACACGGATTCAAGCGAAGCACACATGTCAATAGCAGCTTCTTCCATATTCCCTGGCTTCAGGTTCTGTCCCACTGACGAGGAGTTGATCTCTTATTACCTCAGGAAAAAGCTGGACGGTGATGAAGACAGTGTTCAAGTCATTTCGAAGCTTGAACTTTGCACTTTTGAGCCTTGGGATTTGCCAGGAAAATCTTTCATTCAATCAGACACCGAGTGGTTTTTCTTCTCGCCACGGGGGAGAAAGTATCCCAATGGTTCACAGAGTAAAAGGGCAACTGAATGTGGGTATTGGAAGGCCACAGGAAAAGAACGCATCGTAAAATCCGGTCAGGATGTTATTGGTACCAAACGCACTTTAGTATTTCATCTTGGTCGAGCTCCTAAAGGCGAAAGAACTGAATGGATTATGCATGAGTACTGTGTCAATGACAAATCTCAGGATTCATTGGTGATTTGTCGGCTGAAGAAAAATACAGAATTTCGTGCTAATGATCATTCTAATAAAACTTCACATGATAGTGATTGTGGAGTCTCAGAAGGAGCTACAGTTCAAGGGGGCACTTATGTGCCTATTCAAGACAAAGAAACTGGATGCAGCTCCAAGAGAACTAGCAGTAGTAATAGTTCTCCTTCTACTACTACTGGCCAAATTGAATCCAGTCATAGAGTTGTCAATGAAGCCAATCAAGCCAATGAAGTCAACATAGCCAATGAAACCAATCAAGTCAATGAAGCCATTGAAACTAATGTAGCCAATGAAGTCAATATAGCTAATGAAGTCAATCAAGTCAATCAAGCCAATGAAGTCAATGTAGCCAATGAAGTCAATCAACTCAATGAAGCCAATGAAACCATTCAACTCAATGAAGCCAATGAAGCCAATGTAGCCAATGAAGTCAATATAGGTAATGAAGTCAATCAAGCCAATGAAGTCAATATAGTCCATGAAGCCAATCAAGTCAATCAAGCCAATCAAGCCAATGTAGCCAATCAAGCCAATCCCAGAAATCACCCTCAGGTGGACGAAGATGATTGTTATGCAGAGATCCTAAATGATGATATCATCAAGTTAGATGAATCAACACTCTCACGGCCATCGCCACCACAAGGGACAGCAAACAGGAGAATCAGGCTAAGGGTTCCCAAATCAACAGTTCCCAAATCAAGGGTTCCCAAATCAACGGTTCCAACAGGAAATGGTTGCCATTGCTCCACGCAATCATCAAACAAAATTAACACCTTCCTGCCATATGCTTTGGTGGTCTTCACTTTCTTCGTTTTCACTTTGTTAGCTCTAGGCTTCTTTCTTATTAGGAGGTCCCAAACTACTGCGCAATATTCTCGAGACCTCTCTAGAGTTAATTAAGACTATTAATTAATTAGTGTTTAGTGTTGCATAGCCTTTGGATCAATGTTAGTATGTTACGTTGTATACAAAATTACAAACAAATATGCACTCTTCATAGTGAAATAGTAAGGATAAGGATTATATTATTCGTTACCTTCCTTTATTTTGCATCTGAATACATATTAAACCAACGTTGAA

## >AhNAC74-Arahy.CK11CG.1

CCAATTTTTCAAATATATATATAAAATCCACTATTTATAACCATTTAATAAGAACAACATCTTCATTCAACTCTACTCCTTCCATTATTCTCTCTCTCTCTTTTTCCTTCTTTTCAGTTACATAACAATTTAATACATCAATTATTTTATCATCAAGACCAATAATAATAGAAATAATAACATAGAAATGAATACAAAGATTGAACTGCCACCAGGTTTCAGGTTTCATCCAACAGATGAAGAGCTCATAACTCACTACCTCTCTCAGAAGGTTGTTGCTAGCTGCTTCTATGCAACTGCCATTATTGGAGAGGCTGATTTCAACAAGTGTGAGCCTTGGGATTTACCTTGGAGGGGCAAAATGGGAGAAAAAGAATGGTATTTTTTCTGTTTGAAGGACAAAAAATACCCAACAGGTGAAAGGACAAATAGAGCCACTGGTGCTGGGTACTGGAAGGCCACAGGAAAAGACAGAGAGATATACAATGCAAAAGCAAAAGCACTTATTGGGATGAAGAAAACACTTGTTTTCTACAAAGGAAGAGCTCCAAATGGTGAAAAGACAAATTGGGTCATGCATGAATATAGGTTGGAAGGCGATAATAAACCTTCTATATACAATCTTCCCAAAACAACCAAGAAAGAGTGGGCTTTGTGCAGAGTTCTACACAAAAGTGAAAAGAAAGTAATGCATGTTCCACAACCACAGGGATTGGTTGAGTTCAGCTCTTATGAAAATAAGGAACTTCCCCAATTGATGGATTCTTCACAAGTAACATTCTTTTCATCAGACCCAAATAATCAAAGTGAGGATCCAAATCCAATCACACGTGATGATGATAATAATAATAATGATGACATCATAGTTGATAGCATTGAAACTCCTTTCTTGGAACAACAACCACCTTATTATTCTTCATCCTATGATTCTTCAGATTTAGACACCCTTAACCCTGCCACATGGGATATTTCCGAAAATGCCCCTACAAGTAATGCGTCTAAGGAGACGGACTTTGATGCTGACATGTTCTCTTTGATGTACAACAATAGAGAAGTGTTCCAAACATCATTTGAGAATCAGGAATATTATGCATATGATTCTATGGGACATGTGGACAATGGTTCCCTATGGAATTTTTAGAGAATTAGGATTATGATTAGAATTGGTTTAATTTTTCTAATAATTTCAACTTATTAAGGTTAACTCAGTTGATTAGGATAACATAGTTTAGCTTCCTTCTTCTGTCTTTAGATCTTGGGCTTGAACCCATTTGTAATAAAAAGAAAAAATTATATATTA

## >AhNAC75-Arahy.CRX62L.1

TGGGGTGTGATGAATGAATACACTTTTGCAGAATACTAAGCTCTCTAACAATCAAATCAATACAAGAGAGAAAAATAATATAATGTCACCAGTTGGATTACCACCTGGGTTTAGGTTTCATCCAACAGATGAAGAGCTTGTTAACTATTATCTAAAGAGGAAGATCAATGGCCAAGAAATTGAACTTGATATCATTCCTGAGGTTGATCTCTACAAATGTGAACCATGGGAATTAGCAGAAAAATCATTTTTGCCGAGTAGAGATCCAGAGTGGTATTTCTTTGGACCAAGGGACAGAAAATACCCTAACGGATTTAGAACAAATAGAGCAACACGAGCAGGGTACTGGAAATCAACAGGTAAAGACAGGAGAGTTTCAAGCCAAAGCAGACCAATTGGTATGAAGAAGACTTTGGTTTATTATAGAGGAAGGGCTCCTCAAGGAATCAGAACTGATTGGGTTATGCACGAATATCGTCTCGATGACAAGGACTCTGAAGACACCACCGGTTTACAGGATACTTATGCTTTGTGCCGTGTGTTCAAGAAAAATGGAATATGTACGGATGTTGAAGAGCAAGTAGGGCATTGTAGTAACATGTCTTCACTAATTGAGAGCTCACAAACCATAATCAATAATAATAACAATAATAGTAATAATAATAATGAGTATTGTGAAACCATGTCACCAGACATAGCAGGGGTTTCATCTTCATGTTTGGAAGAGGAAGACAAAGATGATTCATGGATGCAGTTCATCACGGAAGATGCATGGTACTCTTCTAATGCACCAAATATGGTTGGTGGTGAAGAAGTTTCACATGTTACATTTACAAGCTAATATATATTTTTCAAGATTGGTGGTCAATAATTAATAACTTTGGGAACATCTCATCATATATATAGTAATTAATAAAAAAAATATCTAAATACTTTTTTTAATTTACACCATAATTAACTTATTTTTATTATTGAGATACTAGTTAACACTTTCTTTACTCTAATTATTATGGTTAATTAATTGGTGAGGATGGTTTGGTTTGGTTTCTTTTTTTTTTTTTTTTTTGGGTGGGTTTGTTAGGGGTCATATTATTTTGGATTTTTGTACTAACCTCATCTCGAGGTTACGTGGGTGTCACCTGTGGAATAAAGAAAAAAACTACCACACAAAGTGTAAGTTTCATTATGTTATTTTTTTTTTCCTCCCTATGGAAGGGATTATAACACTTGAAAGGCAATTGAATATAATACTATAGTTCGATTAAAAAAT

## >AhNAC76-Arahy.CSHQ77.1

AAAACAATACTTATCCCACATATATGGTTATGGTGTGAACTGGGTTTTGAAGTGGTGGGCCATTTTTCCTTGGAATTTTTGGGAGCACTTTGGGACAAGTTATCTCATACTTTCGGTGTGTTTTGTGCAATTTTGGACTGTTGGTTCCCCTTCCCTTCTCTCCCTCCAAATGCTATAAATATCACTCCACCCATGCCTTATGCTTTTACTACTCAAATTGTTAACATACTACTCAAATTAAACTTGTTTTGAGTTTAACCAATTTGATTTCTTAATTTTCTTGTTATGCAAAAAATAATTTATTAGAGGTCAAAAGTAGTTGGTGGTTGAGTTAGAGTAATTTGAACTTTCTGGGTAAGTTAGTTCATTTTATCATTTGAGGGTGGGTGGTTTGGGATTAGGATAGTGGTAGAGATAGTGTTATTTCAAAGAGAATGATCATTGCATACAGTGGTTGCAGACTACTGTGGAAGTATCACTTGAAAACTATTGTTATCCACAAAAGCTTCTATTTTTGGATCTTCCAAATCTTCAATACATATGGGCATGCCACTTAGCATCATAAAGCTTTGAGCTTTCTTGCTGGAAAATTGTGGGTTTATTTTTTTGGTTACTTTCTTGCTAATAAGTTTTCATAAACTTTGGTTGGTCTTTTTTGTTCTTTTAATCATAATGGGAGGGGCATCACTGCCACCTGGATTTCGTTTCCACCCAACGGATGAAGAATTATTGGGATATTACCTAAAAAGAAAAGTGGAAGGGCTTGAAATTGAGCTTGAGGTTATTCCTGTGATTGATTTGTGCAAGTTTGATCCTTGGGAATTGCCCGAGAAATCATTGTTGGCAAACAGAGACATGGAATGGTTCTTCTTTTGTCCAAGGGACCGCAAGTACCCAAATGGATCAAGAACTAACAGAGCCACCAAAGCTGGTTATTGGAAAGCCACTGGAAAAGACAAGAAAGTTGTGTGCCAATTTGATACTCCTTCCACTGTCACAGGATATAGAAAAACCCTTGTCTTCTACCGTGGCAGAGCCCCTTTAGGTGACAGAACTGATTGGCTCATGCATGAGTATCACCTCGCCGATGATCTCGGCCTAGCATCTACATGTTTTCAGGGTGGTTATGCCTTGTGTCGGGTTATTAAGAAGAATGAGAAGGTGAACAATGGGAATGATGCGTCAATGAGATTCTCCAATGAGCCCTTCTCCATTTCTGCTGATGCTTCATCTTCTCAACCAAGTTATTTGAACAGTGAGAGTGTTTACTCAAGCCCCAATGCTTCTTCACACAATGTGGCTCCAATGGCAGACTCTAACCAAGCTTCTATAAACACCAGTTCTTCATCAGAATTTTGGGTGTCCCCTGATCTGATTCTTGATTCTTCAAAGGACTACCCGCAACTAGAAAATACTTTTACAAGGTGTGACATACCAAGTAGTACAATGACACCATGCCTCTCATTGGATCAACCTGAAATTTCACCTTGTTCATCATACTCAAATTTTAATGGGCAACTTGGATTTTCCGATGATTTCAATATGATTGGCGGCATGTCACCTTACTCAATACAAGAAGATTTTACGTACTTTCATGGAAATGATGGGGATGTTTCTTATGGAAGTTATGATCATATTAATTCAGTTGAGTACCCTGAATACTTCTGAAAACAAAGAAATATCATTCTACAACTTTTCTTGATATGCAAGGTGTCATGACTGAAGTCGCTAGCAATCGAAATTTCAAGCGCAAACATAGTTTAGATGTTAGCACCAAAGAGCGAGTTACGATTTGGAAAGTGGAAAAGAAAATCAAGGGTTATAAGGTACAATACTAGCATGATTGTGCACATTACTATTTAAAGTGTAATTGAAGTTTAAAGGAGCTAAATCTTGGGATCAAATTCTTGTGGGGGATAGTTATTTTAGATGAATTTTATTTGGTGTGATCTGATAAAGGTTGCCTAAGACAACTCTTTTTAATGTATGTCTTAGACTTAAGAGTGATAAATAATGTGTACAATACTCTTTTGATGTCATAGTATCATGTTTACTTATCTGTCTCTTATCTTGTTCACATTGAGTAATATACATGTGTGCTTTATAATATAAGCTTGTATAATGAAAGAGAGCTAGCTTC

## >AhNAC77-Arahy.CSZ51X.1

GCTATATATAATATGCTTATAAATAAGCATACACAAAGCTTCATTTGATCACCAATAAGTTGGTCCAGTTTACTAAGTTTTTATTATTTATAATTATTTTATGATGAGCAAGAAGATGAGGTTTGTTAAGAAGAACAAGAATGGAGTGAGATTATTGCCACCTGGATTTAGGTTCCAACCAACAGAAGAGGAGCTTCTATTTCAGTATTTGAAATGCAAGGTTTTCTCTTTTCAGTTGCCAGCTTCAATCATTCCTGAGATCAATGTATGCAACTATGATCCTTGGGATTTGCCAGGGAATAATAATAATTATGGGGAGGAAGAAGAGAGATACTTGTTCAGCTCAAAGGAAGTTAAGTATAGAAACGGTAACCGAATGAACAGAATAACGAAATCTGGATATTGGAAAGCAACTGGATCAGACAAAAGAATAATTTCAACATCATCCAATAATAATAATAATAATAATATTGTTGGGATAAGAAAAACTCTTGTATTCTATCATGGAAAATCTCCAAATGGCTCTAGAACTCATTGGATCATGCGTGAGTATCGACTTGTCACTACTCCTTCTAATTCATCCCAGAAGTATGTAGAAGACTTAGGGAATTGGGTTCTTTGCCGCATATTCAAGAAGAAAAGAAGCATAGAAAGTCAACATCACATGGTCAAGAACAAAATTAATAATAATGTTGTCGAGGTGGCTAATAATAATAATAATAAGCCAATATTCTTTGATTTTATGAGGCTATATGACTCGCCAATATCTTCTTCTTCATCTTCATCTTCTTCTTGTTTAAGTTCTGATTTCATAACTCAAATGTAAGAACATATGTGGTTATATTATGTATGAATTTTGATTAATTTTGTAGAATTTTCAATAAATTCTTAGAATTTAGAAGTTTATTA

## >AhNAC78-Arahy.CTTQ97.1

GTTAGATATATGCATTTAACTATGATATCTAATATGAGGATTCTTCTTGTTGAAGTTGTGAGGAGTTATAAAACCATAAAGTGGAGAACCATTTAAGAACCTCATCTTGGTCATGTTATAATTGATGAGGATTCTTCACATCATAGCTTGGTAAACTGAAATTAATTAAGCTCATCATCAAAGGGGTTTTAGGGTTCTTCATAGGAAGAGTAATTGATGGAAGAGAATCTACCTCCTGGATTCAGATTCCACCCAACAGATGAAGAGCTTATAACATATTATCTTACAAGAAAAGTCTCTGAAAATGGATTCACTTCTAAAGCTATTGCTGTTGTTGATCTTAACAAGTCTGAGCCTTGGGACCTTCCAGGTAAGGCAAGCATGGGTGAGAAGGAATGGTACTTCTTCAGTTTAAGAGATAGAAAGTATCCAACAGGACTAAGAACAAATAGGGCAACAGAATCAGGGTATTGGAAGACCACAGGCAAAGACAAAGAGATATTTCGTGGTGGGGTTTTGGTTGGAATGAAGAAAACCCTAGTCTTTTATAAGGGTAGGGCTCCAAGGGGTGAGAAAAGTAATTGGGTCATGCATGAATATAGACTTGAGAACAAGAATCCCTTTAGAACTAAGTTAATAATAGGGGTGTTCAAGATCCAGTCTGGATTTGAGGATGAATGGGTAGTGTGCAGGGTATTCCAAAAGAGCATAGCAGCGAAAAAACCGCCGCAACAAACATCATCCTCCCAACCTGAATCCCCATGTGATGACACAACCTCTTTGGTCAATGAATTTGGTGATGTTATTGAGCTTCCAAATCTAAACACCAACATTAATAATAATAATAATTCTTCATCATCATCCTCAAGTGCTTTATTCCCTAACAACATTCTTATTTCAGGACAACACATTCATCATCACCATGACCTAACCAATAATAATAACAATAATAATGTTAACACAAACATGAACTTAGCAATGAATTGGCCACCATCAAGTGATCATAATATTAATAATGTTCCATGGCCTTCAGTAGGGTTGTTGAATCCAAGTATTTCATCAATGAATTCCTTGATTCTCAAGGCATTGCAGCTTAGGAATAATTATCAACAAAGAGAAGTTGCATCCACATTTGCACCATCATCATATATTATGCCTCATCATCAAGGACTAGTAGTTCCTCATCAACAAGTTATTGGAACCAATAATAATGATGACCTAATAACAACTTCTTCAAATCTCATCAATGCTTCTTCTTCTTCATCAAAAGTTTTGGAATGTATGCCACATCAGCAACAACAACAACAACAACAGGAGCAACCATTCAATTTGGACTCCCTTTGGTAAATTGATGAATGAATTGAAAAGCATATAATAATGTATATGAAGCTCTAGCTTGCTAGATAGCTAACATCCATCTCATCTTGTAAAAGTTCTTGTGAATTATTATTATATTAATATATATGTCCATGTAACGCTCTGATTCCTACATGAAAAAATTGTAACATTTTCAAAATTGTAATAAACATAAGGATAGTTTCCTAATTAATTATTAGTAGTTATGTATGTGTCCTTTCTTTGCCCTTCGGTAATAAATAAAAAGTCAACCAACCTTAATGTTGATGATGTGAATAGTGATAATAATCAGTTGTTGTAATTAGGTGTAAAATTAGGGAAGTTTAGATCTTTGTCAATCACTCAAGAATTTTCTATAAAATAACCGTTTTTAT

## >AhNAC79-Arahy.D4BTID.1

AATTGGTGCAGAGATCCAAATAAAAGAGAAAAAAATTTAAGGACTCAATTAAAAAAATATTTTAGTACAATGACTCAATTAAAAGAAAAAATATAAAGACTTAATTAAAATTTTGCAAAACTATAAGAACCAACAAAATAATTAAAGCTAAACATATAAAATTATAATAAAAATCAATGGAAAGAAAAGAAAATAACTGACCCTCTTTGAATTTCACGGGAGGGGGTTTCCCAAAAGGCTAATCAGTAGCCCAAATTATCTTATTCTCACTAGAAAGAAAAGAAAAAGCTATGTTCATAGTTACTGAGTTGTGTTCTCGCCAGCACCTTCTCCCAAGGCTTCTTCACTCCCTCAAAGGTGAAAGTGTTTCTTGCCTTCCACATATTCCAACACAGTATCGCTACTAAAATGGTCTCTTTGCTTTCATTCTGTCTTCGTCCAAAGTCTGTCTTCGTTTCTAACCACCATTCGTGAAACTGTTCCGTGGCTGTGGAAGGGAGCTACTGATCAGGTAGCTATTGGAAAAACAGATTACATGGAAAAAGGAAAATTAATTCCGGGATTTCATTTCAATCCCACTGATGTAGAGCTTCTAAAGTATTTTTTGAAGAGAAAAGTGACAGGCAAAAAGCTACCCAATGTGATTGCTGAGATTAACGTTTATCAGTATTGTCCCTGGGATCTCCAAGGTAAGTCACATCTGAAAAGTGGGGATTTGGAGTGGTACTTCTTCTGTGCAAGAGGAAAAAATTATGGAATTGGGTCGAAGACGAACCGTGCTATAAAGAATGGGTACTGGAAAGCTACTGGTATGGACAAAGCTATTGTTCAGCATGACAAGCAAACAGTGGGGATGATGAAAACCCTTGTTTTCCACACTGGGAAACCGCCTCATGGGACCCGAACTGATTGGGTTATGCATGAGTATAGGCTTCAAGATAAAGACCTTACTGATAAAGGAATTGCTCAGGACTCTTATGTTATCTGTAAGGTGTTCCGAAAGGAGGGTCATGGCCCACGGAGCGGTGCACAATATAGGAAACCATTTAATGAAGAAGATTGGGATGATGATGATGACGATGATGATGATCATCACGTTGTTGAGGAGGGGGGAACCCCTTCAACTGCTTTGGTTGCGCCGGTTTCCATTCAGTCTATGACATTGGATGGCTCTTCTTACATGAAGGCAACCTCTGTTTCATGTGAGTCGGGACCTGTGGCTACTTCTCCTGTCCCATCAACTCCTTCTTCAGATGCAAGCATTCACACGGTTAATAATTCAACAGTGACTGATCTCTCCAAAGACGAAAAAACAGTACCTAAGGAAAATATTGCTGCCGGTGACCTCTTGAGTAAGTTTTTTGAGGGTTTGGAAGACCTCGAGTCTGAATACACTCCAAATGGAATGGGCTTGGATGACTTCGCCCCAAATGGAATAAACTATGATGACTTGGGGCACTTGGATTTGATTGATTGCAATTTCCTCTAGTTCTACTTCTAGCCGTCTCACAATCTAAAAGTTGTAGATTTTTGTTTAGATTTAACAGTTGCTTTTTCATTTTGTAAATGTCTAATTTGTTGTTTATATTACATGTTTTTTTGGTTCAGATCACAGGTTCGATCATATTTTTTTTATTTTATGATTTTTCTGTGCTACACATAAAACACACCTAAGGATAGAACTCCCATGCACGTATAATGCAAGCAAATATACAACGATCTATGTGCTTTAGATTAAACCCACCTTTAACCCAACTATATAATTGCTTTCTGCTTGGTTCATTGTACGAGGTCCATTGCCTCTTTCAGTCCTTGGAGTTGCTAATACTAATCGATTTTATTAGATAGACTATAACTTTTTTAAATAATGTGAATAATAGATTTTATAGTTTTAAAATTGACTCAATAAAGTAAAAAAATACTTCATCCTCTAATTACTTCTTAAACTTTAATT

## >AhNAC80-Arahy.D5FDJH.1

ATGGTGGATAATAGCACAGATTCATCATCAGGAGCGGGTGAACAGCATCATCACCCTCAGCTTCCTCCAGGCTTTCGATTCCACCCCACAGACGAAGAACTCGTCGTTCACTACCTTAAAAAGAAAGCTTCTTCTTCACCACTCCCTGTCGCCATCATCGCCGACGTTGATCTCTATAAGTTCGATCCATGGGAGCTCCCAAGTACCCAACTTTTCCACACCCACATGCCCAATTCGTCTATCGGTTTCCCTCCTTCTATCCCTCTTGATCCACTCAAGAAGACTTCTCTCAGGCTTGATGATTGGGTTTTGTGCCGAATATACAAGAAGAGCAACAGCAGTAGTAGCAGCCTTCCAATTCCAAGGCCAGCGTTTTTAATGGATGAAGAGAAGGATCTAATTTCCATGGAGAGCAGCATGGTGCCAACTATGTCAATGTCAAAACCAAGAAGCACTTCAACAACAGGTTGTTATGGACCCATGGCACTTGAAAACGATGACAACTTCTTCGATGGTATATTGGCAGCATCAACCGATCATCACACCATGCAAAATGGGTCTCCAGGGTCCTCATCTTCAAGCAAGAGATTCCATGGTGATCTTAATAATGGAGACAACACCTCCTTCGTTTCTCTTCTTAACCAGCTTCCTCACAACACACCCTTTCACCCAAACTCCATTCTTGGCTCCGTTGGAGACGCCGTCTTGAGGCAACAATTTCAACTTCCAGGCTTAAATTGGAACTAATTAATTAATTAATAATAATATTTTGCGTTGCTTTGGTGGGGGGTATTATAATTATATTATATTAATAGGTCTCTTGATCCATATTGTGTAGCTTAGTATATATATAGTTATTCACTTTTGAATGTAATAACCATAGCATACACAAATTAAAGAACAAGAGTTTTCATGATCATAACTATAGGCCTATATTTTGAAACTGAAGCAAGCTTACCCTAGATGAAATTTCATAAATGAATTCGGTTACACATGTTCATGGAGCATCTAATTTCTCTTCTGTGTTTTCTATATGTATATATAATCAAAGCCACACGTGAAAAACCTATGAATAGTATTATAATATATATATTATTGTTTTTGCCTTTA

## >AhNAC81-Arahy.EHBV2Z.1

ATGGAATCATCGTGTGTCCCACCTGGTTTTCGGTTCCACCCAACGGATGAAGAGCTTGTTGGTTATTATCTGAGGAAGAAAGTGGCATCTCAGAAGATAGACCTTGACGTTATCAGAGAGATCGATCTCTATCGTATTGAACCCTGGGATCTCCAAGAGAGATGTAGGATCGGGTATGAAGAGCAGAACGAGTGGTACTTCTTCAGCCACAAAGACAAGAAGTATCCGACGGGGACTCGAACGAACAGGGCCACCATGGCGGGGTTCTGGAAGGCCACCGGAAGAGACAAGGCAGTGTACGACAAGGCGAAGCTGATCGGGATGAGGAAGACTCTCGTGTTCTACAAAGGGAGAGCCCCTAACGGCCAGAAAACAGACTGGATCATGCACGAGTACAGACTTGAATCCGATGAAAACGGACCCCCTCAGGCAAGCCTTCTAGATTGTTCTATATAG

## >AhNAC82-Arahy.F01Q5M.1

TTAATGAAGAATGAAATTCTATTATGGAAAACTGTGTTCTCTTTCCTCCCTACCTAGCTCTAACAATATATATATATACACATAGATACACACCTTAGTGGCTCTCTATCTATAAATAAAAAAATAGTTAGGTTGATGAATTGATATACATTGAGATGGGGCTTCGAGACATAGGAGCATCACTTCCACCAGGGTTCAGATTCTACCCGAGTGATGAGGAATTGGTTTGCCATTATCTTTATAAGAAGATCACAAACCAGCAACTTCTTAAAGGCACTCTTATTGAGATTGATTTACACATATGCGAGCCATGGCAGCTTCCAGAGCTTCTACGATGGAATGGAGTAATGAAATACGTGGTATATGCAGAGGTGGCAAAGCTAAATGCAAATGAATGGTACTTCTTCAGCTTCAGAGACAGGAAATATGCGACGGGGTTCAGAACGAACAGAGCGACGACAAGTGGTTATTGGAAAGCAACGGGGAAGGATCGAACGGTGCAGGATCCACTTACGCAAGAGGTTGTAGGGATGCGGAAGACTCTGGTGTTCTACCGGAACAGAGCTCCCAATGGCATCAAAACTGGCTGGATCATGCATGAGTTTCGGTTGGAGACCCCACACATGCCCCCTAAGGAAGATTGGGTGTTGTGTAGAGTGTTCCACAAAAGCAAAGAAGAAAACAGTGGCAAACTTATCATGTATGATTCCATTTCCACACATCATGAATCATCAAACTCTATGGCATTGGTATCAACCCATCTTTTGAACCCCATCAACAACCATAATGCCATGAATAATTTCCTTCATCACTTCTCATCATCAAGGGATGATAGTCAAACAAATAACGCCAATAATAATAATAATAATAATAGTCCCAAGGGTTATGATGGATATGGCTTCATATGGGATCACATGGATCTTGAAGATCATAGTGTGCCCTCATCAGATTTCCAGGTCGACAATAATAATAATAATAATAATATTGCATTGCTATAAGAAGCTAAGATACTAATTAGGTGTATTATTGTATTATTTGTAAATATGCATATTGTATATGGAAAGTGTGAAGAATTCGTTGTTGTATGTATTCATTATGGTGTGTGTGTGTGGCTTCGAGTGGCCAAGTCACGTGATATGTACTATATTATTCATATATATGCTCATTGTGTAATTAGTAGTGTTAGTGTACGTATTATTGGATTAATTAGGCTCTTTATAATTTTCTTATTTTGGTGTATCCTTCTTCTCCTTAAAATTAGCCCTTTCTTTATTTTTCTCATAATGTATGTATCCCAGTTACACTTATTATATGTATAGTCAGATTCTATCTA

## >AhNAC83-Arahy.FD63AG.1

ACCCACTACCCTTTTTTTAACCTCCTTTTTTTCTTATGAATTAAGGGTTTAAATCATTAATTAACCATGCACATTTTCATATATATATATATACTCTTAATTTTTCTTAACTAAGTCAACGTAATTAATCCTTCAAGTACACCAACAATGAGATCATTTTCATTTTCTTACATTATATAATTACCCCCAGAACTAGTGCAGCTAACTATTTATTATTTAGCCCTTGATTAATTAATTGATTCAACCGTATATACCAGGACAAAGAGAAACTTGGCTTCCAAGCTTCTTTGCTATATGTAGAACCCACTTGTGTTCCCCACCTTTTTTTTTTATCTGTTATTCTCTCTCTCTCTCTCTCTCTCTCTCTCTCTCTCTCTCTGCTTTATCTCTTTCTCTCTCTCCCTCTATATTAGAGAAGAAGTTGGCGAAGTAGTAGTGCAAGGCGTTTGGAAGACCAAAAAGCACAGTTTCTCTATGAATCTTAAACAGAGAAGAATAATAATAGAAATATAGAAAAGATGGAAAACTAAGTAGATAGAGCAGACTGGTAGGGAGCTGGTGATCATCGAAAGGGCCGGGTCTGGAAAAATCAAAAGAAATGACATGGTGCAATAGATCATCAGTTGTGGAGGGGGGAATCGAAATAATCAACCACCCCAATCTCAATATTATTGCAATCCCTAGAAATAGTAATGACAATAATAATAGTGTTATTAGTGTTAATCACGCAAATACTCCTCCTAAACCAACCGAAATCCGAGCCGTTACTTGCCCCTCTTGTGGTCATCACATTCAAATACAACAAGATCAGGGTGGGGGAATTCAAGACTTGCCAGGATTGCCAGCTGGAGTGAAATTTGATCCGAATGACCAAGAGATACTGGAGCATTTGGATGCAAAAGTGCAGTCTGATGTGAGAAAGCTTCATCCTCTAATTGATGAGTTCATACCAACTCTTGAGGGCGAGAATGGAATTTGCTATACTCACCCAGAGAAGCTTCCAGGAGTAAGCAAAGATGGACAAGTGCGTCACTTCTTTCACAGGCCTTCAAAAGCATACACAACGGGAACAAGGAAGAGAAGAAAGGTTCACACCGACCAAGAAGGAAGCGAGACTAGGTGGCACAAAACTGGCAAAACCAGACCCATCTCTGTGGCTGGTTCCGTTAAGGGTTTCAAGAAGATTCTTGTTCTCTACACCAACTATGGCAGGCAGAAGAAGCCCGAGAAGACCAACTGGGTCATGCATCAGTACCATCTCGGCTCCAACGAAGAAGAGAAAGACGGCGAACTCGTCGTTTCTAAGGTTTTCTATCAAACACAACCTCGACAGTGTGCAAACAAGGATCCTTATGATGAAAGATTATTGATGACTTCACAAATTAACAGTGTCAATGACATTAGCATTCACGCACTACCCAAGAACAACAACAACAACAATGCAGGTTTTGTGGATTATTATAACCCCGGTTTCATGAATATGAATTATGAACAGATGAACGAGACTACCTCACCGCAACTGATTCCGAATATGGTGGTGCAAGGTGACAGCTCTTCTTTCATTCGGTTACCCAGGTTGGACAGAAAGTAGTATGACAATGACTGCATGCATGTTGATGTTTTTTTAATGCCATATATATGTGTTTATAATTATTATATATATGATGACTGTATTTATTATTCTATTTATATATATAGGAATATTAGAATGAAGGGGAATCAGTAGGGTTGTTAAGGAAATAATTATTTAGGCCTGAATCGGCTTCCTTAATCCCTAATATATATATGTTGACTTTTGCTGATTTGATGTTGAGACTTGAGACTTGAGAGACCATCGACCAACAAGGTCTTTCCTGTACTGTACTGTACTGTACTTTTATCTTAATCTTAGTGTACAGGTAAATTCGCAAAACTCTCTTTCATCGCTCGCTTAATCTGAAATCTCCACTCCTTTCTTCTTTCAACAATAAAATAAAATTCATTACTATTATTGTTGTTTTTTATATATGTACGAAACAAGTGACGCATATGTCTTATTTACATATATATGTTTACAATTTCGCTACGTTACTAACAGTATTTCTGCTAATTTCTGCCAACTCTTATTTATAACTGTGTTTAATGAAGATGTCTTTGTGGATGTATCTAATAAAAATGTCTTTTTTATGAATGTATTTAACAAAAGTATCTTTATAAATATATTTTCTGGATACGTCTCTTTATATATGTGTTCAAAATATAATAATTAATTATTATTGGTAATATTTTTCTATATGTTTATGCCTTTGTGAACATAATTCTTTGCACTTTATCTCACAATGCAAGCTTGATTTTTCCGATCCATTTATCTTATTCGTTTCAATTGGGAGTTTAGAGTGCGTTTATTTACATACAAGACACTAGACAGTGAGATATACACAAAAAATATTATGTATAAAAATATTAAATAATGTATTTTGTGCTACAC

## >AhNAC84-Arahy.FFKU3L.1

TGTTTTGCCATATTTATCCGTCTTGAATATAACAGCTTGATTATTTAGGAAGTGTTAGCTGACAAAAGTATTATTTAGTATCTATGTAAGTATTAAAGTAGTAAAGTGCTAGAACCTTCTTTCTCCTCTAACATGGGGTGGGAACTTTGAACTTTCTATATCACCAAAAACGACTCAAAACGGGTCTCGTTTTGAACCTTGAACCCTAAATCCCCAATTCAAACCACTCTCTCTGATACTCGCAAAGCTCGCTCTTTTAGCTGATTAACCTGCTGTTCAATCGTCATCGTGATCGTCATAATCATACATGAGTCGAATACTCGGTCCCGGTTTCCGCTTCCACCCTACGGACGACGAACTAGTTCAATACTATCTCCGCCGGAAGGTCATCGGAAAACTCAACCACCACGACCACATCGGCGTCATCAATATCTACGACTATGAGCCATGGCAACTCCCCGAATTGTCGAAGCTGAATACTAGAGATTTGGAATGGTATTTCTTCACGGTTCTGGACAAGAAGTACGAGAAGGGGGAGAAGACAAAACGCGCCACCGTCAACGGTTACTGGAAGACCACCGGCAAGGATCGTGGAATCAAGTATGGCGATCGCCAAGTAGGCATGAAGAAGACCCTCGTTTACCATGAAGGAAGGGCCCCGACTGGCAAAAGATCAAATTGGGTTATGCACGAGTACCGGATGGTCGATGAGCAATTGGCGGAAGTCGGATATCAGCTGGACGCTTTTGTGCTGTGTAGAATTTTTGAGAAGAGCGGGATGGGCCCTAAGAATGGAGAGAAGTATGGTGCTCCCTTTAGAGAGGAGGACTGGGTGGAGGATGGCGACCTGCTTGAACCGATTGCTGATGAACCTGTGGTTGAGCTGTCTGTTGACCAGAGTGATGCTTTCCTTGAAACTGATGACCTTGAGAAGAAACTTGGTACGCATGTGGTTGATGGAAGTGCTGATTTACCACCAAACCCTCCCAACTACTTTTATGGGGAGTGTAGTCACTATCCTCAGCATCAAGAAGAATTTGTTGAAGTTCCGAAACCTTTGGAAGGTACTGAAGGCCGGAATTTCGATGTAACTGGGCCATATGCTGAGGATACCTGTTTAGAAAATCATGAAATGAACCATAATGGGAATTCTTCAGGATTCATTTATGGTGATGTTAATTCAGATGAAATCATGGATTCCATTGTTGATCCTCTGATTGGTGCTGAATTATTCCTGGAAACAGATGATCTTCTGAACCCAATCGAGGGAAATTCCTCTGGGGCAGATCCTTATACAGTTGAGGGAAATCACCCCAGGGCCGATCCCTACGCAGCTGAGGGAAATCCCCCTGGGCCAGATCCCTACACAGCTGAGGGAAATTATCCTGGGACAGATCCTTATGCTGTGGATATGTTAGATGAGTATCTTGCACTTCCAGATGATGATATTCTGAGGTATATATCCTTTGATGATTCTCCTCCATCAATGGAGGGTGAAAACCCTATTCTAGAGCAGATACCACCTCTTATACAGCAGAATGTGGAGGAAGAGGCCAAGGATGTTTCAGAGGAGAAACAACAAAAGGTGGAGGGAGAAGCCACAAATATTTTCAAGACAAACAAACATGACCTTGAAGCAAATTCTAGCCGTGGAGGATCTGCTTCAGATGATGCAAATCCAATTGCAAAACGCTTCAAGAAATGGTTGGAAGACATCCCAGCTGCTCCCGCATTTGCTGCAGAGCTTCCATCCAAGAAGGATGCACTCCAGCTTCATTCTGCACCTCAGTCTTCAAATACTACTCATGTAACTGCAGGAATGGTCAGCATTACAAACATTACTGGAAGAGGCAATGACATGAATCCGATGGTGGCAAAGATTGGAGGAGGGTTCAACCATCCCATTATCTCTGCTGTTGTTTTGATACCTGTTTCTGGCTTACTTTGTGGCAAGACTCTGTTTGTGCTGACATATGGATGGGCTTTTCTGGTGACATTTTCATTTCTGTTTGCCACCGTGACTTGCAAAATTGGAACCTTCATGTATTCTGGAAAATGAGAGTTATGAGGTCTGCTGTGAGACAATGGGGTGCGGTTGGTTATCAATTGGTAGTGATGGTACTAATTGTGTGACTCAAAGATACTACTCTATTTGAAAGTTGCACAACGAATACCCATAACCGCCTGCTGATATCCAACCACCGCCCATTGGCTCCTTGTACACAAACCCCATCCAAATATATTTATCTATCAAGTGGGAACGTAACATGACTTGATTGGAGGTGTTCATTAGTAAAGCTAAAGATATATAGTCTTTTTGTCATAGATAGTCTTTTGTCTTGGTCTCTTAGATGGACTTCATCTGAAAGGTTATAGGGGTGGCTGTCCATAATAAATTGGAAGATAGTTGGACGCTTAGAGTTGGGGCATCTCGCTACCAACATGTTGGTTAATCATTCATTGGAATATCTTGTATTAGTGGGATTTTCATACCAGAATGAATGAATTTATGTTTCATAGAATTGATGGCGTGCTTATTCAGCTGTTTCATGATACTTACATTTTGTA

## >AhNAC85-Arahy.FHJ4BK.1

ATGGAAGAGCCTGTCGTAGTTAACAAAGGCAAGGAGCCGCTGGATTTGCCACCAGGTTTCGGATTCCACCCAACAGATGAAGAAATCATCACTTATTACCTCACCGAGAAGGTCATGAACAGCAGCTTCAGTGCAACTGCCATAGGTGAAGCCGATTTGAACAAATCCGAACCCTGGGATTTACCAAGTAAGAAAGAAAGCAAAGATGGGAGAGAAGGAGTGGTAAAGTAA

## >AhNAC86-Arahy.FKL2A7.1

TTTCTCTCTCTCTCTTTTCTCTCTCCCTCCTTTCTTTGCACTATAAATAACACACATACCTTGGACCACCAACACCAACACCTTCATTCTCCGTTTTCTCACATACCCTTTTTGTAATAGCAGCAGCTGGTTTGGTTTTTTTGCTTCAATGGGGGATAGCAATAATGTCAATCTTCCACCGGGGTTTCGATTTTATCCCACTGATGAAGAGCTTGTAGTCCATTTCCTTCAGAGAAAAGCAGCACTTCTACCTTGCCACCCTGATGTCATTCCTGATCTTGATCTCTACCCTTTTGATCCATGGGAACTTGATGGTAGAGCTTTGGCAGAGGGGAACCAATGGTACTACTACAGCAGAAGGACACAAAATAGGGTCACTGCCAATGGTTATTGGAATCCAATGGGAATTGAAGAGGCAGTGGTTTCAAACTCAAGCAACAGGAGAGTTGGTATCAAGAAATTTTATGTGTTCTATGTTGGAGAAGCCCCTCATGGTAACAGAACCAATTGGATCATGCAAGAGTATCGTCTTTCAGATTCTGCAGCATCCTCTAGCAGATCATCAACCAAAAGAAAATCACAACCAAAAACAGATCATAGTAAATGGGTGGTATGTAGAGTTCATGAAAGTGATGAAAATGATGATGATGGTGATGGTGATGGAACAGAACTCTCTTGTTTGGATGAAGTTTTCTTGTCATTGGATGATCTTGATGAAGTAAGCTTGCCAAATTAGATGATGCAAAACTTTTGTTAGCAGCATTATTATCCCAAATGGGGATAATTAATTAAGGCTAAATAGCTGTTTAATGCAAATTTGTTAGTACTTAATGTAGTTAAGTTTTTTTTTTTTTTTTGTATTTAGCTTTAGCAGTGTAGCTGCAGAAATTGCAATCAAAAAGTGTTTTAATTAAGTTGCCAGAGCTAGAAGATTTTCTATAGCAACCTTTGAAGTTGGGGCACTAGCTAGGTGAAATTTATGGCCAAGCAATATGCATTGTGACGTTATGCTTTCAAATATACCATTGAAACTTACTTGCTTACTTACTTTATTTTGATGCTGACACGTGGTTAATTACATGCCGAGTAATCAAAAAGTGTCCATTGTCAACCACTAATATCAGCAGTAATTATTTGATCTGTAATTAAAATTCCATATAAGATATGCAAAAGCAAGAGGGACGTCTCATTTCAATGCAAAATTCTACTAGGAATAGGAATTTCCTGGTTCAATGTAGTGTAACATGATGAAATCCAAACCAAATCCAATTGAAATTCTCACGTGCAAGGATTGTTGAGTGAATTAAGATTTGAATTCATAGAATACAAGTTGAAACAGAGAGGAAGGTTGTGGAGCCCATTCAGCACTGTAATGGAAGGTTGCAAGTGAACATAGCATATAGATCACAATAACATTGATGGTATTTGGTAGCTAGTTGTCTTGTGTCGCAATGCTAAGGACATTTCATTTTTTTATTTTTTTTTGTTATTGCATACTTGGATTCCTGCTTAGGGAAGCATCAAGTCATGTGTGATGCAAGCATTCGCATGCTTTCAAGCACCAACACTAGCACTATATTTAATAATAATAATAATAATATGCATATATTATGA

## >AhNAC87-Arahy.FU1JML.1

ATAAAGAACACTCCCTAGGGTTGTTCAGAATAATATTTGTGAATGATGTCTTCGGAAATGGGACACAGCAGATAATATTTGTAAGCAGCAATCTACTTTCAATATCAGAAAATCCAATACAATGCACCCACCGAAACGATTGAACCGTACCGGTAACAGATAAGGTCGGCCGCAGAGAATGCTTTATGAGTAGAGGGTACGTGGCGCAATCTGACAGGACAGTAACTCAAGTGGTCCCCACACCCCAACATTACCGTACTTATCTCTTTCTCCTGTCTTCCCTATAAATACCTTCCTCTGTTTTCCAGTTACTGAAAAAGAGAAGAAAAAAGAAAAAAAAAAAAAAAAGAAGAGGAGATTTACTACGGTCTGATTTTCGAGAATCTAGCTTTTTGATTATCTGAAGAAGATTAAAACAAAATGGGAATTCAAGAGAAAGACCCTCTCTCGCAATTGAGTTTACCGCCGGGTTTCCGATTTTATCCGACGGACGAGGAGCTTCTCGTTCAGTATCTGTGCCGCAAGGTTGCTGGCCACCATTTCTCCCTGGAAATCATTGGCGAAATTGATTTGTATAAGTTCGACCCTTGGGTTCTTCCAAGTAAGGCAATTTTTGGCGAGAAAGAATGGTACTTCTTTAGTCCGAGGGATAGGAAGTATCCGAATGGATCGCGACCCAATCGAGTAGCCGGGTCGGGTTACTGGAAAGCTACCGGAACCGATAAGACTATCACGACCGAAGGAAGGAAAGTTGGTATCAAGAAAGCTCTGGTTTTCTACATTGGTAAGGCACCCAAAGGCACCAAAACAAACTGGATCATGCACGAGTATCGCCTCCTAGACTCTACCCGCAAGAACGGGAGCACCAAGCTTGACGATTGGGTTCTGTGCCGGATATACAAGAAGAATTCAAGCGCACAGCAGAAGGTACCAAACGGCGTCATTTCGAGTAGCGAGCAATATGCCACGCAATACAGCAACGGATCTTCTTCAAACTCCTCTTCCTCCCACCTCGACGAGGTGCTCGAGTCCCTGCCAGAGATCGACGACCGTTGCTTCGCCTTGCCACGTGTCAACTCCTTAAGAGCGCTGCAGCAGCAGCGCCATCACCAAGAAGACACCAAGGTCGGCCTACTCCAACAGCAACAGGAACAGGGTCTCGTAGCCGGCACCGGTAGTTTCTTGGACTGGGCTTCCGGGCCGGGGATTCTGAACGATTTGGGCCAGGCCCAACAGGGGATTGTTAACTACGGAAATGACCTCTTTGTCCCTTCGGTGTGCCACGTGGATTCCAATTTGGTGCCAGCAAAGATTGAAGAGGAGGTTCAGAGCGGCGTGAAGACTCAATCCGGATTCTTTCAGCAGGGACCGAACCCCAATGACTTTACACAAGCATTCTCAAACCAGCTAGATCCTTACGGGTTTAGTAGGTACTCGGTTCAACCGGTGGGGTTCGGGTTCAGGCAATGAACCAGGGTGAGGAACTAGATGTGAAATAACTAAAAGAAAGTGTATTGAATTTTTGACTATTTGTTGAGGTGCAATTGGGGGGTGTAAATAGGGATTCTTTTGGAATATTCCAAGAAAGAAATGTCTTGCATTTAGAAAAAGGGTTGGGTTGTAATTTTCTTTCTTGGTGGTCCTCTTTTTCGGCTGGAGATAGAGTGGAGAAAAGAAGAATTCAATGCATCGAGAGGTGGAAAAAAGGGGGAAAAAATTGTGATTGCACTAAATTACTAGTCTTTGGAATTTTAATTAGTTTGAAGATGTACACAATTTTTGGCTGCACCGGCCGTGGTGTGAGCCACATGAATCACGTTTTGTACTTTTGGAAGATTTAGCATCAATCGATATACAATTGTTTACAATTCCAATGTTAGGTTGCTTTTCAAATGTTTGATGTGATCATTTTAGCTAATAACCACAACATTGGTGGCCACAAACTCATTCGTGTTCATGGGATAGGACTATGTTATAAGAGTATTTTCAATGCGTAATTTTAGTTTAGTTTGCTTTTGAGTTTTACAAAATGGTATATTATGTAAAAAATTAATTTGTGATTCAATGTGTGATTATCTATAAAATATGAATATTTTGTTAAATTATCGTGTTTGCATGTCAGATATATTTTTGGATACGATATTTACGGTTTGACATGCGTATTTGTTGTATCTAATCGTATCTTAGTAAAAAATAAAAAAATTTTAGACATATTTAAACATTTAAATATCA

## >AhNAC88-Arahy.G1V3KR.1

TTTCCATTTCCAGGTAAAACTGACCTCTAAATTCCATATTAAAATTCTTTAGCATTTGGAAGGAATATTTGTATTTCAATTCCATGGAAATTTACCTAGGCGAATGGCGAACAATGAGGCCCCTCGCACAAGAAACAGAAATTAATAAAAAAGAAAAAGGAAAAACCCCAATTAAAACCACATATATAAACAAAACCCTGGCCGCTTCTCACTCTCTTGGTAGTCCAAAGATTCAACGATTAGGAGTGCGAAGTTTCAAGTCTGTGTTCTTCTCACTTCCCTCTTTCCTTCCCTGGGTTCCACTGTGCCGATGAATTTTGTTCTCAGAAAGGGACCGTCTTCATCCATTGTGGGTTTCCAGTTCCATTTTACCTTCATGAACCGGGGAAGGCTTCGCTGTTCTCGCAATTTCTTTCGCCACTGGACTTGACGCTTCGCACCCTCGATTCTTTTAGGGTTATTTAATTCCGTTCATCATTCTCTAGTTAAATTTCTCGTCTTTTTTATTTTTTATTTTTATTTTTAATTGGGAGTACCTACAAAGTTGGTTTCTTTCTTCACTGCTGGTGAAGTGGTTCTTTGTTGTTGGATTGTTTGATTTAAGGGAAGTTGTTAGATTCTGAAGGTTTTACTATTTAAGCTTTCTTTATTCTTCTGCGATGGCCGCAATGAAGTCAATTCCAGGGTACCGGTTTCATCCAACCGATGTTGAGCTGGTTCAGTACTTTCTGAAAAGGAAGGTGATGGGGAAGAGATTCCCTTGTGATGTGATTGCTGAACTTGATATATACAAATATCCGCCGTGGGATCTACCAGATCATTCTCTGCTTAAAACTGGAGATTTAGAATGGTACTTCTTTTGCCCTCGAGGGAAGAAGTATTCGAGCGGAGGGAGGATGAATAGGGCCACAGAATGTGGGTACTGGAAGACTACTGGCAAGGATAGATCCGTCGAGAACAAGAAGCTTGTTGTGGGCATGATAAAGACTCTGGTGTTTCACAATGGTAAAGCACCCAAGGGAGATCGAACTGATTGGGTTTTGCATGAATACCGACTTCAAGATAAGGACCTTGCTGATAAGGGTGTTCAACAGGATTCTTATGTGATATGTAAAGTGTTCCAAAAGGATGGTCCTGGTCCTAGGAATGGTGCACAATACGGAAGGCCATTTAATGAGGAAGACTGGGATAAAGAGGATGAAATTGACTGTGTAGAATCTGCACCTGTTGCTGCTCTACCTGCTGCAGTTCCTATACAACCCGCTTCATGTCATAGCTCTGTTGTGAATAACGTGAATCTCTCTGTGAGTGAATGCTATGGGTTGACCTCTGTTTCGTGTTTAACAGGGCCAATGCCTTCTTGCTCAGCACATCCTTCAGCTCCAAGTAATCAAGTTGATGGTGATATTACACCAGTGCCTGGTTCCGCCATAGAAGATAACATAATGGCTCCTACTGAGAACACCACAACTGAAAAGGTTGACAATCCTCCTGACATAAACAATGCTGAAGGAACACCTTGCTTTGATCCCAATGAGATTTTTGGGGGTCTGGGTGACCTTGATGGTTTGTTCGAAATGGGTGGAATTGGACATGGTTTTTCCTGCGGCCAAAATGGTGGATATACTGTGAATGAAATGCTTTCTGCGAGTGATGGGTTGCGTTTCCCTGATCCCCTGGACTACTTGGAGTTGGGTGACCTCGACACCCCATTGTTATGGGAGACTAATGAACAAGGAAATTGGAGCCAGGACAATAAATGAGGCTTCAAAATTTGACGAAGCATCAACATGAGGTTGGTAGAAGAACCGTTGCCGTGTGTTGCTGATCTTGATTGTCTTTCCATTCTCATTTATAAACTCTACTGAAGCATATATTCTGTACAAACATTCCTTTCTGTAATAAGCTAACACATGATAGCAGTTGATAAAATCTTTAGTGTTAGTTCTATTTACTAGGTTGGATTAATCATTAGCCTCACAAATTTTCTTCGGATATTTTCTGAGTTGTGTTTGTTAGAGTGAGTTTTCAACATCTATTACGTTGAAATCTATGCATGTCTTTCGTAGTAATATCAAATGATGGAAGTTGACACATTTAACCCTAAACTTCTAGTATTCTCAATTTTGTTTTATTGGAGAACGGTTAGTTGATGAGGGAACTGTGATAGGCCTTTGCCCCTTTACTTTTCGTCGCGCTGGAAAGTACGTTATCTAAACCAGTATAGCTTGATCTTGTAAGGTCTTTTCGTGATTTATATAGAGATCGACTAAATCCCATTATTAGAAAGATTTTGTGATAACTAGTAGATTTAGAATTGAACATAAAACTAGCACTATGA

## >AhNAC89-Arahy.G3FV2L.1

CATATACAGGACTATTTATTTTTCAAACTTGTATTTTTTAGTTATATATATACTTTAATGGTTCCAAATATATATATAATTTTGTGCCGTTTTGTGCTTTATTTGCTTTAGCAGTGAAGGCTGAAAGAAAGGTTCTGCCTGCCTCCTCAAGTAGTAGAAGGAAGCAGCATACACATACAAGAGAGAAGAGACGAAGGCAGCATGGGCTTTCAACTTTAATTAGTTAGAATAAACCAAACGGCCTGGATGGTTTTCTTTTTTTCTTTTTCTTTTCTTTTCTCCTTCTTTCTTTATTATATCCTGTTTCTGTTTCTGTTTCTCTCTCTCTTTATTAATTTCTCCTTTTTCCTCTTCTCACATAATTAAACCGCGCAGCCTAGCTATTACTACATACATCCGCTTCATAAATTAGCAAAGTCTTAAAGGTGCTATTCTGTGCTATGTGTTTGAAGCCACACCCAACTTTTCCTTTATTATAAATTTTTATTCGTCACTGCAGAGAGAGAGAGAGAGAGAGAGCTCTATGTGGTTTATGCTTAATTTCTATGGTTAAAGCAGCAGCCTGGAGCCACTAGCTAGTTTCTAAATAATCGAATCGTTATCTGATGAATTAGATGATTGCAAGATGTGATCCAGTTCCAATATATATTAATCAAAGTGTTGGATTTGTGAGTTATAGATAGAAGAGAATGATGATAAGTAGTAGTAGCAGTAGTGGATTGTTGCATAGAAAAAGAATTGAAAATAACAAGGAAAGAAGTAGAATCGATGAATAAGATGGATCTGATAGATGCGAAGCTGCAAGAAGAGCATCAATTGTGTGCATCATCGTGGAAACAGTGCCCCGCTTGTGGACATAAGTTTGAAGGCAGCAGCGGGAAGAAGGGGGAGTGGGAGTGGGTAGGTCTGCCAGCAGGAGTGAAGTTCGATCCAACAGACCAAGAACTGATAGAGCATCTAGAAGCAAAAGTAGAGGCAAAGAGATCGCACCCTTTGATCGATGAGTTCATTCCCACCATCGAAGGAGAAGATGGAATCTGTTACACCCATCCCGAGAAGCTTCCAGGTGTGACGAGGGATGGGTTGAGCAGACACTTCTTTCACAGGCCATCAAGGGCGTACACCACTGGAACACGGAAGAGAAGAAAGATTCTTCAAAACGATGAGGCGGAGGCCGAGAGAGGAGAGACACGGTGGCACAAGACCGGTAAGACAAGGGCCGTTATGCTCAAGGGAAAGCAGAAGGGGTGCAAGAAGATTCTGGTGCTGTACACCAACTTCGGCAAGAACAGGAAGCCCCAGAAGACCAACTGGGTCATGCATCAGTACCACCTCGGACTCCATGAAGAGGAGAAAGACGGGGAGCTCGTCGTCTCTAAGATTTTCTACCAAACTCAGCCCAGGCAATGCAGTTGGTCTTCTTCTTCTTCTTCTTCTTCAATTACTGCTGCTGCTGCACCCTCTGTCAAAACTAATAATGACACGTGTCCCGTTCTTGGATTCCCTCCTATGGAACATTTCAGCAGCTTCATCCCTCTCAGAAAACCCCTCCATAATGAGGAAGTTGGAATAGGAGGGGAAACTTGCACACCAGCGTCACATATTCCTTCATCAAATCCTCTTGGAGTCTTCCATCACAACACTTCCATCATCCTTGACGACCTTATCTCCGCTAGATTCATGACTCCTCCTCCTCCTCCTCATTTCCACCAGCAGCATGATAATAAAGTAGTAGGAGGAACCTCTGCTTCTGGTTTAGAGGAACTCATCATGGGCTGCACTTCAACTTCAACCACTCATAATATCACCAAAGAGGCATCAATGACAAACACAAACCCACAAGAAGCTGAGTGGTTGAAGTACTCTTCTTATTGGGCTGACCCTCAGCCTCAGCCTCAGCCTCATCTTCATGGGTAATAATAACAGACCCCATTTGGCAAAGTAATCATCCACACTTACGCTTTCCTCTTTTTTTTTTCTTTTTTTTTTTTCCATTAATGCAAATATATATATAAAAGGGCGTGGCTTTTCGAGTCTCAACGGAAATAACTGCTGCTTGTGTGCACTGTAACGGCACATACATCAAGTTGGAAACAAACAACAAAGGAGGGAAGAACAAATTGAATCAAATTCAAGAAAATGAAAATAACCGCAAGAAAATTAAAGCTGCTGTTCTACCTCCTCATCACACAAAGAAACAGTTTGATAATGCATCAAAATCATCACTATTACCAATATAGTATCTTTTTTTTCTCTCATTATTCATATTATTTTATTATTTTAATTAAGAGGTTTGTATAATTTCGCTTTTGCTGACTTGCTTCTTTTACACATTACATGAAATATCAAATATACCGATGAGTTTCTGCAAACTGCTTTGCCCTAAT

## >AhNAC90-Arahy.G3YZJ0.1

CAAAACAGTATTGAAAGCTATTGATTCTATAGTCTCTTGTGTGATCCTTATTTTGGTCTTTCCCAAAATCATTATATATTAATACTGGTCTCAAATGGTGGAATTGAGACACATCCATTAAAGGAGAAATATAAATATATCCTTAATGAAAGGGAGGCAGAAGCTTAGGAAGCATATATAAACATTCTTGGGAGGCACATAAATTTCCTTGGACTAAACAAATTTATTTGAGAAATTGAATTGGAGATGGAGGAAAGGAATATTGAGATGGAGAATAAGATTGAAGATGAGATGATGCCAGGTTTCAGATTTCACCCAACAGATGAAGAGATTGTTGGTTTTTATCTAAAAAGAAAAATTCAGCAAAAATCTCTTCCTATTGAATTGATCAAGCAAGTTGATATCTATAAGTATGAGCCATGGGACCTTCCAAGCCTTCATGAAGATCCAAATCATCATCATTACAATAATAATAGTAGTGGGTTCTCATATGATGATGATTCAGGTGTAATTACAACAATTGCTGGGTTCCCATTCAATTTGCCTCCAAATGATGATGATGCTGCTGCTTGGAATAATAATAATAATAAGCCTAATACTACTCTGCCATGGGATTACTCATCAGACATGTCCACTACCTATTCCACTAATAAATCTTACACT

## >AhNAC91-Arahy.GDX8G8.1

TATTGCACTGATCCACACACACATTACACATCTCCCTCTCTTTCTCTCTCCTAAAAGTTAAAAGCCACATACATACATAGATACATAGATACAGGTCCGTGCCCTTGAAGCTAAGAAACTGTGCAAACTAAGCCTTCTCTTTGCTTGTTTGTCCCCTCTTTCCTGTGAGCTTCTTTGTCTTCTTTCCTTTGTCCTCAAACGCTCATTTCCCTTGTTGCTTCTCACTAAAGGTAATTAATTAAAAGCAGAAGAATAATCAGAATGATGGCAGGTAGTGGACAACTAACAGTTCCACCAGGGTTCCGGTTCCATCCAACTGATGAGGAGCTTCTCTACTATTACCTAAGGAAGAAAGTTTCTTATGAAGCCATTGACCTTGATGTCATTAGAGAGGTTGATCTCAACAAACTTGAACCTTGGGACCTCAAAGATAAATGCAGAATAGGATCAGGGCCTCAGAACGAGTGGTATTTCTTCAGTCACAAAGACAAGAAGTACCCAACAGGAACAAGGACCAATAGGGCAACCACTGCTGGTTTCTGGAAAGCCACTGGGAGGGACAAGGCCATATACCATACTAGCAATTCCAAGAGGATTGGGATGAGAAAAACCCTAGTTTTCTACACCGGCCGTGCGCCCCACGGCCAGAAGACTGACTGGATCATGCATGAGTACCGCCTCGACGAAGACGACGCCGAGGTTCAGGAGGATGGGTGGGTCGTGTGCAGGGTTTTCAAGAAGAAAAACCAAAGCAGAGGGTTTCGACAAGAAATTGAAGAAGAGGAACATCATCACTTAGCAGCAGCACATCAACACATGAGAGGAGTAGCAAGCCAACAAGTTCTGGACCCAAAACACCACCACCACTTGCAACATCATCAAGGACTCTATGATAATGAAAATAATAATAATTACACCAATAATTTTGATGGATCCATGCATCTTCCACAGTTGTTCAGTCCAGAATCTTCCGTGGCTACCGTGGCGGCGCACACTTCCATGAATGCCATGGACATTCTTGAATGCTCCCAGAACCTTCTAAGGCTCACAACAACAAGTGGATGTGGACTCAATCTCATGCAACAACAACATGGAGAGAGGTTCAATGGTGATTGGTCTTTCTTGGATAAGCTTCTTGCTTCACACCATGGCAGCACCATGGATCATCATCAGCATCATCATCATCATAGCAAATGTAACAATAATCTTCATCATCAGCATTCTGCAATTGCTATTGGAACTACTTCATCTCAGAAATTCCCATTTCACCACCTTGGTTGTGACAACCATGATATCATGAAGTTTTCCAAGTAGGGTTATTATGAAAAATAATAATATAATAATTATTATTATTATTATTATTATCATAAAAGATGATGAGGTGAGAATCAGAAACTTCCTTTCTAGTATATGTATTTCAGAGTATCCTCCTGTTTGTTAGGTACTCATTGGAAAAATTCTCCCACTCATTAATATTTATTAGCATTTCTTTATTTTGAGTATCAATTGTGTATAGGTATGTATCTTTGTTATATTTTCAAGCTGCTGTTAAATAATATGATACTATTCATGAATGCTCATTATTCATGTTGTTGGATACCATGGCGATCTACTTTC

## >AhNAC92-Arahy.GPRR9Y.1

GTGATACATAGATGATGATGATGATGATGCTATCAATGGAAGAGCTAGCGTGTGAGCTGAGTGATCATGAAAAGAGAAACGCTCAAGGTTTGCCACCGGGTTTCAGGTTTCACCCAACTGATCAAGAACTCATTACCTTCTATTTGGCTTCCAAGGTCTTCAATAATACAAATGCTACTACTGCTACTACTACTACTACTCATGTCAACTTTGTGGAGGTTGATCTCAATCGATGCGAGCCATGGGAACTTCCAGAAGTGGCAAAGATGGGGGAGAGAGAGTGGTATCTGTACAGTGTGAGAGACAGAAAATACCCAACGGGCCTCAGAACTAACAGAGCAACCGCTGCTGGGTACTGGAAGGCTACCGGCAAGGACAAGCAAGTCTACGGCGGCGGTGGCCTTGTTGGGATGAAGAAGACGTTGGTGTTCTACAAAGGGAGGGCCCCCCGCGGTCAGAAGACTAAATGGGTAATGCATGAGTTCCGGTTGGACCCTCACAGCTCTCCTTCCCTCTCTAAGGATGAGTGGGTAATATGCAGAATATTTCATAAAAGTGGGGAAAAGAGAGCTCCTACTACTACTCCTCCTCCTCTGCTACTTCATCATCAACTGCAGCATCAGCAACAACACGATCCATTATTATTATTCCAAACCCCATCATCCCTGTTCAATGACCATATCTCCCACTCTCATAATCATAATCAAAACCTCCTCTCGCCATTGCTTCATCCTTTCCCAATCCCTGAAGAAACCACTAAAACCAGATCATCAACAATTAACAGCAACCATTACCCTCCACCACCACCTTCCTCCCAACACTCGCTTAAGCTCAACAAGTCTACTAAATTAACAAAAACAGTGCCTCCTTCTCCATCATTCTTCCAATACCAACAGCTTCTAGAAGATTATCCCAACTTATTGCATTGGATCGACAGTGGTAATAACAATAATAATAACTGCAACGCTAATAATACTGCTAGTAGTGTTGAGATAATGGATGCTGCTGCTGCTGGCTTGATAGCATTCTCATCAGGAGGACCTTCACCTACTCCTAATAATAATAATAATAATAATAATAACAATGCTGAAATAATGATGATGTCTTCTTCTTCGGCTTCTATGCTGCACATACTCGACGATGCTCCTCTTGGGATTCAATCTTGGCCTCATCATCATCATCATCACCTTCTGTAAAATAATGTATGTGCATGCATGCATATTTACCTTAATTAGTTAATTAATTAATTATTAACTTATTGTATATGCGTACC

## >AhNAC93-Arahy.GU1UJS.1

GAGAGAGCATCAATGGATATGGAATCATGTGTGCCTCCAGGATTTAGATTTCACCCAACAGAAGAAGAACTTGTGGGGTATTACCTCAAGAGGAAAATTAACTCCCTCAAAATTGATCTAGATGTTATAGTTGAGATCGATCTCTACAAAATGGAACCATGGGACATACAAGAAATAATAATGTGGACCTTGCTAGAAGAAGGATGGGTTGTATGTAGAGCATTTCGAAAACCAAGTCCAAGTCATCAAAGGCAATTAGGTTATGATCCATGGTGTAGTAATCATCATCATCAACCACATTATTTCAGAGATCAGAGTAGCTATGGTGGAAGGCCCTTATCAATCACAGATCTTCTAACTTCAGAAACTCATCATCATCATCATCTGCTGAATCACCCTACTGAAGGTACAAATTTTAGTCATCCCTTCGGTTCAGATCATCATCATCAACAAGAACAACAAGAGTTTGTAATATCAAATAATCATCAACAACTCATTGAGCTTCCACAGCTAGATAGCCCTACTAGTGCTTCTCTCTCAGCACCACCAACAACAAGTTTTGCAGTCAAAGAATCATCTTCCATTAATAATAACAATGAAGAGTATTGCAGTGATGAGAGGAACAACAACAACAATATTGATTGGAAAAGCTTGGATAACTTGTTTGCTGATACTTCTAATTACTTCTCAAATCCAAACATGTCCCAATTCATGACCATCAATCATCATCTAGGTTGTTTCCCTGGTTCATAA

## >AhNAC94-Arahy.H91V8V.1

GCTGCGCGCTAATGGCATTGTGGCAATTATTTCCAAAACCAAGGTCCAAAGCAGCACACAATAGTATAAACGAGGATTTTTTTTTTGGGTAAATAAAACGAGGAATTTTTATTTTAACGCGCTTCACTTCTCACTTCTCAGTTCTTCCTCAAACGAAAATCCAAATAGTAAAAATACTGCACTAACCCTTCTCTCATCTCTGATATTTTTCTTTATTATATATATATATATATATATATATATTTTCTCTTTCGATCACTGTCTTCTAGAAAAAAAAAATTACTAAAATTCTCAGAATCCTCTCTGATTCCAGTTCAGCTTCACTTAATAGAAACCAATCAAAAGATCATCGTGAAATTTTTTTCTTGTTACTTTTTTTCTTCGTTTGGTTGTCACGAACGGTGCCGTTTTATATATACATAAAATAGAGACACCGTCGTTTTGACACAAACGGTGGTTTTGTTATGGGTGCGGTGGAGGTGTTTCAACAGCAGCCGCTGGTGGTGGACGCTGCTCCGGTTTTGTCGCTGAACTCGCTGCCGTTGGGGTTCCGTTTCCGACCGACAGACGAGGAGCTCGTTGACTTTTACTTGCGGCAGAAGATCAACGGCAATGGCGATGAGGTTTGGGTCATTCGAGAAATCGATGTTTGCAAATGGGAACCTTGGGATTTGCCTGATTTGTCAGTGGTACGGAACAAGGATCCGGAGTGGTTCTTCTTCTGTCCACAGGACAGGAAGTATCCAAATGGACACCGGCTGAACCGAGCAACAACTCATGGATATTGGAAGGCGACTGGCAAGGATCGGAAGATTAAGTCCGGCTCTACCGTGATTGGAATGAAGAAGACTCTTGTATTCTACACTGGCCGTGCTCCCAAAGGGAAGAGGACCAATTGGGTGATGCATGAGTACAGGCCTACCCTGCAGGAGCTTGATGGTACCAATCCTGGACAGAATCCATATGTCCTTTGTCGATTATTTAAGAAACAAGATGAGAGTCTTGAAGGTTCAAACGGTGAAGAAATGGAGCGTACTACTTCAACTAATTTAACTGCAAATTACTCTCCAGAAGAAATACAATCAGATCCAGCTGTTAAATCGGTTTCTTCTTCACAGGCTACAGAAGATGACAAGAAACTAGCAGTTATCCCTTTGACCCCTGAAGAAGCAATTTCCAATGTTATAACCCCGGTCGGTTGCCAAAACGATGGATGTGATGCTTATGATGCACAAAATCAAATCGCAGCAGGAGATCCATCTAAGGAGGAGGACTTACAAGTGAACATGGACATATTTTATGACCCGAGTGAGCTATTTGACGATAAATTATTCTCCCCACTCCACAAGCATATTCCAGAAGAACTTTTTCATCAATCAAACAATGAAGCCAATGGACATTTTGGGCTGCAACATCAGTGTGGAACAAATGAGATCAGTATTTCTGACTTCTTTGACTCTGTTATTAATTGGGATGAGATCTCCGGTGACAATTCCAGCGGCCAAACGCCAAACTCTGCTTGGTTTGATGTACAGCACAATGAATCATGGGGAAACTCAAATGTGGATATGGTCCATGCCAGGGTGAGTGACAGTGTTAACCCTGTTTATCTTTTGCCCCTACAAGTAGGGGGTGCAGATTATCCAGGGGATGCAACCGAGGGAAAGCTCCCTTTGTTGAAAACTAGAGAATTCAATCCCAACACCTCTTATGACAATGCGCTCAGCAACAACATGGGATTATTTCATAACCATTCCCAGATGGCTTTTTCATCTGATGTTAATATGCTCCAAGGTTACCATGCAACCAACAATTATGAGCAACCGACAAACTTCAATATGGCTATGGCTAATAGTGACAACACTGGAATTAGGATAAGGTCTCGGCCACCAGGTTATGAAGGGCCAAACGTAAACTCCAATATGCAACCACAAGGTACTGCACCTAGGAGAATACGGTTGGCACGATCTCTTGCACCTCAACACATGTCCAATGAGGCGGCAAAAGATTCGAGTTACGAGTCAAAAGATCAAAATTCACAACTAACCACTGCCAGGGAGATGGAAACTTCCAAAGACCTTGCTGCTGGTGAGAGTGTTACTGTTACTAGTGATGTGGAGGAACAGGAGACATCACCAGTTGAAAATAAGGAATTCGAAGACTTCAACACAGTCCAGCAGAGCACATCATCAGCTTCCTCCAATCTTTCCACGTGCTCTTCTGATTCTGAAGTTTCTTATGAGGCAGAAAAAGAATCTGGTTGGACATCAGAAGACCATAGTCCAAAACCAGCTGCCGCGGGGGCCAGTAAAGCTTCCGAAGACCAAGTTCCCAGCGAGTGCGTCAATGATATCACTGATGATGTGGATGAACCCAGGATACCAAACGCTTATACTCTAGAGGTCTCAAAGGAGGAATCCTTCTCGGACTCTCAGTCGAAAGACTCTCTATTGCGTAGAAAGGTGTGTTACCCATCGAAGTCTTCCTCAAATCTAGCCAAGTGGTATTCGGTTATTGCAGTCTCAGCCACTTTGGTGGTGTTACTAGCATTCCTTGTTAATACATGGGGTTATGGATATTACCTTAAAGTTTAACTGCATAGTAGGATATGTATCATTGCATAGAGTATATTTTGCCTCCATTGCGTTTTTGGCCTTAGTTAAGGAGGCATATATGTAAGGGTTGACTTTGTAGCTTTCTTTGAATATAATCTGTATGTGCATATGTTATATATAGCAGAATATGCCTAATCTGCTACTAGCTTGTACTATATGTGGAGTTGTAAACTCATGTTTATAAGTTAGATTCAGAATTTCAGGGTTTCCAATTTTTTGGAATTTAATTTTGATTGCCCGTGCGTGACCACCATTGGAGAATATGCCTGCCTAGCCAAGAGTGTATTTTTAGTAAAGAAAGGTTTAGGCAGATTCGGCTGTTTGATTCATTCATTTATTTATTCTATTTTTGTTGTTGGTTTAAAGGTAAGCTTTTAGTTAAGAATGCTAAAAGATATAGAACATTGAAAATCTGGAGAAAAAAAGATAATGTATATATAAGAAATCAATCATTTTACATAGAATATATTTTAATATATTAAAATATAAAATATATATTAAAAATAAATTAAATAATATATTTAATTATTTATGCTAATTTTGTGACTGATTTTTTATATTTACGTA

## >AhNAC95-Arahy.HHSK2A.1

ATGGCTTTAACAAATTTGCCTTCAGGTGCAAGCAAGAAGTTCAAACCTACAGATGAAGAACTCATTCAAGATTTTCTCCGTAACAAAATTAATGAGAGGCCTCTACCAAACTATGGAACCATTCTTGAAGGTGAATTGTTTGGTACGGAGAAGAATCTATCGGAAATTTGGAAAGAACACGTTGAAAATTCTTATGACGGGAAGGACCTCTACTTCTTCACTACTCTGAAGAGGAAGTTCTCGAAGCTTGAGGATGGTTCGCACCATCGGGTTGGGTTCTTGGGAAGCTGA

## >AhNAC96-Arahy.HJ0R1G.1

ATGGCTTCAACAAAGTTGCCTTCAGGTGCAAGCAAGAAGTTCAAGCCTACAGATGAAGAACTCATTCAAGATTTTCTCCTTAACAAAATCAATGGGAGGCCTCTACCAAACAATGGAACCATTCTTGAAGGTGAAATGTTTGGTACGGAGAAGAATCCATGGGAAATTTGGGAAGAAAACGTTGAAAATTCTTATGACGGGAAGGACCTCTATTTCTTCACTACTCTAAAAAGGAAGTTCTCAACTAACAGCTTGCGGATGGTTCGCACCATTGGGTTGGGTTCTTGGGAAGGTGAAGACATCGGAAAAGAGATTATGGCCAATAAAACTAACCAGCGCATTGGAATGAGGAAACGATATCGCTTTGAGAAGAGTGGTACTAGCCATGATGGTGGATGGATCTTGCATCAATATAGCATTGATTCTTCTTTGTTACCAAATCCATCCAATATGAATAATTATGTTTTATGCAGATTTAGAAAGAATAACAGTAAACCTCGTCAAAAGAAAAGGAAAATTGTGGCTCCTGAAACTGTGGCTACTGTAATACCGTTTATAGCTTATTGA

## >AhNAC97-Arahy.I1Q9WS.1

ATAAAAAAAATTGGGATGCAGAAGTTAAGGAAACAGTGTGAGAGGTGTGAGAGTGGGGATGAAGATAAAAGCGGAAGCACGGACTAGTTGCGATCGAAGTACTTGCTAATGACACGTTAAAAGAAAAATAATGGCATCAAACATGCTTGGTTCCCTCGCCGACCCTATTCCACTCAAAGACCAAACGCCTAAATTGCCATACTAAATTGTTGTCACTCACCAACAAACAAAGAACAGAACAACATAACTCTCTCTAACACCAAACCCTTTTACTTTCACCTTAGAATCATGGTGGATAGGGATTCAAGTGAAGCACACATGTCAATAGCCGCTTCTTCCATGTTCCCTGGCTTCAAGTTTTGTCCCACTGACGGTGAATTAATCTCTTATTACCTCAGAAAAAAATTGGACGGTGACGAGGACAGTGTTCAGATCATTTCGGAGCTTGAGCTTTGCACCTTTGAGCCTTGGGATTTGCCTGAAAAATCTTTCATTAAATCAAACGATGAGTGGTTTTTTTTCTCGCGACGGGGGAGAAAGTATCCGAATAGTTCACAAAACAAAAGGGCAACTAAAAGTGGGTATTGGAAGGTCACAGGAAAGGAGCGACAGATAGAGTCCGGTCAGAATGTGATTGGTACCAGACGCACTTTGGTATTCCATGTCGGTCGAGTTCCTAAAGGCGAAAGAACTGAATGGATTATTCATGAGTACTGCATCAATGACAAATTTCAGGATTCTTTGGTGGTTTGTCGGCTCAAGAAGAACACAAAATTTCATACAAATGATGATTCTAACAAAGCTTCACGCGAGAGTGGTTGTGGAGTCTCAGAAGGGGGTTACAGTTCAAAGGAGCACTTTTCCAAGAGGAGTAACAATAGTAATAGTTCTCCTTCTATTACTGTCCAAATTGAATCCAGTAATAGAGTTGCTAATGAAGTCAATCCCAAAGCTTCTTCCAATGAATCCAGTGATAGAGTTGCCAATGAAGCCAATCCCAAAGCTTCTTCCAATCACTCTAAGGTGGATGAAGTGGATTATTATGCAGAGATCAACTTAGATGATATCATCAACTTAGATGAACCAGCACTCTGACTGCCATAGCCCATAGCCACCACAAGGGACAGCAAATAGAGATCAACAATCATCAAACAAAATCAAAGGTTTCAACGGAACTTCTTGACCAATGCTCCAACCAATCATCAAACAAAATTAACACTTTCCTGCCATACCGTTTAATGCTCTTCACTTTCTTTGTTTTCACTTTACTAGCTCTTAAGCTTTATCCTTATCGGGAGGTCTCAAACTGCTGTATAATATTCTCCAGACCTCTCTCGAGTTTAATTATGATTATTAGTTAATTAGTGTTAGTTACATTGTATATAAAGTTACAAAGAGATATGCACTCTTCATAGCGAAATAGTAAGGACAAGGATTATATTATTCATTACCTTACCAATGTTGAATGTATGGACATCATCACTAATATTAACTAACTAAAAATATACGATTAAACAAGCTAAAGCAATTGACTAGCACCTTCCTGATCAAACATGAAGATGTAGGCAATGACATGTGGCTGATGCTGCTAGGAGGATATGGCCGTATGGTCTGGTCATGGGTATGTCG

## >AhNAC98-Arahy.I4FPAQ.1

ATGGCTTCAACAAAGTTGCCTTCAGGTGCAAGCAAGAAGTTCAAGCCTACCGATGAAGAACTCATTCAAGATTTTCTCCTTAACAAAATCAATGGGAGGCCTCTACCAAACAATGGAACCATTCTTGAAGGTGAATTGTTTGGTACGGAGAAGAATCCATGGAAAATTTGGGAAGAAAACGTTGAAAATTCTTATGACGGGAAGGACCTCTATTTCTTCACTACTCTGAAAAGGAAGTTCTCAACTAACAGCTTGCGGATGGTTCGCACCATTGGGTTGGGTTTTTGGGAAGGTGAAGACATCGGAAAAGAGATTATGGCCAATAAAACTAACCAGCGCATTGGAATGAGGAAACGATATCGCTTTGAGAAGAGTGGTACTAGCCATGATGGTGGATGGATCTTGCATCAATATAGCATTGATTCTTCTTTGTTACCAAATCCATCCAATATGAATAATTATGTTTTATGCAGATTTAGAAAGAATAACAGTAAACCTCGTCAAAAGAAAAGGAAAATTGTGGCTCCTGAAACTGTGGCTACTGTAATACCGTTTATAGCTTATTGA

## >AhNAC99-Arahy.ILS8DP.1

AGCTGACAAAAGTATTATTTAGTATCTACGTAAGTATTAACTAAAGTAGTAAAGTGCTAGAACCTTCTTTCGCCTCTAACATGGGGTGGGAACTTTGAACTTTCTATATCACCAAAAACGACTCAAAACGGGTCTCGTTTTGAACCTTGAACCCTAAATCCCCAATTCAAACCACTCTCTCTGTGATACTCGCAAAGCTCGCTCTTTTAGCTGATTAACCTGCTGTCATCGTCATCGTCATCGTCATAATCATACATGAGTCGAATACTCGGTCCCGGTTTCCGCTTCCACCCTACGGACGACGAACTAGTTCAATACTATCTCCGCCGGAAGGTCATCGGAAAACTCAACCACCACGACCACATCGGCGTCATCAATATCTACGACTATGAGCCATGGCAACTCCCCGAATTGTCGAAGCTGAATACTAGGGATTTGGAATGGTATTTCTTCACGGTTCTGGACAAGAAGTACGAGAAGGGGGAGAAGACAAAACGCGCCACCGTCAACGGTTACTGGAAGACCACCGGCAAGGATCGTGAAATCAAGTATGGCGATCGCCAAGTAGGCATGAAGAAGACCCTCGTTTACCATGAAGGAAGGGCCCCGACTGGCAAAAGATCAAATTGGGTTATGCACGAGTACCGGATGGTCGATGAGCAATTGGCGGAAGTCGGATATCAGCTGGACGCTTTTGTGCTGTGTAGAATTTTTGAGAAGAGCGGGATGGGCCCTAAGAATGGAGAGAAGTATGGTGCTCCCTTTAGAGAGGAGGACTGGGTGGAGGATGGCGACCTGCTTGAACCGATTGCTGATGAACCTGTGGTTGAGCTGTCTGTTGACCAGAGTGATGCTTTCCTTGAAACTGATGACCTTGAGAAGAAACTTGGTACGCATGTGGTCGATGGAAGTGCTGATTTACCACCAAACCCTCCCAACTACTTTTATGGGGAGTGTAGTCACTATCCTCAGCATCAAGAAGAATTTGTTGAAGTTCCGAAACCTTTGGAAGGCACTGAAGGCCGGAATTTCGATGTAACTGGGCCATATGCTGAGGATACCTGTTTAGAAAATCATGAAATGAACCATAATGGGAATTCTTCAGAATTCATTTATGGTGACGTTAATTCAGATGAATTCATGGATTCCATTGTTGATCCTCTGATTGGTGCTGAATTATTCCTGGAAACAGATGATCTTCTGAACCCAATCGAGGGAAATTCCTCTGGGGCAGATCCTTATACAGTTGAGGGAAATCACCCTAGGGCCGATCCCTACGCAACTGAGGGAAATCCCCCTGGGCCAGATCCCTACACAGCTGAGGGAAATTATCCTGGGACAGATCCTTATGCTGTGGATATGTTAGATGAGTATCTTGCACTTCCAGATGATGATATTCTGAGATATATATCTTTTGATGATTCTCCTCCATCAATGGAGGGTGAACACCCTATTCTAGAGCAGATACCACCTCTTATCCAGCAGAATGTGGAGGAAGAGGCCAAGGATGTTTCAGAGGAGAAACAACAAAAGGTGGAGGGAGAAGCTGCAAATATTTTCAAGACAAACAAACATGACCTTGAAGCAAATTCTAGCCGTGGAGGAGCTGCTTCAGATGATGCAAATCCAATTGCAAAACGCTTCAAGAAATGGTTGGAAGACATCCCAGCTGCTCCCGCATTTGCTGCAGAGCTTCCATCCAAGAAGGATGCACTCCGGCTTCATTCTGCACCTCAGTCTTCAAATACTACTCATGTAACTGCAGGAATGGTCAGCATTACAAACATTACTGCAAGAGGCAATCACATGAATCCGATGGTGGAAAAGATTGGAGGAGGGTTCAACCATCCCATTATCTCTGCTGTTGTTTTGATACCTGTTTCTGGCTTACTTTGTGGCAAGACTCTGTTTGTGCTGACATATGGATGGGCTTTTCTGGTGACGTTTTCATTTCTGTTTGCCACCGTGACTTGCAAAATTGGAACCTTCATGTATTCTGGAAAATGAGAGTTATGGGGTCTGCTGTGAGACAATGGGGTGCGGTTGGTTATCAATTGGTAGTGATGGTACTCATTGTGACTCAAAGATACTACTCTATTTGAAAGTTGCACAACGAATACCCATAACGGCCTGCTGATATCCAGCCACCGCCCATTGGCTCCTTGTACACAAACCCCATCCAAATATATTTATCTATCAAGTGGGGACGTAACATGACTTGATTGGAGGTGTTCATTAGTAAAGCTAAAGATATATAGTCTTTTTGTCATAGATAGTCTTTTGTCTTGGTCGCTTAGATGGACTTCATCTGAAAGGTTATAGGGGTGGCTGTCCATAATAAATTGGAAGATAGTTGGACGCTTAGAGTTGGGGCATCTCGCTACCAAGATGTTGGTTAATCATTCATTAGAATATCTTGTATTAGTGGGATTTTCAGACCAGAATGAATGAATATATGTTTCATAGAATTGATGGCGTGCTTATTCAGCTGTTTCATGATACTTACATTTTGTA

## >AhNAC100-Arahy.JB9PK4.1

AATAATTCAATCAAACCTTGGGAGTTTGCAACATCTTTCTCTCAGACCTATTCTTTTCTCTCTTCTCCCCTTCTTCCTAGCAAGCAGGCTCTCACTATATATTAATTTAATTTGTTGAAATTAAATAGAAAAAAGAATAATGAGCAACATAAGCTTGGTAGAGGCAAGGCTTCCACCAGGGTTCAGATTTCATCCAAAAGATGAAGAGCTTGTGTGTGATTACTTGATGAAGAAGTTCACGCACAATGAATCCCTTCTCATGATTGATGTCGACCTCAACAAGTGTGAGCCATGGGATATTCCTGAAACAGCATGTGTGGGAGGGAAGGAGTGGTACTTCTACACACAGAGAGACAGAAAGTATGCAACGGGGCTGCGTACAAACAGAGCAACGGCATCAGGATATTGGAAGGCCACTGGCAAGGACAGGCCTATCCTTAGGAAGGGCAGCCTTGTTGGTATGCGAAAGACTCTTGTCTTCTATCAAGGTCGGGCTCCCAAAGGCCGTAAGACTGAGTGGGTCATGCATGAGTTTCGCATTGAACCTCCTCTTCCTCCCCCCAACACTTCTTCTAAGGAAGATTGGGTGTTGTGTAGGGTGTTTTACAAGAACAGAGAAGTTGGTGGCAAACCTAATAGCATGGGAAGCTGCTATGATGACACAGGCTCTTCATCTCTTCCAGCATTAATGGATTCTTTCATCAGCTTTGACCAACAACAACAACCTCAAACCCATCTTCATGCTGATGAGTATGAGCAAGTGCCCTGCTTCTCCATTTTCTCTCACACCCAAACAAGCCCTATTTTCAACCACATAATGGAGCCTAAGTTATTCCCTACCAACAACAACAATAATAATGCAACTTTATATGGTGGAGGAGGAACTACTACAACATCCAATTTGGGTTCTTGCTTAGACCCTTTTTCATGTGATAGGAAAGTATTGAAAGCTGTTTTGAGTCAGCTCACAAATATGGAAAGAAACATACCTAATAATAATAACAACAATACAAATAGTATAAAAGGGTCACCAAGTTTAGGAGAAGGTAGTTCTGAGAGTTACTTATCTGAGGTTGGCATGCCCAACTTGTGGAACAATTATTGATGTGGTAGTCCTTATAGGCTTTGTAATTTGTTTATATACTTTCCCCCAAAATTAATTTATCCATGTTCTTCATATGCCACAAGGAAGTGGAAAAAATATATATGAAGAATCAATAGAATTTTGGAGGAGGGGGAAATTCTCATTGGAATTTTAAGTCATTGTGGTGGGTATCTAGGCTTCACCCATTGTGTGCATGTCCCAATATATTAAGAAAACGGGACTTGTAATTGAAACTCAAAAATATTTGTAGTTAATTTTAGTTAGTAGATGAATTGTTTCTTC

## >AhNAC101-Arahy.JBNT97.1

GAACTACCAGGAAGCATAGGATCATTACCACACAAGAACACAACATATTAAACCCAAGCTTGAATACAAAGATAAAAAGAATCTTCCATTCCATTTGATCGAAAAAGAGATAGTGGGACAACCCCAATTTGCTTCTTCATTATATTCTCTTTTTGTCCTCTTTGTTAAAAGCCCAAGCAACCCCAACATTCCACCCTCCTCTTCCCTCACCTATTCTCTCGCTCTCTCTCATTTTTCCACATTAAGGTCTCTACTCTTTTAATGGAAGGGAGAGCATTATTATTGCATCATTGGAGATCATCGCTTGGATTATTGAGGTTTCTGATAGTTTGTTAAGTGGGGTATTAGAGATCATATAATATTTAGATATTATAATGGCACCAGTTTCATTGCCTCCTGGTTTTAGGTTCCACCCTACAGACGAAGAACTGGTTGCTTATTACCTTAAAAGGAAGATCAATGGCCGTAAAATTGATCTCGAGATCATTCCTGAAGTTGATCTATACAAGTGCGAACCGTGGGACTTGCCAGGGAGGTCGTTGTTGCCGGGTAAGGATTTGGAGTGGTACTTCTTTAGCCCTCGGGACAGGAAGTATCCAAATGGGTCAAGAACCAACAGAGCAACGAAATGCGGGTACTGGAAGGCCACTGGAAAGGACAGGAAGGTAAATTCGCAGAGCCGCGCCGTAGGGATGAAGAAAACCCTTGTGTACTACCGAGGCAGGGCGCCTCACGGGTCTCGCACTGGTTGGGTCATGCACGAGTACCGTCTTCATGAGAGGGAATGCGAAACCAATGCTGCTTCTGGCTTGCAGGATGCCTATGCTCTTTGCCGTGTCTTCAAGAAGGCGGCGGTCATAATCCCCAAAGTGGCAGATCACTATGCTGGTAACAATATCATGATGATGACAACTGATTCTCAAGGAACACCCCAAGTCTTTGACACCATGCCTTGGGATCATCATATTGGGCATAATGGTAAATGCCCACACTTATCTCAGGATCCATTCCTCAATAACCTTCCATCTTCATCGTCCTCCTCATTTCCTCATTATGGAGCCCTAACTTACTCTCCATCTAAGATGGATGTAGCACTAGAGTGTGCAAGGATGCAGCACAGTTTTTCCATGCCTCCATTGGAGGTAGTGGAGGAATTCCCTAATGTGGGAATTTCAGAGCTCAACATTATGACACGTGGCACCACTTCAATGTGTGGAGGAAGCATGAACAATAACAATGAATCGGATATCTTGCAACAGATTCTTTCACTTGCTAATGCTAATGTTTCTTCCCATGAATTCACAAATCAATCAAACCATTCACACACATTATTGGGTGGCAACAATGCAAATTATTCTGCTCCTCATCATCACGAACATGATTTTGCTTTTAATGCTGGCACAAGTTACACTAATCACGCCGTAAATGATATGAACCCCATGAGATATGAAATCCAACATCAAAACCTAAGAACAATAGAGATTGGAGATCTTGAAAGCGAGTTCAAGAGCTTTATGGAAGAGCAAAAGACGGTTCCGATTGAGGATATATCAAGCTTCCAAACAAACATACAAGAAAATGAGGTTCAAGCAGAATCTGAGCTACACAATAGCAACAAAGAATTTAGTGAAGCTGACATTGACAATTTCTCAATGGGGTTCATCAACGATGGTGACCCAAATGAGAACTTCATCGATGATGATGACAACATTGATTATTCAAATTCCACAAGCTTTGAGGTCCTTGAAGAAACCAAGGTTAGCCATGGAATGTTTGTGGCGACTCGCCGAGTAGCCGACACATTCTTTCATCAGATTGTTCCTTCACAAACCATCAAAGTTCAACTGAATCCAGTAACAATAATGGGCAACAATTCTTCCATGGAGATGCTCAAGAATAATCAAGAGTCTTTGTTCAAGAAGCTGATGATGATGAAGTCACCAAATACATTACCAAGTGCTATTGTATTTATCTTTGCACTATTGTTGACGCTTTGTGTTAATTTGAAGGGGCAAGTTGAAAATTATTGGGCATCAAGAAGTGATGATGATACGATTAATGTGAAGAAGAAATGTTGCTATGGTGCTAATAGAAGCATGAAGAGAATGAAACAAGTAGCTCACAAGATCATATGGAACCAGCAAGAAAAATCTTGGTGTGTTGGAATTAAAAGTGGGAGAGGATTTAGTGTGGTGTTGAAGAAAATTGGTATTTTCCTCTCCATTCTTTGGCTCTTTGTACCATGTGGGTTAACCATGTTACAATTAGTCCTTAATTGACCTAGTAATTTTATTTTCTTTTAGCTTCTAACTTAATTAAGGATTTCATTATTTAGAAGGATACTTGTATTTTCTTCTCACAACAATTCTTGTATTTTACTTGGTCAATATATTATTTAATTAAGAATTAGTATTGTACTCAATTGTATCAATAGAAATTGATATATAATTCATTTTCATTTAAACCATTTAT

## >AhNAC102-Arahy.JBU48Q.1

CTTTCAAAAATGGCATCGGAGCTTCAATTGCCGCCTGGATTTCGATTCCATCCAACAGATGAAGAACTCGTGTTGCACTATCTCTGCCGTAAATGCACTTCACAACCGATTGCTGTTCCGATCATTGCCGAGATCGACCTTTATAAATATGATCCCTGGGACCTTCCTGGTATGGCATCCTATGGTGAGAAGGAGTGGTATTTTTTTTCACCTAGAGATAGAAAATATCCGAATGGGTCTAGACCGAATCGGGCTGCAGGAACTGGTTACTGGAAGGCAACCGGAGCCGATAAGCCTATTGGACAACCCAAACCAGTTGGGATTAAGAAAGCTCTGGTCTTTTACTCTGGAAAGGCTCCCAAAGGAGATAAGACCAATTGGATTATGCACGAATATCGTCTTGCAGACGTAGATCGTTCTGTTCGCAAAAAGAATAGCCTCAGGCTGGATGACTGGGTGCTGTGTCGCATCTACAATAAGAAAGGATCAATCGAGAAGCAACAACCGAGCAGCGGCGTCAGTACCGTCGTGAACCAGAAGGCCGAATCTTCGGAAGTCGAAGACAAGAAGCCCGATATAGTTCCACGTGGCGGCGGCGGGGGCGTACTTCCACCGCATCCTCCTACGGCTCAGGCTTCGGCAGGCGGCGTGACGACAGATTATATGTACTTCGACAACTCCGATTCGGTTCCGAAGCTTCACACGGACTCGAGTTGCTCGGAGCAGGTGGTGTCGCCGGAGTTTGCGAGCGAGGTTCAGAGCGAGCCAAAGTGGAACGAGTGGGATAAAAACCTCGAAAGTGCGTATAATTACCTCGATGCCACACTCACCAACGGTTTTGGGTTCCCGTTTCAGGGTAACAATCAGATGTCGCCGCTCCAGGATATGTTCATGAACCTTCCGAAACCGTTCTGAACGCGCTAGTAATAGTAGAACACCAGAGAAAGAATCTTTTTATGGAAAACCTTTGATATGCAGTATGGTGCATTCCACGGCAGCACGAGACGTTAAGGTCCATGAGTGTGCCTGTGCGGGTGCGAGTCCATGGATGGGTCAACGAGATGGAACAGCAAGGGGAGAATCTGAAGAAGGGAAAATGAAAAAATGCATGCGTTAGGTGTGCAGCATTGTGCGCCCATTGATTTGGGTCGTGGGATTAGAATATCGTTTGGGGAGTCTGACGGTGGATGTTGGGTAGTCGCTAGGTTAGGCTGTGTATAGTTTATTTTGTACAATGTAGAGTGTAAGACCGGAGGGGGTAAACTATACGGTGGATGGTGGTGTATGTTGTTTCCATCTATTTATGATTAGTCAATATAAATGGTTAATTTTATTCTGTGCCACATAATTTCTTAAATTATTTGAAATTTAAATTTTAAATTTTAATTTTAATTTTAATTTTAAATTTCAACATGAGAATTAAAAATAATTTTATTTCATTGATATTAATGATAAAAAATAAAATTTGTTTCAAGTAGTTTGTATGAT

## >AhNAC103-Arahy.JE37KP.1

AATAATAAAAATAGTGAAAATAGTTGAATATGTATGTATGGTGGAAGAAAGGTCCAACGGCACCGCTCTGGGGCTGTAGCCACCCTGTCTTGTTCTTGAGATGTCATTTTCTGATCTTAAAAACACTCTTAGGGCAAGCATACTACCCTCATCCCCATGTAGGGCCTGAAAGCTATGTAGATCTTTCTATTAACTCCCATCCTCTCTCTCCTCTTTTTTCTCAACACCTATTTAAAACACACGCCCCATTTTCTCTTCTTTTTATATATTAACTCTGCCTCAAACACCCAGTTTAATTTCTTCAATTTCTACATTTTAACCATCAATCTCTTATCTCTGCCTAACACCACTGCACCACCCAATTCGTACAACTATTACACTCTCTAAGTATAAAGACAGAGAGAGAAATTAAAGGGGGTCCAAACTCGAAGCACCAACTACTACTACTACGATAGATATAGTATTATTATCAGTGTTGGATGGCAATTGCAGCGCCGAATTCATCTCCGACGATGAGTCTGAGTCACAGCCACAGCCACGAGGACGGAGGGACAACGACGGCGGCGTCCACCACGAACGACAACCTGAACGGGAACGGGAAGCAAGAGGATGATGATCACGAGCATGACATGGTGATGCCGGGGTTTCGTTTTCACCCGACAGAAGAAGAGCTGGTGGAGTTCTACCTTCGCCGTAAGGTGGAGGGCAAACGTTTCAACGTTGAGCTCATTACTTTCCTTGATCTTTATCGCTATGACCCTTGGGAGCTTCCTGCTTTGGCGGCGATAGGAGAGAAGGAGTGGTATTTCTATGTGCCTCGAGACAGAAAATACAGAAACGGAGATCGTCCGAATCGAGTGACGACGTCGGGTTATTGGAAGGCAACGGGAGCAGATAGGATGATAAGGACGGAGAATTTCAGGTCCATCGGCCTCAAGAAAACCCTAGTTTTCTACTCTGGGAAAGCTCCTAAAGGCATCCGTACAAGTTGGATTATGAACGAGTACCGTTTGCCCCAACACGAAACTGAACGATACCAAAAGGCGGAGATATCGCTGTGCCGGGTTTACAAGAGAGCTGGAGTTGAAGATCATCCATCGTTGCCGCGGTGTCTGCCAACAAGGGCTCCATCTTCAAGAACTGTTGATCACCAGAAGAACAAGCAGCAGCCTCACAACGATCAACTCAACATGGGATTTGGGGGGAACACCGCCGATGGAGCTTCTGATAATCGTGATCATGATGTAACCACCGCTCTCGCCCTCTCCAAACACAACACAAATAATACTAATAATGCTTATCGTGCTCCTTCAATGGGACTCCCACCGCTGCTTCTTCCCTTGGACGACGAAGCCGCCTTCGTCCTCATGCAGCAGCAGCACCATGCTGGCCCTTCTTCCGGCACCACCATGATGGATGATCTCAACAGGCTTGTAAGCTATCAACACCAGTACTACAACAGCAGCAGTAGCAGTAGTAACAATAATAATCCCAATCATCATCATCACCTGTTAATGCATCAACAACAACAACAACAACAACAACAGCAGCAGCAGCAAAATCCTCCTGCAATAATGTCTCTGAATAACACTCCTTCTCCGCTTGCAACCGCCTTCTCTGACCGCCTGTGGGAGTGGAATCCACTCCCGGAGGCCAACCAGCGGCAGTACAGCAACATGTCCTTCAAGTAATAATAATCATGCAATATTATTAATATATTTATTTATATATTATGAACTGCACATACATATAGAGACAATTATCTGTCCTAATTTCCATCCTATCAACTCTCTATATATGTTCCCAGCAGCTTGTTTATTATTTCCTTACTCTAATTCATCTTCTGCTGCGATGCATGCTAGCTATCTCTTAGATTGATTCCAATAACATAATCATTGTATGACTCTTATTCATCACATTTCAATATATCTATCTATGGATATTATGTTGATGATGATTGTAGTGCCTGCTACTAGTACTCTATATGTTAGGGTTTTAATTAATTAATTTTAAGTACGCCAGTTTCTATTATTGTTTACACAATTTCAATTCTCCATTTGAATCAAGTTGGGATAGAAAGGGTACGGCATCTTTTAATTTGTAATTTGCTTAGCTTGTAGTTTAATTTCATCTAAAATGGGGCTATAGCTTCAT

## >AhNAC104-Arahy.JHHH6T.1

ATGCACAGTCTCTTCCCATTACCAACAAAAACCCCAGGCTACAATTTCCCGCTCACTCTACCGACTGTCGCTTTCTTTGTCTCTCTGTCGATTGAAAATGGGAAACCGATGCTGAAGCTCAATACTCTCCACAGTTCTGCTTCAGCTCAAGCTTCTTCCTCTTCCTCTCTGCAATGGCTAGGTGAGGTTTTAGAATCAGATTCAGATATGGCCACATTGCATAATGAGATACACGTTTTCTTCCTTAGTTTAGAAGGGGAAGGGAAAGAAGTGAAAGGTTTGAAGAGTAAAAGGAGCTGGCTTATTGACATAGGTGGATTTGCAAAGAAAGTGAAAAGCACTAATTTATCTCCAGCTGATCAAATCAAAGATTGTGGGGCATATCGTGATTGTCCAAACTGCCATTACCGTATTGATAACCGTGATGTTTCTACTGAGTGGCCTGGCTTTCCTGTTGGTGTGAAGTTTGATCCTTCTGATGTAGAACTCCTAGAACATTTAGCAGGAAAATGTGGCATTGGAAATGCTCAGCTGCATATGTTTATTAATGAGTTCATTCCAACAATAGAAGAAGAAGAAGGTATTTGCTATACACATCCAGAAAATCTTCCAGGTGTCAAGAAAGATGGGAGCAGTGCCCATTTCTTTCATAGAACAACCAATGCCTATACTACTGGTCAACGGAAGCGTCGAAAGATTCATCATCAATGTTTGACTGAAGAGCATGTACGGTGGCATAAGACTGGTAAGACAAAAGCTATATTGGAGGATGGAGTGCATAAGGGCTTTAAGAAGATCATGGTTCTTTATATAAGACCTAAGAAAGGGTCCAAACCTGATAAAACGAATTGGGTGATGCACCAATATCATTTAGGAACTGATGAAGAGGAGAAGAACGGTGAATATGTGGTTTCAAAGATTTTTCAGAAGCAAACTGAGAAAAATGAGGAGAATCCAGCGGTCGAAGATTCTGACCAGAATGAGAAAAATGAGAATCGATTGGCTGATGATTCCAACTGTATAGCATCCCGAACCAGTCCTAGAACTCCGAAACCAAATCCCCCAAATCCACCTAGAGCTGGAAATTTTGTTGACAATGATGATAATATTGACGAAACTGAACTTCCATTCACTCAGGATGTGAAATGTGTCCCACAATGTGATGTTCTGGATCAGAACAATGCTGGTGACCCTGCATGGCTGGCAGGTGAATCGCAGGCTGTGGAAAACTTCGACTTTGATGGCTTGGATGACATCTTGTTCTGCAATGAAATATTCGATTCATCATCTCTACTAGATATTTCTGGAACGGAAACCATGATAATGATTCCAAACGATATGCTTGGGAATGATGGTTTATCATACGGAACTTCCGTTCTTGATACCCTTGACTTGGGTACTCCCCCAGATTTTGATCTTTCAAATCTGAATTTTTACTCTCAAGATAGTATTTTCGACTGGGTCGACAGATTATGAAGTGATTTCTGAAGTTTGAGTCCATCAGAATGCATGTTTGTTCAGCTCTTAGATGCTTCAGATTCAAATATAGTAAGAGAAACATAGGTTCTCACGTGATTTGCAAGCAGAAATAACAGTATTTGTTTGTCCCTGGTTCTTGAAATTCTGCAAATTTTGTAATCCCACACATTTGGGCATTGTTCAGGTGGGTGTATTGTTCATGTGGATTTTAGGTATTATTCACGTGGGTTGTATTTGTGTCAAATAGCTTTTGTGGTATATGTTGTTTCCATAGCCATCTAAACTTGGTTGCCTCATTCTGTTGTGGTAGATACATCTCTGGTAGCAAAGTCCAAACTTATATAAGTACTTGATATTCGACTGTTTTTATGTCAATACTAGAATAACTATCCGCGGCATAGTTGTCAGAACCGAACCGGTGATCGAACCGGTCAGGTTACTGGATCACTGGGTCACTGGTTCAACCGTTGGATCACTGG

## >AhNAC105-Arahy.JUA047.1

ATGGAAGAAGGAGGAGGAGATCAACATGCCTCTAACAGCAGCTACACTTTTCCACCAGGTTTCAGATTCCACCCTTCTGATGAAGAACTCATAGTTCATTACCTACAAAACAGAATCAGCTCTCGTCCACTTCCAGCTTCCATTATAGCTGAGATTGATCTTTATAAGTATAACCCTTGGGATTTGCCAAAGAAGGCTTTGTTTGGAGAGGAAGAATGGTACTTCTTTAGCCCGAGAGATCGCAAGTATCCAAATGGATTGAGGCCAAACAGGGCAGCAGGTTCAGGGTACTGGAAGGCTACCGGAACTGACAAGCCGATTCTCACTTCTTATGGATCGAAGCGCATCGGAGTGAAGAAAGCTCTTGTCTTCTATTTAGGTAGACCTCCAAAGGGGACTAAAACTGATTGGATCATGAATGAGTATAGATTGGTTGACACAATCACCAGCCCCTCCAGGCTCAAAGGTTCCATGCGCTTAGATGACTGGGTACTTTGTCGCGTTCGACACAAAGGCTACTCATCGAAGAACTCATGTGAGAATCAAGATAATCCTTGTGAACCAAACATGCTATCAAATCTGCCAAGGTGTGATGAAGGTTATCCAGCAACAAACATGAACTTCCATGCTGATATGATCACTGATTATCAATACAAAGACTATCAGATCCTAGCTTCTATCCTTGTTGGTGGCCATGTTCCTACCACTGAGAGCATGTCAAGTTTGAACTTGAAGGATGGCAAAGGCAATGATCCAATAACTTCAGTTCATGAAGATGGTTTCCACAGAGAAGATTCTTCTACAACAGTTTCTCCTTTGGACTGTTACTTCAACTCACTGAAAAGAAAATCTAATGAGGATAGCCAATATGAGAATCTCATTTCCTTTAACAGGAAGTTGAACATGGAGACCACAATGGATGATGAATCTTCTATCATCAATGGAGGTTTGAACTTCTACAATCAAAACCAGTCTCAAGATGACATAATATTCAATAAGAGAGCAGCAGAGCCTAGCATCAACTTTCAAGAGCTAAAGCAATCAGCTTTTATAGGAAGATACCCGCAATGCTCAAGTGATTGA

## >AhNAC106-Arahy.K4491K.1

CGGTTCACGGTTCGACCGGTTCGACCGGCCGGTTCGAACCGGTTTTCAGAACATTGGTCCTACCCCTGCCATTAAGTACTCCATATGAAAAGTGATTGGGGTAAACAATGCAAGAATCTGTAACGTTACTTTACATTTACTGATTTGCCCTTTTTTAGCTTGGCGACCAAGGATGCAAGGTTAAACTTGTAATTCTGGCCCTTACACGGTTTGCGTCTTTACTCTTTACGAAACTCGACTTTCTCTTTCCGTCTCAATATCCGAACCAATAGGCTCGGTTTTGCTTTGGCTCATTTGTTCGACAGAACAGCACTGACGAAGCACAGGTTCGAATGCACAGTCTCTTCCCATTACCAACAAAAACCCCAGGCTACAATTTCCCGCTCACTCTACCGACTGTCGCTTTCTTTGTCTCTCTGTCGATTGAAAATGGGAAACCGATGCTGAGGCTCAATACTCTCCACAGTTCTGCTTCAGCTCAAGCTTCTTCCTCTTCCTCTCTGCAATGGCTAGGTTTAGAAGGGGAAGGGAAAGAAGTGAAAGGTTTGAAGAGTAAAAGGAGCTGGCTTATTGACATAGGTGGATTTGCAAAGAAAGTGAAAAGCACTAATTTATCTCCAGCTGATCAAATCAAAGATTGTGGGGCATATCGTGATTGTCCAAACTGCCATTACCGTATTGATAACCGTGATGTTTCTACTGAGTGGCCTGGCTTTCCTCTTGGTGTGAAGTTTGATCCTTCTGATGTAGAACTCCTAGAACATTTAGCAGGAAAATGTGGCATTGGAAATGCTCAGCTGCATATGTTTATTAATGAGTTCATTCCAACAATAGAAGAAGAAGAAGGTATTTGCTATACACATCCAGAAAATCTTCCAGGTGTCAAGAAAGATGGGAGCAGTGCCCATTTCTTTCACAGAACAACCAATGCCTATACTACTGGTCAACGGAAGCGTCGAAAGATTCATCATGAATGTTTGACTGAAGAGCATGTACGGTGGCATAAGACTGGTAAGACAAAAGCTATATTGGAGGATGGAGTGCATAAGGGCTTTAAGAAGATCATGGTTCTTTATATAAGACCTAAGAAAGGGTCCAAACCTGATAAAACGAATTGGGTGATGCACCAATACCATTTAGGAACTGATGAAGAGGAGAAGAACGGTGAATATGTGGTTTCAAAGATTTTTCAGAAGCAAACTGAGAAAAATGAGGAGAATCCAGGGGTCGAAGATTCTGACCAGACTGAGAAAAATGAGAATCGATTGGCTGATGATTCCAACTGTATAGCATCCCGAACCAGTCCTAGAACTCCGAAACCAAATCCCCCAAATCCACCTCGAGCTGGAAATTTTGTTGACAATGATGATAATATTGACGAAACTGAACTTCCATTCACTCAGGATGTGAAATGTGTCCCACTATGTGATGTTCTGGATCAGAACAGTGCTGGTGACCCTGCATGGCTGGCAGGTGAATCGCAGGCTGTGGAAAACTTCGACTTTGATGGCTTGGATGACATCTTGTTCTGCAATGAAATATTTGATTCATCATCTCTACTAGATGTTTCTGGAACGGAAACCATGATAAATGGATCTGCTTCAAACGATATGCTTGGGAATGATAGTTTATCATACGGAACTTCCGTTCTTGATACCCTTGACTTGGGTACTCCCCCAGATTTTGATCTTTCAAATCTGAATTTTTACTCTCAAGATAGTATTTTCGACTGGGTCGACAGATTATGAAGTGATTTCTGAAGTTTGAGTCCATCTGAATGCATGTTTGTTCAGCTCTTAGATGCTTCAGATTCAAATATAGTAAGTGAAACATAGGTTCTCACGTGATGATTTGCAAGCAGAAATAACAGTATTTGTTTGTCCCTGGTTCTTGAAATTCTGCAAATTTTGTAATCCCATGTAAACACATTTGGGCATTGTTCAGGTGGGTGTATTTTGGTGTTCATGTGGATTTTAGGCATTATTCACGTGGGTTGTATTTGTGTGTCAAATAGCTTTTGTGGTATATGTTGTTTCCATAGCCATCTAAACTTGGTTGCCTCATTCTGTTGTGGTAGATACATCTCTGGTAGCAAAGTCCAAACTTATATAAGTACTTGATATTTGACTGTTTTTATGTCAATACTAGAATAACTATCCGCGGATTCA

## >AhNAC107-Arahy.K9ZHT4.1

GTTCCTCTCCACTTCGTTGAAAAATTGTGCCACAAACCTTATTAACCGTTTTTGTCTTCTAATAATATCCCTTACTAGCTTCTTCATCTTCCTTAGAGAGAAAGAAAAAACACACACACACACACAACACAAAAAGAATAAATAAGAGAACATCATTGTTATCATTATTGTTATTATAATTGTTATATATGGGGGTCAATGAAGATTTGATGATGAAGGATGATTCATATGCATCATCAGTAATGGAGGAAGAGGATGATGTTCCACTTCCAGGGTTTAGATTCCACCCAACAGATGAAGAACTTGTGAGTTTCTATCTAAAGAGGAAGCTTGACAAGAAACCAATCAGCATCGAACTCATCAAACAGATTGATATCTACAAGTATGATCCTTGGGATCTTCCAAGAAATCTGGACATTGTGTAGAATATTCAAGCGAAATGTGTCACAAAGGAAGCACACAGTAGACTTGAGATCACATTTAGTAACAGCTAATAGTAACAAGCACAAAACCACTAGAACCCATGTTGTTCAATCCAATAATAACAATATTAATCAACATCAAGAATCTTACATCAACTTTGGTGCAACAATCATTGGCCATCACCATTACCATCATCATCGTCATCAAAATGAACAGAAGCCAGTGACTAACTACACAGCATGCAATAACAACACTGATCAAATCCAAAGGAACAATAGCAATCATCATCATCATCATCAGTTGAACTATCACCCTTCTTCAGCGGTGGCTACTACTGTGCCACAGCAACAACAACAACAATATCATCATCATCATCAGCTAATGACGGCTCCAGCTTCTAACATGTGGATTAATCCTTCTGCGATGAACGATTTGTTTGCATTTGATGATGACTGGGATGAGCTTGGATCTGTTCTCAAATTCACTGTTGATACCCCTAGCTTGTAAATTCATATATATATGTATATATATATATATCTTAGTTTATTTGAGGAGCAGTCATTAGATATATACTTTATAACATATATATATATATTAGTGTGTTTGTGTAAGAGAACATATGTTAATTAATATCGAAAATGGAGGTTTTAGCTTGTTAGCGGACTTTTATATATGGGAGATTCTACGGTGTTTTTTAAATTTTTGTCTAACTTGTCTAGATAAGACTCATTTGTAGTGATGAGAAAAAATAGAGCTCACTCGTTTATATTTGCATTGAGCAACTTAGTTATAGGCACCACAGCAATAATTAATTGGGTAGTGTTAGGTAGCCAAAAGATAATGTATACTATAGATATAATACAAAATAAACGTCAGTTTCATGATAAGTGAGTGGAGTACACTTGCTTATCAATAATATACTTATCACTCTACCTTCTTTCCCATTCTTCGAGTAATTAATTCCATTCCCAAAATTAATTT

## >AhNAC108-Arahy.KK00U0.1

ACTGTGTGATACATAGATGATGATGATGATGATGCTATCAATGGAAGAGCTAGCGTGTGAGCTGAGTGATCATGAAAAGAGAAACGCTCAAGGTTTGCCACCCGGTTTCAGGTTTCACCCAACTGATCAAGAACTCATTACCTTCTATTTGGCTTCCAAGGTCTTCAATACTACTACTACTACTCATGTCAACTTTGTGGAGGTTGATCTCAATCGATGCGAGCCATGGGAACTTCCAGAAGTGGCAAAGATGGGGGAGAGAGAGTGGTATCTGTACAGTGTGAGAGACAGAAAATACCCAACGGGCCTCAGAACTAACAGAGCAACCGCTGCTGGGTACTGGAAGGCTACCGGCAAGGACAAGCAAGTATACGGCGGCGGTGGCCTTGTTGGGATGAAGAAGACGTTGGTGTTCTACAAAGGGAGGGCCCCCCGCGGTCAGAAGACTAAATGGGTAATGCATGAGTTCCGGTTGGACCCTCACAGCTCTCCTTCCCTCTCTAAGGATGAGTGGGTAATATGCAGAATATTTCATAAAAGTGGGGAAAAGAGAACTCCTACTACTACTCCTGCTCCTCTGCTACTTCATCATCAACAACACGATCCATCATCACTGTTCAATGACCATATCTCCCACTCTCATAATCATAATCAAAACCTCCTCTCGCCATTGCTTCATCCTTTCCCAATCCCTGAAGAAACCACTAAAACCAAATCATCAACAATTAACAGCAACCATTACCCTCCACCACCACCTTCCTCCCAACACTTGCTTAAGCTCAATAAGTCTACTAAATTAACAAAAACAGTGCCTCCTTCTCCATCATTCTTCCAATACCAACAGCTTCTAGAAGATGATCCCAACTTATTGCATTGGATGGACAGTGGTAATAATAATAATAATAACTGCAAGGCCAATAATACTGCTAGTAGTGTTGAGATAATGGATGCTGCTGCTGCTGGCTTGATAGCATTCTCATCAGGAGGACCTTCACCTACTCCTACTAATAATAATAATAACAATTCTGAAATAATAAGGGACATGATGATGATGTCTTCTTCTTCGGCTTCTATGCTGCACATACTCGACGATGCTCCTCTTGGAATTCAATCTTGGCCTCATCATCATCATCATCACCTTCTGTAA

## >AhNAC109-Arahy.L5HHP2.1

CCAACTGGGTTGCCTTACATTCCACGTTTCTTTCTTTCTTTGAAATACAAACACAAACACAAACACACACACTGGTATTTAGGGGGATTCTTTTTCTCTCAGCTTTTTTTTCAAATCTCTCTCGATCACTTCCATGATTCTCATCACCTTTTCTTTCCCACTTTCTGCATACTCGATCTCTTCTCTTTCGATTCTCATCAGCAATTCCTAGAATTCACCCTTTCATTTTGGGGGTTCTTACATATCAAATGGAAAACATTTGTTCAGAGGTTGAGATGGATTTGCCACCGGGATTCAGGTTTCACCCAACTGATGAAGAGCTTATAAGTCATTACCTATACAACAAGGTTATTGACACTAACTTTTCAGCCAGAGCCATTGCTGAGGTGGACTTGAATAGGTCTGAGCCTTGGGATTTGCCATGGAAGGCGAAAATGGGTGAAAAAGAGTGGTACTTTTTCTGTGTAAGGGACAGAAAGTACCCAACAGGATTGAGGACAAACAGAGCAACAGAAGCAGGGTATTGGAAGGCCACTGGAAAAGACAAGGAGATATACAGAGGCAAATCACTTGTTGGCATGAAGAAGACCCTTGTCTTCTACAAAGGTAGGGCTCCCAAAGGTGAGAAATCTGATTGGGTCATGCATGAGTTCAGGCTTCATGGTAAATTCAATCCCCACAACCTCCCCAAATCTGCAAAGAACGAGTGGGTGATTTGCAGGGTGTTTCAGAAGTCTTCAGCCGCCAAGAAAATCCATCTTACCGGGATAATGAGGTTGGACTCTTCTGTTTTCTTGCCACCATTGGCAGATTCCTCATCATCACCTTCCAACACTGCTACTACAGCACCTTACGTGCCCTGCTTCTCCAATCCAATCATTCACAACCAAGTTGGGATCTTTGATCCATTTAGCAACACCCCTTTTGTTGCTGATTCATTCTACTCTTCTCAAGGGATGCCAATGCAACATGCTCAACCACCAAGTTCCACATATTCCGCTTCCGGCTACACCACTCATGACCATTCAATTCTCAGAGCCTTGCTTCAAAACAATTCTTCAAACCTCAGGAATGGTTTCAAGCCTGGAGAGAGGGAAATGGCCCATCATCAAACTTCTCTTGTTGATGCCAACAACAACAACAACAATGGAATCACTTCTGTTGTTGCCCCACAGGACCTTTCTAGCCTCTGGAATTACCAGGTTCAGATCAAGTAGCTATGGAAACTGAATTCTGAGGGTACAAGAGAAGCCATTCTGTTGTCATTTATTCTCTGTCTTTTGGTGTTGTTGCTTGTCTTAATTGATTTTAAGTAAACCATGTATGAATATTTTGTGATGTCAGTAATAATCTTGTGAATTTATACTATTTTGGTCCTTTTGATACTTTGCTATTGTTATGGTTGCAATGACCTAAATTATCATTATCATTATTCTAGGGTTTAATTGTAGTTTTTTTCCTTACATTACAT

## >AhNAC110-Arahy.L9IK9Y.1

TGGTGATAAAATTTGTAGTGATAAAGTATAAAGAGTATCCAGTTACATAAGTGCGACAACAAAGTGATGCTCCATCAACATACACTAGCATGGTGATAATTAGATGCATGGTATTTGGGCAAGTGAGCAGAAAACAAAATTAGCGAAAGTATTATGACACAATAATTATCTCATCTCATCATAAATCAAATAATTTGGAATGGGAATGAGTGCTTTAGCCTTATAAACAAAAAACCAAGGAAGTTGCCTTACATGCCACGTTTCTCTTTCTCTCTCTTCCCCCAGATAGCAGTAGTATTTAGGTGCCTCCTTTTTCTCTCCTTCGTTTTCCCTTCTCATTATTCACATCCATCTCCATATTCCATCTCTCTTTCCTCTCCCCCCCCCCCTCTCTCTATATATTGAAAGTACAATAATATAAGTAAGAAAGAAAGATCAGAGCGAGTGAGTGAATGAAATGGAAAAAGTGGCATCATTGGTGTTGAAGGAAGAGGAGCAGATGGATTTGCCACCGGGGTTTCGGTTTCACCCAACTGATGAAGAGCTCATAACCCATTACCTATACAAGAAAGTTATTGACACCAACTTCGCCGCGAGAGCCATTGGCGAAGTCGACCTTAACCGCTGCGAGCCTTGGGATTTGCCATGGAAAGCGAAAATGGGAGAGAAAGAATGGTATTTCTTCTGTGTGAGGGATAGGAAGTATCCGACCGGGTTGAGGACAAACCGGGCGACCGAGTCTGGGTACTGGAAGGCAACCGGGAAAGACAAGGAGATTTTTCGAGGCAAATCGCTCGTCGGAATGAAGAAAACGCTTGTCTTCTACAAAGGAAGAGCACCAAAAGGAGAGAAAACAAACTGGGTCATGCACGAGTTCAGGCTGGAGGGGAAATTCTCCATCCATAACCTCCCAAAAACCGCAAAGAACGAGTGGGTGATTTGCAGGGTGTTTCGGAAGAGTTCAGCTGGCAAGAAGGTTCACATCTCTGGAATCATGAGGCTCGACACTTTCCGAACCGAATTGGATTCTTCCGGTCTACCTCCCTTGACGGAGACCTCGCCCTCTTTCGACACCATCCATGACGAATCGCCTTACGTGCCCTGCTTCTCCAATCCAATTGATGTTCCAAGAAACCAAGCCGCAGGCGGAAGCGGAGGAGGAGGAGGAGTCTTTGGTGGTTCCTTCCCCAACAACTCCTCCTCTTCTGTTCCCGCCTATGCGGTTTCTTCCAACATTCTCCCAAGGATGCCGATTTGCGGCGGTTCCTTGTACTCTACTCAACATCAAGATCAGAGTATCCTGAGGGCGTTGTATGAATCGAACGAAAGGGAAATGATCAGTGTGTCACAGGAAACAGGCCTCACTACTGAGATGAACGTGGAAACCAATTCCGTGGTGTCCAATTTTGATTTAGGGAGGGCTCACTTTGAGAGTTTGTGGAATTACTGAAAATTTCAAGATTTAATTAACTACTAGGGTTTGATTGTGTTACTCTTGTTTGTTTGTTTGTTTGTGTATTTGTCTGTAGATTTTATGATTTACATTTACGTGTGTAAAGAGATTGTGCTCATGATACATGAATGTGTCATCATGTTATTATGTAAGTAAATCTTAAAAGTTGAATTTGTGAAATTAAGCTCTGTGATTTAGCATGTTATTTTTGTTTTCATGATAAAAAAAGAAAGATATGAGAATGAAAAGTTATTATTTCAGTACCGTAGCTTGTTCGTGTTGCTTATCTGGATTGAATCAGTACAGTCAGCTAATAGTAAAAGCATGAGGCGTGACC

## >AhNAC111-Arahy.LV3APC.1

CATTTTTTTTAATTTAAATTATTCATACTTTATTTTATAATATATATATATATAATACATTTTCTCTAATGTATCTTTTCGGTCCCTTGTGTCCCACAGCACATATATATAGTGTTCCTTTACATACACATTGCAGTACACACAACACACCACCCTCTCATTCATTACATCTTTTTCTTTTTTCTTCCTTATCCCTTTGAAAGTGTACCTATTTGAATGTCTTCTCTCTTCTTCTTCTTCTTCTCTTTCTCTTGGGACCCTTTGGTGAATTGAACAACAATCAAGAACAACCAATTTTAGACCTTTATATATTACATACATATCTTTGATTTGTTAGTTGTTGACCTGAATGCCAGAAAGCATGAGTATATCAGTGAATGGACAATCTCAAGTTCCACCTGGATTCAGGTTCCATCCAACTGAGGAAGAACTCCTTCAATACTACTTAAGGAAGAAGGTCTCTTATGAGAAGATTGATCTTGATGTTATTCGTGACGTTGATCTCAACAAGCTTGAACCATGGGATATACAAGAGAAATGTAAGATAGGAACCACCCCACAAAATGATTGGTACTTCTTCAGCCACAAAGACAAGAAGTATCCGACCGGAACCCGGACGAATAGAGCGACCGCGGCCGGGTTCTGGAAGGCCACCGGCCGCGACAAGGTGATATACAGCAACGGGAAGAGGATTGGAATGAGGAAGACGTTGGTATTCTACAAAGGAAGAGCCCCTCATGGCCAAAAGTCTGATTGGATCATGCATGAGTATAGGCTTGATGATAACACCACCAACGATGCCAATATTGTTTCAAATGTGATGGGAGATGCAGCACAAGAAGAAGGGTGGGTGGTGTGTAGAATATTCAAGAAGAAGAACCATCTAAAAACCCTAGATAGTCCTTTAACCTCTTCTATCTCCGGTGACGGAGGTAGGAGGAGCCACCACCACCACCACCTGTTCGACTCGTGCGACGAGGGCGCCTTAGAGCAAATTCTCCAACAAATGGGAAGAGGTGGTGGTGGTGGTGGTTGCAAGGAAGAGATCAACAACTATGATCAATCTAATAACAACAACAACAATAACAACAACAACTATGGTGGATCATCATCGTTAACAACAAGGTATGCAAGACCTTTTGACACAATCAACAACAATGTTGATAGCAGGTTCTTGAAGCTCCCAAGCCTAGAGAGTCCAAAATCAACAAGCATGGATCATAATAATAATAACAACAACAATGATAATGATGATAGCAATGAAAATAATGGGTACCATCCTATTATTCCAGTTGAGATGGTAACTGAAAATGAAGGGTCATTCACATGTGACAATCCCAACAACATGTTTCATCATCACCATTTGGGTGGTGGCGGTGGTGGCAGCAGCGACGGCGGTGGCGGTCTTACAAATTGGGTAGCGCTAGATAGGCTTGTTGCTTCTCAGCTTAACGGTCAGACCGAAGCTTCTAGAAAACTCTCTTGCTTCAATGATCCCACCATGGGGTATGGCACTGGAAATCATGATCTTCTATTTCCATCCGTCAGATCTACTTCATCGTTGACGTCATCGTCAGCGTCAATAAATCCAAGGGCTGTTATTAGTGCGGGTGCAGGTGCATACATTTCTCCAGGCGCACAGGATTATACCACCACAAGCGAGATTGACCTGTGGAACTTTGCTAGATCCACTTCTTCGTTGTTGTCATCCTCTGAGCCATTGTGCCACGTGTCAAACACGTCAGTGTAGCCAGAATAATAAAATCAATTAAAAAAAAAAAAGAAAAAAACACGCTACACTTATTTTTACTACTTCACCTCTTTCCTCCCATGTTCGATTGTTAAAGTATAGGGAAAATAATTTAGTCTTTGATCATATCATCATGTTTAGTTAATTTTCCTCTTGATGAGTCAGAGGATTATTATTATTATTATTATTGCATACTGTTAAATGTGATGATGTTACTACTATAGTATACACGGTACTTTTTTTTATAATTTTAAAATTGGGTTTGAATCTTGTTATTGGTAATGATAGGACATGTGTAAGTATGTAATAGTAATAGTTTGTATGTAGTATTATTATAAATCACCACTATACTTGTTATATGATGATGATGATGATATCACTTAGAATTTTAGTTATATGCTTTCCCATTAAAAAAAAAAAAACAAATTAAATCGTATTCTATACATCTTTGAATGAT

## >AhNAC112-Arahy.M99KVR.1

TGCATGCTATCATATATACTATATATGTGTCGCTTTTCTCTTGTATAAATATAGTTCCATGTTAGCTTAACTGAATTTCATCGATGTGCATGTATAGTATCAGAGAGTAGAGAGAATATAATACAATCAAATAATGGAAAAGTTAAATTTTGTGAAGAAGAATGGGGTAAGTAGAATGCCTCCTGGATTCAGATTCCAGCCAACGGATGAAGAGCTTGTGTTTCAGTATTTGAAATGTAAGGTCTTCTCATTCCCCTTGCCCGCTTCCATGATTCCTGACATCAATCTCTCCAACTATGATCCTTGGGATTTGCCAGGAAATTGTGATGAACATCAAGAGATGTATTTCTTCAGCAGCAAGGAACCCAAGTATAGAAATGGAAGCCGCATGAACCGAACAACCACCTCTGGCTATTGGAAGGCAACAGGATCCGACAAAAGAATCATTTCATCTTCTAATAATAGTGACGATAATAGCATTCTTGGCATTAGAAAAACCCTAGTGTTTTACCAAGGGAAATCTCCCAATGGCACTAGAACTCACTGGGTCTTGCATGAATATCGCCTGGTTAGTACTACTCTACATGCTAATAACAATGCTTGCGATATAGGAGATTGGGTTCTGTGTCGCTTATCGGTGAAGAAAAGGAGTGTTGGGAGTGGTAGTATCATCATAAGCAAGAAAGCACGTTCTTCACCATCTTCATCCTCATCTTCTTCCACTTCAAGTAATAACGTCATGGAAGTATCTTCTTCATATGCTTCTTAATATCAACAATGCACTTCCTCCTCATGGTCTTGCTGACTATTGATTAAAACCACCATACATATTCTCACTCACAACCCGTCGTTTCTGCAACTAAATGGGTTCAGACCCTTTAAAATTAACAAGAGAAATATTAGTGCGCTATTAAAATTTATTATTTTTGGTCATCACTTTTAGCCATTAACTCAATTTCTTTAGTTTAATAATTCAATGACATGTTTTTAGACCACACTTTTAAATATTGATGGCTAACGAATGATCAATAATAATAAATTTTAATGGTCTTCTAGTATTTTTCGATTAACAACACTAAATATGCTAATTAAGACTTTTTTATGTATCTTATATATTGTTGTTTCTTATTATAATATTGTAATGTACTTATCT

## >AhNAC113-Arahy.MFVS6B.1

GCTGCGCGCTAATGGCATTGTGGCAATTATTTCCAAAACCAAGGTCCAAAGCAGCACACAATAGTATAAACGAGGATTTTTTTTTGGGTAAATAAAACGAGGAATTTTTATTTTAACGCGCTTCACTTCTCACTTCTCAGTTCTTCCTCAAACGAAAATCCAAATAGTACAAATACTGCACCAACCCTTCTCTCATCTCTGATATTTTTCTTTATTTTTTATATATATATTTTCTCTTTCGATCACTGTCTTCTAGAAAAAAAAAAATACTAAAATTCTCAGAATCCTCTCTGATTCCAGTTCAGCTTCACTTAGTAGAAACCAATCAAAAGATCATCGTGAAATTTTTTTCTTGTTACTTTTTTTCTTCCTTTGGTTGTCACGAACGGTGCCGTTTTATATATACATAAAATAGAGACACCGTCGTTTTGACACAAACGGTGGTTTTGTTATGGGTGCGGTGGAGGTGTTTCAACAGCAGCCGCTGGTGGTGGACGCTGCTCCGGTTTTGTCGCTGAACTCGCTGCCGTTGGGGTTCCGTTTCCGACCGACAGACGAGGAGCTCGTTGACTTTTACTTGCGGCAGAAGATCAACGGCAATGGTGATGAGGTTTGGGTCATTCGAGAAATCGATGTTTGCAAATGGGAACCTTGGGATTTGCCTGATTTGTCAGTGGTACGGAACAAGGATCCGGAGTGGTTCTTCTTCTGTCCACAGGACAGGAAGTATCCAAATGGACACCGGCTGAACCGAGCAACAAGTCATGGATATTGGAAGGCGACTGGCAAGGATCGGAAGATTAAGTCCGGCTCGACCGTGATTGGAATGAAGAAGACTCTTGTATTCTACACTGGCCGTGCTCCCAAAGGGAAGAGGACCAATTGGGTGATGCATGAGTACAGGCCTACCCTGCAGGAGCTTGATGGTACCAATCCTGGACAGAATCCATATGTCCTTTGTCGATTATTTAAGAAACAAGATGAGAGTCTTGAAGGTTCAAACGGTGAAGAAATGGAGCGAACTACTTCAACTAATTTAACTGCCAATTACTCTCCAGAAGAAATACAATCAGATCCAGCTGTTAAATCGGTTTCTTCTTCACAGGCTACAGAAGATGACAAGAAACTAGCAGTTATCCCTTTGACCCCTGAAGAAGCAATTTCCAATGTTATAACCCCGGTCGGTTGCCAAAGCGATGGATGTGATGCTTATGATGCACAAAATCAAATCGCAGCAGGAGATCCATCTAAGGAGGAGGACTTACAGGTGAACATGGACATATTTTATGACCCGAGTGAGCTATTTGACGATAAATTATTCTCCCCACTTCACAAGCATATTCCAGAAGAACTTTTTCATCAATCAAACAATGAAGCCAATGGACATTTTGGGTTGCAACATCAGTGTGGAACAAATGAGATCAGTATTTCTGACTTCTTTGACTCTGTTATTAATTGGGATGAGATCTCTGGTGACAATTCCAGTGGCCAAACGCCAAACTCTGCTTGGTTTGATGTTCAGCACAATGAATCATGGGGAAACTCAAATGTGGATATGGTCCATGCCAGGCCCCTACAAGTAGGGGGTGCAGATTATCCAGGGGATGCAACCGAGGGAAAGCTCCCTTTGTTGAAAACTAGAGAATTCAATCCCAACACCTCTTATGACAATGCGATCAGCAACAACATGGGATTATTTCAGGACCATTCCCAGATGGCTTTTTCATCTGATGTTAATATGCTCCAAGGTTACCTTGCAACCAACAATTATGAGCAACCGACAAACTTCAATATGGCTATGGCTAATAGTGACAACACTGGAATTAGGATAAGGTCTCGGCCACCAGGTTATGAAGGGCCAAATGCAAACTCCAATATGCAACCACAAGGTACTGCACCTAGGAGAATACGGTTGGCACGAGCTCTTGCACCTCAACACACGTCCAATGAGGCGGCAAAAGATTCGAGTTACGAGTCAAAAGATCGAAATTCACAACTAACCACTGCCAGGGAGATGGAAACTTCCAAAGACCTTGCAGCTGGTGAGAGTGTTACTGTTACTAGTGATGTGGAGGAACAGGAGACATCACCAGTTGAAAATAAGGAATTTGAAGACTTCAACACAGTCCAGCAGAGCACATCATCAGCTTCCTCGAATCTTTCCACATGCTCTTCTGATTCTGAAGTTTCTTATGAGGCAGAAAAAGAATCCGGTTGGACATCAGAAGACCATAGTCCAAAACCAGCTGCCGCGGGGGCCAGTAAAGCTTCCGAAGACCAAGTTCCCAGCGAGTGCATCAATGATATCACTGATGATGTGGATGAATCCAGGATACCAAACGCTTATACTCTAGAGGTCTCAAAGGAGGAATCCTTCTCGGACTCAGAGTCGAAAGACTCTCTATTGCGTAGAAAGGTGTGTTACCCATCGAAGTCTTCCTCAAATCTAGCCAAGTGGTATTCGGTTATTGCAATCTCAGCCACTTTGGTGGTGTTACTAGCATTCCTTGTTAATACATGGGGATATGGATATTACCTTAAAGTTTAACTGCATAGTAGGATATGTATCATTGCATAGAGCATATTTTGCCTCCATTGCGTTTTTGGGCTTAGTTAAGGAGGCAAGTATGTAAGGGTTGACTTTGTAGCTTTATTTGAATATAATCTGTATGTGCATATGTTATATATAGCAGAATATGCCTAATCTGCTACTAGCTTGTACTATATGTGGAGTTGTAAACTCATGTTTATAAGTTAGATTCAGAATTTCAGGGTTTCCAACTTTTTGGAATTTAATTTTGATTGCCCGTGGTGACCACCATTGGAGAATATGCCTGCCTAGCCAAGACCCAAGAGTGTATTTTTAGTAAAGAAAAGTTTAGGCAGATTCGGCTGTTTGATTCATTCATTTATTTATTCTATTTTTTGTTCTGTTGGTTTAAAGGTAAGCTTTTAGTTAAGAAGCTAAAAGATATAGAACATTGAAAATCTGGGGGAAAAAAGATAATGTATATATAAGAAATCAATCATTTTACATAGAATATATTTTAATGTATTAAAATATAAAATATATATTAAAAATAAATTAAATGATATATTTAATTATTTATGCTAATTTTGTGACTGATTTTTTAATATTTACGTA

## >AhNAC114-Arahy.MGN032.1

TATATATATATATATATATATATTCTTTGGGTTCTGTCTATGTTGACTGTTTAGAAAGAAAGAGCGGTGTTTGGATAAAATCGAAACTGATTGGTGCTGGCATCTTGGGTTGGAACTAACTAACCAGGCTTGTTTCGTTGTGCAATAACCAATAAGTGAATAGTTTTCGCAAAAGCCAAATGAATAATATAAACCATGGTTAATATAATGTCAAACCAAAAGGGAGGTTGAGTCTGGCTTACGCCAAATGTGTCGTCTTATTCTTCCCCCTCCTTCCTTGCTTGCTTGCTTCTTGTCAGACCCATTACTATAAAAGAAGGCACACTTCACCCGTTTCCATGCCCTTTTGCTTTCTTTATCCTTTCCTTCCAATCTAATAATAATAATAATAATAATAATAATAATAATATCAATAGATTAAAATTATTTTTAATATGAAGATGAATATAATATTTTAATTAAATATTAATATTTAAAATGTATTACATAATTATAAACATATAAAAAATATATTCAAATACATATTTTTATATTAAATAAAATGTTAACATATATTCAACATGTATCCTACTTAATATAAAATGTATCTGCGAATTAACTTTTTACCTATAAAATCCATTTATATGTAATTATACTAAATAATTTTATTTTAAAAAAAAATATTTCTTTCATATAATAAAAATAGTTAATACTAGCCTTTTTTTTTTGTTTTAATTTTTTCTTTTCTACATCTTTCTCTCTTCCTTTTCCTGGCCCTCCTTTTGTGCTTTTGTTATATTCTTCCTCTTTTCTTCTTTTGCTTGTGCTCTCTCAACTTTGATGGGCTAACATTGGGAATTGGAGGCTGCACGAGTGCCTTCAGTCTCTTCGCTGTTTGTCACCCAAGCGGACAAACATAAATACATAGTCTTACCACTGACCATTGTGGGGAGCATAGAACAGAAAAGGATGGAGGGGAGAGGGAGTAGTTTTGTGAAGAATGGGGAGCTGAGATTGCCTCCAGGATTCCGGTTTCACCCGACGGATGAAGAGCTGGTGGTTCAATACTTAAAGCGCAAGGTCTTCTCCTGCCCGTTGCCGGCCTCTTTCATTCCTGAGGTTGATATTTGCAAGTCCGATCCATGGGATTTACCAGGTGATTTGGAGCAAGAGAGGTACTTCTTCAGCACGAGGGAGGCCAAATACCCCAACGGGAACCGATCCAACAGAGCCACCAACTCGGGCTACTGGAAAGCCACGGGCTTGGACAAACACATCGCAACTTCAAAAGGCCACCAACTTATTGGCATGAAGAAGACTCTCGTCTTTTACAGAGGCAAGCCTCCTTATGGATCAAGAACAGATTGGATCATGCACGAGTATCGCCTTGTCTCCCACCCCCACCTGCTTCCCATGCAAAATTGGGTTCTCTGTCGCATATTCTTCAAGAGGAGAGCACCTGCTACTGCTAAGAATGTTCTACTGGATCACAATTCCCATTCGGCATCAGCATCAGAAGAGGCCTTCACCATCAGCCATGAGGGCAGCAACTCTAAGGTGGTTTTCTACGATTTCTTGGCACAGAACAGGGCTGATTTGAACCGCGTGACCCCTCCTGCTTCTTCGACCTCTGGCACCAGTGGAATCACCACCGAATCCGATGAGCATGAAGACAGCAGTAGCTGCAACAACTTTCCTTTTTTCAGTTGGTACTAG

## >AhNAC115-Arahy.MI72XM.1

TCTCTATTTTCAAGTAAAGCTGACCTCTAAATTCCATATCAAAATTCTTTAGCGTTTGGAAGGAATATTTGTATTTCAATTTCATGGCAATTTACCTAGGCGAATGGCGAACAATGAGACCCCTCGCACAAGAAACAGAAATTAATAAAAAAGAAAAAGGAAAAACCCCAATTAAAATCCACATATATAAACAAAACCCTGGCCGCTTCTCACTCTCTTCTTAGTCCAAAGATTCAACGATTAGGAGTGCGAAGTTTCAAGTCTGTGTTCTTCTCACTTCCCTCTTTCCTTCCCTGGGTTCCACTGTGCCGATGAATTTTGTTCTCAGAAAGGGACCGTCTTCATCCATTGTGGGTTTCCAGTTTCATTTTACCTTCATGAACCGGGGAAGGCTTCGCTGTTCTCGCAATTTCTTTCGCCACTGGACTTGACGCTTCGCACCCTCGATTCTTTTAGGGTTATTTAATTCCGTTCATCATTCTCTAGTTAAATTTCTCGTCTTTTTTATTTTTTATTTTTATTTTTATTTTTAATTGGGAGTTCCTACGAAGTTGGTTTCTTTCTTCACTGCTGGTGAAGTGGTTCTTTGTTGTTGGATTGTTTGATTCAAGGGAAGTTGTTAGATTCTGAAGGTTTTACTATTTAAGCTTTCTTTATTCTTCTGCGATGGCCGCAATGAAGTCAATTCCAGGGTACCGGTTTCATCCAACCGATGTTGAGCTGGTTCAGTACTTTCTGAAAAGGAAGGTGATGGGGAAGAGATTCCCTTGTGATGTGATTGCTGAACTTGATATATACAAATATCCGCCGTGGGATCTACCAGATCATTCTTTGCTTAAAACTGGAGATTTGGAATGGTACTTCTTTTGCCCTCGAGGGAAGAAGTATTCGAGCGGAGGGAGGATGAATAGGGCCACAGAATGTGGGTACTGGAAGACTACTGGCAAAGATAGATCCGTTGAGAACAAGAAGCTTGTTGTGGGCATGATAAAGACTCTGGTGTTTCACATGGGTAAAGCACCCAAGGGAGATCGAACTGATTGGGTTTTGCATGAATACCGACTTCAAGATAAGGACCTTGCTGATAAGGGTGTTCAACAGGATTCTTATGTGATATGTAAAGTGTTTCAAAAGGATGGTCCTGGTCCTAGGAATGGTGCACAATATGGAAGGCCGTTTAATGAGGAAGACTGGGATAAAGAGGATGAAATTGACTGTGTAGAATCTGCACCTGTTGCTGCTCTACCTGCTGCAGTTCCTATACAACCCGCTTCATGTCATAGCTCTGTTGTGAATAACGTGAATCTCTCTGTGAGTGAATGCTATGGGTTGACCTCTGTTTCGTGTTTAACAGGGCCAATGCCTTCTTGCTCAGCACATCCTTCAGCTCCAAGTAATCAAGTTGATGGTGACATTACACCAGTGCCTGGTTCCTCCATAGAAGATAACATAATGGCTCCTACTCAGAACACCACAACTGAAAAGGTTGACAATCCTCCTGACATAAACAATGCTGAAGGAACACCTTGCTTTGATCCCAATGAGATTTTTGGGGGTCTGGGTGACCTTGATGGTTTGTTCGAAATGGGTGGAATTGGACATGGTTTTTCCTGCGGCCAAAATGGTGGATATACTGTGAATGAAATGCTTTCTGCGGGTGATGGGTTGCGTTTCCCTGATCCCCTGGACTACTTGGAGTTGGGTGACCTCGACACTCCATTGTTATGGGAGACTAATGAACAAGGAAATTGGAGCCAGGACAATAAGTGAGGCTTCAAAATTTGACGAAGCATCAACATGAAGTTGGTGGAAGAACCATTGCCGTGTGTTGCTGATCTTGATTGTCTTTCCATTCTCATTTATAAACTCTACTGAAGCATATATTCTGTAGAAACATTCCTTCCTGTAATAAGCTAACACATGATAGCAGTTGATAAATCTTTAGTGTTAGTTCTATTTACTAGGTTGGATTAATCATTAGCCTCACAAATTTTCTTCTGATATTTTCTAAGTTGTGTTTGATAGAGTGAGTTTTCAACATCTATTACGTTGAAATCTATTCATGTCTTTCGTAGTAATGTCAAATGATGGAAGTTGACGCATTTAACCCTAAACTTCTAGTATTCTCAATTTTGTTTTATTGAAGGGTTAGTTGATGAGGGAACTGTGATAGGCCTTTGCCCCTTTACTTTTCGTCGCGTTGGAAACTGGAAAGTACGTTATCTAAACCAGTATAGCTTGATCTTGCAAGGTCTTTTCGTGATTTATATAGAGATCGACTAAATTCCATTATTAGAAAGATTGTGATAACTAGTAGATTTAGAATTGAACATAAAACTAGCACTATGA

## >AhNAC116-Arahy.N4TQEE.1

ATATTGCTGGTAATTAACTTTAGTGTGTGTATATATGTGTCTTAAAACCAAAGTAGTTAGCTTTTGTTGTTAGTTTCCTTGGCCGCCTTTAATTAACCTTGTGATATTATGACATGTTCCAAGTACCGCCGCCTTTATAATTTCCAGCAATCTATCCATCCATTATTATAATTGTGAAAAATAATCAACCCGAATCTTTATCTCTCTACTCAGCATATATATAGTGACAAAATAAATGGATTATGGAAGTGTAGTTATTACTATACCAAACTCAACAATTCATACATACACCACCAATTCTATGATGATGATGACTGCAGATTATGAAAGCGTGAAGCAGCTTCCTCCTGGGTTTTTGTTCTCTCCAACGGATGAAGAACTTGTCCTTCACTTTCTCTATGCCAAGGCTTCTCTTTTGCCATGCCATCCCAACATCATCCCTGATCTTGATGTCTCTCTCGCTCATCCTTCCCAACTCAACGGTAAAGCGTTGTCAAGCGGAAATCAATACTATTTCTTCAGCAAAGTGAAGGAAAAAAGAATAACAGAAAATGGGTATTGGAAGGAAATAGGTGAAAGTGAAGCAATATTGTCATCAACGTTTGAGAAGAAAGTAGGGACAAAGAAGAACCTTGTATTCCACATAGGAGAAGCTCCACACGGCATTGAAACCAGTTGGGTCATGCAAGAATATCATATTTGCCCATCCTCTAACATTATTTCTACAACTAGAGCCAGAAGAAAACACGATCATCAAATTTGGAGCAAATGGGTTTTGTGCAAAGTGTATGAAAAGAAGGGGTCCGTACGAGGTGTAAACTACTGTAGCGACGATGATGACAGTGGGACAGAGCTATCTTGGCTTGACGAAATTTATCTCTCGTTGGATGATGATCTGGAAGAAATTAGCGTCTCCATTTTAGATTGAATATGTATCAATTACCTGAATTTTAGGTTTAATATATATGTAACGGTGCCGCTATATATGTTGATACTACTGTATATTGAATTGGGAGTTCAATGAAAATGATTATACCTTCACTAAATATTTTAAATGGAAGACAATTGAACAATAAATTCTAAAATTTGGTTCAATAAAATAAAAATACATTGCATTTTATTTTATCCACCTAAATAAAATACTTTAATATACTATTT

## >AhNAC117-Arahy.NB8KRW.1

ATGGGAGTTCCAGAGAAGGATCCTCTCTCTCAATTGAGCTTACCTCCTGGTTTTAGATTTTATCCCACAGATGAGGAGCTTCTTGTTCAGTACCTATGTCGCAAGGTTGCTGGCAACCATTTCTCACTTCCTATCATCGCGGAAATCGATTTGTATAAATTCGACCCTTGGATCCTCCCAGGTATATATTTGATAAGGGATAATGACATTATTTCTGTGATTGAGAGCAAGATGTTTATTTATTTGGTTTGTTTTTGTGCAGGTAAAGCAATATTTGGGGAGAAAGAATGGTACTTTTTCAGCCCCAGGGATAGAAAGTATCCGAACGGTTCGCGACCGAACAGGGTTGCTGGCTCTGGGTACTGGAAAGCCACAGGAACAGATAAAGTAATCACTACCGAAGGCAGAAAAGTTGGAATCAAGAAAGCACTTGTTTTCTACATTGGCAAAGCACCCAAAGGCACCAAAACAAACTGGATCATGCACGAGTACCGTCTCCTCAACGGTTCTCAAAAGAGCCTCGGCAGCACCAAGCTAGATGATTGGGTTTTGTGTCGGATATACAAGAAGAACTTGAGCTCATCGCAAAAAGTCAATATGCCAAGCTTTACGAGCAAAGAATGGAGCAATGGATCGTCGCCTTCTTCATCGTCTCACATCGACGACATGCTCGAATTGCCGGAGATCGACGACCGGTGCTTCGCCTTACCGCGGGTTAACTCACTGCAGCACGAAGAAAAGCTCACCCTTGGCGGCACAGGCAATAATTTCCCGGACTGGGTCAACTCGGGGGGTCTCGACTCGGTCCCTGAGTTTGGGAGCCAATCTCAGGGGATGACAAGTTACGATGGAAATGACCTATATGTCCCCTCCGCGTCACAGTTCTGCCACGTCAACACAATGGTTGTACCGGGTAACCCGACGGAGGAGGAAGTCCAGAGCGGCATCAGGACCCAGCGGATTGATGAGAATTTCGGGTTATTTCAACAGAATTCGAATGTATTCACCCACCGGTATTTGTCGAGTTCGGGTGACTCATTCGGATTCGGATACCCGAATCAGCAATTTGGATTCGGATTCAGAGAATGAAAAGATGTCATGCATTGTCATTGTCAAGGCCTTGTGGTTTTAGTGTAGGAAAGGGCAATTGTGTAAATAGCTGTTGGATTCTTTGGCTAACACTATATTATATGTGTAGTGGTAATTTGGTTTAATAGATTTTTCAAAAGTGGCAAAAGTGGGGAAAAAAATGCAGTTTTGATAAGTGCTAGGCACATTCGATGGTTCGATGTGAAACTAAGCAGAATAAACATAGTTCAAGTAATTGTGTGAAAGCATTCATAATTCTGAAAGTTTTTTAGAAATGTATATGCATTATTTGGCTACAATAGCTACCCAGGTTCACTCATCAGCAACCTGAATGAAAATGTACTAAAAAGGCATTAGCAATGTTTGGTTTAAGATATTTAAATTTTCTTCTCTTTTTTCGGATGTTATTTAATATTTATCATTTGTTCGTTAAGCGTAACGTTCATGCCTTCGTGACCACAACCTTATTGACAGCAAACAATGAGATATTGATATTATCTCAACATTATAATTCAAGTTGGTAAATATAATATCGCCCTCGTATTAATTGTCTATTTTAA

## >AhNAC118-Arahy.NLE5K3.1

GAGCAAAACAAAAAGCTCTCATCTTTGTATATTTAAATCTCAAAAACATATGATAATTGCTAATAATTATGAATGCACATACATATATAAATACACTCCAATTAGGTGGGGGCCACCCCAGCTCAATTGATCTGAGACCAAACCCATACCAATGCATGACACGTGGAAAACAGCAGCTTGATGATATCAGCATGAAGTGTGATGATGATCCATATACACAATAAAAATCACCTTATTCATCACCAATGTATTAACTAAGTCCTGTTAGATCATAAATCCTTGTCAAGATAATATGATCTGCTTTCACTCCTAATGAAATTGTTTCAATCTGGTGTCACACACTCCTTCCAAGTATTCTGTTTATAAATTATAATGAAAAATATTTGGTTGATGATAAACACCAGCTTGGGTTTCACGTCAACACCCTTATTATAAATACCTTAGCTTCTCTATTTGTACTATAAATACCACTCATCTTCACCATTACTCTAACTTGACACATTCCCTCCTCTTGTTTTCTCTGCCTATCTTCTTAATGGGAGATAACAATGTGAACCTTCCACCGGGGTTTCGATTTTATCCAACAGATGAAGAGCTTGTGGTCCATTTTCTTCATAGAAAGGCAGCACTCTTACCTTGCCACCCTGATGTCATCCCTGATCTTGATCTCTATCCTTATGATCCTTGGGAACTTGATGGTAGAGCGTTGGCAGAGGGAAATCAATGGTACTACTACAGCAGGAGAACACAGAGTAGGGTGACTGAGAATGGATATTGGAAAGCAACGGGAATGGAAGAACCAGTGATGACAAGCTCAACCAACAAGAGAGTTGGCATCAAGAAATACTTTGTTTTTCATCTTGGTGAATCCCCTTCTGCTATCAAAACAAATTGGATAATGCAAGAATATTGCCTTTCCGATTATTCTGCTTCCTCTAGCAGATCCTCCAAAAGAAAATCAGATTATAGTAAATGGGTGATATGTCGTGTTTATGAGCGCAATGGAGATGATGATGATGGAACGGAGCTGTCTTGTTTGGATGAAGTTTTCTTGTCACTCGATGATCTTGATGAAATAAGCTTACCAAATTAAATTAATCAAGCTAGCTAGCTGCATTAATTAATATAATCCAAAATGGATATAATTATTAAGTATATAGCAGTGTGATGTTGCAAGTATAGGTAGATTTAGATTTCTATAGCAGCCCTTTAACACAAGTGGAAGTGGGGCACTATGTGAAATTCATGACCAAACAATATGTACTATGACGTTATGCTTTCAAAGATTCCATTTATCATTTTACCATTTTCTCTGCTCTTTTCTCCATATAAACACACGTGGATAATTGCCCTCCCATTCTCTCAA

## >AhNAC119-Arahy.Q3Y7SF.1

TGTATTGTTGTATGTATATACTAAGGTGTATAAAGACTATATATGCATGAGATATAAATAGAGAAGAGAATAATAATAATATTCATTCACTGATCCATGATCATGGTGGATAATAGCACAGATTCATCATCAGGAGCGGGTGATCAGCATCATCACCCTCAGCTTCCTCCAGGCTTTCGATTCCACCCCACAGACGAAGAACTCGTCGTTCACTACCTTAAAAAGAAAGCTTCTTCTTCACCACTCCCTGTCGCCATCATCGCCGACGTTGATCTCTATAAGTTCGATCCATGGGAGCTCCCAAGTAAGGCAGCGTTTGGGGATCAAGAGTGGTACTTTTTCAGTCCTCGGGATAGGAAGTACCCGAATGGAGCTCGGCCAAACAGGGCGGCTACTTCTGGGTATTGGAAAGCCACCGGGACGGATAAGCCTATTCTCTCTTCTGATGGGAACAAGCAGAAAGTTGGAGTCAAGAAAGCGCTTGTTTTTTATGGTGGCAAGCCCCCCAAAGGTGTCAAAACCAATTGGATTATGCATGAGTATAGGCTCACCGATAACAATAACAATGCTTCTTCTTCTATTTCATCTAAGCCTCCTTCTATCCCTCTTGATCCACTCAAGAAGACTTCTCTCAGGCTTGATGATTGGGTTTTGTGCCGAATATACAAGAAGAGCAACAGCAGTAGTAGCAGCCTTCCAATTCCAAGGCCAGCGTTTTTAATGGATGAAGAGAAGGATCTAATTTCCATGGAGAACAGCATGGTGCCAACTATGTCAATGTCAAAACCAAGAAGCACTTCAACAACAGGTTGTTATGGACCCATGGCACTTGAAAACGATGACAACTTCTTCGATGGTATATTGGCAGCATCAACCGATCATCACACCATGCAAAATGGGTCTCCAGGGTCCTCATCTTCAAGCAAGAGATTCCATGGTGATCTTAATAATGGAGACAACACCTCCTTCGTTTCTCTTCTTAACCAGCTTCCTCACAACACACCGTTTCACCCAAACTCCATTCTTGGCTCCGTGGGAGACGCTGTCTTGAGGCAACAATTTCAACTTCCAGGCTTAAATTGGAACTAATTAATTAATTAATAATAATAATATTTTGCGTTGCTTTGGTGGGGGTGTTATAATTATATTATATTAATAGGTCTCTTGATCCATATTGTGTAGCTTAGTGCCTTAGTATATATGTAGTTATTCACTTTTGAATGTAATAACCATAGCATACACAAATTAAAGAACATACAAGAGTTTTCATGATCATAACTATAGGCCTATATTTTGAAACTGAAGCAAGCTTACCCTAGATGAAATTTCATACCATCGCATCATAAATGAATTCGGTTACACATGTTCATGGAGCATCTAATTTCTTTTCTGTGTTTTCTATATTTATATATAATCAAAGCCACACGTGAAGAACTTATGAATAGTATTATAATATATATTTTTTATTATTGTTTTTGCCTTTAATTTGTTTCAATGGTAATGGTGATTTACCACCATTCCTTAGGATGAAAATTCTGGTAAGGTCGTAACCACCTTCCTCTTTTTGGCATTTATGACTTGAAGAAAAAGTGTGAACTGGGAAACAAAAAATAACGAAACACCGCCGATAAAGTTAGGCAGAAAATTTTGGCCTTTTACTTGTCTCACTAAAACTAAATGGTGTCATTTAAGTTACCTACTGTATATTTGTGTATAAATATATTATTTAAATTATTTTAAATATATATTTTATACTAACGACTAAT

## >AhNAC120-Arahy.QDSH2R.1

AGCAACGCACAATAATTTGACCTTAGCTTAAATGATCAATTACATTACATATATAGCAGCTAACTTGTATTGTTAGCTTCAATGGCCGTCTATCTTTGTTGTTTGGCCCTACTTAGTTCCTTATGATGGAATTCCACGTACAGCCGTCTTTCTTCAAACAGCTCAACGCTTTTTTTGAATCCTAATCAATCCAACCATAAATCTCTCTCTCTCTGTATGTGTATATATATATATATATATATAGCATGACAAACTTGACCCAAATTAATAAAGTGCCAAAGTCCAAAAAGGAAGAAAGAAGAAATTAATACAATGACGAACCTTCCTCCTGGGTTTTGCTTCTCTCCAACAGATGAAGAACTCATCCTTCACTTTCTTTATTCCAGAATTTCTCTACCATTCCATCCCAGCATCATACCGGACCTTGATCCCTCTCAACTTCATCCATGTCAATTAAATGGTAAGGCGTTTTCAAGTGGGAATCAACACTATTTCTTCACCAATAAAGTGAAGGAAAACAGAAGCACAGAAAATGGGTATTGGAAGGAAATAGGTTTGAGCGAACCTATAATCTCAGCTGATGCAAACAAGAAATTGGGAATCAAGAAGTATTTTGTCTTCAATCTTAACGAAGGCACAGAAACCAATTGGGTCATGCAGGAATACCATATTTCCTCATCTATGTTCCACAACCCTATTTCGTGTTATGCAAATGGAACTGCTCATCGAAGATTATTAAAACCTGATCAAAATCAGAACAATAAATGGGTTTTGTGCAGAGTGTATGAGAAGAACAAGTCACAATCACAACAAGGTGCAACTGCAAACTCCTACTATAGCGACGAGGATGATTGTGGATCAGAACTTTCATATCTAGATGAGGTTTATCTGTCACTTGATGATGATCTTGAAGTCATAAGCCCCCCTAATTAATTAGAGTGATGCAATTTAATAATAGTGTATATTATTATAAGATATATATAAGATAATGTTACATGATCTAGCGTGCTAGAATTCCTGATAATGTTACACATGTATCTTGAGTATAGCAGATCTATTAGCTAGGGACATAACACTACTATATTAGTTACATATACACATGCAGGACCCCAAAGTTGAGTAATTAATTAGGTTCAAAGTTTTATTGTCCTTATACATACACCATATACATATATATATATATAGAGAGAGAGAGAGAGAATATGTATATTTTTGCTTCCATAGATTATATATATATA

## >AhNAC121-Arahy.QL5RCW.1

AACTTGACGTCCAAGTAACCTTTCCTTATATCTAGAACCGCTTCCTTTGGTTCCCACCTTTTCTTTTTATTTCATCTATCGCTTATACCTTTTGTCCTCTTCTTTATCTGATCTTCCCTCTTTCTCTCTCTATCAGTGATCACCAATATCATCAAGCACATAAGAACGAATTCAAGACGGTGGCCATGGTAATCATAAGCTAAGGGAGATAGAACGTGTCCCAAAAGAGTACAAATAAAGTACTACTATGCTTTCTACCTTATTTTACCTTTCTCTTAACTATATACTTAACTTGTAATTTTTTCTCCTGTTCAATTTTTGGGGGTTTTTTGTTTTTACAATTCTAAGTATAAGGGAAGAAAAATTAGAGGGTGGTCTCATTTATTGTGATGGCATGGTGCAATGACACTCATGAGAAAGAGATAATTGCTTCCAATAATAGTACTATTACTCTTAGACCTAAATCCGACCCAAACGTAACTTGCCCCTCATGTGGCCATAACATTCAAATAATCCAAGAGCAGGGTGGAATTCATGAGTTGCCGGGGTTACCAGCTGGAGTGAAGTTTGACCCAAATGACATTGAAATATTGGAGCATTTGGAGGCAAAAGTTATGTCTCATGTGCCCAACCTTCATCCTCTCATTGATGAGTTCATACCAACACTTCAAGACGAGAATGGCATCTGTTATACACACCCAGAGAAGCTACCAGGAGTAAAGAAAGATGGGCAGATCCGGCACTTCTTCCACAGGCCTTCAAAAGCATACACAACAGGAACAAGGAAGAGAAGAAAGGTTCACACCGATGAAGATGGAAGCGAAACAAGGTGGCACAAAACCGGAAAAACAAGACCGGTGGTGGCCGGCGGCGGCCTAGTGAAGGGTTTCAAGAAGATTCTAGTACTATACACCAACTATGGGAGGCAAAAAAAGCCTGAGAAAACTAACTGGGTGATGCATCAATACCATCTTGGAAGCAATGAAGAAGAGAGAGATGGAGAACTAGTAGTTTCAAAAGTGTTCTACCAAACACAACCTAGACAATGCGGCAATTCCATTGTTATAAAGGAAGATAATGATGATGATCTTCCCTATGGAAAGATATTGATGATGAATAACAGTAGTAAGAAGCACAAAAATAATAACGATAGAAATGTTGCTGCTCCTGTTGTGGACTACTACATCAACTATGACCATGTTGATCATCATAATCACAATCATAATAGTCAAAGATGTTCATCACCTACTCAACTTATTCCAAACTTGGTTCTCCAAGGTGATTCCTCTTCTCTTTTTCGCTTTGCTTCATCATCACTCGATGGGAATACCAACAAAACAAGACTTTTTGAGAGAAAGTTGTAGTAATTCGTTCATTATTATTATTTCTTTCACTAAGTGACAATATTTATTCTATACATATATGATATATGGGATTAATATAATTAGAGTGACATGTAATTAAGTTGATTGGCTTTGATGTGAATTAAGGACTCACTCATATAATCAAAGATTGGTTTTTATTATGTTAATTTTAGTGCTTAGGTACATTCAAATATTGTGGTATAAATAGTAGGGTTATTATTGTCCATTCATAACTTTGTTAAATAATGAATTAAAATTTT

## >AhNAC122-Arahy.QVEY1G.1

ATGTGGTATGCAGAGGTGGCAAAGCTAAACGCAAATGAATGGTACTTCTTCAGCTTCAGAGACCGGAAATATGCGACGGGGTTCAGAACGAACAGAGCGACGACAAGTGGTTATTGGAAAGCAACGGGGAAGGATCGAACGGTGCAGGATCCACTGACACAAGAGGTTGTAGGGATGCGCAAGACTCTGGTGTTCTACCGGAACAGAGCTCCCAATGGCATCAAAACTGGCTGGATCATGCATGAGTTTCGCTTGGAGACCCCACACATGCCCCCTAAGGAAGATTGGGTGTTGTGTAGAGTGTTCCACAAAAGCAAAGAAGAAAACAGTGCCAAACTTATCATGTATGATTCCATTTCCACACATCATCAATCATCAAACCCTATGGCATTGGTATCAACCCATCATTTGAACCCCATCAACAACCATAATAACACTTATCATGCCATGAATAATTTCCTTCATCACTTCTCATCATCAAGGGATGATAGTCAAACAAATAACGCCAATAATAATAATAGCAGTGTTACTCAAATTAGTCCCAAGGGTTATGATGGATATGGCTTCATATGGGATCACATGGATCTTGAAGATGGTGGTGTGCCCTCATCAGACTTCCAGGTCGACAATAATAATAATCGTAACAACAACAATAATAATAATATTGCATTGCTATAA

## >AhNAC123-Arahy.QZZL54.1

AGGAAGGAAAAGAGTTCAAGAAACCAAAAACGTTTTAGCATAATGGGAGTTCCAGAGAAGGATCCTCTTTCTCAATTGAGCTTACCTCCTGGTTTTAGATTTTACCCCACAGATGAGGAGCTTCTTGTTCAGTACCTATGTCGCAAGGTTGCTGGCAACCATTTCTCACTTCCTATCATCGCGGAAATCGATTTGTATAAATTCGACCCTTGGATCCTCCCAGGTATATATTTGATAAGGGATAATGACATTATTTCTGTGATTGAGAGCAAGATGTTTATCTATTTGGGTTCTTTTTGTGCAGGTAAAGCAATATTTGGGGAGAAAGAATGGTACTTTTTCAGCCCCAGGGATAGAAAGTATCCGAACGGTTCGCGACCGAACAGGGTTGCTGGCTCTGGGTACTGGAAAGCCACAGGAACAGATAAAGTAATCACTACCGAAGGCAGAAAAGTTGGAATCAAGAAAGCACTTGTTTTCTACATTGGCAAAGCACCCAAAGGCACCAAAACAAACTGGATCATGCACGAGTACCGTCTCCTCAACGGTTCTCAAAAGAGCCTCGGCAGCACCAAGCTAGATGATTGGGTTTTGTGTCGGATATACAAGAAGAACTTGAGCTCATCGCAAAATGTCAATATGCCAAGCTTTACGAGCAAAGAATGGAGCAATGGATCGTCTCCTTCTTCATCGTCTCACATCGACGACATGCTCGAATTGCCGGAGATCGACGACCGGTGCTTCGCCTTACCGCGGGTTAACTCGCTGCAGCACGAGGAAAAGCTCACCCTTGGCGCCACAGGCAATAATTTCCCGGACTGGGTCAACTCGGGGGGTCTCGACTCGGTCCCTGAGTTCGGGAGCCAAACTCAGGGGATGACAAGTTACGATGGAAATGACCTATATGTCCCCTCCGCGTCACAGTTCTGCCACGTCAACACAATGGTTGTGCCGGGTAACCCGACGGAGGAGGAAGTCCAGAGCGGCATCAGGACCCAGCGGATTGATGAGAATTTCGGGTTATTTCAACAGAATTCGAATGTATTCACCCACCGGTATTTGTCGAGTTCGGGTGACTCATTCGGATTCGGATACCCGAATCAGCAATTTGGATTCGGATTCAGAGAATGAAAAGATGTCATGCATTGTCATTGTCAAGGCCTTGTGGTTTTAGTGTAGGAAAGGGCAATTGTGTAAATAGCTGTTGGATTCTTTGGCTAACACTATATTATATATGTAGTGGTAATTTGGTTTAATAGATTTTTCAAAAGTGGCAAAGGTGGGAAAAATATGCAGTTTTGATAAATGCTAGGCACATTCGATGGTTCGATGTGAAACTAAGCAGAATAAACATAGTTCAAGTAATTGTGTGAAAGCATTCATAATTCTGAAAGTTTTTTAGGAATGTATATGCATTATTTGGCTGCAATAGCTACCCAGGTTCACTCATCAGCAACCTGAATGAAAATGTACTAAAAAGGCATTAGCAATGTTTGGTTTAAGATATTTAAATTTTCTTCTCTTTTTTCGGATGTTATTTAATATTTATCATTTGTTCGTTAAGCGTAACGTTCATGCCTTCGTGACCACAACCTTATCGACAGCAAACAATGTGATATTTTTCAAGTCCTTCAAATCTAAGTCCATTTGGATTTTTCTTTAAAATTTGCAGATTTT

## >AhNAC124-Arahy.R9WKT4.1

ATGAATACAAAGATTGAACTGCCACCAGGTTTCAGGTTTCATCCAACAGATGAAGAGCTCATAACTCACTACCTCTCTCAGAAGGTTGTTGGTAGCTGCTTCTATGCAACTGCCATTATTGGAGAGGCTGATTTCAACAAGTGTGAGCCTTGGGATTTACCTTGTCAGACAAATAGAGCCACTGGCGCTGGGTACTGGAAGGCCACAGGGAAAGACAGAGAGATATACAATGCAAAAGCAAAAGCACTTATTGGGATGAAGAAAACACTTGTTTTCTACAAAGGAAGAGCTCCAAATGGTGAAAAGACAAATTGGGTCATGCATGAATATAGGTTGGAAGGCGATAATAAACCTTCTATATACAATCTACCCAAAGAAACCAAGAAAGAGTGGGCTTTGTGCAGAGTTCTACACAAAAGTGAAAAGAAAGTAATGCATGTTCCACAACCACAGGGATTGGTTGAGTTCAGCTCCTATGAAAATAAGGAACTTCCCCAATTAATGGATTCTTCACAAGTAACATTCTTTTCATCAGACCCAAATAATCAAAGTGAGGATCCAAATCCAATCACACGTGATGATGATAATAATAATGATGACATCATATTTGATAGCATTGAAACTCCTTTCTTGGAACAACAACCACCTTATTCTTCATCCTATGATTCTTCAGATTTAGACACCCTTAACCCTGCCACATGGGATATTTCCGAAAATGCCCCTACAAGTAATGCGTCTAAGGAGACGGACTTTGATGCTGACATGTTCTCTTTGATGTACAACAATAGAGAAGTGTTCCAAACATCATTTGAGAATCAGGAATATTATGCATATGATTCTGTAGGACATGTGGACAATGGTTCCCTATGGAATTTTTAGAGAATTAGGATTATGATTAGAATTGGTTTAATTTTTCTAATAATTTCAACTTATTAAGGTTAACTCAGTTGATTAGGATAACATAGTTTAGCTTCCTTCTTCTGTCTTTAGATCTTGGGCTTGAACCCATTTGTAATAAAAAGAAAAAAAATATATAT

## >AhNAC125-Arahy.RC5QY0.1

TCCCACTCTGATCTGATCCTGCCATTAATTTCATTGATTACAAAGCAAGGCAAAAAGCATATGGGTAAGGGTAGGTCTACACTACTACTTCACTCCATGAGCAGCAGCTGGTTTCGTTTTCTTGCTTTAATGGGGGATAGCAATGTCAATCTTCCACCGGGGTTTCGATTTTATCCCACTGATGAAGAGCTTGTAGTCCATTTCCTTCAGAGAAAAGCAGCACTTCTACCTTGCCACCCTGATGTCATTCCTGATCTTGATCTCTACTCTTTTGATCCATGGGAACTTGATGGTAGAGCTTTGGCAGAGGGGAACCAATGGTACTATTACAGCAGAAGGACACAAAATAGGGTCACTGCCAATGGTTATTGGAATCCAATGGGAATTGAAGAGGCAGTGGTTTCAAACTCAAGCAACAGGAGAGTTGGTATCAAGAAATTTTATGTGTTCTATGTTGGAGAAGCCCCTCATGGTAACAGAACCAATTGGATCATGCAAGAGTATCGTCTTTCAGATTCTGCAGCATCCTCTAGCAGATCATCAACCAAAAGAAAATCACAACCAAAAACAGATCATAGTAAATGGGTGGTATGTAGAGTCTATGAAAGTGATGAAAATGATGATGATGGTGATGGTGATGGAACAGAACTCTCTTGTTTGGATGAAGTTTTCTTGTCATTGGATGATCTTGATGAAGTAAGCTTGCCAAATTAGATGATGCAAAACTTTTGTTAGCAGCATTATTATCCCAAATGGGGATAATTAATTAAGGCTAAATAGCTGTTTAATGCAAATTTGTTAGTACTTAATGTAGTTAAGTTTGTTTTTTTGTATTTAGCTTTAGCAGTGTAGCTGCAGAAATTGCAATCAAAAAGTGTTTTAAACTTTTAATTAAGTTGCCAGAGCTAGAAGATTTTCTATAGCAACCCTTGAAGTTGGGGCACTAGCTAGGTGAAATTTATGGCCAAGCAATATGCATTGTGACGTTATGCTTTCAAATATACCATTGAAACTTACTTGCTTACTTACTTTATTTTGATGCTGACACGTGGTTAATTACATGCCGAGTAATCAAAAAGTGTCCATTGTCAACCACTAATATCAGTAGTAATTATTTGATCTGTAATTAAAATTCCATAAGATATGCAAAAGCAAGAGGGCCGTCTCATTTCAATGCAAAATTCTACTAGGAATAGGAATTTCCTGGTTCAATGTAGTGTAACATGATGAAATCCAAACCAAATCCAATTGAAATTCTCACGTGCAAGGATTGTTGAGTGAATTAAGATTTGAATTCATAGAATCCAAGTTGAAACAGAGAGGAAGGTTGTGGAGCCCATTCAGCACTGTAATGGAAGGTTGCAAGTGAACATAGCATATAGATCACAATAACATTGATGGTATTTGGTAGCTAGTTGTCTTGTGTCGCAATGCTAAGGACATTTCATTTATTTTTGGTTTTTTTTTTTTTTTGTTATTGCATACTTGGATTCCTGCTTAGGAAAGCATCAAGTCATGTGTGATGCAAGCATTCGCATGCTTTCAAGCACCAACACTAGCACTATATTTAATAATAATAATAATAATAATAATAATATGCATATATTATGA

## >AhNAC126-Arahy.RU4C7B.1

TCACTCTCGTCTCTCTCTCTCTCTCTCTCTCTACTCTCTTAACCATTATTGCTCTATGGCTCCAATGAGTCTCCCACCTGGTTTTAGGTTTCACCCCACAGATGAAGAGCTCGTTGCTTACTACTTAGAAAGGAAGATAACAGGTCGCTCTATAGAGCTTGACATTATAGCTGAAGTTGATTTATACAAATGTGAACCATGGGATTTGCCAGATAAGTCATTTCTACCAAGCAAGGATATGGAGTGGTATTTCTACAGTCCAAGGGATAGGAAGTATCCAAATGGATCAAGAACGAACAGGGCAACAAGAGGTGGGTACTGGAAAGCGACTGGAAAGGACAGGGCAGTGCAGTCTCAGAAGAAGGCAGTTGGTATGAAGAAGACTTTGGTGTATTACAAAGGAAGAGCTCCACATGGAATTAGAACCAACTGGGTCATGCATGAGTACCGCTTGATTGAATCCCTCCCTGGTACTCCTCACTCCTCTTTCAAGGATTCCTTTTCATTGTGTCGGATTTTCAAAAAGACAATTCAAGTTCAAGACAAATCTAAAGAAGAGAAAGAACATCAAGCATTACTAGAGGAAGATCACTCAAGTGGCATTGAGATTTCAAGAGAAATGGAAGCCATGAATGATAATAATAACAATAATATAACTCTAAATAGTAATGAACAATATCCTAATAATAATAATAATAAACTCCCTAATTGTGATGCTTCATCTTCTGATCTCACTCAAGGAACATGTACACCCACAGAAACCGGTATAGCAGATGATTTTCATGCCCAATTTGCTTGTGATGAAGCAAACAGTAGTGCCGCTAATTCTTACTCAATGGGAATAGCATACCCCTCAAACGTTTTTCAGGACATAGAGATGTCTATGTATGGAAGCATGCATAATTATCAATTCCCACAAACGCCTTTGGTGATGGAAGATTTTCCACAAATAGATTTTGCTGAGACAAAGTTATTGAAGCCAGAGGTGACTGAAGATTGCATGTTCTATGATAGATACGGTAGGGATTGTATGAATGGAACACTAGAAGAAATCATCTCATTGTGTTCCTCTCAAGACAACTCTGTGGCTTTGCCCATGCTAGAATGATTATATTACTAACGAAAAAAAAAAAAAACAAAATGACACAAAAAAAAAAAAAAAAAAAACAGTATCAAAATATTCATTTGACACAGAAGAAACTATAGCTACTTGTGTTGGCCAAAATAACCACACACAAAAGGAGACATTTTTTCTAGCAAGAGAGAAGCAGCAGCTACATATATAATGTACTAAAGATACATAGTAGAATATGCAGAGCCAGGGAGGCTGGCATATAGTGCACTTGGTTTCTTTTTCCTTTTTCTTTTTGGGTGTTCATCTTTTTTTGAACGGGGTGTATGTCTATCTACAATATAATTTTTAATATATATATATTTGTGTTCAAATCTCTTG

## >AhNAC127-Arahy.S9FEUH.1

ACAGCACCGCTTATTATTGTATATTTAAAACTTTGTTCTTTTAAGATTATTTCGTGGACTACAATGATTTTGTTGTTATTGGTTCGGCCAAATCATTCTCAATACAACAACTTCAGAGATCCAAGGATTCAGAAAATGATCCGCTGACTGCTGCATGCACAATAATATTCCAGAGTGGGTGGAACTGGAACTAGTTGAATTGAATGGAAATGAAACCCAATATGGATAAAGACGTGCTTTGTTTAGATTCAAAGAATCAGAGATAATCAATCACGTGGCCAATAACCGTTGATTCCAATTATTCTTAAATTTTATGATTAAAATTAAACGTCAAAATTATTAAATAAAACACAAATTAAATATATCTTGTAATGGTCCCCTTCCCCAAATGGATAACGGCAAGGCATTGCCAGATGACTAGGTATAACAATAACAAAGTGTTGTCTATCAATCAATTAAATCCATGCAACTTCACAAAACGACAAAGAGACCCTCACATAAAAGGAACACACATCTCATTGCAAACAGTTCAAACTCCTTAGCTTGTTCAATTTCACATAACAATAATGGAAGGAAGTAGTAGAAGTTGTGAACTACTACCACCAGGGTTTAGATTCCACCCAACAGATGAGGAGCTAATTGTGTATTACCTTTGTAACCAAGCAACATCAAAGCCCTGCCCTGCTTCCATCATCCCTGAAGTTGACATCTACAAATTTGATCCATGGGAATTGCCAGGGAAAGCTGAGTTTGGGGAGAAAGAATGGTACTTCTTTAGCCCAAGGGACAGGAAGTATCCGAATGGGGTTCGGCCGAACCGTGCAACGGTTTCTAGGTATTGGAAGGCCACAGGGACAGACAAGGCTATTTACAGCAAGTGTAAGCATGTTGGTGTCAAGAAGGCCTTGGTTTTCTACAAGGGTAGACCTCCAAAGGGGATCAAGACTGATTGGATCATGCACGAATATCGTCTTCTTCAACAATCTAATCACAACAGCAGGACCACTGGTTCTATGAGACTGGATGACTGCGTCTTGTGTAGGATATATAAGAAAAAACATGCTGCTAAAGCATTGGATCAAGGACAGGAATACCCAACAACAGTTCAAATTAATCTAAATGCATCAACCAACAATGATGATCAGAAGGAGTTGATGATGATGAAGAATCTTCCAAGGACTTGTTCCCTTACTTATCTTTTGGACATGAATTACTTTGGTCCAATCTCACAGCTATTGTCTGATGGATCCTACAACAACTCATCAACCTTTGAAATATGTCAACATAGCAATAGTGTTGACAACATTGGAATAGTGGATCCTCTTGTCAAAACTCAAATGGTTGAAATGGATGATAGCTATTATGCTCAAGATTCAGGCAAGTCCCAAGTGATGAAACAAGGGAATGATTTAAGAGGATATTACTAAGTAATTATTAACAAAAGAAAAAAGAAAAAATGTTATATTGTTTAGATTAGACGTGATGAATTATGATAAGAGTCTTCTATAAGATAATCATGAAGATTTTAAATATGTCAAATGTGTCCTATCACAGAAATGAAATTTTCAAATAGAATTTAGTATTCTAGTTCACGTTGACATTGTTCACCTTATCTGTACATAATAAGGACAAGACTATTACTGGTTCTTAATTGAATTTTCGGACTTATGAAAATAGTTTAAAAGATGCCTGGATCACGCCTTCTTAAGA

## >AhNAC128-Arahy.SJ3Y3C.1

ATGGAATCATCGTGTGTCCCACCTGGGTTTCGCTTCCACCCAACGGATGAAGAGCTTGTTGGTTATTATCTGAGGAAGAAAGTGGCATCTCAGAAGATAGACCTTGACGTTATCAGAGAGATCGATCTCTATCGTATTGAACCCTGGGATCTCCAAGAGAGATGTAGGATCGGGTATGAAGAGCAGAACGAGTGGTACTTCTTCAGCCACAAAGACAAGAAGTATCCGACGGGGACTCGAACGAACAGGGCCACCATGGCGGGGTTCTGGAAGGCCACGGGAAGAGACAAGGCGGTGTACGACAAGGCGAAGCTGATCGGGATGAGGAAGACTCTGGTCTTCTACAAAGGGAGAGCCCCTAACGGCCAGAAAACAGACTGGATCATGCACGAGTACAGACTTGAATCCGATGAAAACGGACCCCCTCAGGCAAGCCTTCTAGATTGTTCTATTAGATCTAATTAA

## >AhNAC129-Arahy.T50ENK.1

CATAGCTTTTTTGCTTTTTGATGTAGTCAAGGGTCTCTCAAAAGAAAGGAGTAGCTTAGCTTGAAGAATTGAAGACCTTGCTTCAATAACTTTGAGCTTTATTAATTAATTAGCTAAGAAAAAACCAACCAGGTTTTCTCTCTTTTATCTCTTTTCTCTTTCCGTATTCTTCTTCTTCTTCTTCTTGATCTCAAAATTTAATTAACTAGAAGCAAAGTGTTAAAGCAACTACTACTCTATCTATATGTCAACAAGATATTATTGAAGAAACAAGGACACATGCTCATTTGTGGTGGAGAACTGTTCCATAGAAAAAGAGATCTTCAAATAATTGAAGAAGCTATATCAACTTCTTCTTCGTCTTCCTTCTTCTTCTCGTGTTTTTATATCAACAACAATAACAGTGCAAATTGCGAGTCGCCGCCATGAATAATAATAAGATAAGCAACTTGAGCTCCGTGAGTAGCTCCGATCTCATAGATGCCAAGCTTGAAGAGCATCAGTGGTGTGGAGGATCCAAGCAGTGCCCCGGTTGCGGCCACAAGTTTGAATCCAAACCGGATTGGCTAGGTTTACCAGCAGGAGTGAAGTTTGATCCAACAGATCAAGAACTAATAGAGCATCTTGAAGCCAAAGTGGAGTCAAAGAACATGAAATCACACCCTTTGATAGATGAATTCATTCCCACCATTGAAGGTGAAGATGGGATTTGTTACACCCATCCTGAGAAACTTCCAGGAGTAACAAGAGATGGATTAAGTAAACACTTCTTCCATAGGCCTTCAAAGGCATACACAACAGGAACAAGAAAGAGGAGAAAGATTCAAAATGAGTGTGACTTGCAAGGTGGAGAAACAAGGTGGCATAAGACCGGTAAAACAAGACCGGTCATGGTTAACGGAAAACAGAAGGGTTGCAAGAAGATTTTGGTACTCTACACTAACTTCGGCAAGAATCGGAAGCCGGAGAAGACGAATTGGGTGATGCATCAATACCATTTGGGGCAACATGAGGAGGAGAAAGAAGGAGAGCTTGTTGTGTCTAAGATATTCTACCAAACTCAGCCTAGGCAGTGTAATTGGTCGTCCGATCGGAGCGCCACCACCACCATCGCAACGGCCGAAGGGAGTGGAGAGCCATTACAAAATAGTAGAAGGGATAGTGGGAGTGGAAGTTGTTCTTCTAAGGAAATTAACATCGGTCATAGGGATGAGATGTCTGCTGTGGTTGGAGTTAATAATACTCCAATCACGAGCTTCACTCATCCCTTGGACATTCATCATCACCTCAAATCGGATCATTTTAGCTTCATTCCATTTAGGAAAAACTTTGATGAGGTTGGAATAGGAGAGGCTTCAACAGCAAGAGAAGTTCAAGCATCAGGGTCATGTGATGAAGTAGTACATGAACATGTAAATCATCATCATCAGCAACAACAACAACATCATCATCATCAACATCATCAAATTGCAACATCGGCATTCCATATAAGTAGGCCTTCACATCCAATCTCTACCTTGATATCTCCACCACCACTTCACCACACATCCATCATCTTAGACGATAATTCCTACCAAGTCTCCAGAATAATGCTCCAAAATGAACATTTCCAGCAACAACAACAACAACAACATCAACACCATCATCATAAAATTGGAGCAAGGTCTGCGTCTGGTTTGGAGGAACTCATCATGGGGTGCACTTCTTCTGATATCAAAGAGGAGTCATCCATCACAAACACACAAGAAGCTGAATGGTTGAAATACTCTTCCTATTGGCCAGACCCTGACAACCCGGATCATCATGGGTAGAACAAAGAGTCAATCAATTTTTTTTTTAACAAAAAAAAAAAAAGAAAAAAGGGAGAAGAAAGAGAGAATAAACAAAACCACAGAGACACCATCATTGTCATCATCATTAATGCTTGAAGCCTCCCTTAAGTTTCCTTTCAACAAGAAAAGGACAAGGAAATCATCATCCAAATAGTTTTCTTTTTTTTTTTTCCTTTTTTTTTAATTATAATTTTTTCTCTTTTTCTTTCTTTTCCAATGAGGCCTGTTTATAATTATAACAGTGTTTTACACTGGACAAGCTGGAAAGAAAAGGAGAGAAAAAAAAAATAGAAAGAACAAAGAGGAAAAGGAAAGAAAATCAAAGAAGAGAAAAGAAAAGAAAACACCAAGAAAAGCTGCTCTTAGTTCTGACAAGAACATATAGAGAGGAACAGAGAACCAAAGATGCATGAAAATCATCACTATTTTGATTACAATATATATATACCTAGTATTCATATTATTATTTTATTATTTCTATAAATGTTTGTACAATTTATTTTTTACTTTTCTCTCTTTTTTTTATTTTTAATTTATAATAGAAATATATAGCAATGAGTTTGTGCAAACTGCAAACCACCTTGTTACTTGTGCTTTGGTTACTCACTTTGTTATTATTAATTATATATAGCAAAGTATAAATTATTCTTATACTAGTCCCTTTTATGTTTCTATATATTTGTGTATAGAGGAATGAAAATGGGCAAAAGAGTATGTCATAGAGCTTTACCTTCTCTACTTCTTTTTCACATTTTTTGAAAGGTTTAGAAAGGAATCATGTAGCTTTCACTTTATCTTTTAGAAATTTTATTATTGATTAGGAGCTTTACATGGAACCTTAAGCGGTGCAAGAATTTTCAGATTAAGTGTGGTGTCAGTTTCAAATTATAGTTGTTTGTCGCAAAATGAAAATATTTG

## >AhNAC130-Arahy.T5AJQY.1

ATCACGTCCCAAAAATTAAAAAGAATAAAAACCAACATTTATTCATCTACAAGTTTTCATATACCAAGTAATATATACAAAGCATACCTAAACTTGAAATCAAAGCAATGTTTTTATTCCTTTTTAATTTTTTTTATTATTCTTATCTCTCTCATTGCAACCAATCCTATCTTCTATTCTTCTATCTCATTCTCTCTATTTAGTTTTGTTTTTGGGGGAAGGAAACTGCTTCTTGAAAACTGAAAAGAAAGCACAAAAAAGAGAAAGTGAGGGAGAATTTAATTCTTTAATTCCTTCCCACACCTCAACAAGTGCTAGCATGAGGGGTGGTGCAATCTTTAGAAGAGACAAGTTTATAAACTTTTTCTCCCTTTAATTTTATATTTAATTAATAATTATTACTACTTACTTTATTATTAGATCCCTCTTTTGAAGTACATACTCAACCCTTCTATCTCTCACACACACACACACACAAACACAACTTTCTATCTTTCTCTTTTTAGTTTTTGTTCTATTTGTTTTATAGAACAAGAGAAAAACAAAGGAAGGAAAAGAAGATATCCATAAGTGGAATATGAATGTGTGAGAAGGATAATAAACAATAAGGAGAAATGACTGAGTGCAATGAACATGAAAACAATCATGGCAACATCATAGTGGAGGGAAGAAAAGACAGTTTAATTAGAACTTGTCCAACATGTGGTCATCACATCAAATGCCAAGATCAGGGTGGTGGACTTCATGACTTACCTGGACTTCCAGCTGGAGTGAAGTTTGATCCAACAGATCAAGAGATTCTTGAACATTTGGAAGCAAAAGTGCGATCTGATATTCACAAGCTTCACCCTTTAATTGATGAGTTCATCCCAACTCTTGAAGGAGAGAATGGAATCTGCTATACTCATCCAGAGAACTTGCCAGGAGTAAGCAAGGATGGGTTGATCCGGCACTTCTTCCACCGGCCGTCGAAAGCATACACAACCGGAACAAGGAAGAGGAGGAAGGTGAACTCGGACGAAGAGGGAAACGAAACCCGTTGGCACAAAACAGGCAAGACCAGACCAGTCTATATTAGGGGGAAGCTGAAAGGATACAAGAAAATCCTTGTTCTCTACACAAACTATGGTGGGAAGCAAAGGAAGCCAGAGAAAACCAATTGGGTGATGCACCAATACCACCTTGGCAATGATGAAGAGGAGAAAGAAGGGGAGTTGGTTGTTTCCAAAGTGTTCTACCAAACACATCCTAGACAATGTTCTTCACTCTTGGTCAATAACAACAAAGACTCTTCAACAACACTTGTCAAGGGTAATAATAATAATGGGTTTGTTGAGTATTACCATTCAAATTTCATATCATTTGATCAAGGGGAACACCAACATAGATCTAGTGGGGCTCAAGTCGTCATTTCACATTTTCCTCTCCATGAAGCTGCTCCTAATTATCATTCTTTGAATCAAAAGGAGTAGTGTAGAAAAAATAGTAAATTACCATTTTATCTATGAAAGAATTAGCTTGCTGATAAAATGGTTTTCTGGAGATTGATAATTATTTTATGGTACTCGATTTTTTTTTTTTTGTTTAACAAAATTACTTAAAGCAATCATAATTTGTTGTTGTTGAAATGGTAATTTATTTGGGGAAATAGACATATAAGGTGAAAGAGTTTGTGAAAAGAGCTGAGGAGAGGAAAATCATAAATAGGAGTTTTTTTTATATAATATTGAGGCATTATTGTAACATATTATGAAAAGTAGTTAAGAGGGCACATAAGTGAACAATAATAATTATTAATAAGGAGAGAATTTTGTTCATTTCCAATACTACTGTGTTTGTTGAGGAAGAAATCAATTAAGAATTCGGCGAAGTGAAGAACATGTGGTATTTGTTATGTCACTGTGTTTCTGCTGTCTCCCGTCTAAAGGTATATTATTATTATTGTGATTGGGTGAGTTCTAACCATCAATCTGTATAGACATGACTTATGTAAATTGGTGCGATTTATGCCGCACTTTCTAAAAGAGATAAAATAATTGTCCTTTAATACAATTTATATGTAAATAAGTTGTTACTGCGATTTATCTATAAAAAAGAGGGCATATATAAGGAGAATAGGATTGAAAGGTACCAAAGATTAGTTGAAAAAGGAGAAAACTAAGAGCTAACAAGAGAAATGCAGAGAGAGTTGAGAGAAGAGATGGCAGAACCTTTTAGTTAGCATCTGCATCTGAGTTAATTAACATTTATTACAAACTAAAT

## >AhNAC131-Arahy.TCTP66.1

ATGGAAGAAGGAGGAGGAGATCAACATGCCTCTAACAGCAGCTACACTTTTCCACCAGGTTTCAGATTCCACCCTTCTGATGAAGAACTCATAGTTCATTACCTACAAAACAGAATCAGCTCTCGTCCACTTCCAGCTTCCATTATAGCTGAGATTGATCTTTATAAGTATAACCCTTGGGATTTGCCAAAGAAGGCTTTGTTTGGAGAGGAAGAATGGTACTTCTTTAGCCCGAGAGATCGCAAGTATCCAAATGGATTGAGGCCAAACAGGGCAGCAGGTTCAGGGTACTGGAAGGCTACCGGAACTGACAAGCCGATTCTCACTTCTTATGGATCGAAGCGCATCGGAGTGAAGAAAGCTCTTGTCTTCTATTTAGGTAGACCTCCAAAGGGGACTAAAACTGATTGGATCATGAATGAGTATAGATTGGTTGACACAATCACCAGCCCCTCCAGGCTCAAAGGTTCCATGCGCTTAGATGACTGGGTACTCTGTCGCGTTCGACACAAAGGCTACTCATCGAAGAACTCATGTGAGAATCAAGATAATCCTTGTGAACCAAACATGCTACCAAATCTGCCAAGGTGTGATGAAGGTTATCCAGCAACAAACATGAACTTTCATGCTGATATGATCACTGATTATCAATACAAAGACTATCAGATCCTAGCTTCTATTCTTGTTGGTGGCCATGTTCCTACCACTGAGAGCATGTCAAGTTTGAACTTGAAGGATGGCAAAGGCAATGATCCAATAACTTCAGTTCATGAAGATGGTTTCCACAGAGAAGATTCTTCTACAACAGTTTCTCCTTTGGACTGTTACTTCAACTCACTGAAAAGAAAATCTAATGAGGATAACCAATATGAGAATCTCATTTCCTTTAACAGGAAGTTGAACATGGAGACCGCAATGGATGATGAATCTTCTATCATTAATGGAGGTTTGAACTTCTACAATCAAAACCAGTCTCAAGATGACATAATATTCAATAAGAGAGCAGCAGAGCCTAGCATCAACTTTCAAGAGCTAAAGCAATCAGCTTTTATAGGAAGATACCCACAATGCTCAAGTGATTGA

## >AhNAC132-Arahy.TY7WD8.1

TCTACCTCATGACCAAGTCATAACCCAAATCACCATCATTAATCACAATTATTAAATAAATAAAGCCACAAATTAAATAGTTATGAGCTCCATTAACACAACCAACTCCACCTCCTTATAAATATACACACCCCTCTCAATATCATATTCATTTAAACATAACATACAACACAAGAAGAGAACCATAATTAAGCAAGCGACATATACATAATACATAATTGATGATTGATATGGGTTCTTCATCAGTAATAGAAGGTGAAGTTACACTTCCAGGATTCAGGTTTCACCCTACTGAAGAAGAGCTCCTTGATTTCTATCTCAAGAACATGGTCGTTGGAAAGAAGCTCCGTTTTGATGTCATAGGCTTCCTCAACATCTATCACCATGATCCCTGGGACTTGCCAGGATTGGCTAAAGTGGGAGAGAGGGAATGGTATTTCTTTGTGCCTCGGGACAGAAAGCATGGCACCGGGGGAAGGCCAAACCGGACCACCGAGAAAGGGTTCTGGAAAGCAACCGGTTCCGACCGTAAGATTGTTACCTTGTCTGATCCGAAGCGCATAATTGGATTGAGGAAGACACTGGTTTTCTATGAGGGAAGAGCTCCACGTGGATCCAAGACCGATTGGGTCATGAATGAGTACCGTTTACCTGACAATTGCCCCTTGCCTAAGGACATAGTGTTATGCAAGATATATAGGAAGGCGACTTCATTGAAAGTGTTGGAGCAAAGAGCAGCAATAGAGGAAGAGATGAAGCAAATGGTAGGTTCCCCTGAATCCACACCTTCCTCCACAGACACCATGTCCTATGAAGAACAACAACAGAATCAGAATCAGAATCAGAATCTGCAATTGTTACCACCACAACATGTTGTTACTAAGAAAGAGGTTGAAGCTGAACTTGAAGAGGAAAAAATGGTACATGTTACATTGGCAACAACAAAGCAAGAAAACAAGGACACAACAAAGAACAATAAAAGTAGTTGTTGTGGTAACACAAACACTAACAGTAACACAAGTAGTCTTCAATTGCCATTTGGGAAGGATAAGATCCCAGAGCTTCAAATGCCTATGATGATCACTGATTGGACCCAAGACACATTTTGGGCTCAATTGAATAGTCCTTGGCTCCAAAACTATACCTACTCCAACATATTAAACTTCTAGTAATATTATCAATCCAAACTTCCAAACCATTCACTTCAAGTGCAACTTAATTAGTTATTATTTATTACCTATTTTATTTATTTTGGAATATGTATATGCAGTGAATTAATTAATTAATAACATAAGTTATCTACCTCAGTTATTTAATTTAGGGCAGGTGTTTATTCAAGTTTAACAAAAATTGTGCCAAGCTGTAACTTATAGTGGACCCTATACCCTATTTTTAGTGGGATTTATTGGCTTATTTATTTGGCCAGTTTCATCACTGTATAGGGAGGGAAATTCGGGTTGTAAAGTTTATTGTTATTATCTCAGCACAATAATTTTGTGATTTATTTATTATGTAAGTTACTATGAAATTTCTTTAGTTCTTTTACTTGAATTCAAGCTTCGCAAAGGTTGTTCTGAAACTCTTCACTCTTGACTCCTTCAAATGAAATCATTGTATGTATCTGTCGCAGTTCATGTTGAATTTCTTTACTTCTTTTTACTTGAATGAGATAAAGCTTTCAAAGATTATCGTGTGGAACTTTTGGGTCTTAGACTCGTTCAAAAT

## >AhNAC133-Arahy.U16Y2L.1

TACCGCGTATTTTGCTTCAGCATCCACTCCATAGTCCCTACAAGTTGGTCCCAGCAATGGCAGTTTAAAAAATGGAGCCAGATTGCTTTTAACAATCTACATGTGAATTTTGAGGTGAGAATATAAAAGAGACTCTCTGAAAGTTTTTTAGGAGACTTGGACCCTCACCCTCTTTGATGACACAATCACATGAATTAAAGTGTTCAATCCGACACAATATGATGTGGATTTAACTTGATGTTCAGGGTCTTGGTCGCATCATCCATCCATTTAGGTACCCTATTCCTGTTTTTTTACATGTTCACACAAAATTGAAAATTCTTTGCTTTCTCAGCAAAATTAAGTGATATGATATGTCATATGTGTTCAACCAAAATTGTCCTGCAAAGTTTGTGCTGAGATTTGGGAATTGAAATGTAGTCAACAGTCACCAATAATTTGAACCGCCAAAAATAAACTAAGAAAACATGTTATGAGTCTATATCAAATGTGAGAAACTATATGAATATATGGACTTATATATATATATGAACAGAACTAGAAAACTAAAACTTTATAGGTGTGAACGGGGCTGATGAAGTGGTTGGTGGGGCCATTTGCTTTGGAATTTTGGGTGCACTTGAGACAAGCAATCTCAATTCTCAAACTTTCGGTGTGTTGTGTGCAATAATCTTTAACTGTTAATTCCTTCTCCCAACTGTTATATATAGCACACCATCTCTGGCTTCTTTTACTCTACTCTACTCTACTCTCTACTCTCCACTCTCCACTCTCCACTCTCTTCCTTATTTCTATCAATTTTTTTCTGTTTCCAACTCAATTTCACATTCTCCATACAAAGTAACTACATTACTATTTAATTTGGTTCATCTTAACTTCTGGGGCTTTCAAAAGCTTCCAACTTTTTTGGAAATCTTTCCTACTCAAATTTTGGACTAGTGCCCATTTTTCTTTTGTGAAAGTTGGTATTTGGGTTACACTGATTTGAACATCTGGTTACTTACTCCTCAGTTTGGTTCATTTTTTCTTAACGGGGATTAATATCAAAGGGGTGTTGGTGGGGGAAGGGTGTGATAGCTTCATAAGAGACCTCAAATTTCTTTTTAAGACCATGATTCTGGCACACAACGGGTGCCTTCTGTTGAATTCTCGCCTTATTCTTCACAAAGAATATATCATAGACAAAAGTTTCTACTTTTGGATCTTTCAGATTTTTAATAGCTATAGTCTTGGTACCTAACAGGAAAATTCTATATCCTTCACCAACATTCTTGAAAATACTATCTTTTTTGTTGTGTTAAATTGAGACCGGAAGAGACTAGAAAACTGCATATACCTTTGGTTCCTTGTTTCTCAATTATACACTTTCCATTCTGTAAGTGAGAATGGGAGGGGCATCACTGCCTCCGGGTTTTCGTTTCCACCCTACTGATGAAGAACTGATAGGATACTACCTGAAAAGAAAAGTTGAGGAGCTTGAAATTGAACTTGAAGTTATCCCTGTGATTGATTTGTACAAGTTTGATCCTTGGGAGTTGCCGGAGAAGTCATTCTTACCAAAAAGAGACTTGGAATGGTTCTTCTTTTGTCCAAGGGATCGAAAGTATCCGAATGGATCAAGAACAAACAGAGCTACCAAAGCAGGATACTGGAAAGCCACTGGAAAAGACAAAAAGGTTGTGTGCCAATCTAGTCCATCAACATCAATCATGAAAGCCACCGGATATCGCAAGACCCTTGTTTTCTATCGCGGAAGAGCCCCTTTAGGCGACCGAACGGATTGGGTTATGCACGAGTATCGCCTCTGTGATGATCTTGGCCAAGACTCACCAAGTTTTCAGGGTGCTTATGCTTTGTGCCGGGTTATTAAGAAGAATGACAAGGCCAGTGATTACAAGGGTAAAAGAGGTGTCAGCAGTTCCAAGAATGAGAATGAGAATGAGAATGAGAGCTCAATGAGATTGTCATCCTCTAAGGAGCACTTGAGCATCTCTGCTGATGTTTCTTCTCAAGCAAGTCAGCTATGCAGCGAGAGTCGTTATTCGAGCCCTATAGCTTCTCCTTGTGCATACAATGTGGCTGCAACGGCCGGGTTTGAGCCACCTTCTGTGGACACTAATCCTTCAACCTTCTTGGTCTCCCCTGATATGATTCTTGATTCTTCAAAGGACTTTGCTCAAACACAAGATGTTATTTCAGGATTCTTTCCGCATCATGAATTGCCAAGTACAATGACACCATGGCAATCATTGGAACATACAGAGATTTCATCCAGTTCATCCTACTCAAATTTCAATGGGGAGATAGAATTCTCTGATGAACTCGGCCTAATTGGCCGAATGTCGCGTTACTCAGGACAAGTAGACATGTTAGACTTCTATGGAAATGAGGAAGTGCTGTATGAATATGAAGGATATGACCAGATCAATTCAATCAGAGATCCAAGACAATTCTGAAGTGAAAAGGAAAATTTCTCCTTTTCTTGTAATGCTAAGCATATATGGGAATGGAAGTAAGGGAGATCTTGATGATGGAGGGATCTCTGAGGCAACGCCTTTTAATGTGTGTCTTGGAGTGAAAAATAATGAGTACAAGCTGCTTCTTCTGCACAAATGCTCCTAGCTATGTTTATCATTTCATGCAGTTGTGTAAGATGCTCGGGTGACATCACATTTTTTAAATTGCACCTGGTGTTTGGTCTTAAATTCATTCACCTTATGTAATGAAGGAGAATTTCTTATGTTTCTTATATTTATTCACCTTTCTTTACATACTAACTTAGTAACTTACATGAATTGAAATGCATGCTTATGCATTTTTGCTTTTCCTCCAATGCTTGCCTAATTTGTTAACACAGCAAATAACTATGGGATATGAGGCATGAATCATTCTTTTTATATTAAATCTTCATTAAGGTAAGTTAGAGTTGAATTGTATTCTCCACCCTACTCCAAAAGCAAAGAAATCAAAATGTTTGGCTTTGAAAAGTTCCAAATTATATGCAGACAAACTAAAATAGGTAGAATGAGCATATGAACAAAATCAAACTCC

## >AhNAC134-Arahy.U487DX.1

AATTTATTTACGGAAACAAAATTACTAAATTCCCATAGACACCTAAGCATTGGTGGTGTAGTAATAGGATGGATTTGATTGGGCACACGTGTGACAGCGGGTATTTTGCTGGATTTGCACGTGTGAGGAAGGCACCTCCCTTGGTCCGTTAGTACATTTATCTGTACCACAAAGTCCCATATACACTGATGAATACTCATCTATCTGATCCTACCCCACCCACGCATCACGCATGGATTCATGTCAACCCCAACTCCCACCGGGATTCAGGTTCCACCCAACCGACGAAGAACTCATCGTTCACTACCTCAAGAGAAAAGCTTCCTCTGCTCCTCTCCCCGTTGCCATCATCGCCGACGTTGATCTCTACAAGTTCGACCCATGGGAGCTTCCAAGTAAGGCCACGTTTGGGGAGCAAGAATGGTATTTCTTTAGTCCGAGGGATCGCAAGTATCCGAATGGGGCTCGCCCAAACAGAGCAGCTACGTCTGGATATTGGAAGGCCACCGGCACTGATAAGCCTATTATTGCGTCTGATGGCCAACACCGACTCGGCGTCAAGAAAGCTCTCGTTTTCTATGGCGGCAAGCCTCCAAAAGGGGTCAAAACCAATTGGATCATGCACGAATATAGACTCACTACTACTCATAACAATAATTCTATCTCATCAAACTTGATGATTGGGTGTTGTGCCGAATATATGAAAAAAGCAACCGTGGCAATTTTGCAAGAACAGCGTTGATGGAGCACCATGATCATGATGATGATGATGACAACAAGGATCAGCTTTCCGCGGAAACAACGAGTATGATAGAAAACATGTCCACTATGAGTCAGAATTCCAAGCCCACACAACATTATGGACCATTGCTGGTTCAAAACGATGACAACTTCTTCGATGGAATCTTAGCCGCTGATCATCAAAATCATCAACATCACAACTTGCCAATGAAGAGGGCACTGGTGAATATGAATAATTCGCAGTTTTGGAATGAGACAAACAAGAGGTTCCATTGTGATCTCAATAACAACACTAACACTGTTGCTAATATTGCTAATAATGATGAGGATAACACTTCCTTTGTTTCACTGCTTAGCCATAATCAGATTCCTCATCCTACTAACAATGCTTCTCTTCTTGGCCCTACTGTTGCTGATGGTGTTTTCAGGCAACACTTTCAACTTCAAACAATTAATTGGAACTTATAAATTAATTCTCATCTGACTAGCTAGCTAGCTATATATTATTATATGTAGTGTGATCAGAGTGGTCTAATATCATTGATTATATATACAGTACATGTAATTAGCTATTTTATTGTCACTTTTCTATTTGAAGCTTAATTAATTAAGCTATACAATGTTTGTATACAAAATATACTATTCTATGATGTAAGTATACCTATATACCAAGCTTATACAAAATTAGCTCTTGCATTTTGAGTAGTCAATATGCTGCCTCTAGTTAATTTACTATGTTAGCTTTCTCCCATGTTGTTAACAAAAATGTTTGGGGGATATAATTTGAAAATTATAGATAGCTATAACT

## >AhNAC135-Arahy.UCK419.1

ATGGCCGGATCATCGTGGTTGGTAGACAAAAGTAGAATTGCAACCAAAATAAAGAGTGCATCTGGAGCAAGTGGGAAAGTTTTATGGAAAAGCAATCCTACCAGAACTTGTCCGAATTGTCAACATGTTATTGATAACAGTGATGTGGCACAAGAGTGGCCTGGATTACCAAAAGGTGTGAAATTTGATCCATCTGATCAAGAAATAATATCGCACTTGCTTGCAAAAGTTGGTGCAGCAGGTTCAGAGCCTCACCCTTTCATTGATGAATTTATTGCTACTCTTGAAGTGGATGATGGAATTTGTTATACACATCCTAAACATTTACCAGGTGTCAAGCAAGATGGTAGTGCTACACACTTTTTCCACAGATCAATCAAGGCTTATAATACCGGCAATCGAAAGCGTCGGAAAATAAATGACCAGGACTCTGGCGATGTCCGTTGGCACAAGACTGGAAAAACTAAACCTGTCATCTCGGACGGGGTTCAGAGAGGCTGTAAAAAGATTATGGTTCTATATATGACTTCAGTTAGAGGAGTAAAAGCTGAGAAAACTAACTGGGTTATGCATCAATATCACCTCGGAACAGACGAAGATGAAAAGGAAGGAGAGTATGTTATCTCTAAAGTGTTTTACCAGCAACAAGTTAAGTTTGGTGAAAAAGATGATCATGATGTTCCTGGAACCAATGAAGCAACTGTTGTGAAAGATGATCCAGTCACTTCGGAACCTCCTCATAGTGAAAAGCAATGTTCATATCTCCACATAGGAGAAAAATCACATCAGATTCCTCAGGGTCCTCAGACAGATTGTGTAGAAGACATTCAAGCCGAGTGTGAAGAGATTGTGAAAACTGATGTAGCCATGGCAGATGCTCAAAATAATGAAGGAATGGATAATGTAGAAAATAATGCTGATGGAGAACAAAAATGGTGGGACAGTGAGTCACAGAATTTGTTAGATTCACAACAACTTGTTGAAGCATTGGCCTTGTGTGATGATCTCCTCCATAGCCAGTGTTCCAATAAGGATGATGAAAATGTAGAACACAAGGAGCACTTGAGTCTTTCCATCTATGCTCATCTAGGACCAGAGCATCTGAAGAAAGATCTTGAAGAGTGCCAAAACCTTAATCTTGATCCTGCAAACGTAGAGCTCGAGACACCACCTTCAGAGTTTCGACTAAGTCAGCTGGAATTTGGTTCACAGGATAGCTTTGTTTCCTTGAGCGGTGGCAAGGCAGTCGACTAATCCGGCCCTTCTTCGGCTAATATGGGAAACAAACACATTTCTAACTACTTTTGTTGTAACATTTGATCAACAGTGGTGTAATTAATACTGTCTTTTGGTGTAGTGAAAGCCATGGTATGTTCCTTACAAGTTCTCTAGAAAGTTGCATGTGGACCTGACTTGTCCAAAAATCACTACATGTTTTTATTTCATTTTGAAAGTAACATATGAAGATGAACATGTACATTTATAATTTTATATCCCTATCTTGAAATGTGTTGTCTTGTCTCCCATAAAAATCTATTGTATGTATATTTGGTGGCGACAGATTCATGAATGC

## >AhNAC136-Arahy.UK39BN.1

ATTTACACACTATAAGAAGATATGAAAATATGTTAATATAAATATAGAGAAAGCATAGAGAATAGTTTTCACATGTATGTGTTGTTTTACTTGAGACTTTAAGTGTTTTTAGAGTCATGGATGTGGCTAAGTTGTACATGAACAACGACTACTCCGAAGAACATGAACATGAACATGAAGATGAAGATCATGAGATGATGAAAGAGGAGAAAGAAGTTGTGCTTCCTGGGTTTAGATTCCACCCAACAGATGAAGAGCTTGTTGGGTTTTATCTTCGGAGGAAGGTTGAGAAGAAGCCTCTTAAGATTGAACTTATCAAACATGTTGATATCTACAAATATGATCCATGGGATCTTCCAACTAATAATGCTACCAAGGATCTTCATGAAGCTGAAGTGTGGACACTATGCAGAATATTCAAAAGGATTCCAACATACAAAAAGTACACACCAAATTTGAAAGATTCATCAACATCACCACTGATGAACAAACCCATCAATAACATTAACCACCAAACTGATTCCTCAGTAACTTCCATATCATGCAGCTTAGAATCTGACAACAACAATAGCAAGCCATTCTTGACTTTCACTAACACTATGACTATGGGCATTCAACAATGTGAAAGGAAGCCTCTTGTTATTGGACATGTTGATGAAAGGAACAACAACTTTTTCTTAGACCATTCATCAATACATCATCAACAAGCTCCAACAACAATTACTACTACTGCTTTGTCATCATCATCATACTCATCATGGAACCAGCACCATCTTGTGGAGGATTACTTGTTTGCAAATGAGAATTGGGATGATCTTAGATCTGTGGTTGAGTTTGCCACTGACCCTAATAATTCCAAGGTTTATCTATGATTGTAATTAAAGAGTTTAATATATATTTTGAGAGGGTATTATTATTAGGGTATTTAGCTTCTTAAAACTACACTACTACTAGTACTACTACTACCAATAATAGTGACATGTATTACTATAATGTATATCTATCTTACTCTAACCCCAAATTCTTTTCTTGGGGTTGAAGTGAGAAAGTTTGATAGAGTATGTTTGTATGAAAAATAGGCCTTCAGTTTTGTTTTGACATTGTTCACATACACTTGGTTCTTTTGCTAATTTAATATAAACATATATAGTTTTATTTATACCTAATACACTCCTGGTTTAGTTTACTAATAACCACTTTTTAAATTTCAAAATCCAAATCTTAGTTGTAAATCACTGCCCTTTTACAATGTTGTGGTCAAAATATCTTTAG

## >AhNAC137-Arahy.UX5JN7.1

TAGCTTTTTTGCTTTTTGATGTAGTCAAGGGTCTCTCAAAAGAAAGGACTAGCTTAGCTTGAAGAATTGAAGACCTTCAATAACTTTGAGCTTTATTAATTAATTAGCTAAGAAAAAACCAACCAGGTTTTCTCTCTTTTCTCTTTCCGTATTCTTCTTCTTCTTCTTGATCTCAAAATTTAATTAACTAGAAGCAAAGTGTTAAAGCAACTACTACTCTATCTATATGTCAACAAGATATTATTGAAGAAACAAGGACACATGCTCATTTGTGGTGGAGAACTGTTCCATAGAAAAAGAGATCTTCAAATAATTGAAGAAGCTATATCAACTTTTTCGTCTTCCTTCTTCTTCTTCTCGTGTTTTTATATCAACAACAATAACAGTGTAAATTGCGAGTCGCCGCCATGAATAATAATAAGATAAGCAACTTGAGCTCCGTGAGTAGCTCCGATCTCATAGATGCCAAGCTTGAAGAGCATCAGTTGTGTGGAGGATCCAAGCAGTGCCCCGGTTGCGGCCACAAGTTTGAATCCAAACCGGATTGGCTAGGTTTACCAGCAGGAGTGAAGTTTGATCCAACAGATCAAGAACTAATAGAGCATCTTGAAGCCAAAGTGGAGTCAAAGAACATGAAATCACACCCTTTGATAGATGAATTCATTCCCACCATTGAAGGTGAAGATGGGATTTGTTACACCCATCCTGAGAAACTTCCAGGAGTAACAAGAGATGGATTAAGTAAACACTTCTTCCATAGGCCTTCAAAGGCATACACAACAGGAACAAGAAAGAGGAGAAAGATTCAAAATGAGTGTGACTTGCAAGGTGGAGAAACAAGGTGGCATAAGACCGGTAAAACAAGACCGGTCATGGTTAACGGAAAACAGAAGGGTTGCAAGAAGATTTTGGTACTCTACACTAACTTCGGCAAGAATCGGAAGCCGGAAAAGACGAATTGGGTGATGCATCAATACCATTTGGGGCAGCATGAGGAGGAGAAAGAAGGAGAGCTTGTTGTGTCCAAGATATTCTACCAAACTCAGCCTAGGCAGTGTAATTGGTCGTCCGATCGGAGCGCCACCACCATCATTGCTACGGCCGAAGGGAGTGGAGAGCCACTACAAAATAGTAGAAGAGATAGCGGAAGTGGAAGTTGTTCTTCTAAGGAAATTAATATCGGTCATAGGGATGAGATGTCTGCTGTGGTTGGAGTTACTAATACTCCAATCACGAGCTTTGCTCATCCCTTGGACATTCATCATCACCTCAAATCGGATCATTTTAGCTTCATCCCATTTAGGAAAAGCTTTGATGAGGTTGGAATAGGAGAGGCTTCAACAGCAAGAGAAGTTCAAGCATCAGGGTCATGTGATGAAGTAGTACATGAACATGTAAATCATCATCATCATCAACAACAACAACAACATCATCATCATCATCATCAAATTGCAACATCGGCATTCCATATAAGTAGGCCTTCACATCCAATCTCTACCTTGATATCTCCACCACCACTTCACCACACATCCATCATCTTAGACGATAATTCCTACCAAGTCTCCAGAATAATGCTCCAAAATGAACATTTCCAGCAACAACAACAACAACATCAACAGCATCATCATAAAATTGGAGCAAGGTCTGCGTCTGGTTTGGAGGAACTCATCATGGGGTGCACTTCTTCTGATATCAAAGAGGAGTCATCCATCACAAACCCACAAGAAGCTGAATGGTTGAAATACTCTTCCTATTGGCCAGACCCTGACAACCCGGATCATCATGGGTAGAACAAAGAGTCAATCAATATTTTTTTAAAAAAAAAAAGAAAAAAGGGAGAAGAAAGAGAGAATAAACAAAACCACAGAGACACCATCATTGTCATCATCATTAATGCTTGAAGCCTCCCTTAAGTTTCCTTTCAACAAGAAAAGGACAAGGAAATCATCATCCAAATAGTTTTCTTTTTTTTTTCCTTTTTTTTAAATTATATTTTTTTTTCTCTTTTTCTTTCTTTTCCAATGAGGCCTGTTTATAATTATAACAGTGTTTTACACTGGACAAGCTGGAAAGAAAAGGGGAGAAAAAAAAAAACAGAAAGAACAAAGAGGAAAAGGAAAGAAAATCAAAGAAGAGAAAAGAAAAGAAACACCAAGAAAAGCTGCTCTTAGTTCTGACAAGAACATATAGAGAGGAACAAAGAACCAAAGATGCATAAAAATCATCACTATTTTGATTACAATATATATATATATGGTGGCTACTCCAATGAAGATTTAATGATTATCATCATGTGAAGATACATCATTTTGACCATTG

## >AhNAC138-Arahy.V0X4SV.1

ATTTGGTGATTGGTCTGAGCCGTTACAATCAGATAGAGGGAAGATGAATACATTTTGTCATGTTCCACCGGGTTTTAGGTTCCATCCAACGGATGAAGAACTCGTTGATTACTACCTTAGAAAAAAAGTTAATTCATGTAGGATTGACCTTGATGTCATCAAAGATGTTGACCTCTACAAAATCGAACCCTGGGATCTTCAAGGATGGGTTGTGTGTAGAGTGTTCAAGAAGAGAGTGACTTCCATTATGCGTAAGATGAGTGATCATGATTCCCCATCCTGCACTTGGTATGATGACTCCTCTTTCATGCACCAACAACCAGATCACTTTGACAACTCTTGTTCTTCTTCATCAAAGCACCAACTAATCCCTAATAATAACTGTGATGTCTTCTACCAACAACACAACAACAACTTGCCTCTTCATCATCTTCCACTTCTTCATCAAACTGCTGCTCTTTCCAATCATAATAATAATAATCCAATCATGGCACCACCATTTGCTGCTATTAATAATAATGAAACTACTGCTTTTCAAGAACAAGGGAAAAGCTTAATTCATCATCAGGCACTACTCTATGGAAATTTAAATGAAGAGCAAGCTTCTTCTTCAGCTGATTGGAGACTTGTTGACAAGTTTGTTTCATCACAGCTTAGAGAAGATCATCATGTCTCCAAACAAGAATTGATGATGCCAGAAAATAATAATAATAATAATAATGATAATGGTGCCTCAACATCAAACTCAAGCTGTCCAATAATG

## >AhNAC139-Arahy.V20ZHW.1

ACCCCTCTATATTCCATCAACTCAGTCCGCGCCATATACTTCAGAAGTGGCAAAATTGTAGTTTCGGAAGCAAAAACCAAGGGTAAACAATGGTAACGCGGGATATACACGAGGAACCGTGAACGCGCTTGTCACGTAAACTATGAAAGCAGTATTAGTACTCTACAGTGTGTAGCACTGACGAAACTTTCTTTCTTCTCTCTCTTTCTTTTTCACTTTCTGTGCAACTTCTTTTACTTTGTTGGCGACTACTTTGTTAGTCTGATAAATAAAAAGTGAGCAAAAGCTAAAGGGGCCACTCACCCATCACACCCCAAATTCTCACCAAATATTGAATCGAATTATTCCTCTGATTCCTTCCTCATCTCTTCATTTGCTTTTATAATTCATCATCCCATTCTCTTTCGCTTCTTTTTTCTCTCTTTCGTTTCTCTCTGTTTTCGTCTCTGCTGCAAAATCATAAACCGGAAATATTAGAGCTCGTCCAATGGGAGCCGTTGTTGACTGTTATCCGCCGCACGCCGGCGAGGTTGCAGTTTTGTCTCTCAACTCGCTTCCCTTAGGTTTCCGATTTCGACCTTCCGACGAAGAGCTTGTTGATTATTATCTGAGACAGAAAATCAACGGAAATGGAGAAGAAGTCTGGGTTATTCGAGAAATCGATGTTTGCAAATGGGAGCCTTGGGACTTGCCAGATTTGTCGGTGATAAGAAACAAGGATCCGGAGTGGTTCTTCTTCTGTCCACAGGACCGGAAGTATCCAAATGGTCACCGGTTGAACCGAGCAACCAATCATGGGTACTGGAAGGCCACAGGAAAAGATCGTAAGATCAAGTCAGGTTCCACCTTGATTGGGATGAAGAAGACTCTGGTGTTCTACACAGGTCGTGCTCCCAAAGGGAAGAGAACCAATTGGGTCATGCATGAGTACCGCCCCACCCTCAAGGAGCTTGATGGCACCAACCCTGGACAGAATGCGTATGTACTCTGCCGATTATTCAAGAAACAAGATGAGAGTCTTGAGGTTTCAAACTGTGATGAGGTGGAACAAACAGATTCCGCTCCCATGGCGGCCAATTACTCCCCTGAAGAAATACAGTCTGATCAGGCTCTGGCTGAAGTATCGCCGTCTCAAGTTACAGATGAGAAGCACCAGGGTGTTATCCCTGAGATCTCTGAGGAAGCGGTTTCCAACGTTATAACCTCTGCTGATTGCCATAGTGACGGATATGATGCTTGTGAAAGGCGAAATCAAGCTTTTGAACTACCTGCTGAGGACATTCCGCCGTTGAATTGGGACATATTCAATGACCCCGAGGACAAGATATTTGATGACAAATTATTCTCCCCAGTCCATAGCCATATTCCACCAGAATTTTACTACCAAGCAAACAATGAGACAAATATTGCAGACATCTTAAATTCTGTCAATTGGGATGAGATCTCCTATGAGGATCCCTATAGTCAAGCACAGAACAACTTTTTTAATAATGTTAAGCAAAGTGTATCAGGTAGCGAACCAGATGCAGGGCTGACCAATATGACATGCATACACCCGACGAATGTTGTTTATCCCGAGGAGGCAATTCACAGAAAGGTTGCTTTGGCAACAACTCCGCAATTTTGCAGCACCTTCACGTCTGACTTCAGTGCTGATGAGCAGAAGAGCAGTGTCGCGTTAATTCAAAACAATTCCCAGATGGCTTCTTTTCCGGATGCCAGAACAGGCCAAGTGTATAACGTATTCAATGATTATGAGCAGCCGAGAAACCTTAATACCTATGTTAGTGGTGATACTGGAATCAAGATAAGGACTCGACAAGTGCGAAATGAACAACCAGCAATGATCTTTACAGATCAAGGTAATGCAGCAAGGAGAATCCGATTGTTAAAGCAGTGTGCAAATGTCTCAAACAAGATGGCAGATGATGGGAGTCCTAAACAAGAGCATGATTCAAAACCAATAATTGCAGGGAACAAAAACAAAACTTTCAAAAGTCACACTGCAGATAAGCATGATACTGCTAATGATCTGAATGAACGCCAGGAGAAAACTGAGTCAACTGATAAAAGAAACATGATATCTAAACTTGCAAAAGGAGGTTCTTCCATGTTGGGGTTGAAGGGATTATTGCGCAGAAGGCTTAGTTACATATCAAAGGCCTCCTCCAATTTCAAAATGTGGTCATGTGTTGTTGTGGCTTCTGCCTTTGTATTGGTCTCGTTTCTGTTCTTTGCTAACATATGGGGATATATTAACTTATGAACTTCTAGGAGATCATTCCTTTATGCGTGTAATGTGCCTCCATTTTTTTTTTCCTTTCTTTTTTTAGGGCCAGTTGTGGAGGCTTTTAGGATTGACTCCTAGATTTGTTGATAACTCTATTATGAGAGAGTATGCGTTTCAATATGTAGCTGTATAATATACTTAGTTGCTTACCTCGCTGGCTAGTAGAATTTAGATCAACACTTGTGCTACTGTTTTCTTCTCTCTTAATTACTTGCTTACTGCCTATGAATGCCAAGAAAGTTATTTGTGAAATAATGATGAGAGTTGTTCATGCTCTGATTCAATACTATGGTCCAAATTCAAGTAAGTCAAAAAGGCTCAATCCAAAGGATGGCCTTTACTGTTCACCTGACCTTCTCAAAGAGGTCGGATTCAGCTGGCAAATAACACTTATTCAAATAAGTAATTGTCTTC

## >AhNAC140-Arahy.V6VDUM.1

ATCACGTCCCAAAAATTAAAAAGAATAAAAACCAACATTTATTCATCTACAAGTTTTCATATACCAAGTAATATATACAAAGCATACCTAAACTTGAAATCAAAGCAATGTTTTTATTCCTTTTTAAATTTTTTTTATTATTCTTATCTCTCTCATTGCAACCAATCCTATCTTCTATTCTTCTATCTCATTGTTAATCTCATTCTCTCTATTTAGTTTTGTTTTTGGGGGAAGGAAACTGCTTCTTGAAAACTGAAAAGAAAGCACAAAAAAGAGAAAGTGAGGGAGAATTTAATTCTTTAATTCCTTCCCACACCTCAACAAGTGCTAGCATGAGGGGGTGGTGCAATCTTTAGAAGAGACAAGTTTATAAACTTTTTCTCCCTTTAATTTTATATTTAATTAATAATTGTTATTACTAGTTACTTTATTATTAGATCCCTCTTTTGAAGTACATACTCAACCCTTCTATCTCTCACACACACAAACACATACAACTTTCTATCTTTCTCTTTTTAGTTTTTGTTCTATTTGTTTTATAGAACAAGAGAAAAACAAAGGAAGGAAAAGAAGATCTCCATAAGTGGAAATGTGGAATGTTGTGTGAGAAGGATAATAAGTAGGGAAATTAAACAATAAAGAGAAATGAGTGAGTCCAATGAACATGAAAACAATCATGGCAACATCATAGTGGAGGGAAGAAAAGACAGTTTAATTAGAACTTGTCCAACATGTGGTCATCACATCAAATGCCAAGATCAGGGTGGTGGAATTCATGACTTACCTGGACTTCCTGCTGGAGTGAAGTTTGATCCAACAGATCAAGAGATTCTTGAACATTTGGAAGCAAAAGTGCGATCTGATATTCACAAGCTTCACCCTTTAATTGATGAGTTCATCCCAACTCTTGAAGGAGAGAATGGAATCTGCTATACTCATCCAGAGAACTTGCCAGGAGTAAGCAAGGATGGATTGATCCGGCACTTCTTCCACCGGCCGTCGAAAGCATACACAACCGGAACAAGGAAGAGAAGGAAGGTGAACTCGGACGAAGAGGGAAATGAAACCCGTTGGCACAAAACAGGGAAGACCAGACCAGTCTATATTAGGGGGAAGCTGAAAGGATACAAGAAAATCCTTGTTCTCTACACAAACTATGGTGGGAAGCAAAGGAAGCCAGAGAAAACCAATTGGGTGATGCACCAATACCACCTTGGCAATGATGAAGAGGAGAAAGAAGGAGAGTTGGTTGTTTCCAAAGTGTTCTACCAAACACATCCTAGACAATGTTCTTCACTCTTGATCAATAACAACAAAGACTCTTCAACAGCCGCACTTGTCAAGGATAATAATAATAACGGGTTTGTTGAGTATTACCATTCAAATTTCATATCATTTGATCAAGGGGAACACCAACATAGGTCTAGTGGGGCTCAAGTCGTCATTTCACATTTCCCTCTCCATGAAGGTGCTCCTAATTATCATTCTTTGAATCGAAAGGAGTAGTGTAGAAAAAATGGTAAATTACCATTTTATCTATGAAAGAATTAGCTTGCTGATAAAATGGTTTTTTGGAGATTGATAATTATTTTATGGTACTAGATTTTTTTTTGTTTTGTTTAACAAAATTACTTAAAGCGATCAAAATTTGTTGTTGAAATGGTAATTTACTTGGGGAAATAGACATATAAGGTGAAAGAGTTTGTGAAAGGAGCTGAGGAGAGGAAAATCATAAATAGGAGTTTTCTATATAATACTGAGGCATTATTGTAACATATTATAAAAAATAGTTAAGAGGGCACGTAAGTGAACAATAATAATTATTAATAAGGAGAGAATTTGGTTCATTTTCGATACTACTGTGTTTGTTGAGGAAGAAATCAATTAAAAATTCGGCGAAGTGAAGAATATGGTATTTGTTATGTCACTGTCTTTCTGCTGTCTCCCGTTTAAAGGTATTATTATTATTATTATTATTATTATTATTATTATTATTGGGTGAGTTCTAACCATCAATCTGTATAGACATGACTTATGTAAATTGGTTCGACTTATGCACTTTCTAAAAGAGAAAAAATCATTGTCTTTTAATACAATTTATTTGTAAATAAGTTGTTACTGCGATTTATCTATAAAAAAGAGGGTATATATAAGGAGAATAGGATTGAATGGTACTAAAGATTAGTTGAAAAAGGAGAAAACTAAGAGCTAACAAGAGAAATGCAGAGAGAGTTGAGAGAAGAGATGGCAGAACCTTTTAGTTAGCATCTACATCTGAGT

## >AhNAC141-Arahy.V88TU5.1

AATAATTCAATCAAACCTTGGGAGTTTGCAACATCTTTCTCTCAGACCTATTCTTTTCTCTCTTCTCCCCTTCTTCCTAGCAAGCAGGCTCTCACTATATATTAATTTAATTTGTTGAAATTAAATAGAAAAAAGAATAATGAGCAACATAAGCTTGGTAGAGGCAAGGCTTCCACCAGGGTTCAGATTTCATCCAAAAGATGAAGAGCTTGTGTGTGATTACTTGATGAAGAAGTTCACGCACAATGAATCCCTTCTCATGATTGATGTCGACCTCAACAAGTGTGAGCCATGGGATATTCCTGAAACAGCATGTGTGGGAGGGAAGGAGTGGTACTTCTACACACAGAGAGACAGAAAGTATGCAACGGGTCTGCGTACAAACAGAGCAACGGCATCAGGATATTGGAAGGCCACTGGCAAGGACAGGCCTATCCTTAGGAAGGGCAGCCTTGTTGGTATGCGAAAGACTCTTGTCTTCTATCAAGGTCGGGCTCCCAAAGGCCGTAAGACTGAGTGGGTCATGCATGAGTTTCGCGTTGAACCTCCTCTTCCTCCCCCCAACACTACTTCTTCTAAGGAAGATTGGGTGTTGTGTAGGGTGTTCTACAAGAACAGAGAAGTTAGTGGCAAACCTAATAGCATGGGAAGCTGTTATGATGACACAGGCTCTTCATCTCTTCCAGCATTAATGGATTCTTACATCAGCTTTGACCAACAACAACAACCTCAAACCCATCTTCATGCTGATGAGTATGAGCAAGTGCCCTGCTTCTCCATTTTCTCTCACGCCCAAACAAGCCCTATTTTCAACCACATAATGGAGCCTAAGTTATTCCCTACCAACAACAACAACAACAATAATGCAACTTTATATGGTGGAGGAGGAACTACTACAACACCCAATTTGGGTTCTTGCTTAGACCCTTTTTCATGTGATAGGAAAGTATTGAAAGCTGTTTTGAGTCAGCTCACAAATATGGAAAGAAACATACCTAATAATAATAACAACAATACAAATAGTATAAAAGGGTCACCAAGTTTAGGAGAAGGTAGTTCTGAGAGTTACTTATCTGAGGTTGGCATGCCCAACTTGTGGAACAATTATTGATGTGGTAGTCCCTATAGGCTTTGTAATTTGTTTATATACTTTCCCCCAAAATTAATTTATCCATCCATGTTCTTCATATGCCACAAGGAAGTGGAAAAAATATATATGAAGAATCAATAGAATTTTGGAGGAGGGGGAAATTCTCATTGGAATTTTAAGTCATTGTGGTGGGTATCTAGCGTTTCACCCATTGTGTGCATGTCCCCAATATATTAGGAAAACGGGACTTGTAATTCAAACTCAAAAATATTTGTAGTTAATTTTAGTTAGTGGATGAATTGTTTCTTCTTGCCAAGTAACTAAGAGTAGTAATATT

## >AhNAC142-Arahy.V9YDBD.1

AACTTGACGTCCAAGCAACCTTTCCTTATATCTAGAACCGCTTCCTTTGGTTCCCACCTTTTCTTTTTATTTCATCTATCGCTTATACCTTTTGTCCTCTTCTTCATCTGATCTTCCCTCTTTCTCTCTCCATCAGTAATCACCAATATCATCAAGCACATAAGAACCAATTCAAGGCGGTGGTAATCATAAGCTAAGCCAAGGGAGATAAAACGTGTCCCGAAAGAGATACAAATAAAGTACTACTATGCTTTCTATCTTACTTTACCTTTCTTTTAACTACATACTTAACTTGTAATTTTTTCTCCTGTTCAATTTTTGGGTATTTTTTTTTTACAATTCTGAGTATAAGGGAAGAAAAATTAGAGGGTGGTCTAATTTATTGTGATGGCATGGTGCAATGAGACTCATGAGAAAGAGATCATTGCTTCCAATAATAGTACTATTACTCTTAGACCTAAATCCGACCAAGAAATTCGAAACATAAGTTGCCCCTCATGTAGCCATAACATTCAAATAATCCAAGAGCAGGGTGGAATTCATGAGTTGCCGGCTGGAGTGAAGTTTGACCCAAATGACATTGAAATATTGGAGCATTTGGAGGCAAAAGTTATGTCTCATGTGTCCAACCTTCATCCTCTCATTGATGAGTTCATACCAACGCTTCAAGACGAGAATGGCATCTGTTATACACACCCAGAGAAGCTACCAGGAGTAAAGAAAGATGGGCAGATTCGGCACTTCTTCCACAGGCCTTCAAAAGCATACACAACAGGAACAAGGAAGAGAAGAAAGGTTCACACCGATGAAGATGGAAGCGAAACAAGGTGGCACAAAACCGGAAAAACAAGAGCGGTGGTGGCCGGCGGCCTAGTCAAGGGTTTCAAGAAGATTCTAGTACTATACACCAACTATGGGAGGCAAAAAAAGCCTGAGAAAACTAACTGGGTGATGCATCAATACCATCTTGGAAGCAATGAAGAAGAGAGAGATGGAGAACTAGTAGTTTCAAAAGTGTTCTATCAAACACAACCTAGACAATGCGGCAATTCCATTGTTATAAAGGAAGATGATGATGATCTTCCCTATGGAAAGATATTGATGATGAATAACAGAAAGAAGCACAAAAATAATGATGATGCTGCTCCTGTTGTGGACTACTACATAAATTATGACCATGTTGAGCATCATCATAATCACAATCATAATAGTCAAAGATGTTCATCACCTACTCAACTTATTCCAAACTTGGTCCTCCAAGGTGATTCCTCTTCTCTTTTTCGCTTTGCTTCATCATCACTGGATGGGAATGCCAACAAAACAAGACTTTTTGAGAGAAAGTTGTAGTAATTCGTTCATTATTATTATTTCTTTGACTAAGTGACAATATTTATTCTATACATACATGATATATGGGATTAATATAAGAAAAAGTCTAGGGGCCAGCAGTTTTATTGAATTTTGGCCAGC

## >AhNAC143-Arahy.VCF3H0.1

GAGTGTAACTACCATTTTTCATGGTTATGGTGTCTCAAGTCTCATGCATGCACAAGACCAGAAGCACACAACGACGGTCAACCATGTCCCCCACCAAATAGAAAAATACTAAGCCGACACTTTGATCTTTGACATAAAAAAAATATTACTAGAAAATAAAATGGTAGGGAAAAAACGAGAGAAAGGTATTGGTGACGCAAGCAATGACAGAATCAAGAACTGGAACCCCCACACGTGTCGACCAACCAGTAATGCGTCGCCACGTGGCAGTAGCAGTCAGCATGAGAAAAGAGGAGGCTGAGGTGTAGGTATAGCCTACTAATAAACGCTGGAACAATGCCACCTTCACCACGTTTTCACTACTTATCACGCACGTGTCTAAATTCCTCTCTCTTGTTCCTTCACGCGGTTATATAACTCCCCTCTCGTGACTCTCTCATACGTTTCAACTCGAAATTCAGCATCATTCGCCACCACCAGCACACACACAGAGATTCGATTCGGTTTGGTTCGGTTCAATTCAATTCATTCAATTTCAGCTAGAGTTGCAGCTTCTCGAGAATTCAAGAAGAAGAAGAAGAAGAAGGAGGAACAATGCAAGGTGGATTAGAGTTACCGCCAGGGTTCAGGTTTCACCCGAGCGACGAGGAATTGGTGAACCACTATCTCTGCAAGAAATGCGCAAAGCAATCAATTGCTGCTCCAATAATTAAAGAAATCGATTTGTACAAGTTCGATCCGTGGCAGCTTCCAGAGATGGCGTTGTACGGAGAGAAGGAGTGGTACTTTTTTTCGCCGAGGGATAGGAAATATCCGAACGGATCCCGGCCGAACCGGGCGGCTGGGAGCGGGTACTGGAAGGCGACCGGGGCGGATAAGCCGATAGGGAAGCCGAAGGCGTTAGGGATAAAGAAAGCGCTAGTGTTCTACGCCGGAAAGGCCCCGAAAGGAGTGAAGACTAATTGGATTATGCATGAGTACCGTCTCGCCAATGTTGACAGATCCGCCGCCAACAAACTCAACAACAACAACTTGAGGCTTGATGATTGGGTGTTGTGTCGAATCTACAACAAGAAAGGGAAGATTGAGAAATTCAACTCTGCCACAACAGGGTTGGAACAGAAACTACCAAAGTTTTCCCCAGGAGAGATACTTCACTATGATCATGAGCATGAGCATGAGACCAAGCCAAAGATTATCCACAATTTCTCCAACAATGAGCACCAATTGTACATGGACACATCAGATTCCGTTCCAAGGCTGCACACGGACTCTAGCTGCTCGGATCACGCGGTTTCGCCGGACGCCACCTGCGACAAGGAGGTGGAGAGCAACCCAAAGTGGAGCAATGAGCTAGATATGCAGCTGTTTGATACCTTTGATTTTCAGCTCAACAACTATGATAATAACCTCCCAATGAATGATGATGACCTTTTTGGAAATCAGTTCCAAATGAATCAGCTCATGTCTTTCCAAGACACATTCTTGTTCCCACAAAAGCCATTTTGATTCTTTTGACCATTAAGAATTATTGGGAATTGGAAGAAGAGGGGGAGAAAAAAATTGAACCTTTTTTTCTGGGAAAATGTGGTGGAGGACTATTGCCTAATGAGATCATGGGGACCAAAGCATATGTTCATTTTTTGTAGTGAAAACTCAGCATTTACACAATTCACACCTACCTAACAATTTCACTTTTGGACCATTGGGTTTTGGTGGCTATTCTTTGTTCTTGGGAGATTAGGGAGGGAGATGTGAAGGAAAAAAAAGCCAAAAAAAAAAGAAAAAGAAAAAGAAATGGAGATCAGGTTCTGTACTCTGTAATATAAAGAATAAGTAGCAGAATGATCTCAAAAGTGGGTGTAGATCCTTTGGGCCGTTTGATGATATTTTAGGGTGTTAAATGTGTAGTGGTTAGGACTCTTAGTAGGGGAAATTCTTGCTCCATTGGTTTTGTTAGTTGATTTTTCTATATATTAGTAATATAAAATATAATATAATTCATTATTATTTGCAAAACAAAAGAAACAATCAAAGAGAATGTTTCTTTCACGAATCTTTCTGTTCAGAACCAACAGTGATTTAGTGTGACAGTTGTAGCAAAGCATCTTTTCTGTTGAGAGAAAGGGTAGCTGTTATTTCTAGAAGAGTAAAAAGGATAAGATTCCATTGTAGCATATGGATTAAGAAGATTTGTTTCTACAGTCAAATTTGAATAGATCAAAAACCATTTGAGAAAGCAGATGGATTTGTGGTACAGCACTGAATTGATTAATTATGAAATAAATATATGATATTTGCATCAACTTGCATTACTCTGCCTTATCAGTTCCAAATGGAAACCTCAGAGCTGTAAATTTTAATGTAATCGTCGTATGACTAATTAATTTCTAGGTTGTAACCAGCTGTAACTACACTATTAATATTATTTAAGGTAAATTCCCACATCACCAGTATATCATTATGTTTTCTACTATTTATAAGGCTAACTTCACAAACAGATCAACATTCATATGATACAACACAACCACTCTGTTCTATAAACAGTGAGAAATAACTGCTACTTAGTGACAAATAAAGGAGAG

## >AhNAC144-Arahy.VI6QZG.1

GAGCAAAACAAAAAGCTCTCATCTTTGTATATTTAAATCTCAAAAACATATGATAATTGCTAATAATTATGAATGCACATACATATATAAATACACTCCAATTAGGTGGGGGCCACCCCAGCTCAATTGGTCTGAGACCAAACCCATACCAATGCATGACACGTGGAAAACAGCAGCTTGATGATATCAGCATGAAGTGTGATGATGATCCATATACACAATAAAAATCACCTTATTCATCACCAATGTATTAACTAAGTCCTGTTAGATCATAAATCCTTGTCAAGATAACATGATCTGCTTTCACTCCTAATGAAATTGTTTCAATCTGGTGTCACACACTCCTTCCAAGTATTCTGTTTATAAATTATAATGAAATTATACATGATAAACACCAGCTTGGGTTTCACGTCAACACCCTTATTATAAATACCTTAGCTTCTCTATTTGTACTATAAATACCACTCATCTTCACCATTACTCTAACTTGACACATTCCCTCCTCTTGTTTTCTCTGCCTATCTTCTTAATGGGAGATAACAATGTGAACCTTCCACCGGGGTTTCGATTTTATCCAACAGATGAAGAGCTTGTGGTCCATTTTCTTCATAGAAAGGCAGCACTCTTACCTTGCCACCCTGATGTCATCCCTGATCTTGATCTCTATCCTTATGATCCTTGGGAACTTGATGGTAGAGCGTTGGCAGAGGGAAAGCAATGGTACTACTACAGCAGGAGAACACAGAGTAGGGTGACTGAGAATGGATATTGGAAAGCAACGGGAATGGAAGAACCAGTGATGACAAGCTCAACTAACAAGAGAGTTGGCATCAAGAAATACTTTGTGTTTCATCTTGGTGAATCCCCTTCTGCTATCAAAACAAATTGGATAATGCAAGAATATTGCCTTTCCGATTATTCTGCTTCCTCTAGCAGATCCTCCAAAAGAAAATCAGATTATAGTAAATGGGTGATATGTCGTGTTTATGAGCGGAATGGAGATGATGATGATGGAACGGAGCTGTCTTGTTTGGATGAAGTTTTCTTGTCACTGGATGATCTTGATGAAATAAGCTTACCAAATTAAATTAATCAAGCTAGCTGCATTAATTAAGATAATCCAAAATGGATAATTATTAAGTATATAGCAGTGTGATGTTGCAAGTATAGGTAGATTTAGATTTCTATAGCAGCCCTTTAACACAAGAGGAAGTGGGGCACTATGTGAAATTCATGACCAAACAATATGTACTATGACGTTATGCTTTCAAAGATTCCATTTATCATTTTACCACAAATTTTTCTCTCCTCTTTTCTCCATATAAACACACGTGGACAAT

## >AhNAC145-Arahy.W5I9MA.1

TTTAGTGTAAAACCAAGATTGTTAAATTAATTAAATACGGTAAGATCGCAATCCCCAGATGCTGCTAGTTTGGTGGTGTATGGAGATTCCAGCTTTTGGGTCATAGACAAATAACACACAGCTCTTTAATTAAGTTGAAAGTATGAATGAACAGTGGTACTTAATGATGAAAATAATGTGAATTAATTGGGTTTGATTTCTAATTAAGTTGGTAGTTGTTCTCTTTTTAAGAGCTTCATAGCAACGCGATCCTCGCAAAACACACGCTCCTTTAAGAGTCACCACAAAAGACGGCTGAACCTTCGGGTACAAGATCCCTTCTCAGGAAGCATATAATCACTTCACTACTTCAACTGTGTTCTCTCTCTATCTATCTATCTCTTCATTATTAGGGTTTTCATTTCTTGTTTCAAATAAACTTCTTCTATATATCCACCTCTTTCAACTCTCTACTCTTTCTTGAGGAGATGGAAGGTGAGAAGCTTGATGAGATCATGTTACCAGGTTTCAGGTTCCACCCAACTGATGAGGAGCTTGTCGGGTTCTACCTTAAGAGAAAGATTCAGCAAATGCCTCTGTCCATTGAGCTCATCAAGCAACTTGATATCTATAAATATGATCCTTGGGATCTTCCAAATCTTATAAGCAACGACTGTTTAATACCCTCTTCTACTACTCCACTTGAAACATCCTCTAATAGTGCAAAACCTACTATGGATTTTTCTTCATTGTTGCTGAACATGTCATCTTCTGTTCTTGGAGATTTTGCTGGAAAGACATCATCGTCGTCGTCATCCCAAGAGGGTACAGCAGCAGCAGCAACAGCAACAACAATCACAAGTAGCTTCGGTGGTGGAATGCAGGAGCACTACCCAACAATACCATTACTGCGTCAGATGCATCAAGGGAACAACAACAACAACAACAACATTGGCATCAACAACAACAACGTGTCTGCTGGCGGTGAAGAACAAGAGTTGGAGAAAGTTGGATCCATTGTTGGGTTCCCATTCATGAACATTGGGGATGCATGGAAGTCAAATATGCTTTGGGATACTTCTTGTCCCTTGTGA

## >AhNAC146-Arahy.W8FFAE.1

ATGGCTTCAACAAAGTTGCCTTCAGGTGCAAGCAAGAAGTTCAAGCCTACAGATGAAGAACTCATTCAAGATTTTCTTCTTAACAAAATCAATGGGAGGCCTCTACCAAACAATGGAACCATTCTTGAAGGTGAATTGTTTGGTACGGAGAAGAATCCATGGGAAATTTGGGAAGAAAACGTTGAAAATTCTTATGACGGGAAGGACCTCTATTTTTTCACTACTCTGAAAAGGAAGTTCTCAACTAACAGCTTGCGGATGGTTCGCACCATTGGGTTGGGTTCTTGGGAAGGTGAAGACATCGGAAAAGAGATTATGGCCAATAAAACTAACCAGCGCATTGGAATGAGGAAACGATATCGCTTTGAGAAGAGTGGTACTAGCCATGATGGTGGATGGATCTTGCATCAATATAGCATTGATTCTTCTTTGTTACCAAATCCATCCAATATGAATAATTATGTTTTATGCAGATTTAGAAAGAATAACAGTAAACCTCGTCAAAAGAAAAGGAAAATTGTGGCTCCTAAAACTGTGGCTACTGTAATACCGTTTATAGCTTATTGA

## >AhNAC147-Arahy.WB1HDB.1

TGGTAACACTACATGGTGATGCATGCAGCTCAAGAGGGTTAAAATAGTCATTGTAGTTAGTCAACACTGCTAGGTGTCTCCTTAGAATTTACAACTTGCTATAAACCTGAGGCAAACTTGATGATGAAGACCCACATTTGGTGGAACCTTGAGAAACACACTTATCCATGGTTTTTCTTTCTTCATTTGCCTTTGTATACTTAAACCCTAATTAACTCTAGTTTCTTTCATGCATAATTGATTAGTAGTAGCAGTGATAAGGAAGCTCCATACTCTTCCCTCTCTTTTAATTTGATCTTATTATTTTGTGGTAACCCTCAAGTCAAGTGTTTTCTTCTCAAAGTGAATCACAAAAATTCAACCCAAAATTGAACAAAGAGTTTTGTCTCTCCATGCAAAAGGAAAAGGAAGCAATCACCGCCAATAAGGAGGGCACTAATAAGAAGGACATAGGAATAATGGAATGTTGCCATGGAAAAGAGGAAACCCTACCACCTGGGTTTCGATTTCATCCAACCGACGAAGAACTCATTACTTGCTATCTCATAAACAAGATCTCGGATTCAAACTTTTCAGGCAGGGCAATAACTGATGTTGATCTCAATAAATGTGAGCCATGGGAGCTTCCAGGGAAGGCGAAGATGGGAGAAAAAGAATGGTACTTCTTCAGCCTGAGAGATCGTAAGTACCCAACTGGGGTGCGAACGAACCGAGCCACAAACACGGGGTATTGGAAGACCACCGGAAAAGACAAAGAGATCCTTAATAGTGTTACATCGGAGCTAGTTGGGATGAAGAAAACTTTGGTTTTCTACAAAGGAAGAGCCCCAAGGGGAGAGAAGAGTAATTGGGTCATGCATGAATATCGCATTCATTCTAAATCCACCTTTCGAACAACCAAGGATGAATGGGTGGTTTGCCGTGTGTTCCAGAAGAGTGCCGGTGCAAAGAAGTACCCTTCTTCCAACCATGCAAGTAGGGCAATGAACCCTTTCAACCTTGAAATAGGTCACCACAATATTGTGCCGCCGCCGCCAATGATGCAACTCGGAGACCCCGCCGCCGCTCATTTCCTCTATGGAAGGAACTATATGAATACTGCAGAGTTAGCAGAAGTAGCTAGGGTTTTGCGTGTTGGTACTGGATCAACCAGTACCAACCTACCCGGGATGCAGCCTCAGATAAATTATCCAGTGGCTGCATCATCCCCAGGAGTTGGATTCACAATTTCAGGGCTCAATTTAAATCTAGGAGGCGGAGGAGGCGGAACAGTAGTGGCCACAACACAACCAGTTTTGCGGCCCATGCAGCCGACTCCTCCGTCCCAAACATTGGGTATGGTTCCTCATCATCAAGTTCATCATGATGTGAGTTCCAACATGATTTCTGGTGCTGAGAATGTGGGTTACGTCAATGAAATAAGCAACACAAATGGTGGTCATGGAAATAGGTTTATGGGCATGGATCATTGCATGGATCTTGATAATTACTGGCCTTCCTACTAAATAAAGGAGAGAGACCCTACTTCATTTTGTTTAATTAATTATTATACATAATTTAGTGTTATAAGCTAAGTTGGATAATTAAGCTACGTAAAACTTTTTTCTTTCTTCTTTTTTTTTTTTTAATTATCGTGTTTCTAAGTTTAATGCAGGTAAAGAGACCTTGCAGTATCAAAGTGAATCTTGTTCTTATTTGGAGTTACGAAACTTCTTTAATGTTATTGCTTTTATCTAGTCAATAATGCTACGTATAGCAAAAAATTCAATCATTGCTTTTGGGATGAGTGGTATCATCTATAGTATTAGAGCGCTAGATCCGAAAGGTCAA

## >AhNAC148-Arahy.WF4CBH.1

TATATATATATATATATATATATTCTTTGGGTTCTGTCTATGTTGACTGTTTAGAAAGAAAGAGCGGTGTTTGGATAAAATCGAAACTGATTGGTGCTGGCATCTTGGGTTGGAACTAACTAACCAGGCTTGTTTCGTTGTGCAATAACCAATAAGTGAATAGTTTTCGCAAAAGCCAAATGAATAATATAAACCATGGTTAATATAATGTCAAACCAAAAGGGAGGTTGAGTCTGGCTTACGCCAAATGTGTCGTCTTATTCTTCCCCCTCCTTCCTTGCTTGCTTGCTTCTTGTCAGACCCATTACTATAAAAGAAGGCACACTTCACCCGTTTCCATGCCCTTTTGCTTTCTTTATCCTTTCCTTCCAATCTAATAATAATAATAATAATAATAATAATAATAATATCAATAGATTAAAATTATTTTTAATATGAAGATGAATATAATATTTTAATTAAATATTAATATTTAAAATGTATTACATAATTATAAACATATAAAAAATATATTCAAATACATATTTTTATATTAAATAAAATGTTAACATATATTCAACATGTATCCTACTTAATATAAAATGTATCTGCGAATTAACTTTTTACCTATAAAATCCATTTATATGTAATTATACTAAATAATTTTATTTTAAAAAAAAATATTTCTTTCATATAATAAAAATAGTTAATACTAGCCTTTTTTTTTTGTTTTAATTTTTTCTTTTCTACATCTTTCTCTCTTCCTTTTCCTGGCCCTCCTTTTGTGCTTTTGTTATATTCTTCCTCTTTTCTTCTTTTGCTTGTGCTCTCTCAACTTTGATGGGCTAACATTGGGAATTGGAGGCTGCACGAGTGCCTTCAGTCTCTTCGCTGTTTGTCACCCAAGCGGACAAACATAAATACATAGTCTTACCACTGACCATTGTGGGGAGCATAGAACAGAAAAGGATGGAGGGGAGAGGGAGTAGTTTTGTGAAGAATGGGGAGCTGAGATTGCCTCCAGGATTCCGGTTTCACCCGACGGATGAAGAGCTGGTGGTTCAATACTTAAAGCGCAAGGTCTTCTCCTGCCCGTTGCCGGCCTCTTTCATTCCTGAGGTTGATATTTGCAAGTCCGATCCATGGGATTTACCAGGTGATTTGGAGCAAGAGAGGTACTTCTTCAGCACGAGGGAGGCCAAATACCCCAACGGGAACCGATCCAACAGAGCCACCAACTCGGGCTACTGGAAAGCCACGGGCTTGGACAAACACATCGCAACTTCAAAAGGCCACCAACTTATTGGCATGAAGAAGACTCTCGTCTTTTACAGAGGCAAGCCTCCTTATGGATCAAGAACAGATTGGATCATGCACGAGTATCGCCTTGTCTCCCACCCCCACCTGCTTCCCATGCAAAATTGGGTTCTCTGTCGCATATTCTTCAAGAGGAGAGCACCTGCTACTGCTAAGAATGTTCTACTGGATCACAATTCCCATTCGGCATCAGCATCAGAAGAGGCCTTCACCATCAGCCATGAGGGCAGCAACTCTAAGGTGGTTTTCTACGATTTCTTGGCACAGAACAGGGCTGATTTGAACCGCGTGACCCCTCCTGCTTCTTCGACCTCTGGCACCAGTGGAATCACCACCGAATCCGATGAGCATGAAGACAGCAGTAGCTGCAACAACTTTCCTTTTTTCAGTTGGTACTAG

## >AhNAC149-Arahy.WPHD30.1

TAATATTAATTCTTAAAAACTTAAAAATACCCTTAATATCAATTCCTTAGTTTAATATTTTATTGTATATCATATTACCTAATTAAAAGTATAGCGGTATTGGCTAGTTGAAGACACCACCACATACATAAGTAATGGACAATGGTCAAACCATTAATAATAGTGAAGGTTGAAGAAGAGGGACTTTGGCAATATAAATAAATAATCATAAGAGGAGGAATATTATTTCTTTGAGGGAGGGACAAAAACAAAAAGAAATTAGGTGGAGACATGGAGGGAACAGGATTGAATCATTTTGGGCAATATAAAAGGAAATTCAACAAGGTGACATGGCTAACCCCTGTAATAAGTGAGCGCTGCCGCTACACTACACTACAATACAAGTAATGAGTTCAATCTTCAATCTCCATTTCAATTTTGTGTATTTTGAAGAGTCAAATGTTATAGTACAGGCGGCGTGACATGACTGGTTAGGAGAAGGGTGCAGGTTGGAGAGCACACAGAATCCACATGATTTACAGTCAACTTGACTTAACCCTTTTCTTTTGCTGATCTAAGCAGTGACAGAATCACTATCCCTGCTTCCAAGTTGCAAGTTCCAAGTGCCAAATGCACACGTGTCAATTTAATTAACCCTCCCTATATAGACCTCAGCTTCTCCAAGCCTCTCTCTCACGTTCCAACTTCTAAGTTCTAAGTCGCAAACAAGAAGAAAAAAAGAAAGGAAGAGGCCGCAATGAAGAGTGAATTGGAATTACCACCTGGATTCAGGTTCCACCCCACTGATGAGGAACTTGTGAATCACTACTTGTGCAAGAAATGTGCTTCACAGTCAATTGCTGTTCCTATCATCAAGGAGATCGATTTGTACAAGTTTGATCCATGGCACCTTCCAGAGATGGCTCTATACGGCGAGAAAGAGTGGTATTTCTTCTCTCCCAGGGACCGCAAATATCCGAACGGATCACGCCCAAACCGGGCTGCGGGTACAGGGTACTGGAAGGCCACAGGTGCCGATAAGCCCATTGGAAAGCCCAAGGCCCTGGCCATCAAGAAGGCACTGGTGTTTTACGCTGGCAAAGCCCCTAAAGGAGTGAAAACCAATTGGATCATGCATGAATATAGACTCGCTAATGTTGACAGATCCGCAGGCAACAAGAAAAATAACTTAAGGCTTGATGATTGGGTGCTATGCCGAATTTACAACAAGAAAGGAAAGATTGAGAAATACAACCATCTTGGGGCGGCGGATCACAAATCAGCATCTTCGTGGGAGGAGAATGAGAGGAAGCCGGAGGTGAAGGAGCGATTGCATATGGATACGTCGTCGGATTCGGTGGTATCGGCGGATGTGACGTGGGAGAGTAGGGAGGTGCAGAGCGAGCCAAAGTGGACTGACCTGCTTGACCAAGTCTTTGATTTCCAGTTAGGCAGTTTCGTTGATTTCTCATCGGCTGGAGATGACCCTTTTGCCCCCCAGCTCTCTCCTTGGCACCAGGACACGTTCATCACATTTTAATTCATTCTTTCCAACACAACAAGAAAAACTAAGATTGTTTAGTTTAGCCACCATGACTCATTTGGATTTAACTCGGGAACCAAGGACGATGAGATCATATATGTAAAAATAAATAAGAAAAATAATCTCACCAATGCCAGCTAGTTTGTTTTCATGTTACCATTGTTGGAGTGTTAAGCATCTACTCCTGTGGAAAATTCTTCCTACCATTTTTTAAAATATATGTTATTATTTAATTCAACATATTCAAGGAATGATTTCGTTTATTCATGTCTGTTTTTCTTTAATTTTTTTTTAAAATAAAAACAACTTTGGTTAATCGATTCTATAACTCAGTACTAGATTATTGATGTAAAAGGAAATGGTGGCAAAGCGAGCACGAGCACACGTACACAAGGAAAAGTTTCTAACGTTGAAGAAAGATTTTGATTTAGCAAACATGCAACGTATGAAACAATGTGCAAGAAATGCACCTCCCATTCCTATGAGCAATAACCCTTGTCCAGTCCATCCTATTAAGTACCTTTCAATTCCATTCCTTCTTATTCAGGTTCTAAAAACCCAGTTGTCCAACTCATCGAACCACTACGTTTAGAAGTTAAAAATGCAAAAGTTCAATTGAAGTTAAACCAAATATAATATTGAGAAATAAGGCACAGGATTCGGTGGTCTAGAAACCTTTAGACTATTTAGTGGTCCAAGTAGAAAACATGTTTTTAGAGTTTTTTTATCAATTGTTACGTAGTTCTTGAACTAATTT

## >AhNAC150-Arahy.WULW7H.1

TTCTTCTTCTTCTTCGCTTTCCGATCCTAATATAACCCCAATTTCTCTATTCCACTATATATATCGCCGTTTAAAACGCGTTGAATTCAAAAAGATATTTAGTTTGATTGTGTTAGATGGAAGAGCCTGTCGTAGTTAACAAAGGCGAGGAGCCGCTGGATTTGCCACCAGGTTTCAGATTCCACCCAACAGACGAAGAAATCATCACTTATTACCTCACCGAGAAGGTCATGAACAGCAGCTTCAGTGCAACTGCCATAGGTGAAGCCGATTTGAACAAATCCGAACCCTGGGATTTACCAAAGAAAGCAAAGATGGGAGAGAAGGAGTGGTACTTCTTTTGTCAGAAGGACAGGAAATACCCGACGGGGATGAGGACCAATAGAGCAACGGATTCCGGTTACTGGAAGGCAACCGGAAAAGACAAAGAGATTTTCAAAGGGAAAGGGAATCTCGTTGGGATGAAGAAAACGCTTGTTTTCTACCGAGGTAGAGCTCCCAAGGGTGAAAAGACCAATTGGGTCATGCATGAGTTCAGATTGGAAGGCAAATTTGCCAATTATAACCTCCCCAAGGCTGCAAAGGATGAATGGGTTGTGTCGAGGGTTTTTCACAAGAACACAGATGTAAAGAAGACTACTACCCCATCATCATCATCATCAATAATTCCTGGCCTTTTGAGGATCAACTCAATAGGCGATGATCTTCTAGATTGTTCCACACTCCCTCCTCTCATGGACCCTCACCCTACTCCTCTCGATACAACAAAATCCGACGGCTATTATTTCCCCTCCTTCTCATCATCACATCAGATTCTCAATATCAAGCCCGAAGAACACAACACAAGCCACCAAATTCCCATCACCAACTACCAGATTCCAAATTTCAATACCACACTCTCTTCTTCATCATCTCATCAAATCAGACTCCAAAATCATCTCAACTTGTTCTCATCATCATCATCAAATAACAATTACCATAATAGCTCGTGGCCAAGCTATTATGATGAGGTCCACCACCATCATCAAGATGATATTCTACTAAGAGCAATCGCCTCAAAGAACTATAGCAACGGAGGAGGAGGAGGCGGCGGCGAGTGCAAGGTGGAGCAATTCTCCTCCGGCAACCAGTCAGTGGTGAGCGTTTCGCAAGACACGGGGCTGAGCAACGACAGAACCACCAATGACACGTCATCGGTGGTTTCGAAGCAGCAGCATAATAATAAAACATTGTACGAGGATCTTGAAGGTCCTTCTTCATCAGTTGCACCTCTTTCAGATTTGGAATGCTTGTGGGATACCTACTGAAAATTCATCAACATCAAGTTTCCAATCCTATCCGTGTTTTTTTAATTCATATTCATATAAGTATCATCATGCATATAGTGATTTACCTATATACTATGTTGCTGGTTTTTCTTTTTTTAAAATCAGGCTTCTATACTGATTAATTATTTATATATTATTATTATTGTTATTATCATATATACATATATCATTGTAGGATTAAGGAGGAAACTAAAGGTTATTGGATACACATATATACTGTATAGATTTATGATATCCATGACTCTTTTTTTTCTTAAGTGTTAGGTTTTCCCATCATAGGTTATATCGAGGACAAGGACCTGGTGAAATCATGTAAATCTATATATTCTTTTGTTATATTAGTGTGTTTGTTTTATTCTTTTCTTTATTCTATTTTTTTTTTTTTTTTGTTCAAGGTTCAACTCATGACTCTTGTATACTCCTCAGAAATTTCGTTTGATGCCTTTGGAGAATTATTACTACAATTATTATTTATTATTATTATTATCAATTATTTGTTCCTCTATATATTCTTTTAATTGCAAAATATTAATCTTGGCAGACTCATTTTAATTTTGAGCCGTTCCATTCTTGCTGCAGCAATATATTTATATTTATTTTATGCTGTTCGAATATATATATCTATGTGGCACCCCACCAAGAATGGGAAGCATTAATTCCTAAGAAAATTGATGCATTATTTTTCTCTCAACTTGTGAATAAAATGTTCTAGTTGCTTAGCTGCTAGCTAGTGCCTCCCACCACTACTGTTATTATTTCCTTTGCAAATTTGGATTTGATCGAGAAATGGTACGA

## >AhNAC151-Arahy.X47CQ0.1

TAATATTAATTCTTAAAAACTTAAAAATGCCCTTAATATCAATTCCTTAGTTTAATATTTTATTGTATATCATATTACCTAATTAAAAGTATAGCGGTATTGGCTAGTTGAAGACACCACCACATACATAAGTAATGGACAATGGTCAAACCATTAATAATAGTGAAGGTTGAAGAAGAGGGACTTCGGCAATATAAATAAATAATCATAAGAGGAGAAATATTATTTCTTTGAGGGAGGGACAAAAACAAAAAGAAATTAGGTGGAGACATGGAGGGAAAAGGATTGAATCATTTTGGGCAATATAGAAGGAAATTCAACAAGGTGACATGGCTAACCACCTGTAATAAGTGAGCGCTGCCGCTACACTACACTACAATACAAGTAATGAGTTCAATCTTCAATCTCCATTTCAATTTTGTGTATTTTGAAGAGTCAAATGTTATAGTACAGGCGGCGTGACATGACTGGTTAGGAGAAGGGTGCAGGTTGGAGAGCACCCAGAATCCACATGATTTACAGTCAACTTGACTTAACCCTTTTCTTTTGCTGATCTAAGCAGTGACAGAATCACTATCCCTGCTTCCAAGTTGCAAGTTCCAAGTGCCAAATGCACACGTGTCAATTTAATTAACCCTCCCTATATAGACCTCAGCTTCTCCAAGCCTCTCTCTCACGTTCCAACTTCTAAGTTCTAAGTCGCAAACAAGAAGAAAAAAGAAAGGAAGAGGCCGGAATGAAGAGTGAATTGGAATTACCACCTGGATTCAGGTTCCACCCCACTGATGAGGAGCTTGTGAATCACTACTTGTGCAAGAAATGTGCTTCACAGTCAATTGCTGTTCCTATCATCAAGGAGATCGATTTGTACAAGTTTGATCCATGGCACCTTCCAGAGATGGCTCTATACGGCGAGAAAGAGTGGTATTTCTTCTCTCCTAGGGACCGCAAATATCCGAACGGATCACGCCCAAACCGGGCTGCGGGTTCAGGGTACTGGAAGGCCACAGGTGCTGATAAGCCCATTGGAAAGCCCAAGGCCCTGGCCATCAAGAAGGCACTGGTGTTTTACGCGGGGAAAGCCCCCAAGGGAGTGAAAACCAATTGGATCATGCATGAATATAGACTCGCTAATGTTGACAGATCCGCAGGCAACAAGAAAAATAACTTAAGGCTTGATGATTGGGTGCTATGCCGAATTTACAACAAGAAAGGAAAGATTGAGAAATACAACCATCTTGGAGCGGCGGATCACAAATCAGCATCGTCGGAGGAGGAGAATGAGAGGAAGCCGGAGGTGAAGGAGCGATTGCATATGGATACGTCGTCGGATTCGGTGGTATCGGCGGATGTGACGTGGGAGAGTAGGGAGGTGCAGAGCGAGCCAAAGTGGAATGACCTGCTTGACCAGGTCTTTGATTTCCAGTTAGGCAGTTTCGTTGATTTCTCATCGGCTGGAGATGACCCTTTTGCCCCCCAGCTCTCTCCTTGGCACCAGGACACGTTCATCACATTTTAATTCATTCTTTCCAACACAACAAGAAAAACTAAGATTGTTTAGTTTAGCCACCATGACTCATTTGGATTTAACTCGGGAACCAAGGACGATGAGATCATATATGTAAAAATAAATAAGAAGAAAAATAATCTCACCAATGCCACCTAGTTAGTTTTCATGTTACCATTGTTGGAGTGTTAAGCATCTACTCCTGTAGAAAATTCTTCCTACCATTTTTTAAAATATATTTTATTATTTAATTCAACATATTCAAGGAATGATTTCGTTTATTCATGTCTGTTTTTCTTTTATTTTTAAAAATAAAAGCAACTTTGGTTAATAGATTCTATAACTCAGTACTAGATTATTGATGTAAAAGGAAATGGTGGCAAAGCGAGCACGAGCACACACGCACACAAGGAAAAGTTTCTAACGTTGAAGAAAGATTTTGATTTAGCAAACATGCAACGTATGAAACAATGTGCAAGAAATGCACCTCCCATTCCTATGAGCAATAACCCTTGTCCAGTCCATCCTATTAAGTACCTTTCAATTCCATTCCTTCTTATTCAGGTTCTAAAAACCCAGTTGTCCAACTCATCGAACCACTACGTTTAGAAGCTAAAAATGCAAAAGTTCAATTGAAGTTAAACCAAATATAATATTGAGTATGGTTCTTATTCCGAAGGTAGAAAACCCGGTTTCCATGAAGGATTTCCGACCAATTAGTCTCTGTAATGTGGTTTACAAAGTTATAACGAAGGTCCTGGTCAATAGACTTCGTCCCCATCTCAAAGAGATTATTGGGCCCCTTCAAGGAGGGTTTATCCCGG

## >AhNAC152-Arahy.XB6K25.1

AATAATAATAATAATAATAATAATAATAATAATACTTATTATTATTATTATTAATCCCACCCCATGAAGCCCTTATAAACCAAGCACCCTCCTCCATACCAAAAGGCACCACATACAGCAGAACAACAGAAGAAAGAAAAAAGTGATCCAAACCCCATTATTAATCTCCTTTCACTCTTTTGCTCCTTCTGCTTTTTCTTCAAGTTCTTTCCATTCTCATACAATCCAAATGGCCCCTTAAGAAATACAAATAGATCGAAGAAGAAAGAAAACACAACCACCAAGATTCTCCTCATTCATTCATTCCAACAAAAGGATCTTTACTTAGTTCCTTTTGTTCCTCCCCAAAAAAGAACACTCTTTATTTATTTTCCTTTGAACACTCTTCACCATGGACAGCTTCTACCACCACCATAACCACCACTTTGACAACAGCGACACTCACTTGCCACCTGGATTCCGATTCCACCCCACCGATGAAGAACTCATCACCTACTACCTCGTCAAGAAGGTTCTCGACAACACCTTCACCGGAAGAGCCATAGCTGAAGTTGACCTCAACAAGTGTGAGCCATGGGAGCTCCCTGAGAAAGCGAAGATGGGTGAGAAAGAATGGTACTTCTTCAGCTTAAGGGACAGGAAGTACCCAACTGGGTTGCGCACAAATAGAGCGACGGAAGCTGGGTACTGGAAAGCCACTGGAAAAGACAGAGAGATCTACAGCTCCAAGACTTGTGCTCTTGTTGGAATGAAGAAGACACTTGTTTTCTACAGAGGAAGAGCTCCCAAGGGTGAGAAGAGCAACTGGGTGATGCATGAGTATCGCCTTGAAGGCAAATTTGCCTACCATTATCTCTCTAGAAGCTCCAAGGATGAGTGGGTGATTTCTCGTGTGTTCCAGAAGACAACCACCGGGGGTGGCGGTGGAGGCGGAGGCTCAGCCGTGTCAACCACCGCCGGCGGGTCCAAGAAGGCAAAAATGAGCACTTCAACCACCACTTCTACAATGAGCTTCTGCCCTGAACCAAGTTCTCCCTCTTCGGTTTACCTTCCACCACTTCTCGACTCCTCACCTTACACTACCGCCACCACCGGCTCCGTCACCTCCGCCGCCGCAGCATACGACGGCCGCCAGAGCTCCTCCTTCGACAACAACAACAACGATAGCACAAGGGAGCACGTGTCCTGTTTCTCCACAATCTCCAACAACTTTGTCAATGGGTTCTTCGATCTTGCTCCTATGGACTCCTTCGCTCGATTCCAAAGAAACAACAATGTCGGTGTTTCTGCATTCCCAAGTCTAAGGTCTCTGCAAGATAACCTTCAAATTAACCCTTTGTTTTTCTCCGCCGCAGCGGCGCAGCCTCTCCACGGCGGCGAGCTTCACGCCGCGGGGGCCTGGCCGGTGCCGGAGGATCAGAGGGTTGCCGAGGCTGCTGCCGCCGGCATGGCTTTGGGACATTCCGAGCTTGATTGCATGTGGGGCTATTGAAGTCTTTTCCCATTGACCCTTTCAAGCGTTGACTTTCATGGTTAGGATTTGACTAAGAAGTGTCTTTCTACTTTCTAATGCTTTGTTATTATTAGGGTTTATGTTAGACTTGAATTACAAGTTAGTAGTACTTTTTATGGTAGCTTATTAATTATAAGATCATTGCTAATGCTAATTATTCTATCTCTCTCTTGCTAGGGTAGTTTGAATTCGGTTATTGTAACGTGTTTGGGTGTTGAAGTTTAAATTCGGTTACTCTAATGTGTGTAATTTCTTAGGGTACTATTATTGTTATTGGAGTACAGTGTACTGTAATATAATGTATGTGGACCACTAGAAGTGTGTTGTAATTGTCTCTAATGTGTTTGGGAAGTGTGTTTTATGTGATATGTGTATTTAGTTCATAAACCATGGTGTGTGGAGTGTGGACCAGTCATGTTTTTTCTTATCTTACTTAGAACTGATTTTGTATTCTCTCTGACCTATATGTTAGTGAAATTAAATGCAATGTGTGAAGTATTATTATCAGAAAAGTGTGCTGA

## >AhNAC153-Arahy.XKF840.1

AAACCCCAAGCAAACCCCACATTCCAACCCCCCTCTCCTCTTTTGCTCTTTCATCTATTCTCTCCTTAGCCTCTCCCACACTTTCTACCTTTGGATCTTCTTCTTCCCCAATTGAGTTATACTTTGTACTTTTGTTTATAAGAGGAAGAAGGAGGTCCAAAGCTAATTAGGTCATTCGGATTCAAATTATATATAGAGAGAACATATGGCACCAGTTTCATTACCCCCAGGTTTCAGGTTCCATCCAACTGATGAAGAACTTGTTGCTTATTACCTCAAAAGGAAGATCAATGGCCGTAAGATTGAGTTGGAGATCATTGCTGAAGTTGATCTCTACAAGTGTGAACCATGGGACTTGCCAGGGAAGTCATTGTTACCGGGGAAGGATTTGGAGTGGTATTTCTTTAGTCCTCGAGATAGGAAGTATCCAAATGGGTCAAGAACGAACCGTGCAACAAAATCTGGGTATTGGAAGGCGACAGGGAAGGACAGAAAAGTAAATTCACAATGTCGTGCTGTGGGTATGAAGAAAACCCTAGTTTACTATCGTGGAAGGGCACCTCATGGCTCTCGCACTGATTGGGTTATGCATGAATATCGTCTTGATGATAGAGAATGTGAAAATGCTTCTTCTGGCTTGCAGGATGCATATGCACTTTGTCGTGTGTTCAAGAAGAGTGCAGTTATAACCCCTAAAGTTGATGAGGAACATCATCATCACCATCACTATGTTAATGCTAATAATCACAATAATAGCAGCCATGCTTTGCCAATTACAAGTGATCAATCGTCAAGTATGGAGTTATATTCTGAAGGAAGGGGTGAAGATTTGGATAATAGCTCTAATTATTTGGTTCCCATTGATACTACTTCCACACTACCCCTCAACAACATGGTGATGAACAATAATAATAGTGATGCTTCTTTCAATAGTAGGGATAATAATGGAAAATGGTCACAATTTATTTCAGAAGATCCATTGTTCAGCTTTCCAACTTCCTCCTCATCATTTGCTAATAGTTATGGATCTATAACATACCCTCCATCCAAGGTGGATATAGCACTAGAGTGTGCAAGGATGCAACATAGGTTCACCATGCCTCCATTGGAGGTAGAGGACTTCCCTCATGTTGGAACCTCGGAGCTGAAAATGACAGAATTAGCCTCGGGTGCCGGATCCACCGTGCACGGAACCCGAAACGAAACCGATATCTTGCAGGAAATTCTTTCGGTTGCTCATGCTTCCCAGGAGTTGATAAACCACTCCAGCTACTCATCATCATGGGGTGGTGATGGTGGTGGCAACCATGAAAATTGTGCAACTCATGGAGATGATTTCACTTTCATGGTTGGTAGCACTAACTACAATAATAATAATTTGAATGACATTAACAACATGAGATATGTTGATAGAAATTGGGAAGATCCAAACACTTCAAGATCCATTGATATTGGATATTTGGATGAAGAATTTAAGGGAGAGAGGATGGTAGAGAATTTAAGATGGGTTGGAATGTCTACAAAAGATTTAGAAAAGAACTTCACGGAAGAGCAAAAGATTGTTCCAATAGAGGATATATCAAGCTTCCAGACAAATAATAAAGAAGAAAATGAGGTGCAAGAATCTGAGCAACACCATAGCAACAAGGAACTATTGATCAATGATTTCTCATTAGGGTTCAACCCTAATAACAACAACAGCGAGAACTTTCTAGATGATGATCATAACAACATGGATAATGATGATTACTCAAGTTCTCCAAGCTTTGAAGTCATTGAGGAAATAAGGGTCAGCCATGGATCAATGTTTGTTTCAACTCGCCGCGTCGCCGACACATTCTTCCACCAAATAGTTCCTTCACAAACCGTCCAGGTTCACCTCCTCAATCCAGTGATAACAAGCAATGAAGAAGAGACATTGATGATGATAATGGAGAGGAATCAAGGGTATTTCGGGGATTTTCTTTTCAAGACAATAGCAACTGCGTTTGTGCTCATCTTTGAACTTCTATTCATGCATTGTGATTATTTGAAGGAAGAAGTGGAATTGGTGAAGAGAAAGAGATCATCACAATCATCATCTAAGATCATGAAATGGAACAACAATAATAATAAGGTTTGGTTTGTTGGTTTCAAGAGTAGTGAGAAGGGCTTTGGTGCAATTTTGAAGAAAATAGGGATTTTTCTCACAATATCTTTGGCTCTTTGTACCATGTGGGCTAACCATGTTATAGTTAACCCTTGATTTATTCCTCCTAGTTTCGTTTATTTTTGTCTCTACCTTATGGAATTCTCTTGACATGGCTCAAATTAAGTTTGGGCTTTTTCCTAATTAAATTAAGTAGGAGGCTGATGTATCCATATACTTATTATAATTTTACTTTGACATATGTTGTTTGATTAATATTATAGATAA

## >AhNAC154-Arahy.XMHS8A.1

GCGCATCCGACACACACACAATTTTGGGCTTCTTTTTATTTGTATCATTTGAAAATTTTGGATGTTTCTCAGACAGAAAGAGACAGACCTGCTTTTGAATTTGTGACATTGGTTTGAATCATCAAAAACCTCTCTTCGTTCGATGGCAGGAGCATCATGGTTGGTGGACAAAAGCAGAATTGCAACCAAAATCAAGAGTGCATCAGGAGCTTGTGATCCTAACGAAGTTATTTGGAAAACCAATCCTACCAGGATTTGCCCGAATTGTCATCATCCTATTGACAATAGTGATGTAACTCAAGAGTGGCCTGGCTTACCAAAAGGGGTTAAATTTGATCCGTCAGATCAAGAGATAATCTGGCACTTGCTTGTAAAGGCTGGTGTAGGAAATTTAAAACCTCATCCTTTCATTGATGAGTTTATTACTACCCTTGAAGTGGATGATGGGATTTGTTATACTCATCCTCAACATTTACCTGGTGTCAAGCAAGACGGAAGGGCCTCACATTTCTTCCACAGAGCAATAAAGGCTTATAATACCGGTACTCGAAAGCGTCGAAAAGTACATGGTCAGGATGACGTTCGTTGGCACAAGACTGGAAGGACTAAACTGATCACCCTGAACGGGGTTCCAAAGGGTTGCAAAAAAATCATGGTTTTGTATACAAATGCGGTGAGAGGAGGAAAGTCAGAGAAAACTAATTGGGTTATGCACCAATATCACCTTGGGACAGAAGAAGATGAAAAGGAAGGAGAATATGTTATTTCTAAAGTGTTTTATAAAGAAGACCAGGATATACCTGAAGCCGCAGAAAGTAAAAATGCAACAGTTGCGAAAGTAGATCCAGTCACTCCCAAATCCACGACTCCTGAACCTCCTCGTAATGAAAGGCAAGATTCAGATCTAGGCCTGGATCTAGACCTAGGGCAAGAAGCACTTGTTTTTACTGAGATGGATTGCTTAGATGAAATTCAAGCTGACTGTGAAGAATCTGCGAAAGCTAATCCACCAGTACTGGAGACACAAGAAAATGAAGGGATGGACAACAAGGAAACTAATGCTTATGAAGCACAACCGTGGTGGGATAGTGATTCACAGAATCTATTAGATTCACAACAACTCGTTGAAGCATTAACTCTCTGCGAAGATATATTTCACAGCCAATCTTCCAACAAAGACGATGAAAATGATAAGAACCAAACCGGTCTATCTGTGTATGCTCATCTAGGACCAGAGCATCTGAAGAAGGATATTGAGGAGTGTCAAAAGCTTGCTCCTGCAGGACCAGAGCATCAGAAGGATATTGAAGACGGCCAAAATCTTGACATCGACCTTGCAAATATAGAGCGGGATACTCCTCCTGAGCATCGACTAAGTCAGCTGGAATTTGGTTCGCAGGACAGCTATACTTACTGGGGTTTTCAAGGCGTAAACTAATATCTTCCTTGATCTCTCGGCTTTGAACCAATTTTTTTGCAACTACATTTTGTTGATGTGTTATTTGTCTTTTGCCTTTGTGTTATGCTAAACTATGGGGCAAACTTAACTGTTACGAGTAAGTGGTTACTACTAACTATGCAGTAATGTAAGACTGTGAACCTGATATATATTTTGATCAACCTTGGTGTAAATGTAATTCAAACTTGCAATTCTCGGCTGGTTGAAGTTTTGTTCTATATTCTTGCAGTTCTGGATGTGGACTCTAGAGAGAAAGATGCATTTAGATTTCTACTCTATTCAAGCAGCTATGGAATTATCAAAGTTGCTTTTAGATTTCTGATAATGGCTTAAGACTGAAAAATAGTAATGTTGGCTTTGTTCTACAGTACCAAGTAGTATCAGGATGTGAATTTTATCCAAAAATAATTTCTTCAAGACCAAAAATGATATATAACCAGATTTATGTGAAAATTTTGAATATTACATTA

## >AhNAC155-Arahy.YB9YM9.1

ACAACACATGACGTTCCGTCTTAACGTGATCTTCGTCTGAATCCACCGTAGCCAGAACCAACCAACCAACTAACCAAATCCATAAAAAAAGAGGGAGAATTATTGAAAGGAAAAGGAAGAAGAGAAGGAGAAGAAGAAGAAGAAGAGTGAGAGATCCATAAATATGGAGAGCACCGACTCATCCACCGGTTCGCAACAACCGAACCTTCCACCGGGGTTCCGGTTCCACCCCACCGACGAGGAGCTCGTTGTTCACTACCTCAAGAAGAAAGCTGCATCAGCTCCTCTCCCAGTCGCCATCATCGCCGAGGTTGATCTCTACAAGTTCGATCCATGGGAGCTACCAGCTAAGGCAACGTTTGGGGAGCAAGAGTGGTACTTCTTTAGCCCAAGGGACAGGAAGTATCCGAACGGTGCTCGGCCAAACAGGGCGGCAACTTCCGGGTACTGGAAGGCAACCGGGACGGATAAGCCGGTGCTGACCTCCGGTGGGACCCAGAAGGTGGGTGTGAAGAAGGCTTTGGTCTTCTATGGAGGGAAGCCACCGAGAGGGATAAAGACAAATTGGATCATGCATGAGTATAGACTTGCTGATAACAAACCTAACAATAGGCCTCCTGGTTGTGACTTGGGTAACAAGAAAAACTCTCTAAGGCTTGATGATTGGGTATTGTGCCGAATCTACAAGAAGAACAACACACATAGGTCTCCAATGGAACATGAGAGGGAAGATTCTATGGATGACATGATTGGAGGGATTCCTCCTTCCATCAACGTGGGGCAAATGAATGCAAGATTTCATCTCTCAAAAATGTCAACAAGCTTTAGCAACGGTTTGTTGGAAAACGACCATCACCATCACCAGAATCTTCTGGAAGGTATGATGCTAGGAGGAGGAAACAACAACAACAACAATGTTGTTCCTCCAAACATGTTGGGGTTGGGATCAGCCTCAAACACCATTAACAATAATAGTAATAAGGCAGAGCTTTCATTTGTACCAACCATGACTACATCTTCAAACACCAAGAGGTCTATCATCTCTCTATTGGAATGAAGATGATGTTGCTGCTTCCAACAAAAGATTCAATTTGGAAAGTGGAGATCATAACCATGGAGAGAATAATGGTACTAGTGCTAGTTCTATTGCTACTCTGCTGAACCAACTTCCTCAAACCCCTTCATTGCA

## >AhNAC156-Arahy.YH9HLJ.1

GATCTAAATAATTATTCTATCAAAAAATAAACTGAGAACTGATATGTATGAGATGTGAGAGTAAAAGAATTTATTTACGGAAACAAAATTACTAAATTCCCATAGACACCTAAAAAATGGTGGTGTAGTAATATGATGGATTTGATTGAGCACACGTGTGACAGCGGGTATTTTGCTGGATTTGCACGTGTGAGGAAGGCACCTCCCTTGGTCCGTTGGTACATTTATCTGTACCACAAAGTCCCATACACACTGAATACTCATCTATCTGATCCTACCCCACCCACGCATCACGCATGGATTCATGTCAACCCCAACTCCCACCGGGATTCAGGTTCCACCCAACCGACGAAGAACTCATCGTTCACTACCTCAAGAGAAAAGCTTCCTCTGCTCCTCTCCCCGTCGCCATCATCGCCGACGTTGATCTCTACAAGTTCGACCCATGGGAGCTTCCAAGTAAGGCCACGTTTGGGGAGCAAGAATGGTATTTCTTTAGTCCGAGGGATCGCAAGTATCCGAATGGGGCTCGCCCAAACAGAGCAGCTACGTCCGGATATTGGAAGGCCACCGGCACTGATAAGCCTATTATTGCGTCTGATGGCCAACACCGACTCGGCGTCAAGAAAGCTCTCGTCTTCTATGGTGGCAAGCCTCCTAAAGGGGTTAAAACCAATTGGATCATGCACGAATATAGACTCACTACTACTCATAACAACAATTCTATCTCATCATCAAAGTCTTTTCCTTCTCTTCCTTCTCATCTTCCTTCCGCCAATAACAAGAAGAATTCCTTGAGGCTTGATGATTGGGTGTTGTGCCGAATATATGAAAAAAGCAACCGTGGCAATTTTGCAAGAACAGCGTTGATGGAGCACCATGATCATGATGATGATGATGATGACAATAAGGATCAGCTTTCCGCGGAAACAACGAGTATGATAGAAAACATGTCCACGATGAGTAGTCAGAATTCCAAGCCCACACAACATTATGGACCATTGCTGGTTCAAAACGATGACAACTTCTTCGATGGAATCTTAGCTGCTGATCATCATAATCAACAACACAACTTGCCAATGAAGAGGACACTGGTGAATATGAATAATTCACAGTTTTGGAATGAGACAAACAAGAGGTTCCATTGTGATCTCAATAACAACACTAACATTGTTGCTAATAATGATGAGGATAACACTTCCTTTGTTTCACTGCTTAGCCATAATCAGATTCCTCATCATCCTACTAACAATGCTTCTCTTCTTGACCCTACTGTTGCTGATGGTGTTTTCAGGCAACACTTTCAACTTCAAGCAATTAATTGGAACTTATAGATTATATTCTCATCTGACTAGCTAGCTAGCCATATATTATATGTAGTGTGATCAGAGTGGTCTAATATCATTGATTATATATACAGTACATGTAATTAGCTATTTTATTGTCACTTTTCTATTTGAAGCTTAATTAATTAAGCTATACAATGTTTGTATACAAAATATACTATTCTATGATGTAAGTATACCTATATACCAAGCTTATACAAAATTAGCTCTTGCATTTTGAGTAGTGAATATGGTGCCTCTAGTTAATTTACTATGTTAGCTTTCTCCAATGTTGTTAACAAAAATGTTTGGGGATATAATTTGAACATTATAGATAGCTATAACT

## >AhNAC157-Arahy.YSLF5V.1

TATTGCACTGATCCACACACACATTACACATCTCCCTCTCTTTTTCTCTCCTAAAAGTTAAAAGCCACATACATACATAGATACATAGATAAGGTCCGTGCCCTTGAAGCTAAGAAACTGTGCAAACTAAGCCTCCTCTTTGCTTGTTTGTCCCCTCTTTCCTGCGAGCTTCTTTGTCTTCTTTCCTTTGTCCTCAAACGCTCCTTTCCCTTATTGCTTCTCACTAAAGGTAATTAATTAGAAGCAAAAGAATAATCAGAATGATGGCAGGTAGTGGACAACTAACAGTTCCACCAGGGTTTCGGTTCCATCCAACTGATGAGGAGCTTCTCTACTATTACCTAAAGAAGAAAGTTTCTTATGAAGCCATTGACCTTGATGTCATTAGAGAAGTTGATCTCAACAAACTTGAACCTTGGGACCTCAAAGATAAATGCAGAATAGGATCAGGGCCTCAAAACGAGTGGTATTTCTTCAGTCACAAAGACAAGAAGTACCCAACAGGAACAAGGACCAATAGGGCAACCACTGCTGGTTTTTGGAAAGCCACTGGGAGGGACAAGGCCATATACCATACTAACAATTCCAAGAGGATTGGAATGAGGAAAACCCTAGTTTTCTACATCGGCCGTGCGCCCCACGGCCAGAAGACTGACTGGATCATGCATGAGTACCGCCTCGATGAAGACGACGCCGAAGTTCAGGAGGATGGGTGGGTGGTGTGCAGGGTTTTCAAGAAGAAAAACCAAAGCAGAGGGTTTCAACAAGAAATTGAAGAAGAGGAACATCATCACTTAGCAGCAGAACATCAACACATGAGAGGAGTAGCAAGCCAACAAGTTCTGGACCCAAAACACCACCACCACTTGCAACATCATCAAGGACTCTATGATAATGATAATGATAATAATTACACCAATAATTTTGATGGATCCATGCACCTTCCACAGTTGTTCAGTCCAGAATCTTCCGTGGCTACCGCGGCGGCGCACACTTCCATGAATGCCATGGACATTCTTGAATGCTCCCAGAACCTTCTAAGGCTCACAACAACAAGTGGATGTGGACTCAATCTCATGCAACAACAACATGGAGAGAGGTTCAATGGTGATTGGTCTTTCTTGGATAAGCTTCTTGCTTCACACCATGGCAGCACCATGGATCATCATCACCATCATCATCATCATAGCAAATGCAACAATAATCTTCATCATCAGCATCCTGCAATTGCTATTGGAACTACTTCATCTCAGAAATTCCCATTTCACCACCTTGGTTGTGACAACCATGATATCATGAAGTTTTCCAAGTAGGGTTGTTATGAAAAATAATAATATAATAATAATCATTATTATGATCATAAGCGATGATGAGGTGAGAATCAGAAACTTCCTTTCTAGTATATGTATTTCAGAGCATCCTCCTGTTTGTTAGGTACTCATTGGAAAAATTCTCCCACTCATTAATATTTATTAGCATTTCTTTATTTTGAGTATCAATTGTGTATAGGTATGTATCTTTGTTATATTTTCAAGCTGCTGTTAAATAATATGATACTATTCATGAATGCTTATTTTTCATGTTGTTGAATACCATGGCGATCTACTTTC

## >AhNAC158-Arahy.YV5C93.1

TTTAGTGTAAAAACAAGATTGTTAAATTAATTAAATATGGTAAGATCGCAATCCCCAGATGCTGCTAGTTTGGTGGTGTATGGAGATTCCAGCTTTTGGGTCATAGACAAATAACACACAGCTCTTTAATTAAGTTGAAAGTGTGAATGAATAGTGGTACTTAATGATGAAACTAATGCGACTTAATTGGGTTTGATTTCTAATTAAGTTGGTAGTTGTTCTCTTTTTAAGAGCTTCATAGCAACGCGATCCTCGCAAAACACACGCTCCTTTAAGAGTCACCACAAAAGACGGCTGAACCTTCGGGTACAAGATCCCTTCTCAGGAAGCATATAATCACTTCACTACTTCAACTGTGTTCTCTCTCTATCTATCTATCTCTTCATTATTAGGGTTTTCATTTCTTGATTATTACCAGCTTGCTTCTTCTATATATCCACCTCTTTCAACTCTCTACTCTTTCTTGAGGAGATGGAAGGTGAGAAGCTTGATGAGATCATGTTACCAGGTTTCAGGTTCCACCCAACTGATGAGGAGCTTGTGGGGTTCTACCTTAAGAGAAAGATTCAGCAAATGCCTCTGTCCATTGAGCTCATCAAGCAACTTGATATCTATAAATATGATCCTTGGGATCTTCCAAATCTTATAAGCAACGACTGTTTAATACCCTCTTCTACTACTACTCCACTTGAAACATCCTCTAATAGTGCAAAACCTACTATGGATTTTTCTTCATTGTTGCTGAACATGTCATCTTCTGTTCTTGGAGATTTTGCTGGAAAGACATCATCGTCGTCCTCATCCCAAGAGGGTACAGCAGCAACAGCAACAACAATCACAAGTAGCTTCGGTGGTGGAATGCAGGAGCACTACCCAACAATACCATTACTGCGTCAGATGCATCAAGGGAACAACAACAACAACATTGGCATCAACAACAACAACGTGTCTGCTGGCGGTGAAGAACAAGAGTTGGAGAAAGTTGGATCCATTGTTGGGTTCCCATTCATGAACATTGGGGATGCATGGAAGTCAAATATGCTTTGGGATACTTCTTGTCCCTTGTGA

## >AhNAC159-Arahy.YXGX3A.1

ATGGAGGATCCACCAACTGGTTTTCGGTTCTATCCAACAGAAGAAGAGCTAGTTGCTTTCTACCTAAACACCCAGCTTCAACTACAAGGCCACACGACTCACATCAACAGGGTCATTCCAGTGGTTGACATCAATGGCGTTGAGCCCTGGACTCTTCCATCACTGGCGGGAGAGCTGTGTAGGGAAGAGAAGGAACAATGGTTCTTCTTTGTGCCTCGCCAAGAGAGGGAAGCCAGAGGGGGGAGGATCAACAGAACCACTGCTTCTGGTTACTGGAAAGCCACCGGATCACCGGGGTACGTATACTCTTCCGATAACCAAGTGATCGGAGTGAAGAAAACCATGGTTTTCTACAAAGGAAAAGCTCCCACCGGTCGCAAAACTAAATGGAAGATGCATGAATACCGCGCCATCGTTCAAGCCCCTAACCAATCTCCCACGGCTATTCCTCAGTTGAGGCACGAATTCAGCTTGTGTCGCGTGTACGTGATATCCGGAAGCTTCAGAGCATTTGATCGACGGCCACGGGAGGTGGTAGTGCCAAGAGTTCTTCATCATGGTTCTTCTACAACAAGTGCTCAGCAGCATCAAGGAGAATCATCAGCAAGGGTGCAGGCCAATAATAATAATAATGGGTCGAGCTCGTCGGAAACTTCCCTTTCATCAGGTGGTCCTGATTTGCCACCAGATACTGGAGGAGGAGGGTCATGTAGCAATTGGAATAGTAGTGAGGTTCAAGTTCAAGCTCAAGTTCAAGAACCACTATGGGAATGGGAACAACTCGATTGGCTATAAGCATGACTAATAAGAATATCAATTCGTCAAGCCATGCATGCATTTCATCTCTCATCAAGACCCCTTCTCCACCACTTCAATAATTCCTCTGTTTTAAATTAATGGTCGCTTTCACTTTTTCGTTTTAACTTCATTTATTTAGAAATAGTAAAATAGGGTATTTGTATAATTAACATGTGTAACACTATCAGGATTTGATTTTCACCGAGAAAATAAAGTAGGTGTAACTCTAATTAAGGAGAGATCATGTAATATTAGAACTTAATTAGAAGCTAAAGTATAATTTTTTACTTTTTTTAAGCATATTGTATCATTTTATTTAGTTTGCATAAAGGTTAATTTGTAACCAAATTCTTCTCATTCAAAAATTCATATATAA

## >AhNAC160-Arahy.YY4A03.1

ATTTGGTGATTGGTCTGAGCCGTTACAATCAGATAGAGAGAAGATGAATACATTTTGTCATGTTCCACCGGGTTTTAGGTTCCATCCGACTGATGAAGAGCTCGTTGATTACTACCTTAGAAAAAAAGTTAATTCAACTAGGATTGACCTTGATGTCATCAAAGATGTTGATCTCTACAAAATCGAACCCTGGGATCTTCAAGGATGGGTTGTGTGTAGAGTGTTCAAGAAGAGAGTGACTTCCATTATGCGTAAGATGAGTGATCATGATTCCCCTTCTTGCACTTGGTATGATGACTCCTCTTTCATGCACCAACAACCAGATCATCACTTTGACAACTCTTGTTCTTCTTCATCAAAGCACCAACTAATCCCTAATAATAACTGTGATGTCTTCTACCAACAACACAACAACAACAACTTGCCTCTTCATCATCTTCCACTTCTTCATCAAAATAACAATAATCCAATCATGGCACCACCATTTGCTGCTATTAATAATAATGAAACTACTGCTTTTCAAGAACAAGGGAAAAGCTTAATTCATCATCAGGCACTACTCTATGGAAATTTAAATGAAGAGCAAGCTTCTTCTTCAGCTGCTGCTGCTGCTGCTGATTGGAGACTTGTTGACAAGTTTGTTTCATCACAGCTTAGAGAAGATCATCATGTCTCCAAACAAGAATTGATGATGCCAGAAAATACTAATAATAATAATAATGATAATGGTGCCTCAACATCAAACTCAAGCTGTCCAATAATG

## >AhNAC161-Arahy.Z03JM7.1

TGCATGCTATCATATATACTATCTATGTGTCGCTTTTCTCTTGTATAAATATAGTTACATGTTAGCTTAACTGAATTTCATCGATGTCCATGCATGTATAGTGTCACATAGAGTAGAGAGAATATAATACAATCAAATAATGGAAAAGCTAAATTTTGTGAAGAAGAACGGGGTAAGTAGAATGCCTCCTGGATTCAGATTCCAGCCAACGGATGAAGAGCTTGTGTTTCAGTATTTGAAATGTAAGGTCTTCTCATTCCCCTTGCCCGCTTCCATGATTCCTGACATCAATCTCTCCAACTATGATCCTTGGGATTTGCCAGGAAATTGTGATGAACATCAAGAGATGTATTTCTTCAGCAGCAAGGAACCCAAGTATAGAAATGGAAGCCGCATGAACCGAACAACCACCACTGGCTATTGGAAGGCAACAGGATCCGACAAAAGAATCATTTCATCTTCTAATAATAGTGACGATAATAGCATTCTTGGCATTAGAAAAACCCTAGTGTTTTACCAAGGGAAATCTCCCAATGGCACTAGAACTCACTGGGTCTTGCATGAATATCGCCTGGCTAGTACTACTCTACATGCTAATAACAATGCTTGCGATATAGGAGATTGGGTTCTGTGCCGCTTATCGGTGAAGAAAAGGAGTGTTGGGAGTGGTAGTATCATCATAAGCAAGAAAGCACGTTCTTCAGCATCTTCATCCTCATCTTCTTCCACTTCAAGTAATAACGTCATGGAAGTATCTTCTTCATATGCTTCTTAATATCAACGCAACAAACAATGCACTTCCTCCTGATGGTCTTGCTGACTATTGATTAAAACCACCATACATATTGTCACTCAGACCCCGTCGTTTCTGCAACTAAATGGATTCATACCCTTTAAAATTAACAAGGGAAATACCCAAGAGCTATTAAAATTTATTGTTTTTGGTCATTACTTTTAGCCATTAATTCAATTTCTTTAAGTTAATAATTCAATGACATATTTTTAGATTACACTTTTAAATATTGATGGCTAACGGATGATCAACAATAATAAATTTTAATAATCTTCTAGTATTTCTCGATTAACAACACTAAATATGCTAATTAAGACTTTTTATGTATCTTATATATTGTTGTTTCTTATTATAATATTGTAATGTACTTATCT

## >AhNAC162-Arahy.Z8VU36.1

CTCTCCAAAAAAGGAAGAAAAAAAAAATTCATTTTTCAAAAAGGCCGCGCCCGTGTACGTCGCGGCCGCACACAGCACCATGCACCACCGCTCATCACCCCACACATCACACAACCCCACCGCTCTGTCATCACAGCCAATCACAACACGCCACGTCACCTTGCCACCCCCCAAAACCCACACAAATTCTCACCGGCGGGAACACGAGTGAGAAGAAAGCTTGCGCAACACGAATCTCTCTCTTCCGCGTTTTTTCCCCTTAAACCCTTATAAAAACCCTAACCCCGATTAAAAAACCCCAAACTTTACCCACTCTCTCTTATTTATATATGATTCGATTGCTTCTTCTTCGTTTTGAGTTTCAATTCTCCGCACTGCGATGGAACAAGAAGAAGAACCACAACAAAATGAGCCACCTCACTCTCACTCTCAATCTCGGTGCGTGACGCTACCTCCCGGTTGCCGGTTCCATCCTTCGGAGGAGCTTCTATTGCGTTACTACCTGACCAACAAAAACGGCACGGGGAACTGGAATGGTAACGGTGGTTTGGGATTCGATGGTTCTGATTTGATTCGGGAGCTGGATTTTTACGATTACGATCCTTTTGAACTGCCGGATTTTGCGTGCTTTGCGTACGGCTACGGCGGAAGGAGGAGGCACTGGTACTGTTTCACCTCCGTTAGGGTTTCGAGGGGAGAGAGGTGGAAGAGGAAGAGGAAGGTTAAGAGTGGGTTCTGGTTGAGGAGGGGAAGGGTTTCGAATGTTAACGGTGTTGGGGAGAACGTGGTTTTGGGAACGAGGACGAGGTTCGTTTTCTATATGGGTGATTCGGCGAAGAACGGTGCCAGGACGGATTGGGTTTTGTATGAATACGCATTGGTTGATCATGTTATGGCCTCTTATGTTCTTTGCCGGGTATTTAGTAAGCCTCGTTATAAGAATAGTGCATCAGACATCGGCCTGAGTTGTTGTGCAGAAGAGAGTGTATCAGCAGTGCGCCATATTGGTATTCAGCATGATGAACATGTTAAATTGGATGCCGTTGAAGCTAAAGTATGTGATGATATCTCCATTGACCACAACAATGAAATATGTGCTGGTGGAAACAGCGATAATGATAATCAAGTTAAGAATGCACATGATATAGATGCTCTACGTTGTTTGGCGGGTCCTCAGGGCAGTCAGCAGCTATCAATTGCAAACTTCTACATCAGCAATAATCTGAAAAGTCTAGTCAGAAATGGGGCTATCAGTGTCGGGAGTCAATTAGCGTTTCTATCTTCTTTACAATTACCTTCCCCCTTTTTAGATTTGCTAGATTTGAGAACTCATTCATATGATCTTGGTTGCATTCCCCTTTTTTTCCCTGCTAATATCATTATTAATATTAGATTAAATGTGTTATTCTTTCTTCTGAATGGGAAAACATATTACTGTTCAGTAGAAACCGCTATAAGTAAATCTAGCAGTAATATGTCCTTCCATCCAGCTCTTGCTTTCCAGCAGACTTTTGCTTTAGCATGCTCTTTTGCCAGTGAATCTGAAAGGCTTCCTTTACTCCCCAGCAGTAGTACAATGTTCATTGAAGCAATTTCATCTCCACAACAATTACTTTCCATCACGGAGGAAGACTTCATAGAGAGTATTCCCCTGAAACTCATTAGTTCCGAATGGGTAAATATCCCATCAAAGTGGATAAACATTCGCAACGTCGGAGAAATATTTATGATATCAGGTAACCTTTGGCTGTCATATGAAGATCATATTGTCAGATCAGGTGAAGGAAAACATCACAGGGTGATCGCTTCACAAAATAAGGCAAGTTATGCAACTGGCTCTAGAGGAATAGAGTTAGGAGTTAGTTTATTGGCATTGGTGATTGCAATATGCAATTTACTTAAGTATGGCAAACCTGACGGTTTGGAGTTGTTAGAATTTTCCATCCACCCATTAGCAAAGCATATTCCCAGTAGTCTCCTCTCCTTTCATCTTCATAGCTTATATTGTTCAAGGAGCAAATATGATAAATTCCATATTTTATTGCTCACATCTTGA

## >AhNAC163-Arahy.ZDQ75D.1

ATATATATATATATATATATATATATATATAACAAGCTCATGAGAAGTGCACTACACAAAATTGAATTAAAGAGGTGGAGAAGATGGAAAACATGCCACCAGGTTATCGTTTCTACCCTACGGAAGAGGAGCTGATTTCATTCTATCTACGGAACAAGCTTGAAGGAGTGAGGGAGGACATGAATCGGGTTATTCCAGTTCTTGATATATATGAATATAGTCCAAGCCAACTCCCACAAATATCGGGAGAGGCAAGTGTTAGAGACAGCGAGCAGTGGTTCTTTTTCATTCCGCGGCAAGAAAGCGAAGCGCGTGGAGGGAGGCCGAAGAGGCTCACAACAACTGGGTACTGGAAAGCCACTGGCTCCCCTAACCATGTTTTCTCTTCCGATAATCGCGTCATCGGAATGAAGAGAACCATGGTTTTCTACTGCGGTAGAGCTCCTAACGGAACTAAAACCGATTGGAAGATGAATGAGTATAAAGCCATTGACACTCATCACGCTTCTTCTTCTTCTTCTTCTTCTTCTTCCTCCAACAACAGGGCGGTTCCTATGTTAAGGCAAGAATTCAGTTTATGCCGAGTGTACAAGAAAGCAAAATGTTTGAGGGCATTTGATAGAAGACCACCTCCAAGGAGGGACACATATCCTCCTAGCCAGAACAACGGTTCATCATCTTTTGATCATCATCATCAACATAATCAAACGGTGGAGAAATCTTCAGGTGCAGGTAGCTCACCGGAGAGTTCGTGCTCCGAAGACCACGGACAGTGTTCTCATCGGACGGAGGACGTGGAAAACGCTAACGAGCCATTTCTCGATTGGGAGCAAATCGATTGGTTCTTAGGATCTTCTTCACCGCCGGAACCATGAATGCACAGGTATAACTAATTGGAACTCCATCAACGAAAATTCGCAGAAGATCAGAAGGCGGCTTCCGTATGATTATTAAGTTATATATGAAGCGTAATTTATTAATTACTTAACCACTTACTAGTTTCTTTAATGGAAAAGCCTCTTTGCTACAATTAGAGTGTTCAATAATAACAATAATTTTAGAAAGATATGCATGTAAATTGCAATGCCCTATATTATCAGAATAACTACTTCGTCTTGTGATCAACCATATCAATCACCTTTTCTTCATGCATATCTTCCTAATGGATGGAAGATATGTAGTAACTATATTTTATTATTCTAGTATTTTATGTTGCCATGCTAGATAATCTTTTCTCAATCTTTTAAGGAGAAGAGAGCGAATAAATATTCAATTTTGTCATTTATTTATAAAAACAATGTTAGAGAATTATTA

## >AhNAC164-Arahy.ZM1I0C.1

ATGCGATGTAAAAATGTTAAAAAACGCAAGATCTACGTACAGCGATTCTACTTGGAGTTCTTAGGTGCAAGATATATCAAGCTAAGGAGGTTTGAAATGAACGCATTTTCACATGTTCCTCCTGGGTTTCGGTTCCATCCTACGGATGAAGAACTTGTTGATTACTACCTGAGGAAAAAGATAGCTTCGAAAAGGATTGATCTGGATGTGATAAAAGATGTGGATCTCTATAAAATTGAGCCATGGGATCTTCAAGAAATATGCAAAATAGGAAGCGATGATGAAAATGAATGGTACTTCTTTAGCCATAAAGATAAGAAGTACCCAACAGGGACTCGCACCAATAGAGCTACAAAGGCAGGATTCTGGAAAGCCACGGGAAGAGACAAAGCCATATACTCAAGCTCAAGCCATTGCCTCGTTGGTATGAGAAAGACTCTTGTGTTCTACAAAGGACGAGCTCCCAATGGCCTCAAATCAAACTGGATCATGCACGAGTATCGTCTTGACTCCAATCAGGAAGATGGCTGGGTTGTGTGCAGAGTCTTCAAGAAGCGGATGCCCACGCTACGCAACGTGGTAGACTATGATGATCAACTTCCCTTCATGCAAGGATCTCCATCCACTCACTATCCCTGCAAGCACGAGCTTCATCAATTCCAATACAACACCAATGATGCTTTTCTCCAACTTCCACACCTTGAGAGCCCTAATCAAGTTTTGAGTTGCGGCAGCCCCGTTATTGCACCCTATGCCTACGCCGAAAACAACAACAACAATAATGGAACTAGTAGTACTAGTGCTTTGCAGTCCTATTCATCTGAACGCATTCAGCAACAACTTCACTTGCTTTACGGTAGCAATATTGAGCAAGCAGTAGTGGTGGACCAAGTCACGGATTGGAGAGTGCTTGACAAATTTGTTGCTTCTCAACTCATGAGTCAAGATCAAGATCAGGCTTCCAAGGAAACCTGCAGCATGGCTGATGAACAACATGTTGCTACTACTGTGCTTCCAAATGGATCCACGAAGCAGGAAATGGCGCCTCAGGACGACTATGTTTCAACGTCTGCCTCCAGTAACTGCGATATTCACCTGTGGAATTGA
