# Supplementary material for: Comprehensive genomic characterization of NAC transcription factor family and their response to salt and drought stress in peanut
Source: BMC Plant Biol. 2020 Oct 2;20:454. doi: 10.1186/s12870-020-02678-9 (PMC7532626; doi:10.1186/s12870-020-02678-9)
Supplement: Supplementary file 5 — Additional file 5. NAC proteins of two wild peanuts. [file 12870_2020_2678_MOESM5_ESM.docx]

**>AdNAC1**

MDVAKLYMNNDYSEEHEHEYEHDEDDDEMMKEEKEVVLPGFRFHPTDEELVGFYLRRKVEKKPLKIELIKHVDIYKYDPWDLPKVGSSMGEKEWYFFCIRGRKYRNSIRPNRVTGSGFWKATGIDKPIYSANYNNNYNNNSNNNSKEHGDHHECIGLKKSLVYYRGSAGKGTKTDWMMHEFRLPPNNNNGAKLLSNNQEANNATKDLHEAEVWTLCRIFKRIPTYKKYTPNLKDSSTSPLMNKPINNINHQTDSSVTSISCSLESDNNNSKQFLTFTNTMGIQQCERKPLVIGHVDERNNNFFLDHSSIHHQQAPTTITTTALSSSSYSSWNQHHVVEDYLFANENWDDLRSVVEFATDPNNSKVYL

**>AdNAC2**

AWVSRQHPYYKYLSFSICTINTTHLHHYSNLTHSLLLFSLLIFLMGDNNVNLPPGFRFYPTDEELVVHFLHRKAALLPCHPDVIPDLDLYPYDPWELDGRALAEGNQWYYYSRRTQSRVTENGYWKATGMEEPVMTSSTNKRVGIKKYFVFHLGESPSAIKTNWIMQEYCLSDYSASSSRSSKRKSDYSKWVICRVYERNGDDDDGTELSCLDEVFLSLDDLDEISLPN

**>AdNAC3**

MGSSNNGGVPPGFRFHPTDEELLHYYLKKKVSFQKFDMDVIREVDLNKMEPWDLQERCRIGSTPQNEWYFFSHKDRKYPTGSRTNRATNAGFWKATGRDKCIRNTYKKIGMRKTLVFYKGRAPHGQKTDWIMHEYRLEDSNDPQANANEDGWVVCRVFKKKNLFKIGNEGGGGSTHTSSDQQLNNSTATNARSFMQRENHYLLHHHQQQQNPRNGNPSSSSSGFDELDKPELGLHHYPHMQNPHYSLFHHSQPLLHPQAHKPIVYDYSYTPALPSDPPVTAKQLMTNPRDCDSGGSESLRYQQVSEPGMEVGSCEQAQEMGAAAAARGGGEGMNEWGVLDRLVTGNLGNEDSANKGIRFEDANPHQINQLSLRGEMDFWGYGKQ

**>AdNAC4**

MAIAAPNSSPTMSLSHSHSHEDGGTTTAASTTNDNLNGNGKQEDDDHEHDMVMPGFRFHPTEEELVEFYLRRKVEGKRFNVELITFLDLYRYDPWELPALAAIGEKEWYFYVPRDRKYRNGDRPNRVTTSGYWKATGADRMIRTENFRSIGLKKTLVFYSGKAPKGIRTSWIMNEYRLPQHETERYQKAEISLCRVYKRAGVEDHPSLPRCLPTRAPSSRTVDHQKNKQQHHNDQLNMGFAGNTADGASDNRDHDVTTALALSKHNTNAYRAPSMGLPPLLLPLDDEAAFVLMQQQQHHAGPSSGTTTMMDDLNRLVSYQHQYYNSSSSSSNNNNPNHHHHLLMQQQQQQQQQTPPAIMSLNNTPSPLATAFSDRLWEWNPLPEANQRQYSNMSFK

**>AdNAC5**

MVFYKGKAPTGRKTKWKMHEYRAIVQAPNQSPTAIPQLRHEFSLCRVYVISGSFRAFDRRPREVVVPRVLHHGSSTTSAQQHQGESSARVQANNNNNGSSSSETSLSSGGPDLPPDTGGGGSCSNWNSSEVQVQAQVQEPLWEWEQLDWL

**>AdNAC6**

MTETTILPVGYRFRPTEEELLVHYLNNKHLRNDAEIKNTVSQIDLYNFDPWDLPEQSKVKWDDQEWFFFNELKHIKNKRCNGKTNAGYWKITGKERIVKRTGIDNVIGTKRTLVFYKRPHSVKPIGFFMNIMHFI

**>AdNAC7**

MMAGSGQLTVPPGFRFHPTDEELLYYYLRKKVSYEAIDLDVIREVDLNKLEPWDLKDKCRIGSGPQNEWYFFSHKDKKYPTGTRTNRATTAGFWKATGRDKAIYHTSNSKRIGMRKTLVFYTGRAPHGQKTDWIMHEYRLDEDEAEVQEDGWVVCRVFKKKNQSRGFQQEIEEEEHHHLAAAHQHMRGVASQQVLDPKHHHHLQHHQGLYDNENNNNYTNNFDGSMHLPQLFSPESSVATAAAHTSMNAMDILECSQNLLRLTTTSGCGLNLMQQQHGERFNGDWSFLDKLLASHHGSTMDHHQHHHHHSKCNNNLHHQHSAIAIGTTSSQKFPFHHLGCDNHDIMKFSK

**>AdNAC8**

MAWCNETHEKEIIASNNSTITLRPKSDQEIRNISCPSCSHNIQIIQEQGGIHELPGLPAGAKFDPNDIEILEHLEAKVMSHVPNLHPLIDEFIPTLQDENGICYTHPEKLPGVKKDGQIRHFFHRPSKAYTTGTRKRRKVHTDEDGSETRWHKTGKTRAVVAGGLVKGFKKILVLYTNYGRQKKPEKTNWVMHQYHLGSNEEERDGELVVSKVFYQTQPRQCGNSIVIKEDDDDLPYGKILMMNNRKKHKNNDDAAPVVDYYINYDHVEHHHNHNHNSQRCSSPTQLIPNLVLQGDSSSLFRFASSSLDGNANKTRLFERKL

**>AdNAC9**

MDAEDHNHALDLPPGFRFHPTDEEIISYYLTHKVLNTSFTATAIGEVDLNKCEPWDLPQKAKMGEKDWYFFWQRDKKYPTGIRTNRATESGYWKATGKDKEIYKGRNLVGMKKTLVFYRGRAPHGHKTNWVMHEFRLEGLFATYNLPKPAKEEWVVSRVFHKNTTEKLNPTIPSGLFRIMKNVNSIEDDDLVDFSSLPPLMDPSNNYDDEHTTTTNNMFASSSDYNITIQQNKKDMMGIRNNNIRALLMYDGPSSSSEVVAPPLSDLELCLWDL

**>AdNAC10**

MNTFSHVPPGFRFHPTDEELVDYYLRKKVASKKIDLDVIKDVDLYKIEPWDLQELCKIGSDEENDWYFFSHKDKKYPTGTRTNRATKAGFWKATGRDKAIYSKQHCLIGMRKTLVFYKGRAPNGHKVFKKKMATVRKIGDYDSPCSWYDEQVPFMQDLESSSPIKPPIINNNHYASSYNHHQLQLPCKPEFHQPMQYNNMNMPRHDDAADNNNNFLQLPQLESPNAGISPFLQQQDPHQLLQQQNSNNSNYHLDQVTDWRVLDKFVASQLMSHGDDDGHNHNNNVSKE

**>AdNAC11**

QNSNDEAQVRMHSLFPLPTKTPGYNFPLTLPTVAFFVSLSIENGKPMLRLNTLHSSASAQASSSSSLQWLGLEGEGKEVKGLKSKRSWLIDIGGFAKKVKSTNLSPADQIKDCGAYRDCPNCHYRIDNRDVSTEWPGFPLGVKFDPSDVELLEHLAGKCGIGNAQLHMFINEFIPTIEEEEGICYTHPENLPGVKKDGSSAHFFHRTTNAYTTGQRKRRKIHHECLTEEHVRWHKTGKTKAILEDGVHKGFKKIMVLYIRPKKGSKPDKTNWVMHQYHLGTDEEEKNGEYVVSKIFQKQTEKNEENPAVEDSDQTEKNENRLADDSNCIASRTSPRTPKPNPPNPPRAGNFVDNDDNIDETELPFTQDVKCVPLCDVLDQNNAGDPAWLAGESQAVENFDFDGLDDILFCNEIFDSSSLLDVSGTETMINGSASNDMLGNDSLSYGTSVLDTLDLGTPPDFDLSNLNFYSQDSIFDWVDRL

**>AdNAC12**

MAELSAAATFTPSDEELIHFLSDKVKGQSMDEDAAINIHECEYLYGRNKNPWDIWRDFAGDVDAGRTALFFFSPNKKHHSTASRPIGAGVWEAEAETIDGEGIVGKGKNRRIGTKKCFIFDKSGTSYDGAWILHEYTLHGSSLHTNTSVDNSYVICKLIKNVEGEAHPVEVQFGDKRKRHAQSATTSGVQIDVNAPHSYRNTKEQEVQFIPNELGRRMLLERFEGLPITNENLIRNLMQLQGGTRRGGKGL

**>AdNAC13**

MENICSEVEMDLPPGFRFHPTDEELISHYLYNKVIDTNFSARAIAEVDLNRSEPWDLPWKAKMGEKEWYFFCVRDRKYPTGLRTNRATEAGYWKATGKDKEIYRGKSLVGMKKTLVFYKGRAPKGEKSDWVMHEFRLHGKFNPHNLPKSAKNEWVICRVFQKSSAAKKIHLTGIMRLDSSVFLPPLADSSSSPSNTATTAPYVPCFSNPIIHNQVGIFDPFSNTPFGADSFYSSQGMPMQHAQPPSSTYSASGYTTHDHSILRALLQNNSSNLRSGFKPAEREMSHHQTSLVDANNNNNGITSVVAPQDLSSLWNYQVQIK

**>AdNAC14**

MGSPESNLPPGFRFHPTDEELILHYLRKKVASIPLPVSIIAEVDIYKLDPWELPAKAAFGEKEWYFFSPRDRKYPNGARPNRAAASGYWKATGTDKTIVVSPAATVTRRVGQESSVGVKKALVFYKGRPPKGVKTNWIMHEYRLVDNNRPIKLKDTSMRLDDWVLCRIYKKSKFSVSSPEESPSSEVQAAEENGLFKNTILRSPIPTPSPSPPPPLPQPLLSQKSVSFSNLLDAMDYSMLSTILSENNNNSTLDQQQYSQINTNQLNHSSNMENTSNSNMMVMRSKRQIEEETTTVLHPSKKFHHQLMGSSSCSFPNNINNTNTAQYENPQWNYLVKQSFLNQHLLLAPHLRFQG

**>AdNAC15**

MMSKKMRFVKKNKNGVRLLPPGFRFQPTEEELLFQYLKCKVFSFQLPASIIPEINVCNYDPWDLPERYLFSSKEVKYRNGNRMNRITKSGYWKATGSDKRIISTSSNNNNNNNIVGIRKTLVFYHGKSPNGSRTHWIMREYRLVTTPSNSSQKYVEDLGNWVLCRIFKKKRSIESQHHMVKNKINNNVVESFQ

**>AdNAC16**

MGAVVDCYPPHAGEVAVLSLNSLPLGFRFRPSDEELVDYYLRQKINGNGEEVWVIREIDVCKWEPWDLPDLSVIRNKDPEWFFFCPQDRKYPNGHRLNRATNHGYWKATGKDRKIKSGSTLIGMKKTLVFYTGRAPKGKRTNWVMHEYRPTLKELDGTNPGQNAYVLCRLFKKQDESLEVSNCDEVEQTDSAPMAANYSPEEIQSDQALAEVSPSQVTDEKHQGVIPEISEEAVSNVITSADCHSDGYDACERRNQAFELPAEDIPPLNWDIFNDPEDKIFDDKLFSPVHSHIPPEFYYQANNETNIADILNSVNWDEISYEDPYSQAQNNFFNNVKQSVSGSEPDAGLTNMTCIHPTNVVYPEEAIHRKVALATTPQFCSTFTSDFSADEQKSSVALIQNNSQMASFPDARTGQVYNVFNDYEQPRNLNTYVSGDTGIKIRTRQVRNEQPAMIFTDQGNAARRIRLLKQCANVSNKMADDGSPKQEHDSKPIIAGNKNKTFKSHTADKHDTANDLNERQEKTESTDKRNMISKLAKGGSSMLGLKGLLRRRLSYISKASSNFKMWSCVVVASAFVLVSFLFFANIWGYINL

**>AdNAC17**

MQKEKESITTNKEGTNKKEIEIMEGCNGKEETLPPGFRFHPTDEELITCYLINKISDSNFSGRAITDVDLNKCEPWELPGKAKMGEKEWYFFSLRDRKYPTGVRTNRATNTGYWKTTGKDKEILNSVTSELIGMKKTLVFYKGRAPRGEKSNWVMHEYRIHSKSTFRTTKQDEWVVCRVFQKSAGAKKYPSSNHASRAMNPFNLEIGHHNIVPPPPMMQLGDPAAAHFLYGRNYMNTAELAEVARVLRVGTGSTSTNLPGMQPQINYPVAASPGIGFTISGLNLNLGGGGGETVVATTQPVLRPMQPTPPSQTLGMVPHHQVHHDVSSNMISTNSLGAENVGYVNEISNTNGGHGNRFMGMDHCMDLDNYWPSY

**>AdNAC18**

MIMVDNSTDSSSGAGDQHHHPQLPPGFRFHPTDEELVVHYLKKKASSSPLPVAIIADVDLYKFDPWELPSKAAFGDQEWYFFSPRDRKYPNGARPNRAATSGYWKATGTDKPILSSDGNKQKVGVKKALVFYGGKPPKGVKTNWIMHEYRLTDNNNNASSSISSKPPSIPLDPLKKTSLRLDDWVLCRIYKKSNSSSSSLPIPRPAFLMDEEKDLISMENSMVPTMSMSKPRSTSTTGCYGPMALENDDNFFDGILAASTDHHTMQNGSPGSSSSSKRFHGDLNNGDNTSFVSLLNQLPHNTPFHPNSILGSVGDAVS

**>AdNAC19**

MDYGSVVITIPNSTIHTYTTNSMMMMTADYESVKQLPPGFLFSPTDEELVLHFLYAKASLLPCHPNIIPDLDVSLAHPSQLNGKALSSGNQYYFFSKVKEKRITENGYWKEIGESEAILSSTFEKKVGTKKNLVFHIGEAPHGIETSWVMQEYHICPSSNIISTTRARRKHDHQIWSKWVLCKVYEKKGSVRGVNYCSDDDDSGTELSWLDEIYLSLDDDLEEISVSILD

**>AdNAC20**

MEKLNFVKKNGVSRMPPGFRFQPTDEELVFQYLKCKVFSFPLPASMIPDINLSNYDPWDLPGNCDEHQEMYFFSSKEPKYRNGSRMNRTTTTGYWKATGSDKRIISSSNNSDDNSILGIRKTLVFYQGKSPNGTRTHWVLHEYRLASTTLHANNNACDIGDWVLCRLSVKKRSVGSGSIIISKKARSSASSSSSSSTSSNNVMEVSSSYAS

**>AdNAC21**

MSESNEHENNHGNIIVEGRKDSLIRTCPTCGHHIKCQDQGGGIHDLPGLPAGVKFDPTDQEILEHLEAKVRSDIHKLHPLIDEFIPTLEGENGICYTHPENLPGVSKDGLIRHFFHRPSKAYTTGTRKRRKVNSDEEGNETRWHKTGKTRPVYIRGKLKGYKKILVLYTNYGGKQRKPEKTNWVMHQYHLGNDEEEKEGELVVSKVFYQTHPRQCSSLLINNNKDSSTAALVKGNNNNGFVEYYHSNFISFDQGEHQHRSSGAQVVISHFPVHEGAPNYHSLNRKE

**>AdNAC22**

MESTDSSTGSQQPNLPPGFRFHPTDEELVVHYLKKKAASAPLPVAIIAEVDLYKFDPWELPAKAAFGEQEWYFFSPRDRKYPNGARPNRAATSGYWKATGTDKPVLTSGGTQKVGVKKALVFYGGKPPRGIKTNWIMHEYRLADNKPNNRPPGCDLGNKKNSLRLDDWVLCRIYKKNNTHRSPMEHEREDSMDDMIGGIPPSINVGQMNARFHLSKMSTSFSNALLENDHHHHQNLLEGMMLGGGTNNSNPNMLGLGSASNTINNNSNKAELSFVPTMTTSSNTKRTLSSLYWNEDDVAASNKRFNLESGDHNHGENNGTSASSIATL

**>AdNAC23**

MNTFCHVPPGFRFHPTDEELVDYYLRKKVNSCRIDLDVIKDVDLYKIEPWDLQELCRLGTEEQNEWYFFSHKDKKYPTGTRTNRATAAGFWKATGRDKAIYSKHDLIGMRKTLVFYKGRAPNGLKSDWIMHEYRLETDQTSAATPHEEGWVVCRVFKKRVTSIMRKMSDHDSPSCTWYDDSSFMHQQPDHFDNSCSSSSKHQLIPNNNCDVFYQQQNNNLPLHHLPLLHQTAALSNHNNNPIMAPPFAAINNNETTAFQEQGKSLIHHQALLYGNLNEEQASSSAAAADWRLVDKFVSSQLREDHHVSKQELMMPENNNNNNNDNGASTSNSSCPIMDVWK

**>AdNAC24**

MPGFRFHPTDEEIVGFYLKRKIQQKSLPIELIKQVDIYKYEPWDLPRVASNGEKEWYFYCPRDRKYRNSARPNRVTRCGFWKATGTDRPIYSSEAQSIIGLKKSLVFYRGRAAKGFKTDWMMHEFRLPSLSSDSAKKCSDKTTPASDSWAICRIFKKTNTMSMAQKASLPHHPYNWNHHNQLFDDILTHQQHQHQHPIIPNSNNNFIFYNSNSTLEPTKEIDATTTSSSIVISSNIGLHEDPNHHHYNNNSSGFSYDDDSGVITTIAGFPFNLPPNDDDAAAWNNNNNKPNTTLPWDYSSDMSTTYSTNKSYT

**>AdNAC25**

MSRILGPGFRFHPTDDELVQYYLRRKVIGKLNHHDHIGVINIYDYEPWQLPELSKLNTRDLEWYFFTVLDKKYEKGEKTKRATVNGYWKTTGKDRGIKYGDRQVGMKKTLVYHEGRAPTGKRSNWVMHEYRMVDEQLAEVGYQLDAFVLCRIFEKSGMGPKNGEKYGAPFREEDWVEDGDLLEPIADEPVVELSVDQSDAFLETDDLEKKLGTHVVDGSADLPPNPPNYFYGECSHYPQHQEEFVEVPKPLEGTEGRNFDVTGPYAEDTCLENHEMNHNGNSSEFIYGDVNSDEFMDSIVDPLIGAELFLETDDLLNPIEGNSSGADPYTVEGNHPRADPYATEGNPPGPDPYTAEGNYPGTDPYAVDMLDEYLALPDDDILRYISFDDSPPSMEGEHPILEQIPPLIQQNVEEEAKDVSEEKQQKVEGEAANIFKTNKHDLEANSSRGGAASDDANPIAKRFKKWLEDIPAAPAFAAELPSKKDALRLHSAPQSSNTTHVTAGMVSITNITARGNHMNPMVEKIGGGFNHPIISAVVLIPVSGLLCGKTLFVLTYGWAFLVTFSFLFATVTCKIGTFMYSGK

**>AdNAC26**

MGDSNNVNLPPGFRFYPTDEELVVHFLQRKAALLPCHPDVIPDLDLYPFDPWELDGIILSLCSNTCMHIARELKLMNTIMCVSVCVCAGRALAEGNQWYYYSRRTQNRVTANGYWNPMGIEEAVVSNSSNRRVGIKKFYVFYVGEAPHGNRTNWIMQEYRLSDSAASSSRSSTKRKSQPKTDHSKWVVCRVHESDENDDDGDGDGTELSCLDEVFLSLDDLDEVSLPN

**>AdNAC27**

MEGEKLDEIMLPGFRFHPTDEELVGFYLKRKIQQMPLSIELIKQLDIYKYDPWDLPKVAGTGEKEWYFYCPRDRKYRNSARPNRVTGAGFWKATGTDRPIYSSEGSKCIGLKKSLVFYKGRAAKGVKTDWMMHEFRLPSLVDSSSSDKTTIPANVGIYHSLKPFLTGSISHFLADFNFNMQDSWAICRIFKKTNATAQRALSHSWVSTLPETTPTTTTNDTDHIFNMPTMMAKKTSFMTQFCTNYTSDTQIQDVASSYKPPFININPLLYKQFDHHHHQLPPIISNGDLISNDCLIPSSTTPLETSSNSAKPTMDFSSLLLNMSSSVLGDFAGKTSSSSSSQEGTAAAATATTITSSFGGGMQEHYPTIPLLRQMHQGNNNNNNNIGINNNNVSAGGEEQELEKVGSIVGFPFMNIGDAWKSNMLWDTSCPL

**>AdNAC28**

MENMNSFCHVPPGFRFHPTDEELVDYYLRKKVSSRKIELDVIKDVDLYKIEPWDLQEICRIGREEENEWYFFSHKDKKYPTGTRTNRATAAGFWKATGRDKAIYSKHDLIGMRKTLVFYKGRAPNGQKSDWIMHEYRLETDENAAPQASLILYFSVIEIFAYIHNRHECMHAWMQEEGWVVCRVFKKRVTTMRKVMMREHDESPNSSCWYDEQELMMMESPTKQQSSILLHQSTNNNHSNLMQLPPYPLIKKELHHPSSSYPFLQLPLLESHQQSAAAPSSISEQLIMPPPIGGGGGEQVPSFQSFFNNEQQEVGVLDWRVLDKFVASQLSQDDNHASSNSIVQDLTQEIVMVPHNDAASTSNSLTCPIDLWK

**>AdNAC29**

MNNNKISNMSSVSSSDLIDAKLEEHQLCGGSKQCPGCGHKFESKPDWLGLPAGVKFDPTDQELIEHLEAKVESKNMKSHPLIDEFIPTIEGEDGICYTHPEKLPGVTRDGLSKHFFHRPSKAYTTGTRKRRKIQNECDLQGGETRWHKTGKTRPVMVNGKQKGCKKILVLYTNFGKNRKPEKTNWVMHQYHLGQHEEEKEGELVVSKIFYQTQPRQCNWSSDRSATTTIATAEGSGEPLQNSRRDSGSGSCSSKEINIGHKDEMSAVVGVTNTPITGFAHPLDIHHHLKSDHFSFIPFRKSFDEVGIGEASTAREVQASGSCDEVVHEHVNHHHQQQQQHHHHHQIXXXXXHPISTLISPPPLHHTSIILDDNSYQVSRIMLQNEHFQQQQQQQHQQHHHKIGARSASGLEELIMGCTSSDIKEESSITNPQEAEWLKYSSYWPDPDNPDHHG

**>AdNAC30**

MRFKPTDEELVSYYLNHKLLNDNFPINIIPDIDLCKVEPWQIPALSKIKSDDPEWFFFSGRDYKYGKSKRSNRATKGGYWKATGQDRYIKERGTTNVIGSKKTLVFYSGRVPNGVKTNWVIHEYHATTFDDSQRNFVLCRLMKKAERKSEDGTDAQACDEGEPSTHMEEADESVPSMFESPDVDMGSIFHTLPQDGSSSQHSPVSIEQQESFPFSPSENYYLVNEDRSMHIQFETNEEKQDAEKFADSILDGGNIAMFEERQQHHTFMNNHLRSVPSMRVCYESSDTDAEVVSRRAGSREYHVSKMVQSSHGAACTDKTRSISSEDFWGLDSSSCDSNADKPFEINSIEISSPPSALSRSKNQYIPRLSQTHRKVSSNAIPHLEDKKKLTTVEQSRRDQEKAQKTSPGKKLETRSSDVNRIGSFIHLEPCSSSESLTPRAVYLVNVVIGILLLLAISWDVLSC

**>AdNAC31**

MQEEIMTQCNSNNDYPENNHSTIVERNKDSLISRTCPSCGHHIKCQQDHQGAGIHDLPGLPAGVKFDPTDQEILEHLEAKVRSDIHKLHPLIDEFIPTLEGENGICCTHPEKLPGVGKDGLIRHFFHRPSKAYTTGTRKRRKVHTDADGSETRWHKTGKTRPVYISGKLKGYKKILVLYTNYKKQRKPEKTNWVMHQYHLGNNEEEKEGELVVSKVFYQTQPRQCAGSLLIKDSSSFPAKLKDQGGVHHHEVTNNHKNNGFVEYYNASFISFAQGEQQHRSNNPTLISHFPAHDGAPFIP

**>AdNAC32**

SKEQNNITLSDTKPFYLHLRIMVDTDSSEAHMSIAASSIFPGFRFCPTDEELISYYLRKKLDGDEDSVQVISELELCTFEPWDLPGKSFIQSDTEWFFFSPRGRKYPNGSQSKRATECGYWKATGKERIVKSGQDVIGTKRTLVFHLGRAPKGERTEWIMHEYCVNDKSQDSLVICRLKKNTEFRANDHSNKTSHDSDCGVSEEVTVQGGTYVPIQDKETGCSSKRTSSSNSSPSTTTGQIESSHRVVNEANQANEVDEDDCYAEILNDDIIKLDESTLSRPSPPQGTANRRIRLRVPKSTVPKSRVPKSTVPTGNGCHCSKQSSNKINTFLPYALVVFTFFVFTLLALGFFLIIRRSQTTAQYSRDLSRVN

**>AdNAC33**

MEGRGSSFVKNGELRLPPGFRFHPTDEELVAQYLKRKVFSCPLPASFIPEVDICKSDPWDLPGDLEQERYFFSTREAKYPNGNRSNRATNSGYWKATGLDKHIATSKGHQLIGMKKTLVFYRGKPPYGSRTDWIMHEYRLVSHSHLLPMQNWVLCRIFFKRRAPANAKNVLLDHNSHSASASEAFTISHEGSNSKVVFYDFLAQNRADLNRVPPPASSTSGTSGITTESDEHEDSSSCNSFPFFR

**>AdNAC34**

MDSCQPQLPPGFRFHPTDEELIVHYLKRKASSAPLPVAIIADVDLYKFDPWELPSKATFGEQEWYFFSPRDRKYPNGARPNRAATSGYWKATGTDKPIIASDGQHRLGVKKALVFYGGKPPKGVKTNWIMHEYRLTTTHNNNSISSKSFPSLPSHLPSSNQKNNSLRVSILDDWVLCRIYEKSNRGNFARTALMEHHDHDDDDDNNKDQLSAETTSMIENMSTMSQNSKPTQHYGPLLVQNDDNFFNGILVADHQNHQHHNLPMKRALVNMNNSQFWNETSKRFHCDLNNNTNTTVANNDEDNSSFVSLLSHNQIPHPTNNASLLGPTVADGVFRQHFQLQTINWNL

**>AdNAC35**

MAAMKSIPGYRFHPTDVELVQYFLKRKVMGKRFPCDVIAELDIYKYPPWDLPDHSLLKTGDLEWYFFCPRGKKYSSGGRMNRATECGYWKTTGKDRSVENKKLVVGMIKTLVFHMGKAPKGDRTDWVLHEYRLQDKDLADKGVQQDSYVICKVFQKDGPGPRNGAQYGRPFNEEDWDKEDEIDCVESAPVAALPAAVPIQPASCHSSVVNNVNLSVSECYGLTSVSCLTGPMPSCSAHPSAPSNQVDGDITPVPGSSIEDNIMAPTQNTTTEKVDNPPDINNAEGTPCFDPNEIFGGLGDLDGLFEMGGIGHGFSCGQNGGYTVNEMLSAGDGLRFPDPLDYLELGDLDTPLLWETNEQGNWSQDNK

**>AdNAC36**

MGGASLPPGFRFHPTDEELIGYYLKRKVEELEIELEVIPVIDLYKFDPWELPEKSFLPKRDLEWFFFCPRDRKYPNGSRTNRATKAGYWKATGKDKKVVCQSSPSTSIMKATGYRKTLVFYRGRAPLGDRTDWVMHEYRLCDDLGQDSPSFQGAYALCRVIKKNDKASDYKGKRGVSSSKNENENENESSMRLSSSKEHLSISADVSSQASQLCSESRYSSPIASPCAYNVAATAGFEPPSVDTNPSTFLVSPDMILDSSKDFAQTQDVISGFFPHHELPSTMTPWQSLEHTEISSSSSYSNFNGEIEFSDELGLIGRMSRYSGQVDMLDFYGNEEVLYEYEGYDQINSIRDPRQF

**>AdNAC37**

MEEGGGDQHASNSSYTFPPGFRFHPSDEELIVHYLQNRISSRPLPASIIAEIDLYKYNPWDLPKKALFGEEEWYFFSPRDRKYPNGLRPNRAAGSGYWKATGTDKPILTSYGSKRIGVKKALVFYLGRPPKGTKTDWIMNEYRLVDTITSPSRLKGSMRLDDWVLCRVRHKGYSSKNSCENQDNPCEPNMLSNLPRCDEGYPATNMNFHADMITDYQYKDYQILASILVGGHVPTTESMSSLNLKDGKGNDPITSVHEDGFHREDSSTTVSPLDCYFNSLKRKSNEDSQYENLISFNRKLNMETTMDDESSIINGGLNFYNQNQSQDDIIFNKRAAEPSINFQELKQSAFIGRYPQCSSD

**>AdNAC38**

GFFGLSMGAEAGATECFSKAMASMPGFRFHPTDEELVMYYLKRKICGKKLKLDVILETDVYKWDPEELPEISVLRTGDRQWFFFTHRDRKYPNGARSNRATRQGYWKATGKDRNVTCNSRSVGVKKTLVFYRGRAPNGERTDWVMHEYTMDEEELNRCQDIKDYFALYKLYKKSGPGPKNGEQYGAPFKEEEWADDECVDFNINSADREEVNTVPVNDQLPPLADDEVTDMINQILDNELALDQQFGDGLEFPQVVAEETQSTVVDQFSEAVTDPEYNDIYHSTSQHYDVQNVNFNQSVASHLHAPEGSEVISTANIQVEDYNFQEDDFLEINDLNGSELTIPNMETPVENLQFEDGLCELDLFQDAEMFLRDLGPINEETIPHSYMNNAAGSNIENQNYHLLPNPEDATQNVHEFWMHDERNTSSLFEGFDDSLSQQNPGAVSTEGYDNQSSIAEDVATSRFSSALWSFVESIPTTPASAAENALVNRALNRMSSFSRVKINIKPTNTAAGKDTATTKRVGRKGFSFLFFPIIIALCAFLWVSLGTFRLLGRCIAP

**>AdNAC39**

MEKVASLVLKEEEQMDLPPGFRFHPTDEELITHYLYKKVIDTNFAARAIGEVDLNRCEPWDLPWKAKMGEKEWYFFCVRDRKYPTGLRTNRATESGYWKATGKDKEIFRGKSLVGMKKTLVFYKGRAPKGEKTNWVMHEFRLEGKFSIHNLPKTAKNEWVICRVFRKSSAGKKVHISGIMRLDTFRTELDSSGLPPLTETSPSFDTIHDESPYVPCFSNPIDVPRNQAAGGSGGGGGVFGGSFPNNSSSSVPAYAVSSNILPRMPICGGSLYSTQHQDQSILRALYESNEREMISVSQETGLTTEMNVETNSVVSNFDLGRAHFESLWNY

**>AdNAC40**

MNKMDLIDAKLQEEHQLCASSWKQCPACGHKFEGSSGKKGEWEWVGLPAGVKFDPTDQELIEHLEAKVEAKRSHPLIDEFIPTIEGEDGICYTHPEKLPGVTRDGLSRHFFHRPSRAYTTGTRKRRKILQNDEAEAERGETRWHKTGKTRAVMLKGKQKGCKKILVLYTNFGKNRKPQKTNWVMHQYHLGLHEEEKDGELVVSKIFYQTQPRQCSWSSSSSSSSITAAAPPVKTNNDTCPVLGFPPMEHFSSFIPLRKTLHNENLFYLIGETCTPASHIPSSNPLGVFHHNTSIILDDLISARFMTPPPPPHFHQQHDNKVVGGTSASGLEELIMGCTSTSTTHNITKEASMTNTNPQEAEWLKYSSYWADPQPQPQPHLHG

**>AdNAC41**

MISELLRSNGVMKYVVYAEVAKLNANEWYFFSFRDRKYATGFRTNRATTSGYWKATGKDRTVQDPLTQEVVGMRKTLVFYRNRAPNGIKTGWIMHEFRLETPHMPPKEDWVLCRVFHKSKEENSGKLIMYDSISTHHESSNSMALVSTHHLNPINNHNAMNNFLHHFSSSRDDSQTNNANNNNNNSPKGYDGYGFIWDHMDLEDHSVPSSDFQVDNNNNNNNIALL

**>AdNAC42**

MNTKIELPPGFRFHPTDEELITHYLSQKVVASCFYATAIIGEADFNKCEPWDLPWRGKMGEKEWYFFCLKDKKYPTGERTNRATGAGYWKATGKDREIYNAKAKALIGMKKTLVFYKGRAPNGEKTNWVMHEYRLEGDNKPSIYNLPKTTKKEWALCRVLHKSEKKVMHVPQPQGLVEFSSYENKELPQLMDSSQVTFFSSDPNNQSEDPNPITRDDDNNNNDDIIVDSIETPFLEQQPPYYSSSYDSSDLDTLNPATWDISENAPTSNASKETDFDADMFSLMYNNREVFQTSFENQEYYAYDSMGHVDNGSLWNF

**>AdNAC43**

MAGASWLVDKSRIATKIKSASGACDPNEVIWKTNPTRICPNCHHPIDNSDKIAYYQFKRWFKGPVLLDIHVTQEWPGLPKGVKFDPSDQEIIWHLLVKAGVGNLKPHPFIDEFITTLEVDDGICYTHPQHLPGVKQDGRASHFFHRAIKAYNTGTRKRRKVHGQDDVRWHKTGRTKLITLNGVPKGCKKIMVLYTNAVRGGKSVKTNWVMHQYHLGTEEDEKEGAYVISKVFYKDDQDIPEAAESKNATVAKVDPVTPKSTTPEPPRNERQDSDLGLDLDLGQEALAFTEMDCLDEIQADCEESAKANPPVLETQENEGMDNKETNAYEAQPWWDSDSQNLLDSQQLVEALTLCEDIFHSQSSNKDDENDKNQTGLSVYAHLGPEHLKKDIEECQKLAPAGPEHQKDIEDGQNLDIDLANIERDTPPEHRLSQLEFGSQDSYTYWGFQGVN

**>AdNAC44**

MTSTKLPSGASKKFKPTDEELIQDFFRNKINGRSLPNYGTILEESMENLGKNVKNSYDGKDLYFFTTLKRKFSTNNLRMVRTIEFGSWEGEDIGK

**>AdNAC45**

MGAVEVFQQQPLVVDAAPVLSLNSLPLGFRFRPTDEELVDFYLRQKINGNGDEVWVIREIDVCKWEPWDLPGTILNSLFFQICQWYGTRIRSGSSSVHRTESGSTVIGMKKTLVFYTGRAPKGKRTNWVMHEYRPTLQELDGTNPGQNPYVLCRLFKKQDESLEGSNGEEMERTTSTNLTANYSPEEIQSDPAVKSVSSSQATEDDKKLAVIPLTPEEAISNVITPVGCQSDGCDAYDAQNQIAAGDPSKEEDLQVNMDIFYDPSELFDDKLFSPLHKHIPEELFHQSNNEANGHFGLQHQCGTNEISISDFFDSVINWDEISGDNSSGQTPNSAWFDVQHNESWGNSNVDMVHARPLQVGGADYPGDATEGKLPLLKTREFNPNTSYDNAISNNMGLFQDHSQMAFSSDVNMLQGYLATNNYEQPTNFNMAMANSDNTGIRIRSRPPGYEGPNANSNMQPQGTAPRRIRLARALAPQHTSNEAAKDSSYESKDRNSQVTTAREMETSKDLAAGESVTVTSDVEEQESSPVENKEFEDFNTVQQSTSSASSNLSTCSSDSEVSYEAEKESGWTSEDHSPKPAAAGASKASEDQVPSECINDITDDVDESRIPNAYTLEVSKEESFSDSESKDSLLRRKVCYPSKSSSNLAKWYSVIAISATLVVLLAFLVNTWGYGYYLKV

**>AdNAC46**

MKVVIARISLPFHPSIIPDLDPSQLHPCKAFSSGNQHYFFTNKVKENRSTENGYWKEIGLSEPIISADANKKLGIKKYFVFTLNEGTETNWVMQEYHISSSMFHNPISCYANGTAHRRLLRPDQNQNNKWVLCRVYEKNKSQSQQGATANSYYSDEDDCGSELSYLDEVYLSLDDDLEVISPPN

**>AdNAC47**

MGGASLPPGFRFHPTDEELLGYYLKRKVEGLEIELEVIPVIDLCKFDPWELPEKSLLANRDMEWFFFCPRDRKYPNGSRTNRATKAGYWKATGKDKKVVCQFDTPSTVTGYRKTLVFYRGRAPLGDRTDWLMHEYHLADDLGLASTCFQGGYALCRVIKKNEKVNNGNDASMRFSNEPFSISADASSSQPSYLNSESVYSSPNASSHNVAPMADSNQASINTSSSSEFWVSPDLILDSSKDYPQLENTFTRCDIPSSTMTPCLSLDQPEISPCSSYSNFNGQLGFSDDFNMIGGMSPYSIQEDFMYFHGNDGDVSYGSYDHINSVEYPEYF

**>AdNAC48**

MGLRDIGASLPPGFRFYPSDEELVLHYLYKKITNEEVLKGTLMEIDLHTCEPWQLPEVAKLNANEWYFFSFRDRKYATGFRTNRATTSGYWKATGKDRTVLDPLTREVVGMRKTLVFYKNRAPNGIKTGWIMHEFRLETPHMPPKGKTDNSAKLSPQFMYEATPSSLTLASSSSSPPTNQTNCNNLHENSTITQLSPKGGGGGDDGGYGFMWDMDLEENSFHDGGVIASNLNDMRFEVDNNTMVML

**>AdNAC49**

MEGSSKSCELLPPGFRFHPTDEELIVYYLCNQATSKPCPASIIPEVDIYKFDPWELPGKAEFGEKEWYFFSPRDRKYPNGVRPNRATVSGYWKATGTDKAIYSKCKHVGVKKALVFYKGRPPKGIKTDWIMHEYRLLQQSNHNSRTTGSMRLDDCVLCRIYKKKHAAKALDQGQEYPTTVQINLNASTNNDDQKELMMMKNLPRTCSLTYLLDMNYFGPISQLLSDGSYNNSSTFEIFQHSNSVDNIGIVDPLVKTQMVEMDDSYYAQDSGKSQVMKQGNDLRGYY

**>AdNAC50**

MDNRLATNSSYASLRLPVGYRFCPSDEVFVSCYLKNKALSKTLDFDVVPVFDVFNTEPKNLPSGGKVFLETKYFYFDLKERVFEDNNKIEAGKGHWKRVGKGNQELLNNNNKLIGFKTKFVFWRKKNRTQFLKTKWVMFEFRVFLNPSQIMSSWAGYKIYLKKDKRRNKKAKFSCEESSDDDEEEAERASEVNFADEISGINTGPPSPTSSNESSVTN

**>AdNAC51**

MGVNEDLMMKDDSYASSVMEEEDDVPLPGFRFHPTDEELVSFYLKRKLDKKPISIELIKQIDIYKYDPWDLPKASGSGGEKEGYFFCKRGRKYRNSIRPNRVTSSGFWKATGIDKPVYSHGGEGSDCIGLKKTLVYYRGSAGKGTKTDWMMHEFRLPSATTENKTSLLANNKNNNNINNADVAQEAEIWTLCRIFKRNVSQRKHTADLRSHLVTANSNKHKTTRTHVVQSNNNNINQHQESYINFGATIIGHHHYHHQNEQKPVTNYTACNNNTDQIQRNNSNHHHHHQLNYHPSSAVATTVPQQQQQQYHHHHQLMTAPASNMWINPSAMNDLFAFDDNWDELGSVLKFA

**>AdNAC52**

MESSCVPPGFRFHPTDEELVGYYLRKKVASQKIDLDVIREIDLYRIEPWDLQERCRIGYEEQNEWYFFSHKDKKYPTGTRTNRATMAGFWKATGRDKAVYDKAKLIGMRKTLVFYKGRAPNGQKTDWIMHEYRLESDENGPPQASLLDYLYYVYEEGWVVCRAFKKRTTNGQTKTMEGWDSSYFFMCKQEIENMHANIAAEQFVQLPQLESPSLPLVKRPTTTTSTMALVSESNEEHNMLSCNNTKKVVTDWRDLDKFVASQLSHGGDNSRHETETDDAAVLPSFMDNNNHDNNGSISDMLMMSMSPFLNTSSDCDIGICVFEN

**>AdNAC53**

MRNLLSITSSARFAAKASDLMQFLRLTSTGANPGTLQSRLKTRDQEWYFFSALDKKYGNGGRMNRATSKGYWKATGNDRPVKHEQRTVGLKKTLVFHSGRAPDGKRTNWVMHEYRLVDEELERARSGSSQPQKDAYVLCRVFHKNNIGPPNGQRYAPFVEEEWDDASALVPGAEPVEDVTVTVAHPLRIESNGRTLCSDRRNNVAQDTQSNNKVPFDVNKLPIETQSLLAVCKRESMAEFPSPEKEDNSKRQIDEYPLPQTENTKPISQIYKRRRHYLNVNHSNVNGDSVRTIQEPPCSSTITTAATTLPTVATTASTAITNVAPKKHFLSALVEFSLMESLESKGNASVQPPEFDDASLEASVPPNCVKLIKRMQGEIYKLSEERETMRFEMMSAQAMINMLESRIEILSKENEELKSMINNNP

**>AdNAC54**

MEGSRRSSNSELPPGFRFHPTDEELIVHYLCNQATSKPCPASVIPEVDIYKFDPWELPDKTSFGENEWYFFSPRDRKYPNGVRPNRATVSGYWKATGTDKAIYSGSKHVGVKKALVFYKGRPPKGIKTDWIMHEYRLVGSRRQPTKQIGSMRLDDWVLCRIYKKRSIAKSMLEPKEEFPTMPQINHHLTSSSNDGNDNNDDEQEMMMKFPRTCSLTHLLEMDYLGPISQILSDGSYNSTFDFQLNSANVGNMIMDPFVKQPQILEIPNKNNPNNPYYDVDSGKNNLVKQNSTINPTIFVNQFFDHSGS

**>AdNAC55**

MVDRDSSGAHMSIAASSMFPGFRFCPTDHELISYYLRKKLDGDEDSVQIISELELCTFEPWDLPEKSFIKSNDEWFFFSRRGRKYPNGSQNKRATKHGYWRVTCNERQIKSGQNVIGTKRTLVFHVGRAPKGQRTEWIIHEYCINDKFQDSLVVCRLKRNTKFHASDSSNKALRKSGGGVSEGVTVQRSTCVPIQDRSNKTSCKSSASRKSSCGVSEGITIQRSTCAPIQDRSNKASRKSSSSRKSSCGVSKGVTVQRSTCVLIQDRINKASRKNCCGVSEEGVTVQRSTCVPIQDKEVGCSSKKGNNNNSSPSTTAQIESSRIVANEANPKASSGHSKVVDEVGYYAEINLVDIINLDETAL

**>AdNAC56**

MEENLPPGFRFHPTDEELITYYLTRKVSENGFTSKAIAVVDLNKSEPWDLPGKASMGEKEWYFFSLRDRKYPTGLRTNRATESGYWKTTGKDKEIFRGGVLVGMKKTLVFYKGRAPRGEKSNWVMHEYRLENKNPFRTKDEWVVCRVFQKSTAAKKPPQQTSSSQPESPCDDTTSLVNEFGDVIELPNLNTNINNNNNSSSSSSSALFPNNILISGQHIHHHHDLTNNNNNNNVNTNMNLAMNWPPSSDHNINNVPWPSVGLLNPSISSMNSLILKALQLRNNYQQREVASTFPPSSYIMPHHQGLVVPHQQVIGTNNNDDLITTSSNLINASSSSSKVLECMPHQQQQQQQEQPFNLDSLW

**>AdNAC57**

MAETRVLPVGYRFRPTEEEILIHYLNNKHLGNDAEIKNTISQVDLCNFDPWDLPEQSKVKSDDQEWFFFNELKYMKNKRCNRKTNMGYWKITGKERIGTDSVIGTKRTLVFYERPHNVKTNWVLHEYHAFDQKVGSCQSNIVLSRVIMNAEKREQKLKTKASNIVEEEEACVSSERQQPQVIDYEILSSGQQSSVAHSGNENNNAAEATWRQDADMNIEYFWNLLFSSIDADPHAEFLNSVLAGDDQLYVDSGHH

**>AdNAC58**

MPESMSISVNGQSQVPPGFRFHPTEEELLQYYLRKKVSYEKIDLDVIRDVDLNKLEPWDIQEKCKIGTTPQNDWYFFSHKDKKYPTGTRTNRATAAGFWKATGRDKVIYSNGKRIGMRKTLVFYKGRAPHGQKSDWIMHEYRLDDNTTNDANIVSNVMGDAAQEEGWVVCRIFKKKNHLKTLDSPLTSSISGDGGRRSHHHHHLFDSCDEGALEQILQQMGRGGGSGGCKEEINNYDQSNNNNNNNYGGSSSLTTRYARPFDTINNNVDSRFLKLPSLESPKSTSMDHNNNNNNNDNDDSNENNGYHPIIPVEMVTENEGSFTCDNPNNMFHHHHLGGGGGGSSDGGGGLTNWVALDRLVASQLNGQTEASRQLSCFNDPTMGYGTGNHDLLFPSVRSTSSLTSSSASINPRAVISAGAGAYISPGAQDYTTTSEIDLWNFARSTSSLLSSSEPLCHVSNTSV

**>AdNAC59**

MEQEEEPQQNEPPHSHSQSRCVTLPPGCRFHPSEELLLSYYLTNKNGTGNWNGNGGLGFDGSDLIRELDFYDYDPFELPDFACFAYGYGGRRRHWYCFTTVRVSRGERWKRKRKVKSGFWLKRGRVSNVNGVGENVVLGTRTRFVFYMGDSAKNGARTDWVLYEYALVDHVMASFVLCRVFSKPRYKNSASDIGLSCCAEESVSAVRHIGIQHDEHVKLDAVEAKVCDDISIDHNNEICAGGNSDNQVKNAHDIDALRCLAAPQGSQQERLPLLPSGSTMFIEAISSQQQLLSITEEDFIELNDLT

**>AdNAC60**

LRILGIYLITSIILECTRPILIDREISHQVIPRTRKIYVQRFYLEFLGARYIKLRRFEMNAFSHVPPGFRFHPTDEELVDYYLRKKIASKRIDLDVIKDVDLYKIEPWDLQEICKIGSDDENEWYFFSHKDKKYPTGTRTNRATKAGFWKATGRDKAIYSSSSHCLVGMRKTLVFYKGRAPNGLKSNWIMHEYRLDSNQEDGWVVCRVFKKRMPTLRNVVDYDDQLPFMQGSPSTHYPCKHELHQFQYNTHDAFLQLPHLESPNQVLSCGSPVIAPYAYAENNNNNNGTSSTSALQSYSSERIQQQLHLLYGSNIEQAVVMDQVTDWRVLDKFVASQLMSQDQDQASKETCSVADEQHVATTVLPNGSTKQEMVPQDDYVSTSASSNCDIHLWN

**>AdNAC61**

MDKDTSLEIHLPPGFRFHPSDEELIVHYLRNKVTSSPLPASFIAEIDLYKFNPWELPSKALFGEEEWYFFTPRERKYPNGVRPNRAAGAGYWKATGTDKPIITSGGMKSIGVKKALVFYKGRPPKGSKTDWIMHEYRLHDSLLSNSHKRGSMRLDEWVLCRVRQKTGSPRSTLEDPSELIYEPTKKIQQMNDENFNPELVKASIVHNEFPMLPYILASRSTLPNSIGVSSSTGFVRNCDMKQYGSVHEDNNLNVIGAQFLASAMEGLYNNPLKRKFIEQEENHLEYAPPNKKISLELGDDVDNSDDKPSLVMDTNKGYNFGFFDQWNSIIQPQELNSLAFMGYS

**>AdNAC62**

MIDMGSSSVIEGEVTLPGFRFHPTEEELLDFYLKNMVVGKKLRFDVIGFLNIYHHDPWDLPGLAKVGEREWYFFVPRDRKHGTGGRPNRTTEKGFWKATGSDRKIVTLSDPKRIIGLRKTLVFYEGRAPRGSKTDWVMNEYRLPDNCPLPKDIVLCKIYRKATSLKVLEQRAAIEEEMKQMVGSPESTPSSTDTMSYEEQQQNQNQNQNLQLLPPQHVVTKKEVEAELEEEKMVHVTLATTKQENKDTTKNNKSSCCGNTNTNSNTSSLQLPFGKDKIPELQMPMMITDWTQDTFWAQLNSPWLQNYTYSNILNF

**>AdNAC63**

MSNISLVEARLPPGFRFHPKDEELVCDYLMKKFTHNESLLMIDVDLNKCEPWDIPETACVGGKEWYFYTQRDRKYATGLRTNRATASGYWKATGKDRPILRKGSLVGMRKTLVFYQGRAPKGRKTEWVMHEFRVEPPLPPPNTTSSKEDWVLCRVFYKNREVGGKPNSMGSCYDDTGSSSLPALMDSYISFDQQQQPQTHLHADEYEQVPCFSIFSHTQTSPIFNHIMEPKLFPTNNNNNNATLYGGGGTTTTPNLGSCLDPFSCDRKVLKAVLSQLTNMERNIPNNNNNNTNSIKGSPSLGEGSSESYLSEVGMPNLWNNY

**>AdNAC64**

MAPVSLPPGFRFHPTDEELVAYYLKRKINGRKIDLEIIPEVDLYKCEPWDLPGRSLLPGKDLEWYFFSPRDRKYPNGSRTNRATKCGYWKATGKDRKVNSQSRAVGMKKTLVYYRGRAPHGSRTGWVMHEYRLHERECETNAASGLQDAYALCRVFKKAAVILPKVADHYAGNNIMMMTTDSQGTPQVFDTMPWDHHIGHNGKCPHLSQDPFLNNLPSSSSSSFPHYGALTYSPSKVDVALECARMQHSFSMPPLEVVEEFPNVGISELNIMTRGTTSMCGGSMNNNNESDILQQILSLANANVSSHEFTNQSNHSHTLLAGNNNANYSAPHHHEHDFAFNAGTSYTNHAVNDMNPMRYEIQHQNLRTIEIGDLESEFKSFMEEQKTVPIEDISSFQTNIQENEVQESELHNSNKEFSEADIDNFSMGFINDGDPNENFIDEDDNIDYSNSTSFEVLEETKVSHGMFVATRRVADTFFHQIVPSQTIKVQLNPVTIMGNNSSMEMLKNNQESLFKKLMMMKSPNTLASAIVFIFALLLTLCVNLKGQVENYWASRSDDDTINVKKKCCYGANRSMKRMKQVAHKIIWNQQEKSWCVGIKSGRGFSVVLKKIGIFLSISLALCTMWVNHVTISP

**>AdNAC65**

MDSFYHHHNHHFDNSDTHLPPGFRFHPTDEELITYYLVKKVLDNTFTGRAIAEVDLNKCEPWELPEKAKMGEKEWYFFSLRDRKYPTGLRTNRATEAGYWKATGKDREIYSSKTCALVGMKKTLVFYRGRAPKGEKSNWVMHEYRLEGKFAYHYLSRSSKDEWVISRVFQKTTTGGGGGGGGGGSAVSTTAGGSKKAKMSTSTTSTMSFCPEPSSPSSVYLPPLLDSSPYTTATTGSVTSAAAAYDGRQSSSFDNNNNNNDSTREHVSCFSTISNNFVNGFFDLAPMDSFARFQRNNNVGVSAFPSLRSLQDNLQINPLFFSAAAAQPLHGGELHAAGTWPVPDDQRVAEAAAAGMALGHSELDCMWGY

**>AdNAC66**

MQGGLELPPGFRFHPSDEELVNHYLCKKCAKQSIAAPIIKEIDLYKFDPWQLPEMALYGEKEWYFFSPRDRKYPNGSRPNRAAGSGYWKATGADKPIGKPKALGIKKALVFYAGKAPKGVKTNWIMHEYRLANVDRSAANKLNNNNLRLDDWVLCRIYNKKGKIEKFNSATTGLEQKLPKFSPGEILHYDHEHEHETKPKIIHNFSNNEHQLYMDTSDSVPRLHTDSSCSDHAVSPDATCDKEVESNPKWSNELDMQLFDTFDFQLNNYDNNLPMNDDDLFGNQFQMNQLMSFQDTFLFPQKPF

**>AdNAC67**

MDMESCVPPGFRFHPTEEELVGYYLKRKINSLKIDLDVIVEIDLYKMEPWDIQDRCKLGYEEQNEWYFFSHKDKKYPTGTRTNRATAAGFWKATGRDKAVMSKNRIIGMRKTLVFYKGRAPNGRKTDWIMHEYRHQTSEHGPPQARWVVCRAFRKPSPSHQRQLGFDPWCSNHHHQAHYFRDQSSYGGRPLSITDLLTSETHHHHLLSHPTEGTNFSHSFGSDHHHHQEQQEFVISNNHQQLIELPQLDSPTTTSFAVKESSSINNNNEEYCSDDRNNNNNNIDWKSLDNLFADTSNYFSSNPNMSQFMTINHHLGCFPGS

**>AdNAC68**

MAPVSLPPGFRFHPTDEELVAYYLKRKINGRKIELEIIAEVDLYKCEPWDLPGKSLLPGKDLEWYFFSPRDRKYPNGSRTNRATKSGYWKATGKDRKVNSQCRAVGMKKTLVYYRGRAPHGSRTDWVMHEYRLDDRECENASSGLQDAYALCRVFKKSAVITPKVDEEHHHHHHYVNANNHNNSSHALPITSDQSSSMELYSEGRGEDLDNSSNYLVPIDTTSTLPLNNMVMNNNNSDASFNSRDNNGKWSQFISEDPLFSFPTSSSSFANSYGSITYPPSKVDIALECARMQHRFTMPPLEVEDFPHVGTSELKMTELASGAGSTVHGTRNETDILQEILSVAHASQELINHSSYSSSWGGDGGGNHENCATHGDDFTFMVGSTNYNNNNLNDINSMRYVDRNWEDPNTSRSIDIGYLDEEFKGERMVENLRWVGMSTKDLEKNFTEEQKIVPIEDISSFQTNNKEENEVQESEQHHSNKELLINDFSLGFNPNNNNSENFLDDDHNNMDNDDYSSSPSFEVIEEIRVSHGSMFVSTRRVADTFFHQIVPSQTVQVHLLNPVITSNEEETLMMIMERNQGYFGDFLFKTIATAFVLIFELLFMHCDYLKEEVELVKRKRSSQSSSKIMKWNNNNNKVWFVGFKSSEKGFGAILKKIGIFLTISLALCTMWANHVIVNP

**>AdNAC69**

MAPMSLPPGFRFHPTDEELVAYYLERKITGRSIELDVIAEVDLYKCEPWDLPDKSFLPSKDMEWYFYSPRDRKYPNGSRTNRATRGGYWKATGKDRAVQSQKKAVGMKKTLVYYKGRAPHGIRTNWVMHEYRLIESLPGTPHSSFKDSFSLCRIFKKTIQVQDKSKEEKEHQALLEEDHSSGIEISREMEAMNDNNNNNITLNSNEQYPNNNNNKLPNCDASSSDLTQGTCTPTETGIADDFHAQFACDEANSAANSYSMGIAYPSNDIEMSMYGSMHNYQFPQTPLVMEDFPQIDFAETKSLKPEVTEDCMFYDRYGRDCMNGTLEEIISLCSSQDNSVALPMLE

**>AdNAC70**

MENMPPGYRFYPTEEELISFYLRNKLEGVREDMNRVIPVLDIYEYSPSELPQISGEASVRDSEQWFFFIPRQESEARGGRPKRLTTTGAPNGTKTDWKMNEYKAIDTHHPSSSSNNRAVPMKAKCLRAFDRRPPPRRDTYPPSQNNGSSSFDHHHQHNQTVEKSSGAGSSPESSCSEDHGQCSHRTEDVENANEPFLDWEQIDWFLGSSLPEP

**>AdNAC71**

MSTQKYTQQAKSGFFNVAGANSIFNQDFSVMKISSKSHLKSGDLEWYFFCARGKNYGIGSKTNRAIKNGYWKATGMDKAIVQHDKQTVGMMKTLVFHTGKPPHGTRTDWVMHEYRLQDKDLTDKGIAQDSYVICKVFRKEGHGPRSGAQYRKPFNEEDWDDDDDDDDDHHVVEEGGTPSTALVAPVSIQSMTLDGSSYMKATSVSCESGPVATSPVPSTPSSDASIHTVNNSTVTDLSKDEKTVPKENIAAGDLLSKFFEGLEDLESEYTPNGMGLDGFAPNGINYDDLGHLDLIDCNFL

**>AdNAC72**

MARSWVIDIGGLAKKVKNNTLPLADQINDCGAYCECPICHYHIDNIDVSPEWPGFPAGVKFDPSDIELLEHLAAKCCVGNKVPHAFIQDFIPTLEGDQGICYTHPENLPGAKKDGASVHFFHKTTNAYATGQRKRRKINHQLGLSEEHVRWHKTGKTKAVTENGVHKGFKKIMVLYVRPKRGAKPNKSKWVMHQYHLGSEEGEKEGEYVVSKIFYQQQKKTKKNKLNPLVAEDSVMALQASPRTPNPNPPKRPRTGKSVDFEETDLMLFTQGGKPTIHGESLEPPPSEVHGDENNGGFNNTALLSVETQPVNSDFIGLDDILLCKEQTLDSSSAHLNDSGLKSNNLKGFACNANGNAGELFGDVNDCYGISVLDNLGLDSPPDFDLSVSTRSPLSS

**>AdNAC73**

MSPVGLPPGFRFHPTDEELVNYYLKRKINGQEIELDIIPEMNDCISKSRTFCLFLEKSFLPSRDPEWYFFGPRDRKYPNGFRTNRATRAGYWKSTGKDRRVSSQSRPIGMKKTLVYYRGRAPQGIRTDWVMHEYRLDDKDSEDTTGLQKNGICTDVEEQGIVVTCLH

**>AdNAC74**

MEEPVVVNKGEEPLDLPPGFRFHPTDEEIITYYLTEKVMNSSFSATAIGEADLNKSEPWDLPKKAKMGEKEWYFFCQKDRKYPTGMRTNRATDSGYWKATGKDKEIFKGKGNLVGMKKTLVFYRGRAPKGEKTNWVMHEFRLEGKFANYNLPKAAKDEWVVSRVFHKNTDVKKTTTPSSSSSIIPGLLRINSIGDDLLDCSTLPPLMDPHPTPLDTTKSDGYYFPSFSSSHQILNIKPEEHNTSHQIPITNYQIPNFNTTLSSSSSHQIRLQNHLNLFSSSSSNNNYHNSSWPSYYDEVHHHHQDDILLRAIASKNYSNGGGGGGGECKVEQFSSGNQSVVSVSQDTGLSNDRTTNDTSSVVSKQQHNNKTLYEDLEGPSSSVAPLSDLECLWDTY

**>AdNAC75**

MEELACELSDHEKRNAQGLPPGFRFHPTDQELITFYLASKVFNTTTTTTHVNFVEVDLNRCEPWELPEVAKMGEREWYLYSVRDRKYPTGLRTNRATAAGYWKATGKDKQVYGGGGLVGMKKTLVFYKGRAPRGQKTKWVMHEFRLDPHSSPSLSKDEWVICRIFHKSGEKRTPTTTPAPLLLHHQQHDPSSLFNDHISHSHNHNQNLLSPLLHPFPIPEETTKTKSSTINSNHYPPPPPSSQHLLKLNKSTKLTKTVPPSPSFFQYQQLLEDDPNLLHWMDSGNNNNNNCKANNTASSVEIMDAAAAGLIAFSSGGPSPTPTNNNNNNSEIIRDMMMMSSSSASMLHILDDAPLGIQSWPHHHHHHLL

**>AdNAC76**

MKSELELPPGFRFHPTDEELVNHYLCKKCASQSIAVPIIKEIDLYKFDPWHLPEMALYGEKEWYFFSPRDRKYPNGSRPNRAAGTGYWKATGADKPIGKPKALAIKKALVFYAGKAPKGVKTNWIMHEYRLANVDRSAGNKKNNLRLDDWVLCRIYNKKGKIEKYNHLGAADHKSASSSEENERKPEVKERLHMDTSSDSVVSADVTWESREVQSEPKWNDLLDQVFDFQLGSFVDFSSAGDDPFAPQLSPWHQDTFITF

**>AdNAC77**

MGIQEKDPLSQLSLPPGFRFYPTDEELLVQYLCRKVAGHHFSLEIIGEIDLYKFDPWVLPSKAIFGEKEWYFFSPRDRKYPNGSRPNRVAGSGYWKATGTDKTITTEGRKVGIKKALVFYIGKAPKGTKTNWIMHEYRLLDSTRKNGSTKLDDWVLCRIYKKNSSAQQKVPNGVVSSSEQYATQYSNGSSSNSSSSHLDEVLESLPEIDDRCFALPRVNSLRALQQQRHHQEDTKVGLLQQQQQQGLVAGTGSFLDWASGPGILNDLGQAQQGIVNYGNDLFVPSVCHVDSNLVPAKIEEEVQSGVKTQSGFFQQGPNPNDFTQAFSNQLDPYGFSRYSVQPVGFGFRQ

**>AdNAC78**

GPGLEKSKEMTWCNRSSVVERGIEIINHPNLNIIAIPRNSNDNNNSVISVTHHANTPPKPTEIRAVTCPSCGHNIQIQQDQGGGIQDLPGLPAGVKFDPNDQEILEHLDAKVQSDVRKLHPLIDEFIPTLEGENGICYTHPEKLPGVSKDGQVRHFFHRPSKAYTTGTRKRRKVHTDQEGSETRWHKTGKTRPISVAGSVKGFKKILVLYTNYGRQKKPEKTNWVMHQYHLGSNEEEKDGELVVSKVFYQTQPRQCANKDPYDERLLMTSQINSVNDISMHALPKNNAGFVDYYNPGFMNMNYEQMNETTSPQLIPNMVVQGDSSSFIRLAMDANKPRLDRK

**>AdNAC79**

MAGSSWLVDKSRIATKIKSASGASGKVLWKSNPTRTCPNCQHVIDNSDVAFTITCSPSPSSHLCFHYCACVPFIYSSFHDFFDHDSVTLAGATNFIGSVVLWILIFLYEARHMKIFERATVTVQVAQEWPGLPKGVKFDPSDQEIISHLLAKVGAAGSEPHPFIDEFIATLEVDDGICYTHPKHLPGVKQDGSATHFFHRSIKAYNTGNRKRRKINDQDSGDVRWHKTGKTKPVISDGVQRGCKKIMVLYMTSVRGVKAEKTNWVMHQYHLGTDEDEKEGEYVISKVFYQQQVKFAEKDDHDVPGTNEATVVKDDPVTSEPPHSEKQCSDLDIGEKSHQIPQGPQTDCVEDIQAECEEIVKTDVAMADAQNNEGMDNVENNADGEQKWWDSESQNLLDSQQLVEALALCDDLLHSQCSNKDDENVEHKEHLSLSIYAHLGPEHLKKDLEECQNLNLDPANVELETPPSEFRLSQLEFGSQDSFVSLSGGKAVD

**>AdNAC80**

MGVPEKDPLSQLSLPPGFRFYPTDEELLVQYLCRKVAGNHFSLPIIAEIDLYKFDPWILPGKAIFGEKEWYFFSPRDRKYPNGSRPNRVAGSGYWKATGTDKVITTEGRKVGIKKALVFYIGKAPKGTKTNWIMHEYRLLNGSQKSLGSTKLDDWVLCRIYKKNLSSSQKVNMPSFTSKEWSNGSSPSSSSHIDDMLELPEIDDRCFALPRVNSLQHEEKLTLGATGNNFPDWVNSGGLDSVPEFGSQTQGMTSYDGNDLYVPSASQFCHVNTMVVPGNPTEEEVQSGIRTQRIDENFGLFQQNSNVFTHRYLSSSGDSFGFGYPNQQFGFGFRE

**>AdNAC81**

MESMENVRMQREKDQKFELPSGFRFHPTDVELINYYLVKKVLDDKHFCSIAIADADMNKSEPWDLPGLAKMGETEWYFFSMKDRKYPTGQRTNRATEAGYWKATGKDKEISKENSKIGMKKTLVFYKGRAPRGEKTNWVMHEYRLEGNKSVYNLSQPERGEWVICRVFEKGNNGKRLNIAKLERLNSSGKEPLPLPKPTPLMPPLMDSSSSRTTPGELSQATCYSSDPNQADVQNNLHDDIVESRETPILNFSPASINEELIQIPNQIENPDYYTLPQENNGSIARQNQKSEFDADISSLIYNNDMFYRFFGNQEHSSSASADICNLWNY

**>AiNAC1**

RVTLFILGVLTYQMENICSEVEMDLPPGFRFHPTDEELISHYLYNKVIDTNFSARAIAEVDLNRSEPWDLPWKAKMGEKEWYFFCVRDRKYPTGLRTNRATEAGYWKATGKDKEIYRGKSLVGMKKTLVFYKGRAPKGEKSDWVMHEFRLHGKFNPHNLPKSAKNEWVICRVFQKSSAAKKIHLTGIMRLDSSVFLPPLADSSSSPSNTATTAPYVPCFSNPIIHNQVGIFDPFSNTPFGADSFYTSQGMPMQHAQPPSCYTTQDHSILRTLLQNNSSNLRSGFKPAEREMAHHQTSLVDANNNNNNNGITSVVAPQDLSSLWNYQVQIK

**>AiNAC2**

MNNNKISNLSSVSSSDLIDAKLEEHQWCGGSKQCPGCGHKFESKPDWLGLPAGVKFDPTDQELIEHLEAKVESKNMKSHPLIDEFIPTIEGEDGICYTHPEKLPGVTRDGLSKHFFHRPSKAYTTGTRKRRKIQNECDLQGGETRWHKTGKTRPVMVNGKQKGCKKILVLYTNFGKNRKPEKTNWVMHQYHLGQHEEEKEGELVVSKIFYQTQPRQCNWSSDRSATTTIATAEGSGEPLQNSRRDSGSGSCSSKEINIGHRDEMSAVVGVNNTPITSFTHPLDIHHHLKSDHFSFIPFRKNFDEVGIGEASTAREVQASGSCDEVVHEHESSITNTQEAEWLKYSSYWPDPDNPDHHG

**>AiNAC3**

MAWCNDTHEKEIIASNNSTITLRPKSDPNVTCPSCGHNIQIIQEQGGIHELPGLPAGVKFDPNDIEILEHLEAKVMSHVPNLHPLIDEFIPTLQDENGICYTHPEKLPGVKKDGQIRHFFHRPSKAYTTGTRKRRKVHTDEDGSETRWHKTGKTRPVVAGGGLVKGFKKILVLYTNYGRQKKPEKTNWVMHQYHLGSNEEERDGELVVSKVFYQTQPRQCGNSIVIKEDNDDDLPYGKILMMNNSSKKHKNNNDRNVAAPVVDYYINYDHVDHHNHNHNSQRCSSPTQLIPNLVLQGDSSSLFRFASSSLDGNTNKTRLFERKL

**>AiNAC4**

MNKMDLIDAKLQEEHQLCASSLKQCPACGHKFEGSSGKKAEWEWVGLPAGVKFDPTDQELIEHLEAKVEAKRSHPLIDEFIPTIEGEDGICYTHPEKLPGVTRDGLSRHFFHRPSRAYTTGTRKRRKILQNDEAEAERGETRWHKTGKTRAVMLKGKQKGCKKILVLYTNFGKNRKPQKTNWVMHQYHLGLHEEEKDGELVVSKIFYQTQPRQCSWSSSSSSSSITAVAPPVKTNNDTCPVLGFPPMEHFSSFIPLRKTLHNEEVGIGGETCTPASHIPSSNPVGVFHHNTSIILDDLISARFMTPPPPPQFHQQHDNKVVGGTSASGLEELIMGCTSTSTTHNITKEASMSNTNPQEAEWLKYSSYWADPQPQPQPHLHG

**>AiNAC5**

MVDRDSSEAHMSIAASSIFPGFRFCPTDEELISYYLRKKLDGDEDRKSFIQSDNEWFFFSPRGRKYPNGSQSKRATECGYWKATGKERVVKSGQNVIGTKRTLVFHLGRAPKGERTEWIMHEYCVNDKSQDSLVICRLKRNTEFRASDHSNRTSHDSDCGVSEGVTVQGDTYVPIQDKETGCSSKRTSSSNSSPSTTGQIESSHRVNEEEDCYAEILNDDIIKLDESTLSRPSPPQGTANRRIRLRVPKSTVPKSRVPKSRVPTGNGCQCSKQSSNKINTFLSYPLVVFTFFVFTLLALGFFFIRRSQTTAQYSQDLSRV

**>AiNAC6**

MGAVVDCYPPHAGEVAVLSLNSLPLGFRFRPSDEELVDYYLRQKINGNGEEVWVIREIDVCKWEPWDLPDLSVIRNKDPEWFFFCPQDRKYPNGHRLNRATNHGYWKATGKDRKIKSGSTLIGMKKTLVFYTGRAPKGKRTNWVMHEYRPTLKELDGTNPGQNAYVLCRLFKKQDESLEVSNCDEVEQTDSAPMAANYSPEEIQSDQALAEVSPSQVTDEKHQGVIPENSEEAVSNVITSADCHSDGYDACERRNQAFELPAEDIPPLNWDIFNDPEDKIFDDKLFSPVHSHIPPEFYYQANNETNIADILNSVNWDEISYEDPYSQAQNNFFNNVKQSVSGSEPDAGLTNMTCVHPTNVVYPEEAIHRKVALATTPQFCSTFTSDFSADEQKSSVALIQNNSQMASFPDARTVQVYNVFNDYEQPRNLNTYVSGDTGIKIRTRQVRNEQPAMIFTDQGNAARRIRLLKQCADVSNKMADDGSPKQEHDSKPIIAGNKNKTFKSHTADKHDTANDLNERQEKTESTDKRNMISKLAKGGSSMLGLKGLLRRRLSYISKASSNFKMWSCVVVASAFVLVSFVFFANIWGYINL

**>AiNAC7**

MASELQLPPGFRFHPTDEELVLHYLCRKCTSQPIAVPIIAEIDLYKYDPWDLPGMASYGEKEWYFFSPRDRKYPNGSRPNRAAGTGYWKATGADKPIGQPKPVGIKKALVFYSGKAPKGDKTNWIMHEYRLADVDRSVRKKNSLRLDDWVLCRIYNKKGSIEKQQPSSGVSTVVNQKAESSEVEDKKPDIVPRGGGGGVLPPHPPTAQASAGGVTTDYMYFDNSDSVPKLHTDSSCSEQVVSPEFASEVQSEPKWNEWDKNLESAYNYLDATLTNGFGFPFQGNNQMSPLQDMFMNLPKPF

**>AiNAC8**

MASWVIDIGGLAKKVKNNTLPLADQINDCGAYCECPICHYHIDNIDVSPEWPGFPAGVKFDPSDVELLEHLAAKCCVGNKVPHAFIQDFIPTLEGDQGICYTHPENLPGAKKDGASVHFFHKTTNAYATGQRKRRKINHQLGLSEEHVRWHKTGKTKAVTENGVHKGFKKIMVLYIRPKRGAKPNKSKWVMHQYHLGNDEGEKEGEYVVSKIFYQQKKKTKKNKLNPLVAEDSVMALQASPRTPNPNPPKRPRTGKSVDCDDNFDETDLMLFTQDGKPTIHGESLAPPPSEVHGDENNGGFNNTALLSVETQPVENSDFIGLDDILLCKEQTLDSSSAYLNDSGLKSNNLKGFACNANGNAAELFGDVNDCYGISVLDNLGLDSPPDFDLSNLQFCSQDNIIDLQWLDIP

**>AiNAC9**

MGVPEKDPLSQLSLPPGFRFYPTDEELLVQYLCRKVAGNHFSLPIIAEIDLYKFDPWILPGKAIFGEKEWYFFSPRDRKYPNGSRPNRVAGSGYWKATGTDKVITTEGRKVGIKKALVFYIGKAPKGTKTNWIMHEYRLLNGSQKSLGSTKLDDWVLCRIYKKNLSSSQKVNMPSFTSKEWSNGSSPSSSSHIDDMLELPEIDDRCFALPRVNSLQHEEKLTLGGTGNNFPDWVNSGGLDSVPEFGSQSQGMTSYDGNDLYVPSASQFCHVNTMVVPGNPTEEEVQSGIRTQRIDENFGLFQQNSNVFTHRYLSSSGDSFGFGYPNQQFGFGFRE

**>AiNAC10**

MVDRDSSEAHMSIAASSMFPGFKFCPTDGELISYYLRKKLDGDEDSVQIISELELCTFEPWDLPEKSFIKSNDEWFFFSRRGRKYPNSSQNKRATKSGYWKVTGKERQIESGQNVIGTRRTLVFHVGRVPKGERTEWIIHEYCINDKFQSSNQIRILWWFVGSRRTQNFIQMMILTKLHARVVVESQKGVTVQRSTCVPIQDKEVGCSSKRSNNSNSSPSITVQIESSNRVANEVNPKASSNESSDRVANEANPKASSNHSKVDEVDYYAEINLDDIINLDEPAL

**>AiNAC11**

MHKNTRDVNSYVKGYLINWVKCSCYITIYIKTNNNRNNNIEMNTKIELPPGFRFHPTDEELITHYLSQKVVGSCFYATAIIGEADFNKCEPWDLPCQYLNFFNDIIFYLASFDLFLQNLFLFIILIFFNEGRGKMGEKELYFFCLKDKKYPTGERTNRATGAGYWKATGKDREIYNAKAKALIGMKKTLVFYKGRAPNGEKTNWVMHEYRLEGDNKPSIYNLPKETKKEWALCRVLHKSEKKVMHVPQPQGLVEFSSYENKELPQLMDSSQVTFFSSDPNNQSEDPNPITRDDDNNNDDIIFDSIETPFLEQQPPYSSSYDSSDLDTLNPATWDISENAPTSNASKETDFDADMFSLMYNNREVFQTSFENQEYYAYDSVGHVDNGSLWNF

**>AiNAC12**

MEENLPPGFRFHPTDEELITYYLTRKVSENGFTSKAIAVVDLNKSEPWDLPGKASMGEKEWYFFSLRDRKYPTGLRTNRATESGYWKTTGKDKEIFRGGVLVGMKKTLVFYKGRAPRGEKSNWVMHEYRLENKNPFRTKDEWVVCRVFQKSTAAKKPPQQTSSSQPESPCDDTTSLVNEFGDVIEFPNLNTNINNTNNNNNNSSSSSSSALFPNNILISGQHIHHHHDLTNNNNNNNVNTNMNLAMNWPPSSDHNVPWPSVGLLNPSISSMNSLILKALQLRNNYQQREVASTFAPSSYIMPHHQGVVVPHQQVIIGTNNDDLITTSSNLINASSSSSSKVLECMPHQQQQQQEQPFNLDSLW

**>AiNAC13**

MEGSRRSSNSELPPGFRFHPTDEELIVHYLCNQATSKPCPASVIPEVDIYKFDPWELPDKTSFGENEWYFFSPRDRKYPNGVRPNRATVSGYWKATGTDKAIYSGSKHVGVKKALVFYKGRPPKGIKTDWIMHEYRLVGSRRQPTKQIGSMRLDDWVLCRIYKKRSIAKSMLEPKEEFPTMPQINHHLTSSSNDGNDNNDDEQEMMMKFPRTCSLTHLLEMDYLGPISQILSDGSYNSTFDFQLNSANVGNMIMDPFMKQPQILEIPNKNNHNNPYYDVDSGKNNLVKQNSTINPTIFVNQFFDHSGS

**>AiNAC14**

MHSLFPLPTKTPGYNFPLTLPTVAFFVSLSIENGKPMLKLNTLHSSASAQASSSSSLQWLGLEGEGKEVKGLKSKRSWLIDIGGFAKKVKSTNLSPADQIKDCGAYRDCPNCHYRIDNRDVSTEWPGFPVGVKFDPSDVELLEHLAGKCGIGNAQLHMFINEFIPTIEEEEGICYTHPENLPGVKKDGSSAHFFHRTTNAYTTGQRKRRKIHHQCLTEEHVRWHKTGKTKAILEDGVHKGFKKIMVLYIRPKKGSKPDKTNWVMHQYHLGTDEEEKNGEYVVSKIFQKQTEKNEENPAVEDSDQNEKNENRLADDSNCIASRTSPRTPKPNPPNPPRAGNFVDNDDNIDETELPFTQDVKCVPQCDVLDQNNAGDPAWLAGESQAVENFDFDGLDDILFCNEIFDSSSLLDISGTETMINGSASNDMLGNDGLSYGTSVLDTLDLGTPPDFDLSNLNFYSQDSIFDWVDRL

**>AiNAC15**

MDVAKLYMNNDYSEEHEHEHEDEDHEMMKEEKEVVLPGFRFHPTDEELVGFYLRRKVEKKPLKIELIKHVDIYKYDPWDLPSTKTDWMMHEFRLPPNNNNGAKLLSNNQEANNATKDLHEAEVWTLCRIFKRIPTYKKYTPNLKDSSTSPLMNKPINNINHQTDSSVTSISCSLESDNNNSKPFLTFTNTMTMGIQQCERKPLVIGHVDERNNNFFLDHSSIHHQQAPTTITTTALSSSSYSSWNQHHLVEDYLFANENWDDLRSVVEFATDPNNSKVYL

**>AiNAC16**

MGAEAGATECFSKAMASMPGFRFHPTDEELVMYYLKRKICGKKLKLDVILETDVYKWDPEELPGISVLRTGDRQWFFFTHRDRKYPNGARSNRATRQGYWKATGKDRNVTCNSRSVGVKKTLVFYRGRAPNGERTDWVMHEYTMDEEELNRCQDIKDYFALYKLYKKSGPGPKNGEQYGAPFKEEEWADDECVDFNINSADREVVNTVPVNDQLPPLADDEVTDMINQILDNELALDQQFGDDLLEFPQVVAEETQSTVVDQFSEAVTDPEYNDIYHSTSQHYDAQNVNFNQSVASHLHAPEGSEVISTANIQVEDYNFQEDDFLEINDLNGSELTIPNMETPVENLQFEDGLCELDLFQDAEMFLRDLGPINEETIPHSYMNNAPGSNIENQNYHLLPNPEDTTQNVHEFWMHDERNTLSLFEGFDDSVSQQNPGAVCDSASFPTTEGYDNQSSIAEDVATSRFSSALWSFVESIPTTPASAAENALVNRALNRMSSFSRVKINIKPTNTAAGKDTATTKRVGRKGFSFLFFPILIALCAFLWVSLGTFRLLGRCIAP

**>AiNAC17**

MAAMKSIPGYRFHPTDVELVQYFLKRKVMGKRFPCDVIAELDIYKYPPWDLPDHSLLKTGDLEWYFFCPRGKKYSSGGRMNRATECGYWKTTGKDRSVENKKLVVGMIKTLVFHNGKAPKGDRTDWVLHEYRLQDKDLADKGVQQDSYVICKVFQKDGPGPRNGAQYGRPFNEEDWDKEDEIDCVESAPVAALPAAVPIQPASCHSSVVNNVNLSVSECYGLTSVSCLTGPMPSCSAHPSAPSNQVDGDITPVPGSAIEDNIMAPTENTTTEKVDNPPDINNAEGTPCFDPNEIFGGLGDLDGLFEMGGIGHGFSCGQNGGYTVNEMLSASDGLRFPDPLDYLELGDLDTPLLWETNEQGNWSQDNK

**>AiNAC18**

MRTLHTEVAKLNANEWYFFSFRDRKYATGFRTNRATTSGYWKATGKDRTVLDPLTREVVGMRKTLVFYKNRAPNGIKTGWIMHEFRLETPHMPPKEDWVLCRVFHKGKTDNSAKLSPQFMYEATPSSLTLASSSSSPPTNQTNCNNLHVIGYNQLPNFSSSSSPMAIHHNHHHHHHHQNQNGSSSLMNLLQFSTKENSTITQLSPKGGGGGGGGDDGGYGFMWDMDLEENSFHDGGVIASNLNDMRFEVDNNTMVML

**>AiNAC19**

MDSFYHHHNHHFDNSDTHLPPGFRFHPTDEELITYYLVKKVLDNTFTGRAIAEVDLNKCEPWELPEKAKMGEKEWYFFSLRDRKYPTGLRTNRATEAGYWKATGKDREIYSSKTCALVGMKKTLVFYRGRAPKGEKSNWVMHEYRLEGKFAYHYLSRSSKDEWVISRVFQKTTTGGGGGGGGSAVSTTAGGSKKAKMSTSTTTSTMSFCPEPSSPSSVYLPPLLDSSPYTTATTGSVTSAAAAYDGRQSSSFDNNNNDSTREHVSCFSTISNNFVNGFFDLAPMDSFARFQRNNNVGVSAFPSLRSQQDNLQINPLFFSAAAAQPLHGGELHAAGAWPVPEDQRVAEAAAAGMALGHSELDCMWGY

**>AiNAC20**

MAPVSLPPGFRFHPTDEELVAYYLKRKINGRKIELEIIAEVDLYKCEPWDLPGKSLLPGKDLEWYFFSPRDRKYPNGSRTNRATKSGYWKATGKDRKVNSQCRAVGMKKTLVYYRGRAPHGSRTDWVMHEYRLDDRECENASSGLQDAYALCRVFKKSAVITPKVDEEHHHHHHYVNANNHNNSSHALPITSDQSSSMELYSEGRGEDLDNSSNYLVPIDTTCTLPLNNMVMNNNNSDASFNSRDNNGKWSQFVSEDPLFSFPTSSSSFANSYGSITYPPSKVDIALECARMQHRFTMPPLEVEDFPHVGTSELKMTELTSGAASAVHGTRNETDILQEILSVAHASQELINHSSYSSSWGGDGGGNHENCATHGDDFTFMVGSTNYNNNNLNDINSMRYVDRNWEDPNSSRSIDIGYLDEEFKGERMVENLRWVGMSTKDLEKNFTEEQKIVPIEDISSFRTNNKEENEVQESEQHHSNKELLINDFSLGFNPNNNNSENFLDDDHNNMDNDDYSSSPSFEVIEEIRVSHGSMFVSTRRVADTFFHQIVPSQTVQVHLLNPVITSNEEETLMMIMERNQGYFGDFLFRTIATAFVFIFALVFVHCDYLKEEVELVKRKRSSQSSSKIMKWSNNNKVWFVGFKSSEKGFGAILKKIGIFLTISLALCTMWANHVIVNP

**>AiNAC21**

MKATESWVTSFCMLVCDLLSGLCYMGINGFNFKLNMICLEKIAYYQFKRWFKGPVLLDIHVTQEWPGLPKGVKFDPSDQEIIWHLLVKAGVGNLKPHPFIDEFITTLEVDDGICYTHPQHLPGVKQDGRASHFFHRAIKAYNTGTRKRRKVHGQDDVRWHKTGRTKLITLNGVQKGCKKIMVLYTNAVRGGKSEKTNWVMHQYHLGTEEDEKEGEYVISKVFYKEDQDIPEAAESKNATVAKVDPVTPKSTTPEPPRNERQDSDLGLDLDLGQEALAFPEMDCLDEIQADCEESAKANPPVLETQENEGMDNKETNAYEGQLWWDSDSQNLLDSQQLVEALTLCEDIFHSQSSNKDDENDKNQTGLSVYAHLGPEHLKKDIEECQKLAPAGPEHQKDIEDGQNLDIDLANIERDTPPEHRLSQLEFGSQDSYTYWGFQGFWMWTRERKMHLDFYSIQAAMELSNLRLKNSNVGFVLQYQVVSGCEFYPNPQLYDGVG

**>AiNAC22**

MKSELELPPGFRFHPTDEELVNHYLCKKCASQSIAVPIIKEIDLYKFDPWHLPEMALYGEKEWYFFSPRDRKYPNGSRPNRAAGSGYWKATGADKPIGKPKALAIKKALVFYAGKAPKGVKTNWIMHEYRLANVDRSAGNKKNNLRLDDWVLCRIYNKKGKIEKYNHLGAADHKSASSEEENERKPEVKERLHMDTSSDSVVSADVTWESREVQSEPKWNDLLDQVFDFQLGSFVDFSSAGDDPFAPQLSPWHQDTFITF

**>AiNAC23**

MMMMTTTSTTADYESVKQLPPGFLFSPTDEELVLHFLYAKASLLPCHPNIIPDLDLSLAHPSQLNDKALSSGNQYYFFSKVKEKRITENGYWKEIGESEAILSSTVEKKVGTKKNLVFHIGEAPHGIETSWVMQEYHICRSSNIISTSRARRKHDHQIWSKWVLCKVYEKKGSVRGVNYCSDDDDSGTELSWLDEIYLSLDDDLEEISVSILD

**>AiNAC24**

MTNLPPGFCFSPTDEELILHFLYSRISLPFHPSIIPDLDPSQLHPCKAFSSGNQHYFFTNKVKENRSTENGYWKEIGLSEPIISADANKKLGIKKYFVFNLNEGTETNWVMQEYHISSSMFHNPISCYANGTAHRRLLKPDQNQNNKWVLCRVYEKNKSQSQQGATANSYYSDEDDCGSELSYLDEVYLSLDDDLEVISPPN

**>AiNAC25**

MTECNEHENNHGNIIVEGRKDSLIRTCPTCGHHIKCQDQCNEHENNHGNIIVEGRKDSLIRTCPTCGHHIKCQDQGGGLHDLPGLPAGVKFDPTDQEILEHLEAKVRSDIHKLHPLIDEFIPTLEGENGICYTHPENLPGVSKDGLIRHFFHRPSKAYTTGTRKRRKVNSDEEGNETRWHKTGKTRPVYIRGKLKGYKKILVLYTNYGGKQRKPEKTNWVMHQYHLGNDEEEKEGELVVSKVFYQTHPRQCSSLLVNNNKDSSTTLVKGNNNNGFVEYYHSNFISFDQGEHQHRSSGAQVVISHFPLHEAAPNYHSLNQKE

**>AiNAC26**

MENMNSFCHVPPGFRFHPTDEELVDYYLRKKVSSRKIELDVIKDVDLYKIEPWDLQEICRIGREEENEWYFFSHKDKKYPTGTRTNRATAAGFWKATGRDKAIYSKHDLIGMRKTLVFYKGRAPNGQKSDWIMHEYRLETDENAAPQAIFKKRVTTMRKMMMREHDESPNSSCWYDDQEFMMMESPTKQQSSILLHQSTNNHSNLMQLPPYPLIKKELHHPSSSSSSYPFLQLPLLESHQQSAAAPSSISEQLIMPPPIGGGEQVPSFQSFFNNEQQEVGVLDWRVLDKFVASQLSQDDNHASSNSIVQDLTQEIVMVPHNDAASTSNSLTSPIDLWK

**>AiNAC27**

MAPVSLPPGFRFHPTDEELVAYYLKRKINGRKIDLEIIPEVDLYKCEPWDLPGRSLLPGKDLEWYFFSPRDRKYPNGSRTNRATKCGYWKATGKDRKVNSQSRAVGMKKTLVYYRGRAPHGSRTGWVMHEYRLHERECETNAASGLQDAYALCRVFKKAAVIIPKVADHYAADNIMMMTTDSQGTPQVFDTMPWDHHIGHNGKCPHLSQDPFLNNLPSSSSSSFPHYGALTYSPSKVDVALECARMQHSFSMPPLEVVEEFPNVGISELNIMTRGTTSMCGGSMNNNNESDILQQILSLANANVSSHEFTNHSNHSHTLLGGNNANYAAPHHHEHDFAFNAGTSYTNHAVNDMNPMRYEIQDQNLRTIEIGDLESEFKSFMEEQKTVPIEDISSFQTNIQENEVQAESELHNSNKEFSEADIDNFSMGFINDGDPNENFIDDDDNIDYSNSTSFEVLEETKVSHGMFVATRRVADTFFHQIVPSQTIKVQLNPVTIMGNNSSMEMLKNNQESLFKKLMMMKSPNTLASAIVFIFALLLTLCVNLKGQVENYWASRSDDDTINVKKKCCYDANSSMKRMKQVAHKIIWNQQEKSWCVGIKSGRGFSVVLKKIGIFLSISLALCTMWVNHVTISP

**>AiNAC28**

MARSATIPFPILDFIPVGFRFKPTDEELVSYYLNHKLLNDNFPIDIIPDIDLCKVEPWQIPALSKVKSDDPEWFFFSGRDYKYGKSKRSNRATKGGYWKATGQDRFIKERGTMNVIGSKKTLVFYSGRVPNGVKTNWVIHEYHATTFDDSQRNFVLCRLMKKVERKSEDGTDAQACDEGEPSTHMEEADESVSTMFDSPDVDMDSIFHTLPQDRSSSQHSPVGIEQQESFPFSPSENYYLVNEDSSMHIQFETNEEKQDAEKFADSILDSGNIAMFEERQQHHTFMNNHLRSVPSMRVCYESSDTDAEVVSRRADSREYHVSKMVQSSHSAARTDKTRSISSEDFWGVDSSSCDSNADKPFEINSIEISSPPPALSGSKNQYNPRLSQTHRKVSSNAIPNLEDKKKLTTVEQSRRDQEKARKTSPGKKLETRSSDVNRIGSFIHLEPCSSSESLTPRAVYLVNVVIGILLLLAISWDVLSC

**>AiNAC29**

MESMENVRMQREKDQKFELPSGFRFHPTDVELINYYLVKKVLDDKHFCSIAIADADMNKSEPWDLPGLAKMGETEWYFFSMKDRKYPTGQRTNRATEAGYWKATGKDKEISKENSKIGMKKTLVFYKGRAPRGEKTNWVMHEYRLEGNKSVYNLSQPERGEWVICRVFEKGNHGKRLNIAKLERLNSLGEEPLPLPKPTPLMPPLMDSSSSRTTPGELSQATCYSSDPNQAEVRNNLHDDIVESRETPILNFSPASINEEFFQIPNQIEKPDCYTPPQENNGSIARQNQKSEFDADISSLIYNNDMFYRFFGNQEHSSSASADICNLWNY

**>AiNAC30**

MEELACELSDHEKRNAQGLPPGFRFHPTDQELITFYLASKVFNNTNATTATTTTTHVNFVEVDLNRCEPWELPEVAKMGEREWYLYSVRDRKYPTGLRTNRATAAGYWKATGKDKQVYGGGGLVGMKKTLVFYKGRAPRGQKTKWVMHEFRLDPHSSPSLSKHQQQHDPLLLFQTPSSLFNDHISHSHNHNQNLLSPLLHPFPIPEETTKTRSSTINSNHYPPPPPSSQHSLKLNKSTKLTKTVPPSPSFFQYQQLLEDYPNLLHWIDSGNNNNNNCNANNTASSVEIMDAAAAGLIAFSSGGPSPTPNNNNNNNNNNAEIMMMSSSSASMLHILDDAPLGIQSWPHHHHHHLL

**>AiNAC31**

MPESMSISVNGQSQVPPGFRFHPTEEELLQYYLRKKVSYEKIDLDVIRDVDLNKLEPWDIQEKCKIGTTPQNDWYFFSHKDKKYPTGTRTNRATAAGFWKATGRDKVIYSNGKRIGMRKTLVFYKGRAPHGQKSDWIMHEYRLDDNTTNDANIVSNVMGDAAQEEGWVVCRIFKKKNHLKTLDSPLTSSISGDGGRRSHHHHHLFDSCDEGALEQILQQMGRGGGGGCKEEINNYDQSNNNNNNNNNNYGGSSSLTTRYARPFDTINNNVDSRFLKLPSLESPKSTSMDHNNNNNNDNDDSNENNGYHPIIPVEMVTENEGSFTCDNPNNMFHHHHLGGGGGGGSDGGGGLTNWVALDRLVASQLNGQTEASRQLSCFNDPTMGYGTGNHDLLFPTVRSTSSLTSSSASINPRAVISAGAGAYISPGAQDYTTTSEIDLWNFARSTSSLLSSSEPLCHVSN

**>AiNAC32**

MSNISLVEARLPPGFRFHPKDEELVCDYLMKKFTHNESLLMIDVDLNKCEPWDIPETACVGGKEWYFYTQRDRKYATGLRTNRATASGYWKATGKDRPILRKGSLVGMRKTLVFYQGRAPKGRKTEWVMHEFRIEPPLPPPNTSSKEDWVLCRVFYKNREVGGKPNSMGSCYDDTGSSSLPALMDSFISFDQQQQPQTHLHADEYEQVPCFSIFSHTQTSPIFNHIMEPKLFPTNNNNNNATLYGGGGTTTTSNLGSCLDPFSCDRKEDWVLCRVFYKNREVGGKPNSMGSCYDDTGSSSLPALMDSFISFDQQQQPQTHLHADEYEQVPCFSIFSHTQTSPIFNHIMEPKLFPTNNNNNNATLYGGGGTTTTSNLGSCLDPFSCDRKAL

**>AiNAC33**

MDKDTSLEIHLPPGFRFHPSDEELIVHYLRNKVTSSPLPASFIAEIDLYKFNPWELPRKYPNGVRPNRAAGAGYWKATGTDKPIITSCGMKSIGVKKALVFYKGRPPKGSKTDWIMHEYRLHDSLLSNSHKRGSMRVGQLHP

**>AiNAC34**

QIYVQGFYLEFLGAGYIKLRRFEMNAFSHVPPGFRFHPTDEELVDYYLRKKIASKRIDLDVIKDVDLYKIEPWDLQEICKIGSEDENEWYFFSHKDKKYPTGTRTNRATKAGFWKATGRDKAIYSSSSHCLVGMRKTLVFYKGRAPNGLKSNWIMHEYRLDSNQEDGWVVCRVFKKRMPTLRNVVDYDDQLPFMQGSPSTHYPCKHDLHQFQYNTHDAFLQLPHLESPNQVLSSPYAYAENNNNNNGTLQSYSSERIQQQLHLLYGSNIEQAVVVDQVTDWRVLDKFVASQLMSQDQDQASKETCSVADEQHVATTVLPNGSTKQDDYVSTSASSNCDIHLWN

**>AiNAC35**

MGIQEKDPLSQLSLPPGFRFYPTDEELLVQYLCRKVAGHHFSLEIIGEIDLYKFDPWVLPSKAIFGEKEWYFFSPRDRKYPNGSRPNRVAGSGYWKATGTDKTITTEGRKVGIKKALVFYIGKAPKGTKTNWIMHEYRLLDSTRKNGSTKLDDWVLCRIYKKNSSAQQKVPNGVVSSSEQYATQYSNGSSSNSSSSHLDEVLESLPEIDDRCFALPRVNSLRALQQQRHHQEDTKVGLLQQQQQQGLVAGTGSFLDWASGPGILNDLGQAQQGIVNYGNDLFVPSVCHVDSNLVPAKIEEEVQSGVKTQSAFFQQGPNPNDFTQAFSNQLDPYGFSRYSVQPVGFGFRQ

**>AiNAC36**

MPGFRFHPTDEEIVGFYLKRKIQQKSLPIELIKQVDIYKYEPWDLPNEKIWPNRVTRCGFWKATGTDRPIYSSEAQSIIGLKKSLVFYRGRAAKGFKTDWMMHEFRLPSLSSDSAKKCSDKTTPASDSWAICRIFKKTNTMSMAQKASLSHHPYNWNHHNQLFDDILTHQQHQHPIIPNSNNNFIFYNSNSTLEPTKEIDATTTSSSIVISSNIGLHEDPNHHHYNNNSSGFSSSSIMMMQPNIMATSDDDDSGVITTIAGFPFNLPPNDDDAAWNNNIKPNTTLPWDYLSDMSTTYSTNKSYT

**>AiNAC37**

MAGSSWLVDKSRIATKIKSASGASGKVLWKSNPTRTCPNCQHVIDNSDVAFTITCSPSPSSHLCFHCCACVPFIYSSFHDFFDHDSVTLAGATNFIGSVVLWILIFLYEARDMKIFERATVAQEWPGLPKGVKFDPSDQEIISHLLAKVGAAGSEPHPFIDEFIATLEVDDGICYTHPKHLPGVKQDGSATHFFHRSIKAYNTGNRKRRKINDQDSGDVRWHKTGKTKPVVSDGVQRGCKKIMVLYMTSVRGVKAEKTNWVMHQYHLGTDEDEKEGEYVISKVFYQQQVKFGEKDDHDVPGTNEATVVKDDPVTSKSLTSEPPHSEKQCSDLDIGEKTHQILQGPQTDCVEDIQVECEEIVKTDVSMADAQNNEGMDNVENNADGEQKWWDSESQNLLDSQQLVEALALCDDLLHSQCSNKDDENEEHKEHLSLSIYAHLGPEHLKKDLEECQNLTLDPANVELETPPSEFRLSQLEFGSQDSFVSFSGGKAVD

**>AiNAC38**

MEKVASLVLKEEEQMDLPPGFRFHPTDEELITHYLYKKVIDTNFAARAIGEVDLNRCEPWDLPWKAKMGEKEWYFFCVRDRKYPTGLRTNRATESGYWKATGKDKEIFRGKSLVGMKKTLVFYKGRAPKGEKTNWVMHEFRLEGKFSIHNLPKTAKNEWVICRVFRKSSAGKKVHISGIMRLDTFRTELDSSGLPPLTETSPSFDTIHDESPYVPCFSNPIDVPRNQAAGGSGGGGGVFGGSFPNNSSSSVSAYAVSSNILPRMPICGGSLYSTQHQDQSILRALYESNEREMISVSQETGLTTEMNVETNSVVSNFDIGRAHFESLWNY

**>AiNAC39**

MRSLLSITSSARFVAKASDLMQFLRLTSTGANPGTLQSRLKTRDQEWYFFSALDKKYGNGGRMNRATSKGYWKATGNDRPVKHEQRTVGLKKTLVFHSGRAPDGKRTNWVMHEYRLVDEELERARSGSSQPQKDAYVLCRVFHKNNIGPPNGQRYAPFVEEEWDDASALVPGAEPVEDVTVTVAHPLRIESNGRTLCSDRRNNVAQDTQSNNKVPFDVNKLPIETQSLLAVCKRESMAEFPSPEKEDNSKRQIDEYPLPQTENTKPISQIYKRRRHYLNVNHSNVNGDSVRTIQEPPCSSTITTAATTLPTATTTASTAITNVAPKKHFLSALVEFSLMESLESKGNPSVQPPEFDDASLEASVPPNCVKLIKRMQGEIYKLSEERETMRFEMMSAQAMINMLESRIEILSKENEELKSMINNNP

**>AiNAC40**

MNRITKSGYWKATGSDKRIISTSCNNNNNSNIVGIRKTLVFYHGKSPNGSRTHWIMREYRLVTTPSNSSQKYVEDLGNWVLCRIFKKKRSIESQHHMVNNKINNVVEILIIFHNVT

**>AiNAC41**

MGLRDIGASLPPGFRFYPSDEELVCHYLYKKITNEQLLKGTLVEIDLHICEPWQLPEVAKLNANEWYFFSFRDRKYATGFRTNRATTSGYWKATGKDRTVQDPLTQEVVGMRKTLVFYRNRAPNGIKTGWIMHEFRLETPHMPPKEDWVLCRVFHKSKEENSAKLIMYDSISTHHQSSNPMALVSTHHLNPINNHNNTYHAMNNFLHHFSSSRDDSQTNNANNNNSSVTQISPKGYDGYGFIWDHMDLEDGGVPSSDFQ

**>AiNAC42**

MESSCVPPGFRFHPTDEELVGYYLRKKVASQKIDLDVIREIDLYRIEPWDLQERCRIGYEEQNEWYFFSHKDKKYPTGTRTNRATMAGFWKATGRDKAVYDKAKLIGMRKTLVFYKGRAPNGQKTDWIMHEYRLESDENGPPQASLLDYLYYVYEEGWVVCRAFKKRTTNGQTKTMEGWDSSYLYEEGSGGGGGPNMHANIAAEQFVQLPQLESPSLPLVKRPTTSTMALVSESNEDHNMLSKKVVTDWRDLDKFVASQLSHGGDSSRHETETDDAAVLPSFMDNNNHDNSDSISDMLMMSMSPFLNTSSDCDIGICVFEN

**>AiNAC43**

MTWCNRSSVVEGGIEIINHPNLNIIAIPRNSNDNNNSVISVNHANTPPKPTEIRAVTCPSCGHHIQIQQDQGGGIQDLPGLPAGVKFDPNDQEILEHLDAKVQSDVRKLHPLIDEFIPTLEGENGICYTHPEKLPGVSKDGQVRHFFHRPSKAYTTGTRKRRKVHTDQEGSETRWHKTGKTRPISVAGSVKGFKKILVLYTNYGRQKKPEKTNWVMHQYHLGSNEEEKDGELVVSKVFYQTQPRQCANKDPYDERLLMTSQINSVNDISIHALPKNNNNNNAGFVDYYNPGFMNMNYEQMNETTSPQLIPNMVVQGDSSSFIRLPRLDRK

**>AiNAC44**

MPPSPLGLISFSTWRQIPSTIKSPSLLTSPKISILSSSLFLDFSLLHQTSLSLLLFLLFLTKPITNILLHQIIRLSKMGSPESNLPPGFRFHPTDEELILHYLRKKVASIPLPVSIIAEVDIYKLDPWELPAKAAFGEKEWYFFSPRDRKYPNGARPNRAAASGYWKATGTDKTIVVSPAATVTRRVGQESSVGVKKALVFYKGRPPKGVKTNWIMHEYRLLDDWVLCRIYKKSKFSVSSPEESPSSEVQAAEENGLFKNTILKSPIPTLSPSPPPPLPPQPLLSQKSVSFSNLLDAMDYSMLSTILSENNNNSTLDQEQYSQINTNQLNHSSNMENTSNSNMMLMRSKRQIDEETTTVLHPSKKFHHQLMGSSSCSFPNNINNTNTAQYENPQWNYLVKQSFLNQHLLLAPHLRFQG

**>AiNAC45**

MEEPVVVNKGEEPLDLPPGFRFHPTDEEIITYYLTEKVMNSSFSATAIGEADLNKSEPWDLPKKAKMGEKEWYFFCQKDRKYPTGMRTNRATDSGYWKATGKDKEIFKGKGNLVGMKKTLVFYRGRAPKGEKTNWVMHEFRLEGKFANYNLPKAAKDEWVVSRVFHKNTDVKKTTTPSSSSSIIPGLLRINSIGDDLLDCSTLPPLMDPHPTPLDTTKSDGYYFPSFSSSHQILNIKPEEHNTSHQIPITNYQIPNFNTTLSSSSSHQIRLQNHLNLFSSSSSNNNYHNSSWPTYYDEVHHHHQDDILLRAIASKNYSNGGGGAGECKVEQFSSGNQSVVSVSQETGLSNDRTTNDTSSVVSKQQHNNRTLYEDLEGPSSSVAPLSDLECLWDTY

**>AiNAC46**

MEDPPTGFRFYPTEEELVAFYLNTQLQLQGHANNINRVIPVVDINGVEPWTLPSLAGELCREEKEQWFFFVPRQEREARGGRINRTTASGKAPTGRKTKWKMHEYRAIVQAPNQSPTAIPQLRHEFSLCRVYVISGSFRAFDRRPREVVVPRVLHHGSSTTSAQQHQGESSARVQANNNNNGSSSSETSLSSGGPDLPPDTGGGGSSSNWNSSEVQVQAQVQEPLWEWEQLDWL

**>AiNAC47**

SFSRNQEQEEEGGIMQGGLELPPGFRFHPSDEELVNHYLCKKCAKQSIAAPIIKEIDLYKFDPWQLPEMALYGEKEWYFFSPRDRKYPNGSRPNRAAGSGYWKATGADKPIGKPKALGIKKALVFYAGKAPKGVKTNWIMHEYRLANVDRSAANKLNNNNLRLDDWVLCRIYNKKGKIEKFNSATTGLEQKLPKFSPGEILHYDHDHEHETKPKIIHNFSNNEHQLYMDTSDSVPRLHTDSSCSDHAVSPDATCDKEVESNPKWSNELDMQLFDTFDFQLNNYDNSLPMNDDDLFGNQFQMNQLMSFQDTFLFPQKPF

**>AiNAC48**

MTQCNSNDYPENNHNTIVERNKDILISRTCPSCGHHIKCQQDHQGAGIHDLPGLPAGVKFDPTDQEILEHLEAKVRSDIHKLHPLIDEFIPTLEGENGICCTHPEKLPGVGKDGLIRHFFHRPSKAYTTGTRKRRKVHTDADGSETRWHKTGKTRPVYISGKLKGYKKILVLYTNYKKQRKPEKTNWVMHQYHLGNNEEEKEGELVVSKVFYQTQPRQCAGSLLIKDSSSFPAKLKDQSGVHHHEVTNNHKNNGFVEYYNASFISFAQGEQQHRSNNPTLISHFPAHDGAPFIP

**>AiNAC49**

MGSSNNGGVPPGFRFHPTDEELLHYYLKKKVSFQKFDMDVIREVDLNKMEPWDLQERCRIGSTPQNEWYFFSHKDRKYPTGSRTNRATNAGFWKATGRDKCIRNTYKKIGMRKTLVFYKGRAPHGQKTDWIMHEYRLEDSNDPQANANEDGWVVCRVFKKKNLFKIGNEGGGGSTHTSSDQQLNNSTATNARSFMQRENHYLLQNPRNGNPSSSSSGFDELDKPELGLHHYPHMQTPHYSLFHHSQPLLHPQAHKPIVYDYSYAPALPSDPPVIAKQLMTNPRDCDSGGSEGLRYQQVSEPGMEVGSCEQAQEMGAARGGGEGMNEWGVLDRLVTGNLGNEDSANKGIRFEDANPHQINQLSLRGEMDFWGYGKQ

**>AiNAC50**

MVDRDSSEAHMSIAASSMFPGFRFCPTDGELISYYLRKKLDGDEDSVQIISELELCTFEPWDLPEKSFIKSNDEWFFFSRRWRRYPNGSQNKRATKSGYWKVTGNERQIESGQNVIGTKRTLVFHVGRAPKGERTEWIIHEYCINDKFQDSLVVCRLKRNTKFRANDDSNRTSRESGCGVSEGVTVQMSTCVPIQDKEVGCSSKRSNNSNSSPSITVQIKSSDRVANEDNPKASSNESSDRVANEANPKDRVANEANPKASSNESSDRVANEANPKASSNHSKVDEVDYYAEINLDDIINLDEPAL

**>AiNAC51**

MGDSNVNLPPGFRFYPTDEELVVHFLQRKAALLPCHPDVIPDLDLYSFDPWELDEGNQWYYYSRRTQNRVTANGYWNPMGIEEAVVSNSSNRRVGIKKFYVFYVGEAPHGNRTNWIMQEYRLSDSAASSSRSSTKRKSQPKTL

**>AiNAC52**

MIMVDNSTDSSSGAGEQHHHPQLPPGFRFHPTDEELVVHYLKKKASSSPLPVAIIADVDLYKFDPWELPSKAAFGDQEWYFFSPRDRKYPNGARPNRAATSGYWKATGTDKPILSSDGNKQKVGVKKALVFYGGKPPKGVKTNWIMHEYRLTDNNNNASSSNSSKPPSIPLDPLKKTSLRLDDWVLCRIYKKSNSSSSSLPIPRPAFLMDEEKDLISMESSMVPTMSMSKPRSTSTTGCYGPMALENDDNFFDGILAASTDHHTMQNGNLLPSLITFKQIKLTSSTGSPGSSSSSKRFHGDLNNGDNTSFVSLLNQLPHNTPFHPNSILGSVGDAVLRQQFQLPGLNWNLINYFALLWWGVL

**>AiNAC53**

MMAGSGQLTVPPGFRFHPTDEELLYYYLKKKVSYEAIDLDVIREVDLNKLEPWDLKDKCRIGSGPQNEWYFFSHKDKKYPTGTRTNRATTAGFWKATGRDKAIYHTNNSKRIGMRKTLVFYIGRAPHGQKTDWIMHEYRLDEDDAEVQEDGWVVCRVFKKKNQSRGFQQEIEEEEHHHLAAEHQHMRGVASQQVLDPKHHHHLQHHQGLYDNDNDNNYTNNFDGSMHLPQLFSPESSVATAAAHTSMNAMDILECSQNLLRLTTTSGCGLNLMQQQHGERFNGDWSFLDKLLASHHGSTMDHHHHHHHHSKCNNNLHHQHPAIAIGTTSSQKFPFHHLGCDNHDIMKFSK

**>AiNAC54**

MSRILGPGFRFHPTDDELVQYYLRRKVIGKLNHHDHIGVINIYDYEPWQLPDLEWYFFTVLDKKYEKGEKTKRATVNGYWKTTGKDRGIKYGDRQVGMKKTLVYHEGRAPTGKRSNWVMHEYRMVDEQLAEVGYQLSQGLFVLVYLCFWWWWAKDAFVLCRIFEKSGMGPKNGEKYGAPFREEDWVEDGDLLEPIADEPVVELSVDQSDAFLETDDLEKKLGTHVVDGSADLPPNPPNYFYGECSHYPQHQEEFVEVPKPLEGTEGRNFDVTGPYAEDTCLENHEMNHNGNSSGFIYGDVNSDEIMDSIVDPLIGAELFLETDDLLNPIEGNSSGADPYTVEGNHPRADPYAAEGNPPGPDPYTAEGNYPGTDPYAVDMLDEYLALPDDDILRYISFDDSPPSMEGENPILEQIPPLIQQNVEEEAKDVSEEKQQKVEGEATNIFKTNKHDLEANSSRGGSASDDANPIAKRFKKWLEDIPAAPAFAAELPSKKDALQLHSAPQSSNTTHVTAGMVSITNITGRGNDMNPMVAKIGGGFNHPIISAVVLIPVSGLLCGKTLFVLTYGWAFLVTFSFLFATVTCKIGTFMYSGK

**>AiNAC55**

ACFFYISTSFNSLLFLEEMEGEKLDEIMLPGFRFHPTDEELVGFYLKRKIQQMPLSIELIKQLDIYKYDPWDLPKVAGTGEKEWYFYCPRDRKYRNSARPNRVTGAGFWKATGTDRPIYSSEGSKCIGLKKSLVFYKGRAAKGVKTDWMMHEFRLPSLADSSSDKTTIPPNDSWAICRIFKKTNATAQRALSHSWVSTLPETPTTTTNDTDHIFHFCSSNMPTMMAKKTSFMTQFCTNYTSDTQIQDVASSYKPPFININPLLYKHFDHHHHQLPPIISNGDLISNDCLIPSSTTTPLETSSNSAKPTMDFSSLLLNMSSSVLGDFAGKTSSSSSSQEGTAATATTITSSFGGGMQEHYPTIPLLRQMHQGNNNNNIGINNNNVSAGGEEQELEKVGSIVGFPFMNIGDAWKSNMLWDTSCPL

**>AiNAC56**

MAETRVLPVGYRFRPTEEELLIHYLNNKHLGNDAEIKNTISQVDLCNFDPWDLPEQSKVKSDDQEWFFFNELKYMKNKRCNRKTNMGYWKITGKERIIKRTGTDSVIGTKRTLVFYKRPHNVKTNWVLHEYHALHQKVGSCQSNIVLSRVTMNAEKREKKLKTKASNIIEEEVKCEDEPCSEITGCVTQATTEDAILPDNACVSSEWQQPQAMDYEILSSGQRSSVAYSGNENNAALLPMEATWRQDAGMNTECFWNSLFSSIDADPHAEFLNSVLAGDDQLYVDSGHH

**>AiNAC57**

MESTDSSTGSQQPNLPPGFRFHPTDEELVVHYLKKKAASAPLPVAIIAEVDLYKFDPWELPAKATFGEQEWYFFSPRDRKYPNGARPNRAATSGYWKATGTDKPVLTSGGTQKVGVKKALVFYGGKPPRGIKTNWIMHEYRLADNKPNNRPPGCDLGNKKNSLRLDDWVLCRIYKKNNTHRSPMEHEREDSMDDMIGGIPPSINVGQMNARFHLSKMSTSFSNGLLENDHHHHQNLLEGMMLGGGNNNNNNVVPPNMLGLGSASNTINNNSNKAELSFVPTMTTSSNTKRTLSSLYWNEDDVAASNKRFNLESGDHNHGENNAIVVVVMVIP

**>AiNAC58**

MSPVGLPPGFRFHPTDEELVNYYLKRKINGQEIELDIIPEMNDYISKSRTFCLFLEKSFLPSRDPEWYFFGPRDRKYPNGFRTNRATRAGYWKSTGKDRRVSSQSRPIGMKKTLVYYRGRAPQGIRTDWVMHEYRLDDKDSEDTTGLQDTYALCRVFKKNGICTDVEEQVGHCSNMSSLIESSQTIINNNNNNSNNNNEYCETMSPDIAGVSSSCLEEEDKDDSWMQFITEDAWYSSNAPNMVGGEEVSHVTFTS

**>AiNAC59**

MEQEEEPQQNEPPHSHSQSRCVTLPPGCRFHPSEELLLRYYLTNKNGTGNWNGNGGLGFDGSDLIRELDFYDYDPFELPDFACFAYGYGGRRRHWYCFTSVRVSRGERWKRKRKVKSGFWLRRGRVSNVNGVGENVVLGTRTRFVFYMGDSAKNGARTDWVLYEYALVDHVMASYVLCRVFSKPRYKNSASDIGLSCCAEESVSAVRHIGIQHDEHVKLDAVEAKVCDDISIDHNNEICAGGNSDNDNQVKNAHDIDALRCLAGPQGSQQERLPLLPSSSTMFIEAISSPQQLLSITEEDFIELNDLT

**>AiNAC60**

MASSLPQSLSSFPTTIRETVPLLWKGAADQVAIGKKDYMEKGKLSPGFHFNPTDVELLKYFLKRKVTGKKLPNVIAEINSHLKSGDLEWYFFCARGKKYGIGSKTNRAIKNGYWKATGMDKAIVQHDKQTVGMMKILVFHTGKAPHGTPTDWVMHEYRLQDKDLTDKGIAQDSYVICKVFRKEGHGPRTRSGAQYRKPFNEEDWDDDDHHVVEEGGTPSTALVAPVSIQSTTFDGSSYMKPTSVSCESGPVATSPVPSTPSSDASIHTVNNVNNSTVTDLSKDEKTVPEENIAAGDLLSKFFEGLEDLESEYTPNGVGLDDFSPHGINYDDLRHLDLIDLNFL

**>AiNAC61**

MIDMGSSSVIDGEVTLPGFRFHPTEEELLDFYLKNMVVGKKLRFDVIGFLNIYHHDPWDLPGLAKVGEREWYFFVPRDRKHGTGGRPNRTTEKGFWKATGSDRKIVTLSDPKRIIGLRKTLVFYEGRAPRGSKTDWDIVLCKIYRKATSLKVLEQRAAIEEEMKQMVGSPESTPSSTDTMSYEEQQQNQNQNLQLLPTQHVVTKKEVEAEVEEEKMDKIPELQMPMMITDWTQDTFWAQLNSPWLQNYTYSNILNF

**>AiNAC62**

MKKTLVFYTGRAPKGKRTNWVMHEYRPTLQELDGTNPGQNPYVLCRLFKKQDESLEGSNGEEMERTTSTNLTANYSPEEIQSDPAVKSVSSSQATEDDKKLAVIPLTPEEAISNVITPVGCQNDGCDAYDAQNQIAAGDPSKEEDLQVNMDIFYDPSELFDDKLFSPLHKHIPEELFHQSNNEANGHFGLQHQCGTNEISISDFFDSVINWDEISGDNSSGQTPNSAWFDVQHNESWGNSNVDMVHARPLQVGGADYPGDATEGKLPLLKTREFNPNTSYDNALSNNMGLFHNHSQMAFSSDVNMLQGYHATNNYEQPTNFNMAMANSDNTGIRIRSRPPGYEGPNVNSNMQPQGTAPRRIRLARSLAPQHMSNEAAKDSSYESKDQNSQLTTAREMETSKDLAAGESVTVTSDVEEQETSPVENKEFEDFNTVQQSTSSASSNLSTCSSDSEVSYEAEKESGWTSEDHSPKPAAAGASKASEDQVPSECVNDITDDVDEPRIPNAYTLEVSKEESFSDSQSKDSLLRRKVCYPSKSSSNLAKWYSVIAVSATLVVLLAFLVNTWGYGYYLKV

**>AiNAC63**

MNTFSHVPPGFRFHPTDEELVDYYLRKKVASKKIDLDVIKDVDLYKIEPWDLQELCKIGSDEENDWYFFSHKDKKYPTGTRTNRATKAGFWKATGRDKAIYSKQHCLIGMRKTLVFYKGRAPNGHKSDWIMHEYRLETNENGTAPEEGWVVCRVFKKKMATVRKIGDYDSPCSWYDEQVPFMQDLESSSPIKPPIINNNHYASSYNYHQLQLPCKPEFHQLMQYNNMNMPRHDDAADNNNNFLQLPQLESPNGGISPFLQQQDHHHQLLQQQNSNSNYHLDQVTDWRVLDKFVASQLMSHGHDDNDDDGHNNNNNVSKEVINSYSDASILHVAQQIAMLANGSSSSSSSRRPQISHQEYAASTSTSSSQIDLWKSSS

**>AiNAC64**

MEEGGGDQHASNSSYTFPPGFRFHPSDEELIVHYLQNRISSRPLPASIIAEIDLYKYNPWDLPKKALFGEEEWYFFSPRDRKYPNGLRPNRAAGSGYWKATGTDKPILTSYGSKRIGVKKALVFYLGRPPKGTKTDWIMNEYRLVDTITSPSRLKGSMRLDDWVLCRVRHKGYSSKNSCENQDNPCEPNMLPNLPRCDEGYPATNMNFHADMITDYQYKDYQILASILVGGHVPTTESMSSLNLKDGKGNDPITSVHEDGFHREDSSTTVSPLDCYFNSLKRKSNEDNQYENLISFNRKLNMETAMDDESSIINGGLNFYNQNQSQDDIIFNKRAAEPSINFQELKQSAFIGRYPQCSSD

**>AiNAC65**

MDMESCVPPGFRFHPTEEELVGYYLKRKINSLKIDLDVIVEIDLYKMEPWDIQDRCKLGYEEQNEWYFFSHKDKKYPTGTRTNRATAAGFWKATGRDKAVMSKNRIIGMRKTLVFYKGRAPNGRKTDWIMHEYRHQTSEHGPPQARWVVCRAFRKPSPSHQRQLGYDPWCSNHHHQPHYFRDQSSYDHHHQQEQQEFVISNNHQQLIELPQLDSPTSASLSAPPTTSFAVKESSSINNNNEEYCSDERNNNNNIDWKSLDNLFADTSNYFSNPNMSQFMTINHHLGCFPGS

**>AiNAC66**

MEKLNFVKKNGVSRMPPGFRFQPTDEELVFQYLKCKVFSFPLPASMIPDINLSNYDPWDLPGNCDEHQEMYFFSSKEPKYRNGSRMNRTTTSGYWKATGSDKRIISSSNNSDDNSILGIRKTLVFYQGKSPNGTRTHWVLHEYRLVSTTLHANNNACDIGDWVLCRLSVKKRSVGSGSIIISKKARSSPSSSSSSSTSSNNVMEVSSSYAS

**>AiNAC67**

MEGSSKSCELLPPGFRFHPTDEELIVYYLCNQATSKPCPASIIPEVDIYKFDPWELPGKAEFGEKEWYFFSPRERKYPNGVRPNRATVSGYWKATGTDKAIYSKCKHVGVKKALVFYRGRPPKGIKTDWIMHEYRLLQQSNHNSRITGSMRLDDCVLCRIYKKKHAAKALDQGQEYPTTVQINLNASTNNDDEKELMMMKNLPRTCSLTYLLDMNYFGPISQLLSDGSYNNSSTFEIFQHSNSVDNIGIVDPLVKTQMVEMDDSYYAQDSGKSQVMKQGNDLRGYY

**>AiNAC68**

MLSGFRLLFFILRVVVHIWLNYIIEQSKVKSDDQEWFFFNELKHIKNKWCNRKTNAGYWEITGKERIVKGTGTDNVIGTKRTLVFYKRPHSVKTNWVLHEYHALDQKSNIVLSRVIKNVEKREKKVKRKASNIIEEEVTCEPCSEMTGYVTEATTEDAIIPDACVSSEPQPPQDIDYEILSPGQQSSVAHSGNGSNNAPLLPFEGMWKQDAEMNTDIDADLDAEFLNSVLAGDD

**>AiNAC69**

MAIAAPNSSPTMSLSHSHSHEDGGTTTAASTTNDNLNGNGKQEDDDHEHDMVMPGFRFHPTEEELVEFYLRRKVEGKRFNVELITFLDLYRYDPWELPALAAIGEKEWYFYVPRDRKYRNGDRPNRVTTSGYWKATGADRMIRTENFRSIGLKKTLVFYSGKAPKGIRTSWIMNEYRLPQHETERYQKAEISLCRVYKRAGVEDHPSLPRCLPTRAPSSRTVDHQKNKQQPHNDQLNMGFGGNTADGASDNRDHDVTTALALSKHNTNNTNNAYRAPSMGLPPLLLPLDDEAAFVLMQQQHHAGPSSGTTMMDDLNRLVSYQHQYYNSSSSSSNNNNPNHHHHLLMHQQQQQQQQQQQQQNPPAIMSLNNTPSPLATAFSDRLWEWNPLPEANQRQYSNMSFK

**>AiNAC70**

MDAALDLPPGFRFHPTDEEIISYYLTHKVLNTSFTATAIGEVDLNKCEPWDLPKKAKMGEKDWYFFWQRDRKYPTGIRTNRATESGYWKATGKDKEIYKGRNLVGMKKTLVFYRGRAPNGHKTNWVMHEFRLEGLFATYNLPIPAKEEWVVSRVFHKNTTEKLNPTIPSGLFRIMKNMNSIGDDDLVDFSSLPPLMDPSNNYDDEHTTTTNNNYVNSMFASSSDYNITIQQNKKDMMGIRNNNNNIRALLMYDGPSSSSSEVVAPPLSDLELGLWDL

**>AiNAC71**

MEEEDDVPLPGFRFHPTDEELVSFYLKRKLDKKPISIELIKQIDIYKYDPWDLPKASGSGGEKEGYFFCKRGRKYRNSIRPNRVTSSGFWKATGIDKPVYSHGGEGTDCIGLKKTLVYYRGSAGKGTKTDWMMHEFRLPSATTENNTSLLANNKNNNNINNADVAQEAEIWTLCRIFKRNVSQRKHTVDLRSHLVTANSNKHKTTRTHVVQSNNNNINQHQESYINFGATIIGHHHYHHHRHQNEQKPVTNYTACNNNTDQIQRNNSNHHHHHQLNYHPSSAVATTVPQQQQQQYHHHHQLMTAPASNMWINPSAMNDLFAFDDDWDELGSVLKFTVDTPSL

**>AiNAC72**

MGGASLPPGFRFHPTDEELIGYYLKRKVEELEIELEVIPVIDLYKFDPWELPEKSFLPKRDLEWFFFCPRDRKYPNGSRTNRATKAGYWKATGKDKKVVCQSSPSTSIMKATGYRKTLVFYRGRAPLGDRTDWVMHEYRLCDDLGQDSPSFQGAYALCRVIKKNDKASDYKGKRGVSSSKNENETSMRLSSSKEHLSISADVSSQASQLCSESRYSSPIASPCAYNVAATAVFEPPSVDTNPSTFLVSPDMILDSSKDFAQTQDAISEFLPHHELLSTMTPWQSLEHTEISSSSSYSNFNGEIEFSDELGLIGRMSRYSGQVDMLDFYGNEEVLYEYEGYDQINSIRDPRQF

**>AiNAC73**

MQKEKEAITANKEGTNKKDIGIMECCHGKEETLPPGFRFHPTDEELITCYLINKISDSNFSGRAITDVDLNKCEPWELPATNTGYWKTTGKDKEILNSVTSELVGMKKTLVFYKGRAPRGEKSNWVMHEYRIHSKSTFRTTKDEWVVCRVFQKSAGAKKYPSSNHASRAMNPFNLEIGHHNIVPPPPMMQLGDPAAAHFLYGRNYMNTAELAEVARVLRVGTGSTSTNLPGMQPQINYPVAASSPGVGFTISGLNLNLGGGGGGTVVATTQPVLRPMQPTPPSQTLGMVPHHQVHHDVSSNMISGAENVGYVNEISNTNGGHGNRFMGMDHCMDLDNYWPSY

**>AiNAC74**

MAPMSLPPGFRFHPTDEELVAYYLERKITGRSIELDIIAEVDLYKCEPWDLPDKSFLPSKDMEWYFYSPRDRKYPNGSRTNRATRGGYWKATGKDRAVQSQKKAVGMKKTLVYYKGRAPHGIRTNWVMHEYRLIESLPGTPHSSFKDSFSLCRIFKKTIQVQDKSKEEKEHQALLEEDHSRTCTPTETGIADDFHAQFACDEANSSAANSYSMGIAYPSNDIEMSMYGSMHNYQFPQTPLVMEDFPQIDFAETKLLKPEVTEDCMFYDRYGRDCMNGTLEEIISYSFDTEETIATCVGQNNHTQKETFFLAREKQQLHI

**>AiNAC75**

MGGASLPPGFRFHPTDEELLGYYLKRKVEGLEIELEVIPVIDLCKFDPWELPEKSLLANRDMEWFFFCPRDRKYPNGSRTNRATKAGYWKATGKDKKVVCQFDTPSTVTGYRKTLVFYRGRAPLGDRTDWLMHEYRLADDLGLSSTCFQGGYALCRVIKKNEKVNNENDASMRFSNEPFAISADASSSQPSYLNNESVYSSPNASPHNVDSNQASINTSSSSEFWVSPDLILDSSKDYPQLQNTFTRCDIPSSTMTPWLSLDQPEISSSSSYSNFNG

**>AiNAC76**

MNTFCHVPPGFRFHPTDEELVDYYLRKKVNSTRIDLDVIKDVDLYKIEPWDLQELCRLGTEEQNEWYFFSHKDKKYPTGTRTNRATAAGFWKATGRDKAIYSKHDLIGMRKTLVFYKGRAPNGLKSDWIMHEYRLETDQTAAATPQEEGWVVCRVFKKRVTSIMRKMSDHDSPSCTWYDDSSFMHQQPDHHFDNSCSSSSKHQLIPNNNCDVFYQQHNNNNLPLHHLPLLHQNNNNPIMAPPFAAINNNETTAFQEQGKSLIHHQALLYGNLNEEQASSSAAAAAADWRLVDKFVSSQLREDHHVSKQELMMPENTNNNNNDNGASTSNSSCPIMDVWK

**>AiNAC77**

MDSCQPQLPPGFRFHPTDEELIVHYLKRKASSAPLPVAIIADVDLYKFDPWELPSKATFGEQEWYFFSPRDRKYPNGARPNRAATSGYWKATGTDKPIIASDGQHRLGVKKALVFYGGKPPKGVKTNWIMHEYRLTTTHNNNSISSSKSFPSLPSHLPSANNKKNSLRVSIIHFLPTNIFDLDISIKNLDDWVLCRIYEKSNRGNFARTALMEHHDHDDDDDDNKDQLSAETTSMIENMSTMSSQNSKPTQHYGPLLVQNDDNFFDGILAADHHNQQHNLPMKRTLVNMNNSQFWNETNKRFHCDLNNNTNIVANNDEDNTSFVSLLSHNQIPHHPTNNASLLDPTVADGVFRQHFQLQAINWNL

**>AiNAC78**

MAELSAAATFTPSDEELIHFLSDKVKGQSMDEDAAINIHECEYLYGRNKNPWDIWRDFAGDVDAGRTALFFFSPTKKHHSTASRPIGAGVWEAEAETIDGESIVGKGKNRRIGTKKCFVFDKSGTSYDGAWILHEYTLHGSSLHTNTSVDHSYVICKLIKNVEGEAHPVEVQFGDKRKRHGQSATTSGVQIDVNAPHSYRNTKEQEVQFIPNELGRRMLLEMFEDDEDGLTLSDGLTHQKPHAAARGNKKRWKRHLIIA

**>AiNAC79**

AWVSRQHPYYKYLSFSICTINTTHLHHYSNLTHSLLLFSLPIFLMGDNNVNLPPGFRFYPTDEELVVHFLHRKAALLPCHPDVIPDLDLYPYDPWELDGRALAEGKQWYYYSRRTQSRVTENGYWKATGMEEPVMTSSTNKRVGIKKYFVFHLGESPSAIKTNWIMQEYCLSDYSASSSRSSKRKSDYSKWVICRVYERNGDDDDGTELSCLDEVFLSLDDLDEISLPN
