# Supplementary material for: Comprehensive genomic characterization of NAC transcription factor family and their response to salt and drought stress in peanut
Source: BMC Plant Biol. 2020 Oct 2;20:454. doi: 10.1186/s12870-020-02678-9 (PMC7532626; doi:10.1186/s12870-020-02678-9)
Supplement: Supplementary file 6 — Additional file 6. Arabidopsis NAC proteins. [file 12870_2020_2678_MOESM6_ESM.docx]

[>At1g52890 4](#_Toc536650256)

[>At1g01720 4](#_Toc536650257)

[>At5g08790 4](#_Toc536650258)

[>At3g15500 5](#_Toc536650259)

[>At3g15170 5](#_Toc536650260)

[>At5g53950 5](#_Toc536650261)

[>At1g76420 5](#_Toc536650262)

[>At1g56010 6](#_Toc536650263)

[>At5g04410 6](#_Toc536650264)

[>At1g52880 6](#_Toc536650265)

[>At1g69490 7](#_Toc536650266)

[>At3g10490 7](#_Toc536650267)

[>At3g10500 7](#_Toc536650268)

[>At1g34180 7](#_Toc536650269)

[>At3g10480 8](#_Toc536650270)

[>At3g04420 8](#_Toc536650271)

[>At1g77450 8](#_Toc536650272)

[>At3g29035 9](#_Toc536650273)

[>At3g17730 9](#_Toc536650274)

[>At1g02230 9](#_Toc536650275)

[>At3g04410 9](#_Toc536650276)

[>At1g65910 10](#_Toc536650277)

[>At5g39820 10](#_Toc536650278)

[>At1g33060 10](#_Toc536650279)

[>At3g44350 11](#_Toc536650280)

[>At1g01010 11](#_Toc536650281)

[>At3g49530 11](#_Toc536650282)

[>At5g04400 11](#_Toc536650283)

[>At5g24590 12](#_Toc536650284)

[>At5g22380 12](#_Toc536650285)

[>At5g39610 12](#_Toc536650286)

[>At4g10350 13](#_Toc536650287)

[>At4g28530 13](#_Toc536650288)

[>At4g35580 13](#_Toc536650289)

[>At4g17980 13](#_Toc536650290)

[>At3g55210 14](#_Toc536650291)

[>At5g18270 14](#_Toc536650292)

[>At2g24430 14](#_Toc536650293)

[>At5g07680 15](#_Toc536650294)

[>At2g46770 15](#_Toc536650295)

[>At2g02450 15](#_Toc536650296)

[>At2g17040 16](#_Toc536650297)

[>At2g27300 16](#_Toc536650298)

[>At2g43000 16](#_Toc536650299)

[>At5g17260 16](#_Toc536650300)

[>At5g22290 17](#_Toc536650301)

[>At1g79580 17](#_Toc536650302)

[>At1g02220 17](#_Toc536650303)

[>At1g02250 18](#_Toc536650304)

[>At1g54330 18](#_Toc536650305)

[>At1g61110 18](#_Toc536650306)

[>At1g28470 18](#_Toc536650307)

[>At1g32870 19](#_Toc536650308)

[>At1g34190 19](#_Toc536650309)

[>At5g61430 20](#_Toc536650310)

[>At5g46590 20](#_Toc536650311)

[>At3g56530 20](#_Toc536650312)

[>At3g61910 20](#_Toc536650313)

[>At5g13180 21](#_Toc536650314)

[>At3g04060 21](#_Toc536650315)

[>At3g03200 21](#_Toc536650316)

[>At3g04070 22](#_Toc536650317)

[>At4g37130 22](#_Toc536650318)

[>At1g60280 22](#_Toc536650319)

[>At1g60300 23](#_Toc536650320)

[>At1g60340 23](#_Toc536650321)

[>At1g60350 23](#_Toc536650322)

[>At1g60380 24](#_Toc536650323)

[>At3g18400 24](#_Toc536650324)

[>At1g32770 24](#_Toc536650325)

[>At3g15510 24](#_Toc536650326)

[>At2g33480 25](#_Toc536650327)

[>At1g26870 25](#_Toc536650328)

[>At5g63790 25](#_Toc536650329)

[>At3g57150 26](#_Toc536650330)

[>At5g09330 26](#_Toc536650331)

[>At5g64060 26](#_Toc536650332)

[>At3g56570 27](#_Toc536650333)

[>At5g56620 27](#_Toc536650334)

[>At5g39690 27](#_Toc536650335)

[>At5g39540 28](#_Toc536650336)

[>At3g56560 28](#_Toc536650337)

[>At3g44290 28](#_Toc536650338)

[>At4g28500 28](#_Toc536650339)

[>At1g76420 29](#_Toc536650340)

[>At1g33280 29](#_Toc536650341)

[>At1g32510 29](#_Toc536650342)

[>At4g27410 29](#_Toc536650343)

[>At5g24590 30](#_Toc536650344)

[>At2g18060 30](#_Toc536650345)

[>At4g36160 30](#_Toc536650346)

[>At5g66300 31](#_Toc536650347)

[>At1g12260 31](#_Toc536650348)

[>At5g62380 31](#_Toc536650349)

[>At1g71930 31](#_Toc536650350)

[>At1g62700 32](#_Toc536650351)

## >At1g52890

MGIQETDPLTQLSLPPGFRFYPTDEELMVQYLCRKAAGYDFSLQLIAEIDLYKFDPWVLPNKALFGEKEWYFFSPRDRKYPNGSRPNRVAGSGYWKATGTDKIISTEGQRVGIKKALVFYIGKAPKGTKTNWIMHEYRLIEPSRRNGSTKLDDWVLCRIYKKQSSAQKQVYDNGIANAREFSNNGTSSTTSSSSHFEDVLDSFHQEIDNRNFQFSNPNRISSLRPDLTEQKTGFHGLADTSNFDWASFAGNVEHNNSVPELGMSHVVPNLEYNCGYLKTEEEVESSHGFNNSGELAQKGYGVDSFGYSGQVGGFGFM

## >At1g01720

MSELLQLPPGFRFHPTDEELVMHYLCRKCASQSIAVPIIAEIDLYKYDPWELPGLALYGEKEWYFFSPRDRKYPNGSRPNRSAGSGYWKATGADKPIGLPKPVGIKKALVFYAGKAPKGEKTNWIMHEYRLADVDRSVRKKKNSLRLDDWVLCRIYNKKGATERRGPPPPVVYGDEIMEEKPKVTEMVMPPPPQQTSEFAYFDTSDSVPKLHTTDSSCSEQVVSPEFTSEVQSEPKWKDWSAVSNDNNNTLDFGFNYIDATVDNAFGGGGSSNQMFPLQDMFMYMQKPY

## >At5g08790

MKSELNLPAGFRFHPTDEELVKFYLCRKCASEQISAPVIAEIDLYKFNPWELPEMSLYGEKEWYFFSPRDRKYPNGSRPNRAAGTGYWKATGADKPIGKPKTLGIKKALVFYAGKAPKGIKTNWIMHEYRLANVDRSASVNKKNNLRLDDWVLCRIYNKKGTMEKYFPADEKPRTTTMAEQSSSPFDTSDSTYPTLQEDDSSSSGGHGHVVSPDVLEVQSEPKWGELEDALEAFDTSMFGSSMELLQPDAFVPQFLYQSDYFTSFQDPPEQKPFLNWSFAPQG

## >At3g15500

MGLQELDPLAQLSLPPGFRFYPTDEELMVEYLCRKAAGHDFSLQLIAEIDLYKFDPWVLPSKALFGEKEWYFFSPRDRKYPNGSRPNRVAGSGYWKATGTDKVISTEGRRVGIKKALVFYIGKAPKGTKTNWIMHEYRLIEPSRRNGSTKLDDWVLCRIYKKQTSAQKQAYNNLMTSGREYSNNGSSTSSSSHQYDDVLESLHEIDNRSLGFAAGSSNALPHSHRPVLTNHKTGFQGLAREPSFDWANLIGQNSVPELGLSHNVPSIRYGDGGTQQQTEGIPRFNNNSDVSANQGFSVDPVNGFGYSGQQSSGFGFI

## >At3g15170

MDVDVFNGWGRPRFEDESLMPPGFRFHPTDEELITYYLLKKVLDSNFSCAAISQVDLNKSEPWELPEKAKMGEKEWYFFTLRDRKYPTGLRTNRATEAGYWKATGKDREIKSSKTKSLLGMKKTLVFYKGRAPKGEKSCWVMHEYRLDGKFSYHYISSSAKDEWVLCKVCLKSGVVSRETNLISSSSSSAVTGEFSSAGSAIAPIINTFATEHVSCFSNNSAAHTDASFHTFLPAPPPSLPPRQPRHVGDGVAFGQFLDLGSSGQIDFDAAAAAFFPNLPSLPPTVLPPPPSFAMYGGGSPAVSVWPFTL

## >At5g53950

MDIPYYHYDHGGDSQYLPPGFRFHPTDEELITHYLLRKVLDGCFSSRAIAEVDLNKCEPWQLPGRAKMGEKEWYFFSLRDRKYPTGLRTNRATEAGYWKATGKDREIFSSKTCALVGMKKTLVFYKGRAPKGEKSNWVMHEYRLEGKFSYHFISRSSKDEWVISRVFQKTTLASTGAVSEGGGGGGATVSVSSGTGPSKKTKVPSTISRNYQEQPSSPSSVSLPPLLDPTTTLGYTDSSCSYDSRSTNTTVTASAITEHVSCFSTVPTTTTALGLDVNSFSRLPPPLGFDFDPFPRFVSRNVSTQSNFRSFQENFNQFPYFGSSSASTMTSAVNLPSFQGGGGVSGMNYWLPATAEENESKVGVLHAGLDCIWNY

## >At1g76420

MMLAVEDVLSELAGEERNERGLPPGFRFHPTDEELITFYLASKIFHGGLSGIHISEVDLNRCEPWELPEMAKMGEREWYFYSLRDRKYPTGLRTNRATTAGYWKATGKDKEVFSGGGGQLVGMKKTLVFYKGRAPRGLKTKWVMHEYRLENDHSHRHTCKEEWVICRVFNKTGDRKNVGLIHNQISYLHNHSLSTTHHHHHEALPLLIEPSNKTLTNFPSLLYDDPHQNYNNNNFLHGSSGHNIDELKALINPVVSQLNGIIFPSGNNNNDEDDFDFNLGVKTEQSSNGNEIDVRDYLENPLFQEASYGLLGFSSSPGPLHMLLDSPCPLGFQL

## >At1g56010

METEEEMKESSISMVEAKLPPGFRFHPKDDELVCDYLMRRSLHNNHRPPLVLIQVDLNKCEPWDIPKMACVGGKDWYFYSQRDRKYATGLRTNRATATGYWKATGKDRTILRKGKLVGMRKTLVFYQGRAPRGRKTDWVMHEFRLQGSHHPPNHSLSSPKEDWVLCRVFHKNTEGVICRDNMGSCFDETASASLPPLMDPYINFDQEPSSYLSDDHHYIINEHVPCFSNLSQNQTLNSNLTNSVSELKIPCKNPNPLFTGGSASATLTGLDSFCSSDQMVLRALLSQLTKIDGSLGPKESQSYGEGSSESLLTDIGIPSTVWNC

## >At5g04410

MGRGSVTSLAPGFRFHPTDEELVRYYLKRKVCNKPFKFDAISVTDIYKSEPWDLPDKSKLKSRDLEWYFFSMLDKKYSNGSKTNRATEKGYWKTTGKDREIRNGSRVVGMKKTLVYHKGRAPRGERTNWVMHEYRLSDEDLKKAGVPQEAYVLCRIFQKSGTGPKNGEQYGAPYLEEEWEEDGMTYVPAQDAFSEGLALNDDVYVDIDDIDEKPENLVVYDAVPILPNYCHGESSNNVESGNYSDSGNYIQPGNNVVDSGGYFEQPIETFEEDRKPIIREGSIQPCSLFPEEQIGCGVQDENVVNLESSNNNVFVADTCYSDIPIDHNYLPDEPFMDPNNNLPLNDGLYLETNDLSCAQQDDFNFEDYLSFFDDEGLTFDDSLLMGPEDFLPNQEALDQKPAPKELEKEVAGGKEAVEEKESGEGSSSKQDTDFKDFDSAPKYPFLKKTSHMLGAIPTPSSFASQFQTKDAMRLHAAQSSGSVHVTAGMMRISNMTLAADSGMGWSYDKNGNLNVVLSFGVVQQDDAMTASGSKTGITATRAMLVFMCLWVLLLSVSFKIVTMVSAR

## >At1g52880

MESTDSSGGPPPPQPNLPPGFRFHPTDEELVIHYLKRKADSVPLPVAIIADVDLYKFDPWELPAKASFGEQEWYFFSPRDRKYPNGARPNRAATSGYWKATGTDKPVISTGGGGSKKVGVKKALVFYSGKPPKGVKSDWIMHEYRLTDNKPTHICDFGNKKNSLRLDDWVLCRIYKKNNSTASRHHHHLHHIHLDNDHHRHDMMIDDDRFRHVPPGLHFPAIFSDNNDPTAIYDGGGGGYGGGSYSMNHCFASGSKQEQLFPPVMMMTSLNQDSGIGSSSSPSKRFNGGGVGDCSTSMAATPLMQNQGGIYQLPGLNWYS

## >At1g69490

MEVTSQSTLPPGFRFHPTDEELIVYYLRNQTMSKPCPVSIIPEVDIYKFDPWQLPEKTEFGENEWYFFSPRERKYPNGVRPNRAAVSGYWKATGTDKAIHSGSSNVGVKKALVFYKGRPPKGIKTDWIMHEYRLHDSRKASTKRNGSMRLDEWVLCRIYKKRGASKLLNEQEGFMDEVLMEDETKVVVNEAERRTEEEIMMMTSMKLPRTCSLAHLLEMDYMGPVSHIDNFSQFDHLHQPDSESSWFGDLQFNQDEILNHHRQAMFKF

## >At3g10490

MNRATNRGYWKATGKDREIRRDILLLGMKKTLVFHSGRAPDGLRTNWVMHEYRLVEYETEKNGNLVQDAYVLCRVFHKNNIGPPSGNRYAPFMEEEWADDEGALIPGIDVKLRLEPPPVANGNDQMDQEIQSASKSLININEPPRETAPLDIESDQQNHHENDLKPEEHNNNNNYDENEETLKREQMEEEERPPRPVCVLNKEAPLPLLQYKRRRQSESNNNSSRNTQDHCSSTTTTVDNTTTLISSSAAATNTAISALLEFSLMGISDKKEKPQQPLRPHKEPLPPQTPLASPEEKVNDLQKEIHQMSVERETFKLEMMSAEAMISILQSRIDALRQENEELKKNNANGQ

## >At3g10500

MGFVLLTLLWNCGKDAFVLCRIFQKSGSGPKNGEQYGAPFVEEEWEEEDDMTFVPDQEDLGSEDHVYVHMDDIDQKSENFVVYDAIPIPLNFIHGESSNNVETNYSDSINYIQQTGNYMDSGGYFEQPAESYEKDQKPIIRDRDGSLQNEGIGCGVQDKHSETLQSSDNIFGTDTSCYNDFPVESNYLIGEAFLDPNSNLLENDGLYLETNDLSSTQQDGFDFEDYLTFFDETFDPSQLMGNEDVFFDQEELFQEVETKELEKEETSRSKHVVEEKEKDEASCSKQVDADATEFEPDYKYPLLKKASHMLGAIPAPLANASEFPTKDAAIRLHAAQSSGSVHVTAGMITISDSNMGWSYGKNENLDLILSLGLVQGNTAPEKSGNSSAWAMLIFMCFWVLLLSVSFKVSILVSSR

## >At1g34180

MVDSSRDSCFKAGKFSAPGFRFHPTDEELVVYYLKRKICCKKLRVNAIGVVDVYKVDPSELPGLSMLKTGDRQWFFFTPRNRKYPNAARSSRGTATGYWKATGKDRVIEYNSRSVGLKKTLVFYRGRAPNGERTDWVMHEYTMDEEELGRCKNAKEYYALYKLYKKSGAGPKNGEQYGAPFQEEEWVDSDSEDADSVAVPDYPVVRYENGPCVDDTKFCNPVKLQLEDIEKLLNEIPDAPGVNQRQFDEFVGVPQGNSAEVIQSTLLNNSSGEYIDPRTNGMFLPNGQLYNRDSSFQSHLNSFEATSGMAPLLDNEKEEYIEMNDLLIPELGASSTEKSTEFLNHGEFGDVNEYDQLFNDISVFQGTSTDLSCLSNFTNNTSGQRQQLLYEQFQYQTPENQLNNYMHPSTTLNQFTDNMWFKDDQAALYVQPPQSSSGAFTSQSTGT

## >At3g10480

MGRESLAVVSSPPSATAPSTAVSATSLAPGFRFHPTDEELVSYYLKRKVLGKPVRFDAIGEVDIYKHEPWDLAEKILCSYLALNCFKNFVFACATVMFACATVMFALFSKLKTRDQEWYFFSALDKKYGNGARMNRATNKGYWKATGKDREIRRDIQLLGMKKTLVFHSGRAPDGLRTNWVMHEYRLVEYETETNGSLLQDAYVLCRVFHKNNIGPPSGNRYAPFMEEEWADGGGALIPGIDVRVRVEALPQANGNNQMDQWADLLKLHNSIKFAITFCRTQLNLTALSNERCSTREIFIVFWLICKEMHSASKDLININELPRDATPMDIEPNQQNHHESAFKPQESNNHSGYEEDEDTLKREHAEEDERPPSLCILNKEAPLPLLQYKRRRQNESNNNSSRNTQDHCSSTITTVDNTTTLISSSAAAATNTAISALLEFSLMGISDKKENQQKEETSPPSPIASPEEKVNDLQKEVHQMSVERETFKLEMMSAEAMISILQSRIDALRQENEELKKKNASGQAS

## >At3g04420

MENPVGLRFRPTDKEIVVDYLRPKNSDRDTSHVDRVISTVTIRSFDPWELPCQSRIKLKDESWCFFSPKENKYGRGDQQIRKTKSGYWKITGKPKPILRNRQEIGEKKVLMFYMSKELGGSKSDWVMHEYHAFSPTQMMMTYTICKVMFKGDVREISSSSASYGSEIEQSRDSLIPLLVNDSEEEAQIEDAIPIEEWETWLTDDGVDEQVNHIMNMKDDRNNHRPQKPLTGVLIDDSSDDDDDSDLLSPTTNSIENSSTCDSFGSSDQINLVSLTQEVSFILSLFFSDTNNP

## >At1g77450

MMKSGADLQFPPGFRFHPTDEELVLMYLCRKCASQPIPAPIITELDLYRYDPWDLPDMALYGEKEWYFFSPRDRKYPNGSRPNRAAGTGYWKATGADKPIGRPKPVGIKKALVFYSGKPPNGEKTNWIMHEYRLADVDRSVRKKNSLRLDDWVLCRIYNKKGVIEKRRSDIEDGLKPVTDTCPPESVARLISGSEQAVSPEFTCSNGRLSNALDFPFNYVDAIADNEIVSRLLGGNQMWSTTLDPLVVRQGTF

## >At3g29035

MDYKVSRSGEIVEGEVEDSEKIDLPPGFRFHPTDEELITHYLRPKVVNSFFSAIAIGEVDLNKVEPWDLPWKAKLGEKEWYFFCVRDRKYPTGLRTNRATKAGYWKATGKDKEIFKGKSLVGMKKTLVFYKGRAPKGVKTNWVMHEYRLEGKFAIDNLSKTAKNECVISRVFHTRTDGTKEHMSVGLPPLMDSSPYLKSRGQDSLAGTTLGGLLSHVTYFSDQTTDDKSLVADFKTTMFGSGSTNFLPNIGSLLDFDPLFLQNNSSVLKMLLDNEETQFKKNLHNSGSSESELTASSWQGHNSYGSTGPVNLDCVWKF

## >At3g17730

MAPVGLPPGFRFHPTDEELVNYYLKRKINGQEIELDIIPEVDLYKCEPWDLAEKSFLPSRDPEWYFFGPRDRKYPNGFRTNRATRGGYWKSTGKDRRVTSQSRAIGMKKTLVYYKGRAPQGIRTDWVMHEYRLDDKDCDDPSSLQDSYALCRVFKKNGICSELESERQLQTGQCSFTTASMEEINSNNNNNYNNDYETMSPEVGVSSACVEEVVDDKDDSWMQFITDDAWDTSSNGAAMGHGQGVY

## >At1g02230

MMNPVGFRFRPNDEEIVDHYLRPKNLDSDTSHVDEVISTVDICSFEPWDLPSKSMIKSRDGVWYFFSVKEMKYNRGDQQRRRTNSGFWKKTGKTMTVMRKRGNREKIGEKRVLVFKNRDGSKTDWVMHEYHATSLFPNQMMTYTVCKVEFKGEETEISSSSTGSEIEQIHSLIPLVNSSGGSEGSSFHSQELQNSSQSGVFANVQGESQIDDATTPIEEEWKTWLNNDGDEQRNIMFMQDHRSDYTPLKSLTGVFSDDSSDDNDSDLISPKTNSIGTSSTCASFASSNHQIDQTQHSPDSTVQLVSLTQEVSQGPGQVTVIREHKLGEESVKKKRASFVYRMIHRLVKKIHQCYSISRT

## >At3g04410

MVNPVGFRFRPTKEEIVDHYLRPTNFDGDTSHVDRNIMFMQDNRNDYRPPNSLTGVFSDCSSDDNDSDLLSPKTVS

## >At1g65910

MAPVSMPPGFRFHPTDEELVIYYLKRKINGRTIELEIIPEIDLYKCEPWDLPGKSLLPSKDLEWFFFSPRDRKYPNGSRTNRATKAGYWKATGKDRKVTSHSRMVGTKKTLVYYRGRAPHGSRTDWVMHEYRLEEQECDSKSGIQDAYALCRVFKKSALANKIEEQHHGTKKNKGTTNSEQSTSSTCLYSDGMYENLENSGYPVSPETGGLTQLGNNSSSDMETIENKWSQFMSHDTSFNFPPQSQYGTISYPPSKVDIALECARLQNRMLPPVPPLYVEGLTHNEYFGNNVANDTDEMLSKIIALAQASHEPRNSLDSWDGGSASGNFHGDFNYSGEKVSCLEANVEAVDMQEHHVNFKEERLVENLRWVGVSSKELEKSFVEEHSTVIPIEDIWRYHNDNQEQEHHDQDGMDVNNNNGDVDDAFTLEFSENEHNENLLDKNDHETTSSSCFEVVKKVEVSHGLFVTTRQVTNTFFQQIVPSQTVIVYINPTDGNECCHSMTSKEEVHVRKKINPRINGVSSTVLGQWRKFAHVIGFIPMLLLMRCVHRGNSNKNRGSEGYSRQPTRGDCNNRGTILMMENAVVRRKIWKKKKEKNMVDEQGFRFQDSFVLKKLGLSLAIILAVSTISLI

## >At5g39820

MVLVMDDEESNNVERYDDVVLPGFRFHPTDEELVSFYLKRKVLHKSLPFDLIKKVDIYKYDPWDLPKLAAMGEKEWYFYCPRDRKYRNSTRPNRVTGGGFWKATGTDRPIYSLDSTRCIGLKKSLVFYRGRAAKGVKTDWMMHEFRLPSLSDSHHSSYPNYNNKKQHLNNNNNSKELPSNDAWAICRIFKKTNAVSSQRSIPQSWVYPTIPDNNQQSHNNTATLLASSDVLSHISTRQNFIPSPVNEPASFTESAASYFASQMLGVTYNTARNNGTGDALFLRNNGTGDALVLSNNENNYFNNLTGGLTHEVPNVRSMVMEETTGSEMSATSYSTNN

## >At1g33060

MNQIKNKTLPEMTTEQALLSMEALPLGFRFRPTDEELINHYLRLKINGRDLEVRVIPEIDVCKWEPWDLPGLSVIKTDDQEWFFFCPRDRKYPSGHRSNRATDIGYWKATGKDRTIKSKKMIIGMKKTLVFYRGRAPRGERTNWIMHEYRATDKELDGTGPGQNPYVLCRLFHKPSDSCDPAHCEEIEKVNFTPTTTTRCSPDDTSSEMVQETATSGVHALDRSDDTERCLSDKGNNDVKPDVSVINNTSVNHAETSRAKDRNLGKTLVEENPLLRDVPTLHGPILSEKSYYPGQSSIGFATSHMDSMYSSDFGNCDYGLHFQDGASEQDASLTDVLDEVFHNHNESSNDRKDFVLPNMMHWPGNTRLLSTEYPFLKDSVAFVDGSAEVSGSQQFVPDILASRWVSEQNVDSKEAVEILSSTGSSRTLTPLHNNVFGQYASSSYAAIDPFNYNVNQPEQSSFEQSHVDRNISPSNIFEFKARSRENQRDLDSVVDQGTAPRRIRLQIEQPLTPVTNKKERDADNYEEEDEVQSAMSKVVEEEPANLSAQGTAQRRIRLQTRLRKPLITLNNTKRNSNGREGEASHRKCEMQEKEDISSSSSWQKQKKSLVQFSSVVIIVAVIVVLVEIWKESRDAKCSFLFHQLDSFKGMFT

## >At3g44350

MGEELSVGFRFYPTEVELLTYYLRIQLGGGNATIHSLIPILDVFSVEPTQLPNLAGERCRGDAEQWIFFVPRQEREARGGRPSRTTGSGYWKATGSPGPVFSPDNRVIGVKKTMVFYTGKAPTGRKTKWKMNEYKAVETASVSTIPKVHNHFFKMKTFIK

## >At1g01010

MEDQVGFGFRPNDEELVGHYLRNKIEGNTSRDVEVAISEVNICSYDPWNLRFQSKYKSRDAMWYFFSRRENNKGNRQSRTTVSGKWKLTGESVEVKDQWGFCSEGFRGKIGHKRVLVFLDGRYPDKTKSDWVIHEFHYDLLPEHQRTYVICRLEYKGDDADILSAYAIDPTPAFVPNMTSSAGSVVNQSRQRNSGSYNTYSEYDSANHGQQFNENSNIMQQQPLQGSFNPLLEYDFANHGGQWLSDYIDLQQQVPYLAPYENESEMIWKHVIEENFEFLVDERTSMQQHYSDHRPKKPVSGVLPDDSSDTETGSMIFEDTSSSTDSVGSSDEPGHTRIDDIPSLNIIEPLHNYKAQEQPKQQSKEKVISSQKSECEWKMAEDSIKIPPSTNTVKQSWIVLENAQWNYLKNMIIGVLLFISVISWIILVG

## >At3g49530

MNQNLHVLSMDSLPVGLRFRPTDEELIRYYLRRKINGHDDDVKAIREIDICKWEPWDLPDFSVIKTKDSEWLYFCPLDRKYPSGSRQNRATVAGYWKATGKDRKIKSGKTNIIGVKRTLVFHAGRAPRGTRTNWIIHEYRATEDDLSGTNPGQSPFVICKLFKKEELVLGEEDSKSDEVEEPAVSSPTVEVTKSEVSEVIKTEDVKRHDIAESSLVISGDSHSDACDEATTAELVDFKWYPELESLDFTLFSPLHSQVQSELGSSYNTFQPGSSNFSGNNNNSFQIQTQYGTNEVDTYISDFLDSILKSPDEDPEKHKYVLQSGFDVVAPDQIAQVCQQGSAVDMSNDVSVTGIQIKSRQAQPSGYTNDYIAQGNGPRRLRLQSNFNGINTKNPELQAIKREVRITPTF

## >At5g04400

MHIDAYVLCRVFFKQNNTGSRYAPFLEEEWDDDNGERVAIHVPDDGPLPLCVLNKEAPLPLIQYKRKRRISSSQTTQDHRSFTETIIDSTASAEPLDISERIALRALNGMLDDLEKEQEPVVVDGNKINEIQQESQLQRKKLIDLNLKEDAPSPLCVVNKETPSPLKYMIDDLEKEQEPATKRINDLVLKENDEIVVFREMQERESMKAEMEISFLEAQIDALDRKIDHPHK

## >At5g24590

MKEDMEVLSLASLPVGFRFSPTDEELVRYYLRLKINGHDNDVRVIREIDICKWEPWDLPDFSVVKTTDSEWLFFCPLDRKYPSGSRMNRATVAGYWKATGKDRKIKSGKTKIIGVKRTLVFYTGRAPKGTRTCWIMHEYRATEKDLDGTKSGQNPFVVCKLFKKQDIVNGAAEPEESKSCEVEPAVSSPTVVDEVEMSEVSPVFPKTEETNPCDVAESSLVIPSECRSGYSVPEVTTTGLDDIDWLSFMEFDSPKLFSPLHSQVQSELGSSFNGLQSESSELFKNHNEDYIQTQYGTNDADEYMSKFLDSFLDIPYEPEQIPYEPQNLSSCNKINDESKRGIKIRARRAQAPGCAEQFVMQGDASRRLRLQVNLNSHKSETDSTQLQFIKKEVKDTTTETMTKGCGNFTRSKSRTSFIFKKIAAMGCSYRGLFRVGVVAVVCVMSVCSLVA

## >At5g22380

MADEVTIGFRFYPTEEELVSFYLRNQLEGRSDDSMHRVIPVLDVFEVEPSHLPNVAGVRCRGDAEQWFFFVPRQEREARGGRPSRTTGSGYWKATGSPGPVFSKDNKMIGAKKTMVFYTGKAPTGRKTKWKMNEYHAVDETVNASTIPKLRREFSLCRVYITTGSSRAFDRRPEGVLQTERMLTSDVAVAETSFRVESSLETSISGGEHIDVSMNTEFVDGLSEPMWDWEQLTWP

## >At5g39610

MDYEASRIVEMVEDEEHIDLPPGFRFHPTDEELITHYLKPKVFNTFFSATAIGEVDLNKIEPWDLPWKAKMGEKEWYFFCVRDRKYPTGLRTNRATEAGYWKATGKDKEIFKGKSLVGMKKTLVFYKGRAPKGVKTNWVMHEYRLEGKYCIENLPQTAKNEWVICRVFQKRADGTKVPMSMLDPHINRMEPAGLPSLMDCSQRDSFTGSSSHVTCFSDQETEDKRLVHESKDGFGSLFYSDPLFLQDNYSLMKLLLDGQETQFSGKPFDGRDSSGTEELDCVWNF

## >At4g10350

MLSFYFLEERCKIGSTPQNEWYFFSHKDRKYPTGSRTNRATHAGFWKATGRDKCIRNSYKKIGMRKTLVFYKGRAPHGQKTDWIMHEYRLEDADDPQANPSEDGWVVCRVFMKKNLFKVVNEGSSSINSLDQHNHDASNNNHALQARSFMHRDSPYQLVRNHGAMTFELNKPDLALHQYPPIFHKPPSLGFDYSSGLARDSESAASEGLQYQQACEPGLDVGTCETVASHNHQQGLGEWAMMDRLVTCHMGNEDSSRGITYEDGNNNSSSVVQPVPATNQLTLRSEMDFWGYSK

## >At4g28530

MGLKDIGSKLPPGFRFHPSDEELVCHYLCNKIRAKSDHGDVDDDDDDVDEALKGSTDLVEIDLHICEPWELPDVAKLNAKEWYFFSFRDRKYATGYRTNRATVSGYWKATGKDRTVMDPRTRQLEDWVLCRVFNKGRDSSLQDNNYYNNDNQTQRLEVNDAPDLNYNNQLPPLLSSPPHNHQHEKMKIQVCDQWEQLMKQPSRTTGHPYHHHCHHQTIACGWEQMMIGSLSSPSSHGPDHESLLNLLYVDNNNSVNISGDHHQNYEKILLSSLDMTSLDHDKTCMGSSSDGGMVSDLHMECGGLSFETENILAFQ

## >At4g35580

MGAVSMESLPLGFRFRPTDEELVNHYLRLKINGRHSDVRVIPDIDVCKWEPWDLPALSVIKTDDPEWFFFCPRDRKYPNGHRSNRATDSGYWKATGKDRSIKSKKTLIGMKKTLVFYRGRAPKGERTNWIMHEYRPTLKDLDGTSPGQSPYVLCRLFHKPDDRVNGVKSDEAAFTASNKYSPDDTSSDLVQETPSSDAAVEKPSDYSGGCGYAHSNSTADGTMIEAPEENLWLSCDLEDQKAPLPCMDSIYAGDFSYDEIGFQFQDGTSEPDVSLTELLEEVFNNPDDFSCEESISRENPAVSPNGIFSSAKMLQSAAPEDAFFNDFMAFTDTDAEMAQLQYGSEGGASGWPSDTNSYYSDLVQQEQMINHNTENNLTEGRGIKIRARQPQNRQSTGLINQGIAPRRIRLQLQSNSEVKEREEVNEGHTVIPEAKEAAAKYSEKSGSLVKPQIKLRARGTIGQVKGERFADDEFVVVTGTGAEQKETRREAMEGGCNGNGGCDGWGRDGYMEDTGEFMTHETQRERERESNVV

## >At4g17980

MGSSCLPPGFRFHPTDEELIGYYLSRKIEGLEIELEVIPVIDLYKFDPWELPGKSFLPNRDLEWFFFCPRDKKYANGSRTNRATKAGYWKATGKDRKITCKSSHVIAGYRKTLVFYEGRAPLGDRTNWFMHEYRLCDIDDHSQKSPNFKGAFALCRVVKKNELKKNSKSLKNKNEQDIGSCYSSLATSPCRDEASQIQSFKPSSTTNDSSSIWISPDFILDSSKDYPQIKEVASECFPNYHFPVTTANHHVEFPVSSCYFNVDQDIDQSMQTGYWTNYENDQTGSFDYSNLF

## >At3g55210

MSPPSTIAYVLPPGFKFVPNDEEVIHCYLKPYSDGNTNVLLHVPIHLVNIYESNPQTLSEEFQKGNDKEWFIITERNKVDQGLSQTKRVGYGAKRQKRVDTNGGYWHATVAAQKINAGDGVVRNKRPLAYYVGKPSEGVKTDWLMQEYSLDHSSHNNDKDYTLCKIYLTPQATKMNKEVGEEKKKQKKGEAVVSVAPVEALEEQLPCNVEYHQPLAPLDSCQPQPHDLAYQQQQFCPGPLDSYQPQPHDMENQQPHNEKLKKEEDVEQLDLHQPDQGKGC

## >At5g18270

MAVVVEEGVVLNHGGEELVDLPPGFRFHPTDEEIITCYLKEKVLNSRFTAVAMGEADLNKCEPWDLPSKAKMGEKEFYFFCQRDRKYPTGMRTNRATESGYWKATGKDKEIFKGKGCLVGMKKTLVFYRGRAPKGEKTNWVMHEYRLEGKYSYYNLPKSARDEWVVCRVFHKNNPSTTTQPMTRIPVEDFTRMDSLENIDHLLDFSSLPPLIDPSFMSQTEQPNFKPINPPTYDISSPIQPHHFNSYQSIFNHQVFGSASGSTYNNNNEMIKMEQSLVSVSQETCLSSDVNANMTTTTEVSSGPVMKQEMGMMGMVNGSKSYEDLCDLRGDLWDF

## >At2g24430

MEQGDHQQHKKEEEALPPGFRFHPTDEELISYYLVNKIADQNFTGKAIADVDLNKSEPWELPEKAKMGGKEWYFFSLRDRKYPTGVRTNRATNTGYWKTTGKDKEIFNSTTSELVGMKKTLVFYRGRAPRGEKTCWVMHEYRLHSKSSYRTSKQDEWVVCRVFKKTEATKKYISTSSSSTSHHHNNHTRASILSTNNNNPNYSSDLLQLPPHLQPHPSLNINQSLMANAVHLAELSRVFRASTSTTMDSSHQQLMNYTHMPVSGLNLNLGGALVQPPPVVSLEDVAAVSASYNGENGFGNVEMSQCMDLDGYWPSY

## >At5g07680

MDLPPGFRFHPTDEELITHYLHKKVLDLGFSAKAIGEVDLNKAEPWELPYKAKIGEKEWYFFCVRDRKYPTGLRTNRATQAGYWKATGKDKEIFRGKSLVGMKKTLVFYRGRAPKGQKTNWVMHEYRLDGKLSAHNLPKTAKNEWVICRVFHKTAGGKKIPISTLIRIGSYGTGSSLPPLTDSSPYNDKTKTEPVYVPCFSNQAETRGTILNCFSNPSLSSIQPDFLQMIPLYQPQSLNISESSNPVLTQEQSVLQAMMENNRRQNFKTLSISQETGVSNTDNSSVFEFGRKRFDHQEVPSPSSGPVDLEPFWNY

## >At2g46770

MMSKSMSISVNGQSQVPPGFRFHPTEEELLQYYLRKKVNSIEIDLDVIRDVDLNKLEPWDIQEMCKIGTTPQNDWYFFSHKDKKYPTGTRTNRATAAGFWKATGRDKIIYSNGRRIGMRKTLVFYKGRAPHGQKSDWIMHEYRLDDNIISPEDVTVHEVVSIIGEASQDEGWVVCRIFKKKNLHKTLNSPVGGASLSGGGDTPKTTSSQIFNEDTLDQFLELMGRSCKEELNLDPFMKLPNLESPNSQAINNCHVSSPDTNHNIHVSNVVDTSFVTSWAALDRLVASQLNGPTSYSITAVNESHVGHDHLALPSVRSPYPSLNRSASYHAGLTQEYTPEMELWNTTTSSLSSSPGPFCHVSNGSG

## >At2g02450

MAIVSSTTSIIPMSNQVNNNEKGIEDNDHRGGQESHVQNEDEADDHDHDMVMPGFRFHPTEEELIEFYLRRKVEGKRFNVELITFLDLYRYDPWELPAMAAIGEKEWYFYVPRDRKYRNGDRPNRVTTSGYWKATGADRMIRSETSRPIGLKKTLVFYSGKAPKGTRTSWIMNEYRLPHHETEKYQKAEISLCRVYKRPGVEDHPSVPRSLSTRHHNHNSSTSSRLALRQQQHHSSSSNHSDNNLNNNNNINNLEKLSTEYSGDGSTTTTTTNSNSDVTIALANQNIYRPMPYDTSNNTLIVSTRNHQDDDETAIVDDLQRLVNYQISDGGNINHQYFQIAQQFHHTQQQNANANALQLVAAATTATTLMPQTQAALAMNMIPAGTIPNNALWDMWNPIVPDGNRDHYTNIPFK

## >At2g17040

MGKDIELPGFRFHPTEEELLDFYLKNMVYGKRSSVEVIGFLNIYRHDPWDLPGLSRIGEREWYFFVPRERKHGNGGRPSRTTEKGYWKATGSDRKIISLSEPKRVIGLKKTLVFYRGRAPGGSKTDWVMNEFRMPDNCSLPKDVVLCKIYRKATSLKVLEQRAEMEAKMNQTCPNSPLSSSETISFVGKEENMMTSFRAPQVIAMEEANKIQMHQENAKTEEKQREAETKEPSSSLKLPFGSLPELQLPKPGVEWDQLLSISPWLQNLTPIVNIYW

## >At2g27300

MSKEAEMSIAVSALFPGFRFSPTDVELISYYLRRKIDGDENSVAVIAEVEIYKFEPWDLPEESKLKSENEWFYFCARGRKYPHGSQSRRATQLGYWKATGKERSVKSGNQVVGTKRTLVFHIGRAPRGERTEWIMHEYCIHGAPQDALVVCRLRKNADFRASSTQKMEDGVVQDDGYVGQRGGLEKEDKSYYESEHQIPNGDIAESSNVVEDQADTDDDCYAEILNDDIIKLDEEALKASQAFRPTNPTHQETISSESSSKRSKCGIKKESTETMNCYALFRIKNVAGTDSSWRFPNPFKIKKDDSQRLMKNVLATTVFLAILFSFFWTVLIARN

## >At2g43000

MRGRKYRNSVRPNRVTGSGFWKATGIDKPVYSNLDCVGLKKSLVYYLGSAGKGTKTDWMMHEFRLPSTTKTDSPAQQAEVWTLCRIFKRVTSQRNPTILPPNRKPVITLTDTCSKTSSLDSDHTSHRTVDSMSHEPPLPQPQNPYWNQHIVGFNQPTYTGNDNNLLMSFWNGNGGDFIGDSASWDELRSVIDGNTKP

## >At5g17260

MAPVSLPPGFRFHPTDEELITYYLKRKINGQEIELEIIPEVDLYKCEPWDLPGKSLIPSKDQEWFFFSPRDRKYPNGSRTNRATKGGYWKATGKDRRVSWRDRAIGTKKTLVYYRGRAPHGIRTGWVMHEYRLDESECEPSAFGMQDAYALCRVFKKIVIEAKPRDQHQQQHQPYVHTSSNISGSSSFDVCSDLEISSNTPYNTAAHIQPRFGNANAISDHDDWSQYLSQNMPTSFSDYGSPYGPYLTQSKVNTEVQCEMFQHQMSLPPLRVENSQAQTSDFSKRLHQNSGQSGFDDFTFAASNSNQFYNSNVDDHLIHIGNLDEQSYIEEQELILPSFQSNDQDLELYGGSRTNTIDNIEIDDFFSFENQAQDNDNSNVTPNSAGFEMIGEEIIVNHKMLISTRQTTEILYYQVVPSQILKIHINPVHGNEERTMLMEEDSDDSWFQKAENVAKMKLKQISLVAKRYYKCLTIIF

## >At5g22290

MGYWKATGKERDVKSGSEVIGTKRTLVFHIGRAPKGERTDWIMHEYCVKGVSLDDAMVVCRVRRNKEYNSGTSQKAPKPNSSAEKHAKVQNGATSSGSPSDWDNLVDFYLAGESGEKLLAEMAESSENLQVDNDEDFFADILRDEIINLDEAVMTGNTPNEVPTLESASMEIRVLPLPNMIDKQMSSLLEERPSQKKKGKDATESLSSCFVGLYSIKSVNKARWDVIIGVVALIAMLFYLE

## >At1g79580

MEIGSSSTVAGGGQLSVPPGFRFHPTEEELLYYYLKKKVSYEPIDLDVIREVDLNKLEPWELKEKCRIGSGPQNEWYFFSHKDKKYPTGTRTNRATAAGFWKATGRDKSIHLNSSKKIGLRKTLVFYTGRAPHGQKTEWIMHEYRLDDSENEIQEDGWVVCRVFKKKNHFRGFHQEQEQDHHHHHQYISTNNDHDHHHHIDSNSNNHSPLILHPLDHHHHHHHIGRQIHMPLHEFANTLSHGSMHLPQLFSPDSAAAAAAAAASAQPFVSPINTTDIECSQNLLRLTSNNNYGGDWSFLDKLLTTGNMNQQQQQQVQNHQAKCFGDLSNNDNNDQADHLGNNNGGSSSSPVNQRFPFHYLGNDANLLKFPK

## >At1g02220

METPVGLRFCPTDEEIVVDYLWPKNSDRDTSHVDRFINTVPVCRLDPWELPCQSRIKLKDVAWCFFRPKENKYGRGDQQMRKTKSGFWKSTGRPKPIMRNRQQIGEKKILMFYTSKESKSDWVIHEYHGFSHNQMMMTYTLCKVMFNGGMREKSSSSPSSSGVSGIEQSRRDSLIPQLVNNSEGSSLHREDPSQFGDVLQEAPIEDAKLTEELVKWLMNDEDDAQIEDAIPIEEWETWLNDIDDAKEKSIMFMHDNRSDYRPPNSLTGVFSDDVSSDDNDSDLLTPKTNSIQTSSTCDSFGSSNHRIDQIKDLQESPTSTINLVSLTQEVSQALITSIDTAEKKKNPYDDAQGTEIGEHKLGQETIKKKRAGFFHRMIQKFVKKIHLCSSISRT

## >At1g02250

MANPVGFRFRPTDGEIVDIYLRPKNLESNTSHVDEVISTVDICSFDPWDLPSHSRMKTRDQVWYFFGRKENKYGKGDRQIRKTKSGFWKKTGVTMDIMRKTGDREKIGEKRVLVFKNHGGSKSDWAMHEYHATFSSPNQIMTYTLCKVKFKGERREFSVATGSGIKHTHSLIPPTNNSGVLSVETEGSLFHSQESQNPSQFSGFLDVDALDRDFCNILSDDFKGFFNDDDEQSKIVSMQDDRNNHTPQKPLTGVFSDHSTDGSDSDPISATTISIQTLSTCPSFGSSNPLYQITDLQESPNSIKLVSLAQEVSKTPGTGIDNDAQGTEIGEHKLGQETIKNKRAGFFHRMIQKFVKKIHLRT

## >At1g54330

MAPMSLPPGFRFHPTDEELVAYYLDRKVNGQAIELEIIPEVDLYKCEPWDLPEKSFLPGNDMEWYFYSTRDKKYPNGSRTNRATRAGYWKATGKDRTVESKKMKMGMKKTLVYYRGRAPHGLRTNWVMHEYRLTHAPSSSLKESYALCRVFKKNIQIPKRKGEEEEAEEESTSVGKEEEEEKEKKWRKCDGNYIEDESLKRASAETSSSELTQGVLLDEANSSSIFALHFSSSLLDDHDHLFSNYSHQLPYHPPLQLQDFPQLSMNEAEIMSIQQDFQCRDSMNGTLDEIFSSSATFPASL

## >At1g61110

MENMGDSSIGPGHPHLPPGFRFHPTDEELVVHYLKKKADSVPLPVSIIAEIDLYKFDPWELPSKASFGEHEWYFFSPRDRKYPNGVRPNRAATSGYWKATGTDKPIFTCNSHKVGVKKALVFYGGKPPKGIKTDWIMHEYRLTDGNLSTAAKPPDLTTTRKNSLRLDDWVLCRIYKKNSSQRPTMERVLLREDLMEGMLSKSSANSSSTSVLDNNDNNNNNNEEHFFDGMVVSSDKRSLCGQYRMGHEASGSSSFGSFLSSKRFHHTGDLNNDNYNVSFVSMLSEIPQSSGFHANGVMDTTSSLADHGVLRQAFQLPNMNWHS

## >At1g28470

MSWCDGSDDNYDLNLERVSNTDHPSVQLKDQSQSCVTSRPDSKISAETPITTCPSCGHKLHHHQDDQVGSIKDLPSLPAGVKFDPSDKEILMHLEAKVSSDKRKLHPLIDEFIPTLEGENGICYTHPEKLPGVSKDGQVRHFFHRPSKAYTTGTRKRRKVSTDEEGHETRWHKTGKTRPVLSQSGETGFKKILVLYTNYGRQKKPEKTNWVMHQYHLGSSEDEKDGEPVLSKVFYQTQPRQCGSMEPKPKNLVNLNRFSYENIQAGFGYEHGGKSEETTQVIRELVVREGDGSCSFLSFTCDASKGKESFMKNQ

## >At1g32870

MKNKIFPQNKTLLPFGTEKSKLFRVFFFVITREKKVMDLSVENGGLAPGFRFHPTDEELVVYYLKRKIRRKKLRVEAIGETDVYKFDPEELPEKALYKTRDRQWFFFSLRDRKHGSRSSRATERGYWKATGKDRVIHCDSRPVGEKKTLVFHRGRAPNGERTNWVMHEYTLHKEELKRCGGEDVKDAYVLYKIYKKSGSGPKNGEQYGAPFIEEEWAEDDDDDVDEPANQLVVSASVDNSLWGKGLNQSELDDNDIEELMSQVRDQSGPTLQQNGVSGLNSHVDTYNLENLEEDMYLEINDLMEPEPEPTSVEVMENNWNEDGSGLLNDDDFVGADSYFLDLGVTNPQLDFVSGDLKNGFAQSLQVNTSLMTYQANNNQFQQQSGKNQASNWPLRNSYTRQINNGSSWVQELNNDGLTVTRFGEAPGTGDSSEFLNPVPSGISTTNEDDPSKDESSKFASSVWTFLESIPAKPAYASENPFVKLNLVRMSTSGGRFRFTSKSTGNNVVVMDSDSAVKRNKSGGNNDKKKKKNKGFFCLSIIGALCALFWVIIGTMGGSGRPLLW

## >At1g34190

MADSSPDSCFKGGKFSAPGFRFHPTDEELVMYYLKRKICRKRLRVNVIGVVDVYKMDPEELPGQSMLKTGDRQWFYFTPRSRKYPNAARSNRGTENGYWKATGKDRVIEYNSRSVGLKKTLVFYRGRAPSGERTDWVMHEYTMDEDELGRCKNPQEYYALYKLFKKSGAGPKNGEQYGAPFQEEEWVDDDNEDVNAIAVAVPEQPVVRYEDARRVDERRLFNPVILQLEDIDELLNGIPNAPGVPQRCIPQVNSEEELQSTLVNNSAREFLPNGQQYNRPSSFDSLETAEVTSAPLVFEKEDFIEMDDLLLIPEFGASSTEKAAQFSNHGEFDDFNEFDQLFHDVSMSLDMEPIDQGTSANLSSLSDSANYTSDQKQQLLYQQFQDQTPENQLNNIMDPSTTLNQITSDIWFEDDQAILFDQQQSFSGAFASPSSGVMPDSTNPTMSVNAQGHEIQNGGGTTSQFSSALWALMDSIPSTPASACEGPLNRTFVRMSSFSRMRFNGKANGTPVSTTIAKKGIRNRGFLLLSIVGALCAIFWVLVATVRVSGRSLLLKD

## >At5g61430

MAKMGEKEWYFFCVRDRKYPTGLRTNRATEAGYWKATGKDKEIYRGKSLVGMKKTLVFYRGRAPKGQKTNWVMHEYRLEGKFSAHNLPKTAKNEWVICRVFQKSAGGKKIPISSLIRIGSLGTDFNPSLLPSLTDSSPYNDKTKTEPVYVPCFSNQTDQNQGTTLNCFSSPVLNSIQADIFHRIPLYQTQSLQVSMNLQSPVLTQEHSVLHAMIENNRRQSLKTMSVSQETGVSTDMNTDISSDFEFGKRRFDSQEDPSSSTGPVDLEPFWNY

## >At5g46590

MGSSCLPPGFRFHPTDEELIEYYLKRKVEGLEIELEVIPVIDLYSFDPWELPDKSFLPNRDMEWYFFCSRDKKYPNGFRTNRGTKAGYWKATGKDRKITSRSSSIIGYRKTLVFYKGRAPLGDRSNWIMHEYRLCDDDTSQGSQNLKNEIKTNTKIRKIPSEQTIGSGESSGLSSRVTSPSRDETMPFHSFANPVSTETDSSNIWISPEFILDSSKDYPQIQDVASQCFQQDFDFPIIGNQNMEFPASTSLDQNMDEFMQNGYWTNYGYDQTGLFGYSDFS

## >At3g56530

MAIPQRNKRKARSSPERLTQPPELPHNSDVPSSSSSSAADNFFSWSTKQFAFPPGYRFVPKDQELIFHYLKPFSQGNKCSLLNVPIHRVNIYESNPQHLSEKYEKGNDKDWFYISERTKTGKAGRSNKRVDNGGYWSATVAAQKINAGNGIVGYKTSLEYYVGKQSNSVKGDWLMQEYWFESSDDNNNEKVDHALCKIYLTPAAAKKKKAEEAENEKLKKEEDVEQLDLNQPDQLQLQQPHDIVYQPQYCLLPEHHQPQPFPDNFSELISFQQQPVMIPDDFEDFLAEFTKPHSLDGDEEFNNYGLFEGFFDTEGMIKH

## >At3g61910

MNISVNGQSQVPPGFRFHPTEEELLKYYLRKKISNIKIDLDVIPDIDLNKLEPWDIQEMCKIGTTPQNDWYFYSHKDKKYPTGTRTNRATTVGFWKATGRDKTIYTNGDRIGMRKTLVFYKGRAPHGQKSDWIMHEYRLDESVLISSCGDHDVNVETCDVIGSDEGWVVCRVFKKNNLCKNMISSSPASSVKTPSFNEETIEQLLEVMGQSCKGEIVLDPFLKLPNLECHNNTTITSYQWLIDDQVNNCHVSKVMDPSFITSWAALDRLVASQLNGPNSYSIPAVNETSQSPYHGLNRSGCNTGLTPDYYIPEIDLWNEADFARTTCHLLNGSG

## >At5g13180

MDNVKLVKNGVLRLPPGFRFHPTDEELVVQYLKRKVCSSPLPASIIPEFDVCRADPWDLPGNLEKERYFFSTREAKYPNGNRSNRATGSGYWKATGIDKRVVTSRGNQIVGLKKTLVFYKGKPPHGSRTDWIMHEYRLSSSPPSSMGPTQNWVLCRIFLKKRAGNKNDDDDGDSRNLRHNNNNNSSDQIEIITTDQTDDKTKPIFFDFMRKERTTDLNLLPSSPSSDHASSGVTTEIFSSSDEETSSCNSFR

## >At3g04060

MVEEGGVVVNQGGDQEVVDLPPGFRFHPTDEEIITHYLKEKVFNIRFTAAAIGQADLNKNEPWDLPKIAKMGEKEFYFFCQRDRKYPTGMRTNRATVSGYWKATGKDKEIFRGKGCLVGMKKTLVFYTGRAPKGEKTNWVMHEYRLDGKYSYHNLPKTARDEWVVCRVFHKNAPSTTITTTKQLSRIDSLDNIDHLLDFSSLPPLIDPGFLGQPGPSFSGARQQHDLKPVLHHPTTAPVDNTYLPTQALNFPYHSVHNSGSDFGYGAGSGNNNKGMIKLEHSLVSVSQETGLSSDVNTTATPEISSYPMMMNPAMMDGSKSACDGLDDLIFWEDLYTS

## >At3g03200

MAPVSLPPGFRFHPTDEELITYYLKRKINGLEIELEVIAEVDLYKCEPWDLPGKSLLPSKDQEWYFFSPRDRKYPNGSRTNRATKGGYWKATGKDRRVSWRDRAIGTKKTLVYYRGRAPHGIRTGWVMHEYRLDETECEPSAYGMQDAYALCRVFKKIVIEAKPRDQHRSYVHAMSNVSGNCSSSFDTCSDLEISSTTHQVQNTFQPRFGNERFNSNAISNEDWSQYYGSSYRPFPTPYKVNTEIECSMLQHNIYLPPLRVENSAFSDSDFFTSMTHNNDHGVFDDFTFAASNSNHNNSVGDQVIHVGNYDEQLITSNRHMNQTGYIKEQKIRSSLDNTDEDPGFHGNNTNDNIDIDDFLSFDIYNEDNVNQIEDNEDVNTNETLDSSGFEVVEEETRFNNQMLISTYQTTKILYHQVVPCHTLKVHVNPISHNVEERTLFIEEDKDSWLQRAEKITKTKLTLFSLMAQQYYKCLAIFF

## >At3g04070

MISKDPRSSLPPGFRFHPTDEELILHYLRKKVSSSPVPLSIIADVDIYKSDPWDLPAKAPFGEKEWYFFSPRDRKYPNGARPNRAAASGYWKATGTDKLIAVPNGEGFHENIGIKKALVFYRGKPPKGVKTNWIMHEYRLADSLSPKRINSSRSGGSELDDWVLCRIYKKSHASLSSPDVALVTSNQEHEENDNEPFVDRGTFLPNLQNDQPLKRQKSSCSFSNLLDATDLTFLANFLNETPENRSESDFSFMIGNFSNPDIYGNHYLDQKLPQLSSPTSETSGIGSKRERVDFAEETINASKKMMNTYSYNNSIDQMDHSMMQQPSFLNQELMMSSHLQYQG

## >At4g37130

MSFFPPQQQQTPQPLFQTQQTSLFQPQQTNSIFSQSQPQQTNSIFSQSQPQQTNSIFSQPQQQQQTSLFQPQQFQQQQQQLNQQQQQQVQQQLYLFTNDKAPANYSTKWADLHPDSQKLLLQIEEKILEHRSESQRLDQCSRLYDSSVSSEGFEFDASRIVQELGGINTAMDRQKAVLHELMIVAKDMLRNAEIAVRSFMMLQPRFPHWKQGGGVVSVGSQPSQGQGTNPAPASSGQQQAVTTTVQVSDFYRGIPKKPTAFLLQTVVRFEKYLNECRQWVEELEQLLALDSDKYSRHASLLESLPKVMSNVHDFFVHVAAKVESIHQYIESMRTSYLADQRRRGECHDPFLEADRRETAKQEAAAKRVHPTLHLPASTTSTQPSTQVAGLIASSATPGGSNPPQTSVPTSNPSSGAGFSFLNTPASGPSSSLFATPSSTAPTSSLFGPSPTPTQTPLFGSSPASTFGSTQSLFGQTTPSLTMPSQFGGATPGSGASFGSMTKSSRPKSRTTRR

## >At1g60280

MKVEDEATYELIKDELMKAEDEATYWLIKEELIKAEDDVIISRYLKRMIVNGDSWPDHFIEDVDVFNKNPNEEFHSQSPRFVIVKPRTENCGRTDGCQSGCWRIIGRDKLIKSKETGKILGFKKILKFCLKKKPREYKRSWVMEEYRLNNNLNCKQDHVICKIRFMFDAEISFLLAKHFSCLSTRSPLPANQLLPAYGVCFFDSEAEGAFYLETIIGYDGNTWPSYVTNDVYRLHPLTLVDPQDDKFKEFGTCIFANRTKTCGKTDECDGGGCWRIVEGHRVIKSKGKVLGYRRIFQFSENEEPRNVCEGEDPKKTAWFIEEYRPDENNKKDKVLCVIKFLIPLNQR

## >At1g60300

MEDDDAAYDLIKHELLYSEDEVIISRYLKGMVVNGDSWPDHFIEDANVFTKNPDKVFNSERPRFVIVKPRTEACGKTDGCDSGCWRIIGRDKLIKSEETGKILGFKKILKFCLKRKPIDYKRSWVMEEYRLTNNLNWKQDHVICKIRFMFEAEISFLLSKHFYTTSESVLENELLPSYGYYLSNTQEEDEFYLDAIMTSEGNEWPSYVTNNVYCLHPLELVDLQDRMFNDYGTCIFANKTCGETDKCDGGYWKILHGDKLIKSNFGKVIGFKKVFEFYETVRQIYLCDGEEVTVTWTIQEYRLSKNVKQNKVLCVIKLTYDR

## >At1g60340

MEDNDAAYDQVKSELLNSEDEVIISRYLKPMVVNGDSWPDHFIEDANVFTKNPNEVFNSERPRFVIVKPRTEACGKTDGCESGCWRTMGRDKLIKSEETGKILGFKKILKFCIKWKPIEYKRSWVMEEYRLTNNLNWKQDHVICKIRLLFEAEISFLLAKHFYTTSESLLRHELLPSYGYLSNTQEEDEFYLKTIMTSEGNDWPSYVTNNVYCLHPLELVDLQDVMFQSYGTCIFANGTCGESDKCDGGYWKILHGDKLIKSNFGMVTGFKKVFEFYETVIHRYFCDGEEVKEETERLGHQRCQGLLLPHRRGHVQTKSCGVVTVKEKRSLVEQPEEIVAAPETPRQGKSEHNDPLKRKKAIGCVGEKAEM

## >At1g60350

MEEDAAFDLLKAELLNAEDDAIISRYLKRMVVNGDSWPDHFIEDADVFNKNPNVEFDAESPSFVIVKPRTEACGKTDGCETGCWRIMGRDKPIKSTETVKIQGFKKILKFCLKRKPRGYKRSWVMEEYRLTNNLNWKQDHVICKIRFMFEAEISFLLAKHFYTTSESLPRNELLPAYGFLSSDKQLEDVSYPVTIMTSEGNDWPSYVTNNVYCLHPLELVDLQDRMFNDYGTCIFANKTCGKTDRCINGGYWKILHRDRLIKSKSGIVIGFKKVFKFHETEKERYFCGGEDVKVTWTLEEYRLSVKQNKFLCVIKFTYDN

## >At1g60380

MADTLLNAEDEVIISRYLKPMIVNRVSWPDLFIEDADVFNKDPYVKFHAEIPSFVIVKPRTKACGKTDGCDSGCWRIIGRDKLIKSEETGKILGFKKILKFCLKWKPREYKRSLVMEEYRLTNNFNWKQDHVICKIRLLFEAEISFLLAKHFYTTSDSLPRNVLLPAYGFCSPDKQEEDEFYPVTIMISEGKDWPSYVTNNVYCLHPSELVNVHDGKFHDNGICIFANRTCGVTDKCNEGYWKIKHREKLIMSRYGQTIGWKKVFQFYETEKERHFGNGEEVKVTWTLKEYRLTRKMNKNKVVCVIKYKVKCLPRITS

## >At3g18400

MEENLPPGFRFHPTDEELITHYLCRKVSDIGFTGKAVVDVDLNKCEPWDLPAKASMGEKEWYFFSQRDRKYPTGLRTNRATEAGYWKTTGKDKEIYRSGVLVGMKKTLVFYKGRAPKGEKSNWVMHEYRLESKQPFNPTNKEEWVVCRVFEKSTAAKKAQEQQPQSSQPSFGSPCDANSSMANEFEDIDELPNLNSNSSTIDYNNHIHQYSQRNVYSEDNTTSTAGLNMNMNMASTNLQSWTTSLLGPPLSPINSLLLKAFQIRNSYSFPKEMIPSFNHSSLQQGVSNMIQNASSSSQVQPQPQEEAFNMDSIW

## >At1g32770

MADNKVNLSINGQSKVPPGFRFHPTEEELLHYYLRKKVNSQKIDLDVIREVDLNKLEPWDIQEECRIGSTPQNDWYFFSHKDKKYPTGTRTNRATVAGFWKATGRDKIICSCVRRIGLRKTLVFYKGRAPHGQKSDWIMHEYRLDDTPMSNGYADVVTEDPMSYNEEGWVVCRVFRKKNYQKIDDCPKITLSSLPDDTEEEKGPTFHNTQNVTGLDHVLLYMDRTGSNICMPESQTTTQHQDDVLFMQLPSLETPKSESPVDQSFLTPSKLDFSPVQEKITERPVCSNWASLDRLVAWQLNNGHHNPCHRKSFDEEEENGDTMMQRWDLHWNNDDNVDLWSSFTESSSSLDPLLHLSV

## >At3g15510

MESTDSSGGPPPPQPNLPPGFRFHPTDEELVVHYLKRKAASAPLPVAIIAEVDLYKFDPWELPAKASFGEQEWYFFSPRDRKYPNGARPNRAATSGYWKATGTDKPVLASDGNQKVGVKKALVFYSGKPPKGVKSDWIMHEYRLIENKPNNRPPGCDFGNKKNSLRLDDWVLCRIYKKNNASRHVDNDKDHDMIDYIFRKIPPSLSMAAASTGLHQHHHNVSRSMNFFPGKFSGGGYGIFSDGGNTSIYDGGGMINNIGTDSVDHDNNADVVGLNHASSSGPMMMANLKRTLPVPYWPVADEEQDASPSKRFHGVGGGGGDCSNMSSSMMEETPPLMQQQGGVLGDGLFRTTSYQLPGLNWYSS

## >At2g33480

MMITGDCESEMYFFSTREAKYPNGNRSNRSTGSGYWKATGLDKQIGKKKLVVGMKKTLVFYKGKPPNGTRTNWVLHEYRLVDSQQDSLYGQNMNWVLCRVFLKKRSNSNSKRKEDEKEEVENEKETETEREREEENKKSTCPIFYDFMRKDTKKKRRRRRCCDLNLTPATCCCCSSSTSSSSVCSSALTHTSSNDNRQEISYRENKFCLFL

## >At1g26870

MGDRNNDGDQKMEDVLLPGFRFHPTDEELVSFYLKRKVQHNPLSIELIRQLDIYKYDPWDLPKFAMTGEKEWYFYCPRDRKYRNSSRPNRVTGAGFWKATGTDRPIYSSEGNKCIGLKKSLVFYKGRAAKGVKTDWMMHEFRLPSLSEPSPPSKRFFDSPVSPNDSWAICRIFKKTNTTTLRALSHSFVSSLPPETSTDTMSNQKQSNTYHFSSDKILKPSSHFQFHHENMNTPKTSNSTTPSVPTISPFSYLDFTSYDKPTNVFNPVSCLDQQYLTNLFLATQETQPQFPRLPSSNEIPSFLLNTSSDSTFLGEFTSHIDLSAVLAQEQCPPLVSLPQEYQETGFEGNGIMKNMRGSNEDHLGDHCDTLRFDDFTSTINENHRHHQDLKQNMTLLESYYSSLSSINSDLPACFSSTT

## >At5g63790

MNLPFVYKSSMDFALFSSISIFEINHKDPPIRRFIKTQNRILSTRKQQGTFPKMKAELNLPAGFRFHPTDEELVKFYLCRRCASEPINVPVIAEIDLYKFNPWELPEMALYGEKEWYFFSHRDRKYPNGSRPNRAAGTGYWKATGADKPIGKPKTLGIKKALVFYAGKAPKGIKTNWIMHEYRLANVDRSASTNKKNNLRLDDWVLCRIYNKKGTMEKYLPAAAEKPTEKMSTSDSRCSSHVISPDVTCSDNWEVESEPKWINLEDALEAFNDDTSMFSSIGLLQNDAFVPQFQYQSSDFVDSFQDPFEQKPFLNWNFAPQG

## >At3g57150

MAEVDISHSKKKKQDKTENDAADTGDYMIKPQSFTPAIDTSQWPILLKNYDRLNVRTGHYTPISAGHSPLKRPLQEYIRYGVINLDKPANPSSHEVVAWIKRILRVEKTGHSGTLDPKVTGNLIVCIDRATRLVKSQQGAGKEYVCVARLHSAVPDVAKVARALESLTGAVFQRPPLISAVKRQLRIRTIYESKLLEYDADRHLVVFWVSCEAGTYIRTMCVHLGLLLGVGGHMQELRRVRSGILGENNNMVTMHDVMDAQFVYDNSRDESYLRRVIMPLEMILTSYKRLVVKDSAVNAICYGAKLMIPGLLRFENDIDVGTEVVLMTTKGEAIAVGIAEMTTSVMATCDHGVVAKIKRVVMDRDTYPRKWGLGPRASMKKKLIADGKLDKHGKPNEKTPVEWSRNVVLPTGGDAIIAGAAAAPEEIKADAENGEAGEARKRKHDDSSDSPAPVTTKKSKTKEVEGEEAEEKVKSSKKKKKKDKEEEKEEEAGSEKKEKKKKKDKKEEVIEEVASPKSEKKKKKKSKDTEAAVDAEDESAAEKSEKKKKKKDKKKKNKDSEDDEE

## >At5g09330

MGKTQLAPGFRFHPTDVELVRYYLKRKILGKKLLVDAIAEVDIYKFEPPDLPDMSFIRSGDLKWHFFCPREKKYASGVRANRATECGYWKTTGKERPVLCNSEVVGKIKTLVYHFGKSPRGERTDWVMHEYRLDDKVLTQMNVPQDTYVVCVLFKKDGPGPRNGAQYGAPFKEEDWSDEEVRTDVPSTSNPTNLLEPSKETTLALTAPDDSNKDCFGGMISESCVSDFLPATTNTTSELPHPSDAATTPMSTAPLAETVQTPNNDDLYSMLDLFDDDEEFLGFNNNEVRYDPGVSAPVCLEEEGIFNGLPELSSMPRTASYDLVENSELYLELQDLTAPLNPQTGLQDLTAPFNPQTGLQDLTAPFNHQTGLQDHTAPFNPQTGLQDHTAPFNHQTGLQDLTAPFNPQTGLQDLTAPFNPQTGLHDLTSPFNPQTGLQDLTAPLNPQTGNRNDPRSSSFLYNQGHFDFSGGNDDDPYGFSASMRHRPKM

## >At5g64060

MGKTNLAPGFRFHPTDVELVRYYLKRKVMGKKFQVDAIAEVDIYKFEPPDLPDKSCLGTGDLKWYFFCPREKKYPKGGKANRSTECGYWKTTGRDRDVSYNDEVTGKIRTLIYHYGKIPRGDRTDWVIHEYRLEDKVLAQKNVPQDTYVLCVLFKKNGLGPRHGSQYGAPFKEEDWSDKEEEYTQNHLVAGPSKETSLAAKASHSYAPKDGLTGVISESCVSDVPPLTATVLPPLTSDVIAYNPFSSSPLLEVPQVSLDGGELNSMLDLFSVDNDDCLLFDDFDYHNEVRHPDGFVNKEAPVFLGDGNFSGMFDLSNDQVVELQDLIQSPTPHPPSPPAQASIPDDSRSNGQTKDD

## >At3g56570

SADLDGSLGLSVALMYERSLGEESPWAGYLQILPIQEDLPLVWSLEDLDSLLSGTELHKLVKEDHVLIYEDWKENILPLTSSLPQNVDSDSFGIKEYLAAKSLIASRSFEIDDYHGSGMVPLADLFNHKTGAEDVHFTHESDSEADESDNDDAANETTDEDEPSSKISSSPEQSFEEVPGENTDDEAKEEEEEEEEEEEGEEEEEGEEEEENSSMLQNDQSGLKMIMVKDVSAGAEVFNTYGLMGNAALLHRYGFTELDNPYDIVNIDLELVTEWSTSSFTSRYTRARLALWRKLGYTGCESQNSEYFEVSSTGEPQTELLILLYILLLPDDTYNKLDLAESTTGASPSKEGKRSSSYEITIGKHKFVYGESGNDILLTDGVCEALLTIVDKRESLYGSLSSLEDDIVRVKTCCLPRDRRLYHSLVLRVSERKILKKLRSYIHTQTNESSSGKRRKKMVPKS

## >At5g56620

MKNSKCNLIDSKLEEHHHLCGSKHCPGCGRMIQAATKPNWVGLPAGVKFDPTDQELIEHLEAKVKGKEENKKWSSSHPLIDEFIPTIDGEDGICYTHPQKLPGVTRDGLSKHFFHKPSRAYTTGTRKRRKIIQTDHDSELTGSSETRWHKTGKTRPVMINGQQRGCKKILVLYTNFGKNRRPEKTNWVMHQYHLGINEEEREGELVVSKIFYQTQPRQCVSNTNWSDHHGSKDVIGIGVGDEISSVAATLQSLGSGDVVSRVNMHPHTRSFDEGTAEASKGRENQHVSGTCEEVHDGIITSSMSSHHMIHDHHNQHHQIGDRREFHMSSSYPMTPTITSQHESIFHVTSTMPFQRQQLRGRSSGSGLEDLIMGCTTATCTEDEHSEANPQRNAEWLTFPQFW

## >At5g39690

MAKKEKIEQVISMGGIMWEGLNSSLIKVDEALLKQQIREFEKGNDKEWFIITERNKVDQGLSQTKRVGNGAKRQKRVDTNGGYWHATVAAQKINAGDGVVGNKRPLAYYDRKPSEDVKTDWLMQEYSLDHNNDKVRLHFVQDLSYSTSNKEVGEEKKKQKKGEPVEASEGQQPCNAEYHQPLAPLDSCQPQPHDLAEQLDLHQPEQLQLQQPHDIVYQPQYCLLPEQHQLQPFPDNFSELNSFQQQPVMIPDDLEDFLAELMEPHSLDGDEESNNYGFFEGLFDTEGINDKTLH

## >At5g39540

MGFSNPTIMSFEVRSEKFDTIALPSGSFANMLIPYQGRLACVTNTMKNDVNGGIILWTLDDAEKHIWSCKLFLAPFAHMVIIVCMYVLLSHNSNKDYNFCKIYLTPQAIKKKKEVEEEKKKQKKGEAVVSVAHVEALEEQQPCNVEYHQPLAPLDSCQPQRHDLEYQQQQFCPGPLDSYQPHPHDMESQQPHNPLL

## >At3g56560

MFDFKIFTFSFAQSKYLENIREKTLTEVSTSDIELLRRNSQPFRRATPCSLTVDLQANSFTPKLQSYLMMMPGRNKRKERSWPEPQTQPQSEIPSSSSSLAADNMSPPSTIAYVLPPGFKFMPNDKEVINCYLKPYSDGNTNVLLNVPIHRVNIYESNPQTLSGRNVATLTEDIGTQKWLPKRFKAGDGVVGNKRPLAYYVGKPSEGVKTDWLMQEYSLDHSSHNTTR

## >At3g44290

MAAAPPIEPAVTTTFPGFKFSPTDIELISYYLKRKMDGLERSVEIIPEVEIYNFEPWDLPDKSIVKSDSEWFFFCARGKKYPHGSQNRRATKIGYWKATGKERNVKSGSEVIGTKRTLVFHIGRAPKGGRTEWLMHEYCMIGVSLDALVICRLRRNTEFQGSTIQKPPQPSLPLDKHVNLRNEAISESIYGWETMVDFYLSSESGQELLSEIAESSQSSQNPQEFFHV

## >At4g28500

MTWCNDRSDVQTVERIIPSPGAAESPVASLPVSCHKTCPSCGHNFKFHEQAGIHDLPGLPAGVKFDPTDQEVLEHLEGKVRDDAKKLHPLIDEFIRTIDGENGICYTHPEKLPGVNKDGTVRHFFHRPSKAYTTGTRKRRKVHTDSDVGGETRWHKTGKTRPVLAGGRVRGYKKILVLYTNYGKQKKPEKTNWVMHQYHLGTSEEEKEGELVVSKVFYQTQPRQCGGSVAAAATAKDRPYLHGLGGGGGRHLHYHLHHNNGNGKSNGSGGTAGAGEYYHNIPAIISFNQTGIQNHLVHDSQPFIP

## >At1g76420

MMLAVEDVLSELAGEERNERGLPPGFRFHPTDEELITFYLASKIFHGGLSGIHISEVDLNRCEPWELPEMAKMGEREWYFYSLRDRKYPTGLRTNRATTAGYWKATGKDKEVFSGGGGQLVGMKKTLVFYKGRAPRGLKTKWVMHEYRLENDHSHRHTCKEEWVICRVFNKTGDRKNVGLIHNQISYLHNHSLSTTHHHHHEALPLLIEPSNKTLTNFPSLLYDDPHQNYNNNNFLHGSSGHNIDELKALINPVVSQLNGIIFPSGNNNNDEDDFDFNLGVKTEQSSNGNEIDVRDYLENPLFQEASYGLLGFSSSPGPLHMLLDSPCPLGFQL

## >At1g33280

MSSSNGGVPPGFRFHPTDEELLHYYLKKKISYEKFEMEVIKEVDLNKIEPWDLQDRCKIGSTPQNEWYFFSHKDRKYPTGSRTNRATHSGFWKATGRDKCIRNSYKKIGMRKTLVFYKGRAPHGQKTDWIMHEYRIEDTEDDPCEDGWVVCRVFKKKNLFKVGNDVGSNISNNRLEARSFIRRESPYQGISMFELNKPEEISVHQYPQPPMFQPHHKPLSIGYDYSLALLPRESEYQQACQPSGVEVGTCKAVSEWGIVNCNMVSHEDSSRAMRFEDDGNNTSSTVQPPSNLLSLRGENGFLGLF

## >At1g32510

MVGSFLPPGFRFYPTDEELVGYYLHRRNEGLEIELEIIPLMDLYKFDPWELPEKSFLPNRDMEWFFFCHRDRKYQNGSRINRATKSGYWKATGKDRKIVCHSSSSSSSSSITGCRKTLVFYMGRAPFGGRTEWVMHEYRLFDNDTSQGSLNFKGDFALCRVIKRNEHTLKKCEIISPEVSDESLSNNVNNFCQASDLEKGSCDASNTRLSSPDFILESSFQGNSHSKTEEDSGFQVFTLPEFEYPLEVFADLNFDLEMEDPFMFDYHPEPHMNNEVMSHHIRG

## >At4g27410

MGVREKDPLAQLSLPPGFRFYPTDEELLVQYLCRKVAGYHFSLQVIGDIDLYKFDPWDLPSKQTCFTFVGEYNCNYLGKALFGEKEWYFFSPRDRKYPNGSRPNRVAGSGYWKATGTDKIITADGRRVGIKKALVFYAGKAPKGTKTNWIMHEYRLIEHSRSHGSSKLDDWVLCRIYKKTSGSQRQAVTPVQACREEHSTNGSSSSSSSQLDDVLDSFPEIKDQSFNLPRMNSLRTILNGNFDWASLAGLNPIPELAPTNGLPSYGGYDAFRAAEGEAESGHVNRQQNSSGLTQSFGYSSSGFGVSGQTFEFRQ

## >At5g24590

MNQNLHVLSMDSLPVGLRFRPTDEELIRYYLRRKINGHDDDVKAIREIDICKWEPWDLPDFSVIKTKDSEWLYFCPLDRKYPSGSRQNRATVAGYWKATGKDRKIKSGKTNIIGVKRTLVFHAGRAPRGTRTNWIIHEYRATEDDLSGTNPGQSPFVICKLFKKEELVLGEEDSKSDEVEEPAVSSPTVEVTKSEVSEVIKTEDVKRHDIAESSLVISGDSHSDACDEATTAELVDFKWYPELESLDFTLFSPLHSQVQSELGSSYNTFQPGSSNFSGNNNNSFQIQTQYGTNEVDTYISDFLDSILKSPDEDPEKHKYVLQSGFDVVAPDQIAQVCQQGSAVDMSNDVSVTGIQIKSRQAQPSGYTNDYIAQGNGPRRLRLQSNFNGINTKNPELQAIKREVRITPTF

## >At2g18060

MAGFWKATGRDKAVYDKTKLIGMRKTLVFYKGRAPNGKKSDWIMHEYRLESDENAPPQEEGWVVCRAFKKRATGQAKNTETWSSSYFYDEVAPNGVNSVMDPIDYISKQQHNIFGKGLMCKQELEGMVDGINYIQSNQFIQLPQLQSPSLPLMKRPSSSMSITSMDNNYNYKLPLADEESFESFIRGEDRRKKKKQVMMTGNWRELDKFVASQLMSQEDNGTSSFAGHHIVNEDKNNNDVEMDSSMFLSEREEENRFVSEFLSTNSDYDIGICVFDN

## >At4g36160

MESVDQSCSVPPGFRFHPTDEELVGYYLRKKVASQKIDLDVIRDIDLYRIEPWDLQESCRIGYEERNEWYFFSHKDKKYPTGTRTNRATMAGFWKATGRDKAVYDKSKLIGMRKTLVFYKGRAPNGQKTDWIMHEYRLESDENAPPQEEGWVVCRAFKKKPMTGQAKNTETWSSSYFYDELPSGVRSVTEPLNYVSKQKQNVFAQDLMFKQELEGSDIGLNFIHCDQFIQLPQLESPSLPLTKRPVSLTSITSLEKNKNIYKRHLIEEDVSFNALISSGNKDKKKKKTSVMTTDWRALDKFVASQLMSQEDGVSGFGGHHEEDNNKIGHYNNEESNNKGSVETASSTLLSDREEENRFISGLLCSNLDYDLYRDLHV

## >At5g66300

MMKVDQDYSCSIPPGFRFHPTDEELVGYYLKKKIASQRIDLDVIREIDLYKIEPWDLQERCRIGYEEQTEWYFFSHRDKKYPTGTRTNRATVAGFWKATGRDKAVYLNSKLIGMRKTLVFYRGRAPNGQKSDWIIHEYYSLESHQNSPPQEEGWVVCRAFKKRTTIPTKRRQLWDPNCLFYDDATLLEPLDKRARHNPDFTATPFKQELLSEASHVQDGDFGSMYLQCIDDDQFSQLPQLESPSLPSEITPHSTTFSENSSRKDDMSSEKRITDWRYLDKFVASQFLMSGED

## >At1g12260

MNSFSHVPPGFRFHPTDEELVDYYLRKKVASKRIEIDFIKDIDLYKIEPWDLQELCKIGHEEQSDWYFFSHKDKKYPTGTRTNRATKAGFWKATGRDKAIYLRHSLIGMRKTLVFYKGRAPNGQKSDWIMHEYRLETDENGTPQEEGWVVCRVFKKRLAAVRRMGDYDSSPSHWYDDQLSFMASELETNGQRRILPNHHQQQQHEHQQHMPYGLNASAYALNNPNLQCKQELELHYNHLQSNIAHEEQLNQGNQNFSSLYMNSGNEQVMDQVTDWRVLDKFVASQLSNEEAATASASIQNNAKDTSNAEYQVDEEKDPKRASDMGEEYTASTSSSCQIDLWK

## >At5g62380

MESLAHIPPGYRFHPTDEELVDYYLKNKVAFPGMQVDVIKDVDLYKIEPWDIQELCGRGTGEEREWYFFSHKDKKYPTGTRTNRATGSGFWKATGRDKAIYSKQELVGMRKTLVFYKGRAPNGQKSDWIMHEYRLETDENGPPHEEGWVVCRAFKKKLTTMNYNNPRTMMGSSSGQESNWFTQQMDVGNGNYYHLPDLESPRMFQGSSSSSLSSLHQNDQDPYGVVLSTINATPTTIMQRDDGHVITNDDDHMIMMNTSTGDHHQSGLLVNDDHNDQVMDWQTLDKFVASQLIMSQEEEEVNKDPSDNSSNETFHHLSEEQAATMVSMNASSSSSPCSFYSWAQNTHT

## >At1g71930

MDNIMQSSMPPGFRFHPTEEELVGYYLDRKINSMKSALDVIVEIDLYKMEPWDIQARCKLGYEEQNEWYFFSHKDRKYPTGTRTNRATAAGFWKATGRDKAVLSKNSVIGMRKTLVYYKGRAPNGRKSDWIMHEYRLQNSELAPVQEEGWVVCRAFRKPIPNQRPLGYEPWQNQLYHVESSNNYSSSVTMNTSHHIGASSSSHNLNQMLMSNNHYNPNNTSSSMHQYGNIELPQLDSPSLSPSLGTNKDQNESFEQEEEKSFNCVDWRTLDTLLETQVIHPHNPNILMFETQSYNPAPSFPSMHQSYNEVEANIHHSLGCFPDS

## >At1g62700

MNSFSQVPPGFRFHPTDEELVDYYLRKKVASKRIEIDIIKDVDLYKIEPCDLQELCKIGNEEQSEWYFFSHKDKKYPTGTRTNRATKAGFWKATGRDKAIYIRHSLIGMRKTLVFYKGRAPNGQKSDWIMHEYRLETSENGTPQEEGWVVCRVFKKKLAATVRKMGDYHSSPSQHWYDDQLSFMASEIISSSPRQFLPNHHYNRHHHQQTLPCGLNAFNNNNPNLQCKQELELHYNQMVQHQQQNHHLRESMFLQLPQLESPTSNCNSDNNNNTRNISNLQKSSNISHEEQLQQGNQSFSSLYYDQGVEQMTTDWRVLDKFVASQLSNDEEAAAVVSSSSHQNNVKIDTRNTGYHVIDEGINLPENDSERVVEMGEEYSNAHAASTSSSCQIDL
