# Supplementary material for: Comprehensive genomic characterization of NAC transcription factor family and their response to salt and drought stress in peanut
Source: BMC Plant Biol. 2020 Oct 2;20:454. doi: 10.1186/s12870-020-02678-9 (PMC7532626; doi:10.1186/s12870-020-02678-9)
Supplement: Supplementary file 7 — Additional file 7. Rice NAC proteins. [file 12870_2020_2678_MOESM7_ESM.docx]

[>Os01g01430 6](#_Toc517537223)

[>Os01g01470 6](#_Toc517537224)

[>Os01g09550 6](#_Toc517537225)

[>Os01g15640 7](#_Toc517537226)

[>Os01g29840 7](#_Toc517537227)

[>Os01g48130 7](#_Toc517537228)

[>Os01g48446 8](#_Toc517537229)

[>Os01g59640 8](#_Toc517537230)

[>Os01g60020 8](#_Toc517537231)

[>Os01g64310 9](#_Toc517537232)

[>Os01g66490 9](#_Toc517537233)

[>Os01g66120 9](#_Toc517537234)

[>Os01g70110 10](#_Toc517537235)

[>Os01g71790 10](#_Toc517537236)

[>Os02g06950 10](#_Toc517537237)

[>Os02g12310 11](#_Toc517537238)

[>Os02g15340 11](#_Toc517537239)

[>Os02g18460 11](#_Toc517537240)

[>Os02g18470 12](#_Toc517537241)

[>Os02g34970 12](#_Toc517537242)

[>Os02g36880 13](#_Toc517537243)

[>Os02g38130 13](#_Toc517537244)

[>Os02g41450 13](#_Toc517537245)

[>Os02g56600 14](#_Toc517537246)

[>Os02g57650 14](#_Toc517537247)

[>Os02g42970 15](#_Toc517537248)

[>Os03g01870 15](#_Toc517537249)

[>Os03g02800 15](#_Toc517537250)

[>Os03g03540 16](#_Toc517537251)

[>Os03g04070 16](#_Toc517537252)

[>Os03g12120 17](#_Toc517537253)

[>Os03g21030 17](#_Toc517537254)

[>Os03g21060 18](#_Toc517537255)

[>Os03g39050 18](#_Toc517537256)

[>Os03g39100 19](#_Toc517537257)

[>Os03g42630 19](#_Toc517537258)

[>Os03g56580 19](#_Toc517537259)

[>Os03g59730 20](#_Toc517537260)

[>Os06g33940 20](#_Toc517537261)

[>Os06g23650 21](#_Toc517537262)

[>Os06g04090 21](#_Toc517537263)

[>Os06g01480 21](#_Toc517537264)

[>Os06g01230 22](#_Toc517537265)

[>Os05g48850 22](#_Toc517537266)

[>Os05g43960 22](#_Toc517537267)

[>Os05g35170 23](#_Toc517537268)

[>Os05g34830 23](#_Toc517537269)

[>Os05g34600 24](#_Toc517537270)

[>Os05g34310 24](#_Toc517537271)

[>Os05g25960 24](#_Toc517537272)

[>Os05g10620 25](#_Toc517537273)

[>Os04g59470 25](#_Toc517537274)

[>Os04g52810 26](#_Toc517537275)

[>Os04g43560 26](#_Toc517537276)

[>Os04g38720 26](#_Toc517537277)

[>Os04g35660 27](#_Toc517537278)

[>Os03g62470 27](#_Toc517537279)

[>Os06g36480 27](#_Toc517537280)

[>Os06g46270 28](#_Toc517537281)

[>Os06g51070 28](#_Toc517537282)

[>Os07g04560 28](#_Toc517537283)

[>Os07g09740 29](#_Toc517537284)

[>Os07g09830 29](#_Toc517537285)

[>Os07g09860 30](#_Toc517537286)

[>Os03g61319 30](#_Toc517537287)

[>Os03g61249 31](#_Toc517537288)

[>Os03g60080 31](#_Toc517537289)

[>Os12g43530 31](#_Toc517537290)

[>Os12g22940 32](#_Toc517537291)

[>Os12g23090 32](#_Toc517537292)

[>Os12g41680 33](#_Toc517537293)

[>Os12g29330 33](#_Toc517537294)

[>Os12g05990 33](#_Toc517537295)

[>Os12g03050 34](#_Toc517537296)

[>Os12g03040 34](#_Toc517537297)

[>Os11g31380 34](#_Toc517537298)

[>Os11g31360 35](#_Toc517537299)

[>Os11g31340 35](#_Toc517537300)

[>Os11g31330 35](#_Toc517537301)

[>Os11g08210 36](#_Toc517537302)

[>Os11g07700 36](#_Toc517537303)

[>Os11g05614 36](#_Toc517537304)

[>Os11g04960 37](#_Toc517537305)

[>Os11g03370 37](#_Toc517537306)

[>Os11g03310 37](#_Toc517537307)

[>Os11g03300 38](#_Toc517537308)

[>Os10g42130 38](#_Toc517537309)

[>Os10g38834 38](#_Toc517537310)

[>Os10g33760 39](#_Toc517537311)

[>Os10g27390 39](#_Toc517537312)

[>Os08g33910 40](#_Toc517537313)

[>Os10g27360 40](#_Toc517537314)

[>Os10g26240 40](#_Toc517537315)

[>Os10g25640 41](#_Toc517537316)

[>Os10g25620 41](#_Toc517537317)

[>Os10g21560 41](#_Toc517537318)

[>Os10g09820 42](#_Toc517537319)

[>Os09g38000 42](#_Toc517537320)

[>Os09g33490 43](#_Toc517537321)

[>Os09g32260 43](#_Toc517537322)

[>Os09g32040 43](#_Toc517537323)

[>Os09g24560 44](#_Toc517537324)

[>Os09g12380 44](#_Toc517537325)

[>Os08g44820 45](#_Toc517537326)

[>Os08g42400 45](#_Toc517537327)

[>Os08g40030 46](#_Toc517537328)

[>Os08g10080 46](#_Toc517537329)

[>Os08g23880 46](#_Toc517537330)

[>Os08g33670 47](#_Toc517537331)

[>Os11g45950 47](#_Toc517537332)

[>Os12g07790 47](#_Toc517537333)

[>Os05g37080 48](#_Toc517537334)

[>Os07g12340 48](#_Toc517537335)

[>Os07g13920 48](#_Toc517537336)

[>Os07g17180 49](#_Toc517537337)

[>Os07g27330 49](#_Toc517537338)

[>Os07g27340 50](#_Toc517537339)

[>Os07g37920 50](#_Toc517537340)

[>Os07g48450 51](#_Toc517537341)

[>Os07g48550 51](#_Toc517537342)

[>Os08g01330 51](#_Toc517537343)

[>Os08g02160 52](#_Toc517537344)

[>Os08g02300 52](#_Toc517537345)

[>Os08g06140 52](#_Toc517537346)

[>Os09g38010 53](#_Toc517537347)

[>Os01g28050 53](#_Toc517537348)

[>Os01g47670 53](#_Toc517537349)

[>Os03g61650 54](#_Toc517537350)

[>Os04g39960 54](#_Toc517537351)

[>Os04g40140 54](#_Toc517537352)

[>Os04g42940 54](#_Toc517537353)

[>Os05g26049 54](#_Toc517537354)

[>Os05g26026 55](#_Toc517537355)

[>Os05g27749 55](#_Toc517537356)

[>Os12g04230 55](#_Toc517537357)

[>Os07g31410 55](#_Toc517537358)

[>Os10g27380 55](#_Toc517537359)

[>Os11g04470 56](#_Toc517537360)

[>Os11g31370 56](#_Toc517537361)

[>Os12g22630 56](#_Toc517537362)

[>Os11g04360 56](#_Toc517537363)

## >Os01g01430

MMTIDLQLPAAACGDHHTAAGAGLPPGFRFHPTDEELLLHYLGKRAAAAPCPAPVIAEVDIYKYNPWELPAMAVFGESDGEWYFFSPRDRKYPNGVRPNRAAGSGYWKATGTDKPISISETQQTVLLGVKKALVFYRGRPPKGTKTSWIMHEYRLANAAASSSSSYTSNMKQLASSSSSSSSSASMRLDEWVLCRIYKKKEANQQLQHYIDMMMDDDNDDEHNLQVQQQQQQQAQSHRMPRPPSISDYLLDYSDDLPPSTDQTPSLHLGFTAVNEGNNKRHKTMEEYYSISISTADMLHASSSTSNNKSTQINFSSIFEPQTPAAAGHQLMSSHNDDTSI*

## >Os01g01470

MGEQQQQVERQPDLPPGFRFHPTDEEIITFYLAPKVVDSRGFCVAAIGEVDLNKCEPWDLPGKAKMNGEKEWYFYCQKDRKYPTGMRTNRATEAGYWKATGKDKEIFRDHHMLIGMKKTLVFYKGRAPKGDKTNWVMHEYRLADASPPPPPSSAEPPRQDDWAVCRIFHKSSGIKKPVPVAPHQVPAAANYQQQQQMAMASAGIIQVPMQMQMPSMSDQLQMLDDFSTTASLSLMAPPSYSTLPAGFPLQINSGAHPQQFVGNPSMYYHQQQQMDMAGGGFVVSEPSSLVVSPQDAADQNNNAADISSMACNMDAAIWKY*

## >Os01g09550

MYTSPTLLALSFALLLLILLLYYSSSLLYSQLATRRRLTHACARSLPDAYESGMNRGHISSSELIDAKLEERRISTAKHCPSCGNKLDCKPDWVGLPAGVKFDPTDQELIEHLEAKVREEGSRSHPLIDEFIPTIEGEDGICYTHPEKLPGVTRDGLSKHFFHRPSKAYTTGTRKRRKIQTECDVQKGETRWHKTGKTRPVMVSGRQKGCKKILVLYTNFGKHRKPEKTNWVMHQYHLGDLEEEKEGELVVCKIFYQTQPRQCSWSSDRGGGAAATASAVTTAAVQQDQQRRRDSGSGSCSSTRDHEVSATSYSTAGYAVAAAVEMQHLKHAADHFSFAPFRKSFEEVGISGDQVHSNQLGRSEQQHAGQEQQPHRPLLATTTAVPATAFLISRPTNPVSNIVPPAMQHASVVLDHDQFHVPAILLHHDKFQQQQQKLDRRSAGLEELIMGCTSSSTKGEASIPHSQETEWPYQPYWTPDNQDHHG*

## >Os01g15640

MESLRDMVLPPGFGFHPKDTELISHYLKKKIHGQKIEYEIIPEVDIYKHEPWDLPAKCDVPTQDNKWHFFAARDRKYPNGSRSNRATVAGYWKSTGKDRAIKMGKQTIGTKKTLVFHEGRPPTGRRTEWIMHEYYIDERECQACPDMKDAYVLCRITKRNDWIPGNGNELDNSDPHPEPYDAPPSVISTEQLNPAAEPVVGVEAAPVTVAEPDGVTTSAITANIPSPSDDINLDDWLNELFDPFFDPEQSLASADLSPDEQNVESSNVGALAPKVEQDYSSPNENVVDDTEYLLPEDVYNILHPGTDDFNMLQNPLDQYPIQYATDVWSGIQKEELWSPQANAEPSQSNEAADNGIIRRYRSMKTPETSVPQFKGKTQAKMRVGINKMATSSSESINQTIKFENSGRLVEHQKNQAHDVASTKRSDAGKPSTELSSNRGFLRGIRNAFAGCSDARWNMILVAGFAIGVAVVALHIGQRLGLSQRDQQHT*

## >Os01g29840

MGEQQQQVERQPDLPPGFRFHPTDEEIITFYLAPKVVDSRGFCVAAIGEVDLNKCEPWDLPGKAKMNGEKEWYFYCQKDRKYPTGMRTNRATEAGYWKATGKDKEIFRNHHMLIGMKKTLVFYKGRAPKGDKTNWVMHEYRLADASPPQPPPPPSSAEPPRQDDWAVCRIFHKSSGIKKPVQVPMQMPMQMQMPVAHQVPAANYQQQMAMASASIIQVPMQMQMPSMSDQLQMLDDFSTGSLMAPPPPPPSYSTLPGFPLQINGGAQQFVGNPSMYYQQQQQQQQQQMDMAAGGFVVSEPSSLVVSPQDAADQNNAADISSVACNMDATIWKY*

## >Os01g48130

MTWCNSFNDVRAVENNLATAAAVAAAKKQQQQQQVSQHVNLIKTCPSCGHRAQYEQAAAAATIQDLPGLPAGVKFDPTDQELLEHLEGKARPDARKLHPLIDEFIPTIEGENGICYTHPERLPGVGKDGLIRHFFHRPSKAYTTGTRKRRKVHTDEQGGETRWHKTGKTRPVFTGGKLKGYKKILVLYTNYGKQRKPEKTNWVMHQYHLGSDEEEKDGELVVSKVFYQTQPRQCGGGSAATAKDLSVDLVAGNNIKASNAAAEHHHNDGVGGGGHGGNNSSMLKEAAGIVDFYNPAAALIGYSQAAPNNRAAASAHLTMPNFEVHTGGAGFGP*

## >Os01g48446

MSPSRPDEADPAADFGSHPTDQELVTKYLRRHVDSGGNPWRYVHEADVYAADPDDLTGKYSPAVASDGSRAWYFFTTVRSKSTGGQRRARAVGDGGCWHSEAGAKDVVGGIRSPRPIGRRQFFSFVNKEGPRRVRSGWIMVEIGLKYAQQNASSDELVLCKVYRSPRAPPAAAAANKSMAAPPPTATKSKTEEATPPPDDVKPVVAAAQTPDTKILRAAKEAAATGCKRKADVKSSGARRGKRLCSRCRAETSESDSETAVLDRSPSIEDETADSSEIHGSSDGKFIRFL*

## >Os01g59640

MAESSESAWPQQSQQLQISSTMPAGSAWPEEENLENLEQPLPLLMPSSEDHREQQLVPVPWLQQDQDQEWHEQEQFLPLKNQNQEQLQDQQPLQDQEETRRYLGVPGIRFVPSDIELILDFLRPKLRGEQLPSYSYMHVCDVYSDHPKELTSKLGPSREGNWYMFSPRNRKYNKGKRPSRSTGQLGFWKSTTKNEAVLDALSDNMLIGYKACLTYHEYDESMPTPKLKKENAIKTPWKMWEFVCSNSNRPFDAEEEPMRLNDWVLCKVTNKDNKVTTKKFKPQRSKKPKKPKKLQQEEQPQNQGIVIRQPSESGSASSSHQEIPGSSLPGAGGDAAAAAATAAAVVDPMPLHMIPPSSWNYFSTGVTADGIVMDDSTGVDSYGCVDGAGALNFQRNIFYHR*

## >Os01g60020

MEMAAAVGGSGRRDAEAELNLPPGFRFHPTDEELVVHYLCRKVARQPLPVPIIAEVDLYKLDPWDLPEKALFGRKEWYFFTPRDRKYPNGSRPNRAAGRGYWKATGADKPVAPKGSARTVGIKKALVFYSGKAPRGVKTDWIMHEYRLADADRAPGGKKGSQKLDEWVLCRLYNKKNNWEKVKLEQQDVASVAAAAPRNHHHQNGEVMDAAAADTMSDSFQTHDSDIDNASAGLRHGGCGGGGFGDVAPPRNGFVTVKEDNDWFTGLNFDELQPPYMMNLQHMQMQMVNPAAPGHDGGYLQSISSPQMKMWQTILPPF*

## >Os01g64310

MADGGGRRAPGFRFYPTEEELICFYLRNKLDGLRDDIERVIPVFDVYSVDPLQLSEIHHEMLGGGGEEGEPWFYFCPRQEREARGGRPSRTTPSGYWKAAGTPGVVYSADRRPIGMKKTMVFYRGRAPSGTKTAWKMNEYRAFHYPDASSASASSAGAAAPPNHLPPQLRSEFSLCRLYTRSGGIRQFDRRPLAGGGDENPGPSMAAAAASPEENDGSGSSMQQLELMDQGGAVDPDWDQWDDLATLTALLYWPRD*

## >Os01g66490

MHMRRGRAAGGEGEAAAVVVMNRYDNNGHAAAAAAAAVAGGGGGGGGNKAAGEVDGHEDDLVMPGFRFHPTEEELIEFYLRRKVEGKRFNVELITFLDLYRYDPWELPAMAAIGEKEWFFYVPRDRKYRNGDRPNRVTASGYWKATGADRMIRAENNRPIGLKKTLVFYSGKAPKGVRSSWIMNEYRLPPADTDRYHKTEISLCRVYKRTGIDDGHGQVSTARSSAHSRGGGAAPVQDNKQGSSSTSTPTPPPTPSKLHLLSSECTSPPAIVTDHAAMVAHKAPSPRHHQQQQQLHAAKPCGGYLQNSSMASAAGGDQQQQFQQDFAAALYQQYSKNTSGAFASTYSLLNLVNAASMGSSAAAIDELSSLVGHGTPSYINPAAGSHNYSQFLHLPTTPSSHQPTPAPLGTTTAAAAATLPMSLAAFSDRIWDWNNPIPEAGGRDYSTSTGFK*

## >Os01g66120

MSGGQDLQLPPGFRFHPTDEELVMHYLCRRCAGLPIAVPIIAEIDLYKFDPWQLPRMALYGEKEWYFFSPRDRKYPNGSRPNRAAGSGYWKATGADKPVGSPKPVAIKKALVFYAGKAPKGEKTNWIMHEYRLADVDRSARKKNSLRLDDWVLCRIYNKKGGLEKPPAAAVAAAGMVSSGGGVQRKPMVGVNAAVSSPPEQKPVVAGPAFPDLAAYYDRPSDSMPRLHADSSCSEQVLSPEFACEVQSQPKISEWERTFATVGPINPAASILDPAGSGGLGGLGGGGSDPLLQDILMYWGKPF*

## >Os01g70110

MAAEAASGGGGGYRMLPQAGLPIGFRFRPTDEELLLHYLRRKVMSRPLPADVIPVADLARLHPWDLPGEGDGERYFFHLPATSCWRRGGGGSRAGGGGGAWRASGKEKLVVAPRCGKRPVGAKRTLVFFRRGGARTDWAMHEYRLLPADDHPPEANDVWVVCRVFKKTTTLAHRRSPPSIRGAPRRRAAAADDDDMPSSPSSCVTDGGDAGEEGEESSSCSVVASNCP*

## >Os01g71790

MGKEMNLIREDEYGGGGVGFEPTEDELMLHFLRPQLRGFAPRVAGAVVEADPCGAAPWELLARHGRREEGFFFSARARRKPSVRRTVAGCGGGGGGGGAWMHSSTKNGQSVTDLGVVVRWCRINYCFYVRGEMGQQRSTGWMMAEYEITDPRCYRRADDGEEDDFWVLCHVRKSSRPQAAKISPAKPARRRKPAAAAAADVRAA*

## >Os02g06950

MVEARLPPGFRFHPRDDELVVDYLSGKLRSGDGGAASGGGAAGAGCPTPTLIDVDLNKCEPWDLPEIACIGGKEWYFYNLKDRKYARGQRTNRATESGYWKATGKDREITRKGSLVGMRKTLVFYRGRAPKGERTDWVMHEFRQELDHANHHHHLKVLAHRFRFQFALDCIISHSHASWQLDYMQEGWVLCRVFYKSRTEAVAAPTMESTLPPRYINGGTSRSPLPPLVDSSISFNHGGYEEVLPCFSSSHHQQPSPASMNASAAADDDQDYHHLSEGQRHYSDKKMMRDVQNDQVTTRFDGHLAVKREMSLKKDLSEDEQAAPNADAGGFSILLKYSVSKMTSLMKPIQRNISTLQEFLNQKKEAILEKVEIFTKLLLPSRLGSAVFQLCLEHLIKNHKVGISWDGIWELSDWEVADNEVVLKMVGQCSAPADSKSKDLKRLFDLLRPYYDQEGKDPHLFFEHLKFDFTDVLKTIVTDAKWEWFWKYLLNHVFVMPPTGNTY*

## >Os02g12310

MAMTPQLAFSRMPPGFRFQPTDEQLVVDYLQRRTAAQPCVTPDITDIDVYNVDPWQLPAMAMYGSDHDRYFFTMAAREAQARRTTPSGFWKPTGTKKTIFVVAGGHEVPTAVKRRFVFYLGHHQPSGSNNNNKTSWIMHEYRLMNSPRAAVPSSSSVNRLPTDDLTEEMVLCRISNKDLPKPPFIHNSLLQFSSVGLNGDGYNYLILDHLEPPAMEYPNVGIGNVDDAAAGTDDPGDLDEEIDDSMQRNHGG*

## >Os02g15340

MHPNGAPLAVPPGFRFHPTDEELLYYYLRKKVAYEAIDLDVIREIDLNKLEPWDLKDRCRIGTGAQEEWYFFSHKDKKYPTGTRTNRATVAGFWKATGRDKAIFLGSGGGTRIGLRKTLVFYTGRAPHGKKTDWIMHEYRLDDDNVDVPEEGWVVCRVFKKKSIHQRGFDQPDMAAAADEDELRYQLLHGAGMSSSPVDQKHVLLQEQLVAHGAHGGGFVVPAFEASMHLPQLASADAAPCGGGGGGHVAFASMNPLDAAGCGSQNMMTMKMAATSGGEMLLMSGGGVDGGRFGAAADWSILDKLLASHQNLDQLFHGKVAGAHQQQQQMAMDAASSLQRLPFHHYLGLEAADLLKFSM*

## >Os02g18460

MAAAARSQGLSPGFKFNPSDQMLVELFLLPYLIDGELPVRGLVFVEDDHLGGLPLPPWILLDRHGRGDEDEAYFVAPMGAGDGARQVRSVAGGGKWVKQRSEGKGEVVVAPGGEAFLWENFSLNFHRDDRRSGSTGWVMHEYIVSPPAGSAVAASHRATHIAFTGHGQNRKRVPDGYVLVLDDAVPPAAAAAAPPPESEQSNQEEQEYAAYTDQIQQQCFVPEQQMSNQEYFPEAAAEQSNQQFFVPAEEQSSHQLFLPAEEQSNQQFFMPAEEQSSHQFLPAEEQSSHQFLPAEEQSNYQQFLPALEQMTQSNQEFAYGEQSQCYIVPEQQQLSNQEYAYSEQSQCYILPEQQQLSDQEYAYSEQSQCYILPEQQELSNQEAEYAFVCYDEQQQQQQQSNQEAEYAFACYDEQQQQQQQQYLHGDLTSWQEPFVTSSSSSSQQFLGQEQLLPDGLLLDGFGEISQQQGDQEYAYCEESQCYIMPEQQQQSNQEAEFAFACYD*

## >Os02g18470

MATAAESQGLSPGFKFNPSVEQLLCFFLLPYLQHRRLLVDGVVFLDDPASAPPWALLHRHGRGGEDEAYFIGPVPAGDGHGGRRQQQVSRTVTGGGGGKWIKQRTERPRGEEEPVVVFGGETFRWEEFSLNFHADERCRSGSTGWVMHEFAVVPPAGSRVAATHTACRIAFTGHGQKRKRVPDGYVFVDVHVQTAAAAAAVAPPLPMLSSYGEPPHEHFSDDHPPPHSYTYYTQEYQQFLPAAEQSDQEQEYCAPEQQNFQDYHVAAAEQTDQDYFYTEMINQEQDYAYQQQQQHLFHGDFLATSQQFLGQDHEVMFTGLGGGLVVSDNGEHASAAAPATEPPVHDVFLETLVPEPPENAYVDGAGESAMASASSAGGAPLLEQPFATPPQQFLDQEPAPAGLNDGGAMIYNNNGDGEHDAAPAAQPPARYYSGPVPAVDSVFLDKMREYLMADAKGLCRIDAPINNGEHAAAPAPAADDPLAAQHGHGDAPPLPVPPDAAELERVVGHLLREVEDIIKVAAAGGYGGSSDKPLSEFDKAQNQILAKLMAVFNQVAES*

## >Os02g34970

MGGATNLPPGFHFFPSDEELVVHFLRRKVSLLPCHPDIIPTLLPHRYNPWELNGKALQAGNQWYFFCHLTQSRTSSNGHWSPIGVDETVRSGGRNVGLKKTLLFSIGEPSEGIRTNWIMHEYHLLDGDCVAGGSSNLTSSSSNRRSHRKRGHSSMESNNWVLCRVFESSCGSQVSFHGEGTELSCLDEVFLSLDDYDEVSLPNK*

## >Os02g36880

MRLARQQQQVVVAATMEHDVHHHRQMMQQQQQQEMDLPPGFRFHPTDEELITHYLLRKAADPAGFAARAVGEADLNKCEPWDLPSRATMGEKEWYFFCVKDRKYPTGLRTNRATESGYWKATGKDREIFRGKALVGMKKTLVFYTGRAPRGGKTGWVMHEYRIHGKHAAANSKQDQEWVLCRVFKKSLELAPAAAAAVGRRGAGAGTDVGPSSMPMADDVVGLAPCALPPLMDVSGGGGGAGTTSLSATAGAAAAPPPAHVTCFSNALEGQFLDTPYLLPAADPADHLAMSSASPFLEALQMQYVQDAAAAGGAGMVHELLMGGGWYCNKGERERLSGASQDTGLTSSEVNPGEISSSSRQQRMDHHDASLWAY*

## >Os02g38130

MARSWLITGRGVAKKIRNAPHCSSRPISELGAEAQMECPNCKHVIDNSDVAIQWPGLPAGVKFDPSDLELLEHLEQKIGLGGSKPHTFIDEFIPTIDNDEGICYSHPENLPGMKKDGTSGHFFHRVSNAYGCGQRKRRKISNCDHVVSVEHVRWHKTGKSKAIVEKGVTKGWKKIMVLYKSSQRGAKPDKANWVMHQYHLGAEEDEKDGELVVSKISYQLHGKQIDKSETGNADEESDAFAARVGPKTPKSNTPQPCRLKNSPCETENYDPILEDQDEEESNIPIVSLKDDAGNPAWCAGETQAAREAVQACPNLDESLRCHEVLDSFYHETLLPSDRPILSQGGNEILDRNLNAVYGLPDLYNVDLGTPPDFQLADLQFGSQESIGNWLDSI*

## >Os02g41450

MRVFVLSKFIVVAICAYTNAEVAKLTASEWYFFSFRDRKYATGSRTNRATKTGYWKATGKDREVRGSSSSSSSRAVVGMRKTLVFYQGRAPNGVKTGWVMHEFRLDSPHSQPREDWVLCRVFQKRKGDGDGPQDSGGAASPTFTGSMSTTTLSQLQPPDHRRHAAAAAGGYYVGSQQLAAGYDSAAGFANPTQPAVPHYQYGGAVIGFPEEFGGGGGVADEYGFGTYLDLGFELDDTASVLGGIRSFPQGWN*

## >Os02g56600

MGLRDIELTLPPGFRFYPSDEELVCHYLHNKVVNQHRFAGVGGAAAAGGGTMVEVDLHTHEPWELPDVAKLSTNEWYFFSFRDRKYATGLRTNRATKSGYWKATGKDRVIHNPKLHAAAHRRASIVGMRKTLVFYRGRAPNGVKTNWVMHEFRMENPHTPPKEDWVLCRVFYKKKAETETESSYSMENEQEAVIAMARSAAAIKAGGCYSNSSSSHDPAAAGHHSPPPFPASLAACSSSHHYSSHPPPPPDHHHHHHMPVTGGGGGSLNEFIPTTSMALYSSIFDFSQHLDGGAVAASASAAGSRVDGGEQCGLMELGLEEHYNYNGLMPM*

## >Os02g57650

MSQSPPDSSSAAAVPLAPGFRFHPTDEELVSYYLRRRILGRRLRIDAIAEVDLYRLEPWDLPSLSRIRSRDAQWYFFARLDRKVTGAGAGGRGGPGNRTNRATPRGYWKTTGKDRDVHHRGKLVGMKKTLVFHSGRAPKGQRTNWVMHEYRLLDADGTQDLHVVCRIFQKNGSGPQNGAQYGAPYLEEDWEEEDDAIENMPASGAFAEMAAVTDTADESTEEDGNFSLKTNDEPLQTQEYQPEITPVKAQGSNEETNGGGYSCDVFSLDEILQEPENVCKNEEQNAIDDKFTIAELSGYPRQDDGYVGENGPVNWIDPSNGDNTNWPLRAYSTQNHVNGTLSADGFFDTVNGTNSYSGPSDNQNLYLQDDGLTSSHQVGDNMPFYDASSNHKWVDGKDDYLNLNDLLYPPAENQPLFDAGDDLMAYFDATEDDFKFDIMGTEDSNSQLPDMSNFVQKDDNNNKFTLDGISNTALYGASSSGSHGNMYPDTAVPDMPMDDTVDKSFGKRLASMLGSIPAPPAMASEFPPSTGKSVVPLSAVNPSSSIRVTAGIIQLGGITFTGSTEHLQKNGDFNLLLSFTVEGDVSTKSIGFEPDTQMSTTPMVLRSGMYLFFVSAMILMLSYKVGLCIYSR*

## >Os02g42970

MDTFSHVPPGFRFHPTDEELVDYYLRKKVASKKIDLDVIKDVDLYKIEPWDLQEKCKIGMEEQNDWYFFSHKDKKYPTGTRTNRATGAGFWKATGRDKPIYARSCLVGMRKTLVFYKGRAPNGQKSDWIMHEYRLETNENGTTPEEGWVVCRVFKKRVATVRRMADGSPCWFDDHGAVGAFMPDLSSPRQLLPHHHHHHPGSSAALYHGHHHQQLQQMYGHCKPELEYHHLLPQEAFLQHLPQLESPKPPPPPPAAAAYIGGHLGSSSSTALTTHDDEASGSAAQQQPPSLEAVYMAGAGVGIGVDASVTDWRLLDKFVASQLLSKESMSSYGSHPAQVFQAADGGKHEEALDYASTSAGSGGGEADLWK*

## >Os03g01870

MDGLISDDSMMMGGEVRAIESRLPPGFRFHPSDEELVGYYLRNKQQQQQQQTAATSMLVEVDLHACEPWDLPEVAKVGSDEWYFFSWRERKYATGWRRNRASKQGYWKATGKDKPILHPTVAGARKTLVFYSGRAPNGRKTAWVMHEFRLLHHHHHPNPNIQNMQQQEGDDWVLCRVFRKGNNSNGQPLATSSPPAHHLVESLISSPAPTIMSDHDRLFTIQLPHHQHCDEQYFFLDDDEQHQQQLLDLSVLQAPTSFESEQAPGHGGMEINIAEMESFDTTCAALQDASDYCMQLY*

## >Os03g02800

MAPVSLPPGFRFHPTDEELIIYYLKRKINGRQIELEIIPEVDLYKCEPWDLPEKSFLPSKDLEWYFFSPRDRKYPNGSRTNRATKAGYWKATGKDRKVNSQRRAVGMKKTLVYYRGRAPHGSRTDWVMHEYRLDERECETDTGLQDAYALCRVFKKTAPGPKIIEHYGVVHHHVEQPQWMTSSIDRSPTLDVSCDGRGDDFESSSFSFPTETPMDSMHGGFGMQMSAPHEDGKWMQFLSEDAFNATNPFLTNPVSANFSCLPSKVDVALECARLQHRLTLPPLEVEDFPQDVSLDTKIGILRSNPNEVDILQEFLSVATASQELINGSTSSYPEMWLGASTSSASYVNELSSLVEMGGVGTSNHHESARLQVEIADMEVFKDEKKRVENLRGVKLVNNDLGEIVVEGDESNPTEDIIAQYPIKVTADNSGEAGHRMTDPTDVGGIDTAPIFSQSQPDDFAAGFDDVNPNASFDLYEKVDVNHRLFVSRVAAAKTFFHRIEPSKKVSFHSNPAATAVSKATEKFHFPVTTKVSGRVSIFSKFKALIRDKFLMMRPSHSYQRLGSKETTVNELLQIVSLLLAPKQINGCPTEQELVKKKAKEVMKPGWGREGSNKLWLPLSKGKGISSMFLSGKWTFLTSALAISTPAECDH*

## >Os03g03540

MESCVPPGFRFHPTDEELVGYYLRKKVASQKIDLDVIRDIDLYRIEPWDLQEHCGIGYDEQSEWYFFSYKDRKYPTGTRTNRATMAGFWKATGRDKAVHDKSRLIGMRKTLVFYKGRAPNGQKTDWIMHEYRLETDENAPPQEEGWVVCRAFKKRTAYPARSMVETWDYSLHERNIMSAAAAAAFADPSAAYAQMRRQHRSGRFKQEAELDGAATALLHYSSHLAELPQLESPSAAAAPLQPNPSQLATAGEDDDCKGDNGGRRAKKARAAGDKVATTTDWRALDKFVASQLSPGECGSMEATAEAAAAAVAGVSSPLDHGDDDMAALLFLNSDERDEVDRWTGLLGSGAGASGVDGDLGICVFDK*

## >Os03g04070

MAAAVAVAGPSMEVEQDLPGFRFHPTEEELLDFYLSRVVLGKKLHFNIIGTLNIYRHDPWDLPGMAKIGEREWYFFVPRDRKAGNGGRPNRTTERGFWKATGSDRAIRSSGDPKRVIGLKKTLVFYQGRAPRGTKTDWVMNEYRLPDYGAARAAAPPPKEDMVLCKIYRKATPLKELEQRASAMEEMQRGSSHGDYTATRASLVHDASASTGDDYFSSDDVHDSGFLIQSSSSSAAPSGSSSKNGGAGAPREAKKEEADVTVTVASATSLQLPAVSQLPSLQLPAMDWLQDPFLTQLRSPWQDQHCLSPYAHLLYY*

## >Os03g12120

MAKDKEVGPPPPPCVDSDHEDLPLAERRRRLLRPPAESKPPAPERREASAAAAAAEDSGGAAQQGWPGLPRGVEFNPTDSDLLWHLAAEVGNGQARRHPFINEFIKSVDETIGFGYTHPQDIPGIRQDGCASYFFHKNFKECANENSKCIRWQKSGNPISITLDGNLQGCKEVFVLYAYETDGNNPQITDWRLHQYHIESTEKDEGELVVSKIFYELEKNQFKWAEKSHAQSAQGASAIDDDSKEELQLDNHSFNMITENSSVQGNENKQKQTQTGTCPNLDKLSYFNVVSNMHIGNQINDHDEIEELDHMSLQERYRILMAENHSSSAVVSSEQCAIDGLENSCKPGTNGMIPKRIHEGTAFRDGMYSMLQEISSAPAIIGSIDNDNNRRLLTEGLSNNQQSHEAGCESGFLSTSSSAAPPQCQVVCSHDLLVNGKTLIYSRDPSSSSTPTFGDKNIQLEGTDDRTLLVDIKLEPALEGDFTEKITSSVQRTDPNHGTEGSNLVGSINSVSSAISKRISEAARSNPENSHVEGLLPSSRIKSEVTGSELPLVVCGLTSISIAELTAKKTNTLNHDGVLAYCSRKRKRRKTLRDPSEKTLEEDSLRNDEGTAYFSRQRRRRKTATDSIETALEEDAPGLLQILLDKGILVKEIKLYGVEEEDDMVPDCTESDFQDLENVITKLFPQRTSLLKSALRHEKGEKAIYCLTCLISLIEQSRYLQFRDCPVEWGWCRDLQSFIFIFKSHNRIVLERPEYGYATYFFEIVKSLPIQWQIQRMVTAMKLSGCGRTALIENRPLLIGEDLTEGEARVLEEYGWVPNSGLGTMLNYRDRVVHDRWNERSGTDWKTKIGKLLMNGYSEGHLVLSHFPTKVGKIEDDTEIKQEDPL*

## >Os03g21030

MSEVSVMAEVEETAAAAPLDLPPGFRFHPTDEEIVSHYLTPKALNHRFSSGVIGDVDLNKCEPWHLPAMAKMGEKEWYFFCHKDRKYPTGTRTNRATESGYWKATGKDKEIFRGRGILVGMKKTLVFYLGRAPRGEKTGWVMHEFRLEGKLPSQLPRSAKDQWAVCKVFNKELALAAKNGPMAVTEATADDAGIERVGSFSFLSDFIDPAELPPLMDPSFVADIDGVDDAKVSASTSGQAAIAAGFHVASQVMSYQQVKMEEPLPLPYLHQQPPRMLHSGQYFSLPAVHPGDLTPSAIRRYCKAEQVSGQTSALSASRDTGLSTDPNAAGCAEISSAPTSQPFPEFDDGILGLDDFWN*

## >Os03g21060

MVLSNPAMLPPGFRFHPTDEELIVHYLRNRAASSPCPVSIIADVDIYKFDPWDLPSKENYGDREWYFFSPRDRKYPNGIRPNRAAGSGYWKATGTDKPIHSSGGAATNESVGVKKALVFYKGRPPKGTKTNWIMHEYRLAAADAHAANTYRPMKFRNTSMRLDDWVLCRIYKKSSHASPLAVPPLSDHEQDEPCALEENAPLYAPSSSSAASMILQGAAAGAFPSLHAAAAATQRTAMQKIPSISDLLNEYSLSQLFDDGGAAAAAPLQEMARQPDHHHHQQQQHALFGHPVMNHFIANNSMVQLAHLDPSSSAAASTSAGAVVEPPAVTGKRKRSSDGGEPTIQALPPAAAAAKKPNGSCVGATFQIGSALQGSSLGLSHQMLLHSNMGMN*

## >Os03g39050

MAGADGLPPGLRFDPSDDELVSRYLLRRIQQKPLPLDGVIVDADPLSVPPWTPLADHTRGDEAFFFAGARAKNGKGKRQKRTVEGGGFWQGQRMAVDGERLVVPGDGGGGVDGSGGGGLEITWRKYVLSFFAEGEQGSSGWVQKRKREPQCLDSHDDEDGGDQERAAPRRGVALPAADGTDQGSYGVIDGEPSLVSHCLPDQIVPPAEEADATAGAVDEERWSPPQPASPTAALVKQNSYDLMVISSLLFSDLPDRIDDDDLSVSQTEGTELSEQGSSGVIDDDYWREADATGGAEREEIALLDEERCPQPQPAPPTDALVPPLQGQNSYDVMADSSLLFADLPGRIDDDELQRSLRVSDMPDLFLSQTEEAGAGGGGGAAPVLNKQSNSSPLGVMDSEVPIVLSDLEFPESIDEVLSYIDFATDDCLDFDMDELFSDMPAD*

## >Os03g39100

MAGADGLLPGLRFDPSDDELVSRYLLRRIQQKPLPLDGVIVDADPLSVPPWTLLADHTRGDEAFFFAEARAKNGKGKRQKRTVEGGGFWQGQRMAVDGERLVVPGDDGGGGGDEGLEITWRKYVLSFFAEGERGSSGWVMHEYAVTSPADLASSQLRLYRVRFSGHGKKRKREPQCLDSHDDDDGGDQESATHRRAVAETTLFDGYVPRPAADGTDQGTYGVIDGESSLASHCLPDQIVPPAEEADATAGVENPLLDEERWSPPQPALVKQNSYDLMAISSLLFSDLPDRIDDDDLSVSQTEGTELSEQGSSGVIDDDYWRVFHGLSDLIALPAEEADATGGAEREEIALLDEERCPQPQPAPPTAALVPPLQGQSSYDLMADSSLLFADLPGSIDDDELQRSLRASDMPDQFLAQTEEAGAGGGGGAAAALNKQSNSSPLGVEVPMALSDLESPESMPLSDLEFPESIDEVLSYIDFTTDDTSCLDFDMDELFSDMPAD*

## >Os03g42630

MEEGLPPGFRFHPTDEELVTYYLARKVSDFGFATRAIADVDLNKCEPWDLPSKASMGEKEWYFFSMRDRKYPTGIRTNRATDSGYWKTTGKDKEIFHGGALAGMKKTLVFYRGRAPKGAKTSWVMHEYRLQSKFPYKPAKDEWVVCRVFKKLQCHLAKPRPPHDDVDGDGASPPEMVDASSLGELGELDVSSILLGGFAPPSGELCHGGGGGDGFGAHRLHVGAYMSWLQAAAAANQGMFQWPAATQAGLVGGTVFAAAHKAAGTMPFGGGCSQQQARDVGVSLANVGGGDALFGGAPLAKVDMECGEQAPQLDMDDSTWRAF*

## >Os03g56580

MVTSKEFARDQAAMDQKIKSDVGEVVLAGDEEEDGDVVLPGFRFHPTDEELVTFYLRRKVARKSLSIEIIKEMDIYKHDPWDLPNASTVGGEKEWYFFCLRGRKYRNSIRPNRVTGSGFWKATGIDRPIYSAAVNSNSGESIGLKKSLVYYRGSAGKGTKTDWMMHEFRLPPAIAAADASPCMQEAEVWTICRIFKRSITYRKQQQQQAWRPPATVTVKAPPPGDSSSNTGSFESDGGGDEFMNCGLTPAISQQQQHGGRHQMMSTMSCNGGYFFNDGIHHSHSHHKLHSQWGSLQMAPPEPKPEPEQKPLSSPAMTIAFHQNDHGFPAAAADFYKDGYLEEIARMMEVADPSPTGFYDCRY*

## >Os03g59730

MAAINPVAGEVAAATAKAPPPAMATVRAPLPANHYSPYHSASAAGSYAANTQSTSSPVSPASPAMISSSSSSLPPQQQRTWQPQPTTFSQANPGHAYQQDHLPAVAGRRFFPPPAMQMQYYHQQPDGVAMVGSGHPMAAPVHSSPLATTSGSNHAVVPDAPPQEPAKRRRRNTAAAATARRGRGRPRGATASSAHSAPPPPQQQQQPTTSAPAITAQRNDDVNQEDDNQSSKNSAEEAVVVDGGEPPAATSALAIVPRHGDVGDADRPVSPYSDIPGVRFTPTDQELIIHFLKPKYNLRDAMPTNIIVIKQLDVCKLNLDELHGDLGLGKSLDGAWYVFSPRSRYKERGVRPARGIKTTAVGYWKSNSAEADVVDDDGEVIGRVNSLTLALGHQPRGKATHWRMKEYRIPQFQIPLGQEDSNRLLTPMLSQDIVLFYFLQLDEWVLCKLYHSFAYKQKGKCKVHEEGSKSDRGVQDLSIDDDRKTCDIEANKPNGV*

## >Os06g33940

MHPSGGALSVPPGFRFHPTDEELLYYYLRKKVAYEAIDLDVIREIDLNKLEPWDLKDRCRIGTGPQNEWYFFSHKDKKYPTGTRTNRATTAGFWKATGRDKAIFLANACRIGMRKTLVFYVGRAPHGKKTDWIMHEYRLDQDNVDVQEDGWVVCRVFMKKSYQRGLNPADMAAVDDDDLLHHHHHPFPPAQLHGGAADHKHDGAGGHHHHHLMQPHHHYDDFPSFDPSMQLPQLMSADQPPPPPPSLLPGVPPSAAAALSSLDVECPQNLMKLTSAAAGGGATGLLHAGGDHRFATAATDWSILDKLLASHQNLDQLFQGRVIAGASSPAAMAAPSHHQHLMDQLAGGGGGTASSLQRLPLQYLGCEAADLLRFSK*

## >Os06g23650

MERCSVLGLGGGGGGGGRLDGELPPGFRFHPTDEELITYYLLRKVVDGSFNGRAIAEIDLNKCEPWELPEKAKMGEKEWYFYSLRDRKYPTGLRTNRATGAGYWKATGKDREIRSARTGALVGMKKTLVFYRGRAPKGQKTQWVMHEYRLDGTYAYHFLSSSTRDEWVIARIFTKPGVFPVVRKGRLGISGGGGDTSCFSDSTSASVGGGGGTSASSALRAPLAEASLFAAAAAPAVDGADSSNYGGGGGAGSATATANLVTGLELVPCFSTTAHMDASFGTGQYNPAPLAVEPPPPPPAFFPSLRSLQENLQLPLFLSGGMQAGVSSQPLSGGGAFHWQSGMDVKVEGAVGRAPPQMAVGPGQLDGAFAWGF*

## >Os06g04090

MSISVNGQSVVPPGFRFHPTEEELLTYYLKKKVASERIDLDVIRDVDLNKLEPWDIQERCRIGSGPQNDWYFFSHKDKKYPTGTRTNRATAAGFWKATGRDKAIYSSSNRIGMRKTLVFYKGRAPHGQKSDWIMHEYRLDDPSSASASVSVNLPSYYSSSSSSSSPMHGVAGDQGAQEEGWVICRVFKKKNLVHHGGGAAAASHHAAAKLAAAAMEGSPSNCSTVTVSDHVKAQMLHSSASDDALDHILQYMGRSGCKQETKPAAMSASSAAAAAALEQHLSTPQYGKFMKLPPLEHVAGGVGLLAAAGGGGEYCSAADASGIADWDTLDRLAASYELNGALSDVASGKNMAGFFDVVDQPAGAAAFSSGDGDLWSLARSVSSSLHADLTTMNNV*

## >Os06g01480

MDGSSMSSSSTQQQAQVPPGFRFHPTDEELVDYYLRKKVAARRIDLNVIKDVDLYKIEPWDLQERCRINGGSAAEEQNEWYFFSHKDKKYPTGTRTNRATAAGFWKATGRDKPIYATKQHSLLVGMRKTLVYYRGRAPNGHKSDWIMHEYRLETTETAPPQEEGWVVCRVFKKRLPTTRRDSDHDAPCGSWYVDEDAPGAFMSPMMITRSSILRPHQHHAGITLQEQHLHTTYKHRDLTTKIQQLQVPAAGHHLLNTMPHDLESSTSSFHSLLVSPDHHQINMHHAQADPFFDDMHAVDQATTTDWRVLDKFVASQLSNDATNKPADHYTDEGDILQVSDKQQEVAAADYASTSTSSSQIDPWK*

## >Os06g01230

MKQGKVEMTPAVAAAAVLPVGFRFRPTDEELVRHYLKGKIAGRSHPDLLLIPDVDLSTCEPWDLPAMSVIKSDDPEWFFFAPRDRKYPGGHRSNRSTAAGYWKATGKDRLIRSRPAGPLIGIKKTLVFHRGRAPRGLRTAWIMHEYRTTEPHFQSGKNGSFVLYRLFNKHEQDDTHTPASNLDQQLSTSSQGNPQNGTPAVQPALASIMKDHQTLPSSGFSQLTEIQDASTSVHDKEQTVAHDDAFLDVLSQLPDLEPEQRYNGFPNITSPIRPYSDHPFVGNLGEQDLSAHFGSTLSEQDLQSLLFSPNYTKMDKHPTGNVESNPTASSNNPNNNTLLMDSWRKNDSYQMLLIQTTQMLLAVLLQSMHHKQKQVMLILKQEPRVAAWSTVE*

## >Os05g48850

MTWCNSFSDVRTAVDSSLSPAAAVAAAAGKKAAASLAVLVKMCPSCGHRARYEQETTTIQDLPGLPAGVKFDPTDQELLEHLEGKARPDSRKLHPLVDEFIPTIEGENGICYTHPERLPGVSKDGLVRHFFHRPSKAYTTGTRKRRKVHSDEVDGGETRWHKTGKTRPVMANGRPRGYKKILVLYTNYGKQRKPEKTNWVMHQYHLGSDEEERDGELVVSKVFFQTQPRQCGSTAAAAAAKEASAAVAAAVVNSNYSIVHGHQGGGGGSFLKEANVVHEFYDPAATMGYRPPAPAAHFAPNFAVHAARNSFGGP*

## >Os05g43960

MAGLGKGESSGGGGGGGGEMGFRFKPREAEAVEYYLLPRLQGRPPVPNPAIVVENVYEFEPERLINEKCNGGVAGEGEEGWYFLSPRDRKYRNGKRPSRSTEDKAGRWKASTGKTEGKDPITECYGWVKFCVTSLVYFKGPVKTEKKTKWLMREFTIPHFENKLDKTAAAGGSSNQRQLDQYVLCRIYTSPKKGADDGEQAEVVRGGGGGGEDIDEWAEACAVFDLGPETAEGSDNADAAAAEGDMRSAKQAGKRPVAAAAVAEQPSKRPWLPPSPSTPCDGGPSQAMGNRQVPMQGLSLMHNFPPPPTTFCGHAPFQQGFPVHNNRAQMRWPTMQHNCMPSPAHSFQPRPVQRRPVLVGQAPPQRRPVHHVGGHAPMHMHQAQWTPVHIAQAPMQQLPFDDWVFDPFDDPPPPMQQLPVMMNNYQPQAPMQLPPMMNNDQPAMVHGGELQAPMQLLPATTHGGEVQAPMPLNVYEEEQRPSQEDGGQCTNAEG*

## >Os05g35170

MAQTCLPPGFRFHPTDVELVSYYLKRKIMGKKPLIQAISDVELYKFAPWDLPAQSCLQSRDLEWFFFCPRDKKYPNGSRTNRSTPNGYWKTSGKDRTIELNSRIVGSKKTLIFHEGKAPKGNRTDWVMYEYKMEDNQLVSAGFSKDDFVLCKIFKKSGLGPRIGEQYGAPFNEEEWEHADAEMFPLLPNVETSVFPLLPSSEVVNSTDDTRVQPSVAARAIEELPVQHLPHVCAGNGSTYQNITVTGESALMELPSQHSVESIGDEVVSVDNCSNVVNNADSPVIEGLVLEELSRFLTDSPHHGNPVGEHSGLPPMSEAEAHAFEVSTNDLYNEIAGLAELGVPNGDGFSPSNAGVTEQQPTYFGVPNSENYVNMDDIFAPDTRLSYAYPLPNNQFWHYPMDQFTYSTTLSAAFPSGDSRPTMRIVDDLPAAANNGGFASKPSMQFPLS*

## >Os05g34830

MSGGGEGAAAAERQELQLPPGFRFHPTDEELVMHYLCRRCAGLPIAVPIIAEVDLYKFDPWHLPRMALYGEKEWYFFSPRDRKYPNGSRPNRAAGSGYWKATGADKPVGTPRPVAIKKALVFYAGKAPKGDKTNWIMHEYRLADVDRSARKKNTLRLDDWVLCRIYNKKGGVEKPSGGGGGERSNMMSHGETASAGSPPEQKPAVLPPPPPPYAAAAPFSELAAFYDVRPSDSVPRAHGADSSCSEHVLTTSASSGGVVERPEVQSQPKIAEWERTFAGAAAPAGAVSTAGPILGQLDPAAAVAGGGDPLLQDILMYWGKPF*

## >Os05g34600

MSRDDVDDVTAGAAGSGEEAAADQEEAAAAVAGDSHENDLVMPGFRFHPTEEELIEFYLRRKVEGRRFNVELITFLDLYRFDPWELPAMAVIGEKEWFFYVPRDRKYRNGDRPNRVTASGYWKATGADRMIRGENSRPIGLKKTLVFYSGKAPKGVRSSWIMNEYRLPPPAADADLFYKSEISLCRVYKRSGIDDGHGHHQRPAGNVQASSSSAAARPPEQHSGNNTAAGLPACRHRPSPSSSSTTTAQQHTSFHQLLQGECSAAAAAPPPPPSLPASATTRNSNASQLLMPPPPPRPPCAAAYTSAAAAPTESAAVLAAASTYSLLAAAGSSSTHIDELSTLLAGHSHGGAYGNNHIVAGSHHHFPLPPSQLMPQLGTLPISPPLAAVSDKLWDWSSVPDTSTARDYDSSGFSDPK*

## >Os05g34310

MAMQLSLPVLPTGFRFHPTDEELVINYLQRRATGLSCPIPIIADVEIYNFNPWELPSMALFGEHEWYFFTLRDHRYPNSVRPSRSAASGFWKATGTDKPVQVANMQSTPVAMKKALVFYVGRPPMETKTTWIMHEYRLTNTGGSTASHPSLSSSTAHPSVKLDEWVLCKIFNKSPEPDNTAPPSNVVSRLQCSPPLPPPAAPPGNYPPLPVGATNDGGVFAGAGDMLFTIQEHQEGTPSMLPPIPNLEPPAATIGNSSLNGTAAAAAAADGHGRLEEEDTSAYTFTDQEMEQMLMDLMDQDFFGNDQPQE*

## >Os05g25960

MAAADGLLPGLKFDPSDHDLVGRYLLRRLQGQPLPLDGVILEADPLSAPPWKLLADHGRGDEAFFFAEAHAKNGKGKRQKRTVEGGGFWQGQNTCVDGERLCVPDDGDGSGGGGGGLEIAWRKYVLSFFANGERGSSGWVMHEYAVTAPDGLASSQLRLYRVRFSGYGKKRKREPQCPGAHGDDDGELQCAPPPRSMAETALLEERGPLPHPVLGPASVVDQCTDQGSSGVIDDSSLVFRDLPDLIDLPVAEEADASHGAETALLNEHLPLPPPQLFVPPTAVPLDLADDSNGADQNSYGMMGDDQLLLPDLPGTINDDMPDLFVSQAEEASAVPAISYHSSGFMGNEVAALSDFELPESYSSSDAMDGEALALSNYEFPESFEEDLSCIDFATANASSLGFPMDGYPMDELFDDMPDQGSSGAMDDSSVVFRDLPGLINLPAAEEADAIGDAETALLRDLADDSNGTDRNSYGVMGDDQDRLLLPEIPRRIDMPDLFVSQAEEAGLGGGAALDSSSGAMDGEALALSDFEFPESVEEVLSCMDFSTVDMSFLDVPIDELLDDLPAD*

## >Os05g10620

MGAGSIQARMSSDGASGSIGMKHDDGDHRPSTGASSRRCPSCGHDPDCNKPFDMVGMPAGVRFDPTDQELIEHLEAKVKDGGSTSHPLIDEFIHTIQGEDGICYTHPENLPGVTRDGLSKHFFHRSAKAYPTGTRKRRKVLADQQPDDHPQASKGRNVAAAETRWHKTGKTREITVRGQPKGCKKILVLYTSFGKKRKAEKTSWVMHQYHLGELDDEKEGELILSKVFYQTQTRSAAAAEAPVSSGAAMEVQGQQQQVLKLQADDGHFSSAPTKKRLHQDVVAQVKVDRGHHCMPAQRQVNFNLKVTPVPTTSSFPVVVDKQLYSPVALFRSEHLHVGKNFNSSAPKSRLASPALAS*

## >Os04g59470

MEEEWCCVAPGFRFHPTEEELVGYYLARKVVGQQDDGIIQEVDLNSIEPWDLLQAQQHDQEYYCYFFSYKDRKYPSARGTGTRTNRATAAGFWKATGRDKPVLSSSRSSSSPAVIGMRKTLVFYRGRAPNGCKTDWIIHEYRLVAHHQQPDGSCWVVCRAFHKPTTTTLQHQLHLHRPAPLLHHPGYYDDQYLYPPPAAAAGGGGLLCSPALDMELEDEEDESKMMILSNDNIPLVVSPTAVHTQGTGGDIINDATTAPAAAAAADHRRHLAPPPPLHLLANSVD*

## >Os04g52810

MVFFTCHESLAYFLHNLIIFPARARARAAGRRRREDAARVGATEWYFFSLHDRKYATGQRTNRATRSGYWKATGKDRAIVTRRRAAAGEAVAGGEVVGMRKTLVFYQGRAPRGSKTEWVMHEFRVDGHAVADHPSSSTSSSSSNLLKEDWVLCRVFYKSRTATPRAVVSGEAAVSLSGELSLPPPLPPPVAPAVVDGYTGGGYYEQDSSAGYHHHHHHRPPPSAALPFKDLTDFRDLLSNMAQGGGGGAAAAKTEGFHLGWSEEESSGYVQQSAMASQAWNPF*

## >Os04g43560

MGLREIESTLPPGFRFYPSDEELVCHYLYKKVSNERASQGTLVEVDLHAREPWELPDVAKLTASEWYFFSFRDRKYATGSRTNRATKTGYWKATGKDREVRSPATRAVVGMRKTLVFYQGRAPNGVKSGWVMHEFRLDSPHSPPKEDWVLCRVFQKSKGDGEQDNPTSAASPAATFAGSSQAAVPGQAAYSSDDHTGSSMGFAPRQNEILDSSSHQLLNLAMLQCNSVLDHFPQEVNSSPMMGLAGSIGIGDEYGFFYDTGFEETASLGGMRFPQGWS*

## >Os04g38720

MEQHQGQAGMDLPPGFRFHPTDEELITHYLAKKVADARFAALAVAEADLNKCEPWDLPSLAKMGEKEWYFFCLKDRKYPTGLRTNRATESGYWKATGKDKDIFRRKALVGMKKTLVFYTGRAPKGEKSGWVMHEYRLHGKLHAAALGFLHGKPASSKNEWVLCRVFKKSLVEVGAAGGKKAAVVTMEMARGGSTSSSVADEIAMSSVVLPPLMDMSGAGAGAVDPATTAHVTCFSNALEGQFFNPTAVHGHGGGDSSPFMASFTQYGQLHHGVSLVQLLESCNGYGGLVDMAASGSQLQPAACGGERERLSASQDTGLTSDVNPEISSSSGQKFDHEAALWGY*

## >Os04g35660

MAGASNLPPGFHFFPSDEELIIHFLRRKASLLPCQPDIVPTLILNLYDPWELNGKALQSGNQWYFFSHATQTRTSPNGHWKPIADETVISGGCNVGLKKTLIFFIGEPFEAIKTNWVMHEYHLMDGSTNCSSSSTSSSSSKRSHKKKGHSDTESKNWVICRVFESSYDSQVSFHEEGTELSCLDEVFLSLDDYDEVSFAK*

## >Os03g62470

MDDDEIALEPGYAFRPSDDGLITLFLRPKIAKIPFEHRLINHADVYSADPTELVGEHRPAPGTHGSGSVWYFFCSPRYTSKRKASGRRQRAVGGESVWKSEGGKKAVIGADGRRVGYLQKFSYHYTGQGHL*

## >Os06g36480

MDGVVAEDQAGGSGSGHRRLIGSRIEEHRKYMSEESCCPRCGHKIDRKLDWVGLPAGVKFDPTDQELIEHLEAKVRPGGEAAAHPLIDEFIPTIEGEDGICYTHPEKLPGVSKDGLSRHFFHRPSKAYTTGTRKRRKIQPPAAAASSGGGGGNASSSSSASAAAAVARHGHQQQQQQQRSETRWHKTGKTRAVVGGGRQRGCKKILVLYTNFGKHRKPEKTNWVMHQYHLGEAEEERDGELVVSKIFYQTQPRQCAAADAAATASASAVDRRTTSLRDRAAAAAAAAAAAAPMASANVSVAAFHGGAAGIDEFSFAQFRSSFEEAGMGASSSDHQSAMVDQRRRQQQHDDDEHDHRRGGGGHHYVGQQQSVAATFHVVSSPADPIARLMSPPPAHQGTVMLRQPEPPYIYHHQEDERPHQPRKFDGRSTSGSGLEEVIMGCTSRRSKGGETSGGKDGTEWQYPSFWPSDSQDHHGDKDDIEAKQKGETEKEKGEEKQSFSRYTFAKKYSMNKETLKISSILHSGILSESKLPENFPVCVVEGEYDEHGNAWMNLILPFSDSSKLRLM*

## >Os06g46270

MSGMNSLSMVEARLPPGFRFHPRDDELVLDYLERKLLDGGVGGAAAAAAAVTIYGCPVMVDVDLNKCEPWDLPEIACVGGKEWYFYSLRDRKYATGQRTNRATESGYWKATGKDRPISRKGLLVGMRKTLVFYKGRAPKGKKTEWVMHEFRKEGQGDPMKLPLKEDWVLCRVFYKSRTTIAKLPTEGSYNNIDSVATTSLPPLTDNYIAFDQPGSMQNLEGYEQVPCFSNNPSQQPSSSMNVPLTSAMVDQEQNNMGRAIKDVLSQFTKFEGNVKREALQSNFSQDGFDYLAESGFTQMWNSLS*

## >Os06g51070

MEQQRSRSTAAGGEVEVEQLPGFRFHPTEEELLEFYLKQVVQGKKLKFDIIPTVHLYRHDPRELPGLARIGEREWYFFVPRDRKQATGGGGGGRPSRTTERGFWKATGSDRAIRCAADPKRLIGLKKTLVYYEGRAPRGTKTDWVMNEYRLPDAAAIPDTMQLQMQHDDMVLCKVYRKAVSLKELEQRVAMEELARSTTSSGTHNTGSPLQQDSSSISISSSSDAMKKEVVGVDEASAAAHELVRPATLSLPQLEVARPQSGLEWMQEPFLTQLRSPWMETWSPYYASVLNF*

## >Os07g04560

MKRGCEDELGAGDVILRGVEEVEEEDDDDLVLPGFRFHPTDEELVTFYLRRKIAEKRLSIEIIKEMDIYKHDPSDFLKTSTVGSEKEWYFFCLRGRKYRNSIRPNRVTGSGFWKATGIDRPICSAAGGGGGDCIGLKKSLVYYRGSAGKGTKTDWMMHEFRLPPPPADDLAAGRSSPPPSLQEAEVWTICRIFQRNITHKKQPQPQLAVAAAAVPAPVPDATSSITGSLESDSAGDDVVEYMNTLQPPPASNVNGGYSNQRYFQEQWNSSSNDNTTVFHQHAAAAPPPEPSPATAMAGFGHDQSVLSSPAPSDFYYKDGCNDDIYRMVMELADPSLFYDHIYA*

## >Os07g09740

MASAAGDGLPPGLKFEPKDDELVARFLLARIQGKPLPLHGVILDADPLCAPPWRLLADHGRGDEAFFFADARAKNGKGSRQKRTVEGGGYWQGQRMCVDGERLVVPDGGLEIAWRKYVLSYFADGEKGSSGWVMHEYAITTPADLASSTMRLYRIRFSGHGKKRKREPESQSAHHDDGRARCAPQIAMPETALLEDSAPPPQPVLPPAAVVNSVSDGAVPPPAPVVNCDSDVTDEDELQSFVPEFSARNLFVSLPQGSHEAEADVVGGALPAQSMSSFADVGGPENMDDQSCSGVVFANLSDLIVLPPVEASGAAPAPSWASSLDNQNDEAPVFFEFPESMDDIIGCFDFATMDDPSCTSAISEEPFLPPAAMVNHDDGYASDNADQGCSGAVPLPSAVVDLPNETDGADQSCSGVVDDSSMVFANIHPLDSPAEGGHEAEAGAGGGRAAPAPSWVSSLDNQNDEAPMFFELPESLDDMVSCFDFAAMDGQSCTSAVSETALIEELVLPPAAMVNHHDDSVSDIADHGCSGAAPPPSAVVDLPDDSDGADQSCSGMVDDSLPGYYEAELKVPLEYAARNPVDSPSKGGHDHEAEVDASGGAGSMMSSPDKEKEHSSSGVMDVEATGFGVPDSMDGLSCIDFAETMDDLSCIDFTIDDELFDLWS*

## >Os07g09830

MAAAAGDGLPPGLRFEPKDDELVARFLLARIQGKPLPLHGVILDADPLCAPPWRLLADHGRGDDAFFFAEARAKNGKGSRQKRTVEGGGYWQGQRMCVDGERLVVPDGGGGGGVEIAWRKYVLSYFADGEKGSSGWVMHEYAITAPADLASSTTRLYRIRFSGHGKKRKREPESQSDNHQIAVAETAMLEDSAPPPQPVHPPAAMVNCVSDDTDQGFSGAVPPPAPVVHHTNDSDVTDRYSSLVFSDQPGSIYEDELQSFVPEFPARNLFVSLPEGSHEAEPAQSVSSLADVGGPENMDDQSCSGVVFANLPELIVLPSAPSLDNQNDEAPVFFEFPESMDDIVGCFDFAAMDNQSCTSAIPEEPFLPPAAMVNHDDGYASNNADQGCSGAFPLPAAVVDLPNETDGADQSCSGVGDNSTLLFSDVTGSIDEDELQSFVPEFAASNLFVSLPQGSCEADAEADSGGGVAPAQFAEFGGSESMDDSSMMYPLNFPAEAGGGGGRAAPASSWVSSQHNQNDEAPMFFELPESLDDMVGCFDFAAMDGQSCTSAVSETALIEELVLPPAAMVNHHDDSVSDIADHGCSGSVPPPSAVVDLPNDSDQSCSGYYEAELKVPLEYAARNPVDSPAKGGNEAEVDASNGAGSMMSSPDKQKEHSSSGVMDVEAIGFGVPDSMDSLSCIDFAETMDDLSCIDFTIDDELFDLWS*

## >Os07g09860

MAAAAGDGLPPGLRFEPKDDELVARFLLARIQGKPLPLHGVILDADPLCAPPWRLLADHGRGDDAFFFAEARAKNGKGSRQKRTVEGGGYWQGQRMCVDGERLVVPDGGGGGGVEIAWRKYVLSYFADGEKGSSGWVMHEYAITSPADLASSAMRLYRIRFSGHGKKRKREPDSQSAHDEHGRARCAPQIAMPETALLEDSAPPPQPVHPPAAVVDCVCDVTDQGSSLVFPDQPGSIYEDELQSFVPEFAARNLFVSLPEGSRDVVAEAALIEDLALSPQPVPPPAEVVNQADDSDGADQGCSSVFAALPDLIVLPPEEACGSGGAAPAPSWASSLDNQNDDAPAFFEFPESMDDMVGCFDFASMDNQSCTSAVSEIAVLEEPFLPPPTMVNHDNNSVSDGADQSCFGVGDNSTLVFSDLTGSIDEDELQSFVPEFVSLPQGSCEADAEADSGGGVAPAQFAEFGGPESMDDPLNFPAEASGGGDRAAPASSWVSSQDNQNDEAPMFFELPESLDDMVGCFDFAAMDGQSCTSAVSETALIEELVLPPAAMVNHHDDSVSDIADHGCSGAVPPPNSEVVDLPNDSVGADQSCSGMVDDSLSGYYEAELKDASGGAGSMMSSPDKQKEHSSSGVMDVEATGFGVPDSMDGLSCIDFAETMDDLSCIDFTIDDELFDLWS*

## >Os03g61319

MADDDEIALEPGYVFHPSDDGLITLFLRPSIAKIPFEDRLINHADVYSANPAELVGEHRPAPGTHGSSSVWYFFCSPRFTSKRKTSGRRQRAVGGGGGGESVWKSEGGKKAVIGADGRRVGYLQKFSYGVYESSSSGSARTFTRLGWCMTEYGLDDDAIDGADKQVLCKVYRSPRAVCAEARTAAAAKSADSPCSGSKRKADDGADHPEAPPSARPRQEEAGSEQPAILPELDLDALLSAPMDDSLGVEFDTATTEQYMRYLMNDEPLPWAPTMEVAGGGDEFIETTNGPCMGEEEIIQRLASGETLDDILGSNPN*

## >Os03g61249

MADDDEIALEPGYVFHPSDDGLITLFLRPSIAKIPFEDRLINHADVYSANPAELVGEHRPAPGTHGSSSVWYFFCSPRFTSKRKTSGRRQRAVGGGGGGESVWKSEGGKKAVIGADGRRVGYLQKFSYGVYESSSSGSARTFTRLGWCMTEYGLDDDAIDGADKQVLCKVYRSPRAVCAEARTAAAAKSADSPCSGSKRKADDGADHPEAPPSARPRQEEAGSEQPAILPELDLDALLSAPMDDSLGVEFDTATTEQYMRYLMNDEPLPWAPTMEVAGGGDEFIETTNGPCMGEEEIIQRLASGETLDDILGSNPN*

## >Os03g60080

MGMGMRRERDAEAELNLPPGFRFHPTDDELVEHYLCRKAAGQRLPVPIIAEVDLYKFDPWDLPERALFGAREWYFFTPRDRKYPNGSRPNRAAGNGYWKATGADKPVAPRGRTLGIKKALVFYAGKAPRGVKTDWIMHEYRLADAGRAAAGAKKGSLRLDDWVLCRLYNKKNEWEKMQQGKEVKEEASDMVTSQSHSHTHSWGETRTPESEIVDNDPFPELDSFPAFQPAPPPATAMMVPKKESMDDATAAAAAAATIPRNNSSLFVDLSYDDIQGMYSGLDMLPPGDDFYSSLFASPRVKGTTPRAGAGMGMVPF*

## >Os12g43530

MEEEKRLERIIKEIDSPISPGGGAALLAEDDDLVFPGFRFHPTDQELVGFYLTRKVEKKPFSIDIIKEIDIYKHDPWDLPKVSHGAVALQGSSSSSSLSTAAAAEKECGYFFCLRGRKYRNSIRPNRVTGSGFWKATGIDKPIYSSSLAAAAAAAGAGDCIGLKKSLVYYRGSAGKGTKTDWMMHEFRLPSSISDSDHLQDASETWTICRIFKRSMTYTKGRAAAAAASMNKRISHELQHIHHHQQQQFYYHEVVHDGHGHHHRRHLQHYAGSASMAAAAANIVDVIDHSSDAETTTRSHSHSQSHLVADIRHRQSPFMLDFHAGTASSSSSTAAGWSEVMSFSRDGGSSSGSSWDELGRIMDISTNSANNNYYL*

## >Os12g22940

MEKGKLDFLLELGFRFNPSPEQVVTYYLPCLVAGQQPKDTEGCIHSADVYGADEPRDLAGKYAPVARSSNGDRFFFTGCKRMKGKFSRSAGGGTWVSQSSKDLKNREGIKIGEVKNFRFKKDGKNTDWLMEEYHLCGQESGDVVEPVVCRIYVSPRAAPDSVAHQESAVLQPQEPAPLPVPAAPAPPRQVPVVTQQAPPPPPPLVPVITQDAPPLKRPAPVAAPPCAKKMRGDVSAFPVVRQSCVAAPRCAPRVVAPPPRHPPIQTYPTDPFESAPLDPFEPPPAAASVTGGHHTPQPSVPVPATPEQGFSLAASNSPELDPANIGIDMDELMRYLGNTPLDGVLPSQLFVLPTNDDEDVELAKVLEDGLQGGGGRQWQSTAVCDSSSSATGILARHGATAASSTHPDLSQGSVRAQ*

## >Os12g23090

MASLDLLLKLGFRFNPSQEEVITYYLPRLIAGHPPKDTEGYIHRADVYGADEPRDLAGKYAPVARSPNGDRFFFTGCKRVKGKFSRSAGGGTWVSQSSKDLKNREGIKIGEVKNFRFKKGGNNTDWLMEEYHLCGKEAGGVVEPVVCRIYVSPRAAPDSVAHQESAALPPPQELVPPPQELAPPPYPAAQAAPQAPAPPRQVPVITQQQAPPQKRPAAPVAEPPCATKKMKGAVSAKPMAPQSSVTASAAPPRCAVAPSQHHPPFQTYPTDPFEPPAPAASVTQPSVPATPEQGPAYVPDPADIGMEMDELMSFLDSIPVDGILPSQLYEYDELAKELEDALQGGGEEDGNDNPPRRRGRGGCDKQSQGGYRVLLKDMGDDQIDQQWLKVSLKDYHHLMKSCKL*

## >Os12g41680

MSMMSFLSMVEAELPPGFRFHPRDDELICDYLAPKVAGKVGFSGRRPPMVDVDLNKVEPWDLPEVASVGGKEWYFFSLRDRKYATGQRTNRATVSGYWKATGKDRVVARRGALVGMRKTLVFYQGRAPKGRKTEWVMHEYRMEGVHDQQASSFSSKEDWVLCRVICKRKSGGGATSKSRSLTTTTTTIVHDTSTPTSSPPLPPLMDTTLAQLQASMNTSSSSAIAAVAALEQVPCFSSFSNSIASNNNNSNSATVNAQQCYLPIVTGSNNNGMSYLDHGLPEFGSFLDTQSCDKKMLKAVLSQLNSIGGEVLPGLPPPSEMAAAVSSSWMNHF*

## >Os12g29330

METTAAKKLPPGFRFRPTDEELVVHYLRRRALGSPLPPAVDIPDVRLLAHDPSDLLPPGWSEQERYFFTCKEAKYVKGRRANRATGAGYWKATGKEKPVAVSVAAAPRSQAAAVVVGMKRSLVFYRGKPPTGKKTDWVMHEYRLAGAGLAPCRRAATADHPARPAEGWVLCRVFRKKGSAAASTASPTADADDDDATTERADDAAAGVRFIDFFARADARRRRAASPVSSSCVTDASAEHCREQETTSRNGGAAAGDASD*

## >Os12g05990

MVMSGGGGGARIVSDPAATPGFRFYPTEEELIGFYLRHRLAGTRADDVARVIPVVDVYGYHPSQLAAMAGVATAGDREQWFFFCPRAERELHGGRPARTTPSGYWKATGSPSFVFSSSAAAAARVIGVKRTMVFYQGRAPSGTKTRWKMNEYKAVAAAAADDDHNAAGVAVQLPPMAPPPSSSACVRLRNELSVCRVYVSTGTLRSFDRRPLDAPPVISHHQPQLQQQQRQLPSSAAAAATNGNLIALAGGYECSHDSSGGSSEDAAIDWSSLITAATDSATAAVDFSFNDDIDFSPAAVGPWAPQL*

## >Os12g03050

MVESTTSLVKLEQDGGLFLPPGFRFHPTDAEVILSYLLQKLLNPSFTSLPIGEVDLNKCEPWDLPSKAKMGEKEWYFFSHKDMKYPTGMRTNRATKEGYWKATGKDREIFRQPAAVNTSSYGGSSNKKKQLVGMKKTLVFYMGRAPKGTKTNWVMHEFRLHANLHNHHPNLRLNPKDEWVVCKVFHKKQGDEAINNQQQQPQYAAVDQYSAETPNSGSSVVQAGDIDGGDDFFQLDDIIDPSIYFVSNSSNILSAPPNNNNAVYSVSASTTTTNTTAVSFQQQPNYYSLINKSSSSSSSNYSAPLQQHVSSWNITPGAGGAHGIGSSYYNLQQQQAAMVKALENVIAVPNFGTLLPSSNKLKGLSKSAMAGLTQQNPLGVPQYKIENYGDHYISRQ*

## >Os12g03040

MPSSGGAMPALPPGFRFHPTDEELIVHYLMNQAASIKCPVPIIAEVNIYKCNPWDLPGKALFGENEWYFFSPRDRKYPNGARPNRAAGSGYWKATGTDKSILSTPTSDNIGVKKALVFYKGKPPKGVKTDWIMHEYRLTGTSANNTTTTKQRRASSMTMRLDDWVLCRIHKKSNDFNSSDQHDQEPEGSTVDEQLEDIHDNNSSSQQPPAPPDMNNQQSDFQPMTAMSMSKSCSLTDLLNNLDCAALSQFLLDGSSDAIAELPAPPSPLIYPNQTLNYNINNNMPHAFESRLDHHDGYVNNYNVNGLRRKRMMACSATSFDDGSSSSSSDFLHVAKKPLLLPSDSRGSGFGGGYCNQQLSETATGFQFQNGNMLSHPFPLNQQLLLNNHLQMQ*

## >Os11g31380

MHLPAVGMSHPTEGELVFHYLYRRAVNMPLPSEFICDVNVLPHNPWDIVPGSEKPVYYNQGGGSDCMLVGMRRTLTFYFGNSRTAERTKWGMQEFRLAGNGLSPYPAMKHATGDGSKPPCNCAETTIAKRNDGLSAVLRNVLAVTPLVETVVEPDGSWLICRIYRTRQRALPVITPPAIENAREIIIPPANGNAREAQVRFIDFLQQGSHIESSSPCSCIVGPSLAEGSDESAGSVDQKD*

## >Os11g31360

MAGPGVCINLLNGTTMHLSVGCVFRPTEGELVVNYLYRRAMQEPLPCDFITDVDIQCHNPWEIVPAGEKKNGKHFFTRKENSHPRDYESNHAAGDGFWRLAGTEVPIYNKPSGGADEKLVGMKRTLVFHFRKSSSTERTGWVMPTCRCQPCALPCDEAGHRCHLYHGCTLIDYMRTVLQKNNGSPSAAHTHAPLVETMVEPDNSWMICRIYKKRQRAPQVIIPPSIGNAREAVLAVPAIGNAGDRQVTSLTSQGIDVSRRGVMSLPMSSQRTRAVMVMGRTNRRKKLGLV*

## >Os11g31340

MASPGVCINLVNGTSTRLPTNADLVVHYLHRRAIQEPVPCDFITNVDILQHNPWDIVPAEEKTNGKYFFIHEENERLGNHHSNRAAGDGFWRPVGSEVPIYHKRSGGADEALVGMKRTLVFHYGNSSSAKRTEWVMQEFRLAGATLIPCPVTRPATGDGSMLPCHRTGTTIATENNGSPSAGQTHGPLEKTMVEPDSSLRICRIYKKRQRTPQFIIPPSIGDARELILALPTIGNTREVALALPAIDFLGQPSFEEGSDVSADVITDDKDGYGHGMN*

## >Os11g31330

MANTGLSIPMVNGATIHLLPGFRFRPTDDELVIKYLYPRAFHVPLPCAIITDVDIHHHNPWDIVPVAEREKGKHFFTRKEVKYPGSRRSNRVAGNGFWRAAGSEVPIYYKPEGAANDMLVGMRRTLVFHYGKSRSAERTEWAMHEFQLAGAGLLPHPMMRHATSNGSEPPCGCLEATIAKKSDGLSATLRAKRDSAPLMRIMVEPDSSWVICCIYKKRQRAPPVVIPPVIGDVGEAIIPHAIGDAREGQLHFIDFLGQPARNDPSSPHSCTIDPSSLEEGSDESAGDGEDKDGDGMNEAN*

## >Os11g08210

MECGGALQLPPGFRFHPTDDELVMYYLCRKCGGLPLAAPVIAEVDLYKFNPWDLPERAMGGEKEWYFFSPRDRKYPNGQRPNRAAGTGYWKATGADKPVGSPRAVAIKKALVFYAGKPPKGVKTNWIMHEYRLADVDRSAAARKLSKSSHNALRLDDWVLCRIYNKKGVIERYDTVDAGEDVKPAAAAAAAKGGRIGGGGGAAAMKVELSDYGFYDQEPESEMLCFDRSGSADRDSMPRLHTDSSGSEHVLSPSPSPDDFPGGGDHDYAESQPSGGCGGWPGVDWAAVGDDGFVIDSSLFELPSPAAFSRAAGDGAAFGDMFTYLQKPF*

## >Os11g07700

MASTSRKRSARSLQEDEQTSSAAEAPAAVREDEERVAVAGMEAWRFGFSRFSWFPAFKFDPTDADIVASYLLPRALYGRGHAAVIQDDVSRCEPWTLMREHGHATSAHAFFVHDHESVGGGGGGGRRKVQRAVKNGGGVWRIQKGEVAILTIVRGGGGGGGELDVVYKRRNLSFHRRGESSSSGWVMHEYEITSPPLPATVLSRIRATPRAKDKKLCIKEEPSCSTSAAGERSGPNPDHTAAGAGDSATANHNNTTSAATTTMAAAV*

## >Os11g05614

MATTRSGVGGAISDPFATPGFRFYPTEEELLGFYLRHRLAGTRPDVERVIPVVDVYGYHPSQLAALAGEASARDTEQWFFFCPRAERELHGGRPARTTPSGYWKATGSPSCVISSATNRVIGVKRTMVFYQGRAPTGTKTRWKMNEYKAVADDADAAAAAMLHPMAPPRLRNELGVCRVYISTGTLRSFDRRPLDNQAAAPTQQQVMPSLTAAAAVNTNLCGGGGGVVFAGAQGDSSRDCSSSSGSRELAGGADGSEDDAIDWNSLISSATADDLGFNTVVGFDPSIVGSWPQV*

## >Os11g04960

MNPTIENGGSGGDGSAAAAAAEGSAIWKSELVPQLQLPPGYHFVPTDEELVDFYLRGKIEGRDPPRHFISEENIMRYDPQKLIEKYKGYGEDRWYFFMVREPSKTKKKDEPNRKVVVDGVEEGSWSATGSVVQIHSTKETNRKAIIGSKRVLTYKSARSAENDMWSMHEYVLAGKSQMGQYVLCAIQLKQTYEREEKAREEQKNDNKRNKKAARRKNMQQQPTACQAQDEQQETAPTPGEETIVDPDQFMDIAHSMHMMFGGVDQDAPPFMPSLIAPCNNDDGMLQLQPLQLQNPNPAMLYSNQLEPSYIGDQSMFTPCCCDRNCISCRQLQFYQQQQAEDGSVAFGEADLYQQHDRALGNTGVYPDNVWVDGNMADYAQRQIYNDQDNGGVLMQGPEDSATFPDNFLMLDEMAAGSDDASGFDYEVDQSMAVVPHVADQTVDDIMSSLLN*

## >Os11g03370

MVETSTSLVKLEQDGSLFLPPGFRFHPTDAEVILSYLLQKFLNPSFTSLPIGEVDLNKCEPWDLPSKAKMGEKEWYFFSHKDMKYPTGMRTNRATKEGYWKATGKDREIFNLQPTSYGGSSNNKNNKQLVGMKKTLVFYMGRAPKGTKTNWVMHEFRLHANLHNDNPNLRLNLKDEWVVCKVFHKKGDDREAINKQQAQAAAVDQYSAGTPNNGSSVEAGDDDDDLFQLDSIIDPSIYFSNSSAANILSAPPNMSNSVVAANYGASTTTTGTASAGSFQQQPNYCSLINKSISSSNVSSWNNMPPPPPVAEGGVHGIGSSYSLQHQAAMVKALRDVIRLPNPLGMPQYKLDDAYLWDSS*

## >Os11g03310

MGNNEWYFSRKDMKYPTGMRTNRATKEGYWKATGKDREIFKPAIYEGSSKNNKQLVGMKKTLVFYMGRAPKGTRTNWVMHEFRPHANLHNHYPNLRLNPNEWVVCKVFHKKQGDEAINNQQQQPAVDQADDDDIFQLDDIFADPSIYDFSNSSANILSAPPNNNAVHSSVSAGTTMTSTTTASSFQHQPNCYSAPLQQHVSSWNNTPGAGGAHGIGSSYYNLQQQQQQAAMVKDLEDIIAVPDYGTLLPSSNKGSSIRSATAGVSQQNPLGVPQYKIENYGDHYISRE*

## >Os11g03300

MPSSGGAMPALPPGFRFHPTDEELIVHYLMNQAASVKCPVPIIAEVNIYKCNPWDLPGKALFGENEWYFFSPRDRKYPNGARPNRAAGSGYWKATGTDKSILSTPTSDNIGVKKALVFYKGKPPKGVKTDWIMHEYRLTGTSANSTTTTKQRRASSMTMRLDDWVLCRIHKKSNDFNSSDQHDQEPEESTVEQLEDIHDNNSSEQPPAPADMNNQQSDFQPMTAMSMSKSCSLTDLLNTIDCAALSQFLLDGSSDAIAEPPAPPSPLIYTTPHPNYQTLNYNINSNSSMPHAFESRLDHHDGYVNNYNVNGLRRKRMMACSATSFDDGSSSNDFVHAVVKKPQLLPSDSRGSGFGGGYCNQQLSETATGFQFQNGNLLSHPFPLNNHLQMQ*

## >Os10g42130

MGTMTLPPGFRFHPTDDELVGYYLKRKVDSLKIELEVIPVIDLYKFEPWELPEKSFLPKRDLEWFFFCPRDRKYPNGSRTNRATSTGYWKATGKDRKIACAGEVFGLRKTLVFYKGRAPGGERTDWVMHEYRLCQDLAHGVSNFIGAYALCRVIKRHEAGLHGEPPAAKAKGMISKVSSSSSLVTVEHQLSSRGNASPSFTPTNNGSPLVDEMFVGGGGGDPFQLLPSCVPYHGGDACGFDLPPLCMPQTQDPFFSDAGFTQAAPPLYGDVMGSVSEHELKWDTLGGYSGGGGGELWNAAAAPLLCRQASDGDDLTAWFTAADDNMSVF*

## >Os10g38834

MVIMESCVPPGFRFHPTDEELVGYYLRKKVASQKIDLDVIRDVDLYRIEPWDLQEHCRIGYEEQSEWYFFSYKDRKYPTGTRTNRATMTGFWKATGRDKAVRERSRLIGMRKTLVFYKGRAPNGHKTDWIVHEYRLESDENAPPQEEGWVVCRAFKKRTMQPPRSSIGAWEASYSYHDPAVFVGGGEHFKQEAAAELDGVAAAAGANAFLRYSTRLAELPQLESPPLPSQGSQAASAVVDGEEDNADSSRRPGGGGGAAAAVTTDWRAFDKFVASQLSPEEQHTCRATDDDDMAALLLLDGGGQEDDAGRWLGSAGLLSAVAADATTDCGLGTSCVPGDIN*

## >Os10g33760

MAGLREMESTLPPGFRFCPSDEELICFYLRNKVANHRVASGTLVDVDLHAREPWELPEVAKLTAEEWYFFSFRDRKYATGSRTNRATKTGYWKATGKDRIVHEGTTRAVVGMRKTLVFYLGRAPNGQKTTWVMHEFRLETPNSQPKEDWVLCRVFDKKKPSTIEAEGGGSSGSDLFIPGATDGSTDPSSPTTMAPLLGSSPDPTVVDRFDHRSAAVPPLMVLMQGGGDQMISGSGVHCSNNDNSGSSSALLNLTMLQYSFLEHRPTGDDMAVGAHFGTCQGGNNDATMALGMGFEEHGMGEIIEMEPAWRQGGSNCVYRDELYF*

## >Os10g27390

MAAPEDGEDKNFGKNKHGLPIGFYFAPTDQELLAILEAKRLGRPLSRAHDAFFHDIRILDFHPAELYEKYAKDEEKGYFYFFSKREFPTSSKKRPLRVAEGGAWNSSGAVYKVVKSSKSGGGYDVGHKKTLVFHQRFPGDKEAVKTNWAIQEFTRIIGPQNEVPDLAVYRLYKMRKEGRETPADLAADEAAAAAAMNNRGQQASAAAMALPPPATGLPGGRMMSMADKANMASTSKAYGPSKSSSSQLQQDAAAAAAPPNAAGASNWAPRPCNCRECAPAAGHYGYFAAAAAMNNRGGQASAAAKALPLPAPGLPGVRRMSMADKANMASTSKAYATSQSSSSQLQQGAAAVAAPPNAAGPSNWAPRPCNCRECAPAAGQYGYFASMVPRPSLDRKGKGKAPMDCAEQAGGGGGCHAESTSTPAPPKGAEYYGCSVAVEDDDEELLKFLQAMVRGEEVEGDGDHAMADERGPQQGSSPVAAAAASGSAPAGHDGRRGSLQGGHHGSSSPTSLAAAAATGDDVTSAASAGDDVSGSQQEDHPAR*

## >Os08g33910

MEERNDVNMDKSDEILLPGFRFHPTDEELVSFYLKRKIQQKPISIELIRQLDIYKFDPWDLPKLASTGEKEWYFYCPRDRKYRNSVRPNRVTTAGFWKATGTDRPIYSTEGTKCIGLKKSLVFYKGRAARGIKTDWMMHEFRLPTLTDPSLPKKPIDKNIPLNDSWTICRIFKKTSSMAQRALCQTWGAQLPGTIDPDIFSTLQSVQASQFALESSSCSLQAAATAAHQITSKYALQGNNNNQQQQQQHKPSNPLDGSSCKVINFNCSQSAEVQNSQIILPFEAHTSQKTATPLLFDTQFGQPDQISRFVVDSSVNANGGGISNKSQDPSARKPGSGFSMNSDWDGVARINFPFDLGADSSEDWRSSIPWESFLSPTTVHAEMPN*

## >Os10g27360

MAAPGDGEEKKAGSGRNKHGFPRGFRFVPRDQELLDILDDKLRGAPLDRALDAVFHDTRILDFHPAKLYGMYAEDEENGYIYFFSTIEFKAAKPKQKKWPRRAAQGGRWKAVLGSSQMVEVGGVPVGRKLSMEFYVKGVRTNWGMHEFVRIIGPNIEVADLAVYRLHKLWTNGEEKPGDLAADVAKSTNQSGQASAADYYQTYQNAVSQAYAYAPPYVLQPGWSQGYPYDVAAAPPTAPWPVCWAPPSAPGSYDCCYASTFSRPPPPPPIAASTLDKAPITSTDHGASTNTSAATPVANNKPPPPPVAATATTLGKKGEGKGKAPTTTSTDHAGSTNTSAPPAANYQPPPTTTTPPLQGTQHVFAPGVVVGHEDEEGYLIVDEVNTWRNTQQLVLEDDDDDDDGRAAGAGAGEGGASASGR*

## >Os10g26240

MGSGRGRRRWCPSTARTGTACLSAATSSPRTLELFAILRCKLVCGQLPGALNNVFEHIRILEFHPALLHETYIGKEEDGYIYFFNRWQFATKAGNKRRPTQVAKGGTWKASSGSKTVRSKKVGGIDIGQKLTMMFYERRFEGDRNPIKTNWGMHEFTKIIDDSKNQKPGSPC*

## >Os10g25640

MEGVDRIGWNLGLGFLRKERPPVHGDSGGSGGSNSNDGKWKGKEKVVPEYGKNRHGMPVGFYFVPKDLELLAILMCKLVRGKVPGALNNVFKHIRILNSTPPSSMTYIETMEDGYIYFFSKRQFATKARNKRRPMRVADGGTWKASGGSKKVGGIDVSQKFTMVFYERRFEGDRNPVKTNWGMHEFTKIIPGTKN*

## >Os10g25620

MAELPPEGRGGGGNNNGKWKGKEKVVPEYGKNRHGMSVGWYFVPKDLELFAILKCKLVRGQLPGALNNVFEHIRILEFHPALLHEMYIKNEEDGYIYFFSKRQFTTKAGNKRRPTRVTKGGTWKASGGSKTVRSKKVGGIDVGQKLTMVFYERRFEGDRNPIKTNWGMHEFTKIIDGTKNQLEDLAVYRLYKIKRKEDEEPSNTAAAASSTDEPSTSSALPPPTPPRPLPDMAGPSSATPLLPLQLPGLAGSSSAMSLPALQLPGMAGSSSAMPLPPLSLPGLAGGMMSMADQANMASTSQASTPSSELLQDWYDEFEITYGAVAPPSPSTISWVAPQSSPTGWWPSPNGGPVQHDGYLGMAADPTSYMLENLLPTAAIPPEPMMPPTSSPAPPPAVDHHHRLSPPHDAAGSNYNHPELADYNGGVQAQHEHQYHPQEPQASLVDAEDGYSAMAGGDDAQLGGAELDTERIAEMVNHIMDGEFEFKFEDNTVLKYNEVFPDNDEVVAAPMMIDGGRDGDGADGGDGDDPFDN*

## >Os10g21560

MSSKVPGLALLNTSISKFWSDEELVRFLAERKEAHSLPENVFVGMNISLIDPRNSEDIWYMNFSDDPQSPKNGENAIIKSKTGYWKVVGTVRIPTSTVIVGMKVSLDHYEGEAPSGKRTGWVMDEYLIEQNDEANLPQDYKNLCTIFFQGDDILNAGDKQICLNANVPNERKEFYLQYLAELEEQNAAWSNQAVSVNEQDVSSSKGLDGQKTSAADDQSVNHAPSREGYIELNDFLNSDSSASTSEYSSQRTMISEEYFDSDAFLREIRNDHNAADEEHTDSKFSVAAASKSDCVVISPPEQGFVNNLDNHATIAGDSPQKSVKNDKVDEHSSEEHPQHSPTTSCFPSHVKRSLSSSSSSSQGTSNSKSPQRQRERSTKKIGKLGKYWCCGSL*

## >Os10g09820

MTAESNGGNSSAAATTASNGGRRRHELVPKLRLPPGYHFVPSDEELVDFYLRGKIEQRRPPMDFINEVDIMSFDPVKLIEKYKGYGENRWYFFTVRKPSKTKKKDEPNRKVVVDGVEEGSWSATGSVAYICGKDHETVIGTKRVLTYKSARSAEEDKWSMHEYVMLDKSQILDTTRDTMSMDQYVLCAIQLKQTYEAEKKAQEEEERGVKRKRTATRKRRKGDIDQTTSQEQEDQQQETPPPGDPHDQSVVDAPYYSTQMALGGEEEVAPVPWCADCMAQPDRIEYPAVWYNQQEQQPSQLVDRSMMTQGYIGDLSYIQNQFDQQQAHDHGSINAFDEALDQCHDTNFAWDNAGIYPGNNLLDGNLDDDTQDQFGNQSTLGALTGELEYGTGYQFHDALQATPGSDDASAQSMGIQPAAGHSMGDDDETCCNDDLSSLLADISRILLDGNGVINNEGNPEGSNQGLHICKDGHQWPLEESTMPTVECVLQHPGESA*

## >Os09g38000

MEKVQASCEGDQGICKRGAKGGHPIDQNLVGGMTMSTGVPTTVPPTPALAALKNLLIRVVAAAAGKGLSSSAEEARCLWAKLPAPEEEEEAGQQPERPPSSYRRPPTSTLMAPVGLPPGFRFHPTDEELVNYYLKRKIHGLKIELDIIPEVDLYKCEPWELAEKSFLPSRDPEWYFFGPRDRKYPNGFRTNRATRAGYWKSTGKDRRVVHQHGGRAIGMKKTLVYYRGRAPQGVRTDWVMHEYRLDDKDCEDTMPIKDTYALCRVFKKNAICTEVEELQGQCSMALLEGACQQLLASGGGGSQEQYETPSPPDVPVGSTFGGADADAEDDPDKDDSWMQFISDDAWCSSTADGGAEESTSCVALAG*

## >Os09g33490

MATPGQQLPPGFRFHPTDEELVVQYLRRRALCRPLPAAVIPDVHDATVLDPWDLPGAGDGEAYFFSFRQLAAASGGGGWRRRRAGSGYWKATGAEKPVFLRGFGCGGGGGGGGQHLVGVKTTLLFLRAKPPSRTHWVMHEYRLAAAGAVAVAAAGQTKRGNHSCMAQPGEWVVCRIFLKNNRSSRRRAGDADGETPVTGVHGHRRRQPSPSPSSSSCVTAEVSDGEGEEEVSSGSINGAPSASQREA*

## >Os09g32260

MAMGMEGSGGGGSAKKKEESLPPGFRFHPTDEELITYYLRQKIADGGFTARAIAEVDLNKCEPWDLPEKAKMGEKEWYFFSLRDRKYPTGVRTNRATNAGYWKTTGKDKEIFTGQPPATPELVGMKKTLVFYKGRAPRGEKTNWVMHEYRLHSKSIPKSNKDEWVVCRIFAKTAGVKKYPSNNAHSRSHHPYTLDMVPPLLPALLQQDPFGRGHHPYMNPVDMAELSRFARGTPGLHPHIQPHPGYINPAAPFTLSGLNLNLGSSPAMPPPPPPPPQSILQAMSMPMNQPRSTTNQVMVTEQMIPGLANGVIPQGTDGGFTTDVVVGGTGIRYQNLDVEQLVERYWPGSYQM*

## >Os09g32040

MGMENPPLRWPPGFRFSPTDEELVLYFLKRRIATGRPTPYIADVDVYKSHPSHLPERSALRTGDKQWFFFSRMDRKYPNGSRASRTTGEGYWKATGKDRSICNGGGGGTASGRAVGSKKTLVYHHGRAPRGERSDWVMHEYTLLADALPPAARDREAYALYKLFHKSGAGPKNGEQYGAPFREEDWLDDDDHHHDQLPAEAALPAPATTSGRAATTEEHADFELPGGDLDVLLAQIENDQDIIEAQLDFSTHVTSQVQIQHRVHQGWLSDDGGKSDVADATTSGSALLMAENTCAELPIDGLEQLLMQISDDQQTVEMLSGFSASVPQSQLQHDYHQGCLGVHREEVGVADSTTVSSAVVTEECTVRELQDIEGLLMQIENDQENAESLPDFSTPVHLHDCHQAAFGDFQGSQRATFNIANLSTMVQESPNFDLQTGPSNQITESILTTEPMNGETNAVEETSPLRSMSVLGSYDRQDGDDEFLEINDFFDPEDLEQILGSTRSQNLIPADDGVFDSLQYSDAPMFLPGSFDRTGVVAENHYVEFGASGIQNQGFQHTTELLAHNQVALNVRNHMKDNHVVFSHSSDATIIHTVNEQPPNRSSNASQSWFNGALSALLDSVPSSPAMAAENIGLNRTLQRISSFRSQQPAREEVSSTLINTRRRGGGLIFISLMVLLVAIMWTFSNGSAVKLSKGLWKFPST*

## >Os09g24560

MSARGGVTMAGGGGGDRAPSSSSTAMISRLLPPGFRFRPTDGELVAHYLARKAADAGFTSAAIRDADLYRAEPWDLLPPPRCDAAAEEEEEEEERCGYFFCTRSFRWPSGTRTNRATATGYWKSTGKDKAVLHGGGGGGGRPVGVKKTLVFYRGRAPRGEKTSWVMHEYRLLHGGAAATASSSPTPTTVVARSEWVICRVFVRKTPDGNNDRGTTEHHLPSDDAHLRSSPAPANSVDGAGHASCSFFSGANESMAPSDHFNIGDDMILHGHDEEELLMMNCSSAFDLPELLDYESFSLDL*

## >Os09g12380

MADADADACPAVFASRHPTEQELISSYLHPRLLLTTTKPAAAVAAGGVPSFIHHADAYAADPADLTARHLPARAADGSRAWYFFSPVRTTTERGTRRARAVESGDGCWHSESGVRAVVDAAGRRVGHRQFFSFVKKREEDGKRVRTGWLMVELGVDNDAASASSSNELVLCKIYMTPRMPPPSPPSAVTSSAAATMELMPRAPPPSAPSAVTSPAATTMELMAGGVHKRRKISDEIAAAATPPHPQQQRRQRCVPDNDGSKESSGESSSVVILDDDDDDADAPEDGGAVRSKLRSDDGVMLADARDDEQHAATSDSMAGTSGGAVTGGGHGKLLPDLNVVATVAHDDEGRHARGAPRPQDGGTSTTTTMVASAGAERGSTTGHLPAATAGYRRTLMLFLEEEDDDAVEDEQQQQQAPPLPPATSTATTTTRTAAEANVQRQRQPPCCTFVVHPCAVHAKMRHGAAYGCGCRVTGAVRRGGYHLPRRAVHTTTTGQ*

## >Os08g44820

MSHPSSSSSSAPPAAAEATSLAPGFRFHPTDEELVSYYLKRKVHGRPLKVDAIAEVDLYKVEPWDLPARSRLRSRDSQWYFFSRLDRKHANRARTNRATAGGYWKTTGKDREVRNGPTTVGMKKTLVFHAGRAPKGERTNWVMHEYRLDGQTTIPPQDSFVVCRIFQKAGPGPQNGAQYGAPFVEEEWEEDDEDVGLLPVEEKDNSDDQEKEISGAMEKGYLQMSDLVQNLVDQNENGTIALPVSDNSNNSNHSEDVDGNSGDILSDQNLGSNFLHHVEPVEQNGLVLNENMFSSANAGDLFNISSPNDGFLELKDFADIADLENPLANESTIWPSDGWPWKSTDSMEAVNGASNEFSPLAGEQIFQPEELEQLLQSLQEDSHMGSTISDPPHSSITNLAKPEEDCLMFYDAPFDSSMCDDGFRQLNGFLGSPSTNLSGIDMVDDGMPYYDAMDDNLFNDLLSSVQPSAGSSSHAFSGPVLTQEVNNSTYTYSPTQKVLEPNFVVGAPSSARLPEAGSQLNYVVLPDSQTKSSLIGKRFVKILDSISAPPAFAAAEFPASLRKSLAPISGAHHNTIRVSAEVISIGSLTPDSQDKWSLEKDEGMELLFSAGFEPDTRVHFGCNTITAVLRGGFCLFFFSAIMLLVSYEVGMCIYGK*

## >Os08g42400

MERAAAAAPVVVRHGGVVLPPGFRFHPTDEELVVQYLRRKAFGLPLPAAVIPDLHNLFKLDPWDIPGASSDGDKYFFAVRPPAARGRRQHVTASGGCWKPAGGRDKPVVVARCGGSHLVGVKKGMVFVPRQGRKAPAAAAAAAGGGCWVMHEYSLALPMHKKGCLAEAEEWVVCRIFQRSSSGSRSPRRPDNDVRRTMPAVAELGRSPSPSSSSSQSSCVTSSSDQEEVSSG*

## >Os08g40030

MGDALWEMLGEEMAAAAAAAGEHGLPPGFRFHPTDEELVTFYLAAKVFNGACCGGVDIAEVDLNRCEPWELPEAARMGEKEWYFFSLRDRKYPTGLRTNRATGAGYWKATGKDREVVAAAAAGGALIGMKKTLVFYKGRAPRGEKTKWVLHEYRLDGDFAAARRSTKEEWVICRIFHKVGDQYSKLMMMKSPASYYLPVSHHHPSSIFHDLPPVPFPNPSLVPFHHDLPTSFHPPLLQHSHANSKNSSSNNGGFVFPNEPNTTNSSDNHISCNGAMAAAAAAAFPSFSCASTVTGKGGPPAQLGVNAGQQEPPPPTWMDAYLQHSGFIYEMGPPAVPRGA*

## >Os08g10080

MSFIGMVEARMPPGFRFHPRDDELVLDYLLHKLAAGGRGGGVYGGGGGVAIVDVDLNKCEPWDLPDAACVGGKEWYFFSLRDRKYATGHRTNRATRSGYWKATGKDRSITRRSSISSGEPSSSAAAAAVGMRKTLVFYRGRAPKGRKTEWVMHEFRLEPQPLHLKEDWVLCRVFYKTRQTIPSPSSEEAVTLPNELDLPATPSLPPLIDAYIAFDSAPTTTPSMVGSYEQVSCFSGLPALPMKGSISFGDLLAMDTSAEKKAIRVLHNSNTAKLELSPDWGQESGLSQMWNPQ*

## >Os08g23880

MAAAKMAGLTPGFKFEPSDEQLVQFFLLPYLRELPVPLGGLVIRDDPRSVPPWKLFARNGRGDEEDAYFLAPADGEGRQARTCDGGRGRWITQRLERTGNLRLAGGGGSGEAVVFEKHRLNYHAGEGRCGSTGWVMHEYAVVKPAALGARHRACHIAFTGHGQKRKRVPDGYVDVEDDGSKASTNAAAAVPPSSTAAMSACPSNVTYNQGCHISPEQSIEQHFPAEHNNIQIQQQAYYQSQDHEQCQYSDEEKYLLQQIKQEQYYYNQQNCFLPGQGNQELYYNDEQQQIFSLPEHQCSQEQYCHHDDQQDCVLPEQHSQELHGYNNEEQGYLLPPEPIDQEEQALFVGGEPQHEQQPLTSTPRQALLDYDDGKLLPPVGVNGAIAIPPQDAAVASNDDDGGQATEAPAAKMTAEEKKWFMEELLTEGCWSGPLLFDQPYYGSALKN*

## >Os08g33670

MEEEQRLPAGFRFFPTDEELVTYYLARKAMDATFTSAAIRDVDLYTSDPWHLPCDSSAASTGGGGGGECYFFCRRSSKYPSGARVRRATAGGYWKSTGKDKGVYAAGGGGGLVGTKKTLVFYEGRAPRGEKTSWVMHEYSRAPSTNFIRGAQARTHNLLDIIYSEWVICRVFKKQPPIEHWLEMEEVETTTTTTTVQEHTPNRRRLPPAEAAAAPPPPSGQPWQHTSRRSGDGRAAIDGGNREEEEDEHGLAREESSSPVVISSPSRCTSSPSSRLLNHEHLGASSSDDLPELMEFGDIYGGIAAGGPTDQQASSSNSNSICNFLDEPYYCWNF*

## >Os11g45950

MGAGAGEPWFYFRHHERWMHRMDRSTPSGYWKTAGKASFVYSADRHPVGLKKSMLFYRGPEPSGRKTKWKIDEFWALDNAANGSGELLAQLCRSRQNVGLMPRFPLSLCRLYSTKSSSERHVLATSSDEISEDESD*

## >Os12g07790

MDVDPPPPLPPPPPPPPPPATPQQNKAVELPPGVYFNPTREEAMHHYLNRWIAGKTIPEMEAGFVAGADVYGDGPDALRRRHRPGYWCNCVYKWFFLCHRKRQSSRRTTGNKRAERVVAAGGRWKVEQGKKVLGGGGGGGERDSLGFYSSNSTKKTSWIMEEYTSSAADGAAAARGEEDRMEPVLCKIYLSPRAPAGEKRALFGEDGVAVGPDGRKRNARVTVLATLFDDVAALLGQPVAAPPLPAPGEQLGHGHGHFDDVAARFGQVAVAALPASGDLGHGDFDDDAAALLGQVAMAPAPAPGHHQQGYVVAEAPLPESGYLGHYHHDGHLAQAAAAPEQDHYLGYHSHDAHVVDAEATPEQGYHDDAHVAVAPAPEQGDLGHDQGHLAAALTPEECGEIVGAYEFHPEMVQMLSIGFAAPDEQLLPQLLDPTGGGYDMASSAIAAVGDVNAYAAAAPTPRIRPNAAEAMAATATAETMPPPLDAVAAELSAPPRGLPPELAFSALPSVQQEPSCDDGDNFGELVAEAMPPLIGENAGVDAGSDEPLPDLAGIMTELDFGHDFFSNQHRE*

## >Os05g37080

MGELPPGYRFYPTEEELVCFYLRHKLDGGRRVPDIERVIPVADVCSLDPWQLPEAHQGAWTGDGEPWFYFCPRQEREARGGRPSRTTPSGYWKAAGTPGWVYSSDGRPIGTKKTMVFYRGRAPAGAKTKWKMNEYRAFEEDDDNAAAAAPAQNHYLQTRSDFSLCRLYTRSGCPRQFDRRPPSSSVAGGGGENRAAPSSTAAAFANEDAAESSGKSQKRKRSAPDDSLDSTSSSDDNGGCDGSMLQQQQQRQRGTDEELVECSMTDWADLLDWF*

## >Os07g12340

MAAAKRRVRDAEADLNLPPGFRFHPTDEELVAHYLCPRAAGRAAPVPIIAELDLYRHDPWDLPHRALFGRREWYFFTPRDRKYPNGSRPNRAAASGYWKATGADKPVLHNGRTAGIKKALVFYHGKPPRGVKTEWIMHEYRLAKKGGAAAAAGAGALRLDDWVLCRLYNKKNEWEKMQSRKEEEEAMAAAQSWGETRTPESEVVDSDAFPEMDYSLPAASFDDALLPKEEARDDDWLMGMSLDDLQGLGSLLQADDLSMLAPPPAAKTEPLGAPFF*

## >Os07g13920

MAAAGADGLPPGLRFDPSDDELVGRYLLRRLQGQPLPLDGVVLDADPLSAQPWRLLADHGRGGDEAFFLAEAHAKNAKGKRQKRTVEGGGFWQGQRMCVDGKKLLVPGGDDGGGGGEVLEIAWRKYVLSFFAEGERGSSGWVMHEYSVTAPADLASSPLRLYRIRFSGYGKKRKREPEDDGRAHGAPRRAEAETALFDLEVGPPPPPLLVPPPAAAAADHGTDQSSSGVTDMVFRDLPDLIADAGAALPDQNQQDWSEVADQSSFCVMGDDSSLLLPDLPGMIDDNEHQQFVRECDMPHLFVPQAEEAIAGGGAASAPSADNQNCEFNDGEDMALSDFEFPESIDEVLSYIDFSTSDTSCRDFTMDELFDLPVD*

## >Os07g17180

MAAGGGGADGLPPGLRFDPTDGELVSRFLLRRLQGKPLPLNGVILEADPLSVPPWKLLAEHGRGDEGFFFAEARAKNGKGSRQKRTVEGSGLWQGQRVCADGEKLLVPDGGGVEVEIVWRKYLLSFFAEGERGSSGWVMHEYAVTSPAELAASPIRLYRVRFSGHGKKRKREPQSGEDGVGRARAAPQSAGTETALLEERVMPPQPAPQSVGTEDALVEERIPPPQPVPIPPIAGTEDALDVGTEDVRGRAAPQSAGTESALLEECVLPPQTAPQITGTGVALLDEVVPPPQTVSISPPAALVDAVDDADCANQGCSGVMDDSTMVFSHLPDMITLPAEEGDAAGGAALASMDYSWADFEFPEINMDELPNCIDFTTTDPSCLDIELSMGDLHEPQSTGIESDLLEEFVPQPQPVLVPPLAALVEVADSSEGPDQGCSVVMHDSSAVFTHLSDPIVLPEEEEADRPDAPAGTMSLDYQNYSLSDFEFPEYPLLDVAGDADGADQCSSNVMDDSSMVFSHLEDLITLPAEEAEADACSAAPAPSLDNQKYSSQGIIDSEAPALSDFEFPETIDEVLNSINFTMADPSCLDMEFSMDDLLDFDPPAD*

## >Os07g27330

MAAAADGLPPGVRFDPADDELVSRYLLRRLRKQPIPLHGVIHEADPLGAPPWMLLAAHGRGGDEAFFFAEARAKNVRGKRQKRTVEGGGFWQGQRVCIDGERLRVPGDGGGGEVGGELEIEWRKYMLSFFAEGERGSSGWVMHEYAITAPADLASSPIRLYRVRFSGHGKKRKREPERLGARVHDDDVDGGERAAPRRAVAETALFVQQSSAVDCAESADQSFSGVIEPVFHDLPDMMPEQADAGDTAETTAAVVNLTDAMTEQPVLPLAADGDDQSSYGVIDPAFRDLADLMVLPPVLAQQEPPLAPVAMVDLPPGNADCADHQSCSGVIDPAFRDLPDMTVLPPEQADTGGGAETTTAMVSLTDKLKYSSSMDGEAAPAWCDFDFPESTDEVLSYMNFTAGAHDNNDGSVGRAAPWRPVSEIAMFEQPSAVDLPPGDADCTESADQSFSGVIEPVFHDLPDMIREQADAGDTAETTAAVVNQNYSMALCDFDSGIDFTAGAHDSGMERATPWTPMSEAALFEQQGPPLAPAAVVDLPPGNADCADHQSSYGDMIVLPSEQAGAGGGAETTEALFDQPVPPLAADCANQGSYGVIDPVFRDLADLIVLPPEQADAMDGEAAPAWCDFDFPENIDEALSYVDFTAGAHADNDGGVSETAMFEQPGSPPQHDPLLMDADGADQSSSSGALIDTVFGDHAEPIVLPLEQADTGGGAAAAVKLMDKQKYSSSSMDGEEAPAWCDSDFPESIDEVLSYVDFSTDGASCDFSMDELFDLAD*

## >Os07g27340

MAAAADGLPPGVRFDPADDELVSRYLLRRLRKQPIPLHGVIHEADPLGAPPWMLLADHGRGGDEAFFFAEARAKNVKGKRQKRTVEGGGFWQGQRVCVDGERLSVPGGDGGGEVGGGLEIEWRKYMLSFFAEGERGSSGWVMHEYAITAPDDLASWPIRLYRVRFSGHGKKRKREPERLGARVHDDDVDGGQRAAPRRAVTETALFVQPSAVDCAESAGQSFSGAIEPVFHDLPDMMPEQADAGDTTETTAAVVNLTDAMSEQPVLPLAADGDDQSSYGVIDPAFRDLADLIVLPPEPDDGGMERATPCTPMSETALFEQQGPPGNADCADHQSSYGVIDPAFCEQADAGEAETTVSAAVVNQNYSMALCDFNFPEVLSYVDFTAGMEPSWQQRWPPMSESAPFEQQEPPLAPVAMVDLPPGNADCADHQSCSGVIDPAFRDLPDMTVLPPEQADTGGGAETTTAMVSLTDKLKYSSSMDGEAAQAWCDFDFPESTDEALSYIDFTAGAHTDNDGGVSETAMFEQLGSPPQHDPLPMDADGADQSSSGPLIDTVFRDHAEPIVLPLEQADTGGGAAAAVNLMDKQKYSSSMDGEAVPAWCDSDFPESIDEVLSYIDVSTDDTSCIDFSMDDLFDLAD*

## >Os07g37920

MESPDSSSGSAPPRVLRRQQQQPGSAPELPPGFRFHPTDEELVVHYLKKKAASVPLPVTIIAEVDLYKFDPWDLPEKANFGEQEWYFFSPRDRKYPNGARPNRAATSGYWKATGTDKPIMSSGSTREKVGVKKALVFYRGKPPKGVKTNWIMHEYRLTDTSSSAAAVATTRRPPPPITGGSKGAVSLRLDDWVLCRIYKKTNKAGAGQRSMECEDSVEDAVAAYAPSSQQHATAAAGMAGSDGAGGVAAAHGGDYSSLLHHDSHEDTFLVNGLLTAEDAAGLSTGASSLSQLAAAARAAATPCDATKQLLAPSPTPFNWFEAFLPRAKEFPSGLSRSSRDIGDMSLSSTVDRSLSEAGAVAIDTGDAANGANTMPAFINPLGVQGATYQQHQAIMGASLPSESAAAAAACNFQHPFQLSRVNWDS*

## >Os07g48450

MEMTMSSAATSLPPGFRFHPTDEELILHYLRSRATAGQCPVPIIADVDIYKFDPWDLPSKAVYGESEWYFFSPRDRKYPNGIRPNRAAGSGYWKATGTDKPIHDSATGESVGVKKALVFYRGRPPKGTKTSWIMHEYRLAADPLAAAANTYKPSSSSRFRNVSMRLDDWVLCRIYKKSGQASPMMPPLAADYDHDEPSGVLDDAYSFYAPPMISTTLIPKLPKIPSISELFDEHALAQIFDAAADPPADHHQHALAVHPSLNQLLGVGDNFLAECYPSTASTATVAGGKRKASPAGDYAGGGHTPAKRLNGSCFDVAPQSVVGGLQATPSSVLAGLNHQMLPPQLF*

## >Os07g48550

MSESEVSVINQLEEEETRLELPPGFRFHPTDEEVVTHYLTRKAQDRSFSCVVIADVNLNNCEPWDLPSKAKMGEKEWFFFCHKDRKYPTGMRTNRATASGYWKATGKDKEIFRGRGLLVGMKKTLVFYMGRAPRGEKTPWVMHEYRLDGKLPPNLPRSAKEEWAVCRVFNKDLAAKIAQMPPPPFPRNDSFDLDLDDFLHLDADLPPLIDDPFASTSTLKTEPPPPANLMHNHYGYFSLPASATNYNHSSGAMADQAIRRFCKAEASTACFSGADADVDPVVDELLSFPDSITDYSYIWKA*

## >Os08g01330

MDRHEEEAGESPCVPPGFRFHPTEEELVGYYLARKVASQKIDLDIIQELDLYRIEPWDLQERCKYGGHGGDEQTEWYFFSYKDRKYPSGTRTNRATAAGFWKATGRDKPVLSSPSTRVIGMRKTLVFYKGRAPNGRKTDWIIHEYRLQSNEHAPTQEEGWVVCRAFQKPMPNQQQHRLSYGCIPGSYGAGAYAAVPDNYSLLLHHDNPSFAGRPLMSAAASALFANNNNNSVVDHSNILSSESKLHFSDMMPPLESPTIVDGEGYVSQASSCVDVDQQAGIVDWNLLTSLLPPPAHQLFHHLPSASSSKNSNNISSSGFIDDRD*

## >Os08g02160

MRGSDHHQDVVAAPRGGGGGGDDGQAHDMVMPGFRFHPTEEELIEFYLRRKVEGKRFNIELIAFVDLYRYDPWDLPALASIGDKEWFFYVPRDRKYRNGDRPNRVTPSGYWKATGADRMVKVEGDRPIGLKKTLVFYVGKAPKGLRSSWIMNEYRLPHGDADRYQKEISLCRVYKRPGIEDNFHLTGTTTKSSGSKAAAAMGKKHAAANRTSSTAAAAAPRLAPMFDGGGGGQARATASAAKKQAATAAAAKRRHPRRHRRPWSLQRRRHSTSSRVCSRVCHRWYGAPLTHGSPAGAGTGDISHPTTCAGSGSGGEVGEQGGGVPVQPTPLPTL*

## >Os08g02300

MSISVNGQSCVPPGFRFHPTEEELLNYYLRKKVASEQIDLDVIRDVDLNKLEPWDIQERCKIGSGPQNDWYFFSHKDKKYPTGTRTNRATAAGFWKATGRDKAIYNAVHRIGMRKTLVFYKGRAPHGQKSDWIMHEYRLDDPATDTAAATPTVTSAAAAAAAMAAAADGGQEDGWVVCRVFKKKHHHKEAGGGGGKHGGDGSAGAKAAHAYSSSDDALDQILQYMGRSCKQEHELPSPQASGGGGAGAGSRPASRYLRPIDTVLGGHGFMKLPPLESPSAATALSSTPSTGGDAASSAAAAAADHLLLHHHHRTDWAMMDRLVASHLNGANSDAPDDQLCFDAADDDGLAYYSAAATRLLGGANAGTDDDLWSFARSAAPPPPPPPPSSATPERLSHVAL*

## >Os08g06140

MTVMELKKLPLGFRFHPTDEELVRHYLKGKITGQIRSEADVIPEIDVCKCEPWDLPDKSLIRSDDPEWFFFAPKDRKYPNGSRSNRATEAGYWKATGKDRVIRSKGDKKKQQVIGMKKTLVFHRGRAPKGERTGWIMHEYRTTEPEFESGEQGGYVLYRLFRKQEEKIERPSPDEVDRSGYSPTPSRSTPDNMEPIEDGNTPLNRESPESALHESPIDLPALTEAQAAPITRWLADRTDNATTNEVNISHMPHHGLDGGAKQASPSAGAFPQLIGSQQNIHDNNELATVSAPMLPHEDFNNFPLGAIGNFDGNMNPRDPVEEFLNQTIADPDEHSSTTSKAQYDSDTGIIPTEFENHGVMQGEFMDDLSGLENLDFWPDDRNPQLSALYEDTPLLPYDSTDQDVLSMDSGAESLQDLFNSMDDSNARNNVWGNEPFLQGTGFPMSWPLQPNSAFPNQGTANRRLMLQLSESLSPDFDVSMTRDECEDEEPGIVVTSKYVNEAPEESTAEKDMPSDGDDAEPTGITILRRRHAPTASSFSDGDDAESTGITILRQHQAPNASLLSDGDDAESTGITILRRRQAPTASSASSFTQQGAAVQRVRLQSNLDAAPCSSVDGSSSCIINEGESERTMEKPEIEENAGSTLAEGGTCHEDDQKEHDASAANAKSVLRLRKTAEGSDKENKQEEEEGVLASHVRAPGNKRGFPSYIIWLVLSVALVLLISLGIYGWV*

## >Os09g38010

MAPVGLPPGFRFHPTDEELVNYYLKRKVHGLSIDLDIIPEVDLYKCEPWELEEKSFLPSKDSEWYFFGPRDRKYPNGCRTNRATRAGYWKSTGKDRRINYQNRSIGMKKTLVYYKGRAPQGIRTSWVMHEYRIEESECENAMGIQDSYALCRIFKKNVVLGEFDKKGECSSSQAKGNEEVTDFGDAGQSSGANENDKDNSWMQFIAEDLWCTNKLK*

## >Os01g28050

MAICAAAADHSRASPSSAATQPTTICWRPSKVKEEVEEDGMVVDPPDDASEASDNLRATGFQDTPTVGELVLRHLRPRLRGFHCADGDVPVIGVRDDPAAAAPLDLVARHGGAADRRRGEAFYFVRRRRCRRPNVRRTVAEGGGGGGGAGGLWKKSWTGSGKSVTDLGVVVPWSKTCYCFYRRDEGGRLSTFGGGWVLAEYEITEPGTYRRADEEEDDDDYWVLCHVRKTASKKRKRNRCDEAVAARAVAGTESKSYLLCGLTAN*

## >Os01g47670

MGAASRPRRQCRAPRRLDGGGGGSMDVHPSELLPRSRCTAPRRLDDDEMDVHPSEQELIETGFAPAVARSSGDEAWYFFSAVRGLKGGRKARTVDDGAGCWHSEAGAKPVLAASSGRRLGHRQSFSFITKDDDGQRVRSGWLMVELSLDVDEEEQLVLSKVYFSPRAPGARKPTTAAAMSRHKRKLSTTDIASPPRRQRRHRVVPSSPPEEPNTSPSPAAAPPDQQEGGDDDPDRGSISWWLRRVFGLTATFTEEESIELNPWLKDILRPFPPPLPPTPPPPCPSPRRKLIDMPEIREFIMRGSYLGGGPAPPRYECDHPAMVMTGGDDQQQLDEQRRDDVGDDRAHYDRVDGQLQFERHYLQL*

## >Os03g61650

MAEIGKGLVFSPSDDQLTDGYLRSYLVRTSLDDLPSAATSYFHVADVYSAPPDQLVAGLAPAPGTGDGDGRVWYVFTPVRVLGSRGARKARTVGGGCGECWHAEGGPKDVKGSAAGGKLQKFSYKIKTALGAVVKPGWLMVEFSFPGSDHLALCKVYRSPRTSRYGAPSPPSSAASSPSRAAPPPVSSTSGRKRKAEEESDHPEAPASSAPRRTLPASEQHVDVDAAAASEPDQGGYLSTDQLDSVAAFVQEHEAFVQEHEGDEEFCKSLGFDERSDPQCWTNFFLSALEEFGPAPETDAAAVAVAAVEPGPCPEYEEHDDTATTAASSHAYDSATAELVNLSDKEFYDIIFSGDQQGGAAVAG*

## >Os04g39960

MASSAHMAAVLNLPWGYRFRPSDRQIIANYLGPMAIHGADSLPQRGDVVEGVDVFATRPAAIPFEPRRHVFGRDEVRAYFFGDQPTDSRGREVPGGAWLPCGGGDKAYSGGADGGEAVAYRRKYEFRAANEEADRAGEEAATPARPRWRMKEYRLNKSAAEFRRAYAQPNPKANMDCVVREIYTKAVPPPTPPSGRSGDEEMQEGSDYSVMDEDELVDYLLQGFEDGNFDEDQDQPAAAEDGDYSDEDEDQPAAAEDGDYSDEDQDQPAAAEDGDYSDEDEP*

## >Os04g40140

MAQNWKIQQIYHNGVLKGWKKILVLYKGSKKNKIVQANWVMHQYNLGVEEGGEDGELVVSKVFYQLSSKQTGTPEMDSVTEEASDALTIRSDPITPITNPPLPRCLMNSPCDTEQNGTISHDQEGECSTSTLRPMVEAGNRAGCSAGASTAGDFNEDLLQRCEFPEDPVPTLDDTLPFLYTDETDLFSWEDFQFGSQESFGWVDGDHT*

## >Os04g42940

MLPMRPRASQAAAPETAAAAESLREEETEDGWVFLAGRSRATRPPPPPPSPMARAVASGSSGGGGGQPFDPTAEDIVNRYLPLRRALRCDALPRQVHDADVYGAHPALLASVYPAANERFEWFFFVCRRQCPGGRRRAGPGDYRLSQEAKHRGNAFCHSFRYYEYEDAGGGFRETEWRMVEYGDRGRDAGAGGSEGFELVVCKVYPARGGALHERLGADRAVLATRHRADEDAKPQVLVQLYLASLRLGNPLACRVHRADDVFDAHPAVITAALPAANDRCEWFFAAVRPRGHAQGHGDGAPPRPRKAGPGAYVPVRECRVVDGRRGDMGCRLVFWYREDDEEARRASRRTEWWMDEYRFGPDFPYGELPAPMARGEDEELVVYKVYPRLVGNRR*

## >Os05g26049

MAAAAAGADGLLPGLKLDPSDDELVGRCLLRRLQGQPLPLGGDILEADPLSAPPPWNLLADHGRGDEAFFLAKKGNGKRQRSSVEGQRMCVDGGRLRVPDDGRGGGGGLAFLPSIFPSPPPVPRCSTPLSPSSPPFKPSRVIVRWWAPPGGWCKLNFDGSVYDDGSRRASIGGVIRGCDGGVVLAFAEITEHWTVGVVEARAMIRGLRLALACFVERLVVEGDDLVLVQLIRGEETQTRIPAAMHEEILDLLRCFADVEVRHIYREGNSMAHTLCRQAYVHPGLWTDCATLPAAVWEKIDDDLRGVVHERLCNKKKKSSA*

## >Os05g26026

MAAAAAGADGLLPGLKLDPSDDELVGRCLLRRLQGQPLPLGGDILEADPLSAPPPWNLLADHGRGDEAFFLAKKGNGKRQRSSVEGQRMCVDGGRLRVPDDGRGGGGGLAFLPSIFPSPPPVPRCSTPLSPSSPPFKPSRVIVRWWAPPGGWCKLNFDGSVYDDGSRRASIGGVIRGCDGGVVLAFAEITEHWTVGVVEARAMIRGLRLALACFVERLVVEGDDLVLVQLIRGEETQTRIPAAMHEEILDLLRCFADVEVRHIYREGNSMAHTLCRQAYVHPGLWTDCATLPAAVWEKIDDDLRGVVHERLCNKKKKSSA*

## >Os05g27749

MAAAAAGADGLLPGPKLDPSDDELVGGYLLRRLQGQPLPLEADPLSARPRNLAADHGRGDEAFFLAEAQAKNAKGKRQRSTVEGQSMCVDGGRLRVPDDGRGGGGLAFSHFLPLSPSIVPSPAPSPRCSTSTPLSPPKLLADHGRGDEAAFFADAWAKNGKRQKQRSTVEGGGLWQGQGMLVDGERLRVADDGGGGSAFLPPSILPSLLPAPRCSTPLSPSSPPFRPSRVIVRWARPPPGWCKLNFDGSVFNDGSPRASIGGVIRDSDAGVVLAFAETTEHWTVGVVEARAMIRGLRFALACFIERLVVEGDDLVLVQLIRGEETQTRIPAAMQEEILNLLRCFAEVDVRHIYREGNSVAHTLCRQAYVCPGIWSQRGGGMPAAVWDKVDDDRRGVVHERIRKNK*

## >Os12g04230

MAEEDDKKQKGPDVTVPSGYFFVPKPEQLIRDYLNHWITGRPIEELRDIVREADVYGSDPATLTEAHRAYGHDGKSWYFLTVAKWKGGRGGAGTAGRLNRCVEGGGTWHNSQRRRVIEGYGDRQAFEYRAPGNKKTNWLMEEIASNLPAAITDEGIMVICKVYLSPRAKEATADEEERQETNVVPGPKRLREAEATGYDAPAPETPQPDVGCSYSGGGETSQATASMDYCCSTTTHTADDTANAAAYYYGDVDAIKPDAYDGGDYGIGINADGELVLCGNGHGGIGTQGQMPLAMQNTNGEMTLFSPMNGYGVGFNEEVRQEPQVGGEVEMNDFFNDLFVDFDGAGDPNPNPNEGGDSHGHILCE*

## >Os07g31410

MAPLADPAAEGFRIPFLPSDSDLLDCLLRPKIASGRVDPRFAPLVHDVADAFALPPAQLAAAHAPAPGAGGAEAWYFFSVRPRARARARAGSKRAASRAVGGGGGKRWCSMGAKKAVEGGGYCQRFRYKERTAAGVVAPRWMMVEYGVAQEHDGEGVAQEHGGEGVAELVLCKIFRSPEPSRRSESGSPSSSSSASASPSCSGGRKRKAAE*

## >Os10g27380

MAAPEGQNKFAGVRFLPKDLELLAILDAKLRGSPLGPVEAIFHDTQILDFHPYKLYGIHRLDRSISLSLSLCRSPSSSPATEMYAEDEEEEGYIYFFSTMQFRCRKIVERAAQGGRWKVNNCETLEVGGVAVGRKFTMNFYEHIGGDNDLIWTNWGMQEFARIIGPNKELADLALYRLYKKKITRGTGEEKPEDIAAASDGDTESSMNKRRRVEASAAAMALPPPPPSPGLPGTMMFMAADQANVASTSQEWHGQFANGAAAAPSPSGCWPWAPPPTPSAVEPFSFWASASAATPPAAANYHPSPQPQPLPPQGGEYYSRHGAFSVAPVPASACSTPSPEAATSCLLATTSPLPAAGTEGSDSQQQEPPCELMEF*

## >Os11g04470

MAEEDDKKQKGPDVTVPSGYFFVPKPEQLIRDYLNHWITGRPSEELRDIVREADVYGSDPATLTEAHSAYGHDGKSWREKTTGASQQNIFTISRRGGFEGGGTWHNSQRRRVIEGYGDRQAFEYRAPGNKKTDWLMEEIASNLPAAITDEGIMVICKVYLSPRAKEATANEEERQETNVVPGPKRLREAEATGYDAPAPPQPDVGYSYSGGGETSQATASMDYCCSTTTHTADDTANAAYYHGDADAIKPDAYDGGDYGIGFNADGELVLCGNGHGGIGTQGQTPLAMQNTNGEMTLFSPMNGYGVGFNEEVRQEPQVEGEVEMDNFFNDLFVDFDGAGDLNPNPNGGGDSHGHILCE*

## >Os11g31370

MAGPQMSLPYDFITDIDILHHNPLDIVPTRQEKKNGKHFFTRKEKKHHGDNCRNHAAGVGFWRKSSFAESMEWAMQEFQLAGSYLLPCFVMRFATSDGTEQPFGCTRVTIANMRHCILTPYCCFIG*

## >Os12g22630

MATNLHLLLELGFRFNPSPEEVVTYYLPRLIAGHPPKDTESCIHRANVYGAEPRELAAQFAPVARSSNGDRFFLTECKRIKGKVSRVAGGGSWVSQTSKDIKNREGIKVGEAKNFRFKKDGTNTDWLMEEYHLCLRQASDLEPVLCRVYVSPRAAKDSAAHQESAALTPQEPAPPLAHAPAPAPIQEPAALPRQELAPAPPRLEAVITQQQATMKMGGSVPASKATRQSCVTASAPPPRRVAPQPAPPSLRTAPAAVAPPRQVPVITQQQAPPLKRPAPPVPSPPCAKKIRGPVSASPAARQSCVAASAPPPWCVPPPPRPAPPSRRVMAPLPPYPMDPFETPPSPHAPRHDPFEPPPSPDPPIQSYAIDPPIQSYAMDPFEQPPSPYAPHGVDDMDEFTRSLEAQLEEADGDEIAAATVAPPMAQNVAPDDDMDEFTRSLEAQLEEADGDDKIDDDEIDEEIFQIPLKD*

## >Os11g04360

MATLPQMAATKERQEAANPTTTTRTLVESVTNWIRVYSDGSVDRLGPPEAAAFMVLVPPYDDPRDGVTVHDVATDHGVDVRLYLTTTAPARRRPVLVHFHGGGFCLSHAAWSLCHRFYARLTVDLDVAGIVSVVLPLAPEHRLPAAIDAGHAALLWLRDVASGGSDTIAHPAVERLCGAADFSRVFLIGDSAGGVLVHNVAARAGEAGAEALDPIRLAGGVQLHPGFILPEKSPSELENPPTPFMTQETVDKFVVLALPPTKDTEKFICRADVYGSEPSDLAGKFAPVPRCEKGGRLFFTSCKRHKGSSTRKERTAGDGTWVRQNSKGVKNKAGVKVGETQNFRFKKDGSYTDWLMEEHHCCRQQAVAGDEEPVICRMYVSPRAPPDSAARQESAAFVQQQPAPQVSEPPCDKKKRDDVAEEAPAAA*
