# Supplementary figures and images for: Comprehensive genomic characterization of NAC transcription factor family and their response to salt and drought stress in peanut
Source: BMC Plant Biol. 2020 Oct 2;20:454. doi: 10.1186/s12870-020-02678-9 (PMC7532626; doi:10.1186/s12870-020-02678-9)

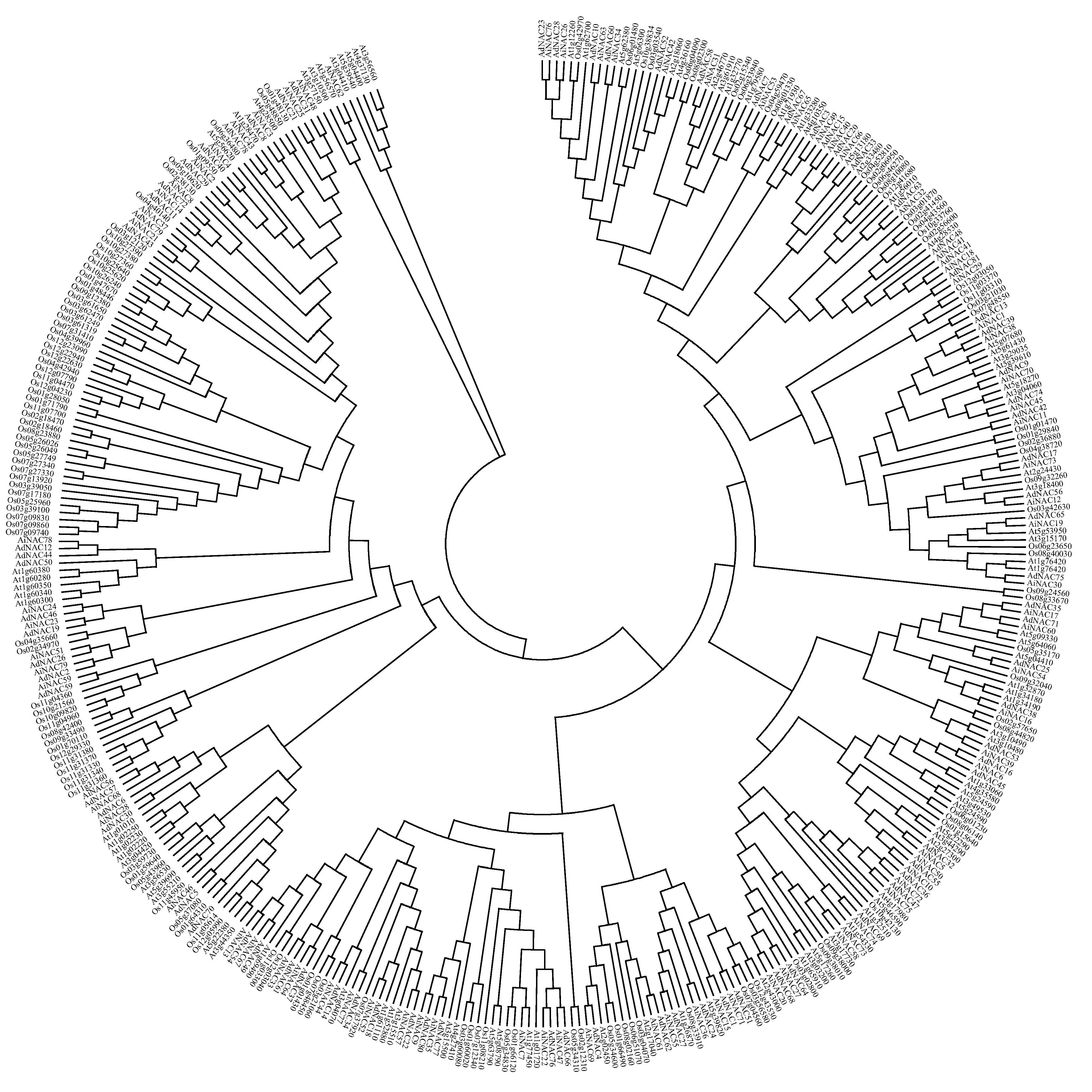

Supplement: Supplementary file 8 — Additional file 8. Phylogenetic tree analysis of NAC proteins among Arachis, Arabidopsis and rice based on conserved NAM domains. [file 12870_2020_2678_MOESM8_ESM.jpg]

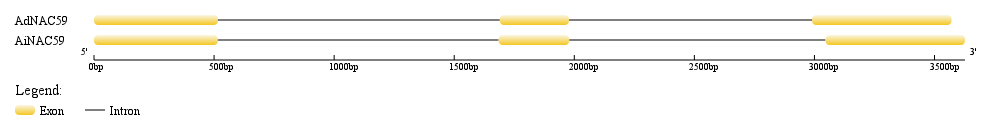

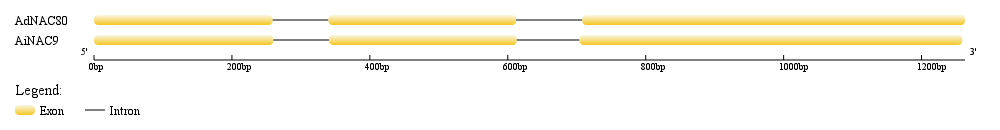

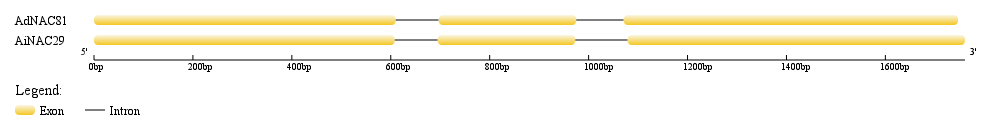

Supplement: Supplementary file 9 — Additional file 9. Exon-intron structure comparison between AdNAC59, AdNAC80, AdNAC81 and their orthologues AiNAC59, AiNAC9, AiNAC29. [file 12870_2020_2678_MOESM9_ESM.docx]

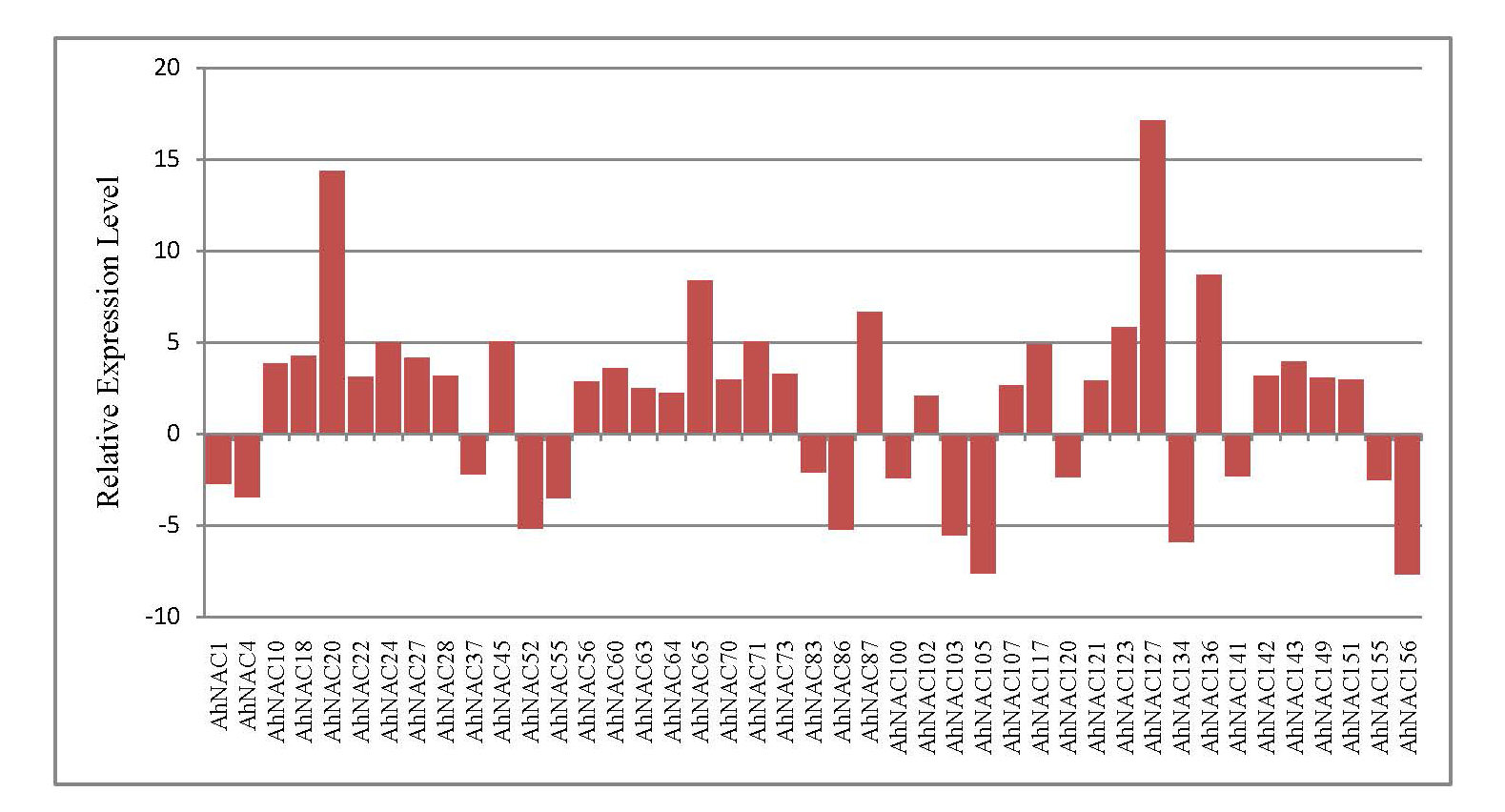

Supplement: Supplementary file 13 — Additional file 13. Genes involved in the salt response based on comparative RNA-seq data. The Y-axis represents the fold change compared with the level in un-treated plants. The X-axis shows the genes whose expression was upregulated and downregulated more than 2-fold under salt treatment in cultivated peanut. [file 12870_2020_2678_MOESM13_ESM.jpg]

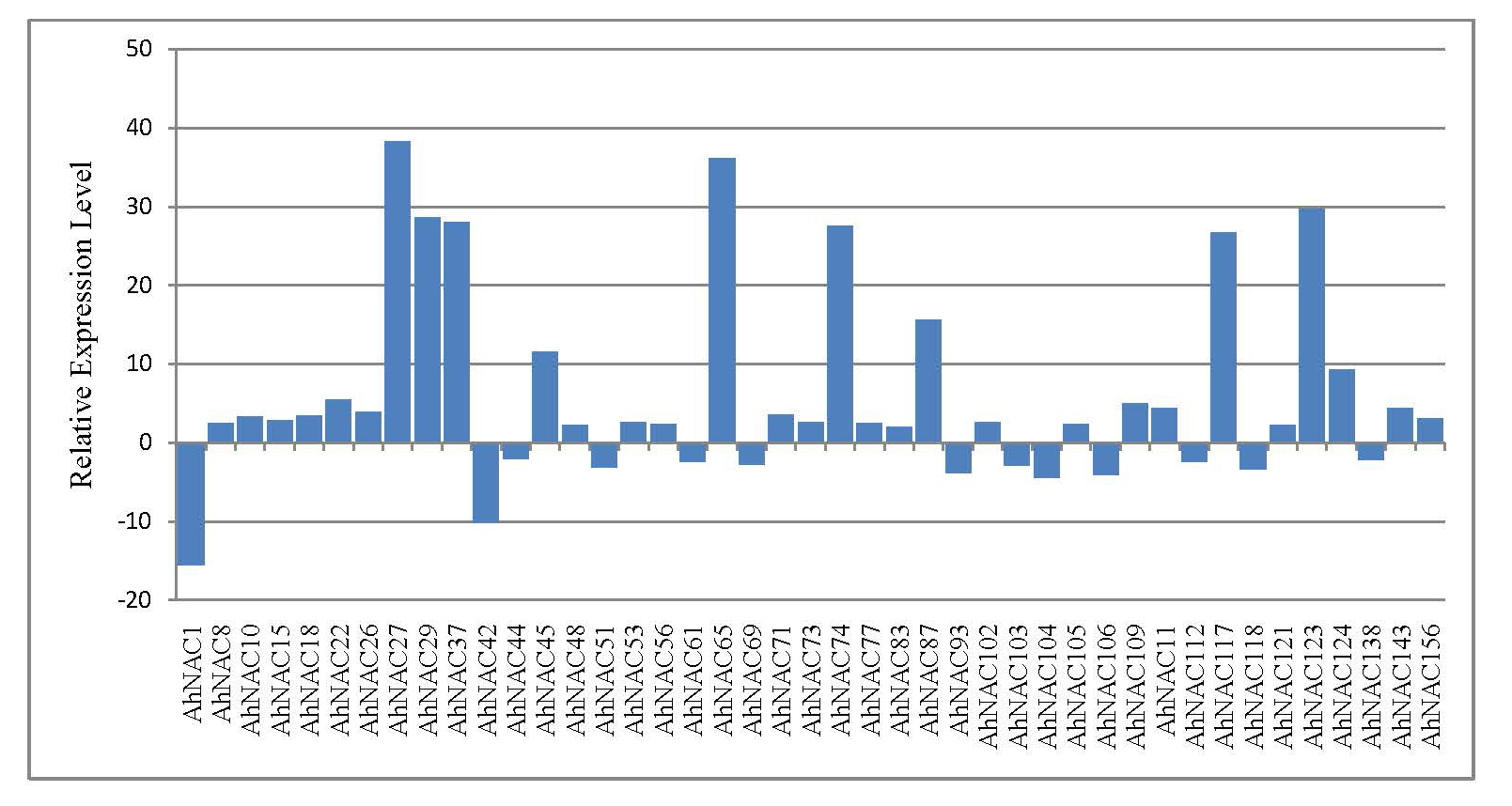

Supplement: Supplementary file 14 — Additional file 14. Genes involved in the drought response based on comparative RNA-seq data. The Y-axis represents the fold change compared with the level in untreated plants. The X-axis shows the genes whose expression was upregulated or downregulated more than 2-fold under drought treatment in cultivated peanut. [file 12870_2020_2678_MOESM14_ESM.jpg]
