# Supplementary material for: Comprehensive genomic characterization of NAC transcription factor family and their response to salt and drought stress in peanut
Source: BMC Plant Biol. 2020 Oct 2;20:454. doi: 10.1186/s12870-020-02678-9 (PMC7532626; doi:10.1186/s12870-020-02678-9)
Supplement: Supplementary file 10 — Additional file 10. Sequence logos for the conserved motifs within NAC proteins. [file 12870_2020_2678_MOESM10_ESM.docx]

| Motif | E-value | Sites | Width | Logo |
| --- | --- | --- | --- | --- |
| Motif 1 | 4.1e-2385 | 116 | 27 | 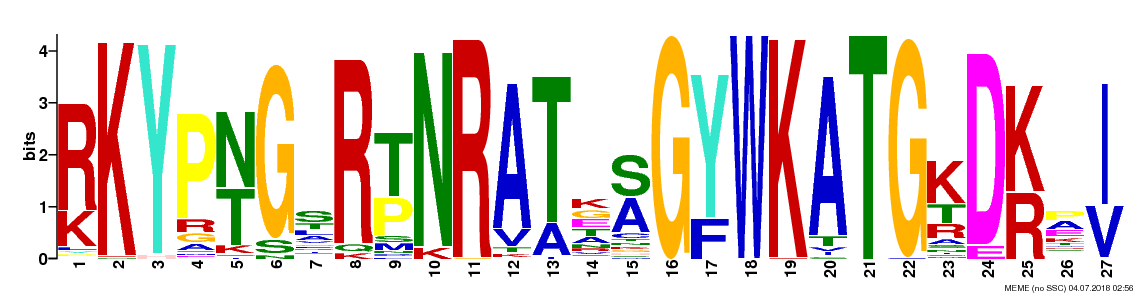 |
| Motif 2 | 4.3e-1649 | 156 | 15 | 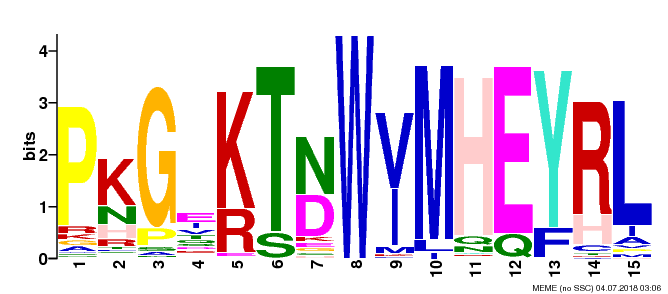 |
| Motif 3 | 3.8e-1626 | 147 | 15 | 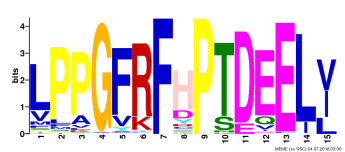 |
| Motif 4 | 3.9e-1308 | 148 | 15 | 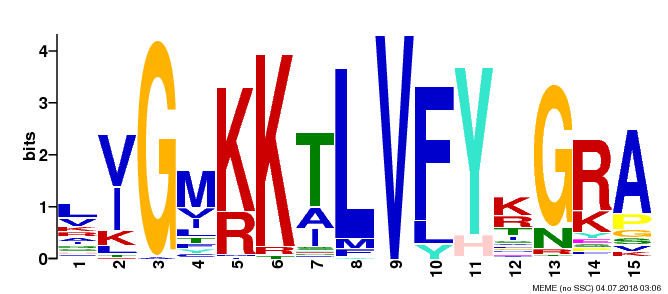 |
| Motif 5 | 7.9e-1299 | 118 | 21 | 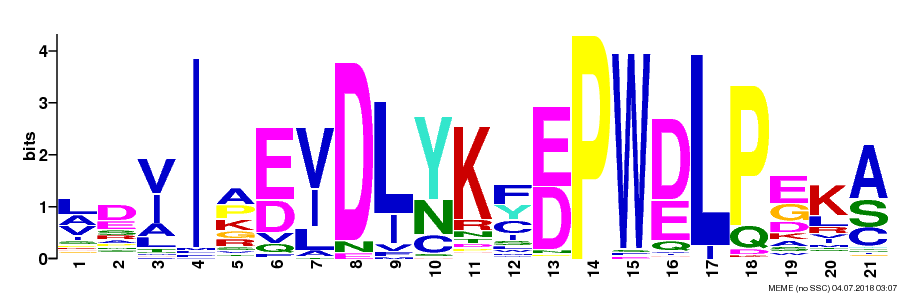 |
| Motif 6 | 2.8e-848 | 150 | 20 | 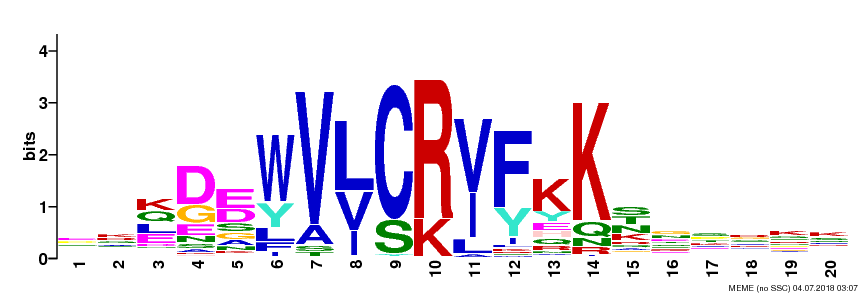 |
| Motif 7 | 4.7e-766 | 20 | 50 | 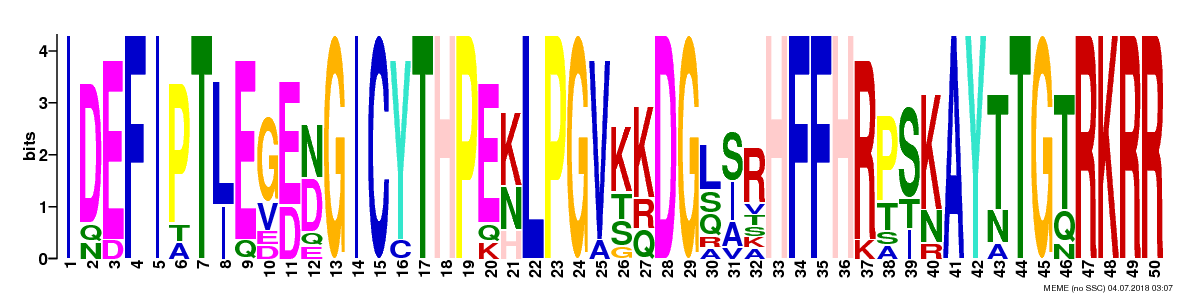 |
| Motif 8 | 9.9e-752 | 116 | 11 | 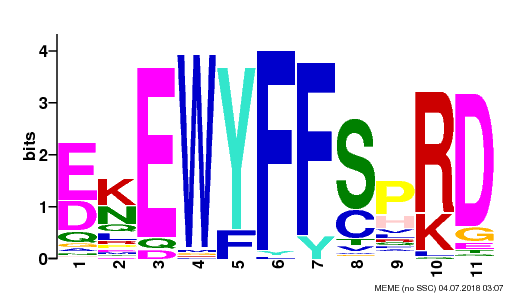 |
| Motif 9 | 1.3e-385 | 155 | 11 | 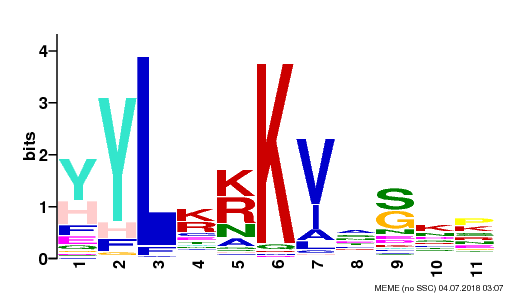 |
| Motif 10 | 8.6e-199 | 20 | 21 | 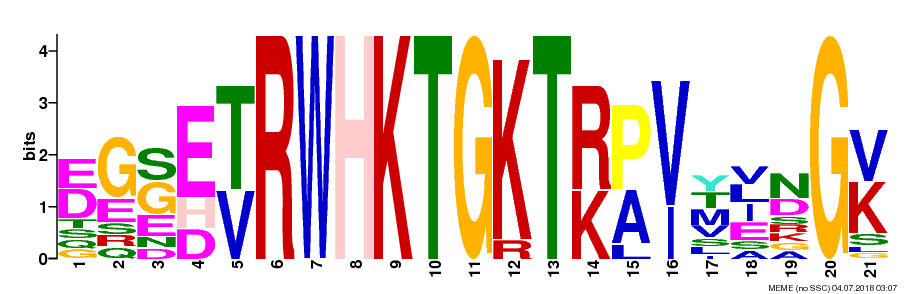 |
