# Supplementary material for: Comprehensive genomic characterization of NAC transcription factor family and their response to salt and drought stress in peanut
Source: BMC Plant Biol. 2020 Oct 2;20:454. doi: 10.1186/s12870-020-02678-9 (PMC7532626; doi:10.1186/s12870-020-02678-9)
Supplement: Supplementary file 11 — Additional file 11. 2500 bp promoter region of NAC genes from two wild peanuts. [file 12870_2020_2678_MOESM11_ESM.docx]

[>AdNAC1 5](#_Toc518555407)

[>AdNAC2 6](#_Toc518555408)

[>AdNAC3 8](#_Toc518555409)

[>AdNAC4 10](#_Toc518555410)

[>AdNAC5 12](#_Toc518555411)

[>AdNAC6 14](#_Toc518555412)

[>AdNAC7 16](#_Toc518555413)

[>AdNAC8 17](#_Toc518555414)

[>AdNAC9 19](#_Toc518555415)

[>AdNAC10 21](#_Toc518555416)

[>AdNAC11 23](#_Toc518555417)

[>AdNAC12 25](#_Toc518555418)

[>AdNAC13 27](#_Toc518555419)

[>AdNAC14 29](#_Toc518555420)

[>AdNAC15 30](#_Toc518555421)

[>AdNAC16 32](#_Toc518555422)

[>AdNAC17 34](#_Toc518555423)

[>AdNAC18 36](#_Toc518555424)

[>AdNAC19 38](#_Toc518555425)

[>AdNAC20 40](#_Toc518555426)

[>AdNAC21 42](#_Toc518555427)

[>AdNAC22 44](#_Toc518555428)

[>AdNAC23 45](#_Toc518555429)

[>AdNAC24 47](#_Toc518555430)

[>AdNAC25 49](#_Toc518555431)

[>AdNAC26 51](#_Toc518555432)

[>AdNAC27 53](#_Toc518555433)

[>AdNAC28 55](#_Toc518555434)

[>AdNAC29 57](#_Toc518555435)

[>AdNAC30 59](#_Toc518555436)

[>AdNAC31 60](#_Toc518555437)

[>AdNAC32 62](#_Toc518555438)

[>AdNAC33 64](#_Toc518555439)

[>AdNAC34 66](#_Toc518555440)

[>AdNAC35 68](#_Toc518555441)

[>AdNAC36 70](#_Toc518555442)

[>AdNAC37 72](#_Toc518555443)

[>AdNAC38 73](#_Toc518555444)

[>AdNAC39 75](#_Toc518555445)

[>AdNAC40 76](#_Toc518555446)

[>AdNAC41 78](#_Toc518555447)

[>AdNAC42 80](#_Toc518555448)

[>AdNAC43 82](#_Toc518555449)

[>AdNAC44 84](#_Toc518555450)

[>AdNAC45 86](#_Toc518555451)

[>AdNAC46 87](#_Toc518555452)

[>AdNAC47 89](#_Toc518555453)

[>AdNAC48 91](#_Toc518555454)

[>AdNAC49 93](#_Toc518555455)

[>AdNAC50 95](#_Toc518555456)

[>AdNAC51 97](#_Toc518555457)

[>AdNAC52 99](#_Toc518555458)

[>AdNAC53 100](#_Toc518555459)

[>AdNAC54 102](#_Toc518555460)

[>AdNAC55 104](#_Toc518555461)

[>AdNAC56 106](#_Toc518555462)

[>AdNAC57 108](#_Toc518555463)

[>AdNAC58 110](#_Toc518555464)

[>AdNAC59 112](#_Toc518555465)

[>AdNAC60 113](#_Toc518555466)

[>AdNAC61 115](#_Toc518555467)

[>AdNAC62 117](#_Toc518555468)

[>AdNAC63 119](#_Toc518555469)

[>AdNAC64 121](#_Toc518555470)

[>AdNAC65 123](#_Toc518555471)

[>AdNAC66 125](#_Toc518555472)

[>AdNAC67 127](#_Toc518555473)

[>AdNAC68 128](#_Toc518555474)

[>AdNAC69 129](#_Toc518555475)

[>AdNAC70 130](#_Toc518555476)

[>AdNAC71 132](#_Toc518555477)

[>AdNAC72 134](#_Toc518555478)

[>AdNAC73 135](#_Toc518555479)

[>AdNAC74 138](#_Toc518555480)

[>AdNAC75 139](#_Toc518555481)

[>AdNAC76 141](#_Toc518555482)

[>AdNAC77 143](#_Toc518555483)

[>AdNAC78 145](#_Toc518555484)

[>AdNAC79 147](#_Toc518555485)

[>AdNAC80 149](#_Toc518555486)

[>AdNAC81 151](#_Toc518555487)

[>AiNAC1 154](#_Toc518555488)

[>AiNAC2 155](#_Toc518555489)

[>AiNAC3 157](#_Toc518555490)

[>AiNAC4 159](#_Toc518555491)

[>AiNAC5 161](#_Toc518555492)

[>AiNAC6 163](#_Toc518555493)

[>AiNAC7 165](#_Toc518555494)

[>AiNAC8 166](#_Toc518555495)

[>AiNAC9 168](#_Toc518555496)

[>AiNAC10 169](#_Toc518555497)

[>AiNAC11 171](#_Toc518555498)

[>AiNAC12 173](#_Toc518555499)

[>AiNAC13- 175](#_Toc518555500)

[>AiNAC14 177](#_Toc518555501)

[>AiNAC15 179](#_Toc518555502)

[>AiNAC16 181](#_Toc518555503)

[>AiNAC17 183](#_Toc518555504)

[>AiNAC18 184](#_Toc518555505)

[>AiNAC19 186](#_Toc518555506)

[>AiNAC20 188](#_Toc518555507)

[>AiNAC21 190](#_Toc518555508)

[>AiNAC22 192](#_Toc518555509)

[>AiNAC23 194](#_Toc518555510)

[>AiNAC24 196](#_Toc518555511)

[>AiNAC25 198](#_Toc518555512)

[>AiNAC26 199](#_Toc518555513)

[>AiNAC27 201](#_Toc518555514)

[>AiNAC28 203](#_Toc518555515)

[>AiNAC29 206](#_Toc518555516)

[>AiNAC30 208](#_Toc518555517)

[>AiNAC31 211](#_Toc518555518)

[>AiNAC32 212](#_Toc518555519)

[>AiNAC33 215](#_Toc518555520)

[>AiNAC34 217](#_Toc518555521)

[>AiNAC35 220](#_Toc518555522)

[>AiNAC36 222](#_Toc518555523)

[>AiNAC37 223](#_Toc518555524)

[>AiNAC38 225](#_Toc518555525)

[>AiNAC39 227](#_Toc518555526)

[>AiNAC40 229](#_Toc518555527)

[>AiNAC41 231](#_Toc518555528)

[>AiNAC42 233](#_Toc518555529)

[>AiNAC43 235](#_Toc518555530)

[>AiNAC44 236](#_Toc518555531)

[>AiNAC45 238](#_Toc518555532)

[>AiNAC46 240](#_Toc518555533)

[>AiNAC47 242](#_Toc518555534)

[>AiNAC48 244](#_Toc518555535)

[>AiNAC49 246](#_Toc518555536)

[>AiNAC50 248](#_Toc518555537)

[>AiNAC51 250](#_Toc518555538)

[>AiNAC52 252](#_Toc518555539)

[>AiNAC53 254](#_Toc518555540)

[>AiNAC54 256](#_Toc518555541)

[>AiNAC55 258](#_Toc518555542)

[>AiNAC56 260](#_Toc518555543)

[>AiNAC57 262](#_Toc518555544)

[>AiNAC58 264](#_Toc518555545)

[>AiNAC59 266](#_Toc518555546)

[>AiNAC60 268](#_Toc518555547)

[>AiNAC61 270](#_Toc518555548)

[>AiNAC62 272](#_Toc518555549)

[>AiNAC63 274](#_Toc518555550)

[>AiNAC64 276](#_Toc518555551)

[>AiNAC65 278](#_Toc518555552)

[>AiNAC66 280](#_Toc518555553)

[>AiNAC67 282](#_Toc518555554)

[>AiNAC68 284](#_Toc518555555)

[>AiNAC69 286](#_Toc518555556)

[>AiNAC70 291](#_Toc518555557)

[>AiNAC71 292](#_Toc518555558)

[>AiNAC72 294](#_Toc518555559)

[>AiNAC73 296](#_Toc518555560)

[>AiNAC74 298](#_Toc518555561)

[>AiNAC75 300](#_Toc518555562)

[>AiNAC77 304](#_Toc518555563)

[>AiNAC78 306](#_Toc518555564)

[>AiNAC79 308](#_Toc518555565)

## >AdNAC1

AATTTGAGGCACGCGTTAAAATTTTATGATACAATTTAGGTCAATTAAAAATTCAAGGGTTAATTTAAATCAAATTAGAGATCAAATTTCAAGAACTATTTTGAATATCAACTCATTAAACTAGACATTCCTTTATTATTTGATGAATGACTTGTAGATAGTATAGAAATTGTTTCCGTTTGTACAAGAAATTAAATATATTTTGCATATGGTAATATTTAGGTGTATTACGAAAAAATGTTATAATTTTATTACTTTTTCGTATCTAGTGCATATATATGAAAACTTATCACGGATGATTTTTCCTATGACCATATTTTTCTAACGTAACGTTTTTAAAATTCATCTTCCGTGTTTTCTATTTAAAAGAAAAATGAATCTTTCCCAACAAATTCTATTTTTCAAAAAATAAAAAATTGAATTCGTCCTTACGGAATTGTTAATTTTGAAAATGTTTTCGACAATTTTATTTTGTCTCTTGAATTTTCAAAAATTACAAATAATCTTTATTTTTGGAGATTTTGTCCCTCCGCCAATCATATTAAATTTTTTAATCAATTTCCAACATTTTTTTAATTTATTTTTCTTGATGAGATTGTGATCTCCTCACATCCTATATAAATAAAATAGATTATCAATAGTCACCACCATAAAAGAAAAAGATAGGTTGCTTATCATCTAAACAAATTAAAACTTTGGTGGAAGAGAGATTTCAAAGTAGAATTAATAGGTACAGGAAAATATGAAAAGAATGTCGTCACTCTTGCTAGTTGTGGATTGATTTTGATATGTCATGACATGCATGCCAATTGAATATATAGATAGTGTTTTTCATATGGCGCAGACACCCATCTTTATCTTCCCTTATTAGTTGAAATAACCTATTTCATAAAAAAGAAATTGATTCTCTAATTGAATCCATATTATATTATCAATGGTTTTAGTTTTATATATATAAGTTTCTCCTAATCATCAATTGACCACAAAAACAATTTTTCTTTAAAAAAAAATTGTGCTACCTCGTAAAAAATAAAATTTTTACCAAACCGAATATTATTTATTTAATTTCTTTACTTTTTTCTCATGTTAGCTACTACATGCATAGTAATTAAAATTTCAATTTAATTTTTTAAATTTTTTAATATTACAATTTTAAATGACTTTATTAATTTTATAAAATTTATATTAAATTTAACAATTTTACAATTTAAATATTATTAACATTTTACATTTCACTTCTTAATTTATTTTTTTAATTTTATATAAAATTTCGATTTTCTCTACCTTAACCACATGCATGTATATCGACTATCAAAATGTATTATTGTTGTATGTTCATATAATATGACTCAAAATAAATATTTAGTATTAGTTCAAAAACTATAATTGTTAATTGAAGGATTTATTTTTGGAATTAGTTAAAGAGTGTCCAGAGGATATTAATTGAAAAAATTAAAGTAGTAATAAGTTTTTACACTTTTAATACATTAAAAGTTTAAAAAAGTTCGAGGTGCAATGAAGAGATATAAATGTCAATTAAGGCTGCCCAACAAAATACAGTGATGAACCTAGTCAATAATAAAACCAACGGTATATATACATATATAGATAGATATTATATTTTGGATGAATACAATTTTTACTTTTTTGGTTAAACGGATGAAATACATTTTAATATACATCTTCTTCAAAATTTATTTATTTTGGATCTTTTCTTTGACTTTTTCCTTACGTGGTTATTTATGAATGTACTCTATGTCCCACACCATCCAGATTTTTAATAGTCAAAGTTGTGCCCACTCTCTAGACTTTGTTTTATTTGTTGTTGCACTAAAAGCTAGCCATGTTTTACAAATTCTAGTCAATTGGATTTTCACCCTACATTAATTCTTTCTTCGTTCTTATTATTTTATACTTACAATATATCAAAATTATATATATTTAATTTTAATGTAGTATCAGTATAAAATAATTTTACATTTGTATCTAATTACTAATACCATATCAATAAAAATAATTATTTTTTACATTGATCGTACAAAAAAAACGAATGTAATTGTATGACTGTGTTACACCGCATCAAAATTATATATATATCGAAAACTTTTAACTCAAAATCGCTAATTAGTAAAGAAAAAACCGCCACTGGAAATAACTATAGTGGTCTCTTTTCAACTTCAATTATTATTAATTAATTAATTAACCTTTGGTGGCTTGATGAGACCCCCCTTAAGAAAAATCGATATATGTAACATTAGGTCAAAATCTATTGTAAGCTATAGAACTCTCTTTTAATCAAGGCCATGATGGGGACCCCAAGAAATGGAAATGTTTGACTTGACTTCATCACCTAAATATTCAATACATTTACACACTATAAGAAGAAATGAAAATATTATATGATAATAATAATATGTTAATATAAATATAGAGAAAGCATAGAGAATAGTTTTCACATGTATGTGTTGTTTCACTTGAGACTTTAAGTGTTTTTAGAGTC

## >AdNAC2

ACCAATTAATCTGTGGTGTAGGTTCTAGCTACAGTAAATCATTGGTAGGATATAATTCATTCTATTTAAATTTTCCTACCCTACCCTTTGATGTTGACCAAGAACAAAACAAAAAGAAAAGGATGAAGTGATAAAAACGTATGGGAGTAAAACTTGCCTAACTCAGACCCACCACCACTCAATTATCTTAACATTCATCCTCATAGTCAGCTAAAACTAGCTTACTCATTCCATTTGGAGCATTATAGTGCTCTTTTAGCACACTTTTGATTCTAACTGAGGATATATTATACTATATCAGTATCAGATACAAGAAAACAAGAAAATATATTAGTGAACTTGTTATTATTCTGATGTATGCATTATCTTTATTAATGTCATTAACCACTTCAGGATATGGTGGTGATATTGAAATATCCCAAAATGCAAATTATGAGTCTGAGTAAACTTGTGAGTGCTCCAACAGTGGTAGATAAATGTCATTTTTTATTTATGTCAATATATCTATGTATAGCTATCTATATCCATAAATCTATATTATATCCACTATAGTGGAAGGGTAATTATATGAGAATAAGGAATCAAATGTGGCTAATTAATGACTGAGGAGGATCATGTTAGATATTTCTATGAGTCTATCTTGCAATCTGTAGTTTTCTTAAGAAAAGGGTAGCCATGAGGCTGGAATTGTGTATACTGCTTTACAGTCACCTCCTTGTTTAGTCTTTTATCTCATGATAACCCCACCATGTAAGATCCAGCAGATATGTTAAATTCTTCTAAGAATAATATTTTTAGATATTTCCTTTAAAGAAAATACAAAATGCCCCACAACTTTTTCCATGGGTCAATTACACTTATCTCTTCTATTCTTAGGTAAGATTATACATGAAACTTTTTCACTTGTAAAAATATGTATTAATTACTTATGAAATTTAGGTTATGTCAATGATTGGTATCCTTATAGTCATAACAACTCAAAAAAAGTCATATGTAATATAGTCTAATCTTACTAAAGATCTCATTGTAAGTTAGTCCTGAATAATTTACATTAATTTGAAAAATCTCTTCCAAAGATAAAATGGTACAACATACTGAAACAGAGGACTATTCTTTGTTGTGTCCCCATATAAAGAAAAGTTCTAGAAACAATTAAGAATGTGCATGAGCAATTTCTTTGTGGAAGAAGCACTGACTCCCAATAAGATGGCTAGAGTAAGTACCAAAGGCACATGGCATTGTGGTTTGTGGATGAGGAAATAGACTCAAAATTTTAGGAACTTTGCTCACTGAGCCTTTGTATATGGATGGAATTTGCCTTTTAAGTGGAAGGCTGCTTCTGAATTCTTGGTCCACTTGAGCCCAGAAATAGTACATCCTCCACTTCCACATATATCTAAGATGTGTAACATGTTCTTCCCAACAAAATTTGGATCTCTCACATTTTTTTTTTTCTTATAAACTTTTATATACTGTGTTGGTGTCACAAACTGTAGCCAGAATTGGTACAAGCACTGTCTGAAATCAATGGCAAGCCTTGGATATGGAGATCTAACATGGTACTAATAAATTAACAGAATTGTTGAAACTACAAATTGTAAAAGAATTAGAATTTGGAAAGCCATTGTCTTGGTGGTTGATGCTCTTAGTTTGGTTGCACTCTCTTTTGGTACCAACTTATAGAGCATATGTCTACTTCAGTAATAATCCAAATAGGTCATTATGCCACAAAAAAAAGGTACTTAGGTACCCTTCAATGGCCCCCAACATCATGTCTTGCAATTTATGAAGCACAAGATAAATAGATAATGACCTACTTAGAAGACTGCATACAGTAATTAAGTAACACTCTTACATGCATAAGCATAATAATTAACTATGAAGCATGATCATAATTGGAGTTAGAAAACCTTGGCTTAGTCATAAGAGACAAAATAATGATGTCTACTTTAATGGAATAAATATTATTGCTCGGTGCTGACTTACTAAATCAGAAGAAGAGATGCAAAATTAATGGTTGGAAAAGTTATAAGCAAAGTAGTAGTTCATTGTTAAATGTTTAATGTACTTGTTTCTTTCTCCTGCAAGATTTAATAATTGATTAATTATATAAATAAATAAATAATGTGGGGTCCAGAAAATGGGCCAAGGAAATCAGTTAAACTCCCTCTTCCAAAGAAATAATTGATGATTGCTTTGTTTTCATGTGTGGGATGGGAGATATGTTATGTGCAGTTCCCACTTCCCACTGTGCTTGCTACTCCATAGAGCAAAACAAAAAGCTCTCATCTTTGTATATTTAAATCTCAAAAACATATGATAATTGCTAATAATTATGAATGCACATACATATATAAATACACTCCAATTAGGTGGGGGCCACCCCAGCTCAATTGGTCTGAGACCAAACCCATACCAATGCATGACACGTGGAAAACAGCAGCTTGATGATATCAGCATGAAGTGTGATGATGATCCATATACACAATAAAAATCACCT

## >AdNAC3

AGCTATGGCGACTGCGTCTCCAACTGTTTCTATGGACATTTAAGACTGACCCAAATTTTCTCAAAAGTTTTATGCGATCGTCAATAATTTTGTATAAAAAAATTATAAAGAAGAATTTTGTTAGTATAAAACTTCGGCCGTCCAATGAAATGCGGAAATCCCAAGAAGCTTTAGTAGCTATTTTTCCACACATTGCTTTAACAACAATCAAATAACCTATATTTTTTTTCTCGGGATCGTTTACTGATTTAATTATTTGCTGAAAACTGGTTAACCTATTTTTGTAGCAAGATTTAGTTGACAGCCTTCTGCCACGCGCGTGGATTATACTATATGCAACAATATATATATAGCATCAACATGGTAAATTTTATATTATCCTCCCTAAAATAAAAAGGTAAATTACTCTTTATAATATATATATTAAAAGTGATTCACCAAAATGAGTTTTATAATATAAAAGAGATTTTTACAGATGCAATTGATGCACTTAAAGGTAAAAGAAAACTTATGATCTGTGATAATAGAAGAAGTCATAAAAATATTTGAAATGATTCTTTATATTTTCAAGTTATAAATATAGTTAAAGACATTGTTGTTGATAATGTCAAATACAATTTTTAAGGGATTGTAGATTTGGATTCTCATGAGATTAAAGACATTGTTGTCGATGAGGTGGAGATAATAGGATTTGCAAGATGAGGTGACAATTATTATGTATAAGAATTTAATCACTTTTATGGGACAAAAATTTTGTATATAGTGAATTTCGAAAGTGTTATCACCATACAGAAGAGAATGGATAGGAGGCACAGTAGAGATTCTTCGTCATAGACTAGTGGTGCTAAATTAAATTTAAATAATCACAAAAGAGGAAAAGAAAAATAAAGAACATATACATAATTATTATGATTAACATTCAATTTAATTATAATTATAGATACTATATAAATTACAGAGAGTAATTTACCTTTTATTTTATAGAAGACATAATAGCATTCACCTATTATTATTCGCTATTATTATTCATAGACTATACATATTCTTTTTCACATTTGATATATATATATACATACCCTATACATTAATACTGGTATGATTGTAAACATATACAATCCGAAGGATTTTTAACCGCCGCTATCTGCCGCTCCGATTTGTAGTGATAGCTAGCATGAAAAATCGTTATAATGTGAATAATAGTGGCCATGGTAATGTTGGTGCTGACCTTTAGATAAAAAATACAATGTCCTTGATTTTGCATGGTAGCTCCCCGTACTATAGTACTATATTCTCCAGTCATTGTTGGGTTTTATTTGGGGCACGAACCCCTCTTCCTTTGAAGTATCGAAAATTATCCTAAAGCAGAGGGTTTTGACCAAGTTAGCTTTAACCACAAATTAAGCATGTTGCTTCTATATACCTTAGGTTCGCATTAACGTAATAATTAGTTCGGACAATCACACGGGTCCCTGATAACATAAATTCCACCATATTAAGTTTAATTTACTGCTAACATAATATCAAGATAGGGAAACGAGACATCATATATCATACCTTTACGCATAAGCACGGTTCTGATAATGGTGCACACGAGCAAATTAAATTTAAGATATTTAAGTTTCATTATTAATTAACGGCTTTGCTGTTAATAATCATATCGTCGTCAAATTATTAGTAGGGCATGCAAATGTATTCATTACCTTTAAGCATAAGCACTGAGATTAACTATAGTCATAGCTGCACCATATATACCCTCCATTATTTGTACTCTAATATTTTGATTTCTGCTAATAAATTAAATATACCACCTATTTTCATCTTTCGCATTATTAATTGTAGATCAAACCAATCAGGTTTGGTTTTTTTTTTTTAACTAAAAACAAAGAGACTCGAATTTGCGACTTCTTAGATGAGTATAAAAAGACTATATTATGTCATTTAAACTATAACTTGTTGATAACCAGTCAGTTTAATTTAATAAAAAATTAGTTATTAGACCGATTCAGTTTAAAATAAATATTAATTGGTAACAAATTAATTAAATTAATAAAAAAGTCGGATTGAAGTAATTGGTTTAAAATAATAAATTATATTTTTGTTAATTATATCCAATTCAACTCCATGCACATGAATTTCTGAATGTTTAATTATTATTATGAAAAATAATAATGTAGACTAATATACGGATAGGGGAGTGTAGCTTACGCTCCTTATACGCTAGTGGGGGAAGAGGTATGATTAAGAAGAGAAAAGAATTAAGAATAGAAAAATAAGAATGATAGAGATATAGGTAAGAGATAGTCACATTCATTTGGGCAAACTTGGTTTTTACGATAAGTAGCTTTAACCATTCCCGTCGGCGTAGTCACCGGCCGTCAACCCTTTCTCTATAGTTACATACAGTGGGTGGCGTTGACAACCATGACGGTATATAAGTCCCATGCCTCAATCACCACCTACAAAATTTATTGCTAGCTTCCATCATCATCTATCACTTTCC

## >AdNAC4

ATAAGTAAAACCAATTAGGATATTAAAGTTTTTATTTTTTTCTTTTTGTATTATTTAATTATAGATATATAATTTAATTTCCAAATAATTAAGAAGCTTTAATTATTATTACACAATTCTTATCCGGGGAATTAAGCACATATATCTCTTTATTCTCTATGCATCTGCCGAAATAAATAAAGAAATAAAAATACTATGCTTATTAATTTATAACATCAATCAGTCGTGATGATTTCTATTAAAAATTGTATGTAAAGAGATCAATTTAAATTACATGAGATTAATTAGGGTATATATTTAGAAGAAGGATCAAGATACCAACAAGTAATCGGTGCTACTATTTTAATTAAAACATGATTTTTTTTTTCTCTTTTATCTTTTTAATACATTTAATATATCAAGCGTTAAAAAATAATAATAATAATCAATCAAATCTTCAATATTCTCAAAGAAAATGTTATTTTTTGGGTGTTAAGGGAAAAAAGAACAAGTGATGGATATATAAGGATTTTTTAATTTATTCACATGTCAAAATTGATATTACGTATAATTCAGTATTTCAAACTAACAAGCAAAAAGTTAGTTATTAGACCAAAATAACCATAATAAATAAACTGACACGTATTTTTATGTTTAGGCAAAGATTAGATTTAAAAGAGAATTGTTTTTTTGTACATTGAAGGGATTTAATCTAACATCAGAAATTAAGGTGAAAAAGAATCTATCTCACAACTTTAAGAGTACTTAACAGTTTAATTAACCTATCCTAATTGGTGACCACATAACTACGAGCTGGAGTCACTAAGCTAAATTAATTACATACTTTACTTTTTATCTTCCGTTACATTAGAGCTGGTTATAGCATACATTGTACCAAGAAGATGTCAAATTGTCAACGCTAGCTTATCTTTTGCTATTTGTTTTGTTGTTACAACTTACAACTAGCTTGCACATATTCCCAAAACCGTCACATCCAAGACTTCTTAAGGGGCCAATGCTTAAGAGAAATTGTGAAAAAAAGGGGAGTAATTAGGATGTAAATTAATCTTAGGGTTTACTTTTTTGTTTACCAATAGAAAATATTTTTAGTGTATTATTATTATTATGTTTTACAAATTATAACATAAAGTGAAAATAATGTGATGTTCTTACAATTATAAATAAAAATAAAAAATGACAACGAAAACAGAAGATAATACTTGATATCTTTTAGAGGAATTTTGATATTAAATTTCGAGTAATTAGATAGAAATTTAAATGCAGTTAATTTTATATAAAGTTGATAAATGATGATCGTTAAATGAAATAAACAGATTTAATTAAATTGTCATCTAACGATTCTTCAACTATCAATTTTAAATAAAATTAACTATATTTAGTTTTCAACAAGTAATTAACCTTATAAATCTCGTAGATTGTAGTTATTAGCGCGTTCATTAATAGACACCTATAATGCATATTGCGACATTTTCATAAATATCAGGCCGTTAATGTCTACTAATTAAAGTAGAAAAATTGCACTCTCTAACTCTGGTAGCTGGTAGAAAAAATAAAACTTCAGAAAGAATAATAAGACAGAATAAGCAACTAAACAAATTATTACCGATGCGAACATTGTGATAGTTCGGCATGATTGATGAGACTAATTATAAGATTAATATATATAATAATGTTTATGATACTTAAGTAAACAAATAAAGCCATCGACACAGTATATACTAGCTCTCTCATAAGTCACTATTGGATTAATTTGATGAAAATAATAAAATAAGAAGTCCAATAATTTGAAGCATTGATAAATATATGACATGGAATGAGTCTCGGATTAAAAAAGATTCATGTACTGCATGTGAACTGGTGGAGTACATACATATACATACAAAATTAAAGATTTAAAGGTTAAACCTAATTGACACACTAACGATACTAAAACAATTTTCCTGGATCCAACAAGTTTATAAACCTAATGATACGATGAATATATAAAAGAACAAAACAGTCGCATAGGGAGAAAATCACGAATTCACCAATTTTACTTTTTTTTTTCTCAATGTTTACCTCATGTATGTGTGGCATTTATATCTAATGTCAATTTCGTGAGCTAAGAATTAATTAATATTTCATTAAAAGTTAGTTTTGGGTAAAATAATGCTAAACTAGCTAGGCCTAACCTTACAAGATAGAAAAAGAAATAAAAAAAATAAAAAGAGTAGTATTAATAGTGTTAGTGAAATTGAAAAATGAAAAGGGAGAGGAAGGAAGGTAAGAAAGAGCACACGAGTGTTTGAATATGTAATAAAAAGGTATAATATTTGTGTGGGGTAAAAGAGACACGAGTGTATGGCTTAGGTGAATAAGAAAAATAAAAATAGTGAAAATAGTTGAATATGTATGTATGGTGGAAGAAAGGTCCAACGGCACCGCTCTGGGGCTGTAGCCACCCTGTCTTGTTCTTGAGATGTCATTTTCTGATCTTAAAAACACTCTTAGGGCAAGCATA

## >AdNAC5

GTTATATCAGCATTCTGTTTATCTAGTTATAGTCGGAAAGAGTCGAGAGACCATATTGGTGCCCAAACTTGAATCTGAAAGACTAATATAGATAATTTTAAATTTTAAGAACTAAATTGAGTAATCGCGTGAATCTCAGACACCAAAATGGATATTTAGTGAACTTATAAGTTATAACACAAAATCGGAAAGACCTATTCCCCTGCCGTGGCTGCTCACGCTTCGTGATTATTAGTTTATTACGTATTTATTTCTTTTTATTTTGATGTGAGGATTACGTATTATTATTTCATTGAACGTCATGATGATGCATTATTTAATTGCTCATATTTCGCTTCGAATAGTTTATAATGAACTCCTCTCTCTATTTCTCATGAGAGATCAATAATGCTTCAAGGTTCGTGGCTCGTGAGTTGCATGAAAATGTATACGAAGGATTAATATTTTTTTATTAATATTAATTAATATTATATTTTTTAATTACTAAAATTTATAATTTAAACTTTAGTATTCAAAAATTTAAAATTTAATTAATATTATATTTTTTGAATATTACATATAAAAATATATTTATTAACTAATTATTAATTTAAAATAATAATTTTTATTAATCATATAAAATTAGTTATTCACATTATCAACAAAATTTTAAACTATCTTTGGTATTTTATTTGTTTCAATTTTATTTCCATTTCATTTTAAATTTGTTTCAGGTATGTCTTTAAAGAGTAATATTACCAATAGAAACACCATTAATATCTATGGTGAAATCACTTTTTTTTTAATTTAAACTGATGGAAGACNTAACACATTATTTAATATAATAGTCACTTTTCGATCTACATAAATTTTTTGCATTAATTTTTTTATTTATTTTATGCTATTTTTTATTTTTTTTAAAAAATAAAAGAATAAAATTTTATTTTGATCATAAAAATTTTGATATTATATATATCATAAAATAATTTTTTTAAAAAATTTAAACTGATAAAAAGAGACACATAAATAATTATATCTTTAATATCAAATTGAAGTATTAATAAAATGTGGTAAGCTGGTATATTATTAATTAATAGGCTATTTTAATATTACTAAGTCATATAACATTGACGCCTGGGAATATAACCAAAGCATATTTAAAAGTATATAATTATATATTGTATATACAATACATATTATTTATTAGTAATTAAGGAACACTTTTAAATATTTAATTACAATAATGTATTAAGATATGTACACTTAATTCGCGCAAAAACATACTATAGAATCCCATTAATTTGCTTCTCATTCTATCTATAGAACTTCTTTATTTCTTTTGATATAATTTACGTTTAAGTTATATTTTTCAATTTTACTAGACATTGGAGTAAAACAAATTTTTAGTGTTACAAAATTGCACCTTTAGATGAATAAAAAATTATCTACAAAACTTAGGATATAGTAAGTAAATCAGAGAATTACTATACTTGTGGTAGATATTATTTGATGAGAAATGTTAGAGAATATTAAAATTTATTATTTTTTGTTATTAGTTAGTTATTAATATTTAAAAATATAGAATAAAATATATTATTAAATTATTAAACTAAAAAAATTAAACTAAAACAAAAAATAATAAATTTTAATAATTCCTAATATTTTTGTTATTTATTATTTAATTTATGTGGTAGCTAGGCTTTGTGATTTGATTTAATGAAGGACTTGTTCCACCTATAAGATGATCTGGCCCAGCAGATAAGCAAAAGGATATGAGATTGACGGGTTTGGGCCTTTGGTGACTCGTTCAGCGATTCCTTTATGGGAGAGTCACACTCACATTCACATATATGCGTCCTAATAAGCATAACTATACCATAAATAATCCATAATAATGCATTAATATTGGGGGCAAGCTAAGTAAGACGCTTTCTTGTTCCCACAACCGCGTGGAAGTTTCTACTTTCTAACATACATACATCGTCTCAGTTCAGAAGAAAATAATAAATAAATATACCTAATTTTACTCGGTGATTTTTCCATCTATATATATATGTTTGTCCCTGCTAGCTGTGCTTCCAAAATGTGCAACAACACATAAAGTAGTGTAAGTACACATCACCATTTTAATATAGTATATGGAGGATCCACCAACTGGTTTTCGGTTCTATCCAACAGAAGAAGAGCTAGTTGCTTTCTACCTAAACACCCAGCTTCAACTACAAGGCCACACGACTCACATCAACAGGGTCATTCCAGTGGTTGACATCAATGGCGTTGAGCCCTGGACTCTTCCATGTACGTAACCAAAACACATATATGTATGCTTCTGTATTTCTCTGTCTTAAGACAGTTAGTTATTTTGTATTGGTTGCAGCACNNNNNNNNNNNNNNNNNNNNNNNNNNNNNNNNNNNNNNNNNNNNNNNNNNNNNNNNNNNNNNNNNNNNNNNNNNNNNNNNNNNNNNNNNNNNNNNNNNNNNNNNNNNNNNNNNNNNNNNNNNNNNNNNNNNNNNNNNNNNN

## >AdNAC6

GACTTCTCCAGCGACGGACCCAGAGATCAGCAACTACAGCATTGGACTGACGCTGGTTTGGGTGGCTGGTCAGATTTTAGTGGTATCCCTTCACCGTCAGTGCCGGTTGATCCACGTAGGGCATCAGTAGATATGGGACATGGTTTGCGGGGTCGTACTTTCAACCACTCGTCAGAGAGTGCGTCATGTGATAGTGGTTTTATCCAGCGTCAGCGGGCATACGCAGTTGTTGACACGTTCAACCCCGGTCCATCCGCTCCAGCTGAGGCAGGCGGGTCGACCGATCCGACAGAGGCAGGTGGGTCAGCTGCTCCTACAGAGGTAGGCGGTTCGGGAGCTCCGGGGGTGGATGACTCAGTTCGAGGTCACCCATATGACCTACGGACGGAGCGAAATTCACCTGATAGGTATACTCCATCACTGTTAGAGACGGGCATTATGCGTTAGGGACTGCACTGGTGTTGGAAAGAAGTGAGCTTTGGTTGTTAACTTAATGTAAATCGTGTTTAATGTTTGGTTTGTTAACTTATGTAAATCGTGTTTAATGTTTGTTTTATTAACTTATGTAATCAGTTAATGTTTGTTTTGTTAACATATGTAAATCGTGTTTAATGTTTGTTTTGTTAACTTATTTAATCAGTTAATGACAATGTTAAGCGACCATTTTAATATCAATGGATAGACTTCATTGAAAAATACAAACACGTCTAATTTCAAAAGCATCGACTAACAAAATACAAATTAAAAAATACAAACATGACAATATTAAACTACGAACCAGCACCACTCGGTCTAGCACGCTGAGGACACCTACTCTGACTATGACCCTGAGCACCACAGAGACGGTATATCCGAGGACCACGCATATCACGTGAGTCCATTTCATTCAAGTATCGGGTCAGTTTGGGCCTGCCTTTAGACGTTCGCCTCTGCCCGGGATTAGCGACCAATGTGGGTCCCTTATAAGCAGGCCATCTATCGGGATCACCTAATGGTGTGAACTCAAACCTATATACTTTAGGAACCTCTGTCATCTTGTACACATCATGCACATACAACTGCCAATTGAGATGCTGGTTAGCACAGCAAGCAATAACATGGCGACATGGTATTCGTTCAACCTGAAAGTGCCCACAGTCACACGTCCGTCGCGCAAGATCAACAACTAGCACCTTTCCAGTAGTCATTTCGTGCACCTCAAACACCTCATTTCGTCTATCAAAGCGGTGCACAACTATATTCCCAGCCTGTTGCATACTTGCTTCTATCCGCTGTTGCGCAAATGCGGAGTACGTATATCCAGCACGCTTGCGTTCATGAGTCTTGGCACTCTTCCATGTAAAAAGTTCATTTAACTTATAATATATTTCTCGGACCAACGCCAACACAGGTAGATTACGGGCACCCTTCAACACTGAGTTAATGCACTCGACAAGGTTCGTTGTCATATGGCCCCATCGATGTCCCTCGTCGAATGCCAATACCCAATGTCTGAGTCCAATGGCATCGCACCACCTGGCATATGCCTCGCCTCGCTCCTTCATGAGTATTACGTATTATCTCACCGTTGCGATACACCACCAAATTTGCGGTGCCCTCCATTACACCAACAACAACCTAATTCTCATAACACACTCAAATCTCACTCAATATGGAAAACACTTTCGAGCTCTACCTTATATAGAGCAGTGCACTTAACACGCCCCCCACGTGCTGTCTGCCTCAACCAATCACGCTTTGACATGTGTCAGAACGTCCCTGCGTGCTGAGTCATCACGCCCCCCATGTGTTGCATACTTGGACTAATCACTCCTCGCCACATCAACACGCCCCCCACGTGCTGACTCATCACGCCCCCACGTGATGCACCTCCTGGCCAACCAAATTCTGCCACGTCAGCACGCCCCCCACGTGCTGACTCATCACGCCCCCACGTGCTGCATCTCCTGACCAACCAAATTCTGCCACGTCAGCATACCCCTGCGTGCTGACTCAGCACGCCCCCTGCGTGCTGCTTAGGTGGCTCAACCACAGCATGCCACCTAGCCTCCATGCCCCCCACGTGTTGAAGGCACCACGCCCCCTGCGTGTTACCCCTTCATCTGACACGTCCATCTATTTGTAATACACACAGTTACTCCATTTTCACCCAATACACCAAAATATTTTTCATATTTAAATTAATGAACCTAATTTTAAAATATTAATATTTTTTACATTATTAAAACACTACAGAATTTGCCTTAATAAGAAAGGAAGAGAGGGAGAAAGAAAAGAGTTGAGTTGTACAAATATTTTGATTTTTTCACTTCACTTCACATCTCTGTATTGGAAGTTAGTTCTTTCCTTCTCCTTTCCTTTTTTTTATAGTTAAAAAATGTTCTCCTTTCCTTGTTCTTTTTCTTTCATCCATTGTTTCCCTTCCTTATTTATTTTCTTTTTCCTTTTTGCAAGTTAGTTCTTGTTTTTGCTTCTATACCATTAAGCTTAGATTTTAATA

## >AdNAC7

TCAAGTAACCACCAAAAAAGAGTTCCATCTCTCTCAATTAATAATGTAACAAACAAAAAGACTCAGAAAATAAGCATAACGATATATTAATTAGATTTTTAAAGTTGTGAGGTTTGAATGGTCATTTTAGTTTAATTAGCTTGCTTAGCTTAACTACCTCATTGATTTTTATTACCATCACTTTGAGAAACACATTATAAATATAACTATTATAAGATAAATAAACAAAAATTATGTAAAATATTTACATATTTTTAAAAAATATAATTATTTATGTATGTTTTTTAATCAATTTAACAGAAAAAAGTGCCTTCTTGACAACTACTACGTGGCTAATTAAAAGAAGTGAGGATCGAATGGTCATTTTAGTTTAATTATTAGCTTCCTTAGCTTAATTACTTCACTGATTTTTATTATCAGCACCTAGGATCCGGAATCATGATAAAACTAACGTCGTCCTTAGGCAGCTGTAGCTGCATCCCAATAACTCGAAAGATATAAATCGGTAATTAATTAAATAAATTAATAATATTGCGTATGCTGTTAGGCGTGGTGGTGAATAATGACTATACTTGATAAGAGAATTAGAACCCCTAGGTTTGACACTTACACTGAATTTGATGCAAATTCTTCACTTTTATGAACATGATTCAAGGCCTGCCGCTAGATCTCTTCGTCCTTTTTGATTAGTTGACTGCAGCTTTACATTTGATGTAATCACAAAAGTAATTAATTATTTATTTATTTAATTAATTAATATTTACAGATATGAGTGCTAGATAGCTCCTCCTCATATGGCTGATTATAATTAACAAGATATAAAGAAGCACACGCTCGTCACTTGTGCTTGTTCAATTATTCAAACTCATGCATCAAGATATAATTAATTATTAATTCTTGATTACTTGTGGACCATGATACAATACTTTTTAAATAATTAAAAGTCATTGTGAATTATACAAATATCTATAATTTTCGTAATTATTAATAATAGTATTATAATGTCACCAAAGGTGCATGTATTGGGAATATTGGAATGAGCTGAAAATGCCTTATATCATTCTTTCTTTGACTTGGACTAACTGTCCGTGAGAGAGGGACTAATTGAAGACAGATCAGATCTATTTTCATATTGGTAAATAAAATGAAGAATAAAAAAAAATAGAAGTTACTGTTTATTATTTGGATTTAGTAATAGCCAGTTAAGCAGATTTTCTTCATTTTAATGGGGCGAAATTTAGGACTAAGGTCTCCTATTCAACGGACATTTACCAAAGCCTATTTCATATATTATTAACAAATTGTTTCTGTATCTACCTATCTATAATAATGGTTCATATGATATGATGATAATATACATATAAATATCTATAAAAACCAATAATTTACCCAATGGAAAATTTAATTTATATCGTAAGGCATAGTATTCAAGATTAAGCTGTATATATTAGTGCACACTTTTATGATATCATCATAAGCCATATCACAGCTATATAGCAGCTCAGATACTTAATAATTTACTCAACTCTTTGTCATTCATCTTCTTGTATATTTGGTTCAACAACACGAAAGCAAAATTAAATTAATTTGATACAGGTCGAAGTATATCTTAGGTTAAGCAAGAATAAAAAGAGTTAAAGTTATATTTAGATAATATTATATGAATAATATAATTTAATATTTGAGTTTTTATAATTTAATAGTTTTTTTCACTATCAAAAAAATTATTTTAGCAACTATTTATCTCATTTTTAGGAGGAATTAAAATAATCGCTAATCTATTCACCGATAATTAAATATTAGGTCCTATGTTTTGTCATTAAAATATTTTAGCGATAATTATATAATTTTTAAAAAAAATTTACAAAAAATATATAATTACTATTATAACATATAATAACAATAAATATATAATTGTCACAAAAACATAAATTAAATAAGATATATACGAATCATTATATTATTATTACTAAAAATTTGACACTAAATTATTTTTTTTTGTAATGTTTATGTTATGATATTATTTTTTTAAAAATTTTAAGATAGTAAAAAATATACATAGTAATTAGTTAAAAATTTTGAGTTTGCGTAAATTTTGTATAAATATTTTATTTTATATTTTTTTTTGAATTAATAAGAATTGAACATTAAATCTTCAGATCATAAAAAATTTAATTAAAATATTATATTATTTTTTAAAACTTAAATTAAAAAGAAGTATATAAATAGCTATGTGTCTAACATTCTAAAAATATGATAAAACAATAATGAAATAAAAAAATGTTTTTATATTTTTTATATATTTCTGTGCCATTCTCGGTTGTCAGGCCTTGTTAGAGAAGCTAGCATATTGCACTGATCCACACACACATTACACATCTCCCTCTCTTTCTCTCTCCTAAAAGTTAAAAGCCACATACATACATAGATACATAGATACAGGTCCGTGCCCTTGAAGCTAAGAAACTGTGCAAACTAAGCCTTCTCTTTGATTGTTTGT

## >AdNAC8

AGGAAAAAAAATTAGAAAATATTTAAATTTTTTAATAGTATAAAATTATGTGTTTGGATATTTTAATTGAAATAATTTATAAAATTTAAAGAATTTTGATAAATAAAATAAAAATTTCAATTTCCACTTGTAATGAGAAAATTTTAAGTTCTCTTTTTTCTATATCTCACTTAAAATTAACAAGAAGGAAAGTCTAAACATTATTAAAGAAGAAGAAAACAAGTTGGCAATTCTATTACCTTATCCATATAAAGAATTTTATATATACAAGTAGTATATTAACTTCATCCAAATAAAGAATTTTGTAATTAAATATTAAATTTTCCATGCATTTAAAAGGTTCCTAAATTTAAAAATATTCCATCCAAATACAATTTTATAAATTATACACAATAATTAATAATTAAAAAACAAAATTATACACACGAGTTTTCTTATGTGGGATATGGAAAAAAATATTTGAATCTTTTAAATTTTAAAATTTTTTTAAGGGTAAAATATAATTTATTATTATTTATTTTATAAATAAAAAATATAAAAAATATTTAAAAATAAAAAATTATATTTTATCATCTCAAATAAAAATTTAAAATTTAAAAAATTTAAATTTTTAAAAGAAATGTGAAGCTTAATGCCTAAGCTTCTGTTGCTTGGCTTCTATGACAAGCTCACAAAGACGTGTCGGCTGCCGCATACAAAAGTCACTATTTCTTAATTAATTCAAGCCTTATAATCTCCTGTGCTCCTGTCTTTGCTATGTACCGTACCTCCAAAATCCAACCAATAGGATCCCTCCACGTCATTATCCCTTTCATGCAAATTTACCAGAACGTCACTCTATCACAAACCCTTTAATAATTATATCATAATAAGACGCTGATTGTTAACATTCGTTTTCCCCAAGCTATATGTGATTTACAAAACCACGTTGTAATCCAGCATTCTTCATCATTCTTGATATTGTTAATAATCAAGGTTTGTAAGTCCATCCTCTATACAAATGCCAATAATTAAGCACATATAGTTAATTAAATTAAATTACTGTAAGTTTGTGCCAACAAGCTAATATGCTCTCTCATTAGCTTCCAGCACAACTTTGTTGAATGAGAATAAAGATATTCAGTTCCCAGTGTGAAGGGAACAGAGATGAATGGAGGGGGGAAAATGAGAGAGATTCGTGAAGAATTGAATATTAACGAGAATATGCAGTACTAGTGTGTATTTTTTTTTTGTTGGTTGGTGCAAAAAAATATATTATATTTTTAACTAAATTTCATTCTTTGTATATGTTTTATTCATTCATTTCTTATGATATGCACTATTTTTTTATATTTTTATTTTAGTACTAAAAATAATTTATATAAACAGTGAAAATATCATTTTGTTGTATATGTAGATATGTGTACCAAATTTTTAGGGTGAAGTATAATTTTGGTTTTTATAGGTCGAAATTTTTTTTTATTCCAAACATTTTTTTGCATATAAAATTATTTTAAGATTTAACTTAATTTTAAAATCGTTCTTATTTTAGGGACCAAAATTGTATAGAAGTGACTGCAGAAGCAAAGGCACATATGGATTGTAAATAATTTTTTCTTTATTTCTCTCCTCTTCTTCTTTATTTTTTTTTTATAGAAATAAAAACACTCTCTTTCTTTTTTTCTTTTTTTATTTTATAATTTTTTTGTTATCAATAATTTAATTTAAAATTTTAATATTTAAAAANNNNNNNNNNNNNNNNNNNNNNNNNNNNNNNNNNNNNNNNNNNNNNNNNNNNNNNNNNNNNNNNNNNNNNNNNNNNNNNNNNNNNNNNNNNNNNNNNNNNNNNNNNNNNNNNNNNNACCAAAATTATACTTAACCTAAATTTTTACACAATTTTGAGGGTACTCCAATGCAATTTGACACTAGCTACTAGCAGCTCATCACTATTCAGCTTTGGACATACACAAAACCTTAATTATGTGGATCTCTTTCTCAAAAGAATCAGGTTTGCTCCATCTCCACTATTACCTTAGATCACCTTTTTAAAGTCGTATATTTAATACTTATTTGGATATCATTATTTTGATAAAAAAAAATTTAAATAAAAAAATCTATTTTTTATTTTTAGTAGTAAAAAATAAAAGTACTAAAAAATAAAACCCAAATAACCTCATAAAACTTTTATGTATAATAATAAATTAATAATAATCATAATGAAACCTCTTGTTTGGCCTTAATATATAATAAGTGAAATAATTTAATTTTTTAGAACATCATAGATATAGATGTAGGAATGCAAGAAAAAAAAAAAAAGAAAAGTCTAATAGTCTAGGACGAATTTATTCTATTTTATAAAAAGCGTATATACCATAATATTAACTACTACCAAACTTGACGTCCAAGCAACCTTTCCTTATATCTAGAACCGCTTCCTTTGGTTCCCACCTTTTCTTTTTATTTCATCTATCGCTTATACCTTTTGTCCTCTTCTTCATCTGATCTTTCCTCTTTCTCTCTC

## >AdNAC9

TTACTTTATATTATCTCATATGAATTATTTTAACAAAGTTACTATTAGTAGTAAAGTAATAAACATGTTGAATAAAATAAAGAATGCTTCAGCGGAATCTGTTCCGTATCATTAGATATGATTATTGTCTCACAAGTTAATAATTAATGTTAATATCATCCACCCATTAGAGACCGCGTAGCTAATTTAAATCTTAGGGGTTTGGATTTCATTTGAGGATGGTGTATGTCATGAAAATACAATTATTATAATATATGAGAATATAATATTAGAATTGAAGAAGAATAAAAACGAAGTTATGCGTATCTAATTTATGACTTATGAGCTTTGGACATTTTATGATTTTGATTTGTGAGATTATGTGATTTGCATGGTTGAGGGAGTGAAAAATTAAATATTTTGGTTGAAATATAGAGAAATAGTGTGAGTGAATTTTTTTTAATAAAAATGATCAAATTTATTACAGTAGAATAATCAAATATCTTGTATAGAATAATAAAATAAAAAAGTATTATAATTTCGGATTCTTTTCAAAAGGAGGCAATAATAATGCTTTTTAATCAGCTGTATTATTGTTTGGAGAATCCTATTCCAAAATTTGCACTATAAAAAAAGGTTTTAGTAGTATATTTAGAAGAAAATTACTCTACTTAATAATAATTACGTACATATACCTCCCTAATAATTTGGAAGCAGCAACTAACATTTCATATTAAATATTTTAACTATTGTTATATAAACCTAAGTATGTTTTAAATTTGTTTATACACTAAATATTTTATTTTCTTATTAGTCTTTTATATCTTTGTTTTTTTTTCAAACGTCAATTATGTGTATATACATACTCAGAATATTTTCAACAAATAGCCGAACCATATTAGAAATTGTAGTTACCTAGCTAAAAAAAACAACCACCATCACCCACCTAAAGCTGCGCAAGAATATAGACAAATCAAATATATATATATATTCAACTAAAGCAGCAGCTACTACTAAAATTACTACTATATTTTTAAAAGAGCAAATAATATAGAATTAATTAATTAGGACAAGAATTACACTTTTTCACAACTTTAGCTAATACGCTTTTTAGATAATATATAGTTATTTATTTATATATTTTTACTTAATAGTTTAAACTTGTTAAAAAGGCAACAGAAGTTTTACATAAAAAAACGTATATTAAATAATATAATTTATTTAGAGTTTGTTTGAATGTGATTTAAAAAAAAAAATCTTTTTTTAATAATTTTTTAAAAAGATTTTTTATGAAAATAAAAATAGTTTTATATTTAATTTAAATATTTTATGTAAATTTTTTTATTTATCAATTATATTTAAATAATCAAAAAATATATTTTTTATTTTTTTAATATTGTTTTTTATTATTAAAAATTTACTAAATACATTAAAAAATTAAAAAAATTATTAAAAAATATTTTTTTATCGATTTAACAACGTTAAAACAAACACTTAGATGCTTTCACTCAAATTATTTGTCCTCATGTATATGTTAGTGTTTTCTTCTTCCGAATTAACCCTCGAGCAGTAAAACTTCTTGAGAGGTGTTGGAGTACGATGGAGAATCGGCAATGGAAACAAGGCTATGAAAAAAAAATATTTAATTCAACTGAATCCAAGAAAAAAGTGGTGGGGACTTGGAAGCAAGGGTCCACACTCCACAAGGATACACCACCGTTTCATTATCGCCATTTTCTTTGATCACATTCGTTTTCAAACGGGTAGAGAATTAGATATATAGTTATAGCCAACAAACAACATAGGTGGAACCGTGGAACATATACCAATACGAATCACGAGCTTTTTTTAAAGAGAAATATGGTCCAGACTTCTATGATTCTATCCTAACACTTTTAGTATACTAATAATTAAGAAAAATAATGTTGCATGCCCTATTTTATGTATATTTTTTAAACAACAATACTATATAGTCATTAAATATTATTATTTTTAACCAGCACTTAACTAAAAATAATTTACAAAAATATTTACAAAAATTTTGTACATATAAATTGTAATTATCTTTACTAAAATTTTACACACATAAATTAATAACACATAAATTAATACGGTAGAAATTCAGGTGCAGTCAATTTTACGTGAAGTTGATAATTGAGAATCGTTAAATGATTTAACAGATTTGACTAAATTATCATCTAACGGCTCTTAGCTATCAATTTTCTCGTAATTAATAGAATAATATAGAAATAAAAAAAGATGAAAACTCAGATGCAGTCGACTTTACGTAAAGTTGATAGGTGAGAACCGTTAGATAGTTTGAATGATTTGACTAAATTTTCATCTTACAGCTTTAAATTATCAACTTCACGTAAAGTTAACTGCACATGAGTTTCCACCACTAAAAAAATGCATTATGTTTGTAAGCTTGCATAGGAAGTGGCTTGCAAAGCACGGATCCGTGAAGCGTAACGTGTGAGAGGTGCGTGTAGTGTATCTCTTCTCTTCTCTATCTTGTCTT

## >AdNAC10

TGGTATGTTGTATATTTTGATTGTCAAATTTGTAATTAATCATTCATAATGATATATAAGAGAAATAGAGTGAGTGAAGGAATGAGAGTAGAGATAGAGAAAAAGAGAGGGAGGAAGAGAGAATTCTTTAATTTTAGAGAAAAAGATTTGATTTTAATTACAATAAGAGAGTGACAAGTGACATATTTTGGTTGTAAAATTAGTAATATTTAGTAGATATATAATATATAATATAACTATATTTTAATTTTAATTTAATTTGAATGTAATTAGAGAATGTTATGTTGTATATTTAATTGTCAAATTTGTAATTAGTTATTGATAGTAATATATAGAAGAAATAAAATAGTAAAGGAATGAAAAGAGATAGAAAAAAAAAGAAAAAGAAAATTTTTTTTAATTTTACAAAAAAATATTTAATTTTAATTATAATGAAAAAGTGACATGTGATATATGTGACATATTTTAATTTATTTATAAAATTGAAAATATATAATAAATTTTTTATTTTTTTTTAACCTTTACTCATTCAATATTTTATTAACTAATTATCCATAATAAAAAGTTAATTCTTTGTCAAAACCATTATTCATAGTATACTCTTAAATGAAAGTGTTAACCACAATATATCCTTTTATATACAATGATATATTATATAAATGTGATTATTATCTTCATTCATTGTACATACTAGAAAGCTTTTAAACCAGATGATGTGCACCACGCGGGTTGGATCAACGGGGCACACGCATGAAACCTGCTCAATAATAAAGGTTAAAAGAATAGGACGCGATGTGATGAGTACAATATAATTAAAAAATATATATAGTATCGTTTTTGTCCTTAGCTTAGGGTAAGTCTTAAAGTTGTTCATAACATTTCAATCGTCTTATTTAAGTTTTTAACGTTTCAAAATTGACTCAATGTTATCTTACCGTTAAGAATCCATTAACAGAATTGACGGCGAGACAAAATTGAGATAATTTTAAAACATTAAGGACTTAAATAGGATAAAAACATTGGGGACAAAAACGATACAATACAAATAAATTTTAATTTTATCCTTTAATATATAATATTAATTTTATCTTCTAAGTAAATTACATTTAATCATATTACTTTCATTCTAAATAAATTAATTTTTATAATTTTATTTTTAAAAATTTTTAGTTATCATAAAATATTTGTAGAATAACTACTATATAAACTTACAGAAAAAAAAAATAATATATATACAATAAAATATAAATTATACTTTTTGTTTCTAATGTATCAAAATTCTTAAAAATTATAAAAAAATAAATTTATTTAAATTGAAAGTAATGTGATTATTAAGTGTAATTTACTTAGATGTAATTAAAAATAATTAAATACTATGTACAGTAAAAAATTGATATTATTAAAAGATAAAATAAAATTAAAATTTATTTTTATGTATCGTTTTTTGTCTCCAATATTTTCGTCCTATTTAAGTCTTTAACGTTTTAAAATCGTCTCAATTTTGTCCTACCGTCATGTCTGTTAACGGATCCCTAACGACAGGACAACATTGAGTCAATTTTAAAATATTAAAGACTTAAATAGAACGATTGAAATGTTAGAGACAACTTTAAAATTTATCCCAAACATTGAGGACAAAAACGATACTCTACTCTTAAAAAAAATTAAGATTTTCTTAAAAGAAAATTACGAGAGTTTAAATGAACACATTTGTTTATTGAAAATGCTATATGTATATAAAAATAAGAATATTAAAAGATTATTAGAATTTATTATTTTTGTCTATTAATTAACTATTAATATTTAAAAATATAAAATTAAAATATATTGTTAATTTACTAAACTAAAAAAATTAAGTTATAATTAAAATTAATAATAAAAAATAATAAATTTTAACAATCTTCTAGTATTTTTCTGTGTATATCAAAAAGAGCTTTAATTGGTGGTAATTAAGGGTTTGATGTTTAATTTTGAATCAATTTCTGGGGTCCCTTCACAGGCAGCTGAAATAAACGTTAACATCTACATATAATTATTACTTATATATATTCTGTATGAGTTGGTGGTAGATAATGGAGTTAGATGGTGACACCATCACCTCTATGAAGATTGATGGCGTTTAAGCATAGCGCCACCCTTATTCACACTCTCACTCAGCTTTTCCTACAATCTAGCAAGCCAACTTTAGGGGAGCGACACAACACACACATATAATATATATTCAAAAATATTATTTGTACACTAAAATTAGCTATTAAAATTAGTCACTAATATATTTGTGTATAAATATATATGTGGTTTAATTTATTTTTAATGTGTATTTATATTTTAACATATATTTTATTCTGATAGTTGATTTTAGTGGCTGATTTTAGTGTTCACATAACATAACTCATATATATTTTATTAAATTTTATTTTATTAGGGTTACCTTTCATCTTATCATAATACCTTGCTATATATAGTTTTCATCTCCTTATTGTTCCTTTTGCTTCCTATT

## >AdNAC11

TATTATCATCTATCCTCTTTTTATAGGTTAGTTAATTTTTGGCCATTTTTAGCTACCATAAAACTTTTTAATAACAATATATATAAAAGATAACTAAGGTAATGCTCAATTTTCCAACACTATAAAAAAGTCTTTAATTTTTTTAAATAATAAAATTAGTTTCTGAATTTATAAATTGTTTCGACTCTAATCAATTGTTGCTAATATAATATTGACATAGAGTGTTATTACAAACTCGAATTCCACAGCCTTAAATGCTTAATAATGACTCGATAAAGTGTTCATAATAACTCCACGAAGGCCCAATGCCTATAGCCTCTTACTATCACTCAATTCAGCGCATCTCTAAAGTTTTTTTGGTTGGAAAGAAAAAAATAAAAGATAAAAAATAAAAAAAAAACAAAAGAAAAAAAAATAAATTGAGTGAAATTTTATTTTTTTAAATATCTTTAGATGAAAGAAAAATAAAGAAAAAAATATATAATATTATAAAAAAATAATTTTATCTTTAAATATTTTAAATTATATTAAAAAATAGAAGATAATATTAGAATATTAATATAAATTTTCAATAAATTCTACTTATTTTTTTACATTATTTATTATTTTTTTAATTTTTTCGTTCAACCAAACAAAAATAATAACTATTTTTTTTTAATTTTTTTCTTTCATTTTTTCTTTTCTATTTTCACCTCAAACAAACACACCTAATAGATTTTTGAATTTGAAAGCCTAGAGATGTGCTAATAATCACTTAGGAGATATGATAAATCACGAACTTATAAAATCGGTTTCTCCAAGTATGATCTATCTGAACCTTTACAAACCCTATCTCTTACGTATCCAATGACTTGGGTTTTATTTGCACCTCATTTNNNNNNNNNNNNNNNNNNNNNNNNNNNNNNNNNNNNNNNNNNNNNNNNNNNNNNNNNNNNNNNNNNNNNNNNNNNNNNNNNNNNNNNNNNNNNNNNNACGTAAAAAATATATTCTTTTAAAAAAATTTAAAAAATATAAATTATAGCTTTTAAAAAATTTTTTTCTTTTAATTTTTATAATGTTTTTAGTTTTATTATTAAAAATTTACTAAAATATAAAAAACATCTTTTTTATTAATTTTTTTATCAATTTAATAACGTTCAAACAAACATTTAATCGTTAAAATATCTTTGTAAATCACCTCTACCATTCACACATCAAGAGTCCGAAGGCCGGAAGAAATCTTGCATCCATAATTGACGATCCCAAATATCTGAGGTAACTCACAAACAAGTGTTAAAATATTATCGTAGCATCTTATAATAATAAGGTGACATATTTAGAACTTTAGTATCATTAAACTAGTGTTTGTGATGAAGATGTCTCTAAAATTTTATAAAAAAAAGCAAAAATAAAAAAAATAAATAAATAAATTAGTTAATTAACAAACTAATTAAAAGCGTAAATTTTATAATTAACACTAAATTATCTGTTAATAAATTGGATTTTTTATTTAAATTATTTAAATATAATATTTATAAAAATATGTAATCTGAAACATATTTATCTATATACATAATATTTCATATTTCGAATACAATATCTAAAAATCATGTGTTTAGACATATTGATCTCTAAGATAAAGAATATTATTTTATCAATCTTTTACTCTAAAAGAACTTAATCCTTATTTTGTAATGCAGCTTAAAAGCTTTTATTTAATAAATAATAATATATATTATTTTTAAATTTATTACAACAATATACATGTTAAGAATAAAGTTGGATACACCGACATATGATGGTAATTAAATGTATCTAAATATGTTTAAGAAAAATTTTTTATTTTTTATTAAGATACTATTGGATACGGCAGATATGTGTGTCAGATAAGTGTCTTGTTCAAAATATATCCGACACCCAGATACGACAAATTTAGCGACATATCCGTATTTCATAGAATAGGGTTGTTAAACGGATTAGTCTAGCCATTTTAAGTTTAGTCGCGAAATCTATTTATTTACGAGTCATAATTTTTGGAGTATTTATAATCAATTCAATAGTCCGTTTAATCTTTTAATTTATTTTTTAAAAAATAATTTTTTTAAAAATTTAATTTTTAAGTGAAAAATTTTAAATAAATAATGTTTTGTTAGTTGATTGAATAAAAAAATATTGGCTAAAAATATTTTTTTAAATACAAATAAATTAAATAAATCGCGTGTTTAATCTACAGACTAACCTATTATTTTTTTTTGAATTAACTAAACTCAATTCATTTAACCCAAAATCCTTTTAACCCAAAATTTAGATGAAGTTAGTTTTAGAAGTAAGTTTGCCTTTTTTTTTTAATTTTTTTTCCTTAAATTTGTCGCGTAAATAAATAAACTATAGTATTGAGTTATAACATCAATTTTAACAGTCCTACCCCTGCCATTAAGTACTCCGTATGAAAAGTGATTGGGGTAAACAATGCAAGAATCTATAACGTTACTTTACATTTACTGATTTGCCCTTTTTTAGC

## >AdNAC12

GTTATTGTATGTTCTCTTTATTACTAAATTATATTATTTTTTATTATTTTATATACTAATTATTAATTATATAGTAAAAATATACAATAATTTTATTAATATTATTTTAATTTTATATTCACTTATTTTTAAATTAATTATATTTTTTATTATTCATAATTATTAATATAAATATTAGAGGTAAATACAAAATCGGTATTCAAAAGATTCTGGTGCTGACAAAATAGTACTTAACTTTTGTTATTGATAAAATAGTCCCTAAAAGATTTAAAATTTGACAAGCATGTTCCTGAACTCGCCCAACTAATAACTTATCAATTGGATCAATAAATTAATAAATTAATAGTCTAATCAGTTCGATTACTAATTCGATTCTTACAACTACGATTATAAATAAATTTGATTATTCGTCTATTATAAATTTAATTTTTTATTATAACAAATTTAATTATTCTAGTGATCAATTATTAATAAAAAATATGTGTAAATTAATATGAAAAAATATAAAAATATATAATGATTGATTTTTTTTGTGACTGAAATAAATTAAACAAAGAAAAACAAAACAAATAAAATAAGGAACTGCTTAAATAGAGGGGCGGTCCTATTTAAGACTACTCTTAAACTTCTTCTAAGGTGAAAGAAGCTCCACATGCGAAAGTTGTAACTTCATTGTCATCTTTGCCATAGTATCTGTCACCGTGTTTGCATCTCTCATAATCAAACGAAAGTCAACACGCCAATTCCAATGCATGATATCTCTTATTTTGAGCACCAATGGATCAATAAACCCAAAACCATCTTGAGTAACAAGATTAAATGTTTCCATACAATCCGTCTCACAGATAACATCTCGTTGACCCACATCTCAAGCTAAGAGATATTCTCTCCAAATAGCAAACAATTCTCCTTGAAGAATACTATTACTCTCAATCATTCCCAAACACCCCATTTGCCAGCTCCCATTACAATCTCTAATAATACAAGCAAAACTAACACTATTATCCAAACCAAAATAATTAGCATCACAATTAATCTTAAAAGTACCAATAGATGGAAGATTCCAAAACCCATTTAAAATATAATGATTGATTTTGAGTGTTTATAATTTTTTTTCTGTAACTGTACACATCTTTTAATGAATGACAAAATTATAGTTTTGCCCTGGTGTCTATATAAGGCTATAAGCACAACTCTCCCTCCCTTTCTTCTTCATTTTCACTTCGTCTTCTCTCTGATTTTCCTGAAATCTCTTCCAAAACCATTTCTTCCTCTTCTCTGTGACACCGTTATTCCATTTCTTCAAGTTCTTCTTCAACATTCGCCTTTCATTTCATATTACTGCTTGTATTCGTTGAAGTTGCTACTGTCAGAATCATCTTCACACGTAACTTCCATATTCCCTCTACACCAAAGGTTAATACTTTTTTTTATAAGAAGGCTCGAACTCATGACTACTTAATATTACTGTCTTATTTATTAATTTTTATTAACTAAAATGGATTTTATTTAATTTCTAGCTAAATCCATAATTAGATGGCACTTTAACTATAATATTTAACATTGCTTCAATATAATTTAAATAGTATAATACTACAATTTTTTTAGCTAAAATGAGTAGAGCTGCACATGGATCGGATAATATCCGTATATCTGCGGTAATTATCCGCATCCGATCTGAATTTTGCGGATATTATCCGATCCGCAAAGCCATCGGATCGGATTGCACTATGGTAAGATCGGATTGCGGATTCGGCAGTGATATCTGCGGATTCGATCCGCAAATCCGCATATCCGCACATCACATATAAATAGCATAGTTTAAGAAAGTAAACCTTAATCTGATATGAATTTTAGTGTGTTATTTTATGAATTTTATGATATTTTATTTTTAATTTCTTATGTTGTGCTCCAACTTAAAATAATTAAACTTAAATCTTGTGTTATTATTTTGTTTTTGTTATTCAAGAGAATTATTATTGATAATATTTTAGTAGTAAATAGGCTTAAACAGATAAAAAATAAATTTTTTGAATATTTTTTTGTAAAAACAACCAAACAGAATCTTAAAATAATTTTTTTTAATTACGCGGATATACCTGATATCCGATCTGATCCGATCCTCGACCTAAAAAAATACAGATATCGTATCCGATCCTATGAGTCCAGTGCAGATCAGATAAAATTTTAGACTATATCAAATTCGATCCAATGCGTGTCATGTGCAGCCCTAAAAATGAGCAAAACTAAGAATTACTTATCCGATCCAAAACACTTCTTTACTTATAATAGGTTTAAGTTATCATCCTTAATTAAGTAGTTACATTATTTTATTTATGATTATTTCATTTCTCTTTATTTATTTATTTAATCATTGTAGTTAGTTTTTTTTAGAAAATCCAAAATTTAAAAATGAAATCAAAGAGATCAAAATCAAGTAGTTTGAACTGATGTGATTTTTCCTTGAAAAACAAACCAAACCCTAAATCATAGTTTAATA

## >AdNAC13

AGTTACATTGGATGGTCTTCATAAATTTATGGTATGTAATTAGTTCGTTAGCTTTTGATTAAAGAAAAAAAATACAAAGGTCTTAGATCTGATTATGAGAGCTGTTAATAGTTAATAATTAGTGTATCTATCAAATTATTTCTATATATGTAGTATAGGAATGGCTTCAACAAGCTAAGGTCTTAGATCTTGATTTTGGTTATGATATTGAGATTTGGTTTAATAAAGTTTCTTTGGGGAAGTGCATGCACTTTATTTTATATTTAGTAAATCATCATGTACATATATTTGTAATAGAAAAATAAGTGTTTTTAAAAATATTTTAAAAAAAATTTTAAAATTAATTTATATTTATTAAAATTTAAAAATATAATATAATATCGTATATTAACAAATATTTAAATTTACTCTTGAATGTCAAGATTTTATGAAAGTCAATTAAACCTTTCTTTTAGCTTTGAAGCCATTTAATGACATAAAACTTCCAAACATAGCATTACTATATTAATGCATATTCAAAATTTCAAATGTTAGTATGTGAATTGTTTTGGCTATTCTTAATTAAGTATTAATCTACAAATTAGTGCGTCTCTGTGTGTGTGTGTGTATATATATATATATATTGATATTAATTATTGATTATTGATTTAAATAACGAGTTGGACATGAGAGTAAGCAAACTACACGTACAACATGATAATAATGTACCAACCTGGTCAAACACTAAAAAAAAAAAGAGAGAGAGAAAGAAAAAAAACACCACCAATCACTGCTTTTCTTGTCTATTTTGCTATTCATTTATTATTATATATACACTTCTTCTTTTTTTTAATTTACCAAAAGTTTTTCCTTTAAACAAATATCAAGGTTATACTTATACATCACAAATTCAACTTGACATAAGGGGAGAAATTTTTCCTTGCAACCAGCAGCCAAGTAATATAATTACTTTAATTCATTTTCAGTATTTTCAACTTTAATATTCCCCAAATATCCTTGCCATGTCTGTTTCGGAAACTTTTATAAATAAAAAGGCCAGTTGTTTTCTCTCTCTCTCGTCGTTTTGTTCCCAATAAACCTTGAAGGCCATATTGGTAATTTTGATAGGCTCACGAATAATGCTCTTTTATCTTCTCGTAATGGAAAAAATAATGAGAAACAAGGGGAAAGATTCTTTAATTTTGGTGTTTATCTATTTTAACAAAAATGGATATTTAAGTTATTAACTACACCACCTGACCCTATTTTATTTTCATGTAATGACTGAGAAAAGAATTCCATGATGAGTTGATCAGAGGCACCAATCAACATCGTTGTCTCGCTTGATTATTATTTATTAATTTGTGTCACAATAATAATATGAAAGAATCAATATCCCAAACAAAAGAATAAGAGACTCTGTAATATATTGTGTACGAAATTATTAATGTTTTTTCATTATTAAATAGTGACTTTACAAAGTAATCCACATTGGATTATCATAATCATATGCATGATATGGTGGCGACTGGTGTGTTGAGCCATAACATCTTGTAAGGGTAAAATTGAATAACAATGACATATAGAAACAGTCACAAAATTAAAGAAATTAAAAGATATATTTGTATCTCATTTTGTAATGGATTAACTATTTACACATTATTAAGGTTATTAAAATTAAATGTTATTTCTCATTAAATTAAGAAAAGAACGATAAAATAAAAACAATATTACATGACCAGTAAATATTATTATTTTTAGTCAGTATTTTGTTAGTAATAATTTATACTAATATTTAAAAAGGTTTAATACACAAAAAATATATAATTTATACTTATATTTATCAAAATTTATACACATGAATCAGTACAATTTAATTATTGTACTAGTCAAATAATAGCTAAAAATAGCTGACCCCAAAAATTTCTCATAAAATAATTCGTTTTTATATATATACTTCACATTTAGAACTTATTATGATAAAATAAAATGAATATCAAAGTTTACCCTACAATAATTACTAAAAAAATGGTTTTATTAAGTAGAGAATAATTTTTCTAAACAAAGTGAATAATAGGTTCTAAAATTAACCCAATAAAGTTAAAAAACACTCCAACTCAAATTACCTCCTAAATCTTACCATTAGAATAACTATCCGCACACCTAATGAATTGAACATCCAATTATTGTTAACTGTGCAGGAGTAAATCGAATAAAAAAAAATAACCATTCAATTAAGAATAATTAACATAATCATCTGCATACCTATTGAAATGAACATCCAACATATCCATTGTTTACATTGTTTAGTATTCTCATTATTTTCTTATACGTTTTCTTTAAAAAAATAAAAAAGGCCTAAAAACGTTGTGTGTAAAATTATGAAAAGATAATAGATATAAAGTTGAAGAAAGTGGGCAATAAACGACCAACTGGGTTGCCTTACATTCCACGTTTCTTTCTTTCTTTGAAATACAAACACAAACACAAACACACACACTGGTATTTAGGGGGATTCTTT

## >AdNAC14

CAAATGCCACATGCACAATAATCCAAGGTCTCACCTCAATCCACGACGATGACAACGGCCACTACCACATTAATATACATTTTCTGTTTTAGAATTGAATGAGATACTACTGTTTCTCTTTTCCCACTAAAATAATAATAATTCATTAATTTTCCTAGGACTGGAGCTGGACCAACACCAACCACCAGAGGTAATTACAAGATAAGATTCATTTTCGGGGAAGACGATTCTCTTAATTTTTTTTTAATTAATATTGTTAAAGCTGGTTTGTTATATAATATTTTCTCATTTTACTTAAAGAATATATAATAAAAGATTATACCTAACAATACCAATTGAGAAAAGAATATTGAAAAGAACCATTTCCTTCAGTCCGGTACTTGAATCCTCCAATAATTAATATTTAAACATATTTGGTTCGAATTATGTTAGGTATACATTAAAATTAGTTATTAAAATCAGTCATTAATATAAAATTTATATTGAAGTATAAATATATATTAAAAATAAATTAAATTATATATATATTTATACATAAATACATTAGTGACTGATTTTAATAATTAATTTTAGTATATAAATAGTATTTTGATTTGGTTCGTTGTTAAGTTTTATAATTATGGATCAAGTTAATTTTTTGTCATAATTTAATTAATAAAATACTTATATATATATGTACAATTGCCTAGTGACTAGTTAGCATTATACTATAAGTGCATGACTTAATTCAATCCTCAATATAATGAGATTAAATTGAGGGATCAAATTAACTCTCTTAAGAAGCTAAATAGAGTTCAACACTTTTAGTGTTATATGAATTAACTATTAAACTGATCGGTAATATTAAGAAAATAAAAAATAATCAAAATTTATCTTATTTAATATTTATTAATTATTTAATAATTAATAAATATTAAATAAAATAAGTTTTAGTTTGATTTTGGTTAATTTTTTTCCTTCTGATTGATAATGGATAGCTAACTATTCACATAAGTGTTCTGATTTTAAAAAATTTGGTATAGAAGACTAGATGATCTTCACTGTATCATATTGTGATTTAATAGGGAGATCGATAAATATATTATAAAAATTATTAACAATATCATTTTTCTTAAATTGTCATTAATGTCATTTCATCTTATAAAATATAAATATAAGTAAATGTTAATTTGATATTTAGTTTTTCACTATTGTATAAAGTGAAATATTCTTTGTGAACTATCTAATAAATAAAAGATAAACTTTGTTAGGTAGATAATAGTTTTTATAAATAATATAAAAATAATGAGTTTTAAAATTGACTCAATAAAATAAAAATACATTACATTTTTAAATTATCTACTTAAATCTTAATATTATAATAACCATTCACACATTTAGTAAATTAAATATCTAATATATTCATTATTTATATTATTTAATATTTTTATTATCTATTTATACTTTTTCATAAAAGATATAAATAAATCAATTTTCTTACTATAAACTCCTAAATTTAACACTTTATCTTTAGATATAAAATTTAATTTTGATATATTATTAACACAAAAATCAATACAGACATATAATAATATGTTATTATAACAATAAAATTAATAATTTTTGAAACTAAGAAATATTATAAATATTTGAATTTTATTTCAAAATTATGTAAAATTTTTACGTTATGAATATATTCAAATTAAAATTATTTTGGTACGTACTTTGAACGGAAGAGATTTATGTTAAGCTAGTCTACATGAATATTTCTAGTTTTCTACTATTCAGAAAGGTGTTGCTGAAATTCCCACTTTATCCTGAATGATGCCCTTAATGATTCTACTTTATGGGGCCTTGAATGACACTTGTGACCATCTTATCTCAACAACGATGATGTCAGTCACCAGCCGCCGCCATTACACAACATTAAGTAGTATAGCACACCATGCCAAATCCCTTTCTTTCTATAATTAATATTAATCGCATATATATTTGATATTTTTATGTCAAGGAATAAAAAATAGAATATAAAATTAATATGATATCGTTCAATTATTTTTTTGATCAATTTATATCATTCAATTATTATTTCTTATCTTTTTATTGATATTATGACTCTTAAAAAAAATTAAGAATTTATTTTTAAAAATATCTATAATAAACAAATATATTATATTTTTGAAATATTAAAAATAATTCTCAATTATATGTAGCATTAAAAGATAATTATTTTATAATACTTGAATATAATATTTTTTATATAATATATTAAAAATATAATTTCTTAAAATATACTTCTTAAAATACATGGTGACTAAAAATAGTTATTTTTATTAATATATCATGCACTACATATATATTGTTTTTACTTTAACAATATTTTAAAATTAAATTTAAAAAAAAAAGTTGAGTATTATCAACACCAAACACAAAAATTGATGTGTATATAGTTGTTATTTTGTTATTCATTCTCTCTTTTATACAAATGGAGCCCACTTCATGGAAAATTTTGAAGTTGATC

## >AdNAC15

ACCAATCTTATTATTATTATTATTATTATTATTATTATTATTATTATTATTATTATTATTATTATTATTATTATTATTATTTAGGTANNNNNNNNNNNNNNNNNNNNNNNNNNNNNNNNNNNNNNNNNNNNNNNNNNNNNNNNNNNNNNNNNNNNNNNNNNNNNNNNNNNNNNNNNNNNNNNNNNNNNNNNNNNNNNNNNNNNNNNNNNNNNNNNNNNNNNNNNNNNNNNNNNNNNNNNNNNNNNNNNNNNNNNNNNNNNNNNNNNNNNNNNNNNNNNNNNNNNNNNNNNNNNNNNNNNNNNNNNNNNNNNNNNNNNNNNNNNNNNNNNNNNNNNNNNNNNNNNNNNNNNNNNNNNNNNNNNNNNNNNNNNNNNNNNNNNNNNNNNNNNNNNCACGTGTCAATTTTAATTTTCAAAGTTCAAATAAATAAAACTAGCTATGTGTATATAATTATATATTGACATACAGGGCAATGCTAATGGGTGATAATTCAACCCTTTTTTTTTTTTACCTGAAGTTTGTTATTTTCTTCAAATATATTATCGTCACATATCTAGCTCCATACTTTATTTATATCATCCCAATTTAGAAGTACCGACATAAAATGCTTGGAAGAACTAAAAACAAGGTTTAAGTACATGTCTAGCTCCTAATTAATATTAAACATTAAATGCAAATAAGGTTTTATAAACAATATATTATCCTTTTTTTATAATTATTTTTTCGATAATATTAGAAAGACAAAAAAAATAATAAAATTTATCTTATAAATAAGATAAATTCTGACTATTTTTTATTAATTATTTTTTGTTATCAAATATTTTTATTATCTTTTACTTTGTCATTACATATCCCAAAAATTTAAACTAAAATAAAGACATGTACACAAACAATTATAGAACAAAATCTCTTCTTGAGTTTGAAGCATAAATTTACATAGATATATTTTTATAGTTACATATCTAATAGACTTTTTCTATATATATGAAATAATACAAAGATGTTTTAAATTTTATATAATAAAATAGCATTTTATTTCAACCCAGTCCTCAACTAAAAGTTTATTTTTCTATATTTTTCTTTTTTATTTTTCCTTCTCTCCCTATTATGCATTTTTTTCCGCCTTCTCCTTCATGTTATGTGTTATTTGTACAGAATCTATATATTTGTTGTTCTGTTATAAATTTTATGTTCTTTAATTTTGCAAAAATTTATGTTTTGGTTTTAAAAAATTTCTATATTTTTCCATGAATTCCTTTGTTATTTTAAATAAATTTTTGTGTTGGTTTCAATATTTAAAAAAAAAGTTGGAAAAAATTTAACTTTTTGAAAATTTAGTTAGTTGGACGGTATATTTTGGAGACTTATTATAAAAATACTTATTCATCTAACATTATTTATGTTCGTTAATTTGATGGTTTATTTGTAGTATATAATTTATTACCTTTTAGATATTTATATAGATTTTATAAATAATTAAAATAAAAAAATTGTTTCTTATTAAATAACTTTTTTTTAAACTTTTTCAGTTTTAGAAAAATAAAGGCTGTCAAATCACTTTTTTTTTTCTTTTAATATAAAATCAAAAAACAAATCCACCATTATTCTATGTTTTACGTCAAACTATACATGCTAAGCATGACACCAGCAAATAGAGGATTGACTTATTATTATCAAAGAAATTAAAAAGAAACATAAGAGATATAATTTCACATAGGAGATTCCAAAAACCTATCCAACAAACCCTAAGTTTGATTAAATCATTAAACCAAATCCAACAACTCCAGTTGAAAATAGTCAATCACCACCGCCAATCCAATATTAGATGAACATATATATAGAAAATTTTAAATTTCAAAATTTTAATTAGGTCTTCATTAATTCTTTTGCTAATTAATTATCTCTCAATACTTGTTAATACTTAATTGGTAGAACCATGGAATCTGAAATCATATTTCCATGACTCTAAGAAAAGCATGCAAAATATTTAATTTTATGACCAATCACTTTATATTTTATGATACAAAAGGTGGTGGTCAGGCATAGCAAAAAAGGCAAAGAAAAAGATCTTTTTAAATTTGGTATATAAAATATAAATTTAATTTTGATGTAATGTTAGTGTAAAATAAATTACAATTACGCAAAGTCATATCATCAACAAAAATAACTACTTTTTACATTGATTGCGTGAATAGTCATCCAAAAGAATAGATGTAATTATACGACTGTGTAAAATGCTTTACATTGTCGGTAAATCAAAATTAAACTCAATATAATATATAACAACTTTTAATTAATTAATTAAAATTTCAAAGAACAAACTTAGTCAGTTAAGCATTGTCCCCTTTATTACATGCTTAAGTTCCCTCCCAACTAACTCCAAAAAAATTAAATAACCTAGCTATATATAATATGCTTATAAATAAGCATACACAAAGCTTCATTTGATCACCAATAAGTTGGTCCAGTTTACTAAGTTTTTATTATTTATAATTATTTT

## >AdNAC16

ACTCACTACATCAATTATGATATAAATAAACTTTGCATTAGGTTTTATCTCGGTCAAAACAAAATAAAAAAATAAACACATAATTACCTAAATATTTAACTAAATACTAATTTATATTTAACTAAACTAATAACTAACTAAAAAAATTATTATCTTAAACTTACTTAAATAAACACAATAAACAAATGCTAATTGAGTATATTTTCACATTTCACCTAACTATTACGTCCATCGATTAACATAAATTATTACAAAATTCTAATCCTTTTTATAAATATAGGCAATTATGTACCTGCACTCATATATTCCGAAAACTCCGAAACATGCATGGCTTCCAAATCCAACACTCGCATGAAAGATGGTTCCTCAAACGGCACTTCATTCGCGAGTGCATTTGCCACGTCTGTCACATTTGGAACCACGGTGTCGTCACTTTGATCTTCATCTCCGTCTGGACCAACAACTTCGTAATTGCTTTCGAACTCTTCTTCACTGTCACTATTATAATCTTCTCATACAATATTCCGGTCGGCCTCAGATTGTTCGAATTCAATGTACAACTTGATGAATGAGATTCGAGCACGACTTTCAATATACATTGAAAACATCACTTGCATACTCGCTTCGTCCATTACATATTTGGTTTCAAATTGGACGAATCCACCAAACACCGGTATAAAATATATGATATTTTTCCTGACATCAAAGAATCTATCTTCTCACAAATCACACCTTTGAGCTCTTCAAATGAGATTGTGAAGGGAATAACAACATCTAATGGATTTTCACAAATAAATTTCACTCCTTTAGATTTTTGTAACAAAATCTAACCAAAGTAATACACTTTTAAAAGAACTCTATCATTCATTTCTCTCACTCACCTAAATAAAAACAGAAATTTCACTACCAAAATTTTTTTACTTCATATCAGAGAAGAGAGAACATTGGTAGAAGAAAGAAGAAGAAGAAGCCGAGCAAGAAGAAGAAGAACACGGGATCTGAAATCTTCTTTGCGAGTTACACACTATATATATATAATTTGGTTAATACATTAATAATAATAATTCTATAATATACTATTAGTATTATGATCTAAATAATTTTCACAATAAAATAAAAGCTAATGAACACATTATTTGATATATAGTAAAAAAATTTTGAGTAATAACAAATTTAATTTTTTTTATCTCTTTATTTGATATATCATTTAGTATTAGACTATTAGTAGCCTACTCATAGTGTATTTTAATGCCAAGATATCAAATAAAATTATTAATTTAGTTTAAAGAGATAAAAAAATTATGTTGAGTAAAATATATGTAACATTTAATTAAATTTTAATATAAATCTCTGAATAAGTATTAGTAGCTTCGTCATAGTGTATTTTAGTACAAAGATATCAACTAGAATTATTAATTTAGTTTAAAGAGATAAAAAATTAAATTTGTTATTACTCAGAAAAATTTTACTATATATCAAATAATGTGTTCATTAGTTTTTATTTTATTGTAAAAATTATTTAGATTATAATACTAATAATATATCATAGGATTATTATTATTATTAATGTATTAACCGAATTTTAATATTTATTATTTATATATATTTAAAAATTATATTTGAGAATTATTCATAATAAATGATATTTTTAAATTTGAATAATTTATTAATTAGTTTGTACTTATTATACCATATATTCAATAATTTATTAAAAAAAGAATATTAATATAATGAATAATTTTTTTAAAAAAATATCATTTAAAAAATATGGACAACTAAATTCCTAACCAATTTAAATTTAAAGACAATTAAGTTTTTGTTCATTTTTGAATCTGACACGTCAGTATTTTTTGTTCAAAATTAACAAAAAAATACTCACACAAACTACGTTGGATCACAAAAATAAAAAATAAGAACCAAAATGAATTATTTTTATAGTGAGAAATCGTATTATCATTTTAAAAAATAGACAGAAATCGAATTAATAGTTCCTCAAAATATGACTAACATCAAATTAAAAAATATTGTGAAAGAGACGAGGAGCACTAAACAAAAGTGTATATCTATTTTTTTTTTCCATTTAAATTTTTGGGGCAAAACTTGCGTTTCCTTACTCACGACGTAAAATGAGGGTCCTCTCGTCCGTTTCAGGTACACGCGTCAGCTTTTCATTTGCTTGACAGCATGTCCTTTTTTCCCTCCTTCTGCAGTTTCCTTTTTGAACAACAACAACAACACTCATAAGTTTAATTCCAACTGTACCCCTCTATATTCCATCAACTCAGTCCGCGCCATATACTTCAGAAGTGGCAAAATTGTAGTTTCGGAAGCAAAAACCAAGGGTAAACAATGGTAACGCGGGATATACACGAGGAACCGTGAACGCGCTTGTCACGTAAACTATGAAAGCAGTATTAGTACTCTACAGTGTGTAGCACTGACGAAACTTTCTTTCTTCTCTCTCTTTCTTTTTCACTTTCTGTGCAACTTCTTTTACTTT

## >AdNAC17

GTTACAAATTCTTTTTGTTTCAAAGTTTCGGTTTTTTGTAAGAAATTTTTTTGTTACAAATATTGCTTTTTCTTGTAGTGGTAATAATACAAAAAAACAACATATTGTAGCGCCAACATTACCATCAGAAAAATTCGCCCTAACCAAATGATTTTTCGGTCAGGTAATCCGTCGCAAAATCCGTTAGTAATTACTATCGGATGAAAAAATCCGACCGTAACTATTTACCGGCAAGGTTTATACCGTTCGATTCCTTTAGATGGTAAATCTGAGGTAACTTAATTAACGTCGAATTTTATTCGTCTTTCCGACGATAAATTTGACGGTACTCAAGTTTTTCTTGTAGTGAAAATCAGAAGTAATAAATAGTTATATAATATAAAAAGACTAACCATATTTATACAATATGTATTTAAAAAATAATTAAAATATTATATTAATTAACTTATCATTTAATTTGTGAAATTAAGTGTCGAACTTATGCTTTGATGACATTACTTCTAACTCAAATTAAATTTTTCAGAACATAATGTCATCAAGCGTACGTTCTATTCTTATTCGTTTTAATGTAGATATTTTTTATTGACATTTATATTTTAAAATTAATAAAAATATTAATAATATGTATAAATAGTAAATCGAATTAACTCAAATCGATTTATTATGTATATGTTATATAAGTAATCAATCGAATTGGATATAATATATACATAAAATCAACTTAATTTAATTTATTATTAGTACATAATACACATAATAAATAAAATAAAGTTAATTCGATTTACATAAAAATATTTAAGATAAGACGTATAACCAATTCTACTTCAATACATTTACATAATTTTAAATTTTATTTATTTTATCAAGGTGAATTATCCGAATTTTTTTCTTGTCATTAATTAACAAAAAATTTTAATGTTATATATATTGTTACAGACCACCATCTCACATTATTTAATTACTGAAATACAAATATGTAAAATCATAATCACATAGGAATATCAAGAGAAATAAAATAAGAATGAAGGGGTGTGGATTTCTGGAAATTGGTCAGGGAAAAGTGGAGCTGTCTTCCATAAAACAATATTTTCTTTGTTTCTTTTTCTAATGATAGTTGGTGGTATCCATAAAAACACACCATTATATATCATGTTATTATTTGTTCTTTATTTCTAGCTATTCTCGTATACATTTAATTTAATTTCTATTTTTCTGTGTGCATTACTTTCCTTTGACCTTATTGTTGTCATGACTTGTATATATATATAAGTATATGTGTGATGATCAAGTAAATGTTTTTGGCAGTTACTCCCACGAAAATGTCAAAATTATCTTCTCATGATGATTTTTGTTTAAAAAATATAATTTATTTATTTTATAACATTTTAAATAAAATTAAGTCATACAATTTATTATTTAATAGTTAAAATAATATATCTTCACATAATAATAATTATTAAATTTTTATTGGAGTAGCCACCAATATTTTTTAGATGAGTAATGTACATGCATGTTTATTTCATTTTATAATAAAATTTTATTTTTATTTTTATTTAAAGAAATTAAAATAATAAAAAAACTAATTCTATGTATTTTAATTATAAAAAATTTAAAATGATATCATAAATTATTTTTAAAAATTTAAATTATTAAATAAAATGGAATAAAAATTAAAAATGCACCAATAATTTATACAATACAATAAGTTTATAAACAACATTAGGTATATAGTGGAGCCCACTGCATCACGTGTGCATGAATGATTCACGTGCATGACGCCTTTTGTTTTTGGGTGCTGAAATAAATGTGGATATTATATATTATATATTTGTGCTTAATAGTAAAATAATTAAGATTCTCTTGGTATTTATAATTTCTTTTAATTATAATAATTAAGTTTTTATATTTGTAAAAACTTTTAATTGAAATTTTAGATTAATTTTAAATTTGTAACTAAATCTTAATAGTACAAGTTGTAAAAACATAGATTAACAAAACTCTTTATTTTATTGTATAAAGCAAACATATGTATGTACTCAATATATATCAAATAAATAAATACTGAATACACATATATTGAATACATATTTAGCTATAACTTTTGACAATGAATAATTTTTTTAATAAAAAAATTTATATAATATAATTTACTTAGTTATTATTAAAAGTAATAATGTAATTATATATTATATTGATTTATGAAGGAACAAATTTAATTGGTAAGAAGAGTGTTAGATAAATAATGATTTTTTTGAACAACATGAACAACCACCAATCAAATAAGAACACACTACACCTCTAAATTATCCATCTAAATTTTAATATTAAAATAATCATCTGTACACCTAATAAAATAAACATTTGATATATCTATTATTCATATTATTTATTATTTTCATTGTTTACCTATACTTTTTCAATTGATAAATATAATTTCTATATTATTTTAGCTACCATCTATGTTTAATATTTTTGGTAAATAATTTCTTAAACCAAGATG

## >AdNAC18

GATTCGGGTATGCCTCGAAGATACCGACCCAACTGGTGGCATAGGTCGTACAGAAGGCGAGTCGGTCGTGGCTCGGGTTAGGTGTCAAAAAACATGAGTGTTTTGTAGAATTAAAAGAGTGTGAAGGTGTGTATTATATTATCCTTCAACCTTACATCTTAAGAAAGATAAAGTGTATATATATATTGACAACCGATGTCTAACACCAAATAAGTAGAAAATGACATCAAATCTGGAATAGTTTTTTCATTTTACAACATGATGAATAATATAAACAATAAATAGAATAATCATAAATACTTGAAAAATCTACTTAAAATGAGTATCATCTGAAAAAGATTAATTAATAAATCCTTAATGAGGTAGACACCTGATCAATTACCACCAGAAATCTGATCACAATACTTTTCACAAGCTGGGCCACAGACTTCAATCGTAGCTCCCCTCTCTTGCAAGCATACAGGCATGCATTGTTTGGCACAATCTGATAAAGTTTCCGCATAATTAAAACATACAATAGCTAACACCATGCATATTAGCATTGCAACTACGTATTTCTTTAAGACCAACATTGTTGAAAATTTTTTGCTTTTAGAGTTTGTAGTCTTCTGGTTATAGGATAATGAATTAATTAATTTTCTTCTTATATTTATATGCGTTCAAAAGTCAATTAAATATATTTTTAATCATTATTTTATAAAAGTAGATGATACTGCTCCTCCTCCTGTTTTTAAACGGTGGGGAGAGAAACATGGTAATAGCTTAGAATTTTTAGTTTAATTACTAATTTTGGAAGTATATGTATTTTTAGTTGTGTTAGGAGCCACATTTAAAAGAAAGTAAAATCATGTTTTAGTAATTATTAAAAACTATATAAGTATAAATGTATTTGTTCCAAAGTATTGTGATCATATTTCTGGTGATAATTGATCAGGTGTCTAGCTCATTAAGAATTTATTGATTAGTCTTTTTGAGATGATACTCATTTTATATATATTATTTTTCTTAAGATGTAAGGTTGAAGGATAATATGATACACACTTTCACACTCTTTTAATTCTACAAAACACTCATGTTTTCTGACACCTGTTCTGAGTCACGCTCGACTCACTTTCCGTACGACCCATGCCATCAGTTGGGTCGGTATCTTTGGGACAGCACCTAAATTTCCGATCTGAAGTCCGGTTCGACCTACCCCTTCCACGTGGACTAGAACAGTTCATAGAAATTTCTTAGTCAGTGGACTAGGTTATTCATGGACCCGTTCAGTACCATATGTAAAGGGAGAAATTAGCTCTCGCGCCAAAGTACGTAACACTTTATCTAATTCGGCCATCACCTCGTACGAATATTGACTTGATCGTTGGAGTGTCTTTACAGGTGACCACCCCCTCCGCACGTCATCTTTAGCGTCTTCAATTCACTCTTCCATCAACTCAGGGTTCAATCCGCTCACTATTCCATCTCTGCCTGACCTGTCGAACACCCGAGATCCCAAGTAACGAACACATTCTTTAATTTTTTGGGGAGGATGAATGAGATTGGAAACACCAGAGATGCTTTTAATATTGCATTTCACATTGTATTTGTGTTTGGGAGGGGGGAGGTGGGATGTGTGTGAGGGAGAGAAGGTGGCTTGTTTGCATAAGAAAAGCATGACATCAAAAACGTTCCATTCCCAAACTTCTCAACCCCTTTCTTTTTCCCAGGAAAACAGCCATCCTTTCCTCGTTGCAATCGTGGTCCCCACATCCACGTGTCAAANNNNNNNNNNNNNNNNNNNNATTTAGATATATAATTAAAAAAAATTATATGCATAATTTTGTATGCTCTTTTGTATATGTCTTTTTATTTTTTTACTAAAATAATTGATAAGAAATATTTTGTTATACTATAAAAATATACAAACAAAAATTAGTACAAATATTATTTCTTGTTACAAAAACTCAGATTTCCTCTCCTAATATCAAAATCAGCAAATTAAATAAATAAAGTGTTCACATACTCGCATAGGATATGTTCATCTCCTTTAAAATTAAGTATATTACACTAAAAATATTGTTTTTCTAATGTTTTTCATTTGTTTTAACGTTTTATAAAACATTTCAATAAAATCTATTTATCAAAATATAATGAATAATACTAATTTAATTTAGTTTATTTTTATTCAATTTTTGTAATTGTAACTAAACTAAACTGCTCGATAATAATAAATTTTTTTGTCACAACTTACAAAACAAATAACAATGGCGAATTTAAAATAATTTTTTATTTGGACGATATTGATGAATTTTGAAAATTTGAGTCAAATATTTTTTAATTGAATTTGAATTAATTTTTAACGTCCCTAATAATTGTCCCCGTTATCTACCTCCAGCGATGTCACTATCAGAGGGCTACTTTTTTCCCAAAATTCACTGAAGAAATAAAATGAAATGGAAAAAAACAAAATAGGTATTAAAAAAGTTGTATTGTTGTATGTATATACTAAGGTGTATAA

## >AdNAC19

TTTTATAAAAAGTCTTAAACATCTTTCTTTTATTTGATTAGACAAAAATATCCTTTTAAATTAATAAAATTTTAGAATTCAATTAATTCTAATTTTATAGTTTCAAATAATTTAAGCAACAAAACAAAAACATATTAAAAAACAAAAAATCAAAATTTCTTCATCTTCTCCAATTTCTCTAATCATTCGTGCCCCCTCTAAACAAAACATATAAAAAAAATAAAAAATCGATCGTTTTTCATCTTATCCAATTACCCCTCTAAAATAAAATAGTAAAAATCCCAAATCAAAATCCTAAATTTTTTGCCGCCGTCGTAAGACTGCATCAGCAGAAGGGATCGATTTGAACATGTCACCGGCAACAAAGTTCAAATTCCCATCTCCTTTGAGGTTACCAACAACCTCCGACTGGTCGAAAACGGTGCACTTCAACTGCGAAAACTCTTGATGAATAAGTTTAGAGACACCGCCAACATCAACAACAGAGCTGAGATTCTCAAACACGTGCCTGCACTCCTTGAGCGCAATCTTGAACATGTATGAATCAGCGGCGAAGAACTTAGGGTTTTAGTTTGGGATTTTTACTGCTTTGCTTCAGAGGGGTAATTGGAGAAGATGAAGAACGATTGATTTTTTATTTTTTTGATATGTTTTGCTTAGAAGAGACACAAATGATTAGAGAAATTTAGAAAAGATGAAGAAATTTTGATTTTTTATTTTTTTAATATGTTTTTGTTTTATTGTTTAAATTATTTAAAATTATAAAATTATGATTAATTGAATTCTAAAATTTTGTTAATTTAAAAAGATATTTTTGTCTAATCAAATGAAAGAAGAATGTTTAAGATTTTTTATAAAATTAAATAAAAAATATTTTTTTATTTTTATTTAATTAAAGGGATATATTAGAAAAAGTGGTGATATAATATATATTTAAAAAATAAAAATTAAATGTTGATGTGGAAAATAAATTCCACGTGGATTGTTATTATTTGTCCATATTTTAAACATATCGGTACGTATGAATTTATCATCGTACCGTAACAAACACCTTTGTGTGTGTGTATGTGGCCACAAATTTGGTGGCTATGGTGGGGTGTGTGTGTGGCTATGGACTTTACAAAATGATATTTTTAACTATTTTCAATCATACAAAATGAAAAACTGAAGCACGTTTCAGTTCTATTAATAAATAATGACATGTTTATGAGTCTGTAAATAAAAGAAAAAAATTAGATCATTTATCATATTTTTTGATAAAAAAATAATCTATATTTTCTCTCGTTGTCAGAAATAAAGTCGCTACCAATGCCCAAAATTTAAAAAATAATAGTATCTAAAAATACTTATATTGCATTAATTTTTTTCACGTTAATAAATAATTATTTATATATTTTATTTTTAATTAAAATAATCATTTTATTCCGCAATAAAAAATTGAAGTCTAAAGTTGCTGTTATTAATTAAATTTTAATATTAGTCAAGTGAAATTTAAAATTATCAATTACAAAAAAAAAAGTATAAGTAGGCAATAAAAATATTAAATAATGTGAATAATAGATATATCGGATATTTAATTTATTAGGTGTGCAAATAATTATTCTAATATTAAAATTTAAGTTAGTAGTTTAAAATATAGTATATTTTATTTGAATTATATAAATAATTTAAAAAAAGTCTAATAGTTAATCTATTATTTTTTTTATTTTAGTCCAAATTAAATATTTTTTATTATTTTAAAAAGAAAATCTTTTTGAAAAATTTAATAAGAGAAAGTATAACGAGCCAATGTCCTAAACGTACAATGTGTACAATAGAGGTTTAAAAAGTATCAGAGATATGACCATTAGTGTTACATTGTTCTATCAGGTTATGCTATTGGGATGAGTGGTTTCATAACATGGTATTAGAGTTCTAGATCCAAAAGGTTAAGAGTTCGATTTTTGGTGAACCCCAAAATCAGCTTATCTTTGGTGAACCCTAAAATCAGCTTAAGCTTATTGTACGCATAGGCCATTTACTCCCTAGCAGTACTCATTTAATAATATGTGATGAGAAACATAGAAACATCTTTAAAAATAGATGAAAGTGGTCACTTTAAAATATTTGTAAATGACGCGTTTATTGTGTAACTTTGCATGCAAAAGCAATACATAATTAACAAGTGGCAAATTGTTATTCCATTGTCATGATTCAATAATTATGCATGTTAAGTTTATAAGAAAAGAATTGATAATTAAATAATTGATTAACGTAAGTAAGAATATTTTAATATTTAAAGAAACACTAATTAATTCCCTATTTTCTTCTACTAATATTATTAAGTTTCTATCATTTTCCAAAAACAATATTGAATTTGGTGTATAACGTCATTGCAAAGTCAACTAACTTGTCATACATGTTATTGTTGAATAATATTGCTGGTAATTAACTTTAGTGTGTGTATATATGTGTCTTAAAACCAAAGTAGTTAGCTTTTGTTGTTAGTTTCCTTGGCCGC

## >AdNAC20

AATTGAAAGGTGTGATTTGTGAGAAGATAGATTCTCAAAGATGTAGGAGAGTATCATGTATTTTGTACAGGTATCCTTTATCTGTGTTTGGTGGGTTTGTTCAATTTCAGACCAAGTACGTGACGGACGAAGCGAGTATGCATGAAATGTTTTCAATGTACATGGAAAATCGCCACCAAATGTCGTGCATCGAGTTATATATTGAGTTTGAGCAATCTGAAGCGGACCGTAACATTGAATTGGAATATTATAATAGTGAAAGTGAAGATGAATTTGAAAGTAACTATGAGATCGTCGGTCCAGGTAAAGACGAAGATGAAGCTGGCGGCACCATGAACACATATGTGGCAAAAGTTACAAATGCACTAGCAAACCCGCATCCGTTTTAGGAGCCTTCTTTCATGTAGTCGTTGGATTTAGAGGCTATGCACGCACTGGAGTTTTCGCAGTATATGAATGCAGGTGCGTAAACCTAGGTAGCTATGTGAAACTCTGAAGTAGCAAATCAATAAGTCGGATGAGTAATGTATGTGATATGTGACTAGGCATTTGTGACCACATCGTTTGATTTTTTTATTAATTAATAGTTCTTTGTTATGAATTATATTTAGTTGTTCTTTACATAGATGGAATAGCGAAAAATAAGACTGTAATGATCTTACATGGTAAAGTGTGTTTATTAATTTTGATGTATGCAGCGCCTCCTATTGTAGCGGATGGTGAGTTTACAGTGGGGATGTAATTCAGTTCAAGGGAGGCAATAATCAAGGCAATGAAAGATTATACCATCTAGAGAGGTGTGGACTATCGGGTATATGAATCGGAACCGACGACATTCTATGCCAAATGTACAGAATATGGGAATGGTTGTGACTGGTTGATAAGGGTTACCAAAATGCAGAAGAAGTACTGTTGGGAGATAAGGAGGTACAATGGAAGTCACACTTGTACCAGGTCTACTATTTCTCAAGACCATTTGAAGCTGGATTCCAAGACAGTTGCAGAAGCAATTAAGCTGTTGGTAGAGGTTGACCCGTCTATAAAGGTGAAATCAGTAATTGTTGAAGTCCAGTCAAAGTTTAACTACAGCATTAGTTATCGCAAGGCTTGGTTAGCGAAGCAGTAGGCGGTGGAATCAATTTTTGGAGGTTGGGAAGCATCATATGAAGCTTTGCCCATATGGTTTGAGGCCATGTGTCATAAGGAGCCATCAACAGTGATTCACTTTGAAATAATGCCTGCTTACCAGGGGGATGATTTGGTTCCTGATATACGTGTACTGCATAGAGTCTTCTGGAGTTATTACCCTTGTATAAGGGCCTTCAGACACTGCAAACCAGTGGCGCAGGTGGACGGGACTCATTTGTATAGAAAATACAAGAGTTGTTTATTGGTTGCAGTCTCACAAGATGGTAATAACAACATCGTGCCTATTGCATTCACCATAGTGGAGGGAGAGACTTCTGATGCATGGTACTTTTTCCTGAGTAACTTGCATCAACATGTGGTGACACGTGATGGTGTGGGACTTATCTCTGACCGTCACGATTCTATTAGGTCAGCTATTGAGAGAAGTAATGGGGTTTGATCTCCTCCTAGAGCGTTCCATATGTTCTGTATCCGGCATATTGAGTCCAACTTCTTGAGGAAGTTCAAGGCACCTTACTTGCAGAAGCTTATCGTCAATATTGGTAACTTTAAATACAATGAACTTTGAATTTATTGTAAGTACGATCAAATAGAATGGTGTTCATTGTTGACTGAATGTTTTTCATAGGATACTCGAGGACGATCAGGGAGTACCAGATGCGCTATGAACGATTAAAGGAAAGGGGTGAGGCTTACACCAACTGGCTTGATCGAATCCCACGTGAGCAGTATACTTTGGCATTTGATGGTGGATATCGATGGAGTCATATGACCACCAATCTTGTGGAGTGCATCAACTCCGTCTTAAAGGGTGCATGCAATCTCCCGGTGACTGCGCTTGTTAAGGCTACATTTTACAGATTGAATGAGTTGTTCACTAGGAAAAGAGCTAAGGCTGAAATCCGAATTAATGCTGGACTTGTGTTCTCTGAGATGGTGACCTCCAAAGTGCATGCAAATCAACGAACATCGGGTAACATACAGGTTAGTTGTTTTGATAGAGAAAATGAAGTATTTGAGGTACGCGAGATGCCAAGTGGGGTTGAGTATGCAGTTGACCTAGTAATATTATTTAAAAAAAATTCTTAAAAAAGATATAATTCTAACATAATTATTTCAATTTAATCACAAATTATATATTACTATTGATGAGTAATTATTATACTTTAAAAATAAATAAACTTTCTATTTAAAAAATAATTTTTTTATATATTTTTATATATTATCACGGACACAACTACATATAATAGTAATGTATAATAACAGTCTTTCCTACGACGACTATGACGACTTTATTACGATATAAAGGTTAGGCATCATGTCAAAAGGGAAAGAAAAAGATCTTTTAAGA

## >AdNAC21

TTAATTACATTGGAGACTCGTATATATTGCAGGATCACTAGGATTGACACTTACCAAATAAAATGCACTAATGTATATCTTGGGGCTTGAAAATGAAAATGATCAAATTCTTGCATGCATGTGTCTCAAAAGCACTAGAAGAGGGTCCGAAGTTTTTCCATTAAAGTCAATAGAGCCATCGACAATTTGGTCAAAACTATTACCAAGTATATCTTGGGGCTTGAAGATGGATAATTGGAAGAGGCGGTGGAGTTTTCTAAAAAGATATCATGCGACATGCATGGTAAAATTTTGATATTAGTCATGAAAAATTTTCATACTATAAAGATGTTTCTAATGAAAGATTGGTTAAACTCAAGTTGAGTTACACCAGAATTTATTATATATAAATAAATATTCATTGTAAGTTTGTAACTCTAACTGAAAATAGTTGTAATTGTACTTGATTAAATAAAAAATTATAACCATAAAATCTAAGCATAACGACTCTAACTAAATTTATACTGAAGCAATTTGAAGGAAATAGTCCTTCCAAATCCTAGGGTGATATGAAGATAAAATTTTTGAAGAGAAAAATATGACATTAGAATCAAATTTTTAGTGTGTTGAATAAATTAAAAAATTTGAAGAATTTTAAATTTAGATATTTGTAATATTTATTAAAAAACTTATATATTTAAAATATATAATTATGGTTAATAAAATCTATATATTATTGAGACAAATGTTTAAAAAAAAAGAGAGCATATAAATAAATAAATAAATAATTTTCTTAAACACCGATCCACTTGACTTTAAAACTTAAGTACCACATAAACTGTTACATAAACATACTTTCACCTGCTGCTATTAATTTGTTAGCTTGATATGATATAAGTCTAGGATAATATAAAATGAAGTAACCAATAATATAATGGTCCATATAAATCAAGGCGAAAGGATGGTTGGGACCTAATGATTTTCACCTTTATACTCAAATAGAGCAATATGCTTTCTAACAACCACCAACACTAGACAAAAAGTAGGGACGCTACTTCCAATCTTAGTTAATCTCATAAAATTTACAAAATCTACCTCATAGATCCATAAGCACACAAACTAAATTAATTATATATATAATAATTATGAATTTCATAAGTAAGGAACTCATCAGAGAGGAGGGAGTAGCATTTGGATCACTTGTACTCCCTTCATTTTAAGAGATTGTTAGTTTTGTTATTTGATAAGCAATGTTGTTAGAAACTTACGATTCGACCTCTTTAACATTCTCAATATAATGTCTGTAATAAACACAATTATATTTAGGAAAAGTATAGGTAAATAATGAAAATACTAAATAATGTGAATAATGGATATATTAGATATTCATTTCACTAGATGTGCGGATGATTATTCTAATATTAAAATTTAGAGAGATTCGATTCCGCAACCTCTAAATGAATATAGAGAGACTATGTCATTTGAGTTATAGCTCATTGGCATTCTTTAGCATTGTTTTTTATTAAATGCAATTTGAAAAATTTATAAAAGGATATGTTATACCTAGTAACAATCGTAGTTGTTGGCTTCATAATAATAATAATAATAATAATAATAATAATAATAATAATAATAATAATAATGACTTAATCCATTATTATTAGTAGAGACAAATTAGATTAAACCAATACTTTCGTTACAAATCAAATATTCCCACTCAATATGAAAATATCTATTGAGTGGGAACGCTAGGGGTTTTCTTGCTATGCATGAGAAGTCTACGTGAAGTAATAGAATTATGTTGGGCTATGAAAACAATTGTTCTCTTCAATTCAATTTCCCCCCTTATTATTAATAGAGTCTTCTTGGTATTTATATTTTTTACTTTATCAACACTCTAGGGTGACATTGGACCTAAATTCTTACTCTTTGAGCTTATACATTCGGTTGCCCCAATCCCACTCTAGCTCTAAATCCATATTTTAATCGTTTCTTTCTAAGTTTAAACATGATTTATTTAATTATTTCATCAAATTCATCATCAGAGCAGAGCTATGTACCTTTAATCAGTGTAAAATGGACAAAATCACGTCCCAAAAATTAAAAAGAATAAAAACCAACATTTATTCATCTACAAGTTTTCATATACCAAGTAATATATACAAAGCATACCTAAACTTGAAATCAAAGCAATGTTTTTATTCCTTTTTAATTTTTTTTTATTATTCTTATCTCTCTCATTGCAACCAATCCTATCTTCTATTCTTCTATCTCATTGTTAATCTCATTCTCTCTATTTAGTTTTGTTTTTGGGGGAAGGAAACTGCTTCTTGAAAACTGAAAAGAAAGCACAAAAAAGAGAAAGTGAGGGAGAATTTAATTCTTTAATTCCTTCCCACACCTCAACAAGTGCTAGCATGAGGGGGTGGTGCAATCTTTAGAAGAGACAAGTTTATAAACTTTTTCTCCCTTTAATTTTATATTTAATTAATAATTGTTATTACTAGTTACTTTATTATTA

## >AdNAC22

ATGTAACACATCAAAAAAGGTAATTTATATATTACTTTAGAGAGGATAGGATAACATTCACCAAGTGCTAATGAACTTAAAAAAAAAAGCGTGAGTGTTTTTTAGAAGATGACCTACTATTAGAGACAACAAAGGAAAATTTTAGACCTTTAATTTTGGATAAAATATATTTTTTATTTCTGAAATTTGCTAAAATTTTTAAAAATATTCTAAATATAAATTTTATTTTGTTTCAATTTTGTCTTAAAACTGTTCGATTTGCATCAAAATATTAAATATATCTCTGACAACTAATTTAACAATAATGCATGGGAAATAATATGTTTGATTTGCTTACGTTAAAAGTTGTTCATGTGGAATTATTCTTAAATTGGTTATAATTTATTTAAAAAATTAGTCGTTAAGAGTAAATTTAATGTAAATTGAAAATTTTTAGAAAAAAATTAAAACAAAATAAAATTAAAAAATATTTTTAAAATTTTTAACAAACTTTAAAAATAAAAAAATACTTTCTCTTTTAATTTTTATTATCTTACAAAAAATAAATAAAAATTAGTATTTATTTAATTAACAAAAAATATAATATTCTTACTTAATAAAAAATATTTAAGAATAAGTCTAGTTGTATAAATATATCAGATAAAAAAAGCGATATAAAAGAACACAACAACCAGAAAGATAACAAGCCAAGCTAAGATAAATTAAATAAGGCTTTTACATTAAATTCCCATAGACTCTATTGATTTCGAAGAAATTATTGAATTTTAAATTTTATTTTCAATAATTCTATTAACTCCATTCGATCCAACTTTATGAAAGTCTTATAAAGAGAGAATAAGAATCTCTCACAATATTAATATCTGTTATGTATTATAATAACCAATTTTTATTTTGATATAATTTCAATTTAATATCTTAAAATTATTCTTAATATTCATAAGCCCTTCAATTATTTGTGCACAAACATTGTCAATTTAAGAGTCGTTTGAAAAATTTTAAAAATGATATTTTTTAAATTTTTGATTTATGAAAAGTAATAATATTAATGTTTAGTGTAATTTTTAAAATTAAATTATAATTTTTAAAAAGTTATTTAAATGTTTATAAAAAATAAAAAATAACTTTTTTTATAATAAAAAATTTGAATTCATTAATAGCATGTTGATTATGATAATGAGTCATGATCCATGCCATGACTTGAAATAATGATGTATTATTGTTGTTACCATCCCTTGATGGTAGTAGTGGGTCCCACGTGGTAATTTTCATGTTTACCCTCATGTTAATTAATGGTGCTAATTGCGCAACTAAAGCAAGAAGAGAGACTGAAAATCTTATCTCCACTCTCACGCCCCACATACACTTATCATTCCAATGAATATACCCTCTTTTCAATTTCTTCTATCTTTTCTCTTTTTACAATAATATCATTATCAGTGAAAAATATAAAAGGAAAAATAAAATAGCACTGTCCTTTGTGGCCCCCACCCACGTGTCAACACTACATGATTTGGAATCCCACGTGTCATGCTCTCAACGGCCAGAATACCAGTTCACGTCATCATCAGGTACCCACCTCATCGCATGCACCCGCATCCACCTCATCCCTCTTTAGTGTTCTGTGTTCACACACCACATACACAAGTCAACACCCAAGAAACAAAATTTAGGTAATTATGGTCATCTATCATACTAAACAATTTTCAAACCACTTAAATTATATTATGTAATAAATATATTTATTAGTGTAAACATATTTTATATAAACACTTTGTTTTTATTAAATGATGTATAGCCATTACTAAAAGTACTATTTATACACTAAAAATTTATATTTTAATAAAAGATAAACAATAAATTTAAATTAATCAAATAATTGATTTATTTATCTTTTTAAATGAATATTAAAATTAAATTTCACTCTAAAAAAATTTTAAAAGTCAGTATTTTTATTAAAATTTAATTAACATTTAATCATAAAAATATATAATTTTTTATTATTAAATATAAATACTAATTATAAATATAAATTATAAAATATATCGATTCTCTAACACTATTATTTCACCTTATACATACAGTAGTTTATTAACTAACATAATTCTAACGTAAAATAAATATGAATGCTAATATTTTTTAAAGACTATTTTACTATAACTTGGAAAACTTTATGGTAATGAAGATTTACTGTTTACGAAAAGACATATCAGTCCTTGAATATGAGAGACACGTCATGAGAGCACAGGATGGAGAGATAGAGATGACCTCAGTGGATCAAAGCCGATGGTGCCCATGCCACATATAAAACGCAAGCTTCAGCACTTATTGAGGGGTAATGCAAATTACAAGAACAAAACAACACATGACGTTCCGTCTTAACGTGATCTTCGTCTGAATCCACCGTAGCCAGAACCAACCAACCAACTACCCAAATCCATAAAAAAGAGGGAGAATTATTGAAAGGAAGAGGAAGAAGAGAAGAAGAAGAAGAAGAAGAAGA

## >AdNAC23

CACTGGAAAACTAAAGGCAATATCATGGGAAGAACAATGGTCATCATCAGGCTGTTATTGCTGGTATCTTCACTTGTCTTATCAGCATCAAGAAGAATACCTTCTTCTTCTCATTCTTCTGGTAACATTGATTGGTGGTGCAACCTAACGCCACACCCTGGAACATGCAAGTATTACTTAGGTAAAAGCAACCAACAGCACACAACAATAATCAAGCACAAAACCGAGTTGAGGAGCATGCTTGTGAAATCTGCATTAGAGGAAGCAACCATCATGCAAAAGGAAGCACACGGTTTGGACCAAAACTTGATCAAGACAAAGAACCATGAAGCTGTGCATGGCGATTGCTTGAAGCTCTACGACGACACCATCTTCCATCTCAAGCGTACCCTCGAATGCCTTAACAACAACAACTGTTTAGCGGTTGATGCACAGACATGGCTCAGCACTGCTCTCACAAACATCCAAACGTGTCAAACGGGTGCACAAGAACTCAGCGTTCAAGATTTCAAGGTTCCATCTAAGAACACCAACGTCACTGAGATGGTGAGGAACAGCTTAGCCATCAACTTGGATTTTGTCAAGATGATGAAGCAACAACCACAAGCAAATCGCACATTAGCAGAAGCAGAAGTAGAGGGAGAAGAAGAAGAAGCAGAAGACGATTTTCCGAGCTGGTTTTCCGGTCACGAAAGGAGGCTTCTTCAATCTAGCGCGATAAAGGCTCACGTTGTGGTGGCGAAAGACGGATCGGGGAATTTCAAGACGGTGCAAGAAGCGCTGAACGCGGCGGCGAAGAGAACCGTGAAAACAAGCAGATTTGTAATACACGTGACAAAAGGAGTATACAAAGAAAACATAGAGGTGGAGAAAAACAACGATAACGTGATGCTGGTTGGTGACGGCATGAGAAACACCATCATTAGCGGCAGCAGAAGTTCTCAAGACGGTTACACAACATACAGCTCCGCAACCGCCGGCATAGATGGGCTTCACTTCATCGCAAGAGACATCACTTTCCAAAACACCGCGGGCCCACGCAAGGGCCAAGCTGTGGCCCTGAGATCCGCCTCCGACCTCTCTGTGTTCTACAAGTGCGGCATTGTGGGCTACCAAGACACGCTCATGGCCCACGCCCAGCGCCAGTTCTACAGACAGTGCTACATCTACGGCACCGTTGACTTCATCTTCGGCAACGCCGCCGTGGTCTTTCAAAACTGTCACATATTTGCAAGAAAGCCCCTGGATGGGCAGGCCAACACTATCACCGCACAGGGCCGAGGGGATCCCTTCCAGAACACCGGCATCTCCATCCACAAATCCGTTATCAAAGCCGCACCCGATCTCGTTCCTGTTTTGGACAAGGTTCAGACCTTCTTGGGCCGGCCCTGGCAGCAGAACGCTAGGGTTGTTGTTATGAGGACTTACTTGGACTCTCTAATAAGCCCATTGGGCTGGGATGAATGGAATGGATCTGACTTTGCCAAGGATACTTTATATTTTGGAGAGTATGAGAATTCTGGGCCTGCTTCCGATACAAGCAAAAGAGTGAAGTGGCCCGGTTTTCATGTGATATCAAACCCAAAAGAGGCTTCACAATTCACTGTGACTTCTCTTCTTGCTGGTCGCACCTGGTTGCCTACCACATCTGTTCCCTTCAGCTCTGGCCTCTGATTCTCATGTAGTTTGTTTTCAGCATTCATTCTTTTGTACATTTGAGCAAGCGAGAGAAATTATTATTTGTGTGTCTTTGATCGAAATTAAATTAAATTAAATTAAATTATTATTTCAATTATATCCTACTCGATGTATTCTCTTTTGTATGAGTTAAGACAAATGAATGAAATGATGATCGTCACTTTTATAAAGACTGTTGAGTTTAGCTTTTTGCTCTATATGGTAATTATTAGCCACTGGATGACTGTTTATTATATGTTGTCCAATTAGAATTAGCAAAAGGTGAATTATATATTATTAGACTAATTATTATAAGGCCAATATGAAAACCAATAATAATGGTACAAATTACACTTATATTAGGTAGATGAAGAGATGGATGAAATAAATCTAGTGTAAGTGAAGATGATGATTATTATGAAGGAGTGTGGGCGTGGAAGCTGGCTGCTCCATTACGTCAACTTATCCCTTTTTGTGTTTGTTTCTTTCTTTTAAGATTTGGTGATTGGTCTGAGCCGTTACGTTAGTTCCTCTTTCATTCATTCAGCGCTTAATAGCAAAGATTAGTATATAGAGATAGAAAAAGACCCATAGCTAGCATGTTCTCTTGTTAAGCACTCTTTGTTTGGTGGCGGGTAGAAACAAACACTTTGATCATCCCTTATGCCATATTTTCTGCCACATTTTTTTGCCCGCTTTCGCTTCTAAAAGCTTTTCCACCAATTCAGTCCTGACAGTGAGACATGGATCATGACACAAATACAATACATCCATTTATAGAATCACTTTAGTTTCATTGCCACAAAACCAAACACACACAATT

## >AdNAC24

AATATTGTTATATAATTATAATTAAGATAATTTTCTGAAATAAATTTAAAAGAGATTAGTATAATTATAATAAAGAGATTATCTTAAATTAGTATAATTATGTATCATTCATTACATACTAATTATAATATAATTATATCAATTAAAGATAATTTTTAAAAATAAATTTAAAAGAAAGAAATAGTGCAATCATTTTGGAAGAAAAATGGGTTCACGAGAAAGTGACACCTTACTTTCATTAGTTGAGGGAAAACTCAATTTTAGTATATTAAATAGAAGTATATTGGTATAAATTATAATAAATTATATAACATTTAAAAAGAAAATAAAATGAATAAGAAGAAAATAAAAATAAATAGAATAGACAGAAGACTAAAATAAAATAAATTGAATGTGAGAACTTTTTTTTGTACATTTCATATCACATCCGTTTCAATAATAATAATAAATAAAAATACGTCAACTTGATATCTAAGAAAAAATGTTGAGATAAAATCATAATACAGTTCTAAAGTAAAAGCTTAAATTAGAACATAATATCATTATACAACACATATTGAAAGTAATTTCACACTACAATATATCACAAACAGGTAAATGACTTAAATTTAAATACGAAATATTTTTTTATTTATGCATACATGATAACACCATGGTCTATAAATACTTCATAGATAACATATCTTATAGAGCTCCGAATGATGAGCACGAAAGTTGGAGAGTGTGGTAGTTTGTGTCCACAATAGTTGTCTTCTTGCAATGACGCCTCATAGGAAGAAAAAGAATTGTTGTAAAAGCTATCAAATGAAAGAGTAATTGGTAAACAAATAATATTTTTTTCTAGCATACATGGTCTTTTATAGTGATAAATTAAAAATGGAAGGAATTAAGTTTTATATTTTTATATTAGGAATAGAATTTGATATTTATTCTAATAAAATTATTATTATTATCAATATAAATAGGTTAACAACTTGTAATTATGCATTATTCATTAAAATAATTAAAAATATTTTTTATTAATAATTGATAAGTAGTTTAATAATACATTAAATATTGATTTTATATAATTAAAATAAAGATATTTTCCTAAATTAGTATAATTATGTATCATTTATTAAATGCATTTCTTTTGGAGGGAAAATGGGTTCACGAGAAAGTGACACCTCACTTTCATTAATTGAAAAAAAATCAGTTTTAATATATTAAGTAGATTTTAAAGATACCTATCTAGACAACTCATGACGTGTCACTTGTTCTTATTCCTCACAAAGCATGGGGAATAGATAGGAGGTGTGAAAATAATGGGATTGAAATGAGAATTTTGGATACACATGAATTGTTGTTATAAGGGAAAAAAATGATATTTTTTTCCAAAATCGGCCATAGAAGAATTAGGTATATAGAAAACAATCAATCATACTGAATATAACATTAAGATTATTATATTAAGAACTAGGTCCGGCCAACAACTTACAAAGAACAATTTTAAAATAAAAACAGAATTGCCTTATTTTATTAGTAGAGAAAATGAAATAATTGTTACTACATTCAAAGAAGCTTGCTTTGTCATTTTCCCCTTGTCTTTGTATGTTGAAGTTGAAAGGGAGAAGTTTATGAGCATCAGCAAGTGTGAAAAATGGAATAATCTCCTTTTTTTTTCTTTTTTCAAAAAAAAAAATAAATTGGAAGAAATAAAAAAGGAAAAAAAACAAAACAGTATTGAAAGCTATTGATTCTATAGTCTCTTGTGTGATCCTTATTTTGGTCTTTCCCAAAATCATTATATATTAATACTGGTCTCAAATGGTGGAATTGAGACACATCCATTAAAGGGTATGTTTTTATTTTATTTTATTTTATTTTATTTTATTTTATTTTATTTTAACTTGTCCTTCATTTAAGTGACATAATTCCTATTCACTAAGGAATTTTTGTGATCTCAATTAGAGAAATATAAATATATCCTTAATGAAAGGGAGGCAGAAGCTTAGGAAGCATATATAAACATTCTTGGGAGGCACATAAATTTCCTTGGACTAAACAAATTTATTGTAAGTGCAACAACTTGAAACTTCTCAACATCTATTTTTTGAGGTATACTGTTTCATCAATTATTTGTTCTATCTTTTTGTAGTATTATTAGAGAAAACAAACATCACATTGATAATTGGTTTTGTTTGGTTTTTCCAAAAATTATTGCAAACCACAAAGTTTTTAATATTCATCTATAGAAAGATGGTACTAATCTAGCAATTAAATCTTACATAGCTTAATTATATATATACTTAATATGCTTTTCTTCTCATTATTGTACAAATTTATCAAGAAGTCTTCTCTTATATATCTTCGAATTTGATTGTTAGAGATACAAGAGTTTTTTTTTCTGAAACCGTTGCTAAAAAACTGCCGCTATCTGTCGAATTTGGTACAGTGAGAAATTGAATTGGAGATGGAGGAAAGGAATATTGAGATGGAGAATAAGATTGAAGATGAGATG

## >AdNAC25

TAGAAAAAAGTCACTCAAGCTATAGTAAAACATAATCTTGTGTTTAGCTTTGTTGAGTATGAGGGTATTAGAGATTGGATTAATTACATTAGTCCAACGATTATAATGCCTTCTAGAAACACTTTAGTTTCAGATCTTCAAATGATTTATTCAACAGAAAAAGAGAAATTGAGGCAGAAGATGTCTAGGATACCTAATAGAATTTGTTTGACATCTGATGTGTGGACAGCATCTACCACTGAAGGATATATTTGTCTGACAGCTCATTTTGTTGATGAGAATTGGCGACTAGTGAGTAAGATTTTGAATTTTTGTCGAATGATTCCTCCCCATACTGGAACCGATATGGAAGCAGTCTTATTTAACTCTTTGAAGCAATGTAGTATTGATAAGAAAGTACTATCTATTACTTTAGATAATACTTCTGGAAATGACAACATGCAAAACATCTTGAAAATCCATCTACGTAGCAAAATAGTTTGCTTTACAATGGTGAATATTTTCATCTGTGTTGCTCTGCTCATGTTTTGAATTTAATTGTGTAGAAAGGGTTAAAGGTGGTTGCTGAAGCTTTATTTAAAATAAGAGAAAGTGTAAAATATTTGAAAGCTTCATATGGGAGAATTGTGAAATACAGCAAGCAGAAATTGAAGAAGGTGTTGGTCTAAAATCAGATGTTCCAACTCGATGGAATTCTACATATATGATGTTGGAAAGTGCAATTAAATTTGAAAAAGCGTTTGACATCCTTAGTGTTGTAGATGGAGCTTATAAAGATTGTCCGACAAATGAAGAATGGAGCTTAGCAAAAAAAATGTGTGAATTTTTAGAGCCATTTTATGAAACTACAAATCTCATTTCGGGTTCATCATATCCAACATCAAATTTGTATTTTATGCAAGTTTGGAAAATTGAATGTCTTTTGGAAGACAATCAAACTTGTGATGATGTTGTTATTATGAACATGACTTTCAGAATGAAGATGAAGTTTGATAAATATTGGAAGGATTATAGCACTGTCTTGGCTTTTGGGGCAATTCTTGATCCTCGATTAAAGTTAAAGTTCTTGAGGTTTTGTTACAAAAAACTAGATCCTTCAACCTTTGAATTGAAGGCAAATGAAGTATTGGAGAAATTTAAAAGGTTGTATGGAGAGTACATAAATACTTTTGGTGGTTCAACAATTTCTCAAAGTAGTAATCAATCTCCTATGTCACCTGAAGAAGGAAGGCTTACAAAGAAGAGCAAAATGGTGATGAAGGTATTTAATTTTAATATAGTATTATTTTTCTCCTATTATGTTTATTCGTTACTTAAATAATCTTTATACTACATCTTTTAGGAGTTTAGAGAATTTGACTGTGAAACCCAAACTTCCAAAGATAAAGATGAATTAGAGATTTATCTAAAAGAAGGTTTGATTCACACCAATGAAGATGATTTGAAGTATGATGTGCTGAATTTTTGGAAGATTAATGAGGATAGGTTTCCCACTCTGTCAGTTATGGCCAGAGATGTTTTAAGTATTCCCATCACTACGGTAGCATCTGAGTCTGCATTCAGTATTGGTGGACGTGTTTTAACAAAATATAGAAGTTCCACTCTTCATGCGCATGTTCAAATGCTTATTTGCACAAGGAGTTGGTTACGTGGATTTGTTCCAAATCATGATGGTAAATATTTATGCTATTTTTTCCAAATAATATTTTAAATTGAGTTTCTAATTGTTTTTTAATTGGTTGATTTTTGATGATGAAATTGGTGAAATTCATGAAGAAGAAGCGTCAACAGAAACGATGCATCCCCCTCAACATTNNNNNNNNNNNNNNNNNNNNNNNNNNNNNNNNNNNNNNNNNNNNNNNNNNNNNNNNNNNNNNNNNNNNNNNNNNNNNNNNNNNNNNNNNNNNNNNNNNNNNNNNNNNNNNNNNNNNNNNNNNNNNNNNNNNNNNNNNNNNNNNNNNNNNNNNNNNNNNNNNNNNNNNNNNNNNNNNNNNNNNNNNNNNNNNNNNNNNNNNNNNNNNNNNNNNNNNNNNNNNNNNNNNNNNNNNNNNNNNNNNNNNNATATTAAAATTTTTTATTTATTTCATAATAAAATTTATATATTTAAAATTTAGAAAATCTAAATTCTTAACATTTATGTTAACGAAGTATTATTTATTATGTGATTTTTATGTTTTATTGTTTGTTTTGCCATATTTATCCGTCTTCAATATAGCAGCTTGATTATTTAGGAAGTGTTAGCTGACAAAAGTATTATTTAGTATCTACGTAAGTATTAACTAAAGTAGTAAAGTGCTAGAACCTTCTTTCGCCTCTAACATGGGGTGGGAACTTTGAACTTTCTATATCACCAAAAACGACTCAAAACGGGTCTCGTTTTGAACCTTGAACCCTAAATCCCCAATTCAAACCACTCTCTCTGTGATACTCGCAAAGCTCGCTCTTTTAGCTGATTAACCTGCTGTTCAATCGTCATCGTCATCGTCATCGTCATCGTCAT

## >AdNAC26

GTCAGATTCCAACATTGGTGATCCATGAATCAAATGCACACTATACCCTTTTTCTTATTCTTTTCTTCTTTCAGATTCAAATCCTAAGAACCATTGTATGTGCAAAAGTACTAATACATATGACTGTGTTTGTTTACAGAATAGTACTGAAACATGAATATAAAAATACAAAATTGTATTTTTTCTCAAATTTTTTGAATGAAAAAAAAATGAAAATAAATTATATTTTTATAATTTGTTTTATTTTATTACCAAATATAATATACGAATACGGTGACACATGTTTGCTGTTTGATTTGGTTAGACACAAATTTTCAAAGGACACAGAAGAATATTAAATTTGTGTACTTTCAATTTGGTGAGACACTGAAACACAACTTATGAGATACTAATTTTTTTACTCTTTTACTTTTATTTAATTTTTTATTTTTAAAATTTGTTCTTCTATCTTTCCAATTTTTTTTAATAAAGGGTAATTCAATCTTTTTTAAATATTTGTGTGTTTTGTTTATTATTTTTACCAAACACAATACATAGACACAAATATTTTATGTCTATGTCTTTAATGTTTATTTCTTTGTGTCTATGTTTCATCTTATATATCAACCAAACAGAATAATTTATCTTATCCTGTTCTCATAAACAAACGCAATATGCTCATATATCATACATTCAATGAATTAAGTAATTAACATAGTACTATATATTGGATCATAAGTGGCTGTTTCATTACAACAAAACATAAGCTTTCAGATTTTTGAAACCCCATTACCTAATCAAATAATGCTGTCTAAAAAGGAGATAGAGCTTCATTCACAACTATACAATTACTGTGTGTAACAGCTACTAGTCATAGTAACATTCTTTTTTAACCATAAAAATGAAATTACATATATATAATTTTTTTATGTCGGCCAACAAAAGGAAAAGAAGGATAAAGGAATAAGAACGTGTGAGAGATAAACATGCCTAACTCTCTGACCCACCACTCAATTACCATAAACATCAGCCAAACTTGCCTACTCAAATATTTGTGCCATATCATAATTCTCTTTTAGTATTCTTGATTTCTTTCCACACTCTGCCTAATTGACCTTCATTAATTATACATAATTGGGATTCTTTTGGAGGTCTCAGTCATCTTCAATTGTATAATTACTTGACATCATGGCAACAAAATTGTACACCATGGATTTTATTGCAACTTGTGACTTATGTTAAAGAATCAAGGTAACTATGGTGATCAAGAACCATGCTCATAGTCATAGTTTAGGAAACTTGCTCACTGGGCCTTGTGGAACAAACATACACAATTAAAAGTACATGACAATATATGTAATTTGATCCGTTCCCAAATATTATACAAATAAAAATACTAAGATATAATAATATAATTGCTGATGTCAAAGCCAGAAGAAAAAATCAATGATAATTGAGCTGCAAAATACCATTTATGGTTAGAAAAGTTATAAGCAAAGTAGTAGATTCCTAGTGTGTAGTTATTCTTTTTTATATAAGATGTTTATGTCTTCTGCAAGATTTAATAATTGATTAATTGTATGAAATAATGTGGGGCTTGTTTGGCTCCACCAAGGAAACAAGAAAATCAGTTAAACTCTCCTTTGCAAGGAAATGTTTTGAGTGCTTTGTTTTCATGTGGAATGTATGTTCAAGTTCCCACTCTGATCTGATCCTGCCATTAATTTCATTGATTAGAAAGCAAGGCAAAAAGCATATGGGTAAGGGTAAGGGAAGCAAACAAGAAAAAAGGTTCTTGTATTTAAAATACAAACCTATGATAATTGCTAACCATTATGAAGCATATGTAGGTAGGTCTACACTACTACTTCACTCCATGAGGTTGGTCACCCCAATTCAACTGCTCCAAGGAACCCAAAAATTATACTCATGCTTGCCACGTGGATATCACAGGTTGATGATATCAGCATGAAGTGTCATCATGACCCACACTATAGCAGTACATACATTTATGTTATTATATATCAAACATTTTTGTCTTTTGAATCTAGTTTTTTACACCATACTTATAAACAAAATTTCTCACATTTTTTTTCTCTAAAAAGCTATATTGTATCTGTTTCTATAATAAACGAATCAATAAAGCTATCAAAAGATTTCTTCTGCTACATACACATTATGATTAAACATTTAGAGGCCAATCCTCCAGTAAGATTCTGTTACACTTCCGCCGCCTTGTATGCTTGGTGTTCAGTGCTTAAAGTGCCTCAAACTTTATCCAAAAATATTTTTTGATTTGATGAAATATTGAAAATTTATATCAGTGCTGTATTTGTACATGATAAATAAGACTCCAGCTTCAGTTTCACGTCATTTCTCTCTCTCTCTTTTCTCTCTCCCTCCTTTCTTTGCACTATAAATAACACACATACCTTGGACCACCAACACCAACACCTTCATTCTCCGTTTTCTCACATACCCTTTTTGTAATAGCAGCAGCTGGTTTGGTTTTTTTGCTTCA

## >AdNAC27

AAATATATTTCGAACTTTTATCACTTATACCATTCTTTTTGATAATATGATAAAAAATTATTATGTATGCACTTAAAATTATAAGATGGAATTTGACTTCCATTTAAAATATTTCAAACGCTAAAGATCAAATGACTAATAATATATTCGAATACAACATTTACTTTTTAGAAAATGATATAATGCTACTCTTATTTTTTCTGGCATCATTTTACATATAATTTTAAAAATGATATATTTATATAAATTTAGTGGGATAAATTTGCATTGTTGACTATATTTGTATAAATTTTATAAAAAAATTAAATAACATTATAAAAATAGGAGTGAGAATACTGGTTCTCGTATTTAGAGGGTTGATATGTATCACGCAAAAAGAGGCATAAATGGTTGTGTTTGGAGGGTTTGACTCGGTTTTAAACTTTGGTTACACGCTTGGGATGCAAACCCTAGGTCAAGGAACAGACAAAAGGTGGTTCCAACCGAGGGTTAGATTTTTGGGACCTTCACTGAATTTGGAGTCCCAAGTTTGCCACTTGACTCGGTGAACCTTTCCCATTAATTAGTCGTATGGCACTTGCCATCCAACCACTAGACATATTTGGCAAACTTTGGTGTAAAATATGTTACAAAATGTGTCAAAATCTATGATGTTATTAGTACTATTAAAGTTAGTGGGAGGTCATGAATGAAATTGAGAAATTGAGAAATCCCATAATTATTTTGTATTCCCAAAGCCTAGAGGAAATTGCCTTTCACGTGTGCTATTACCACAACGCCCCCGTCAAATAACAAGTTCCTAAAGACATTTTTTAAAGTTCGTCATGTCAAATAATTTGAGGCTCTTTTGTAATCTATAATTTTTTTTATTTGTTGAAATTTAGGTTGCGTTTGTTTTTTTTTTAGAACAGAATAAGATAAAATATTAAAAATAAGACATAAAAAAATATAAAATTTAATATTTTTAATATTTTTGTATTTTATTTTATTAGAATGAACACAAAATACACTAATTTAATATTTTTAAACATAATATCTCTATTTATATTCAATCTATTAAACACAATTTTATGTCTCATACTCTTTGTCTCAATATCTTGTACTTATAAAAAACCTAACCCTAAAGATTGCTTTTGGTCAAAGTTCATCTTTCTAATATGTTGCTTAAAACCTGATGATCTTATCCATATACAATTATGCATACACCTTGGAATGGACCCGTGATAATCCTATTGGATCATGTCTCAACTATTTAATTTCTAACTGCTACATATTAGTCTAGTTCAAAGCCGATGATTTATGAAAACACAAGTTAAAATTCAAACAAGATTACCTAAACCATAACTGCTATGTAGAAAGTCACTAGCATTATTAAAAATAAAAGATTACACGAACAAGTACATCACCTAAACGGCGAAACCCTAAACTAGTGTTCGAATTTTGATGATAAAGACAAGAAAAAAATGGTGGAGAATTTTCCTTTGGCCATAGAAGAATGACAAAATGATTATAAAGTGTTTAAAAATTTATGACTAGAAGAAGCTAAGTTATAAGTCTGAGTATGAAAGCGAAGAACTGAAAAACAAAGTTGGTTAATTTAATTAGTAGCCTTCGGGCCATTGAAAAGATAGATTGAGTGAAAATTAAGAAGGTCAAATAATAAGCCCGAAGTCAGAAGCATCATATATTGCAGTAGTCATAAATTTTGGCTAACTCTGAAATTGATAGAGTAAAAGCGGTCCCCTACTCAACCACATTAGATTTTGATCACACGTCCGATTCTATCTCTTGTTGTTATCTAAAATTTGACATTAAACAAAAACTATTAAACTGTTTCGACCTGACAAACCCAGTGGAATAGAAGCTCTGGGGAATGGAATAGAAACGGGATTTTCGGATTCTGAGAAGCCTTAAAACCAATAATATAATCCAAAGTTAGTCTAACTGGAATTCGATGTTGAATTATATATTGGAGCTCTGTTTTGGAATCAGGTTCAGGTCCACGTTTCCAAATGATATTTCAAAGTTTCAGACCCTCATACATAATTTTATTATTTTTCCTTGGAACTTTGCTTTGTCTTGGCCTCTAGCGGATAGGCTCAAAACTTTCATGTCATTACTAATATGTTACTTACCAAAAATGCATATTAATCTTAAATTAGTATCCACAAAACTTAATCGATACCTATATACAATGAAACTTGTCAAATTTAAATATTTAATAATAATAAATAATCAACTACACTAAATATAAACAATAAAATTTATCTGTGTCTAATATATAATAATTATATAGTATTTTTTATAAACAATAAAGTTTATGTGTGTCTAAATAAACACCAATGTTTTTAGTGTAAAACCAAGATTGTTAAATTAATTAAATACGGTAAGATCGCAATCCCCAGATGCTGCTAGTTTGGTGGTGTATGGAGATTCCAGCTTTTGGGTCATAGACAAATAAAACACAGCTCTTTAATTAAGTTGAAAGTATGAATGAACA

## >AdNAC28

ATATCATTAACAATATTTTTATTCAAAAATTCATGTGCATGCTAATTAAAAATTAGTCACTATATATTTATATATAAATATATATTATTTAATTTATTTTTAATATTTATTTTATATTTAAATATATATTTTATATCAGTAATTAATTTAATTTAATAGTTAATTTTTTGTGTATATACATAATATGATTGACTTTTATTAATATTTATTTTTGACGATCAATAATAACGTAACTAATTGTTAATAAAGATATTGTTAATGAAGATTTTTTTTTATGATTAAGTGACTGTTAAATATATCTTTGAATATTTTTTAAAATAATTATCATTGATCGTATTAAACTTTTAAATTTTTTTTGGGACGAATAAGTAGACCATTTAACCAATCCAAGTTAAACGTTTTAAATATCAATTTCGTTACGTTATCAACAGTATTTTTGTCAACTTCTACCAATTCTTATTTATAATTGTGTTTAATAGAAATGTCTTTGAAGATGTGTCTAATAAAAATATTTTTTTATGACTATATTTAATAAAAGTGTCTTTATAAATATATTTTCTGAATGTGTCTCTTTATATATATTTAAAATATAATAATTAATTATTGTTAGCAATAAGTTGAGAAATAATATGTTGGCACCTCTACTTTTCCTTTAAATATATGTTTATTTATCTTGTTTCTTTTGTATCGCTCAAGACAAATGAAATAATTAACATTGAGGACATACATACGTACTTTTGCTTTGCACTTTTACTACCCTTTGATGATTATTAGAGCTACATTATTGTCATTTCCTAGTGCTGTATAGTGAACAAACAACTACTCAGTAGGTGTCTAAATATATGTGGTCTAATTAGCTAGGTTGGTTAAGATGATGAGATCATTTTTTACATTAACACACAGATACAAAGCCTGCAAGGATTGTATCATTAGCTTAATAATAATAAGCTGAACAACCATCACTTAGCTTATGAATCTCAGAGAATTAATTAGAAAGGGACAAGAATAAGAGAGAAGAAGGAGCTTATCAAGATTTGGCACATGTATGTCTAATGTCTCAATAGAACATCCATAGTGTCACTTCATGATTGATGATCACAAAATTTGATTCATCAATTACAGTAGCAAGTAAGAAGGGGATAGATGGTGAAAAATGGTAGGGACTAAGAATTGGAATGGGGCCAAATAGGGGCCACCATGCCTCATACATAGCGTAGTAACTTTTCCTTTTTCTTTTAGGGCGTTGGCTGTTCCATTACGTCACCACCAACATACACACCCTTTTTAGACATTTCTTTACTCTCATTTTTAAGAATAGAAAAAAAAATTAAATTGAGTAATGTTATGAATTTCGTGGGGATAATATTTAGAAAAGATTTCATCTCGAAGGCAACACTTGAAAAAGAGTTTTACAATGCTTTTAACTCTTCGTTCATATAAATTAACTTTTGTAATGATAATAATTTTCTTAAGGTCTTGTAACATTGAGAACTAACCAATTAAAAAGGACAATTTGCATAGAGTTCTAATTAGCAAAAGGCCAAATGAAGTATCAAAAAATAGAATTGTTGAAATCCTGGCTCTTAAAAAGAATATTATGTTATGAGTATTGTGGGATTAACATATAAAAAAGAGTTTTATATCGCAAGGATAAAAAAATCTTATAAAATTTCAAACTCTTTCACTATATAAACTAGTTATTGTAGTAATAGTTCTCCTAAAATTTTATAGCTTATCATAAATAATTGCAATAGCAATAGTATATTATACATTAACAAAAGAATTAATACAATAAATTTAGAAGTTCCAATAGATGATAATATTATATATATTACAAAGAGCAATGATACGTAACTAATAAAATTTATTGTTTTTAATTAATTAATATTTGACTAACAAAAATAATTTTAAATCGATATAAAAAATATAATCATATTATATACATCCTACTAAAATCAATTATTAATATAAAATATATATTATAAAAAATTAAATAATACATATATGTATATATAAATAATAAATAACTGATTTTAATGTATAAATAATATTTAAAAGAAATGTTAATTTTAGGAACTCCTAAAGTCCCTTTAAGATTCTTCATGTGTCTGTCCTCTTAGTTAGTTCCTCTCACATTCAACCCTTTAACTTCCTCTCTCTCTCTCTCTCAAGCTTCTACACAAGACAGATATGGATTTAGAAAGAGACTCTTAGCTTATTGAGTGAGAGAGAGGGAGAGCAAAATCTGGCATTGTCCTCTGTTGGTTTATGGTTAGGTGATGGTGGTAGAGGTGGCAGCTGAGAGATCCAACCATTTGGCTCTATCTGCCATCTTCAATTCCTTCTCTTCAAAACTTTTGCACCAATTCAGTGACATATTCTTATTCTTTGCCACATAACAAACACAACCAAACTGCATCCACTCATTAGGGTTTCTATTTTATTCCTCATCACACCTTGCTTTCTGTGCCTTCTTCACTTCTTGTGTTTTCTTTCTCCT

## >AdNAC29

TTAATGATAGAAGAAACACATAATTTAAGAGAATTATGTTCCAATATCATACACTTAGCTCCTTAATGCGTAAAAATATTTGTATTTCTTTGACATATATCATCAACTCATAATTTATTTATTACTGAATAATATTTTTAATAGATTTAGACCAATTTTTTCTATGATAAATAAATTGTGGATGAAAAATATACCATTTGATATGATCTCCACAAGAATGGTTATATTTATAATACGTTTTTTCATATAAAATTTTTTATTTATCTTATATTTTTTTTTGGAATAATTTTAGNTATTAATAGTTAAAAAAATATACAATATTTAATATAATTGATTAGTTGCATATAATAAAATTTAAATAATTTTAATTATGTGAAAATAGATAAATTAAAAAATTTTAAAATTTTAAAAATATTTAATTATTTTATTGGTACTTATAGTTTCACCAAGTTTATAATTAGATACTTATATATCTTTTTCTTTTAATTGGGTTTCTATACTACTTTTAATTTTATAATAAAATTTTTTTTTATCTAAAAATATTAGAGTTAACCGAATATTTTTTTACAAATTGAAAATATTCACAATTAAGAATATAATTAAATCTTTAACCACTTACTTTTTTAAGAGAAATATTTTATTAATTTGAATGTTTTTGATATAAAAAAATTTAATTATAAAATTAAAAACAATAAAAAAATTCAATTAAAAAAATATAAAAATTTAATTATAAATATAATAAAATTATAAAAATCAATAAAAGAATGAAAACATCAAAAGAGAAAGATACGCTAAATAATATATGAAATTGAATAATTAGATGGAGATGTATTATATACGTAAAGAAATACAGACAAAAATTCTTGGGTTGGTATAGTAATTGGTACACACTTGAAAAAGGGATCGTGAATAGTAACTAGAAGATGAGAGGCATATATGTATATATGTATATGTATATGTGGCATAGCTTTTTTGCTTTTTGATGTAGTCAAGGGTCTCTCAAAAGAAAGGACTAGCTTAGCTTGAAGAATTGAAGACCTTCAATAACTTTGAGCTTTATTAATTAATTAGCTAAGAAAAAACCAACCAGGTTTTCTCTCTTTTCTCTTTCCGTATTCTTCTTCTTCTTCTTCTTCTTGATCTCAAAATTCAATTAACTAGAAGCAAAGTGTTAAAGCAACTACTACTCTATCTATATCTGTAAGTGTGCTTCCTTCTCCTTTTTCTTTTATTATTATATTCATATTCATCACTTCCTTCTATCTATTCTCTCCTAATTTCCATGCTTAAAGGCTTAAAGCACAGGTTTATAGCCTCATACTTTTCCTCTTTTTTTTAGTTTCTTCTTTCTTCTCAATATTTTCCCTTTATTTTCTCCGGTTTTTCCTGCATATTCTCCTCTTTCTTTTGCAGATATTTTCTTTTCGAGTTCATGTTTTCATTCATCTTCTTTTGTCCATATTCAAGGTTCTTTGTTTTTTTATTTTTTCAAAATTTTATTTTTAATTTTAATTTTTCTCTTCCTTTTATTATTGCTTTATTTATTTATGTTCATGTCCAGTTTTTTCAATCGGATAATAATCATTCTCCTCATGCATATCTTGATATAATATATATAGTTCATATATATATATATCAACTTGTTATTTTAAAATAAAAAAAAAAAATTCCAGTTCTTATTTTATTTTATTTCTTGGTNNNNNNNNNNNNNNNNNNNNNNNNNNNNNNNNNNNNNNNNNNNNNNNNNNNNNNNNNNNNNNNNNNNNNNNNNNNNNNNNNNNNNNNNNNNNNNNNNNNNNNNNNNNNNNNNNNNNNNNNNNNNNNNNNNNNNNNNNNNNNNNNNNNNNNNNNNNNNNNNNNNNNNNNNNNNNNNNNNNNNNNNNNNNNNNNNNNNNNNNNNNNNNNNNNNNNNNNNNNNNNNNNNNNNNNNNNNNNNNNNNNNNNNNNNNNNNNNNNNNNNNNNNNNNNNNNNNNNNNNNNNNNNNNNNNNNNNNNNNNNNNNNNNNNNNNNNNNNNNNNNNNNNNNNNNNNNNNNNNNAATAGAGAGAGAAAAAATGGGGTTAGATTTTCTTGAGTGCTCTCTCTCTCTCTCTCTCTCTCTCATCTCTCTCTCACACACACACACTCTCTTTCTCACACACACACAGAGACAGCGACTTGAACTGCTGCTTCTTCTAACAACTACTAAGGTAGGTTGGTTCCTCTCTCCCTCTCTCTATAACTGCAATTTATTATATATATATATATATTTTATTTTTATTTAATTTTAATTTTTTCATATATTGATCTGAATGGTTATAATTCTCAATATTCTTTTTTGCAGCAACAAGATATTATTGAAGAAACAAGGACACATGCTCATTTGTGGTGGAGAACTGTTCCATAGAAAAAGAGATCTTCAAATAATTGAAGAAGCTATATCAACTTTTTCGTCTTCCTTCTTCTTCTTCTCGTGTTTTTATATCAACAACAATAACAGTGTAAATTGCGAGTCGCCGCC

## >AdNAC30

AACTTGTATTAATTGTCTTGAAAATATGAAAGAGAAAAAATAAAGTGATTTGATGGCTTTGATGGAAGTCTATACATTGCTACACAAAGCACGGAGAATTGCACTTAATTAATTTATCTTTGATGATATTCATAGCTTTGCTTTTCCTTTCTTTTTCTCTACTCTTTTTTTCTCTCTCTCTCTCCGTCATTTTTCATCAGGAAACATGGAAGCATTAGCAAACTACCAACAAGAAAAAGAAGGAGCTAGTAAAAAGACCATTACCATTATGGCAAGAAAACATAAAAAATATAGTTGTTGAGATTCTTATCACTTATGGTAAGATATGGTGATAAGATCTTGACTTCTCCATACCAAAAATGGTTGAAGAAAGATTTCGGCCAGCAAGGAGAAGATCTTTGGAATGGCTTGTCTCTGCTTTTGGGTTCACTCATCACAGAAGGTAGCTAGAGTGGCTATGTGAGGGAGGAAGCAAAAGTGGAGCAGATGAAGCTATCATCATCATCATGAAGCATCAAGGGGCAGAAATTCATCTTGGAGAGCAAGCCAAGGATGGAGCGCTCAGATTGATGAAGATTGATGACTAAGGAATGACTAGAGATAATTGCATGTTGGATTTTGTATAGGTTATCTATTCTCTCTCTCTGGCCGAACCGGTTTGCATGGAGAAGAAGAAGTTGGCTTGGTTTTTGGTTTCAACTATGGAGGCTTCCCCCTTCTATAAAAATGGAGAATAGCCACGGCTTGAAGCAAGGAGAAAGTGAGAGTGCACGGCACAGAGTTCTTAGAGCTACTTGAGCTAACAGTTTTTCTTCTCCTTCAATGTATTCTGTTTAGTATTTTTCTATTTAATTTTGTCATGTCTTGAGTCTCATAAAAAAAGGCAATCAGTGAGGTTTGTATGAAAAAGTCATAGAGCGAAAAAAGGCAGAGAGTGCAAAATTAAAAGAAAAAGCCATAGATATCCTTAGAGTTCCTTTATTCATCTATGTTGTGTTTCATGATAAATAAAATCAAACCAAATATTTGATTATAAATATAACTTTTATATTAAAAGAATGATAATTTAATCACAATTAAGTAATTAAAATAAATTAACTATTAATTTATTAGAAAACCATCTCAATGGAAATAAATACATCGCATGCTTAAAAATAATTTGAATTAATCCTATTAATTTACAAACTTTAAGAAAATAAGAATAAAAATTTAAACACAAAAAAGCCAAAGCTCTGATACTACTGAAAGATTACCATGTGTTCATAACACGTAGCGGAAGAAAAGAAAGTAATCCTTAAAAATCTATTTTTGTGCTTAAATTTTATTCCAATAATATATAGATCAGATCAAAATACCTTTAGACGTTTTAGTAAAAATCACTTTATCCTTTGATGGTACGAAGGCGCTATGCGTATCCACACCGAGACCAACCTTTACTGTCAACTCCTTGACTAATGGACATCTGATTTAAATTGATAAACTTCTTGCAACTCTTTACATGCCATAAATTTCTTCTCCAAGAATCAGGCTCACATACGTTTATGTGTGTAGAGGTAAAGGAATAAAAACTCTTGTTATTCTCTCGTTTCTGTGATTCCTCTACTCTTGCATACACATATATATAGTATGAGATTTGTTAATGATTTTAAATTTGAATCTCAATTCAAATTTGAATCATATCTTAAAATTCTAAATCTGAATCCTTCAAATTTATGAATCATATCATAACTCAATTTGAAACAGAATGAGTTAGGATCTCGTCCAAATTTAGAATTCAAATTTTGAAATAGAATAACTAATTATTCTTAAATATCATTTAATATTCTCAATTATCATATTATTATTATATTCTTGGTGCTAGCAAAAAATATAATAATATTCCATTTGAATTAATATAATTATTTGTTTGATCAAATCAAAATAATAATTAAATAATTCTACAGCAAAGATTAGAACACTCGTTAGTGTGTGACTCCATAGGTTCAATACTAAGCGAGTAGTAAATTAGTCATACTAAATTTACTAATCAAGGTTGCGTCTAGCAACGCTCCTCAACGACCCGATAGTATGAAGTAATATTTTTTACTAAGAACCTTAGAAGAACAAAGTATAATTCCTTCCATCTTTCCAGCTCTTGGTTAATCCTTAGAGTATGGTTTAATTGTCAAACTCTAACATGTTACCATTATTATAATGAACTGTGAATGACTTAAGAAACTCATTTATTCATTCATTCAATTTCCTTGGCCAAGGTTTCATTCATCTCAGTCATTATAATCATAGAACTCAAACTCTTTACTGACAGTTGACGGATTCCTTATTGACTAATTATTAATTCTACAAGTATTTAAATCATACACAATGTCCATTCAACTAGCACCCTAGGGTATTAGGTGTCCGAAATCAAAGTATAATAAATACATTGTTAATTACTATGACAGTCGCAGGTCAAAGAAAACTCTATTACTATGTTCATCTTGAGAATATCCTATTGACAAAT

## >AdNAC31

TTCTCATTGTTTCTCTATACTTTTTCAAGTTCAATTTTAAATATAATTAAAGTTATCATATTATAAATTCTCAACAATCTTAAAAGGGTTAAATTCATCAAATTCAATAATTTATCTCAAATATTTATTGGTGTATATTTTTACAGCAGATATAGGAATAAGACGAGTATTAGGGGTACACATGGGCCGGGTGAAGCCGGGTTTGATGGGATCTAGATCCGACCCAAGATATATACCGGGTCTATTTATTAGACTTGAACCCGACCCTAGACCCAATAAAACCAATACACTTTCGGGCCACAATTATACCGGGTGAAACCGGGCCGTTAACATTACATTACGTTGATACCTTCTTGTAAGCTAACATGTAAAAATATCCAAATTTCCAAGACTCCAACCATTATTTAACATGGTAAAATTCACTTAGAAAAATATAACAAGAACTAACCCTTCCTTAAAATTAAAGTATAACCACAATCAATACTAAAGCAAAACCACAATCAATACTAATATCGTCTAATAATACAAAATATTTAAATCAATACAAATAACACAATATTATGCATTAGTCTAAAGTCTTATGTATTCTAAACATAAAACATTAACTTATAGTCTTATAATGACTAATAACACAAAATATTAAGGTTTACAATACTTAAATTTCACATAAGAATAGTCATGATCCATTACTAATAACACAAAATATTAATTGTGTATGATGACCGGATCTATTTTTGAGACCCTTACCCGATCCTAAACCCGATGAAATCACACCAAATTAGTCTCTAAAGTATTTGAGACCAAACCGAACCTTCGAACCGAGTCGGGTCTGTGCACCCCTAACGAGTATAAACTCGGAACAACCAGAATCTAGCTACCCCATATCTAAATTATCACAAGATATTATCCTCACTTGCTCTAAATCCCAGTCAACCATTAAAAAACTCATCCCATTCGGAGCGAAACATGTCGGATCAACTCAAATTGTTATCCCTATTAGTTGGTTAAAAAAAAAACACTTATTCACTGGCTTATCATTTCTCTTCTCAATATCCCCCTTTGCGTTTCCAATTTCATTTATCATTTTGTGTATTATTAGGATCAATTGTCACTTATGTCAATTTAAGTAAAACTATACCACTTATAACTTTTTGTAGGATGTATACATATTTTAGCGATCAAGTTATTAATAAATTACAATAATAACATATAATGAAGATTATTTATATCAAATTTTATTAAAATTGGCCATCAACATAATTGATTTTTTTAAATTACACAAATAAAAATTATTAAAATAAGATAACATTTACATAGATTGACACCCTGTCTGTTTTTCTAGCTAACTCTCAAATAATATACATCGCATTCTTCTATACGTGAATCTCTCCCATTGTTTCCCTTATTTATTTATTTATTTATTTTTTTGAGAGGACGGGGTAACAAGAATTCCTTCCATCTTAATACACAAACGCAGCAGCGGCTAATGGATCGATCAAAATAATAAAAATGTAGGGACACTAAACTTCCAATTTAGGCAAAGTTCATTATTTTATGGAATCTTTGTGGCTTATATAAATTTCTTTTATTTTTTTATTTTTATCAAACAGATAGAAGACACCTCATAGGAGAATGAACTTAAGAAGTACGGATATTAATCATCCTTAACTAATGAACATAATATATTCTTTTTCAATATAATAAATAATATTATTTAAATATAATATATAATTGTAATTTTGTTTTGTGTTGACATGCATATTATCATTATAATAAAATACAATATAAAATCCATTTATATTTGGATACATTCTATATATTTATCTAATAATAAAAATACAAAAAAAGTATCTATACATTAAAAATTAGTCATCAATTTAATCATCGATATAAAATATATATTAAAATATAAAATAAATATTAAAAATAAATTAAATAATATATATTTATATCTAAATACATAATAGCACATTTGGTGTCTGATATTTTATGTACATATAATATTTTGTGAGTATATTTAAGTAACAGAATTTTGCTATAATATTATATTACACATATAAGTTTTGTGTTTGCTTGAGATAGTGGCTGCTGGGTGGTGCATATCAGCAAATGGTTGACACAAGGTAGAGTTTGCTTTTGCCATATGGCAACAGATATAGTATGCAGCAGCCATCATTGATGTTGAGAAGAATAGAGCGCTTATTAGCTTCCTTGCTATGCATGAGAAGTCTACGTGAAGGAATGAAGTTATGGTTGGCACAAAAATCATATAAAATATATATATGTAGTTTTCAATTATTTTTCTTCTACTTGTTATGATTTTTATCATATACATTTTCATTTATTTTATAGCTTTATGTACACTCCAGGGTGACATTGGACCTAAGTTGATCTTACTCTTTGTGCTTAGACATTAGGTTGCCCCATTCCCACTCAACTCTTCCATCCCTTATTTAATAGTCTCTTTCTAAGTTAAAACTATGGTCTTAATTAATAAGAT

## >AdNAC32

ATCAATTCATCTTTATGAACAAAAAATTTGTTATTTTTTTAAAAGAACTAATTTAGAATATTACTCCTTGTTTGGACATTGTTTGACATCACCAAACACGTATAATATCTCCTATGCATGATATGCAGTTCAACAACTTATAAACATATCATGCATAAAAAATATTATGTTTTTTTTATATCAATATATCCATCAAGTCAGAAATTTAATGACTAATCTCTCATATACTACAGAGATAAAATGGATGACTTTCTCAAATAAGTAAGTTTTATTCTTATTCTCCAATGAATATAGATTCTAGAAAAGATGATTAAAAGATACAAACTTTTATCTATCTATGCCAACTTATTTAATGATGCCAAACAAAGCCCAAACAAGTTCAACGAGACCCATTAGTTTAGTTACGGCAATGGATATATAACCGGCCTCGTATTGCACAAAAAAATGGTCTAGCAGAAAAACCCATCTCCCACGGATACAGAAGTTCAAGACCAAAAAAAAATTATCCGCTATGGATTACTGAGGACAAACCAAAGATAGTTCCCAACAACAAAAAAATCTGACGATGGCTTACTTTTATTGAAATGAAAATTGATTTTTTTTAATTATAAAGAAAAAAGAACTAAAAAAAATAAACACACATTAAAATTACAAATAATTTTTTTATTAAAAATATAAAATATTTAAAATTTTCTATTAATAATAAATTTATAATATTTTTTATTTTTAGAATATACGTTAGCTAAATCCTATATATATTTAATTAGCAATTATGGCCTTTTTTATATTGTATGAGTTCCTCATTCATATCACAAATGAAATAAAGAAAAGAACAACATATGTTCACCGCTAGTTTATAACTTTCGGTGATTAATATTAATGGACATTGAAAATTGTATTAATTGACCAAAAGAAAACAATTGTATTAATTAATTGACTTTCTAAGTAGAAACCAAAAATTCAATCACTCAAAATATGCTTCATTTAGTAGGAACCATAGATATACAAGCATGTTAACTGCACCTGCAATCAAATTGAAACGACTAAGTTAAGATATTCTAATAGTTATTCCTAAATTCTACTTGTTTTTGCTTATGATGCAAGTAGATGCTAACCTTTACTTTGTTTAAGGTATCTTTCTTTTCCTTGTCCAGAGATTAGAGCTTTTGTGTGCATGGAAAGTTACTTTTGTTACGTAAAAAGTCTGTATTACTAATCAGTAAAGAGAAATGCATTATTATTAAAAAAATATTCCCGACCAAGTGGTCATCCACACATGTACTCTTGAACACAATACAGTGCTATAAATTAAACATCATAATGAGCAACATTTTAAGCTAAAATTGTGTATTTATCAGGCGGTAAGATGATAACGTATTTATGAAAACTGAAAACAAGAAAAAAAGACAAATATTATACTAGTTAAGTTATGAAATAGATGATCGTATTTTTTTTACTATTAAATTATTAACATGTTATCTTATTGTTCAAAATTTGACCAAAATCTGATATATTTGAAATTCTATCAAAAAACATGAAGAAATTTTTAAGGTGATGTTAATCAATTTACAAAAAGACAATAAATTCAATCTTTTTGAAAGTCAAAATTTTTTATTTGGTTATTTTTTTTTTAAATACAAACATGATTTTGAATTTTAATTTTGTTCACCAAAAACAATAAATAGTGGTAATTTTTGTGTTTGCATTCAATTATTAGATTGAGAAGTAATTGAAGAATTTTTTTTATGCTAATTTTGCTATTTAATTTCATGGGTAACAAGATATCAGTAACAATTATTAGAATTTTTGTATTTTTTATGGTCGGTACTTTACAAACAATTATTCTTTTTATATTATCATTTTTAGTACTTTTTTTAAGTTTTGATTTTTATTTTATCAATTTTTCATTTTTTTTTGTTCATAGTATCATGTTTTTGTTTAAAGCTTCTTCTTTAACGTTCTCTAATGCATATAGTTGTATTATTTTAATAGAATTTTTAATTAGTTTTATTAACATATATATTTTTTATGAAATTTTATTATTTTTTGTTTGACTTTGTATCATTTTTATTATATATTTTTAAATTAGTATGTATTTTATTTATTATTATTATTTTTTATAATATAAATTATTAATTTTATTAAAAATGTCAAATTAAATAAAAAAGAGTACTAATAAAGAACAATAATAAAAAATTATGATAAAAAATACTAAATTTTAATACATAAANATATTTTAAATTATATAAATTTATATTTTAATTTTTATTTAAACATAATTTTAAATAATATAATCTAAATAATATTTATTTTATTATAATTTATTTTAATACAAAATTATTAAACATAAATTACATTAACATTAATTCACTTCTTATTAAAATCAAATCTATAAAATTAATTTTATATAAATTTTTTGTGTGTAAACATTAATACAAACAAATACTTAATGGATTAAGACAAGAAAATAAAAAATGAGATGAAGCAGCTAAGGAAACGG

## >AdNAC33

ATCTCCGGTAACTATGAACGGAAAACGCTGAGCTGTCACGGTAAGCGTGTATTAGCTTGCTGATGTGGATGAATGCTGCAGACGTGGACACCTCAAATGGTCAGAGGGTTAATTGTCCCCATCCACGTCAACCAAAACAAACTCTCTCTTCCATCTTCGCTCAACTGCTATGCTGGAATGGAAGAACCGCTACCACCTCCCACTTCCGCCATCCCCAACCAACCTCCTGCATGGGATCCAATTGCTGTTGTCATTTTTTTTTATCACCAATGGTGTTGCAATGAGTAGCACTAGGAATACCTCAAACCTGAATTGGCTCAGCTCCATTGTCACTACGTTTGTTATTGTGTTTATCATCGTAGTTGAATTTGTTCACGGTAAAGTCTCAAACCTGACGTCATTCTTCCCTATGGGGATAAAGGGAGTGTTCAATGCTGCTGTGGTTGTGTATTGGTCTTATACAGGCTTCGACATGGTGGCAACTATGGCTGAGGATGGTGGAGGTTGGTTAGAGATGGCGGAGGTGGGCGGTGGTGGCGGTTTCTCCATTCCAGCATAGCAGTTGAGCGAAGATGGAAGAGGGAGGTCGTTTTGGTTGATGTGAATGGGGACAATTAATCCCCTGACCATTTGAGGTGGTTGGGGATAATTAACCCCCTGATATTTGAGATGTCCACGTCTGGAACATTCATCCACATCAGCAAGCTAACACACGCTCACCGTGACAGCTCAGCGTTTTCCGTTCATAGTTACCAGAGATTCACCCGCAAGGATCGTCTTGTGTCACGAAAGTATGGGACATAGATCGAAGTGAATTGCTTTATTCGTCAGGGGTTACTTTATCATTCTACGAAATAGACAGGGGTCGAGTTGGTAGTCCACTCTTTAAATAGATATATGTTAAAAGATATATTGAGGTTAAAAGGAAAAAAAAAACTTTAAAAAGATTAAAATTTTTAAATTCTTAATAAATTTAATCTGAATTAAAAATGATAAAGTGCAAAAAATTTTAGTCCAATACTATTATTATAATACAAGAGTAATATAATTTAATATTCTAATTGATATAATAAAATATAATTTTTTTATTAGTATTTATATTTTTGTATGTTTATTTATTATTTTATTGTAATTTAATTATTTTAATTAAACTACAATAAATTAATTAAATTACTAAACTAATAAATAAATGGTTGCAATAGTTCGATCACATATTTATTTTGATTAATTCTATGATATTTATAAATTGTTGTATAAATTTTATCTAACTAATTTTATAATATTTATAAATTGTTACATAAATTTTGTCTAACTAATTTTTTATAATAAATTTTTATTTTTTTAAAATAAATATTTTTATATTTTTTAAATTAAATATTAATTTTTAATTCTTTTTTAATAAATTAGACCTCAACTCTTTTTTAATCAATTAGACCTCATTTTTAGGCACAAATTCACCCTTCAGTTTTGCTATGTTCACTAGAAAAGAAGCTGCTACTGATAAAATTCCTTTGCCTTTTGGGCTGGTGGTCAGGTCACTTCCAGGCTCCAGCTGGAACCCATGACATAAGCTGACTTCACCAATTGCCTCACTTTCAATTTAACAACACCATTTAATTATCTATTTTTTAATANNNNNNNNNNNNNNNNNNNNNNNNNNNNNNNNNNNNNNNNNNNNNNNNNNNNNNNNNNNNNNNNNNNNNNNNNNNNNNNNNNNNNNNNNNNNNNNNNNNNNNNNNNNNNNNNNNNNNNNNNNNNNNNNNNNNNNNNNNNNNNNNNNNNNNNNNNNNNNNNNNNNNNNNNNNNNNNNNNNNNNNNNNNNNNNNNNNNNNNNNNNNNNNNNNNNNNNNNNNNNNNNNNNNNNCTCAGCTCTGAAATAACCAACATAATCCACAAGAAACAATGAAATAAATACAGGCGAATTGAAAACTGACATAAAATTCCACCCCTCTTTTATTTATTTTTTATATATATATATTCTTTGGGTTCTGTCTGTGTTGACTGTTTAGAAAGAACGAGCGGTGTTGTTTGGATAAAATCGAAACTGATTGGTGCTGGCATCTTGGGTTGGAACTAACTAACCAGGCTTGTTTCGTTGTGCAATAACCAATAAGTGAATAGTTTTCGCAAAAGCCAAATGAATAATATAAACCATGGTTAATATAATGTCAAACCAAAAGGGAGGTTGAGTCTGGTTTACGCCAAATGTGTCGTCTTATTCTTCCCCCTCCTTCCTTGCTTGCTTGCTTCTTGTCAGACCCATTACTATAAAAGAAGGCACACTTCACCCGTTTCCATGCCATTTTGCTTTCTTTATCCTTTCCTTCCAATCTAATAATAATAATAATAATAATAATAATAATAATAATAATAGATTAAAATTATTTTTAATATGAAGATGGTTTTTTTGTTTTAATTTTTCTTTTCTGTCTTTCTCTCTTCCTTTTCCTTGCCCTCCTTTTGTGCTTTTGTTATATTCTTCCTCTTTTCTTCTTTTGCT

## >AdNAC34

TAACAACAGAAACAATTCAGAACTAAAAAATTAACAACCAGAATTAACAAAGAATAAAAAAAAATTAACATAAATAACTCAGAATCATAATGATAGCATCAACAACTTAAAATTAAAAAAAATATTAGCATAAATAATGCAATGAAGGATTGCACCAAAACACTATCAACCGTTCAAAATCAGAAAAGTTAGCAATCAACAATTTAAAAATTAGAATAAATTAAATTAACAAAATACCTAAAACTAAATTAAGACAACAGTGTTTATCGGTGTCGAAGAAGAACGGAAAATGACAGCGCAATGAATACTTTACCGCTGAATAGAGAGAGACAGACACCAAAAAATCAAGCAGAGATGATGACGGAATAGCCTTTCGTAACGATGATGCCAACAGTTTCAAACAAATCTTTGTGTGATGTTGAGGAGAAGTGAGCAGACCTTCCAAAACAATGACAACATCCTCTACCATAGAAGAAGAGTTCGACGGTGAATTTGAATTTGTTGGCTTAACTATTATTGTCAAAAGTTTAAAGTTGGAACGTTGGGTTTGTTGGTCTGAAAGAAAGAGAAAGTATTTTTTATTTTTAAAAAAATAAAATAATACTATTTTAGAAAGGGATTTTTTTTAACTAAAATCGCTTCGAATTGGTTTTAACAATTCATCTATTAGTTGTTAATTTGATCGATTCTTTTATTAATTTTTTGTAATACATACAATTTTTCACATTAATCGAATCAATAAAAAAATAATTTTTAATTTTAATCGAATCGACTAATCTGATTCGATTTTTAGACATTAGTTCTTAATATATTTGTTGGTCGTAACTTTGCCAGGAGGGTTGCTTTTAAATTGTCTGCATAACAAATGCATTATGCATGACATCAAAATTCTCCTATCAATTGGCAGTTAGCTCCCTTCTCATAAGTCCCATTTTTCTAAAGAAAGAATTGAATGCTTTAGAATCATACTTAATATATATTCTAAAAAAAAAGTATAGAAAGACAATTAATTTAGTATATAATGTATATAGTAAAGATAAAATTAAAATTATAATTAGTTAATAATTAATTTAATTAATTTTTAATAATTTTAAATTTTAAATTTAAAAACATAAGAATTTAGTTATGAAAAAGTTATGTATTCTTTTTTTTTACGTGTGTGGAAAACGTGCCGTAGTTTTTTCTTCTCCAGCAGTCTCTTCCACTCTTTTCGAATTGACGCTGCGAATATCACTACTATTCGCCGTCTAAAATGCAATCCGATGCCATCCAGCTGTCCAAAAGCGTTTGATGAATACACCCCTTCATGCACGTTGCGTCCATCGCGCAGTTCAAAACCGCTATGGCCGCCGCCACCTTCAACGACGTGGCGGCTGAATCCTCCTAGCCCTCGCTGTAGCACTCGCCGGAAAGGTCAATGGCAGCGGCGGCCACAAGAGCCCCCTGTGTCCGTGTGGTTTTTTTGTTTGGCTTGGCGTTCGTCATCTTCATGATGACGGTTATCCAACCGGCGATAAAGCTGGTGGCGCGGGGGTGCTCGTGAAAGCATGATACGATGGACGAGATGTATATCTGCTTAACCCTCGCCGGAGTGATGGTATCGGGATTCGTGACGGATTTGATCGGGATCCACTCGATCTTTAGCGCATTCGTGTTTGGATTGACGATTCCGAAGGGAGGGGATTTCGTGGAGAGGCTGATAGAGAGGATCGAGGATTTTGTGTCCGAGTTGTTGATGTTGCTGTACTTCGCTTCGAGTGGGTTGAAGACTGACGACTTTTGATGCTTGATGTTCAATTTACTAGGTATGTAGATGATTATTTTAATTCTTAATTGGATGGTTATTTTGTTTGATTCAGTTTAAGTATATACAATTAACAAATGCTAGATAGTCATTTCACTAGGTAGTCAGATGATTATTTTAATAACTACGTAGACAGTTGTTTGATAGCCAGTGAAAGAGCTTCTAACACCGGAGAGGTGGGATGATTGATGAGGATGGGATAGTAGATGATTGGGAGAAGGGGAGAAAAAAGGTATTTGGGTAAATTCCATCGGTAAATTAAGGTTGAGATGGTTAATTTTTTGAAATAACTGTTTGAAAATTTTTTTTCCTTGTATTTGGTCAAATTTAAAATCCATTGTACATATTGTCAAAATTTCTTATTGTCTTTTTAACGAAATTCATTCTAAAATTCATTTATAAACATATTTATTTAAAATATTTTGAAGTGTAGATACTCGTATAAAATATTTTTTTATAAAAATAATAATTAAAAATTAATTGTTAAATCAACCATTACATATAATTAAATTGATAATTGATCTTTTATACGTGGATGAGAGAATTTATTTACCGAAACAAAATTACTAAATTCCCATAGACACCTAAGCATTGGTGGTGTAGTAATAGGATGGATTTGATTGGGCACACGTGTGACAGCGGGTATTTTGCTGGATTTGCACGTGTGAGGAAGGCACCTCCCTTGGTCCGTTGGTACATTTATCTGTACCACAAAGTCCCATATACACTGATGAATGAATACTCATCTATCTGATCCTACCCCACCCACGCATCACGC

## >AdNAC35

GGATTGACTCACGATGATCGGAGATTATACCCACGCGTCTCTTCTTACAACATGTGTTCATAAATTACTGAGAAAAAAGTATCACGCATCAACAGTCTCACCCTCTACAATGGCAAAATCAATCTGTACAATGTTTTGATTCCCATCTTGCGCAATTGTAACCAAAAGACAACCTTTATATTTTCCGTATAAGTGGGTCCCGTAAACCTGAACAAGCAGCTTGCAATATTTGAAAGCTCTTATGCATGGATTGAAACTCCAGAATACCCGATGAAGTATCCTAACACCATCCAGGTCTTGACTCATGTTATACAGAGGTTGTGTTTCTATTTGGACTTGTGAACCAGGCATCTTCTGAACCATTGCGAAGAACCACAATGGTAAAGCTTGGTAAGATTATTCCCATCCACCGAAAGCCAAGCCTTCTGGTAACTAATAGTGTAGTTGAATCTTGCCTGGACTTTCGCAATTATAGATTTGACCTTTATCGATGGGCCGGATTCAACCAATGGCTTCATAGCCTCAGCAATCGTGTCCAAGTCTAACTTGGAGTGATCCCATGAGATCGTTCCCATAGAACACGTGTGCCTCCCATTGTATCTCTGAATCTTCCAACAGGCTTTTTTCTGTATCAAGCTGGCTCGGATAAGCCAGTCGCACCCACTTTCATAAGTCTTGCACTTTGCATATGTGGCTCGGATTGATAAACAACGTAATCAACTCCTCTGGAGATAGTGAAACTCCGAATTGCCGCAATGACTGATTTTCTTGAACCGTATTCCATTCTAATTCTAAACTCTCCATCCTCAGGATCAGCAACACCTATAAGAAAACAAATTTGTCACTCATCGATCCGTTAAAACAAAATCAAGTAAACATTCTACCCACTGCTCACATACCTATGTTTGCATATTCGGAAAACTCTGGTGTATGCATGGCTTTAAGATCCAAGCTACACATAAAAGGTGAAACGTCCATCGGTTGACTAACTGCGGCTGGAATCACTAAAGTTTTCGCCACTGCCTCACCTCTCCCATCGTCATCTTCATCCTCATCACCGGCTTCGTACGTAACTTCAAACTCCTCATCGCTATCATCGTTCATTCCCAAGTACACCTCAGCTCTGTCATCTTGCACATCTATGTTATATTGTACCTCATCTGCAGCAATATGCTCAAACTCAACGTATAACTTAACCTTCGGGTGTTGCACCTGAGTTTGTTGGTGAATATGAAACATCCGCTGAATACTAATTTTGTCGACGATTGGCATAACCTCAAACTGTATTAGGCCGCCAAATACGACAACTGGACTCTTATAGAGAATGTTGGTCACCCTCTTTAAAATATCAGCCTCTAGCTTTGACAGAGCCCAAACTGTAGTTCCGCAAATGTTATTTTGCACAGAACAACAAACGAAAACATATTCTCACATGCAAAACTCACATCCTCATACGTATTTCGTACGACTTCACCATTGCGATAAATCACAATATTTGTAGTGCCCTCTATCGTATCGATCATGGAGGAAAATACCTCTCTCACAATGAAGCAAAATGGTTACGAGTTTTGATTTTTATAGACAAAAGACTTAATTCGCAGACTACGTGTTGCAACGCCATCCACTAACGTCTGCCACATGTAAAACACGTTACCTACGTGTTGAAGAGATGAGCACCACATAGCAACATGTTATCTGTATGTTGCATGCAATTCTTGACACGTGTCCAATATATTAGTTGTGTGCTGAATCATGATGTTATCTACCTGTTGTATTCGTATATTTACACTACTTGTATATACACCAAAAATCTCTATTTTCAAGTAAAGCTAACCTCTAAATTCCATATCAAAATTCTTTAGCGTTTGGAAGGAATATTTGTATTTCAATTTCATGGCAATTTACCTAGGCGAATGGCGAACAATGAGACCCCTCGCACAAGAAACAGAAATTAATAAAAAAGAAAAAGGAAAAACCCCAATTAAAATCCACATATATAAACAAAACCCTGGCCGCTTCTCACTCTCTTCTTAGTCCAAAGATTCAACGATTAGGAGTGCGAAGTTTCAAGTCTGTGTTCTTCTCACTTCCCTCTTTCCTTCCCTGGTAAGCTTCCTTTCTTCCCAAAACCCCAATCTTCGCGATACAATAACACCTTCCATCTCTTCGTCGTCTTTTTTTTTTCTTCTCTGTTCTGGGTAAGCTCCGTCGAATAATTGTAATCGATGAATTTGCAATGTGGCTGTTGGTGTTGTTATTAGGGTTCCACTGTGCCGATGAATTTTGTTCTCAGAAAGGGACCGTCTTCATCCATTGTGGGTTTCCAGTTTCATTTTACCTTCATGAACCGGGGAAGGCTTCGCTGTTCTCGCAATTTCTTTCGCCACTGGACTTGACGCTTCGCACCCTCGATTCTTTTAGGGTTATTTAATTCCGTTCATCATTCTCTAGTTAAATTTCTCGTCTTTTTTATTTTTTATTTTTATTTTTATTTTTAATT

## >AdNAC36

AAGATAAGGATAAGAATATAAGAGAGGATTAAGGTTGGAAAATAAATGGAGTGTGACACAAGGCAGAAAAAAATTGCTGATAAAGTTGTAGATGTGTGTATAACTGTATATATATAAGCCACTTGTGTTTAAATAGACCAAACTAGCAATACCGTTTCCTATGCACAATGTGAACCCAAAATTCCGTTGGATTAATTAAAATGTAAAAAGAAAGAAAGGGAGAATGAGAATCACATCAAAACTCAAAATAGAAAAACAAAGGCTCACAGCATTGGCAAGAGGAACACAATTATTTTACTCCTTTTGCCTTTTTTTCACATCTTAAAAAGGAATAGTCCCACTTGATTGCTGCTTTGTCTAATATAACTTGTTAGCCACATCAGCATAGAAAATAGACCAATGTATAAAATATAAATGAAACCAAGAAGTTTTTAAATGGTCCAATAATGAGAAAATTAACTTTTATTCTGCCCCTGGATGGATGGGTCGCTCTGCTTTTCTTTGTTTTTTTTTTTCCATGTCACAATGTTGAAAAAGCAAAAGCTTTTGTTCATGTGTCTTTGATTTCTTCACTAATAAATGTAAAGAACAAAATGCTTAACCTATAATTTTGTAATGTGATTGTATATAGTTTAGTTTTTTCTTTTCTTTTCTCTTCTTTTCTTTGTTTTTAGAAAGAAAGTAGATATTACAAATATTAACCATGGACCATTATTCATGCAAGATTAATATAGAAAGAGCCCAAAAGAAAACTAAAAGGTCTTGCATATTTAAGAAAGAAAAAATGTCACAAATATTCTCATTCTCAATCCCAAACTTGTAACTCTGCATTTTTCCTCCATTCAACCTCAAATGTTATAAAATCCAATGCAAATACCCATGATAGTATGATACCTTTTACAAAAAATTCACAAGGAGTAGGGTCAGATTTTATTTATTTATTTAATTTATTTTAGTATTTTTTTAAAATTAAAAAAAAAAAAGTTGATTGTGGAGTGGTAAGAATATGGCAGAAAGGACAAAGTTGAAAGCATAATAAAACTGACGTCAAAGCAAGCTTTAATTTTTAACTTTTCTTTGTGCTTTCTTGGTCATACACTCATATCTACGCAAGACAATTCCACTAATTACCGCGTATTTTGCTTCAGCATCCACTCCATAGTCCCTACAAGTTGGTCCCAGCAATGGCAGTTTAAAAAATGGAGCCAGATTGCTTTTAACAATCTACATGTGAATTTTGAAGTGAGAATATAAAAGAGACTCTCTGAAAGTTTTTTAGGAGACTTGGACCCTCACCCTCTTTGATGACACAATCACATGAATTAAAGTGTTCAATCCGACACAATATGATGTGGATTTAACTTGATGTTCAGGGTCTTGGTCGCATCATCCATCCATTTAGGTACCCCTATTCCTGTTTTTTTACATGTTCACACAAAATTGAAAATTCTTTGCTTTCTCAGCAAAATTAAGTGATATGATATGTCATATGTGTTCAACCAAAATTGTCCTGCAAAGTTTGTGCTGAGATTTGGGAATTGAAATGTAGTCAACAGTCACCAATAATTTGAACCGCCAAAAATAAACTAAGAAAACATGTTATGAGTCTATATCAAATGTGAGAAACTATATGAATATATGGACTTATATATATATATGAACAGAACTAGAAAACTAAAACTTTATAGGTGTGAACGGGGCTGATGAAGTGGTTGGTGGGGCCATTTGCTTTGGAATTTTGGGTGCACTTGAGACAAGCAATCTCAATTCTCAAACTTTCGGTGTGTTGTGTGCAATAATCTTTAACTGTTAATTCCTTCTCCCAACTGTTATATATAGCACACCATCTCTGGCTTCTTTTACTCTACTCTACTCTACCTTCTACTCTCCACTCTCTTCCTTATTTCTATCAATTTTTTTCTGTTTCCAACTCAATTTCACATTCTCCATACAAAGTAACTACATTACTATTTAATTTGGTTCATCTTAACTTCTGGGGCTTTCAAAAGCTTCCAACTTTTTTGGAAATCTTTCCTACTCAAATTTTGGACTAGTGCCCATTTTTCTTTTGTGAAAGTTGGTATTTGGGTTACACTGATTTGAACATCTGGTTACTTACTCCTCAGTTTGGTTCATTTTTTCTTAACGGGGATTAATATCAAAGGGGTGTTGGTGGGGGAAGGGTGTGATAGCTTCATAAGAGACCTCAAATTTCTTTTTAAGACCATGATTCTGGCACACAACGGGTGCCTTCTGTTGAATTCTCGCCTTATTCTTCACAAAGAATATATCATAGACAAAAGTTTCTACTTTTGGATCTTTCAGATTTTTAATAGCTATAGTCTTGGTACCTAACAGGAAAATTCTATATCCTTCACCAACATTCTTGAAAATACTATCTTTTTTGTTGTGTTAAATTGAGACCGGAAGAGACTAGAAAACTGCATATACCTTTGGTTCCTTGTTTCTCAATTATACACTTTCCATTCTGTAAGTGAGA

## >AdNAC37

TTTGTTTGTAAAGCATAGTCATAACAAAAATTTCAAGAACTTTGACGATTGTTTTTGGTATTTTTATTGTAGATGAAATTAAATGGCAAAATTAGTAAAAAGTCAATTAATTTTTTAATTTATGAATTGAACGTAGAAACTAAATCCAAAAGTTAGAATTTATTGCTTCTCGTGAACCAGAGTTCAAATCCAGAATGATGTTCACCTTTAGAAGAAAAAAACTAGCCAAACAAAAATTACAACATTCAAAAAGATTGAACTTACTTTCTATACTTCCAACGTGTTTATTAAACATATCCTAAGTCGTACAAAAATAATAGCTGCATTTGTACGACTTATTCCTTATGACGATTACATTTGTATGAATTCGTGCATTTTTTATCATATACACTGATATTCATGATTTTTTTTATAGTGAGTCACTTTTCTCATAATCCGCTATAGGGTGTAGCGGATTATGCTTTGTGCCAGTTTGTTCATAAACTGGTATAGGGCGTAGCGGATTACGTATATTTGGAATCCATGTTAGAGTGTCGCAGATTACATACAATTAGGATTGTTGTATTATTTTTGCAGTTTCGCAATATTGTATTTGTGTAAAGGTTTTTCCAACTTATTTTATTTGTGTGAATTGCCCAAATAGAACGATATTTTTAATTAATTATAGTATTGTTTTTAAGAATAAACAAAATAAGACTTTTTTCTTTTTAGCATGGAAGTAAAAATAGTGATATATCCTAACATTGTAATTTTTGGTATTTTTTAAAATTGTGAAAAATACATAAGTTAGAGGATTCAATTTCTGTACCTCAAATATTTTAATTTTTTCAACACAGTTCAATTTCTGTATCTCTACAAATCAGACAGTCCGATTTTTGTACCTCTAACAAATCCGACGATCCAATTTCTACTTTTCTAATTAAACAATTTCACATTTAAAAATAACACCACATCATTCCACATCTCAAAAAATCATCATTATACCGTAGTTGCCAATGATGGGTTCCACGGAAAATTAAAGAGGACAAAAAGTTTAGCCTCATCTTTTCCATTGCCCATATTTGTGACATTACATACTGAATAGGAAAAGTCTAGGGGCCAGCAGTTTTGTTGAATTTTGGCCAGCATGTAACCAGCAGAAAAATGTGAGTCATTGGATGAAATCTCACACCAATCTCACACCATTAAATCATCATTGATGGTTATTTGCTGGCTACCAATCACAAAAGTTGCTGGCCCCCTAGCATTGCTCTACTGAATATATTAGTTTCCAAATTTCAATAACATTGATCTTATTTTGGATATCATACAAATTATTGAAAGTAGCTGAGAGCTAAAAAAGTGCACTTCAATTTTGTCGAGAAAACAGCACGTCATCATGAACTTGTTCTAGACTTAGCTTGGAGAACACGCAAGAGATGATCAGAAGGTGGCTTAAGCTTTGTGATGGACGAAAAGCATGAAATGCTGGTCCAAGAATTCGAGGTTGACATACAAAAGGTGACAAGTTTTGAATCGATTGGAACTATGTCTCTCACTGTTATGCTGTGTGTGTATGGGATATTTGCCAATCATATTTACACCAGGTAACTTCCAAATCTAGAGTAGCTTTCTCTACTCTCCACCAAATTCAAAACCTGGGATTCATCGGTTTTTGTTTCAAACCACACTCTTATTGCTAACACAAAACTTTAAGATAATTAATGAGCCGATAAAACTTTAGTTACTATTCACATGCAGTTATTTTTGTACGAAGATAGATAATAATTGAGGACTGTCAAATAATTTGACATATTTAATTAAATTACTATCTAATAATTTTTAACTATTAGGATGGTGGGAAACCAATTGTAAGTAAGTGAGCTGTATAATATGTTAAAGCTAAGTGTCATGCAAGGTGTAAATGAAGCTAAGCTAGGCCCCCACGTAGTTTGTGGTTCATATTGGTAGAGTAGTGTTTGGAAGCTGTATAGTAACAAGTAGGAAGAGAAGACAAGGAAGAAGGGACAAAGTTAATGCTAAAACGAGGAAGTTTCAAGGCAATAATAAAAAGTGCGGTTGGGTGGCTTTATTATGTCTTTCTAATAAGACAAGTGACGTACTTGTCCAACCGCAGATACCTATGTACGTGCCTATGTCATCCCTGTGCCACTTTCCTTCTTTCACTTCTTATGATTACCCTACCACCATTATTGGTAACAAGTAATTAGTAACATCATTTGAAGGATCTCAGTCATAAGGTAACGGTATCAACTCTGATAAATTTAGTTCAGAAAAAAGAGCTCCTAGAACAGCTATAGTTTTAGACCCAATATGGGAGTTGGACCTTAACAATTACTTTGCTTTATCTAACTAGGCTATCGTATTGCTCCTATCTATTGTGTATGGTATCCAATTAAATGAGCAACTCTCTATTTAGTTCTTAATCTGATTTGTTTTGGATACAAATATTTTCTCTTTCACATGATGAGTTCTATAAACAACATTGCTGAGAAAGTGA

## >AdNAC38

GTAGGGGAAAAAGAAAAATTAGTCCACCTACACCATTTGATTAGCAACATGCTAGATTATTCATTGAGTTTTTGAGAATCTTCTACGAAATAACTTTAAGTTTCTCATCTTCCTTGCATGTGACATCAAACAAATATTTTCATGAAATCGCATCTCAATTAACATCTTGAAGCCAAAATCATTTTGAATTATTAGGAACTATGGCATGTTCTATGAAGAATAAATATCATAAATATTGGGGATAGGTTGACAAATTTAACCCATTGTTAGTTCTTGTTGTTGTATTTGATCTTCGGTACAAACTAGATTATCTTTGTTGGTGTTTGGAAGATGTTGATGACAAGAAAGTGTCTACTAATATGACTGATTTTGTTAAACTCATATTGGATACCTTATATAAGTTTTATGAAAAGGAGGTTGCAGATGATAAAGAAAAGGAGGATTGTGAAAGTTCATCTAGAGATGTCTTGGATGACAATACTAAAATCTCAGCTAGTATCAAGGATGTTTTTGAAGATAGAGTGAATATGTGAAAAAAAAAACAAAAAGCAAGAAGACTAATGCAGATAGCAAGTCAGATGTGGAGAGATATTTGGCCGAAGATACTGTAAAAAGATGAGAAATTTGATATATTGGCTTGGTGTAAAGTGAATGCTTCAAAATACAAGATTCTTTCTCTTATAGTCGGTGATGTCTTAGGTATTCCAATTTCAACTGTTATTTTTGAATCATGCTTTAGCACTGATGGATATGTGCTTGATGTCTTCCGCAACTCTTTGTCATTATTACTGGCTGAAACTTTAATACGTACTCAAAGTTAGTTGTGCCCTTCTAAACAATAAGTTGGAGATCAAGAATTTAATCAATTTGATTATAGTCAGAAAATTGTTGAAAGTACATTTATAATATTTTTATTATTTGTGACTTATTCAATTCAAGTAACAATCTTTTATATGAAAATGAGTAATTTTATAGTTTTGTAGGTTTTACTAATGCATCCATATCACAAGGAATAAGTTGACGACATTAATAGCAATTGATGGATGATGTCTATTCTTTTGTGGAATTTAAGTATTGTAAACATTAATATTTTGTGTTATAAAGACTATAAGTTAAATTTCTATGTTTAGGAAGTATAATACTTTAGACTAATACATAATATTTGTGTTATTTATATGTGACTTAAATATTTAGTGTTATTAGACAATATTAGTATTGATTGTGGTTATGTTTTAATTTAGAAAATTGTTAGTTCTTGTTATATTTTTTTAAGTGAATCTCACCATGTGAAATAATGGATGGAGATTTGAAAATTTTCACGTACTAGCCTACAAGAAGGTATCAAAGTAATATGTTTTGTCTTAAACTTGTGTTTTCTACGGAATGTGTAATATTAATTATACATTAATTCTTTTATTTTAATAAAAATTGTGCAACTAGAAAAGAGATATAGAGATACAAGAAATTGTGATTCATGAAGAAATTTAAGTTTAATATTAATTATCAACTTCTTCGTAACAACTAACAAGTAAATTTTTATCTCTTTTTATAAATTATTGCTAAAAACCCAATTTTTATTTTATCTAAAAATGTGTATGTTTCATTGAGTCTAAAACTGAATTAGAATATAAAAAATAAATTTAATATGTATTTTAGATCAGATTTGAGTTAGAATAAATCCAATTTTACTTCGTCCATATACACTCTAGTTAGATTATTGATAGCCAAAGTAAAAATTTATCAAATAGTATCCCGGTCAGGGTTCTCTATAGATATTTGAAAAAAAAACCTAACTTAATTTTATTCAAATAAATAACATTAGAAAAGGTAACTAATTAAATATATTATCCTTTTTATAAAAAAATAAAGTAACAATAAAATTATTAGCTGCATATAATTATAAAAGGATTAATCATCTAGTTTTTTTTTATAAGAATTTAGTTACTATTAATTTCAAAGAAAAAATATTTTATAATGATATTAAAATAAAATAAACTATAAAAAATATATCTAAATTATTTTTTTTATTGATAAAAATTTTAAAATATGCCAAGAATATTCAACTATAATTAGAGACATAATTAGAGACATCTCCCTAATAAAAACATTATGTGACTCATTTGTTAATGTTAAAATTTTATTTGTTATATAAAAAATTTTATTTAACATATTATTATTTTTGATTAACATAAAATGTTTCAATTTACAAATATATATTTTTATCACGTTTTAAATAATTAGAGACATTTTCATCAATAACAAAATTTAAAAATATTTTTGTCATTTGAAAAAAATTTAGGTATTTTTTTTTGGTTTATCCTATTAAAATACTAGATGAAATGATGAATATACCAAATCAAAATCGTGATTTGCTTGTGCGAGATGCCACATAAGACCTAATGTTCACACACAAAAGCTCAATCACATACACCCAAGATCACATAAAACCCAATGCTCACCTCAAACACCCTAATCACACCCTTAATCAACCAAACTTGAA

## >AdNAC39

GTTACAGGGTGGACAAATCTACTCATAGTGGCTGTTAGCAGCTATAGATAACAACATAGAGAGCCTGCCGATGATGCGAATCACCCATTGTGGTTATAGGGCATCCGTTTTTAGCGTAGAAGAGATGGAGCTGGTGGATGGTTGGTCACAGACTTCATATCGTGTTCGTCTAACCGAGCGTACATGCGATTGCAGCTTGTTCCAGTCATTGTATTACTCATGTCGACACGCCTTGGCGGCATGTGCAGCTGCGAGTATTGAGTGGGGTCATTTTGTGGACCCCATGTATACGATGGCCTCTATATTCAAGATATATGAGAGGGAGTTTTTGCTGATACCAGACGAAAAGGTGTGGCCTCCATGGTACGGTGCACGGATGAAGCCCAACTCAGTCATGCGAAGGAAGGCATCGGAAAGGCCGGTATCCACTTGGATCTGGAATGAGATGGATGCGATTAAGCGTGCAGAGAAGAGATGTGGGCTCTGCCGCGAAATGGGCTCTGCCGCGAAAAGGGCCACACCAGACATGGGTGTCCCAATGCGTGCTACTCGGATTCATGACGACGTATGCAATTTAAAGTTTAGGCCTTTTGATTCCAATATTATCACTTTGATGTAATGATTGTTAACTAATGTGTTTAATGTGTAATGAAGCATGATTTCTGTATAACTAGTTACTTTCGATATTACGGGCCCATTTTTTATCTACAACACTACAATATTTTCGTAAAAAAAGCATTCACCAAAATAGATAAACAAATAGAGAACGAGTCTGATAATACCTTGGTAATTTTCCTTTATCTCGGATGCAGTGACCCGAATCAGAAAACGCGCGTATTTCGGGTACAGTGTATCCGAGATATGCTTATGTTCATATCACAGTTGCATCCAAAATATGATTCGCAGACAGTCTATTAGGCTCCGTTTGGTTGATGTATAGGATGAGACATAGACACAAAGACACAGACACTAAAAACATAGACATAAAATATTTGTGTCTATGTATTATGTTTGGTAAAGATAATGAACAAGACACAAATATTTGAAAAAGTCTGAATTGTCCTTCATTCAACCACAAATTTTATCAACACCGCTACACATCCATCACCTCAAAGGCTACATGTCATAACTATTAAATTTTTATTTCTTTCGTATTTTTTTTCTTCTAATATCATTTCAAATTATCATTTAAATATTAAATATATAATTTTTTTTGGAAAAAAATAGAAAATCAAAGTCCGAAATTTATCAAAGATTAATTAAAATTCAAATAAATAAAAAATTAAATGTATAATTTTTTTAAGAAAATAGAAAAATAAATTTGGTTGTAGAATTTAAAAGAGGAAGTAAAAAAAAGAAATATTTGAAGGGATAAGAGGATAAATTTTGAAAATTTAAAAATTGAATAAAGATAAAAGAGTAAAAAAGTAGTGTCTCACAAGTTGTGTCTCAGTGTCTCATTAAATTGGAGGTACACTAATTTAGTGTCTCCGTGTCCATCTGTGTCTTCCCGTGTCCATCAAAAATCTGTGTCTCACCAAACCAAACAGCAGACATGTGTCACCGTGTCCATGTCTCAATGAGACATGGACATCAACCAAATACTACCTTACAGCACGGGGTCAGTGCACCTGAGATGTGGCCATTTGAACAATGACCCTCAGTACTCAAGATGTGACTTAGCATGAATTTTAATAATTTCTTCAACGTTTATTTATTTTGATAAATACTATATTTATTTAATTTAAATAAAAAAATCTCTTTTAATATACATGTATATTTAAAAATTAAAATTGCATAAATATTTAATCATATATTATTACATCAGTACAGGTATTTGATTATTTAACTATTTTATTTATTGTATTATTCGTTTAAAAATATAAATATATAACTGAATAAAAGAGAAAAGAGCAACCTAGTGTAAAGTGTTATACAAAATGTAATATGTAAATGGTCTAATATATATAATGCAAAGTTTCAGTGTTTAATTTCGTAATTTAACCCATAACATATAAAAAATCCTACAAATACATCTCAACTGGTATCCCAACATTTTCTTTTTACCTAACTAATAAACACTAATGTAAAAAATTTTTACAATGTTACTGCACAGCAATGAATCTGAATCTTCTCAAGATTAGCATATGACTAATCATGAAGAATTCAAAGTTACTTACATGATAACAACAAATTACCTAGTTTAATTAAAAAGAAAAATAAATAAAAAAGCGTCTGGTGATAAAATTTGTAGTGATAAAGTATAAAGAGTATCCAGTTACATAAGTGCGACAACAAAGTGATGCTCCATCAACATACACTAGCATGGTGATAATTAGATGCATGGTATTTGGGCAAGTGAGCAGAAAACAAAATTAGCGAAAGTATTATGACACAATAATTATCTCATCTCATCATAAATCAAATAATTTGGAATGGGAATGAGTGCTTTAGCCTTATAAACAAAAAACCAAGGAAGTTGCCTTACATGCCACGTTTC

## >AdNAC40

CCTAACCACATCATCTGATAAAGAGGAGCCAAAGATTTCCATCTTGATGCCGGACCCACCTATGTTCTTGGTCGTCTACTCTTTATATATTTATGTTCTCTAATATAATATATGATGATTACGATCAATATTATTATTATTATTATTCAAGAGTCATACCTGAACAAAAATAATAATGTTGATATACATCCCAAAAAGAAATTATTAATGTTTGTTTTTCATGTTAAGCACGGTGATTTGTCGATTATTACTGTGGGGATTGAACTGTAAATAGACAGATTTTTAAGTCTAACAACATCAATTACTTGAATGGGTATTTTTAACGAAAACGATAGCCAATCGGTAATACACAGTAGCGCGCTAAAAATTGGGTAAGAGCATTTGTTGTCGAAATAGGTTGACATTGTTGTTGAATGGTTAGGGTTAGGAATAGTGTTGGTGGTAAAACAATTGGATGATTTTTTATAATCATCATAATCACGACAAAACTATGGCCATCAGAAAATGCACCCTTTTATATATATATATATATAAGTAAAGAATATAGTAGTAAAGAAAACAGTAAACTATTTGGTGTGTGTGCTTCTAGTTCAAGTTTCGTTTCAGAAGTTTAAATCCAAAAATATATATTAATATATGATTTAGTATAATAATAGTTGTAGTAATTAGAAGAGAGGGTGGTGGTGGTGGTGGTCCATCAAGTACTAGACTGCACATTTATTTTCCTTGATTATCAAGCAACCTCTCTGATCTCCGAGTGAGTGAGTGTGTGCAAAACATTCATGCCAAAAGGCATCATCATCACCACTCCAGCTCAATACCCATGTGAGTTTCTATCAGCCTTTGCCCCCCTCCCCCCTATTTTTCTCTTCTCTCTCTCTCTCTCAGATAATTTTGGCTTTATTATGTATGTAGATATGCTCCATATACATGAATTTGTTATATGTGCACTGAAAATAATGTAGGTGTTAATTAATAAAAAAAATTTTAAAATAAGTATAATTTATTATTTTTGATCAATATTTTTAATTATTAATTTAATTTTTTTAATTTAAAAATAATTCAACCATATACTTTTAATATATAATTTTAAGCATTATTAATTAACTACCGACTAAAAATAATAAATTTTGTTCCTTCTCTAATATTTCTATTAATTAAATTGTAATTCTTTAAAAGTTAAAGCTATATATTAGTCATTTTTATTACTTATTTATGGATATTCTGAGCTTAATTATTTTTATGATTCTGACACGTATTAATAAGTGATCAGTTAATTTTAAGAATTATATTAGATAATTAATGACTTTTTTAAATAATATGAACAATAAATTTTAAATTTAATTATTATGAAATATTAATTATTCAGACCATTCATTAAATTTAAGATTAATTTACTTTTTTAATTCCATTATTCATATTATTCAGGAATATTGTTGTTTACTTATACTTTTTCTAATTTTAATGTGTATGGTAACATTATTACATGGTTCCCAACTTCCCATAGCTTAACTATGGTAGAAAGCATTGCAATATATATGTTTCTCTCTAATCAATTATTTGTTTGTCTTCCTTTTCTTATTCTTTGTTTTCATATACAGGACTATTTATTTTTCAAACTTGTATTTTTTAGTTATATATATACTTTAATGGTTCCAAATATATATATAATTTTGTGCCGTTTTGTGCTTTATTTGCTTTAGCAGTGAAGGCTGAAAGAAAGGTTCTGCCTGCCTCCTCAAGTAGTAGAAGGAAGCAGCATACACATACAAGAGAGAAGAGACGAAGGCAGCATGGGCTTTCAACTTTAATTAGTTAGAATAAACCAAACGGCCTGGATGGTTTTCTTTTTTTTTCTTTTTCTTTTCTTTTCTCCTTCTTTCTTTATTATATCCTGTTTCTGTTTCTGTTTCTCTCTCTCTTTATTAATTTCTCCTTTTTCCTCTTCTCACATAATTAAACCGCGCAGCCTAGCTATTACTACATACATCCGCTTCATAAATTAGCAAAGTCTTAAAGGTGCTATTCTGTGCTATGTGTTTGAAGCCACACCCAACTTTTCCTTTATTATAAATTTTTATTCGTCACTGCAGAGAGAGAGATTAATTTCTAGGTTTCCTGCATATATAGTGCATACTTAATTTCATATATTCAGCACAAATTAATTTTCTGTTGATACTCTAAATAAAATATATATGTAGCCTAGCTACCACTGGTTGCTTTTCTTGAGAAAAAGTTATTAGTATTTTCGATTAAGGTATAATAATAATTTTGTTTTAATTATTCGTTGTAGCCTGGAGCCACTAGCTAGTTTCTAAATAATCGAATCGTTATCTGATGAATTAGATGATTGCAAGATGTGATCCAGTTCCAATATATATTAATCAAAGTGTTGGATTTGTGAGTTATAGATAGAAGAGAATGATGATAAGTAGTAGTAGCAGTAGTGGATTGTTGCATAGAAAAAGAATTGAAAATAACAAGGAAAGAAGTAGAATCG

## >AdNAC41

TAATTGGTATATTTGATTTTTAAATCATTATTAATTAATAATTATTTAAATTTTATTTTTAAAAAACACAAAATAATCAATCACTATTAATTATTACAGTTATTTAAAAGTGACTGATTAGGAGTTGTTTATCTATATTTTTCAATACTAAATTATCTTATGACACAAGCATTCCCCGGTCTTGGTGGAATTAATTAATATTATTGTAGTAATTAATGAAGAATGAAATTCTATTATGGAAAACTGTGTTCTCTTTCCTCCCTAGCTAGCTCTAACAATATATATACACATAGATACACACCTTAGTGGCTCTCTATCTATAAATAAAAAAATAGTTAGGTTGATGAATTGATATACATTGAGATGGGGCTTCGAGACATAGGAGCATCACTTCCACCAGGGTTCAGATTCTACCCGAGTGATGAGGAATTGGTTTGCCATTATCTTTATAAGAAGATCACAAACCAGCAACTTCTTAAAGGCACTCTTGTTGAGATTGATTTACACATATGCGAGCCATGGCAGCTTCCAAGTATGTATCTTCTTTTCTTTTCTCAATCAATAAACAACCACATGGATGCCCTTTTCTTCCATATCATTCTTTCTCTTCATTCACATCAATTAATTAAAACCTTCTATTTCTATATTGAACCATTTATATCTTCTCTCTCACTCAATAATCTCTCTTCTCGTAATCAATATTTTTTTTACTTTTATATTTAAATCAAGACTAGCCAATTTTTTTCCTTTTTTTTTTTGCTCTTTATTACACTGCTACTAATTAAAAGTAGGTAGAAACTCAGGTGCAGTTGACTTCACGTGAAATTGATAGTTGAGAACTGTTAGATAAAAATTTAGTCAAATCAGTCAAATTATCTAACGACTCTCAATTATCAACTTTACATAAAGTTGACTGCACCTGAGTTTTCACCTTAAAAGTATTGAACTCTTAACATTTTTTATTTATTTTCAATTTTTTACAGCCTTATTCCTTGAATGTTGTTGGGTGTAACTACTTCCATTGTTCTACACATTTATTATTTTACTTTATCTCCTTGGAGAATAGAATGATGCAATTTTAATTTGCTTCTGTATTTCGAGAGCCAAAATATAATAAATCCTTTTTTTTTCTTCCCTAACCTAATTGATGTTAGAAAGTGAACATTGAAGCCATTTGTTTTAAGCTCTATTGTCATCGGCATGAAGTTTTTTTATTTATTAGATCCATTCTCTTCCTAAGCTAGTTCTTAAATCTTAATTTATTTTCCTTCAATTATATTCTAACATTACTTCGTTTTATGGTCTTTAATGATTTTGTCTTTTGTATTTTAGTTGCTTGGTTTATAGAATAATGATAACAAGAATAAAAAATTGTATAGTGGCTAGTGGTTGGTGTATATTATATATAAACTTGTTTTCTCCTCTATATTCGGATTATTATTTCTTTAAAAATAATTGTGTGTGTGATTATATCCGCCTTATGTATGTATATATGTATGTATACAGTATTTTTAGAATAATACAGGGATCTCTAGCTAGCTGGCAGATATATATTCAAGAGTAGGTTCAAATATAGAATGAATAATGATCATATTTTATTTTTTCTTTAGTTTAGGTATAAACATGGTAATTATATTTTTATTTAATTATAACAAAGTTGACAAAGCAATACATTCTTATTAAGCAACCAAACTATATATAGAGATGATTAATTATTATAAAGATAATTATTCACTTAATTATTATTATATGTATATATATACGTGTATAAATGTCTCATTTACCTTTCTTGTGCATGATTCGCTATCTTGATGATTTTGACTCTTTAGCACTCATAAATATATAATTAGTAATTAAATGTTCCCTCAAGAAATGTTAAGAAAATCTAACTTTACACATGAAAAGACATACTTGATCTTTGTTGCGTAGAATAATTAATCAGCCCTCATCTCTTTGATTATATATCGGTGGAAACTCAAGTGCAGTCGACTTCATGTGAAGTTGATAATTGAGAGTCATTAGATAATTTGACTAATTTGACTAAATTTGCATCTAACGATTCTTAACTATTAACTTCATGTGAAGTCGACTGCACCTGAATTTTCACTTTATCTATTATGATGATTCATGCACAACATTATTATGAAATATGTGAAAAGAGGGTGAAAACAAGCTAAGGGAGAGGATAAAAAAGGTCACGTATTTACATAAATAGCAACCTTGAAACTTCCAAACACTGTGTTGGTGTATGTCATATACCACTATTCCTCTTGTCACCTTTGTCTGCTGAGATTGGAAACTTGAAAATATCATGTCATTCACGACCTAGTTGACACATGATGCTCATTCGACACAAGACCTAATTATTGTTATATTATTTTAGGATTAGAATTTTTCTAATAAAACTAATTTAATATTTATTAATTAAGGATCAGATTAGTTCTTTTAAAAATTTAAACTGATAAAAGATACGAGTATTTTTCTATCATGGTATCATAAT

## >AdNAC42

CATATATACATGTATATAAGAGTAAAAGTGAGTTAAGTCAGTTCAAGTTCGACTCCTTAATAGTTTGATAAGCCGAACTCGTGAATTGGTGAGTTGAATTTGAGCTTAAAATTGAGATTTTATAGTCTTTTTATATATATTTTTAACGTAAGATATAAATAAAAAATTTATAATTGATCGATAGACAATATATAAAATTGATCTTTTCTTAATATATATAAGTTACAATTTATTGATATAGAATTATAGACTATGTTCCTATTATTTGAGCCAGCTCGTGAACTTTTGTTGAGTCGAACTTGAACTTACAAAATAAGTTCGATTGTTAATGAGTCGAGTTATAAGTCAAGCTTAATTTTTGAGAACTGAGCTTGAGTTTAGTCTAACTCGACTCATCTTGACTCACTTCTAATCATATATATATATATAAAACTCATTTTTATATTTATCGAACGAATAATTATTTTAAAAATAAATATAAATACTATATTAAGTATTTAATATATTTATACATAAAAATTAGATTATTTCTCCATTTGTTTGTTAACCAACTTCTTCTATGAAACTTCCCACATTGTTCCAAACTATAACACACTTCTTTTTCTTTAATTTTGTTATTCATTTAACGTAATTTATTTGTACTTAAATTATAATTTCAACATGCATATTGACCATGCTTCTGGATAAGGTTGATCTGGACCAATTTTTCTCCTGAGAAAAAAGTTGGTTATATATATATAACATGCTAGAGGTGTGTTCTTATTTTATTTATTTATTTATTTTGATGAAAAGAGTGAGGTTTTTCGTCTGTTGTGGATAAATATCAACAGTACACAATTATTAAAGTGTAAAGCAATGAATGGGTTGGAGGTAACTCCTTTTTTCTTTTAGTTAGGGGGATCGGATTCTATCCGCATATCCGCAGTGTTTATTTGAATTTGATCCGAAAATTGTGGATATGGATTTGATTTGTAAGGCTATCGGATCAGATCGGATCATATTCGCACAATAATTGGATCAGATTGCGGATTTTGTGTAAGTATCCACATATCCGATTCGCATATCCGTGTATCCGCAAAAATAAAAAATAAAAAAATAAATATTCTTTTTATGTTTTATTTCAACTAATAATTATCATATATGTTGTATTATTTTAATTTATTATTTAAGAAAAGTATGTTTAATATTATTTTAAGAGTAAACGTATTTTAAAAAATAAAAAAATTAATTTTATTGATATTTTTTAATAAAAATAAGTTTTTAAAAATATTTTTGTATTTTGCGGATATATCTGATATCCGATCCGTAAATGTGCGGATCGGATCCTAACTAAAAAAACTACGGATATTGAATCCAATTCGATATGATGATTTTAGTGCAGATTTGATCGAAATTTTGGCCATATTCAATTCGCGTTAACCCTTACTTTTAGCGTTAAAATTAAAAAAAAAAACAATAATATGTACACTAAAAAAATAATATGTAATATGTACACATCATATGTATGGTCAACTTTTATGAAAGTACAATGCGAGAGTAAAAACTTCATATTTTAAAAAAATTATGTTATGGGATAATATTAAAATTTATGTAAAAATTTAAAATTTCATTATTATATGCTGTTTATATTTTCTAAAATAGATTTTTATTGTAAGGTATATGAATGGAGAGCACAGGATATCAACAGGGACATGTATAATAATAATCTAGGATTTACAAGTATTTTTTAACTAAATTATAATTATATTTTAATATTTTATTGATATTAAAATATAAATTAATTTTTTAATTATATAAAATATTTAAACTATTTTTTATTTTAATAATTAATAATATATACTATTTTTAAATTATTCATCGAATACATGTTAAAAATAAGATAGGACATGCTGACACATGATGAAATTTAGGTATGTTTAAATGTGTCTAAAAAAATTTTGTTCCTTATTAAAACGCGGTTAGGTATATAAAATATGCATATCGGACGAGTGTCGATGAGTATCGTATTCGAAACGTATCCGACATACGAACACGATAAATCAAAGAAGTATCCAGGAAAAAAATAATAACAAAAAAAATTAAAATAAAATAGAGAAGTAGCTATGTATAAGTTAGTGAATCATTGTTTGAGTCAAGAAGTAAAAGGATAATTACTAGAGATATGTATGCACAAAAACACAAGGGATGTAAACAGTTACGTGAAAGGCTGTCTAATTAATTGGGTGAAGTGTTCTTGGTCAAGTTTTCAGTTTTGGATATCAAAACTTAACTTATCCCATAAAAGTAATAAAGACACAAACCTAAAATATAAAAAGCATGAAACTTCCCAATAGTTTTTCTATCTATAACTATAAAGCCAAGGTTCTCTGAACTATGCACCCAATTTTTCAAATATATATATAAAATCCACTATTTATAACCATTTAATAAGAACAACATCTTCATTCAACTCTACTCCTTCCATTATTCTCTCTCTCTCTTTTTCCTTCTTTTCAGTTAC

## >AdNAC43

AAGAAAGGAAGAGAGATGTTAGTAGAAGAGAGGAGTGGGTAACAGTGAAGAAAGAAGCTCACACGATCAAGAAGATTGGAGTTGCGCACTGCGTTTTAACGCAACCTCCCTCTGTTGTAGTGTGACACTAGTTTTCTGCAGAACAAAGAATTTGTAGCTATTTTAGAAAAATAATAGAGTGATAATTAGGTATCGAAGATTTCAATTTTAGATAAATTAATTTTTAAAAAATATTAATTAAATTTTGGACAATTTACTAAAATAAATAATTTAGCTTCCAATTTTATCAGAATAAACATTTTATAAAAAGGATAGTATTTTTTTTTATAAACTTTTTTTTTATAAACTATGAAAACTGTGCCGATGAGTAATAGCTCAAATGGCATAGTCTCCCCAATTCTCAGGTTATTCTACTTTCTTTTTTATATATTTTTTGTTAGATTTTTTCGTGATGCTTTTGATTATTTTGTTTAAATTTTATCAAAAGATCAATTTTTTTGAGATCTTGGTATGTTGTGTTTATTTATTTATTTTTGTTCTTATTTTTCTAGACTATGCCTTTTCTAACTACGGACTCATTTCATTGGCTAAGTTTTCTGTCATTCTAATCAAGGAACTCTCAATAATGATTCTGATTCTTTCTTCAAGTTTAAGAATTTATTTTTATATAATATCAAATATGAAATAAATTATAATTTTAAAATAATTAATTGCATTTATATAGATCAACCATTAAAAAATAAGATTGATAAGTTAATATATTATTCATCATAACTCTTTTAATTCAGGTAATACATACTCTGTACTAAATAAAAAAATTTAACAAATATACTTGTTCAATCCTAATTTTTATAACTATAAATTTTAGGACAATTTACTATATTAAATAATATAAAAACCAATTTTATCAGAATACACAAAATGGAAAATAGATACCAAAATACATTTTTTTCCTAATCTATGTTAACCGCCATTGGAGTCCCGCGACTTACGAATACTAATCCACTATAGGGGTGTCACGGATTACATGTAACTAAAAATGACCATAAACCGCGACAGGGTGTCGCGGTTTACGTGTTGGCATAGGATGTGCATAAACCGCGATATGGGTGTAGCGGTTTATGCATTAGTATAAATACGTTAAAGGGATTTCACAGATTACATTTGTGCTGGAAGAAAAATATTTCTAGAGAGAAGTAGTGATTTTCTAAAGAGAGGAAAAAAGAGAGAAGTGGTTGAGTTAAGTTGAGTTGTTCGTGTATTTTTGTGTACTCCTATATACTGTTTGTAGATCTATATACTATTTGGAAATGATTATAATTGATGAAAAAGGCAGAATTCAAAAAACTTAAAAGGAAGTCGCCAGGCAAACTTGACAATTTTTATAGAGTTTGCCAGAATCTAGCAAACTTGCTTAAATGTAAAAAAATTAAAGTAAAAAAACCATGTTTGTGAAAATATGGATTTATTTTATTTTATTATTAAAAAAATTCCTGTACTATCACCATCACATCATCATGGACTAGAGAATCATGAAACAAGGTATAAGCGATAACCACCTTCATCAGTTGCAACAGGCGCGGCCAACACCGACTTTCATAAACCCATATGTTTGTCACCAATTCATTAGTTTCTTTAGCAGCATTTTGGTGTCGAGTGTGAATTTCCTTCTGGATGAGGCGGTGACGGCAACATCAAGAACCCCTTTAGATTCTCTCAGATAATGCACTCCAACTCTCAACGAATGTGCCCAGGAATATGGTAGCAATGGCAGTCCGATGAAAAACGATGGTGACTGTGCTGCGTTTAGATACGATAACAACATTATTTATCTTAGGGTGAGAAGAAGGAAAGGAAGGAAGAGGGAGAGAGAAAAATTTTCTAATCAAATTGAGAGCAAGAAGAAGAGGAAAGAGTAAAACGACGTCGATTAAGTACAAACAGCAAAACGACGTTGTTTCTCCCGTGATGAAAATGGATTTGAATTCTTTAAAATTTGAATTTCACTTTAAACAGTAAAATGAGATTTTCTACTTTTAAACATTTTCTCTTTTATATTTATTTTTTGTCCTACCTATAAAATAAATGGTGAGAGATCACATTTTACTCTTTAAAGTGAAATTTAAACTTTAAAGAATTCAAATCTGATGAAAATGGATGGAGAGGCCAACTTGAGTCACGCCTTAAAAGTTTTAAGATACCATTTGAACCAATTAAAAATTAAAAAATTAAATTGAATCGGATGTTAAATTTTAAGAACCATTTTAAAGATTAACTCTCAATTTCATGTAAAAAAAATTATAAAAATAAAACTATATATAATAATATAGAAATGTTTGTTATAATTATATAATTTTCATGTCATTTCATAGCTTTTTATTTTTCTAGAAAATAAAATTTATTTACATTTAAAAAATAAAAATAATGTTGTTTATTTTCTGTTTTAATTTTTTCAGATTATAAATAAAAAAATTAAAAAAAATAGCGCATCCGACACACA

## >AdNAC44

CCTAAATCAAACTCAATCAGCGACATTACCTCACCCTCTTCATCAGGTTCAGGAACAACATAATTCAAAGTACTATAAACAGCCCTAACCAAGTGCGGGTAATACTCATCTCTAGTTTGTACAAAATCAACTAAGTGTTGTGTTTGCAATGCATTCAACACCAAATTAAAACAGGAGTGGATGTTGGTGAGATTGGAGAAGAAGAGAGAAGAAGATGAAGCTTTCTGGTTCGATGAGAGAAGAGAGCGGTGCAGAAATGGGGGTTCGAATCTTTTAAAACGTTACTTCTAATTCAATACGCATGGTTTGGGATTAATATATAGCGATTTTGAAATTGCAATCGATTGGATTACAATACCAATCGATTGAATGCTGAGAAAAACACATTTAGGACACTTCCCAATCGATTGAAGTTTCAAACCAATCGATTGAATTAGAAGTTACGTGCAAAGTTCAATTGATTTTTAGATAGAAACAATCGATTGAATATTAAGACCAATCGATTGTTTGGAAGAATTCAATCGATTGAAAATCATAAACAATCGATTGAATTACATAAATCTCACATTTAATTCATTGTTCAATCGATTGGTTACTATGACCAATCGATTGATTTATGCAAAACCATGCCTACCAGGAATATACAATCGATTGTTTTGTGATAAACTGAAATCCAATCGATTGGGTTATCATTCAATCGATTGGTTTGATAGTCTAATCGATTGGATTTCAGCGAAACACGATTTGAAAGTCATGGAGGAACTTCACGAGATCAAACATAATATTTTACTCGACTGGAATATAATTAGTTGTTGTTTTGTATTTTTTTGTTAATAAATATAATGGTTAATTGATAAATAAATAATTTGTAAAATAAAAAATAGTTTCTATAATTAAATAAAATATAAGGGGTTAATTTGCAATTAAAAATAGTTCAAGGACTACATAGCAATTGATAAAAAACTCTAAAAATATGTTTTCTACTTGGACCACTAAGTAGTCCAAAGGTTCCTGGACCACCGAATCCTGAGCCTTGCTTGTGCATGTATTGCGTTTTGAAATCTATTTTAATTCTTTAAAATTAATATTATTTAATATCAATTTGTGACTTTTAATTAAGTATTTTAATTTAAAATGTAAGATAATATAATTAATTTTCTTCATTCATTAAATTAAAAACATTAAATTTTAAAATTTCAATTATTTAAATTAAATCATATAAAATTTTTATTCTTTTATAACTACTAAAATTTAAAATTTATATATAAAAATATCAATAATTATAGAATTAAGTAGAATTTCTATTTTAATTATCAAAATTTGATTTAATTATTTTTAATAAAATTATTTTTAAAAATAAAATCTTTTAATAAATAAATAAATTTAAAATCGGCTCATAAATAAATAAAGTCTTTATATAATTCGAATTCAATAAATTCAATTTATATAGAAATGTAAACGGAGACAAGTAGGTAACGATTTTGAGTTTAGAAAATTCAGGTAATTTTTGGGTGGCTTTGGTTTATCAACATAAATTGCCCTAAAATATATGAGTATTTTTTAAAGTATCTAAGGTTGACTTTTTCAAAAATGACTTGTATTTATCAAAATTAAAAAAGTCTAATATAATCTCATATATTAATTAATATCCAAAATTAATTCTTATTAATGTCTATTATAATATTTTTAAATTTTAATTATTTTACCAAATGCACTACCACTTATTGTTATTTGTGCTTATTGAAAGTCATTTTTAATTTAAATTATCAAACATAAAAGATAATTTTTATAAAGTAATTTTTATAAGCTACTTTTGAAATGTAAAAATTTTATCAAATTAAGCGTAAGTGTTAGTTTTAGTGATGTATTTGAGTAAAAAAAATTATTTTTATAAAAAAATAAATATTTTTTAAAAAGAATTATATATTATTTAGAAAATATAATTAAAATATGCTTTTAATATTTGAAATTTTTTTAAAAAAATTTACCTAAATATAAAATAATTTTTGTCTATTAATTTTTTTAAAATATAATGTTTTTAACTAGGTGATTGTATCTTCTCCCCTATAAATTTCTGTCTTCAGATCTTTTCATAATTAATTAGAAAGATATTTTATTAATATAAGATAACATATGATATCTTTTTAAGTTCAATTAGAAGATATAATATTAAATTTAAAATATGATTTAATTATTTTTTGATGATATGATATTAAAAATTGACATATTTAACTTTAGTTGATGTCAACTAAAAAGAATAATTTTTATCTTAGATTTTTTTTGAAAAAATAAAATAAAAAGATGACATAATTATTTTGAACCAAAACATCCCAGACAAAATAAAAACGAACATAAATTATCATATTTCAATCTTTTTATTCTTTCAATCAATAGATGCTTTCATCCAATATATAAACTCTACTATCGCATTATTAAACCACTTACTTCAATAACTTCTTAATTCTTTAATTATTTGTTTCTTTCTTTTTTAATCTTGAAT

## >AdNAC45

TTTCTCAAATTCCATAGCCAAAGAAGTTTTATGTGCCTCAAGTTCTTGTGTGCCAATTTCAGGAAGATCTTGACGAGTGGATTTTTTATAGTTATCAATCTCACCAAACAACCTATAGCTATCAAACACCTTGAAAAGAATCTCTTTCACCTTTGAAATCAAGAATTCAGCATCTTTTTCATACAACTTCTCAAAACTCCACTTAATCAACTGAAACTTATATCTAGGATCAAGAACTACTGCAATGAAAATCATCATGTTTGTGTTTTTTATATTTCTCCAATACTTATCATACTTAGATTTTATTTTCTCAGTCATGCTTGAAAGTACCGGATCTAAACTTTTCATCCAATGTTTAAGTGTAGACAATATTTTACAAAAGTCATTAAAATATTGAGAAGATGTCACAAATGTTGAACCAGAAACTTTGTTGGTTACGTCATAAAATATTTTTAAAAACTTGACAAAATGCCTTGCGTTCTCCGAATCCTCAGACTTAGGGATCCCACCAGCCATCATAGCATATTCAGAATCTCTCTCTCCCAATCGTTAAATACCTTTTGAAATTTCAAAGTACTTTCAAGCATTAAAAATGTAGAGTTATATCTGGTAGGAACATCTAAATGCACAGTACCTTTTTCCAGAATTTATGGTGATGCACGCACATGCCTAACTGCATTTCTAATCTTTAAAATTAAATCATACATTTAAAAGAACCAATTTGTGTATTCTTAATTTATGTAATCGAACCATTTAATTACTATTCTTTTACTCGGATTAGGGTTGTTCACGATTTAGTTATTCCAAAAACCAAACCTGTTTTTGGGTTAGCCAAAAATATAATTTGCTTAATTAACCAAATTGGTTTTATGTGATAAAATTAGTTATTAACTAGTTAAAAAATTCAAAATTCGATTTTTAACCGGTTTAAAATAAAAATCAATTTTGAAAAATAACTAATTAAAAAAAAACTAATATTTTACATATTTTTTTATAAAAAAAACTGATTTTAAAAAAAATAAAAAATAAAAATCGATTTTTACAAAAAAAACCAATTAACTAATTTGAATACAAAAATTACATAAAATTTAATTTTAATTTTAGTTAATCAATTAAAAATTAATTTTTAAAATTTAATTTTGATTAATTATTTTTTAATAATATAATATAATTTTAAAAATTTTTTTTGAAAGAGGAATTCAACACAACAAGTAAAGCATAGAGGACAATACCATAAAAACACTAACTAATGAGAAAAAAAAACACAAACCAACAAAGCAATTATAGTGTCTTCTCTATTATTTTTGGCATTGCCATAAATAACAGAAGGAATCTGCATCTAGCCACTTCTTGTAGCTCAAAAAGGTCATGTTAATAATCTCCTCAACTCCTTTTTCTTTATTCTGAAAAATTCGCTGGTTTCGTTCCAACTATATGTTCCAAATTATCGCACAAAAGCACACCATCCATCTTTTACACTCCTCTTTTTTATGTGAGATCTCAGTCCAACTTTAGAAGTGCTCATTTACCATATCCGGATAAATTCATCGTAAACCAACATATAAGATCTAAACACACCAAACCTGCCAAGAAAAAGTACAGTCAAGAAACAAATGGTGGCCACATTCAATACCATTGTTACATAAAACACACAAAGCATCTTCTTAGTGAATAACTCCAAACTGAATCAGTCTTTTCTTCGTATTAATTTTGTCGGTGAGAACAAACTAAACAAACAGTTCTACTTTAAGTGAGACCAGACCTTTTCAAATAATCCTAGTAAAACTATAGTTTGATATATTTTCTGATTAACCATAATTTAATTATTTTAATACAATCATCACAGTTCGATAAATCTTCCAACACCCCAAAGGCCCAAATAACATCGCTATACAGCAATCCTACCTCCATGTCAAAATTAAAAAAGTGGCGTAATACTTGGTAAGAAATTCATTACCGAGTGCCCCATCCGCGTTTAAAAAAAAAAAAAAACTGAAATCCTTAACACGCGTCGGACACGTCACCTTCCTGTTTCGGCTTTCAGCTGCGCGCTAATGGCATTGTGGCAATTATTTCCAAAACCAAGGTCCAAAGCAGCACACAATAGTATAAACGAGGAATTTTTTTTGGGTAAATAAAACGAGGAATTTTTATTTTAACGCGCTTCACTTCTCACTTCTCACTTCTCAGTTCTTCCTCAAACGAAAATCCAAATAGTACAAATACTGCACCAACCCTTCTCTCATCTCTGATATTTTTCTTTATTTTTTATATATATATTTTCTCTTTCGATCACTGTCTTCTAGAAAAAAAAATTACTAAAATTCTCAGAATCCTCTCTGATTCCAGTTCAGCTTCACTTAGTAGAAACCAATCAAAAGATCATCGTGAAATTTTTTTCTTGTTACTTTTTTTCTTCCTTTGGTTGTCACGAACGGTGCCGTTTTATATATACATAAAATAGAGACACCGTCGTTTTGACACAAACGGTGGTTTTGTT

## >AdNAC46

NNNNNNNNNNNNNNNNNNNNNNNNNNNNNNNNNNNNNNNNNNNNNNNNNNNNNNNNNNNNNNNNNNNNNNNNNNNNNNNNNNNNNNNNNNNNNNNNNNNNNNNNNNNNNNNNNNNNNNNNNNNNNNNNNNNNNNNNNNNNNNNNNNNNNNNNNNNNNNNNNNNNNNNNNNNNNNNNNNNNNNNNNNNNNNNNNNNNNNNNNNNNNNNNNNNNNNNNNNNNNNNNNNNNNNNNNNNNNNNNNNNNNNNNNNNNNNNNNNNNNNNNNNNNNNNNNNNNNNNNNNNNNNNNNNNNNNNNNNNNNNNNNNNNNNNNNNNNNNNNNNNNNNNNNNNNNNNNNNNNNNNNNNNNNNNNNNNNNNNNNNNNNNNNNNNNNNNNNNNNNNNNNNNNNNNNNNNNNNNNNNNNNNNNNNNNNNNNNNNNNNNNNNNNNNNNNNNNNNNNNNNNNNNNNNNNNNNNNNNNNNNNNNNNNNNNNNNNNNNNNNNNNNNNNNNNNNNNNNNNNNNNNNNNNNNNNNNNNNNNNNNNNNNNNNNNNNNNNNNNNNNNNNNNNNNNNNNNNNNNNNNNNNNNNNNNNNNNNNNNNNNNNNNNNNNNNNNNNNNNNNNNNNNNNNNNNNNNNNNNNNNNNNNNNNNNNNNNNNNNNNNNNNNNNNNNNNNNNNNNNNNNNNNNNNNNNNNNNNNNNNNNNNNNNNNNNNNNNNNNNNNNNNNNNNNNNNNNNNNNNNNNNNNNNNNNNNNNNNNNNNNNNNNNNNNNNNNNNNNNNNNNNNNNNNNNNNNNNNNNNNNNNNNNNNNNNNNNNNNNNNNNNNNNNNNNNNNNNNNNNNNNNNNNNNNNNNNNNNNNNNNNNNNNNNNNNNNNNNNNNNNNNNNNNNNNNNNNNNNNNNNNNNNNNNNNNNNNNNNNNNNNNNNNNNNNNNNNNNNNNNNNNNNNNNNNNNNNNNNNNNNNNNNNNNNNNNNNNNNNNNNNNNNNNNNNNNNNNNNNNNNNNNNNNNNNNNNNNNNNNNNNNNNNNNNNNNNNNNNNNNNNNNNNNNNNNNNNNNNNNNNNNNNNNNNNNNNNNNNNNNNNNNNNNNNNNNNNNNNNNNNNNNNNNNNNNNNNNNNNNNNNNNNNNNNNNNNNNNNCATTTAAATTAAAAAAATTATATATTTTACTCTTGGATTTAACAAAATATATTTTTAATAAATTATATATGATGAAAAATATTTTGTGAAAGATATTTGTATACCAATTTCTCATAAATATATTAGAAATAATAAAAAATAAAAAATGATCTTATTATAAATATATAATTATAAATATTATATATATAAAAATTATAAATATTAAGTTAAATAAGATAATTTTGTGTTTGACATTTCCTAACATTACAACCCTAGAAGAGAGATTCCACGTTGAGCCCTGGCCTAATTGTTGATATGAGAAAGCACATATATACCTTTATTTCTTTAATTTGATTTTGGAAATAATAAATAAATTTTAATGTTAATTTGTTTGGAAAAAAAAAAGTCAGGCTAATTTAGGGGCACTATCAACATTCAACACCCTCGTAGGTTGTACACCTTGCCGATAAGGTGCGGTTAAAATGATTAATGGATGGAAACATATGGCGAAGATAAACAGGAACACTTGTAAATCATTTTATTCCAAAAGGTGAAAGATCAAACCTTTTGTCATGCTTGAGGCCCATCATTAGGATTCATCCAGAAACCTGCACATTTTTTTAAAAGATTTTTTTTTTTTTTGGGACCAAGTCACAGCAGGCAGATTATAACAAATCAAACAATGGTCAATTCATGTTGGTGATACATCCAAATAGTAAAGTGAACAATAAAAAGAATGCTCAAACAATAATTAAGATCTTGTATATACAGCAAGGAACAATATCCTCTTTTGCGGGGTGTAGATAATAATTTTCGGAGAATGTTAGTTATATGTCCGTATATTTTTTATTCTTAGTATTAAAAATAATTAATAAAAAAGTAAGATAAAATTAAAAAATTGTCTTATATTAAGAATTATGCTTTTCACTGCATGGCGTAATTGTTTTTTACCCCTAAATGTAATAACGAAATAGATATATTTCACATTACTATTAATGTTAAGTTCGGAATTCATATATATTGAATATATGTAAACAAAATCTGAGATTAAAAGTTGTGATAGAGTCATAGAGATCTCATTAATTCGTCATCAGGCCTTTAATTTATATATAATTAATTTCCATATTATAATAAGTATTATTTTCCTAAATTTAATTACTAATTTAATGTTTCAAAAAGTTGTTTTATCAGTTTAATTTTTAAAATAATATATTTGTGGAAATTATATATGAAACAAACATAAACGGCGTTCCCCGCGTGAATCTCCAAGATGAGATGATCAATTTGGATGTGATGCATTAATTCATGCCAGGCAGAGTAAATAAGAATTAAGAACTTAAAAAGGTATGAAAGCAAGACATGAGTATAACCTTATCCTAATAAATTAAACGTTATCCAAGGC

## >AdNAC47

TGGATTCAGAGTATAAACCAGCTGAAGAAGAGGATGACACTAAAGATGATTTACACTTTACCAACAGTGAAGATGAGCTTGATCCTACTGTTAGTGGGTTTCAGGATGTTAACGTCATGAATGAAAAAAAAAGGGAAGTGAAAAAGAAGTGTGTCACAACTAACAACTTTGAAGATGAGGAGGGAGCAAGGAGTGATGAATTAGAAATTGATCATGAGGTTGGAACTGATGTGTCAGATTCAGACCACTAGGGACAGAGGTATCCAGTTCACAAGCATCAGAAAGACATGAGCCAATACAAGTGGGAAGTGGGCACAGTGTATGCATCTTGGGAAGAGTTCAAGGACACTGTGACTGCATATGCCGTGCAGACCGCTAGGACAATCACACTTAGGAAGTGTGATCTGCAGAGAGTTAAGGCTGTTTGTAATGGAGAGTGTCCCTTTTGGCTGTATGCTGCAAAAATTCGAGAAGTGGATACTTGGCAGCTTCGCAGCCTGAATTTGTCTCAAACATGTACACAAGCACACAGGGTGGAAATTTTACACTTGAAATGGCTTGGCAAGGCATTTAAGAAGAAGGTTGAATCAAATTCCAAGGTGAAGATAAGGGAGTTGGACATAATAAAAGCAGATGCCCTAAACCTATTGAGGATGAGGTATGTTGATTAATTTTTAACTTTGCTTGTCTGAGATTTTTTCATAAGTCTTATGTTCATCTCTATTGACTGCAACTGTTTCATAGGTCCAAAATTCCAAGAAACTAAGCAAAGGCAAGCAGAAGAAAGAAAGCAACAAATCACCCATTCTAGCTGCTAAGTGAGGAAAGAAGGCTGCATCTACACAGCCCACTCCTAAACTCACTATCAAGCAAAAAGCTGCTTCAACAATACAGCCACCAATTTCAACCCAGTCCAACTTTGTAACACAGCTAAAAAGGCCTAGAGGTAGGCTCATAGAAACCACTAAATCCAACTCTTCAGCCCAACATGATCCACAACCCAAGAGGATTGCCAGCCCAAAAAGCTTAGTACATTCTACATCCTCATCACAGCCAACCACCAGAACTCTTCCAGCATCTCAGCCAACCCTTGCCATAGCTTCTAGCCAACCCACAGGACACTTCTCTGTCAGCCTTTCTAGAGGACCTCACATTTTTCCAAGAAAACTTAAATTAATGGCAAAGTTGCTTCCAAGAAAATGGGGATTACTTTAGGAAAATAAAAAAATTAGCATCAAATTGTCATATTTCTATATCATGCATGTTGGTTTATTTTGATTTAAGTTTGTGTTAAGGTTCTGGTTAAATCAGTGTGGACTTGTTAGCTACTTTTTGTTGCTTATATTTTGCCTGTGTGCTATTGATTGTTGTACTGAATTATTGGCTTGTTCATAGCCAATCCTTTTGTTTCTTAGTTTATTTTGAGTTAAATTTCTTTTAAGGTTCTGGAAGTAAGAAACACTATGCTTATTATGTATTTTGACATTTACCATGTTAATGACACTGTTATGGAATTTTTCGGTATTGGAACTCATGTATTTGCCAAAAAAATTTTTCACTCCCATTTCATTCACCAACCATGCTTACAAAATAGTCATTTATACATGTTGAAATAGATTCAACATAACAGATTTAGGGAACCCCCTAATAGATAAACCTTAGCCCAATTTACCTACAACAACAACAGAATACACCTACATGTTTCATACCAGGTTCAATGGAATTACACACTGTTGCGTTGCTTTAGAGAAAGTCTACAACAGCAAACATACAAACCCAACCCACCCAAAAAATCTTAAAACAGCAATGACCTTTAGCTTCCACTCTACCGCTTTCATTCTTGATTTCAGCATCGAAACCTTCTTCCTTATTCTAGCAATTTCTGAGTGTTCCACCTCAGTCTCTGGATCTGCCCAATGAAAGAAATTATAGCCTTGTTGCACCTGCACATACCCACCGTGATCATCACTATCCATTCCATCACCCAAACGACCCAATTCAGCGGAAAAGCAAAAAAAAAAAAAAACAAACCTCAAAGTAGACACATCCCCAAAATCTGCGACCTAGGTTCTCCTTTGTACCTGAGATTCGCAGCACCGACTTCTCACCATGGGAACAAAGTAGCAGCCTACTATGTGACGAAGATCTGCTTCGAGACGAGCATGAGCTTTGTGCAGCCATGGCATTCTCCCTCCAACAGGTCCTCGACAGCAAACTCTGCAACAGTGGCTTCCACGTTGCTTTACCCTTTCTTCCTTTCAATTCAATTTATAATTACAAAGTTTTTTCCTTTTTTTATTATCTATACATATATATTTATAATTGTTTTCTGTGGTCATAACTACCTCATAACGACGAAAGGGCATTTATGTCCGAAAAAATTATAAAGGACGATTTTAAATTCAAATACGACTTTAAGGATAATTTTAAATCATAAATTACACCAAGGACGGTTTCGATTCTAACCCTCAACGTTAGGGACCAAAACAATACTTATCCC

## >AdNAC48

TAGAATAGTTATAGTGCTTATGACTGATCTATATATTTCTATTTTATTTTATTTTATTTTTTATAATGGGGCCAATGGCTGCTCACTTCTTAACTCATGTAGGGTGGTGATCTGTGATTGGCTCACTAGCATTTTATATAGTGAAATTTTCATAGATGCTTTTAATGTAGTGTAAGAGGTGTTTATTTTAATTTTGATTCGTTCTTCCTTAGCTTGTTTATCATGTTAACTTTGTCGGTTATTGTCAATAATCATTTTATGTAATTGAAAATTTTATTTGACTTTGTCTCATGCATATATATTATTTCTTACCATCAAATGACAAGAATTAAAACGGTCTTAAAATAATTTTTTTCAAAATTTAAATTAATAAAAAATATATAATCAAATTAACAAAGATCAAACTCAAAAGTTTTAGATCTTAAAAGCTTTGATATTATGTCATAAAACGAATTCTCTTATAAAAATTTAAACTGATAAAAAATTGGAGGCAAAAAAAAATAGTATATTTCTGATAGAAACTAAATATATAAAAATTTCACAAAAAACAGTTAATTAGTAGTAATTAAGAAATTAGTAAATAATAAAGTAACCTAAATTTTTTAATAAAAGAAAAATAAAAAATAATCATTTCATATAGATAATTTGCTCTAATTAGTTGTTAGTATTCTTTTAATGACACACTAGCTACCTTCTTCACAAAACTTATATTTGACAAAGAAACAAACAAAACTTGTTTTGGTTACACAAACAAGAAATCTTTAAGTGAATCAAAGTAAGAGAAATGTTTTTCTCTAAGAGCAAATTAAAAATGTTTCTTTTATAATTTTAATAACCTAGCCAATAAAATAAGCATAGAGTCACACTTGTCCTTGATGATGTGGAGCAATTTTTTTATGCACCACAGAGAAAGATTCAACATTTAGAAATAGTTTCTCACTAAAGCACTGGAAAGAAAAAAACGAAAAACGTGATTTTGGGGGGATTTACAAGTAAATTATCTTGATTCAATGTACGCTCTATACTATAGAATTTTTTTAAAAAAATATATGTATAATAAAACATGATTAATTAATTGAAGTGCATGCGAATCTAGAGAACTAATTAGAGTAGGAAAAGTGTCGGACTATTAACAATTAGGTCCCCTTTTTTTTTATATATTTTTGAGCCAACTTTTTTAAACAAGAAGGAACCAAAGTTGATTAACGAACGTCGTTAACGAAAAGTAGACGAAAATGATTATCAAAATATAGAAACCGAAAAACACGGTTTGGTAAAAAAAAAATACTCAAATTAAAATATTAGAGAACTTTTACAATCATATATATATAATTATTCATCTATCTTTTTCTATCTGTTTAACTATTTAGAGGAGGAATTTCATAACATAGGATCAGAATTTTTATAATCTAAGGCCGCGTTTGTTTTTGAAAATAGGATAAGATAAGATATTAAAAATAAGATATAAAAGACAGAAATATAAAATTTTGTGTTCTTGTATCTTATTTGGTGATAAATTAAAATAAATTATGAAAATTTAATTTAATCTTATTTTTTTTCATTCAAAAATTTTAGGAGAAAAATATAATAATAAAAAATATAATTATAAAAAATCTGTCAGGATGAACACAAAATACACTAATTCAGTGTCCCTGGACACATTATCTCTATCCATATCTCTTCTATCAAACACGATTTTGTATTTCTATATTTCTATCTCAATGTCCTGTCTCTGTCTCTGTAGATAAACGTAATCTAAAAGTCTAGAATTCAATTCTTATTAACCACAAAAGAAAATAAAACAAACATCGATATAAAAAATTACACAAACTCAAAAGAAGTTCTTATGTGATAGAAATATACCCAATTATTTATTAGAAATGTATAAATGTCTATAAATAATTATTATGTAGTAGTATATATTATTAATTTAATTTCATTACATTTGTGATAATAAAATTAATGATTCTATCTAATTAAACACAACGATAATTAAATTCAAAGATCACTTGTGGCGGAAGACAATGGGAGAGAAAAAGTAACTTTTTTAATAATACAAATAAAAAGGGGTCTTTTGGAGGGTTTCTATCTCTAATAATTTTTGGCTAGTTTAGGCTTTTGCATATAGGTTTCGGAAAAAATATAAATCATCAATTATTATTTATTTAGTAAATTATAATATTATTATTGTTATTATACTATTAAACAGTTATGTAAGATTTCTTGTAACTTTGGTATTTCTTGAAACTTCCTACCATGTCTAACCAGTTACACAAGCATGGCCCTGGCCTAAAAATATAATTGTATAGTTAAAGAAATTTAATTTTATATGGCAAAGTCTCTTATCTTCAAAACTATATATATAAGGCACCAACCTTTCTGCCATTATACCTTAATATTTCAAATACACAACATAGCAACTACTCTCTCAAATTTAAACCACACACCTCTTTCTCTACCATATACTAAAACTCTCTCTCTCTTTCTCTTTCTCTTTCTC

## >AdNAC49

CAAAACGAAAAATAGACCTCCTGACATAATTTCATCACTACACAATTTTATATTAAAAAAATATAGGTAGACAATAAAAGTATTAAATAATATAAATAATAAATATATTGGATGTTCATTTTATTAAGTGTGCGGATGATTATTCTAATATTAAAATTTAGATAGGTAATTTAGATGTGTCATGTATTTTGATTTAATTGGTGGTTATTTATATTGTTCAAAAAAATTATTGGTTACCTAACATATTCGGTTTTTTTCAATTCAAATCGATTTAAATATGTTCATTTAATTTAATTTAAATCAAAAACTAATTAAAAATGTATTAATTTATATTTGGTTGTATTTTATTTTTAACAAATTATGTATATTGGATCAAATTTTGGATTTATTTTTTAAAATTAATTTAATCCAATTCAAATTGTACTGCTATTATTAAAATATTATCAAAGTTATTAGTAGTTATTAAGAGTTAAGTTAGTAATGACCAGTAGTAATTAGTAAATAGAATATTAATAAATTAGTTGGTAATATTTTTATAAATAAAATGATTATTGTATCATGTAACAACAACTTCAACACAATAATATCACATATAATATTATATTTTTATTCTCCTCTTCTTCAAAAAATTTAACATAGTATCAGAGTTATAGTATCCTCCTTAGAGAAAATAGAATATCATTTTTTTGATAAAATCATTGTGTTTTCTTTCACAAAATTCTTTTTCCTATGTATTGTTGTTCTTTTTTTTTTCTTTTGACACCTGTTTTTGATGTTTTCTCACTTTAATAATAAGTTCATATTTTTCATTTCATCTTTTTGAATTCAATGGGTCAAAAATCACTGGCCCAACGCTCTTCTACACGCTCTTTGAAGATCATTGCTTGCTTTCTCTCCCTCGCGCAACATCTCCTTCTCTTTCTTCTTCCTCTGCTCACGAAGGCAGCCCTAGCTCCGCTGCCACTATCATCTTTTTCCTTTTCTTTCTTTACATTATCGCGCCGCATGTATCGTCTCTTCTGCCTCATCGCACGCCACTTACGTCTTCCCCTAACTAAAGCAGAAGTCCTGATCCTCCTTTCCGTCATGACTAGAATTGTGGCCCACGCCGCGCCTCTCTCTTCCTTTGTATTCCTCTGCTTCTCGCGCCACACGTCTCTTCTTCTGCGTTACAGCGCCGCACGCCTCTTCTTCAATTTCATCGAAACTTATCCAAGTTGAAAATTATTGACATCTTCCATAAAAAATGTAATAATGTAAGAAAAATGTTTTAGAATAGAAAAGAATAAGAGAGATTTTGACAAGAATTAAAGGAGAGAGATAATTTTATTGAAAAAATTTTTAAAAAGAAAAGATACAATTACTAATTTTTTTATTGTCAATTACATTTCTATTAAAATTAGTAGTATAAAAAAATTTAATTTAATATTATCAAGAAAAAATAAAATATATATATAAGGACTAATTTGATTAATTTTTAAAATTTTAGGAATGAAAATTACTTACGTCTGAACTTTCAAGGATTATTTTGATCATAAATAACTTTTTTACATGTCAAGTGCCACGTGGCATGCCACGGGTCACTAATCTGACACGTAGCATGCCAGGTGTCACTGATCTGACACGTCAACCAATCATCTTGTGACACGTGGTATCAGCCCTCTATATCATTATATCCCACGTGGCACTTAACGTGACACGTCATCATCCAACTGACGGAAAGACTAACGTGACCAATCGTATATCTTTTGGGAACGATTTTGATTAACTTTATCTTCCGAAGACTAAAATAAATATCGAAATATCTTTAAGGGACGATTTTGACTATTTACTCAATTTAAATTACACGGCGAACACGCCTAGTTTTCTGCAAAGCGAGATAGTAGGAGACTATATATATGTTCACACCAAACTATAGCTAATTAAGATATGTTTGAACCACGTTTAAAACTTGTAACTCCTTTAGAAGATGATGATCCAAAAGAATGCTACTGCATAGTGCCTAGTGGGTAACTCAATTTTGTACATTATTATTTTGCTTTGCTTTGATGCGGTAGTCCCCACTCACTCCACACGTAGAAATAAAAAGGAGGGAGAAATACTTTCGTCATTGCTGAGTTCATACATTCATTTACACAACACAGCACCGCTTATTATTGTATATTTAAAACTTTGTTCTTTTAAGATTATTTCGTGGACTACAATGATTTTGTTGTTATTGGTTCGGCCAAATCATTCTCAATATAACAACTTCAGAGATCCAAGGATTCAGAAAATGATCCGCTGACTGCTGCATGCACAATAATATTCCAGAGTGGGTGGAACTGGAACTAGTTGAATTGAATGGAAATGAAACCCAATATGGATAAAGACGTGCTTTGTTTAGATTCAAAGAATCAGAGATAATCAATCACGTGGCCAATAACCGTTGATTCCAATTATTCTTAAATTTTATGATTAAAATTAAACGTCAAAATTATTAAATAAAACACAAATTAAA

## >AdNAC50

ATTATATTTTTGTAAGTGATAAAAAATTTTTATATTTAACAAACAATTTATTCATTTGATTTAAATTTTTGTGATAATATTTAAATAAATATTTAGAAAGTAAAAAAAAATCAGAGCAAAATTTAATCTCAAATTCTTTTTTTATTTTTAAAAAATATGATAATTCAAAATATTTTTTAAAAAAATTCATTTAACATAAAATTATCAAATTTAAAAATAAAATATTTTATTTTTCTAATAATAATTACAAAAATTTGTATCAAAATATAATCTCTAAAATTTTTTTTTAGTATATATATTTGATATCTAGTTTTTTTGTTGTATTTTAAAATCTTTCGAGAACTTATTTATTATTAGCATTTTTGGGGATTCTTTTTGTCAGCGATAAAAACTTTCGAGTATCATTTTGCTAATTTATTCTATAGCACTACTATAAGAGAAATGTTAGGTGCATGAGAATTTATTATTTTTAAAGGATAAAGTATTAAATTGGTCTCCTATATTTGGGCATAATCCTGTTTTAGTCCTTAAGATTTAAAGTATCTTATTTGAATAAAAAAAATCATTTAGCTTCAATGTAGTCTCATCGTGAGGTCAAAGTTAAATAATTAACAAAATATCCTGCATGACAGTTGTACAATAACAAGGTCGATAATTTGGGCTGCGTTTGTTTCTGAGAGCAGGACAAGACAATACACTGAGAACAAGACACAAAGGACAGAGACACAAAATTTTGTGTTCTTGTATCTTGTTTGGTGATAAACTAAAATAAATTATGAAAATCAAATTTATTCTCGTTTTTTTTCATTCAAAAAATTTGAGACGAAAACTATAATAATAAAAAATATAATTATAAAAAATTAACAAGGATAATGAAAGAAAAAATGAAAAATAAGTTGTGTCCTTTGTTAGTGTCTCCTTGTCCTTCCTGTTAGGATGGACACAAAATACACTAATTCAGTATCTCTGGACACATTGTCTCTGCCTCCTCTGTCAAACACAATTTTGTGTCTCTATGTCCCTGTTTTAGTATCTGTCTCTATAAATAAACGCAGCCCTGGAGAACAAATACGAGCTCCAAAAACACAAAATTAACCGTGGATGCATCAATATATTGATTTATCATTCTCCTTAATTCTATAGAAAATATTTCATTTAAATTGTAAGAAGAATGATAAATAAATGTATTGATGTATGGTTGATTTTGTGCATCTGAAGTTTGTATTTGTTCTCAATATTATCAACCTTGTTCTTGTACTGTTGTCATGTAGGACATTCTATTAATTATTTAACTTTAACTTTACGGTGAGACTATATTAAAGTTAAATGAATTTTTTTTGGATTCAAATAAGACATTTTAAATTTTGGTTTTCTAAATTGAGAAGAGAGAGGCGACTGTGTTAGGAGATGGATTATGTTCACATTTCTGATTCAGATATCAGGATCTTGGACCTTTGGTCATCTGGACAGTGGAACCTTGAGAATATATATTCTCCTCTGAATCAGTCTCTGCAGAGCAACATTAACTCTTACAACCCGAATGTTCAAGCTGGTTTAGAGGTCGGTTGGTGTTGGACTGGTGCGGCTTCAAAGGTTTATGATGCTCGTAGTGGTTATTTATGGCTCAGTAAGATGTTTACTTGGGACGATAGAGGTAATTAGCTTTGGCTTTAGCGTCAACATGTTTCAGAAAAGTACAAATTTTTGGCCTGGCTATGTCTTCGGGAGGCTCTTTCTACTGCTGCATTTTATTTTAGGAGGCGCATTTCGAACATGGATAGTTGTCCACGATGTTTCTCAGGTCAGGAATCGGTTTTATATTGTATTCGGGATTGTCCAAAAACCCAATTAGTTTGGCAAACTTTAGGGATTTCCGATCAACCAGTGAATTTAATGAGTTGGTTCTTACATAATAGCAAACAGCGTCCCTTTAGATTCTTTTCTGGTCTCTGGTGAATTTGGCGTTCGAGGAACAACGAGATCTTTCATCCTCACGAGCATTGGATCATAAATAAAGTGATTGGTATGACTTTGTCCTTAAAAAAAAATTTCAAAATATTTTTGAGTTGTAACTACTGTCTATCTCATCCATTATTAGTGGCTCTTAAATTCTCCTATCAATAAATACTTTTAAGATTAACAGAAGAAAGAGAATAAATCATAATAGAAGTCCACAGTGCTATAAGCCAATAAATCATAATAGAAGAGAGAGAGAATAAAATTAAAAGCAGATAAAAGGTCCCTTAGAAAGTCAAACAAGGAATTAAGGATATTGCTGGGTCATAATTGGAGTTATGATCCTTCCACTTTTGCCTCTTTCTCTCTTTGATAGCACTGCATGTCTTGTATCTATAAATTAACCCACACCCACTTTTCTCTGTCCTCACACATAAAACAACACTGCTCTTTGTCTCATCCTTCAAATCCCTCTATCTTCTTCTTCCAAAGGGGAGTTCCACTCAAACCCCCCTTCTTCTTCTTCTTCTTCTTCTTCTTTGAG

## >AdNAC51

CTCCTCTAATCTTCATCAATCGCCAATCTCTTGGTCACTTGATTTCGATTAGAGGGTTAAGTTCAAATCTAGTTTATGGCCACGAAAACCCTAATTACCAAAAGCTAAATGGATTATATGTCACATATCCAGATTAGTTCAAGTAATTAGCAATTTAGGAGGAATTTAATTTCAAGCTGTGTATGGGTGAGAGACCTCTCCCAAGGGTCACAAGAACGCATCTAGAATAAGGGTCATACTCTCGTTCCACTCAAATTTATAAAATTAAGAACAAAAGTAATCCAAGAAATTAAAATCAGTACATTAATTAAAATAGAAGACTAATGATCTTAATCCATAGAAATAAACAAAACTCCTAACATTAACCAAGGCAATTTAGTGGCTCATGACTTACAAAGAAAATAAGCATTCTGAAAAGTATGAAAGTGCAGAAGTGAGAAGATCCCTTCAAAGGTGAATCTTTTTCCTTCTATATCTAACCTAATTTAATTTGAAAATAAAATAAAATAAGAATTCCTATAACTAAAAGATACTGTTTGCAAATAAAAATTACAAAAATAAAAGATAAGATAACTAATAAAAGCTAAATCTACTAGAGATAGTCCAAAAGTGTGGGTTTCAATTCGGACCGCCTGGAACTAAACGCTAGTTGGTCGTTTAGCACCCATAGAAGGCAGAGGCATTGCTGTTTTGTGGGGCTTGCTGGCGCTAAATGCCAGTTGGGCGTTTAGCATCCAGAATGGCAGATCCCAATCTTCTCCTTTTTGCACAAAACTATGCCAACTTGTTCCGAATTTTACCTGAAATAATAGAAACATCAGAAAAACTCAAAGTAGCATCCAAAGAGAATTTTAAAAGTAAAATATAATAAAACTTACTAAATTTTAACTAAAAACGACTAAAATACAAAGAAAAAAGGGTACAAGATGCTCACGCATCAGGCACTATTTTGATGATGGCTTTTCAAACACTTAAAACGGCTTTGTTTTATAGCTGACGTGTATTCCTAACGTGTCACATTAATTTTCGTTAACGGCGTTAGCAAGAGAGATGATGGAAAGACTAACGTGACTAGCTGAAAATCTTTCAAGAATGCATTATTTGATTAAAAAAAATCTTTCAAAAACTAAATTGGAGATCGCGTTAATTTTTAGGGACCATTTTGATTATTTACTCATAAAAGAAATTTAAAAATAGGTTTGAAAATATATAAAATTTTTACACATTAATAATATTTCATTAAAATTAAATTAACATTTATTTTTTTTTTTTGTTATGCACGTGACATATACAATATTCACGTTTAATTTATAAATGAAAATTCAAAATTATCATGATAAGTCAATATAACTCAACCCAGCCTCTTTTGAATGTATTTGAAAACGCCTGTAAAATAACCTTTTGCTTTTTGTGTTCCTTTTCTAGCAAAAGTTGTTTTTCTTTCATTAAAATAAAATATTTTGTTTGAAAAAATTATGAAAATTGATTTAATAAAATTTTAAAAGCGAAAATATAAGTATAATAAATATTTTGATAGCGTTTATTAAAATATCTCTTTTTAAAAATATTTTATTTAACATTTTAAATTTTAAATTTACTCACTATATAGAGACTTAATTTAATTTTTATCTTTTAATTTTTGTTTCGTAGTCTTAGTCTTTCAATTTCAGCCTCCCTTCTAAATTGAAGTTAATTTACAGTTATAATCAGAAGTTAATAATTATTTGTGAATTACTATATATGATTTTATCAGATTATTACTTTCCATGATCAAATAATAAATTATATATATAAATAATTATTGCATAACTTTATCTTAATATACTTATAAAAGTGTGGGAAAAATCATGCATATATATTCCTAATAAAACAATTTATTTTAAGAAATCACATTCTTAGCTATATCCTTCATGTAAAATGAATTCTCACAAAATTTATTACTTTATAAACACAAAGCTTTAGTTTTCCTCTTGTATGTACCTTATATAAATAAATAAATATATGCTTGGGGTAGATAGCAGCATAATTTGTCATGTTTGATAGGACAGTTGTGTATATAATTTATTATGGTTCAAGACTTCAACCCAAGAATTCGAACAACAATTATTGGTATAGTAGCTTCTACAAAGATCAGAGAGAGAAAATAGATAGAGAGATAGATAGGAAGAAAAGTATATATTTGAAAGGAATAATTCCACCCTTTTTTGTATATATGCATTAACAAAAACACACATATCTTAAGTCTCAAATGGTGACTTTAAAATAACAATATATAATACCATATATAGAACAAGTCCACCATCAGCAAAAATCATCACATATGGCCACAACAAGTCAAACCTCATGACTTGACCTTATAAGCTTCAATTCATCAAAAATCATACCAAAATAAATAATAATAAAATAATAATAACAATCTTCATCCATTCCACGACTCTCGATAATAATATAAAAGAAGCTAAGGTTCCTCTCCACTTCGTTGAAAAATTGTGCCACAAAC

## >AdNAC52

AAACTTTAAATTCTCATATTATTATGTACGCTGAAATTTTCATTATTAGTAAAGAACTAATTTTTATCTGTACTAGCCAGGATTACTAAAAAATAGATTAAAATATTTCATTGTTAATAAAGAATTTTTATTATTTTGGTTAATATTTGATGAATCTTAAATTTTAAGACCTAAATTATAATAATATAAGAATAAAATATTGATAAAAAATATTGACTAACATTAATAAAAATATTAAATTTTTAATATTTTTCTTATAAATATAACAATAAGTACAAAATATTGTGATTGTTTTCGTCTAACAAATGCACCATTTTTAATAAATGGTATAAATAATTTGTGCAGCAAATTTGATTATTGTAGAAAATATTTAAATAATAAGTAATGCTATATATTAAAGTCATTTTATGAATTAAGTTTAATTAAATTAATTAATAAAATTTAGAATAATAATATTAGTCATTTTATGTATAGTATTATTACTTAAAAAATATATGTAAAAAACTAGATATAAAATTTGAATGATAGAAAATTATTTTTTCATTTTGTAACTTTTAGTCATCATTCACCTAAAGTGAAAGTAACGGAAAAAATTAATTTAACATAAAATTACTAAATTCAAAAATAAAAAATTCTCATTCTTCAAAACATCTCGTAAAACAAAATTGTACCCAAATCTGATTATTTTTAACCTTTTTTTTAAATACATCTTTAGAGTTTTGGTATACTTTATGAAGCAAAAAAATCTTTCAGAAAATTGATGAAAAATGGGATTTATTTTATGAAAGATTGATTGTTGTGGGAGGATAAAAGACACAAGGTTGGGAAATAGAGAAAGGAGAGAGGAAAAAGGTAGAAAGAAGGGCAGGGAAAGGGAAGGAGAAAAGAAACAAAAAGAAAAGAAAAAGGAATGTTAAGTGGGTGGTATAGGCAATGACGTCACCTACTGGGGGGACAACCATCAACCCATGCGCATTTCAGCGGTCCTTCTTTCTTCTTTCTTTTATTTATATTCTCTATTCTCTCACTCCTAGCTGAATCTCTCTTTCTTCCTCTTCCAATTCCATCTCCTTCTTCTTCAACCCTACTTACATACATTCAGGTGAGCTTATTATATTCTTACTATTATTCTTCTATCTGCATCTATTTCTAATTAACTATTCATATGGCGCCATCTTATCTTAGCTTCCATGCATAGATACATGTTCCCACGCTACACCATGAATGTAGTATATATCATACGTCATATATCAAGACATAATACAATTGCAGCTACAAAACTACCACTAGTACGTAGTTTTCATCGTACGATCATGAATGGATCCGATTATCTTTCTTTCCAGAAACTTCCTTCTTCGCTCCAATTTCACCTAATAACTAATAGAAATAGAAGTACTGGAAATTAGAAAGTAGAAGAAAGAGTGAAGGTGCAAAACATAGGGCACAGGCACATACATACATTCCCCTTAAAGTTGTGTATTTTTGGGGTGAGCACCACACCACATTCTCAAAGTCAGATATGTGCCATGAAAATTCTCGACAAGCAGACAATAAAGTCAGAGTCCCCAGTTTCCCCATTCAGAAGACTCTCTACTCTTCATTCTTTAATTTCTCTCCTTCAAACGTCTTTTTCTTCTTTATATTATCTCCTCCTACAAACTACGGCAGGCAGGCAGGCCTTGATCCAATACAACGGTAAACGCCCGCGCGTGCCCCCATTCCTCATAACGTCACTCATATACTATACTCTTTTCTTTAATTTAACTATACCTAGCTACTATATACTAATCACTAGTAATCAGATGCCTCTACAGCTCTATTACTATTTGTTTTTCCCTTGCTTAAGCTTCAAATTAGAACTCTTATAAATATTATTATATTGTTTCCTAAAAAAGTTGGTTTTGTTTGCTTATGGTTTAGTTTGGTTTGGTTTACCCATATTATTAGTTATTTTAATAAGGTGAAGTGATTATAAGTGAAAATAGATACATAATTATTAATAGTAATGTGGGAATGGGGAACCCCGTTCTTGAAGAATGATTCCCTCCATTCACTAACACAAGACAACATCATTAGCACAAGCCTCTACCTGTCCGTTTCTCACCCTCTCTTGCATAACACATATACTAGTAGTGCCTAAGCCCTCAACTCCTTCTTCTCATGGTTTTTATCAAAATACATTTATACCAAACCCTCATCATTTGCTTCTCTCTCAACCGGGGCCAGCAGCGAACAAAAATCATTATTGTTTTCTGAATTTATGGACTATTGTTAAATAACACGCGCTTATATTTTATTTATCCCTGTTGGTATGACTAGTGTTGCTCACCACCAAATGACCCTATCATTATCTCTCATAACACTTGTCACCTTCTATTAATTACCTCATTCCTTATTATTTTTTTAACTTTTTAGTCTCACTTCATCTATCACTATTCATTCGTCTTGCTTATTATTTATACATGATGATGAACGAGTTATGTGGTGTGTGGTGAATTTG

## >AdNAC53

TAAGATTAATATAATCATAGAAATACTAATAATACAAGAGATTTTATCACTAATATGAACATGTTATGATAATTAAAGGTTAATAAAATGATGAGAAAAATATTCTCTTTTTAACACAAAAAAATAATATTTTAATAATTAGAAGTTGTTGAACAATAATTTTAGGTTAATTACTAAGTTTGAGTTGTCCAACAATTTTAACTTGTTAAAATGATACAACAAAACCAAAATTAAAAATAAATCGTATCAGATGTGTATGAAATTATAAATTAAGAAGTCGATAAGGATTAAAGGTTAATAAAATATAATAGACATTTTTTTTTCTTGGGAAGAGTTTAATATTCTATCTGATGGAAGAACCTCCTACCAAAAACCCTAAAGGCCCCTCAGAAAGACATGGAAACGAGACCGGGTGATAAGAAAAAATGAAATTTGATTTTTTTTTTGCGTAAAAATAAAAGTTGAATATATATAAAGCAAATATATAGTTATATTACTCTAAATAATTTTATACAGTTCAGCAAAAGTTAATGATGACAGAACTTAACAGACTGCTTAACTAAACAAGGGAGTACTAAAAGAGTATAATTTTTAATTTTTTATTAAATAATAATCAGCAACTAGTATAATCAAGTAACAGTAAATGTTATATAATAACAAAAAAAAATATTTGATAGAACAAGCTAAGAATGTTGAAAATAAAATAAAAATGATTTTGAGTCACCATTTATTTTTGTTATAAGAAGTTACAGTAAATTAATATATTCATGAGTTTTAAAAATCTCATAATCTCTTCCTACCAAAATAAAAGATAACAATAAAATCATATTAGAATTTACAAAGATGAGAAAACAATAGCAAAAACTGTATGGTTAGCATAAAATGCATAACAATTATTAAAAAAAAAAAACTCAAAAAGAAAGTATGTATAATTAACTTATTGCTATTAAAGATAAAATTAAATAAAAAATACAAAAAAATTAAATTTTAAATTTTAAATTTAAAATCTTAAATTATAAATTTTAAGTATAAATTATTAATATAAATAAATAAATAATTAACATTTATTTAATTAATAACAGATTAACAGTTACCTTTACAATACTATCTACTTAGTAAAAGATATTTGAAGATAAATATATAATTATGTATCCTAACTCCTAAGAATTTCTCATCAACTAGTGATATTTCCTTATTAAAATAATAAAACAAGCAATGAATTTGATTTGCAAAATATATTTGGCTTGACAAAGAATATATACATAGATATAGAGTCCTTGAACAAAAGGATAACAACAATGGGCCATATATGGGAGATGTGCTCCAAAAATGGTCCCAAATTACTACCTCATAAATAGAAAAAAGAAATAAATCACCCAATGATATATTTCCAAATTTGCCACTGTAAATTAGTAAAAGCAAGCAAACCCAGATTTTCTGGTTTCTTTGGAAGTCCCATCTGAATCCCTTATTATCCAATACCATTTAGAAATTATTCATTTACCCTGGCGCTACTCACCAACTAAAAGAATATCTTTTCTAGAAAAATCCAAAGAAAAGAAAAGGATTTTTTTTCGAAAAAATTATTATTATTATTATTATTATTATTATTCTATTTTTTAGTCAAAACTACCACACACACAATATATATATATGTTATATTCTTTTGTTTTTTTTATAAAAAAAACTTACTTGAACAAAAAGTTATCGATCAACATAATCAAAGTCCTTGATTCTAAATTTTAATGTTATCATAGTACTCAATATTTAAATTTTAAATTTTTTGATTCTAAATTTTAATGGTGTCATAGTACTTAATATGTAAATTTTAAATTTCTAGCTTGCATTAAAATAATAGTTTAATTTTAATATACTAATAATTTAAAACATATAATCGTCTAATTATTATTTACATGTTTAATATAAAAAATTAATTATTTTTATTTATATAGTAATATATAATTAATATATATTTTTTTAAAAAACATTAATATCAAATAATTTATTAAAATTAAATAATAAGTTTTTAATTTTAATGTTTTTTTCAAAAACAAACAAATGAGTGGGTTCAAATTCCAAAAGAAATAATAAAACAGCCACATAATAATATTTAACTTTTCTTTAAAAAAAAAAAAATCCCTGGAAGGCACGAAGTGTTGTTAAGAATTTCTTGACATTGAAGGTACCCTTTCTCTCTCTCTCTCTCTTTTGTTGCTTGACCCTCAATCAAAGACGCCCCATCTTTTCCGTTTTGCTTAGGCAAACTTCACACAGACACAGACTCTTCNNNNNNNNNNNNNNNNNNNNNNAACAGCCACACACACCCCACCACCACCGCCGCCGCCACTGCCGTCTCTACCTTCATTGACACAAGCAGGACCATCAGGAGGAGTATCAGCATCTGCACCTTCACCATCTGCTTCACCTTCTTCTCCTGCTATTGTTGCTACTACTGCTGTGGCTACTGCAGTTGCTGCTCCTCCTACCTCTCTTGCTCCTG

## >AdNAC54

GTGAACACGTAGAAGGCACGTTCCCAACCCCATTAGCTTTTATTCTTTTTTTTTTTCCTGCAAAGGAGTGGCGTCATTATTGATGATTTGCTAAAAATTCATTCGTTTATTTTTCTTAAAAGAAAAAATTATAAAGTTGTGAGGATTTTTTTTTTATTTTACAATTTTCTTAGGAAATGGAATATTTAAAGTTATTTTTTCTTCCAACAAATAAATGGAATTAAAAATGATTATCATATGCTCTTTGTTTCTCCGAAATATTTGAAGTCTCCTATGATAAGTTAATAAGTTAATAACCAATTAATTAACAAAATAAGAAACACTTCTAATCTAATCACATGCTTCCAAGTCAGACGCTAAATTAAATATGCAAGTGGAATGCTTTTTATAATTAGTAACACTAAAAGGAAGCTTTGATGATTTTCCAAATCCTTCTTTTCCTTGGGAGATTGTGGCATATGTACCAATTAATAATGGTATAATAATAATACGGTAATAATGGTAGAAAAATAAAAAAATAGTCAAAATTTGTAGCATTTATTTATTAATTATTATAATAATTAATAAATATTAAATAAAACAAATTCTGACTGTTTTCGATTAATTATTTTTTGTTATCAAATATTTTTTATAATAATAATATCTATGGATTTAAGATTTCAAAAAACTTGATTGATGACTTAATGATAATGAAATATATTTTGATAGAAAAATTTATCTCAAAAGTTTAAACTATTAAGAAGAGACAATATAATGGTTATATTTCTAACATTTTTTCTAATCTTTTTGGGTTTATTTAATGATATTATGTTATGAAACCATTCATCCAAAAAATTTAAACTGATAAAAGGAGATAACATAAATAATTATGTTTCTATTATTCTATTTTTTATTTATTATTATATAATCACTTTTTTACATTTTTTGAGTAATTTTAAACATGCATTAAAAAACAGTGAAAATAATGTCAATTTTAAAGTTAATCAGACCATTTATTTTTTGTGTGATTTTTCATATTATTCCTCATTTTCGTCATTTTATTTTTCATAGAATTTTCTATTAAAGGTAATTTGGAAATGGAAAAGTATTGGTGGCCAACCCTTACTTAAGAAATTTTAGCCAATAAATTATAAGTGCACGCATTAACATTCTTATAATCCCAGTTATTAAATGAATATAACTCATGCGCCCTTTATATCAAAATTTCACTTAAACCATTTTGCATTCATTATTTCAATTGATACTATCTTTTGTCCAATCAATCCTNNNNNNNNNNNNNNNNNNNNNNNNNNNNNNNNNNNNNNNNNNNNNNNNNNNNNNNNNNNNNNNNNNNNNNNNNNNNNNNNNNNNNNNNNNNNNNNNNNNNNNNNNNNNAATAATAATAATAATAATAATAATAATAATAATAATGATGATGATGATGATGATGATGGTGATGATGATGATGATGTACCATTGATTAGTCAATCTCCTTGTTTTCTTATTGATGGAGTATTTCCACCTCCGGTAATTATTAGCTTCACTATTTTCTTCCTTTTTATACAATTATTGTAATTATTTAAAAACCCTTTCTAAAATGAATCCAGCAATACATTTTAAAAACGATAGATGGAATAGTTATTTCAAAGGTGATAAGAAGAAAAAAATAAAAGAAATTAGATCCTTCAAAGAACTTTAAAATAATAAGGTGAAAGGAAAATTGCTACTCTTCAATCATTTGTTTTAATTTAGTTGCAAGTGGATTGCATTTTGCCTAATGATGATTCAATTTAAGGATGATGTTCCTTATTTTGACACTTTATAAATGATATAATCGTTATTTAAAATATCAAATTGTATGCTCTACTATTTTCTATTTAAATTATTCGTATGAAAAATCTAATACCTAAATTCATTCCACTTTGTTTAATTTATCTACTATACATAAGGACATGCAATATGCCAATAATATTCTATGGGTGACTTGAATGCGACCCAATCTGGATAAACACGCGGAACATCAGCGTTAGTTCAAAGTTCAAAGAATAGTACCCAAAAAACAAAAATATTATGTATTCGTACACCAGTTNNNNNNNNNNNNNNNNNNNNNNNNNNNNNNNNNNNNNNNNNNNNNNNNNNNNNNNNNNNNNNNNNNNNNNNNNNNNNNNNNNNNNNNNNNNNNNNNNNNNNNNNNNNNNNNNNNNNNNNNNNNNNNNNNNNNNNNNNNNNNNNNNNNNNNNNNNNNNNNNNNNNNNNNNNNNNNNNNNNNNNNNNNNNNNNNNNNNNNNNNNNNNNNNNNNNNNNNNNNNNNNNNNNNNNNNNNNNNNNNNNNNNAAAAATAATAAATTTTGATGATATATTTTATTTTATATGAAGATAAAGATATATATGTTTTTTAATAATTGGAGCATGCATAAATAAGAAGGCATAAGGTGACGCACATAGAAAAAGTCGTAGCAGGAACCAATGAAATCCATGCACCTAAATCAGAAACGACAAGAAACCTCAAAGATAAAAGGGG

## >AdNAC55

TGTATTTTAATTTTCATTCACTATAAACAAATGATGGCTTTTGGATTTATCTTTCATTAAAAATATTTATTTCTGTTCTGGTTTTGTCATTTAATCTCATTTGTAGGAAGAGATACTAGAAATAATATTTTTTTTCATTTGCCATTCAATCATTAGACTTTTTGTATTTATTTTTTTATGGCTATACTTTGTAAATAAATATTTTTTATATTATTATTTTTAGTACTCTTTTTTAAGTTTTGATTTTTTTAATCAATTTTTCATTTATTTTTCTTATTCATAATATGAATGTTTTTGTTTAGATTTTTTTATGTTCTTTAATAATTTTTAATACCAACGGCTCATTATTCATATATTTTTTAATTATTTTTATTAATGCAAATTCTTTTATGTAATTTTTTATTTTTTGTTTAACTTTGCATAATTTTCATTGTATTTTTGAATAAATATGTACTTTATTTACTTATTGTTATTTTTTATAATATAAATTATTAATTTTATTAAAATATAAAATTAAAACAAAAAATACCAAAGAAGAATATTGAAAAATGATGACAAAAAGGAAATACTAAAGAAGAGTACTAGTCGATATAGAATTATAAATATAAAGTATTTTTTATATAGTCATTCTTATTTTTTTATAAATATAAAATATACTAAAGTAGAAAAGACTTTAGTATAGTCATCCATATATTTTATATTCTTCATAACTCTCAATAATAATAGATATAGAAAATTGTCAATTGAAAAGAAAAAAAAGGTTGATTGAAAGAAAAAGGTTAAATTATACGGTTGATTTCTATATTTTTAGTAAAATTATAAATTTGTCCCTTACTTTAAAAAGTTTATAATTGGATTTCTAAAGAGAATTAAAATTTATAATTGATTTAATCGAATATTATCAGAATATGCTAAAAATACTTTGTTAACATAGAAAATAGGTTGAAAAAATTGTGTTAAATCAAACACTTCTTGAAAGATGAAAACTAAATTATAAATTTTAATTTTTTTAAAGACCAATTTACAATTTCACTAAAAATGTAAGAAGTAACTGTGTAATTTAACTAAAAGAAAATGACCATGTTAATTAATTGACTTTTTAAGTAATATAAAATAACGATTCAACTAAGAGATAAAATTTGATAAAAACAAAATCAGTGCAACTAAATATTTATTAAAAATTATCCAAAATCAAATTCAATAACTCAAAACATCGTTAATTTAGTAGGAACCATGATATCCAAGCATGTTAACTGCACCTCCAATTAAATTGAATAGAATAAGCTAAGATATTATACTTGGTTTTCGCTTAGAGTTTTTGTGTGCATGGAACGTTATGTAAAAAGTTTGTATTATTAGTTAGTGAAGAGAAATGCATTATTATTAATTAAAAAAAATATTTCCTGCGAAGTGGTCATCCACGCATGCACTCAAACGAACATAATGCAGTGCTCCAAATTAAACATCATAATTGTAAAATAGTATTCATTTCCTAATGTAATAAATTGATAAAGTAGGAGAGACACTAATAGAATCTGAATCTACAATCTGACGTGCGAAAGAACAATTGATAACAAGTAGAAGAAGATGAAAGACTCAAGAGTGAAAGAAAAGAACAAAAAGAGAGAGAACTAGTGATATATGAATTTTGAATTATTCACATAAAATAAAGACACTCACATTATCAGTGTTTTATGTACAATGATTAAATAGTTAGATAAAATAACAAACCAACTAAATACTAGGTTAGCTCACTGTAAGGAAAAAGAATTGCTGCTACATTTTTTTCTTGCGACGCTTTTAGAGTGTGGACAAAAGGGAGTCAATAACCACGCTTTTATAAAGGTGAGATTGATTAGAGATTTAGTTATATCTTTCTGTGCCATGCTTTAAAAGTGTGGCCAAAAAAGGTTAATCACCACAATTTTATGAGGAGGACGATTGATTAATGATTTAATCACACTTTTTTTGCCACTCTTCAAAAATGTGGCTATAAAAAAATAAACAAGCATGACTAATATGTTTTTCTACTGTCACAATTTTAAAACGTGCCCATATCTTTTAATTATTTGCCACTCTAAAAAAAACTAGCAATAGAGTTTCCTAACTCTAGGTATAACAAACTAAGCATAACAAATCATTAGCGAACTGTACAAGAGCTGAATTTGAATTTGAATTTTATTCATGTTCATGTTTATCATATTTTGTTACAATGATGAGCAATATTTTAAGTCAAAATTATGTGTATTATGAAAACTGAAAACAAGAAAGAAGGGACAAATATTATATTAGATAATTTATGAAAGAGAATTTTTTTGGTGACTCGGTTATAAAAGAGATGATCGTCTTTTTCTTTACTATTAACTATTAACATGCAATAAAAATTTGATTAAAATATGATGTATTTGAAATTCTACAAAAAGATAAAAAATAAGTTCCAAGGTGATGTGAACAAGACAAATTTAGATGGATTAAGATAAGGAATAAAAAAAATAGGATG

## >AdNAC56

TGATAGTATTAATCTACCTTTAATTTTTTATGTATATCAAATGTGAATAATAATAATAAATTTTGTTTTGGCTAAAACGATAAAAATACATTATATTAAAAACCGAAAAATCATGAATTATAAATGATAATGCATATTTTTTTTAAAAAAAATCTACTATCTCTCTTTGATATCTCAATTATATAGTAGATATATATAGACATAAATATAAATACATGCATGGATAAATGAAGAAAAAGAGTAATTTAGAAGGCTATTTTATTGTGTAGGCATTGTTGAATTTGTAGGTCGGACAGAAAAGAAGGAGATAGATGAGTAGGTTAATGGAATGAAACGAAACAACAGATGTTGAAATCTTAATTATTTTGCATCAACACACATTCATCTCACATGTGCTCTTTAATTTAACACAATTAGCTTACGTTGATCTTGTATTTTTTTATTGACTTTGTTTTGTGTATATATACATATGGTTAGAAATATAACTATTTAATTTGTTTTTTTTATTAGTTTAAATTTTTGAGATGAATAATTTTATAATATCGTATAAAAATTTTAAGTCATCAAAAAAATAAAAAATTTAATTTTTAGTGAGTCCTAAAAATAAAAAAAGAAAAGACAAATTAAAAAAAATAAATATACAAAAATTAAATAAACTCAAAAAAGAGACTTACTTTAAAAAAATGTTAAGAATATGACTACTTATATTGACTTTTTCTATTAAGTGATTTCGTGACACCTATAATTCAGTTTTCAATTGGCAAATTAAAAATGTTAGAATAATTAATTATTGGGACTTATTTGCTTTCTTCTAGAAATCCTAGGCTATACACTATAGTAGTGAGGAAACAAGAAGATATATATGCAAGATTCACAAAGTTCTTGTTAGGATTGACATTTATGAATGATACTTTGAAGATTAATTGATGAATTAATTAACACTTGAATAATTATTATGAGAGTTATATATAAAGGGTTCCTAACAGAAAGGTTTACCCTCATGTATATACGGTCTTCTTGGTTGGTCACCATTTTAACCACCAGTAATAACATTATTAGCTCTCCAACACTCCTTAGTTATATTGAAATTAAATTCACATTTTTTATACTAAAAATATTATTAGCATATATAATTATATATAAACAATGCTATACGACAGTAAATATTAATAAATTTTATTTATTAAAAATAATTTAATATTTATAATAATCAAATAATAATTAAAAATACTAAAAATTATTGCCTTCCAAAAATTTCTCATTATATATACAACACTATAAAATGTATTGTTATTTATTAACTATATATGGTTAGTTTTATAGATAAATGTATGTGATTGAATAATGGTAGTATTAAACTTTTTTCCTACTAATAATCAATAACAAATCATAACATAAATATATATTTTTTCACTACAAACTATGAAATTGCTACATGCATATAATTTATTATATTATTTAAAGACAATGTTACCTGCTACATGACTAGAAAATGTACTGCAAAAGAACTAAGCTGTATGTGATGGTAGAAGTCTGATCAGTTAAATGATTATATATAATTTAATTACGAATTAATTTGAAATATACAGAATTATTTGAGGGAGAGAAAAAGACGGCAGTGGCGTAGATAATATATAATTAATAGATTAATACAAGTGTGAGTAATTAAAAATGAATTTAAGAGAATATGGTTACAATAAGTCATGTTCTCATTGGACTAACGTGAAAGGTTTTGGTGGGTAGAAATGGGTGAGGTGGTTGGACAGGATACATCTTATTAGAAGAAGAAAACATGCAAAATAATAATAATAATAAAAATAAAAATAAAACTAGTATGGTTTAACATAGACTCAACCCCCTCGTGCAAACATAATATTAATTACTTTTCAAATTAATTAGTTTAAAAAATAATAAAAAATTTAGTGTGTTGTTTTACACCATCGTATAATTATATCATTTTTTTTAAATTATTATTCACATAATTAATATAAAAATAGTTATTTTCATTGACATGATGATATATAATTAAATACATACGTAAAATTACTATAAACTGAAAATATATTAAAATTAAATTTAAATAAAAATCATATTAAATATATATTTATTATTGAAGAATTTAGCTCTTTTGAAATAAAAAATTAATAAAATAATAAAATAAAAGATTAATTATCATTAAATTTATAATTTTTATCGTATATAAATTTTATTATTTTAATTCAAAAATAATTAAATACTTATTTTTTCATTTTATTAATCTCTTCACTTTTCATAAGCCTGATTCATTTTGAATATGACACATAACACATCACATTGATATAGTCAAGGTCGCTGGTTATATATACTTCATTTTGTCTAGTAAAAATACCAAAACCCTCGTGGCAATTTGGTCAATTCCAAATAATTAATTAATTAACCTCATCATATAATTAATATATTATATATTTATTTAATTTAAATGTATAGTTTTGTTGATGTAGGGTACCATATTTGTCAAAGG

## >AdNAC57

TGTTGTCACAGCAGTGAACAGCCCTGCTTCCAAAAGGCACACCTTTTCTAAAAAGACTCGAATTGTCCTGAAATTTGGACATAAAATACTATACATCCTAATATTTCCCCTCTTTTTAGTTGCACCTCCAAAGATTTTCAGAGATGTGATTTTGAAGTTGTTATTCCAGAATTCGGACAGCATTATTTCTGCAGAAAATGACCATTTTCTAAAAATCATATCTCCCAAACCACACATCAAAAAATTCTAAAATTTTAGGGGAACAATTAGACATCTCAAGGTTTCATAGAAAAATAATTTCACTCATTTTGAGTGGCTAAATTGCTCCCAGTTTTGTTCACAAAATGCTGCCCAAAACTGCATAATTCTGCAAGACTGCAACATGTAACCTCGGATTTTGAGAGGTCAAAAATTGATTCCTAACTTTTCACTCACTTTAACCTTGCATTCTAAATTTCTTTTACCTATTATGACTTGTTAAAAAGATTAAATCAACCCCAAATACTCAGAATTAATAAATCACTATTTTTTGTCACTTTCACATTTAAGCATACTCATATAAAATAAAAATTTTTTGCATTACTCAACATAGCAATCCAGACACAAATTACTCAAACAATCCACATATATTATCATCAATTTATTATTGCATCATATATAACAAACAGTCCAGTATCATCTCAAATAATCATTAGTATGTATCATCAATTAATTCAATCATAGAACAACCGCTGCAACATTATACACAATCGTTACCGATCGATTTGTCATACTTCACATCATTAGGGGAATTCATTTTTTTGCATCCAATAGCAGTTGCAGCATGAATTGAACCTAACATAAACTTCTCTCACACACGTCAAAAACAATGAACCAACGTAAAGGAAACGGAAAAGTAGCATTCCAAAAAAAAAAAAACGTGCTTACATTATTGCTTCCTACGACAATCTATCTAAGCCAACTCAAATTAAAAAAGAATCCACAATTACAAAAATCAGAAAATAGATAGAAAACCTCTACAATTTTCAGTTGAAGAAGCAAAACATCTATTCAAAGGAATTACCTAATTTGGAAGAAACGACGAAAATGACGAAAAAATTCACCTTCGTCTTGAGAAGGAAAAGATGTAGTTGTGATGGACCTGTGACGACAATAATAACACTGTGGTAGCGTTGTGAACAACGACGATGAGACTTTGATGACGCTAGTGACACTAAAAATAAAGAAAAACAAGAGATGAATTTTAAGATTTGAAAAAAAGTGAAATAAAAAATTTTAATTTCTCTATTTAGTAAAGAGCAAATATGTAATTGTGTCATTATTTATTTTTTTATCTATTTCATTTTATGGTAGAAAAAAAATTAACTGTACATCATTACACTTTTCATTCCCATGCAATCTGATTTCTTAATTAATAAAAAAAATAAAATTAATGGCGTGTTGGTTAAGGCATAAGGCATTAAAAAAATATATAAGTAAACTGAGAAATTGTTTTACTGGTTTAATTTGTTTTACTAGAAAATATGAAATGGGAACCGCAAAAGAATTTTCATGCTAGAGCTAGAGTCGGTGAGAAAACCGACCAAAATTTTGATGTGTGAAATTATATTATTAACTTTTATGTATTATTATGTAATAAGTTTAATTATAGATATTTTATATATGAAATAAATATTTTTATATATTTTATTTAAATATAAAACATGATCTAATTTATTAAAAAATATATTTAATTAAAAAATATAAAAATAGTTTTTAAAAATATTTAATATTTGTTGACTTTACTTTTAAAACACTAACACATTGTTTAGGTTAAAGAAATTTGAAGGAAAAAAAATAGATGAAAAAATAAATTTATTTTTTGTTGTTTGGATGAGAAGAGAAAATAAAATGAAAAAAAAAATATGTGGAGTCCACTAAAATATTTTCATCTCAAAGATGAGATAAAAATGAAGAAAAATATTGTAAACATAAATAAAATTATACATTTATCCTTTATCATTAATAAATTATAATTTATATATAATATAGATAAAAGTATTAATGTAATTTTATACTATTATAATTTTTTTTTTATTTTTCTTTTCATTTAAACAAATTTTTTTTTTATTTTTTTATTTATTTTTTCTTCATCCAAATATCGTACAAATAACTTTATTTTTCATTTTATTTTTTTTTCTCATTTTTTTCTTAATTTCTTTCTTTCTATTTTCTTTCCTCCGTAGAATTTGTCTTGATAGAATTGAAATGAAGGAGAAAAAAGAGAGTTGAGTTTGTATAAATACTCTTACTCTTTTCACTTCACTTCCCATCTCTCTACCTCTTTCCTTCTTCTTTCCTTTATTTTTTTTTTAATAATTAAAAAAATATTCTCCTTTCCTTGTTCTTTTTTCTTGCATCAATTGTTCTTCTTACTTGTTCCTTCTTTTTTTTCTTTTTGCAAGTTAGTTCTTGTTTTTGCTTCCATGCCATTAAGCTTGTTGTAATAGATTTCTTAACA

## >AdNAC58

TAGCATTGCTATGCCTTCCCCTCTCTATATAGTAGAATGAAAATAGCCAATAAGAATCGCCACAAATTCTGGAGACCCCATATTTATTTCCTAAAAGACATAAATTTAAAATTTTCTATCATTATGAAGATCTTTTTATTAAAAAAAAAAACTAAATAATAGATATATAGTTATTCATATATCTTAATAAAACTAATTTTCAAATGTTTAAGTTGATGCTTAAAGATTCTCTGAAGTTAAAAAATTAGTAAAATATTAAAGTGAAGAGTTAATTATTCATAAATTAATAAGATAATGAGATATAAATTTTATAAAAGTTATAAATTTAATAATAATTAATTTCTCACATTAGCATTTTTTTATTTTTTTTATTTTAAAGAATCTAAATTTTAAGTCGATAAAAAAGAAACTTATTTTTAATACGTTCTTTTATGCAAGAACATTTTTAGAAAAATTTGTATAGATTTTTTTATATGTGTGATGCTAAAATTTTTATTTAGAAATCAATAAGAATCAAACATAGATAATTTGGTTATAAAAATTATGAATGATACCTGTCATGAAATCGTTCATTTTAAAAACTTAAACGATAGGAAAAAATAATACTTTCTTTTAAGTAAGAATCTCTCTTAAATTTGTTTAATTTTTTACATATATTTAACTTTTGACCTTTCGAACATAGAATTATAATACTATGTTATGAAATCACTCGTTTCAAAAGTTTAAACTGATAGAAAAATATAATATTAATGATCATATCTCTAATAATATTATATGTTATAAAATTATTATTTTTTAAAATTTAAATTAAATATATGAAATCATTTGATTACTAGGTATCTTTAATTTTTTTAATGCCTATGACTACCTATTTTGATAACTATTAGTGTTTAAATTTAAATTCTTCAAAAATAAACAAAATTTACACCAAAACTTGATGTGTGAAGGAAAATTAAAGCAAGAATGAAAAGAATAGAAAGATAGAGAGATAGACAGGTTAATTTTGGAGATGGTCCATGGTTGTTTGAGGGGTGATTTTCCATGGAGATTTTAAGGTCAGAAGTCGTAGTTGTGGATGTGGGCGTGCGCCCTTACTCTTCTGTGAGTTCCCTATAACGTCCATTCCTTGTGGGACCCTTAAGTGATACCCACATACACATGGGTCCCACTTTCAAAAAAATTTTGCTCCCTTTTTGAAAATTTTTTTCTCAAACACATACTACAGTAAACTCTCTTTCTCCATCTTTCTTGGTAGGTGGCACTTCTTCCAGCATAGCCATATAGCAATTAGCAAAACTAACCCCACATCATATTCATTTTTCTATTTCAATTTCTTTGTATCATTATTTATTATTATTATTATTATTATAAGCCCACATATACCCAATTGGTTATTGGTGTCTGGTGTCCCCCAGCTTTAGCTATGCGACAAATATTATGGAGTACTGTTGCTATTTGTTATGTCAATATATTCTCATTGTATTTTAGATGTTAACTAAATTAAATAGTAATAGTATGGATATATAGACATATACAATATCTTATAATTTTAATTCTCGTGTTATTTTTAAATTATTAATTAACAATGATTATATTACTGTTGGTATGAAAAAAATAATATTATATAATNNNNNNNNNNNNNNNNNNNNNNNNNNNNNNNNNNNNNNNNNNNNNNNNNNNNNNNNNNNNNNNNNNNNNNNNNNNNNNNNNNNNNNNNNNNNNNNNNNNNNNNNNNNNNNNNNNNNNNNNNNNNNNNNNNNNNNNNNNNNNNNNNNNNNNNNNNNNNNNNNNNNNNNNNNNNNNNNNNNNNNNNNNNNNNNNNNNNNNNNNNNGAGAGAGAGCTTAGCATTATTATTGTTATCATAATTATCATTATTATTGTTTTCATAATTATGATAATATCTTGCATTTATATTGACACACATTACAAATGTTGAGATCAGAGTATGAGTATGCATCTGTAAGTGAGAGCATTTGATAAAGCCAATAACATATACCAAGATAGAAAAACCAAAGGAAACTGAGAAGGGTTTAGACTTTTCCATAAAACAAATTATTATGATTTAAATTTCAGTTGAAAGGAGGTATTCCGCGTTCCCCTCCATATTTAAATTTAATAATCTGATCATATCTTTTAAACAAAGATGTATCTATTAAAATATAAAATTAAAAGAACGAAAAATATATATATACCAAGACACAAATAGTAGTCATCATTCTAGCACCATACAAGAGTTTCTTAACAAAGACATTTTTTTAATTTAAATTATTCATACTTTATTTTATAATATATATATAATACATTTTCTCTCATGTATCTTTTTGGTCCCTTGTGTCCCACAGCACATATATATAGTGTTCCTTTACATACACATTGCAGTACACACAACACACCACCCTCTCATTCATTACATCTTTTTCTTTTTTCTTCCTTATCCCTTTGAAAGTGTACCTATTTGAATGTCTTCTCTCTTCTTCTTCTTCTCCTTCTCTTTCTCTT

## >AdNAC59

ATGTTCCACTCCCAGGGGTTGCCGGAGGAATGTTGTCAGTCCCCTATGACATAGGTGGTCTTCCAATCCGCGATGGTGTAGGGCAGCCAGTTGGAATTCAGGCCTTGGCTACTGCTCTTGCAAATGCTCCTCCCGAACAGCAGAGGACTGTAAGTCCGATTTCCTGTAGCAGTGCAACTTCTTTAGCACATTTGTTTGGAATTCTTAGTTGCATGTGCACAATTCTTATGGTTATACTAATTATTTCTCATGAATGTTCCTAGATGCTTGGTGAAGCTTTGTACCCGCTAGTGGACCAGCTGGAGCATGATGCAGCTGCTAAAGTTACAGGCATGCTATTGGAGATGGATCAGCCAGAGGTATTACACCTGATTGAATCACCAGATGCTCTCAAGGCAAAAGTTGCCGAGGCAATGGATGTGTTGAGAAATGTTTCTCAACAAGCCAACACCCCTGCCGATCAACTCGCCTCACTCTCCTTGAATGAGAATCTTGTCTCTTAGATTTTTTTAATTCAGTTTACTTTAGGCAATAAACCCTACACTGGTCATCACTATCTGCTTTTGTTCCAGTTTTAAGGTCTTAGATTGGTGATTTCGGTCAGTACTAAGGAAGCTTTTCATGTTCTTTGCAGTTTTTTGTTTCTAGGATTAGCAAACTTGCTGGATTATTTAGTTTTGTTGTCTTGTTTGTGGATTTATATTTATATCAGTTGTACTGGATTGTTTGAAAGTGTTGTGTTAATTTTTGTTTCCGATTAATTGCTCCGCTCTATTTCAGTGATAACTGTGAATTTTGACAGTTCAAGGCACGAGATAGATCCAATGTAGGCGTCCTTTATTGAAAGAAAGAATTTCGTTTCCGATAAAACTTTTTTTGGGCTCATGACCTGAGTGTCATAGAAATAATGAATTAAATTGACAGAGATGCCTAATAGAGTAATAATAAAAGTGGAAAATATTTTACACAAACAAAACATGACCCCGAGGAAGAATGTCCATGGGATCAAAGTCTTGATCATTTTCCTGTGAAACATTGTGGCTTCTTAAGGCTATGTAAAACAAAGCAATGTTTCTCCGTTAATGCAATACAATACACCAGCTCGGAATTGAAATAACATGCAATAGGGAAGGGCACAGCTTAAGCAGGGTTTGTGGTCCTTTTTCAACAGAATAGGTTGATGAGCAATGCAGATTGCAAACTACTTGATGTAGTTGAAGGCCATAAAGAGAATTTAGTGTGGGTACAAATTGTAAGGTCCCACGTCGATTGGGGAGGGGTTTCGGCTATCATCCCTATCGTCAAAGGCAAAACCGTGAGGCCTTGTGTGCTATAGCGGACAATATCGTGTTAGCGGGTGATTTGGAGTGTTACACAAATGTCACTTCACTTGTTTCAAATAGATTTGATAAGCTATTTAATGTAGAAAAAATCTCTAAAATTAAGAGATTGAAAAAGTGATAAAAGTAAAAAATTAGTTACTATTAAATTTATAATTTATAATTACTAGTGAAAGATCTCACCCTTCATTGCATTCTCTCATTTATTTTTGGTTTCACATGGAAGATTACACTTTATTCTCTTCAATGACAAAAAAATGGAGAGGATCTATTTCCAGTTATACACTTATACCCTTCTCTTTTTGGGGCAAGAACTAGGCTTCATCTTGTGGACATTGGCTAAGGGAACAGGTAATTAGACTCTCCTCGGTGGTGAGTTTTCCCACGTAGGCTGTAGCATCATCCTATATTAATTTGATGTCACATTTTTATTAAATTTTACGAATTTTTTTATTATTGCCACTATAAGAGAATTTTACATACATAATTAATTATATATTAATACAGCAATAATTTAATTTGTAAACTCAAATCTTACACACATATTAAAATTCATTCAACTTTTGTACTATCAATCAAATCTCTTTTTACCCTGTAATTTTGAAGGACAAAAAATCACACGCATTAGGGGTGGTGACTGTTAATTTTCAAATTCTAGTTAATATAGAAAGGTAGAAATTGAATCTTTGGGGGACAGTTTATTCTCGAATTGGAGTTTGGACGATGTATTACTAAGTGGGACCCAGCAACAAGATTCCTGGAGTGGAATGTAGCTGATACCTTCTCTCTCCGAAAAAGGAAAAAAAATATTCATTTTTCAAAAAGGCCGCGCCCGTGTACGTCGCGGCCGCACACAGCACCATGCACCACCGCTCATCACCCCACACATCACACAACCCCACCGATCTGTCATCACAGCCAATCACAACACGCCACGTCACCTTGCCACCCCCAAAACCCACCCAAATTCTCACCGGCGGGAACACGAGTGAGAAGAAAGCTTGCGCAACACGAATCTCTCTCTTCCGCGTTTTTTCCCCTTAAACCCTTATAAAAACCCTAACCCCGATTAAAAAACCCCAAACTTTACCCACTCTCTCTTATTTATATATGATTCGATTGCTTCTTCTTCGTTTCGAGTTTCAATTCTCCGCACTGCG

## >AdNAC60

TGCTATAGCACATCGACGGCTCGGCGCCATTGGCAATCGAAACTAAGACCTAACAATTTTAAATTTAAATAGCACATGAGTAAGTAATGGGAGTGAGTAAGTATAAGATTTTGTTTGAATTGTTATAATTATTTTGTTACTTTTTCAAGAGGTTGAAGCCATTATTAAGGAAACTAAGAGTGAGGAGCAAACGCAATTTTTAGGACAGATTGAAGAGAGTTTAGGAGTGAAGTTAGTCAGAGAGTGAGGGAATGGGAGTGGTGACAGGTGCTGTGGCAGTGGAAGTGAAAGAAAAGGGAAGGAGATCGGAGGAGATAGTATATGACAAAGAAACTTGTAACAGAAAGTGGTGAAAGGTGAGAAGGAGAGGGAGAGGTGGTGGAGGGTGGAAAAAGAAGAGGGAGAGAGAGATGAAAAAGAAGATGATGATGAGGTGAAGTGAGTGATGATGCCACGTGACAGTAGTGGTGCCACATCGACAGTTTGGCTGTTGACTCATGTTAGAAATTAGTTGAGGGACTAATCTAGAGGTTTAAATTAGTGCAATTAAAATTTCAAGAATCAAATTATTGCATAAATCGAATCTAAAAGACCACTTTAGAGATTTCGGACCCGTTTGGAAAGCTTTAAAAGTAAATATTTTCAGCTTTTGACTTATAAAAAGTAGTAGTATTAATGTCTGGTGTAATTTTTAAAACCAAATTGTAATTTTCTAAGAAATTATTTAAAAACTTATAAAAAAGTTAAAAAAATGACTTCTCTTTTAATAGTACTATTTTTTATCATATTTTAATAAATAAATATTTTTAAAACTAAAAATTCAAATACAAAATAATTTATATAATTATTTATTATTTAAATTATTTTTTTATTTAATTATACAATTTAATAAAAGGCTTAATTTTTTATATACTATATTGCGGACCAACCAACCAGAGGCCGCGTGCGGTTGCTCCAACTGAAAACCAATTATTATTTTACTAAACCCTTAAATAATAAATACTATATACTATACTCCTCTTTCTTCTTTCTTGTCTTGTCTCTCACTAATTTATTTGCGCTTTTTTTTCCTCATCCTCATGCCGCAATGCACATAAATATGTCAATATAATGTAACACACCACACTCAACATATAACAAGCTGCTAGCTAGCTAATGCAACGTCCAATCACTGGAAGGCTGGTAGTATATGCTAATAATTATAATTATCAAGCCTAACTAAATTATATTAGAGAACTTTGATCAACAATAATCAAGTAATGCTTATTAGGATATAAATTAACAATATTAGGTTAATACTATATCAGAAGAAGGAGCATATATTATATATGGGATATGCTACTCAGCAACGCATACATATAAATAAAGAGTTCCCCAAAAAAAAAATGCTCAACACGGAATTTGAGACAAATTAACTTTAATGTTGATGATGAGTGTACTAGAAGTTTGTTACCTTTTTGTTTAATTGGTATGATGAATCTTCCCTTTTACAATTTCATTTATAATTACTACTAAAAAATTATATACTGCATATTTTTAGCATTTATTTAAGGATAAAGCATTATTGTGATTTGTGATCCATCTAGAAGAACTGTACATCATATATAGTACCTGTGTTGCAATTATTATAACAAATACTAAACAAAAAAGAAGGCATGCTGAACAATAAATAAATATATAAATCAAAATGTGATCTTATACACCAAATGCGATGTAAAAATGTTAAAAAACGCAAGGTTTTTTGAAATTTTGATGCCAGGGAGGATGTCCAGCATTTTTTTTATAAACTGCATGCTTATTCTTTATTTAATTGAATGTATTAAATCCTAGTTGCTGAGAATTTGTTTAATTTAGATGTTCACATAATTTAAACCCAAAACTTTGTTTAAAGTGAGATGAAATAAAATGGGTAGTAATGATGAAGAGAAATAAAACTCCATTGATGATTAGATATTGGGAAAATTTCAGCATATCATCAACTGAATTTTATTCGCTCACATGTCTGAATATATCAACACTTGGTCCCTTTTATTAGAAAAGAAAATGTGCATTCGTGGTTCAGCATTTGATGAGTTGATCTATGCTATGCTACATGGATCATATATATTCATTCTCTTTCACTCTCTCTCATTATTGTTTATTATATCATCTATAATCTCTTCATTCTCTTTCTGGGGTCCCATGCTGGCCCTCTGAAACGTTAACACCACATCATTCTTTTCTCGTCACCATCAATTGCTGGTGGGTGGAGAGAGATTCCTCTCTCTCTCTCTAGCGCCACACTTTTATGAACTGAAAGCTTTTCCAGTAATCTAGCTCCAACTCAATACATCGATAAGGAGAGAGAAAAACTTTTGAGAGGGATCATTCTTTATTCCTTAAATTTATATATTTGTTTCTTGGTGTTTTACTTCCTATTTAACCCTACCTCTTCTTCATTCTCCCATTCCCTGAACGAACCAACCTTTCTTTCTTTTTCCTTTTACTTCTCTTTTTGAAG

## >AdNAC61

AAAAAAAAATAAAAAATATCATTATGTAAAATATTATTAATGTAAAACTTAATTGTGTGAGTACACAAATCAAGTATTCTATTTTAGATCCTACAAAAAAATTCGTCAATAACAATAAGATAAATTAAGCCTAAAAACAAATGTGTGTAACACACATTTTGAAAAGTCAGGCTATTATATTCTCTAATATTTTTTGTTGTAAATTTTGTTCCATCTTTTTTATATCTTTAAAGACTTCAACAAATTCTAAGTATACCGGTCCAAATTTTCATATTTAATTACTTTTAAAATAAATATATAATATTAGATATAATAAGTATATAATTTTAATATTATATATATAAAATTATGATTTTATTAATTTGTGTTCTAAAAACATAAGTTAAATATACTATAAAAAGACATGTTTTATAAAAGTTATTGTAAAAATTAATTTTTTTTATATTTTAAATGTATTGAATATATTAAAAATATTAAAAAAATTATTATTATTTTTTAAAATATGTTCTTAAAATATAAATTAATTAAATTTATAAAATTATAAATATTAAATTAAATAAGATAACTTTAAATTATTATAACATACATGGGTATGGCTGTAGTCTTATCAAGTGCTAAATTGCCAATTGGCTAGAGTTGTACATGCATAATACATTCACTCTATGTTACAAATTAATTAGTAGCCAAATAATTGATAATTGAGAGTTTGAAACATTTTGAGCCATGCCATGCCATTAATAACCTGTGTTGCAATTAAGGATGGCAAAATTTTTTGAGATGCGGAAATTTCTGCGGGAACTGTTTCAAATGGAGATTCGACCGTGGATAATTTTTTTTGCGAAGATGGGATGGGGGACAAAATTTTTCCGAGACAAGCGTGGGGATCTGAGTGGGGATTCCCGTCTTGTCCCTGCTAGCTAATCCCCAAAATTCATGAATTTATTAAATTATCCTTAATAGTTTATTTCTCATATATGTTTTTTAGTCATTTCACACACATATATATATAGAAACCCAAAAACACCTAATCCTCATTTGTATAACCTTTCTTCAATTCAGAAATTTCTAACACTGTCTATACTCCGTCCAACCTCTCTGCAATCACTCCCTGACCACTCTCCCTTCTCACCGCATCGTCACACCTCACTGTGCCATCACCCTAACTGCATCGTGCTCTCTATTTGCGGCGTTTCTTTCCGTAACTCTTTCGTCGTGTCTATTCTCCTCTATGTTGTCGCGTCTCCGACTTCGTCCACTGCTCTGTCCTCTGATTCGCCGTTGCGTCCTCCCTCTGTGTCATTTTGAAAGAAGTCACCATCTTATCGTTTAGGTTTTTGTTTATAAAATCTTACTGTTTTATATAGAATGGATACTTGCAACTCTATTTTATATTGAAATTAATAATTAGTTAGAACTATCAGATAGATGTGGAATGGGATTATCACGAAAAATAGAACCCTGTGAGGAATGGAGATTAAGAGAAATATTCTTCTACGGCGGAAAATGGAGACAGGGACGGGGAATAAATCTGAAGATAGGGATGGAAAGTAGAGAGACATCTCCAGTCCCCGCCCTACTCAGTTGGCATCCCTAGTTGCAACCATTTCTCACATATTGCTCCACAGAGCATAGAACTGTTTTCTTATATCGAAATATACTTGTATACTTAAACATATTTAACTGTATTTCAAAAACTTTTATATCACCTGTTCTTAATTTTGTTATATAATGTCTAAATATTATAATGATCTCATCTTATCAAAACTGACCCACCAAAGTTCATCGTCATATGTTCTTAAATTTAGGAACTGACTATTAGCATTTATCATAAGTTAGTGCCTTCTTTACTTAGCCATTCTATCTTGATATTTGACATGGTAAAGTAAACTCTGTCCACTCCTCTGATGGACTACGGTATGATTTTTGGAATCAATATCCAGAGTAATCATAACAACTAACATGACTTCAATTATGAAATTGCAATTATATTTTCTTAATTACCATATATTCAAATTTTGCTCATGGATGAATCAAATAGGTATTTTCAAATTATCTGATCTAAATAATTTTATTAGTTAACCTATTTACACACTATAAAAATATATCGATATCCACCACAAAAATGATATTATTTTACTACGTTTTACTAAAACTTAACGGTGAGATTAGCATAGATCGGAGGACACACAAACAGAGAACAAAATGATGAATTAATTTGATTCAACAGACATTTAAGGAACTAATTTGATCAAATTTAGTCATATTACATAAGTAATTTGCATATAAATATTAGATGATTTAATAAATGAAATAAAATTGACAACCTTTGTCGCTAGAGTTTCAAAATCATGCTATGTCCTGTTAAATCAGCTTCACATTGGAGACCATTAGCTAGACTCAAATCTTTTAGCTAAATTGCAAAGTAAGCGTGCTAATTAAATTATATCAAACACCCTTGGAAGAATGTGGGTCATAGAACATGAGACAAATTGGTTGCTTT

## >AdNAC62

CAAAAAGCAATTGAACAATGTCTTCTTCAGAGGTTAGAATTTGTCCTTGGTTATAATTTTTTTTACAGTAATTGATGTATATATTGTCAACAAAATCCTAATCTGCAATATTTTTTCAACACTATATATAATTTTAGTGAAATTTTTAGTGATATTTGTACTTGATTTAACCTGAATAATTGTTTTTAGTGTCTTTTTGAATATATATGTGTAGCTGTCTCTGATTGTTTATGGTCATTTAAATTCTGTTGATTGGCTTTAAAAATGAATTATTTATGATTCTGTTTATGAGCTGTTTTGGTTTTGTTTATGAGCTGTTTATGAGCTGCTTATGTCTTAGTAAATTTGTGTTGAATTATCTCAAAATAATGTATGGCAGTTAGTGAGTATTAAATGATGCAAATAAACTAGCTAGTTGTACTATTATGTGAGTTTCTAAATGTATGTTGAGAAACAAAAGTTTTGGTGCAAGGTGTTGAAGTTTCTAAATGTACTGTTAAAATACTTTTGACATATTGAATTGGTGGAAGGTAAATTCTAGCAAGTATCCTATCTTATTTCAAATAGTTAAAGATGTCTTAGCAATGGCAGTCTCGACTGTTGCTTCAGAATCAGCTTTTAGCATTGGTGGAAGAGTGCTTAACAACTATAGGAATTCTTTAATTCCAAAGACAGTTGAGGCATTGATATGAACACAAAATTGGCTTTGTGCTTCTCCAATGACAACTGATTTTGAGGAGTTTATTGAAGAGTTTGAGAAACTTGAATTAGGTATGCAAAAAAGAAATTTGTAGTTCCTTTCATGGTTTATGTTTATATTTATAATCTATATTATGTTTATAATCTATATTGATTTTTTTGTTTTATTTTTGTAGAAATTACACCAACCGGAGAAGATGAGGATAAGTCTGGTGTGGATTCAGATTAAGCATGGTGGCTGTTTGATTTTTATTTGTTTTAAAGTGACTGTTTCGGTTTAATGTGTATTTATTTTTGTTGTTTTGCTAGACATTTTTAATTGTCTTTGTTTTATGTTTAATTAAGTTGAATTTGTATTGAATTTGGATGTGATATACTCTTATTATTCTAGTTTATTGAAGGTTTTAAACATCATTTCATAGTATTTTAAATTCAGATTATTAGGTATAAAAAACTGAAAAAACTAACTGAACCAAACCGCTGTTGTTTCGATTGTTCAAATAGCAGTCAACTAAATGGTTGGTATCATTGTAAAACCGATCTAGTCGGTTTGGTTGGTTTTCGATCCAAAATCAAACTAAACCGAACTAATTACAACCCTAGAAAAAAGTAAAACGTAAAATAATGTAGGACAATTAAAAAATTGACAATTTTGTTCATATTTATTATCTATTATCTCTATATATTACATATGTAAAAAAATTATGAAAAAAATTTAGTGCACATTTTTTTTGAAGTATTTTTTATTATATAAAATTTAATATATATATATATGCTATTTTAATTAATTGATATTTTCTATAGGAATAGTACGGATGTACCACAAAAATAATGGAAGAATTTAGCAAAGTAATGACTAAGTAGCATAATAATGTGAAAAGGGAGGGAAAGTGTAGAGAAAGAATGGCAAGAGTGAAAGAGGAATTTCCGGGTAAAAGTCCAAAACTGAGAGAAAATGAGGTCAAGGCATAAAATAAACTTATCAACACTAGCAATAGCATTAACATTAACATGTCTCTCTCTAACACCGCACAATTTTTCTCTTTCTCAATCCTACACCTCAGTTTAGTCACCAGACTTTTTGCTTCAAGCCGCGTGGGTCCACTAACTCTATCGTCCCCACCAAATAAATCTCACTACTTTCATAGTTTGCACTCACAACAATAATAAATACGATTATATTTAGTCTACATTAAAATTAATTATTCATATTAAATATATATTAAAATATAAATATACGTTAAAATAAATTAAATTATATATATTTATAATTAATTTTAATGATTAATTTTAATATATAAATAATATCTAATAAATTTAGATTCTTTTAATCATTAGTTTATTTATTCGTTTAAATAAATATAATGAATTTGAGCGTTATTATATATAATAAGTTATTGATGACAAACTTTTAAATATAAAAATATCTGATAATAAAAATTTAATAAAAATAATTAAAATTTATTTTATTTTATTTTATTTTTTTAGTATTATCACCTTAAATAAAATTTAAATATGCGATCCATTAGTTGTTAGCATATTGAATTGGAGAATTCCAAAAAAATGGGTAATCTCTTTTTTTTTTTCCCTCAAATAATATATATCAACATTCAAGTCTTTTCTTTTCTGTCCTCTTCTATGTATAGTCTACCTCATGACCAAGTCATAACCCAAATCACCATCATTAATCACAATTATTAAATAAATAAAGCCACAAATTAAATAGTTATGAGCTCCATTAACACAACCAACTCCACCTCCTTATAAATATACACACCCCTCTCAATATCAAATTCATTTA

## >AdNAC63

TATATTATAAATATGTAAGATTTATAAATTAATTCACCGTTTTTTATTATCTATTAATTTTAATTCCATAAAATTCTAAACACGTGAGTAATTTTTAGCAAATGGGTATAAAAGGTGACACAATATTATAAAATTATTTGTCTTAAAAGTTCAAATTAATAAAAAAATTACGTGAATGATTATATTATTATATCATCAAACAAAATTAATTAAGACTAACATTTTAATTGAAAATTTTATAAAGTGGTCATGCATTTATTTGAAATTAATATGGGGCTAAAAATAATATTCAATTTATACATTAATTTAGTCAAACATTTTGAATAAATATAAAATATAAAGACATTGACATTTTAAATATAATAAATTGGAAGATGATAAACATACACCAGGTTAAGTTAGTTAATTAATAGATTCCTTACACATAAAGATCCATTATTTTATTGAAGAAATTCTTGTACAAAATTAGCCGTTTTGTTGAATTTTCATAAGATATAAAGAAAATAAGGTAATTTTACAAAATAAAATAATTTTGTGGAGACAATAACTTAAAGTAATTTTATTTAACTTAATATCTATAATTTTATATATATAATAGTTATAATTACATATTATGATAGATAATATTATCTAATTATTCTAACAATAATTAAAAAATATAAAATAAATTTATTTTAAATTGTTTTTTACTATTTTTTTAGTGTCTTCTAAATATTTTTGAAAATTATACTTGATAACCCTATACTAATCACGAAATCTCACTTCATCTTTTTGAACTTTTAAATGGAAAGAAAATATCTTCAATTCAACCTCCTTGTTGAGTGAATAGTTCAGCAAATTGAAGGTCTTGTTTAATTTAGGTAAAAAGAATTAACCACGAGGATTGACTTCTCTCTCATCATTGCCAAGAGTTGCTCAAACTCTAGGTTTCAGTTTCATAATTAAGCGGTATTATATAATTCATTAACTAATTAATATTCACTAACCTATATGATTAAATAGAAGTTTCGGAGGAGCAACCTCTTTTATTTAACTGTTGGATTAATTTATACCGTCACTTCAATTATTACTCAAGCTAAACTATGGCTAAAGGTCCGGTTAAAAAGTTTTAAAAATAATTTTTTTAAGTTTTTGATTTACAAAAATAATAATATTAATGTTTGGTGTAATTTTTAAAATCAAATTGTAGTTTTTTAAAAAATTATTTAAGAGTTTATAGAAAAATTAAAAAAATAACTTTTTTTATAATATTATTACTTTTTTTTACGTTTCTATAAAATAAGTATTTTTATTAGAACTAAAAACCCAAACACAAAATAATTTATTTATAAGCTATTTTTAATATAATTATTTATTATTTGAACTATTTTTTAAAAGAAAAACTTAATTAAATTATTTACCTAAATTGAGTCTAAGTATTTTCTCTATATGTCTTTGCAATTTTTTTTTCTCTCATCTCTTATGGCATCTTTTAGTTTGACAAGATAAAAATTAATTTATTGTGATCAAAATTTTATTTAAAATTTATTATTATTTAATAAATTATTATATGTATAAGAAATTCAAACTTTCGATATTTATTTNNNNNNNNNNNNNNNNNNNNNNNNNNNNNNNNNNNNNNNNTTTAATATATAATATATTATTTTGACGAATTGAAATTGATCACCTAACTCCCATGTCCTCCTATCAGATTTCAGCACAAAATACTCTTCAGCCAATATATCCGCTTCCTTAATTTGTCTCCATGCCATTAGATATAAAAATTATTCACCATATGACCTACGAGCATAAAATTGATAAATAACGACTTACATACTATTGTTTTAAATAAAAAGGCTATTCAAGAAAATTCCTGTGGATGCCAGCTTATCAACAACCAATTTGATGAACACAACTAATTAATTTAAATAATAATAATAATAATAATAATTCTTCATATGGAGATTATTTATCACAATTTTCAGACCAGGCAGATGCACAAAAACCCATCAATTTATGACGAAGATAAATCATGAATCAGAAATAATAAATTAATAGGGAAGATAATAGATATGGGTATATAAAGCAGAAATGGCCAAAATTCAAACGAATGAGACTATGTCATCATCATCACTAGGGCTTTGTGTGCCAACTCTCTAATTATTATTATCATATAATTTGCTTAATTTTATATTTGGAAATAATAATGATAAAATGTGTTATATTATTGTTATTTTGGAAATTATTTACTTAGCATTATCTCCAGACTAAATTAATGCAATGCACAAAAATTGTTTAAAAAGATGGAAAGAAAAGAAGCAAATGATCCCTAGCTAGAATTATATAGATACATGTCAGCAAGTTGGCAGGAATTGGTTTGTCCACGTCATCATGCCCATTCTCTCCTTATAGAATTACAATATTACCCTCCATTGTTGCATTTAATAACAAGTTGACTTAGCCACTTGTGAGTTCCTTCTCCCATTTATATATACACCTTAGCACCTCTTCCTTTTCTAAT

## >AdNAC64

AGTAACAAAAATTGAAAATATTCAGTTGATTTCTTTTTCACTATTTTCTACTTGAAGAACTGGATCATGTGGGCCCTGCATTCTGGCCAGCTATTTTGTTTAGGCCTGAAAACTGAAAAGGATTCCATATAGTCTCCTTTCGGCCTTGTATATGCTTTAAATAGCCATAAAACAAAAATTTGTATTTTATCTACATCTCTATCTCTAAGTTATTGCAATTGTGTCCGAATACAAGGTTAAGGGGCAAGGCATTAAATCAATTAACGAATTTCATATAAAAATGAGGGCAAAGTCTTGATAAACATGTTACACGTTACGTGCGTATGTTGAGACTTCAAATGGAGTTCGCTTTCCAAATGAATACACCGACATTTCTTTTCTGTGATTAACAACAATTATCTCCTTTCTAAGTCGGTCTTCTGATTATATATAAAATAAAATAAAGACACATATTTCTATTCTAGTAACAACTAATCACTCATTTGGCTCCGTGGTTATCCTTTTCAATTTTCATTGTTTTTGCCTTACAGAATTTATTAATAACAAATGTAAGAAATCCTAAATGAAATCTTACCACGATTATCTATTCTTGTTTTTATATAATCAGATTTCTTTTTCTTTTTATAATTGTATCCATTGGATAGAACTTCTATCCTCCATAATTTTTACCAAATATATATATAAAAAATAAAGTCGAACATTTATATTCTATATCTATATCCTAATTAAATTCTAAGCCATGAAGAAAAAAATAAAAATTTTATTGACATGTGTCCTAAGGTATAATTTAATTTATTTTTAATATTTTTAATAACGCAATACTAGGATTTTCAGAATCAGAAAATGGGTGTGGAGTTTGTAATAAGAAAGACTATGCTAAGCTACAACGAATGGACTGAGAATGAATTTTTTGGTGGTTGATGATGTTGTCGAAAGATACAGGAATTTTAGGTTTTTATGACATTTTTGTTGTTTCTTTCGTTATTCCACTTTAGTTTGTTGAGCTTATTTTGATTAAAAAAATATATATTTAAAATTTTAACATATATTTTGTTCGAGTAAATGATTATTTGATGTCTGATTATTTGTCTATATTAGCATGATTGAAAAGTTATTGAAAGATATTTTTTTTATATTTTTAATATATTAAATATATAAAAAATTTAAAAAATTTTATTATTATATTTTTAAAATGTGATCTTAAAGACACAAAAAAACTAAATTCTAAATTCAATTATTTTAATATAAAACATTATTTAATTATTCTAGTATCTGATTATTTTAATTTATTTAAAAAACATGTAAAAAAATATACAAAAATATGTAACGATGAATTAGTGATTGATTTTTAGATGATGTTTAAAATTATTGTTAATTAATTTGTATTTTCTCTAAAAAATTTATATTTGAATAAAATAAAATCGCATGTCATGTTAGGATAATGAGGAGAAAAATCGTAAGCAAATATAATGAATTATTTCCGAAATTAAATTAAACAATCCGATGGCAGCATAAACTGAGAAAGGAGAAAAGCACCAAAAGATGGATTGGTGAAAGGGAAAGGGAAAGCATGAGATGACATAAGACTAAGTTATAGTAATATACTACCTATTTAGTTAGGGAACATTTAATTTGCATCCATCTATCAGCTATATGGGAAAAGCATCAGATAACTCACAAGTCATCCTGCCATGCCACGTTGGTTCTTTATTCATTTCACGTGGCAAAATCTGGCCCCACATGGGTCCCACTTCCAAATTACCTTTCATTCATTGGCTGATCCAACTATAATAATATATAATCATTTTCTTTGCATGCACAAAATTTAAGTAATCTAAGTTTTTATTTCATTCATAATTTATAAATGAAAATTTAGGTGTAATGGATTTAAGGTGAAATTAATATTTGAAAATAATATTTGAAAATCGTTAAATAATTTAATTAAATTTTTATCTAACGACTTTCAGATATCAACTTGACGTAAAATTTATCTCACCTAAGTTTTCACCTAATTTATATGGATTAAGATATAACTTTGAAAACAAAAAATGGAGGAGCATACAAGTTGAATAAGTGGATAAGGAATAGTAGAGAAAAGGGGTGGATGATGATGAAACTCCATGCGAGTAGTAGTCAAGTGTGAACGGTATGTAGTTTTCTTGGGTTATGAGGAAGAGCCGCCAAAGTTGGTTTTCCTACTGTTGGTCACGTTAATGCAGATTCTTGGTCTGATGAAAAAGGTTTTGAAATGCATCCAATTAATATATTCCATGCATTGTCATGTGGAGCATACCAGAATCCAAGAAAGAACTACCAGGAAGCATAGGATCATTACCACACAAGAACACAACATATTAAACCCAAGCTTGAATACAAAGATAAAAAGAATCTTCCATTCCATTTGATCGAAAAAGAGATAGTGGGACAACCCCAATTTGCTTCTTCATTATATTCTCTTTTTGTCCTCTTTGTTAAAAGCCCAAGCAACCCCAACATTCCA

## >AdNAC65

GGTGGGGAAAACCTATGAACTAAAAAGAAAATAAAATTAAGCCTCAAATTTATTTACCTAGCTTTCAAGAATAGATTTATCATCTATTTCTTTTGTCTTATATATGTCACACACTGTACCACTACATCATCACCATCCCTATAAATTACCCTAAATTTACTTCAATTTTATTATTATCTAAAACTCAAAAATATGTATAGCTAATTAAGTAATTATTGTCTAGAGGGTTAAGTGAACAACATTACTAAAATAATAAATAGAGTGGATAGTAAAAATTATTCTCTTATGAGTTGTTATAGAAAGCGAATGCACTTTCTCCTTTGAGATCACAAACAATTCAAGTAACCAAAAACTGAAATAATCATGAGAGTTTATAAGACAAAGAAAATAGAGAATAGATTTAAAAAAAGAGTGGTTGAGATGGAGAAAGGGGGAAGCAGATGAAGAGTAGTACCAAATCTTACATAAATAAAAAGTGAGGGAAGTTGGCGTGTAATAAACATGACTTGCTGTTTCTGATTATATAATTTTAGTACTGTGTACTGACAGGGAAGATATGAAACAGCTTTATCACAATATATATACATATACACATATATGTGCATGGTGAAGGAACCTCATCAATCATCAATATTTTTTTATTTTCTTTTTTTTTTCTATTTTCTTTCTTTCGTATTAAATCCAAAAACACATGTCTTCCATCAGCTTTTGTTAGTTTATATTGTTATTATTTGAGTTTGAATTTCATAAATATCAAATTACTTATACCTTTTTATATTTCATTCAATGGATCTAACGAAATAGACAGACTTGTGACACGTGCTTCTTTTTCCTTCTTTCTTTTATTTTTTCTCTTTTGAAACTTAATTATAATCGACTCATCTTTTATCATAGAATAGTGATATTGGTATAAAAATATCCGAATCTAAGTATCAATTAATACCAAGCATAAAATAGTCATCAAATTCAAAAGTCAAAACGTTAAAGTGGGAATGATACAAAATGAATAAATAAATATCAATACGATGGACAAAGGCTTGCTATAAAAATATTATTTTCTTAATTGATGGGTCCAACATATAAATCAATTAGATTATTTTACACCTAATTAAATAATAAAAATAAATAAATTGACTTACTAAACCAAAAACATATAATATGATTATTAAAAGAAACATTTCTCAAAAAATAAGTCTATATTCATCATTCATTATAACAAACTTAAACTAATTTAGTTTAATTGACTGGTCTGCTCATTTTTTTATTTAAACAAATATTAGGTGGTCAAATTTTATTTTATATATACAATAATTTATTAATTAATTATAAATTTTTAAATAAAATTTAAATTTACGATAAATTAATTTTTAGTCTATCAATTAAAAGATATTCTGAAAAAAGAAATTATAATAAATTCTTATTAACAAAACATTACATTTATCAAAAGTTAGTTAAGAGATGATAGAAATATTTAAATTTAAATTCATTTTATATGTAGTTTAATTTATTTTATATTTTAATATATATTCTCATGGTATCTCCTAATTCGGGGGTTAAGGACTAATTTGTCGCGATACTGAGCTCCATTTAAGGGTTTACTGCTGGTCAATGAATTACTGCATGCATAGAGCGAAATTCGAACCCCCGACACTTACTTAAACGGACTAGCAAGCTAATCACTAGGCCAACCCAACTTGATTATATATATTTTATACTAATAATTAATTTTGATGTACAACTAATATAGTTATTATTATTTCCCTAATTAACCTTGTTACTTAGAATGATTTACAATTTCTACTATCTTGTTATTACTAAAAGCAAGTCTAACACAAACATATTGACATGTAACCTAATGAAAACTCATTATTGGATCAAAAGGCAAATCTAGCAAACATTTGATTCACCATGGATCATCATAAGTAAAGAGACATATAAAACCTTGGATGAAGATGAAATCCAAAATTGTTTGAGTGATGTAGGTCTGGGCTGGTTCACTCTCTCTACCATAAAGAAAAAACACTACTTTCTGTTGCCACTTTCTTCACTGACAAAGGAATACCTGCACACCTTCCTCACACACGCACGCATACTCTCTTTCTCTCTCTCTCTCTCTAGCATTGTGGATTATGATAAGGACCCTGTCAGGGAGAGGAGAGTAGAGAGAGATTTTCAGAATAGGACACTGCACACACAAGAAGAAAACACACACATTTTTCCAACCCCATATCATACATACAAATTCAATAATAATAATAATACTTATTATTATTATTATTATTATTAAACCCCACCCCATGAAGCCCTTATAAACCAAGCACCCTCCTCCATACCAAAAGGCACCACATAGAGCAGAACAACAGAAGAAAGAAAAAAGTGATCCAAACCCCATTATTAATCTCCTTTCACTCTTTTGCTCCTTCTGCTTTTTCTTCAAGTTCTTTCCATTCTCATACAATCCAAATGGCCCCTTAAGAAATACAAATAGATCGAAGAAGAA

## >AdNAC66

TTAAATTTATGGCAACGTTGGTTATTATTCTCTCTCTTCATTAATAAAATTATTTTAATTTTAAATTTCTTAATAATTTCGTATTTAAATTAAAATAGTAAAATTTATATATAACTATATAAATTCAATTATAATTAAATTATTATTTTTTTATTTTATTCATGCTCTTTATTTTAGAGAATGTTTTTTATATTAAATTATACTACACTAGTATAAGGTTTGGGTACCAGTGTAAATGTTACTAGAATTATATACACACCGAGTCCCTGATAATTTTTTAATCCGATAATTTAATTACTAAAATATTTTTTATTTTAATTAGATAAATTAATTATCATTTTAAATTATTTGTTTATATAAAAAATCAATTTATTTTTTAAAAAAACTTATTTATGTTTTCTAAAATACTGACATAAATTAATTTATTTAAATTTTAAGTAAAATATAATAAGAAAAGTAAAAAATTTTATTGATTAAAATTTGTTAAAATTTAAACTGTTCTATTTAAAATTCGGTGAATACTGATTTAGCATTTACACATTTATAATATTTTTTCCAAAACTCAGAGAATGCGAAAGGGGTTGGCGGGTCACAGCTGAAATAAACTCCAAAATACAGCTCAGGAGCTTAAGGATGACGTCATAGGCTTACATAGGTAGGAGTCAAAGTAGTTGACCAACAATTAAACGCAGTGTTGAATCCACCTCCCCAAATCCCAACCTCAGAGCCTCAACCTCCGTCTTTACTCTGCGGGCTAAACTTTTTGTTAACAAAAATATTTTTAAATTTTGTTATTGAAATATTTTTCACATCTTATATTTTATTTATCAAATATAATAAAATATATTTTTATTTAAAAAAAATAAAAAATAACAAAAAAAGTCAAAAAATTTTTGTTACGTTTTTAGATAATTTACAAATAATTTTATGGATAATAAAATTAAAAATATTTTTGTTAATATCAAAATAAATTAAGTATAATTTTTTGTAATTTATCCAGGTTTTAATCCCTCCCACCCCAACATATATACATGGCAAAATTATAAAAATAAATTTATACAAAAAATTTAAATTATATGTTGTCACACAAATTAAATTATTTTAAACTAATCACTCCTGATTGAATAAAATATTCATATATATTATAACTGTTTTATTAAAAAATATAAATTCTTTTATAAACTTAATTTTTGTTGTATACTTGATAATTGTTGAATTTTTGGGAGAGAAAGCAGAATAAGAACATAATATTATATATAAAGTTGTTATATTGAAATTGTTTTAATGGTACAAAAATCATGCTATTTATAAAAATACTTTTAACTAATTTCATAAATATTTATCTACTAACTACTAATTATTACTTATTTAATTCCTAATTACTAACAATTCTAATAATATTTTATAACGTATATTTTTAAGCTGTAAATATTTGATGATTATACTGTAAACATTATAATACACACACTTTTAATTAAACATGCGACTTCTTATTTTTTTAATATTACTCACATTTAATCCCAATCACTACTGTATGTCACCCTATATCTGAATTTTACAATCAAATCTATAGCTTTCAGTAAAAAAATTTTAGCTAATTGTTTTTAAAGGAAAAAATTATTGATCCCAAATTGAATCAACCAAAATTTTTTTTTTCTTCCATGACTAGCTAGTATGCTTTTGAATGAGCTTGACTATAGATATCAAAGGTGGATACCTAGTATAGTATTAAAAAATGATTAAAATCTAACAAAAGTTTAACCACAAAAGGAGATCATCATAGTTTAAAACTATAATAAATAATTTATTAGTACATTTACATTTTGATATTAAAATTACAGAATTATGAATAATTTTTTGGGCGTTAAAGCATAGGATTATGATGATCCGTTAGAACATTTGATAAAGGATGAGTGTAACTACCATTTTTCATGGTTATGGTGTCTCAAGTCTCATGCATGCACAAGACCAGAAGCACACAACGACGGTCAACCATGTCCCCCACCAAATAGAAAAATACTAAGCCGACACTTTGATCTTTGACATAAAAAAAATATTACTAGAAAATAAAATGGTAGGGAAAAAACGAGAGAAAGGTATTGGTGACGCAAGCAATGACAGAATCAAGAAGTGGAACCCCCACACGTGTCGACCAACCAGTAATGCGTCGCCACGTGGCAGTAGCAGTCAGCATGAGAAAAGAGGAGGCTGAGGTGTAGGTATAGCCTACTAATAAACGCTGGAACAATGCCACCTTCACCACGTTTTCACTACTTATCACGCACGTGTCTAAATTCCTCTCTCTTGTTCCTTCACGCGGTTATATAACTCCCCTCTCGTGACTCTCTCATACGTTTCAACTCGAAATTCAGCATCATTCGCCACCACCAGCACACACACAGAGATTCGATTCGGTTTGGTTCGGTTCAATTCAATTCATTCAATTTCAGCTAGAGTTGCAGCTTCTCGAGAATTCAAGAAGAAGAAGAAGAAGAAGGAG

## >AdNAC67

NNNNNNNNNNNNNNNNNNNNNNNNNNNNNNNNNNNNNNNNNNNNNNNNNNNNNNNNNNNNNNNNNNNNNNNNNNNNNNNNNNNNNNNNNNNNNNNNNNNNNNNNNNNNNNNNNNNNNNNNNNNNNNNNNNNNNNNNNNNNNNNNNNNNNNNNNNNNNNNNNNNNNNNNNNNNNNNNNNNNNNNNNNNNNNNNNNNNNNNNNNNNNNNNNNNNNNNNNNNNNNNNNNNNNNNNNNNNNNNNNNNNNNNNNNNNNNNNNNNNNNNNNNNNNNNNNNNNNNNNNNNNNNNNNNNNNNNNNNNNNNNNNNNNNNNNNNNNNNNNNNNNNNNNNNNNNNNNNNNNNNNNNNNNNNNNNNNNNNNNNNNNNNNNNNNNNNNNNNNNNNNNNNNNNNNNNNNNNNNNNNNNNNNNNNNNNNNNNNNNNNNNNNNNNNNNNNNNNNNNNNNNNNNNNNNNNNNNNNNNNNNNNNNNNNNNNNNNNNNNNNNNNNNNNNNNNNNNNNNNNNNNNNNNNNNNNNNNNNNNNNNNNNNNNNNNNNNNNNNNNNNNNNNNNNNNNNNNNNNNNNNNNNNNNNNNNNNNNNNNNNNNNNNNNNNNNNNNNNNNNNNNNNNNNNNNNNNNNNNNNNNNNNNNNNNNNNNNNNNNNNNNNNNNNNNNNNNNNNNNNNNNNNNNNNNNNNNNNNNNNNNNNNNNNNNNNNNNNNNNNNNNNNNNNNNNNNNNNNNNNNNNNNNNNNNNNNNNNNNNNNNNNNNNNNNNNNNNNNNNNNNNNNNNNNNNNNNNNNNNNNNNNNNNNNNNNNNNNNNNNNNNNNNNNNNNNNNNNNNNNNNNNNNNNNNNNNNNNNNNNNNNNNNNNNNNNNNNNNNNNNNNNNNNNNNNNNNNNNNNNNNNNNNNNNNNNNNNNNNNNNNNNNNNNNNNNNNNNNNNNNNNNNNNNNNNNNNNNNNNNNNNNNNNNNNNNNNNNNNNNNNNNNNNNNNNNNNNNNNNNNNNNNNNNNNNNNNNNNNNNNNNNNNNNNNNNNNNNNNNNNNNNNNNNNNNNNNNNNNNNNNNNNNNNNNNNNNNNNNNNNNNNNNNNNNNNNNNNNNNNNNNNNNNNNNNNNNNNNNNNNNNNNNNNNCGGTCATAAAAATTTTGTTATCATGAAACTATTTTTTTAAAATACTTAAACTAATAATAAGAGGAAGTATATTAATGATTATATCTCTAATACTACGATAAAAAATAATAAAATAATATTTTATGAAGTGAATAATATAATATATGAAATAGAATGAGTTGAGATAGATATAAAATTGAATTAAGAGAAGATAGATATATTGGGTTGATAAGAAGAGAAAGAATATGAGAANNNNNNNNNNNNNNNNNNNNNNNNNNNNNNNNNNNNNNNNNNNNNNNNNNNNNNNNNNNNNNNNNNNNNNNNNTCTGCCACCCTAACCACGCGCAATCAATACCCTTCCTTCTATTAATATAAATATAAATATAATATTCTCTTCTATCTGCTTCTTTTCTCTACATATATACCTTACAACAAACATTCCTTCCTACGCATAAATACACACCCTCTTCTCTCTTTAAATAAACATTCACATCCCCTCTCTCCCTCTATCTATTCTCCCATCCTCACTCTCTCTCTCTACCTTCAATTAACAAGATCCAAGATATGACACTAACACAAATTCCTGTGAGTACATACTATTATTTCATGCATCTCCTTTCTAATAAACACCATCATTATTATTATTATTATTATTTCATCACATTTTATACAAATCCCATAACTCCACATTCTCATGTGTTGCCTAGCTAGCTTAGTTAGTACCTTCTCATACATTATTCTTTTTAGATTTTTTCTTTATATTCTATATATAGAAATAAAGGGTCCCAATCCAATGTCTAATATGTTTTGGATTTTGGCAAAAGTGTAATCAGATCAAGTTTATTTCAAAATTTCTTGTCATCATTCAAAGAAAGAATTATTTCCCCAAATTTTTAATTTTTTTCTCAAAAGTTATTTAGTGTTAGGGTTAGATGTAGAATTGTGTCTAGCTAGCTAGGTTTGTAAAGAATGTTATATATTATATCAAAATTGTGAGATGAATGGTGATGATGATGATAATGGTGGTGGTAAGAAGGTCTTTCTCCACCAGCAGAATGAATTAGGAGTAGTAGTGTATGTATGTGTTGAGAGTGCACTACACTCAAAGAAATGAAAGTCAAGCTTTTCGGCCACAACTTATTGCATATATTTGTGTCTCTTTTTCAATCAATATCATGCTATTGCTACTTATATATATAGGACTAATCATAATATATATATCTCATATTTTTGTTTCCATTTTAATGGTGGGGTTCCACACATGGTCTGCCAATTTTTCTCATCGCACATGCTTCACTAATATTATTCTTTGGGTCCAATACCAATGTTACATCATCATCATTACTTATGTATATATGTCAGCCCTAGATACCTACATTATTTATATATCTCTCAAATCCAATAATTTTGG

## >AdNAC68

GATTTAAAACCTTAGGAATGATTTTGTATACAAAAAAATGTTAGGAACGAAAAAAATTTTCAGCCTATACCTTAAGGACCGAAATCGTACTTAACTCTTATTATTAATATAATTAATTTTAAAAACAAAAAAATATGAGTATCATTTAAATATATCAAAAAAGAGAAGAATGTCAATTTTTTTAAGGAAGCCACTCATATAAAGACGCCTAAAACGTCTTTTTTTAAAGATGTTTTTTAGTAATTAAAATTTAACATATATAATCGATTAAATCATGTTATTTTTGTCAAAATTAGGCTAGACAAATTGATTTAACCAAAAAAATGGTGAATCAAATCTTAAACTAGTCTAAATTAATATTATTTTTTTTTATAAAAAATTACTACAATACTCTTATTATAGAGAATGATTAAAATACTCTTATTATATATATATAATAGAAGTATTTTAGTCATTTTTTATATTAGGGTATTGTAGTCATTTCTATAAAAAATAATATTAATTTAGACTAGGTCAAAATTTGATTCACCATTTTTCGGTCAAATTAATTTGTCTAGCCTAATTTTGATGAAATAACATGATTTAATCGATTATATGTGTTAAATTTTAATTATTAAAAAATATCTTTATAAAAAGATGTTTTCAGCGTCTTTATTTGAGTGGCTCTTTTTTTAAAAGTTATTTAATTGGGTTGTAAACGTACATAAATGGTCATATTGGTTGTTCTCTACATGTTTTTTTTTTTTTGGAAATAAAGGAGCTCAACACAATAGAGTGGAACAAAATTAAAAACAAACAGACAAACAACACCTAGAACAGACACAATAAAAGAAGCTCTCCATTGTCGTCTCCAGCATAGCTATCAACAATACGAAAAGCTCACACCTTCCCACTCTTTAATGCCGAGCACGGACATCCTTATGATATCTCTTCAACACCTTTTATTTCATTCTGAAAGATCCTCCTATTTCTTTCCAGCCAAATATTCCAGATGATTGCACAGAAACATCTCAATCGCTGCTTGCGTTCCTCCTTCCTACACGGCTCTTCTGTCCAACTTAAAAAGTGGTCCTTCATCAATTCTGGGAGAGATCATGACCGACCAAACGCAGATATCCAAGCATTCCACACCTGCCAGGCAAAACCACATCCAAAAAACAAGTGATAGACCTGCTCAACATCCCTATTGCATAAGACACACCTCGTATCCTCCTGATTGAAAATTTCAAATTGACTCAATCTTTTCTTCGTGTTCACCCTGCCTATCAATACAAACTAGATAAACAGTTCTCTACATGTTTATTGTTCTGCCTTCATCAATTACTTTGTCTTTGGTGTACAGTATAAACGGGGTGATGTTTCTTTGAAGCTGTTCCTTGGTATATGTTTTTAAAATGAATTGTAAAATTTACTCTCCATGAAATGGTTGAAGCTCATGTGTGAATAAGGGACATCTGAAAGATGATTGCATTCATTATTTATTTGTTAGAAGGCTTGTGAGAAGATTCCTAGTTATTAAGTATTAACCATGGTATGTTGCTCGAGAGAATACACAGACAAGTAGAATAGTGATATTTGGTGATGATATTAAAGAAAATGAAGAGGAAAGAAGTTGCCAAGAAAATAATAATTTTTGCCATATAGGACAATTTTTTAACAGAATCAATGTCTCATACTTGTGTTAAACTAAACATGTCTTTTAAAATGTAATTTTACTTTTTATATGATTAAAATTGTTAGTATTTTATTCTTTGAAGTAGAAAAAAATATTAAATTTATTTACAATAGTAGAATAAAAAAGTTTATAATATCAAATATGCTTTTCTATCATAAGTCATGACTAATTACTGAATTTTTTTTTGTATATACATATTAAAACTTAATTAAAAATTAATTTTTTTAACTAATTTTTAAATTTTTTATCTTAATTTTTAATTTAAATATTTAAAAAATATTTAGTTAATATTAATTAGTTAAAAATTAATTTTTTATACTTATTCATTCACAAATCATTCATTGCTTCAAAGAAGATAATTAAAGCTAAAAGAATGAATGATGACAATAAAAGAAATGTGAAAGGACCGTTTGTTCAGTTAAGAACAGCCGCCAAAGTTGGTATCCTTCAGATTGGTCACGTTAATGTTCTTCTTCTTCCAAAGAATAGGGGCTTGCCACATGGATAGGCCCAGAATCCAAGAAAGAACAAATTAAACAAGAAGCATAGGATCAATGGCTTGGAACATATATAATAATAATAATAATAAACATTATAATATTAATTAATTTCATATTCAATCTTGATGGCAAAGATAATAGGAATCCATCCATTTGATCCAAAAAAAAAATGTAAAAAAGTACTTAAAAGAGAAAAGAAAAAGAAAGAGGTGTGAGACTGTAGCACAACAATGTTAGCTTTATTTATTTTTTTATTTTTAAATTTTGCCCCTCAATATTCTCTCTCTTCTCTTTTTGTCCTCTTTATCAAAACCCCAAGCAAACC

## >AdNAC69

AACAGACGTGGGAGGAAACATCTTTTATTGGATTTCCTAATTTCAGTCAATTTTGAGAAACATCTTGACCATTTTCTAAGTTAAAGCCAATCAAACACATTCGGATTATGGTCTATTATAAATTATACTAGAGCAAGAATAATCAAAGTAAATTTATCCATTTTAAGAACAAAATTGATAGTATAGTACTAACACATAAATGTGAAATGCAAGTTAAAAGAGAAATGATATCATTTTTTCAATTGAAGGGCGCAATATAGATTTACCTTAACAAGGACCTTTGCCTTCTTTGATTTAAGATCAACAGAATCCTTCTCAGATTTCATTGAAACTAACTTATTCTTCCGTTTCACCCTTCTCTGAGCAGCTGCTTCTGCCTCTTTATCTTCAGAGCTTCGAAACTCTGTTATGCTTTTGATTTTCAAATGCGTTCATTTCCTAGTTTATTTCATTTTTCTAAACTCAACCACAGAGCTCCAACTCTACATGCACAACTTTTCACCTCCAACATATAAGAAAAATAATAGACTAATACATAAAAATAGAATTGAAATGTATCTAAAATTAACAAAATCCTCCATGTTATCTTCCTCTTCAAGTTCATCATAACCTTCCACATACTCTATCTCTACTTCCTGTTTAATAACCAAAAACGAAAAAAGAAATTAAATACAAGGCAACATATTGTTCAAGTAAAATTTCAAATAGTTTTGCATGAAACATATATGACAATAGAAAATGCAGATGCATACCTCCTCCTCTTCCTCAGCAATAAAAATGAAACTAGTCAATGCATTCAGGAACCTTGATTTCACGAACAAGTTTATAAGAACACAAAATAAAAAACTATAACCGTTACAAGTTCTTATCATAGAATAGGAAATAGAATCATAGTTAGCGATGGAACATAGGATAATATAATTAGAGTTAGCGATGGTATTGAAAGAAAGAAAACAGCGATGAAAGAGAAAGGAGATTAGGGTTACCTGCATAGCTGAGGCAAGATCGGAGAGAGAAACACAGCAACACGCGGAAAAGAGCAAGCACTAATGGTGAGATCGAAGAGAGAAACTCACATTAGGGTTCGAAAGGGAGCGATGCGAGCAAGGCCGACGGAAGGGGTGCGACGAAGAAGAACCGGGAAGGGGTGCGACGGAAAGGAGCGATGCGAGCAAGGCCGATGGGAGTAGAGTGCAATGGAGGTTGCGTGAGATGGCGGTGCACGGCTGATAGCGCCGCCGATGAAGCATTCAGAGGGATGAGTGAGGAATCGCGAGAGTGTAGTGTGAAATGAAGAGATAATCGTGATCTTTAAATCTTTATTCTAATATTTCCGACGAAAATTTTAAATTACAGACGGATTTTCGGTCTGTAATAATTTAATAAAACAAAGCATTTTGTCTATTTAATTACAGACGGAAAATCCGTCTATAATTATTTTCCATAAAAAAAATTAATTTTACCGATAGAATTATCGACGGATTTTATTTTTCGTCTGTAATTTATGCTAATTCATTTTTTTCATTTTTCGACAAAAAAAATCCTCTAAAATTTCGTCTGTATTTTCGAAGGATAAAATCCGTCTGAAATATCCGTCTGTAGTAACTAATTTTCTAGTAGTAATATATGCTGTTTCAAAAAATTACAATATGATAAATTTAAAATTTAATTATTTTATTTCAATAACTTACCCTATATTTCACTTGCTAAAAATTAAAATAACGTATATTTTAGTCCGACACAAATAACTACCTACTAATGATCATTATAATTATATATGTAAATTAATGATAGTTATTTGGTTGTTTCGAATAATAATAATATAAAATTTGTATTCATTATTATTATAATAATTTTAACTCTTTTAATATTTATCACGTAATATAATATATTTTAACTTGTTATAAAACTATTTTATCTAAAATTATTATATATTTCATTGTTATAAATGTAATAAAACATTTATAATCTTCGATGTAATAATTTTTATTCTTAACAATTTATTATTGAAATATAGAAAAAAAATAAATTAAAATTTATTATAATAATAATAAATGTCTAAGTTATATTACTTTTATTATTTGAATGAACCAAATGATGATTAGTACTAATTTATATATAATTATAATAAAATAGATTAATATATATTTTATAAATAAAAATATTATTTAGCATCTCGAAAAGGGTAGAGTCTAGTAAGTAGTATTGCGTGTTGTAGGGTATGGATGGTGTCGTGGCCCACAGTGCCTTTTGAAGGGGTCCTGTTTTTTTTGTTAGTTTGTGAGCAGACAAAAGCAGAGAAGCTTCTTATCCTCTCCCACTAGGTTTTCACACAGCACCATACACATAGAATCTCTTCTCTCTTCATAAAGCACAACACACAACAAAATAATACACTTGAATTCAGGGGCAAAATGGTAAATCCGCAATCACCGTTTGTTCTCAACAGTGGCCTATTTAGTTCAATATCGTACAAGTCGGTTTAGTTCATAATATCAGG

## >AdNAC70

AGCTACATGCTTCGAAAGGGGAGATATTAGATGATCCGACTCTATATCGAAGGCTCATAGGCAAACTCATGTGCCTCACAATTTTCTAGCCTGATATTACCTTTGCAGTAACAAAACTGGATCAATTCATGGCTGACCCTCGCACTCCACATCTCACTGTAGTTCACCAAGTCCTTTGTTACCTCAAGGCCATTTCTAGACAAGGCATTCTATTTCCTATAGTAAATAAGTTCAACTTGTCTATATATATCGATGCCGATTGGGGGAGTTGCTTAGACACGAGAAGATTAACCATTGGCTACTATGCCTTCTTTGAATATTCACTAATCACATGGAAGAGCAACAAGCAATCACTGGTCTCAAGAAGCTCAATCGAGGCTGAGTAATGTGAAATCATATGATTAATCAGGCTGTTAAGTTTCCTAAATGTCCAAATCAGTTTAGCTATGCTATTCTGTGCAATATATCAGCTATTTACATGGCTACCAACTCTGCAGTACATGAACGCTCTAAGCACATTGAATGGATTGTCATTTCATTTGTGAAAAGGTGGCAGATGGCACTGTAAAGCTAATTCACATCCCAAGTAAGCACCAATTGGTATATATCCTCACCAAGGCACTCCCTCCTTCACAATTCAAGTTCTTGATGACCAAGTTGAGCATATACAACATATTAGGGGTGTTATTGGTTCGGTTTGGTTTGGTTTTGGACCAAAAACTAACCGAACCGATATGTTCGGTTCCTACAACACACAACCGTTCAGTTTGCTGCTTTTACAACAATCGAACCGAACCAACAACGGTTTGGTTCGGTCGATATTTTCGGTTTTTAATTTTTAATGAGCAAAATATGAATCACAATAATTAGAGGTTCAAAATAGTCAATAAACTAAAATAATAAGAGTAAAACATTAAAATAGTTAGGGGTTGGAGATTTTTGTTGTTAGATTAAAGATTAAAATGGTTAAAACATTGAAATAGATGCATATGTTCGGTTCGGTTTGGTTCGATTAGGTTTCTATTGAGCCAACCGAAAATCGAACCAAACCGATCAGTTTTGTCAGAAAAAAAAACCAAAAAAACCGATTTTTTCGATTTTCAAATCCGTTATTGTAATTTTCGATTCGGTTTGTGGTAATTTTTGGTCGGTTCGATAACTTACACCCCTACAACATATATGACTTGAGGAGAGATATTAGAATAGTCAGCTTATTTATTTATAACACACTTGATAAGTTAGTTACATTGCAGTTGTAATTAGTTACTCTTAGTTCTAGAATTTGTTATTCTATTATTTTTCGAGTTTTAGCACTTGTATGTATATATACATTTAACTAGACTATTCAATACAGTCTTTTTTTATTGCTCAATCCCTTTCTCTCTTCATCTCTGCCTTATAACATTTTGTTTCAACAATCCATCAATACTCTTTTTAATTTGTAATAATATTTTCCTCATAGTTCATAATATATTTATTTTTAGGTAAGGTTAAATATGTTTAGCATTTTAAATAAACCCTATTAAATGGCTAAAAATTGTTTGGTCAACGTATGACCAACTCTTACATTTATGCTTAGATTATTATAAGTGAAAAAGGTAATATAAATAAGTAGACTATATATTATTCAAAATAATTTTATTCTTTATTTATATCAGTTAAATAATATTAATTATCTAAAATTTTTCATTTATATACAAAACATGCACTTGTCTAGGTTCATTCTCTTACATTTATTAATTTATTAATATAAATTATCAAAAGCATGGGTACGGTTAACATTCAATATTTTCCTTTCTCTTCATATTAACCGTTTCTTTTACTCTTTAAATTTAATAAAAAAATGACGTATTTGAAGATGTNNNNNNNNNNNNNNNNNNNNNNNNNNNNNNNNNTCTTAAATTCTAAATTTTGAATTATAAATTTTAAAACAAAGTTAACTAATGTTAGCCATCTAAAAATTGACTTCTTGTATTTTCTTGTAGTAATACAAAGTATGGAACTAACGAAATAGCAAAAGTTAAGAAAGAAGGTTTGAATTTTGTTTAGCTCGTATGTATTTTTAGGACAGATATAAAAGATATTATTATAATATTTTATTTAAATAAATATATTAAAACTTAAATTTTATTACTTTTTTAAAAGTTTTTTTAATCAGTAATTTTAATGTGTATTCTTTAAGACAAGTACTATCTAAATTCCATTTATTAAAGGCCATGTACGGACACCTATATAAGAATGATTAGGATGTTAGAAGCGCGTGGAGAGACTGGGAAATTTCCAAGTAAAAGTAGGGATGATGGTGCAAAATTTTCTCATTTCACTCGTATCTCTGTTTACACTATTAATTATTAATTATTAGCATCGGAATTGCGAACTTTTTCCTACTCACTAATTCTCCGAAACCGCTAGTGGTGATGGATCTATATATATAAATAAGATGC

## >AdNAC71

TCTCTAAAATGTTATTGTGTGTTTGGTGACAAACTGAAGGCATATCTAATATAGTAAAGTAGCTGGTTGTGTGAGTTGGTAAAATTGTCGAGGTTGGTCATGATTTTATTTGGGATAAATTAAAAGGGACGACCAATTTAACCACAAAAAGATTATATGAACACAATGATAATTGATTGGTACAATGTGTATAGTACTAGACTTGAATGTGAAACTTGGATAAACTGAAAAAATAAAAACTTATTTGAGGATCTCCTTGAATAAATCATGATGTCCAGTTAAAAAATTCATACTAAAAGGAATAGTGATAAGGAATATATTCACATTGCTTAGCTTGAGGTGTGAGACGCAGTTGCTAAAGAATTGAAACTCTAAGCAAAGGGTTGGGTGCCCAATCAAAAAGTTAGTTTGGCAGTTGACAAAGTCAAGCCAACTTATCTAGTTGAAGTGCTCATCGATAAAGACATTATGGATGAAACAACATCTTTGCTCAATCCTGAAGCAAGTTCAGACCGCAACTCTAATGAAGATGACGATGGAGCAAGACTGCTGATTGATGTAAGTGTGTAACTGGCATATCAGCTAAAAAAGATCAAATTTACATGTAGAACCAAACTTACAGATTTCTCACTACTTATAATGGGTGCAAGTTGCAAAGTGGTGCCTCTTATCCTTTGATTATCAAACACAAACATTAGATGATTGATAGTTAAGAATTTACAAGCAATAATAGTCTTACCTTAAGCCACTGATGGCCCAAAAAAAAAATCCCATTTTCACATTGACTCAGCTCCGCTATAACATAAAGTAAGACTAAAAAAAAGAGTTAGTCTAAAATCTTAGACTAACCTCACATCAACTCTTCTACAATACAACTAAATAATTCAACCGGTTGTAAGTTCAACTTAGACCTTAGTTTTTTCTGTCCACTATAATCAATACATGTGAATTTACACCGTTTATGTAAATTAATTTAAATCTCGATGGTGCAAATGTATGAAATACCAAATTCCTTTCGGATAGAGTGCTGTATGGAACCGCTTCCTATACTAACTAGTCCTAAATATAAGAAACATTTAAATTTAATAAGAGATATTCTAATCAATTTTTTCTAAATTAAAAATTAAAAACTCAACACAAAAGACCATATTCTGAAATGGATATAGACTTAACTAAAGCCCAATGCCAAGCAAAGTCCTAGACTACAAGAAGAAATTGGGTTCCACTAACCCAACATGAGGTGGAGCCTTAACACAAAACAAGATGGGACCTATTAGGATAGGATGCAGGACCTGGGACCCCAGAATTTGTATGAAAGAAGAGCCTACAGGAGTGCCACTCAACCGAGCCACCACGTTTGAAAATAAGGTGGTACCCAATGGGAACCAAAAAAGAGAGGATACCACGTTGACATTGGGCAGAAGACGATCCAAAAAGCGAAGGCTCACCCCCTTCTCACTTAAAGGAGAGGCGATCGGCAGTGAAGGATTTTTCTAAATTAAAAATTAAAAACTTAACACAAATAAAAGTATTTAATTGTTTACTCTAATCTGTGATACTTAGGGCCGAAAGATCCTTTCCTTTTTACACAACTAGATATTTTAAATTTCAAACTACAACATAAAAACCAACAACCCACTTCTTCTCAGTTTTCTTATTGGAGCCAACACTAGCGTGTAATCCTATTTTCCACTCTTCTTCTGTCCTTTCCCGTTTTTTTCTTTTCTTTTCTTTTACATATGTGAATTGTGATTTTCCTCTTTTATCAATTCTGTTTCGCACTATCTCCATAATTATTACCCTTCGCAATTCATCTTGTTATTAATCTTGACTATCCTCTCTCCTTTGTGATGCACATCAGACTCTGTTCTTCTTCCATAGTCTTAGGATTTAGGATTGTTATCTCGTCTAAAATTAGGATTTAGAATTTAGAAATTAAAATTTGACCTTTTCTTTATATTGACAAATTATAGATTACATGGAAAAAGGAAAATTAATTCCGGGATTTCATTTCAATCCCACTGATGTAGAGCTTCTAAAGTATTTTTTGAAGAGAAAAGTGACAGGCAAAAAGCTACCCAATGTGATTGCTGAGATTAACGTTTATCAGTATTGTCCCTGGGATCTCCAAGGTTAGAACTAAGCGCATCTCTTTGTCTTTGGCTGTGTTTGGTTTAGCATTGGTAAAGAGAAAAGTGTCTTTTAAATTTCTTTAATACTTTTATTTTGTGTTTGATTATTTCTTCTCCTTTGAATGCAGATGTGATTTTACATTCTAAATCCATTTTTATGTAAAAAAAATTATTTTCAAGAACACTTTATTTTAACTTTAATCTCTGAAATGCGTTTTTTTTAGGATGAGTTCATCCAACTCTTTGGTAAACTATAATTTTATAGAGTTCACCCAATCCTCTAACGAACTTCATCCAAATTCTCTCTAAAAACCTGGCTGAGAATAAAATTGAAATTTTTAAGTCATTATCATCGTCTCCAACAATAT

## >AdNAC72

TCCAGTAGGTGGTGCCGTATTATTATGGAACTCATTATTACTTTCTTGTTTGGTGGCAGCTTTATAGCACCGTCACTCTGCGACAAGGCGAGAAGAAACATTACAACCATAAGGTTGATGCTTACAGCTTCGCGATTGTGTTGTGGGAGCTGATCCATAATAAGTTGCCATTTGAAGGCATGTCTAATTTACAGGCCGCATACGCGGCTGCTTTTAAGGTAAGATGACATTTCAATACTTGCTTCATATTCAATTTGGCTTAAGATGATCATATAGAATTTTGTTGCACCACTTACAAAAGGTCTCCATTCTACAGTTATGCTGAGTTACATTTTTTTAAGGGTCCATAAAGGCATATTTAACCTTTTCATTTGGGGTCTAATTTGGTCTGATATATTAATGCATATTTAATATTTATACTGTCATGAAAAATAGACGATTTTCGTAGAATGTTAAAAGTCTATTCAATTTTTTTATTCAAGTATGATCAAAGTGAGCAAAAAATATGCAACATTTTGATTATCAGACTTCAAAAATTTGAAGGAACCATTTCATTGTACGTGTTTGTTTCAACTTATTTTGGAAAAAAATGGTTTTCATAAATAATACAGGAGGAGGTTTGTGTTAGGCCTTTTAACAGCCAGTATAAAAAATTAAAAACTTTGTCGAATCTTTATGACATGGACCAAACACCTTATTGCGCCAAAGCTAGGTCATTTCCCGGAAGCAACGGGTTGTATGGCTTGAGTAATAGTGTCAAATGAGCAAGATGCGTTGCATTGTTGCCCGGGGGTAGTGCAAAATGAGTAAAGGTTCCAACGTTTTCGTGAATGGACGAGAGTAAATAAATTAGTTCACCAAGAAACTTGGTGGATAAGGTTTTGTTTTTATTGTTTCCATAAACAATTGTACTTCTTAGGTTATGCCATTGGGTTGTTATACTTTCTAGTTGTTATATTCTCATTATTATTTGATTTTACTAAGATTCATTATACAACTATTTTACTCTTTCAGAACACAAGGCCTAGTGCTGATGACCTTCCTGAGGATTTAGCCATGATTGTAACTTCATGTTGGAAGGAGGATCCAAATGACCGACCGAATTTCACGCAAATCATACAGATGCTTCTCCGATATCTCTCCACCATTTCTCCACAAGAGCCGGTTGTTCCTCAGCGGATGAATTCATCGGAGAATGCCGTATTGCCACCGGAGTCCCCCGGCACAAGCGCTTTGATGTCTAGGAGAGATGACTCCGGGGAAATCCCGAAAGCCGGTATGGAAGACAGACCTAAAGGGTTTTTCTTCTGCTTTAACCAGTGTTACTGATTATGATCCCACAAAAGGGCACCCCTCAATATCAGTTAGGGAAGAAAAACTAAATAAAGATCACATGCACCAATTGTAACTGCTAGAATCTTGATAAGAAAATGTTAACTATGATTATCAAATAAGTCAAGTTTAGTAGCAATTGTAACAAAGAAGGCACCTTAGCTCTCAGCAAGAGTTGCAAGCTTTTTAACATTATGTTGACTTATTTCTGACTGGCATATCCAAGAAAAGCTCAGAAAATTTTGGGGTCAAGGTTGCTGAGATGTAATATCATTGTAAATTATAAGGTTTCATATAATTGCCTTCACCCCCCTCTTGTTATATATACATATAGATGTGGCATGGATAGTAAAATCATAAGGGTGAAAAATGTTCTGTTGGTCCATTTATTTGTTCCATTCATGAGTGTTTGTGATAGTCAATTAAGCAGCAAAAATGTTCATGAACTAAATTCAGATTAAAAGATGAACCTAATCTTGTAGTAGCTAAAAACATGTATTAGTTATTGAACTGAAACGACTATAATTTTTTTAAGTAATTGAGAAACAACAAAAGTAAAATGAGAAAATGGGAACATATTGTCAAGATTTTGGTTAATTGAATTAAGAATCAAAGTAAAAAATTTAATCGCCGTTGCCGGGGATCGAACCCGGGTCGTCCGCGTGACAGGCGGAAATACTCACCACTATACTACAACGACTAGCTGAGACGTGAACAACAAATAATTGTTTATCAAATATTACATGTCAAATCTTACATACTCGATTCATCTCGGGGTTAGTGCTGAGAAGATTGTTAATAACGCTATTAAAGTTTAGCTTGGGGAAAAAGAAAGAGTAATTTACCATAATGCCACTGTCAAGTGTCAACGAGTGAAGACTTAACGCCAAAGCAAAGGTTCGGATTGGTGGAGTCTATCGTGGGTCCCACTCCGAACCAAAGACATAGAGATGTTGTTCTTACAAGAGTTTAGGTTCGGATAACCGAACCAACGAGTGTGTTTTCCCGCTTTCTCTCTCATTCTCTGACTACCTTACTCACTTGCCTAAGCAAACCGCGACAGCATAAATACTCAAACTCGCTTTCAAACGAGCCTTTCTCTTTTTGATTATCTCTCTATCTATCTATGATCATTTCTTCTTTGTGTCTTAGATTCTGATTCTTGATT

## >AdNAC73

TATCACATAAATTTAAAGAATGTGCTTATATATTAACTATTGTAACGTGTTTATTTATAAAAAATCGTTACGTAAATAATAATTTTTGAAATAAAATTTTATATATTTTAATAGGATTTGTGATAAATAAAAAATAATTGATAAATATTATATAAAAAAGCAAAAAGATTATGAATAATATTTTATTATTTAAAAATATATTATTTTTATGCTCATGTGTCAGTAATAAGACAACACTATTATAAATAATGAAATATATAAATAACTCAATTTCGTTTCGTACTATTTATTGATGGACATACATATCCATTATTTATTATCACTTAATTGATAATTTTTTTTAAGAGCTAATTAGATTGATGTCGAAATCTTTAAAAATATAATGATCACTTTAGTCTTTATAATTTAAAAGGTATCATATTTGAGATCGAAAAGAGATGAGAGAGATATGTGTAAACTATGAATGAATAGTCAAATTAATCTTGAAAAGATCACTCATTCCTTAAATTAATTTTTACAAATTTTTTTTAATCAAATTCGTCTTTTAAAAATTTTAAATTAATCATTTTAGTCCTTCCATCACTTCCACGGCTAATGGTGTTAGAGTCTATTGATGTAATACATTAAGTAACATCATGATATACATACTTAATAGTCCTAATTGACTATTAACATTATTAGTTTATGAAATTAGATCTAATCAACCCTAAATAGAGGGATTTTAATGTTTCAAGTTCTTCTCTCAATTAGGTTTTGATTTGATTTAATTTTATAAATTCGTTATATTAATAGTCAATTAAGACTTATAAATGTGTTTTGTAGTGTCACTTAATGTGTCATATTAGTAAATTTTAACATCATCAACAAAAAAAATGACGAAAAGACTAATTTAAAATTCTTAAAAAATAAATTTAATTAAAAAATATTTCAAAAACCAATTTAAAAAATAAATCATCTTATAGAAATTAATTTGTCTATTTACATATCCTGTAAACTAACTATTGAACATGATATGTAGCACCATTTAATGGCATTTATTATAGAGAGTGGAAAAGGCTCCCGAATACGGAAGAGTTACTCATGAGATTCGCCTATATAATAAGCATGACAAACAACTTCATCTACTTCATATTGTAAATTGCCTAAGCTTATTTCTTAGTCGTTGGCTGCTACTAGTGCTTCTGAAATTTGTACAAACCATTTTCAACTGTTCCATGAAGCAGCACCTCTTTTGCCTCCTGTGATTTCACACAGTAATGATAATGTAGTTTAAAGAAAATATTGATGTCCTTGACAAATTTGTTTACACTTAATTAAACAATTTTGAGCTCAAATCATATTTAAACAAAGAGTTTCCAACATCTGATTTATTTGAAAACTTGATCCATCTCCAACTATGACCTGATTACATCCATCATAAAGCCATTTCTGCATTAGATTGTAGGATCTAATGTCATATGATGTGTAGCTACTACACTTCTTTTTGCCATATTTAATTTGCTCTTGGACCTGAACTGGAATAGTTCCTTGAATCATAACTTCTATCCCCTTCGAATATTATTAGATTTTTTAGAACGAAACAATACTGTATTTATTAAAATAATATTATAAACAATTCAAGAACAATATAACCAAATCAAGCAAAGCACGCGACAGTGTAAAGATACAATAATTAATTAATTAATTGATTAATTAAGCTAGCAAGCCTAGCTGCTAGTATTCAATCACAAGTCTATAATTATATTATATGTAAACTTATTATTGTCTTCTTCTTCTAGAAATTAAAGTGTGTTCGATCTATGCTTGTAGTTGTATAGTTGTATAGCACTGAATGGCTGATCAAAGATACAATAATTAATTAATTAATTAATTAACAGAACTCTCTCATTCTCAAGGCCAGTGGCTAGCTTGGCTTTCTTTTTTGTCCCAAATTGTTCCTACAAAAGAAAAGAAAAAGGGTCATAATGCATTAGTAAGAAAAGCAAATGGTTATTATTATTTGGAGGATTTAATTGGTATTAAAAAATCAGAACAATCTAATAAGGGTGCACAGAATTAATTATAGAAGAATGATAATTAGGATGGTCGAGTGGACAGCTAACTTGTCTCTTTAAATAAATGTCGAAAAATTAAGTTACACTTTGTGTATACAATCATTCATTTGCTAACGATACATCATTAAATAGAGCTCAAATGCAGCAGATTAATCCTTAACTTTTTGGATTGAAAATATTGTAGAAAACAAAAATTGTAGAAGAATGGTATTAATTATTATATTATTGTGAGTTATAGGGGAAAGGCTTGTATCAAACGTTATGCCATAGAATCTGTAAATCTGTAAAAGAAAAAGAAAGATTTAAAAAAACTCAAAGATAAGGAACTTTGGTTCCTTTAATTTGCTCTATTTATTAGCAATGATGGGGTGTGATGGATGAATACACTTTTGCAGAACACTAAGATCTCTAACAATCAAATCAATACAAGAGAGAAAAATAATATA

## >AdNAC74

TAAATTATTTTATATGGAACCATCTACATATGCGGATATCAAATTGTATCTATTTTTATTTTGATTTTCATCATCAAATTATAAAATATATGCATGCTTTAGTGTTTTACCTTCTTCTTCTTCTGACATATGTACATTCTATTCTCCCTTGGTATGTTATTTTCAAGGATATCATTATTTTCATAGGAATGAGGGATACAGTTGTTAGATTTTATACAATTTTTAATTTACGGATTGTAGGAAAACAGTTGTCAAACTTGATTAACTTTGGAGCAATTAGGAAAGAAAATGTCACATTTCATTTCATATTTAAATTTGTTTATAAAAATATTACGTGAATATTAAAGATTCGTGGTTAAATTAGTTATAGTGTATTTATATATAAATATACGTTGTGTACTTTGTAAATAAATTTTATATTTTAATATATATTTTATAAAATCTATATATATCTGAAAAATTTTTTTAAAATAACAATGCTAATAAAATAATAAAATAATACTTTAATTATTTTAAATTTATAATATTTATTATTTTAAATTTTACTATTTTAATCCAACGATAATTAATAATTTGATAGCGTATTTTTTTTAAATGATAATATAACTTTAAAAAAGCAGGATCTATATCCCAATGTGCCTGCCAGCTAAAAATGGAAGGCTATTGAAAAATTCACCCCCTCTTTAATTACTGATTCTTCTGATGACTTTTATCCTCTCTATTTTTCTGGGAAACTGTTGCGAACACATTCCAAAACTTAACTGTGTATAAAAAATAAAGTTTGTTACCATATGGGGNNNNNNNNNNNNNNNNNNNNNTAAACACAAAATAACTAATCCAAATAATAAATTATTAATTAATTATTTATATAAAATATCCATTAAAATATAAAATAACATGTATCAATATCAATACTTAATATATAATAATGAAATTAATATATATTATTTTTAGTGGATATAAATATTTTTAAAATTTAATCTCCATTCCATTACTTCTGATTTTTAACATAGTTGTTTATTTGGATAAATTAACTTTTCAATGTAGTAATTTTCCAAAAGAATTGCGAACTTTTTTTTTTAATATTAGGTCTATATTGGTGTAATTTATTAAAATATCTAAAAATTTGTATTTAACCTGTGGGAATATACAAAATGTCATCGCTGTTTTTAATTTTTTTAATTTAAAAAAATAAAAAACATCAAAACGTCAATGATGTTTTTCTATCTTCAATTTAAAAAAAATATCCAATACAAAAACGGTTTTACTGTTTTTTGAAAATATATATTTTTTTTTAAAATTTTTTTTACATACAAAATGTCATTGACGTTTTATGTCTTTTAAAACTCACATACATTAAAATATTAGAACACATCACACAATTTGTTCCAATATAAAAAAAATCAGCCAAGAATTGCTGGTACTTAATATAGTACTAAATCATATACTATAATTATAAATTACAATATTAAAACATAGAAAGATCTAGGTAGGCACTCATGATGATTAATAATGTGGCCTATTTTTTCAATGAGGTTAGGAATTCGAAGCTAAGCAAATGTGAACCTATATATAAAATATAAATAATTAAATATAAATAAATATGAAGCAGTAGCTGATCCAAGCCAGCTACCAACTGAAACAACCACCACCCACCTAAAGCTGCTATAGTGTTTGTATACAAAACCAAATTAGATTAAGCCACCACCACCTCCAGGCTCCAATAGTAGAAGTGGAACTAGCAACATTTTCTAAAATTGTAGTAGAGAAATTCTCTCCTTAATTAATAATTATTTATTTTTACAAAGAAAGAAAATAATAAGCAGAGGAGTTCACCATGCATTTTGCTTTAATTTTTCATCTCCTTGTAATTATTATTCCACTTCTACTACTATTCAACCTTGTTGAACAAAGACTATTTCATTAGAAGAGAGTACGGTGAAATTCTGAGTTGGATATATAGAAAGATACGGTTGACGGTAACATACATGCAAGATACTGACAAATATTAAATGCATCAGTAGCTACTATTATTTATATATAATTAAATTGATCGTAATTGAATAATTAAATTTATAATAAATAAATATAAATAATTGAATACATATTACCATATATAATCTTTAATTAATAATTAATTTTTAGTGTGCAATTGATAATGAAAATGTGGAAATTAAATTAAATGTTAGGTTGAAGGCTCTAGCTAGCGGATAAAGTTTGTAAATTTTTTTTCAATATAAATAATAATAAAAAGAATTGGTGGAGCCTATAGAAATTAGAAATAGAAAGGATGAGGGTCCACAAAAAGCATTGAAAGCACCCGTTTTTACCATTTCTTTTCTCACCTTGGGGGGGAGGGTAGATGTAGCGAGGAAACCACCAAAGATAGGACATACGTCATGGAATGAGAAAAATACACGTATTTTTTAAATTAATTAAATGAAAATAAAAATTTGAAGAGAGAGAAGAAGAAAACGAAAACAGCAATTGTAGGCTTCTT

## >AdNAC75

ATCCCTCACTGTTCAGCTATGCCACAGTGATATAATCCTGTACCTTATTAATTACTAGCACAATTTCATCGTCCCTTTAACAAAAAGGTAAACCAATAATCTTACTTTAGCAGTGCAAGTCTAAATTTTCACCCCCAAAATCGATTACAAAATTCTGTACAACGCTATCTTTCGCGCTGTGCCTGAAAAAGATGTATGCACTTAGGCTTTGTTTGTTTCAGAGAGAGAGGTAGTTTGAGTCTGAGAATCAGACTTGCAGTTAAAAAAATGGTAAAATAAAAAATAAAAAATTAATTATCTTTAAATAAGATAATAAAATTTATATATAATAAAAATTATAAATCTATTATTGATCGATTATATATTTATTATATTTTTTTATAATTTTTTTTACTGTAGATAATTCTTTCTAGTGGTAACACAAGGTGTTATATATGATAATATATTATTATTTATTATTAAATGCATATGTAAGCCTTTTTGTATTGATAATCTATAGTATAAATTATATTCTTATTTGGTATTTATTTATAAAAAGGTTATTTATACAAAAATGTTAGACACTTTAGATATCAATATTTTTATCTTATCTTTTTTATTTTTTATTTTTGGAAAAATTAAATTTGAAGACTTCAACTGAAAATTTAAAAAATAAAAAAATTTAAGAAACTTTTTTATGTCAATTTTGAGTGTACATATAACAATTATTTTATTTTATCAAAAAGTTAAAAACAATTTTGTTAGTAAAGGATAAAATTATTATAAATAGAATAATTCTTCATAAGATAAGAGCCAGAAATATCATTGAAGTTAATATGTCTTTCTTTTCGAGTAAAGATCCAATCCGGTCCTTGTCCATTTTCATAAAGGATAAAGCGATCCCTGTCCAAAAAAAAGGGACACTTCGACCCTCGACTTTTTTATTTTGGGACAATACGGTTCTTCTGTTAAAAAATTCATTAAATAATAATAAAAATTAGTTTTATGGAGGGTTTTATTTGTATTTTGTGGGGGTTTTAAACTCCCACAAAAATTTTTTCAACAATATAACCAATTACTACCACTACTACCATTATCTTTTTCATCCTTATTATTATCACTCTTATTTCTATTATTTTTATTGTGTAACCATATTTTATCATCATCATTATCTCTTCTACCATCATCATCACTAATACCAATATTATCAACATTAAAGTTTTCTTCTTTTATTTCTTATTCCATGTTATTTTTTTCCTTTAAAAGGTATTATACTATAATTACTTTTATTTTATGGCATCATTTTTATCAATGGTTTTTCTTCATTTAACCACCATTCATGAATGAATTTTTTAAAAACAAAGTTATTAATAGTTTGTGGAGGTTTAAAACCCCCATAAAATACAAATAAAATCCCCATAAAATTAATTTTTATTATTATTTAATAAATTTTTTAACAGAGGGACCATATTGTCCCATAATAAAAAGGTTGAAGATCGAAGTGTCCCTTTTTTTTGGATAGGGATCGCTTTATCCTTCGTGAAAATGGACGAGGACTGAATTGGGTGTTTACTCTTTCTTTTCTTCCACTTTTCCATTCAAACAAACGAAATAAATAATTTTTTTCCATCTAAATATACTTGTGGAAATTAAAAATTCTATTCAGCTTCTTCCTTTTCTTTGCTTTATGGTTAGTTCTCTCTTCCCCACTTCCTTCCAAGTTCCAACCAAACAATGCTTTAATTAATTATAGGTTGATTTGTTCCCCCTTGACCAATTCCCTAAAATGAAAAGTTGGATGCATTAAAGGTCTAAAAATCTCGGTATTCAATCAACGACTTTCTACTCTTGGTTCCAAGTTTTATTTGCAAGAAGAGGAAATGATAGTACTAATAAGCTCACAAGCTCAAAGAGTATAGGCTGATATAGTAGTAGTAAGTAGTAGTAGGCATTTGCTCTCAGACTCTGAATTAAATTCTGCAACCAACCACGCATTTAATGCAACCCTTCTTGGCTTTCTCCTCAAATTACCTTCGAAATTATCCGTTCGTTTTTTTTTTTCAGGAATTTATTTAATGAATTTCCTTAATTCTTGATTTAAGTGTAAAAACAAACATTTCACTCTTAATAATGTCTATTTGAAATGATTATCTTAGAACATTTTTTTTGACAGGGATTTTATTAGAACTTCGCTTTGTAGAATATATTCTAAAATTAAAGTTATAATGTCAAAGGTACGTATAATATTTGTTTCTTAATTAATGTATAAATAAGTAAGTTTGGTAAACCAAAATATAATGTGTGCAAAACAGTATTATTGATTTATTGTATGTGTTTTGAAGTGATGATAGTATACGACAAAATAACTAAATAATTATATAAATAATGGATACATGTTCCTCCTCCTTCAGTTCCCTCGTTTTCAGAAGAGGCAGGTACCTCATTATATATATATATGTATGAGGTGCATGGTTAATGGTTACTGTGTGATACATAGATGATGATGATGATGATGCTATCA

## >AdNAC76

CAAAAATATCTAGACCTTGTGTTCAAGCCAAAAAACAACCCAGTTACAGATAACCCTTACAACAGTTACTATTGCAGAGATGCATTGCGAATTTAAAGAATAAAATGTTGTCTAGTTTTTTCAGTCAGCATATAGTCTGTAATCCTATTTTTCATTTTATAAACAAATCTCCCCTTTTTATTGGGTTCCTTTTTTTTTTTTTGGTCGGTGGATTGGACAACCCCCAATCCTAGGTACCCCAACGGATTGGACAACCCCCAATCCTAGGTACACAATACACACCCACACACTCCTCACATACTTACCAATTTTTTTCTCTTCGGATGCAGCTGATAGGACTCGAACCCGAGACCTTTGAGGTGGGGAGGGGGCGAAATGCCGTGTGAGCTATGGCTCATTGGCCCCTTTTTATTGGGTTCCAAGTGAATATTTTAGTTTTAGTTGGGCCTAAGTGAATCCTTATAAGTGCTTTTTGTACAGAAGTGTTAAATAAAAAATCTATTGGTTAGAAATATCAAATAAACGTAAACAAGCATTTGCTGAAATATGTAAACATATGGACCAACTCAAAAGTCTAGAGGCTGTTAGGACCCCGACCCATTTAGAAGATCAAATAAAAAAAGAAAAGATGAGACCCATAAACAAGGGGCGAAACTTGTCATACGTGGGTCCCCAATTTGACACAGGGAACCGAAGTCAATGATGGCAGCTAGTGCGATTGGTGGAGAACCTTCCCCGAAGGTGCCATTTTAACTAAGCAACAAAATGTTCATTTCTCTCCGACCTATTGGTTCTCTCTTCTAGTTCAAAAAGATTCACACTTGACATGACATTTCTTAACCACGTTCATTAAACTGTATTTCTTTCTAAAATAAAACAATAATTTATGTATTAGGCCATTTACTAATACGGCGGCTAGAATTATAATAATATATATGGAGCAGAACTCAACGGTACTAGCTAGTAGTAGTTAACTTGGTGGCCAAGCATAGAGGTAGTTGCCATATTATACTACTAGTATCAAGAAACACAAAATTCAAAGACCGAGGGAGATGGATATGGAATCCTTTCAAGATCATTCATAATAAGCAGACAGATTCTAGCATGAGCGTTTGTTATCTATTTTTAAGTCAATATAAATAGTTTGTTACCTAAAATTCCTATATGTAATGAGTGTTCATACTTCACACTTGAATCTTGATCACTAACGCACGTACTTACGGAGACTAATTATATATTATAAGGTAATATTGCTTGACAAAATTATTTCAAAATAATAAATTATTCTAGTAAAAAACTTTCAATTCGTGCAAGTCAAAGGAGTTTCAGAGATAATTTGATACAAGGTGGGCCAATTGGAAAAGGAAAATAATGACAGTATCCTTGACCATAGATGGATAAAATACAGGATTTGTTAATTGAATTCAAACCAAGGGGAAGAAATGTCATGTCTTACCTCAACCGTTGTTGGACCCACCCAAAATGTTTGCCCAAAGGCCTAGCTTAGAGAAACAATCCTTAAGGTAACATCCAGGCAGCTTCCTCCCAAGACCAATGTCCTTGTCCATACTGCTAAGTCACACATCACGTCACACCACATGAACTTAGGTTTTGCAGATCCATGCATCATGCATGTTGATGTTACTACTGTTTGTGGATCAATTTTCTTTTTCTAATCACACGCACTAGTATATAGGTTAAGAATGTTCTCGCGTCATAAAGTTCGCGGCTAAGAATATACCAAAGATATGGCATGCACGCTTAACCTTTTATTAGTGTGAGCTTTAATATGTCCAATGTCTTTGACCAAGTAATTCCTATGCTATTAAAAGCTTTTAATACAGAGGAAATTTGTAGGAAGAGTTAAAAATTAATATTAATTCTTAAAAACTTAAAAATACCCTTAATATCAATTCCTTAGTTTAATATTTTATTGTAAATCATATTACCTAATTAAAAGTATAGCGGTATTGGCTAGTTGAAGACACCACCACATACATAAGTAATGGACAATGGTCAAACCATTAATAATAGTGAAGGTTGAAGAAGAGGGACTTTGGCAATATAAATAAATAATCATAAGAGGAGGAATATTATTTCTTTGAGGGAGGGACAAAAACAAAAAGAAATTAGGTGGAGACATGGAGGGAACAGGATTGAATCATTTTGGGCAATATAGAAGGAAATTCAACAAGGTGACATGGCTAACCCCTGTAATAAGTGACCGCTGCCGCAACACTACACTACAATACAATACAAGTAATGAGTTCAATCTTCAATCTCCATTTCAATTTTGTGTATTTTGAAGAGTCAAATGTTATAGTACAGGCGGCGTGACATGACTGGTTAGGAGAAGGGTGCAGGTTGGAGAGCACCCAGAATCCACATGATTTACAGTCAACTTGACTTAACCCTTTTCTTTTGCTGATCTAAGCAGTGACAGAATCACTATCCCTGCTTCCAAGTTGCAAGTTCCAAGTGCCAAATGCACACGTGTCAA

## >AdNAC77

TTTTTCGAACTAAAATTTTGATACAAACTTAATTTGATATCTTATTGAAATTACAAAAAATTAAATAAAACAATATGTTTTAAATATTTTTAATTAGAAATTATATTTTTGTATTATTAATTAATTTACTTGACGAAATCATTCTTTATTTACATATTTCAGTATCTAACTTTTTATTTTCTTTTACACTAATGTGTTTTGACCAAATTAAATGCCCAACCAATAAACCAAAACATGCCGATCCCCTTTGTTTCTTTCACCATCTCAAATCCACGTCCACATAAATCTAAAACGTCACCAATCAATGAAGAATCATCGAGTGCATTATATACACACCCCCTCTCCCAAATATATTATTGACCTTGACCAACCACGTCACAACCCTATCCTTTCACATGAAAACTACCTCCTTCAATACCCACAATAATTTTATACAAACAAAATTTAACACATTATTTTTAACAATTATATTAATTTTATATAAAATATAAAATATATATTAAAAATAAATTAAATTCTATATATATTTATGTAAAAATATATAATGATTGTTTTTATTTTATTTTTTCTTTTTTTATATTTAATGACAAGTTAATATTTTTTAAAAAATATTATCTTTCTTATTTTTTGAGCACATTATTCTTTTGTATCAAAAGTAAAAACAAAAAATGTCCATAAGGCCACGCCCACAACTAAAATTACACGCACATATTACATGAGGTCCCAAAAAAAAAAAGTCATATTACATGGTTGTGGGAGTTGTTGATGAGTGAAAGAAACAACAAATTAGACGAAAAGTGAGAAAAAGGAAAGGAGGGTTTACGTGTCGATCAATGGTGGTGGCCACCGCAACGAGTTACATATTTGTAGTGCTTTCATTTTCGGTTATTTCTTCTTTCTAACAAAATTTCAGCCTAACTCACACTCTCTGACATGCACGTGTCCACCCTTCCCGCGTTCTCTCCTTTTTGCCGACGTGGTTTAAACCCACGCGTGAAATATGACTTCACTTCCTACATTATTTTATCCTTTAAAAAATTGGAAAAGAAAATAATTAACAATTATACTACTCCTAATTATTGGGATGTTAAATTAAAATTAAAAATAAAATAAGTTGATCTGACAGACAAAGATTTCTGTGGAGGAATTCGCTCACAGATCTCGAGTGTGTCAGAAACTGGATAGCAAAATCATGCTTCTAGAACACCCCTCCTGGATTTACACGTGTCGGAATGATCTCGAAGATTTTTTTTTATATTATTACTAGGCAGGTATTAAAAAGAAAAAGAGAAAAAAAATTATATACAGGTACATGTATCGTTCATGCTTTTTATTATGTAGGGGGAATGGGTGGTTGTTTTTGTTGTGCTGGCATATATGAAACGATTTTATTAAAAAATTGATTAAAGCGTATATATTGTGCCAATTACCAAAGGCAATGCAACTATTTTTTTTTATGCGTTAAGAAATAATAATTAGAATTACTGAGAATGTGTCATCAAATAAGATAGTCTCTGATATATTTAAAAATAGTTATATTCTTGTTATTGAACTTAATACAGAGACTAGTTAACTAGTTACTTAATTGTGATCCTTTTTAGTGTTTTTTCTTTTTCCGCTCATTTCTAAAGAAATGAGTTCTAAGCACGATCTCATACTTACGGGTGCCACACATATATATATAGAAAGTTTGAATTCAACCCTTAGATTTTGTACAACACTAGTCACCACTAGAAAATTATACGGCTTCAGCTTTCAATTTCAAAAGCATGAATCATCATCATCATTATCGCCGGTATTTATCAAAACATATATAAAATATGAATTTATTTCTTAATTGTTCTTCGGATATTCTTCTTCTTTCTTCACGTTACTACTAAATATCCATTTCACGTGTTGACATCACCTTGCATTATCAAAAAATATAATTCAATATAATAATTATAAAACATTTTCTAGTAACAGAAATTTTGTTAAGAAAAAAAAAATATAAATCAAATCAAACTGAGGTAGTGACGTGTGTAACAGTATATAGGTGTGAAAGAAATATTGGTACTTTTATATTTTCACTTTTTATTTTATTAAACTTTATTTTACGCTGTGACAGAAAATCCAATACAATGCACCCACCGAAACGATTGAACCGTACCGGTAACAGATAAGGTCGGCCGCAGAGAATGCTTTATGAGTAGAGGGTACGTGGCGCAATCTGACAGGACAGTAACTCAAGTGGTCCCCACACCCCAACATTACCGTACTTATCTCTTTCTCCTGTCTTCCCTATAAATACCTTCCTCTGTTTTCCAGTTACTGAAAAAGAGAAGAAAAAAGAAAAAAAAAAAAAAAAAAAAAAAAAAAAAAAGTAAAATGAAGTAGAAGAGTCCCGATTCCAAACGAACAAATTCAAACTTGATTGAAAAGGATCTTTCTGATTCTCGAAGAATGGGATGGGGGCAAAAGGATTGATCGAGAAAGATTTCTTGTTCTTATTAAAAGATCG

## >AdNAC78

ATCATCATTGATGGTTATTTGCTGGCTACCAATCACAAAAGTTGCTGGTCCCCTATGATTGCTCAAAATAAAAAACTAATAATCAACCATGATGAAATTTAAATATGCGAGTTTATTCATTTTTGCTAAGAAACCAATTTCTTTTTTTTTTCCAGAAGCAGCCTAACAAATAAAGTAGTGTGATGAATAACAAAAATAATATGTACACACTATACATCTTAGCTTTGAGCAAACCCGCAGCTTTGTAGCAAAAGCGAAAGAAGCAGCCCGCATGCATGAACCCATAATTTGCGTAGTGCTATGTATGTATCCATGCCATTCACATATATCGTGTTTTGGTTGATACTCCACATCCATGAAACATATCACGTTTTTCTATAACATAAAGTTTGTTCTTCTCCTCCTTTCATTCACCCTTTACTCCTCCAAAAAACCAACTCCAATCCCTATCCATAGCATCTAATGGACACGTGGACATACATACATTGGGATTATATTGTACTCATAAAAGATATTTAAATTAATAATAAATCATAAGTACATAAAATATTTTCAGTACAAATTAAAAGCATTTGGTGTTATCTAAATTACAGGAAACAAGAATGCATGTCAAAACAAAGCCTTGAAACCCTAGTGTTGAAGGCGTTAAAGTTGATAATAGAAACACAAATTGCAACAAAACGAGGAGCTTAACGGCCAAGTTTCGAGTAGCTTCTTTGAGAAGCCCTCCAAAGACGTGTCGGTTGGTGTATACTATTGAAAATATATAGTTGAGAAATCTTAAGGAAGGATTAATGCTAATATGTGTTAGCGTCTTTTGTCCCTACCTATTACTCTTATGTTTGTTTGTGATCTAAAGCCGTGGTGAAAACGTTCAACAACTTCGGCCGCAAGCGAAAATCTATTGTACTATTTCTTGAAAAAACATATAAGGTGATGATGACCATGACATACATGCATTCATCATATTATCATTATCATTACCAGCTAAAGCCAAATGATATCAACGACACACGTTCAAAATATTTGGAAATTCTGTGTCTTTTGAAACTTTGAATTAATACATATTCTCTTGATTAATGTGAAATTTGTTTTCTTTAATAAGAGTAAAAATATAATTTGGAACCAGTACGTGTGTGCGAGCGTGTTTAGTTTATACGGGATTGTGAAAGGATAAAGAAAAAGAAGAGAGATGCGTTACTTGAATGAAGAAGTATACGGGAAGAAATGAATTGAGAATACTATATATACAGTGTATTATTTGTTGGCGCTAAAGCTTTTATATACATAAAATAAAATCAAAACATATCCTTTGAAATTTTATCGAAAGATATTTTGAATAAAAAATATTAGGTAATTTATATTTATCTTTATTCTAGATTAATATTAATTAATTTTTTATTAAAAANNNNNNNNNNNNNNNNNNNNNNNNNNNNNNNNNNNNNNNNNNNNNNNNNNNNNNNNNNNNNNNNNNNNNNNNNNNNNNNNNNNNNNCGATTTATTTTTTTTGAGCAGTATTACTAAGTGATTTTTTTTTTATTTTAGTTTTACCGTCAATACTTAATTTTACACCTTTACACAATTATATATATATGAAAAGTATTCCTTGGGTTACATTGAACTTTGTCGGTTCGTATGTACATATCTGATAAGTGTGTAAGTTTTCTATTTTATTTTATTTGAAGGTTTTTACATAGCTTTAAGGACACTTCAAATTTGACAGTGCAGCCGAACCCCTAACACACACATGCACACAGCACACTCTAAATTAAAGAGGTGAAACGTCCTCACAAGAATCATCAGGTTGCCCCATCTCCACTCTTACCCACTACCCCTTTTTTTCTTATGAATTAAGGGTTTAAATAATTAATTAACCATGCACATTTTCATATACTCTTCATTTTTCTTAACTAAGTCAACGTAATTAATCCTTCAAGTACACCAACAATAAGATCATTTTCATTTTCTTACATTATATAATTACCCTCAGAACTAGTGCAGCTAACTATTTATTATTTAGCCCTTGATTAATTAATTGATTCAACCGTATATACCAGGACAAAGAGAAACTTGGCTTCCAAGCTTCTTTGCTATATGTAGAACCCACTTGTGTTCCCCACCTTTTTTTTATCTGTTATTCTCTCTCTCTCTCTCTCTGCTTTATCTCTTTCTCTCTCTCTCCCTCTATATTAGAGAAGAAGTTGGCGAAGTAGTAGTGCAAGGCGTTTGGAAGACCAAAAAGCACAGTTTCTGTATGAATCTTAAACAGAGAAGAATAATAATAGAAATATAGAAAAGATGGAAAACTAAGTAGATAGAGCAGACTGGTAGGTAAGATTATACTCGCTCTCCTCTGAAGGTGATCATTCAGGAAAAACGTGTTCCAAAAGGGACAAGCTACTACCTAGCTTTACCTTTTATTTTATTTTATTTTTCCTTTTCTTCTCTCTCTGTTGATTCTATTTTTATTTTAATTATTAGGGAGGTGGTGATCATCGAT

## >AdNAC79

GTATTATTCTTTTTGTGCCGTCAATCGGCTCTTATGCTTCAAGTGTTTGTGCCTAATTCTTATACCTTCACTGTTTTGAGTGGTAGTATATAAAATGCTTAAAGATATTTCTTTCTTATGATATGGGATTCAATTTACTATTTAGGTACAATACTTTCTCCCCTGATACGGTGAAGAAGATGCCTAGAACAGATCTCGTTGAGGAGGTGAAAACCCTTTATCTGATATGTAATTACAGGCTTACAGCATTATCTTTTTGCCAATATTTTCCTCATTTAAGAATGAATTAAACCGCTATATTTTTTGTTAAACCAACTATATAAGCAGGCTTGAATCGCTTTCATATGTCTCGGTAATAACAGTTCTTTATAACTCAGATTTTTAGGCTACAAGCTGCACTGGGTGAGCAGACACAAGTTACAAAGTTTAGCCAAGAGGAGTATGAAAGACTTCAAAATGTAACACTCTCAAACTTGATCTATTATTTTCTGCATTATAATATGTAAATCACCATGTCTTAGTTGTGTAAGTAGTTTAGTTCATAATCAACCTTTGAAGATTAAAAGGAAGTTATATATTGATGGATGAGCATTGTTTTTATCATTTAGTAGGTTATTTTTTTCCAGCATGCTTGAATATTTCTCAACCATATCAGGATCTCACTTTGAATTGATTTGACAAATCTATCAGGAGAAGATCTTATGTAGGGTTTGCTTTGAAGAGCAGATTAATGTTGTCTTGCTTCCCTGCAGGCATCACATTATTTGCAGGTAAGATCCCTGGCAATGTCATCACTCGTCCTCGCTCCTCTCTTATGCTTTCTATCACTGCCTTTGATTTAATTACTTTCTCTTACCATAGCCCGACCGAAAAGGAATATAGTCTTTGCTCGAAATATTACGACATCTCAATCTTCTCTAACAATTATTCCCTCTGTTCCAATTTATAATGATCTCACTTATTTCCTTGACAACTATATTGCCTTTGAATTTGTAAAAGGATGCTGTTTTTATAAAGAATCTATTTACTAAATTGCATGTTGTTGTTACTTAACAAAAATATCTTATACTTACTATGTATTGACATACTGATTTTGTGTTAATGACAGTACTTGCTGTGAGAAATGTAAGAGATGCCCCGTATGCCGAGTCAACATCGAAGAACGGATGCCTGTATATGATGTGTAGACCGAGTTCAATGAAGATGTATAGAGAGATATCCTAATCCTGTTGACCATTCTGGCTTGTGTTTTTAGATGAAACAAGCACACATGGGAGTACCAGTTTCTCTCCTCATAGTTATAGTCATGATGAGCATTGAACAGAAGGAAGTTGTATATACTTTTTTTAGTTATAGTGCATTGATGCAATTCGTATTAAGGAAGATTTCCATTCATAGGAACATTGTTTTTTTTTTAATACATACAGCAAAAATGGGAAGCATGGTCAAGGTCATTGTTGAAGTGTGTATATGAATTCAGAATAACATGCAGTTGAGGATTCATATCCATTCCAACATAACTATTGAGGCTTGTTTGCAGTTTGTTACCTTAAAATCTAATTCTAGATGACTTGAGCAAGTTAAACTTTTGTTAAAAATAATATCATATAAAATAAAGTTTGACTTTTAATGAAAGAAATATGACATGTTATTCTACTTGTAATGAATCAACGTTAAGAAAAACAAAATAACTATGAAGTTTTTAGATTTTTTTTTAAATATTGGGTTAAGATGATGTTATCTCCTAAAATAATTCATTATGTGTGTTGTACTAAATTGTGGAAACGCCGCAATTTTAATTTTTAAATTCGTTAAAATAACAAAATAGCGATTTCGTTTTCTTTTTTAAAATAAAACAGTAATGCCGTTTTCTATTTTAATAATTAAAAAAAATATAATGTCAATGACTTTTTTAAATTTTAAAAAATTGAGACAAAAAATGACGTTTTGTATCTTCTAAAAAGTTAAAAATAAAATTCATAACAGAATAAAACTCAACTAAAGCCAATAACTTGATGTGTAACACATATTTTTTACGAATATTACAAAAAATTAGCCTTATATGGCCTTTGGTGAGTCAAAGTATAAACACTAAAATTTACCAATCATATCACACTGTTCTTAAGGCATAAAAAATTTCTGACTACCGACTATGAAAATTGACTTGACCTGTTCTGCCTTGTGAAATGCCAACCAGAAAACCATCAACAATGAGCAGAAAGACCACAAGAACCTTTAGACTTGGACAAATGATTAGTCAGAGTCAATAGCATCAAATTAACAACCGTGCCACACCACCAATGTAATACTACCATTCTATACTCTTCTTCGAGGGGGACACTTTTCTTTTCTTTTCTTTTGTTCAACATTCACAATTTCACACTACTTTATTATTATCTACCATGCACTATTACTAGTAATAAGCTTCTAACGTGTTTACCAAGAAACCAATTTTTGGACTTTGGATTTTTTATATAACACTTATTAGTCCAAATAAAGG

## >AdNAC80

AGAAGATTATGCGCCTAAATTTTTTTGGAAAAATTGATTTTAATTAGGAATTTTTGAAACTCTTATTATTTAAAATAAAAGCTGCGGTCAATAAATCTTTCTAAAAAGGTAAAAATATCTTTATTTTAAAATTTATTTGTAGGAATTTTTTAAAATTTTTATTAAACAAACTCCTACAAAAAAGTTAAAATAAATATTTAATAATATATTGAAACGAAAATGTTCCATTAATGTGCAGATGCTGCGACGACACTACGTTAGGTGCATGAAATCAATGTCTCTCCACGTAACATGATTATTGTGGCAACTTTTTCAGAACTCTAATCTTCCAAGTTTCTCGCTAAACCATTAAGCTCAAGCCATCTTGCACTCCAATCAAACACGTGGTATATATAAATAACTCTCATCACATCACAACCTCCAAAAATAACTAAAAAAAATACAACACTATCGATTATCCATATCCTACACTGAGCTGCCACTCCTATAATCTTCAACTTTTTCATATTATTTCTCAACCATAAAGAAAAAAAGCTCAACTAAAAACAGAGAAAAATTAAACAAGCCTATTACGTAGTGTATACTTGTCAAAATACTGACACCACATGGCACACAAATACCACACTCCAAACCAATTTACTTAATGACAAATCCCTCCACACGTCCTTTTTTATTGGTGAGCATAATTCTCTATTTTATGCGTTATCTTTTCTTTCACATTTAGATTTTGACCGGCAGATGAGAACTACTTCACATACCAAAAGCCATGCGTGCCTCTGCCATTTTTTATATTATTATCAGTCAACCACAACATACAATTATATGAACAAAATATTTAGATTTTAATTTTTATTATTTTATTTTTTAATATTCCAATATAAGGTACATAAAAAATGTGTTAAATACATACATAATTTTCAGATATTTTTTTATTTAATCACTTAAACTTTTAAAAAAAGATGAGTTACTTTATAGCTAGTATCTTCCTTTTTTTGAACCAATTGCATTTTTGTGATTTTTCTTTTCATTCTTATCTTTAATCTAAAGTTGTCCACGTTCACAAAAATCTAAAACGTCAACAATCAAGGTAGAACAGTTCAGTGAATCACACCACTTCATATTCTTGACCTTGACCAGCCACGTCTCCACCTTTCATTTATCGTGAAAATTACCTGAAAGCTTCAATGGCCACATCCACAATCAATACTTGTAAGCTTTCTCATAGAGGTTGAAGAGTGAAAGAAACGCCTAAGCAGCAAAGCTAAGAAAGAAAGAAAGAAAGTAAAGAGCAAAAACGAATTTCAGTGCCTCTATCTTTTCTATTTCCGTCGTCTCTTTTGTAATGAAAGTTTCAGATCCATCTTTAACCAATCATAAATTACGATAATATATTCCTATTTCAGATGATCAGTTATTTTGCCTAAACAACTTAGATCTAAAAGTGATAAAAAAAAAAGTAATTACCTCAGTGGTCTAATACCTTTATTTAAAATACAAAATTTTATTGAAAAACATAAATTTCACCAAGAAAAAATTAATCATTTATCACGATTTCTAACGAGAGTTTTCTTCATTATTTTTTAATTAAAAAATGACAGTTTTAATAGCAATTTAAATATAATGGAAGTTACATAAAAAAAATAACAAATAATTTGCAACAAAAGTATAAATTGATTATATTAACATATTAATCAATTATAATATAAAATGCAGTAAACGAAATTAACTTCAGATTATCAATTAAACTTTATAAAATTCATGTGTATATCGTTTCCGAACTCAAAAGTGAAATGAATGCCACAAATATATATTGTGGACTGTCAATTTTGCAGTTGGGCACGTGTCCACCGTCCTCTGAAAAAACACACACGTGAAGAATGACATTGACCGGAATTTTTGAAAAAGAACAAAAGAACACACTCGCAGTAAAGGAAAAAAAGGAAAACAACTTAAGCAAACACATTTTTCTTTCTCACTTCTTCACAGACCACCACGTGTAAGCATTATCTGCATGACTGGCTCAATTTTTTATTATTACTTTATTAGTTTTCTTCTTGTGACGGTTATCTACATTGTACCTACTTCCAAAACATTATATGACTTTTTCACATTAATTTGATAAATAAATCATACTTATAATTTCTTGTTTATTATAACTCCATTTTATAGTTCATAAAAAATCTAATTTATAGTTTATAGAATACACGCAATCAAAAAATTAGGCATGATTAATAAAGTACTAAAATTTAAAGTGTTTCATATCATATCATACTACAGTGTAAAACTATTCATTGAATCGAATCCGGCGCAGCCTAAGGACCGTACAAGAAGCAGATAAGGTTGTCCGCGTAGTGATCTTGCCAAACTGAAACGTGGCAGCTTCTCAGACGCCGATACTCTAGTGGGTCCCGCCACCCAATTCTTCGACTAAATGATTTCCCTTATTTTGTCTTCATGCTCTGTTCCTTTATAAATAACAGCCATCTCTTCCGTTTCATGA

## >AdNAC81

TGCAAAAAATGTTGACGACAAAAAACATTTCTAACCTACACTTTAGGGACCAAAATTGTACTTCACCCTAATAATTATTATTAATCTGTATCATCTTTGGTATATTCTAAGAGACTAATTAGGGTACCAATCAACTTTTGCCAATTCGTATTGGCTGCACTCTAAAGTCCACTATAACAAAAAAAAACATCCCTTTATTTATCCATTCTTATCCTAATTTACATTTCACCTATTTCACCTTTCTCTCTTTTTCACTATAACTGCGCTGTTTATCTTCTTCACAGAAAATCGCAATCGTGTCGCTCACCCTCCCACACACGTTGTCTCCATCACCATCGACAACACATCACAGCACCGCCACCGCCTTCTACGCACCTCAACGTTGTTGCCGCCGCCCGCGATCACGCTATCGCCGCTTCCGATGCCTGTAACATTGCTGCGTGGACTCCTCGTCCTCAGAAACCCGCGACGTCGCGCCGCCACCTCCATCATTTCATCAGGTCGCGTCTCTTCTGCCTCTGTCTCCACTCTCCACGCCGCGTGGTCATATCTCCTCTTCTTCCGTTGTGCGCTACTGCTCTTCATTCATCCTAGTGGTTGTCTCCAAAAGGTTAGTAGATATTTACAGTTAAGTGGATGTTTCTTCTTTGTTCCATGGTCCATATACTATGTATAGTAGATGGGTGTTTCTTTGTATTATAGATGGATGCTTCTTATTATCGGATTGCGAATGTTTCTTTTATTTGGTGCTTATTTCGTTACTCTTAGCTGGAATGTATAAAATTTGGAATATTTCTTCTTTTTGTTATTGAATGTTTCTTTTATATATTAGTAGATGTTTCTTTTTGTAGCATGTGGATGTTTTTTGGCGCACTGCGGAGACATGGCAGCAGTCTGCACAACGACACAACGACAGCGACAACAACTCAAGGGGGGAGGGGGTGGGGCGCGACTGCTTCACTCGCATGACTTCTCCCTACAGCGGAGATGTGATCGGCTGCGAGAATCTCCTCCAACGATGATGTGCGTGGTGCACTGCAGAGGTGGCGAACTGCACGACGGGAGCCGAGGCACTTTGGCAACGGCGATGCGGAAGTATACATGAACAAAAACGTTGACACGATGGGAAGGGGTGCAAATGCGATTATGAACTGTGATTATGGGACGGTTATTTTACATAATCCTTATTATTCAAAATATATTTAATGCTAATTATTTAAATTTTATTTTTTAATTTAAAATTAATAATTAATAAATAGAATTGTTTAAATTGGCTGATTTAAATGTTGATTTTCTATACTTTTTCATTCTAAGCTAAACCGTTTTGATTTATATTTAACAAGAAAATACTATTTTTTCTTACAAACTAAATATCATCATTCATAAACAAGATCTGTGATCATCTAAAACAATAAATACATAGGAAATAAAAAAAAATGTGTAGGATATAACTGTTGGTAAAAATGACAATAGCTAGAGTAGAGTACATGGAGCTTAAATTTCATTATAATTCTATTTGGAAATTTTACTAACACTCTAAATATTTCGATCCAGCCTTGTTCGATTAGCAAAAAAAATATTTAATATTTATATCATATATTAAAGATGCGGGTTCGACATTAGAATTGGTTCGACATTATTTGATATTCTTTTATAAAAAAAATTAATAATCACTCTTAATTTGTTAGAAAGAGAACAAAAACACATATTAGACAGAAATGTTTAACTTTAAACCTCAGCAATTAAAAAAAAAGACAATTTATTTACTCTCATAAAATCCTTCATATATCCCTTTTTGAGTAAATAAAAGAGCAAATTCTTGGTGCATTGCCGCCATTAACGGTCTTTTTTCTATGTTGAGCATTTGAAAATACAAAGGAAGAAAAAAGGACAAAAAACAATTGAAGAAGATAGATTTTCAGTTGTATTTTATTTTGCATTAATCCATTTAACAAAGATGCAATAAGAGAATTCAATAACATCAACATTTACATGCAAGCTAACTTGGACACTAGTTAGATCTTATCATCATATATCATGTACTATACAAAATCATAATGATTGTAACCAATTTAAAATGGTAACGATTTACATAACTGAAAAAAAAAGGGGTCAGTTTAAAGTGAAATGAATTCCCACCAAGCATTCTACCCTTAACTTACAATGTTTTTTCTCTCCTCTTATATCAACCTAAGGTAGAATGTAATTTTTGGTATGATTTTCAAGAATTTAATTTTAATATATCATCGATGTAAAAAAAATTATTTAAACAGTCAATTAAAAAAATAATTATTTTACACATTTATCATCTGAATAGTCATATAAAAAATTATATGTAAAAAAATTATTTCGTAAAAAAATTTACCTCATAAATATCTCAAAATTAAAATCTAATTTTCACTTTCCAATTTTGGACATACTCAATCATGCACATACACCATAGAAAAACACACGGTTTAAAGCATGAAAACGCACCCTACCCAAGTTGCCTTATTAGTCA

## >AiNAC1

AAAAATTTTCCTTCCAACCAGCAGCCAAGTAATATAATTACTTTAATTCATTTTCAGTATTTTCAACTTTAATATTCCCAAAATATCCTTTGCCATGTCTGTTTCGGAAACTTTTATAAATAAAAAGGCCAGTTGTTTTCTCACATATATCTCTCTCTCTCTCGTCGTTTTGTTCCCAATAAACCTTGAAGGCCATATTGGTAATTTTGATAGGCTCACGAATAATGCTCTTTTATTTTCTCGTAATGGAAAAAATAATGAGAAACAAGGGGAAAGATTCTTTAATTTTGGTGTTTATCTATTTTAACAAAAATGGATATTTAAGTTATTAACTACACCACCTGACCCTATTTTATCTTCATGTAATTACTGAGAAAAGAATTCCATGATGAGTAGTTGATCAGAGGCACCAATCAACATCGTTGTCTCGCTTGATTATTATTTATTAATTCGTGTCACAATAATATGAAAGAATCGATATCCCAAACAAAAGAATAAGAGCCTCTGTAATAATATAGTGTGTACGAAATTATTAATGTTTTTTCATTATTAATAATTAAATAGTGAGTTTACAAAGTAATCCACATTGGATTATCATAATCGTATGCATGATATGGTGGTGACTACTGGTGTGTTGAGTCATAACATCTTATAAGGGTAAAATTGAATAACAATGACATATAGAAACAGTCACAAAATTAAAGAAATTAAAGGATATATTTGTATCTCATTTTGTAATGGATTAACTATTTACACATTATTAAGGTTATTAAAATTAAATGTTATCTATATATATATATATATATATATATAATTTTACAACTAAAATATACCACATGACACTTTTTCATTACAATTAAAATTAAAATTTTTCCTCTAAAATTAAAGAATTCTATTCTCCTTCCTACTAATTTTACAACCAAAATGTGTTACATGTTACTCTCTCATTACAACTAAAATCAAATCTTTCCCTTCAAAATTAAAGAGTTCTTCCCTCATCCTTACTAATTTTATAACCAAAATGTGTCACATGTCTCATTCTCTCATTGCAATTGAAATTAAATTTTTCCCTCCAAAATTAAAGAGTTCTCTTCTCATCCCTACTAATTTTACAACCAAAATGTGCCACATGTCATTCCCTTATTACAATTGAAATCAAATATTTTCCTCCAAAATTAAAGAGTTCTCCTTTTATCTATTCCTCTCATTCCTTCAACAACTCTACCTCTTTTATATATCATTATCAATGAATAATTATAAATTTGACAATCAAAATGTGCAACATGATATTTTTTAATTATACTGAAATTAAAATATTAAAATTAAAATTAAAATATAGCTATATTATATATTATATTATATTATATATTGTTATTTAATTTAATAACAAAAGGTGTCACATGACACTCTTATTAAATTTGAGAAAAAATATTTTCCTCTAAAATTAATAAATTATCTTCTTAAATTCTCTCATCTATCTCTCTTCTTTTTTCTATTTCTCTCTGTCATTCTCACTCTATATATAATTCTGATATATTTATATATTATTATCTAATTTAATAACCAAAATGTGTCACATGACATTCTCTTATTCAAATTGAGAAAAAATATTTCTTTCCAAAATTAATAAACTCCCTTCTTAAATTCTATCATTTATCTCTCTCCTTTTTTCTATTTCTCTCTACTCTTCCCAGTTTCCACTCTATATATAATTTATAATTTATATTTTATATTACAAGTATAAAAATTAATTATTTCTATCTGTGCAACAGACTTAACACCTAGTCAAAAGTATACAAGTTAAATCGTTTTAAATTTTAGATAACGAATATTAAAATTAATTATTAGTAATAAATCAAACTCTTTCACATGAAAATAATAACTAAAAATTTTATTATTTGATTTTTTATCTACGGAGATCCTGATCTTCTTAGCCGATGGGGATTTTTGTCTCCTACGATTTTTTTGTAAAAGTAGTTTGATTATATAATTCTTTTATGCTATAATCTATAATGTCAGGACAAAATCGACATAGTTTAAAAAGATTTATATTCCCGTAATGTAATTACGGTTAAAATTCTAGTTTCTCGTTAAATTAAGAAAAGAACGATAAAATAAAAACAATGTTATATGACCAGCAAATATTATCATTTTTAATCAATATTTGGCTAATAATAATTTATACCAATATTTATAAAAGTTTTACACACAAAAATATATAATTTATATTTATATTTATCAAAATTTATACACATGAATCAATATAATTTGTACTTATATTTAATTATTATACTAGTCAAATAATAGTTAAAAATAGTTGGTTCCAAAATTTCTCATAAAATAATTCGTTTTTATATATACACTTCACATTTAGAAGTTATTATGATAAAATAAAATGAATATCAAAGTTTACCCTACAATAATTATTAAAAAAAAAAGTCCTAAAAACGTTGTGTGTAAAATTATGAAAAGATAATAGATATAAAGTTGAAGAAAGTGG

## >AiNAC2

CAAACATAATTATTAAAATAATTTGATCATAAAACATCACTACTATTATTTATAAATATGTATTGATTCAGCATATAAAAAATAAATATGAATAAAGACAACTTTTCCGTAGCAATATTGCTATCATCCCAAAGAATTATTTAAGTCCACCACTTTTTCACTTTTCCCTCCATCTTAACCAATTTTATATAGATAAATAAAGGAAATATATAATATAAGGTGAAAATTCAAGTGAAGTCGACTTCATGTGAAGTTAATATCTGAGAACTGTTAGATAAAAAATTAGTTAAATAAGTCAAATCATCTAACAGCTCTTAGATATCAACTTCACGTGAAGTCGACTGCACCTGAGATTTCACTGTAATATAATGCCAAGTAAATTTGTTTTCAAAACTGTTAAAAAACAATTTGGTACTTGTAATCCAAGTTACAAGCATAGATTATTACTATTTAATTATATTTTAGATAATAATTATATTTAAGGTAAATTGTATAGGTAGGTAACTAATTAGAAGAGGTGGTGGCGTGGTCCATCAAGTACTATACACACACTGCTTTGTCTTGTTTATCAAGCAACCTCCTAAATAATCAATCTCTCTCTCTCTCTCTCTTGCAATGTGTGAGTGTTTGTGCATAAAACATTCATGGCAAAAGCATCATCATGATCCATTTTCAGCACTCCATCCACCTCAAAACCCATGTGAGTTCCTATCATCCCTTTTTCCCATATCTCTCTTTCTCTCTTTTTATATTCACATACATTAATCAATAATCGGTTATGTGAATTATATATATAATGAGTGTAATTAATTTCTCCCTTAATAATATATATGTCGTCGTTATTACTCTAAGCTCCTACCCTTATGACACGTCACGCCTATTAACGTGTAATCACTAATCCTTTTCATTAGTGATGATTAGTGATACTAGTTTTCTATTTGCTTTTTTTTTTCTTTAATTTGTTTGACTATGAAAGAGTTAAACTACTGTAAATTAATGATAGAAACACATAATTTAAGAGAATTATGTTCCAATATCATACAGCGTAAAAATATTTGTATTTCTTTGACATATATCATCAACTCATAATTTATTTATTATTGAATAATATTTATAAGAGATCTAGACCAATCTTATCTATGATAAATAAATTGTGGGTGAAAAATATACCATTTGATATTATCTTCACAAGAATGGTTATGTTTATAATATGTTCTTTTATGTAAGAATTTTTATTTATTTTATATCAATTTTTTTTAGAATAATCAAAAATAGAATTCTAGACTCTAAAATTATAAAAATTCTGATATTATATTATAATATTATATAATTACATGTAGATGTATAATATACGTAAAGATATAGAGACAAAAATTCTTGGGTTGGTAATTGGTACACACTTGAAAAGGGGATCGTCAATAGTAACTAGAAGATGAGAGGCATATATATATATGTATGTATGTATATATATATATGTATGTATATGTGGCATAGCTTTTTTGCTTTTTGATGTAGTCAAGGGTCTCTCAAAAGAAAGGAGTAGCTTAGCTTGAAGAATTGAAGACCTTGCTTCAATAACTTTGAGCTTTATTAATTAATTAGCTAAGAAAAAACCAACCAGGTTTTCTCTCTTTTATCTCTTTTCTCTTTCCGTATTCTTCTTCTTCTTCTTCTTGATCTCAAAATTTAATTAACTAGAAGCAAAGTGTTAAAGCAACTACTACTCTATCTATATGTGTAAGTGTGCTTCCTTCTCCTTTTTCTTTTATTATTATTATATTCATATTCATCACTTCCTTCTATCTATTCTCTGCTAATTTCCATGGTTAAAGCACAGGTTTATAGCCTCACACTTTTCCTCTTTTTCTTAGTTTCTTGTTTCTTCTCAATATTTTCCCTTTTCTCCGGTTTTTCCTGCATATTCTCCTCTTTCTTTTGCAGATATTTTCTTTTCGAGTTCATGTTTTCAATCATCTTCTTTTGTCCATATTCAAGGTTCTTTGTTTTTTTTATTTTTTCAAAATTTTATTTAATTTTAATTTTTCTCTTCCTTTTATTTATTTATGTTCATGTCCAGTTTTTTCAATCGGATAATAATCATTCTCCTCATGCATATCTTATCAACTTGTTCCAAAAAAATTAAAAAAAAAAATTCCAGTTCTTATTTTATTTTATTTCTTGGTATATATGCCTTTCAAGTCACTGCAATTTATTATATAAACACATAGTTTGGTTCATGATGAAGGATTTCACTTCTAGTAATACAATATTAAAAAGAAAAAGTTCTCTGAATGGTTGTAATTCTCAATATTCTTTTTTGCAGCAACAAGATATTATTGAAGAAACAAGGACACATGCTCATTTGTGGTGGAGAACTGTTCCATAGAAAAAGAGATCTTCAAATAATTGAAGAAGCTATATCAACTTCTTCTTCGTCTTCCTTCTTCTTCTCGTGTTTTTATATCAACAACAATAACAGTGCAAATTGCGAGTCGCCGCC

## >AiNAC3

TGTAATGAGAAAATTTTAAGTTCACTTTTTTCTATATCTCACTTAAAATTAACAAGAAGGAAAGTCTAAACATTAAAGAAGAAGAAAACAAGTTGGCAATTCTATTACCTTATCCATATAAAGAATTTTATATATACAAGTAGTATATTTACTTCATCCAAATAAAGAATTTTGTAATTAAATATTAAATTTTCCATGCATTTAAAATGTTCCTAAATTTAAAAATATTTTATCCAAATACAATTTTATAAATTATACACAATAATTAATAATTAAAAAACAAAATTATACACACAAGTTTTCTTGTGTGGGTAGAGGCCTAGAGGGTAAACTAAATAAAATATAAAATAATTTGGTTTTAACGGTGTAAAAAGTGAATTTTTTGAATTTTAAAATTTATTTAGAGGATATATTGTAACTTTTTATCATTTATTTTATAGATAGAACAAAAAAAATATAAAATAAAAAGATTTAAAAATAAAAAATCGTATTTTATCTTTTCAAATAAAAATTTAAAATTTAAAAGATTTAAATTTAAAAAAAAAAATTGTGAAGCTTAATGCCTAAGCTTCTCTTGCTTGGCTTCTATGACAAGCTCATAAAGACGTGTCGGCTGCCGCATACAAAAGTCACTATTTCTTAATTAATTCAAGCCTTATAATCTCCTGTGCTCCTGTCTTTGCTATGTACCGTACCTCCAACAATCCAACCAATAGGATCTCTCCACGTCATTATTCCTTACATGCAAATTTACCAGAACGTCACTCTATCACAAACCATTTAATAATTATATCATAATAAGACGCTGATTGTTAACATTCGTTTTCCCCAAGCTATATGTGATTTACAAAACCACGTTGTAATCCAGCATTCTTCATCATTCTTGATATTGTTAATAATCAATGTTTGTAAGTCCATCCTCTACAAATGCCAATAATTAAGCACATATAGTTAATTAAATTAAATTACTGTATGTTTGTACCAACAAGCTAATATGCTCTCTCATAGGTTCCAGCACAACTTTGTTGAATGAGAATAAAGATATTCAGTTCCCAGTGTGAAGGGAACAGAGATGAATGGAGGGGGGAAAATAAGAGAGATTCGTGAAGAATTGAACATTAACGAGAATATGCAGTACTAGTGTGTATGTTTTTTTTGTTGGTTGGTGCAAAAAAATGTATTATATTTTTGGAAGAATTTTAAGTGTATCGGAAACACCAGTGTTCCAGTTGTTTTAACCGTTGATCTGAATTATAAAAAGTATATATAATATATATTAATTAAAATCAACAGTTAAAATAACTGAAACATCGATGTTCCTTGATACACTTGAAATTTTTCCTATATTTTTAACTAAAGTTCATTCTTTGTATATATTTCATTCATTCATTTCTTATGATATGCACTATTTTTTTATATTTTTGTTTTAGTACTAAAAGTAATTTATATAAACAGTGAAAACATCCTTTTGTGGTATACTTAGATATGTGTACGGTGTACCAAATTTTTAGGGATAAGTATAATTTTAATTTTTAAGATATAGGTTGAAGATTTGTTTCGTTCTAATATTTTTTTTATAGAAAATTGTCCCTAAGATTTAACTTGGTTTTAAAATCGTTCTTATTTTAAGGACCAAAATTGTGCGGAGGTGATGGCGAAATCGAAGATACAGATGAATCGTAGACACTTCTTCATTTCTCCCTTATTTTTTCCTTCCTTTTTGTAGAAGCAAAAATATTTTCTTTCTCTTATTTTTTCTTTTTTTTTTATTTTATAATTTTTTTTGTTATTAGTAATATAGTCTAAANNNNNNNNNNNNNNNNNNNNNNNNNNNNNNNNNNNNNNNNNNNNNNNNNNNNNNNNNNNNNNNNNNNNNNNNNNNNNNNNNNNNNNNNNNNNNNNNNNNNNNNNNNNNNNNNNNNNNNNNNNNNNNNNNNNNGACAATTTTAAAATTTAATTTTAAATTTTAAAGACGATTTTATATAAAAAAATATTAAAAACAAAAAAAAATGTATATCTTAAGAACTAAAATTATACTTAACCCAAAATTTTTCCACAACTTTGAGGGCACTCTAATGCAACTTAACACTAGCTACTAGCAGCTCATCACTAATCAGCTTTGGACACACACAAAACCTTAATTATGTGGATCTCTTTCTCAAAAGAATCAGGTTTGCTCCATCTCCATTATTACCTTAGATCACCTTTTTAAAGTCATATATATAAGCTTTCATGTATAATAATAAATTAATAATAATCATAATGAGACCCTTTTGTTTGGCCTTAATATATAATAAGTGAAATAATTTAATTTTTTACAACACCATAGATATAAATGTAGGAATGCAGCAAAAAAGAAAAGTCTAGTAGTCTAGGAGGAATTTATTCTATTTAAAAAAAAACGTATATACCATAATATTATTACCAAACTTGACGTCCAAGTAACCTTTCCTTATATCTAGAACCGCTTCCTTTGGTTCCCACCTTTTCTTTTTATTTC

## >AiNAC4

ATCTAATTGAATAAAACCCTATTATACCCACCTCTAATTGTTAGGTTTTCGGGTTCATGCTTACCAAAACCAAACCAAACCANNNNNNNNNNNNNNNNNNNNNNNNNNNNNNNNNNNNNNNNNNNNNNNNNNTTCAAGAGTCATACCTGAAGAAAAATAATAATGTTGATATACATCCCAAAAAGAAATTATTAATGTTTGTTTTTCATGTTAAGTACGGTAATTTGTCGATTGTTACTGTGGGGATTGGACTTTAAATAGACAGATTTTTAAGTCTAACAACATCAATTACTTGAATAGTTGTTGTAAGAGCATTTGTTATCGAAATAGGTTGACACTGTGTTGTTGAATGGTTAGAGTTAGGGATAGTGTTGGTTGTTCGGAAATGAATAGCTCTGAAGGGGGTAAATTTTGCCGCGGTTGGTGGTTGGAGACGCAGCGGTAATGAAGGCCGTTGCTGAAATAGAATTCAAATTCCACTATGACAAGTAGAACTGACCGACGAAGTTGGGTTTGTTAATCGGCGATAGCCGCTAGCGCATTTCGGACATCTATCTTCTCCAGCGACGAGTACTCACTGACAGTTAGCACTCCGACAATTTCGTGCAATTTTCACAGCAATTTTCCATCAATTTCCCACTATTCCACTTCCTCTCTGTTATAAACAACATGATGTGAATGTCGAATGCCTGCTGCAATGTACCGTCCTCTCAAATACAAAAACCTAACTCAATTATATCTCATCAGCCATCGATTTTGTGTTAACATTTGTTGTTAAAAAATAGAACAATTTAATTAATTATAAATATAGTAATTAATTTTATTTATTTATAATTTAAAATATTGGTTATTAAATGTTTCACCATATTTAAATTATGAGAAGATACCTTTAATATCATTGCAACGTGAATAACGGAAACTGATTCAATTAGTTTCGTCTCTTCACCCTTCTTCTCTCTCCAAATGCCTAAAACATGATAAATCAAAAAACATCCTAATACCTAGTATAAAGTAGAGATTTTGGATTCATCCAGAAACATTTAGTTAATTTTTGGTTAGAACTATCTTGGTTACTAGCATTATTATTTATAATCATCATAATCACGACAAAACTACGGCCGTCAGAAAATGCACCCTTTTATATATATATAGTAGTAAAGAAAACAGTAAACTGTTTGGTGTGTGTGCTTCTAGTTCAAGTTTCGTTTCAGAAGTTTAAATCCAAAAATATATATTAATATATGATTTAGTATAATAATAGTTGTAGTAATTAGAAGAGAGGGTGGTGGTGGTGGTCCATCAAGTACTAGACTGCACATTTATTTTCCTTGATTATCAAGCAACCTCTCTGATCTCCGAGTGAGTGAGTGTGTGCAAAACATTCATGCCAAAAGGCATCATCATCACCACTCCAGCTCAATACCCATGTGAGTTTCTATCAGCCTTTGCCCCCCTCCCCCCTATTTTTCTCTTCTCTCTCTCTCTCTAAGATAATTTTGGCTTTATTATGTATGTAGATATGCTCCATATACATGAATTTGTTATACGTGCAGTGAAAATAATGTAGATGTTAATTAATAAAAAAATTTAAAAATCAGTAAAATTTATTATTTTTTATTAATATTTTTAATTATTAATTTAATTTTTTTAATTTAAAAATGTGATCATATATTTTTAAAAAAATTTAGGGATCAGCATTTTTATTAAAATTTGGTCAGTACTTAATCAACAAAAGAAAAATGAGTAATATATTTTTTATTAGATGAAATCTCACACCATTAAAAATACTATTAATAACTAATTAATAACTACAAATTACAAAATTTGCTAACCCCTTAACATTTCTCTATACTTAAACATTATTAATTAACTAATGGTCTAAAAATAATAAATTTTATTCATTCTCTAACATCTCTATTAATTAAATTGTAATTCTTTCAAAGTTAAAGCTATATATAAGTCGTTTTTATTACTCATCCATGGATGTTCCGCGCCTAATTATTTCTATGATTCTGACACGTATTAACAAGTGATCAGTTAATTTTAATGTGTATGGTTCCCAACTTCCCATAGCTTAACTATGGTAGAAAGCTTCGCAATATATATGTTTCTCTCTAATCAATTATTTATGTTTGTCTTCCTTTTTTCTATTCTTGTTTTCATATACAGGACTATTTATTTTTCAAACTTGTATTTTTTAGTTATATATTCCAAATATATATATATAATTTTGTGCCGTTTTGTGCTTTATTTGCTTTAGCAGTGAAGGCTGAAAGAAAGGTTCTGCCTGCCTCCTCAAGTAGTAGAAGCAAGCAGCATACACATACAAGAGAGAAGAGACGAAGGCAGCATGGGCTTTCAACTTTAATTAGTTAGAATAAACCAAACGGCCTGGATGGTTTTCTTTTTTTTTCTTTTTCTTTTCTTTTCTCCTTCTTTCTTTATTATATCCTGTTTCTGTTTCTCTCTCTCTTTATTCATTTCTCCTTTTTCCTCTTCT

## >AiNAC5

TCAATTCATCTATATGAACAAATAATTTGATATAAAAATTAATTTATAATAAAATAAAACTTTAAAAATTATTTTGATAATTAAAATTTATTAAAATATTTCATTTATTTTTTTAAAAGAACTAATTTAAAATATTACTCCTTGTTTGGGCATTGTTTGGCATCACCAAAAACGTATAATATCTACTGCATGGGAAATGAATCCTCTCTAATGAAAAAATATTTGAGAGAATAAAGTGTGATCTTTCACCTTTAATTCTCTAAGTGAGACTAAAATTAAATAGGAGAGAAAAAATAATGAAGGATTAGATTAAACACGGGATAGTATCCAGTTTTTTTTCACTGGAGAGGATCCACTCTCCTATGCATGATATGCACTTCAACAACTTATAAACATATCATGCATAAAAAATATTATATGTTTTTTTGTGTTTGCATCAAGGTATCCATAAGGCCGGAAGCTTAATGACTAATCTCTCGGGTACTGCAGAGGTAAAGTGGGCGACCCTCCCAGGCAAACAAGTTTCAGTCTTATCCTCCAGTGAGTATCGAACGCGGGAGAGATGGTTAAAGGACACATACTCTCATCTATCTATCCCGATTTTTTTTTCGGTATAATATGCCAACTTATTTAGTGATGCCAAACAATGCCCAAACAACTTCAAACGAGGCCCATTAGTTTAGTTACGGCAATGGACATATAACTTGCCTAGTATTGCACAAAAAAAATGGTCTAGCATAATATTTCTTTATTTTTAGAATATACGTTAGCTAAATCCTATATATATTTAATTAGCAATTATGGCCCTTTTTATATTGTATGAGTTCCTCATTCATATCACAGATGAAATAAAGAAAAGAACAACATATGTTCACCGCTAGTTTATAACTTTCGGTGATTAATATTAATGGACATTGAAAATTGTCAACAGGAGACAACAAACTTGACCAAAAGAAAACAATCGTGTTAATTAATTGACTTTCTAAGTAGAAACCAAAAATTCAATCACTCAAAATATGGTTAATTGAGTAGGAACCATAGATATCCAAGCATGTTAACTGCACCTGCAATCAAATTGAAACGACTAAGTTAAGATATTCATATTCTAATAGTTATTCCTAAATTCTACTTGTTTTTGCTTATGATGCAAGTAGATGCTAACCTTTACTTTGTTTAAGGTATCTTTCTTTTCCTTGTCCAGAGATTAGAGCTTTTGTGTGCATGGAAAGTTACTTTTGTTGCGTAAAAAGTCTGGATTACTAATCAGTAAAGAGAAATGCATTATTATTAAAAAAATATTAAAAAAATATTCCCGACCAAGTGGTCATCCACACATGTACTCTGGAACACAATTCAGTGCTATAAATTAAACATCATAATGAGTAACATTTTAAGCTAAAATTGTGTGTATTTATTAGGCGGACTAAGATGATAACGTATTTATGAAAACTGAAAACAAGAAGAAAGAGACCAATATTATACTAGTTAAGTTATGAAATAGATGATCTTCTTTTCCTTTACCACTAAATTATTAACATGTTATTTTATTGTTTAAAATTTGATATATTTGAAATTATACCAAAAAAAATTATGAAGAAATTTTTAAGGTGATGCTAATCAATTTACCAAAAGAGTGAAAGACAATAAATTCAATCTTTGAAAGCCAAAGCTTTTTATTTGGTTAATTTTTTTTTAAATACAAATATGATTTTGTGTTTTAGTTCGGTTTACTAGAAACAATAAATAGTAGTAATTTTTGCGTTTGCGTTCAATTATTAGATTGAGAAATAATTGAAGAATTTTTTATGCTAATTTTGCCATTTAATTTCATGGGTAATAAGATATACATTAGGAACAATTATTAGAATTTTTGTATTTTTTCATGGTCATACTTTACAAATAACTATTCTTTTTATACTATCATTTTTAGTACTTTTTTTGAGCTTTGATTTTTATTTTATCAGTTTTTTTTTTCTTATTCATAGTATCATGTTTTTATTTAAAGTTTTTTCTTTGANNNNNNNNNNNNNNNNNNNNNNNNNNNNNNNNNNNNNNNNNNNNNNNNNNNNNNNNNNNNNNNNNNNNNNNNNNNNNNNNNNNNNNNNNNNNNNNNNNNNNNNNNNNNNNNNNNNNNNNNNNNNNNNNNNNNNNNAATTAATTTTATATGAATTTTTTATTCGTTAATACAAACAAATACTTAATCGGATTAAGACAAGAAAATAAAAAATGAGATGAAGCAGCTAAGGAAACGGTGGGATGTCAGAATGAGGGTGAAAGATAAAAGCGGAAGCACTGACTAGTTGTAATCGAAGTGGATAACATGCGAATGGCACGTCACAAGAATGACACCGAACCTGCTTGGTTCCCTAGCCGACCCTGTTTCACTCAAAGTCTAAAAGCCTAAATTGCCATACCAAGTTGTTGTTACTCACAAACAAGCAAAGAACAGAACAATATAACCCTCTCTGACACCAAACCCTTCTACCTTCACCTTCGAATC

## >AiNAC6

AGAAATGTAAATAACAAGAAAGTAAATGAAACTCAAATGATAAAAAGGAATAAGAGACAAGAAATGTAAATAACAAGAAAGTAAATGAAACTCAAATGATAAAAAGGTCTTGGCAAGGGTTGATGGTTAAGGATCTTTATCTTTGTCACTAACCACAACATCATAATTGCAAGGATCAATCCCATTAAGTCATCTTCTAACAAGTAAAGAAAAGTCAAATGAGCTACATTAATCCTAATCCATAAGTCCTAACCACCTCACTAATTAACTTAGTGGAAGCTAGAGTCAATGGACATCAATCATCAATCACTTGGAAATTAGCAACTCAAGTTCACCTAAGTTACCATCCCAAGCCAAGAACACAAAAATCTACTCTAACATCCTTCCAAACATTTTATCAAACACTTAGAAGGCACAAAAGGGAAGCAAGATAAAATGACAACAAGAATAAAATCTACAACTACCAATTGCAAGAAATTAACAACAACAAAGGAATTAAACAATAAAAGAAATCAAACATAAATTGCATTAAAGGAAAATAAAAGGAACAAGAGTGCATTAACAACAAAGTAAACAATTACAAGGAGTAAAATACTAAACTAGGAAAGTAAAGGTAAAGGAACAAGAATTTGCAAAGGAAAAGTAAATCAAGGCATGAATTAAACCTAGATCTAAAGAAATCCTAATCTACATCTAACCTAATTCTAGAGAGAAGAGAGAGCTTGACTCTCTAGAATTCTAACCTAAAGCATGGTGAAAACTCAACTAATTGCTTCCCCCCTGACCCCTCTTGAATTCTGCATGAAGTAGCCTCAGAAATGAGTTGGATCTGGGCCTGGGAAGCTTAGAAATCGCCCCCAACGAATTGCCTTTAATGAGGTCACGTGCCGCTTGCCACGCGTACGCGTCGGTCACGCGTACGCGTCATCTTGCAATTCTCCTTGTCACGCGTACACGTCGTTGGCAAATGACCTTCTCACGCGTGCGCGTCAACTTGAAATCTCCACAAGCTCATTTCTTCATGAATTCTCCACTTGCATGCTTTTCTCTTTACTCATTTGATCCATTCCTTGCCTCTTAAACCTGAAATCGCTAACACACATATCAAGGCATCGAGTGGAATCAAGGTGAATTAAAATTAACCAATTAAAGGCCTAAAAAGCATGTTTTCACTCTTATGCACAAATTAGGAGAAAGTCATAAAACCATACCATTTCATTGAATAAATGTGAGAAAAAAAGTTGATAAAATTCTCTAAATTAAGCACAAGATAAACTCTAAAAATGAGGTTTATCACATTCATAATCAGAATCCAGCACACTAGCAGATGCTAAAAGAGAACCACATCCAGAGTTAAGAATATGAATAAAGCAGTGAATGGATAAATGGGCCTCTAATTGATAGTTAAGCTGGGGTCAGAATTTGCAGAGCAATACTTACCTCAATGGACAAAGCCTCTCCTTTAAGGTCATTACCAGCGACGGCTGCACCGTTGCCTCCAATGATGTTGCCCCTCTTGGTTGGTCTTTTAGCCAGGCCTTTACAGGCCAACAATTCCCTTAGAAGACACAATAATATAGATCCTTATTTGCATCAGAAAATTTGGTAGGGTGTCGACTGTCGTGGAAGAACTGGAAGAGAGCTTCTTCTTTTTTAGTAGAGAGAAAGAAGAGAAGGAAGGAGATGAAGAACATCAAAACTTTAGAAATGGTGAACAATTTTTTTATTTTTTAGATGTATTTTTGTTTTATTTTTTAAATTATTTAAAATTTAAAAATATAATATTAAAATTAGTTGAATTTTAAAATTTGATTAATTCAAAAAANNNNNNNNTGTATATCTATTTTTTTTTTTTTTTCATTTAAATTTTTGGGGCAAAACTTGCGTTTCCTTACTCACGACGTAAAATGAGGGACCTCTCGTCCGTTTCAGGTACACGCGTCAGCTTTTCATTTGCTTGACAGCATGTCCTTTTTTCCCTCCTTCTGCAGTTTCCTTTTTGAACAACAACAACCACACTCATAATTTTAATTCCAACTGTACCCCTCTATATTCCATCAACTCAGTCCGCGCCATATACTTCAAAAGTGGCAAAATTGTAGTTTCGGAAGCAAAAACCAAGGGTAAACAATGGTAACGCGGGATATACACGAGGAACCGTGAACGCGCTTGTCACGTAAACTATGAAAGCAGTATTAGTACTCTACAGTGTGTAGCACTGACGAAACTTTCTTTCTTCTCTCTCTTTCTTTTTCACTTTCTGTGCAACTTCTTTTACTTTGTTGGCGACTACTTTGTTAGTCTGAATGTCTGATAAATAAAAAGTGAGCAAAAGCTAAAGGGGCCACTCACCCATCACACCCCAAATTCTCACCAAATATTGAATCGAATTATTCCTCTGATTCCTTCCTCATCTCTTCATTTGCTTTTATAATTCATCATCCCATTCTCTTTCGCTTCTTTTTTCTCTCTTTCGTTTCTCTCTGTTTTCGTCTCTGCT

## >AiNAC7

TTTAAGGTTTATAATCTTTGTGTCGGCGTTCTAGGATTGCCTCTGACATTCCCAGAACCTTATATCTTATGTGCGTGGCACCTTTACCATGCTGAGAACCTCCGGTTCTCATTTCATACTGTGTTGTTATTTTTCAGATGCAGGTCGAGAGGCACCTCGTTAGGCGTCTGGAGCTTTTGCAGTGAAGTGGTTTCTTTTGGGGTTTCTTTTGATCGTTTGATATATGTATATATAGATTCTCCTCTGATATATTTGTTTATTTTGCTCCTCCTAGAGGATTATGGAGAACTAGGGTTTTATATATGTATTTTTTGATATCGAGTTATGTTTATATGTATGTAAATACTCTCCGGCCAACATTGGCTTCGCAGGCTGAGTTAGGAGCTTGTTATCTTGTATCCTTGACCCTCTATTCCTACTTTTTGTTTGATTATACTTATGAACTTTAGTTTTCTTCTCACGCAAGTAATCACGTTTTTTTAGCGTTGCGCTTTTATATTTTGCGATTTTGGCTTTACCTTTTCTTTAAGGTTCATCGTATATTATATCCTTCCATTTATTATACTATATATATTTTATTTTAAGAGGCCGTAGCGCTTCGCAACCTCTGTTTTACATCCTAGGTGTAAAGCTCTGTGTGGTAGGGTGTTACCGTACAAGCTACCTTCATCCGCCATGGTCCCGCTTGAAAATGACACCTTAGGAATTTCAAGTGGCCAAATCATCTCCAAGCCTTTTCCTCTTTAATTTCTCTCAATCCACAACTCTCTCTCTCTAAACAAACAACTTCTCTCTCTAAAATTTTTTGTTTCAATTGAAATGAAGAATCCGCTACTGAATCCAGCAGATTTATAGGCATATATAAACTACTATAGGGGTACTGCGATTTCTGCAATGTATTATTTGTGAAGAAATTATTGTAGGGTATAACAATTTTTTTATTGTTTAGTTCGTTCAAAAATCGCTATAGAAATATCATACCTGCCACAATGTAAACTGTGACACCTTGTAGCAGTTTACATTCAATCCAGAATCTGTCATACCCTCTCGCGGTTTTTGTAGAAATATAAAAATTGTATTCGCGTTTGCAATATTTCAAACATTTTATTTAGGTAGCTTGTTTTTTGTTTATTAAAATACTTCTTAATAGTATAATAATGATTTTTCAATTGTTGGTTTGAAATTGAAAATATAGTAGAATTTGAGCACTATATACTTTCAGACTTAATATTATTTTAAATAAATGGATCAAATAGTATAATTTTACTTAAAATAAATCTTAATTGTAATTTGATATCTTTTTTTCAAAATTTGATTTTTATTTTTTAAACCATAAAATAATATTTAGGTTTTTTAAATATACATTTTATTATAAAATTAAATTACAAAAGTAATATAAAGAATTATATAAATTATGTTTTTTAATAATTTTATACTCTAAAATAATTTTAGTTAATATTATTTAAATAATATTTATTTATTCAAAATTATATTTGACAAAAAATATCCAAACACAAATCATATTAACATCAATTTAATTTCAACCAAAATTAACTATTAAAAAATACATTTATACCAATATTCATTTACAAATATTAATTCTCACACACACACTAAATTCTTTGTTTATAATCATATTGTAAAGATAAATATATATTACTACCATATTATTCGAAACGTTGAATGTAGTATTCTGAATCGAAGGCGGCTGCTAGCTTACTAAGTTGCTTTCTAATTTACCATTTTTATTGAGATATAAAAAGTAGCCACTATGTTATACCGGTTAATGCATTATGCATGTTACTCGTTTCATAATCCGGAAAGAAATAATCATTCATGACAGCAATATTGCATTTTTCAGTTTTCTTCATCACCTACATATATTTAGAAATTAGAACTACCACATTCCTTAATAATAAATAACTTTCACAAAAAGATGAAACAAATATATTTATCTCTATTCTTAATTTCTAATATAGCGAGAGAGCCAGATAAGAAAAATCATTTATACCAATTTCAGAATCAGACAAAACCTAATTAATTTTCTTTTTCTAAAAATTGAGAATCTGCTGATTAAAATAATGAGTGATTATTTTAATTGGTCACTAAAATTTTTTAAATAAACTATTTTGATATGAGTAGGGAAAAAGTTTTGGAGCCGCAAGAGACCACGCGGTATGGTGGGGCATGGTGTCGATGGAGAAGCTCCAAATCATAAATCACGCGAGTCGACGATCGAACGGTAGAAAATCCTCGAACTGCCACGTGCACCCTCATTTCACCGTGAAACACACTGCAACAAAATCAAAAGAAGGCGCCGACTCCATGAGCAGTGAGAAGATGGGGCCCAACCGACTGGGCCTCAGGCCCGTCCAAAACGAGTGTCGTAAGCAGTGACAAAATCATCTCTTGTGGGCTCTCGACACGCACGCACCACACTTGACTGATCGACACGTGTCCAACTGCGTCACCACCCCCTCGCTCTTTAGACCCCCCTCAC

## >AiNAC8

AAATTTCCAATAGGTGGTGCCGTATTATTATGGAACTCATCATTACTTTCTTGTTTGGTGGCAGCTTTATAGCACCGTCACTCTGCGACAAGGCGAGAAGAAACATTACAACCATAAGGTTGATGCTTACAGCTTCGCGATTGTGTTGTGGGAGCTGATCCATAATAAGTTGCCATTTGAAGGCATGTCTAATTTACAGGCCGCATACGCAGCTGCTTTTAAGGTAAGATGACATTTCAATACTCGGCTTCATATTCAATTCGGCTTAAGATGATCATATAGAATTTTGTTGCGCCACTTACAAAACTCAAAAGGTCTCCATTCTGGTAGTAGAACAGTTATGCTGAGTTACATTTTTTGAAGGGTCCATATAGGCATATTTAACCTTTTCATTTGGAGTCTAATTTGGTCTGATATATTAATGTATATTCAATATTTATACTGTCATGAAAAATCGACAATTTTCGTAGAATGTTAAAAGTCTATTCAATTTTTTTATTCAAGTATGATCAAAATGAGCAAAAAATATGCAACATTTTGATTATCAGACTTCAAAAATTTGAAGGAACCATTTCATTGTATGTGTTTGTTTCAACTTATTTCGAAAAAAAAAAAAAGGTTTTCATAAAGAATACAGGAGGAGGTTTGTGTTAGGCCTTTAACACCTAGTATAAAAACTTTGTCGAATCTTTATGACATAGACCAAACACCTTATTGCCCAGGTGTAGTGCAAAATGAGTAAAGGTTCCAACGATTTCGTGAACGGATGAGAGTAAATAAATTAGTTCACCAAGGAACTTGGTGGATAAGGTTTTGTTTTTGTTGTTTTCATAAACAACTGTACTTCTTAGGTTATGCCATTGGGTTGTTATAGTTTCTAGTTGTTATATTCTCATTATTATTTGATTTTACTAAGATTCATTATACAACTATTTTACTCTTTCAGAACACAAGGCCTAGTGCTGATGACCTTCCTGAGGATTTAGCCATGATTGTAACTTCATGTTGGAAGGAGGATCCAAATGACCGGCCGAATTTCACGCAAATCATACAGATGCTTCTCCGATATCTCTCCACCATTTCTCCACAAGAGCCGGTTGTTCCTCAGCGGATGAATTCATCGGAGAACGCTGTATTGCCACCGGAATCCCCCGGCACAAGCGCTTTGATGTCTAGGAGAGACGACTCCGGGGAAATCCCAAAAGCCGGTATGGAAGACAGACCTAAAGGGTTTTTCTTCTGCTTTAACCAATGTTACTGATTATGATCCCACAAAAGGGCACCCCTCAATATCAGTTAGGGAAGAAAAACTAAATAAAAATCACATGCACCAATTGTAACTGCTAGAATCTTGATAAGAAAATGTTAACTATGATTATCAAATAAGTCAAGTTTAGTAGCAATTGTAACAAAGAAGGCACCTTAGCTCTCAGCAAGAGTTGCAAGCTTTTTAACATTATGTTGACTTATTTCTGACTGGCATATCCAAGAAAAGCTCAGAAAATTTTGGGGTCAAGGTTGCTGAGATGTAATATCATTGTAAATTATAAGGTTCATATAATTGCCTTCACCCCCTCTTGTTATATATACATATAGATGTGGCATGGATAATAAAATCATAAGGGTGAAAAATGTTCTGTTGGTCCATTTATTTGTTCCATTCATGAGTGTTTGTCAGTCATTAAGCAGCAAAAATGTTCATGGAACAACTAAATTCAGATTGAAAGATGAACCTAATATTGTAGGAGCTAAAAACATGTATTAGTTATTGAACTGAAACGACTATAGTTTTTTAAGTAATTGAGAAACAACAAAAGTAAAATGAGAAAATGGGAACATATTGTCCCTTTACAATTGGTAATTTGGCATATAAATTCTAATATTAAAATTGTGGCTAATTGAATTAATAATCAAAAGTCGTAAAAAAAAAAAATCACACTTAATAACATTACTTGATAGTAAAAAAACCAAGAGAATTCATTCGCATTTGAAATTAAAAGAAGCCATGATAAGTTGGAAAACAAAGTTGACAAAATATAATCGCCGTTGCCGGGGATCGAACCCGGGTCGTCCGCGTGACAGGCGGAAATACTCACCACTATACTACAACGACAAGATGGGAGGTTGTCAACAAAATCAGTTATTTGTCAAATCTTACATACTCGAGTCATCTCGGGGTTAGTGCTGAGAAGATTGTTAATAACGTTATTAAAGTTCAGCTTGGTGAACAAGAAAGAGTAATTTACCATAATGCCACTGTCAAGTGTCAACGAGTGAAGACTTAACGCCAAAGCAAAGGTTCGGATTGGTGGAGTCAATCGTGGGTCCCACTCCGAACCAAAGACATAGAGATGTTGTTCTTACAAGAGTTTAGGTTCGGATAACCGAACCAACGAGAGTGTTTTCCCGCTTTCTCTCTCATTCTCTGACTACCTTACTCACTTGCTCAAGCAAACCGCGACAGCATAAATACTCAAACTCGCTTTCAAAACGAGCCTTT

## >AiNAC9

GTATTGAACCATAAAAAAATGATTAATTTTTTACTATTTGATATAATCTCACACCATTAAAAATACTATTAATAATCAATTGATGGTTACAAAAAATAAAACTTACTGACTCTTTTAATAATAATATATTGAAACGAAAATGTTCCATTAATGTGCAGATGCTGCGACGACACTACGTTAGGTGCATGAAATCAATGTCTCTCCACGTAACATGATTATTGTGGCAACTTTTTCAGAACTCTAATCTTCCAAGTTTCTCGCAAAACCATTAAGCTCAAGCCATCTTGCACTCCAATCAAACACGTGGTATATATAAATAACTCTCATCACATCACAACCTCCAAAAATAACTAAAAAAATAGAACACTATCGATTATCCATATCCTACACTGAGCTGCCACTCCTATAATCTTCAACTTTTTCGTATTATTTCTCAACCATAAAGAAAAAAAGCTCAACTAAAAACAAAGAAAAATTAAACAAGCCTATTACGTAGTGTATACTTGTCAAAATACTAACACCACATGGCACACAAATACCACACTCCAAACCAATTTACTTAATGACAAATCCCTCCACACGTCCTTTTTTATTGGTGAGCATAATTCTCTATTTTATGCGTTATCTTTTCTTTCACATTTAGATTTTGACCGGCAGATGAGAACTACTTCACATACCAAAAGCCATGCGTGCCTCTGCCATTTTTTATATTATTATCAGTCAACCACACAACATACAATTATATGAACAAAATATTTAGATTTTAATTTTTATTATTTTATTTTTTAATATTCCAATATAAGGTACATAAAAAATGTGTTAAATACATACATAATTTTCAAATATTTTTTTATTTAATCACTTAAACTTTTAAAAAAAGATGAGTTACTTTATAGTATCTATACCTTTTTTTGAACCAATTGCATTTTTGTGATTTTTCTTTTCATTCTTATCTTAAATCTAAAGTTGTCCACGTTCACAAAAATCTAAAACGTCAACAATCAAGGTAGAACAGTTCAGTGAATCACACCACTTCATATTCTTGACCTTGACCAGCCACGTCTCCACCTTTCATTTATCGTGAAAATTACCTGAAAGCTTCAATGGCCACATCCACAATCAATACTTGTAAGCTTTCTCATAGAGGTTGAAGAGTGAAAGAAACGCCTAAGCAGCAACGCTAAGAAAGTAAAGAGCAAAAACGAAAATAAAAGGAGAAGAAGAAGACAAGGAAGTACATATTTCATGCACCACGTGTCACCCAATGGACCCTTCAAAACCGCACCACTGTTTTTTTTTCAGTGCCTCTATCTTTTCTATTTCCGTCGTCTCTTTTGTAATGAAAGTTTCAGATCCATCTTTAACCAATCATAAATCATGATAATATATTCCTATTTCAGATGATCTGTTATTTTGCCTAAACAACTTAGACCTAAAAGTGATAAAAAAAAAAAAAAAAGTAATTACCTCAGTGGTCTAATACCTTTATTTAAAATACAAAATTTTATTGAAAAACATAAATTTCACCAAGAAATAATTAATCATTTATCACGACTTCTAACGAGAGTTTTCTTCATTATTTTTTAATTAAAAAATGACAGTTTTAATAGCAATTTAATAATGGAAATTACATAAAAAAAATAACAAATAATTTGCAACAAAAGTATAAATTGATTATTAACATATTAATCGGTTATAATACGAAATGCTGTAAAGGAAATTAACTTTAGATTATCAATTAAACTTTATAAAATTCATGTGTATATCGTTTCCGAACTCAAAACGAAATGAATAATGCCACAAATATATATTGTGGACTGTCAATTTTGCAGTTGGGCACGTGTCCACCGTCCTCTGAAAAAACACACACGTGAAGAATGACATTGACCGGAATTTTTGAAAAAGAACAAAAGAACACACTCGCAGTAAAGGAAAAAAAGGAAAACAACTTAAGCAAACACATTTTTCTTTCTCACTTCTTCACAGACCACCACGTGTAAGCATTATCTGCATGACTCGCTCAATTTTTTATTATTACTTTATTAGTTTTCTTCTTGTGACGGTTATCTACATTGTACCTACTTCCAAAAAATCATATGACTTTTTCACATTAATTTGATAAATAAATCATACTTATAATTTCTTGATTATTATAACTCCATTTTATAGTTCATAAAAAATCTAGTTTATAGTTTATAGAATACACGCAATCAAAAAATTAGGCATGATTAATAAAGTACTAAAATTTAAAGTGCTTCATATCATATCATACTACAGTGTAAAACTATTCATTGAATCGAATCCGGCGCAGCCTAAGGACCGTACAAGAAGCAGATAAGGTTGTCCGCGTAGTGATCTTGCCAAACTGAAACGTGTCAGCTTCTCAGACGTCGATACTCTAGTGGGTCCCGCCACCCAATTCTTCGACTAAATGATTTCCCTTATTTTGTCTTCATCCTCTGTTCCTTTATAAATAACAGCCATCTCTTCCGTTTCATGA

## >AiNAC10

TCGACCTACATCTGAAAAATAATAAAATAGTATAGGATGAGAACCGGAGGTTCTTAGCATGCTAAAGGTGCCACGTACACAAGACATAAGGCCCTGGAAATGCTAGAGACAATCTTAGAACACCGACACTCAAATTATAAAACTTAAAGAGCTAAAACAAAAACCATAAACAGGTGGTCCTCTAAGGTTCTAACTTAAACCAAATCCGAACTAAAACCCTCAATCTCTTCGCCTTTCCTCCATTCCTCCAACTCCGATGAAATTGCACAGACAGACAAGCAGACAATGGCAAACACAGGTAGAATACAAGTAATACAGATAACAAATATAACAAGTAGCATATTATAATCACTTAGGCAATCCCAAATAATGCACAAGCAAGCAATTCAAACAATATGCATATGATGCATGCATACCCTATGGCTAATGAGTCTCATCTGTCGGTTATCAAGCCAACCCAACAAGTCCGGCTGCTAAATCCTGGACTGTCCCCCGACGCGCATCCCCAAGAGTTTATGCATAGCTTTTTCTCATTTAATTATAATTGCTAAATGGGGGTTAACCTTCTCTAAAATTTATAAGTACGCGGTCACCTCTTATGTTGTAGGGTCAATAGAAATTAAATAGTTTTTGAACGAAGGGACTAAATTACACATTTTAATTCTCTTTAGGGATCAAATTACAAATTTTAAAAGTATAAGGACTAATTTACAATTTCATTGAAAGTGTAAAGATCAACTGTATAATTTAACAAAAAAAAAATGACCATGTTAATTAATTGACTTTTTAAAGTAATATAAAATAACAATTCAATTAAGATATTAAGTTTGATAAAATAAAATCAGTGCAACTAAATAATCCGTTAAAAATCATCCAAAATCAAATCCAAGCATGTTAACTGCACCTCTAGTTAAATTGAAACTAATAAGCTAAGGTATTCTAATATTATTCCTAAGTTCTACTTGTTTTTTACTTAGAGTTTTTGTGTGCATGGAAAGTTACGTAAAAAGTTTGTATTATTGGTCAATGAAGAGAAATGTATTATTATTATAAAAAAAAATATTCCCTGCCAAGTGGTCATCCACACATGCACTCAAACAAACATAATGCAGTGCTACAAATTAAACATCATAATTGTAAAATAGTATCCATTTCCTAATGCAATAAATTGATAAAGTAGGAGAGACACCAGCAGAATCTGCAATCTGATGTGCAAAAGAACAATTGATAACAAGTGGAAGAGGATGAAAGAGAACGAGAGTGAAAGAAAAGAACAGAAATAGAGAACTAATGATATATGAATTTTGAAATATTCATCATAAAAGAATCACTGACGTTATCAGTATTTTATGTACAATGATTAAATAGTTAGATAAAATAACAAACCAACTAGAAACTAGGTTAGCTCACTGTAAGAAAAAAAAGCCTACTGCTACAATTTTTTTATTATTGTGCTTTAAAAGTGTGGCCAAAAGAGGATCAACGGCCACGCTTTTATAAGAGTGAGATTGATTAGAGATTTGGTCATAGTTTTCCGTGCCATGCTTTAAAAGTGTGGCCGAAAGAGGTCAATCGTCACGCTTTTATGAGGAGGACGATTGATTAGTGATTTGATCACACTTTTTTTGCCACACTTAAATAACGTGACTATGAAGAAAAACAGGCACGCTTTTGAAGCGTGACTAAGGCCCAATTTGGATAAACAACTTAATTAAGTTACTTTTGAAGAAATAGCTTAAATAATAAATGCTTATATTAAAAGTAGCTTATGAATAAGTTATTTTGTGTTCGATTTTTTAGTTCTAAAAGTACTTATTTTAAGAGAAAAGTGATAAAAAAATTTTATTATGAGAGAAGTTATTTTTTTTAACTTCTCCTTAAGCACACAAATAACTTTTTAGAAAGTTGCATTTTTATTTTGAAAATTGTACTAGACATTAATACTACATCTTTTCATAAGTTAAAAAAAAATCACTTATAAACCTATCCAAACATGCCCTAATTTGTTTTCTTACTGTTACATTTTTAAAACGTGCACATATCCTTTAATTATTTGGCACACTAAAAAATAGCATAGCAATAGAGTTTCCTAACTCTAGGCATAACAAACTAAGCATAACAAATCATTAACAAACTGTACAGGAGCTGAATGAATTTGAATTTTATTCAGGTTCATGCTTATCATATTCTCTTACAATAATGAGCAACATTTTAAGTCAAAATTGTGTGTATTTATGAGAACTGAAAACAAGAAAGAAGAGGCAAATATTATACTAGATAATTTATGAAAGAGATTTTTTTTTGTGACTCAATCATAAAAGAGATGATCATCTTTTTCTTTACTATATATTAAATATTAACATGTTATTTTATTGTTAAAAATTTGACTAAAATCTGATGTATTTGAAATTCTATAAAAAGATAAAAAATAAGTTCCAAGGTGATGCTAACAAGACAAATTTAGATGGATTAAGACAAGGAAATAAAAAAAATTGGGATG

## >AiNAC11

GATATAGACGATACTAATTGATCCTAATTGATGCTAATGATTCTCTAACAGAATATATGATATTTAGGGGTGTTTATGAATCGGATTCGATCCGTATATTCGCGGTGTTTATCCGAATTCGATCCGAAAATTGCGGATATAGATCTAATCGGCAAGGTTTTTGGATCGGATCAGATTGCGAATTTTGTGTTGGTATCTGCATATCCGTACGCATATCGGCAAAAATAAAGAAATAAATAAGTAAATATTTTTTTTATGTTTTATTTCAACTAATACTTATATTATATGTTGTATTATTTTAATTTATTATTCAAGAAAAATATGTTTAATATTATTTTAAGAGTAAACATATTTAAAAGAATAAAAAAATAAATTTTATTGATTTTTTTTAATAAAAATAAACTTTTAAAAATATTTTTTGTATTTTGAAGATATATCTGATATCCGATCCGATCCGTAAATATGCGGATCGGATTGGATCTAACCTTAAAAACTATGAATGTTAAATCCGATTCGATCCGATAATTTTAATGCGGATCGGATTAAAATTTTGGCCATATATAATCCGATCTGATATGCGTTCACCCCTAATGATATCAGGATGCTTACCTTTAGTCGATTTTGGGATTTTGAAAACAATGACATGCAGTATGACTTTATTTGCATCAAATAAAGAGAAATATCAAATAAACATGCAAAGTAAACTTTGAACTTATTATCACATATTTTATTTCCCTTAACCAATAATAATAATCTCCCACATATGGGTGGTATTGAGTACAAATCGAAGGGATATAAACTGAGAGTCTTCTCTATTTTTATCCTAAAAAGCTTATAAATCATCCTTTTTTATTGAAGAATCACTTCAATTATTTTATCTTTTAGAATAATTTTCTATCGGTTCTTTTACTTAGAATCCTTTTAGAGCTAGAATTCTTTTTTACAGAGCTTCTTGATCTACCTCTAAAGTATGGTGTTGCTGTCTCTTTCCGTACAGAATTTTGTAAAAGAATGAGAAAATCAAGAAATAAATTAAAGTAGTTTGAGAGAAAAAAATTTGGTTGGACTAAATTACAAAACTAATTTATTCATCTAACATTAAAAAATTTTAAATAAATTTAAACTAGGATGCTACTTGACTAAAATGAGTTTGTTTGCATTGCTATTTCTCTCAATTTGAAATTTAAACCTCCCACTTATTTGAAATTAAAATTTAAACTTGATGAGAGAAGGAGCGGGTGTTACACATTTAATTTTTTACATAAATTTATTTTTTTTACCGGATTAAAGAAGTTTATATATACATACATATAGGACTGAAAGTGAGCTAAAGTCAGTTCGAACTCGATAAACTGAATTCGTGAGCTGGTGAGTCGAGTTTGAGCTTGAAATTGAGCTCATAAATTAAATAAGTCAGATATGAGAATGGATGAGCTCAGTTCATTAGCTCATGAGCTAGTTCAATTATATATTTATATATNNNNNNNNNNNNNNNNNNNNNNNNNNNNNNNNNNNNNNNNNNNNNNNNNNNNNNNNNNNNNNNNNNNNNNNNNNNNNNNNNNNNNNNNNNNNNNNNNNNNNNNNNNNNNNNNNNNNNNNNNNNNNNNNNNNNNNNNNNNNNNNNNNNNNNNNNNNNNNNNNNNNNNNNNNNNNNNNNNNNNNNNNNNNNNNNNNNNNNNNNNNNNNNNNNNNNNNNNNNNNNNNNNNNNNNNNNNNNNNNNNNNNNNNNNNNNNNNNNNNNNNNNNNAAAAATAAATATAAATACTATATTAAATATTTAATATATTTATACATAAAAATTAAATCCTTTCTCCATTTGTTTGTTAACCTTCTTCTATTAAACTTCCCACATTGTTCCAAACTATAACACACCTCTTTTTTCTTTAATTTTGTTATTCATTTAACGTAACTTTTTTGTACTTAAATTATAACTTCAACATGCATATTGACCATGCTTCTGGATAAGGTTGATCTGGACCAATTTTTCTCCTGAGAAAAAAGTTGGTTATATATATAACATGCTAGAGGTGTGTTCTTATTTTATTTATTTTTTTATTTTGATGAAAAGAGTGAGGTTTTTCGTCTGTTGTGGATAAATATCAACAGTACACAATTATTAAAGTGTAAAGCAATGTATGGGTTGGAGGTAACTCCTTTTTCCTTTTAGCGTTGAAATTAAAAGAAAACAATAATATTACATATGTACACATCATATGTAAGGTCAACTTTTATGAAAGTACAATGAAAGAGTAAAATTTCATTGAAAAAATTTTTTTTTATTAAGATACTGTTGGACATACAAAATACGTGTGTCAGATGCATGTCGATAAATATTATATTCGAAATGTGTTCGATACGTTTAAACACAATAAATCAAAAAAGTATCTGTGTAAAAAATAATAAAAAAATTAAAATAAAATAAAATAGAGAACTAGCTATGTATAAATTAGTGAATCATTGTTTGAGTCAAGAAGTAAAAGGATAATTACTAGAGATATGT

## >AiNAC12

TGAAACTTCTCCCAATTCCCTCTTCTGGGCAACTAGCTGACATCTTCACCAAGCCTTTGTCTCCTCAACCCTTCCATCTTAATCTGAATAAGCTTAGTATTCTTGACATCTTTCATCCTCCAGCTTGCGGGGGCGTATTAAACCACTCTTCCATCTTAACCCATGACAACAATAAAGACATCTCACGGCCCACAAATTCAGCCCAACAGAATACACAAATTAAATTATAATTTAGTCTTTATCTTATCTTATAATTATCTTTATCTTTATCTTATCTTTATCTACTTTTATCTTTCATAGGCCAATACTCTATATATACTTAGTTTTACCTTCATAATTTACAATTCATTCAATCAACAATCAATAAAATTTCATCTTTTCTTTTTCACAATTCTTTTATTTTCCCATTCTTTCACAATTTTATTACTCTTCTCTAAAGAATTCTTATACCTTACACTAATAAAGAACCACATACTAGGTTAATTTAAATGTATATAAAGTATTTGTCAAACGATACTTGATTAATGCAAAATTTTATAGTACTATTCAATCCAATGATTATTAGTTGGTTTTGTACAAACTTCTCTAATTAAAAAAAATAAATGAAATAAAAACAAGTTTAACTATTAACTTTCATTATATACTAATATATAAATATATATAATCATTCATGTATCTATTTTTTTAATTTAAAAAAAAAGTGAATTCATGATAGTATTAATAGTCTACCTTTAATTTTTTATGTATATCAAATGTGAATAATAATAAATTTTGTTTTGGCTAAAACGATAAATACATTATACTAAAAACCGAAAAATCATGAATTATAAATGATAATGCATATTTTTTTAAAAAGATCTACTATCTCTCTTTGATATCTCAATTATATAGTAGATATATATATATAGACATATATACAAATGAAGAAAAAGAGTAATTTAGAAGGCTATTTTATTGTGTAGGCATTGTTGAATTTGTAGGTCGGACAGAAAAGAAGGAGATAGAGATGAGTAGGTTAATGGAATGATACGAAACGACAGATGTTGAAATCTTAATTATTTTGCATCAACACACATTCATCTCACATGTGTTCTTTAATTTAACACAATTAGCTTACGTTGATCTTATATTTATTTATTGACTTTGTGAGCTTAAGTGATTTCATGACACCTATAATTCATTTCTCAATTGGCAAATTAAAAATGTTAGAATAATTAATTATTGGGACTTATTTGCTTTCTTCTAGAAACCCTAGGCTATACACTATAGTAGTGAGGAAACAAGAAGATATATATGCAAGATACACAAAGTTCTTGTTAGGATTGACATTTATGAATGATACTTTGAAGATTAATTGATGAATAATTACGATGAGAGTTATATATATATATAAAGGGTTTCAAACAGAAAGGTTTACCCTCATGTATATACGTACGGTCTTCTTGGTTGGTCACCATTTTAACCACCAGTAATAACATTATTAGCTCTCCAACACTCTTAATTATATTGAAATTAAATTCACATTTTTTACACTAAAAATATTATTATCTTTTACTAGTATTTGACTAGTAATAATTTACACTTATATTTATAGAAATTTTACACATAAAAAATTATATAATTTACATATATATTTATCAAAATTTCAATATAATTTGTATCTATATTTTTTAAAATTTGTACTTTACACTCATAAATTAATAAAATTTATTTATTAAAAATAATTTAGTATTTATACTCATCAAATAATAATTATAAATACTAAAAATGATTCACTTCCAAAAATTTCTCATTATATATACAACACTGAATAGTCGTTCTTCTTGATATTTAAAAAAAAAATTACTTTAGATTGTTCCTGATCTTCTCCTAGTCTGGACCATAAACTATGCATGATTGAGATTCTGAATGAATTAACTGTGCAATATATATATATATATATACATGCAGTTAAAAGTTGAATTTAATTTCAATATATTTCTAGTATAAAAAAATTGTACNNNNNNNNNNNNNNNNNNNNNNNNNNNNTTTATTAATCTCTTCACTTTTCATAAGCCTGATTCATATTGAATATGACACATAACACATCACATTGATATAGTCAAGGTCGCTGGGTATATATACTTCATTTTGTCTAGTAAAAATACCAAAACCCTCGTGGCAATTTGGTCAATTCCAAATAATTAATTAATTAACCTCATCTTATAATTAATATAATATATTTATTTAATTTAAATGTATAGTTTTGTTGATGTAGGGTACCATATTTGTCAAAGGGTTAGATATATGCATTTAACTATGATATCTAATATGAGGATTCTTCTTGTTGAAGTTGTGAGGAGTTATAAAACCATAAAGTGGAGAACCATTTAATAAGAGCTTCATCTTGGTCATGTATGTTATAATTGATGAGGATTCTTCACATCATAGCTTGGTAAACTGAAATTAATTAAGGTCATCATCAAAGGGGTTTTAGGGTTCTTCATAGGAAGAGTAATTG

## >AiNAC13

TTCATACGTACACATATTATTTTTTAGTTATTGTATAAGTAACTATCAAATTAACACAGATGTTTTTAAATTTTTTCATCATTGAATTTGAAAAAATGTCAAATTTTTAAATTAATTTATTCAATTGATAAATTTACTCATAACATCCATAAATTCATATGCATAACATCGATAATTTCATACATATGATGTCTATAATTAATAAATTTTTATAATTAATATTACCAAATTTTAAACTATGTGTCTTATTGTATTTTTCAAATTATCTCATATGTGCATTAATAGGTTATGATTTTAATTATTTTAATATTTTTTTATTATTTTAATATTTTTTATTTAATATTCATATGTATATGTATGCTTATTTATTGATTTTAATATAACAAGTTAATAATTATTTTTAAAAATTTATTTTTTAATTTTTTATATCTTATCTTTTATTTTTTATTTTCTAATAGTTTATATGTAAAATATGAGTTGTACAGTAGAAGTTGTTAAAATTTTTAAATTTAACCTCCATAATTTCTGTAGAAAAATTGCATTTTAATGGGTAATAATGTGAGTGAGGAATTTTAGGAATTTAATTTGGAAGAAACTGAAATTAATTTTAGAAAGAACATTAATGCTCTAAGCACGCAGAAAAATAGTAGGTAATAAAGAGAATAAGTAATAAAAAAGTATATATCACTTACTTATCAAGAGAATGAAAATTTTTATATGACATTTTTATATTGTAATTAATTTTTGACTACCTAGCACTCCTCTTTTATAATTAGTAATACTAAAAGGAAGCTTTGATGATTTTCCAAATCCTCCTTTTCCTTGGCGATTAATATTGTGGCATATGTACCAATTAATGGTACAAAAATAATACGGTAATAATGCTAGAAAAATAAAAAAAATAGTCAGAATTTATCTTTTTTAGTATTTATTTATTAATTATTACAATAATTAATACATATTAAATAAAACGAATTCTGATTATTTTTTGCTAGTTATTTTTTGTTAATAAACATTTTCCATAATAATAATATCCATAGATCTAAGATTTCAAGAAACTTGATCGATGACTGTATGATAATGAAATATATTTTGATAGAAAAACTTATTCTAAAAGTTTAAACTGATAGAAAAAGATAATATAAATAATTATATCTCTAACACTTTTTTTTAGGTGAGTTTCTATTTTTTTTGGGTTTGTTTAATGATACCATGTTATAAAATCACTCATCCAAAAAATTTAAGCTGATAAGAGGAGGTAACATAAATAATTATATATCTATCATTCTATTTTTTATTTATTATTATAATTACTTTTTAACATTTTTTGTGTAATTTTAAACATGAATTAAGAAACAGTGAAAATATCAATTTTAAGGTTAATCAGATCATTTATTTTTTGTGTGATTTTTCATATTATTCCTCATTTTCATCATTTTATTTTTCATAGAATTTTCTATTAAAGGTAATTTCGAAATGGAAAAGTATTGGTGGCCAACCCTTATTTAAGAAATTTTAGCCAATAAATTATAAGTAGTGCACGCATTAACATTCTTATAATACCAGTTATTAAATGAATATAACTAATGCCCTTTATATCAAAATTTCACTTAAACCATTTTGCAATCATTATTTCAATTAATACTATCTTTTGTCCAATCAATCCTCGTAGTAAATAATAATAATAATAATGTACCAGTGATTAGTCAATCTCCTTAATTTCTTATTGATGGAGTATTTCCACCTCCGGTAATTATTATAGCTTCACTGTTTTCTTCCTTTTTATATAATTATTGTAATTATTTAAAAACCCTTTCTAAAATGAATCCGGTAATACATTAAAAAACGATAGATGGAATAGTTATTTTAAAGGTGATAAGAAGAAAAAAAATAAAAGAAATTAGATCCTTCAAAGAACTTTAGAATAATAAGGTGAAAGGAAAATTGCTACTCTTCAATCATTTGTTTTAATTTAGTTGCAAGTGAATTGCATTGCCTAATGATGATTCATTTTAAGGATGATGTTCCTTATTTTGACACTTTATAAATGATATAATCGTTATTTAAAATATCAAATTGTATGCTCTACTATTTTCTATTTAAATTATTCGTATGAAAAATCTAATACCTAAATTCATTCCACTTTGTTTATCTACTATACATGAAGACATGCAATATGCCAATAATATTCTATGGGTGACTTGAATGCGACCCAATAATGTGGATAAACACGCGGAACATCAGAGTTAGTTCAAAGTTCAAAGAATAGTACCCAAAAATTCTTTATTTTAAAATACAAATTAAATATATCTTATTTTGATATGAAGATAAAGATATATATGATTTTAAATAATTGGAGCATGCATAAATAAGAAGGCATAAGGTGACGCGCATAGAAAAAGTCGTAGCACGAACCAATGAAATCCATGCACCTAAATCAGAAACGACAAGAAACCTCAAAGATAAAAGGGGCATTCATCTCATTGCAAACATTTCAAAC

## >AiNAC14

CAAACAACATACACAGAAAAGATCTTGAAGAGATTTTATATGGATAAGTCACATCCATTAAGTACCCCAATGATCGTAAGATCTTTGAATGTGGAAAAGGATTAATTCCATCCTAAGGAAGAAAATGAAGATATCCTTGGTCCTGAAATACCATATCTTAGTGCCATTGGAGCACTAATGTATCTTGCTAATAATTCACGTCTCAATATATCATTTGCTGTGAATTTACTAGTAAGGTATAGTTCTTCTCTAACCAGAAGACATTGGAATGGAATTAAACAAATCTTTCGATATCTTCGAGGAACGGTTGACATGAGATTGTATTATCCATATGAATCCAAGTCACAATTAGTTGGCTATGCAGATGCTGGATATTTGTCTGATCCACATAAAAGAAAATCTCAAAAAGGATATATGTTCACATATAGTGGTACAGCTATATCATGGAGATCCACGAAATAGACGATAGCAGCAACCTTCTCTAATCATGCTGAAATACTCGCGATACATGAAGCTAGTCGCAAGTATTTTTGGCTCATGAGTTTGATTCAATATATTATGTCATCATGTGGATTGATTGATCATAAGATAGCTCCAACTGTCCTGTTTGAAGATAATACAACATGCATTACTCAACTTAAATGTGGATACATTAAAGGTGATAGAACAAAGTATATTTCTCCCAAATTCTTCTTCACTCATGATCTTCAAAATCAAGGGACAAGTGATATCCAACAGATCTGTTCAAGTGACAATCTGGCAGATTTATTCACAAAGTCACTCCCAAAATCCTCCTTTGAAAAATTAGTACATGAGATTTGGATGCGCCGATTTTGAGACATTAAATGATGTCGACAAGAGGGGGAGACTGTACTCTTTTTTCCTTGATCAGGTTTTTTTTCATTGGATTTTTTTTGACAAGATTTTTAATGAGGCAGTCCCCATCAAAGAATATTGTACTATTTTTCCTTTACTAAAGGTTTTTTCCCATTGGGTTTTTCTTTAGTAAGATTTTAACGAGGCATAATACTGAATGGACATCCAAGGGGGAGTGTTGTGATGATAAGATTAAAATGGTCAAGATGGATGCCCATTAATATGGGAAGCTGGTTTTATTCTCAATACTTAACTTGTCAAGGCAAACCAAATTAATGAAGTTGAAAAATGTAAGACTTCCTTGCCTATAAATAGAGAAGCAACTGAGAAACACTATACACAGCAATAAACAATTCTCCCTCTTTTTTACGTTGCAATACTTCTTTCTCTCTTATATACATCACAATATATAATATTAGTAAACATATTAACTATCTCTATTATATTGAGATAGTAATTGTGATAAATACTACTACTAGAATTATCTAATTATACTTCTTTATTTTATTTTATATTACCTCATCCTTATTTATTTAGTTGTTTTACAACAATTTTATCAATTTTTTACTCTAAAAGAACTTAATCCTTATTTTGTAATGCAACTTAAAAGCTTTTAGCCTTTGGTCATTAAGTAGGGTCTATGATGTACACAACATGAGATTCGTAAATANNNNNNNNNNNNNNNNNNNNNNNNNNNNNNNNNNNNNNTAAAATATAAATTAAATTTTTAATATCTTATTTTATTTATATAAAGTATTTAAAATATTTTTTTGTTTTAATAAATAATAATATATATTATTTCTAAATTTATTACAACAATATACATGTTAAGAATAAAGTTGGACATACTGACACATGATGGTAATTAGGTGTGTCCAAACGTGTCTGATGAAAATTTTTTTATTTTTTATTAAGACACTATTGAATACGGCAGACACGCGTGTCAGATGAGTGTCATGTTCGAAATGTGTCCGACACACAAATACGACAATTCAGCGAAGTATCCGTACTTCATAGAGTAGGGTTGTTAAACGGATTAGTCTAGCCATTTTAAGTTTAGTCCGCTAAGTCTATTTATTTATGAGTCATAATTTTTCAGTCTAAACTATTTATAGTCAATTCAATAGTTCGTTTAACCTTTTAATTTATTTTTAAAAAAATATTTTTTCAAAAAATATAATTTTTAAGTGAAAAAAATTTAAATAAATATTTTGTTAGTTGATAAATTGAATTTTTGAATTGAATAAAAAAATATTAATTAAAAATATTTTTTTAAATGCAAATAAATTAAATAAATTGCCTGTTTAATCTTTAGACTAACCTATTATTTTTTTTGAATTAACTCAACTCAATTCGTTTAATCCAAAATCCGTTTAACCCAAAGTTTAGATAAAGTTAATTTTAGATATAAGTTTGCCTTTATTTTATTTTTTTTCCTTAAATTTGCCGTGTAAATAAATAAACAGTGCCAATAAATTATTACTCAAATGACATAATTTCTATGTTTGGCCAATTTTAACCGTCCTAACCCTGCCATTAAGTACTCCATATGAAAAGTGATTGGGGTAAACAATGCAAGAATCTTTAACGTTACTTTATATTTACTGATTTGCCCTTTTTTAGC

## >AiNAC15

GCTTTCTTGTGGGAGACGAAGGAACGACATGGAAGAGAAGAACTTTTTTGAGTTTTTTGGTGGGAAACTTTGAATGAGGAGGGGGACCATTTCGATTGTTTAATTTGAGGAGGAAAAAGTCAAACGACGTCGTTTTGCATCATAATTTCCTAACCCTTGTGAACGACGTCGTTTCATTCTTGCTCAATGGACAGTTAATATGATAGTTTATTAACAGCCTATTATATCAGTATCTCACTAACATCAATTCAATGAAGAACTAACGGAAAATCACGTTTTATAACGTTAGAAATTTATTTGATAATTTTTTAAAATTAGGGATATAATTGAATCACGATTGAAATTAAGGGATTAAATTGGACATTTCTTCATTATTTGATGAATGTCTTGTAGATAGTATAGAAATTGTTCCGTTTGTACAAGAAATATATTTTACATATGATAATTTTTAGGTGTATTACGAAAAAATGGTATAATTTCATTACTTTTTCATATCTAGTGCATCTATATGAAAATTCATCACGGATGAATTTTTTTATGACAATATTTTTCTAACGTAACGTTTTTAAAATTCATCTTTGTTGTTTTCTATTTAAAAGAAAAGTGAATCTTTCCTAACAAATTCTATTTTTCAAAAAATAAAAAATAGAATTCGCCCTTACGGAATTGTTAATTTTGAAAATGTTTTCGACAATTTTGGTTTGTCTCTTGTATTTTAGAAAATTACAAATAATCTTTATTTTTTGGGATTTCATCCCTCCGCAAACATATTAAAATTTTTTAACCAAATTACTTAATTTCTAACATTTTTTTAATTTTTTTTTCTTGATGAGATTGTGATCTCCTCACATCCTATTCTTAGAAGTTATATTAATAATATATATATAATAGATTATAAATAGTGACCACCATAAAAGAAAAAGATAGGTTGCTTATCATCTAAACAAATTAAAACTTTGGTGGAAGAGAGATTTCAAAGTAGAATTAATAGGTACAGGAAAATATGAAAAGAATGTCGTCACTCTTGCTAGTTGTGGATTGATTTTGATATGTCATGACATGCATGCCAATTTAATATATAGATAGTGTTTTTCATATGGCGCAGACCCCAATCTTTATCTTCCCTTATTAGTTGAAAATTTAACTTATTTCATAAAAAAGAAATTGGTTCTCTAATTGAATCCATATTATATTATCAATGATTATATATATATATATATAAGTTTCTCTAATCATCAATTGACCACAAAAACAATTTTTCTTTAAAAAAAAAAATTGTGCTACCTCGTAAAAAATAAAATTTTTACCAAACCGAATCTTACTTATTTAATTTCTTTTTTTTTTCATATTAACTACATGCATAATAGTTAAAATCTCAATTTAATTCTTAAAAATTTTTAATATTATAATTTTAAATAATTCTATTGATTTTATTAAATTTATACTAAATCTGACAATTTTATAATTTAAATCTTGTTAAAATTTTACGTTTTACTTCTTAATTTATTTTTTCCATTTCACATAAAATCTCGATTTTCTCTACCCGGACCACATGTATATCAACTATCAAAATGTACTGTTGTATATGTTCATATAATATGACTCAAAATATTTAGTATTAGTTCAAAAACTATCATTGTTAATTGAAGTATTTATTTTTGGAATTAGTTAAAGAGTGTCCCGAGGATATTGATTGAAAAAATTAAAGTAGTAATAAGTTTTTACACTTTTAATACATTAAAGTTTAAAAAAGTTCGACTTGCAATGAAGAGATATAAATGTCAATTAAGGCTGCTCAACAAAATACAGTGGTGAACCTAGTCAATAATAAAACCAACGCTATACATAGATATTATATTTTGGATGAATACAATTTTTACTTTTTTGGTTAAACGGATGAAATACATTTTAATATACATTTTCTTCAAAATTTATTTATTTTGGATATTTTCTTTGACTTTTTCCTTACATAGTTATTTATGAATGTACTCTATGTCCCACACCATCCAGATTTTTAATAGTCAAAGTTGTGCCCACTCTCTAGACTTTGTTTTATTTGTTGTTGCACTAAAAGCTAGCCATGTTTTACAAATTCTAGTCAATTGGATTTTCACCTTTCATTCATTCTTTCTTCCTTCTTATTATNNNNNNNNNNNNNNNNNNNNNNNNNNNNNNNNNNNNNNNNNNNNNNNNNNNNNNNNNNNNNNNNNNNNNNNNNNNNNNNNNNNNNNNNNNNNNNNNNNNNNNNGTAATTTTTTCAAATTCAATTATTATTAATTAATTTAATTAACCTTTGGTGGCTTGATGAGACCCCCCTTAAAGAAAAATCGATATATGTAACATTAGGTCAAAATCTATTGTAAGCTATAGAACTCTCTTTTAATCAAGGCCATGATGGGGACCCCCAAGAAATGGAAATGTTTGACTTGACTTCATCACCTAAATATTCAATACATTTACACACTATAAGAAGATATGAAAATATGTTAATATAAATATA

## >AiNAC16

AGGTGGGTTGGTGTGTGGAGAAAAATATATACATGTGAGATGTTGTACTCATGTTCTTAATTTGATAGTGAATGAAGAAATTAAAGAGCAACAAACTTTAATTGAAAGCGTTAGAAATGCTGTCAGGTATGTTAGGTCCTCTCCTCAATAGACTAAAAAAAATTTAAAGATTGTATTGAAGCTGAGATAATTGAATCTAAAAGTTTTGGGTGCTTGGATGTTTCAACTAGGTGAAATTCTACCTATCTAATATTGGAACATGTCGAGAAATTTGAACAAACTTTTGATAGACTTTATAATTAAGAATATGATTTTCTTAGATGATTTGGAGAGAAAAGTGGAGGAAAAAGAAAAATTAGTCCACCTACACCATTTGATTAGCAACATGCTAGATTATTCATTGAGTTTGAGAATCTTCTACGAGATAACTTTGAGTTTCTCATCTTCCTTGCATGTGACATCAAACAAATATTTTCATAAAATCGCATCTATTGCTTCTCAATTAATATCTTGGAGGCAAAATCATTCTAAATTATTAGGAACTATGGCATGTTCTATGAAAAATAAATATCATAAATATTGGGACAGGTTGACAAATTTAACCCATTGTTGGTTCCTGTTGTTGTATTGGATCTTCGGTACAAACTAGATTATATCTGTTGGTGTTTGGAAGATGTTTATGACAAGAAAGTGTCTACTAGTATGACTGATTTTGTTAAACTCATATTGGATATCTTATATAAGTTTTATGAAAAGGACGTTGCAGATGATAATGAAAGGAAGGATGATGAAAGTTCATCTAGAGATGTCTTAGATGACAATACTAAAATCTCAGCTAGTGTCAAGTATGTTTCTGAAGATAGAGTGAATATGTGAAAAAAAAAAAAAAGAGAGAGAAGGCTAATGCAGATAGCAAGTTAGATGTGGAGAGATATCTGGTCGAAGATACTGTAGAAAGAAGAGAATTTTGATATATTGGCTTGGTGGAAAGTGAATGCTTCAAAATACAAGATTCTTTCTCTTATAACCCGTGATGTCTTAGGTATTCCAATTTCAACTGTTACTTCTGAATCATGCTTTAGCACCGGTGGACATGTGCTTGATGCCTTCTACAGCTCTTTGTCACTGTTACTAGCTAAAGCTTTAATATGTACTCAAAGTTAGTTGTGTCCTTCTAAACAAGAAGTTAGAGATCAAGAATTTAATCAATTTGATTATAGTCAGAAAGTTGTTCAAGATACATTTATGATATTTTTATTATTTGTGACTTATTTAATTCAAGTAATAATCTTTTATATAAAAATGAGTAATTTTATATGTTTTTGAAGGTTTTACTAATACATCTATATCACAAGGAATAAGTTGACGACATTAATAGCAATTGATGTATGATGGCTATTTTTTTGTGGAATTTAAGTATTGTAAACATTAATATTTTGTGTTATAAAGACTATAAGTTAAATTTCTGTATTTAGGAAGTATAATACTTTAGACTAATGCATAATATTTGTGTTATTTGTATGTGACTTAAATATTTAGTGTTATTAAACAATATTAATATTGATTGTGGTTATACTTTAATTTTAGAAAATTATTGGTTCTTGTTATATTTTTCTAAATAAATTTTACCATGTGAAATAATGGGTGGAGATTTAAAAATTTTCACATACTAACCTACAAGAAGATATCAAAGTAATATGTTTTGTCTTAAACTTGTGTTTTCTACCTAATATTGTGTAATATTAATTATACATTGATTCTTTTATTTTTAATGAAAATTATGCAACTAGAAAAGAGATACAAAGATGCATGAAATTTTGATTTATGAAGAGATTTAAGTTGAATATTAACTATCAACTTCTTCATAACAACTAACAAGTAAATTTTTATCTCTTTTTATAAATTATTACTAAAATTCCGATTTTTACTCGATCTAAAGATGTGTATGTTTCATCGAGTTTAGAATCGTATTAGAATATAAAAAACACGCCCAATATATCATTTGGATCATATTTGAGTTAGGATAAATCTAATTTTCCTTGGTCCATATACACCCTAGTTAAATTATTGATAGCCAAAGTAAAAATTTATCAAATATGTATCCGAGTCAGGGTTCTTTGTTGGATATTTTAAAAAAAACCTAACTTAATTTGACTCATTTGTTAATGTTAGAGTTTTATTTGCTACATAAAAAATTTTATTTAACGTATTATTATTTTTTATTAACATAAAATGCTCAATTTACAAATGTATATTTTTTTTATCACGTTTTAAATAATTTGAGACATTTTCATCAATAACAAATTTTAAAAATATTTTTGTCATTTAAAAAAAATTTAGGTATTTTTTTTTTGGTTTATCCTATTAAAATACTAGATGCAATGATGAATATACCAAATCAAAATCGTGATTTGCTTGTGCGAGATGCCACATAAGACCTAATGTTCACACACAAAAGCACAATCACATACACCCAAGATCACAAAAACCCAATGCTCAC

## >AiNAC17

CGATGTTTCCTGCACACTGCATGTTTGCCTCTATCCGCTGAGTGGCAAACTTTGAGAAAGTAAATCCAGCATGCTTGCACTGGTAAGCCTCAGTACTCTTATGTGTAAATAACTCGTTCAACCGATAATATGTTGCTCGGACTAGCGTCAAGATAGGAAGATTTTGAGCACCCTTCAACACCGAATTAATGCACTTGACATGGTTCGTCGTCATGTGACCCTATCGATGTCCCTCGTCAAATGCCAACACCCACTGAGGATATTCGATGTTGTCACACCAATGAACATATGCCTCGCCTCGTTCTTGCAACCTCCTGTAGTTGACGTTGTACTCCTCCACTGTCCTTGAATACCCAATATTGACCACAAACTTTTGCAGGTATGGAACCTTGAATTCCCTCAAGAAGTTGCTACCAATGTGCCAAATATAAAACATCCACCATACTCTCGGACGTTTCCAATCACCTCCGCTACGATCTACTGCGGCTCGGATTGACTCACGATGATAAGAGATTATACCCACGCATCTTTTCTTACAACATGTGTTCGTAAATTACTGAGAAAAAAGTGTCACGCATCAGCAGTCTCACCCTCTATAATGGCAAAAGCAATCGGTACAATATTTTTATTCCCATCTTGCGCAATTGTAACCAAAAGACAACCTTTATATTTTCCGTATAAGTAGGTCCTGTCAACCTGAACCAGCGGCTTGAAATATTTGAAAGCTCTTATGCATGTATGGAAACTCAGGAATACCCGATGAAGTATCTAAACACCGTCCACCTCTTGACTCCCGTTATACAGAGGTCGTGTTTGTATTTGGACTTGTGAACCAGGCATCTTCTGAACTATTGCCTAGAACCACAATGGTAAAGTTTGGTAAGAATATTCCCATCCACCGAAAACCTTGGCTATCGACTTCTACTTTACCAGCCAAGCCTTCCGGCAACTAATAGTGTAGTTGAATCTTGCCTGGACTTTATAAAATTGACCTTTATGGATGGGTCGGATTCAACCAATGGCTTCATAGCTTCAGCAATCGTGTCCAAGTCTAACTTGGAGTGATCCCGTGAGATCGTTCCCATGGAACACGTGTGCCTCCCGTTGTTTCTCCGAATCTTCCAACAGACTTTCTTCTGTATCAAGCTGGCTCGGGTAAGCCAGTCGCACCCACGTCCATAAGTCTTGCACTTTGCATAGAACGTCTGTGGTTCGGATTCATAAACAACGTAATCAACTCCTCTAGAGATAGTGTAACTCCGAATTGCCGCAATGACTGATTTTCTTGAACCGTATTCCATTCTGATTCTAAACTCTCCATCCTCAGGATCAGCAACACCTATAAGAAAATAAATTTGTCACTCATCGATCCGCTAAAACAAAATCAAGTAAACATACTACCCACTGCTCACGTACCTATGTTTGCATATTCAGGAAACTCTGGTGTATGCATGGCGTCAAGATCCAAGCTACGCATAAAAGGTGGAACGTCCATCGGTTGACTAACCACGACTGGAATCACTAAAGTTTCTGCCACTGCATCACCTCCCCCATTGCCGTCCTCATCCTCATCACCGGCTTCATACGTAGCTTCAAACTCCTCATCGCTATCATCGTTCATTCCCAAGTACGCCTCAGCTTTGTCATCTGGCACATCTACGTCATATTGAACCTCATCTGTAGCAATATGCTCAAACTCAACGTATAACTTAATCTTCGGGTGTTGCACCTGAGTTTGCTAGTGAATATGAAACATCCGCTGAATACTAATTTTGTCGACGATTGGCATATCCTCAAACTGTATTAGGCCGCCAAATACGACAACTGGACTCCTATAGAGAATGTTGGTCACCCTCTTTAAAATATCAGCCTCTATGCTTTGACAGAGCCCAAACTGTAGTTCCGCGAATGTTATTTTGCACAGAACCACAAACGAAAACATATTCTCATAAGCAAAGCTCGCACTCTCATATGTATTTCGTACGACTTCACTATTGTGATAAACCACTATATTTGCAGTGCCCTCTATCGTACCAACCCTGGAGAAAAATATTTCTCTCACAATTAAGCAAAATAGTTACGAACTTTGGTTTTTATAGATAGAAGACTCATCTCGCAGGCTACGTGTTGGAACGCCATCCAACCAGCGTCTGCCACGTGTGAAACACATTACCTGTATGGTGAAGGGATGAGTGCCACATGGCAACATATTACCTGCGTGTTGTGTGCGATTCTGACACGTGTCCAATATATTAGTTGTGTGCTGAGTCAACACATTACCTGCATATTGTATTTGTATATTTGCACTGTCCATCCACAGTATATACACCAAAAATTTCCATTTCCAGGTAAAACTGACCTCTAAATTCCATATTAAAATTCTTTAGCATTTGGAAGGAATATTTGTATTTCAATTCCATGGAAATTTACCTAGGCGAATGGCGAACAATGAGGCCCCTCGCACAAGAAACAGAAATTAATAAAAAAGAAAAAGGAAAAA

## >AiNAC18

CATGTTTAGTTTCCACAAATACCAAAACCGTTGACACCAAACCTATTTTGCTGGTCCATATTCATGTCTCTATATATATGAATACTTGGATAGATGCACATGCACTACACATTAGAATTATTTCATTTCTTAGTATATATCATTTATTTTTGGTTTTTTGCCCATGATCATTATATGGCCTTATTTAAATTAATTTTAGAAGAGTTGTAGTATGTGTTATATGTGTATGTAGTGTTCACTCTTCTCTTCTTATGAAAATAATATTTAGTTTTATTTATTTTACAAAATATAATATTACTTGTAGTGTTAAATAACCTCCTAATTTTGTGATATTGATGATTATTAAGATTACCTCAACATCATGTAATTAAGAGAAACCGGACATTTGTAATTAACAACATATAGGGCCTAGCTAGCTAGAAACTACTAACTATATATCACAATTCTCTTTCTCTCTCTTGGGATTCAAGTCATCATTCTCACTAATGCATGTCATGCTTGTCACTTCAATTACACGCTTGTTATTATTATTATTATTATTATTATTATTATTATTATTATTATTATTATTATTATTATNNNNNNNNNNNNNNNNNNNNNNNNNNNNNNNNNNNNNNNNNNNNNNNNNNNNNNNNNNNNNNNNNNNNNNNNNNNNNNNNNNNNNNNNNNNNNNNNNNNNNNNNNNNNNNNNNNNNNNNNNNNNNNNNNNNNNNNNNNNNNNNNNNNNNNNNNNNNNNNNNNNNNNNNNNNNNNNNNNNNNNNNNNNNNNNNNNNNNNNNNNNNNNNNNNNNNNNNNNNNNNNNNNNNNNNNNNNNNNNNNNNNNNNNNNNNNNNNNNNNNNNNNNNNNNNNNNNNNNNNNNNNNNNNNNNNNNNNNNNNNNNNNNNNNNNNNNNNNNNNNNNNNNNNNNNNNNNNNNNNNNNNNNNNNNNNNNNNNNNNNNNNNNNNNNNNNNNNNNNNNNNNNNNNNNNNNNNNNNAAGAATTGAACTCTAAATTTTTAGATTATAGAAATTTAATATCATGTCATAATTTTTTTTCCAAAATTTAAACCAATAAAAAAAAATATACGAATAGTTATATCTATATCCATTTTTAGTTTTTTTTTCCTAATCTAAATTAACTGATTTTCAAATTTTGAAAACGGTAAAAGGGATAAAAGCAGAGAGAGAATTTTTTTTTTTTAAAAGATCATTACAACACAATCACAAATATGTGGCAAACACATTGAAACTTCAAAAGTGCCATTGTTTAGGTCATCTTTATCTGATGGGGATTGGAAACTTGAAAATTGCAAGGTCATTGATGAACAAGATGACATACATGAAAGTGATTTCACACACAAGACCTAATTATTACTGAGATAGTGAGATGATCATAATTATATTCACATGGAAACAATTAATTCTACAATACGATAACTAATGTATAGTATCTATCAAATTATCTATATAAAGTATGTGTCTTCTTGTGGAAAATATTTCTTTCTTCATCTAAATTAAATTATGTATGTATGACTTGATTATATATGTGCATATTTATTTTTTATCATCAATATTAAATTTTTATGTATAATTTACAGTCTTACACATAAGAGCATCTTTTATGATTTTATTCTATATATTATACCTATTTGTCTATCTTTTTTTGTGAACACTTCATATGTTAATCTTTATCATTGATTATTAATACCTTTTCTGAATCCCCTATTTAATTTTACTGTTGAGATATTCAATCATGAAAATCACCTATGCATGACAAAATCATATGGAATTTGCATAAAAAAAATACTCTATTAATTACCGTATGAATTGTCTTGTCGAATTCATATATTTTTGTCCAATATCCTAAATTCATACATATCTTTTTATCAGTATATATATAATATACGAGATTTCGACAAAAATTTTTTCGTATGATCTTATCNNNNNNNNNNNNNNNNNNNNNNNNNNNNNNNNNNNNNNNNNNNNNNNNNNNNNNNNNNNNNNNNNNNNNNNNNNNNNNNNNNNNNNNNNNNNNNNNNNNNNNNNNNNNNNNNNNNNNNNNNNNNNNNNNNNNNNNNNNNNNNNNNNNNNNNNNNNNNNNNNNNNNNNNNNNNNNNNNNNNNNNNNNNNNNNNNNNNNNNNNNNNNNNNNNNNNNNNNNNNNNNNNNNNNNNNNNNNNNNNNNNNNNNNNNNNNNNNNNNNNNNNNNNNNNNNNNNNNNNNNNNNNNNNNNNNNNNNNNNNNNNNNNNNNNNNNNNNNNNNNNNTTTACAGTGTAAAATTTTTTATTCGATCTATAGACTTAACGATTTTTGACCACTTAAATAATTAAAAATTTTAAAATTGTAAAAAACTTTTTTTATCTTTAAAGGAAATGATTAAGAAGTGAAAATAATATTTAGTGTAATTTAAATAAAATATAAGATTTCTCTTTCGTGATACCTACCTTACTTAAGTGATGTTGTTGTCCACATATGCTAGAACCATAAACTGACCCATCATATGTAATGTATTTTCATGC

## >AiNAC19

CACCAATGTGTTATAGTATATGGTATATATTAAAAGCTGGTGCATGCATTGGAACATCATGAATTACCCTATTGAGATTAGATTTTATATGTAACAGTAATAATTCTCTTGTTTTGGTTGTATTCACAATGGTACAAAACAAAAGAGCCTCTCTATTCCTATCATCAGAATTTATTAATATCTATACATACATACATACTTTTTCACTTTCATTTTCCTATTTTTGATATGCCATCTCAAAATGATGATAACTATTGTTGGGATATTATGTATGTACTTTGTGGAAAAAAAAAGGGTAATTATTTATTTTATTTTTGCCACCACTAGTTATAATAATTATGATACAACAGTTAAATGGATCAATCAAGCACCATTGATAGGTCATGCAAATCCTGCTAACTATCATGGTAATTCAGAGTTACTCAGCAATTTTTCAACCCACATGGTTATTTTTACTGTGAAGAAAATTATCATTTTCACTTCAAAAAGGAAAAATAAATTGACTCCAAGAGAGAGAGAGGAGGAGTGTGTGTATGGACTATGGAGAAATAATATAATGTTTCTTTTTCTTTTAACCCAAAAATGATTTTAGCAACGGATACCGATAAAGGTTTTATTAGGACATGAACATGCCTATAGTATGATGGGAAAACGAGGTGGGGAAAACCTATGAACTAAAAAGAAAATAAATTAAGCCTCAAATTTATTTACCTAGCTTTCAAGAATAGATTTATCATCTATTTCTTTTGTCTTATATATGTCACACACTGTACCACTACATCATCACCATCCCTATAAATTACCCTAAATTTACTTCAATTTTAGTAGAAGATGTTAATTAATACTATATTATACCAATAATATTATTATCTAAAACTCAAAAGTTAGTGTAGTTTTTTTTCAAATAAATAATAATATTATAAATATATAGTTAATTAAGTAATTATTGTCTAGAAGGTTAAGTGAACAACATTACTAAAATAATAAATAGAGTGGATAGTAAAAATTATTCTCTTATGAGTTGTTATAGAAAGCGAATGCACTTTCTCCTTTGAGATCACAAACAATTCAAGTAACCAAAAACTGAAATAATCATGAGAGTTTATAAGACAAAGAAAATAGAGAATAGATTTAAAAAAAGAGTGGTTGAGATGGAGAAAGGGGGAAGCAGATGAAGAGTAGTACCAAATCTTACATAAATAAAAAGTGAGGGAAGTTGGCGTGTAATAAACATGACTTGCTGTTTCTGATTATATAATTTTAGTACTGTGTACTGACAGGGAAGATATGAAACAGCTTTATCACAATATATATACATATACACATATATGTGCATGGTGAAGGAACCTCATCAATGATCAATATTTTTTTATTTTTATTTATTTTTTCTTTTTTTCTATTTTCTTTCTTTCGTATTAAATCCAAAAACACATGTCTTCCATCAGCTTTTGTTAGTTTATATTGTTATTATTTGAGTTTGAATTTCATAAATAAGAAATTACTTATACCTTTTTTATATTTCATTCAATGGATCTAACGAAATAGACAGACTTGTGACACGTGCTTCTTTTTCCTTCTTTCTTTTTTTTTTCTCTTTTGAAACTTAATTATTATAATCGACTCCTCTTTTATCATAGAATAGTGATATTGGTATAAAAATATCCGAATCTAAGTATCAATTAATACCAAGCATAAAATAGTCATAAAATTCAAAAGTCAAAACGTTAAAGTGGGAATGATACAAATGAATAAATAAATAAATATCAATATGATGGACAAAGGCTTACTATAAAAATATTATTTTTTTAATTGATGGGTCCAACATATAAATCAATTAGATTATTTTACACCTTATTAAATAATAAAAATGAATAAATGGACCTACTAAACCAAAAACATATAATATGAATATTAAAAGAAACATTTCTCAAAAAATAAGTCTATATTCATCATTCATTATAACAAACTTTAACTAATTTAGTTTAATTGACTGGTTTGCTCATCTTTTTATTAATTTAGTTTAGTTGAATGGTATACAATANNNNNNNNNNNNNNNNNNNNNNNNNNNNNNNNNNNNNNNNNNNNNNNNNNNNNNNNNNNNNNNNNNNNNNNNNNNNNNNNNNNNNNNNNNNNNNNNNNNNNNNNNNNNNNNNNNNNNNTTTATTTTATATTTTAATATATATTTTATATTAATTAATAGTTAATTTTGATGTACAACTACTCATAAGGTTGTAATTATTATTTTCCTAATTAACCTTGTTACTTAGAATGATTTACAATTTGTACAATCTTGTTATTACTAAAAGCAAGTCTAACACAAACATTTTGACATGCAACCTAATGAAAACTCATTATTGGATCAAAAGGCAAATCTAGCAAACATTTGATTCACCATGGATGATCATAAGTAAAGAGACATATAAAACCTTGGATGAAGATAGAGTATGTGGAATGAAATCCAAAATTGTTTGAGTGATGTAGGTCTCGGCTGGTTCACTCT

## >AiNAC20

GTAAGAGTGCTCACAAAAATTTGATGCACAACATATAAAAAATTTTGAAAAAAAATAGAATATTGATATCTATCCAACAAAATAGGCATATCACAAAAAATTGAGTGTACAACACAAAAAATTTTGAAGGAATACATGTTTAGAACATTAAATTACTAAACCAACAAAATACATTTGCATAAAATTTTTCTGTGCTGTGTGTGCAAAATTTTTTGTGCTAGGAATGTGTGAAAATTTTTATGTTATATCAATGAGTTAGATGCTATGCACAAAAAATTGTGTTATATGAAAAAAATTATGTATTATATCAATAAATTTATGTGTTCTTTAATAAAAATTTTTGTGCAAAAGAAGCACAAAGAAAAAGAGGGAGTAATGAATGCATGCATTTTTTGGTTGGCTTTATGCCAATTTAATTGGATTTGATTACAAAAATACTTGAACTCNNNNNNNNNNNNNNNNNNNNNNNAATCGTTAATTAACCTTTATTATTAATATAATTAATTTTAAAAACAAAAAAATATATATGAGTATCATTTAAATATATCGAAAAAGAGAAGAATGTCATTTTTTTTAAAGCTATTTAATTGGGTTGTAAACGTATATAAATGGTCATATTGGTTGTTATCTACATGTTTATTGTTCTGCCTTCGTCAATTACTCTCCATGAAATGTTGAAGCTCATGTGTGAATAAGCGACATCTGAAAGATGATTGCATTCATTATTTATTTGTTAGAAGGCTTGTGATGAGAAGATTCCTAATTATTAAGTATTAACCATGGTATGTTCACTATGTTTGTTCGAGAGAATACACAGACAAGTAGAGCAGTGATATTTGGTGATGATATTAAAGTAAATGAAGAGGAAAGAAGTTGCCAAGAAAATAATGATTTTTGTCATATACGACAATTTTTTAACAGAATCAATGTCTCATAATTGTGTCAAACTAAACATGTCTTTTAAAATGTAATTTTACTTTTTACATGATTAAAATTGTTAGTATTTTATTCTTTGAAGCAGAAAAATATTTTCTTTTATTATAAATAAATACACAACCATTTAATATAATATTATTTGCCCAATACGTTGAAATAGAAAATCTTCAATAATAATACAATCGAAAATATTACTCAACCCTCATTATTAACTTTTATTATATCATAATAAAACCACATATTATCATATTCCATTGCATATAATGATGGATTTGGATCACATATTAAAAGAAAAAAAATTATGTATAAACATGTTTGGTTTTTTATACTTATTCATTCACAAATCATTCATTACTTCAAAGAAGATAATTAAAGCTAAAAGAATGAATGATGACAATAAAAGAAATGTGAAAGGACCGTTTGTTCAGTTAGGAACAGCCGCCAAAGTTGGTATCCTTCAGATTGGTCACGTTAATGTTCTTCTTCTTCCAAAGAATAGGGGCTTGCCACATGGATAGGCCCAGAATCCAAGAAAGAACAAATTAAACAAGAAGTATAGGATCAATGNNNNNNNNNNNNNNNNNNNNNNNNNNNNNNNNNNNNNNNNNNNNNNNNNNNNNNNNNNNNNNNNNNNNNNNNNNNNNNNNNNNNNNNNNNNNNNNNNNNNNNNNNNNNNNNNNNNNNNNNNNNNNNNNNNNNNNNNNNNNNNNNNNNNNNNNNNNNNNNNNNNNNNNNNNNNNNNNNNNNNNNNNNNNNNNNNNNNNNNNNNNNNNNNNNNNNNNNNNNNNNNNNNNNNNNNNNNNNNNNNNNNNNNNNNNNNNNNNNNNNNNNNNNNNNNNNNNNNNNNNNNNNNNNNNNNNNNNNNNNNNNNNNNNNNNNNNNNNNNNNNNNNNNNNNNNNNNNNNNNNNNNNNNNNNNNNNNNNNNNNNNNNNNNNNNNNNNNNNNNNNNNNNNNNNNNNNNNNNNNNNNNNNNNNNNNNNNNNNNNNNNNNNNNNNNNNNNNNNNNNNNNNNNNNNNNNNNNNNNNNNNNNNNNNNNNNNNNNNNNNNNNNNNNNNNNNNNNNNNNNNNNNNNNNNNNNNNNNNNNNNNNNNNNNNNNNNNNNNNNNNNNNNNNNNNNNNNNNNNNNNNNNNNNNNNNNNNNNNNNNNNNNNNNNNNNNNNNNNNNNNNNNNNNNNNNNNNNNNNNNNNNNNNNNNNNNNNNNNNNNNNNNNNNNNNNNNNNNNNNNNNNNNNNNNNNNNNNNNNNNNNNNNNNNNNNNNNNNNNNNNNNNNNNNNNNNNNNNNNNNNNNNNNNNNNNNNNNNNNNNNNNNNNNNNNNNNNNNNNNNNNNNNNNNNNNNNNNNNNNNNNNNNNNNNNNNNNNNNNNNNNNNNNNNNNNNNNNNNNNNNNNNNNNNNAGAGGTGTGAGACTGTGGCACAACAATGTTAGCTTTATTTATTTTTTTATTTTTCCTCAATATTCTCTCTCTTCTCTTTTTGTCCTCTTTATCAAAACCCCAAGCAAACCCCACATTCCAAGCCCCTCTCCTCTTTTGCTCTTTCATCTATTCTCTCCTTACTCCTT

## >AiNAC21

CGGAGACCCCTGGAGAAACTTTTTTGTATCCAGATAGGGGCCCTCCCCCACACTTCTCGGAGGAACCCCACAATGGCTGCCTCCACTTCATCCAAGTCATCCAAACTATATTTCTCACAGGGGGAGGCCTCTAACCAGTACAGAGGAAAGCGAGGAGAAGAATTCTCATCCAGAAAAAAGGGGTGGTGACCCTCTACAACTTGAACTTTGAAAAAATAATTTTTGAAATCGTAAAAGGATTCGTCAAAAAAGGTGAAGACTCTCCGACCTTGTATAGCTCGGAAAGACACCCACTGCTGTTTGTTATTTTTCCCACTAAAGGGCTTAGTCATATGAAAGAGGAAAAAGAAAATTCTCAGAGAAGTCGGAAAGTCCAAAGCATGGCTGATGAACTGGTAAATTTTCAGAAAATCCCAAGAGTTTGGGTGAAGTTGGGTAAGAGCAACACGGCAGTGACGTAAGACGGCTATTTCAAAATCAGAAAAAGGAAGAAAAACACCCAAACGGGTAATCATGCTCTCATACATATAGAAAAAATGAGGAGCTGCCTCAGCAGCCCTCGTGAAGCAAACCCGGTCTTCTGGACCCGGGACCATCAACTCGTACTTCGGCTCATCCTCATCAGAAGTACAAATTCTATGATGAGTATGGAGATTGGTAATAAAGTCAGTATCTACAAAGGATTCCTCCCCCAGAACAGTAACGTCCACCCATTGAGCAAGGGTTTCTATAGAAGACATTCTTTCCTAAAAAGGTATGACGAAACCTACAAAGGAAAAACGAAAAAGGATAAAAAAAACAAGGGTCTCTAAAGGGCCTGGAATCAAACAAAACAGAATCACTAGAATCACTAGAAGCCTACCCTCCTCTCTAAAATGAAAACATGCAAACGAAAGCACTTCATAAAAAGAGAGTAGAGAAAAACTAACCTTTGCTTGAAAAAAGGATAGGGGCAGAGAAATGTGAATTCCTTCGAACAAGAGCTTCTTCCGAACAGAGGAGAGGAAGTGTGAAAGTTTTCAGAAACGAAGCAAGGAAAAAGAGGGAAAGTATTTATAAAAACGTTGGGGGCATAATGGTAAAAACGGAAGCCGTCATTTAAAGAGAGGCACCATTCCCAATGTAACTGATCCCCGCGTATGAATACACAAATCCCTAACAGACGCGACATTTGATTAGACGTGACTGTTGAGAATTTTTAAAACACGTCAGTTCTGAAACATCACGTCGGTTCCTTATCAGGTCGGCTACAGTCCGGAGTTACATACTCGACCCCAACTCTTAAAAGAAATTTAGCTCGAGTAGGGGCACTGTTCATACCCCTGGCCCAACGCTACGACCCAAGTCCAAATGAAAGGCCCAACCCAAAGGATTGAGCCTCACCCTACACCGACCTTCCCTTGTAGAAGTCGGTTCTTGACACGACTTGCTCTAAAGAAGTCGGGGACGAAGATTGGTTGGCAGATAAACACTCATTCGAATGAGTAACTGTCCCTAAAATCTCTCAACCCACTTCCAAGAGCCATATCTCAACTTCCCTAAGATAAAGGGACAGTTATCCACCTTAAAAAGGTGGAACTACTTCAACGGTGGTTATTGGCTCACCACTATAAATACACTGACACCCCTCAGGTATCTCTAAGTCCCAATACTCTCTAGACCTGCTTACTCCCTTGCTAACTTAGGCATCGGAGTGTCTTTACAGGTACCACCCCCCATTCACTCGTATTCACAAGTCGGACGGAGGCCCAGGAACGTGATTTCCTTCGAAGGCTTCCCTCTTCAGACGATTGGGACAGCCGTAGCGAGTCCATCCTGTTAATCTCCGGTTACCCATCGTAACAGGTACATTGCCCTAATTTTCATATCATTTCATAATTTTTTATTTTTTTAGAAAATAAATTTTATTTACATTTAAAAAATAAAAATAATGTTTGTTTATTTTCTGTTTTAATTTTTTAAGATTATAAATAAAAAAATTAAAAAAAGAGCGCATCCGACACACACAAAATTTTGGGCTTCTTTTTATTTGTATCATTTGGATGTTTCTCAGACAGAAAGAGACAGACCTGCTTTTGAATTTGTGACATTGGTTTGAATCATCAAAAACCAGTCTTCGTTCGATGGCAGGGTATGTACCCTACTATGTTTGATCATCAAAATCCATTCTTTTATCCGCACATCGTTTCGTAGCAATACTATGTGAACTGAGAGAGATTATTTTTCCACTTTCATTCTATATTAGAAGTGGGGTTACCTTGTTCATGTGGAAAATTATACCTTTTTTGCGGCTTGGTGAGATTTCTTAGTTGTGTTGGTCGTCTTGGCGATTTTCTGTTCATTGATTGAAAGAAAAGTGAACGGATTTAATGGGATTAGTTAGTTTTATGGGCAAAATGACTTCCTTTTTTCTATTTTTTTTTTTTGGGATAAAATTTTTTACTTGGAGTTCGTTTCCACTTTGTTGACTGTTCTATGATCGAAAGATAAATGGATGATC

## >AiNAC22

GTCTTCGAACACACAAAAATTCCCATAACAAATGAGATTGCAGAGATGCGTTGCAAATTTAAAGAATAAAATGGTGTCTAGATTTTTCAGTCAGCATATAGTCAATAATCCTATTTTTCACTTCATAAATAAAATCTCCCCTTTTCTTGGGTTCCAAGTGAATCTTTTAGTTTTGGTTGGGCCTATATGAATCCTTATCAGTGCTTTTTGGACAGAAGTGAGCCCTAAACCTTAAATAAAACATCTATTGGGTTAGAAATATCAATAAACAAGCATTTGATGAAATATGTAAACATATGGACCAACTCAAAAGTCTAGTGGCTGTTAGGACCCTGACCCATTTAGAAGATCAAAGAAAAGAAAAGATGAGACCCATAAACAAGGGGCGAAACTTGTCATAGTGGGTCCCCAACTTGACACAGGGAACCGAAGTCAATGATGGCAGCTAGTGCGATTGGTGGAGAACTTTCGCCGAAGGTGCCATTTTAACTAAGCAACAAAATGTTCATTTCTCTCCGACCTATTGGCTCTCTCTTCTAGTTCAAAAAGATTCACACTTGACATGACATTTCTTAACCACGTTCATTAAACTATATTTCTTTCTAAAATAAAACAATAATTTATGTATTAGGCCATTTACTAATACGGCGGCTAGTAATTATAATAATATATATGGAACAGAACTCAATGGTACTAGCTAGTAGTAGTTAACTTGGTGGCCAAGCATAGAGGTAGTTGCCATATTATACTACTAGTATCAAGAAACACAAAATTCAAAGACCGAGGGAGATGGATATGGAATCCTTTCAAGCTCATTCATAATAAGCAGACAGATTTCTAGCATGAGCGTTTGTTATCTATTTTTAAGTCAATATAAATAGTTTGTTACCTAAAATTCCTATATGTAATGAGTGTTCATACTTCACTCTTGAATCTTGATCACTAACGCACGTACTTACGGAGACTAATTATATATTTTAAGGTAATATTGCTTGACAAAATTATTTCAAAATAATAAATTATTCTAGTAAAAAAACTTTCAATTCGTGCAAGTCAAAGGAGTTTCAGAGATAATTTGATACAAGGTGGGCCAATTGAAAAAGGAAAATAATGACAGCATCCTTGACCATAGATGGATAAAATAAAGGATTTGTTAATTGAATTCAAACCAAGGGGAAGAAATGTCATGTCTTACCTCAACCGTTGTTGGACCCACCCAAAATGTTTGCCCAAAGGCCTAGCTTAGGCCTTAGAGAAACAATCCTTAAGGTAACATCCAGGCAGCTTCCTCCCAAGAACAATGTCCTTGTCCATACTGCTAAGTCACACATCACGTCACACCACATGAACTTAGGTTTTGCAGATCCATGCATCATGCATGTTGATGTTACTACTGTTTGTGGATCAATTTTCTATTTCTAATCCCACGCACTAGTATAGAGGTTAAGAATGTTCTCGCGTCATAAAGTTCGCGGCTAAGAATATACCAAAGATATGGCATGCACGCTTAACCTTTTATTAGTGTGAGCTTTAATATGTCCAATGTCTTTGACCAAGTAATTCCTATGCTATTAAAAGCTTTTAATACAGAGGAAATTTATAGGAAGAGTTAAAAATTAGGAAAAGTATATGGAATACGTTGGAGAATTAGGAGCGGTGCTTCTAATAATCCAAATTCTTAGTATATAAAAAATTTATAAAATATATAAAAGAACATTTATTTAATATGAAAAAGAAATATTTTGATACTTAGTAAAAAAAACATTCTAATGTATAGCATAAACACTATCTACGTAGAGGTTTTGGATTTACTCAAAGACATTTGACTAATTTTTTGCTGGTACCCTCTTGGTTTCCTAACATTGTTGTAAAAATTAATATTAATTCTTAAAAACTTAAAAATGCCCTTAATATCAATTCCTTAGTTTAATATTTTATTGTATATCATATTACCTAATTAAAAGTATAGCGGTATTGGCTAGTTGAAGACACCACCACATACATAAGTAATGGACAATGGTCAAACCATTAATAATAGTGAAGGTTGAAGAAGAGGGACTTCGGCAATATAAATAAATAATCATAAGAGGAGAAATATTATTTCTTTGAGGGAGGGACAAAAACAAAAAGAAATTAGGTGGAGACATGGAGGGAAAAGGATTGAATCATTTTGGGCAATATAGAAGGAAATTCAACAAGGTGACATGGCTAACCACCTGTAATAAGTGAGCGCTGCCGCTACACTACACTACAATACAAGTAATGAGTTCAATCTTCAATCTCCATTTCAATTTTGTGTATTTTGAAGAGTCAAATGTTATAGTACAGGCGGCGTGACATGACTGGTTAGGAGAAGGGTGCAGGTTGGAGAGCACCCAGAATCCACATGATTTACAGTCAACTTGACTTAACCCTTTTCTTTTGCTGATCTAAGCAGTGACAGAATCACTATCCCTGCTTCCAAGTTGCAAGTTCCAAGTGCCAAATGCACACGTGTCAA

## >AiNAC23

AATAAAGAATTAATAGATCGAGACAGAAAATAGTGAGGAGTAATTTTATCTTTCTCTTTTAATTGCTTATATTTTCAATTAAATTTAATGAATATCATAATACCTATCGTAAAATTTAAAAGAGATACTTAATATAAAACAAAAGAAGTATAATGAATAGCTCGTAAGATTCTAAGATGAATTGAAAAGATTTTTTTTTTATAGTTAATTTTAGTGACCATTATTACCNNNNNNNNNNNNNNNNNNNNNNNNNNNNNNNNNNNNNNNNNNNNNNNNNNNNNNNNNNNNNNNNNNNNNNNNNNNNNNNNNNNNNNNNNNNNNNNNNNNNNNNNNNNNNNNNNNNNNNNNNNNNNNNNNNNNNNNNNNNNNNNNNNNNNNNNNNNNNNNNNNNNNNNNNNNNNNNNNNNNNNNNNNNNNNNNNNNNNNNNNNNNNNNNNNNNNNNNNNNNNNNNNNNNNNNNNNNNNNNNNNNNNNNNNNNNNNNNNNNNNNNNNNNNNNNNNNNNNNNNNNNNNNNNNNNNNNNNNNNNNNNNNNNNNNNNNNNNNNNNNNNNNNNNNNNNNNNNNNNNNNNNNNNNNNNNNNNNNNNNNNNNNNNNNNNNNNNNNNNNNNNNNNNNNNNNNNNNNNNNNNNNNNNNNNNNNNNNNNNNNNNNNNNNNNNNNNNNNNNNNNNNNNNNNNNNNNNNNNNNNNNNNNNNNNNNNNNNNNNNNNNNNNNNNNNNNNNNNNNNNNNNNNNNNNNNNNNNNNNNNNNNNNNNNNNNNNNNNNNNNNNNNNNNNNNNNNNNNNNNNNNNNNNNNNNNNTGTTTTAGTTTATACTTTATATTCATTAATTATCTCTGAAATAAATATATAATATTAAATATAATAAATAAATAATTTTAAATATTATATATATAAAACTATAAATATTAAATAAAATAAAATAATTTTAAATTATTATTTCCTTAGCATTATTCTTATGGAAATACTTCAGTGTCCTTTAATTTACGTTTAATGATTAAATCTTTAGGGTGTGAAGTTATTATCAACAATTATTACTAGGACTAAAATGAGAATGGTGTAAGTGTGAAAATTAAACTCCATAATCCATAAAATATATATGATTTCAAATTTCTATATTGAAACTGCAACAACTATGTATAACAACCCAACATATACTTTGACGCGTGTTTCTCCAAAGAAACTTTTCCTAATGTCAGCAGCAGTAGTGCCCATCATTTAGATTTCGCCATACACTTTTTTTAAGAGATCTGAGATAAGATGTCACTCACCACAATGATTTTACGATACTACAATTATTCTTTACGAGTTCTTTTACATAATGTAAGGAAACATAGTTTTAATTTGTGGCCGCGTGTTCGTTTCTATGTTGCAAACTATAATATCATATGAAAAGCTCATATAGACATGTTAAGTAATTTATGCTGCTTCTTTTTATTAATTTAATGTTTTGAGATGAATGATTTTATGATATAATATTAAAATTTTATATTCAAAAATTTTAAAATTTAATTTTTAGAAAATTCTAAAAATAAAAAAATAGATATATAAAAATCAAACAAATTTTAAAAAAGATTTTTACTAANNNNNNNNNNNNNNNNNNNNNNNNNNNNNNNNNNNNNNNNNNNNNNNNNNNNNNNNNNNNNNNNNNNNAAATATGTCTTCTACTTTAATTTTTATACTTAAAAAGAATGTTAGAAAAGTAACAAATTTTATGATTTGTAGTTATTAATTAATTTTTATTAATATTTTTAATGATGTAAAATTTTATTCAATAGTATAAAATTATTTATTTTTTTTAACGATTAAATACTAATCAAATTTTAATAAAAATACGTGCTGACCTTAAACTTTCTCTATCCTTAATTCTAAGACCTCGCTTATTTTATCATCGCAGCTTTTTATAAATACAATTCACCGTTAGCTTAGAGCAAACAAAAAAATTTATCGCAACCTTGTATTTCGATCTTACAAAAGTAGTAGTACTATGAAAAAGATTATCACCTTTGAAACAATATCCATATATATAGAAAAAGAAAAAGTTAAGCACCTCCAAATTAAAATATTTGTAAATGACGCGTTTATTGTGTAGCTTTGCATGCAAACGCGATACATAATTAACAAGTGGCAAATTGTTATTCCATTGTCATGATTCAATAATTATGCATGTTAAGTTTATAAGAAAAGAATTGATAATTAAATAATTGATTAACGTAAGCAAGAATATTTTAATATTTAAAGAAACACTAATTAATTCCCTATATATTTTCTTCTACTAATATTATTAATTAAGTTTCTATCATTTTCCAAAAAACAATATTGAATTTGGTGTATATCGTCATTGCAAAGTCAACTAACTTGTCATACATATTGTTGAATAATATTGCTGGAAATTAACTTTAGTGTGTGTGTATATATGTGTCTTAAAACCAAAGTAGTTAGCTTTTGTTGTTAGTTTCCTTGGCCGCCTTTAATTAACCTTGTGATATTATGACATGTT

## >AiNAC24

GATTTAGAATTTAGAATTTTTAAGTTTAGAATTTCGGATAAAAAAGTAATTTAAAAAAAAACTTGACTGATATTGTATTAATTAAAAGGAATGAAGTTGGTTTTCTTAGTTGAACTCATTTTTAAAAAAATAGGTTTAAAATAATATAAATATGAAGCAACAAAGTTACTGTTTGGCCATGATTCATGTTTGTCCTTTGGTGGACTTTGAAGTTAAAGGGACCATGGCTAGGAGGGAGATAAGGTTTTCAAAGGTGCCATAATGATAAAAAGCACACATAAGCTATAACACTTGTCCATGGATTAACTTGTGTTGCATAAAGGGGTGACCTACCAACAACTACATGAAGAAGAAGCACATACATAAAGCTCTTTTATCCCTTAGTGAAGCGTAATCATTGCTTATGAAACAGGTATACTAAGTCATGACATAAAGTCATGCCCACTATAATTGGTTAATTGTTTTACGTAATGCAAAGCCATATAGTCCTCGGATCCCACTTCGGATGCTATCTTGTGTTTTCTCTATTTGGCCGATTCGGACTAGGAATAACGTTAATTCCAAAATATCATCACTTCTATAAATAGACGAATTAAGATAATAGGCATGAAGTTGGTGGAAGAATAGGACTCATAATTTAATTGGTGTGCATAGCTAGCTGGCACATTCTTATATACCAGAAGTGCTTCTTTAATCTTCAAGTAATTATTAAGAAGCTGAAAATAAGAGGGGATATTATTATTATTATTACACCTTCTTAATTTAAAATATATTTAAGAGACAATACATATTAGTCTCAGATAATTCAATTTTTTATTTTTTAATTATTATAAATATTAAATTAAATAAGATAATTTTGTGTTGAGATTTCCTAACATTACTCCTTAATTTGAAGTGTGTTAGTTTTTCCAACCCTAGAAGAGAGATTCCACGTGAGCCCTGGCCTATTTGTTGATGTGAGAAAGCACATATATACATTTCTTTAATTTGATTTTGGAAATAATAAATAAATTTTAATGTTAATTTGTTTGGAAAAAAAAGTCAGGCTAATTTAGGGGCACTATCAACATTCAACACCCTCGTAGGTTGTACACCTTGCCGATAAGGTGCGGTTAAAATGATTAATGGATGGAAACATATGGCGAAGACAAACAGGAACACTTGTAAATCATTTTATTCCAAAAGGTGAAAGATCAAACCTTTTGTCATGCTTGAGGCCCATCATTAAGATTCATCCAGAAACTTGCACATTTTTTTAAAAGAATTTTTTTTTTGGGACCAACTCACAGCAGGCAGATTATAACAAATCAAACAATGGTCAATTCATGTTGGTGATACATCCAAATAGTAAAATGAACAATAAAAAGAATGCTCAAACAATAATTAAGATCTTGTATATACAGCAAGGAACAATATCCTCTTTTGCGGGGTGTAGATAATAATTTTGGAAGAATGTTATATGTCCGTATATTTTTTATTCTTAGTATTAAAAATAATTAATAAAAAGTAAGATAAAATTAAAAAAATTGTCTTTTATATATATATATATATATATCATAAAAAAATACACAAAATTATAAACAAAAAATAATATAAATATAATTTTCCATCGTGTTAACTGTTAAAATTTAAGAATTATGCTTTTCACTGCATGGCGTAATTGTTTTTTACCCCTAAATGTGATAACGAAATAGATATATTTCACATTACTATTAATGTTAAGTTCGGAATTCATACATATTGAATATATGTAAACAAAATGTGAAATTAAAGGTTGTGATAGAGTCATAGAGATCTCATTAATTCGTCATCAGGCCTTTAATTTATATATAATTAATTTCCATATTATAATAAGTATTATTTTCCTAAATTTAATTACTATGTTTCAAAAAGTTGTTTTATCAGTTTAATCTCTAACTTTTTTCATTATATTTTATTTTATAAAAAATATATTGGTGGAAATTATATAAGAAACAAACATAAACGACGTTTCCATGCAGATGAGATTATCAATTTGGATATGATGCATTAATTCATGCCAGGCAGAGTAAATAAGAATTAAGAACTTAAAAAGGTATGAAAGCAAGACATGAGTATATACATATATATAGTATAACCTTATCCTAATAAATTAAACGTTATCCAAGGCATGAAGGTAGTTATAGCCAGGTTACTTAAACAGTAGTGAAGCAACGCACAATAATTTGACCTTAGCTTAAATGATCAATTACATTACATATATAGCAGCTAACTTGTATTGTTAGCTTCAATGGCCGTCTATCTTTGTTGTTTGGCCCTACTTAGTTCCTTATGATGGAATTCCACGTACAGCCGTCTTTCTTCAAACAGCTCAACGCTTTTTTTGAATCCTAATCAATCCAACCATAAATCTCTCTCTCTCTGTATGTGTATATATATATATATATATATAGCATGACAAACTTGACCCAAATTAATAAAGTGCCAAAGTCCAAAAAGGAAGAAAGAAGAAATTAATACA

## >AiNAC25

TGTTAGAAACTTACCATTCGACCCCTTTAACATTCTCAATATAATGTCTGTAATGAACACAATTATATTTATACCGATTAAACAAGAAGCCTTGAAAATTAGTATTACATATCCAACATTACAGTCAACAATCCATGTAAAGATAAAAGAAAAAATTATTCTAAAGGACCGTTAGGAAGATTTAATTATTAAAAGAGATCTTTAATACCAAAATTATTAAAAAGGACCAAATTTTTTTATCTTCCTAAATTACTGATTATTTATTTACATTCATATATCCATACAACACATTAATTTTTACACCCTTGGATGAATGACCCATTTAGCTACAGCTTTGATAGGATTTTTGAAAGGAAAAAAGAATTTCAAATGAGTTATTTGCACTTTGAATGCAGTAAATGTATTTAGCATATTAGCATAATTGCTAATTCAATTAGCTTGCTATTGTGAACCGAAAGCTGGATGCAATTTTTCTAAATTTGACTGTCAAACTGAGTAACATAAAGAGTTTGTGTAAAATATGGATATTAAAAAGATTTAAAAAGATTTAATTATTAAAAAGGATCTTTAATACTAAAATTATTAAGAAGGACTAAATCTTTTTACATAATATTTAAGAAGAAGAACCAAATCTTTTTATAAGGAGATAAATATATCCTTAACTATTTTTCTTTAACCATATTTATTTCAGTAGTCTTGACTACAGAAACTGAAAATATGTTTACCATTTTAATACATTCTCCAGTTTCGGGAGTGTTTGTAAATGAATATCTTGCAACTTGATGTTATGTATAACATCTTGTTTTCTATTATCATCATCAGCTTCTTTAAATATAACTTTGATTGACTCACAATTCGTAACCCTCAAACAATTTAAATTTGACAATCTTTTAACCGTATTATGAGAAATTATATGGACCAATTCATCACATTTCTCAATGATCAGAGTGTGTAGTTTGCCAAAAGAATTTCCATGAGCTTCAGAATTTCATATTTTGCACAAATTCTTTATGTTACTCAGTTTGACAGTCAAATTTAAAAAAATTGCATCCTGCTTTTAGTTCACAATAGCAAGCTAATTGAATTAGCAATTATGCTAATATGCTAAATATATTTACTGCATTCAAAGTGCAAATAACTCATTTGAAATTCTTTTTTAATTTCCTTTCAAAAATCCTATCAAAACTGTAGCTAAATGGGTCATTCATCGAAGGGGGTAAAAATTAATGTGTTGTATGAATATATGAATATAAATAAATAATCAGTAATTTAGAAAGATAAAAAGATTTGGTCATTCTTTAATAATTTTGATATTAAAGGTCTTTTTTAATAATTAAATCTTTCTAACGGTCCTTTAAAATAATTTTCCCAAGATAAAAACATTCGAAACTCTTCAAAACAAGAATATCCGAATCTCAATTAAAAAATACATGCGAAATTCAAATTGAAAAAAGGTTAATTTTTAGTAGGTTTTATATTGAACTTGTTCGGCAAACTATATTATAGTATTGATTAAAATTGATTACCGTAATATTAATTCTTTAGCATTGTTTTTTATTAAATGCAATTTGAAAAATTTATAAAAGGATATGTTATACTAAGTAACAATCGTAGTTGTTGGCTTAATAATAATAATAATAATAATAACGACTTAATCCATTATTATTAGTAGAGACAAATTAGATTAAACCAATACTTTCATTACAAATCAAATATTTCCACTCAATATGAAAATATCTATTGAGTGGGAACGCTAGGGGTTTCTTGCTATGCATGAGAAGTCTACGTGAAGTAATAGAATTATGTTGGGCTATGAAAACAATTGTTCTCTTCAATTCAATTTTCCCCCTTATTAATAGAGTCTTCTTGGTATTTATATTTTTAACTTTATCAACACTCTAGGGTGACATTGGACCTAAATTCTTACTCTTTGAGCTTATACATTCGGTTGCCCCAATCCCACTCTAGCTCTAAATCCATATTTTAATAGTTTCTTTCTAGGTTTAACATCATTTCTTTATTTCATCAAATTCATCATCAGAGCAGAGCTATGTACCTTTAATCAGTGTAAAATGGACAAAATCACGTCCCAAAAATTAAAAAGAATAAAAACCAACATTTATTCATCTACAAGTTTTCATATACCAAGTAATATATACAAAGCATACCTAAACTTGAAATCAAAGCAATGTTTTTATTCCTTTTTAATTTTTTTTATTATTCTTATCTCTCTCATTGCAACCAATCCTATCTTCTATTCTTCTATCTCATTCTCTCTATTTAGTTTTGTTTTTGGGGGAAGGAAACTGCTTCTTGAAAACTGAAAAGAAAGCACAAAAAAGAGAAAGTGAGGGAGAATTTAATTCTTTAATTCCTTCCCACACCTCAACAAGTGCTAGCATGAGGGGTGGTGCAATCTTTAGAAGAGACAAGTTTATAAACTTTTTCTCCCTTTAATTTTATATTTAATTAATAATTATTACTACTTACTTTATTATTA

## >AiNAC26

TTTCATTACCTTTTTTAGTTTTTCTTTTTTACATATTCATGATATCTTGAGTTATAAATTAAATATACAGAGTCTCTAATATATGTATGACATGTATTGTGGCACTAATTAAATAGAGTATGCTTATTTATGAATTTTGTTAATAACCAATGTTCTTAAAGTAATTATTAACATTTTCAATGACAGAAAGTAGCATATCTTTTGATATTTTGATTCTTTATTTTATTTCAAATATTTAATTTTAGTAACCTTAGCTTCTTAATTAGTACTCTCTTAAAATACTGATTAATTAATAATATCTTAAAGTCATTATTATGTGCAGTTTGTTCTATAAGATGGAGGTATTGAAACTTTTCTGTAGATTAATTTGGAGATGTTAAAAACAAGCAATTACTTAAAAGTGATCAAAATTAATGAAAAAAACAAAAGGGCAGATTTGATTTCCATGTTCATATTTTGAATGGTTGATGATCTCGGATGTTGTGTCTTATTTGGCTAAACATACTGTGACACATAATAAGAAGGTTCATGAACTTTATTCTTTAGAAGCATGGGTCACATAAATCAGTGGGAATCACAATGAGTGACACGCACTATATTGTATGTATATATATGTTGATCTAGTAACATACTTAGTTAGTATTGTGTGTGTGCATGTCCATTATCACATGTGTGTTGACTATGTGAACATTTTCACACGCATAATCATATCATCATGGTCTTGCTTTTTTCTCCATCTACACTTTATAACGACAAACCATATTGGATATCAATTCTTGTTCTTCTTTTACAACATTTTTCACTTCCATGGTTCTTATATGTTATAGGCCACANNNNNNNNNNNNNNNNNNNNNNNNNNNNNNNNNNNNNNNNNNNNNNNNNNNNNNNNNNNNNNNNNNNNNNNNNNNNNNNNNNNNNNNNNNNNNNNNNNNNNNNNNNNNNNNNNNNNNNNNNNNNNNNNNNNNNNNNNNNNNNNNNNNNNNNNNNNNNNNNNNNNNNNNNNNNNNNNNNNNNNNNNNNNNNNNNNNNNNNNNNNNNNNNNNNNNNNNNNNNNNNNNNNNNNNNNNNNNNNNNNNNNNNNNNNNNNNNNNNNNNNNNNNNNNNNNNNNNNNNNNNNNNNNNNNNNNNNNNNNNNNNNNNNNNNNNNNNNNNNNNNNNNNNNNNNNNNNNNNNNNNNNNNNNNNNNNNNNNNNNNNNNNNNNNNNNNNNNNNNNNNNNNNNNNNNNNNNNNNNNNNNNNNNNNNNNNNNNNNNNNNNNNNNNNNNNNNNNNNNNNNNNNNNNNNNNNNNNNNNNNNNNNNNNNNNNNNNNNNNNNNNNNNNNNNNNNNNNNNNNNNNNNNNNNNNNNNNNNNNNNNNNNNNNNNNNNNNNNNNNNNNNNNNNNNNNNNNNNNNNNNNNNNNNNNNNNNNNNNNNNNNNNNNNNNNNNNNNNNNNNNNNNNNNNNNNNNNNNNNNNNNNNNNNNNNNNNNNNNNNNNNNNNNNNNNNNNNNNNNNNNNNNNNNNNNNNNNNNNNNNNNNNNNNNNNNNNNNNNNNNNNNNNNNNNNNNNNNNNNNNNNNNNNNNNNNNNNNNNNNNNNNNNNNNNNNNNNNNNNNNNNNNNNNNNNNNNNNNNNNNNNNNNNNNNNNNNNNNNNNNNNNNNNNNNNNNNGAGATATACAATATAATTTGTTATATTTCCGTCTTCAAATATCACAAATTAAATCCCTATGCTTATAATTGGATTAATTATGTCGTCATTATACTACTACGTACATACTGTAGGAGGTGTAATTAATTTGGTATTGTTTTTCATTATTGTTGGCACTTGTTGATATAATCAAAAGTAAATATTAGATGGTGTAAGTTTATAGCTTAAAAGACGCTACTCTGTAAATATTTGGAAAATGTCCGAATTGGCCTTATTATGAAATAGTTAGGGATTGAAAGAGCAAGAGGGATAAAAGTAACTTGTAGAGTAATAAATTATTATAAAGGTGTTCTTGCATTGTCACTAAGGCTGCGTTTTGTTTTTTCTCAGAATAGTACAGAACAAGACACTGAAAACATGATAGGATAAGACACTGATGAATAGAGACATAAAATTTTATGTTTTTGTATTTTATTTGGTGATAAACTAGAACAAATTATAAAAATCTAATTTATTCTCAATTTTTTTCATTCAAAAAATTTGAGATGAAAAATATAATAATAAAAAAATATAATTATAAAAAATGAATAAAAATAATGAAAAAAAAGAATAAAAAATAAGTTGTGTCCTTTGTTAGTGTTCCCGTGTCCTTTCTGTCAGGATAGACATAAAATACACTAATTTAGTGTCTCTAGACACACTGTTTCTGTTCATATCTCCTCAATCAAACATAATTTTGTGTCTTAATGTCCATGTCTCAGTGTCTTATTTCTGTAAATAAACGGAGCGTAATAAATTGGGCAAGTGAAGGCACAATGCATTAAGGGGATGGAAGTATCTTACTATATATTTTTTGCAGGAGTGAAAAATTGG

## >AiNAC27

GTTTGATAAAATTCTTGGTTGGATTTTAGAGTAGAATTTTACGCTCTTAGCTTGGGAAGGTAACTTAGGAACTCTTGAGTTACTAATGTCCAAGTGATTGACGATTGGGAGCTATTAACTCTAGATCTCACTAATTGATTTGGCGGAGAACTAGGACTTATGGACTTGGATTGATATAGCTCACTTGACTTTCCTCTACTATTAGTTAGGAGTTGACTTAATGGGATTGATCCTTGCCAATTCTCATGTTGTGGTTAGTGATAGGGATAGAGATCCTTGACCACCAAACCTTGCCAAGACCTTTGTAGTTGTTAGTTTATTTTTATTACCATTTACAATTCATGTCTCTTATCCCAAAAATCCCAAAACATACCTCATAACCAATAACAAGACACTTTGTTGTAATTCTTAGGGAGAACGATCCGAGGTTCAATACTTCGGTTTATAAATTTAGGGGTTTGTTACTTGTGACAAACAATCTTTTGTATGAAAGGGTTATTAATTGGTTTAGAAACTATACTTGCCACGAGAATTCATTTGTGAATTCTAAACCATCAAGAGTCCATTCATCACCCCCAAAACAGGCCAACCTTGGGGGCTATGATCCGTTAGGTCGGAACCGAGTCATAACGGGAATCTCGGAACCAGGATCAACACACAACTCCTCCGCAGATCTCTTTCCAGAGCTACCTAAGTTCCGAAATATCAGAACACAACCAAACCAGTAACAAACAATGTGGATAACGGTTCTCCAATTACTCCATTATATACGAGTGATTTACTCAGTCACAGGTACGCTATTCACTCCAATCTTATTCTGAACTCACTCCAATTACTCTAATCTATTTACTCATACTTATTTGAGCGTTGGAGTCCCTTTTTAGGTACCCAACGCCGCCTCCTCAGGAGAAGACGATGTTCCACTCCCTCCACTCTAAGAAAAGCAAGTTCAAGCGCTAGTAGAATAAGCTATACCTCAGAGCTCACTTTCACACAAAAACAGTATCCATTGGATAGAACTTCTATCCTCCATAACTTTTACCAAATATATATAGAAAAGAATAAAGTCGAACATTTATATTCTATATCTATATCCTAACTAAATTCTAAGCCACAAAGAAAATTTTTTTTTTATTGACATGTCATTTAATTATTCTAATATCTGATTATTTTAATTTATTTGAAAAACATGTAAAAAATATACAAAAATATGTAACGATGAATTAGTGATTGATTTTTAGATGATGTTCAAAATTATTGTTAATTAATTTGTATTTTCTCTAAAAAATTTATATTTGAATAAAATAAAATCGCATTTCATGTTAGGATAATGAGGAGAAAAATCGTAAGCAAATATAATGAATTATTTCCGAAATTAAATTAAACAATCCCATGGCAGCATAAACTGAGAAAGGAGAAAAGCACCAAAAGATGGATTGGTGAAAGGGAAAGGGAAAGCATGAGATGACATAAGACTAAGTTATACTAATATACTACCTATTTAGTTAGGGAACATTTAATTTGCATCCATCTATCAGCTATATGGGAAAAGCATCAGATCACTCACAAGTCATCCTGCCATGCCACGTTGGTTCTTTATTCATTTCACGTGGCAAAATCTGGCCCCACATGGGTCCCACTTCCAAATTACCTTGGCCGATCCAACTATAATATATAAGAAAAGTATATACAACAACAAGTGATTACTCAAAAAATAATAATTTAATTAATTTATAATTATATTTAATTAATTTTATTTATTTTTAATTTATAATATTTTTAGTATATAAAAAAATTTATAAAATATATAAAAAAATATCCATTTAGTATAAAAAAGAAACATTATAATACTTAGTAAAAAAATATCTTAATGCCTAACATAAATATCATTCACATACAAATTTTGAATTCACCAAAGATAATTTGCTGATTTTTAGTTGGTATCCTCTTGTTCCTTACCATTGTTGATATATATAATCATTTTCTTTGCATGCACAAAATTTAAGTAATCAAAGTTCTTATTTAATTCATAATTTATATGGACTAAGATATAACTTTGAAAACAAAAAAATGGAGGAGCATACAAGTTGAATAAGTGGATAAGGAATAGTAGAGAAAAGGGGTGGATGATGATGAAACTCCATGCGAGTAGTAGTCAAGTGTGAACGGTATGTAGTTTTCTTGGGTTATGAGGAAGAGCCGCCAAAGTTGGTTTTCCTACTGTTGGTCACGTTAATGCAGATTCTTGGTCTGATGAAAAAGGTTTTGAAATGCATCCAATTAATATATTCCATGCATTGTCATGTGGAGCATACCAGAATCCAAGAAAGAACTACCAGGAAGCATAGGATCATTACCACACAAGAACACAAGCTTGAATACAAAGATAAAAAGAATCTTCCATTCCATTTGATCGAAAAAGAGATAGCTAGTGGGACAACCCCAATTTGCTTCTTCATTATATTCTCTTTTTGTCCTCTTTGTTAAAAGCCCAAGCAAC

## >AiNAC28

NNNNNNNNNNNNNNNNNNNNNNNNNNNNNNNNNNNNNNNNNNNNNNNNNNNNNNNNNNNNNNNNNNNNNNNNNNNNNNNNNNNNNNNNNNNNNNNNNNNNNNNNNNNNNNNNNNNNNNNNNNNNNNNNNNNNNNNNNNNNNNNNNNNNNNNNNNNNNNNNNNNNNNNNNNNNNNNNNNNNNNNNNNNNNNNNNNNNNNNNNNNNNNNNNNNNNNNNNNNNNNNNNNNNNNNNNNNNNNNNNNNNNNNNNNNNNNNNNNNNNNNNNNNNNNNNNNNNNNNNNNNNNNNNNNNNNNNNNNNNNNNNNNNNNNNNNNNNNNNNNNNNNNNNNNNNNNNNNNNNNNNNNNNNNNNNNNNNNNNNNNNNNNNNNNNNNNNNNNNNNNNNNNNNNNNNNNNNNNNNNNNNNNNNNNNNNNNNNNNNNNNNNNNNNNNNNNNNNNNNNNNNNNNNNNNNNNNNNNNNNNNNNNNNNNNNNNNNNNNNNNNNNNNNNNNNNNNNNNNNNNNNNNNNNNNNNNNNNNNNNNNNNNNNNNNNNNNNNNNNNNNNNNNNNNNNNNNNNNNNNNNNNNNNNNNNNNNNNNNNNNNNNNNNNNNNNNNNNNNNNNNAACGTATTGTTGCTACTTTCATACTTGTATAATTAGTGAATATTAGATAAATAGAAAAATGAAACCACTCACACTATTGTTAAAAATTTAAAAACATATACATGGTTGGATATTGATTTTATAGATTAGTCGACTTATTGTTACTGTGAGCGCAAAAAATGTCATTATACGATCGCATCATTTTTTATCTTCATACTGTCGTATTGTATCGTGTTGTTAAGTTTTACGATTACTGGTTTAAGTTTGATGAAGATCTTGTTAGTGTATTTATAGAGCGATGGCGTCTAAAGACCCATACCTTCTATATACCTTTCGTAGAGTGCACAGTTACATTGCAGGATGTTGACTACCAATTTAGCCGTTTAGACTTTCAATAAATGGTGAAACTGTGAGTAGTTGTCTGTCAGAATTTGAATTATACATTTTTAAAGGAAAACCAGCATAAATATGATTTCAAGAGTTATTTAATGAATTTCCTCCTGATCAATATATTCACAAGCACACTGTCTATTAGATTTGGTTCTAGCAGAGATTTATAATTTTTTTACCGGATGCCAATGAAAATACTGTTAGAATTTATGTTTGAGCGTATATTACGGTGTTCTTATCTATTCAGTTATTTGAAGATAAGTTCGGTGCTTGAGTACACATTCGTTGGTTACCCTATGTTGTAAAGCTGGATAAATTAGAAAAATATAACTGAAATTCTACTATGTTGACATGATTGTACTACTGCATGTGTCGTATTACTAACCAGAATGTGATGTAGTAGCTAGTCTGTTAATCTTTCTGTAGCCCTAGTTGTAATTATCAGGGTTGATTTTATATATTTTTTTAAAATCAGTAGTAGGTCCATGATTNNNNNNNNNNNNNNNNNNNNNNNNNNNNNNNNNNNNNNNNNNNNNNNNNNNNNNNNNNNNNNNNNNNNNNNNNNNNNNNNNNNNNNNNNNNNNNNNNNNNNNNNNNNNNNNNNNNNNNNNNNNNNNNNNNNNNNNNNNNNNNNNNNNNNNNNNNNNNNNNNNNNNNNNNNNNNNNNNNNNNNNNNNNNNNNNNNNNNNNNNNNNNNNNNNNNNNNNNNNNNNTGATTAAAAGTATTTTTTATATAAAAATATTTTTTAAATTAGATAATTATTCTTCGTGATTTAATTTTATTATAGAGAAGGTAATGGTGTAGGACAGCGATATGAAGAAATAGCGTGTTTTACAGGCGTGTGGTGTCAAGGAGCTACAAATCGGATGGTACGAGTGCTTCTAAAAAAATCAGACGGTCCAATTTTTGTTTTATTAAAAAAAAATCTTTTTTTATAACACGAACAGTCCGATTTACTTCTTCTAAAATTTAAAATTTCTCTTTTTGCAAATCAAACCATCCGATTTGATATGATATTTTCTTTTCAAAACAATTCGTATGACCCGATTTATACCGTTCCACATCTTGGTTTAACATCACTATACTCTCATAATGCAGCACATCACATACATGCCTCCAACAATAAAAAAATGAGCCATTATTCTTTTGATTTTTTATTATATTTTTTAGTTTTATATGTATAATACAGATTTTTTATATTTACTTAAGCAAACGAATAATCTGATGGATCAATTTGACTAGACAATTTCTAACCGTGTTTTGTTTCTCATCTTACTCCACGAAATTACAATATACCAAAAAAAAGAAGAGAAAAGAAAAAAAAAGTGAAACTTCTCCCTTACGCCAAATTTCAAAGTCAAGTCAATGACTAACTTCGTTGCATTACAAATTCGACACAAACCAATCACTCATAACCAACACTCTCTGCTTCAGTGCTGCTTCTTCTCTTTACTCTGTTTCTTCATCTTCCTCTTCTTCATCAATCATCATCAGCTTCATCACCTTCTTTTTCTTTTTCTTCTTCTT

## >AiNAC29

TATTCTATATTATCTCTGACATGTGCCTCATCCTTAACCAGCAAAACATTAAAAGATTTCAAGAAGAAATATATCCCTGTTTTGAGTGTATTTCTTAAATCCTTTGGGTGTATTTCTGTAATCCTTTCTGTAACCGTTTGTGTGTATTTCTATAACCGTTTGCGTGTATTTCTGTAATCGTTTGGGTGTATTTATGTAATCGTTTGGGTGTATTTATGAAGTTCCATTATCTTCAAAACGATTTCAAAGCTTGATTTCAGATACAATGAAAATCGAGAAAAACGAAGCAAAGAGAGAACGTACGAAGGAAATCCAACAAATTTGGCAAGAAACTCGAAAAAAGAAACGAAATCTTTTGAAAAATGGAAGTTATATATTTGCGCGTTAATTGATTTGAATTGATTTAAAATTCTGTTAAAAATGCACGTAACATAAACAACGCGTTTCATGGGTGATTTCGTTCAGACTTGTAAAGTTTGTAAATACAAAACACTTGTATGTAGAGATTAATTCTTGGAAATTTTACTAACATTCTGAATATTTCGATCCAACCTTATTCGATTAACAAAAAAAATATTTTCACTCAAAAATACTATTTATGTATGATTAGTAAATTGAATTAAGTATATATGCAAAAGGAAACAAATTTAAAGTTCAAATTTTTACTAACTCTATATATGTATACATTACTTCATTAACATTTATATCATATATTAAAGATGTGGTTCGACATTAGAATTGGTTGATATCTGAATCTAGCTCTACCTATTGCGAGTCAGGTTGTCAATCTACTTCGACCGAGTTAAAACTGGTTGGACCGATACCTACAGATACTACTATACTGTTAGTTTTATTAGTGAGATACAAAATACTAGCTATCAGAATTTTAAAAATAAAATAAAATTACCAAACTTTGATAACTTACGAGTTTTTCTTTCGGATGCTTAGATTATAGTATGTTTTGTGAATGTTACCAGATTATATAATATGTTTTCTGTAGAATCTGTCATATTTATTATTTTCACTTGGTGAATTTTTTAATCAAATCAGAACTTGATGGATTTAAACTCATTATTTGATATTTTTTCTTATAAAGAAAATCAATAATCCTCTTAATTTGTTAGAAAGAGAACAAAAACACATAGTAGATAGAAATGTTTAACTTTAAACCTCAGCAATTAAAATAGAGAAATGTTAAGGGGTCAGTAACTTTTGTGATTTGTAGTCATCAAATAGCCATCAATGATGATTTTAATGGTGTAAGATTGGTGTGAAATTTCATCTAATGGCTCACTTTTCTTTACTGGTTACATGCTGGCTAGAATTTAACAAAGTTGCTGCCCCCTAGACTTTTTCATTAAAAAAAAAGACAATTTATTTACTCTCATAAAATCCTTCATATATCCCTTTCTGAGTAAATAAAAGAGCAAATTCTTGGTGCATTGCCCATTAACGGTCTTTTTTTTTTATGTTGAGCATTTGAAAATACAAAGGAAAAAAAAGGACAAAAAACAATTGAAGAAGATAGATTTTTCAGTTGTATTTTATTTTGCATTAATCCATTTAACAAAGATGCAATAGGAGAATTCAATAACATCAACATTTACATGCAAGCTAACTTGGACACTAGTTAGATCTTATCAACATATATCATGTACTATATACAAAATCATAATGATTGTAACCAAATTTAAAATGATAACGATTTACATAACTGAAAAAAAAAAGGTCAGTTTAAACTTTAAAGTGAAATGAATTCCCACCAAGCATTCTACCCTTAACTTTGTTCCAGATACAATGTTTTTTCTCCCCTCTTATATCAACCTAAGATAGAATGTAACTTTTGGTATAATTTTCAAGAATTTAATTTTAATATATCTTCGACGTAAAATAAATTATTTAAACATTCAATTATGTATTATCACATAAAAAATAATTATTTGACACATTTATCATTTGAATAGTCATATAAAAAAAAGGTAAATAGATTTTTTGTCCTTAAAATTTGTTATAAGTTTTAAAAATATTCTTAATTTTTATTTTGTTTCAAAAGTTTTCGAATTGCATTAAATATACCCATGACGGCTAATATTTAAAAAAAAATTTAAAATCAAGTCAGCAACAGTTTCACAAGAACAATCTTTAACACAAGCAAAACCAAACATAGTGATCATGCATTATTACTGGATTAGTCCTAAACTTTCTAAAAATTTAACTGTCAGTAATATATTTGATGCAAATAAAAAACTTTAAAAATAAAATAAAATTTAGGAATATTTTTAAAATATTTGATAAACTTCAAGGATAAAAAATATATTTTACCCTATAAAAAAATATATGTAACATTTTTTTGGTAAAAAAATTTACCTTGTAAATATCTCAATTAAAATCTAATTTTCACTTTCCAATTTTGGACATACTCAATCATGCACATACACCATAGAAAAACACACGGTTTAAAGCATGAAAACGCACCCTACCCAAGTTGCCTTATGAGTCA

## >AiNAC30

NNNNNNNNNNNNNNNNNNNNNNNNNNNNNNNNNNNNNNNNNNNNNNNNNNNNNNNNNNNNNNNNNNNNNNNNNNNNNNNNNNNNNNNNNNNNNNNNNNNNNNNNNNNNNNNNNNNNNNNNNNNNNNNNNNNNNNNNNNNNNNNTTTTATGTTTGAGTGTACATATAACAATTATTTTATTTTATCAAATAGTTAAAAACAATTTTGTCAGTAAAGGATAAAATTATTATAAATAGAATAATTCTTCATAAGATAAGGGCCAGAAATATCATTGAAGTTAATGGGTTAATACTCAAATTCGTTCCCGAAAGATTATGTTATCTTCATTTTCGTTTTCGAATAATTTTTTTAATAAAATTAGTCCGTGAAAGATAAAAAAATAAGTTAAATTAGTCATTCTGTCAGTTGAACGATGACGTGGCACGTTAAGTGTCACGTGGCATGATGACGTGGCACGCCACGTGGCAGATCAACGCCACAGTCAACGCCATGTGGCAGGTCATCGACACGTGGCACGCCACATGGCAGGTCAGTGACACGTGGCATACCCAGGTCAACGTCACGTATCACTTGACATGTAAAAAAGATTTTTATAGTCAAAATAGTCCTTGAAAGTCCAGACATAAGTCATTTTCATCCCTCAAATTTTAAAAATTAGTCAAACTAGTTCTTATATAATTTTTTTATTTTTCTTCATAATATTAAATTTAAAATATTTTTTGATACTACTAATTTTAATAGAAATGTAATTGACAAACAAAACATTAGTAATTGTATCTTTTCTTCTTAAAATTTTTTTTCAATAAAATTATCTCTCTCCTTTAATTCTTCTCAAAATCTCTCTTATTCTTTTCTATTCTAAAACATTTTTCTTACATACATTTTGCTGGAATATATATATATATACTCAAAATTGAAATGTATGTATTTATTAACCTAAACAAAGTTATATGCTCGGAATCAAAATTTATGTATATTAAGCTAAACAAAGTTATACCACTATTTTTTTTCTTACATACATCTTTTTTTTACTGGAATATATATATATTCCAGTAAAATGTATGTAAGAAAAATGTTTTAGAATAGAAAAGAATAAGAGAGATTTTGAGAAGAATTAAAGGAGAGAGATAGTTTTATTGAAAAAAATTTTTTAAGAAGAAAAGATACAATTACTAATGTTTTGTTTGTCAATTACATTTCTATTAAGATTAGTAGTATCAAAAAATATTTCAAATTTAATATTATGAAGAAAAAATAAAAAAAATTATATAAGGACTAGTTTGACTAATTTTTAAAATTTGAGGGATAAAAATGACTTATGTTTGGACCTTTAAGGACTATTTTGACTATAAAAATCTTTTTTACATGTCAAGTGACACGTGGCGTGCCACGTCATCATGCCACGTGGCACTTAACGTGCCACGTTATCGTCCAACTGACAGAAGGACTAATTTGACTTATTTTTTATTTTTCAGGGACTAATTTGATTAAAAAAATCATTTAAGGACGAAAATAAAGATCGCGTAATCTTTCATGGACGAATTTGAGTATTAACACTGAAGTTAATATGCCTTTCTTTTCTTCCACTTTTCCATTCAAACAAACGAAATAAATAAATCATTTTTTTCCCATCTAAATATACTTGTGGAAATTAAAAATTCTATTCAACTTCTTCCTTTTCTTTCCTTTATGGTTAGTTCTCTCTTCCCCACTTCCTTCCAAGTTCCAACCAAACAAAGCTTTAATTAATTATAGGTTGATTTGTTCCTCCTTGACCAATTCCCTAAAATGAAGAGTTGGATGCATTAAAGGTCTAAAAATCTCGGTATTCAATCAACGACTTTCTACTCTTGGTTCCAAATTTTATTTGCAAGAAGAGGAAATGATAGTACTAATAAGCTCACAAGCTCAAAGAGTATAGGCTGATATAGTAGTAGTAAGTAGTAGTAGGCATTTGCTCTCAGACTCTGAATTAAATTCTGCAACCAACCACGCATTTAATGCAACCCTTCTTGGCTTTCTCCTCAAATTACCTTCGAAATTATCCGTTCGTTTTTTTTTTTCAGGAATTTATTTAATGAATTTCCTTAATTCTTGATTTAAAGTGTAAAAACAAACATTTCACTCTTAATAATGTCTGTTTGAAATGATTATCTTAGAACATTTTTTTTTGACAGGGATTTTCTTAGAACTTCGCTCTGTAGAATATATTCTAAAATTAAAGTTATAATGTCAAAGGTACGTATAATATTTGTTTTTTAATTAATGTATAAATAAGTAAGTTTGGTAAACCAAAATGTGTGCAAAACAGCATTATTGATTTATTGTATGTGTTTTGAAGTAATGATAGTATACGACAAAATAACTAAATAATTATATAAATAATGGATACATGTTCCTCCTCCTTCAGTTCCCTCGTTTTCAGAAGAGGCAGGTACCTCATTATNNNNNNNNNNNNNNNNNTGTGTGATACATAGATGATGATGATGATGATGCTATCA

## >AiNAC31

CAATGTTTTTGCCCAAGCAACTTGCTCCAACCTTGTTACCATCCATCACATTTTTCTTGTTTTTCATCTAATGGGTCCCACTAACTGATTCTTTCTTTCTCTTTTTAAATTTTAGATTTTTATTTTCTTCCTTCTTGTTGCGGTGGTTGCCCAAGCCAAACATGAAAAAGAAAGGGATAAAGGGAAGATAAAGAAAGTGGTAGCATTTCTATGCCCTCCCCTCTCTATATAGTAGAATGAAAATAGCCAATAAGAATCGCCACAAATTCTGGAGACCCCATATTTATTTCCTAAAAGACATAAATTTAAATTTTTCTATCATTATGAAGATCTTTTTATTAAAAAAAAAAAACTAAATAATAGATATATTATATATAGTTATTCATATATCTTAATAAAACTAATTTTCAAATGTTTAAGTCGATGGCTAAAGATTCTCTGAAGTTAAAAAATTAGTAAAATATTAAAGTGAAGAGTTAATCATTCATAAATTAAGATAATGAAATATAGATTTTATAGAAGTTATAAACTTAATAATAATTAATTTCTCACATTATCATTTTTTTCCTTTTTTTTAATTTTAGAAAATCTAAATTTTAAGTCGATAAAAAAGAGACATATTTTTAATACGTTCTTTTATGCAAGAACATTTTTAGAAAAATTTGCATAAATTTTTTTATATGTGATGCTAAAATTTTTATTTAGAGATCAATAAGAATCAAACTTAGATAATTTGGTTATAAAAATTATGAATGATACCTGTTATGAAATCGCTCATTCTAAAAGTTTAAACTGATAAGAGAAGACAATACTTTCTTTTAAGTAAGAATTTCTCTTAAGTTTGTTTGATTTTTTGTATATATTTAACTCTTGACTTTTCGAACATAGGGTTATGATACTATGTTATGAAATCACTCATCTCAAAAATTTAAACTGATAGAAGAATGCAACATTAATAATCATATTTCTAATTATATCATTTGTTATAAAATTATTAATTTTTTTAAAAATATAAATTAGTAAAAAAAGATACATAAATAATAATATATCTAACATAGTATATCTTCTTGTATCTCTTTAAATTTTTAGAACAAATAATTTTATAATTTAATATCAAATCTTCTATTAATAAAAAAAAAAAACAAAATTTACACCAAAACTTGATGTGTGAAGGAAAATTAAAGCAAGAATGAAAAGAATAGAAAGATAGAGAGATAGAGAGGTTAATTTTGGACATGGTCCATGGTTGTTTGAGGGGTGATTTTCCATGGAGATTTTAAGGTCAGAGGTCGTAGTTGTGGATGTGGGCGTGCGCCCTTACTCTTCACTGAGTTCCCTATAACGTCCATTCCTTGTGGGACCCTTAAGTGATACCCACATACACATGGGTCCCACTTTCAAAAAAATTTTGCTCCCTTTTTGAAAATTTTTTTTCTCAAACACATACAGTAAACTCTCTTTCTCCATCTTTCTTGGTAGGTGGCACTTCTTCCAGCATAGCCATATAGCAATTAGCAAAACTAACCCCACATCATATTCATTTTTCTATTTCAATTTCTTTGTATTATTATTTTTTATTATTATTATAAGCCCACATATACCCAATGTCCCAATTGGTTATTGGTGTCTGGTGTCCCCCAGCTTTAGCTATGCGACAAATATTATGGAGTGCTATTGTTATTTGTTATGTCAATATATTCTCATCATTATATTTTAGATATTAACTAAATTAAATAGTAATAGTATGGATATATAGTCATAGACATATACAATATCTTACAATTTTAATTCTCATATTATTTTTAAAATTATTAATTAAATGAGAGAGAGAGAGAGAGAGAGAGAGAGAGAGAGTTATTATTGTTATCATAATTATCATAATATCTTGCATTTATATTGACACATTACAAATGTTGAGAGTAAGTGAGAGCATTTGATAAAGCCAATAATATATACCAAGATAGAAAAACCAAAGGAAACTGAGAAGGGTTTAGACTTTTCCATAAAACAAATTATTATGATTTACTAATCTGATCATATCTTTTAAACAAAAATGTATCTATTAAAATATAAAATTAAAAAAATAAAAAATATATATACCAAGACACAAATAGTAGTCATCATTCTAGCACCATACAAGAGTTTCTTAACAAAGACATTTCTTTTAATTTAAATTATTCATACTTTATTTTATAATATATATATAAAATACATTTTCTCTAATGTATCTTTTTGGTCCCTTGTGTCCCACAGCACATATATATAGTGTTCCTTTACATACACATTGCAGTACACACAACACACCACCCTCTCATTCATTACATCTTTTTCTTTTTTTCTTCCTTATCCCTTTGAAAGTGTACCTATTTGAATGTCTTCTCTCTTCTTCTTCTTCTTCTCTTTCTCTTGGGACCCTTTGGTGAATTGAACAAGAATCAAGAACAACCAATTTTAGACCTTTATATATTACATACATATCTTTGATTTGTTGTTGACCTGA

## >AiNAC32

TGATGTAACATACATAAAGAATTGATTTGATTAAAAATATATAATAATCTAATTTTATATTTTTTTGTATTTACTATACAAGTTTTTATTATTACGAATAACCATAATCAGCCGATGGAGTTGGCTATGTCGGCTCGGTCTTTTTAGTTAGGTAGTCAGTCGAACGCGATTGTTGCAAAAGATTTAGTTTTCGATCAATCACCTCGGATAATAGGTGTGATTTGTTTTGGTAGATTTGGATGGTCCGACTACTTCTGAACGGTTCAAGTTATATTAATACTTGATAAAAATAATTTTTTATATTTTAAGAGATCAAATCAGTTTAATTTTAATCAATTTTTTTTTTAAAATAGAAGAATTTGAATTTAGTTATATTATAAATATGTGAGATTTATAAACTAATTCACCGTTTTTTATTATCTATTAATTTTAATTCCATGAAATTCCAAACACGTAAACCTTTTGATTATAAAAACTCTAATATCATATTATAAAATTATTTATCCTAAAAATTTAAATTAATAAAAAAAAGTTATGTGAATGGTTATATTATTATATATCTATTTCATCAAACAAAATTAATTAAGACTAACATTTCAATTGACAATTTTATAGAGTGGTCATGCATTTATTTGAAATTAATAAGTGGCTAAAAATAATATTCAATTTATACATTAATTTAGTCAAACTTTTTGAATAAATATAAAATATAAAGACATTGACGTTTTAAATATAATAAATTGGAAGATGATAAACATACACCAGGTTAAGTTAGTTAATTAATAGATTCCTTACACATAAAGATCCTTTATTTTATTGAAGAAATTCTTGTACAAAATTAGCCGTTTTNNNNNNNNNNNNNNNNNNNNNNNNNNNNNNNNNNNNNNNNNNNNNNNNNNNNNNNNNNNNNNNNNNNNTATAATTACATATTTATTATATATAATATTATCTAATTATTCTAACAATAATTAAAAAATATAAAATAAATTTATTTTAAATTGTTTTTTACTATTTTTTTAGTGTCTTGTAAATATTTTTGAAAATTATATTTGATAACCCTATACTAATCACGAAATCTCACTTCATCTTTTTGAAATTTTAAATGGAAAGAAAATATCTTCAATTCAACCTCTTTGTTGAGTGAATAGTTCAGCAAATTGAAGCTCTTGTTTAATTTAGGTAAAAAGAATTAACCACGAGGATTGACTTCTCTCTCATCATTGCCAAGAGTTGCTCAAACTCTAGGTTTCAGTTTCACAATTAAGGGGTATTATATAATTCATTAACTAATATTCACTAACCTATATGCTTAAATAGAAGTTTCGGAGGAGCAACCTTTTTTATTTAACTGTTGGATTAATTTATACCGTGACTTCAATTACTCAAGCTAAACCATGACTAAGTGTTTCTCTATATGTCTTTGCAAATTTTTTTTCTTCTGATCTCTTATGGCATCTTTTAGTTTGACAAGATAAAAATTAATTTATTGTGGATCAAAATTTTATTTAAAAATTTGTTGTTATTCAATAAATTGTTACTACATGCATAAAATGATATTCAAATTTTTAACATTTATTTCAGCAGATGAGTAAGTTAATCTTTTGACCAATATCCAACAATTAGTTTTTACAATTTGTTTATATATAATATATTATTTTGACGAATTGAAATTGATCACCTAACTCCCATGTCCTCCTATCAGATTTCAGCACAAAATACTCTTTAGCCAATATATCCGCTTCCTTAATTTGTCTCCATGCCATTGGGTATAAAAATTATTCACCATATGACCTACGAGCATAACATTGATAAATAACGACTTACATATTATTGTTTTAAATAAAAAGGCCATTCAAGAAAATTCCTGTGGATGCCAGCTTATCAACAACCAATTTGATGAACACAACTAATTAATTTAAAAAATAATAATAATTCTTCATATGGAGATTATTTATCACAATTTTCAGACCAGGCAGATGCACAAAAACCCATCAATTTATGACGAAGATAAATCATGAATCAGAAATAATAAATTAATAGGGAAGATAATAGATATGGGTATATAAAGCAGAAATGGCCAAAATTCAAACGAATGAGACTATGTCATCATCACTAGGGCTTTGTGTGCCAACTCTCTAATTATTATTATCATATAATTTGCTTAATTTTATATTTGGAAATAATAATGATAAAATGTCTTATATTATTGTTATTTTGGAAATTATTTACTTAGCATTATCTCCAGACTAAATTAATGCAATGCACAAAAATTCTTTAAAAAGATGGAAAGAAAAGAAGCAAATGATCCCTAGCTAGAATTATATAGATACATGTCAGCAAGTTGGCAGGAATTGGTTTGTCCACGTCATCATGCCCATTCTCTCCCTATAGAATTACAATATTACCCTCCATTGTTGCATTTAATAACAAGTTGACTTAGCCACTTGTGAGTTCCTTCTCCCATTTATATATACACCTTAGCTCCTCTTCCTTTTCTAAT

## >AiNAC33

AAACAAAACAATCACACATCAACATACATCATTACATATAAACACGCATTAAGATGAAAACCAAATGGGTTCAGAAGATTTGATAATGGAATACCTAACACGATCTTCTTCCGGAAAGCAAGGGTTTCAAACTCACTGCTGCTGTGGACCTTTAACGTTACTGAACGACAATGTCACTTCTCTGGCTGCGAATCGCAAGCTTTCAGTGACTAGCGGTAGGGTTTCTGGCATTTCCTCTGCGTTTCGTGCTTCACAAGAAGAAGAAGAAGAAGAAGAAGAAGAAGAGTCAGGGTAAGCAGCATGCGTTATGTCCAAAGGCGGGAAGCCTTACACGTTTACTAACCACTATCACCTCTTCCTTGCCACGTCGGACACGGCGTCAATAATTTGACACCCACTTAACGGAAGGACTTGATTGATGCAAATTAAATCTTTTTAGGATTTAAATAGAATAGTTTAAACGTTGGGGACTAAAATAGAATTCACCCCAAATGTAGGGGACCAAAATAATAGTTTACCCTTTTAAAAATATTGTGACAATAAATTTTTTTGATCACTCCAAAAAACTGTGACTATAATTCTTTTTAGGTCATCTTCTAAAACTGTAATCATAGACCTTTTTAGATCATTATTTTTTTAATAACGGCGATCATAAGTACTTTATAGTCACCCTTTTTTTAAAAATTGTAATTATAATTTTTCTTTTTATCACAATAAAAAATCTTAACTACAAACCCAAAATTTTATAGCATGAATAAGCTGATGCATATACTCAATGCCTATAAACAGGACACAACAGAATGGCAAAACAAAATAGATCAATCCATACAACAATCTTTTCTCTTATTTCATCATTTCTATTTTCTCCATTTCTCCATTTCAATGCTTTCTGTGTGAAAAAAAAATGGAAAATATCATTATGTAAAATATTATTAGTGTAAAACTTGAATGTGTGAGTACATAAATCAAGTATTATGTATTGTATCCAACAAAAAATTCGGCAATAATAATAAGACAAATTAAATTTAAAAATAACTGCGTGTAACACACATCTTAAAAAGTTATGCTATTATATTCTCTAATTTTTTTTGTTGTAAATTTTGTTTCATCTTTCTTATCTTTAAAGACTTTAACAAATTCTAAGTATACCTTTAAATTATTATAATATACACCGGCATGGCTGTAGGCTTATCAAAATGTTAAATTGCCAATTGACTAGAGTTGTACATGCATATATAATGCATTCACTCTATGTTACAAATTAATTAGCAGCCAAATAATTGATAATTGAGAGTTTAAAACATTTTGAACCATGCCATACCATTAATAACCTGTGTTGCAACCATTTCTCACATATTGCTCCACAGAGCATACAGAACTGTTTTCTTATATCCAAATATATACTTGTATACTTAAACATATTTAACCGTATTTCAAAAACTTTTATATCACCTATTCTTAATTTTTTTATATAATGTCTAAATATTATAATGATCTCATCTTATCAAAAACTGACCCACCAAATTTCATCATCATATGTTCTTAAATTTAGGAACTGACTTAGTATTTATCATAAGTTAATGCCTTCTTTATTTAGCCATTCTATCTTGATATTTGACATGGTAAACTCTATCCACTCCTCTGATGGGCTACTGTATGATTTTTGGAATCAATATCCAGAGTAATCATAACAACTAACATGACTTCAATTATGAAATTACAATTATATTTTCTTAATTACTAGATATTCAAAGTTTGCTCATGGATGAATCAAATAGGTATTTTCAAATTATCTGATATAAATTATTTTATTAGTTAACCTATTTACACATACACTATAAAAGATATCGATATCCACCACAAAAATGATATTATTTTACTACGTTTTACTAAAACTTAACGGTGAGGTTAGCATAGGTCGGAGGACACACAAACATAAAACAAAATGATGACTTAATTTGATTCAACAGACATTTAAGGAACTAATTTGATCAAATTTAGTCATATTACATAAGTAATTTGCATATAAATATTAGATGATTTAATACATAAAATAAAATTGACAACCTTTGTCGTTAGAGTTTTAAAATCATGCTATGTCCTGTTAAATCAGCTTCACATTGGAGACCATTAGCTAGACTCAAATCTTTTAGCTAAATTGCAAAGTAAGCGTGCTAATTAAATTATATCAAACAGCCTTGGAAGAATGTGGGTCATANNNNNNNNNNNNNNNNNNNNNNNNNNNNNNNNNNNNNNNNNNNNNNNNNNNNNNNNNNNNNNNNNNNNNNNNNNNNNNNNNNNNNNNNNNNNNNNNNNNNNNNNNNNNNNNNNNNNNNNNNNNNNNNNNNNNNNNNNGCACCACTTATGTTTGTATGTGTATATATATAACACATGCACAGTGTTTTCAATCTTACCACCACCTTAATCGATAATACCGGCAACTGATTGATACAGTTTCCTAGTTTTCCTCGAAAGCGTTATAGTAGTTTCCATCTTCTTC

## >AiNAC34

GCCTTTTTGTCGCTTATTATTTTTCATGTGTGAGAGAGAGGTGTATAACAAGAAACTGTGAGGAGAGGTGTGTTTCATACTAGTATAGGATATACAAATCGAAAGCAACGATTTGTTTGTATTATTTTCTTTTTAAACAAACAATCACAAATCAGACGGTCCGATTTGTGATTTCGAAAATAAAAAAAAAATTTAATGTCGGTCGCTTGGTGGGTCCAACTCGTGGAAGCTTTAACAGCAGATTTAATTATACAATTTAATAAAAGGCTTAATTTTTTATATACTATATTGCGGACCAACCAACCAGAGGCCGCGTGCGGTTGCTCCAACTGAAAACCAATTATTATTTTACTAAATCCTTAAATAATAAATACTATAGAAAACATTTCAAATGTATCCAAGAGTATTTATGCACCAGTTATTTTGACTGTTAATTTTAATTAATATATATTATATATATTTTTATNNNNNNNNNNNNNNNNNNNNNNNNNNNNNNNNNNNNNNNNNNNNNNNNNNNNNNNNNNNNNNNNNNNNNNNNNNNNNNNNNNNNNNNNNNNNNNNNNNNNNNNNNNNNNNNNNNNNNNNNNNNNNNNNNNNNNNNNNNNNNNNNNNNNNNTTATAATTTTATAATTTAGATCAACGGTTAAAATAACTGGTGTATCGGTACTTTCAATGCACTTGAAATGTTCCAATACTATATACTATACTCCTCTTTCTTGTCTTGTCTCTCACTAATTTATTTGCGCTTTTTTATCCTCACCCTCATGCCGCAATGCACATAAATATGTGATATCTCAATATAATGTAACACACCACACTCTACATAAAACAAGCTGCTAGCTAGCTAATGCAACGTCCAACCACTGGAAGGCTGGTAGTCTATGCTAATAATTAACAAGCCTAACTAAATTATATCAGAAAACTTTGATCAACAATAATCAAGTAATGGTTATTAGGATATAAATTAACAATATTAGGTTATGCTATATCAGAACAAGGAGCATATATTATATATATATATGATGCTACTCAGCAACACATACATATAAATAAAGAGTTCCCCAAAAAAAAAAAGTTCAACACAGAATTTGAGACAAATTAACTTTAATGTTGATGATGAGTGTATTAGAAGTTTGTTACCTTTTTGTTTAATTGGTATGATGAAACTTCCCTTTTACAATTTCATTTATAATTACTACTAAAAATTTATATACTGCAGATTTTTAGCATTTATTTAAGGATAAAGCATTATTGTGATCCATCTAGAAGAACTGTACATCATATAGGTAGCGTTTGTTTTGAGGTACTGAGACAGAGACTGAGAGACTGAGACTCAGTATCGTGTTTGTTAGTTCAGAGACTGGTACTAAAATTTCTGTCTCTGTCTCTAAAATTTCAGTATTTCAGTACCTCCAAAAAGTAGAGACACAGGGGACTAAAATTTTTAGAGATGGAGACTGAAACTTTAATAACATTTTATACCTAAAATACTTTCATTTCAATTAATTAATTCCAATTTTACCCTTTGTGCAAATTAAATTAGAGTTTTATTTTTGTTTCAATTCCTGTCTCCCATTTTGCACCAAACAGAATACTGAGATTTATTTCAATCCCTGTCTCTTAGTCTCTGTCTCTCAGTCTCAGTCTTTCCGTCTCTGTCTCTCCACCAAACGCTACCATATAGTACCTGTGTTGCTATTATTATAACAACTACTAATATAAACAAAAAAGAAGGCATGCTGAACAATAAATAAATATATAAATCAAAATGTGATCTTGTACACCAAATACGATGTAAAAATGTTAGAAAACGCAAGGCTTTTTGAAATTTTGATGCCAGGGAGGATGTCCAGCATTTTTTCTATAAACTGCATGCTTATTCTTTATTTAATTGAAAGTATTAAATCCTAGTTTCTGAGAATTGTTTAATTTAGTCGTTCACATAATTTAAACCCAAAATTTTGTTTAAAGTGAGATGAAATGAAGAGAAATAAAACTCCATTGATGATTAGATATTGGGAAAATTTCAGCATATCATCAACTGAATTTTATTCGCTCACATGTCTGGATATATATCAACACTTGGTCCCTTTTATTAAAAAAGAAAATGTGCATTCGTGGTTCAGCATTTGATGAGTTGATCTATGCTATGCTGCATGGATCATATATATTCATTCTCTTTCACTCTCTCTCATTATTGTTTATTATATCATCTATAATCTCTTCATTCTCTTTCTGGGGTCCCACGCTGGCCCTCTGAAACGTTAACACCACATCATTCTTTTCTCGTCACCATCAGTTGCTGGTGGGTGGAGAGAGATTCCTCTAGCGCCACACTTTTATGAACTGAAAGCTTTTCCAGTAATCTAGCTCCAACTCAATACATCGATAAGGAGAGAGAAAAACTTTTGAGAGGGATCATTCTTTATTCTTCTTTAAATTTATATATTTGTTTCTTGGTGTTTTACTTCCTATTTAACCCTACCTCCTCTTCATTCTC

## >AiNAC35

ATGTGTAGCACACTGTCATCTGCTTCACTTTATTCTGCGGAGGTAACTCCCCAAACAGCTCACGGAACCACACCCATGCCGGTCTTCCATTTTCCATGAAATTCTCAAACTCACTAAGGCACCCACTCACAGGCTCACCATCAATCGGTAAACCCAGCTGATAGGCCACGTCCTCCAAGGTGACCGTACACTCCCCAAAGGGCATGTGAAATGTGTGGGTCTCAGGACGCCACCTCTCAATGAATGCGCTAAGTAGAGGCTCATCAACCCAGAACCACTGACTGTTCAGCCTAGCCAAGTGATATAAGCCTGCCGTCTCTAGATACGGTATGATACGCTCGTGTAAAGGCATATTCTGTTGTCTTCTCACACCACTAATAACCCTAACAGGCTGTAAAAAGAAAGATAGCGAACTCTCAACAGACAACAACAGCAACAATTTACAGTAATATCAATAAAAAACCCTAAGCATAACAACAATAGAGAAAATAACACTAACAATACTACTAACACGTAAACCTAAAAAATTATTTCTGCCAACAATATAAATTACAAATTCAATAAATATAAAATACTCACTATCATGATAACAGCAAAAAATTCTATTATAACTGTTAAAATATTAATTTAACATTTACCATTAATGATAAGAACATTAAACCAAAAATAATAATAAAAAGCCGGTTTAACCAACCTCTTGGTCGATAAATCCAGCCACATGCGCAACGCGGTTTAACCGGTACATCCGGTCTTCTTCTTCCATCGGTTCTCCACTAAACCGGTTCAACTTCTCAAACCAGCAACTTAACAAAAAAAATTTTTTTTCCCCTCTCACCCACCAACCTTCCCCTCACTCACAAAACTCACAAATGATCCGCCCTCACCAGCCACGGCTTTGCTATTTATACTACTCACTCATAAACCGTGGCTGCCTTTAGGGTTTTATGTTCGAAAGCATTTACTCATAAACCGTGGCTGCCTACCACGGTTTATGCTTCACATTCTCTTCACGTAAACCGTGGCTGCCTACCACGGTTTACATACAAAATGGTTTGATCATAAAACCTCCCTACCTCCCACGGTTTATGTGCATTTGGAATCCGTGGTTGGCTCCCACGGTTTCTATATAAATTGATTCAACGCAAAATGGTAACCAAATTCCAATTGTTGTAATTTGGTAAAGCTTACATCCAATCATTTTAATTTGGTAAATTGCCCAACAATTATACTACTCCTAATTATTGGGATGTTAAATTAAAATTAAAAATAAAATAAGTTGATCTGACAGACAAAGATTTCTGTGGAAGAATTCGCTCCCAGATCTCGAGAGTGTCAGAAACTGGATAGCAAAATCATGCTTCTAGAACACCCCTCCTGGATTTACACGTGTCGGAATGATCTCGAAGATTATTTTTTTTATATTATTACTAGGCAGGTATTAAAATGAAAAAGAGAAAAAAATTATATGTACATGTATGGTTCATGCTTTTTATTATGTGGGGGAATGGGTGGTTGTTTTTGTTGTGCTGGCATATATGGTTAAACGATTTTATTAAAAAATTGATTAAAGCGTATATATTGTGCCAATTACCAAAGGCAATGCAACTTTTTTTTTATGCGTTAAGAAATAATAATTACTGAGAATGTGTCATCAAATAAGATAGTCTATGATATATTTTAAAATAGTTATATTCTTGTTATTCAACTCAATACAGTGACTAATTAACTAGTTACTTAATTGTGAACTTGTTTAGTGTTTTTTCTTTTTCTGCTCATTTCTAAAGAAATGAGTTCTAAGCACGATCTCATACTTACGTGTGCCACACATATATATAGAAAGTTTGAATTCAACCCTTAGATTTTTACAACACTAGTCACCACTAGAAAATTATACGGCTTCAGCTTTCAATTTCAAAAGCATGAATCATCATCATCATTATCGCCGGTATTTATCAAAACGTATATAAAATATGAATTTATTTCTTAATTGTTCTTCGGATATTCTTCTTCTTTCTTCACGTTACTACTAAATATCCATTTCACGTGTTGACATCACCTTGCATTATCAAAAAATGTAATTCATCATGATATAATAATTATAAAACATTTTCTAGTAACAGAAATTTTGTTAAGAAAAAAAAATATAAATCAAATCAAACTGAGGTAGTGACGTGTGTAACAGTATATAGGTGTGAAAGAAATATTGGTACTTTTATATTTTCACTTTTTATTTTATTAAACTTTATTTTACGTTGTGACAGAAAATCCAATACAATGCACCCACCGAAACGATTGAACCGTACCGGTAACAGATAAGGTCGGCCGCAGAGAATGCTTTATGAGTAGAGGGTACGTGGCGCAATCTGACAGGACAGTAACTCAAGTGGTCCCCACACCCCAACATTACCGTACTTATCTCTTTCTCCTGTCTTCCCTATAAATACCTCCCTCTGTTTTCCAGTTACTGAAAAAGAAAAGAAAAAAGGAGAAAAAAAAAGAA

## >AiNAC36

ATAATTTTATTTTTATTGTTATATTATTGTTAGTTTTTCAATATATTACTGAGACTTGTTATGTCATAGTTGGTTATTTAAAATTTGATGTTGAGACTTATTATGTATATTTATCGTTTTTGTCTCTAACATTTGGGGTAAGTCTCAAAGTTATCTCTAATGTTTAAATTGTCCTGTTTAAGTCCCTAGCATTTCAAAATTGTCTCAATGTTATCCTGCCGTTAGGGATCTGTTAACAGAATTGACTGCGGAACAAAATTGAGACGATATAGAAATATTATGGAGTTAAATAAGACAAAGACATTGGGGGACAAAAATAATACATTACTCTTAAAAAAATTATCAAATAATATAAAAAGAAATTANNNNNNNNNNNNNNNNNNNNNNNNNNNNNNNNNNNNNNNNNNNNNNNNNNNNNNNNNNNNNNNNNNNNNNNNNNNNNNNNNNNNNNNNNNNNNNNNNNNNNNNNNNNNNNNNNNNNNNNNNNNNNNNNNNNNNNNNAAATAATTATGTATCATAAAAAATTAAAATTGATTAAAAAAATGAAAAAATAAAATTTAATATAATTAAACATTAAAAAGTTCGATATATTAGAGACAAAAAGATATAAATTTTACTTTATTGTATTTGTACATGCTCTTTTTTTTCTTTTCTAAAAGTTTATATATTAGTCATTTTATAAGTATGAGTAAAAATCTTGAATTTGTAATGTAATTTATTCCCTTAAAATTCATTATCATTTTACTTGATTTGGTAATTCTTCTAAGAGTAATGTATCATTTTTTCCTCCAATGTTTTCGTCTTATTTAAGTCCCTAATGTTTTAAAATCTTTTCAATTTTATCCCACCGTCAATTCTGTTAACATCACTAATGGCAAGACAACATTGAGATGATTTTGTAATGTTAAGGACTTAAATAGAATGATTTAAATGTTAAGGACAACTTTGAGACTTACCAAACGTTGGAGACAAAAATAATACTTTATTCTTTTTTTAATTTACAAAATTACAAATCCAATTCAATCCAAATCCCTTAAAATTAGATTGGATTTAAAAAAAAAAAACATCTAATCTAAGCCGCACTGCAAGTAAAATTAGTGTTTAGATCAGATAAGTTTTTTATTCAAAATCGATCCAAACAGCACTACGAACACCCTACTACTTATCCAATTATCCCTTTTATTTGGAGTTTCTTTTTTTAATTTTTATTTTTAACACTGAGTTATATAATTATATTTTAAAGATACCTATCTAGACAACTCATGACGTGTCACTTGTTCTTATTCCTCACAAAGCATGGGGAATAGATAGGAGGTGTGAAAATAATGGGATTGAAATGGGAATTTTGGATACACATGAATTGTTGTTATAAGGAAAAAAAAAATGATAATTTTTTCCAAAATCGGCCATAGAAGAATTAGGTATATAGAAAACAATCAATCATACTGAATATAACATTAGATATTAAGATTATTATATTAAGAACTAGGTCCAGCCAACAACTTACAAAGAACAATTTTAAAATAAAAATAGAATTGCCTTATTTTATTAGTAGAGAAAATGAAATAATTGTTACTACATTCAAAGAAGCTTGCTTTGTCATTTTCCCCTTGTCTTTGTATGTTGAAATTGAAAGGGAGAAGTTTATGAGCATCAGCAAGTGTGAAAAATGGAATAATCTCCTTTTCTTTTCTTTTTTCAAAAAAATAATAAATTTGAAGAAAAAAAAGGAAAACAAACAAAACAGTATTGAAAGCTATTGATTCTATAGTCTCTTATGTGATCCTTATTTTGGTCTTTCCCAAAATCATTATATATTAATACTGGTCTCAAATGGTGGAATTGAGACACATCCATTAAAGGGTATGTTTTTATTTTATTTTATTTTATTTTCACTTGTCCTTCATTTAAGTGACATAATTCCTATTCACTAAGGAATTTTTGTAATCTCAATTAGAGAAATATAAATATATCCTTAATGAAAGGGAGGCAGAAGCTTAGGAAGCATATATAAACATTCTTGGGAGGCACATAAATTTCCTTGGACTAAACAAATTTATTGTAAGTGCAACAACTTGAAACTTCTCAACATCTTTTTTTTGAGGTATACTGTTTCATCAATTTATTCTATCTTTTTGTAGTATTATTAGAGAAAACAAACATCACATTGATAATTGGTTTTGTTTGGTTTTTCCAAAAATTGTTGCAAACCACAGTTTTTAATATTCATCTATAGAAAGATGGTAGTAATCTAGCAATTAAATCTTACATAGTTTAATTATATATATACTTAATATGCTTTTCTTCTCATTATTCTACAAATTTATCAAGAAGTCTTCTCTTATATATCTTCGGATTTGATTGTGAGAGATACAAGAGTTTTTTTTGAAACCGACGCTAAAAAACTGTCGCTATCTGTCGAATTTGGTATAGTGAGAAGTTGAAATTGGAGATGGGGGAAAGAAATATTGAGATGGAGAATAAGATTGAAGATGAGATG

## >AiNAC37

NNNNNNNNNNNNNNNNNNNNNNNNNNNNNNNNNNNNNNNNNNNNNNNNNNNNNNNNNNNNNNNNNNNNNNNNNNNNNNNNNNNNNNNNNNNNNNNNNNNNNNNNNNNNNNNNNNNNNNNNNNNNNNNNNNNNNNNNNNNNNNNNNNNNNNNNNNNNNNNNNNNNNNNNNNNNNNNNNNNNNNNNNNNNNNNNNNNNNNNNNNNNNNNNNNNNNNNNNNNNNNNNNNNNNNNNNNNNNNNNNNNNNNNNNNNNNNNNNNNNNNNNNNNNNNNNNNNNNNNNNNNNNNNNNNNNNNNNNNNNNNNNNNNNNNNNNNNNNNNNNNNNNNNNNNNNNNNNNNNNNNNNNNNNNNNNNNNNNNNNNNNNNNNNNNNNNNNNNNNNNNNNNNNNNNNNNNNNNNNNNNNNNNNNNNNNNNNNNNNNNNNNNNNNNNNNNNNNNNNNNNNNNNNNNNNNNNNNNNNNNNNNNNNNNNNNNNNNNNNNNNNNNNNNNNNNNNNNNNNNNNNNNNNNNNNNNNNNNNNNNNNNNNNNNNNNNNNNNNNNNNNNNNNNNNNNNNNNNNNNNNNNNNNNNNNNNNNNNNNNNNNNNNNNNNNNNNNNNNNNNNNNNNNNNNNNNNNNNNNNNNNNNNNNNNNNNNNNNNNNNNNNNNNNNNNNNNNNNNNNNNNNNNNNNNNNNNNNNNNNNNNNNNNNNNNNNNNNNNNNNNNNNNNNNNNNNNNNNNNNNNNNNNNNNNNNNNNNNNNNNNNNNNNNNNNNNNNNNNNNNNNNNNNNNNNNNNNNNNNNNNNNNNNNNNNNNNNNNNNNNNNNNNNNNNNNNNNNNNNNNNNNNNNNNNNNNNNNNNNNNNNNNNNNNNNNNNNNNNNNNNNNNNNNNNNNNNNNNNNNNNNNNNNNNNNNNNNNNNNNNNNNNNNNNNNNNNNNNNNNNNNNNNNNNNNNNNNNNNNNNNNNNNNNNNNNNNNNNNNNNNNNNNNNNNNNNNNNNNNNNNNNNNNNNNNNNNNNNNNNNNNNNNNNNNNNNNNNNNNNNNNNNNNNNNNNNNNNNNNNNNNNNNNNNNNNNNNNNNNNNNNNNNNNNNNNNNNNNNNNNNNNNNNNNNNNNNNNNNNNNNNNNNNNNNNNNNNNNNNNNNNNNNNNNNNNNNNNNNNNNNNNNNNNNNNNNNNNNNNNNNNNNNNNNNNNNNNNNNNNNNNNNNNNNNNNNNNNNNNNNNNNNNNNNNNNNNNNNNNNNNNNNNNNNNNNNNNNNNNNNNNNNNNNNNNNNNNNNNNNNNNNNNNNNNNNNNNNNNNNNNNNNNNNNNNNNNNNNNNNNNNNNNNNNNNNNNNNNNNNNNNNNNNNNNNNNNNNNNNNNNNNNNNNNNNNNNNNNNNNNNNNNNNNNNNNNNNNNNNNNNNNNNNNNNNNNNNNNNNNNNNNNNNNNNNNNNNNNNNNNNNNNNNNNNNNNNNNNNNNNNNNNNNNNNNNNNNNNNNNNNNNNNNNNNNNNNNNNNNNNNNNNNNNNNNNNNNNNNNNNNNNNNNNNNNNNNNNNNNNNNNNNNNNNNNNNNNNNNNNNNNNNNNNNNNNNNNNNNNNNNNNNNNNNNNNNNNNNNNNNNNNNNNNNNNNNNNNNNNNNNNNNNNNNNNNNNNNNNNNNNNNNNNNNNNNNNNNNNNNNNNNNNNNNNNNNNNNNNNNNNNNNNNNNNNNNNNNNNNNNNNNNNNNNNNNNNNNNNNNNNNNNNNNNNNNNNNNNNNNNNNNNNNNNNNNNNNNNNNNNNNCTTGATGTGTAATACACATTTTTTACGAGTATTACAAAAAATAAGCCTTATATGGCCTTTGGTGAGTCAAAGTATAAACACTAAAATTTACCAATCATATCACACTGTTCTTAAGGCATAAAAAATTTCTGACTACCGACTATGAAAATTGACTTGGCCTTTTCTGCCTTGTGAAATGCCAACCAGAAAACCATCAACAAGACCACAACTCCACAAGAACCTTAGACTTGCACAAATGATTAGTCAGAGTCAATAGCATCAAATTAACAACCGTAACTTGATGTGTAATACACATTTTTTACGAGTATTACAAAAAATAAGCCTTATATGGCCTTTGGTGAGTCAAAGTATAAACACTAAAATTTACCAATCATATCACACTGTTCTTAAGGCATAAAAAATTTCTGACTACCGACTATGAAAATTGACTTGGCCTTTTCTGCCTTGTGAAATGCCAACCAGAAAACCATCAACAAGACCACAACTCCACAAGAACCTTAGACTTGCACAAATGATTAGTCAGAGTCAATAGCATCAAATTAACAACCGTGCCACCACCAATGTACTACTATTCTATACTCTTCTTCGAGGGGGACACTTTTCTTTTCTTTTCTTTTGTTCAACATTCACAATTTCACACTACTTTATCATTATCTACCATGCACTATTACTAGTAATAAGCTTCTAACTTCTTTACCAAGAAACCAATTTTTGGACTTTGGACTTTTTATGTAATACTTATTAGTCCAAATAAAGG

## >AiNAC38

TGATCCACTATGCCCAGCCTCATGATAGTGCCGACGATTCTGACAGTGAGGGCAATTCAACCTATGTTGCTGGGTCCAGGTCGTCAAGCGATACAGTATCAGAAGATGAGTTTGTACCGGAGACTCCCAGCAGCGTGTTGGTAGGTTTCTGCTGCCTCCCCCTTTGGCTATTCCTCGACTGTCGGATGTTCCTAACCATTATCAGACGTTAAACTTGGATGCAATGCAGCCGAACGATCTTTTGAACGCTGGGGACAAGGAGGATTACAACACGGATGGTGGGGTAGAATTTTGAATTGGGCATAGATTCAGCAACCGAGAAGCATTCCTAATGGTGGTTAAGAACTACAATATTCGACGGAATGCGGAGTACAGAGTTCTGGAGTCAGATAGGCTTAAATATCATTGTCAATACAAGAAATTCACCAATGGTTGTAACATTGCGACTGGTACTTGGATGCTTTGGGTACACTATCCTTGAGATGGTGGATTGGGCTCTTCGTTTCAGGAAGGATTTGTGGTTGCAACATTGCGACGAAGGTCGTCGGTATGGTCACATGACGACGAACCTATCAGAGTGTATAAATGTCATGTTGAAGGGAACGAGGAATCTTCCAGTGGCAGCGATTGTTCGGGCAATGTATGAGAGGTTGCAACAGCTGTTCGTGCGTAGAGAACGCGAAGCACATGCTCAGTTACAGGGTGGACAAATCTATTCACAGTAGCTGTTGGCAGCTATAGATAAGAACGGAGAGAGCCTACCGATGATGCGAGTCACCCATTGTGATCGTAGGGCATCCATTTTTAGCGTGGAAGAGATGGAGCCGGTGGATGGTTGGTCACAGACTTCATATCGCGTTCGTCTGACCGAGCGTACATGCGACTGCAGCCTGTTCCAGTCATTGCATTACTCATGTCGACACGCCCTGGCAGCATGTGCACGATGGCCTCTGTATTCAAGGTATACGAGAGGGAGTTTTCGCTGATACCAGACGAAAAGATGTGGCCTTCATGGTACGGTGCACGCCTGAAGCCCAACTCAGCCATGCGAAGGAAGGCATCGGGAAGGCCGGTATCCACTCGGATCTGGAATGAGATGGATGCCATTGAGCGTGCGGAGAAGAGATGTGGGCTCTGCTGTGGAGAGGGCCACACCAGACGTGGGTGTTCCAATGCGTCCCACTCAGATCCATGACGACGCATGCAATTTAGAGTTCAGGCCTTTTAGTTCCAATGTTGTCACTTTGATATAATGATTGTTAACTAATGTGTTTAATGTGTAATGAAGCATGATTTCTATATAACTAGTTACTTTCGATATTACATTTTTTATCTACAACATTACAATATTTTCGTAAAAAAAGCATTCACTAAAATGGATAAACAAATAGAGGACAAGTCTGGTAATACCCTGGTAATTTCTCCTTATCTCGATGCAGTAACCCGAATCAGAAAACACGCGTATCTCGGGTGCAGTGTATCTGAGATATGCTTATGTTTATATCGCGGTTGCATCCGAAATATGATTCGCAGACACTAGTCTATCACAGTATGGGGTCAGTGCACCTGAGATGTGGCCATTTTGGACAATGATCCTCAGTACTCGAGATGTGACTGAAGATGAATTTTGGTAATTTCTTCAACATTTATTTATTTTGATAGATACTATATTTATTTAATTTAAATAAGAAAGGTATTATACGTTTAAAAATATAAATATATAACTGAATAAAAGAGAAAAGAGCAACCTAGTGTAAAGTGTTATAGAAAATGTAATATGTAAATAGTCTAATATATATAATGCAAAGTTTCAGTGTTTAATTTCGTAATTTAAGCCAATAACATATAAAAAATCCTACAAATACATCTTAACTGATATCCCAAAATTTTATTTTTACCTAAATAATAAATACTAATGTAAAAATTTTTTACAATGTTACTGCACAGCAATGAATATCTGACTCTTCTCAAGATTAGCATATGACTAATCATGAAGAATTCAAAGTTACTTACATGATAACAACAAATTACCTAGTTCAATTAAAAAGAAAAATAAATAAATAAAGCGTCTGGTGATAAAATTTGTAGTGATAAAGTATAAAGAGTATCCAGTTACATAAGTGCGACAACAAAGTGATACTCCATCAACATACACTAGCATGGTGATAATTAGATGCATGGTATTTGGGCAAGTGAGCAGAAAACAAAATTAGCCAAAGTATTATGACACAATAATTATCTCATCTCATGATAAATCAAATAATTTGGAATGGGAATGAGTGCTTTAGCCTTATAAACAAAAAACGAAGCAAGTTGGCTTACATGCCACGTTTCTCTTTCTCTCTCTTCCCCCAGATAGCAGTAGTATTTAGGTGCCTCCTTTTTCTCTCCTTCGTTTTCCCTTCTCATTATTCACATCCATCTCCATATTCCATCTCTCTTTCCTCTCCTCCCCTCTCTCTATATATTGAAAGTAAAAGAAGTAAGAAAGAAAGAAAGA

## >AiNAC39

TCATCCCATGAATAACTTGCCAGAAATTAGGGAAATAGATATCAAGTCTTTGAATATTCCAAAACCACCCCAAGTTAATTGTGAGGTAAATGAAGCATTGAATATTTAATTTTAAGATTATTTTAATTTTGTTTTGTGTGTGCGTGAAATATAAATGAATGAGCATAATTTTTTTTTCTTTCTTTTTATATATACTTCTTAACAGGAATGAGTGTGAGGTGAAAAAAAAAAACAAAATAATGTCTAAGAAAATAATTGCAACTTTTTTATTTGAGTTAATACTTAAAATAATTTTTTAAAGTTAATTCGATATTTAAATTAGTATCCAAAAAATAAATTAATCAAATTAATTTTTTAAAGTATCATAAATAATTAATTGTATTTGTTTTTTTATCATTTATGTGGCTGACATTGTTAAAAAATTTTTATGAAAAATATAATTAAGATTAATATAATCATAGAAATACTAATCATACAAGAGATTTCATCACTAATATGAATATGTTATGATAATTAAAGGTTAATAAAATGATGAGAAAAATATTCTCTTTTTAACCTAAAAAATAATATTTTAATAATTGGAAGTTGTTGAACAATAATTTTAGGTTGTTAATGACTGAGTTTAAGTTGTCCAACAATTTTAGCTTGTTAAAATAATACAATAAAACTAAAATTAAAATAAAATCGTATTAGATGTGTATGAAATTATTAATTAAGAAGTTGATAAGAATTAAAGGTTAATAAAATATAGTAAACATGTTTTTTTTTTTGGAGAAGTTTAACATTCAAAATCTGATGGAAGAGCCTCCTACTAAAAACCTTAAAAGGCCCTTCAGAAGACATGGAATCGAGATGGAGCGATAAGAAAAATGAAATTTGATTTTTGTTTGTGTGAAAATAAAAGTTGAATATAAAGCAAATATATAATTATATTACTCTAAATAATTTTATACAGTTCAGCAAAAATTAACGATAAGAGAGCTTAACAAACTGTTTAACTAAACAAAGGAGTACTAAAAGTGTATTATTTTTAATTTTCTATTAAATAATAATTAGCAATTAGTATAATCAAATACCAGTAAATGTTATATAATAACAAAAAATATTTGATAGAACAAGCTAAGAATGTTAAAGATGAAATAAAAATAATTTTGAGTCACTTGTTTTTGTTATAGGAAGTTATAATAAATTAATATATTCATGAGTTTTAAAAAGCTCATAATCTCCTCCTACCAAAAAAGAAAGACAACAATAAAATCATATTAGAATTTACAAAGATGAGAAAACAATAGTAAAAACTGTATCGTTAGCATAAAATGCATAATAATCATTTAAAAAAAAACCAAAAAGAGGGTATATATAATTAACTTATTGCTATTAAAGATAAAATTAAATAAAAAGAAAAGTACAAAAAATTAAATTTTATATTTAAAATTTAAAATCTTAAATTATAAATTCTAAGTATTTATTATTAAGATTTAATAAAAATTACCTGTACAATACTTTTTACTTAGTAAAAGATATTTGAAGATAAATATATAATTATGACTCCTAACTCCTAAGAATTTCTCATCAACAAGTGATATTTCCTTGTTGAAATAATAAAACAAGTAATGATTTTGATTTGCAAAATATATTTGGCTTGACAAAGAATATATACATAGATGTACAGTCCTTGAACAAAAGGATAACAACAATGGGCCATATATGGGAAATGTGCTCCAAAAATGGTCCCAAATTACTACCTCATAAATAGAAAAAAGAAATAAATCACCCAGTGATATATTTCCAAATTTGCCACTGTAAATTAGTAAAAGCAAGCAAACCCAGATTTTCTGGTTTCTTTGGAAGTCCCATCTGAATCCCTTATTATCCAATACCATTTGGAAATTATTCATTTACCCTAGTGCTACCCACCAACTAAAAGAATATCTCTTTTAGAAAAATCTAAAGAAATGAAAAGGATTTTTTTTTTCTCGAANNNNNNNNNNNNNNNNNNNNNNNNNNNNNNNNNNNNNNNNNNNNNNNNNNNNNNNNNNNNNNNNNNNNNNNNNNNNNNNNNNNNNNNNNNNNNNNNNNNNNNNNNNNNNNNNNNNTTAAGAATTTCTTGACATTGAAGGTACCCTTTCTCTCTCTCTCTCTCTCTCTCTTTTGTTGCTTGACCCTCAATCAAAGACGCCCCATCTTTTCCGTTTTGCTTAGGCAAACTTCACACAGACACAGACTCTTCGAAGCCCTAATCTCTCTCCTCCCGAAACCACCATGGGTCGTGAAACCCTTCTTCATCCACCACCACCATCCACCACCACCGCAACAGCCACACACACCCCACCACCGCCGCCGCCACTGCCGTCTCTACCTTCATTGACCCAAGCAGGACCATCAGGAGGAGTATCAGCATCTGCACCTTCACCATCTGCTTCACCTTCTTCTCCTGCTATTGTTGCTACTACTGCTGTGGCTACTGCAGTTGCTGCTCCTCCTACCTCTCTTGCTCCTG

## >AiNAC40

NNNNNNNCAACTAAAAGTTTATTTTTTTATATTTTTCTTTTTTTTATTTTTCCTTCTCCCCCTATATATTATGCATTTTTTTTTCCTTCATATTCGTATTATGCATTTTTTTTCCGCCTTCTCCTTCATGTTATGTGTTATTTGTACAGAATCTATATATGTGTTGTTCTGTTATAAATTTTATGTTCTTTTGCAAAAATGTATGTTTTGGTTTTAAAAATTTTCTATATTTTTTCCATGAATTCCTTTGTTATTTTAAAGAAATTTTTGTGTCGTTTCAATATTTAAAAGAATGTTGGAAAAATTTTAACTTTTTGAAAATTTAGTTAGTTGGACGGTATATTTTGGAGACTTATTATAAAAATACTTATTCATCTAATGTATGTTAGATGAATAAGTATTTTCATTAATTCGATGGTTTATTTGTAGTATATAATATTTATATGAATATTTTATTTGTTAAACAAAAANNNNNNNNNNNNNNNNNNNNNNNNNNNNNNNNNNNNNNNNNNNNNNNNNNNNNNNNNNNNNNNNNNNNNNNNNNNNNNNNNNNNNNNNNNNNNNNNNNNNNNNNNNNNNNNNNNNNNNNNNNNNNNNNNNNNNNNNNNNNNNNNNNNNNNNNNNNNNNNNNNNNNNNNNNNNNNNNNNNNNNNNNNNTTTTTAATCGTTGATCTTAATTATATATATTATATATTTTTTTTTATAATTAAGATTAACGGTTAAAAATCACTGAAACACCTATATGACGGCACACTTGAAAATTTTCCTATAATTTCACATAGGAGATTCCAAAAACCTATCCAACAAACCCTAAGTTTGATTAAATCGTTAAACCAAATCCAACAACTCCAGTTGAAAATAGTCAATCACCACCGCCAATCCAATATTAGATGAACATATATATATAGAAAATTTTAAATTTCAAAATTTTAATTAGGTCTTAATTAATTCTTTTGCTAATTAATTATCTCTCAATACTTGTTAATACATAATTGGTAGAACCATGGAATCTGAAATCATATTTCCATGACTCTAAGAAAAGCATGCAAAATATTTAATTTTATGACCAATCACTTTATATTTTATGATACAAAAGGTGGTGGTCAGGCATAGCAAAAAAGGCAAAGAAAAAGATCTTTTTAAATTTGATATATATAATATGAATTTAATTTTGATGCACTGTGTCAATGTAAAGTAAATTGTAATTATGCAATGTCATATTATTAGTAAAAATAACTACCTTTTATATTAATCGCATGAATAGTCATCCAAAAGAATAGATGTAATTACACGACTGTATAAAACGCTTTACACTGTCAAAAAATACCATTTAGCAGCGGTTACCATTAACCGTTGTTAAAGGTTTAGCCGCACACTATCTTCTAATGGGTTATCAACTGCTGTTATTCGTTTGACAGCGGTTAAAAATCACTGCTAATCGACTGCATAACCGCTGCTATTTGCTGGATGTGGTGTAGTGAAACTCAATATAATATATAACAACTTTTAATTAATTAATTAAAATTCCAAAGAACAAACTTAGTCAGTTAAGCATTGTCCCCTTTATTACATGCTTAAGTTCCCTCCCAACTAACTCCAAAAAAATTAAATAACCTAGCTATATATAATATGCTTATAAATAAGCATACACAAAGCTTCATTTTATCACCAATAAGTTGGTCCAGTTTTCTAAGTTTTTATTATTTATAATTATTTTATGATGAGCAAGAAGATGAGGTTTGTTAAGAAGAACAAGAATGGAGTGAGATTATTGCCACCTGGATTTCGGTTCCAACCAACAGAAGAGGAGCTTCTATTTCAGTATTTGAAATGTAAGGTTTTCTCTTTTCAGTTGCCAGCTTCAATCATTCCTGAGATCAATGTATGCAACTATGTTGCAACTACGATCCATGGGATTTGCCAGGTAATCAATATAAAAATCTTTACCTCCTTTTTTTTCTTAGTTTTAGTTTCACTCTTAAAGTTACCAAATTATAGTGAAGTATAATTCCGTTTAGTCTTTGCTTCTTAAAATGTTTAATTTAAAAATTTCTTCAAAAAAGGAAAAAGTTAAAGATAGACTAAATTAATTAAATTAAATTGAAACTAAATTGACATTATCTAAGGTTAGAATGAAACTAAGTTAAATTAAAATATTATTTAATTTTTACTCATTTTTTTTTTTTACATATAGCTCTAGGAATAATTAACTAATTAAGGTATTTTTACAATATGTAGTATAAACTCATCATCATTAATGACATATTTTCCATGCAAGAGAGCTCATTAGTATATGTAATACAAAAGTATTACGTGTAGATTAAAAATCATCGATCAAAATCATAGTAATATCTTTTAATTATTACTAAATTGATTAAAAGATGAAGAGAAACAAATATAAATAAACAAAATCACATTAACATAGCATTATTTATTAATGTCATAGATATATATGCTCTTATAACTATGATCTTATTATTTTTTTTTATTTTATTATTAG

## >AiNAC41

TTGCATTTGATGCAAAAATTACAACCACGATTACTTTATAAAAAATGAGATATGTTGAAGGAGTGCTAACCTAGATCCTAAGTTGGGTTAGCCCTTCAGTATAGAGGATGAAATTCTAATTTGATACCTAATTTGGAAAATGATAATTGGAGTGGTACCTGTAAGAATTCTAACAATTAAAACAAAGAAAATTTAAATAATTAATATTCAGGATTATAGAATTAGAATGTGAATACCTTTTGCATAAACCATACCCACAAATTATAATAGTTGGTCGATATTAAAAAAATGTTAAGAACATATTATCCAAAAATTTGAAAATCAAATCCTTTATTAAATTAATAAATCTAAAGACCAAAAAATTTAAAGATTCACCTGAAATTCAACCAAACTTCAACTAGAGTTTAAATTATTCAAAATATATAATGTACTTTTGTTACCGGTCCAATCCCTTTGGGGACATCCATAAAAAAATAATAAAAAAAATTTATTCATCAAGTTCCCTAACCCCAATGCAAGTCATTCTAATAATGTATGTATGATTGACATAGGACTTTTTATGTGTTTGCTAACCAACCAGAACCAGGTAAGAACAAACAGAAACACCCACACTTAATTGTCCTTTGCGAACTTGAATTGAATTAGAGACTCATACATGCATATGTGTGACCACTTGAAGGGGATTGTAAAATGCTAAACAACGAAAACACTTTATTTTGGGACGATCCACAAGCTAACCTAATAAGCAATTAATTAGTTAGTAACCTTCATTCATTATTTGTTATTAGTTTTTTTTTTTTGCGACACTAATTTATCGAGAATCAAAATTTTAATAAAATTTTATCACTACAACCACTTGGTTGATTTATGTACAGAATTCAAATTTCTAACATTTGCTTAAATAAATTATGAATTAATTACTAGATCAATTTAAGTTGGTTTTATATTATATATTATTATTCTAATGTATACATATATATAATCTTTTCTTGAAGATAACGAATTGATTAATTAATTAAGATGCCTGAGAATCTAAAGAAGCGTTGGACTAGCTAGCTACTACGAATGAGGTCTCTTAGCTTGACATTTTAGACTAATACCGAACAAAACTGCTTTTTCACTTTAGACATATAAAAGAATGATTGATAAATAAAATTTGTTAAAGTAAAAAAATTGATGATTGCAAATAGGTTTGGTGGCCACAGAAAAGGTATTTAATGATGAAACTTTGTGTAAATAATTTAATACTAATTAATTATATATACTTAAAAGAACACGACACATACATGAACGGGAAGGAAGATAAATTGTAAAATATATAAAAATGGGTAGTTTATTTTCACCAATAATATGTAGCTGCGTGTGGGGTTCTATACGGATGCCTCTTAAGCTGTCAACAATCTTAATCTGTGATGATCCGCCATTAAAAACAGACAAACAATAAACAAAAACAAATAAATAAATAATGTACGTGTAGTAGTTACCGTTATATATATATAAGGTGTGAAAAAACAAAGGAAATTCAACTTTTAACACAAACTATAAAAGGGGCATTTTGGAGGGATTCTAAACCGTAATTATGTATCTTGATGATTTCTGCCCTTAGCTTGTTATAATCAAAATTTAATTTCTTCTAACTTTGATTTTTCTTAGAGATTATTTTTATTGTTANNNNNNNNGTCTTTCATTCAAAAAAATTTATTAAAGATATAATTATTTATATTTTTTTTTTATAAGGTTACAAGTGATTTTGTAATAATTATGTAAATAGTAACTAAAATACTAACAAATTCGTTAGATAATCAATATGCAATCAGTCAACTAGTAGCCAATTTATCATTAATTGATAATTTTGATTTTTTAAATCATCATTAATTAGTGATTATTTAAATTTTATTTTTTAAAAACATAAAATCAATAACTATTATTAATTACAGTTATTTAAAATTGACTGATTAGGAGTTCTTTATCTATATTTTTTGGATTTGGATTTTCTAAAGTTTGAATTTCACTTTAGAGAATAAAGTGTAATTTCTCATCATTTATTTTATAGGTGGGACTAATAATAAATATGAGAGAGAAATCATTCAAAGATAGAAGATCACACTTTATCCTCTAAAATACAAATTTAAAATTTAGAGGATCCAAATTCTACTTTTTCACTACTAAATTATCTTATGACACAAGCATTTCCCGGTCTTGGTGGAATTAATTAATATTGTAGTAATTAATCAAGAATGAAATTCTATTTTGGAAAACTGTGTTCTCTGTTCTCTNNNNNNNNNNNNNNNNNNNNNNNNNNNNNNNNNNNNNNNNNNNNNNNNNNNNNNNNNNNNNNNNNNNNNNNNNNNNNNNNNNNNNNNNNNNNNNNNNNNNNNNNCACACCTTAGTGGCTCTCTATCTATAGGTTTAACGTTTTATAAATAAAAAAATAGTTTTGCAAAGGAAGGTTTTGGTGAATTGATATACATTAGGGAAGTATCTGATAATTGAATCTGAG

## >AiNAC42

NGACTAACATTAATAAAAAGTATTAAATTTTTAATATCTTTCTTATAAATTAACAATAAGTACAAAATATTGTGATTGTTTTCGTCTAACAAATGCACCATTTTTAATAAATGGTATAAATAATTTGTGCAGCAAATTTAATTATTGTAGAAAATATTTAAATAATAAGTAATGCTATATATTTAAGTCATTTTATAAATTAAGCTTAATTAAATTAAATAATAAAATTTAGAATAATAATGTTAGTTACAATTGATTTTTATTATATTAGATCAATTTGATTGAATTTAATTAACAAAAATATTTGAATAATGTGTAGTATTATTACTTAAAAAATATATGTAAAAAACTAGATATAAAATTTGATTGATAGAAAATTATTTTTTCATTTTGTAACTTTTAGTCATCATTCACGTAAAGTGAAAGTAACGGAAAAAATTAATTTAACATAAAATTACTAAATTCAAAAATAAAAAAGTCTCATTCTTCAAAACATCTCGTAAAACAAAATTGTACCCAAATCTGATTATTTCTAACCTTTTTTTTTTAAATACATCTTTAGATATTTGGTATACTTTATGAAGAAAAAATAATCTTTCACAAAATTCATGAAAAATGGGATTTATTTTATGAAAGATTGATTGTTGTGGGAGGATAAAAGACACAAGGTTAGGAAATAGAGAAAGGAGAGAGGGAAAAGGTAGAAAGAAGGGAAGAAAAAGGGAGGGAGAAAAGAAACAAAAAGAAAAGAAAAAGGAATGTTAAGTGGGTGGTATAGGCAATGACGTCACCTACTGGGGGGACAACCATCAACCCATGCGCATTTCAGCGGTCCTTCTTTCTTCTTTCTTTTATTTATATTCTCCATTCTCTCACTCCTAACTGAATCTCTTCCTTCAACTTTCTCTCTCTTCCTCTTCCAATTCCATCTCCTTCTTCGTTCTTCAACCCTACTCTTAATTACTCTTGTCACATACATTCAGGTGAGCTTATATTCTTACTATTATTCTTCTATCTGCATCTATCTCTAATTAAGTATTCATATGGCGGGATCTTATCTTAGCTTCCATGCATAGATACATGTTCCCACGCAACACCATGAATGTAGTATATATCATTCGTCATATATCAAGACATAAAACAATTGCAGCTCCAAAACTACCACTAGTACGTAGTTTTCATCGTACGATCATGAATGTATCCGATTATCTTTCTTTCCAGAAACTTCCTTCTTCGCTCCTTCGTGAATATTGAAAATCCAATTTCACCAAATAACTAATGACGAGGGGGCCGGGTCGGGGGCTGGGTTTTGTAATAGAAATAGAAATAAAAGTCATGAACTACTGGTGGAAATTAGAAACTAGTAGAAAGAAGCTGCAAAACATATAGGGCACAGGCACATACATACATTCCCCTTAAAGTTGTGTATTTTTAGGGTGAGCACCACACCACATTCTCAAAGTCAGATATGTGCCATGAAAATTCTCGACAAGCAGACAATAAAGTCAGAGTCCCCAGTTTCCCCATTCAGAAGACTCACACTCACACTACATACATACACTCTCTACTCTTCATTCTTTAATTTCTCTCCTTCAAACGTCTTTTTCTTCTTTATATTATCTCCTCCTACAAACTACGGCAGGCAGGCCTTGATCCAATACAACGGTACCTCCCTTTCCACAAACGCCCGCGCGTGCCCCCATTCCTCATAACGTCACTCATATACTATATTCTTTTCTTTAATTTAACTATACCTACCTACTATATACTAATCACTAGTAATCAGATGCCTCTACACCTCTATTACTATTTGTTTTTCCCTTGCTTAAGCTTCAAATTAGAACTCTTATAAATAAATATTATTATATTGTTTCCTAAAAAAGTTGGTTTTGTTTGGTTATGGTTTNNNNNNNNNNNNNNNNNNNNNNNNNNNNNNNNNNNNNGAAGTGGTTATAAGTGAAAATAGATACATAATAATTAATAGTAATGTGGGAATGGGGAACCCCGTTCTTGAAGAATGATTCCCTCCATTCACTAACACAAGACAACATCATTAGCACAAGCCTCTACCTGTCCGTTTCTCACCCTCTCTTGCATAACACATATACTAGTAGTGCCTAAGCCTCAACTCCTTCATCTCATGGTTTTTATCAAAATACATTTATACCAAACCCTCATCATTTGGTTCTCTCTCAACCGGGGCCAGCAGCGAACAAAAATCATTATTGTTTTCTGAATTTATGGACTATTGTTAAATAACACGCGCTTATATTTTATTTATCCCTTTTCATTCCGACTAATTTTTGGTATGACTAGTGTTGCTCACCACCAAATGACCCTATGATTATCTCTGATAACACTTGTCACCTTCTTCTATTAATTACCTCATTCCTTATTATTTTTTTAACTTTTTGGTCTCACTTCATCTATCACTATTCATTCGCCTTGGTTATTATTTATACATGATGATGAACAAGTTATGTGGTGTGTGGTGAATTTG

## >AiNAC43

ACATTATTTTTATTTTTTGTAAAACAGTAAAAATAAAAAACTAATAATCAACCATGATGAAATTTAAATATGCGAGTTTATTCATTTTTGCTAAGAAACCAATTTTTTTTTTTCAGAAGCAGCCAAACAAATAAAGTAGTGTCATGAATAACAAAAATAATATCTAGACACTATACATCTTAGCTTTGAGCAAACCCGGAGCTTTGTACCAAAAGCGAAAGAAGCAGCCCGCATGCACGAACCCATAATTTGCGTAGTGCTATGTATGTATCCATGCCATTCACATATATCGTGTTTTGGTTCATACTCCATATCCATGCTTTTGAGAATGAAACATATCACGTTTTTGTATAACATAAAGTTTGTTCTTCTCCTCCTTTCATTCACCCTTTACTCCTACAAAAAACCAACTCTAATCCCTATCCATAGCATCTAATGGACACGTGGACATACATACCTTGGGAAACCTCAAAATTATCAGAAATTATATTGTACTCATAAAAGATATTTAACTTAATAATAAATCATAAGGGGTGTTAGGTAAATAATAACTATCTTAAATAACATGAACAACTACCAATCAAATAAAAATACACTACATCCTAATTTAATATTACTAATTAAATTTACTCTTTTAATCCTATTAATTCACATTGTTTACACATTGTTAAAAAATATTATTAGTTACCTATACTTTTCCTAAACCATAAGTACATAAAATATTTTCAGTACAAATTAAAAGCACACAGGAAACAAGAATGCATGTCAAAACAAAGCCTTGAAACCCTAGTGTTGAAGGCGTTAAAGTTGATAATAGAAACACAAATTGCAACAAAACGAGGAGCTTAACGGCCAAGTTTCGAGTAGCTTCTTTGAGAAGCCCTTCAAGACGTGTCGGTTGGTGTATACTATATTGTATTGAAAATATATAGTTGAGAAATCTTAAGGAAGGATTAATGCTAATATGTGTTAGCGTCTTTTGTCCCTACCTATTACTCTTATGTTTCTTTGTGATCCAAAGCAGTGGTGAAAACGTTCAACAACTTCGGCCGCAAGCGAAAATCTATTGTACTATTTCTTGATAAAACACATAAGGTGATGATGACCATGCGACATACATGCATTCATCATATTATCATTATCATCACCAGCTAAAGCCAAATGATATCAACGGCACACGTTCAAATATTTGGAAATTCAGTGTCTTTTGAAACTTTGAATTAATATATATTCTCTTGATTTATGTGAAATTTGTTTTCTTTATTTTCTTTAATAAGGGTACAAATATAGTTTGGAAGGGGTACGTGTGTGCGAGCGTGTTTAGTTTATACTGCGGTTGTGAAAGGATAAAGAAAAAGAAGAGAGATGCGTTACTTGAATGAAGAAGTATATGGGAAGAAATGAATTGAAAGTACTAGTATTATTTGTTGGCGCTAAGGTTTTTATATACATAAAATAAAATAAAATCAAAACATATCCTTTGAAATTTTATTGAAAGATATTTTGAATAAAAAATATTAGGTAATTCATATTTATCTTTATGGATTCATCAAAAGAATATTCGATTTATTTTTTATGAGCAGTATTACTAACTGATTATTTTTTTATTTTAGTTTTACCATCAATACTTAATTTTACACCTTTACACAATTATATATATATGAAAAGTATTTGTTGGGTTACATTGATCTTTGTCGGTTCGTATGTACATATCTGATAAGTGTGTAAGTTTTCTATTTTATTTTATTTGAAGGTTTTTACATAGCTTTAAGGACACTTCAAATTTGACAGTGCAGCCGAACCCCTAACACACACATGCACACAGCACACTCTAAATTAAAGAGGTGAAACGTCCTCACAAGAATCATCAGGTTGCCCCATCTCCACTCTTACCCACTACCCTTTTTTTAACCTCCTTTTTTTCTTATGAATTAAGGGTTTAAATCATTAATTAACCATGCACATTTTCATATATATATATATACTCTTAATTTTTCTTAACTAAGTCAACGTAATTAATCCTTCAAGTACACCAACAATGAGATCATTTTCATTTTCTTACATTATATAATTACCCCCAGAACTAGTGCAGCTAACTATTTATTATTTAGCCCTTGATTAATTAATTGATTCAACCGTATATACCAGGACAAAGAGAAACTTGGCTTCCAAGCTTCTTTGCTATATGTAGAACCCACTTGTGTTCCAAGAAGTTGGCGAAGTAGTAGTGCAAGGCGTTTGGAAGACCAAAAAGCACAGTTTCTCTATGAATCTTAAACAGAGAAGAATAATAATAGAAATATAGAAAAGATGGAAAACTAAGTAGATAGAGCAGACTGGTAGGTAAGATTATACTCCCTCTCCTCTGAAGCTGATCATTCAGGAAAAACGTGTTCCAAAAGGGACAAGCTACTACCTAGCTTTACCTTTTATTTTATTTTATTTTTCCTTTTCTTCTTCTCTCTCTGTTGATTATTTTGTATTTTTATTTTAATTATTA

## >AiNAC44

AATCCCAAACTCACATGATAGTTATTATTGGAGTGATTGGAGAGGAACGAAAGAAGATGGAATAGATTTCAAGAATTAAATAAATTTAAGCTGGTGGAATGGTTAATACATTTATCTGTTAAGTTTTTCTTAAAAATATATTTTTCCGGTTAATGATAAATATTAAAGCACAATATACCCAACCTTAATATATAAAATTAAAGTAGTGAAGAGTGATCCAGTTAGAATATATAGATGGTATAAAAGGTTGGGGATCCAGTCAAAGTGTCAAATGGCACCCGCACAATAATCCAAGGTCTCACCTCAATCCACGACGATGACAACGGCCACTACCACATTAATATACATTTTCTGTTTTAGAATTGAATGAGATACTACTGTTTCTCTTTTCCCACTAAAATAATAATAATAATTTATTAATTTTCCTAGGACTGAAGCTGGACCAACACCAACCACCAGAGGTAATTACAAGATAAGATTCATTTTCGGGGAAGACGATTCCTCTCAATTTTTTTTAATTAATATTGTTAAATGTGGTTTGTTATATAATATTTTCTCATTTTACTTAAAGAATATATAATAAAATATTATACCTACGAATTGAGAACAAAAAGTATTGAAAAGAACCATTTCCTTCAATCCGGTACTTGAATTTGAATCCTCCAATAATTAATATTTAAACATATTTGGTTGTTAAGTTTTATAATTATGGATCAAGTTAATTTTTTTGTCATAATTTAATTAATAGAATACTTATATATACAATTGCCTAGTGACTAGTTAACATTGTACTATAAGTGCATGACTTAATTCAATCCTCAATATAATGAGATTAAATTGAGGGATCAAATTAACTCTCTTAATAAGAAGCTAAATAGAGTTCAACACTTTTAGCGTGATGAACTAACTATTAAACTGATAATGGTAGCTATCTATACACATAAGTGTACTGTTACTGTATAAGTGTTCTGATTTTAATAAATTTTGGTATAGAAGACTAGATGATCTTCACTATATCGTATTGTGATTTAATAGTGAGATGGAATGATGGATAAATATATTATAAGAAAATTATTAACAATATCATTTTTCTTAAATTGTCATTAATGTCATTTCATCTTATAAAATATAAATATAAGTAAATGTTAATTTGATATTTAGTTTTTCACTATTGTATAAAGTGAAATATTCTTTGTGAACTATCTAATAAATAAAAGATGGACTTTGTTAGGTAAATAATAGTTTTTGTGAATAATATGAATAATAGGCTTTAAAATTTGTTCAATAAAATAAAAACATACTAAATCCTTAAATTACTCACTTAAATTTTAATATTAAGATAACTATCCACATACTTAGTAAATTGAATATCTAATATATATCTATTGTTCTCATTGTTTAATATTTTTATTGTATANNNNNNNNNNNNNNNNNNNNNNNNNNNNNNNNNNNNNNNNNNNNNNNNNNNNNNNNNNNNNNNNNNNNNNNNNNNNNNNNNNNNNNNNNNNNNNNNNNNNNNNNNNNNNNNNNNNNNNNNNNNNNNNNNNNNNNNNNNNNNNNNNNNNNNNNNNNNNNNNNNNNNNNNNNNNNNNNNNNNNNNNNNNNNNNNNNNNNNNNNNNNNNNNNNNNNNNNNNNNNNNNNNNNNNNNNNNNNNNNNNNNNNNNNNNNNNNNNNNNNNNNNNNNNNNNNNNNNNNNNNNNNNNNNNNNNNNNNNNNNNNNNNNNNNNNNNNNNNNNNNNNNNNNNNNNNNNNNNNNNNNNNNNNNNNNNNNNNNNNNNNNNNNNNNNNNNNNNNNNNNNNATAATATGTTATTATAACAATAAAATTAATAATTTTTGAAACTAAGAAATATTATAAATATTTGAATTTTCATTTTATAATTATGTAAAATTTTTACGTTATGAATATTAAAATTATTTTGGTACGTACTTTGAACGGAAGAGATTTATGTTAACTGTTAAGCTACTCTACATGAATATTTATAGTTTTCTACTATTCAAAAAGGTGTTGCTGAAATTCCCACTTTATCCTGAATGATGCCCATAATGATTCTACTTTATGGGGCCTTGAATGACACTTGTGACCATCTTATCTCAAGAACGATGATGTCAGTCACCAGCCGCCGCCATTACACAACATTAAGCAGTATAGCACACTATGCCAAATGGCTTTCTTTCTATAATTAATATTAATCGCATATATATTTGATATTTTTATGTAAAGGAATAAAAATTAGAATATAAAATTAATATTATATCGNNNNNNNNNNNNNNNNNNNNNNNNNNNNNNNNNNNNNNNNNNNNNNNNNNNNNNNNNNNNNNNNNNNNNNNNNNNTATATTGTTTTTATTTTAGCAATATTTTAAAATTAAATTTAAAAAAAAAAGTTGAGTATTATCAACACCAAACACAAAATTGATGTGTATATAGTTGTTATTTTGTTATTCATTCTCTCTTTTATACAAATGGAGCCCACTTCATGGAAAATTTTGAAGTTGATC

## >AiNAC45

ATTCAAATTCGATAACAATAAATCTGATAAATTGAATATATATTATTTAAAAAGGTAGTATAGTAATTTTTTTTTTTTTTTAAATTTTGGCCATCAACAAATCATTTCATTTAATAACGAGAGTGAGACAGATTCATTAATTCTCCTCCTTGTTGATTTATTATTTTACCTGCTAATTTATACATTATTCAAGTCTAGTTGGAACATCAAAAGGGCAAACATCTTTTTACCATATTTTACTTTCACCAAATTGGTAAAGATATACGCATGTTTAATTTTACCTTCTTTTGACACATGTAAGTACCATCCCTCTTCGCTATGCTATTTTCAGTTATATATCCTTGTTTTGATTTTGATTTAATCCATATATGCATGCTTTGATGCGTAGATTGATTTAGTAGTATCACATTTATAAATTATAATATACCTTTTGAGAATAAATTATTTTATATGGAAGCATCTATATATGCGGATATCAATTTGTATCTATATTTTTATTTTGATTTACATCAAATTATAAAATATATGCATGCTTTAGTGTTTTACCTTCTTCTTCTTCTGACATATGTACATTCTATTCTCCGTTGGTATGTTATTTTCAAGGATATCATTATTTTCATAGGAATGAGGGATACAGTTGTTAAATTTTATACAATTTTTAATTTGCGGATTATAGGAAAACAGTTGTCAAACTTGATTAACTTTGGAGTAATTAGGAAAGAAAATGTCACATTTCATTTCATATTTTAATTTGTATATAAAAATATTACATGAATATTAAAGATTAGTGTTTAAATTAGTTATAGTGTATTTATATATAAATATACATTGCGTAATTTGTAAATAAATTTTATATTTTAATATATATTTTATAAAAATTATATATACATATTAAAAACAATTTTTTAAATAATAATATTAGTAAAATAATAAAATAATATTTTAATCATCTTTAAATTTATAACATTTATTATTTTAAGTTTTATTATCTTAATTCAAAAGTAATTAATAATTTAATAGTATATTTTTTTAAAATGACAATATAATTTTAGAAGAGTAGAATTTATATCCGAATGTGTCACGCTGTCAGGGAAATGAATCCTCTTTAATTTTTTTTATTGGAGAGAATAAAGTGTGATTTCTCACCTTTAATTCTATAAGTAGGACTAGAAATTAATAAAAAAAGAAAATAATAAATAATAAAATCAAACACTGAACACTATCCAATTTTTTTTCCACAAAAAAAATCTATTCCCGGCTGCCAGCTAAAATGGAAGGCTATTGGAAAATTCACCCCCTCTTTAATTACTGATTCTTCTAATGACTTTTATCCTCTCTATTTTTCTGGGAAACTGTTGCGAACATATTCCAAAACTTAACAGTGTATAAAAAATAAATTTTGTTATCATGGCCTACTAAATCATATACTATAATTATAAATTACAATATTAAAATATAGAAAGATCTAGGTAGGCACTCATGATGATTAATAATGTGGCCTATTTTTTCAATGAGGTTAGGAATTCGAAGCTAAGCAAATGTGAACCTATATATAAAATATAAATAATTAAATATAAATAAATATGAAGCAGTAGCTGATCCAAGCCAGCTACCAACTGAAACAACCACCACCCACCTAAAGCTGCTATAGTGTTTGTATACAAGACCAAATTAGATTAAGCCACCACCACCTCCAGTAGTAGAAGTGGAACTAGCAACATCTTCTATAAAATTGTAGTAGAGAAATTCTCTCGTTAATTAATAATTATTTATTTTTACAAAGAAAGAAAATAATAAGCAGAGTTGTTCACCATGCATTTTGGTTTAATTTTCATCTCCTTGTAATTATTATTCCACTTCTACTACTATTCAACCTTGTTGAACAAAGACTATTTCGTTGGAGAGAGAACTGAGAACTGAGGTATTAGAAGAGAGTACGGTGAAATTCTGAGTTGGACGATCTATTAAAGTTTGTTAATTTTTTTCAATATAAATAATAATAATAATAATAAAAAGAATTGGTGGAGCCTATAGAAATAGAAACGTTGAGGGTCCACAAAAAGCATTGAAAGCACCCCTTTTTACCATTTCTTTTCTCACCTTGGGGGGTGACTGGGTGAGGGTAGATGTAGCGAGGAAACCACCAAAGATAGGACATACGTCATAGAATGAGAAAAATACACGTATTTCTTAAATTAATTAAATGAAAATAAAAATTTGAAGAGAGAGAGAGAAGAAGAAAAACGAAAACAGCAATTGTAGGCTTCTTGAGGAAGTGCCTTGCATACCACGATTCTCATCTCTCTCATCTCTCACAGCGCGTGTAAACGCTCTCTCTATTTAAGCACACCATCCTCCTTTCTTTCCACTTTCTTAAAACCTCTCCAATTCTTCTTCTTCTTCTTCTTCTTCGCTTTACGATCCTAATATAACCACGATTTCTGTATTCCACTATATATATCTCGTTGAATTCAAAATATATTTAGTTTGATTGTGTTAG

## >AiNAC46

TAGAACCACAGCTATCCTTATTGTGTTGTTAATTCCTTTTTATTCCGACTGTCCTCCTTTCTCCATGACAAAGACAAATGCCCATTTTTTTACTTTGCAAATGCATAGCCTTCTATTTTTGCTTAACGGTATTTGTTAAGTGCATTGGGTTTGTGCATTAATTTGAAGTATTAAAGGAATATTCAAGCAACATGTTAGATTGAGGATTGAATAATCGATCTGCACCACTAATATACGCATTTAATAATATTGGACTTTTAATTTAAATAATATTTGTCATTATATATGAGTCTAAAATTAAACTTAAGTCAATAACAATAAATTAATTCTTGACTAGTTAAATTATAAGATAATGTGAAAAAAAAAATTATTTCTTAATATATTGTATCTAGGTTCGAATTTTAATGATGATCAAATAATAAATATAATTTTTAAATATGTATGTAAATATATAATGTATTACCAAGGATTATCAAATTGATTCGACTAAAAAAATTCAAGGTAAAAGTTAGGATTTGAATTCTCTAAAGTTTGAATTTCACCTTAGAAAGTAAAGTGTGATCTCTCACCATTAATTTTATAAGTAGGACCTATTCAAAGATAGAAGATCACACTTTATTCTCAAAAGTAAAATTAAAACTTTAGAGGATCCAAATCCTAAAAATTATAACTCTCTAGTTCCAAAAAAAATGTTATAACCCCCCTGATAAAAAAAGAAAAGAAAAGAAAAGTTATAACTCAAAATCCGAAAGACCTATTCCCCTGCCCTGGCTCACGCTTCGTGATTATTACGTATTTATTTCTTTTTATTTTGATGTGAGGATTACGTATTATTATTTCATTGAACGTCATGATGATGCATTATTTAATTGCTCATATTTCGCTTCGAATAGTTTATAATGAACTCCTCTCTCTATTTCTCATGAGAGATCAATAATGCTTCAAGGTTCGTGGCTCGTGAGTTGCATGAACATGTATACGAAGGATTAATATTTTTTTATTAATATTAATTAATATTATATTTTTTGAATATTATATATAAAAATATATTTATTAACTAATTATTAATTTAAAATAATAATTTTTATTAATCATATAAAATTAGTTATATATATTCTACTTTTTTATTCACATTATCAACAAAATTTTAAACTATCTTTCGTATTTTATTTGTTTCAATTTTATTTCCATTTCATTTTAAATTTGTTTCAGGTATGTCTTTAAAGAGTAATATTACCAATAGAAACACCATTAATATCTACGGTGAAATCACTTTTTTTTTTTAATTTAAACTGATGGAAGACGATACATAAATAGTTATATTTTTAATACATTATTTAATATAATAGTCACTTTTCGATATACATAAATTTTTTGTATTAATTTTTTTATTTATTTTATGCTATTTTTTATTTTCTTAAAAAAATAAAAAGATAAAATTCTATTTTGATCATATCAATTTTGATATCATATCATAAAATAATTTTTTTTAAAAATTTAAATCGATTAAAAAAACACATAAATAATTATATGTCTGATATCAAATTGAAGTATTCATGAAATGTGGTAAGCTGATATATTATTAATTAATAGGTTATTTTAATATTACTAAGTCATATAACATTGACGCTTGGGAATATAATCAAAGCATATTTAAAAGTATATATTGTATATACAATACATATTATTTATTGGTAATTAAGGAACACTTTTAAATATTTAATTACAATAATGTATTAAGATATGTACACTTAATTCGCGCAAAAACATGACTACAGAATCCCATTAATTTGCTTCTCATTCTATCTATAGAACTTCTTTATTTCTTTTGATATAATTTACGTTTAAGTTATATTTTTCAAATTTACTAGACATCGGAGTAAAACAAATGTTTAGTCTTACAAAATTGCACCTTTAGATGAATAAAAAATTATCTACAAAACTTAGGATATAGTAAGTAAATCAGAGAATTACTATACTTGTGGTAGACATTATTATTTTTAGTTATTAGTTAATTATTAATATTTAAAAATATAGAATAAAATATATTATTAAATTATTAAACTAAAAAATTAAATTAATAACAAAAAAAAATTCCTAGTATTTTTGTTATTTAATTTATGTGGTAGCTAGGCTTTGTGATTTGATTTAAGAAGGACTTGTTCCACCTAAAAGATGATCTGGCCCAGCAGAGAAGCAAAAGGATATGAGATTGACGGATTTGGGCCTTTAGTGACTCGTTCAGCGATTCCTTTATGGGGAGAGTCACACTCACATTCACATATGTGCGTCCTAATAAGCATAACTAATCCATAATAATGCATTAATATTGGGGGCAAGCTAAGTAAGACGTTTTCTTGTTCCCACAACCGCGTGGAAGTTTCTACTTTCTAACATACATACATCGTCTCACCTCAGAAGAAAATAATAAATAAATATACCTAATTTCACTCGGTGATTTTTCTATCTATATATGTATCTTTGTCCCTGCTAGCTGTGC

## >AiNAC47

TAGTATATAGTATATANNNNNNNNNNNNNNNNNNNNNNNNNNNNNNNNNNNNNNNNNNNNNNNNNNNNNNNNNNNNNNNNNNNNNNNNNNNNNNNNNNNNNNNNNNNNNNNNNNNNNNNNNNNNNNNNNNNNNNNNNNNNNNNNNNNNNNNNNNNNNNNNNNNNNNNNNNNNNNNNNNNNNNNNNNNNNNNTGACATAAATTAATTTATTTAAATTTTAATTAAAGTTTCATAAAAAAAAGTAAAAATTATTTTATTGATTAAAATTTGTTAAAATTTAAATTGTTCTATTAAAAATTCGGTGAAAACTGATTTAGCATATTACACATTTATAATTTTTTTTTCCAAAACTCAGAGAATGCGAAAGGGGTTGGCGGGTCACAGCTGAAATAAACTCCAAAATACAGCTCATGAGCTTAAGGATGACGTCATAGGCTTACATAGGTAGGAGTCAAAGTAGTTGACCAACAATTAAACGCAGTGTTGAATCCACCTTCCCAAATCCCAACCTCAGAGCCTCAACCTCGTCTTTACTTTGCTGGCTAAACTTTTTGTTAACAAAAATATTTTTAAATTTTGTTATTCAAATATTTTTATATAATTTAAAAGGGTGAAAATTCAGGTGAAGTCGATTTTAAGTGAAATTGATACCTAAGAGCCGTTAGTGAAATCAGTCAAATCATCTAACGGCTTTTAGATATTAACTTCATTTAAAATCGACTTCACCTGAGTTTTCACCGTTTAAAAGTGTACCGAAAGAAACATTTTCATCGTTTTTGTTCTGAGACTTCTTACATGCATTTTATATTTTATTTATCAAATTTAATAAAATATATTTTTTTTTTAAAATAAAAAATANNNNNNNNNNNNNNNNNNNNNNNNNNNNNNNNNNNNNNNNNNNNNNNNNNNNNNNNNNNNNNNNNNNNNNNNNNNNNNNNNNNNNNNNNNNNNNNNNNNNNNNNNNNNNNNNNNNNNNNNNNNNNNNNNNNNNNNNNNNNNNNNNNNNNNNNNNNNNNNNNNNNNNNNNNNNNNNNNNNNNNNNNNNNNNNNNNNNNNNNNNNNNNNNNNNNNNNNNTCTGATTGAATAAAATATTTCATATATATTATAACTGTTTTATTAAAAAAATATCAATTCTTTTATAAACTTAATTTTTGTTGTATACTTGATAATTGTACTAGTTAATAACGTATATTTTTAAGCTGTAAATATTTGATGATTATACTGTAAACATTATAATACACACACTTTTAATTAAACATGGCACTTCTTATTTTTTAATATTACTCACATTTAATCCCAATCACTACTGTATGTCACCCTATATCTGAATTTTACAATCAAATCTATAGCTTTCAGTAAAAAAATTTTAGCTAATTGTTTTTAAAGGAAAAAGTTATTGAATCAACCAAAAAATTTTTTTTCTTCTTCCATGACTAGCTAGTATGCTTTTGAATGAGCTTGACTATAGATATCAAAGGGGGATACCTAGTATAGTATTAAAAAATGATTAAAATCTAACAAAAGTTTAACCGCAAAAGGAGATCATTATAGCCAATGAATTATAGTTCAAATGACATAGTCTTTCTATACTCAATTAAGAGGTTGTGGGTTCGAATCTCCTATTTTTAGTAAAAAAAAAAAAAGAGATCATCATAGTTTAAAACTATAATAAATAATAAGGAATGTTAAGGAGCCAGCAACTTTTGTGATTTGTAGCAATCAAATAGCTATCAATGATGGTTTTAATAGTATGAGATTGGTATAACATTTCATCCAATGACTCATTTTTTTTTCTGGTTACATGTTGGTCAGAATTTAACAAAGTTGCTGGCTTTTAGAATTTTCCATAAATAATTTATTAGTACATTTACATTTTGATATTAAAATTACAGAATTATGAATAATTTTTTGGGCGTTAAAGCATAGGATTATGATGATCCGTTAGAACATTTGATAAAGGATGAGTGTAACTACCATTTTTCATGGTTATGGTGTCTCAAGTCTCATGCATGCACAAGACCAGAAGCACACAACGACGGTCAACCATGTCCCCCCCTCCAAATAGAAAAATACTAAGCCGACACTTTGATCTTTGACATAAAAAAAAATATTACTAGAAAATAAAATGGTAGGGAAAAAACGAGAGAAAGGTTTTGGTGACGCAAGCAATGACAGAATCAAGAAGTGGAACCGCCACACGTGTCGACCATCCAGTAATGCGTCGCCACGTGGCAGTAGCAGTCAGCATGAGAAAAGAGGAGGCTGAGGTGTAGGTATAGCCTACTAATAAACGCTCGAACAATGCCACCTTCACCACGTTTTCACTACTTATCACGCACGTGTCTAAATTCCTCTCTCTTGTTCCTTCACGCGGTTATATAACTCCCCTCTCGTGACTCTCTCATACGTTTCAACTCGAAATTCAGCATCATTCACCACCACCAGCACACACACAGAGATTCGATTCGGTTCGGTTCGGTTCAATTCAATTCATTCAATT

## >AiNAC48

AAGCTCTTGAAAATCTGCTTCCTTCGCTTGTAGTTACTTCTTTCCTTGACATTACTCAAATTCTATGAGAATAAATTAATTCAGGGTTCTCCTGTAGAGTAATTCGGAACAACGAATGATTGTGCTCGGATATGATTTTACGTTGTAAAAATTGGATTTCAATTAAGAAAAATAAAAAAAACGGTCCACGTATACCACTCTATTAAAGCTACATCAACCTTAGTTATTTTTGACAAAAAAAACTAATCAATTATAGATATTTGAAGAGTTATGATTTCTATGATTATTTTTGTTATCAAATCAATCTTTTATGATTATTTTTGGTGGTTTAATCGATTAAATAAGATAACATTTACATAGATTGACACAATGTCTGTTTATCTAGCTAACTCTCAAATAATATACATCGCATTCTTCTATACGTGAATCTCTCCCATTATATTGTTTCCCCTTTTATTTTTTTTTATTTTTTTTTTCTGAGAGGACGGGATAACAAGAATTCCTTCCACCTTAATACACAAACGCAGCAGCGGCTAATGGATCGATCAAAATAAAATAATAAAAATGTAGGGACACTAAACTTCCAATTTAGGCAAAGTTCATTATTTTATGGAATCTTNNNNNNNNNNNNNNNNNNNNNNNNNNNNNNNNNNNNNNNNNNNNNNNNNNNNNNNNNNNNNNNNNNNNNNNNNNNNNNNNNNNNNNNNNNNNNNNNNNNNNNNNNNNNNNNNNNNNNNNNNNNNNNNNNNNNNNNNNNNNNNNNNNNNNNNNNNNNNNNNNNNNNNNNNNNNNNNNNNNNNNNNNNNNNNNNNNNNNNNNNNNNNNNNCTTTTTTAATATAATTTTATCTAAATATCTATAATCTATAACAATATATAATACCAAAACATGGAGGTTTGGTATCCAATTTTTTATTTCCTATTTTAACCCTATTAACATAATTCAAAAATATTATACAGTTACTTATTTCACTAACAGTTACAAAAACTCACGCGTAACTTCTATTCAATTCTTTACTCCGACAATCTCTCTTTTTTTATTTTTTTTATTTTGTTCTGGTTCTCCTTCACATGTGATAATATTATTTTTTTAATTTTTAAATTATTAATTATACAGAGTATTATATTTAAAATTTGAGTACATGAATATTTATTTTTAAATTAAACTAAAATAAATGAATTATATTACAAAAATATAATTTATTTTAATTTCTATAATATCATTTGTTTGGCATATGTCACTGGTTATTTGATTAACGTATAAAAGATGTAACATATAAATACTTAGTGATAATTAATTACGGTAGGTGAAGAGAAGTTGGAAGGAGAAGAAAAAAACTAGAAAGAACAAAATAATATAATGAGTATATACCCATTTTGGTCCTCAAAGAATTTTAAACCAGACATTTTAGTCCCCAACAAAAATTAATTACTCGAGTGGTCCCTAACAATTAATTCCATCAGTCACTTAAGTCCTTGGCTCCGTCAACTCTAACGGAAGACAAAATGGTCCCTAAAAACTCTAACATGGAACAATCCTGACAACTCTAACAAGGGACAAAATGATCCCTAACCCCTTTATTCGAAAACGACACTGTTCTTCCCCAATTTTTATCATATCTCGCATAACCCTAACATTCATACTATAAAAAAATACTTATTTAATAAATTTAAAAAATAAATAATATATTAACAAAAAGATAATATTAATTTCTTAAAAACAATAGAAAAGTGGCTGAACGTGCCTGTTGAGCCACTAATAATATATAATTGTAATTTTGTTTTGTGTTGACATATTATCATTATAAAATACAATATAAACTCCATTTATATTTGGATACATTCTATATATTATCTAATAATAAAAATACAAAAAATATATACATTAAAAATTAACTANNNNNNNNNNNNNNNNNNNNNNNNNNNNNNNNNNNNNNNNNNNNNNNNNNNNNNNNNNNNNNATATATACGTAGCATCACTCTTTATTTTATGTGCATATAATATTTTGTGAGTATATTTAAGTAACAGTATTTTGCTATAATATTATATTATACATATAAGTTTTGTGTTTGCTTGAGATAGTGGCTGCTGGGTGGTGCATATCAGCAAATGGTTGACACAAGGTAGAGTTTGCTTTTGCCATATGGCAACAGATATAGTATGCAGCAGCCATCATTGATGTTGAGAAGAATAGAGCGCTTATTAGCTTCCTTGCTATGCATGAGAAGTCTACGTGAAGGAATGAAGTTATGGTTGGCACAAAAATCATATAAAATATATATATGTAGTTTCAATTATTTTTCTTCTACTTGTTATGATTTTTATCATATACATTTTCATTTATTTTATAGCTTTATGTACACTCCAGGGTGACATTGGACCTAAGTTGATCTTACTCTTTGTGCTTAGACATTAGGTTGCCCCATTCCCACTCAACTCTTCCATCCCTTATTTAATAGTCTCTTTCCAAGTTAAAACTATGGTCTTAATTAATAAGATTTT

## >AiNAC49

ACTATTTCTAACTACATCTTAAATTTTAATAAATCTAATAAATTCTAAATTTTAAATGCTAAACTTTAAATTCTAAATTCTAAATTATCTTTATTTTGTTTCACTTTAAAATATTCTTTTTACTTAAATTTTAAATATTCTTATTATTTTTAATGTTAGAGGTTTCTTTTTAACAATTTTTCCCTATATGTTAGCTAAATTTCAAATTAATTAGCTGAATTAAGTTTGATCCCTAGTCAATAAGTTAATGATTGGAACTAATAACTCAATGATTTGTAGCTTCTAACAGGTTATTACTATCATTCTTTTGGTTTTGATATAAAAAAGTTTCTAGATACAATTCACCATTCACCAACTATATTCAATTCTATAAACAAACATCAGAAGAGATATATACATATAATTGATTGATGATATTGAAGGATCGCAAAAGCTCATAGGGAAGGGGGATAATAAATCAGAAAAAAATACAGAAAGAAAGCGTGCAACTGAATCAAACTATAACAATCTGAAGAGGCCTTAGGTCATACCCACATGTGCATGCCCCAGCTATGGCGACTGCGTCTCCAACTGTTTCTATGGACATTTAAGGCTGACCCAAAATTTCTCAAAAGTTTTATGCGATCGTCAATAATTTTGTATAAAAAAATTATAAAGAAGAATTTTGTTAGTATAAAACTTCGGCCGTCCAATGAAATGCGGAAATCCCAAGAAGCTTTAGTAGCTATTTTTCCACACATTGCTTTAACAACAATCAAATAACCTATATTTCTATTTCTCGGGATCGTTTACTGATTTAATTATTTGCTGAAAACTAGTTAACCTATTTTTGTAGCAAGATTTAGTTGACAGCCTTCGGCCACGCGCGTGGATTATACTATATGCAACAATATATATATAGCATCAACATTATTATTCGGTGTTATTATTCATAGACTATACATATTCTTTTTCACATTTGATATATATATATATGCATACACCATACATTAATACTGGTATGATTTTACCCTCTCTATATATGTATTACACTAAATCTGTCCCTCATTGCTAGCATGAAAAATCGTTATAACGTGAATAATACTGGCCATGGTAATGTTGGTGCTGACCTTTAGATAAAAAATACAATGTCCTTGATTTTGCTTGGTAGGTCCCCGTACTATAGTACTATATTCTCCAGCCATTGTTGGGTTTTATTTGGGGCACGAACCCCTCTTCCTTTGAAGTATCGAAAATTATCCTAAAGCAGAGGGTTTTGACCAAGTTAGCTTTAACCACAAATTAAGCATGTTGCTTCTGTATACCTTAGGTTCGCATTAACGTAATAATTAGTTCGGACAATCACACGGGTCCCTAATAACATACATAAATTCCACCATATTAAGTTTAATTTACTGCTAACATAATATCAAGATAGGGAAACGAGACATCATATATCATACCTTTACGCATAAGCACGGTTCTGATAATGGTGCACACGAACAAATTAAATTTAAGATATTTAAGTTTTATCATTAATTAATGGGTTTGCTGTTAATAATAATATCATCGTCAAATTATTAGTAGGTCATGCAAATGTATTCATTACCTTTAAGCATAAGCACTGAGATTAACTATAGTCATAGCTGCACCATATATATATACCCTCCATTATTTGTACTTTGAATATTTTGATTTCTGCTAATAAATATACCACCTATTTCATCTTTCGCGTTATTAATTATTGTAGATCAAACCAGTCGGTTCAATTAGTTATTAGACCGATTCAGTTTAAGATAAATATTATTTGGCAACAAATTAATTAATTGAACATAAAAAAAATTAATTAAAAAGTCGNNNNNNNNNNNNNNNNNNNNNNNNNNNNNNNNNNNNNNNNNNNNNNNNNNNNNNNNNNNNNNNNNNNNNNNNNNNNNNNNNNNNNNNNNNNNNNNNNNNNNNNNNNNNNNNNNNNNNNNNNNNNNNNNNNNNNNNNNNNNNNNNNNNNNNNNNNNNNNNNNNNNNNNNNNNNNNNNNNNNNNNNNNNNNNNNNNNNNNNNNNNNNNNNNNNNNNNNNNNNNNNNNNNNNNNNNNNNNNNNNNNNNNNNNNNNNNNNNNNNNNNNNNNNNNNNNNNNNNNNNNNNNNNNNNNNNNNNNNNNNNNNNNNNATTTTTGAATCTTTAATTATTATTATGGAAAAAAAAATATACGGATAGGGGAGTGTAGCTTACGCTCCTTATACGCTAGTGGGGGAAGATGTATGATTAAGAAGAGAAAAGAATTAATAAGAATAGAAAAATAAGAATGATAGAGATATAGGTAAGAGATAGTCACATTCATTTGGGCAAACTTGGTTTTTACGATAAGTAGCTTTAACCATTCCCGTCGGCGTAGTCACCGGCCGTCAACCCTTTCTCTATAGTTACATACAGTGGGTGGCGTTGACAACCATCACGGTATATAAGTCCCATGCCTCAATCACCACCTACAAAATTTATTGCTACCCTCCATCATCATCTATCAC

## >AiNAC50

NNNNNNNNNNNNNNNNNNNNNNNNNNNNNNNNNNNNNNNNNNNNNNNNNNNNNNNNNNNNNNNNNNNNNNNNNNNNNNNNNNNNNNNNNNNNNNNNNNNNNNNNNNNNNNNNNNNNNNNNNNNNNNNNNNNNNNNNNNNNNNNNNNNNNNNNNNNNNNNNNNNNNNNNNNNNNNNNNNNNNNNNNNNNNNNNNNNNNNNNNNNNNNNNNNNNNNNNNNNNNNNNNNNNNNNNNNNNNNNNNNNNNNNNNNNNNNNNNNNNNNNNNNNNNNNNNNNNNNNNNNNNNNNNNNNNNNNNNNNNNNNNNNNNNNNNNNNNNNNNNNNNNNNNNNNNNNNNNNNNNNNNNNNNNNNNNNNNNNNNNNNNNNNNNNNNNNTATTGTATAAGTTCTTTGTCTATATCATAAATGAAACAAAACAAACCAATGTTCATAACTAGTTCATAACTCTCTCTGATTAGTATTAATAGACATAGAAAATTTTCAATTGGAGGAAAGGAAGAAGTTTACTTAAAAAAATTATACGTTATACGGTTGGTTTCTACATTTTTAGTGAATTGTAAATTGGTCCTTATAATTTAAAAGTTTGTAATTGGGTCTCTGAAGAAATTTATAATTAAGTTCTTTGTCTATATCATAAATGAAACAAAACAAACCAATGTTCATAACTAGTTCATAACTCTCTCTGATTAGTATTAATAGACATAGAAAATTTTCAATTGGAGGAAAGGAAGAAGTTTACTTAAAAAAAAGTTAAATTATACGGTTGGTTTCTACATTTTTAGTGAATTGTAAATTGGTCCTTATAATTTAAAAGTTTGTAATTGGGTCTCTGAAGAGAATTAAAATTTATAATTAGTCTCTCGCTGTTCAAAAAGTGTTAATTTAACTGAATATTATTAGAATATGCTGAAAATATTCTGTTAAAATAGAAAATATATTGAGAATATTCTGTTAAATCAAATATTTTTTAAACGAAGGGACTAAATTACACATTTTAATTATTTTTAGGGATCAAATTACAAATTTTAAAAGTGTAAGGTACAATTTACAATTTCACTGAAAGTGTAGAGATCAACTGTATAATTTAACTAAAAGAAAACGACCATGTTAATTAATTGACTTTTTAAAGTAATATAAAATAACGATTCAATTAAGATATCAAGTTTGATAAAATAAAATCCGTGCAACTAAATATCCGTTAAAAATTATCCAAAATCAAATCCAAGTATGTTAACTGCACCTCCAATTAAATTGAAATGAATAAGCTAAGATATTCTAGTATTATTCCTAAGTTCTACTTGTTTTTTGCTTAAAGTTTTTGTGTGCATGGAAAGTTACGTAAAAAGTTTGTATTATTGGTCAGTGAAGAGAAATGCATTATTATTATAAAAAAAAAATATTCACTGTTAAGTGGTCATCCACACATGCACTCAAACTAACATAATGCAGTGCTACAAATTAAACATCATAATGGTAAAATAGTATCCATTTTCTAATGCAATAAATTGATAAAGTAGGAGAGATACCAGCAGAATCTGCAATCTAATGTGCGAAAGAACAATTGATAACAAGTGGAAGAGGATGAAAGAGAACGAGAGTGAAAGAAAAGAACATGAATTTTGAATTATTCATCATAAAAGAATCACTTACCTTATCAGTATTTTATGTACAACGATTAAATAGTTAGATAAAATAACAAACCAACTAGAAACTAGGTTAGCTCACTCTAAGAAAAAAAAAGCCTGCTGCTACATTTTCTTTTTATCGCGCTTTAAAAGTGTGGTCAAAAGAGGATCAACGGCCACACTTTTATAAGAGCGAGATTGATTAGAGATTTGGTTATATTTTTCCGTGTCATGTTTTAAAAGTGTGGCCGAAAGAGGTCAATCGCTACGCTTTTATGAGGAGGACGATTAATTAGTGATTTGATCACACTTTTTTTGTCACACTTAAATAACGTGACTATAAAGAAAAACAGGTACACTTTTGAAGCGTGACTAATTTGTTTCCTTACTGTCACATTTTTAAAACGTGCCCATATCCTTTAATTATTTGGCACTCTAAAAAAAGCGTAGCAATAGAGAGTTTCTTAACTCTAGGCATAACAAATTAAACATAACAAATCATTAACAAAATGTACAGGAGCTGAATGAATTTGAATTTTATTCATGTTCATGCTTATCATATTCTATTACAATAATGAGCAACATTTTAAGTCAAAATTGTGTGTATTTATGAGAACTGAAAACAAGAAAGAAGAAGCAAATATTATACTAGATAATTTATGAAAGAGATTTTTTTGTGTGACTCAGTTATAAAAGAGATGATCATCTTTTTCTTTACTATATATTAACTATTAACATGTTATTTTATTGATAAAAATTTGACTAAAATATAATGTATTTGAAATTCTATAAAAAGATAAAAAATAAGTTCCAAGATGATGCTAACAAGACAAATTTAGATGGATTAAGATAAGGAAATAAAAAAAAAATGGGATG

## >AiNAC51

GACATGAAGGATGAGTGATATAGATGGCCTAACTGCTATTTTAAATTACGCTACTTTCAGTTGCTTCTTTTGTCCACAAATATAGCAGTCAGATTCCAACATTGGTGATCCATGAACCAATATACCCTTTTTCTTATTATTTTTCTTCTTTTATATTCAAATCCTAAGAACTATTGTATGTGCAAAAGTACTAATACATATGACTGCGTTTGTTTATAGAACAATACTAAGACATGAATATAAAGATATAAAATTGTATTTGACAGATAAAACATACAGAAATATTATGTTAAAAAACATTAGCATATAACACAATTTTTTTTTTGTTAATTTTTATAATTATATCTTTTATTATTATATTTTTTCTCCATTTTTTTAATGAAAAAAAATGAGAATAAATTATATTTTCATAATTTGTTCTATTTTATTACTAAACAAAATATATGAACACTAAATTTTGTATCTCTATCCTTTTAGGTAGCGTTTGGTTGATGTCCATGTCTATGTCTCAATGAGACATAGACACGGTGACACACGTCTGTTGTTTGATTTGGTGAGACACAAATTTTCAAAGAACACAGGAGGATACTAAATTTGTGTACCTCCAATTGAGTGAGACACTGAAACACAACTTGTGAGACATTAATTTTTTACTCTTTTATCCTTGTTTAATTTTTAATTTTTAAAATTTGTCCTCTTATCTCTCCAAATTTTTTTTAAATGAAAGATAATTCAGTTTTTTTCAAATATTTGTGTGTCTTGTTTATTATTTTTGTCAAACACAATACATAAATATAAATATTTTATGTCTATATCTTTAATGTCTATTTCTTTATGTCTAATAGTCTATGTCTCATCTTATACATCAACCAAACAGAATGACTTATCTTATCCTCTTCTCATAAACAAACGCATCCATATGCTCATATATCATACATTCAATGAATTAACATAGTACTATATATTGGATCATAAGTGGCTGTTTCATTACAACAGAACATAAGCTTTCAGATTTTTGAGACCCCATTACCTAATCAAATAATGCTGTCTAAAAAGGAGATAGAGCTTCACTCACAACTATACAATTACTGTGTGTAACAGCAACTAGTCATAGTAACATTCTTTTTTAACCATAAAAATGAAATTACATATATATAATTTTTTTATGTCGGCCAACAAAAGGAAAAGAAGGATAAAGGAATAAGAACGTGTGAGAGATAAACATGCCTAACTCTCTGACCCACCACTCAATTACCATAAACATCAGCCAAACTTGCCTACTCAACCATTTGTGCCATATCATAATGCTCTTTTAGTATTCTTGATTTCTTTCCACACTCTGCCTAATTGACCTTCATTAATTATACATAATTGGGATTCTTTTGGAGGTCTCAGTCATCTTCAATTGTATAATTACTTGACATCATGGCAACAAAATTGTACACCATGGATTTGATTGCAACTTGTGACAATGTTAGAGAATCAAGGTAACTATGGTGATCAAGAACCATGCTCATAGTCATAGTTTAGGAAACTTGTGGAACAAACATACACAATTAAAAGTACATGACAATATATGTAATTTGATCCGTTCCCAAATATTATACAAATAAAAATACTAAGATATAATAACATAATTGCTGATCTCAAAGCCAGAAGAAAAAATCAATGATAATTGAGCTGCAAAATACCATTTATGGTTAGAAAAGTTATAAGCAAAGTAGTAGATTCCTAGTGTGTAGTTATTCTTTTTTATATAAGATGTTTATGTCTTCTGCAAGATTTAATAATTGATTAATTGTATGAAATAATGTGGGGGCTTGTTTGGCTCCACCAAGGAAACAAGAAAATCAGTTAAACTCTCCTTTGCAAGGAAATGTTTGAGTGCTTTGTTTTTCATGTGGAATGTATGTTCAGTTCCCACTCTGATCTGATCCTGCCATTAATTTCATTGATTACAAAGCAAGGCAAAAAGCATATGGGTAAGGGTAAGGGAAGCAAACAAGAAAAAAGGTTCTTGTATTTAAAATACAAACCTATGATAATTGCTAACCATTATGAAGCATATGTAGGTAGGTCTACACTACTACTTCACTCCATGAGGTTGGTCACCCCAATTCAACTGCTCCAAGGAACCCAAAAATTATACTCATGCTTGCCACGTGTATATCACAGGTTGATGATATCAGCATGAAGTGTCATCATGACCCACACTATAGCAGTACATACATTTATGTTATTATATATCAAACATTTTTGTCTTTTGAATCTAGTTTTTTACACCATACTTATAAACAAAATTTCTCACATTTTTTCTCTAAAAAGCTATATTGTATCTGTTTCTATAATAAATGAATCAATAAAGCTATCAAAAGATTTCTTCTGCTACACACACATTATGATTAAATATTTAGGCCAATCCTCCAGTAAGATTCTGTTACACTTCCGCCGCCTTGTATGCTTGGTGTTCAGTGCTTAAAGTGCCTCAAACTTTATCCAA

## >AiNAC52

TATTTAAGTTTATTTTAAAAAAACTCGTACATGTTCCATCTTAAATTTTGGTAGTATTATTTGAATTATTCTGAGACTTTGTTGCTTTTATTTAGTAATTCATGGTTGATTGAAATGATATGCATCTGTGATGTGATTATATGGGGGTGAAAACTCAGCTGCAGTCGACTTTACATAAAGTTATTTTTGGATGGATGACACGTACTACCATTTAATTTTAAAAAAAATTGGTTTATTTTATACAAATTATTTAATTAAAAAATCAATTAATATAGTTATGATGTTAGAATTTTTACCCGTATTTTATTTTAAAAATAAATTGACTAATTTAAATTGAAAAATTGTAGTTAATTGTTTACTATATAATTTTTTAACTAAAATTATAATTAAAAATCATTAAAAAAATAAAATTATCTAGTATATTGGACCAGTTTAATGTTAATAAGATTTTCATTTATAATTAAGATTAATTTAAATTTTAAAAACTAAAATTTCTCTAACTTCCTACACACTTCATAGTTAGTTATTATTTCCTTCTTTTAAGTTTCTTTTCTCTCTATAACACGGTTTTCTTCTTTAATATTTCAATTTGTATCTGCAGAAAAAAATTTCAAAATCAGTGCATTTAGAAAAAAATTAATCCATTACTTATTGTACCTTTGATAAAATGCAGGATTTGTTTTACGAATGGTCGAATATAATTATATACACAAACTAAAATATTTTCAATTGTAGTTTTTTTTTCAATTGTAACACATCTAAAATTATTAAGTTATACAAAAAATATTTTTAAAATACATCTAAAATCATAAATCTAAAATTTAAATTTAAACATTAAAAAAAATTGCAATACATCTAAAATTATGAAGTTATACGGAAAATATTTTTAAAACACATCTAAAATCATAAATCTAAAATCTAAATTTAAACATTAAAAAATAATTGTAACACATCTAAAATTATGAAGTTATACAGAAAAATATTTTTAAAACACTTCCAAAATACAAAATTGCACGTGCAAAAATAATTTAACATTGCCATATATATGAAAATCTCTTCAGGCCATTTTATTCGATTTTTTATTTCAAAAAAATCAAAATTTATTGAGAATAATAAAAAAAGTAATGGTGTCAGAGACCCGTCCAAACCTTAGGTAAGTTTTCAGCGTGGACGAATGGCGACGTTGTTGCAAACAAACGTTTACGAGGAGGAGGAAGGCGGCGACGAAAATGAGTGTTGATAAAGAAAAATGTAGCGATGTGAATGAGCGTCGAAAAGAAGAAAGGCAGCGTTGAGGATGAGCGCGGGAGGTGGCGCGGATGAACGACGATGGAGGATGACTTCTCGATGTGCACTTCTGAGTTCTTCTTTGAGTGTTTTGTACGAGTTTAGTTAATAATTTAATGGTTTAAGGTTTATTTTATAATTTTAAAATCAAAATTTTAAATTTTGAATAATTTGATTTTTAGATGTGTATTTAAATTATAATATATTAATAATTAAATATATTAAATCTGAAACAGAAATTTAAAATTTAAATTTTAAATTTCAGTTTTTTTAGATTGTATTTTCAATGTGTATTTAATTTTAAAAAGACAATAAATCTATTCATAATTTTTAGATGTGTTTGTTTTAATTTTTTTTTAAATTATTGTAATAAAAAGCTGAATATTGTACTATTTTTAAAGAATACCTAGTTGAATTGATATGTTATATTGGTGTTACATATAAAAGCTGTTAATGGAAATGTGTCAAATATAATTATGCATTCTTTGTTGTGAAATATATGGGATGGAATTTTTGGCATATGATTAGGTATCTTAACTCCCCTGTTAGTCTAAGAAGAGAGTAGTAGACATTCTTTAATTTTTTGGGGGAGGATGAATGAGATTGGAAACACCAGAGATGCTTTTAATATTGCATTTCACATTGTATTTGTGTTTGGGAGGGGGGAGGGGGGAGGTGGGATGTGTGTGAGGGAGAGAAGGTGGCTTGTTTGCATAAGAAAAGCATGACATCAAAAACGTTCCATTCCCAAACTTCTCAACCCCTTTCTTTTTCCCAGCAAAACAGCCATCCTATACTAATTTAATTTAGTTTATTTTTATTCAATTTTTGTAATTGTAACTAAACTAAACTGCTCGATAATAATAAATAATAATAATAATAATTTTTTTTGTCACAACTTACAAAACAATAACAATGGCCAATTTAAAATAATTTTTTATTTGGACGATATTGATGAATTTTGAAATTTTGAGTGAATTTTTTTAATTGAATTTGAATTAATTTTTAACCTCCCTAATAATTCTCCCCGTTATCTACCTCGATGTCACTATCTGAGCGCTACTTTTTTCCCAAAATTCAGTGAAGAAATAAAATGAAATGGAAAAAAACAAAATAGCTATTAAAAAAGTTGTATTGTTGTATGTATATACTAAGGTGTATAAAGACTATATATGCATGAGATATAAATAGACAAGAGAATAATAATAATACT

## >AiNAC53

NNNNNNNNNNNNNNNNNNNNNNNNNNNNNNNNNNNNNNNNNNNNNNNNNNNNNNNNNNNNNNNNNNNNNNNNNNNNNNNNNNNNNNNNNNNNNNNNNNNNNNNNNNNNNNNNNNNNNNNNNNNNNNNNNNNNNNNNNNNNNNNNNNNNNNNNNNNNNNNNNNNNNNNNNNNNNNNNNNNNNNNNNNNNNNNNNNNNNNNNNNNNNNNNNNNNNNNNNNNNNNNNNNNNNNNNNNNNNNNNNNNNNNNNNNNNNNNNNNNNNNNNNNNNNNNNNNNNNNNNNNNNNNNNNNNNNNNNNNNNNNNNNNNNNNNNNNNNNNNNNNNNNNNNNNNNNNNNNNNNNNNNNNNNNNNNNNNNNNNNNNNNNNNNNNNNNNNNNNNNNNNNNNNNNNNNNNNNNNNNNNNNNNNNNNNNNNNNNNNNNNNNNNNNNNNNNNNNNNNNNNNNNNNNNNNNNNNNNNNNNNNNNNNNNNNNNNNNNNNNNNNNNNNNNNNNNNNNNNNNNNNNNNNNNNNNNNNNNNNNNNNNNNNNNNNNNNNNNNNNNNNNNNNNNNNNNNNNNNNNNNNNNNNNNNNNNNNNNNNNNNNNNNNNNNNNNNNNNNNNNNNNNNNNNNNNNNNNNNNNNNNNNNNNNNNNNNNNNNNNNNNNNNNNNNNNNNNNNNNNNNNNNNNNNNNNNNNNNNNNNNNNNNNNNNNNNNNNNNNNNNNNNNNNNNNNNNNNNNNNNNNNNNNNNNNNNNNNNNNNNNNNNNNNNNNNNNNNNNNNNNNNNNNNNNNNNNNNNNNNNNNNNNNNNNNNNNNNNNNNNNNNNNNNNNNNNNNNNNNNNNNNNNNNNNNNNNNNNNNNNNNNNNNNNNNNNNNNNNNNNNNNNNNNNNNNNNNNNNNNNNNNNNNNNNNNNNNNNNNNNNNNNNNNNNNNNNNNNNNNNNNNNNNNNNNNNNNNNNNNNNNNNNNNNNNNNNNNNNNNNNNNNNNNNNNNNNNNNNNNNNNNNNNNNNNNNNNNNNNNNNNNNNNNNNNNNNNNNNNNNNNNNNNNNNNNNNNNNNNNNNNNNNNNNNNNNNNNNNNNNNNNNNNNNNNNNNNNNNNNNNNNNNNNNNNNNNNNNNNNNNNNNNNNNNNNNNNNNNNNNNNNNNNNNNNNNNNNNNNNNNNNNNNNNNNNNNNNNNNNNNNNNNNNNNNNNNNNNNNNNNNNNNNNNNNNNNNNNNNNNNNNNNNNNNNNNNNNNNNNNNNNNNNNNNNNNNNNNNNNNNNNNNNNNNNNNNNNNNNNNNNNNNNNNNNNNNNNNNNNNNNNNNNNNNNNNNNNNNNNNNNNNNNNNNNNNNNNNNNNNNNNNNNNNNNNNNNNNNNNNNNNNNNNNNNNNNNNNNNNNNNNNNNNNNNNNNNNNNNNNNNNNNNNNNNNNNNNNNNNNNNNNNNNNNNNNNNNNNNNNNNNNNNNNNNNNNNNNNNNNNNNNNNNNNNNNNNNNNNNNNNNNNNNNNNNNNNNNNNNNNNNNNNNNNNNNNNNNNNNNNNNNNNNNNNNNNNNNNNNNNNNNNNNNNNNNNNNNNNNNNNNNNNNNNNNNNNNNNNNNNNNNNNNNNNNNNNNNNNNNNNNNNNNNNNNNNNNNNNNNNNNNNNNNNNNNNNNNNNNNNNNNNNNNNNNNNNNNNNNNNNNNNNNNNNNNNNNNNNNNNNNNNNNNNNNNNNNNNNNNNNNNNNNNNNNNNNNNNNNNNNNNNNNNNNNNNNNNNNNNNNNNNNNNNNNNNNNNNNNNNNNNNNNNNNNNNNNNNNNNNNNNNNNNNNNNNNNNNNNNNNNNNNNNNNNNNNNNNNNNNNNNNNNNNNNNNNNNNNNNNNNNNNNNNNNNNNNNNNNNNNNNNNNNNNNNNNNNNNNNNNNNNNNNNNNNNNNNNNNNNNNNNNNNNNNNNNNNNNNNNNNNNNNNNNNNNNNNNNNNNNNNNNNNNNNNNNNNNNNNNNNNNNNNNNNNNNNNNNNNNNNNNNNNNNNNNNNNNNNNNNNNNNNNNNNNNNNNNNNNNNNNNNNNNNNNNNNNNNNNNNNNNNNNNNNNNNNNNNNNNNNNNNNNNNNNNNNNNNNNNNNNNNNNNNNNNNNNNNNNNNNNNNNNNGTCTAACATTCTAAAAATATGATAAAACAATAATAAAATAAATAAATGTTTTTATATATATTATATATTTCTGTGCCATTCTCGGTTGTCAGGCCTTGTTAGAGAAGCTAGCATATTGCACTGATCCACACACACATTACACTCTCTTTTTCTTTTCTAAAAATATGATAAAACAATAATAAAATAAATAAATGTTTTTATAATTTTTATATATTTCTGTGCCATTCTCGGTTGTCAGGCCTTGTTAGAGAAGCTAGCATATTGCACTGATCCACACACACATTACACATCTCCCTCTCTTTTTCTCTCCTAAAAGTTAAAAGCCACATACATACATAGATACATAGATAAGGTCCGTGCCCTTGAAGCTAAGAAACTGTGCAAACTAAGCCTCCTCTTTGCTTGTTTGT

## >AiNAC54

CAGAAACTTATGGCATGAAACTTGGGAGTGAGATGAGATTTCTTACCAGGTTTGCTTAGATAGAAACGTAGAGCTCGTCGAGAGCTTCACGTGGCCACAAACGGCTCGTCAATCAGAGCTCCGTAGCTCAAGTTATGGCTTCCGGAAGTGGACGATGAATAGTGCTCCCATGGCTTCTCTTCTCTCTTCTCTCATAACCGAACTCTCTCTTTCTCTCTCTCTCTCTAATGAAAATGAGCTGAAAGCTCATTAAAGGAGACTTATATAAGTTGGGCCCAACTTGGGCCCGGTTCAATCCGTTAGCATTTTTAGCTCGTTCGACTCAATTTTGGGCCAAACCTTTAACACCAACTCCCAGTTTTTCATTCCTAATATTTTTCTAAGGTTTTGGACTGTTTTCACTTTTTTTCGCACAGTACCGAGCAGACTTGAATCGGTTCAACCGGTTTAACTGTCGATTTACGATTTTTCACGGTTTTTTGTAGAAAACACATTTTTTGACTCAGAAAGACCTATTGAGTCCAAAAATCATATTTAAATCCCTAAATTCTCATTCCAACTTTTCGGAACTTAATTTGGGCATTTAAATTATTTTATTCGTGAAAAACCCGATTCTTACATCCTCTCCTTAAAAATATTTTCGCCCTCGAAAATCTAAATCACACGATTTAGTATATATACATATTCTCCACGAAGCATCCTACTTCAACGTTGTCAAACTAACAAGTTGTAAAAGCATCTCGCTCACCATCATCCCACCGTGTCGGCGTTCCCTCTCGCCTTCCGCTGCGTCACTCTGATTATCGTCATCAAGAATCCGAAAGTTTTCTTAAAACAAATACCGATTTTTGGTAATATGTTTCTAAAACCTTCAAAACAAGTTTATTCTAAATGAGTTCATTGTTAAAACTATTTCAAGGAGTCAAAAATCATTTACTAGTAAGTCAAACACTTGAAATCACAGTTTTCTTAAATAAAACTTTTCAGTTCGAATTCCTTTCGATAAGATAATTTGGAAACCAAATTCACAAATGAACATAATCAGAGAACTCACTTTTCTTTTTAAACAATATCTTCCAAACCAAGAATTTCCGACGTAGTTTCGAAACCAAAGCCTGCTTTTGTAACATTTCTCAAAGTTGTGAAAACAAGTTCAATCTAAACCAGTTCATTATGAAAGCTTTTTCAAAAGGCTCAAAAATTCATTTATTCTTAAATCGAAACCTTTCAATTTGAATCCCTTTTTGATAAGATAAATTTGCAAACCAAAATCTCAAGTTATTAAAATCACACAACCCACCTTCCTTTTTAAATCGTCTCATTTGATAAAAAATTCAAATCCTAATTTCAAAACCAAATTAGTTAAACCAAAGCTCCAAACCAGATTTAAAAATCACTCTAGATCTTAAAACAGCAAAAATTATAGTTAAAAACTGCAAGGACGTTAGTCGGTATTTCCGTTGCCACTAGTGCTGAACCACACCCATCACCCACACAGGGTACCGACAAGACTTATAGTCATGCCTGGTAACCATTTCGGTACGTCCACTCGGATTGTTCGTCATCACAAGTCCAAAATCCTCGACTACTACCACTAGAATCCTTCTTTTCCCATGAGGGAATCGCCAAGACTTCTAGTCATACCTGGTAACCGTTCTGGCACGCCAACTCGGATCGTTCGTCATCACAAGTTCGAAATCCTCGGTTGCCGCCGCTAGAATCCTCCTTTCCCCATGGTTTACTTCCAACAAAACTCTAAACTCTTGAAATTCTAGCGACAACTTTACAAAGATAGAACCAAACTAACCTCAGGTCTAGGCAATATAAGACTCAAAGCATACGCTTAACTCTCGTCGGAGGATCCGACCTTGTCGCCTCACGTGCTTCCAAAAAGTAAGCACACGGCCTGGTTGCTGACTCTTACCCACACCTCAGTTCCTCCCTCTACCGCAGTCCTTAGCAAGGTGCCCTGGTAATCCGCAAGTGTAGCATCGACCATTTCCCTTTGCCATGTGGAACTGAGCATTATTGTTCTTTCTGAAATCCCCTTAACCTCGCAGATGTTGAGGAGTGTGTTCATTTCTTTTAAAATTCTGTCTCTTTGGTCTAAGATGATCGTCACGCTCTCTACTAGTATTTTCTCCAGAAGTCTCATTTGACGAGGCTATCATTTTTGCGTATTCTTCAACCACTCTTGCCTTGTTCATCAAATCAAAAAAAATACGAATGTCATTTTTTTTATATATACACTCATCAATATATAAAAGAGTATATATATATATTATTTTAATTATATTAAAATAATAATATTAGTGAATATTAATATTAAAATTTTTTATTTATTTTACAACAAAATTTACATATTTAAAATTTAGAGGATCAAAATTATTAAAATTTATGTTAACCAAGTATTTTTTATTATCTGATTTTTATGTTTTATTGTTTGTTTTGCCATATTTATCCGTCTTGAATATAACAGCTTGATTATTTAGGAAGTGTT

## >AiNAC55

CCCATAATTATTTTGTATTCCCAAAGCCTAGAGGAAATTGCCTTTCACGTGTGCTATTACCACAACGCCCCTGTCAAATAACAAGTTCCTAAAGACATTTTTTAAAGTTCGTCATGTCAAATAATTTGAGGCTCTTTTGTAATCTATGATTTTCCTTATGAATTTCACTCTGAGTTAAAAAATGAACATTACTCATAGTATTTAGAATAATCATTCGAGTACTAGAGCATTTTATATCATAAAATCACTCATCCCAAAAATTTAAGCTGATTTTAGGATTCCTTTTAGATCTAAAGTTCTAATAACATGTCATGAAACTACTCACTCCAAAAGTTTAAGTTGATAGAAAAATATAACACTAATAGTTATATCTCTAATACTAAATTGGACATCTCTAAACTTTTATTGTACACATTGTACAGATATTCCATTGACTCCCTATACTTCCTCTTTTTTTATTTGTTGGAATCTAAGTTGCGTTTCTTTTTTAGAACAGAATAAAATAAGATATTAAAAATACAACATAAAAAATAGAAATATAAAATTTAATATTTTTATATTCTATTTGGTGATAAATTAGAATAAATTATAAAGATCCAATTTATTCTCTTTTTTTTCACTCAAAAAATTTGAGACAAAAAATATAATAATAAAAAATATAATTATGAAAAATTAACAAGAATAATGAAATAAAATAAAATAAATTATAAAAATTTAATTTATTTTTATTTTTTTCATTTAAAAAATTTGCAAAAAAAATATAATTATAAAAAATTAACAAAAATAATAAAATAAAAAATAAAAAAATTGTGTTATTTATTAGTGTTTTTTATTCTTTTTATTAAGATAAACACAAAATACACTAATTTAATATCTTTAAAGACATGCACTATCTCTACCTACCTATGTTCAATCTATTAAACACAATTTTATGCGTCAAGATCCTGTCTCAATATCCTGTTCTTATAAACAACCTAACCCTAAAGATTTTTTTGGTCAAAGTTCATCTTTCTAATATGTTGCTTAAAACCTGATGATCTTATCCATATACAATTATACATACACCTTGGAATGGACCCGTGATAATCCTATTGGATCATGTCTCAAACTATTTAATTTCTAACTGCTACATATTATTAGTCTAGTTCAAAGCCTATGATTTATGAAAAGTGAAAACACAAGTTAAAATTCAAACAAGATTACCTAAACCATAACTGCTATGTAGAAAGTCACTAGCATTATTAAAAATAAAAGATTACACCAACAAGTACATCACCTAAACGGCGAAACCCTAAACTAGTGTTCGAATTTTGATGATAAAGAGAAGAAAAAAATGGTGGAGAATTTTCCTTTGGCCATAGAAGAATGACAAAATGATTATAAAGTGTATAAAAATTTATGACTAGAAGAAGCTAAGTTATAAGTCTGAGTATGAAAGCGAAGAATTAAGTAGCCTTCTGGCCATTGAAAAGATAGATTGAGTGAAAATTAAGAAGGTCAAATAATAACCCCGAAGTCAGAAGCATCATATATTGCAGTAGTCATAAATTTCAGCTAACTCTGAAATTGATAGAGATACATTGGTGGCAATAAACAAATGTTGATTTAGATGAATATAAAAATGAAAATGTTGGTAAAATATATATATAGTAAAAGCGGTCCCCTACTCAAGCACATTAGATTTTGATCACAAGTCCGATTCTATCTCTTGTTGTTATCTAAAATTTGACATTAAACAAAAACATGTAATGCTTATTATTAAACTGTTTCGACCTGACAAACCCAGTGGAATAGAAGCTCTGGGGAATGGAATAGAAACGGGATTTTCGGATTCTGAGAAGCCTTAAAACCAATAATATAATCCAAAGTTAGTCTAACTGGAATTCGATGTTGAATTATATATTGGAGCTTTGTTTTGGAATCAGGTTCAGGTCCACGTTTCCAAATGATATTTCAAAGTTTCAGACCCTCATACATAATTTTATTATTTTTCCTTGGAACTTTGCTTTGTCTTGGCCTCTAGCGGATAGGCTCAAAACTTTCATGTCATTACAATATGTTACTTACCAAAAATGTTATTAATTATGCATATTAATCTTAGAATTAGTATCTACTATACGCTTTTTCTAATTGTAAAGTACGGAAAAAAACTTAATCCATACCTATATACAATGAAACTTGTCAAATTTAATAATAATAAATAATTAACCATATTAAGTATACACTAAATATAAACAATAAAATTTATTTGTGTCTAGCTAATATATAATAATTAATTTTTTTTATGTATATATAGTATTTTTTATAAACAATAAAGTTTATGTGTGTCTAAATAAACACCAATGTTTTTAGTGTAAAAACAAGATTGTTAAATTAATTAAATATGGTAAGATCGCAATCCCCAGATGCTGCTAGTTTGGTGGTGTATGGAGATTCCAGCTTTTGGGTCATAGACAAATAACACACAGCTCTTTAATTAAGTTGAAAGTGTGAATGAATA

## >AiNAC56

ACCCTTGCCCAGTGACGACGAAGTGGTTGAAACTCTAACAATGGCGGCGCAGCAGAACATAGCGTGATGGGAGAGGAGCCAGAGCCCAAGCCCCGCAACGGCGGAGAGAAAGCCCATGGATGCATAGAGAAGATGAGGGGAGCAGCAGAGGGTTGGCGTAGCAGCGACGAAGCGGCTGAACCCCGGCGACGACGGCGATGGAGAACACCAGCGACGGCGGCGCGACAAGCCTCAACAGAAGAGAGAGAGAGACGAAGAACGACAAGCCCATCGCGACAAGTCCTACAGCCACAACGCGACGGCAGCGGCTACCCTCTTCCCCTGCCATCACCTCCCTCTTCTTCCTTTCCTTTTCTTTCTTCTCCCTCTGTTGTTCCTTTTCTTCATTTTGGTTTTTCTGTTTTAGGTGGAAAGGAGTAATGGGGGGTAGGGGTAATGGGTGGGGTACTGTAGGTACTGGGTAGTGATAATAGGAAAGGGTTTTTATGTCATTTCAAAAATTATTATGGACAAAAGGATGATTTTATAACGTTTTGTAATGTTGATGATGATTTTAATAACAAAAAAGGTCGGGGACGATTTTGATTTTCACCCAAGAGGTTAAAGACGAAAAAAGTACTTAACCCCTTACCCATATTTCACCCATTTCAAGTCTTAGAGCATTTTCACTTTTCTAAAAAAAGTTATGCCTCTCGAAATATGATTTTATTTTAAGGAAAGGGTGTTGTCACAACAGTGAACAGTCCTGCTTCCAAAAGGCACACCTTTTTCTAAAAAACTCGAATTGCCCTTAAATTTGGACATAAAATACTATATATCCTAATATTTCACCCCTCTTTAGTTGCACCTCCAAAGATTTTCAGATATGTGGTTTTGAAGTTGTTATTTCAGAATTCGGACAACATTATTTCTGCAGAAAATGACCATTTCCTAAAATTCATATCTCCCAAACCACACATCGAAAAATTCTAAAATTTTAGGGAAACAATCTAGACATCTCAAGGTTTCATAGAAAAATAATTTCACTTATTTTGAGTAGCTAAATTTCTCCCAGTTTTATTCACAAAATGCTATCTGAAACTGCATAGTTCTGCAAGACTGCAACATGTAACCTCGAGTTTTGAGAGGTCAAAAATTAATTCCTAGCTTTTCACTCACTTTAACCTTGCATTCTAACTTTCTTTTACCTATTATGACTTATTAAAAAGATTAAATCAGTCATAAATACTCAGAATTAATAAATCACTATTTTTTGTCACTTTCACATTTAAGTATACTCATATAAAATAAAAATTTTTTGCATTACTCAACATAGCAATTCAGACACAAATTACTCAAACAATCCACATATATTATCATCAATTTATTATTACATCATATATAACAAATAGTCCAGCATCATCTCAAGTAATCATTAGTATGTATCATCAATTAATTCAATCATAAATTAATTCAATCATGGAACAACCACTGCAGCATTGTACACAACTGTTATCAATTTGCCATATTTCACATCATTGGGGGAATTCATTTTTTGATCCAATAGCAGTTGCAGCATGAATTGAATCTAACATAGACTTCTCTCACACACGTCAAAAACAATGAACCAACTCAAAGGCAACGGAAAAGTAGCATTCCAAAAAACAAAAACGTGCTTGCATTATTGCTTCCTACGACAATCCATCTAAGCCAACTCAAATTAAAAAAGAATCCACAATTACAAAAATCAGAAAATGGATAGAAAACCTCTACAACTTCGATTGAAGAAGTAAAATATCGATTCAAAAGAATTACTTAATTTGGAAGAAACGACGAAAATGACGAAAAAACTCACCTTCATCTTGAGAAGAAAAAGATGTAGTTGTAATAGACCTGTGACGACGATAATGATGCCACAGTGGCGTTGTGAACAACGACGATGAGACTTTGATGGTGCTTGTGACAGTAAAAATAAAGAGAAGATAGGAGATGAATTTTCAGATTTAAAAGAAAATAAAATAAAAAATTTTGAACAAATATATAATTGTATCATTATTTGTTTTTTTTTTTTTATCTATTTTCATTTCATTGGAAAAAAAATTAGTCTTTTTGTAACACGTAGCGAGTTACTAACATTTACATGCAATCTGATTTTTTAATTAAAAAAAATGAAAATTAACGGCGTGTTCGTTAAGGCAGAAAAAAAAAGGATAAGTAAACTCGGAAATTGTTTTACTGGTTTAATTTATTTTGCTAGAAAACATGAAAAGAAAATAAATGAAATGGGAACCGCAAAAGAATTTTCATGCTAGAGCTAGAGTCGGTGAGAAAACCGACCAAAATTTTGAGGTGTGAAATCATATTATTAACTTTTATCAGTTTATGTATTATTATGTAATAAGNNNNNNNNNNNNNNNNNNNNNNNNNNNNNNNNNNCTTCTTTTTTTTTTTTTTCATTTTGCAAGTTAGTTCTTGTTTTTGCTTCCATGCCATTAAGCTTGTTGTAATAGATTTCTCAACA

## >AiNAC57

AAAATTATGATTTAAAAAAAAATAGATTCTAAAATATTCAAATGGCAAGATATCTGTCTAAATATATGTTTTATTTGACCAATTGAATCAAACATATATAAATATATAAATAAATTGACTAAATATTATTTTAGTTTGTCAAAAAAATATATCTTTAAAGAGTTATTTTGTTATTTATATCGTTTAAGATTTATTTTGTCAAAATTATAATTTTTGAGGTCAAAATGATTATTTATTTATAATTAATCTATAATCATATAAATACCGTAAGTGCTAATGAACTTAAAAAAAAGCGTGAGTGTCTTTTAGAAGATGACCTACTATTAGAGGCAACAAAGGAAAATTTTAGACTTTTAATTTTGGATAAAATATATTTTTTGTTTCTAAAAAATGCTAAAATTTTAAAAAATATTTTAAATATAAGTTTTATTTTGTTTTAATTTTGTCTCGAAACTTTTCGATTTGTATCAAAATATCAAATATATATCTGACAACTAAATTTAAAAAAATTAAGACTAATCTAACAATAATAACGAATGGTAAATAATATGTTTGATTTACTTACATTAAGTTGTTATCTTTTGTGGAGTTATTCTTAAATTGGTCATAATTTTTTTGAAAAATTAGTCGTTAGGAGTAAATTTAATGTAAATAAAAAATTTTTAGGACAAAATTGAAATAAAATAAAATTTAAGAGTATTTTTAAAATTTTTAACAAACTTTAAAAACAAAAAATATACTTTACCCTTTAACTTTTATTATCTTACAAAAAAATAGTTAATTTGANNNNNNNNNNNNNNNNNNNNNNNNNNNNNNNNNNNNNNNNNNNNNNNNNNNNNNNNNNNNNNNNNNNNNNNNNNNNNNNNNNNNNNNNNNNNNNNNNNNNNNNNNNNNNNNNNNNNNNNNNNNNNNNNNNNNNNNNNNNNNNNNNNNNNNNNNNNNNNNNNNNNNNNNNNNNNNNNNNNNNNNNNNNNNNNNNNNNNNNNNNNNNNNNNNNNNNNNNNNNNNNNNNNNNNNNNNNNNNNNNNNNNNNNNNNNNNNNNNNNNNNNNNNNNNNNNNNNNNNNNNNNNNNNNNNNNNNNNNNNNNNNNNNNNNNNNNNNNNNNNNNNNNNNNNNNNNNNNNNNNNNNNNNNNNNNNNNNNNNNNNNNNNNNNNNNNNNNNNNNNNNNNNNNNNNNNNNNNNNNNNNNNNNNNNNNNNNNNNNNNNNNNNNNNNNNNNNNNNNNNNNNNNNNNNNNNNNNNNNNNNNNNNNATTAATAGCATGTTGATTATGATAATGAATCATGATCCATGCCATGACTTGAAATAATGATGTATTATTGTTGTTACCATCCCTTGATGGTAGTAGTGGGTCCCACGTGGTAATTTTCATGTTTACCCTCATGTTAATTAATGGTGCTAATTGCGCAACTAAAAGCAAGAAGAGAGACTGAAAATCTTATCTCCACTCTCACGCCCCACATACACTTATCATTCCAATGAATATACCTTCTTTTCAATTTCTTCTATCTTTTCTCTTTTTACAATAATATCATTATCAGTGAAAAATATAAAAGGAAAAATAAAATAGCACTGTCCTTTGTGGCCCCCACCCACGTGTCAACACTACATGATTTGGAATCCCACGTGTCATGCTCTCAACGGCCAGAATACCAGTTCACGTCATCATCAGGTACCCACCTCATCGCATGCACCCGCATCCACCTCATCCCTCTTTAGTGTTCTGTGTTCACACACCACATACACAAGTCAACACCCAAGAAACAAAATTTAGGTAATTATGGTCATCTATCATACTAAACAATTTTCAAACCACTTAAATTATATTATGTAATAAATATATTTATTAATGTAAACATATTTTANNNNNNNNNNNNNNNNNNNNNNNNNNNNNNNNNNNNNNNNNNNNNNNNNNNNNNNNNNNNNNNNNNNNNNNNNNNNNNNNNNNNNNNNNNNNNNNNNNNNNNNAAAAATATAATTTTTATTATTAAATATAATTTTATACTATTAAAAATATTAATAATAATTAATTAATGACCATAAATTATAAAATATACGAATTCTCTAACACTATTATTTCACATTATACATACAATAATCTATTAATTAACATAATTCTAAGGTAAAACAAATATAAATGCTAATATTTTTTAAAAACTATTTTACTATAACTTGGAAAACTTTGCTCTTTACGAAAAGACATATCAGTCCTTGAACATCAGAGACACGTCATGAGAGCACAGGATGGAGAGATAGAGATTACCTCAGTGGATCAAACCCGATGGTGCCCATGCCACATATAAAACGCAAGCTTCAGCACTTATTGAGGGGTAATGCAAATTACAAGAACAAAACAACACATGACGTTCCGTCTTAACGTGATCTTCGTCTGAATCCACCGTAGCCAGAACCAACCAACCAACTAACCAAATCCATAAAAAAAGAGGGAGAATTATTGAAAGGAAAAGGAAGAAGAGAAGGAGAAGAAGAAGAAGAAGA

## >AiNAC58

TTCCACTCACCAGTCAGCACACAAAACCACAAATCCTAATCTCTCTTTCCAATGTCCCACGCTGCCGCGGTTTCGTCCTCGCGCCGCTAGCGTCACCGTAGCACCGTCTTCACGCGCCATCGCCGGTCATCCTTACATCCTTCTCTGCCACCTCATCTGCCGTTCGCGCAGGAAAGACAATCACCATCGCGCATCTACTTCACCCTCCTCGCGTCCCATCGCGACTCCCCATCATTCGCCCGACGCATCCTTGCTACTCGCGACTCCCCATCACTCGCCACTTGCACCCGACGGCTCTCCATCCGTTGCGACGTAGCCACCACCAGGTCCCATTCGTGACGCGCGATCAACTACTCCGATCCATGGCGACTTCTTTACTCGTCGCAACACGCCACCGCGAGTCCCCCACTGTCCATGCAACGTCGTCCAAGACGTCATCGTCGGCCACCCTGCGACTCTTCTTCTCTTCCTCCTATTTAGTTTTGAATGATTTTTTTAACTAGTTTTTTATGTTTTTTTTCTACCTTTCGAATGATTCTTTTTATTAGTTTTGGATGATTCTTTTTCCTATGTTTCGTATGGTTTTTTCTTAGGATTTGGATGATTTTTTATCTTATGTTTTGGTGCTTTTTGTTTTTTAGAGTTTCGAAAAAAAATTTTGAAAGAGGAATTAATATTTGCATAATAAATGATAAAAAGTGAATTAAAGTAAGAAGAGGCAATTAATAGTCATTAATTTTGTATCTTTTAAAAAAAATTAAATAACCACTAATTAATAACAATTAATGTGATTAATTAGTATAGAACATAATTCAAAAGGAAAAGTATAGGTAACCAACAAGATTTTTGAACAATGTATAAACAATGTGAATTAATAGGGTTAAAAAAATAAATTTAATTAGTAGTATTAAATTAGAGTGTAGTGTATTTTCATTTGATTGGTGATTATTCATGTTGTTCAAAATTTTCATTGTTCCCCTAACACTCCCCAATTCAAAAATATTTGTTGGCTTATTGTTGGCTGGACCTCTCTTGGTTTCTAGCATTATTGTATTGTAAATTGCAATTTTTCAAGACTCTAGTTGGGACATTTATTGAAGAGTGATCTAACCTATTGTGCCAAACCATTTTCAACTGTTCCATGAAGCAGCACCTCTTTTGCCTCCTGTGATTTCCCACAGTAATGATCATCATGTAGTTTAAAGAAAATATTGATGTCCTTGACAAATTTGTTTATTTACACTTAATTAAACAATTTTGAGCTCAAATCATATTTAAATAAAGAGTTTCCAACATGTGATTTATTTGAAAACTTGATCCATCTCCAATTATGACCTGATTACATCCATCATAAAGCGATTTCTGCATGTCATATGATGTGTTGCTCCAGAATGGGGATACCAATTCGGGCCACTCACTGTTACTGGTGTAGCTACTACACTTCTTTTTGCCATATTTAATTTGCTCTTGGACCTCAACTATTCTCACCTTGTGAACTGGAATAGTTCCTTGAATCATAACTACTATCCCCTTCCAATATTATTATATTTTTTAGAACCAAACAATACTCTATATATTAAAATAATATTATAAACAATTCAAGAACAATATAATCAAATCAAGCAAAGCACGCGACAGTGTAAAGATACAATAATTAATTATTTAAATGATTAATTAAGCTAGCAAGCCTAGCTGCTAGTATTCAATCACAAGTCTATAATTATATTATATGTAAACTTATTATTATCTTCTTCTTGAAATTAAAGTGTGTTCGATCTATGCTTGTAGTTGTATAGTTGTATAGCACTGAATGGCTGATCAAAGATACAATAATTAATTAATTAATTAATTAACAGAACTCTCTCATCTCTAAAGACCTCTCATTCTCAAGGCCAGTGGCTAGCTTGGCTTTCTTTTTTGTCCCAAATTGTTCCTACAAAAGAAAAGAAAAAGGGTCATAATGCATTAGTAAGAAAAGCAAATGGTTATTATTATTTGGAGGATTTAATTGGTATTAAAAAATCAGAACAATCTAATAAGGGTGTACAGAATTAATCATAGAAGAATGATAATTAGATTGGTCGAGTGGACAGCTAACTCGTCTGTTTAAGTAAGTGTCGAAAATTTAAATTACACTTTATATATATAATAATTCATTTGCCAACGACACATCATTAAATAGAGTTCAAATCTGCTGCAACAAATTAACCCTTAACTTTTTGGATTGAAAATATAGTAGAAAACAAAAATTATAGAAAAATGGTATTAATTATTATATTATTGTGAGTTATAGGGGAAAGGCTTGTATCAAACGTTATGCCATAGAATCTGTAAATCTGTAAAAGAAAAAGAAAGATTTAAAAAAACTCAAAGATAAGGAACTTTGGTTCCTTTATTTGCTCTATTTATTGGCAATGATGGGGTGTGATGAATGAATACACTTTTGCAGAATACTAAGCTCTCTAACAATCAAATCAATACAAGAGAGAAAAATAATATA

## >AiNAC59

CAGCTGGAGCATGATGCAGCTGCTAAAGTTACAGGCATGCTATTGGAGATGGATCAGCCAGAGGTATTGCACCTGATTGAATCACCAGATGCTCTCAAGGCGAAAGTTGCCGAAGCAATGGATGTGTTGAGAAATGTTTCTCAACAAGCCAACGCCCCTGCTGATCAACTAGCCTCACTCTCCTTGAATGAGAATCTTGTCTCTTAGATTTTTTTTAATTCAGTTTACTTTAGGCAATAAACCCTACACTGGTCATCACTATCTGCTTTTGTTCCAGTTTTAAGGTCTTAGATTGGTGATTTCGGTCAGTACTAAGGAATCTTTTCATGTTCTTTGCAGTTTTTTGTCTCTGGCTTGTTTCTAGGATTAGCAAACTTGCTGGATTATTTAGTTTTTGGATTTATTTATGGGTTGTCTTGTTTGTGGATTTATATTTATATCAGTTGTACTGGATTGTTTGAAGGTGTTGTGTTAATTTTTGTTTTCGATTAATTGCTCCGCTCTATTTCAGTGTTAACTGTGAATTTTGACAGTTCAAGGCACGAGATAGATACATTGTAGGCGTCTTTCATTGAAAGAAAGAATTTCGTTTCCGATAAAACTTTTTTTGGGCTCATGACCTGAGTGTCATAGAAATAATGAATTAAATTGTCAGAGATGCCTAATAGAGTAATAATAAAAGTGGAAAATATTTTACACAAACAAAACATGACCCCGAGGAAGAATGTCCATGGGATCAAAGTCTTGATCATTTTCCTGTGAAACATTGTGGCTTCTTAAGGCTATGTAAAACAAAGCAATGTTTCTACGTCAATGCAATACAATACACCAGCTCGGAATTGAAATAACATGCAATAGGGAAGGGCACAGCTTAAGCAGGGTTTGTGATCCTTTCTCAACAGAGTAGGTTGATGACCAATGCAGATTGCAAACTACTTGATGTAGTTGAAGGCCATAAAGAAAATCTAGTGTGGGTACAAATGTTACTTCACTTTTAAAGTGTAACATCATTTTGATTTTTGAATAAGAGAGTCTCCTGTTTCAAATAGATTTGATAAGCTATTTAATGTATAAAAAATCTCTAAAATTAAGAGATTGAAAAAGTGATAAAGTAAAAAATTAGTTACTATTAAATTTATAATTTCTATCATTTATAAATATTTTTATTGGTTAAACATTTATTTTCTTACTTTATTAATCTTTTCCCCTTAGAGGAAGTGAGGAACATCATAAATTTGGATTCTCTAGATTTTGAACTTTATTTTAGATAGTAAAATGTGATTTTTCACTATTTATTGATTTTTCACTATTTATTTCATAGATGAGACAAAAAAGAATATGTGAGAGAAATTATTCAAAGATAAAAAATCACACTTTATCCTCTAAAGTGAAAATTCAAAATTTAGAGGATCCAAATTTAACATCCATTTAGCGCTTGCCTAAGATCAAGGTTGTCGGATCTACCAGCTCTCTTATACGGTTATACCCTTCTCTTTTTGGGGCAAGAGCTAGGCTTCATCTTGTGGACATTGGCCAAGGGAACAGGTAATTAGACTCTCCTCGGTGGTGAGTTTTCCCATGTAGGCTGTAGCATCATCCTATATTAATTTGATGTCACGTTTTTTATTAAATTTTACGAGTTTTTATTATTGCCACTATAAGAGAATTTTACATACATAATTAATTATATATTAATACAGCAATAATTTAATTTGTAACTGAAATCTTACACACATATTAAAATTCATTCAACTTTTGTACTATCAAACAAATCTCTTTTTACCGTGTAATTTTGAAGGACAAAAAATCACACGCATTAGGGGTGATGACTGTTAATTTTCAAATTCTAATTAGGCCAATTCTATGGTGCCTATCACTTTGGTGCCGAAATTGCCGAACTTACCTTGTATGGTAAGTTTTAAATATTAATTTTTATCTTCTTTTAGTAAATTAGATAAATATTGATAGGCACCATAAAATGCACCTTCTAGTTAATATAGAAAGGTAGAAATTGAATCTTCGGGGGACACAAGTTTATTCTGGAATTGGAGTTTGGACGATGTATTAGTGAGTGGGACCCAGCAACAAGATTCCTGGAGTGGAATGTAGCTGATACCTTCTCTCTCCAAAAAAGGAAGAAAAAAAAAATTCATTTTTCAAAAAGGCCGCGCCCGTGTACGTCGCGGCCGCACACAGCACCATGCACCACCGCTCATCACCCCACACATCACACAACCCCACCGCTCTGTCATCACAGCCAATCACAACACGCCACGTCACCTTGCCACCCCCCAAAACCCACACAAATTCTCACCGGCGGGAACACGAGTGAGAAGAAAGCTTGCGCAACACGAATCTCTCTCTTCCGCGTTTTTTCCCCTTAAACCCTTATAAAAACCCTAACCCCGATTAAAAAACCCCAAACTTTACCCACTCTCTCTTATTTATATATGATTCGATTGCTTCTTCTTCGTTTTGAGTTTCAATTCTCCGCACTGCG

## >AiNAC60

TTTCTATTTCTCATTTATGCCCTTTTCCCTATTCAGGTGAAGGATAGGCATAATGGAAACCTTTTATTGGATGAAGAAGGTCACATCATACATATTGATTTTGGCTTCATGCTTTCCAACTCACCTGGTGGTGTTAATTTTGAAAGTGCACCTTTCAAGTTAACCCGAGAGCTTCTTGAGGTAAACTTATATTATATCTATTTCATTGGGTATTAGATTCTATAACATTGTTCACAAATTCACAATAATATAATTGTGCTAGGTCATGGACTCTGATGCTGAAGGTGTTCCAAGCGAGTTCTTTGATTACTTCAAGGTGCTAATGAAACTAATCTTTTGAGAAACTTCAAATTGCATATGCTATTGTTAGCTCTAAATTTGTCTGCTTGCTTTGTCATGTTACCCCAGGTTTTATGCATTCAAGGGTTCCTTACTTGCCGCAAGCATGCTGAGCGAATTATTCTTCTTGTCGAGATGTTGCAGGTAATATAAATGTTAGCTTCATTCAGTAATACTCCCAATTCATATTGGGTTTGTACTTTTTTCATTCTTAAAAAATTCCGCTTATGTTGGAATCAGGACTCAGGCTTTCCATGCTTTAAAGGCGGTGCAAGAACAATACAGAACTTACGAAAGCGATTTCATCTGAGTTTAACAGAAGAGGTTCCAATAATAACATTTCTTTTGTAATTACAGTGTTTTGGATGTGATTTTAACCTTTTTTTTTCCTTCTGGCAGCAATGTGTCTCCTTGGTGCTTTCACTTATTAGCAGTAGTCTGGATGCGTGGAGGACACGGCAGTATGATTATTACCAGAAAGTTTTGAATGGAATATTGTGAGATAATAGGTTAATAATGATCACTTGCTTGTTCCATTTTTAACCAAGCTTGCACAGCTGGTGTGTCAATTGATGCTCAGTGGAGACACTTTTGAGGTTTTTGACAGAGCGAAGGATAATAGGTGGTGGCATGCAGCAACCTCTTTTCTCCTGATAGGAGGAAGATTCTAAGAACCAAAATACGTTAACAGTACACCAGGCCAAATTTGCTGAATCCTGGGTCTTTTATATTATCTTTTCTTTCATTACATCTTCATGTGCATGAAGCTGCTACTTGGAGAAAGAAGCTGCATGAGCCTCCTAAGCGTTTGGATAGTACACAGTGTACAGATATTTTTGGGGAATAGAACGGATTCACGTTGGATGTTGAGACTGCTACCTGGTGCAATTACGGATTCATGCAGAGTCAGATGCAGGGCCGGCACACTCTTCTGCATGACCCTTTCATGGAAGAAATCGTGGGCAGGTCTTCGGTGTTGGGCAACTACTCTGTTATTTTTGGTAATGATCTTAGCTGTTCAACGCATCTGTCTTCCCAGATTTGATTCTTCAATACTTCACTCCGGAAAATTGTAATTGAAATTTGTTAATGATGTTGCTGCATTGTTTGCAATATTTAATTTTAAAATGGAAACTAAATTGGTGAAATATATGTAAATGATATTATAATGTGATATACATTAATATGGTTTTCATGCAAATTGCCCAGGGATTTGTATTTGCATTGTCTGTCTGTCCTATTCCCCCTTCTCTCTTTCTAACTCAGGCTAACACCAACACTACTGAGAAATGTGGAATTGGGAACTCATACATCCACCCATCTCTCTTATGTAAAAAGAATAAAATAAAATTAAAATTAAAAAAACTTACTTGCCCAGTTCTTCCATGTTACTCATTTTCTCCTCTTCAATGACATTATATATACCAAAACCAAACTACTCACAAGCATGGTGCCTTTAATGTAAGCAGCAAAGAATAAATACAAATTTAACCTTCATGTTTTGTCGACTTCCATGCACAAGATATTGGAGGCTTTTGAGAAAAAAAAAAAAAGTCTTGAACCATGGCTCTAGGGTAATAACATTGGCAGTGTTGGTACTTGGTAGTAAGAGTATGGTTTAATTATATGGGTGAAGTTCAACCGGTGAACTTGAGTACTTTTTGACCAAAAAAAAGAACTTGAGTACTTGACAAATCCGTTGGGATTTTAGATGAAAAGAGGTGTAAATAAAAAATGCTTCAGCTAGAAAGTTAATAAAAAAAAATGCTTCAGCTAGAAAGTTGACAAATAAGTCTAAGCCAAATCGTTTAATCAGGTAAGGATTAAACATTAAATAAACATATAGTTTAATCATTCTGTTGGTCTCTATAATTTTGCGAAATTTTCAATTAGGTTCTTAAATTTTTTTTTTTAATTGGATATTTATACCAATTTTTTTTTCAATTAAGTCCCTTTTTATAGTAATTGTCTTAATTTTATAGGGACCCAAATAAAAAAATTGGTATAGAGACCCAAATAAAAGAAAAAAAATATACGCGAGGGGGTTTCCCAAAAGGCTAATCAGTAGCCCAAATTATCTTATTCTCAATAGAAAGAAAACTAGAAAAGAAAAAGCTCTGTTCATAGTTACTGAGTTGTGTTCTCGCCAGCACCTCCTCCC

## >AiNAC61

ACACATTGCAATTAAATGAATTTTGGAATCCATCTAATGTTTGAATCTTTGGATCCGTTGAAAGCAATTTTGCTTTCTTCCTTCTTTGGTTTCGGATCATGCATGAGGAACTCTTATAAGATATGGGCTTGATTGCAACAATATTTGATCTTGGCCCATTAATAACATTTGCTTTCCTGATAAAATTTGGAGTATGTCTAAAGCAAATGGAAAGCATGTAACACTCTTGGGCTTAGAGCAATCAAAATCATTTGGGCCCAAGTAACATTATTCATTTTATAAAAACCAAGGCTGAGTGTAAATTGGGCTAGCATCATTTTCTTTTTATTTCGGTCCAATAAGACCTGCAGCAAAATTATTTAATCAATATATGTTGAATGAAAAATTCAGATTAATAATTTTGCAATTAATTAATTTTAATAATGTTTAGTCATCACAAATATTAATTTAGAGTTTTCCAAACTCATCACCAGCGTCTCAAAAGAGAATTCGTATCAGTTTTTCAATGGCGCCTAGTTAATAGTATGGGTGTGCATGTCCGGTCCGGCCCAAAGATCTGACCCGGTCTCGAACATTTTAGGGGATAATTTGGTGTAATTTCACTTGGTCTAGAGCCGGGTAAGGATCTCAAAAATAGACCCGATCATTATTTCGGGTCGGGTCCGGGCCATAGCTTGGGTCATCCGAACTCGGCCCGATGACCCGGTCATCATACACAATTAATATTTTGTGTTATTAATGATGGATGATGACTATTCTTATGTGGAATTTAAGTATTGTAAATCTTAATATTTTATGTTATTAGTTAATTATTATAAGACTATAAGTTAATGTTTTATGTTTAAAATGCATAAGATTTTAGACTAATGCATAATATTGTGTTATTTGTATTGATTTAAATATTTGGTGTTATTAGACAATATTAGTATTGATTATGGTTATGTTTTAATTTCAAAGAAGAGTTGATTCTTGTTATATTTTTCTAAGTGAATTTTACCATGTCAAATAATGGTTGGAGTCTTAGAAATTTGGATATTTTCACATGCTAGTTTATAAGAAAGTATCAAGGTAATGTAATATTAACTGCCCGGTTTTCACCCGATTTTTACCCGGTATAATCGTGGTCCAAAAGTATATATGTTTCATCGGATTTAAAGTCGGGTTCGGGTCTAATAAATAGGCTCGGTATATATTTTGGATCGGGTCTGAGTCACATCAAACCCGATTTCACCCAACCTATGCACACCCCAATTGATAGAATTAGGATTTAGAATTTTTAAAATTAATATATATATAATAAGAGTATTTTAATCATTTTCTAATAAAGGTATTGTAGTCATTTTTTATAAAAAGAATAATATTAATTTAGACCAGTTCAAAATTTGATTCACTATTTTTTCGATTAAATCAATTTGTCTAGCTTAATTTTGACAAAAATAACATGATTTAATCGATTATATATGTTAATTTTTAATTACTGAAAAACATATTTAAAAAAAGACGTTTTAGACATCTTTATTTAAGTGATTCTCTTTTTTATTATATAAAATTTTTAAAAAAAATATACATTTGCTATTTTAATTCATTGATATTTTTTATAAAAATAGTACGGATGTACCACAAAAATAATAGAAGAATTTAGCAAAGTAATGACTAAGTAGCATAATAATGTGAAAAGGGAGGGAAAGTGTAGAGAAAGAATGGCAAGAGTGAAAGAGGAATTTCCGGGTAAAAGTCCAAAACTGAGAGAAAATGAGGTCAAGGCATAAAATAAACTTACCAACACTAGCAATAGCATTAACATTAACATTAACATTAACATGTCTCTCTCTAACACCGCACAATTTTTTTCTTTCTCAATCCTACACCTCAGTTTAGTCACCAGACTTTTTGCTTCAAGCCGCGTGGGTCCACTAACTCTATGCTCCCCACCAAATAAATCTCACTACTTTCATAGTTTGCACTCACAACAATAATAAATAATATCTACTTAATTTACTCATTAGTTTATTTGTTTACTTAAATAAATATTATAAAATTAAATTTTATTGTATATAATAAATTATGGATGACAAACTTTTAAATACAAAAATATTTAATAATAAAAAATAGTTAAAATTTGTTTAATTTATTATTTATTAATAAATACTAAATAAAATATATTCTATTAATTTTTTTGTTTCTATGTATTATCACCTTAAATAAAATTTAAATTTGTGATGAATTACTCATTAACAAATTGAATTGGAAATTTCCAAAAAAAATGGGTAATCTCTTTTTTTTCCCCATCAAATAGTATGTATCAACATTCAAGTCTTTTCTTTTCTGTCCTCTTCTATGTATAGTCTACCTCATGACCAAGTCATAACCCAAATCACCATCATTAATCACAATTATTAAATAAATAAAGCCACAAATTAAATAGTTATGAGCTCCATTAACACAACCAACTCCACCTCCTTATAAATATACACACCCCTCTCAATATCATATTCATTTA

## >AiNAC62

GTAGTCTTTGATCCTGAACAGCATCACTTAAAAAGCGCACCATATTTCTAAACCTCAAAAGTGTATTTTTTAATACAGAAAAACGTAACCTTTAAAAATTAATAGGTAACGGCCGAATAGGCTGAAGCATCTCCATAATAGCCTTTATCTAAAACATAATTTTTTTTTTTATTTTTAACTACACTTTTTAAGTGTGACTAAATAGATATTTTTTTTGTAGTGTATTTTTTTATCTTTTTTTTTATTTATTTATTTATTATTTTTTTGTCTTGAACATAAAAAAAACTGCTTAATTCCATAGTAATAGTAAAATTTATAAATAAAAGCAATTTTATCAAAATGTCCTAAATNNNNNNNNNNNNNNNNNNNNNNNNNNNNNNNTTATCAAGTATTCTTAATCACACGCTTTTTGGAAATTAAATTACATCAGCCATAAAATTGTAAGATCACAGTCATTCATAATTAAATGGAGTAATAAAGATGTGCGCGTAATAAAGCGATTTTTTTTATATGAAAATTACGTGGTCATGGACCTTAGAAAAATTATATATTGTTGTAACAGCGATAATTTTTTAAATTGATGAAAAAAAATTAGATAGTTTGATGTTTTTGTGGGGAGAAAGGAACATAAATTAGATAGTCTAATTTATGTAGAGCAAAATTCTTTGATTTTTTGGGTATAAAAATTGGACTTACCGATTTTATGCATTTAATTTTTTTTTTATTTCGATAGAACTGTAATTGGACGGTCCAATTTGAGTTTTTTAAAAAAATTAAAATAGTAAAAAGGCATTCGAATTGTCCAATTTCTTTGGTTATATATATCCCAATTTAGCTGAACTTTCCACTTCTTCGTCTCTCAATTTTCAAATCTTCTGAAACTCCTTTTCTCTTGTTCTTCCTCTCCATTATTTCAAAAGAAATAATACTGAGGTTGAAAGTGTTCAATGTGAGTATATTATCATGGATGATAGAATTATATTAAAAATATATTATTATGGACAGATTTTACTACAAACATCTAAAGGAGTAAAATTTGTATGTGAGAATCCATTATATATTGTTATTCCATTCACACTATCATTTGAAGAACTAAAAGGTTTGTAAAAAGATGGATTCTCAAATATCTAAAAAAGTGTTGTGTATTTTATACAGTGTTTGGTGGATTCGTTCAATTTCAAATCAAATATGTAACCGATGAAGCGAGCATGTAAGAAATATTTTTAGTGTATATTGAAACTTACTGCCGAATATCGTTCATCGAGCTGTATATTGAGTTCGAGCAATCTGAAGCTGATCAAGATATTGAATTGGAAGATTATTATAGTGATAGTGAGGAGGTGTTTGAAAATAACTATGAAATCGTTCATCCAGTTGTAGACGAAGATTAAGCTGACGACACTATGAAGGCAGATATGGCAAATGCACTAATAAACTAGCATCTATTTGAGGAGCCTACTTTTATGCGCTTGTTGGATTTGGAGGCCATGTATGCATCATAATTTTTCTAATATATAAATGCAGGTATGTAATTTAAATATTTATATGATAGATTAATATTGTAGATTGATTAGGGTACATGACCAACATATGTAATTTATAGAATATAATTTATTTACTAAAATTCTCAGAATCCTCTCTGATTCCAGTTCAGCTTCACTTAATAGAAACCAATCAAAAGATCATCGTGAAATTTTTTTCTTGTTACTTTTTTTCTTCGTTTGGTTGTCACGAACGGTGCCGTTTTATATATACATAAAATAGAGACACCGTCGTTTTGACACAAACGGTGGTTTTGTTATGGGTGCGGTGGAGGTGTTTCAACAGCAGCCGCTGGTGGTGGACGCTGCTCCGGTTTTGTCGCTGAACTCGCTGCCGTTGGGGTTCCGTTTCCGACCGACAGACGAGGAGCTCGTTGACTTTTACTTGCGGCAGAAGATCAACGGCAATGGCGATGAGGTTTGGGTCATTCGAGAAATCGATGTTTGCAAATGGGAACCTTGGGATTTGCCTGGTACAATTCTCAATTCTCTGTTTTTTCAGGTTTCTATTGTAGTTTAATTCTTTGATTTTGCAGTGGAAAGGAGATTTTAAAGTTTTCTTGGTTTATGCTGTTGGAATTGTTTAACTGACTTAGTTTGACTTCATAATTCTAATTATGTGATTCATCTTAAAATATGACAAGCTGAATTTTCTAATTATGCTCTTGAATGGAATTATAGAACTATGTTAGAATAATTTGAGTGAAATTGCAAGAGTTAATTTGGATGCCCATGCTATTTGCTCATTTTTTGTTCCTTTTGGTGGGGGTGTGGACTGGGGTTTGTGTTTCTAGATTTGTCAGTGGTACGGAACAAGGATCCGGAGTGGTTCTTCTTCTGTCCACAGGACAGGAAGTATCCAAATGGACACCGGCTGAACCGAGCAACAACTCATGGATATTGGAAGGCGACTGGCGACTGGCNNNNNNNNNNNNNNNNNNNNNNNNNNNNNNNNNNNN

## >AiNAC63

CATACACTGGGTTTAATTTGGAACACTTGAAAATAAATATGATTTTGATAGTTGATCACTCTGTTGTGATCTGATGCAACTCTATTAGGGTGATACAACGGAGGTAATAAGAAAAATTTAATGGAAAGATCCCGAGTTTGAATTTAGGGAACGGAGAAATTGTGGCTTTTCTTCACTAATAACTTCCAAATTTCTTAGGATAGTTAGAAACTAAGAAAATTAGTAAATCATATTAAAATTGTTAATCACAGTTTAAAAAGCAAAAAGATAGGGGAATAGAGTTAGGTCCACTTTTATACAGAGATAAAAGAGAAATAAAAAAAGAAAAGAAAAGGATAATAATAAGGTGGAATAATGTTATTTAAGAAACAATTTTTCAACTTAGAACCATGTTGGAAACATTCAGATAAAGCTTCAGCTTAAAAAAAAGTGTTAAATGGTTGGACCGTGTTGTCTGATTATTGTTCACTGCATATTCAGAACAGAAAAAGCTTAAGAACAACCTTAAAAGCTTTCAACACTTGAGACAAAATAACTTTAATGCTAATGAGTAGTGAACTTCAAGTTTGTTTACCTTTGTGCTTTATTGATGAACCTTCACTTTTACTATTCACGATCCATCTGCTATACTAGCTAGTGAATGGTTACATTATAAATTAAATTAACAATGCTAAGGATCAACATTTCAGTAAATAGTTTTAGTCAACAATAATTAGATTGATAAAAAAATTCAACATACAGAACAAAAAAACATCACATTAAAATTTTTCTTTTATCATTTTTTATTTTTTTCTTTCTCTCAAGTCTCAATACTATAATTTTATTCATCTATCATTAGTAATGACTTTGACCATTGTTTGCCACTGACAGCCACCCGCTGCTGGCTACCACTGTTAAATTCTAAATTCTATTTCTAAACTCTAAATTTTCTAAAAAATAAGGATTGAAAAAATAATTTTACAATTAAAAAATAACTAATGTTAACTATAAATTACTACTTTATATTTATTCGTTTTAAGTAGTAAATAATAAAAATTAATATAATGTAAAAAGCAATGAGGTTAGTATAAGCAGTTTTAAAAATGTTATATTCTGATAAATAATTATATAAAATATAATAATTAGGCAAAACTATTATGTTTAGAGCATTTGTTATCAAAAGTGAGTTTTCAATTACAAACTATTTTAACATTTAAAATACATAGTCCAAACAAACAATAACTAATGCTAGAAGAAGAAGTGAATGACCACACGCACTAGATCTCTATTCTGTACAATTTATAGATTATACATGAAAAAAAAATTGAAAATTAACAACTTTTACTAATAATCGGTTAATATTTATTTACTAGTATTTTTATCCGTAATAATATTATAGGAATATAAATTTTTTAAAATTACGATCCATTTTGTTCTCACTGTATAGATTATGCTTAATTCTTTGTCGAAACCATTATTCATAGTATACTCATAAATGAAAGTGTTAACCACAATATATATCCTTTTATATACAATGATATATTATGTATATGTGAAATGTGATTATCTTCATCCATTGTACATACTAGAAAGCTTTTAAAGCAGATGATGTGCACCACGCGGGTTGGATCAACAGGGCACACGCATGAAACCTGCTCAATAATAAAGGTTAAAAGAATAGGACGCGATGTGATGAGTACAATATAATTTTTTAAAAAAAAAATCAGGATTTTCTTAAAAGAAAATTACGAGAGTTTAAATGAACACATTTGTTTATTAAAAATACTATATGTAATTAATTATTAATATTTAAAAGTATAAAATTAAAATATATTATTAAATTACTAAACTAAAAAAATTAAATTATAATTAAAATTAATAATAAAAAATAATAAATTTTAACAATATTCTAGTATTTTCTGTGTATATCAAAAAGAGCTTTAATTGGTGGTAATTAAGGGTTTGATGTTTAAATTTGAATCAATTTCCGGGCGTCCCGTCACAGGCAGCTGAAATAAACGTTAACACCCACATATAATTATTACTTATATATATTCTGTATGAGTTGGTGGTAGATAATGGAGTTAGATGGTGACACCATTACCTCTGTGAAGATTGATGGCGTTTAAGCATAGCGCCACCCTTATTCACACTCTCACTCAGCTTTTCCTACAATCTAGCAAGCCAACTTTAGGGGAGGGACACAACACACACATATATAATATATATTTAAAAATGTTATTTGTACACTAAAATCAGCTACTAAAATTAATCATTAATGTATTTGTGTATAAATACATGTGTAGTTTAATTTATTTTAAATATGTATTTATATTCTAATATATATTTTATATTAGTGGCTGACTTTGGTGACTGATTTTAGTGTTTAAGTAGCATAACTCATATATATTTTATTAACTTTTATTTTATTAGGGTTACCTCTCATCTTATCATAATACCTTGCTATATATAGTTTTCATCTCCTTATTGTTCCTTTTGCTTCCTATTCTCTCATCTTCTC

## >AiNAC64

GATTCTTTTTAAAAAAATAATTTAAATAATAAATAGTTATATTAAAAGTAGTTTATAAGTAAGTTATTTTGTGTTTGAATTTTTAATTCTAAAAATGTTTATTTTATAGAAATGTGATAAAAAAATAGTAGTATTATGAGAGAAGTCATTTTTTTTAACTTCTCTAGAAGCTCCTAAATAACTTCTTAAAAAATTAAAATTTGATTTTGAAAATTACACCAGACATTAATACTACTATTTTTCATAAATCAAAAGCTCAAAAAAAATTGTTTTTGTTAATAATATGGGGCACCAGCCCAAAGCAAAAAACTACAAAAAGACCGTACAGGAGATAGAGACTCCCGTTAAACCAGCATTAAGCAAGCTCTGTAGAATAATATGAAGGAAGGCCCTATATAGTGATGAACAAAAGGCCGAACACCAATATGAAGGACTAAAATAGTAGGCCCAAACTAACCAAGAAGAACAATGATCCGTATGCTGTATTTGGGAAATTGGGCTTAACACTCTAAGCATGAAAAAAAAAAGAAAAAAAAAAACTAAGTTCACGTAAAAAGAAATAACAATGGAGTACTTTTTAATTTGGAATATATTTAAAAAAGTTGACATTTTTGGTTATTTATCATACATGGATGGTTTTAGAATTAAGAGAAAAGAGTTCCCAACGATAACTACTTGCTTCTTTCTTTCTTTAATTAAAAGGTAGGATATGAGGCTTGAAATGGAACGATATTTTTAATTAATTATAGTATTGTTTTTAAGAATAAACAAAATAANNNNNNNNAAGAGGCTCCAATTTATGTATTTCAAATATTTTAATTTTTTAATACAAATCAGACAATTCAATTTCTGTATCTTTACAAATCGAACAGTCCGATTTCTGTACTTCTAACAAATTGAATGATCTAATATCTATTTCTCTAATTAAATGGTTCCACATTTAAAAATAACACCACATTAGTCCACATTAAAAAAAACACCACGTAGTAGCCAATGATGGGTCCCACGAAAAATTAAAGAGGACAAAAAGTTTTGCCTCATCTTTTCCATTGCCCATATTTGTGACATTACATACTGAATATATTAGTTTCCAAATTTCAATAATTATACAAATTATTGAAAGTAGATGAGAGCTAAAAAAGTGCATTTCAATTTTTGTCGAGAAAACAGCACGTCATCATGAACTTGTTCTAGACTTAGCTTGGAGAACACGCAAGAGATGATCAGAAGGTGGCTTAAGCTTTGTGATGGACGAAAAGCATGAAATGCTGGTCCAAGAATTCGAGGTTGACATACAAAAGGTGACAAGTTTTGAATCGATTGGAACTATGTCTCTAAATGTTATGCAGTGTGTGTATGGGATATTTGCCAATCATATTTACACCAGGTAACTTCCAAATCTAGAGTAGCTTTCTCTACTCTCCACCAAATTCAAAACCTGGGATTCATCGGTTTTTGTTTCAAACCACACTCTTATTGCTAAAACGTATTTCAGATTAGACGAAAAGTTATAGTAACAAATTACAAGGCTTCTTAACTAACACAAAACTTTAAAATAATTAATGAGTCGAAAAAACTTTGGTTACTATTCACGTGCAGTTATCTTTATGTGAAGATAAATTTAACATATGTAAGTACGTGAGCTGTATAATATGTTAAAGCTAAGTGTCATGCAAGGTGTAAATGTAGCAAAGCTAGGCCCCCACGTAGTTTGTGGTTCATATTGGTAGAGAGTAGTGTTTGGAAGCTGTATAGTAAGAAGTAAGAAGAGAAGACAAGGAAGAAGGGACAAAGTTAATGCTAAAACAAGGAAGTTTCAAGGCAATAATAAAAAGTGCGGTTGGGTGGCTTTATTATGTCTTTCTAATAAGACAAGTGACGTACTTGTCCAACCGCAGATACCTATGTACGTGCCTATGTCATCCCTGTGCTACTTTCCTTCTTTCACTTCTTATGATTACCCTACCACCATTATTGGTAACATAGTAATTAGTAACATCATTTGAAGGATCTCAGTCACAAGGTAACGGTATCAACTCTGATAAATTTACTTCATACTTGAGATTGAAATACAAGTATGCATCTCTATGAAATAGCAGTGTATATATAAGATCAAAAAAGCATTGACGTTCAAGTCAATAATAATCTAAGAAGCATAAATATTAGGAATAAATAACGTATCTTGAATATATTTTAAACTCTTTATTTATAGTGTTTGAAAAGAGAATTTAAAGAGATTCAGATAAATATGTGCGAAATATACACAGGATTTTCTGTAGTGTATATATCCCCAGAAAAAGAGTTCCTAGAACAGATATAATTTTAGACCCAATATGGGAGTTGGACCTTAACAATTCCGATCGTATTGCTCCTATCTATTGTGTATGTTATCCAATTAAATGAGCAACTCTCTCTTTAGCTCTTAATCTGATTTGTTTTGGATACAATTATTTTCTCTTTCACATGATGAGTTCTATAAACAACATTGCTGAGAAAGTGA

## >AiNAC65

ATTATTTAATTTATACTAATCCTTGTAACTAATTTTTGAAGTGATGAGATAGACTACTCACTTAATACCGATTCTAATAGTAGTATATTTGATATGCAATTATGATATGAAAATTAAAGTGTATAACTAACTGAGAATGTAACAAAGACAAATGTAGATTAATGTATAGGGTACATAGTGGTGCAAGCGCACTAGTTATGCTGCTAATAAGAGATTAAGAATAAAAGAGTAATTAATTAAGAATGCAAGTGGATAAGCATTACGACAGAGGGTGGTGGAGGTGTTGTGGGTTTGCATAAGAGAACAGGGAATGCAAATGATTAACGCACCCCCCTTGATGTAGACACCTCTTCAACAAGCTCTTCTCTCTTCTGCCACGTGTCATCCAATCAATGGTGCCAATTGTGGAGGGACGGCGCACCAAACACACACCATTTATCATCTGAAAACCTCAGTACCATGATGTGACATGCATATATGTTCTTGTTTTGCTCTCAAATTTTCATTAGCCTTTAGCCCTTTACCCATATATATATATAATGTAAATTAAAATATATATCGCTGCTTTTACTTCTTTATTTAGTATCACTACTGAAAAAAAATTAGTTTCCTATAGTTTTGCCAAATTTTTAATTATAGGTCCCTATACTTTTTTTCTTTTTAATTGAGTCTTTGCACCAAATTTTTTTTTAATTGGGTCCTACACTTTTTTTTTCTTTTATTTAAGTCCCTATACTAATTCTTTTTTTTTAGTTGGGTCCCTATAAAATTAAGTCAATTACTACTAAGAGGAACTTAATTGAAAAAAAAATTAGTGTAAAGACCCAATTAAAAAAAAAGTATAAAGACCTAATTAAAAATTTCACAAAACTATAGGGATCAACAAAGTAATTAAACCTTATTATTATTATTACAACCTATAATAATAACGACAAATATATAATTACCACAAAAATATAATATAAATAAGATATACAGTGGTTATCATATTATTATTACTACTAAAAATTTGTTATTGTTAACATGGAGTAGTATATTATATAAATTAGAATTCTTATTTATACTTTTATAGTTAAAAATATCAACATTTAAATTTAAAATTAATAATATAATATATGAAATAGAATATAAAATTGAATTAAGAGAAGATAGATATATCGGGTGAAATAAGAAGAGAAAGAATATGAGTAGTGTTGGGGTGGAAAAGGGTTAATAGCGGTGGAAGAAGGAAGAGAAGGGGGGTGGCGTGAAAACTTGTTGTCACGCCGTCCAAACAAGACAACTTTTTGGCCCCCTCTTCCCTTAGCTTGCTTGCATCTGCCACCCTAACCACGCGCAATCAATACCCTTCCTTCTATTAATATAAATATAAATATAATATTCTCTTCTATCTGCTTCTTTTCTCTACATATATACCATTCAACAAACATTCCTTCCTACTCATAAATACACCCTCTTCTCTCTTTAAATAAACATTCACATCCCTCTCTCCCTCTATCTATTCTCTCTCTCTCTCTCTCTCTCTCTACCTTCAATTAACAAGATCCAAGATATGACACTAACACAAATTCCTGTGAGTACATACTATTATTTCATGCATCTCCTTTCTAATAAACACCATCATCATTATTATTATTATTCTTTCATCACATTTTATACAAATCCCATGATCTTTAATTAAACTCCACATTCTCATGTGTTGCCATACATCCTCATCCATCTTTGGAACAACTGAATTATATACATATACCTAGCTTAGTTAGTAGTTAGTACCTTCTCATACATTATTCTTTTTAGATTTCTTGATATTCTATATATAGAAATAAAGGGTCCCAATCCAATGTCTAATATGTTTTGGATTTTGGCAAAAGTGTAATAATCAGATGAAGTTTATTTGAAAATTTCTTGTCATCATCAAAGAAAAAATTATTTCCCCAAATTTTTAATTTATTTTTTTTCCCAAAAGAGCTTTCAAAAGTTATTAGTATTAGGGTTAGATGTAGAGGTGTGTCTAGCTAGCTAGGTTTGTAAAGAATCTTATATATTATTGGAAAATTGTGAGATGAATGGTGATGATGATAATGGTGGTGGTGAGAAGGTCTTTCTCCACCACCAGAATGAAGGAGTAGTAGTGTATGTGTTGAGAGTGCACTACACTCAAAGAAATGTATATATGAAAGTCAAGCTTTTCGGCCACAACTTATTGCATATATAGGACTACTTTATTCATGTCATGCTATTGCTACTTATATAGGACTAATCATAATATATATCTCTGATGATGATCATATTTTTGTTTCCATTTTAATGGTGGGGTTCCACACATGGTCTGCCAATTTTTCTCATCGCACATGCTTCACTAATATTATTCTTTGGGTCCAATACCAATGTTACATCATCATCATTACTCTCTCTATCTTATGTATATATGTCAGCCCTAGATAGCTACATTATTTATATATCTCCCAAATCCAATTTTGGATAATATTTTTCGATCTCT

## >AiNAC66

AGAGAGAGATTGCAACACATGATTATTAGGGGAGGCTCCACAAACAGAAACGCAGTTAAATTTTATTGGAAAAGGACACCTTAGTGCATCATAAATCATAAACTATAAAAAAAGAGCCTTAACCAAAAAAAAAAAAAAAGAAAAAAAAAAAAAAAGAAAAAAAAAAAAAAAGAAAAAAAAAAAAAAAAAAAAAAAAAAGAAAGGAAAAAAAAGACAGAAATTAAAGCTAAGAGAAGTGACTAAGTGAGTCCCATGTGTAATAGAACTAAGCAAGACACTTATTTAAATAATAGTATTGTATTTTACTTACACCTACAGGAACCAACTACTTCTTTTGCCGCGGTCTTAAGCAAGGATTCAAACATGCAAATACGTAATATTGACTTCTTATTTTCTTTTTAATTTTTTTAGTAAGCGCTAGTGAATCTTTCAAAATCTGATTGTAGTGGGAAAAATAAAGCTCTTTAAATCAACATCTTCCTAGTGAAGAAAAGTACCAATACTTTCTTTTAAATCCTTTTCTGATCTTAATCTAGATTTAACAATGGTCATAATATATTTTAGTAAGTAGTGATAACTGATAAGATAACGTATCTTTTTATATGTTTATTTAGAACCGCAATTTTTAACTTAAAATATGATTTACATTATATGTATATAGTTTACAATAATAAAATAATATGATTTAGTGATTTACATTATATAATGTAATAATTACTCTGTTCTAATTAGTTGACTCTATGTGCATGTTTAATAATGTGATGGTGGTTTTGCTTTTTATCGAAAGTATATATGGGCCTGTTCATTTTTTCCCCTTTATATATTTTTCGTACATCCATTTTTTTAATTGAATTCTTTGGAAACTTTAATTTTACAAACAAACAAACTAAAATTAAACAGAGCCGAAGCAAATCGTTGTAAAGAAAACTACTAAAGGAAAAAGAAGCTTCGTCTTTAGAAACACTTGCAGGCAACATAAAGTGAGTCTGAGTCATCTATCCATCAATAATAATTATTCAAATTCATTTTTTTTTTCTTTGAAAAGAGTAGGCAAATAGACAATAATTATATCATGATCAATTTACATGGCAAATATAGGAGAACTTTAAGCACGAAAATTGAATAGAAAAATTATATTAGAAAAGGTCAATAAATATGTAAAACGATAATGCGGCAGACATGAATCACATGCAAGACTTTGGTATATGAAAGTAATAACAAGGTCAGGGTCTTTGATCAGAAGTAGAATTTAATATGAAATTTCTTACGTGACAGGCTCTGATTGATGTGTGTGAGTATACTACTCTTAATAGGGGGATCGGAGAGAAGTTTAATTTTCTGATTTGAAATAATATCTAGTGTAATTTTCATGTTATACATATATATATAGCAAAAGTTAAAAAAAAAATGATGAATAATAGATTGATTAGGGATTTTTAGGACTTGTATGACTTGTATAGTGAAAAAGCTTGTATGTAAATATTAATCCAATTATTTATTTGATTTGATTGAGATGAATATTAAATTATTTAATATTTTATTTGATCACACTAGCAATAACTAACCAATTGCTCTTTTATTCGTTAATACTTTTTTTTTTTACTTTTTATGATGCATTATTTTTACTTTTTATTTTTTTAATAAACTATGAAACATTATTATTCGTTATATTATCCTCTATCTCTTTTATATTTTTTATTTTGTTCCTATTTATTTATTAAGTTTTTGTATTTTTATTAAAAATTTTATTTATTCTATACAAGTTAATTTATCATAACTTTTCACATGTTAAATTTTTTTAATGTTTAAATTATATTTTTCACGTGCTTAATTTCATTTTTAATTTAGAAGATTGAGTGATAAAATAACATCTATAAAAACTATTGTGCGTTGTCTTTTTTTTCTTCTTCGTCTTTTTTGCTTTTCTCTCTTTTTGTGGGGATTATGATTAAATGTAATGGTGCTTATACATAAATGATAAGTATAAATTGTATTATATCTTTTTTCAGTAATTTTTTTTATTTATCTTGAAATAAGAAAAAAAATATTGTAAATTACTTTGTACTAATAAACCCATCATCGTTATAATAAGATATTGTGCATTTATCTTATCTTTAGCAGTTTTAGTAGGTGTTTACGTTAACAAAAAATATGAAACTATTTTTAAGTACATATACAGCTAATTCAACTCAAGAATAGATAAAATATGTTAGTAAATTTTAACAAGTAGTAATATTGGTCATATTACTTTGATTTCAAGAGTAATTAGGATATATAGTAGAATATAAAATATTTCTAAGTAGATTTTAACGAAAATAGTTCTCCCTAATAGATTTTTTTACTATGACAATAAAAAAGTAATTCAAAAAAATTTTTATGTCATGTTAAATTACATAATAGTAATATATAATAATATTCTTTCCAACAACGACTATGACGACTTTATTACGATATAAAGGTTAGGCATCATGTCAAAAGGGAAAGAAAAAGATCTTTAAGA

## >AiNAC67

TTTTGATAACCAAGTCCAACAAAAATCAGCTTCATTATTATCGCATGTGAACATGCTACCTTTTCAGTTTTCATATCCCGTACAAATATTTGTTTGTCATGATTTTTTTTATTACTTTGCTATTGCTGTATTTTTTTCTTTTTCTTTTTGGTAATTTTTGCAACATTATATATGTCGTCTTCAATTCTTCTTTGTTTGATTTTTTTTAATCTTCTTTTGCTTTTATTCTTGTTATAAGGGTAAAATAAAAAAAATTACGAGAAGTTAAAATAAAAAAATAAGAAAAAGAAGATGATGATAATGAAGAAGAATAAGGGGAGGAAAAGTTTTAAGTTATGTATAATTTATCAGAAAATAACACCAAAATTTTTTTATCGTAACAATAAATTTTTTGAGTTACACTTATTGGATTGAATTCTTATCATAAACAAAAATAGCATCGAAATTTCATAATTTTAACATTGAAATTTTATTACAAAAGGCATAAATTTCTTTTTTAATGTTGCATTTTTTTCTTTTTATTTTTCTTTCTTTCTTTCTTTTCTTTTGTTCTTACATATTCTTTTAGGAGTATTACTTTTCATATTAGACTTGAATGAACATGTGTTGCAATATTATTTACTTGAATGAATGTAGATTCGCATTTATTAGATTGAATTCTTACTAGAAACAAAAATAACACCGAAATTTGTTTAAAGTGACACCGAAACTAAGATATCTATATGTTATTAGTAAGAAATTTTAGTGTTATCTTTTTGATAATTTCTGTATAATTCAAAATTCTTCTTCTTTTTCCTCTTTATCTTCTGCTGCTTCTTTTTTTATTTTTTGATAATTATTTTTGTTTCATTTTCTAATGATAACGATAATAATGATGATGATAATAATGATGGAGGAGGAAAAAGAAAGAGGAGAAGAAATTCAAATAAAAAGGAAGAAGAAGGAAAATGAGGAGAAGGTAGCGACGACAACGACAGTGGTAATGGTCATGACAATTATAACAACAAAAGAGAAAGATTAAAAAAAAAGAAAAAGAAGAAGAACTAACAGTGTACAGAAAAAGAGAACAAAAAATACAGGTAAATATAAAAAACGGTCATACAAGTTGGTTGGATTTGATTAGATAAATCATTTAATTATAAAATATTTTTAATATTATTATTCTATTATAACTAATTGGCTATTCTATTATTATATTTAAAATTTAATTCTTTAAAAAAAGATGAAAACTCAGGTGCAGTCGACTTCACGTGAAGTTGATAATTGAGCGTCGTTATATAATTTGACTGATTTGACAAAATCTTCATCTAATTGAGCGTCATTAGATAATTTGATTGATTTGACAAAATCTTCATCTAATAACTTTTAGCTATCAATTTACGTAAATACCTGAGTTTTCATCTTAAAAAAATCATTCCACCTAAATTATATCGCAAATAAAAATTTTTAATTCAAAACCAATTTAAATTACACGGCGAACACGCCTAGTTTTCTGCAAAGCGAGATAGAAGGAGAGTATATATATGTTCACACCAAACTATAGCTAATAAAGATATGTTTGAACCACGTTTAAAACTTGTAACTCCTTTAGAAGATGATGATCCAAAAGAATGCTACTGCATAGCGTAGGAATTTGAATCCTCTAAAATTTGAATTTTACTTTAAAAAATAAAGTGTGATATTCTTCCCTTAAATAATTTTTATTTCATATTTATTTTTAGTCTCCACCTATAAAATATATAACGAGAGATCACATTTTATACTTTAAAATAAAATTTAAATTTTAGAAGATCAAAATCCAATGGCCAGTGGGTAACTCAATTTTGTACATTATTATTTTGCTTTGGTTTTATGCGGTGGTCCCCACTCACACCACACGTAGAAATAAAAGGAAATACTTTCGTCATTGCTGAGTTCATACATTCATTTACACAACACAGCACCGCTTATTATTGTATATATTTAAAACTTTGTTCTTTTAAGATTATTTCGTGGACTACAATGATTTTGTTGTTATTGGTTCGGCCAAATCATTCTCAATACAACAACTTGAGAGATCCAAGGATTCAGAAAATGATCCGCTGACTGCTGCATGCACAATAATATTTACATATGCCAATAATATTCCAGAGTGGGTGGAACTGGAACTAGTTGAATTGAATGGAAATGAAACCCAATATGGATAAAGACGTGCTTTGTTTAGATTCAAAGAATCAGAGATAATCATATAATGTACAAGAATATCACGTGGCCAATAACCGTTGATTCCAATTATTCTTAAATTTTATGATTAAAATTAAACGTCAAAATTATTAAATAAAACACAAATTAAATATATCTTGTAATGGTCCCCTTCCCCAAATGGATAACGGCAAGGCATTGCCAGATGACTAGGTATAACAATAACAAAGTGTTGTCTATCAATCAATTAAATCCATGCAACTTCACAAAACGACAAAGAGACCCTCACATAAAAGGAACACACATCTCATTGCAAACA

## >AiNAC68

AGAACTGGGGAGATTTCGAGCTTACTTCTAGAGTTTCTTGGATGAATTTTGTAGAGCTCTTCGCAAGGAACGCGTAGCCGCTGACGACACGCAAATCGGAGCTTTAGAACTCAAGATATGAGCTTGAGAAGATTGAGGTGATTAGGGTTCCACCTCTCCTTCTCTTCCTCTTTCAATTTCTGAAGTGTGTGTGTGTTTATGAGGTTAGGGTTCATTAAATGAACCTTTATATATGTTGGACTTGGGCCCAACTTGGGCCCGGTTCAACCCGTTAGCGTTTTTAGCCCGTTTGGTCCAACTTCGGGCCAAACCTTTAAAATTAACGTCCGGTTTTCCATTTCTATTGTTTTTCTAAGGTTTTTGACTGTTTCCACTTTTTCTCGCGCAGCACCGGGCAGACTTGAACCGGTTCAACCGGTTCAACTGCCGGTTCGCAATTTTTTACGGTTTTTCGCAGAAAATACATTTTCTGACTCAGAAAGACCCACTGAGTCCTAAAATGATGTTTAAAACCTCAAATTCTCACTCTAACTTTTCAGAATCAAATTTGGGCAATATAACCACTTAATTAACCGGTTCGATTAATTGCGGTTCTTACAATCTATTTGCTGTAGTGGAATACGTTTGTGTTAAACTTTGATTGGGGGAATAATGCGGGTGTAATAAGAAGGATATTTACGGTTTTTCCATTAGCTAGATTATGAAGATTCTGATTTTCTGAAATGTAGATGGATGCAGTGCATAACAATGGTGGTAAGGGAAAAAATTTGAAGAAAATGGGCGCTGCGATGAGGCGGTGGCGGAGGACACTTTAAATGATACTATTGGAAGCGAAACAGAGGGTCAAATGTGTTCGGATTTTGGAGATGTTGAGGGTTTAACTATTGAAGATATTATGCAAAAGACCTTTAGAACTGATGATGATGCATATGAGTTCTACAAAAACTTTAGTAGGTATCATGGTTTTAGTGTTTGAAAAGGAGACTCAAGAAAGGATGACGATGGAAATGTTAGGAGGAGGAGTTTTTGTTTTGTAATAAGGAGGGTTTGAGATATCCCAAGCACTATAATAGATTGGATAGGAGGTGGGTACATAAACCTGAGACGAGAACAAATTGTAAGGCGAAGTTTTCCGTTCACCTAGATAAGAATGCTTCCATATGGCAAGTAAGGAAGATCAACAACAACCACAACCATGAACTGACTCCCTATGTAATGGTGCACCTAATCCCAAAGTATAGGTCATTGACTGAAGGTGCGAAAGCGCAAATAGATGGATTGCGTGAGTGCGGGATATCGACGGCAAAAACAATGCGATATATGGCAGGATTGGCAGGTGGGTATTTATTGGTAGGATTTCTAAAGGACGCGTACAATTATATTGACAAGCAAAGGTGTGAGCAAATTGTTGACGGAGATGTTGAATCTACGATAGCCTATTTAGAAGGAAAGGCAAAGGCTGATTCTATGCTGATGGCACAGTATAATTTGACCAAGGACAGAATGCTTGCAAATATGTTTTGGGCCGACGGGGGAAGTAGGTTGGACTACCAATATTTTGGAGATGTTCTGGCTTTTGACTCGACTTACAAAAAAAATAAGTACAACAGACCACTGGTTATATTCTCATGATCAAACAATCACAAGCAGACCAAGATCTTTGGCTTTGGGCTAGTGTTGGATGAGACGATAGGGTCATATACTTGGCTGTTGAAGAGCCTGTTGGAGGTAATGTGCCAAAAGATGCCATCTGTGGTTGTAACCAATGGTGATGAAGCAATGAGGGAAGCTGTGAGAGTAGTATTCCCTAGAGCAACCCATTGGTTGTTGGTGTGCAAAATTGAACTCCGCAAGTTTCGCACAGATGAATCGGCAAGTGCACCGGGTCATCCAATTAATATCTCAAGTGAGTGAGGGTCGAATCTCACGGAGATTGTCGGATTGAGCAAGCAATGGCTATCCTGTAGATCTTAGTCAGGCGATCAGAAAATATGGTTGTTTGTTTAAAAGCATAAACAAAAAGTAAAGGAAACGAAATACCAGATTAATTTGAAAGCAATGATAGAGATTCAGTTAAGGCTTCGGAGATGCGTATTCTTTTCAGATAAACTTTTCTTACTGTCTACTCCAATAACGAATGATTCATTCAATGGCAGCCGTTATTGACTAATTCATGTAGCATCCTCATCACGTTTGCCTCTTTTAAACCATAGCAGTTCACCATATCCGAGCAACTCATGTAGCATCCTCATCAAGTTAACTCATGGTTTCCACTATAATCGAAGGTGAAGACCTAAGCAATTTACTCCCCTTTGCAATCATACTCAAAACGCCACAGACAAGGTCGGATTTTTCGGATCAGGAAATGTTGCTTCTCAGACTCTAGCCTTAACGCCACAGATACCTTAATAACCCACGGTCAACGGGATTTTATGTCATTTATCCAAAGTCGCCCAAGCACTCTCTTGGAATTCGCAGTGCATTCTCTAAGTGTGGTTCA

## >AiNAC69

AGATGTCAAATTGTCAACGCTAGCTTATCTTTTGCTATTTGTTTTGTTGTTACAACTTACAACTAGCTTGCACATATTCCCAAAACCGTCACATTCAAGACTTCTTAAGGGGCCAATGCTTAAGAGAAATTGTGAAAAAAAGGGGAATAATTAGGATGTAAATTAATCTTAGGGTTTACTTTTTTGTTTACCAATAGAAAATATTTTTAGTGTTTTATTATTATTATTATTATTATGTTTTACAAATTATAACATAAAGTGAAAATAATGTGACGATGTTCTTACAATTATAAATAAAAATGAAATATGACAACGAAAACAGAAGGTAATACTTGATATCTTTTAGAGGAATTTTGATATTAAACTTCGAGTTATTAGGTAGAAATTCAGATGCAGTTAATTTTATATGAAGTTGATAAATGATTATCATTAAATGAATTAGACAAATTTAACTAAGGGTAATGTTAGGTAACCAATAATTTTTTTAAATAATATGAACAATCATCAATTAAATTAAAATACACTACACCTCCAAATTAACCACCTAAATCTTAATATTAGAATAATCATCCACACACCTAGTGAAATGAACATCCGATCTATCAATTATTTACATTATTTAGTATTTTCATTATCTACCTATACTTTTTCTTTAACTAAATTACCATCTAACAATTCTCACCTATCAATTTCAAATGAAATTAACTATATTTAGTTTTCAACAAGTAATTAACCTTATAAATCTCGTAGATTGTAGTTATTAGCGCGTTCATTAATAGACACCTATAATGCATATTGCGCCATTTTCATAAATATCAGGCCGTTAATGTCTACTAATTAAAGTAGAAAAATTGCACTCTCTAACTCTGGTAGCTGGTAGAAAAAATAAAACTTCAGAAAGAATAATAAGACAGAATAAGCAACTAAACAAATTAATACCGATGCTAACATTGTGTGATAGTTCGGCATGATTGATGAGACTAATCATAAGATTAATATATAAAATAATGTTTATGATACTTAAGTAAATAAATAAAGCCATCGACACAGTATATACTAGCTCTCTCACTATTGGATTAATTTGATGAAAATAATAAAATAAGAAATCCAATAATAATTTGAACCATTGATAAATATATGACATGGAATGAGTCTCGGATTAAAAAAGATTCATGTACTGCATGTGAACTGGTGGAGTACACATATACATACAAAATTAAAGATTTAAAGGTTAAACCTAATTGACACACTAACGATACTAAAACAATTTTCCTGGATCATACAAAAGTCCAAGAAGTTTATGAACCTAATGATACGAATAATATATTTTCCTAATTAATATATAAAAGAACAAAACAATCGCATAGGGAGAAATCACGAATTCACTAATTTTACTTTTTTTTCTCAATGTTTACCTCATGTATGTGTGGCATTTATATCCAATGTCAACTTCGTGAGCTAAGAATTCATTAATATTTCATTAAAAGTTAGTTTTGGTAAAAATAATGCTAAACTAGCTAGGCCTAACCTTACAAAATAGAAAAAGAAATAAAAAATAAAAAGATTATTTAAATATTTTTAANNNNNNNNNNNNNNNNNNNNNNNNNNNNNNNNNNNNNNNNNNNNNNNNNNNNNNNNNNNNNNNNNNNNNNNNNNNNNNNNNNNNNNNNNNNNNNNNNNNNAATTTTCAATTAATAAAAAAAATATGTATTTAATAGTTAAAAAAAAGAGAGTTATTCAGATAAAGACGTTTAAAATATCTTTTTTTTAAAGATGTTTTCTAATAATTAAAATTTAACACATATAATTGATTAAATTATGTTATTTTTGTTAAAATTCGACTAGACAAATTAATTTGACCGAAAAATAGTGAATTAAATCTTGAATTAGTCTAAATTAATATTGTTTTTTTATAAAAAATGACTACAATATTCTCATTNNNNNNNNNNNNNNNNNNNNNNNNNGAGATTTTGTAGAAAAAAATTTATATAAAAAATAATATATTTTAAATTGTGTATATTAGCTAATTTAGCATAATAAAAACAGTTTTAATACAGTTTGTTTAATTAAACTAGTTTTTTTATCATTTAGTTTAGAATTTGATCAGATTTAATTATAATAATAAATATTTTTTAATAATGTTATGAGAATAATATCTTTTATTGATTTTGTGAGGATATAACTCCTAACAAAAATTAGATATATGAAAAGAGTATTTATAGTGTTAGTGAAAGTGAAAAATAAAAAGGGAGAGGAAGGAAGCTAAGAAAGAGCACACGAGTGTTTTAATATGTAATAAAAAGGTATAATTTTTGTGTGGGGTAAAAGAGACACGAGTGTATGGTTTAGGTGAATAAGAATAATAAAAATAGTGAAAATAGTTGAATATGTATGTATGGTGGAAGAAAGGTCCAACGGCACCGCTCTGGGGCTGTAGCCACCCTGTCTTGTTCTTGAGATGTCATTTTCTGATCTTAAAAACACTCTTAGGGCAAGCATA

## >AiNAC70

NNNNNNNNNNNNNNNNNNNNNNNNNNNNNNNNNNNNNNNTAATTATTTTTTATGTACATATAGCATGATTATTGTTATGATTATATTTTGTTATTGTAAAATAATATTAGCTTTCAAAATATCTAATATACAAATTAAAACACTAAAATAGTAATTTAAATAATAATATATAATTTAAAATTTTATTTTTTTAGGTTTTATATTTTTAAAAAATTAAGTAATTTTTCTCTTAACACTACCATCAAACTTTCGCTCTTTCCATATATATTACTAAACAATTATTTAAAAATTCACTTCCCAACACAATTAAAAGCCGACTCTTATTGATATTCATAGTTAAGAAAGTTCTTTCAATTAATATAGTTGCTACGCAAAAATTAAAGCTAATTTAAAAAGAAGATAAATAATATTCTCTTGAGTTGATTTTAAAAAAAGAACACTAATTTCGTTTAAATCTGAGAATTGATCATTCTAACGAATATCTAATATAAAATTTTTAAATTAACGATTAAGTACCAAAAGTTAAGTAAAAAATTATTATGAAGTCAAATTTAATAATTTAATAAGGACAAATAAATATTATTATCATATACAACTAAAAAAAATTCTAAATTGTAGTGGGGTGCTTGACCACACCAATATACTTGAAACTGTCCCTGTTGAGTGAGATTATGTGATTTGCATGTTTGAGGGAGTGAAAAATTAAATATTTTGGTTGAAATATAGAGAAATAGTGTGAGTGAATTTTTTTTAATAAAAATGATCAAATTTATTACAGTAGAATAATCAAATATCTTGTATAGAATAATAAAATAAAAAAGTATTATAATTTTGGATTCTTTTCAAAAGGAGGCAATAATAATGCTTTTTAATCAGCTGTATTATTGTTTGGAGAATCGTATTCCAAAATTTGCACTATAAAAAAAAGGTTTTAGTAGTATATTTAGAAGAAAATTACTCTACTTAATAATAATTACGTACATATATACCTCCCTAATAATTTGGAAGCAGCAACTAACATTTCATATTAAATATTTTAACTATTGTTATATAAACCTAAGTATGTTTTAAATTTGTTTATACACTAAATATTTTATTTTCTTATTAGTCTTTTATATCTTTGTTTTTTTTTAAACGTCAATTATGTATATATACATACTCAGAATATTTTCAACGTGGTTGGGTGGCAAATATGAACTTATTGTATAAAATAGCCGAACCTTATTAGAAATTGTAGTTACCTAGCTAAAAAAAACAACCACCATCACCCACCTAAAGCTGCGCAAGAATATAGAGAAATCAAATATATATAGATTCAACTAAAGCAGCAGCTACTACTAAAATTACTACTATATTTTTAAAAGAGCACATAATACAGAATTAATTAATTAGGACAAGAATCACACTTTTTCACAACTTTAGATAATACGCTTTTTTAGATAATATATATAGTTATTTATATATTTTGAGAAAGTCTAGGGGCAGTAATTTTTGTGTTTTATGGTCAACACTTAACCATAAAAAAAAGTAAGTGATCTCCTACTATTAGATGTAATCTCACACCATTAAAAATACTATTGATAGTCAATTGATGATTACAAAATACAAAAGTTACTTATCCCTGACACTCCTTATATATTTTTACTTAATAGTTTAAACTTGTTAAAAAGGTAACAGAAGTTTTACATAAGAAGGCGTATATTAAATAATATAGTTNNNNNNNNNNNNNNNNNNNNNNNNNNNNNNNNNNCTTAACGGCCCAAAGACTTAGATGCTTTCACTCAAATTATTTGTCCTCATGTATATGTTAGTGTTTTCTTCTTCCGAATTAACCCTCAAGCAGTAAAACTTCTTGAGAGGTGTTGGAGTACGATGGAGAATCTGCAATGGAAACAAGGCTATGAAAAAAAAATATTTAATTCAACTGAAGCCAAGAAAAAAGTGGTGGGGACTTGGAAGCAAGGGTCCACACTCCACAAGGATACACCACCGTTTCATTATCGCCGTTTTCTTTGATCACATTCGTTTTCAAACGGGTAGAGAATTAGAGACAGATATAGCCAACAAACAACATTGGTGGAACCGTGGAACATATATCAATACGAATCACAAGCTTTTTTTTAAGAGAAATATGGTCGAGACTTCTATGATTCTATCCTAACACTTTTAGTATACTAATAATTAAGAAAAATAATGTTGCATGCACTATTTTATGTATATTTTTTAAACAACAATATTATATATAGCCATCAAATATTTGCACACAATAAACATATAAATTGTACTTATATTTATTAGAATTTTATACACATAAATTAATACGGCGTGAAATTTTAGATTCAGTCAACTTTATTTGAAATTGATAGTTGAGAATCGTTAAATATTATTATGTTTGTAATCTTGCATAGGAAGTGGCTTGCAAAGCACGGATCCGTGAAGCGTAACGTGTGAGAGGTGCGTGTAGTGTATCTCTTCTCTTCTCTTGTCTATCTTGTCTTGT

## >AiNAC71

ACATTAATTAAAATTGAAGACTAATGATCTTAATCCATAGAAATAAACAGAACTCCTAACCTTAACCAAGGCGGTTTAGTGGCTCATGACTTACAGAGAAAACAAGCGTTCTGAAAAGTATGAAAGTGCGGAAGTGAGAAGATCCCTTCAAAGGTGAATCTTTTCCCTTCTATATCTAACCTAATTTGATTTGAAAATAAAATAAAATAAGAAATCCTATAACTAAAAGATACTGTTTGCAAATAAAAATTACAAAAATAAAAGATAAGATAACTAATAAAAGCTAAATCCACTAGAGATGGTCCAAAAGTGTGGGTTTCAATTCAGACCGCCTGGCACTAAACGCCGGTTGGTCGTTTAGCGCCCATAGAAGGCAGAGGCATTGCTGTTTTGTGGGGCTTGCTGGCACCCAGAATGGCAGCTCCCAATCTTCTCCTTTTTGCACAAAACTATGTCAACTTGTTTTGAATTTCACCTAAAATAATAAAAACATCAGAAAAATTTAAAGTAGCATCCAAAGAGGATTTTAACACTAAAATATAATAAAACTTACTAAATTCTAACTGAAAACGACTAGAAAATAAGGGGAAAAGGGTACAAGATGCTCACACATCAGGTGCTATTTCAATGATGACTTCTCACACACTTAAAACGGCTTTGTTTTATAGCTGACGTGTATTCTTAACGTGCCATGTTAGCTTTCGTTAACGGTATTAATGAGAGAGTTGATGGAAAGACTAACGTGACTAGCTGAAAATCTTTTGGGGATGCATTATTTGATTAAAAAAAATCTTTTAAAAACTAAATTGGAGATTACGTTATCTTTTAGGAACCATTTTGATTATTTACTCATAAAAAAATATTTAAAAATATTTCTGAAAATATATANNNNNNNNNNNNNNNNNNNNNNNNNNNNNNNNNNNNNNNNNNNNNNNNNNNNNNNNNNNNNNNNNNNNNNNNNNNNNNNNNNNNNNNNNNNNNNNNNNNNNNNNNNNNNNNNNNNNNNNNNNNNNNNNNNNNNNNNNNNNNNNNNNNNNNNNNNNNNNNNNNNNNNNNNNNNNNNNNNNNNNNNNNNNNNNNNNNNNNNNNNNNNNNNNNNNNATATTCGCGTTTAATTTATAAATGAAAATTCAAAATTATCATGATAAGTCAATATATAACTCAACCCAGCCTCTTTTGAATGTATTTGGAAACGCCTGTAAAATAACTTTTTGCTTTTTGTGTTCCTTTTCTAGCAGAAGTTGTTTTTCTTTCGTTAAAATAAAATATTTTGTTTGAAAAGATTATGAAAATTGATTTAATAAAATTTTAAAAGCGAAATTATAAGTATAATAAATATTTTAATAGCATTTATTTTCAGAAATTGAGAGAGATTGAGGAACTGAGATTTAATATTGTCTTTGGTGGACAGAGCCTAAAACTAAAATTTTGGTCTTAAAACACAAAATTTCAATCATTTTAATTCGTACTTTCAGAAAGTGGAACACAAAGAACTGAAATTTTTGAGAATGAAAACTAAAATTTTAAAATATCTCTTTTTAAAAATACTTATTTAACTTTTTAAATTCTAAATTTACTCATTATACAGAGACTTAATTTAGTTTTTGTCTCTCAATCTCTGTTTCGCAGTTTTAGTCTTTTAGTTTCAGTTTTCTTTTTGAATGCAGCCTTTGAAGTTAATTTACAGTTATAATCAGAAGTTAATAATTATTTGTGAATTACTATATATGATTTTATCAGATTATTACTTTATAAACACAGAGCTTTAGTTTTCCTCTTGTATGTACTTTAATATAAATAAATAAATATATGCTTGGGGTAGATAGCAGCATAATTTGTCATGTTTGATAGGACAGGTGTGTATATAATTTATTATGGTTCAAGACTTCAACCCAAGAATTCGAACAACAATTATTGGTATAGTAGCTTCTACAAAGATCAGAGAGAGAAAATAGAGACAGATAGATAGGAAGAAAAGTATATATTTGAAAGGAATAATTCCACCCTTTTTTGTATATATGCATTAACAAAAACCCACATATCTTAAGTCTCAAATGGTGACTTTAAACTAACAATATATAATACCATATATAGAACAAGTCCACCATCAGCAAAAATCATCACATATGGCCACAACAAGTCAAACCTCATGACTTGACCTTATAAGCTTCAATTCATCAAAAATCATACCAAAATAAATAATAATAACAATCTTCATCCATTCCACGACTCTCTATAATAATATAAAAGAAGCTAAGGTTCCTCTCCACTTCGTTGAAAAATTGTGCCACAAACCTTATTAACCGTTTTTGTCTTCTAATAATATCCCTTACTAGCTTCTTCATCTTCCTTAGAGAGAAAGAAAAAACACACACACACACACAACACAAAAAGAATAAATAAGAGAACATCATTGTTATCATTATTGTTATTATAATTGTTATATATGGGGGTCAATGAAGATTTGATGATGAAGGATGATTCATATGCATCATCAGTA

## >AiNAC72

AACCAACAACATCGGATAAGCTAATAAGGTTCATTGTCTTCATGTTTTTGTCCACTTCATTCCCCAATGGAAATCTTTTATTTTCTCCATTTTCCACCTCACAACCCAACAACCCAAATTCACCCTTGTCATGTGCATACCAAATAAGAAATAAAGAAAATATGTACATAAAATGCTATCCCTTTATTTTTTGATTTTTCACGGAATTCCCAATCTAACCTGCCAAAGATGAATTCGTCGCGAATTAGAGCTCTATTTAAAAGTTTGTCGTTGACCAATGAATTATTGTATGTGCAGTGAAATTCAAACTCTAACACTTATTTAAACAAATTAGTGAATTAACACCAGACCGACCCAAATTGGTTAATGTTGTTTCTTTATTGTATATATGATGATATATATTCTTTTTATTGCGAAGGAGAATTTCCGTTCCCACCTAGTGACTACAAATCCGAAAAATGAAGTCAATGAAATCTCTACAATGTTCATACGTAATCAATCAAAAGTTTAAATTTCTGTAATAAACACTAAAACAGATAATCTCAACTACTGCTAAGTTCTTTATTTCTTTAGCCATCTATTTGACACTATAGATATCTATAAATTTATTGGTTCGATAATATTCTACCCACATAATAATCTTTGCAGATTTATTAATATATGATATCATCTATATAAAAGTAAAAAGATAAGGATAAGAAAATAAGATATTATAAAAGAGAGGATTAACGTTGGAATAAATGGAGTGTGACACAAGGCAGAAAAAAATTGCTGATAAAGTTGTAGATGTGTGTATAACTGTATATATATATATATATAAGCCACTTGTGTTTAAATAGACCAAACTAGCAATACCGTTTCCTATGCACAATGTGAACCCAAAATTCAGTTGGATTAATTAAAATGTAAAAAAGAAAGAAAGGGAGAATAAGAATCACATCAAAACTCAAAACAGAAAAACAAAGGCTCACAGCATTGGCAAGAGGAACACACTTAATTATTTTACTCCTTTTGCCTTTTTTTCACATCTAAAAAAGGAATAGTCCCACTTGATTGCTGCTTTGTCTAATATAACTTGTTAGCCACATCAGCATAGAAAATAGACCAATGTATAAAATATAAATGAAACCAACAAGTTTTTAAATGGTCCAATAATGAGAAAATTAACTTTTATTCTGCCCCTAGATGGCTGGGTCGCTCTGCTTTTCTTTGTTTTTTTTTTTCCATGTCACAATGTTGAAAAAGCAAAAGCTTTTGTTCTTGTGTCTTTGATTTCTTTGCTAATAAATGTAAAGAGCAAAATGCTTAACCACTTAACCTACAATTTTGTAATGTGATTGTATATAGTTTAGTTTTTTCTTTTCTTTTCTTTGTTTTTAAAAAGAAAGTAGATATTACAGATATTAACCATGGACCATTATTCATGCAAGATTAATATAGAAAGAGCTCAAAAGAAAACTAAAAGGTCTTGCATATTTAAGAAAGAAAAATGTCACAAATATTCTCATTCTCAATCCCAAATTTGAAACTCTGCATAACCTCAAATGTTATAAAATCCAATGCAAATACCCATGATAGTATGATACCTTTTACAAAAATTTCACAAGGAGTAGGGTCAGATTTTATTTATTTAATTTATTTTAGTATTTTTTTTTAAATTAAAAAAAAAAGTTGATTGTGGAGTGGTAAGAATATGGCAGAAAGGACAAAGTTGAAAGCATAATAAAACTGACGTCAAAGCAAGCTTTAATTTTTAACTTTTCTTTGTGCTTTCTTGGTCATACACTCATATCTACGCAAGACAATTCCACTAATTACCGCGTATTTTGGTTCAGCATCCACTCCATAGTCCCTACAAGTTGGTCCCAGCAATGGCAGTTTAAAAAATGGAGCCAGATTGCTTTTAACAATCTACATGTGAATTTTGAAGTGAGAATATAAAAGAGCCTCTCTGAAAGTTTTTTATGAGACTTGGACCCTCACCCTCTTTGATGACACAATCACATGAATTAAAGTGTTCAATCCGACACAATATGATGTGGATTTAACTTGATGTTCAGGGTCTTGGTCGCATCATCCATCCATTTAGGTACCCTATTCCTGTTTTTTTACATGTTCACACAAAATTGAAAATTCTTTGCTTTCTCGCCAAAATTGTCTTGCAAAGTTTGTGCTGAGATTTGGGAATTGAAATGTAGTCAACAGTCACCAATAATTTGAACCGCCAAAAATATATGGACTTATATATATATATGAACAGAACTAGAAAACTAAAACTTTATAGGTGTGAACGGGGCTAATGAAGTGGTTGGTGGGGCCATTTGCTTTGGAATTTTGGGTGCACTTGAGACAAGCAATCTCAATTCTCAAACTTTCGGTGTGTTGTGTGCAATAATCTTTAACTGTTAATCCCTTCTCCCAACTGTTATATATAGCACACCATCTCTGGCTTCTTTTACTCTACTCTACTCTACTCTCTACTCTCTACTCTCCACTCTCT

## >AiNAC73

TGTGATGGTAATCACAAATTGCTCTCACTTAGTTGACCTCTAACAATTGAAGGAAAGTCAAGTGCATACAATTAACTTGAGTTCACAAGTCCTAGTCAACTCATAGTGAGAGACTAGCTTTGGTGAAGTTCAAGCTAACCGACAATCTCCAATTACCATTCAACAAAAGACTTTTGATAACTCAAGAGTCTCTAAATAATCAATCCAAGTCAAAAACATAAAAATCTAAATTAAAATCCACCCAAACATTTTATCAAACACTTGGGAGCCGCAACATAAAGCATAGAAAATTGACAAGACAGAGAAAATTCTAAGACTAACCAAAGCAAAGAAATAACCATAACAAAGCAATTGAGCAATAAAAAACATGAAACATAAATTGCATTAATGAAAATTGAGAGTAACACATGAACTCATAAACTAAATTAGCAAAATAAAGGAAGTAGATAACTAAAGTGCTAGAACAAATAAGCGTAGAAGAGAAACTAAATTAAAGTAAGGTATAAATCTGAATTTGAGAAGGAATAAGACTAAAAACCTTAAAATCTAAAGAGAGGAGAGAGCCTCTCTCTCTAGAAAACCTGCATCTAAAACCTAAAGTATGAATGAATGAGAAGTGAATGATCCCCCCTTCCAGCTTCACTCTGCACCATCTAATCAGTATTTTCGAGCTTGAAACTGAGTCCAAATCAGCCCATAAATCGCTCCCAGTGTTTTCTGTTTAATGCAGCACGTGACGCTCTGTCACGCGTATGCGTCAGCCACGCGTGCGCGTCGCTGGACTTTTTACTGGCCACGCGTAGGCGTCAGCCACGCGTGCGCGTCGTAGGTCGTTGCACATGTCACGCGTACGCGTCGCCCATGCGTGCTCGTCGCTACCAGATTCTGAAATCCTTAATTTCTTGTGTTCCTTCCACTTTTGCATGCTTTTTTTCCATCCTCTAAGTCATTCCTGCCTTGGAAAACCTGAAATCACTTGCACACATATCACGGCATCGAATGGTAATAAGAGATGATTAAAATTAGCAAATTTAAGGGCAAAGAAGCATGTTTTCAATTATAGCACAAAATTAGAAAGAAAAATATAAGCATGCGAATTATATACATAAGTGTGAGAATAATAAATAAAATTCACTCAATTCAATACAAAATAAACCACAAAATAGTGATTTATCATACATATAAATAATCTAATTCGATTTACATGAAAATATTTAAAATAGGATGTGTAACTAATTCTGATATTTACGCAATTTTAAACTTTATTTATTTTATCAAGGTGAATTATCCGAATTTTTTTCTTGTCATTAATTAACAAAAAAAATGAATGTTATATATATATTGTTACAGACCACCATCTCACATTATTTAATTACTGAAATACAAATATGTAAAATCATAATCACATAGGAATATCAAGAGAAATAAAATAAGAATGAAGGGGTGTGGATTTCTGGAAATTGGTCAGGGAAAAGTGGAGCTGTCTTCCATAAAACAATATTTTCTTTGTTTCTTTTTCTAATGATAGTTGGTGGTATCCATAAAAAAACACACCATTATTATATATATCATGTTATTATTTGTTCTTTATTCCTAGTCTCTTATACATTTAATTTAATTTCTATTTTTCTGTGTGCATTACTTTCCTTTGACCTTATTGTATATATAAGTATATGTGTGATGATCAAGTAAATGTTTTTTAGATGAGTAATGTACATGCGTGTTTATTTCATTTTATGCTAAATTTTTATTTTTATTTTTANNNNNNNNNNNNNNNNNNNNNNNNNNNNNNNNNNNNNNNNNNNNNNNNNNNNNNNNNNNNNNNNNNNNNNNNNNNNNNNNNNNNNNNNNNNNNNNNNNNNNNNNNNNNNNNNNNNNNNNNNNNNNNNNNNNNNNNNNNNNNNNNNNNNNNNNNNNNNNNNNNNNNNNNNNNNNNNNNNNNNNNNNNNNNNNNNNNNNNNNNNNNNNATATATTTTAATTATAAAAAATTTAAAATGAAATGGAATAAAAATACACCAATAATTTATACAATACAATAAGCTTATAAACCATATTAGGTATACAATGGAGCCCACTGCATCACGTGTGCATGAATGATTCACATGCATGACTCCTTTTGTTTCTCGGCGCTGAAATAAATGTGGATACTATATATTGTATATTTGTGTTTAATAGTATAATAATTAAGATTCTCTTCATATTTATAGTTTTTTTTTAATTATAATAATTATGTTTTTATATTTGTAAAAATTTTTAATTTATTGATTTATGAAGGAACAAAGATAATTGGTAAATATAATTTCTATATTATTTTAGCTACCATCTATGTTTAATATTTTTGGTAAATAATTTCTTAAGCCAATATGTGGTAACACTACATGGTGATGCATGCAGCTCAAGAGGGTTAAAATAGTCATTGTAGTTAGTCAACACTGCTAGGTGTCTCCTTAGAATTTACAACTTGCTATAAACCTGAGGCAAACTTGATGATGAAGACCC

## >AiNAC74

TACCCAGCGAGAAGTTACTGGTAGGAGTCATCTCGGCCACAATATACTCACACCTTGGCTTGGGAATTGAGTTCCTAGGATACTAGAGTCATGACGTACGATATTTAGTGGTAATTATTGGGTTGTTAGTTGATTCTTGTTTCCATTGACGCTAGCTTTTTACTAAATTAATTAGTAAGTTAGCTAGAACTTATGGATTAAGGCAATTATACTTGCTTGCCTTACTCTTCGATGTTAGGAGTTGACGAAGTGAGATTAACTCATCATAGTTACCATAGTTGTGGTTATGACGATGATAGGATTCCTTAATTCTCATTCCCAAGTCAAGGATCTTTTATGCATTTTCACTATTTTTGATCTTGCTTTCTTTTGATTTGCAGTAATTTCCTTTTTGATCTCTTGCTAGTTCTTTTATTCTTTAGTTCTTTTAATTTTCCATCTCATGCTTAAAAACCCCATGATTTCACAACCAAAAGTGGACACCTAATTTGCATAGTTTGAGGGAGACGACCCGGGATTTCAAACTCCCAGTTATTTGTTTTGGATTGTGACAATCTTTATATTTTAAACTTTGATCGTGGGAGTTATTTTAAACTTTTAAATTCGAATCAGCATAATTCTCTGCATCACTGGCCATAAGACTCTTCCCAATCTCCATAGATCTGTGCTACTGCCTTTTGCTTCGCCATCTAGACCTTCCTGTAATTAGGCCTGAATTCATAGGTTAGTATGGTAACCGTAACACCGGCTCTAACCAACGGAATGATCCTCGCACAAATGACATGATAGTCAAGCTACCGGTGATCGCTTAACATCGATGTAGTCGAGCAAATGTGAGGTCCGTTGTACCTTCTAACCTCCCAGGTACTCTTTCGCTGCCGAAGTGTGATGCGAATCAACCACGTGCAACCTTTACCAAACTCCTTGCATCTCCCATGGTACTTAAGATGATCTGACTCTATCACTCTGTACTCAACTTCACGACGGATGCTATATTCCTTTACACTCAACACAGCTTCCTCCTTACTTTGGAAAGATTGGCCAATTTGAAATTCTGTAGGAGTGGTTCTATCATGCAATCCTTGAGTCCCGAAGGTAGGATCTACGACCGGATGTTGGCCGATGGCATCCAAGTTTAAGGTTGAGAAATGTGGAGGATGTTGCTGTGTACGGAACTTAATGTGCCATGATGTGCAAGTAGATTGCTCCAAATATCCTCATCCTTGTCCCTACCAATGTCAACAAGCTCTTCGTCCGAATCATCCTCTTGCATCACATTTTCAACTTGATCCAGTTCACCCTCGCCTGATAAATCAAGAATCAGACGTGGTGAAACTAGTCATCCCAACTACAATATATGGAGGATCAACCAACGAACAAGAAGGTGCAACCACAGGTATCGAGGTAGAAGCACCCCCCCAACGTAGTCGACTGAGGATTCAGTGCTGATGCCCCAGAATTGTCGACACCATCCTCCAACTTGGCATACAATTCGTGTATTCTCACTTCCAAAAAACTTCGCCGATAATGAAACAAAACCTGCATATCTTCATCGGACCCTATCACAAATATATCGTACTTCACACCGGTTGAAACAACAGCAATAGAGATCTTGTAGAACAACTTCTTCACCCACTTTGCCCTACACACCCCTACCTTTTGTAGTATACTGATTTTCAGCTCTGACAAAGTGTTCGATGACCAGATAAAGACACTCAGTGGTTCCCTATCAGTAAATTTAACACTGTGTCTTTTGCTCTTTTGAATTCTTCCAGAGCAATGCACTAGAGCTAGAAAACTCTTCTTACCATTCATTGTAAAAATGGCACTCTACTTCACAATTGCCTTCTACTGGTATATATAGAGAATCTACCTATACATAAACCGCTATAGAGTGCCAGAAATTAAACTACCTAATGTTTCTTCATAAACCGCTATACCCAGTATTATCTTGTAACAGATTATGTTAAAGTTAAAAATTGCTACACCCTATATCAATTTACAAAAAAAAAATTACAAACATATATGCTATTTCAAAAAGTTGCAATATGATAAATTTAAAATCCAATTATTTTATTTCAATAAGTTACCCAATATTTAAAAGGGTAGAGTCTAGTAAGTAGTATTGCGTGTTGTAGGGTATGGATGGTGTCGTGGCCCACAGTGCCTTTTGAAGGGGTCCTGTTTTTTTTGTTAGTTTGTGAGCAGACAAAAGCAGAGAAGCTTCTTATCCTCTCCCACTAGGTTTTCACACAGCACCATACACACAGAATCTCTTCTCTCTTCATAAAGCACAACACACAACAAACCTCCTTCAATTCTCACATAAAATAATACACTTGAATTCAGGGGCAAAATGGTAAATCCGCAATCACCGTTTTTTCTCAACAGTGGCCTATTTAGTTCAATATCGTACAAGTCGGTTTAGTTCATAATATCAAGTGCCACAGTTCCCAAAATTTCACTCTCGTCTCTCTCTCTCTCTCTCTCTCTACTCTCT

## >AiNAC75

ATTTTCCTCAGGATATAGTGTTCTGATTTCGAGGATAACAAAGATTTTTCAGTTATAAAATTTTTTATATCATATATTATGAAATCACTTCTTTTCAAAAATTAAACTCATAGCTAGAGGAAGAAACATTATATCTCTAACCCCCTAAATGTATGCTTTAAATTTAAATTGAAATCAATTCTCAAACATTCTTTCATCTTTTATGCAGTTTTTTTTTTTTATGGAGATCGATTTTAGCTATAAATTTTAGAAACAAAACAAGAGTAAGAATGATTTGTACCATAAAATATATATACAATTGATAGATAGAAATTAAACTTAAAAAATTTAGTTTAACTACTACAAATAAAAATGTCAAATAATCTAACTCAATTCATTTAGGTTCATGAAATAAAAGCAAACTCCACTCATTTAATAACGGTTCATGCGTTAGTGGATGTAGGTATCAAAAGAAAAGAAGCGTATACACATTTTTTTCTTCATAATTTTCATTTATCATTATATTTTTATTTTGTTCACGGTATTCTGCAATTCGACATGTTAAGGATTAATCCATGACGAATCTGAATTCCATTTAAGTGTCGAATCTCCGACACTTGCTTAAGTCAACGAATGAGTTGACCATTCGACCAACCCAAGTTGGTTGGATAAACCCTGTTCATGATATTACTGCAAATGCGCTAATTTACTCATGATTTTATTATGTTTGATTATGGAGGAAAAAGTAGGATGAAAATTTTTATACATGATTTTGATACGTAAAAATTATACATATGGTTTATTTATGAAAAATTTTAAACATAGAACTTTATTTGTGTTGTGAGCCAATATTTTCAATCAACAATGACATTTGGATTGTCGCTCAATAAAATCGTCAATGTTCAAAGCTAAATGAGTTGAGGCCAGATAAGATTATGCTATTCATTTTTACGATAAGCTTAAGGAAAGGGAAAAGGAAAAAATAGAATGAGAAAAAAAAAAGATGGAAATGTATGTAACTTCGATGATTTTTGTTTTCCTGTGCTACAGATATATATACATACGAATGTTTGACTTCCAATATAGACTTTGAATTTATTTTTTATTTGAATTAAAAAAAGACGAATTTCAAATATATTATCAGTTTGAAATTTATCTACATTATCATTGAATTATGTGTCAATTATAAGAATAAATATGTGTATGCTTTCTATTTGGCTTATCTTTTCATCTCATTTCAAAAGCATAGCAATTATGTGAAGTATATATATCACCATCAAATTATAAGCATAAATAAGAAAACAAAGAGATTCAAAGAGATGAAAGAAATGAGAGGGAGAATATACGGTAGCAGCCCAAAAAGAGTAGTAGAAATTGTAGCTAAGCTATATAGCAGCTTTAAGACACTGTTCATGGAGAATAATTTTGTACTTGAAACAGTCGCATTCGCCTTGGACGGGTCAATATTGAATGATTCTACTCATTAGTTTGTTTGAAAAAAAAAATAAATAAAATTATCAAAAATTGACTTTGATGAAATTATGTTCACAAACTCTGCTTTTGCTCCGGAAAAAAAAAACTCTGCTTTTCCTAATCATTTAGGACAAACCTAGTCATCACTCGTTACTAAGCTGCTTTTGCTAGGCATTATAGTAATTTCATTGTGAACTTTTTGTTTAATTTACAACTTAAAATTTATATTGTTGCAGTTTTATGCGACTCATTACAATTTTGATGTAACTATATGCAAGCAAATCATGTGATTCTAAACTGTGATTACCGATAAATGCAGCCAGGGACAACACAATATTACTAACATGGATGAATTGAAGCTGAAGTTGCATGTACGTATATCGGAATATAGGTACGGTATCTATACGCGGTACGAAGAATACGGATATGGGATACGATAATTTTAGAAAAAAAAAAAGGAGTGTTAGGAGGCCAGTAAGTTTTGTGATTTGTAGTCATTAATTAATTATTATTGGTATTTTTAATGGTGTGAGATTTCATCCAATGGTGTGGGATTACTCACTTTTCTTTTGATGGTTAAGTGTTAGTCAGATTTTAATAAAAGTGCTAGCTTTTTATACTTTCACAAAAAAATATATGAGTGTATAAGTAAATTTTGCAAATTTTTAAAAATAATGATATTTTTTAACTGATAAAATATGTTTTTAGTCTAATTTAATTGATCTTAAATTAACAAATAAATATTTAAATAGTCTAAGTAGTAAATTTAATANNNNNNNNNNGTAATTTTGAAATAAAATAAATAAAAAAATTTATTCAAAATGACGAAGTTTTTGAGTAATTTAAACAAAAAAATGTATCTAATAGTATCTAATACATACATACTCAAAACTACTTTAGACTTAAAAAAAAAAGCCATTTGTTAAATACGTATTTGGACGTACTCAAGCATACCCAACGCGTAGCAGTGCAACGTAGCAGAGAGCAGAAAAACATTATAAGAGTGAGTGACAAGTTATGTTCATGTGAAATGGCGGGAAGTAAACTA

## >AiNAC76

AGAAGAACAATGGTCATCGTCAGGCTGTTATTGCTGGTATCTTCACTTGTCTTATCGGCATCAAGAAGAGTTCCTTCTTCTTCTTCTTCTTCTTCTCATTCTTCTGGTAACATTGATTGGTGGTGCAACCTAACGCCACACCCTGGAACATGCAAGTACTACTTAGGTCAAAGCAACCAACAGCACACAACAATAATGAAGCACAAAACCGAGTTGAGGAGGATGCTTGTGAAATCTGCATTAGAGGAAGCAACCATGATGCAAAAGGAAGCACATGGTTTGGACCAAAACTTGATCAAGACAAAGAACCATGAAGCTGTGCATGGCGATTGCTTGAAGCTCTACGACGACACCATCTTCCATCTCAAGCGTACCCTGGAATGCCTTAACAACAACAACTGTTCAGCAGTTGATGCACAAACATGGCTCAGCACTGCTCTCACAAACATCCAAACGTGTCAAACGGGTGCACAAGAACTCAGCGTTCAAGATTTCAAGGTTCCATCTAAGAACACCAATGTCACTGAGATGGTGAGGAATAGCTTAGCCATCAACTTGGATTTCGTCAAGATGATGAAACAACAACCACATGCAAATCGCACATTACCAGAAGCAGAGGAACAAGAAACAGAAGAAGATTTTCCAAGCTGGTTTTCCGGTCACGAAAGGAAGCTTCTTCAATCTGGCTCTAGCGCGATAAAGGCTCACGTTGTGGTGGCGAAAGACGGATCGGGGAATTTCAAGACGGTGCAAGAAGCGCTGAACGCGGCGGCGAAGAGAACCGTGAAGACAAGCAGATTTGTAATATACGTGACAAAAGGAGTATACAAAGAAAACATAGAGGTGGAGAAAAACAACGATAACGTGATGCTGTTTGGTGACGGCATGAGAAACACCATCATTACCGGCAGCAGAAGCTCTCAAGACGGTTACACAACATACAGCTCCGCAACCGCCGGCATAGATGGGCTTCACTTCATCGCAAGAGACATCACTTTCCAAAACACCGCGGGCCCACGCAAGGGCCAAGCTGTGGCCCTGAGATCCGCCTCCGACCTCTCTGTGTTCTACAAGTGCGGCATTGTGGGCTACCAAGACACGCTCATGGCCCACGCGCAGCGCCAGTTCTACAGACAGTGCTACATCTACGGCACCGTTGACTTCATCTTCGGCAACGCCGCCGTGGTCTTCCAAAACTGTCACATATTTGCAAGAAAGCCCCTGGATGGGCAGGCCAACACCATCACCGCACAGGGCCGAGGGGATCCCTTCCAGAACACCGGCATCTCCATCCACAAATCAGTTATCAAAGCCGCACCGGATCTCGTTCCTGTTTTGGACAAGGTTCAGACCTTCTTGGGCCGGCCCTGGCAGCAGAACGCTAGGGTTGTTGTCATGAGGACTTATTTGGACTCTCTTATAAGCCCATTGGGCTGGGATGAATGGAATGGATCTGACTTTGCCAAGGATACTTTGTATTTCGGAGAGTACGAGAATTCTGGGCCTGCTTCCGATACAAGCAAAAGAGTGAAATGGCCCGGTTTTCATGTGATATCAAACCCAAAAGAAGCGTCACAATTCACTGTGACTTCTCTTCTTGCTGGTCGCACCTGGTTGCCTACCACATCTGTTCCCTTCAGCTCTGGCCTCTGATTCTCGTGTAGTTTGTTTTCAGCGTTCATTCTTTTGTACATTTGAGCAAGCGAGAGAAATTATTATTTGTGTGTCTTTGATCGAAATTAAATTAAATTAAATTAAATTAAATTATTATTTCAATTATAACCTACTCGATGTATTCTATCTTTTGTATGAGTTAAGACAAATGAATGAAATGATGATTGCCACTTTTATAAAGGATGTTGTGCAAAAGGTGAATTATATATTATTAGTCTAATTATTATAAGGCCAACAACAACTAATTAACCATCACACTTTTCAGCAACTCACTATAGCTCTTTCATTTGTCCATCATATATATATATATAAATGTTGTAAAATATGAAAAACCAATAATAATGGTACAAATTACACTTATATTAGGTAGATGAAGAGATGAAGATGATGATTATTATGAAGGAGTGTGGGCGTGGAAGCTGGCTGCTCCATTACGTCAACTTATCCCTTTTTGTGTTTGTTTCTTTCTTTTAAGATTTGGTGATTGGTCTGAGCCGTTACGTTAGTTCCTCTTTCATTCATTCAGCGCTTAATAGCAAAGATTAGTATATAGAGATAGAAAAAGACCCTTAGGCCTTAGCTAGCATGTTCTCTTGTTAAGCACTCTTTGTTTGGTGGCGGGTAGAAACAAACACTTTGTGATCCCTTATGCCATATTTTCTGCCACATTTTTTTGCCCGCTTTCGCTTCTAAAAGCTTTTCCACCAATTCAGTCCTGACAGTGAGACATGGATCATGACACAAATACAATACATCCATTTATAGAATCACCTAAGATCATATCCTTTATTTAGTTTCATTGCTACAAAACCAAACACACACAATT

## >AiNAC77
[truncated: 7,531 more chars]
