# Supplementary material for: Comprehensive genomic characterization of NAC transcription factor family and their response to salt and drought stress in peanut
Source: BMC Plant Biol. 2020 Oct 2;20:454. doi: 10.1186/s12870-020-02678-9 (PMC7532626; doi:10.1186/s12870-020-02678-9)
Supplement: Supplementary file 12 — Additional file 12. Number of different cis-acting elements present within the promoter of NAC genes. [file 12870_2020_2678_MOESM12_ESM.docx]

|  | ABRE | ARE | TGA element | CGTCA | TGACG | ERE | GARE | TATC | P | W1 | HSE | LTR | MBS | TC-RICH |
| --- | --- | --- | --- | --- | --- | --- | --- | --- | --- | --- | --- | --- | --- | --- |
| AdNAC1 | 1 | 2 |  | 1 |  | 1 |  |  |  | 1 |  |  |  |  |
| AdNAC2 | 1 | 1 |  |  |  |  | 1 | 1 |  | 1 |  |  |  |  |
| AdNAC3 |  | 4 |  | 4 | 1 | 1 |  |  |  | 1 |  |  | 1 |  |
| AdNAC4 |  | 1 |  | 1 |  | 1 | 2 |  |  |  |  | 1 |  |  |
| AdNAC5 |  | 1 |  | 1 | 2 |  | 1 |  | 1 |  |  | 1 | 1 |  |
| AdNAC6 | 7 | 6 |  | 6 | 1 | 1 |  |  |  |  |  |  | 2 |  |
| AdNAC7 |  | 1 |  | 1 |  |  |  |  |  |  |  |  |  | 1 |
| AdNAC8 |  | 2 |  | 2 | 1 |  | 1 | 1 |  | 1 |  |  |  |  |
| AdNAC9 |  |  |  | 1 |  |  |  |  |  |  |  | 1 | 1 |  |
| AdNAC10 |  | 3 | 1 | 1 | 1 |  | 2 | 1 | 1 |  |  |  |  |  |
| AdNAC11 |  | 1 |  | 1 | 1 |  |  |  |  |  |  |  |  |  |
| AdNAC12 |  | 4 |  |  |  |  | 2 |  |  | 1 |  |  |  |  |
| AdNAC13 |  | 2 | 2 |  |  | 2 | 1 |  |  | 1 |  | 1 | 2 |  |
| AdNAC14 |  | 1 |  |  |  | 2 | 1 |  |  |  |  | 1 |  |  |
| AdNAC15 | 1 | 4 |  | 2 |  | 2 |  | 1 | 1 |  | 1 |  | 1 | 1 |
| AdNAC16 |  | 2 |  | 4 | 1 |  | 1 |  |  | 1 |  | 3 | 1 |  |
| AdNAC17 | 2 |  |  |  | 2 |  |  |  | 1 | 1 | 1 | 2 |  |  |
| AdNAC18 | 2 | 1 |  | 1 |  |  |  |  |  |  |  |  | 2 |  |
| AdNAC19 | 2 | 2 |  | 1 | 1 |  |  |  | 1 |  |  |  | 1 |  |
| AdNAC20 | 2 | 2 | 1 | 2 | 2 | 1 |  |  | 1 | 2 |  |  | 3 |  |
| AdNAC21 |  |  |  |  |  |  |  |  |  | 1 |  |  |  |  |
| AdNAC22 | 3 | 3 | 2 | 2 | 1 |  |  |  |  |  |  |  |  |  |
| AdNAC23 | 1 | 3 |  | 3 | 1 |  |  |  | 3 |  |  |  | 1 |  |
| AdNAC24 |  | 3 |  | 1 | 2 |  | 1 |  |  |  |  |  |  |  |
| AdNAC25 |  |  | 1 | 6 |  |  |  |  |  |  |  | 1 |  |  |
| AdNAC26 | 1 | 2 |  | 1 |  |  | 2 |  | 1 | 1 |  |  | 1 |  |
| AdNAC27 | 1 | 3 |  | 2 |  | 2 | 2 | 1 | 1 | 3 |  | 1 |  |  |
| AdNAC28 |  | 1 |  | 1 | 1 | 1 |  |  | 1 |  |  |  |  |  |
| AdNAC29 |  | 1 |  |  |  |  |  |  |  |  |  |  |  |  |
| AdNAC30 |  | 4 | 1 | 1 | 1 | 1 | 2 | 3 |  | 1 |  | 2 | 1 |  |
| AdNAC31 |  |  |  |  |  |  | 2 |  |  |  |  | 1 |  |  |
| AdNAC32 |  | 2 | 1 |  | 1 | 1 |  |  |  | 2 |  | 1 |  |  |
| AdNAC33 |  | 2 | 1 | 3 | 1 |  |  |  | 3 |  |  |  | 2 |  |
| AdNAC34 | 2 | 4 | 1 | 1 | 6 | 1 |  | 1 |  | 2 |  | 1 | 1 |  |
| AdNAC35 | 2 |  |  | 1 | 1 |  | 1 |  |  | 1 |  | 1 | 1 |  |
| AdNAC36 |  | 1 |  | 1 | 1 |  | 2 |  | 1 |  |  | 1 | 1 |  |
| AdNAC37 |  | 1 |  | 1 | 3 |  |  | 1 | 2 |  |  |  |  |  |
| AdNAC38 |  | 1 |  |  | 1 | 1 |  |  |  |  |  |  |  |  |
| AdNAC39 |  | 2 |  |  | 1 | 1 |  | 1 | 1 |  |  | 2 | 2 |  |
| AdNAC40 |  | 2 |  | 1 |  |  | 2 |  | 1 |  |  |  |  |  |
| AdNAC41 | 1 | 3 | 1 |  |  |  |  |  |  |  |  |  | 1 |  |
| AdNAC42 |  |  |  |  |  |  |  | 2 |  | 3 |  | 1 |  |  |
| AdNAC43 |  | 2 | 2 |  | 1 |  | 1 |  |  |  |  |  | 1 |  |
| AdNAC44 |  | 6 | 1 |  |  | 4 |  |  |  |  | 1 |  |  | 1 |
| AdNAC45 |  | 9 | 1 | 2 | 1 | 3 |  |  | 1 |  | 1 | 1 | 1 |  |
| AdNAC46 |  | 2 |  | 1 |  |  |  |  | 2 | 1 |  | 1 |  |  |
| AdNAC47 |  | 1 | 2 | 1 | 1 |  |  |  | 2 |  |  | 2 | 1 |  |
| AdNAC48 |  | 1 | 1 |  |  | 1 | 1 |  |  |  |  | 2 |  |  |
| AdNAC49 | 4 |  |  | 5 | 1 |  |  | 1 |  | 1 |  |  | 1 |  |
| AdNAC50 |  | 2 |  | 1 |  | 1 |  |  |  | 1 | 1 |  | 1 |  |
| AdNAC51 | 1 |  | 2 |  | 1 |  |  |  | 1 | 1 |  |  | 2 |  |
| AdNAC52 |  | 4 |  | 3 | 1 |  |  |  |  |  |  |  |  |  |
| AdNAC53 |  | 2 |  |  |  |  |  |  | 1 | 1 |  |  | 1 |  |
| AdNAC54 |  | 2 | 1 | 2 | 1 | 2 |  |  |  |  |  | 1 |  |  |
| AdNAC55 |  | 3 |  |  | 1 | 1 |  |  | 1 |  |  |  |  |  |
| AdNAC56 |  | 1 |  |  |  | 2 |  |  |  | 1 |  | 1 |  |  |
| AdNAC57 |  | 2 | 2 | 1 | 3 |  |  |  | 1 | 1 |  |  | 2 |  |
| AdNAC58 |  | 1 |  |  |  |  |  |  |  | 1 |  |  | 1 |  |
| AdNAC59 |  | 1 |  | 2 |  |  |  |  |  |  |  | 3 | 2 |  |
| AdNAC60 | 1 | 2 |  | 1 |  | 1 |  | 1 |  |  |  | 1 | 2 |  |
| AdNAC61 |  |  | 1 | 3 |  | 1 | 1 |  |  |  |  |  | 1 |  |
| AdNAC62 |  | 3 |  |  |  | 1 | 1 |  |  | 1 | 1 | 1 | 1 |  |
| AdNAC63 | 1 | 1 | 1 | 2 | 2 | 1 |  |  |  |  |  | 1 |  |  |
| AdNAC64 | 1 | 1 | 1 |  | 1 | 1 |  |  |  |  |  | 2 | 1 |  |
| AdNAC65 | 1 | 2 |  |  |  |  |  |  | 2 | 2 |  |  |  |  |
| AdNAC66 | 3 |  | 1 | 1 | 2 |  |  |  | 1 | 2 |  |  |  |  |
| AdNAC67 |  |  |  |  |  | 1 |  |  |  |  |  | 1 |  |  |
| AdNAC68 |  | 1 |  |  |  | 1 | 1 |  |  | 3 |  | 2 |  |  |
| AdNAC69 |  |  |  |  |  | 1 |  |  | 1 | 1 |  | 1 |  |  |
| AdNAC70 |  | 2 |  |  | 1 |  | 1 |  |  | 2 |  | 3 | 1 |  |
| AdNAC71 |  | 2 | 1 |  | 1 | 1 | 1 | 1 |  | 1 |  | 2 |  |  |
| AdNAC72 |  | 1 | 2 | 1 |  |  |  |  | 2 | 2 |  | 1 | 2 |  |
| AdNAC73 |  | 2 | 1 |  | 1 | 2 |  |  |  |  |  |  | 1 |  |
| AdNAC74 |  | 2 |  | 2 | 2 |  |  | 1 |  |  |  |  | 3 |  |
| AdNAC75 |  | 3 | 1 |  |  | 1 |  |  |  | 1 |  |  | 1 |  |
| AdNAC76 | 1 | 3 |  | 2 |  | 1 |  |  |  | 3 |  |  |  |  |
| AdNAC77 | 3 | 1 |  | 2 | 2 | 1 | 1 |  | 1 | 2 |  | 2 |  |  |
| AdNAC78 | 1 |  |  |  |  |  |  |  |  |  |  |  |  |  |
| AdNAC79 | 1 | 2 | 1 | 1 | 1 |  |  |  |  | 3 | 1 | 1 | 1 |  |
| AdNAC80 | 4 | 2 |  | 1 | 1 |  | 1 |  |  | 4 |  |  | 1 |  |
| AdNAC81 |  |  | 2 |  | 1 |  |  |  | 1 |  |  |  | 1 |  |
| AiNAC1 |  |  | 1 |  | 1 |  | 1 | 1 | 1 |  |  | 2 | 1 |  |
| AiNAC2 | 1 | 2 | 1 | 2 | 2 |  |  | 2 |  |  |  |  | 1 |  |
| AiNAC3 |  | 4 |  | 2 | 1 |  |  |  | 2 |  |  |  | 1 |  |
| AiNAC4 |  | 4 | 1 | 1 |  |  | 1 |  | 1 |  |  | 3 | 1 |  |
| AiNAC5 |  | 1 | 1 | 1 |  |  |  |  |  | 1 |  | 3 |  |  |
| AiNAC6 | 1 |  | 1 | 3 | 1 |  | 1 |  | 1 |  |  | 1 | 1 |  |
| AiNAC7 | 2 | 3 |  | 1 |  | 2 | 1 |  |  | 1 |  |  | 1 |  |
| AiNAC8 |  | 1 | 2 | 1 |  | 1 |  |  | 2 | 1 |  |  | 1 |  |
| AiNAC9 | 5 | 2 |  | 1 | 1 |  |  |  |  | 3 |  |  | 1 |  |
| AiNAC10 |  | 3 |  | 1 | 1 | 2 |  |  |  | 3 |  | 1 | 1 |  |
| AiNAC11 |  |  |  |  |  | 2 |  |  |  | 2 |  | 1 |  |  |
| AiNAC12 |  | 3 | 2 |  |  |  | 1 |  |  | 1 |  | 1 |  |  |
| AiNAC13 |  | 1 | 1 |  | 1 | 1 |  |  |  |  |  |  |  |  |
| AiNAC14 |  | 2 |  |  |  |  |  |  |  |  |  |  | 2 |  |
| AiNAC15 |  | 2 | 4 | 1 |  | 1 |  |  |  | 2 |  |  |  | 1 |
| AiNAC16 |  | 1 |  |  | 1 |  |  |  |  |  |  |  | 1 |  |
| AiNAC17 | 3 | 2 |  | 5 | 1 |  | 1 |  |  | 2 |  | 1 | 1 |  |
| AiNAC18 |  | 2 |  |  |  |  |  |  |  | 1 |  |  |  |  |
| AiNAC19 | 1 | 2 |  |  |  |  |  | 1 | 1 |  |  |  |  |  |
| AiNAC20 |  | 2 |  | 1 |  |  |  |  |  |  |  |  |  |  |
| AiNAC21 |  | 2 |  | 3 | 2 | 3 | 2 | 1 |  |  |  |  |  |  |
| AiNAC22 | 1 | 3 |  | 2 |  | 1 |  |  |  | 3 |  |  |  |  |
| AiNAC23 |  | 1 |  | 1 | 2 | 1 |  |  |  |  |  |  |  |  |
| AiNAC24 | 1 | 1 | 1 | 1 |  |  |  |  | 2 | 3 |  | 1 |  |  |
| AiNAC25 |  | 2 | 1 |  |  | 2 |  |  |  |  |  | 2 |  |  |
| AiNAC26 |  | 1 | 1 | 1 |  | 1 |  |  | 1 |  |  |  |  |  |
| AiNAC27 | 1 | 4 |  |  | 1 | 1 |  | 1 |  | 1 |  | 2 |  |  |
| AiNAC28 |  | 6 |  |  |  | 1 | 1 |  |  |  |  |  |  |  |
| AiNAC29 |  | 1 |  |  | 1 | 1 |  |  | 1 |  |  | 1 | 1 |  |
| AiNAC30 | 6 | 1 | 1 | 2 | 2 | 2 | 1 |  |  | 2 |  | 1 | 2 |  |
| AiNAC31 |  | 1 |  |  |  |  |  |  |  | 1 |  |  |  |  |
| AiNAC32 |  | 2 | 1 | 1 | 3 | 2 |  |  | 1 | 1 |  | 1 |  |  |
| AiNAC33 |  | 1 | 2 | 1 |  | 1 |  |  |  |  |  |  | 1 |  |
| AiNAC34 |  | 1 | 1 | 1 |  | 2 | 1 |  |  |  |  |  | 3 |  |
| AiNAC35 | 2 | 3 |  |  | 1 | 1 | 1 |  |  |  |  | 1 |  |  |
| AiNAC36 |  | 3 |  | 1 | 1 | 1 |  | 1 |  |  |  |  |  |  |
| AiNAC37 |  | 3 |  |  |  |  |  |  |  |  | 2 |  |  |  |
| AiNAC38 |  |  |  | 1 | 2 |  |  | 1 |  |  |  | 1 |  |  |
| AiNAC39 |  | 4 |  |  |  |  |  |  | 1 | 2 |  | 1 | 1 |  |
| AiNAC40 |  | 3 | 1 | 3 | 1 | 2 |  |  | 1 |  |  | 1 | 3 |  |
| AiNAC41 |  | 2 |  |  |  |  | 2 |  | 1 |  |  |  |  |  |
| AiNAC42 |  | 2 | 1 |  | 2 |  |  |  |  |  |  |  |  |  |
| AiNAC43 | 1 | 3 |  |  |  | 1 | 1 |  | 1 |  |  |  |  |  |
| AiNAC44 |  | 1 |  |  |  |  | 1 |  |  |  |  | 1 |  |  |
| AiNAC45 |  | 2 |  | 1 |  |  |  |  | 2 |  |  |  | 3 |  |
| AiNAC46 |  |  |  | 1 | 2 |  |  |  | 1 |  |  | 1 |  |  |
| AiNAC47 | 3 | 1 |  | 1 | 2 |  |  |  | 1 | 2 |  | 1 |  |  |
| AiNAC48 |  | 2 | 1 | 1 |  |  | 1 |  |  |  |  |  |  |  |
| AiNAC49 |  | 3 |  | 3 |  | 1 |  |  |  | 1 |  |  | 2 |  |
| AiNAC50 |  | 5 | 1 |  |  | 1 |  |  |  | 2 |  | 1 | 1 |  |
| AiNAC51 | 1 |  |  |  |  |  | 2 |  | 1 | 1 |  |  | 2 |  |
| AiNAC52 | 1 | 2 | 1 |  |  | 3 | 1 |  |  |  | 1 |  |  |  |
| AiNAC53 |  |  |  |  |  |  |  |  |  |  |  |  |  |  |
| AiNAC54 | 2 | 9 |  | 6 | 1 | 1 |  |  | 1 |  |  | 3 |  |  |
| AiNAC55 | 1 | 2 |  | 2 |  | 1 |  |  |  | 2 |  | 1 |  |  |
| AiNAC56 |  | 4 | 2 | 1 | 3 | 1 | 1 |  | 2 | 1 |  |  | 2 |  |
| AiNAC57 | 3 | 1 | 1 | 2 | 1 |  |  |  |  | 2 |  |  |  |  |
| AiNAC58 |  | 4 | 1 | 2 | 1 |  | 1 |  | 1 |  |  |  | 1 |  |
| AiNAC59 |  |  |  | 2 |  |  |  |  |  |  |  | 2 | 1 |  |
| AiNAC60 |  | 3 |  |  |  |  |  |  | 1 | 1 |  |  |  |  |
| AiNAC61 |  | 2 |  |  |  | 1 |  |  |  | 1 |  | 3 |  |  |
| AiNAC62 |  | 3 | 1 |  | 1 | 2 | 1 | 1 |  |  |  |  | 1 |  |
| AiNAC63 |  | 1 |  | 1 |  |  |  |  |  | 1 |  |  |  |  |
| AiNAC64 | 1 | 3 |  | 1 | 1 | 1 |  | 1 | 2 |  |  |  |  |  |
| AiNAC65 | 1 |  | 1 |  |  |  |  |  |  |  |  | 1 | 1 |  |
| AiNAC66 | 1 | 1 | 1 |  | 1 | 1 | 2 |  | 1 | 1 |  |  |  |  |
| AiNAC67 | 2 | 2 | 3 | 3 |  |  |  |  | 1 |  |  | 2 |  |  |
| AiNAC68 |  | 3 |  |  | 3 |  | 1 | 1 |  | 3 |  | 1 | 1 |  |
| AiNAC69 |  | 1 |  | 1 | 1 | 1 | 1 |  |  | 1 |  | 1 |  |  |
| AiNAC70 |  |  |  | 1 |  | 1 |  |  |  | 2 |  |  | 1 |  |
| AiNAC71 |  |  | 2 |  | 1 |  | 2 |  | 1 | 2 |  |  |  |  |
| AiNAC72 |  | 1 | 1 | 1 | 1 |  | 2 |  | 1 | 1 |  | 2 | 2 |  |
| AiNAC73 | 2 | 2 | 1 | 2 | 1 |  | 1 |  | 1 | 2 |  |  |  |  |
| AiNAC74 | 1 |  |  |  | 4 | 3 |  |  | 3 |  |  |  |  |  |
| AiNAC75 |  | 1 |  | 1 | 2 | 4 |  | 1 |  | 2 |  |  |  |  |
| AiNAC76 |  | 4 |  | 4 | 1 |  | 1 |  | 3 | 1 |  | 1 | 2 |  |
| AiNAC77 | 2 | 3 | 8 | 1 | 1 |  | 3 |  |  | 1 |  |  | 1 |  |
| AiNAC78 |  | 3 | 1 |  |  |  |  |  |  |  |  |  |  |  |
| AiNAC79 | 1 | 2 |  |  |  |  | 2 |  |  | 2 |  |  |  |  |
