# Supplementary material for: Comprehensive genomic characterization of NAC transcription factor family and their response to salt and drought stress in peanut
Source: BMC Plant Biol. 2020 Oct 2;20:454. doi: 10.1186/s12870-020-02678-9 (PMC7532626; doi:10.1186/s12870-020-02678-9)
Supplement: Supplementary file 15 — Additional file 15. Primers used in this study. [file 12870_2020_2678_MOESM15_ESM.docx]

| Gene ID | Forward primer | Reverse primer |
| --- | --- | --- |
| AhNAC1 | ATGAACATGCCACGTCACGA | AGAAGGGGCTAATTCCAGCA |
| AhNAC10 | TGTTCATCGAGCGAAAGCCT | AGCACATCCCAACTAATGGCT |
| AhNAC18 | GACTCTAGCTGCTCGGATCAC | TTGCTCCACTTTGGGTTGCT |
| AhNAC22 | GCTTCTTCCAATGAATCCAGTGAT | CCACTTCATCCACCTTAGAGTGA |
| AhNAC27 | TGGGTCATGCAGGAATACCA | CGGTGAGCAGTTCCATTTGC |
| AhNAC37 | CTGGAAGGTATGATGCTAGGAGG | ATGGTGTTTGAGGCTGATCCC |
| AhNAC65 | ATGTCAAATGTGTCCTATCACAGAA | AAGGTGAACAATGTCAACGTGAA |
| AhNAC73 | ACCACAAGGGACAGCAAACA | ACCATTTCCTGTTGGAACCGT |
| AhNAC83 | GAACGAGACTACCTCACCGC | CTGTCCAACCTGGGTAACCG |
| AhNAC87 | CCTCTTTGTCCCTTCGGTGT | TCCGGATTGAGTCTTCACGC |
| AhNAC102 | GAGGTTCAGAGCGAGCCAAA | GGAACCCAAAACCGTTGGTG |
| AhNAC103 | TAACACTCCTTCTCCGCTTGC | CATGTTGCTGTACTGCCGCT |
| AhNAC117 | CTGCCACGTCAACACAATGG | TCTCATCAATCCGCTGGGTC |
| AhNAC156 | ACAGTTCCTTTGTTTCACTGCTT | TCAGCAACAGTAGGGCCAAG |
| Actin | TTGGAATGGGTCAGAAGGATGC | AGTGGTGCCTCAGTAAGAAGC |
